# Cancer Testis Antigen Promotes Triple Negative Breast Cancer Metastasis and is Traceable in the Circulating Extracellular Vesicles

**DOI:** 10.1038/s41598-019-48064-w

**Published:** 2019-08-12

**Authors:** Anbarasu Kannan, Julie V. Philley, Kate L. Hertweck, Harrison Ndetan, Karan P. Singh, Subramanium Sivakumar, Robert B. Wells, Ratna K. Vadlamudi, Santanu Dasgupta

**Affiliations:** 1 Departments of Cellular and Molecular Biology, The University of Texas Health Science Center at Tyler, Tyler, Texas USA; 2 Departments of Medicine, The University of Texas Health Science Center at Tyler, Tyler, Texas USA; 3 Departments of Biology, The University of Texas at Tyler, Tyler, Texas USA; 4 Departments of Epidemiology and Biostatistics, The University of Texas Health Science Center at Tyler, Tyler, Texas USA; 5 Departments of Biochemistry, Sri Sankara Arts and Science College, Kanchipuram, India; 6 Departments of Pathology, The University of Texas Health Science Center at Tyler, Tyler, Texas USA; 7 Departments of Obstetrics and Gynecology, CDP program, Mays Cancer Center, University of Texas Health at San Antonio, San Antonio, Texas USA; 8 Present Address: Fred Hutchinson Cancer Research Center, Seattle, Washington, USA

**Keywords:** Breast cancer, Molecular biology

## Abstract

Triple negative breast cancer (TNBC) has poor survival, exhibits rapid metastases, lacks targeted therapies and reliable prognostic markers. Here, we examined metastasis promoting role of cancer testis antigen *SPANXB1* in TNBC and its utility as a therapeutic target and prognostic biomarker. Expression pattern of *SPANXB1* was determined using matched primary cancer, lymph node metastatic tissues and circulating small extracellular vesicles (sEVs). cDNA microarray analysis of TNBC cells stably integrated with a metastasis suppressor *SH3GL2* identified SPANXB1 as a potential target gene. TNBC cells overexpressing SH3GL2 exhibited decreased levels of both *SPANXB1* mRNA and protein. Silencing of *SPANXB1* reduced migration, invasion and reactive oxygen species production of TNBC cells. S*PANXB1* depletion augmented *SH3GL2* expression and decreased RAC-1, FAK, A-Actinin and Vinculin expression. Phenotypic and molecular changes were reversed upon *SPANXB1* re-expression. *SPANXB1* overexpressing breast cancer cells with an enhanced *SPANXB1:SH3GL2* ratio achieved pulmonary metastasis within 5 weeks, whereas controls cells failed to do so. Altered expression of *SPANXB1* was detected in the sEVs of *SPANXB1* transduced cells. Exclusive expression of SPANXB1 was traceable in circulating sEVs, which was associated with TNBC progression. SPANXB1 represents a novel and ideal therapeutic target for blocking TNBC metastases due to its unique expression pattern and may function as an EV based prognostic marker to improve TNBC survival. Uniquely restricted expression of SPANXB1 in TNBCs, makes it an ideal candidate for targeted therapeutics and prognostication.

## Introduction

A significant number (10–20%) of breast cancers (BCa) do not express *ER, PR, HER2*, and are commonly referred to as triple negative breast cancer (TNBC)^1–4^. TNBCs are highly aggressive, exhibit metastases, lack targeted therapies, commonly occur in young women, more prevalent in African American and Hispanic women, and have a poor prognosis^1–10^. TNBCs are not responsive to approved drugs targeting ER/PR/Her2, however, few new drugs are being tested in clinical trials^10^. While chemotherapy and PARP inhibitors have clinical activity against TNBC in some women, they are not effective in the vast majority (>70%) of TNBC patients and only provide limited benefit on the patient survival^2,6,8,11–13^. Further, with lack of reliable biomarkers, prognostication of TNBC is challenging.

The genetic alterations behind TNBC development and progression are poorly understood^1–12^. Recent deep sequencing and molecular profiling studies identified considerable histological and molecular heterogeneity among TNBCs^14^ implicating involvement of multiple pathways driving cell migration, invasion and metastasis. In this cascade, the extracellular vesicles (EVs) play important role in promoting malignant transformation and metastasis and may serve as attractive tool for biomarker and therapeutic development^15–22^.

In the present study, we identified that cancer testis antigen (CTA) *SPANXB1* is a down stream target of metastases suppressor SH3GL2 (a.k.a. Endophilin A1). Depletion of SPANXB1 in TNBC models markedly reduced migration, invasion and reactive oxygen species production. Mechanistic studies revealed that *SPANXB1* modulate expression and functions of several proteins involved in metastases including RAC-1, FAK, A-Actinin and Vinculin. The TNBC cells with high SPANXB1 expression exhibited spontaneous pulmonary and liver metastasis by 5 weeks, whereas the SPANXB1 depleted cells with an increased SH3GL2:SPANXB1 expression ratio failed to achieve considerable pulmonary and liver metastasis. High SPANXB1 expression was detected in matched primary/metastatic tissues from TNBC patients and exclusively in their circulating sEVs. SPANXB1 expression was associated with TNBC progression.

## Results

### Cancer testis antigen SPANXB1 is a target of SH3GL2

**Figure 1:**
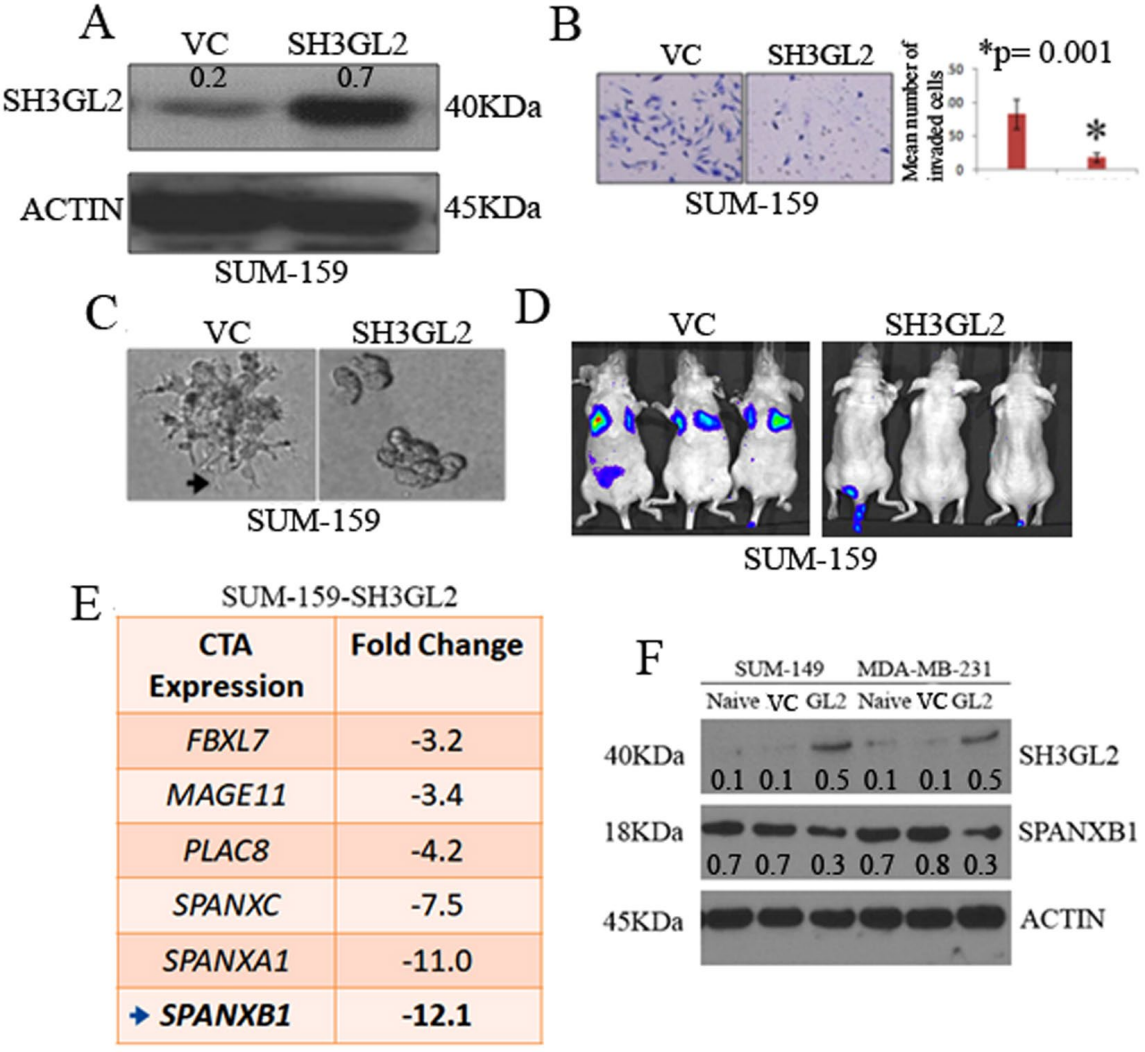
Introduction of SH3GL2 inhibited tumorigenic progression of BCa cells *in vitro and in vivo* and decreased SPANXB1 expression. SH3GL2 overexpression in SUM-159 cells (**A**) reduced invasion (**B**), filopodium like protrusion formation (**C**) and pulmonary metastasis (arrows) *in vivo* (**D**). (**E**) Microarray analysis of SH3GL2-overexpressing SUM-159 cells exhibited 12.1 fold decrease of SPANXB1 mRNA expression (arrow). (**F**) SH3GL2 overexpression reduced SPANXB1 expression in TNBC cells. VC: Empty vector control; SH3GL2: SH3GL2 transduced (**A**–**D**). VC: Empty vector control; GL2: SH3GL2 transduced (**F**). Magnification × 200 (**B**,**C**). In Western blotting, all experimental and control antibodies were run in parallel for the same immunoblot. The Image J software (https://imagej.nih.gov/ij/) was used for Western blot quantification.

SH3GL2 (a.k.a. Endophilin A1), located to human chromosome 9p22, that function as a potential tumor suppressor in human cancer^23–26^. We recently identified a metastasis suppressor role of SH3GL2 in BCa cells including TNBCs^27^. To identify the downstream targets of SH3GL2, we stably overexpressed SH3GL2 in SUM-159 cells (Fig. 1A). Overexpression of SH3GL2 markedly reduced invasion (p = 0.001) and lamelopodia like protrusion formation in 3D culture of the SUM-159 cells (Fig. 1A–C). To assess metastatic capability of the SH3GL2 overexpressing SUM-159 cells *in vivo*, we performed tail vein experimental metastasis assay. The SH3GL2 overexpressing SUM-159 cells failed to achieve pulmonary metastasis by 5 weeks, whereas the model cells expressing empty vector cells achieved extensive pulmonary metastasis (Fig. 1D). A cDNA microarray analysis of the SH3GL2 overexpressing SUM-159 cells identified decreased mRNA expression of a family of cancer testis antigens (CTAs) (Fig. 1E). Among these CTAs, notably SPANXB1a exhibited a 12.1 fold decreased mRNA expression (Fig. 1E, Table S1). To determine whether augmented SH3GL2 expression diminishes SPANXB1 expression at the protein level, we stably overexpressed SH3GL2 in additional TNBC models (Fig. 1F). These studies also confirmed a decreased expression of SPANXB1 protein in the TNBC cells stably overexpressing SH3GL2 (Fig. 1F). Collectively, these studies suggest SPANXB1 is a downstream target of SH3GL2 mediated inhibition of metastatic progression *in vitro and in vivo*.

### SPANXB1 depletion prevents TNBC progression through augmented SH3GL2 expression

**Figure 2:**
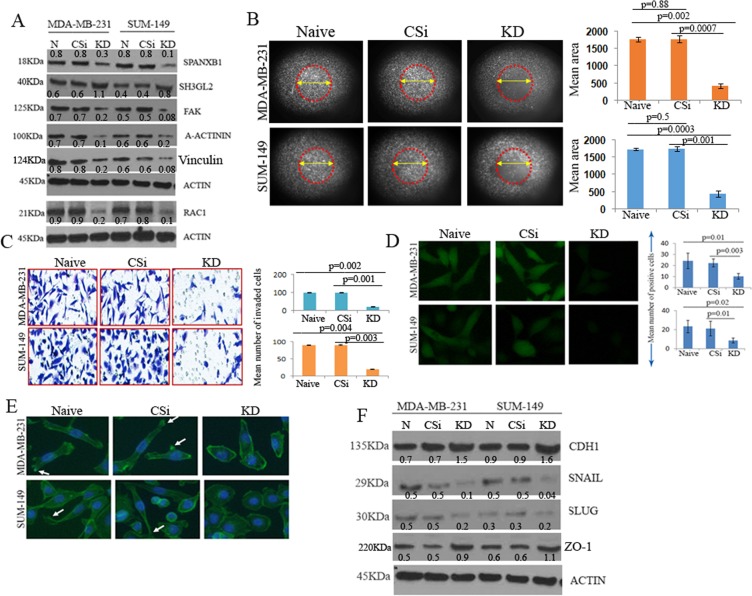
Depletion of SPANXB1 promoted progression of TNBC cells *in vitro*. Silencing of SPANXB1 (**A**) reduced migration ((**B**) low distribution of migrated cells in encircled area), invasion (**C**) and ROS production (**D**) by TNBC cells accompanied by enhanced expression of SH3GL2 and decreased expression of FAK, A-Actinin, Vinculin and RAC-1 (**A**). Mesenchymal to epithelial transition of SPANXB1 depleted TNBC cells ((**E**) arrows) accompanied by augmented expression of CDH1 and ZO-1 and decreased expression of Snail and Slug in TNBC lines (**F**). CSi: Control SiRNA; KD: SPANXB1-SiRNA treated. Actin was used as a loading control (**A**,**F**). Magnification X 200 (**B**–**E**). In Western blotting, all experimental and control antibodies were run in parallel for the same immunoblot. The Image J software (https://imagej.nih.gov/ij/) was used for Western blot quantification and area measurement in the migration assays.

To understand SPANXB1 function in promoting tumorigenic progression, we stably knocked down (KD) *SPANXB1* in two TNBC models using lentiviral transduction system (SPANXB1 SiRNA pool # iV023476), (Fig. 2A). Naïve and control Si-RNA (#LVP015-G) treated cells were used as controls. Pooled clones were selected using puromycin selection. Appreciable depletion of SPANXB1 protein (Fig. 2A) and mRNA was observed (Fig. S1) in TNBC cells transduced with SPANXB1-trageted SiRNAs. Phenotypically, depletion of SPANXB1 markedly reduced migration, invasion (p = 0.001–0.004) and reactive oxygen species (ROS) production (p = 0.01–0.003) of the TNBC cells compared to controls (Fig. 2B–D). The reduction in tumorigenic phenotypes was accompanied by markedly reduced expression of RAC-1, alpha-Actinin, Vinculin and FAK, key promoters of epithelial cell migration, invasion and loss of cell polarity^11,28–35^. An appreciable decrease in RAC1 phosphorylation was also observed in the *SPANXB1* depleted MDA-MBA-231 cells in a time dependent manner (Fig. S2). Notably, in these cells, a concomitant increase in the expression of SH3GL2 was also observed (Fig. 2A). Further, a mesenchymal to epithelial transition phenotype of the *SPANXB1* deficient cells (Fig. 2E) accompanied by an increased expression of the epithelial markers- CDH1 and ZO-1 and decreased expression of CDH1 repressors Snail and Slug in TNBC lines (Fig. 2F).

### SPANXB1 promotes cell migration/invasion of breast epithelial cells and is detectable in the secreted sEVs

**Figure 3:**
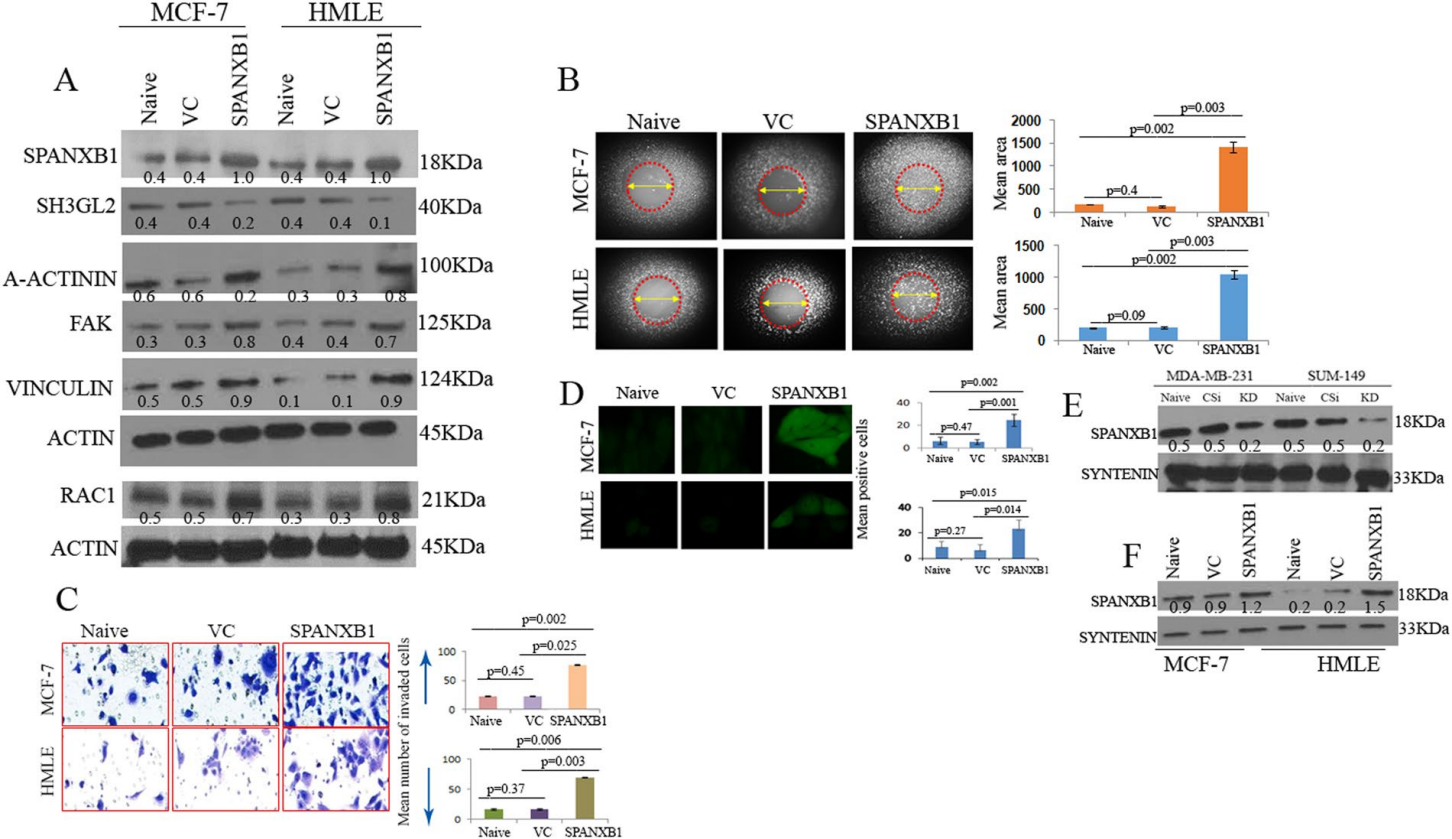
Rescued SPANXB1 expression facilitated tumorigenic progression of non-tumorigenic and tumorigenic mammary epithelial cell. Introduction of SPANXB1 (**A**) enhanced migration ((**B**) high distribution of migrated cells in encircled area), invasion (**C**) and ROS production (**D**) of the mammary epithelial cells accompanied by diminished expression of SH3GL2 and enhanced expression of A-Actinin, FAK, Vinculin and RAC-1 (**A**). (**E**) Reduced or augmented (**F**) expression of SPANXB1 in the secreted EVs from the transduced breast epithelial cell culture. Syntenin was used as an internal EV marker (**E**–**F**). Actin was used as a loading control (**A**). VC: Empty vector control; SPANXB1: SPANXB1 transduced. CSi: scramble SiRNA treated; KD: SPANXB1 targeted SiRNA treated. Magnification X 200 (**C**–**E**). In Western blotting, all experimental and control antibodies were run in parallel for the same immunoblot. The Image J software (https://imagej.nih.gov/ij/) was used for Western blot quantification and area measurement in the migration assays.

To determine the oncogenic potential of SPANXB1, we stably introduced wild type SPANXB1 in non-tumorigenic HMLE and tumorigenic non-metastatic MCF-7 cells (Fig. 3A). Appreciable increase in SPANXB1 mRNA was also observed in these cells following SPANXB1 overexpression (Fig. S3A). Forced overexpression of *SPANXB1* in these cells increased RAC1, Vinculin, FAK and alpha-actinin expression (Fig. 3A), accompanied by enhanced migration (Fig. 3B), invasion (Fig. 3C, p = 0.02–0.003) and ROS production (Fig. 3D, p = 0.01–0.002) compared to the control cells (Fig. 3B–E**)**.

We recently reported presence of cytoplasmic and mitochondrial proteins in circulating and cell secreted EVs^27,36^. To determine the expression level of SPANXB1 in the EVs, we performed Western blotting utilizing proteins isolated from the culture supernatant derived sEVs of the transduced mammary epithelial cells. Similar to SPANXB1 depleted cells, a decreased expression of SPANXB1 was detected in the cell secreted sEVs (Fig. 3E). On the other hand, following SPANXB1 overexpression, an enhanced expression of SPANXB1 was evident in the sEVs of SPANXB1-overexpressing cells (Fig. 3F). Thus, SPANXB1 appears to be a potential promoter of mammary epithelial cell migration/invasion and transported through the sEVs.

### Cross-regulation of SPANXB1 and SH3GL2 occur in breast cancer cells

**Figure 4:**
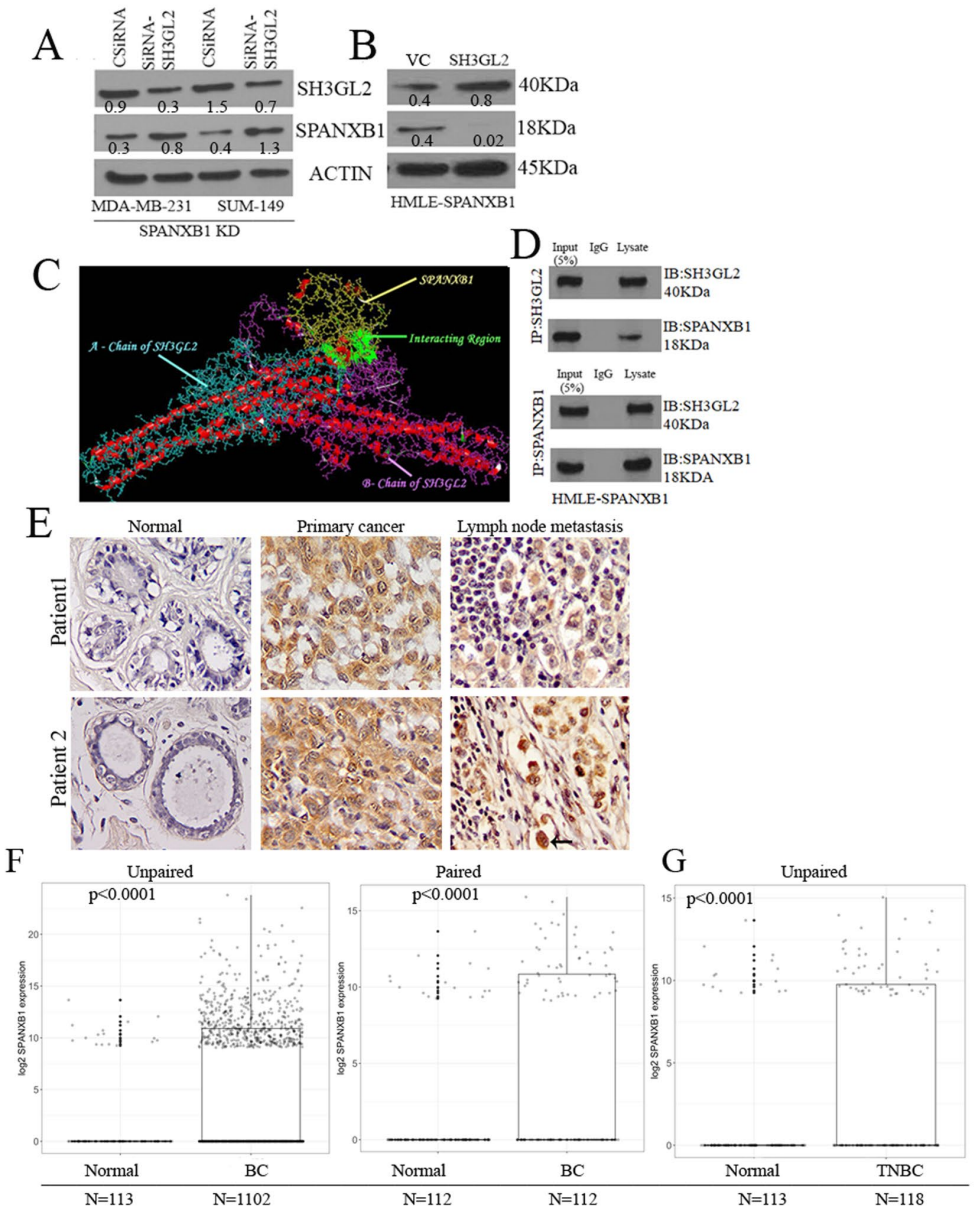
SH3GL2 mediates rescue or abrogation of SPANXB1 expression and are physically associated with SPANXB1. (**A**) Silencing or overexpression of SH3GL2 (**B**) rescue or diminishes SPANXB1 expression in SPANXB1-KD or overexpressing cells respectively. (**C**) Bioinformatics analysis predicted possible interaction sites (green areas) between SPANXB1 and SH3GL2. (**D**) Co-IP analysis pulled down SPANXB1 with SH3GL2 or vice versa in SPANXB1 overexpressing HMLE cells. (**E**) High nuclear (black arrow) and cytoplasmic (red arrow) expression of SPANXB1 in primary cancer and matched lymph node metastatic tissues compared to normal controls (p = 0.0002–0.0001). Magnification x 200. (**F**,**G**) High SPANXB1 mRNA expression in overall BCa (p < 0.0001, (**F**)) and TNBC tissues (p < 0.0001, (**G**)) compared to normal in TCGA datasets, with distribution of data points plotted on top of box plots. Actin was used as a loading control. CSiRNA: Control SIRNA; SiRNA-SH3GL2: SH3GL2 targeted SiRNA pool. EV: Empty vector treated; SH3GL2: SH3GL2 transduced. In Western blotting, all experimental and control antibodies were run in parallel for the same immunoblot. The Image J software (https://imagej.nih.gov/ij/) was used for Western blot quantification.

We observed an augmented SH3GL2 expression in SPANXB1 depleted BCa cells (Fig. 2A), which was reversed following SPANXB1 introduction in the non-tumorigenic and tumorigenic mammary epithelial cells (Fig. 3A). To further confirm these findings and ascertain a possible physical association between SPANXB1 and SH3GL2, we transiently knocked down SH3GL2 expression using SH3GL2 targeted SiRNAs in SPANXB1-depleted BCa cells exhibiting augmented SH3GL2 expression (Fig. 4A). The KD of SH3GL2 rescued SPANXB1 expression in these cells at protein (Fig. 4A)and mRNA level (Fig. S3B). On the other hand, overexpression of SH3GL2 in the SPANXB1 overexpressing HMLE cells diminished SPANXB1 expression (Fig. 4B).

In support to the above findings, our bioinformatics analyses predicted potential interaction between SH3GL2 and SPANXB1at various sites (Fig. 4C). To determine a physical association between SH3GL2 and SPANXB1, we performed Co-IP analysis utilizing lysates from SPANXB1-overexpressing HMLE cells. We could pull down SH3GL2 with SPANXB1 or vice versa in these cells (Fig. 4D). Similarly, we could also pull down SH3GL2 with SPANXB1 or vice versa in naïve MDA-MB-231 cells (Fig. S4). Thus, there appears to be inverse functional correlation between SH3GL2 and SPANXB1 in these cells.

### SPANXB1 is abundantly expressed in human primary and metastatic TNBC tissues

A comprehensive analysis of SPANXB1 expression pattern in TNBC progression is unknown. To assess the expression pattern of SPANXB1 in primary and metastatic TNBCs, we performed IHC analysis of paired normal/tumor and matched positive lymph node tissues (FFPE), from 15 TNBC subjects. We detected high nuclear as well as cytoplasmic *SPANXB1* expression (p = 0.0002–0.0001) in 73% (11/15) of the primary TNBC tissues and corresponding lymph node metastases compared to the matched normal tissues (Fig. 4E). Out of 11 high-TNBC expressing women, 3 were African American (AA) and 8 were Caucasian American (CA). SPANXB1 expression was negative in normal mammary epithelial tissues and surrounding stroma (Fig. S5).

To assess SPANXB1 mRNA expression pattern in overall BCa and TNBCs, we analyzed *SPANXB1* expression data (transcriptome) in BCa patients from the TCGA’s Breast Cancer project (TCGA-BRCA). We observed high *SPANXB1* expression in paired or unpaired analysis of overall BCa tissues (Fig. 4F–G, p < 0.0001). High *SPANXB1* expression was also evident in TNBCs compared to the normal tissues (Fig. 4G, p < 0.0001). 118 TNBC cases from the TCGA dataset further revealed an association (p = 0.004, Pearson’s correlation) between SH3GL2 and SPANXB1 co-expression, with linear regression suggesting an inverse correlation (Fig. S6A). Moreover, analysis of 115 out of these 118 cases revealed an association between increased SPANXB1 expression and progressive clinical stages (p = 0.02, Fig. S6B).

### SPANXB1 depletion reduces primary tumor growth and spontaneous metastasis of TNBC

**Figure 5:**
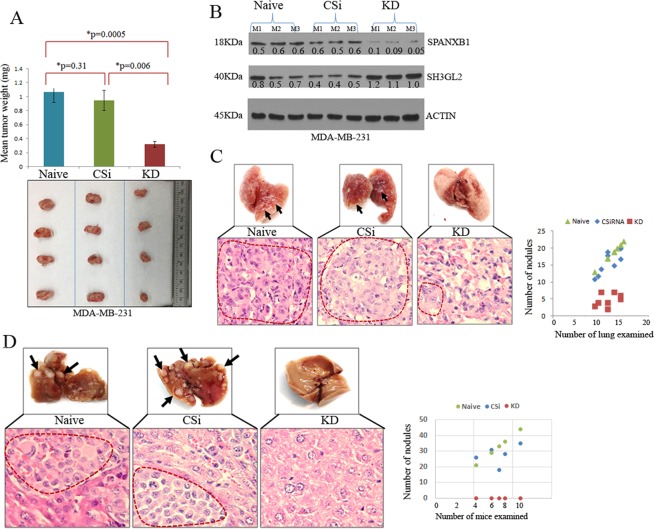
SPANXB1 depleted TNBC cells failed to achieve robust primary tumor growth and distant metastasis. (**A**) Markedly reduced (p = 0.0005–0.006) primary tumor growth of the SPANXB1-deficient MDA-MB-231 cells *in vivo* compared to the controls. (**B**) Low SPANXB1:SH3GL2 expression ratio in the SPANXB1-deficient primary TNBC tumors. (**C**) Reduced pulmonary metastasis of the SPANXB1-deficient TNBC cells compared to the controls. The number of metastatic lung nodules was lower in the SPANXB1-deficient mice group compared to the control groups. (**D**) Absence of visible or microscopic liver metastasis of the orthotopically implanted SPANXB1-deficient TNBC cells in the NSG mice. Control cells achieve extensive liver metastasis (arrows). CSi: Control SiRNA treated; KD: SPANXB1-SiRNA treated. Actin was used as a loading control (**B**). CSi: M: Mouse. CSi: Control SiRNA; KD: SPANXB1-SiRNA treated. Magnification X 200 (**C**). In Western blotting, all experimental and control antibodies were run in parallel for the same immunoblot. The Image J software (https://imagej.nih.gov/ij/) was used for Western blot quantification.

The SPANXB1 depleted TNBC cells demonstrated a reduced cell migration and invasion capabilities *in vitro* with increased expression ratio of SH3GL2:SPANXB1, which was reversed following its overexpression in mammary epithelial cells (Figs 2 and 3). To examine their behavior *in vivo*, we assessed primary tumor growth and spontaneous metastasis pattern of *SPANXB1*-KD MDA-MBA-231 cells by implanting them orthotopically in the mammary fat pad of female NSG mice^27^. The *SPANXB1*-KD cells exhibited reduced (p = 0.0005–0.006) primary tumor growth at the mammary fat pad compared to the control groups (Fig. 5A). An increased expression ratio of SH3GL2:SPANXB1 was evident in the primary tumor tissues obtained from the *SPANXB1*-KD mice (Fig. 5B). A remarkable inhibition in lung metastasis of the *SPANXB1*-KD cells was observed compared to controls (Fig. 5C). The number of tumor nodules was significantly higher in the control groups compared to theSPANXB1-KD group (Fig. 5C, p = 0.002–0.004). On the other hand, no visible liver metastasis was evident in the mice implanted withSPANXB1-KD cells, whereas control cells achieved extensive metastases in the liver in 5 weeks **(**Fig. 5D). Thus, SPANXB1 loss with rescued SH3GL2 expression in SPANXB1-KD cells not only prevents primary tumor growth but also their progression to metastasis.

### Rescue of *SPANXB1* expression promotes primary tumor growth and spontaneous pulmonary metastasis of BCa cells

**Figure 6:**
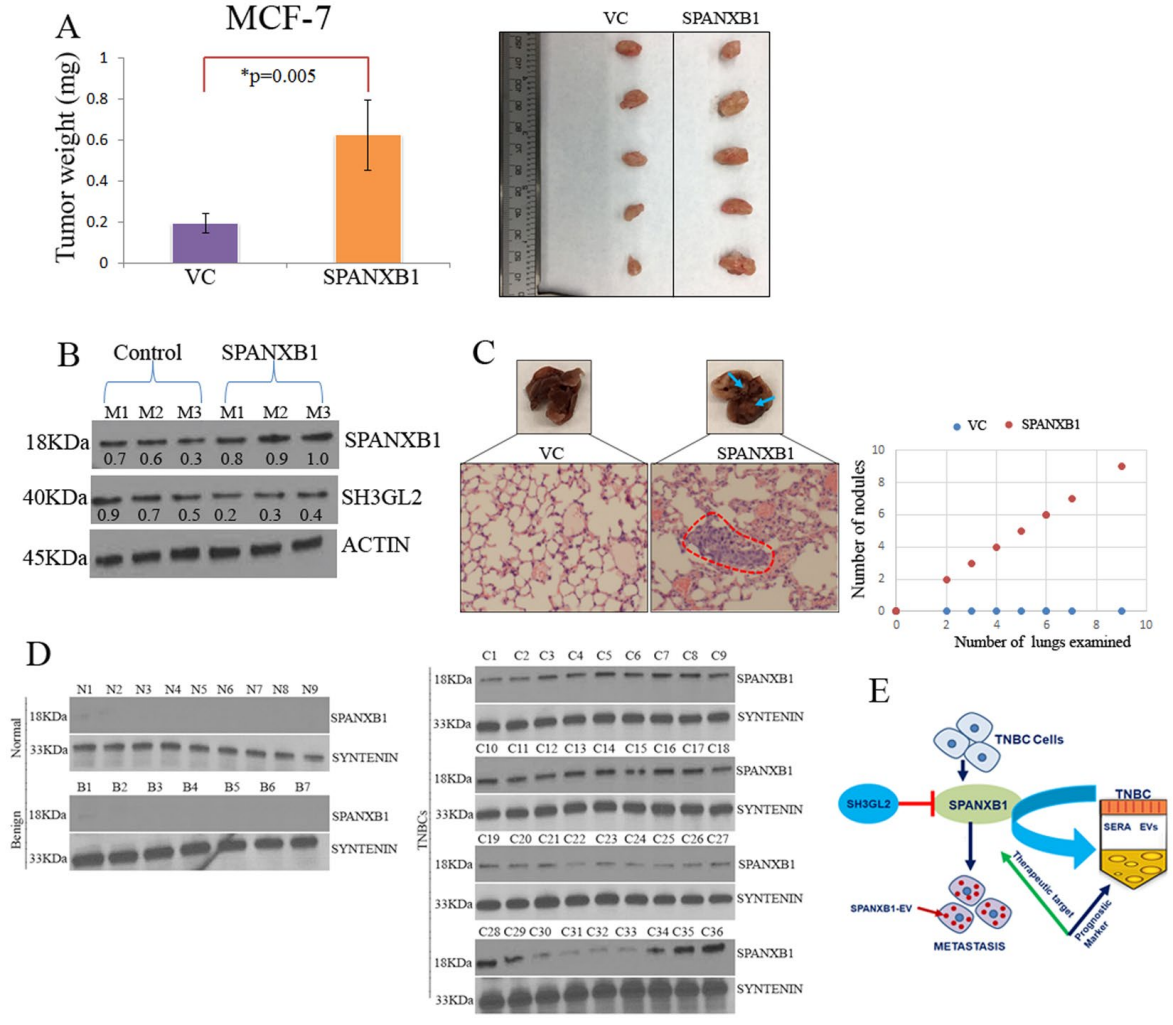
SPANXB1 overexpression facilitated rapid tumor growth and pulmonary metastasis and detectable in the circulating sEVs of TNBC patients. (**A**) Enhanced primary tumor growth of the orthotopically implanted SPANXB1-overexpressing MCF-7 cells accompanied by a high SPANXB1:SH3GL2 expression ratio (**B**). (**C**) Visible and microscopic pulmonary metastasis of the SPANXB1-overexpressing MCF-7 cells evident in 5 weeks compared to control. Actin was used as a loading control (**B**). VC: Empty vector transduced; SPANXB1: SPANXB1 transduced. M: Mouse. (**D**) SPANXB1 detection in the circulating sEVs of the TNBC patients (N = 36) only, which was absent in normal (N = 9) and benign (N = 7) subjects. Syntenin was used as an internal EV marker. N: Normal sera; B: Benign sera; C: Cancer sera. E, Schematic representation of SPANXB1 mediated promotion of TNBC metastasis, opposed by tumor suppressor SH3GL2 and its utility as a therapeutic target and extracellular vesicle (EV) based noninvasive marker for TNBC prognostication. In Western blotting, all experimental and control antibodies were run in parallel for the same immunoblot. The Image J software (https://imagej.nih.gov/ij/) was used for Western blot quantification.

To further confirm metastasis promoting role of SPANXB1 *in vivo*, we utilized MCF-7 model system overexpressing SPANXB1. These cells exhibited increased migration and invasion *in vitro* (Fig. 3). Parental MCF-7 cells form slow growing tumors and occasionally achieve distant metastasis by 25–26 weeks in NSG mice^37^ and thus a suitable model system to study the role of SPANXB1 in promoting metastasis. We orthotopically implanted MCF-7 cells overexpressing SPANXB1 in the mammary fat pad as described above. The SPANXB1 overexpressing MCF-7 cells achieved higher primary tumor growth in 5 weeks compared to the controls (Fig. 6A). The SPANXB1-overexpressing primary tumor tissues exhibited a highSPANXB1:SH3GL2expression ratio as opposed to the SPANXB1 depleted TNBC cells (Fig. 6B). Remarkably, the SPANXB1 overexpressing MCF-7 cells achieved faster pulmonary metastasis in 5 weeks, whereas the control cells failed to achieve lung metastasis by that time point (Fig. 6C). A number of metastatic tumor foci were noted in the lung of the mice implanted with SPANXB1 overexpressing MCF-7 cells but no microscopic lesions were evident in the mice implanted with control MCF-7 cells (Fig. 6C). At this time point, however, no visible or microscopic metastases were evident in the liver of the control or treated group of mice.

### SPANXB1 expression was exclusive in the circulating sEVs of TNBC or ER positive BCa patients

Our preliminary studies identified increased or reduced *SPANXB1* expression in the sEVs secreted by the mammary epithelial cells overexpressing or depleted of SPANXB1 (Fig. 3E,F), confirming the presence of SPANXB1in the sEVs. We further evaluated *SPANXB1* expression in the circulating sEVs from TNBC patients (N = 36) by Western blotting. High to moderate expression of *SPANXB1* was detected in 72% (26/36) TNBC cases (Fig. 6D). A low expression was detected in 28% (10/36, C22-C27; C30-C33, Fig. 6D, Table S2) cases. Nine out of the thirty six women (25%) were AA and exhibited high SPANXB1 expression in the circulating small EVs. However, we did not detect *SPANXB1* expression in the circulating sEVs isolated from healthy women (N = 9) or women with benign breast disease (N = 7). High SPANXB1 expression was significantly associated with advanced clinical stages and histological grades (p < 0.001, Fisher’s exact test, Table S2). We also assessed SPANXB1 expression in sera sEVs obtained from 23 estrogen receptor positive BCa subjects. We detected high to moderate SPANXB1 expression in 61% (14/23) of these cases (Fig. S6D). A low expression was evident in the rest of the 9 cases. Thus, SPANXB1 protein is detectable in the circulating sEVs of TNBCs (N = 36)and may be associated with disease progression in these cases.

## Discussion

Metastatic TNBC is an aggressive disease with limited treatment options and difficult to monitor due to lack of suitable biomarkers^1–12^. Characterization of the key metastasis promoting pathways is critical for better therapeutic targeting and prognostication of TNBCs. We identified a CTA-SPANXB1 as an oncogenic promoter of TNBC progression to metastasis *in vitro* and *in vivo*. A recent study demonstrated an invasion promoting role of SPANX family members A, C and D in BCa cells, reflecting the importance of these CTAs in driving BCa metastasis^38^. However, the expression pattern and role of SPANXB1 in TNBC or BCa progression remains elusive. The CTA-SPANXB1 could be an attractive therapeutic target because of its restricted expression in sperm cells and thus “off-target” effect of targeting this CTA will be less likely, which is a major problem with current chemo- and radiation- therapies. Accordingly, in melanomas, CTAs have been exploited and tailored for therapeutic as well as biomarker development^39^.

The EVs are gaining much attention in this era of cancer research because of their pivotal role in human tumorigenesis and promise for therapeutic and biomarker development^15–22^. Recent studies showed that EV proteins and miRNAs can serve as potential biomarkers for BCa^40,41^. However, there is clear knowledge gap on the role of EVs enriched with specific factors in promoting TNBC metastasis, which can be exploited for the development of EV based noninvasive biomarkers to benefit TNBC prognostication. In this light, detection of the CTA-SPANXB1 exclusively in the sEVs of BCa subjects and its association with TNBC progression could be promising to develop EV biomarker for TNBC prognostication. In addition, CTA-SPANXB1 may also serve as an EV based marker for ER^+^ BCa. Similarly, results from TCGAanalyses in TNBCs further support the potential diagnostic and prognostic values of SPANXB1. A comprehensive analysis using a large cohort of samples from various stages would be the necessary next step. On the other hand, detection of altered expression of CTA-SPANXB1 in the secreted sEVs of the transduced TNBC cells suggest for its potential role in promoting metastasis through the circulating EVs.

Mechanistically, oncogenic CTA-SPANXB1 augmented TNBC migration and invasion possibly through RAC-1/FAK/A-Actinin, key promoters of epithelial cell migration and metastasis and ROS production^28–35^. ROS is a well-known promoter of cancer cell migration and invasion in association with RAC-1^42^. Thus, high ROS production could potentially be associated with metastatic progression in concert with SPANXB1 mediated signaling. We observed aberrant SPANXB1 expression at various cellular compartments in TNBCs, which may aid in cell motility and migration in concert with other oncogenic molecules including RAC1. To our knowledge, there are no studies to date examining SPANXB1 expression in various cellular compartments and the circulating sEVs in the context of its differential activities. However, divergent activities of SPANXB1 could be possible in the light of its expression in the nucleus, cytoplasm and sEVs as observed in the present study.

The SPANXB1 mediated action was inhibited by a candidate tumor suppressor SH3GL2^27^, as restoration of this TSG prevented SPANXB1 driven promotion of metastasis and vice versa. As demonstrated in this study, a physical association between SPANXB1 and SH3GL2 and the SPANXB1:SH3GL2 expression ratio appeared to be the critical determinant of the metastatic fate of the TNBC cells. SH3GL2 also appeared to control SPANXB1 expression at transcriptional level and might possibly act as a blockade for SPANXB1 transactivation. In this regard, identification and characterizing the TSGs that opposes specific oncogenes’ function is of paramount importance for therapeutics development as restoration of their normal expression and function can suppress cancer growth and progression. TP53 is the most frequently altered TSG in BCa and various solid cancers and clinical trials are ongoing using small molecules that can reactivate normal p53 function^43^. As SH3GL2 loss appear to be associated with progression of various cancer types^23–27^, restoration of its normal function may prevent the oncogenic activity of CTA-SPANXB1 and many other oncogenic promoters. At the same time, monitoring the expression pattern of SPANXB1 in cancer tissues and sEVs may serve as a useful biomarker for disease monitoring and surveillance. A single oncogenic factor alone or in combination with others such as ER, PR and HER2/Neu tremendously improved targeted therapy and surveillance of specific subtypes of BCa^44^.

In summary, the results from this study identified a novel metastasis promoting pathway associated with a CTA-SPANXB1 in TNBC (Fig. 6E). High SPANXB1 expression was detectable in TNBC progression and the circulating sEVs. The CTA-SPANXB1 may serve as novel therapeutic target and prognostic biomarker for TNBC (Fig. 6E).

## Methods

### Human tissues samples and ethical statement

Archived formalin fixed and paraffin embedded (FFPE) breast tissues and sera were collected at The University of Texas Health Science Center at Tyler under an IRB approved protocol (#959). Informed consent of the all the subjects were obtained. All patients were de-identified and only relevant clinical information such as age, grade, stage, diagnosis etc. was collected for statistical analyses. All methods were performed in accordance with the relevant guidelines and regulations. All the experimental protocols were approved by The UT Health Science Center at Tyler.

### Microarray analysis

We used GeneChip Human Genome U133A 2.0 Array (Affymetirx) for the microarray analyses as described earlier^45^. Total RNA was extracted from 1 × 10^7^ SUM-159 cells stably transfected with empty vector or SH3GL2^45^ (in triplicates). Cells were re-suspended in 300 μL of Trizol and total RNA was isolated using the MagMAX™-96 for Microarrays Total RNA Isolation Kit (InvitrogenTM Life Technologies, Carlsbad, CA). Microarray analysis was performed in triplicate^45^. Hybridization was done in Affymetrix hybridization oven followed by washing and scanning in Fluidic station 450 and Genechip Scanner respectively utilizing GCOS software. Data interpretation and analysis was done through GCOS manager software specifications. Increased or decreased expression of various genes demonstrated through the microarray analysis was presented in Table S1. All data were submitted to Gene Expression Omnibus Data Base (GEO; #GSE110332).

### Cell lines and culture

Authenticated MDA-MB-231, MCF-7, SUM-149, SUM-159 cells were purchased from ATCC and other suitable vendors and cultured as directed. HMLE cell line was kindly provided by Dr. Guojun Wu, Wayne State University. All cell lines were periodically checked for Mycoplasma contamination using a Mycoplasma detection kit (Sigma # MP-0025)^27^. All tissue culture media and reagents were purchased either from ATCC or Invitrogen.

### Antibodies and reagents

SPANXB1 (#H00728695) and SH3GL2 (#H00006456) antibodies were purchased from Abnova. The CDH1 (#3195P), RAC-1 (#2465), FAK (#3285), Alpha-ACTININ (#6487), Snail (#3879), Slug (#9585), ZO-1 (#8193), Vinculin (#13901) and GFP (#2956) antibodies were purchased from Cell Signaling. F-actin antibody (#A12380) was obtained from Invitrogen. Anti-mouse **(**#115-035-003) and rabbit (#111-035-003) secondary antibodies were obtained from Jackson Immunoresearch. DAPI (#62248) and Phalloidin (#A12379) were purchased from ThemoFisher.

### 3D (three-dimensional) culture

Eight chambered glass slides (Nunc, Rochester, NY) were pre-coated with 3D Culture Matrix™ Basement Membrane Extract Reduced Growth Factor (Phenol Red-free) (Trevigen). Each well of the 8-chambered slide was seeded with 5000 cells per well in complete medium containing 2% 3D Matrix and the media was changed every 3 days. The slides were incubated in a standard tissue culture incubator

### Stable silencing of SPANXB1 in BCa cells

In the knockdown studies, MDA-MB-231 and SUM-149 cells were transduced with a GFP-tagged lentivirus SPANXB1-SiRNA pool (# iV023476, Abmgood). The same lentivirus construct harboring scrambled siRNA was used as a control (#LVP015-G, Abmgood). Stable clones were selected in the presence of puromycin (1ug/ml). Pooled stable clones was expanded and utilized for all subsequent analyses^27^. Naïve cells was used as another control as described earlier^27^.

### Overexpression of SPANXB1 in breast epithelial cells

MCF-7 and HMLE cells were transduced with a RFP-tagged lentivirus construct encoding SPANXB1 (#LVP792912, AbmGood). A RFP-tagged empty lentivirus construct with the same backbone (#LVP691) was used as a control. Stable clones were selected in the presence of puromycin (10ug/ml). Pooled stable clones were expanded and utilized for all subsequent analyses^27^. Naïve control cells were used to examine the influence of the empty vector on SPANXB1 expression^27^.

### SH3GL2 depletion and overexpression studies

In the knockdown experiment, SPANXB1 depleted TNBC cells were transduced with lentivirus SH3GL2-SiRNA pool (#iV022230, ABMGood). The same lentivirus construct harboring scrambled siRNA was used as a control (#LVP015, ABMgood). Pooled clones were selected in the presence of puromycin (1 μg/mL) and protein was isolated for downstream analysis. On the other hand, for SH3GL2 overexpression experiment, SPANXB1 overexpressing HMLE cells were transduced with lentivirus construct encoding wild type SH3GL2 (#LVP303171, ABMgood). An empty lentivirus construct with the same backbone (#LVP590) was used as a control. Pooled clones were selected in the presence of puromycin (100 ng/mL) and protein was isolated for downstream analysis.

### Cell migration, invasion and EMT assays

Cell migration assay (triplicate wells) was performed using the commercially available Oris™ Cell Migration assay kit and protocol (#CMART1.101, Platypus Technologies). The Image J software was used for measurement of the migrated areas^46^. Cell invasion (in triplicate wells) was assessed using the Cell Invasion Assay Kit (# 354481, Corning) as described^27^. The EMT assay was performed as described earlier^27^. Briefly, the transduced cells were stained with Fluorescein-Phalloidin and DAPI followed by washing with PBS and immediately observed under a confocal microscope^27^. In all cases, data were presented as mean ± SE of duplicate experiments.

### ROS and Bioinformatics analysis

Production of reactive oxygen species (ROS) by the transduced cells was determined by fluorescence microscopic analysis using commercially available DCFH-DA assay kit and protocol (#ab113851, Abcam)^47^. The transduced cells were stained with DCFH-DA (1µmol), washed with PBS and visualized immediately under a fluorescent microscope. At least 10 fields were randomly selected to count the ROS positive and negative cells^47^. The bioinformatics analysis was carried out using the FireDock server to predict interaction sites between SH3GL2 and SPANXB1 (http://bioinfo3d.cs.tau.ac.il/FireDock/).

### Western blotting and Co-Immunoprecipitation analysis

Preparation of whole cell lysates, Western blotting and co-immunoprecipitation were performed following protocols described earlier^27,47^. We utilized Phos-tag^TM^ gels (#195-17991, Wako Pure Chemicals) and manufacturer’s protocols to assess RAC-1 phosphorylation by Western blotting. In Western blotting, all experimental and appropriate control antibodies were run in parallel for the same immunoblot. The Image J software was used for Western blot quantification^46^.

### Immunohistochemistry and immunofluorescence analyses

Immunohistochemistry was performed using specific antibodies and conditions in paired TNBCs as described^27,48^. For comparison, all sections were processed in parallel. We used 1:50 dilution of primary antibodies and 1:250 for secondary antibodies in these analyses. At least 10-fields were randomly selected for examining the staining intensity and the distribution pattern of the proteins^27,48^. The immunofluorescence analysis was performed as described earlier^27^. The SPANXB1-KD TNBC cells were stained with Phalloidin and DAPI for EMT analysis^27^.

### Q-RT-PCR analysis

Total RNA was isolated using TriZol reagent. PCR was carried out in 10 µl reaction volume (in triplicate wells) using Bio-Rad iTaq Universal One-Step RT-qPCR kit and protocol (#1725150). SPANXB1 (#qHsaCEP0041842) and control GAPDH (#qHsaCEP0041396) primers were also obtained from Bio-Rad Inc. PCR was run in Bio-Rad’s CFX Connect system and data were analyzed using CFX manager software. GAPDH was used as control to normalized fold changes.

### Isolation of small extracellular vesicles and SPANXB1 detection

The sEVs were isolated from human sera or culture supernatant using commercially available kits and protocols with necessary modifications followed by protein isolation as described^15,27,36,49^. The transduced cells were cultured for 1 week in medium containing sEV depleted FBS (#EXO-FBS-50A-1, System Bioscience). After 1 week, the sEVs were isolated from these cell lines as described^27^. Western blotting was performed using 20 µg of total sEVs protein to detect SPANXB1 expression. Syntenin was used as an internal EV marker^50^.

### Cancer genome atlas data analysis

Data were collected from the National Cancer Institute’s Genomic Data Commons (https://portal.gdc.cancer.gov). All data were assembled from the TCGA’s Breast Cancer project (TCGA-BRCA), which includes 1098 total cases. Gene expression quantification was carried out from transcriptome profiling (RNAseq) and the results were harmonized to FPKM (Fragments PerKilobase Million) that have been upper-quartile normalized (HTSeq FPKM-UQ).

### Experimental metastasis studies with SH3GL2 overexpressing cells

The SUM-159 cells were stably transfected with wt-SH3GL2 plasmid in the presence of the FuGene 6 transfection regent as described earlier^45,48^. An empty vector with the same backbone was used as control. Stable clones were cultured in presence of 300 μg/ml of G418^27^. The SUM-159 cells stably overexpressing SH3GL2 were stably transfected with the luciferase (Luc) expressing plasmid pGL4.50 (Promega, Madison, WI) to facilitate the imaging of the xenografts. The empty vector transfected SUM-159 cells were also stably transfected with the luciferase expression plasmid in parallel for comparative imaging analysis. Luc-transfected control and SH3GL2 overexpressing SUM-159 cells (1 × 10^6^) were injected through the tail vein of female athymic nude mice (5 per group). The mice were monitored and Luc expression was assessed by bioluminescent imaging at week 5. All experiments were performed in accordance with the Animal Care and Use Committee guidelines.

### Orthotopic modeling of spontaneous metastasis with SPANXB1 depleted TNBC cells

For tumor growth and spontaneous metastasis analysis, 1 × 10^5^ control SiRNA and SPANXB1-SiRNA treated MDA-MB-231 cells in 1:1 mixture of PBS and matrigel were injected in the mammary fat pad of 4–6 week old, female NSG mice (Charles River)^27^. All experiments were performed in accordance with the Animal Care and Use Committee guidelines. Each group consisted of 5 mice. Mice were examined every day and mice showing any sign of morbidity were immediately sacrificed according to the University guidelines. All experiments were terminated at week 5 due to the tumor burden associated effects. After 5 weeks, mice were sacrificed and tumor weights were taken. Lungs and livers were removed for the analysis of metastasis. Focal tumor nodules were counted in the lung and liver of all the mice from various groups. Tumors were immediately snap frozen and also processed as formalin fixed paraffin embedded tissues (FFPE) for histological, Western blotting and IHC analyses respectively. All histopathological and IHC staining evaluations of the *in vivo* tumors were done per pathologic guidance^13,14^. Data presented as mean ± SE of duplicate experiments.

### Orthotopic animal modeling of metastasis with SPANXB1 overexpressing breast epithelial cells

For tumor growth, 1 × 10^5^ SPANXB1 and empty vector transduced MCF-7cells in 1:1 mixture of PBS and matrigel were injected in the mammary fat pad of 4–6 week old, female NSG mice (Charles River)^27^. All experiments were performed in accordance with the Animal Care and Use Committee guidelines. Each group consisted of 5 mice. Mice were examined every day and mice showing any sign of morbidity were immediately sacrificed according to the University guidelines. All experiments were terminated at week 5 due to the tumor burden associated effects. After 5 weeks, mice were sacrificed and tumor weights were taken. Lungs and livers were removed for the analysis of metastasis. Focal tumor nodules were counted in the lung and liver of all the mice from various groups. Tumors were immediately snap frozen and also processed as formalin fixed paraffin embedded tissues (FFPE) for histological, Western blotting and IHC analyses respectively. All histopathological evaluations of the *in vivo* tumors were done per pathologic guidance^27^. Data presented as mean ± SE of duplicate experiments.

### Statistical analysis

We employed Wilcoxin-Mann-Whitney, Anova, Chi-square or Fisher’s exact tests as appropriate for the statistical analysis. All p-values were two-sided and all confidence intervals were at the 95% level. Computation for all the analyses was performed using the Statistical Analysis System (SAS).

## Supplementary information

Supplemenraty data

## Data Availability

All data generated or analyzed during this study are included in this published article and its Supplementary Information files. The microarray data files can be accessed via Gene Expression Omnibus database.
