# Author Correction: Cancer Testis Antigen Promotes Triple Negative Breast Cancer Metastasis and is Traceable in the Circulating Extracellular Vesicles

**DOI:** 10.1038/s41598-020-58045-z

**Published:** 2020-01-31

**Authors:** Anbarasu Kannan, Julie V. Philley, Kate L. Hertweck, Harrison Ndetan, Karan P. Singh, Subramaniam Sivakumar, Robert B. Wells, Ratna K. Vadlamudi, Santanu Dasgupta

**Affiliations:** 1 Departments of Cellular and Molecular Biology, The University of Texas Health Science Center at Tyler, Tyler, Texas USA; 2 Departments of Medicine, The University of Texas Health Science Center at Tyler, Tyler, Texas USA; 3 Departments of Biology, The University of Texas at Tyler, Tyler, Texas USA; 4 Departments of Epidemiology and Biostatistics, The University of Texas Health Science Center at Tyler, Tyler, Texas USA; 5 Departments of Biochemistry, Sri Sankara Arts and Science College, Kanchipuram, India; 6 Departments of Pathology, The University of Texas Health Science Center at Tyler, Tyler, Texas USA; 7 Departments of Obstetrics and Gynecology, CDP program, Mays Cancer Center, University of Texas Health at San Antonio, San Antonio, Texas USA; 8 Present Address: Fred Hutchinson Cancer Research Center, Seattle, Washington USA

Correction to: *Scientific Reports* 10.1038/s41598-019-48064-w, published online 12 August 2019

The original version of this Article contained a typographical error in the spelling of the author Subramaniam Sivakumar, which was incorrectly given as Subramanium Sivakumar. This has now been corrected in the PDF and HTML versions of the Article, and in the accompanying Supplementary Dataset file.

