# Supplementary material for: Cancer Testis Antigen Promotes Triple Negative Breast Cancer Metastasis and is Traceable in the Circulating Extracellular Vesicles
Source: Sci Rep. 2019 Aug 12;9:11632. doi: 10.1038/s41598-019-48064-w (PMC6690992; doi:10.1038/s41598-019-48064-w)
Supplement: Supplementary file 1 — Supplemenraty data [file 41598_2019_48064_MOESM1_ESM.pdf]

Cancer Testis Antigen Promotes Triple Negative Breast Cancer Metastasis and is Traceable in the Circulating Extracellular Vesicles

Anbarasu Kannan[1*], Julie V Philley[2*], Kate L Hertweck[3], Harrison Ndetan[4], Karan P Singh[4], Subramanium Sivakumar[5], Robert B Wells[6], Ratna K Vadlamudi[7], Santanu Dasgupta[1,2***]**

Supplementary Figure 1

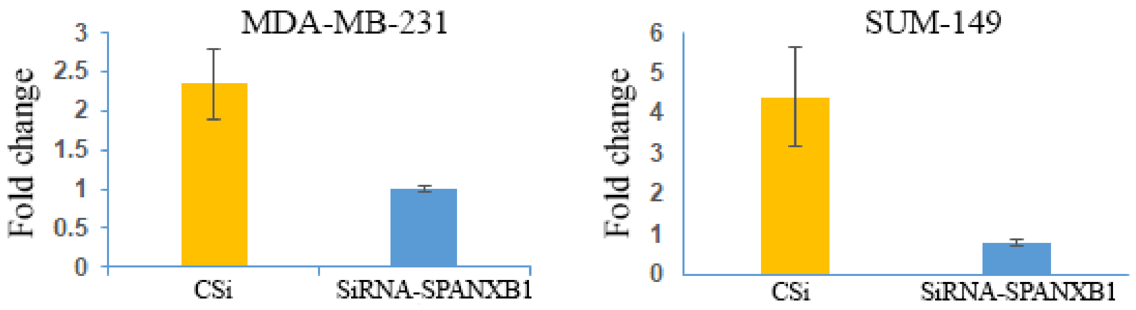

Supplementary Figure 1. SPANXB1 mRNA expression in SPANXB1-KD TNBC cells. Silencing of SPANXB1 markedly reduced SPANXB1 mRNA expression in the TNBC cells. CSi: Control SiRNA treated; SiRNA-SPANXB1: Transduced with SPANXB1 targeted SiRNA pool. Data was normalized to control GAPDH.

Supplementary Figure 2.

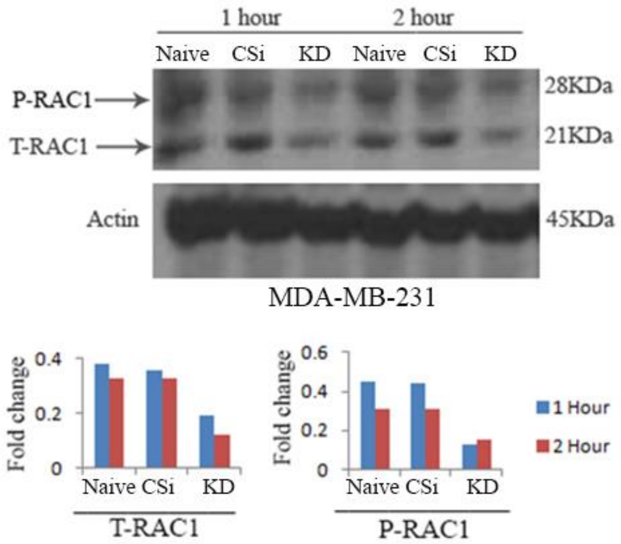

Supplementary Figure 2. SPANXB1 depletion decreased RAC-1 phosphorylation. Decreased RAC-1 phosphorylation in SPANXB-KD TNBC cells. CSi: Control SiRNA treated; KD: SPANXB1-SiRNA transduced. Actin was used as a loading control. Image J software was used to determine fold change with respect to the loading control.

Supplementary Figure 3.

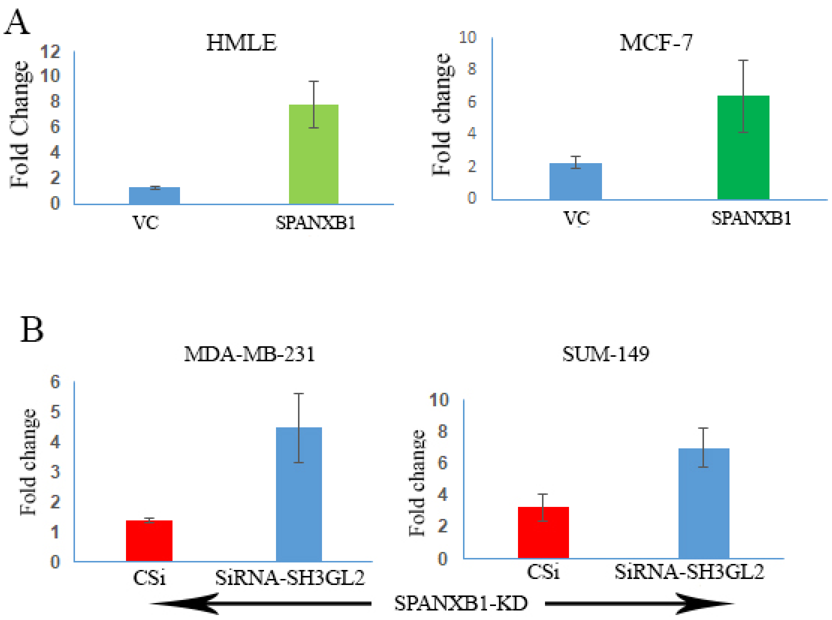

Supplementary Figure 3. SPANXB1 mRNA expression in SPANXB1 overexpressing and SH3GL2 depleted SPANXB1-KD TNBC cells. A, Forced overexpression of SPANXB1 markedly enhanced SPANXB1 mRNA expression in the TNBC cells. VC: empty vector control; SPANXB1: transduced with SPANXB1 lentiviral particles. Data was normalized to control GAPDH. **B,** Silencing of SH3GL2 using SH3GL2-targeted SiRNA pool (SiRNA-SH3GL2) in SPANXB1-KD TNBC cells reduced SPANXB1 mRNA expression. Data was normalized to control GAPDH. CSi: Control SiRNA treated.

Supplementary Figure 4.

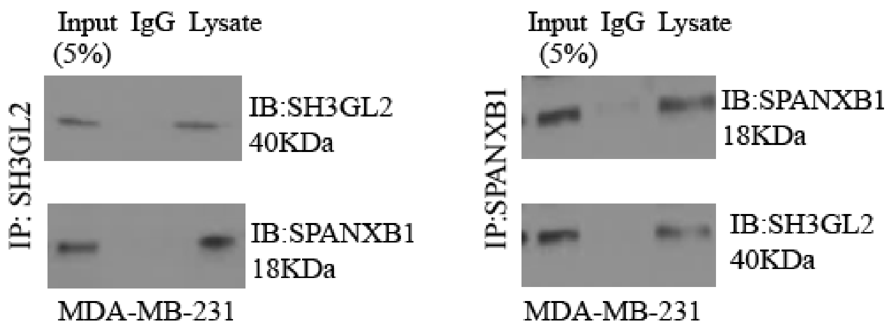

Supplementary Figure 4. SPANXB1 and SH3GL2 association in naïve TNBC cells. Co-IP analysis pulled down SPANXB1 with SH3GL2 or vice versa in naïve MDA-MB-231 cells.

Supplementary Figure 5.

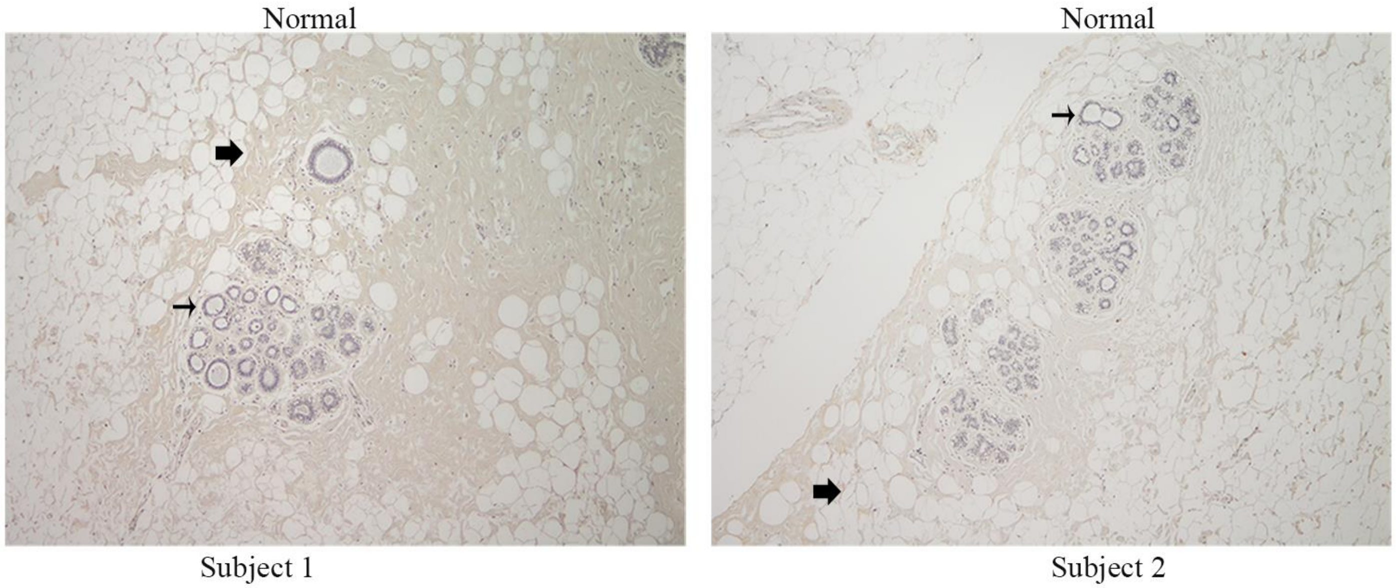

Supplementary Figure 5.SPANXB1 expression in normal mammary epithelium and stroma.

No detectable expression of SPANXB1 in normal mammary epithelial tissues (thin arrow) or surrounding stroma (thick arrow). Magnification x 100.

Supplementary Figure 6.

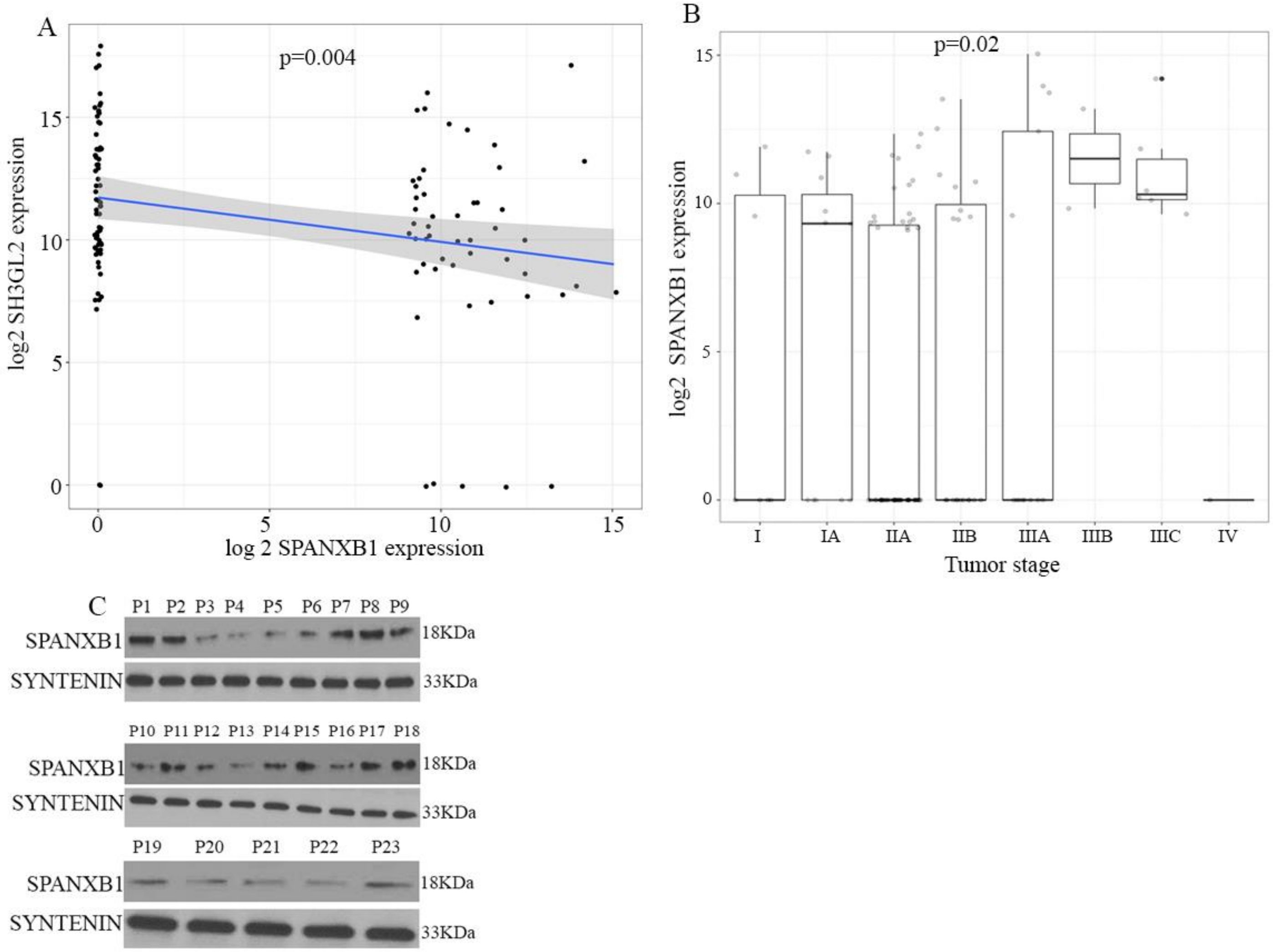

Supplementary Figure 6. SPANXB1 and SH3GL2 relationship in TNBC progression and SPANXB1 in sEVs from estrogen receptor positive BCa patients. A, Association between decreasing SH3GL2 and increasing SPANXB1 expression in 118 TNBC subjects (p=0.004, regression line in blue).Jittering was added to plot to better display distribution at 0, and confidence interval (standard error) associated with correlation (linear model, regression) is displayed. R2 = 0.06803, correlation coefficient (R) = -0.3765, 95% confidence interval -

0.6326829 to -0.1202308. **B**, Enhanced SPANXB1 expression across various stages of TNBC progression ($p=0.02$, N=120).Thelog2 of SPANXB1 expression for tumor stages I, IA, IIA, and IIB were grouped together (N=98) and compared to a second category of tumor stages IIIA, IIIB, IIIC, and IV (N=22) using a t-test. Each data point has also been plotted on top of the box and whisker plots. **C**, Detection of SPANXB1 protein in the circulating sEVs of the ER positive BCa patients (N=23). Syntenin was used as an internal EV marker.

Probe set	Gene Title	Gene Symbol	159 VECTOR	159 VECTOR	159 VECTOR	VECTOR Mean (Log2)
207739_s_at	G antigen 1 /// G a	GAGE1 /// GAGE12	5.71	5.99	6.04	**5.91**
1405_i_at	chemokine (C-C m	CCL5	6.07	5.80	6.23	**6.04**
206640_x_at	G antigen 12C ///	GAGE12C /// GAGI	6.17	5.76	6.04	**5.99**
208235_x_at	G antigen 12F ///	GAGE12F /// GAGI	6.51	6.43	6.21	**6.39**
204655_at	chemokine (C-C m	CCL5	6.77	6.67	6.60	**6.68**
208155_x_at	G antigen 1 /// G a	GAGE1 /// GAGE12	6.68	6.52	6.34	**6.51**
207086_x_at	G antigen 1 /// G a	GAGE1 /// GAGE12	6.86	6.73	6.65	**6.75**
207663_x_at	G antigen 3	GAGE3	6.39	6.48	6.39	**6.42**
203608_at	aldehyde dehydro	ALDH5A1	5.32	5.16	5.57	**5.35**
203153_at	interferon-induce	IFIT1	5.97	6.07	6.12	**6.05**
213478_at	kazrin	KAZ	6.77	6.72	6.68	**6.72**
202718_at	insulin-like growth	IGFBP2	7.16	7.35	7.21	**7.24**
202242_at	tetraspanin 7	TSPAN7	6.57	6.58	6.50	**6.55**
205073_at	cytochrome P450,	CYP2J2	6.75	6.53	6.50	**6.60**
211653_x_at	aldo-keto reducta	AKR1C2	11.58	11.63	11.51	**11.57**
219684_at	receptor (chemos	RTP4	5.11	5.13	5.11	**5.12**
203407_at	periplakin	PPL	7.57	7.59	7.61	**7.59**
200965_s_at	actin binding LIM	ABLIM1	7.84	7.99	8.03	**7.95**
203413_at	NEL-like 2 (chicke	NELL2	6.64	6.78	6.61	**6.68**
219991_at	solute carrier fam	SLC2A9	8.37	8.17	8.20	**8.25**
200606_at	desmoplakin	DSP	7.19	7.22	7.33	**7.25**
204990_s_at	integrin, beta 4	ITGB4	7.22	7.26	7.42	**7.30**
202458_at	protease, serine, 2	PRSS23	8.83	8.87	8.89	**8.86**
209784_s_at	jagged 2	JAG2	7.98	7.89	7.95	**7.94**
216841_s_at	superoxide dismu	SOD2	10.26	10.28	10.40	**10.32**
212813_at	junctional adhesic	JAM3	7.93	7.91	8.02	**7.96**
219911_s_at	solute carrier orga	SLCO4A1	9.41	9.39	9.36	**9.39**
221538_s_at	plexin A1	PLXNA1	8.67	8.74	8.73	**8.71**
202504_at	tripartite motif-co	TRIM29	7.57	7.58	7.64	**7.60**
208747_s_at	complement com	C1S	6.83	6.83	6.78	**6.81**
201487_at	cathepsin C	CTSC	10.34	10.29	10.33	**10.32**
214240_at	galanin prepropep	GAL	10.24	10.11	10.14	**10.16**
211366_x_at	caspase 1, apopto	CASP1	9.59	9.63	9.58	**9.60**
221840_at	protein tyrosine p	PTPRE	7.30	7.42	7.41	**7.38**
209970_x_at	caspase 1, apopto	CASP1	9.97	10.00	9.99	**9.99**
220942_x_at	family with seque	FAM162A	11.50	11.51	11.46	**11.49**
219577_s_at	ATP-binding casse	ABCA7	6.95	6.87	6.94	**6.92**
201494_at	prolylcarboxypept	PRCP	9.48	9.47	9.51	**9.49**
204467_s_at	synuclein, alpha (n	SNCA	7.20	7.25	7.24	**7.23**
207332_s_at	transferrin recept	TFRC	12.86	12.92	12.83	**12.87**
209167_at	glycoprotein M6B	GPM6B	10.03	9.92	9.99	**9.98**
214580_x_at	keratin 6A /// kera	KRT6A /// KRT6B /	7.09	7.09	7.05	**7.08**
209143_s_at	chloride channel,	CLNS1A	12.20	12.18	12.20	**12.19**
203621_at	NADH dehydroger	NDUFB5	11.64	11.62	11.62	**11.63**

210473_s_at	G protein-couplec	GPR125	9.17	9.16	9.20	**9.18**
217928_s_at	SAPS domain fami	SAPS3	8.97	9.03	9.03	**9.01**
203879_at	phosphoinositide-	PIK3CD	6.73	6.72	6.78	**6.74**
209570_s_at	DNA segment on c	D4S234E /// FOXP	5.86	5.84	5.82	**5.84**
201893_x_at	decorin	DCN	9.36	9.28	9.33	**9.32**
205433_at	butyrylcholinester	BCHE	5.43	5.37	5.38	**5.40**
214575_s_at	azurocidin 1	AZU1	4.12	4.09	4.13	**4.12**
208420_x_at	suppressor of Ty 6	SUPT6H	6.76	6.79	6.75	**6.77**
204735_at	phosphodiesteras	PDE4A	8.34	8.35	8.31	**8.33**
212886_at	coiled-coil domair	CCDC69	5.37	5.35	5.37	**5.36**
217411_s_at	ras responsive ele	RREB1	5.08	5.10	5.08	**5.09**
212537_x_at	ribosomal protein	RPL17	14.48	14.46	14.49	**14.48**
207939_x_at	RNA binding prote	RNPS1	9.24	9.23	9.26	**9.24**
214143_x_at	ribosomal protein	RPL24	14.83	14.82	14.82	**14.82**
208645_s_at	ribosomal protein	RPS14	14.81	14.81	14.81	**14.81**
214984_at	SMG1 homolog, p	LOC100271836 ///	3.72	3.72	3.69	**3.71**
200825_s_at	hypoxia up-regula	HYOU1	9.71	9.73	9.72	**9.72**
208737_at	ATPase, H+ transp	ATP6V1G1	11.97	11.97	11.96	**11.97**
204353_s_at	protection of telo	POT1	8.33	8.36	8.34	**8.34**
216651_s_at	glutamate decarb	GAD2	4.45	4.40	4.42	**4.42**
202821_s_at	aryl hydrocarbon	AHR	7.60	7.63	7.60	**7.61**
213815_x_at	chromosome 19 c	C19orf29	6.33	6.30	6.29	**6.31**
220161_s_at	erythrocyte meml	EPB41L4B	11.42	11.40	11.43	**11.41**
210987_x_at	tropomyosin 1 (al	TPM1	11.59	11.63	11.61	**11.61**
218784_s_at	chromosome 6 op	C6orf64	6.63	6.63	6.66	**6.64**
201206_s_at	ribosome binding	RRBP1	8.36	8.42	8.37	**8.38**
212022_s_at	antigen identified	MKI67	10.28	10.24	10.26	**10.26**
222136_x_at	zinc finger protein	ZNF43	4.11	4.17	4.19	**4.15**
211816_x_at	Fc fragment of IgA	FCAR	6.23	6.23	6.20	**6.22**
211976_at	---	---	9.36	9.40	9.35	**9.37**
200760_s_at	ADP-ribosylation-l	ARL6IP5	11.85	11.91	11.86	**11.88**
63825_at	abhydrolase doma	ABHD2	9.29	9.29	9.34	**9.30**
219410_at	transmembrane p	TMEM45A	11.16	11.18	11.14	**11.16**
217369_at	immunoglobulin h	IGHG1 /// LOC100	5.91	5.85	5.89	**5.88**
218292_s_at	protein kinase, AN	PRKAG2	8.95	8.96	8.96	**8.95**
203636_at	midline 1 (Opitz/E	MID1	8.56	8.56	8.56	**8.56**
219582_at	opioid growth fac	OGFRL1	8.27	8.31	8.35	**8.31**
205608_s_at	angiopoietin 1	ANGPT1	6.26	6.31	6.24	**6.27**
205066_s_at	ectonucleotide py	ENPP1	7.43	7.48	7.49	**7.47**
201453_x_at	Ras homolog enric	RHEB	12.86	12.87	12.88	**12.87**
219714_s_at	calcium channel, v	CACNA2D3	8.61	8.63	8.65	**8.63**
211026_s_at	monoglyceride lip	MGLL	9.86	9.85	9.93	**9.88**
202363_at	sparc/osteonectin	SPOCK1	9.49	9.46	9.47	**9.47**
216552_x_at	killer cell immuno	KIR2DS4	6.79	6.71	6.72	**6.74**
205079_s_at	multiple PDZ dom	MPDZ	7.32	7.42	7.30	**7.35**
213059_at	cAMP responsive	CREB3L1	9.09	9.08	9.07	**9.08**
208960_s_at	Kruppel-like facto	KLF6	9.38	9.38	9.47	**9.41**

208811_s_at	DnaJ (Hsp40) hom	DNAJB6 /// TMEM	9.89	9.94	9.93	**9.92**
201615_x_at	caldesmon 1	CALD1	9.43	9.46	9.55	**9.48**
202686_s_at	AXL receptor tyro	AXL	11.67	11.58	11.65	**11.63**
202628_s_at	serpin peptidase i	SERPINE1	11.79	11.91	11.87	**11.86**
221729_at	collagen, type V, a	COL5A2	9.42	9.48	9.44	**9.45**
220014_at	proline rich 16	PRR16	8.67	8.67	8.75	**8.70**
216452_at	transient receptor	TRPM3	4.94	4.96	4.93	**4.94**
209781_s_at	KH domain contai	KHDRBS3	8.91	8.90	8.88	**8.90**
216973_s_at	homeobox B7	HOXB7	7.48	7.47	7.60	**7.52**
201616_s_at	caldesmon 1	CALD1	9.71	9.72	9.80	**9.74**
206796_at	WNT1 inducible si	WISP1	7.80	7.80	7.91	**7.84**
209369_at	annexin A3	ANXA3	8.87	8.87	8.97	**8.90**
213479_at	neuronal pentraxi	NPTX2	11.32	11.32	11.20	**11.28**
214446_at	elongation factor,	ELL2	7.90	7.97	8.01	**7.96**
202555_s_at	myosin light chain	MYLK	8.07	8.00	7.99	**8.02**
211161_s_at	collagen, type III, a	COL3A1	3.98	3.95	3.89	**3.94**
212951_at	G protein-couplec	GPR116	10.19	10.26	10.20	**10.22**
209708_at	monooxygenase, I	MOXD1	6.92	6.94	7.03	**6.96**
221031_s_at	apolipoprotein L c	APOLD1	7.38	7.44	7.41	**7.41**
200953_s_at	cyclin D2	CCND2	7.86	7.84	7.94	**7.88**
209357_at	Cbp/p300-interac	CITED2	9.82	9.93	9.99	**9.91**
221730_at	collagen, type V, a	COL5A2	8.68	8.72	8.72	**8.71**
213436_at	cannabinoid recep	CNR1	10.64	10.71	10.73	**10.70**
219737_s_at	protocadherin 9	PCDH9	9.69	9.80	9.76	**9.75**
202668_at	ephrin-B2	EFNB2	9.43	9.58	9.62	**9.54**
206060_s_at	protein tyrosine p	PTPN22	6.79	6.91	6.89	**6.86**
221541_at	cysteine-rich secre	CRISPLD2	7.48	7.47	7.54	**7.50**
201107_s_at	thrombospondin 1	THBS1	7.23	7.13	7.25	**7.20**
202207_at	ADP-ribosylation f	ARL4C	8.48	8.41	8.61	**8.50**
208951_at	aldehyde dehydro	ALDH7A1	7.85	7.87	7.83	**7.85**
212488_at	collagen, type V, a	COL5A1	9.00	9.08	9.13	**9.07**
218332_at	brain expressed, X	BEX1	9.78	9.91	9.83	**9.84**
203874_s_at	SWI/SNF related,	SMARCA1	5.83	6.06	5.93	**5.94**
203186_s_at	S100 calcium bind	S100A4	10.79	11.02	10.84	**10.89**
201109_s_at	thrombospondin 1	THBS1	11.89	12.04	11.98	**11.97**
202206_at	ADP-ribosylation f	ARL4C	7.98	7.85	8.11	**7.98**
205990_s_at	wingless-type MM	WNT5A	9.87	9.86	9.99	**9.91**
214803_at	cadherin 6, type 2	CDH6	6.94	7.02	7.17	**7.04**
201108_s_at	thrombospondin 1	THBS1	9.80	9.86	9.79	**9.82**
213515_x_at	hemoglobin, gamı	HBG1 /// HBG2	8.46	8.35	8.52	**8.44**
203903_s_at	hephaestin	HEPH	6.88	6.80	7.00	**6.89**
213425_at	wingless-type MM	WNT5A	8.59	8.65	8.79	**8.68**
201438_at	collagen, type VI,	COL6A3	11.04	11.03	11.12	**11.06**
218545_at	coiled-coil domair	CCDC91	6.49	6.51	6.62	**6.54**
202465_at	procollagen C-enc	PCOLCE	8.67	8.68	8.72	**8.69**
218706_s_at	GRAM domain cor	GRAMD3	6.20	6.26	6.50	**6.32**
205919_at	hemoglobin, epsil	HBE1	10.30	10.17	10.32	**10.26**

213249_at	F-box and leucine	FBXL7	8.08	8.08	8.34	**8.16**
210503_at	melanoma antige	MAGEA11	5.84	5.68	5.98	**5.83**
219014_at	placenta-specific	PLAC8	8.80	8.89	8.89	**8.86**
220217_x_at	SPANX family, me	SPANXC	10.83	10.96	10.81	**10.87**
220922_s_at	sperm protein ass	SPANXA1 /// SPAN	12.59	12.72	12.57	**12.63**
220921_at	SPANX family, me	SPANXB1 /// SPAN	11.40	11.46	11.48	**11.45**
37020_at	C-reactive protein	CRP	3.72	3.70	3.73	
201427_s_at	selenoprotein P, p	SEPP1	5.15	5.16	5.42	
209278_s_at	tissue factor path	TFPI2	8.43	8.41	8.44	
204321_at	neogenin homolo	NEO1	6.69	6.60	6.58	
221480_at	heterogeneous nu	HNRNPD	9.79	9.76	9.74	
202141_s_at	COP9 constitutive	COPS8	11.88	11.94	11.93	
209459_s_at	4-aminobutyrate a	ABAT	6.36	6.47	6.53	
207466_at	galanin preprope	GAL	5.54	5.46	5.50	
203074_at	annexin A8 /// an	ANXA8 /// ANXA8I	10.22	10.34	10.21	
208178_x_at	triple functional d	TRIO	8.70	8.72	8.67	
217531_at	---	---	3.70	3.68	3.69	
208782_at	follistatin-like 1	FSTL1	11.24	11.34	11.33	
212608_s_at	---	---	9.82	9.81	9.90	
205412_at	acetyl-CoA acetylt	ACAT1	11.26	11.28	11.34	
201860_s_at	plasminogen activ	PLAT	9.71	9.74	9.90	
202142_at	COP9 constitutive	COPS8	10.28	10.32	10.36	
212489_at	collagen, type V, a	COL5A1	7.17	7.21	7.18	
213610_s_at	kelch-like 23 (Dro	KLHL23	8.71	8.73	8.79	
205016_at	transforming grov	TGFA	5.80	5.80	5.80	
201549_x_at	lysine (K)-specific	KDM5B	7.81	7.87	7.91	
201012_at	annexin A1	ANXA1	12.32	12.40	12.36	
214211_at	ferritin, heavy pol	FTH1	12.52	12.43	12.41	
219938_s_at	proline-serine-thr	PSTPIP2	6.15	6.22	6.26	
218567_x_at	dipeptidyl-peptida	DPP3	8.56	8.59	8.57	
209386_at	transmembrane 4	TM4SF1	10.64	10.80	10.70	
209344_at	tropomyosin 4	TPM4	8.37	8.31	8.36	
202508_s_at	synaptosomal-ass	SNAP25	4.56	4.53	4.71	
212510_at	glycerol-3-phosph	GPD1L	9.89	9.81	9.83	
213947_s_at	nucleoporin 210kI	NUP210	7.50	7.41	7.46	
212843_at	neural cell adhesi	NCAM1	6.72	6.37	6.34	
206218_at	melanoma antige	MAGEB2	4.37	4.44	4.47	
209170_s_at	glycoprotein M6B	GPM6B	9.70	9.72	9.77	
209460_at	4-aminobutyrate a	ABAT	7.09	7.04	6.92	
205405_at	sema domain, sev	SEMA5A	8.67	8.60	8.55	
220617_s_at	zinc finger protei	ZNF532	9.37	9.40	9.40	
215017_s_at	formin binding pr	FNBP1L	6.47	6.47	6.79	
200663_at	CD63 molecule	CD63	12.93	12.94	12.93	
214782_at	cortactin	CTTN	7.39	7.38	7.42	
206382_s_at	brain-derived neu	BDNF	5.94	5.77	5.92	
211980_at	collagen, type IV,	COL4A1	8.54	8.59	8.61	

202626_s_at	v-yes-1 Yamaguch	LYN	9.00	9.05	9.02
221622_s_at	transmembrane p	TMEM126B	11.64	11.74	11.68
218935_at	EH-domain contai	EHD3	7.61	7.71	7.56
204151_x_at	aldo-keto reducta	AKR1C1	12.52	12.56	12.46
202295_s_at	cathepsin H	CTSH	8.42	8.32	8.33
206953_s_at	latrophilin 2	LPHN2	8.44	8.47	8.46
209442_x_at	ankyrin 3, node of	ANK3	8.90	8.88	9.01
216594_x_at	aldo-keto reducta	AKR1C1	11.75	11.82	11.70
212850_s_at	low density lipopr	LRP4	6.24	6.44	6.31
204529_s_at	thymocyte selecti	TOX	7.84	7.83	7.92
202310_s_at	collagen, type I, al	COL1A1	8.46	8.30	8.53
209685_s_at	protein kinase C, I	PRKCB	5.58	5.58	5.55
201042_at	transglutaminase	TGM2	7.83	7.97	7.96
214431_at	guanine monphos	GMPS	11.08	11.04	11.01
204268_at	S100 calcium bind	S100A2	10.13	10.39	10.24
213954_at	family with seque	FAM169A	7.04	7.14	7.24
213412_at	tight junction prot	TJP3	5.86	5.77	5.83
209815_at	patched homolog	PTCH1	8.15	8.07	8.29
206648_at	zinc finger protein	ZNF571	4.21	4.29	4.32
207580_at	melanoma antigen	MAGEB4	3.71	3.66	3.66
213338_at	transmembrane p	TMEM158	10.21	10.22	10.18
205730_s_at	actin binding LIM	ABLIM3	10.27	10.21	10.32
201423_s_at	cullin 4A	CUL4A	10.02	10.03	10.03
210510_s_at	neuropilin 1	NRP1	8.69	8.62	8.68
212021_s_at	antigen identified	MKI67	9.18	9.26	9.20
202421_at	immunoglobulin s	IGSF3	7.45	7.56	7.42
215223_s_at	superoxide dismu	SOD2	12.32	12.43	12.35
206280_at	cadherin 18, type	CDH18	9.09	9.26	9.24
206385_s_at	ankyrin 3, node of	ANK3	9.67	9.52	9.75
203588_s_at	transcription facto	TFDP2	9.11	9.01	9.18
203453_at	sodium channel, n	SCNN1A	8.71	8.57	8.72
203037_s_at	metastasis suppre	MTSS1	10.36	10.32	10.42
212129_at	non imprinted in F	NIPA2	11.52	11.53	11.54
213484_at	---	---	7.57	7.48	7.47
219077_s_at	WW domain conta	WWOX	5.56	5.62	5.53
214074_s_at	cortactin	CTTN	8.65	8.59	8.59
202291_s_at	matrix Gla protein	MGP	9.11	9.01	9.14
209945_s_at	glycogen synthase	GSK3B	8.86	8.84	8.89
203123_s_at	solute carrier fam	SLC11A2	8.01	8.05	8.07
200013_at	ribosomal protein	RPL24	14.59	14.61	14.63
217503_at	serine/threonine I	STK17B	7.03	6.89	7.07
212747_at	ankyrin repeat and	ANKS1A	8.53	8.48	8.51
205937_at	cell growth regula	CGREF1	7.91	8.02	8.03
213397_x_at	ribonuclease, RNa	RNASE4	7.06	6.91	7.12
217374_x_at	TCR gamma altern	TARP /// TRGV3 //	4.83	4.89	4.96
213348_at	cyclin-dependent	CDKN1C	6.82	6.91	6.90
213293_s_at	tripartite motif-co	TRIM22	6.68	6.85	6.77

215545_at	---	---	5.21	5.27	5.19
212296_at	proteasome (pros	PSMD14	12.29	12.29	12.29
214359_s_at	heat shock protei	HSP90AB1	11.72	11.75	11.74
213901_x_at	RNA binding moti	RBM9	9.45	9.38	9.46
204043_at	transcobalamin II	TCN2	6.34	6.31	6.31
212226_s_at	phosphatidic acid	PPAP2B	9.98	9.91	9.86
213169_at	sema domain, sev	SEMA5A	8.83	8.90	8.86
207148_x_at	myozenin 2	MYOZ2	10.95	10.90	11.12
213162_at	Dynein, cytoplasm	DYNC1LI2	4.97	4.98	4.99
212206_s_at	H2A histone famil	H2AFV	9.90	9.99	10.03
201858_s_at	serglycin	SRGN	11.70	11.82	11.76
202007_at	nidogen 1	NID1	4.22	4.39	4.61
210976_s_at	phosphofructokin	PFKM	11.13	11.11	11.13
212298_at	neuropilin 1	NRP1	10.59	10.71	10.75
209699_x_at	aldo-keto reducta	AKR1C2	12.55	12.54	12.42
218298_s_at	chromosome 14 c	C14orf159	8.38	8.41	8.31
216028_at	---	---	8.85	8.91	8.92
212950_at	G protein-couplec	GPR116	11.00	11.13	11.22
201036_s_at	hydroxyacyl-CoA c	HADH	9.84	9.76	9.86
203263_s_at	Cdc42 guanine nu	ARHGEF9	7.15	7.16	7.20
205637_s_at	SH3-domain GRB2	SH3GL3	6.65	6.89	6.91
203056_s_at	PR domain contai	PRDM2	6.84	6.71	6.85
210416_s_at	CHK2 checkpoint I	CHEK2	9.63	9.57	9.65
222258_s_at	SH3-domain bindi	SH3BP4	9.91	9.87	10.01
212067_s_at	complement com	C1R	6.89	6.85	6.95
203705_s_at	frizzled homolog 7	FZD7	8.12	8.22	8.27
213943_at	twist homolog 1 (I	TWIST1	7.98	8.04	8.07
200893_at	transformer 2 bet	TRA2B	12.58	12.59	12.61
212614_at	AT rich interactive	ARID5B	9.69	9.66	9.84
208950_s_at	aldehyde dehydro	ALDH7A1	8.18	8.11	8.12
203488_at	latrophilin 1	LPHN1	7.47	7.38	7.42
209160_at	aldo-keto reducta	AKR1C3	11.26	11.31	11.27
209831_x_at	deoxyribonucleas	DNASE2	8.56	8.61	8.57
216825_s_at	myeloproliferative	MPL	4.10	4.08	4.09
211812_s_at	beta-1,3-N-acetyl	B3GALNT1	6.03	6.26	6.15
204924_at	toll-like receptor 2	TLR2	8.83	8.82	8.78
219271_at	UDP-N-acetyl-alph	GALNT14	9.81	9.84	9.88
222323_at	---	---	6.02	6.04	6.07
214212_x_at	fermitin family ho	FERMT2	9.88	9.84	9.90
201656_at	integrin, alpha 6	ITGA6	9.83	9.98	10.01
202957_at	hematopoietic cel	HCLS1	6.10	6.21	6.05
213782_s_at	myozenin 2	MYOZ2	10.69	10.73	10.89
201287_s_at	syndecan 1	SDC1	9.11	9.19	9.20
202385_s_at	Treacher Collins-F	TCOF1	8.71	8.70	8.75
206237_s_at	neuregulin 1	NRG1	7.53	7.33	7.52
203141_s_at	adaptor-related p	AP3B1	7.81	7.80	7.78
211621_at	androgen recepto	AR	5.49	5.40	5.36

218465_at	transmembrane p	TMEM33	10.04	10.02	10.11
218595_s_at	HEAT repeat conta	HEATR1	10.69	10.71	10.71
207063_at	non-protein codin	NCRNA00185	3.55	3.46	3.52
205505_at	glucosaminyl (N-a	GCNT1	6.89	7.26	7.09
208485_x_at	CASP8 and FADD-l	CFLAR	7.62	7.70	7.62
219987_at	Similar to Unchara	LOC100288413	8.84	8.77	8.83
200693_at	tyrosine 3-monoo	YWHAQ	13.04	13.08	13.08
220647_s_at	coiled-coil-helix-c	CHCHD8	11.12	11.06	11.14
218499_at	serine/threonine		MST4	4.40	4.03	4.35
204719_at	ATP-binding casse	ABCA8	6.68	6.71	6.78
AFFX-HUMRGE/	---	---	13.68	13.66	13.65
208821_at	small nuclear ribo	SNRPB	11.25	11.25	11.25
207712_at	B melanoma antig	BAGE	5.13	5.21	5.50
208436_s_at	interferon regulat	IRF7	7.04	7.01	7.03
219869_s_at	solute carrier fam	SLC39A8	8.28	8.39	8.35
203706_s_at	frizzled homolog 7	FZD7	8.08	8.34	8.26
201579_at	FAT tumor suppre	FAT1	10.13	10.14	10.13
202143_s_at	COP9 constitutive	COPS8	10.88	10.98	10.97
201243_s_at	ATPase, Na+/K+ tr	ATP1B1	10.40	10.47	10.48
218285_s_at	3-hydroxybutyrate	BDH2	9.11	9.12	9.10
214719_at	solute carrier fam	SLC46A3	8.80	8.86	8.88
204312_x_at	cAMP responsive	CREB1	7.13	7.22	7.30
220327_at	vestigial like 3 (Dr	VGLL3	8.26	8.38	8.53
201286_at	syndecan 1	SDC1	9.68	9.60	9.52
200954_at	ATPase, H+ transp	ATP6V0C	8.69	8.71	8.69
218950_at	ArfGAP with RhoG	ARAP3	8.10	8.17	8.14
216977_x_at	small nuclear ribo	SNRPA1	11.28	11.29	11.28
202369_s_at	translocation assc	TRAM2	9.67	9.60	9.72
208241_at	neuregulin 1	NRG1	6.90	7.19	7.15
213581_at	programmed cell	PDCD2	10.18	10.12	10.21
213375_s_at	NEDD4 binding pr	N4BP2L1	4.36	4.42	4.54
201272_at	aldo-keto reducta	AKR1B1	13.29	13.27	13.24
201147_s_at	TIMP metallopept	TIMP3	4.86	4.86	4.65
211864_s_at	myoferlin	MYOF	9.12	9.23	9.23
201110_s_at	thrombospondin 1	THBS1	12.22	12.28	12.46
201422_at	interferon, gamm	IFI30	8.12	8.21	8.24
222227_at	zinc finger proteir	zinc finger protein	9.78	9.71	10.13
218111_s_at	cytidine monopho	CMAS	9.02	8.98	9.04
214577_at	microtubule-asso	MAP1B	9.00	9.04	8.99
205889_s_at	janus kinase and r	JAKMIP2	3.51	3.53	3.53
205529_s_at	runt-related trans	RUNX1T1	5.49	5.70	5.74
222027_at	Nuclear casein kin	NUCKS1	8.23	8.28	8.19
201185_at	HtrA serine peptic	HTRA1	9.24	9.21	9.20
220062_s_at	melanoma antiger	MAGEC2	8.20	8.48	8.02
207177_at	prostaglandin F re	PTGFR	9.58	9.56	9.63
206120_at	CD33 molecule	CD33	7.85	7.88	7.93
202388_at	regulator of G-prc	RGS2	9.51	9.73	9.66

205032_at	integrin, alpha 2 ((	ITGA2	8.72	8.60	8.84
202747_s_at	integral membran	ITM2A	8.28	8.32	8.44
32137_at	jagged 2	JAG2	8.78	8.69	8.62
213730_x_at	transcription facto	TCF3	7.74	7.71	7.74
219978_s_at	nucleolar and spir	NUSAP1	11.34	11.36	11.34
204686_at	insulin receptor su	IRS1	8.42	8.31	8.47
207510_at	bradykinin recept	BDKRB1	6.76	6.83	6.73
215276_at	WAP four-disulfid	WFDC8	5.57	5.44	5.48
202724_s_at	forkhead box O1	FOXO1	9.91	9.81	9.94
212274_at	lipin 1	LPIN1	11.14	11.24	11.18
203778_at	mannosidase, bet	MANBA	8.80	8.74	8.80
203215_s_at	myosin VI	MYO6	9.32	9.42	9.37
203548_s_at	lipoprotein lipase	LPL	4.95	4.67	4.95
204840_s_at	early endosome a	EEA1	5.13	5.16	5.22
202466_at	PAP associated dc	PAPD7	10.50	10.49	10.55
214242_at	Mannosidase, alpl	MAN1A2	5.08	5.09	5.03
207988_s_at	actin related prot	ARPC2	12.00	11.94	12.01
212989_at	sphingomyelin syr	SGMS1	8.31	8.35	8.46
203567_s_at	tripartite motif-co	TRIM38	8.12	8.06	8.13
204466_s_at	synuclein, alpha (r	SNCA	8.86	8.97	8.97
202766_s_at	fibrillin 1	FBN1	7.23	7.30	7.22
202208_s_at	ADP-ribosylation f	ARL4C	8.37	8.26	8.37
211277_x_at	amyloid beta (A4)	APP	7.69	7.69	7.69
203323_at	caveolin 2	CAV2	9.52	9.56	9.63
202685_s_at	AXL receptor tyro:	AXL	7.38	7.33	7.27
205656_at	protocadherin 17	PCDH17	8.73	8.73	8.77
211896_s_at	decorin	DCN	8.05	8.08	8.06
203889_at	secretogranin V (7	SCG5	8.12	8.47	8.55
209514_s_at	RAB27A, member	RAB27A	8.55	8.59	8.59
215034_s_at	transmembrane 4	TM4SF1	9.63	9.66	9.71
205532_s_at	cadherin 6, type 2	CDH6	6.42	6.24	6.60
207732_s_at	discs, large homol	DLG3	4.83	4.83	4.79
209012_at	triple functional d	TRIO	10.70	10.71	10.74
213310_at	Eukaryotic transla	EIF2C2	8.03	8.09	8.20
212897_at	cyclin-dependent	CDK19	7.41	7.33	7.40
203589_s_at	transcription facto	TFDP2	7.40	7.55	7.62
203972_s_at	peroxisomal bioge	PEX3	8.82	8.90	8.86
213513_x_at	actin related prot	ARPC2	12.79	12.85	12.84
221830_at	RAP2A, member c	RAP2A	9.86	9.83	9.86
211380_s_at	protein kinase, cG	PRKG1	4.25	4.20	4.22
203325_s_at	collagen, type V, a	COL5A1	8.90	8.90	8.95
212205_at	H2A histone famil	H2AFV	11.92	12.02	12.11
220242_x_at	zinc finger proteir	ZNF701	5.30	5.40	5.33
202484_s_at	methyl-CpG bindi	MBD2	11.29	11.33	11.31
204604_at	cyclin-dependent	CDK14	9.38	9.34	9.45
212605_s_at	---	---	8.70	8.61	8.78
207090_x_at	zinc finger proteir	ZFP30	6.19	6.40	6.27

200971_s_at	stress-associated	SERP1	11.60	11.59	11.63
209774_x_at	chemokine (C-X-C	CXCL2	9.96	9.98	10.03
211535_s_at	fibroblast growth	FGFR1	9.20	9.21	9.22
213376_at	zinc finger and BT	ZBTB1	9.36	9.38	9.38
219377_at	family with seque	FAM59A	6.63	6.54	6.49
218796_at	fermitin family ho	FERMT1	9.64	9.50	9.62
217233_at	---	---	4.12	4.22	4.20
209387_s_at	transmembrane 4	TM4SF1	9.88	9.88	10.07
218699_at	RAB7, member RA	RAB7L1	10.58	10.58	10.61
209493_at	PDZ domain conta	PDZD2	6.76	6.53	6.71
203086_at	kinesin heavy chai	KIF2A	7.11	7.11	7.10
209225_x_at	transportin 1	TNPO1	12.36	12.41	12.41
213558_at	piccolo (presynap	PCLO	7.15	7.34	7.23
206114_at	EPH receptor A4	EPHA4	6.86	6.96	6.75
220987_s_at	chromosome 11 c	C11orf17 /// NUAK	7.58	7.45	7.45
201879_at	ariadne homolog,	ARIH1	8.68	8.70	8.67
212451_at	SECIS binding prot	SECISBP2L	7.67	7.72	7.64
222162_s_at	ADAM metallopep	ADAMTS1	7.77	7.65	7.72
203474_at	IQ motif containir	IQGAP2	4.78	4.91	5.00
218193_s_at	golgi transport 1 h	GOLT1B	8.89	8.91	8.89
40524_at	protein tyrosine p	PTPN21	5.74	5.84	5.75
206860_s_at	missing oocyte, m	MIOS	11.11	11.20	11.14
209522_s_at	carnitine O-acetyl	CRAT	7.12	7.12	7.03
212323_s_at	vacuolar protein s	VPS13D	8.08	8.09	8.09
214456_x_at	serum amyloid A1	SAA1 /// SAA2	11.51	11.62	11.64
208289_s_at	etoposide induced	EI24	10.63	10.65	10.65
217919_s_at	mitochondrial ribo	MRPL42	11.02	10.98	11.00
210946_at	phosphatidic acid	PPAP2A	10.61	10.62	10.64
215078_at	superoxide dismu	SOD2	7.30	7.60	7.65
212758_s_at	zinc finger E-box b	ZEB1	9.41	9.46	9.44
219566_at	pleckstrin homolo	PLEKHF1	6.89	6.83	6.73
209013_x_at	triple functional d	TRIO	8.74	8.69	8.74
213848_at	dual specificity ph	DUSP7	8.76	8.71	8.83
209267_s_at	solute carrier fam	SLC39A8	10.76	10.83	10.92
214070_s_at	ATPase, class V, ty	ATP10B	4.38	4.45	4.34
211122_s_at	chemokine (C-X-C	CXCL11	3.73	3.66	3.66
211961_s_at	RAB7A, member R	RAB7A	9.51	9.65	9.60
212752_at	cytoplasmic linker	CLASP1	9.51	9.42	9.52
212516_at	ArfGAP with RhoG	ARAP1	7.08	6.99	7.02
211964_at	collagen, type IV,	COL4A2	8.47	8.61	8.48
220098_at	hydrocephalus inc	HYDIN	4.91	4.92	4.86
205483_s_at	ISG15 ubiquitin-lik	ISG15	8.88	8.77	8.92
220032_at	chromosome 7 op	C7orf58	7.89	7.85	7.99
213948_x_at	cell adhesion mol	CADM3	7.73	7.99	7.98
211709_s_at	C-type lectin dom	CLEC11A	6.40	6.42	6.40
201426_s_at	vimentin	VIM	14.69	14.69	14.67
221056_x_at	epidermal growth	EPS15L1	6.61	6.69	6.64

217687_at	adenylate cyclase	ADCY2	4.70	4.65	4.67
210840_s_at	IQ motif containir	IQGAP1	10.07	10.22	10.17
207614_s_at	cullin 1	CUL1	9.28	9.38	9.36
210602_s_at	cadherin 6, type 2	CDH6	5.73	5.64	5.64
204419_x_at	hemoglobin, gamı	HBG1 /// HBG2	8.15	7.91	8.14
215293_s_at	post-GPI attachm	PGAP2	8.21	8.17	8.18
215603_x_at	gamma-glutamylt	GGT1 /// GGT2 ///	7.06	7.07	6.97
203233_at	interleukin 4 rece	IL4R	8.98	8.94	9.02
220407_s_at	transforming grov	TGFB2	4.87	4.64	4.80
203806_s_at	Fanconi anemia, c	FANCA	7.33	7.38	7.33
213687_s_at	ribosomal protein	RPL35A	14.15	14.06	14.06
204036_at	lysophosphatidic a	LPAR1	6.99	6.97	7.17
218281_at	mitochondrial rib	MRPL48	10.33	10.31	10.36
208988_at	lysine (K)-specific	KDM2A	9.19	9.27	9.28
202018_s_at	lactotransferrin	LTF	5.80	5.68	5.68
220170_at	four and a half LIN	FHL5	3.45	3.51	3.58
209125_at	keratin 6A	KRT6A	5.05	5.60	5.49
221483_s_at	cAMP-regulated p	ARPP19	11.12	11.13	11.16
209757_s_at	v-myc myelocytor	MYCN	4.48	4.49	4.44
209707_at	phosphatidylinosi	PIGK	9.31	9.35	9.40
212233_at	microtubule-asso	MAP1B	11.76	11.67	11.70
220477_s_at	chromosome 20 o	C20orf30	10.09	10.03	10.05
200060_s_at	RNA binding prote	RNPS1	11.55	11.53	11.52
218717_s_at	leprecan-like 1	LEPREL1	10.55	10.47	10.55
218176_at	melanoma antige	MAGEF1	8.73	8.65	8.67
AFFX-HUMRGE/	signal transducer	STAT1	13.97	13.99	13.98
202272_s_at	F-box protein 28	FBXO28	9.60	9.61	9.63
207875_at	---	---	3.45	3.42	3.37
213920_at	cut-like homeobo	CUX2	7.50	7.36	7.40
210797_s_at	2'-5'-oligoadenyla	OASL	6.89	6.76	6.81
202134_s_at	WW domain conta	WWTR1	6.11	6.12	6.07
220979_s_at	ST6 (alpha-N-acet	ST6GALNAC5	5.83	5.90	5.89
218984_at	pseudouridylate s	PUS7	9.78	9.82	9.89
222006_at	leucine zipper-EF-	LETM1	8.91	8.90	8.87
207243_s_at	calmodulin 2 (pho	CALM2	14.37	14.43	14.41
214629_x_at	reticulon 4	RTN4	12.96	12.90	12.95
209909_s_at	transforming grov	TGFB2	5.68	5.45	5.97
213560_at	Growth arrest anc	GADD45B	7.54	7.54	7.57
203333_at	kinesin-associatec	KIFAP3	9.46	9.47	9.46
202674_s_at	LIM domain 7	LMO7	5.17	5.27	5.18
211202_s_at	lysine (K)-specific	KDM5B	7.98	7.94	7.96
205728_at	odz, odd Oz/ten-n	ODZ1	7.59	7.52	7.87
200958_s_at	syndecan binding	SDCBP	12.10	12.17	12.17
220975_s_at	C1q and tumor ne	C1QTNF1	7.15	7.07	7.05
209270_at	laminin, beta 3	LAMB3	7.17	7.22	7.06
203355_s_at	pleckstrin and Sec	PSD3	8.45	8.41	8.45
209226_s_at	transportin 1	TNPO1	11.86	11.84	11.88

221043_at	---	---	3.30	3.22	3.28
208607_s_at	serum amyloid A1	SAA1 /// SAA2	10.33	10.21	10.27
209168_at	glycoprotein M6B	GPM6B	7.85	7.80	7.89
202975_s_at	Rho-related BTB d	RHOBTB3	7.83	7.90	7.91
209710_at	GATA binding pro	GATA2	8.44	8.42	8.56
209568_s_at	ral guanine nuclec	RGL1	8.79	8.66	8.80
204018_x_at	hemoglobin, alpha	HBA1 /// HBA2	6.74	6.72	6.76
211506_s_at	interleukin 8	IL8	8.44	8.70	8.73
211561_x_at	mitogen-activatec	MAPK14	7.08	7.15	6.97
211451_s_at	potassium inward	KCNJ4	5.21	5.14	5.10
213006_at	CCAAT/enhancer	CEBPD	6.33	6.41	6.42
213349_at	transmembrane a	TMCC1	6.64	6.73	6.70
208949_s_at	lectin, galactoside	LGALS3	9.44	9.42	9.37
201817_at	ubiquitin protein l	UBE3C	9.93	9.87	9.99
202644_s_at	tumor necrosis fac	TNFAIP3	9.63	9.72	9.63
215257_at	ERGIC and golgi 3	ERGIC3	3.47	3.46	3.42
211237_s_at	fibroblast growth	FGFR4	6.48	6.49	6.46
202705_at	cyclin B2	CCNB2	12.32	12.25	12.27
216265_x_at	myosin, heavy cha	MYH7	5.19	5.22	5.21
209466_x_at	pleiotrophin	PTN	8.00	8.08	8.09
213717_at	LIM domain bindi	LDB3	6.87	6.86	6.82
213725_x_at	xylosyltransferase	XYLT1	7.77	7.85	7.97
200727_s_at	ARP2 actin-relatec	ACTR2	9.62	9.57	9.68
213164_at	solute carrier fam	SLC5A3	9.64	9.77	9.73
202746_at	integral membran	ITM2A	7.66	7.78	7.82
213233_s_at	kelch-like 9 (Drosc	KLHL9	9.24	9.42	9.60
208017_s_at	MCF.2 cell line de	MCF2	5.51	5.55	5.59
214452_at	branched chain ar	BCAT1	8.24	8.26	8.42
215032_at	ras responsive ele	RREB1	5.77	5.74	5.84
205188_s_at	SMAD family men	SMAD5	7.91	7.84	7.91
212044_s_at	Ribosomal proteir	RPL27A	9.41	9.42	9.48
217502_at	interferon-induce	IFIT2	6.71	6.73	6.54
202740_at	aminoacylase 1	ACY1	8.55	8.42	8.49
202087_s_at	cathepsin L1	CTSL1	9.84	9.84	9.77
203277_at	DNA fragmentatic	DFFA	9.23	9.26	9.23
209647_s_at	suppressor of cytc	SOCS5	9.27	9.32	9.30
209277_at	tissue factor path	TFPI2	7.03	7.05	7.18
219427_at	FAT tumor suppre	FAT4	7.12	7.36	7.36
211275_s_at	glycogenin 1	GYG1	11.21	11.20	11.18
212807_s_at	sortilin 1	SORT1	8.89	8.90	9.00
212832_s_at	cytoskeleton asso	CKAP5	9.70	9.68	9.68
204784_s_at	myeloid leukemia	MLF1	8.97	9.06	9.21
201989_s_at	cAMP responsive	CREBL2	9.31	9.33	9.36
215879_at	integrin, beta 1 (fi	ITGB1	3.15	3.08	3.08
221123_x_at	zinc finger proteir	ZNF395	9.05	8.88	9.02
203708_at	phosphodiesteras	PDE4B	5.10	5.03	5.18
206806_at	diacylglycerol kina	DGKI	7.33	7.52	7.22

211282_x_at	tumor necrosis fa	TNFRSF25	6.05	6.06	6.14
214808_at	---	---	7.65	7.69	7.66
215076_s_at	collagen, type III, a	COL3A1	6.43	6.43	6.50
205856_at	solute carrier fam	SLC14A1	5.09	5.04	4.98
212926_at	structural mainter	SMC5	7.79	7.96	7.94
215128_at	---	---	6.76	6.75	6.85
209448_at	HIV-1 Tat interact	HTATIP2	9.92	9.88	9.99
209863_s_at	tumor protein p63	TP63	9.58	9.48	9.63
219474_at	chromosome 3 op	C3orf52	6.84	6.75	6.90
205696_s_at	GDNF family recep	GFRA1	5.05	5.04	4.87
212667_at	secreted protein,	SPARC	9.64	9.73	9.82
202311_s_at	collagen, type I, al	COL1A1	6.82	6.90	6.97
202888_s_at	DNA-damage-indu	DDIT4	10.14	10.23	10.24
201968_s_at	phosphoglucomut	PGM1	9.97	9.94	9.86
203956_at	MORC family CW-	MORC2	9.52	9.55	9.54
206448_at	zinc finger protein	ZNF365	7.62	7.62	7.60
210214_s_at	bone morphogene	BMPR2	6.24	6.24	6.45
49306_at	Ras association (R	RASSF4	5.78	5.80	5.64
212805_at	prune homolog 2	PRUNE2	6.22	6.02	6.24
205923_at	reelin	RELN	6.87	6.73	7.05
205503_at	protein tyrosine p	PTPN14	5.75	5.70	5.73
204848_x_at	hemoglobin, gam	HBG1 /// HBG2	7.76	7.65	7.98
221185_s_at	IQ motif containin	IQCG	8.03	8.12	8.19
212848_s_at	chromosome 9 op	C9orf3	10.66	10.68	10.68
206971_at	G protein-couplec	GPR161	5.50	5.42	5.41
202066_at	protein tyrosine p	PPFIA1	9.43	9.46	9.48
218438_s_at	mediator complex	MED28	9.83	9.83	9.83
208621_s_at	ezrin	EZR	10.74	10.77	10.74
218667_at	praja ring finger 1	PJA1	9.13	9.11	9.09
204082_at	pre-B-cell leukemi	PBX3	8.68	8.73	8.70
218691_s_at	PDZ and LIM dom	PDLIM4	8.19	8.23	8.29
212836_at	polymerase (DNA-	POLD3	8.34	8.46	8.43
205848_at	growth arrest-spe	GAS2	3.95	3.95	4.05
208961_s_at	Kruppel-like facto	KLF6	10.43	10.53	10.56
218109_s_at	major facilitator s	MFSD1	10.78	10.88	10.91
208807_s_at	chromodomain he	CHD3	8.04	8.09	8.06
219588_s_at	non-SMC condens	NCAPG2	8.59	8.41	8.54
218125_s_at	coiled-coil domair	CCDC25	9.50	9.46	9.52
221973_at	---	---	6.00	6.18	6.05
212314_at	sel-1 suppressor c	SEL1L3	9.24	9.23	9.39
205138_s_at	uronyl-2-sulfotrar	UST	5.62	5.76	5.67
214136_at	nudix (nucleoside	NUDT13	6.68	6.40	6.49
201852_x_at	collagen, type III, a	COL3A1	5.97	5.99	5.70
204688_at	sarcoglycan, epsilo	SGCE	10.23	10.18	10.20
213553_x_at	apolipoprotein C-I	APOC1	9.06	8.93	9.01
212686_at	protein phosphata	PPM1H	7.58	7.48	7.72
1598_g_at	growth arrest-spe	GAS6	9.69	9.77	9.65

205480_s_at	UDP-glucose pyro	UGP2	11.23	11.32	11.33
213738_s_at	ATP synthase, H+	ATP5A1	13.61	13.63	13.59
201068_s_at	proteasome (pros	PSMC2	12.04	12.10	12.10
215446_s_at	lysyl oxidase	LOX	6.40	6.12	6.57
212097_at	caveolin 1, caveol	CAV1	12.64	12.79	12.77
202619_s_at	procollagen-lysine	PLOD2	8.65	8.75	8.78
208987_s_at	lysine (K)-specific	KDM2A	8.39	8.33	8.34
214731_at	---	---	6.64	6.68	6.70
214078_at	---	---	3.80	3.81	3.69
208692_at	ribosomal protein	RPS3	14.40	14.35	14.41
219562_at	RAB26, member F	RAB26	7.61	7.63	7.50
216060_s_at	dishevelled associ	DAAM1	6.44	6.65	6.67
217269_s_at	transmembrane p	TMPRSS15	10.48	10.53	10.63
219671_at	hippocalcin like 4	HPCAL4	3.97	3.96	3.87
204205_at	apolipoprotein B r	APOBEC3G	6.12	6.03	6.08
200907_s_at	palladin, cytoskele	PALLD	12.18	12.02	12.07
212261_at	GRB10 interacting	GIGYF2	7.75	7.74	7.75
208945_s_at	beclin 1, autophag	BECN1	10.17	10.16	10.16
203387_s_at	TBC1 domain fam	TBC1D4	8.25	8.34	8.40
215915_at	GULP, engulfment	GULP1	3.60	3.52	3.57
218845_at	dual specificity ph	DUSP22	9.07	9.04	9.09
212481_s_at	tropomyosin 4	TPM4	9.16	9.12	9.11
210015_s_at	microtubule-assoc	MAP2	7.29	7.25	7.39
205112_at	phospholipase C, (	PLCE1	6.87	6.86	6.97
207434_s_at	FXYD domain cont	FXYD2	5.69	5.65	5.64
204346_s_at	Ras association (R	RASSF1	8.17	8.24	8.18
203028_s_at	cytochrome b-245	CYBA	9.19	9.08	9.03
204464_s_at	endothelin recept	EDNRA	8.01	7.84	8.13
201125_s_at	integrin, beta 5	ITGB5	9.81	9.82	9.74
209623_at	methylcrotonoyl-(	MCCC2	10.61	10.56	10.61
213428_s_at	collagen, type VI,	COL6A1	8.13	7.91	7.93
201941_at	carboxypeptidase	CPD	10.24	10.28	10.27
202382_s_at	glucosamine-6-ph	GNPDA1	9.18	9.22	9.18
206315_at	cytokine receptor	CRLF1	5.71	5.67	5.66
206373_at	Zic family membe	ZIC1	7.88	7.97	8.05
219295_s_at	procollagen C-enc	PCOLCE2	9.71	9.62	9.76
203065_s_at	caveolin 1, caveol	CAV1	13.17	13.34	13.25
204715_at	pannexin 1	PANX1	9.00	9.02	9.10
211724_x_at	missing oocyte, m	MIOS	11.16	11.22	11.31
201490_s_at	peptidylprolyl isor	PPIF	8.85	8.76	8.84
221477_s_at	superoxide dismu	SOD2	8.63	8.53	8.66
219428_s_at	peroxisomal mem	PXMP4	8.14	8.16	8.12
207966_s_at	golgi glycoprotein	GLG1	9.24	9.25	9.27
205904_at	MHC class I polyp	MICA	9.66	9.60	9.75
203836_s_at	mitogen-activatec	MAP3K5	6.61	6.56	6.73
201709_s_at	nipsnap homolog	NIPSNAP1	9.76	9.75	9.69
214661_s_at	NOP14 nucleolar	NOP14	9.45	9.35	9.52

210139_s_at	peripheral myelin	PMP22	8.98	9.10	9.00
201982_s_at	pregnancy-associa	PAPPA	5.34	5.29	5.39
221957_at	pyruvate dehydro	PDK3	5.62	5.69	5.58
203623_at	plexin A3	PLXNA3	6.82	6.82	6.81
204889_s_at	neuralized homolo	NEURL	6.54	6.44	6.47
212840_at	UBX domain prote	UBXN7	7.87	7.76	7.89
205533_s_at	cadherin 6, type 2	CDH6	4.36	4.50	4.59
206343_s_at	neuregulin 1	NRG1	8.60	8.41	8.67
207625_s_at	core-binding facto	CBFA2T2	7.47	7.49	7.50
219230_at	transmembrane p	TMEM100	8.91	9.02	9.04
211966_at	collagen, type IV,	COL4A2	5.88	5.92	5.89
202723_s_at	forkhead box O1	FOXO1	9.45	9.30	9.42
205240_at	G-protein signalin	GPSM2	10.39	10.33	10.39
219181_at	lipase, endothelia	LIPG	6.08	6.34	6.29
201998_at	ST6 beta-galactos	ST6GAL1	8.66	8.35	8.66
215047_at	tripartite motif-co	TRIM58	9.00	9.09	9.19
202315_s_at	breakpoint cluster	BCR	7.99	7.93	8.12
202050_s_at	zinc finger, MYM-t	ZMYM4	8.73	8.69	8.73
207348_s_at	ligase III, DNA, AT	LIG3	6.05	6.02	5.86
222094_at	sulfotransferase fa	SULT1A3 /// SULT1	5.63	5.62	5.70
219975_x_at	oleoyl-ACP hydrol	OLAH	9.38	9.38	9.53
212802_s_at	GTPase activating	GAPVD1	9.84	9.88	9.90
222101_s_at	dachsous 1 (Drosc	DCHS1	5.32	5.67	5.50
200830_at	proteasome (pros	PSMD2	11.45	11.44	11.41
208217_at	gamma-aminobut	GABRR2	5.76	5.82	5.87
201279_s_at	disabled homolog	DAB2	8.67	8.51	8.72
222209_s_at	transmembrane p	TMEM135	9.37	9.41	9.44
209069_s_at	H3 histone, family	H3F3B	13.16	13.12	13.14
208745_at	ATP synthase, H+	ATP5L	10.29	10.37	10.33
204347_at	adenylate kinase 3	AK3L1	6.47	6.50	6.46
50376_at	zinc finger protein	ZNF444	7.58	7.55	7.49
222054_at	peptidylprolyl isor	PPIEL	5.65	5.58	5.55
204416_x_at	apolipoprotein C-I	APOC1	9.37	9.17	9.35
213444_at	zinc finger protein	ZNF862	6.85	6.82	6.70
201645_at	tenascin C	TNC	10.43	10.35	10.58
202648_at	---	---	8.08	8.10	8.08
206010_at	hyaluronan bindir	HABP2	5.35	5.36	5.28
218507_at	chromosome 7 op	C7orf68	8.56	8.52	8.62
205568_at	aquaporin 9	AQP9	7.88	8.05	8.05
220240_s_at	transmembrane a	TMCO3	7.43	7.15	7.31
202643_s_at	tumor necrosis fa	TNFAIP3	8.33	8.45	8.45
203060_s_at	3'-phosphoadenos	PAPSS2	4.58	4.38	4.30
222288_at	---	---	8.30	8.19	8.41
201668_x_at	myristoylated alar	MARCKS	7.71	7.87	7.75
212351_at	eukaryotic transla	EIF2B5	9.39	9.33	9.41
215810_x_at	Dystonin	DST	3.75	3.73	3.67
202627_s_at	serpin peptidase i	SERPINE1	12.50	12.32	12.49

214924_s_at	trafficking protein	TRAK1	8.80	8.66	8.70
214366_s_at	arachidonate 5-lip	ALOX5	5.02	4.96	4.93
202217_at	chromosome 21 c	C21orf33	9.68	9.68	9.65
205854_at	tubby like protein	TULP3	8.12	8.13	8.16
202464_s_at	6-phosphofructo-2	PFKFB3	7.69	7.61	7.86
217966_s_at	family with seque	FAM129A	8.90	8.53	8.76
200884_at	creatine kinase, b	CKB	6.72	6.91	6.69
204470_at	chemokine (C-X-C	CXCL1	6.23	6.32	6.24
216640_s_at	protein disulfide is	PDIA6	11.46	11.51	11.46
206155_at	ATP-binding casse	ABCC2	6.23	6.43	6.48
200038_s_at	ribosomal protein	RPL17	14.45	14.47	14.49
218644_at	pleckstrin 2	PLEK2	8.62	8.82	8.72
221922_at	G-protein signalin	GPSM2	10.81	10.87	10.91
209485_s_at	oxysterol binding	OSBPL1A	9.94	9.91	9.91
216812_at	---	---	4.25	4.29	4.31
221481_x_at	heterogeneous nu	HNRNPD	12.85	12.81	12.83
205660_at	2'-5'-oligoadenyla	OASL	6.35	5.66	6.00
60474_at	fermitin family ho	FERMT1	9.17	9.09	9.22
203974_at	haloacid dehaloge	HDHD1A	7.85	7.87	8.03
211376_s_at	non-SMC element	NSMCE4A	10.16	10.16	10.19
213139_at	snail homolog 2 (D	SNAI2	10.44	10.49	10.60
207444_at	solute carrier fam	SLC22A13	4.69	4.79	4.83
211368_s_at	caspase 1, apopto	CASP1	9.23	9.44	9.52
40665_at	flavin containing r	FMO3	3.21	3.25	3.22
204613_at	phospholipase C, g	PLCG2	4.92	5.05	4.97
201150_s_at	TIMP metallopept	TIMP3	4.89	4.71	5.46
202059_s_at	karyopherin alpha	KPNA1	8.56	8.57	8.66
203213_at	cyclin-dependent	CDK1	11.72	11.81	11.89
210793_s_at	nucleoporin 98kD	NUP98	8.60	8.64	8.74
218273_s_at	pyruvate dehyrog	PDP1	7.02	7.20	7.00
220278_at	lysine (K)-specific	KDM4D	5.56	5.59	5.51
207526_s_at	interleukin 1 rece	IL1RL1	7.84	7.84	8.08
207782_s_at	presenilin 1	PSEN1	7.19	7.22	7.10
215091_s_at	general transcript	GTF3A	12.91	12.88	12.92
213812_s_at	calcium/calmodul	CAMKK2	11.08	11.06	11.04
210027_s_at	APEX nuclease (m	APEX1	12.10	12.18	12.22
218856_at	tumor necrosis fa	TNFRSF21	8.64	8.53	8.85
204616_at	ubiquitin carboxyl	UCHL3	10.48	10.48	10.60
221068_at	KN motif and anky	KANK2	7.49	7.50	7.50
213541_s_at	v-ets erythroblast	ERG	6.98	6.87	6.91
204440_at	CD83 molecule	CD83	8.05	8.29	8.25
205955_at	---	---	4.95	4.84	4.94
214114_x_at	Fas-activated seri	FASTK	9.90	9.87	9.77
202411_at	interferon, alpha-	IFI27	8.08	8.05	8.18
219497_s_at	B-cell CLL/lympho	BCL11A	3.71	3.81	3.80
219561_at	coatomer protein	COPZ2	8.55	8.53	8.41
217813_s_at	spindlin 1	SPIN1	8.27	8.46	8.48

222108_at	adhesion molecul	AMIGO2	10.67	10.73	10.92
218611_at	immediate early r	IER5	10.46	10.45	10.48
208499_s_at	DnaJ (Hsp40) hom	DNAJC3	7.62	7.61	7.65
216250_s_at	leupaxin	LPXN	8.04	8.18	8.13
214407_x_at	glycophorin B (MN	GYPB	5.62	5.58	5.48
212008_at	UBX domain prote	UBXN4	7.99	8.08	8.00
208082_x_at	---	---	9.81	9.90	9.94
201906_s_at	CTD (carboxy-tern	CTDSPL	8.48	8.41	8.50
209305_s_at	growth arrest and	GADD45B	9.99	9.87	10.02
217599_s_at	MyoD family inhib	MDFIC	6.49	6.44	6.52
215030_at	G-rich RNA seque	GRSF1	8.44	8.42	8.47
205220_at	G protein-couplec	GPR109B	5.60	5.32	5.36
209310_s_at	caspase 4, apopto	CASP4	8.25	8.25	8.39
218718_at	platelet derived g	PDGFC	10.73	10.56	10.91
204348_s_at	adenylate kinase 3	AK3L1	7.84	7.93	8.03
218627_at	DNA-damage regu	DRAM1	9.25	9.15	9.16
202859_x_at	U2 small nuclear F	U2AF1	10.71	10.94	10.94
213075_at	olfactomedin-like	OLFML2A	8.10	8.14	8.09
202022_at	aldolase C, fructos	ALDOC	7.28	7.18	7.38
218883_s_at	MLF1 interacting p	MLF1IP	9.71	9.67	9.70
216982_x_at	---	---	4.51	4.36	4.42
203358_s_at	enhancer of zeste	EZH2	9.43	9.35	9.53
212092_at	paternally express	PEG10	7.48	7.57	7.49
213184_at	SUMO1/sentrin sp	SENP5	8.46	8.55	8.44
202459_s_at	lipin 2	LPIN2	7.96	7.94	8.02
202370_s_at	core-binding facto	CBFB	11.24	11.23	11.24
200973_s_at	tetraspanin 3	TSPAN3	9.96	9.92	9.90
211165_x_at	EPH receptor B2	EPHB2	5.81	5.56	5.75
220016_at	AHNAK nucleopro	AHNAK	7.65	7.54	7.39
212560_at	sortilin-related re	SORL1	7.74	7.74	7.86
201762_s_at	proteasome (pros	PSME2	11.07	11.04	11.13
220610_s_at	leucine rich repea	LRRFIP2	8.30	8.15	8.30
219682_s_at	T-box 3	TBX3	8.05	8.20	8.13
209822_s_at	very low density li	VLDLR	4.42	4.34	4.49
200845_s_at	peroxiredoxin 6	PRDX6	12.13	12.17	12.14
213720_s_at	SWI/SNF related,	SMARCA4	10.29	10.27	10.27
210998_s_at	hepatocyte growt	HGF	4.40	4.47	4.45
204518_s_at	peptidylprolyl isor	PPIC	8.46	8.49	8.50
218430_s_at	regulatory factor X	RFX7	6.89	6.97	6.87
201240_s_at	signal peptidase c	LOC653566 /// SPC	12.38	12.46	12.39
218229_s_at	pogo transposable	POGK	9.27	9.25	9.32
41660_at	cadherin, EGF LAG	CELSR1	7.07	6.96	7.05
206116_s_at	tropomyosin 1 (al	TPM1	10.81	10.76	10.90
207065_at	keratin 75	KRT75	9.25	9.43	9.28
218460_at	HEAT repeat conta	HEATR2	11.06	11.04	11.06
215778_x_at	similar to mucin	LOC100288496	7.67	7.67	7.63
210609_s_at	tumor protein p53	TP53I3	8.98	9.00	8.80

212426_s_at	tyrosine 3-monoo	YWHAQ	13.43	13.42	13.48
214176_s_at	Pre-B-cell leukemi	PBXIP1	6.08	6.16	6.02
208616_s_at	protein tyrosine p	PTP4A2	13.11	13.11	13.13
214915_at	Zinc finger protein	ZNF362	4.87	4.95	4.82
200667_at	ubiquitin-conjugat	UBE2D3	11.45	11.53	11.49
213587_s_at	ATPase, H+ transp	ATP6V0E2	7.81	8.02	7.77
205968_at	potassium voltage	KCNS3	8.54	8.64	8.67
214857_at	---	---	7.50	7.51	7.40
217244_at	---	---	3.91	3.81	3.84
209572_s_at	embryonic ectode	EED	9.95	10.03	10.03
218559_s_at	v-maf musculoapo	MAFB	5.37	5.58	5.58
209185_s_at	insulin receptor su	IRS2	11.18	11.10	11.20
202873_at	ATPase, H+ transp	ATP6V1C1	5.89	5.82	5.80
211525_s_at	glycoprotein V (pl	GP5	3.49	3.59	3.64
201782_s_at	aryl hydrocarbon	AIP	9.34	9.32	9.27
218258_at	polymerase (RNA)	POLR1D	11.93	11.84	11.89
207321_s_at	ATP-binding casse	ABCB9	6.06	6.18	6.27
201020_at	tyrosine 3-monoo	YWHAH	10.55	10.62	10.66
213033_s_at	nuclear factor I/B	NFIB	8.66	8.57	8.65
209246_at	ATP-binding casse	ABCF2	8.75	8.75	8.68
203837_at	mitogen-activated	MAP3K5	6.85	6.80	7.07
206554_x_at	SET domain and m	SETMAR	6.54	6.67	6.58
209445_x_at	chromosome 7 op	C7orf44	8.92	8.92	8.99
220000_at	sialic acid binding	SIGLEC5	4.58	4.46	4.55
209674_at	cryptochrome 1 (p	CRY1	8.85	8.79	8.94
211075_s_at	CD47 molecule	CD47	10.27	10.32	10.31
212345_s_at	cAMP responsive	CREB3L2	10.74	10.71	10.85
203609_s_at	aldehyde dehydro	ALDH5A1	4.96	4.82	4.80
218149_s_at	zinc finger protein	ZNF395	10.16	10.06	10.07
203011_at	inositol(myo)-1(or	IMPA1	11.13	11.24	11.26
203408_s_at	SATB homeobox 1	SATB1	8.27	8.36	8.38
205105_at	mannosidase, alph	MAN2A1	9.91	9.99	10.11
212470_at	sperm associated	SPAG9	10.15	10.11	10.17
218066_at	solute carrier fam	SLC12A7	9.71	9.54	9.50
219126_at	PHD finger protein	PHF10	8.87	8.89	8.90
204992_s_at	profilin 2	PFN2	12.15	12.20	12.25
200704_at	lipopolysaccharide	LITAF	9.89	9.82	10.00
212162_at	kinase D-interacti	KIDINS220	8.81	8.80	8.78
212873_at	histocompatibility	HMHA1	7.63	7.48	7.63
219572_at	Ca++-dependent s	CADPS2	8.56	8.60	8.71
211379_x_at	beta-1,3-N-acetylg	B3GALNT1	5.98	6.08	6.14
206348_s_at	pyruvate dehydro	PDK3	5.73	5.49	5.49
208605_s_at	neurotrophic tyro	NTRK1	3.94	4.09	4.06
214971_s_at	ST6 beta-galactos	ST6GAL1	4.07	4.10	4.06
205547_s_at	transgelin	TAGLN	7.97	7.57	7.87
221250_s_at	MAX dimerization	MXD3	8.79	8.68	8.68
207825_s_at	growth hormone r	GHRHR	5.92	5.82	5.88

213875_x_at	chromosome 6 op	C6orf62	9.25	9.33	9.31
213370_s_at	Scm-like with four	SFMBT1	7.23	7.41	7.50
205023_at	RAD51 homolog (	RAD51	5.33	5.38	5.33
200997_at	RNA binding moti	RBM4	10.86	10.76	10.85
209169_at	glycoprotein M6B	GPM6B	8.11	8.13	8.24
213874_at	serpin peptidase i	SERPINA4	5.72	5.60	5.60
215507_x_at	---	---	5.19	5.23	5.24
206329_at	exostoses (multip	EXTL1	5.98	5.99	5.90
211783_s_at	metastasis associa	MTA1	9.18	9.22	9.24
208875_s_at	p21 protein (Cdc4	PAK2	8.35	8.42	8.33
202638_s_at	intercellular adhe	ICAM1	7.38	7.20	7.45
211737_x_at	pleiotrophin	PTN	8.20	8.37	8.47
204462_s_at	solute carrier fam	SLC16A2	4.53	4.47	4.53
211615_s_at	leucine-rich PPR-r	LRPPRC	10.96	10.90	10.90
212315_s_at	nucleoporin 210kI	NUP210	6.44	6.54	6.35
207740_s_at	nucleoporin 62kD	NUP62	7.69	7.70	7.71
218754_at	nucleolar protein	NOL9	10.02	10.03	10.05
205528_s_at	runt-related trans	RUNX1T1	5.92	5.43	5.36
200818_at	ATP synthase, H+	ATP5O	12.74	12.75	12.75
214269_at	major facilitator s	MFSD7	6.13	6.08	6.16
214771_x_at	myosin phosphata	MPRIP	10.63	10.62	10.60
210839_s_at	ectonucleotide py	ENPP2	10.38	10.27	10.39
209001_s_at	anaphase promot	ANAPC13	11.36	11.41	11.40
204257_at	fatty acid desatura	FADS3	9.59	9.57	9.52
219765_at	zinc finger protein	ZNF329	6.57	6.48	6.60
209228_x_at	tumor suppressor	TUSC3	10.43	10.43	10.43
210757_x_at	disabled homolog	DAB2	8.89	8.80	8.99
212651_at	Rho-related BTB d	RHOBTB1	7.17	7.11	7.18
216074_x_at	WW and C2 doma	WWC1	8.34	8.35	8.38
40016_g_at	microtubule assoc	MAST4	8.33	8.14	8.30
219041_s_at	replication initiato	REPIN1	10.55	10.52	10.52
210419_at	BARX homeobox 2	BARX2	6.61	6.47	6.52
212390_at	phosphodiesteras	PDE4DIP	6.79	6.83	6.64
204047_s_at	phosphatase and	PHACTR2	7.72	7.67	7.79
210236_at	protein tyrosine p	PPFIA1	7.11	7.18	7.18
207657_x_at	transportin 1	TNPO1	12.47	12.40	12.39
219477_s_at	thrombospondin,	THSD1 /// THSD1P	7.60	7.61	7.69
219833_s_at	EF-hand domain (	EFHC1	8.48	8.46	8.58
215395_x_at	protease, serine, 1	PRSS1 /// TRY6	7.45	7.38	7.40
222196_at	hypothetical prote	LOC286434	6.10	6.20	6.20
202193_at	LIM domain kinas	LIMK2	8.38	8.31	8.12
202691_at	small nuclear ribo	SNRPD1	10.50	10.57	10.49
211518_s_at	bone morphogene	BMP4	6.76	6.92	6.64
205905_s_at	MHC class I polyp	MICA /// MICB	9.38	9.33	9.41
201311_s_at	SH3 domain bindi	SH3BGRL	8.17	8.14	8.14
205662_at	B9 protein domai	B9D1	7.58	7.48	7.47
221636_s_at	MOCO sulphurase	MOSC2	8.61	8.76	8.73

217646_at	surfeit 1	SURF1	6.22	6.25	6.13
207522_s_at	ATPase, Ca++ tran	ATP2A3	4.84	4.76	4.80
201289_at	cysteine-rich, angi	CYR61	11.59	11.60	11.58
203691_at	peptidase inhibito	PI3	5.81	5.81	5.85
206368_at	complexin 2	CPLX2	6.12	5.99	6.03
211973_at	---	---	6.64	6.52	6.41
218355_at	kinesin family mer	KIF4A	9.90	9.90	9.94
219618_at	interleukin-1 rece	IRAK4	6.49	6.42	6.58
204003_s_at	nucleoporin like 2	NUPL2	8.45	8.44	8.52
200755_s_at	calumenin	CALU	10.08	10.13	10.24
203402_at	potassium voltage	KCNAB2	8.93	8.81	8.83
207850_at	chemokine (C-X-C	CXCL3	7.51	7.74	7.79
202109_at	ADP-ribosylation f	ARFIP2	8.14	8.26	8.28
218009_s_at	protein regulator	PRC1	11.76	11.73	11.75
219119_at	N(alpha)-acetyltra	NAA38	10.76	10.64	10.69
213404_s_at	Ras homolog enric	RHEB	11.18	11.19	11.54
204070_at	retinoic acid recep	RARRES3	6.10	5.94	6.20
205211_s_at	Ras and Rab intera	RIN1	7.95	7.88	7.89
210875_s_at	zinc finger E-box b	ZEB1	6.86	6.99	7.15
209242_at	paternally express	PEG3	3.81	3.71	3.78
212713_at	microfibrillar-asso	MFAP4	5.65	5.69	5.72
201054_at	heterogeneous nu	HNRNPA0	11.33	11.31	11.36
214447_at	v-ets erythroblast	ETS1	6.03	6.05	6.04
211113_s_at	ATP-binding casse	ABCG1	7.21	7.04	7.41
203509_at	sortilin-related re	SORL1	8.29	8.24	8.33
217316_at	olfactory receptor	OR7A10	3.95	3.93	4.01
210495_x_at	fibronectin 1	FN1	13.46	13.51	13.56
214845_s_at	calumenin	CALU	8.64	8.69	8.67
200897_s_at	palladin, cytoskele	PALLD	12.11	12.09	12.16
207874_s_at	complement facto	CFHR4	5.22	5.17	5.14
204463_s_at	endothelin recept	EDNRA	6.01	5.57	5.76
214400_at	insulin-like 3 (Leyc	INSL3	5.56	5.58	5.42
209323_at	protein-kinase, int	PRKRIR	10.38	10.43	10.47
205693_at	troponin T type 3	TNNT3	5.79	5.76	5.72
204352_at	TNF receptor-asso	TRAF5	7.58	7.72	7.60
204564_at	polycomb group r	PCGF3	5.93	5.94	5.96
201489_at	peptidylprolyl isor	PPIF	10.49	10.38	10.50
216929_x_at	ABO blood group	ABO	4.48	4.39	4.46
202625_at	v-yes-1 Yamaguch	LYN	7.55	7.57	7.80
204160_s_at	ectonucleotide py	ENPP4	6.86	7.05	7.13
212841_s_at	PTPRF interacting	PPFIBP2	5.74	5.48	5.65
210387_at	histone cluster 1,	HIST1H2BG	5.82	5.85	5.82
200756_x_at	calumenin	CALU	8.95	8.98	8.91
205098_at	chemokine (C-C m	CCR1	3.96	3.93	3.96
203386_at	TBC1 domain fam	TBC1D4	8.49	8.49	8.62
203021_at	secretory leukocy	SLPI	7.69	7.59	7.21
207803_s_at	casein kappa	CSN3	3.87	3.87	3.87

221777_at	chromosome 12 c	C12orf52		9.10	9.02	9.03
214298_x_at	septin 6		6-Sep	4.97	5.10	4.88
206194_at	homeobox C4	HOXC4		8.67	8.68	8.64
202804_at	integrin, beta 2 (c	ITGB2		9.20	9.09	9.18
211178_s_at	proline-serine-thr	PSTPIP1		4.93	4.96	4.90
201790_s_at	7-dehydrocholest	DHCR7		9.55	9.51	9.57
212464_s_at	fibronectin 1	FN1		13.25	13.24	13.32
212509_s_at	matrix-remodellin	MXRA7		12.45	12.40	12.39
215273_s_at	transcriptional ad	TADA3		7.66	7.69	7.68
201502_s_at	nuclear factor of k	NFKBIA		11.90	11.85	11.91
202321_at	geranylgeranyl dip	GGPS1		7.23	7.29	7.38
211000_s_at	interleukin 6 signa	IL6ST		9.55	9.53	9.62
202274_at	actin, gamma 2, sı	ACTG2		6.39	6.31	6.59
209920_at	bone morphogene	BMPR2		8.03	8.01	7.91
218694_at	armadillo repeat c	ARMCX1		4.03	3.80	3.97
208328_s_at	myocyte enhance	MEF2A		9.17	9.01	9.12
213423_x_at	tumor suppressor	TUSC3		10.34	10.42	10.39
220289_s_at	absent in melanor	AIM1L		6.72	6.47	6.50
221235_s_at	hypothetical prote	LOC100288263 ///		8.05	7.98	8.11
201972_at	ATPase, H+ transp	ATP6V1A		11.25	11.33	11.47
206579_at	zinc finger protein	ZNF192		4.07	3.90	3.99
202016_at	mesoderm specifi	MEST		10.33	10.29	10.33
212169_at	FK506 binding pro	FKBP9		9.85	9.83	9.85
218094_s_at	dysbindin (dystrol	DBNDD2 /// SYS1 /		9.30	9.14	9.31
214168_s_at	tight junction prot	TJP1		7.47	7.42	7.55
206417_at	cyclic nucleotide g	CNGA1		5.65	5.65	5.49
213906_at	v-myb myeloblast	MYBL1		9.47	9.41	9.67
215976_at	---	---		3.65	3.64	3.67
205603_s_at	diaphanous homo	DIAPH2		7.09	7.00	7.10
221042_s_at	calmin (calponin-l	CLMN		5.81	5.66	5.70
208385_at	nuclear receptor s	NR2E3		5.13	4.95	5.09
211675_s_at	MyoD family inhib	MDFIC		9.87	9.91	9.95
206783_at	fibroblast growth	FGF4		5.40	5.39	5.46
200607_s_at	RAD21 homolog (!	RAD21		11.06	11.11	11.09
204143_s_at	enolase superfam	ENOSF1		9.79	9.88	9.86
202278_s_at	serine palmitoyltr	SPTLC1		8.20	8.31	8.31
205973_at	fasciculation and e	FEZ1		6.62	6.66	6.81
205438_at	protein tyrosine p	PTPN21		5.97	6.03	6.04
203999_at	synaptotagmin I	SYT1		7.22	7.04	7.31
209010_s_at	triple functional d	TRIO		6.20	6.09	6.27
211476_at	myozenin 2	MYOZ2		5.77	5.75	5.89
210774_s_at	nuclear receptor c	NCOA4		11.73	11.77	11.78
205316_at	solute carrier fam	SLC15A2		5.60	5.40	5.27
221198_at	secretin	SCT		5.12	5.12	5.04
212794_s_at	KIAA1033	KIAA1033		9.44	9.63	9.55
201242_s_at	ATPase, Na+/K+ tr	ATP1B1		9.77	9.69	9.87
201841_s_at	heat shock 27kDa	HSPB1		11.34	11.20	11.26

212806_at	prune homolog 2	PRUNE2	4.12	4.01	3.84
218957_s_at	proteasomal ATPa	PAAF1	9.06	9.10	9.15
201141_at	glycoprotein (tran	GPNMB	5.11	5.15	4.96
204971_at	cystatin A (stefin A	CSTA	8.38	8.09	8.44
217967_s_at	family with seque	FAM129A	9.41	9.07	9.42
205289_at	bone morphogene	BMP2	5.47	5.36	5.56
218531_at	transmembrane p	TMEM134	7.92	7.93	7.96
216304_x_at	YME1-like 1 (S. ce	YME1L1	11.12	11.09	11.15
221695_s_at	mitogen-activatec	MAP3K2	6.12	6.26	6.08
209875_s_at	secreted phospho	SPP1	3.65	3.72	3.76
202345_s_at	fatty acid binding	FABP5	12.06	12.18	12.11
214605_x_at	G protein-couplec	GPR1	7.14	7.07	7.28
210222_s_at	reticulon 1	RTN1	8.92	9.07	8.99
203866_at	notchless homolo	NLE1	6.22	6.23	6.25
211386_at	hypothetical prote	MGC12488	5.14	5.15	5.20
205067_at	interleukin 1, beta	IL1B	7.09	7.19	7.19
221906_at	thioredoxin reduc	TXNRD3	7.99	8.10	8.12
210065_s_at	uroplakin 1B	UPK1B	3.89	3.84	3.92
215851_at	MDS1 and EVI1 cc	MECOM	3.67	3.79	3.84
205330_at	meningioma (disru	MN1	5.87	5.86	5.89
221268_s_at	sphingosine-1-pho	SGPP1	8.01	7.99	8.10
204936_at	mitogen-activatec	MAP4K2	5.20	5.02	5.11
212594_at	programmed cell	PDCD4	7.30	7.10	7.35
201442_s_at	ATPase, H+ transp	ATP6AP2	4.08	4.17	4.13
39402_at	interleukin 1, beta	IL1B	7.08	6.96	7.21
212391_x_at	ribosomal protein	RPS3A	15.07	15.05	15.07
207169_x_at	discoidin domain	DDR1	7.87	8.05	8.04
200677_at	pituitary tumor-tr	PTTG1IP	11.10	11.09	11.11
222338_x_at	similar to L antige	LOC646808	9.14	9.16	9.06
205700_at	hydroxysteroid (1	HSD17B6	4.59	4.49	4.55
206247_at	MHC class I polyp	MICB	9.24	9.26	9.22
218901_at	phospholipid scra	PLSCR4	8.51	8.56	8.76
218557_at	nitrilase family, m	NIT2	10.80	10.79	10.82
211367_s_at	caspase 1, apopto	CASP1	8.28	8.46	8.61
219782_s_at	zinc finger protein	ZNF771	3.76	3.84	3.72
220937_s_at	ST6 (alpha-N-acet	ST6GALNAC4	8.11	7.95	8.09
212324_s_at	vacuolar protein s	VPS13D	6.70	6.66	6.72
208696_at	chaperonin contai	CCT5	13.04	13.12	13.13
209233_at	EMG1 nucleolar p	EMG1	11.77	11.76	11.80
215501_s_at	dual specificity ph	DUSP10	6.71	6.71	6.83
207414_s_at	proprotein conver	PCSK6	7.25	7.16	7.35
203192_at	ATP-binding casse	ABCB6	7.81	7.74	7.84
201517_at	nuclear cap bindir	NCBP2	10.07	10.05	10.23
212928_at	TSPY-like 4	TSPYL4	9.40	9.53	9.53
213811_x_at	transcription facto	TCF3	10.04	10.01	9.99
208967_s_at	adenylate kinase 2	AK2	10.70	10.71	10.64
201013_s_at	phosphoribosylam	PAICS	12.37	12.42	12.47

213457_at	malignant fibrous	MFHAS1	8.37	8.28	8.30
201521_s_at	nuclear cap bindir	NCBP2	8.34	8.27	8.22
218297_at	family with seque	FAM188A	7.98	8.13	8.15
209997_x_at	pericentriolar mat	PCM1	5.82	5.91	5.93
212337_at	taurine upregulat	TUG1	8.75	8.79	8.74
221603_at	peroxisomal bioge	PEX16	6.68	6.68	6.71
211312_s_at	WNT1 inducible si	WISP1	5.54	5.49	5.31
203549_s_at	lipoprotein lipase	LPL	6.03	5.92	5.88
209397_at	malic enzyme 2, N	ME2	10.67	10.70	10.77
212940_at	collagen, type VI,	COL6A1	6.25	6.18	6.17
214352_s_at	v-Ki-ras2 Kirsten r	KRAS	9.70	9.72	9.71
221814_at	G protein-couplec	GPR124	6.23	6.17	6.31
219527_at	MOCO sulphurase	MOSC2	8.07	8.15	8.19
218087_s_at	sorbin and SH3 dc	SORBS1	5.00	4.81	5.11
204808_s_at	transmembrane p	TMEM5	10.38	10.35	10.41
203788_s_at	sema domain, imr	SEMA3C	6.53	6.75	6.75
203032_s_at	fumarate hydrata:	FH	7.58	7.62	7.66
201787_at	fibulin 1	FBLN1	8.74	8.54	8.60
219844_at	chromosome 10 c	C10orf118	4.32	4.26	4.50
220673_s_at	protein phosphata	PPP4R4	6.08	5.99	6.30
213836_s_at	WD repeat domai	WIPI1	8.87	8.81	8.97
208492_at	regulatory factor)	RFXAP	6.40	6.37	6.47
218039_at	nucleolar and spir	NUSAP1	12.09	12.19	12.25
212563_at	block of proliferat	BOP1 /// LOC7279	9.50	9.38	9.40
210529_s_at	family with seque	FAM115A	7.38	7.24	7.17
208691_at	transferrin recept	TFRC	13.17	13.15	13.20
213350_at	Ribosomal proteir	RPS11	9.27	9.26	9.27
205799_s_at	solute carrier fam	SLC3A1	4.56	4.53	4.55
219897_at	ring finger proteir	RNF122	7.48	7.39	7.37
207553_at	opioid receptor, k	OPRK1	4.08	4.07	4.03
214724_at	DIX domain conta	DIXDC1	8.00	7.83	7.89
218542_at	centrosomal prot	CEP55	11.25	11.46	11.42
210487_at	deoxynucleotidylt	DNTT	4.61	4.54	4.43
200707_at	protein kinase C s	PRKCSH	6.72	6.68	6.58
203380_x_at	splicing factor, arg	SFRS5	9.86	10.01	9.95
203409_at	damage-specific D	DDB2	7.85	7.89	7.93
203394_s_at	hairy and enhanc	HES1	9.36	9.09	9.49
217832_at	synaptotagmin bi	SYNCRIP	10.26	10.28	10.23
200911_s_at	transforming, acic	TACC1	10.52	10.48	10.57
212764_at	zinc finger E-box b	ZEB1	10.52	10.60	10.66
209265_s_at	methyltransferase	METTL3	10.21	10.34	10.24
203124_s_at	solute carrier fam	SLC11A2	8.53	8.71	8.63
203965_at	ubiquitin specific		USP20	7.28	7.26	7.27
210729_at	neuropeptide Y re	NPY2R	3.33	3.32	3.31
47560_at	latrophilin 1	LPHN1	7.28	7.40	7.48
204303_s_at	KIAA0427	KIAA0427	7.07	6.99	7.08
204698_at	interferon stimula	ISG20	6.69	6.47	6.58

218217_at	serine carboxypep	SCPEP1	9.33	9.38	9.39
202243_s_at	proteasome (pros	PSMB4	12.59	12.56	12.58
201739_at	serum/glucocortic	SGK1	8.49	8.51	8.46
205930_at	general transcript	GTF2E1	9.25	9.35	9.38
209296_at	protein phosphata	PPM1B	9.36	9.56	9.48
204218_at	chromosome 11 c	C11orf51	8.53	8.51	8.48
204877_s_at	TAO kinase 2	TAOK2	6.27	6.15	6.39
218882_s_at	WD repeat domai	WDR3	10.98	10.89	11.01
204783_at	myeloid leukemia	MLF1	8.08	8.15	8.32
212497_at	mitogen-activatec	MAPK1IP1L	6.06	6.10	5.98
220372_at	DnaJ (Hsp40) hom	DNAJC28	3.91	3.76	3.87
200059_s_at	ras homolog gene	RHOA	13.20	13.19	13.28
209651_at	transforming grov	TGFB1I1	10.06	9.91	10.01
203508_at	tumor necrosis fa	TNFRSF1B	7.35	7.06	7.23
213446_s_at	IQ motif containir	IQGAP1	9.48	9.38	9.28
212473_s_at	microtubule assoc	MICAL2	12.38	12.30	12.33
203126_at	inositol(myo)-1(or	IMPA2	8.86	8.83	8.91
205020_s_at	ADP-ribosylation f	ARL4A	10.18	9.97	10.23
201690_s_at	tumor protein D5.	TPD52	10.72	10.65	10.70
210038_at	protein kinase C, t	PRKCQ	4.83	4.66	4.65
209095_at	dihydrolipoamide	DLD	10.54	10.68	10.73
219843_at	intracisternal A pa	IPP	7.19	7.21	7.26
202215_s_at	nuclear transcript	NFYC	7.86	7.73	7.85
200745_s_at	guanine nucleotid	GNB1	12.14	12.15	12.23
206673_at	G protein-couplec	GPR176	6.57	6.66	6.41
217767_at	complement com	C3	11.89	11.68	11.75
218245_at	tsukushi small leu	TSKU	7.63	7.51	7.72
209553_at	vacuolar protein s	VPS8	7.92	7.88	7.89
219250_s_at	fibronectin leucin	FLRT3	6.88	7.53	7.10
208814_at	Heat shock 70kDa	HSPA4	8.88	9.01	8.92
208878_s_at	p21 protein (Cdc4	PAK2	8.34	8.32	8.46
212230_at	phosphatidic acid	PPAP2B	9.18	9.25	9.34
208780_x_at	VAMP (vesicle-ass	VAPA	12.38	12.40	12.41
210754_s_at	v-yes-1 Yamaguch	LYN	8.34	8.55	8.44
203777_s_at	ribosomal protein	RPS6KB2	7.62	7.63	7.70
201786_s_at	adenosine deamir	ADAR	10.92	10.92	10.84
208325_s_at	A kinase (PRKA) ar	AKAP13	7.87	7.75	7.69
204038_s_at	lysophosphatidic a	LPAR1	6.29	6.28	6.52
215681_at	KIAA1654 protein	KIAA1654	6.47	6.51	6.36
218006_s_at	zinc finger protein	ZNF22	9.21	9.29	9.34
204838_s_at	mutL homolog 3 (	MLH3	5.32	5.40	5.49
202890_at	microtubule-assoc	MAP7	3.70	3.82	3.78
210150_s_at	laminin, alpha 5	LAMA5	7.98	7.95	7.80
220035_at	nucleoporin 210kI	NUP210	5.46	5.25	5.15
200742_s_at	tripeptidyl peptida	TPP1	7.69	7.66	7.76
206185_at	crystallin, beta B1	CRYBB1	6.31	6.20	6.23
216504_s_at	---	---	5.36	5.20	5.12

213286_at	zinc finger RNA bi	ZFR	7.51	7.64	7.68
210650_s_at	piccolo (presynap	PCLO	4.77	5.24	4.99
217367_s_at	zinc fingers and h	ZHX3	8.05	8.15	8.22
203789_s_at	sema domain, imr	SEMA3C	9.52	9.66	9.92
216622_at	laminin, beta 4	LAMB4	3.38	3.29	3.41
200757_s_at	calumenin	CALU	11.19	11.23	11.24
218031_s_at	forkhead box N3	FOXN3	9.59	9.45	9.63
201654_s_at	heparan sulfate p	HSPG2	6.19	6.13	6.04
218257_s_at	UDP-glucose glyco	UGGT1	7.33	7.30	7.42
200966_x_at	aldolase A, fructo	ALDOA	13.23	13.14	13.22
201669_s_at	myristoylated ala	MARCKS	11.64	11.70	11.84
203379_at	ribosomal protein	RPS6KA1	7.58	7.52	7.55
203116_s_at	ferrochelatase	FECH	8.84	8.84	8.80
212673_at	methionyl aminop	METAP1	10.36	10.34	10.44
221938_x_at	mediator complex	MED16	7.74	7.72	7.67
206127_at	ELK3, ETS-domain	ELK3	5.53	5.48	5.63
207861_at	chemokine (C-C m	CCL22	6.55	6.63	6.45
212775_at	obscurin-like 1	OBSL1	6.92	6.92	6.86
205084_at	B-cell receptor-as	BCAP29	6.85	7.07	7.10
201798_s_at	myoferlin	MYOF	11.59	11.66	11.78
203466_at	MpV17 mitochon	MPV17	9.46	9.36	9.45
204444_at	kinesin family me	KIF11	9.44	9.64	9.58
211373_s_at	presenilin 2 (Alzh	PSEN2	7.58	7.71	7.73
216095_x_at	myotubularin rela	MTMR1	10.04	10.17	10.22
212372_at	myosin, heavy cha	MYH10	9.84	9.86	9.78
219579_at	RAB3A interacting	RAB3IL1	7.01	7.10	7.17
213742_at	splicing factor, ar	SFRS11	7.32	7.39	7.46
201899_s_at	ubiquitin-conjuga	UBE2A	10.57	10.67	10.62
206653_at	polymerase (RNA)	POLR3G	8.09	8.26	8.24
211926_s_at	myosin, heavy cha	MYH9	10.70	10.60	10.68
202082_s_at	SEC14-like 1 (S. ce	SEC14L1	11.55	11.44	11.58
205769_at	solute carrier fam	SLC27A2	7.69	7.52	7.81
201032_at	bladder cancer as	BLCAP	9.97	9.81	9.88
212249_at	phosphoinositide-	PIK3R1	9.27	9.13	9.27
213380_x_at	macrophage stim	MST1P9	6.77	6.90	6.88
202055_at	karyopherin alpha	KPNA1	9.25	9.28	9.21
203398_s_at	UDP-N-acetyl-alp	GALNT3	4.95	5.00	5.03
215177_s_at	integrin, alpha 6	ITGA6	8.41	8.66	8.64
213992_at	collagen, type IV,	COL4A6	10.04	10.13	10.12
221829_s_at	transportin 1	TNPO1	13.23	13.25	13.31
203985_at	zinc finger protei	ZNF212	8.11	8.20	8.21
214844_s_at	docking protein 5	DOK5	4.72	4.75	4.71
220122_at	multiple C2 doma	MCTP1	7.47	7.39	7.52
213492_at	collagen, type II, a	COL2A1	4.21	4.15	4.18
213003_s_at	CCAAT/enhancer	CEBPD	7.12	6.95	6.90
202840_at	NADH dehydroge	NDUFB7	8.93	8.93	8.90
206213_at	wingless-type MM	WNT10B	7.56	7.52	7.71

213643_s_at	inositol polyphosp	INPP5B	7.07	7.04	6.81
209616_s_at	carboxylesterase :	CES1	8.51	8.65	8.63
207076_s_at	argininosuccinate	ASS1	11.28	11.05	11.20
207463_x_at	protease, serine, :	PRSS3	8.97	8.88	8.89
211002_s_at	tripartite motif-cc	TRIM29	6.59	6.72	6.45
216359_at	hypothetical LOC1	LOC100129410	4.51	4.44	4.48
219054_at	chromosome 5 op	C5orf23	9.45	9.42	9.37
202182_at	K(lysine) acetyltra	KAT2A	8.95	8.91	8.84
217130_at	---	---	4.24	4.12	4.26
221522_at	ankyrin repeat do	ANKRD27	10.34	10.33	10.43
201129_at	splicing factor, arg	SFRS7	10.91	10.95	11.00
208893_s_at	dual specificity ph	DUSP6	7.86	7.80	7.88
206931_at	zinc finger proteir	ZNF141	3.44	3.48	3.36
218101_s_at	NADH dehydroger	NDUFC2	12.46	12.45	12.54
1255_g_at	guanylate cyclase	GUCA1A	3.71	3.74	3.70
202391_at	brain abundant, m	BASP1	11.88	11.81	11.90
209320_at	adenylate cyclase	ADCY3	8.56	8.47	8.42
218726_at	Holliday junction r	HJURP	10.23	10.10	10.30
213843_x_at	solute carrier fam	SLC6A8	9.42	9.43	9.31
207432_at	bestrophin 2	BEST2	6.36	6.42	6.25
214453_s_at	interferon-induce	IFI44	7.77	7.99	8.02
206592_s_at	adaptor-related p	AP3D1	10.70	10.72	10.69
214791_at	SP140 nuclear boc	SP140L	8.63	8.72	8.71
219951_s_at	chromosome 20 c	C20orf12	5.57	5.58	5.59
209702_at	fat mass and obes	FTO	9.47	9.54	9.54
220143_x_at	LUC7-like (S. cerev	LUC7L	8.63	8.52	8.57
218449_at	UFM1-specific pep	UFSP2	9.50	9.48	9.45
202565_s_at	supervillin	SVIL	8.79	8.81	8.90
201711_x_at	RAN binding prote	RANBP2	8.22	8.20	8.33
201034_at	adducin 3 (gamma	ADD3	7.74	7.90	8.06
208828_at	polymerase (DNA	POLE3	10.17	10.25	10.27
208596_s_at	UDP glucuronosyl	UGT1A1 /// UGT1A	4.99	5.00	4.91
202682_s_at	ubiquitin specific	USP4	10.32	10.33	10.35
207826_s_at	inhibitor of DNA b	ID3	9.47	9.70	9.62
206331_at	calcitonin recepto	CALCRL	4.89	4.83	4.95
213846_at	cytochrome c oxic	COX7C	10.48	10.53	10.49
213813_x_at	---	---	9.37	9.35	9.38
219681_s_at	RAB11 family inte	RAB11FIP1	8.66	8.45	8.54
219297_at	WD repeat domai	WDR44	8.26	8.23	8.36
205214_at	serine/threonine I	STK17B	7.99	8.19	8.32
205609_at	angiopoietin 1	ANGPT1	6.02	6.09	6.49
221989_at	ribosomal protein	RPL10	9.08	9.07	9.23
207717_s_at	plakophilin 2	PKP2	3.51	3.91	3.88
212534_at	zinc finger proteir	ZNF24	9.20	9.21	9.18
221583_s_at	potassium large cc	KCNMA1	7.61	7.56	7.41
204224_s_at	GTP cyclohydrolas	GCH1	7.96	8.09	8.22
205290_s_at	bone morphogene	BMP2	6.11	5.62	6.06

216814_at	---	---	3.51	3.70	3.58
213112_s_at	sequestosome 1	SQSTM1	7.84	7.80	7.91
201101_s_at	BCL2-associated t	BCLAF1	10.03	10.21	10.14
215243_s_at	gap junction prote	GJB3	5.76	5.91	6.10
209212_s_at	Kruppel-like facto	KLF5	8.81	8.84	8.99
210450_at	hypothetical prote	LOC90925	3.99	3.99	4.00
36030_at	intermediate filan	IFFO1	8.12	8.35	8.18
206767_at	RNA binding moti	RBMS3	7.15	7.15	7.26
209709_s_at	hyaluronan-media	HMMR	10.75	10.87	10.84
205395_s_at	MRE11 meiotic re	MRE11A	8.17	8.24	8.38
212931_at	transcription facto	TCF20	9.41	9.37	9.37
208772_at	ankyrin repeat an	ANKHD1 /// ANKH	10.02	9.98	9.98
213132_s_at	malonyl CoA:ACP	MCAT	8.53	8.53	8.61
207121_s_at	mitogen-activated	MAPK6	11.03	11.07	11.10
217933_s_at	leucine aminopep	LAP3	10.47	10.40	10.49
218123_at	chromosome 21 c	C21orf59	10.11	10.17	10.17
208313_s_at	splicing factor 1	SF1	9.78	9.84	9.76
204589_at	NUAK family, SNF	NUAK1	8.13	8.09	8.21
203050_at	tumor protein p53	TP53BP1	8.53	8.53	8.48
211055_s_at	inversin	INVS	3.96	3.95	3.82
212554_at	CAP, adenylate cy	CAP2	9.31	9.27	9.37
202486_at	AFG3 ATPase fam	AFG3L2	9.79	9.71	9.70
202151_s_at	UBA domain conta	UBAC1	9.63	9.61	9.53
201661_s_at	acyl-CoA syntheta	ACSL3	11.81	11.97	12.02
201315_x_at	interferon induced	IFITM2	12.22	12.19	12.22
205665_at	tetraspanin 9	TSPAN9	6.34	6.34	6.40
213008_at	Fanconi anemia, c	FANCI	9.51	9.44	9.48
219520_s_at	WWC family mem	WWC3	7.15	7.16	7.20
207686_s_at	caspase 8, apopto	CASP8	7.20	7.23	7.29
201445_at	calponin 3, acidic	CNN3	8.95	9.13	9.34
208809_s_at	chromosome 6 op	C6orf62	11.64	11.61	11.70
209123_at	quinoid dihydropt	QDPR	10.98	10.96	10.90
215100_at	chromosome 6 op	C6orf105	5.07	5.05	4.96
202886_s_at	protein phosphata	PPP2R1B	6.84	6.95	6.82
212877_at	kinesin light chain	KLC1	8.88	8.88	9.00
217766_s_at	transmembrane p	TMEM50A	9.51	9.63	9.54
220948_s_at	ATPase, Na+/K+ tr	ATP1A1	11.27	11.27	11.22
208578_at	sodium channel, v	SCN10A	4.82	4.78	4.61
201308_s_at	septin 11	11-Sep	6.52	6.61	6.68
203617_x_at	ELK1, member of	ELK1	8.40	8.41	8.34
201294_s_at	WD repeat and SC	WSB1	7.80	8.04	7.84
207574_s_at	growth arrest and	GADD45B	11.41	11.34	11.48
215713_at	---	---	3.27	3.34	3.20
218309_at	calcium/calmodul	CAMK2N1	9.33	9.30	9.46
AFFX-HUMRGE/	---	---	14.46	14.47	14.48
204301_at	kelch repeat and I	KBTBD11	6.71	6.81	6.85
201614_s_at	RuvB-like 1 (E. col	RUVBL1	10.55	10.50	10.75

210130_s_at	transmembrane 7	TM7SF2	7.18	7.00	6.92
218503_at	KIAA1797	KIAA1797	5.82	5.57	5.66
218613_at	pleckstrin and Sec	PSD3	7.57	7.54	7.78
210118_s_at	interleukin 1, alph	IL1A	9.78	9.76	9.95
220104_at	zinc finger CCCH-t	ZC3HAV1	6.82	6.57	6.68
202449_s_at	retinoid X recepto	RXRA	9.58	9.46	9.71
204581_at	CD22 molecule	CD22	5.69	5.62	5.72
216830_at	similar to hect do	LOC100290629 ///	4.49	4.46	4.65
206820_at	ArfGAP with FG re	AGFG2	6.36	6.32	6.40
208442_s_at	ataxia telangiecta	ATM	6.18	6.24	6.20
204667_at	forkhead box A1	FOXA1	5.04	5.00	4.99
200644_at	MARCKS-like 1	MARCKSL1	8.89	8.93	8.76
211638_at	immunoglobulin h	IGH@ /// IGHA1 //	4.39	4.37	4.44
216397_s_at	block of proliferat	BOP1 /// LOC7279	7.35	7.24	7.41
203562_at	fasciculation and	FEZ1	12.89	12.89	12.96
221169_s_at	histamine recepto	HRH4	4.30	4.35	4.30
204366_s_at	general transcript	GTF3C2	10.16	10.20	10.17
217254_s_at	erythropoietin	EPO	5.65	5.74	5.74
210993_s_at	SMAD family men	SMAD1	9.38	9.34	9.54
217110_s_at	mucin 4, cell surfa	MUC4	3.52	3.48	3.52
201375_s_at	protein phosphata	PPP2CB	11.96	11.96	12.03
204878_s_at	TAO kinase 2	TAOK2	6.85	6.86	6.84
203687_at	chemokine (C-X3-	CX3CL1	7.01	6.96	6.81
218587_s_at	KTEL (Lys-Tyr-Glu-	KTELC1	9.17	9.25	9.20
211209_x_at	SH2 domain conta	SH2D1A	6.15	6.17	6.23
201076_at	NHP2 non-histone	NHP2L1	10.57	10.54	10.63
219982_s_at	small EDRK-rich fa	SERF1A /// SERF1E	7.93	7.78	7.84
221563_at	dual specificity ph	DUSP10	7.77	8.07	7.90
216298_at	T cell receptor gar	TRGV5	3.49	3.45	3.47
215314_at	---	---	6.04	6.31	6.35
204533_at	chemokine (C-X-C	CXCL10	5.15	5.24	5.28
218594_at	HEAT repeat conta	HEATR1	10.36	10.29	10.41
212392_s_at	phosphodiesteras	PDE4DIP	5.19	5.12	5.21
221249_s_at	family with seque	FAM117A	6.81	6.78	6.79
209302_at	polymerase (RNA)	POLR2H	10.86	10.82	10.80
218023_s_at	family with seque	FAM53C	9.52	9.45	9.50
202262_x_at	dimethylarginine	DDAH2	6.95	6.99	7.02
204858_s_at	thymidine phosph	TYMP	8.32	8.29	8.18
210145_at	phospholipase A2	PLA2G4A	5.54	5.99	5.87
206609_at	melanoma antiger	MAGEC1	5.80	6.12	5.80
204653_at	transcription facto	TFAP2A	9.94	9.84	9.91
218665_at	frizzled homolog 4	FZD4	6.85	6.85	7.03
208877_at	p21 protein (Cdc4	PAK2	10.11	10.11	10.18
217234_s_at	ezrin	EZR	11.24	11.24	11.04
206307_s_at	forkhead box D1	FOXD1	10.21	9.96	10.22
211307_s_at	Fc fragment of IgA	FCAR	5.29	4.97	5.21
205525_at	caldesmon 1	CALD1	6.19	6.36	6.45

215410_at	PMS2 postmeiotic	LOC441259 /// PM	5.71	5.70	5.53
220645_at	family with seque	FAM55D	4.47	4.56	4.53
202838_at	TRAF-type zinc fin	TRAFD1	7.37	7.72	7.74
215457_at	---	---	3.57	3.56	3.60
219855_at	nudix (nucleoside	NUDT11	8.88	8.99	8.94
213221_s_at	salt-inducible kina	SIK2	7.14	7.31	7.36
213699_s_at	tyrosine 3-monoo	YWHAQ	13.81	13.87	13.84
215887_at	zinc finger protein	ZNF277	6.87	6.84	6.84
204717_s_at	solute carrier fam	SLC29A2	7.42	7.11	7.20
210482_x_at	mitogen-activated	MAP2K5	7.43	7.45	7.50
203001_s_at	stathmin-like 2	STMN2	3.90	3.79	3.83
200619_at	splicing factor 3b,	SF3B2	10.69	10.61	10.58
200824_at	glutathione S-tran	GSTP1	11.77	11.68	11.64
201378_s_at	ubiquitin associat	UBAP2L	8.00	8.12	7.97
206135_at	suppression of tur	ST18	4.37	4.66	4.20
202669_s_at	ephrin-B2	EFNB2	7.53	7.66	7.37
204540_at	eukaryotic transla	EEF1A2	8.12	8.04	8.09
206521_s_at	general transcript	GTF2A1	6.76	6.65	6.84
212104_s_at	RNA binding moti	RBM9	11.40	11.22	11.36
212339_at	erythrocyte meml	EPB41L1	5.67	5.54	5.59
205837_s_at	glycophorin A (MN	GYPA /// GYPB	3.38	3.26	3.22
215971_at	---	---	4.20	4.09	4.00
215537_x_at	dimethylarginine	DDAH2	7.02	6.96	7.03
206519_x_at	sialic acid binding	SIGLEC6	3.15	3.01	3.09
202383_at	lysine (K)-specific	KDM5C	7.04	6.99	6.94
204519_s_at	plasma membrane	PLLP	5.97	5.75	5.49
216442_x_at	fibronectin 1	FN1	13.54	13.53	13.62
219627_at	zinc finger family	ZNF767	6.78	6.89	6.60
210872_x_at	growth arrest-spe	GAS7	4.58	4.45	4.71
204977_at	DEAD (Asp-Glu-Ala	DDX10	9.76	9.67	9.83
203637_s_at	midline 1 (Opitz/B	MID1	8.13	8.26	8.33
209301_at	carbonic anhydras	CA2	6.02	6.07	6.11
206429_at	coagulation factor	F2RL1	5.74	5.77	5.68
206298_at	Rho GTPase activa	ARHGAP22	8.06	7.94	8.04
222081_at	Sirtuin (silent mat	SIRT5	5.52	5.66	5.61
210111_s_at	PNAS-119	LOC100287552	10.08	10.12	10.20
208627_s_at	Y box binding prot	YBX1	13.29	13.34	13.34
215936_s_at	KIAA1033	KIAA1033	7.26	7.41	7.54
202020_s_at	LanC lantibiotic sy	LANCL1	11.58	11.73	11.78
221536_s_at	large subunit GTP	LSG1	10.04	9.85	10.04
214442_s_at	protein inhibitor c	PIAS2	7.41	7.49	7.50
201334_s_at	Rho guanine nucle	ARHGEF12	11.76	11.69	11.78
219217_at	asparaginyl-tRNA	NARS2	9.41	9.43	9.54
206661_at	DBF4 homolog B (	DBF4B	5.32	5.39	5.35
50400_at	polyamine oxidase	PAOX	5.00	5.00	4.91
211317_s_at	CASP8 and FADD-	CFLAR	7.47	7.39	7.52
221447_s_at	glycosyltransferas	GLT8D2	8.03	8.04	8.10

203582_s_at	RAB4A, member F	RAB4A	8.47	8.61	8.68
203545_at	asparagine-linked	ALG8	9.35	9.61	9.57
201496_x_at	myosin, heavy cha	MYH11	4.82	4.67	4.77
210791_s_at	Rho GTPase activa	ARHGAP32	6.43	6.41	6.45
209457_at	dual specificity ph	DUSP5	10.77	10.72	10.99
221933_at	neuroligin 4, X-linl	NLGN4X	3.76	3.69	3.81
208829_at	TAP binding prote	TAPBP	8.81	8.81	8.92
202422_s_at	acyl-CoA syntheta	ACSL4	7.61	7.59	7.73
212763_at	calmodulin regula	CAMSAP1L1	8.17	8.22	8.13
213367_at	zinc finger family	ZNF783	6.94	6.92	6.85
216962_at	RPA interacting pr	RPAIN	5.37	5.35	5.40
208248_x_at	amyloid beta (A4)	APLP2	10.11	10.16	10.15
202267_at	laminin, gamma 2	LAMC2	5.21	5.66	5.52
217982_s_at	mortality factor 4	MORF4L1	13.28	13.31	13.33
210095_s_at	insulin-like growth	IGFBP3	7.24	6.84	6.94
219660_s_at	ATPase, aminopho	ATP8A2	4.30	4.33	4.34
49111_at	---	---	5.71	5.79	5.78
211048_s_at	protein disulfide is	PDIA4	10.76	10.85	10.86
212173_at	adenylate kinase 2	AK2	7.60	7.77	7.74
201474_s_at	integrin, alpha 3 (a	ITGA3	7.94	7.87	8.01
212922_s_at	SET and MYND do	SMYD2	9.41	9.40	9.39
202137_s_at	zinc finger, MYND	ZMYND11	6.99	7.02	7.11
201014_s_at	phosphoribosylam	PAICS	11.46	11.50	11.56
214217_at	glutamate recepto	GRM5	4.02	4.07	3.94
201117_s_at	carboxypeptidase	CPE	4.17	4.33	4.22
212379_at	phosphoribosylgly	GART	8.52	8.43	8.48
210639_s_at	ATG5 autophagy r	ATG5	8.86	8.97	9.10
205003_at	dedicator of cytok	DOCK4	7.31	7.48	7.51
218018_at	pyridoxal (pyridox	PDXK	9.30	9.22	9.22
218286_s_at	ring finger protein	RNF7	10.49	10.60	10.51
209309_at	alpha-2-glycoprot	AZGP1	4.39	4.35	4.51
218209_s_at	regulation of nucl	RPRD1A	9.10	9.12	9.24
200641_s_at	tyrosine 3-monoo	YWHAZ	11.00	11.02	10.95
215069_at	N-myristoyltransf	NMT2	6.62	6.61	6.69
214011_s_at	NOP16 nucleolar p	NOP16	12.07	12.04	12.01
200841_s_at	glutamyl-prolyl-tR	EPRS	9.57	9.62	9.61
209184_s_at	insulin receptor su	IRS2	9.63	9.48	9.63
211126_s_at	cysteine and glyci	CSRP2	8.86	8.89	8.99
202219_at	solute carrier fam	SLC6A8	9.65	9.69	9.74
219838_at	tetratricopeptide	TTC23	7.85	7.90	7.97
204658_at	transformer 2 alpl	TRA2A	10.42	10.51	10.50
221386_at	olfactory recepto	OR3A2	4.42	4.43	4.34
203531_at	cullin 5	CUL5	9.32	9.54	9.44
217715_x_at	---	---	6.67	6.66	6.60
207030_s_at	cysteine and glyci	CSRP2	9.66	9.80	9.75
200906_s_at	palladin, cytoskel	PALLD	11.24	11.13	11.16
213997_at	family with seque	FAM189A1	5.31	5.36	5.38

215933_s_at	hematopoietically	HHEX	4.87	4.99	4.58
201649_at	ubiquitin-conjugat	UBE2L6	9.06	8.98	8.99
205726_at	diaphanous homo	DIAPH2	7.47	7.60	7.54
216021_s_at	glycine receptor, a	GLRA3	4.07	4.04	4.03
205189_s_at	Fanconi anemia, c	FANCC	8.21	8.37	8.30
209116_x_at	hemoglobin, beta	HBB	6.72	6.84	6.63
215377_at	C-terminal binding	CTBP2	4.96	5.05	4.92
219979_s_at	chromosome 11 c	C11orf73	9.19	9.21	9.30
214373_at	---	---	6.70	6.62	6.56
202809_s_at	chromosome 10 c	C10orf26	8.54	8.54	8.48
201318_s_at	myosin, light chai	MYL12A /// MYL12	12.68	12.70	12.77
218854_at	dermatan sulfate	DSE	9.90	9.89	10.01
219013_at	UDP-N-acetyl-alph	GALNT11	8.98	9.13	9.02
220449_at	hypothetical prote	MGC5566	6.86	7.01	6.76
221945_at	F-box protein 41	FBXO41	6.19	6.19	6.31
216867_s_at	platelet-derived g	PDGFA	10.13	10.09	9.95
213085_s_at	WW and C2 doma	WWC1	8.88	8.60	8.69
215723_s_at	phospholipase D1	PLD1	3.77	3.65	3.80
212796_s_at	TBC1 domain fam	TBC1D2B	8.87	8.71	8.76
200051_at	squamous cell car	SART1	8.44	8.39	8.47
210692_s_at	solute carrier fam	SLC43A3	4.65	4.65	4.53
201519_at	translocase of out	TOMM70A	10.57	10.55	10.72
219478_at	WAP four-disulfid	WFDC1	4.17	4.26	4.15
210235_s_at	protein tyrosine p	PPFIA1	7.08	7.26	7.19
202309_at	methylenetetrahy	MTHFD1	11.90	11.92	11.90
220358_at	basic leucine zipp	BATF3	8.12	8.19	8.26
210001_s_at	suppressor of cyto	SOCS1	7.57	7.40	7.55
205787_x_at	zinc finger CCCH-t	ZC3H11A	8.63	8.58	8.56
210910_s_at	POM (POM121 ho	POMZP3	6.46	6.44	6.39
206760_s_at	Fc fragment of IgE	FCER2	5.01	4.99	5.04
215089_s_at	RNA binding moti	RBM10	9.51	9.41	9.54
212479_s_at	required for meio	RMND5A	9.26	9.10	9.14
218540_at	thiamine triphosp	THTPA	6.83	6.81	6.89
202892_at	nitrilase 1	NIT1	9.48	9.52	9.53
206225_at	zinc finger protein	ZNF507	4.59	4.55	4.61
221845_s_at	ClpB caseinolytic p	CLPB	7.10	7.28	7.15
216627_s_at	UDP-Gal:betaGlcN	B4GALT1	4.90	4.87	4.86
213407_at	PH domain and le	PHLPP2	7.82	7.89	7.88
212077_at	caldesmon 1	CALD1	11.74	11.83	12.05
204119_s_at	adenosine kinase	ADK	10.72	10.74	10.77
213891_s_at	transcription facto	TCF4	9.07	9.08	9.11
209209_s_at	fermitin family ho	FERMT2	8.25	8.24	8.41
219884_at	LIM homeobox 6	LHX6	6.73	6.56	6.52
203135_at	TATA box binding	TBP	9.19	9.17	9.15
203620_s_at	FCH and double SI	FCHSD2	6.94	7.00	7.10
218986_s_at	DEAD (Asp-Glu-Ala	DDX60	7.30	7.37	7.53
208370_s_at	regulator of calcin	RCAN1	10.83	10.74	10.89

210018_x_at	mucosa associate	MALT1	8.44	8.53	8.51
200814_at	proteasome (pros	PSME1	11.52	11.51	11.55
213222_at	phospholipase C, I	PLCB1	7.60	7.52	7.81
200951_s_at	cyclin D2	CCND2	6.04	5.93	5.85
202949_s_at	four and a half LIN	FHL2	9.03	9.01	9.11
217684_at	thymidylate synth	TYMS	4.31	4.32	4.35
210517_s_at	A kinase (PRKA) ai	AKAP12	7.65	7.59	7.65
209883_at	glycosyltransferas	GLT25D2	6.45	6.44	6.18
211740_at	islet cell autoantig	ICA1	3.51	3.55	3.58
210455_at	chromosome 10 c	C10orf28	4.53	4.59	4.48
217826_s_at	ubiquitin-conjugat	UBE2J1	9.00	9.10	9.13
200857_s_at	nuclear receptor c	NCOR1	7.87	7.82	7.89
212276_at	lipin 1	LPIN1	10.19	10.32	10.18
212270_x_at	ribosomal protein	RPL17	14.49	14.47	14.56
218471_s_at	Bardet-Biedl synd	BBS1	7.13	7.20	7.06
211385_x_at	sulfotransferase fa	SULT1A2	6.98	7.05	7.04
202368_s_at	translocation assc	TRAM2	7.48	7.44	7.48
214474_at	protein kinase, AN	PRKAB2	6.67	6.64	6.59
202633_at	topoisomerase (D	TOPBP1	9.98	10.05	10.13
209893_s_at	fucosyltransferase	FUT4	5.82	5.87	6.01
209593_s_at	torsin family 1, m	TOR1B	8.84	8.92	8.86
212905_at	cleavage stimulati	CSTF2T	8.98	9.09	9.09
200928_s_at	RAB14, member F	RAB14	4.62	4.79	4.80
204045_at	transcription elon	TCEAL1	8.71	8.79	8.98
205924_at	RAB3B, member F	RAB3B	5.39	5.41	5.40
211874_s_at	MYST histone ace	MYST4	7.52	7.57	7.69
203482_at	family with seque	FAM178A	8.14	8.10	8.12
222271_at	---	---	4.86	5.04	5.03
211429_s_at	serpin peptidase i	SERPINA1	5.20	5.13	5.35
211050_x_at	similar to hCG173	LOC100134822 ///	5.97	5.83	5.98
218326_s_at	leucine-rich repea	LGR4	6.95	7.28	7.34
201499_s_at	ubiquitin specific	USP7	10.38	10.33	10.27
201717_at	mitochondrial ribo	MRPL49	9.66	9.76	9.67
207359_at	calcium/calmodul	CAMKK2	6.24	6.12	6.12
205203_at	phospholipase D1	PLD1	5.81	5.92	5.97
208461_at	hypermethylated	HIC1	5.05	5.12	5.18
217475_s_at	LIM domain kinas	LIMK2	4.95	5.07	4.83
222022_at	Deltex homolog 3	DTX3	6.26	6.10	6.28
210986_s_at	tropomyosin 1 (al	TPM1	12.44	12.45	12.60
219220_x_at	mitochondrial ribo	MRPS22	10.94	10.91	10.93
211827_s_at	potassium voltage	KCND3	3.66	3.70	3.79
215178_x_at	N-acylethanolami	NAAA	7.00	6.96	7.10
201791_s_at	7-dehydrocholest	DHCR7	10.09	9.97	10.16
215404_x_at	fibroblast growth	FGFR1	6.83	6.82	6.86
203214_x_at	cyclin-dependent	CDK1	10.84	10.92	10.97
201602_s_at	protein phosphata	PPP1R12A	8.76	8.82	8.96
204128_s_at	replication factor	RFC3	8.19	8.24	8.31

221218_s_at	thiamin pyrophos	TPK1	8.63	8.83	8.74
217846_at	glutaminyl-tRNA s	QARS	11.28	11.25	11.31
205661_s_at	FAD1 flavin adeni	FLAD1	7.54	7.67	7.62
220750_s_at	leucine proline-en	LEPRE1	8.15	8.12	8.23
205492_s_at	dihydropyrimidina	DPYSL4	5.18	5.17	5.15
216682_s_at	Family with seque	FAM48A	6.54	6.63	6.52
203833_s_at	trans-golgi netwo	TGOLN2	8.17	8.20	8.15
209603_at	GATA binding pro	GATA3	6.80	6.72	6.98
214172_x_at	RYK receptor-like	RYK	9.21	9.15	9.21
203810_at	DnaJ (Hsp40) hom	DNAJB4	9.19	9.50	9.51
203401_at	phosphoribosyl py	PRPS2	10.01	10.12	10.18
220399_at	non-protein codin	NCRNA00115	5.74	5.67	5.63
212294_at	guanine nucleotid	GNG12	12.73	12.73	12.76
202085_at	tight junction prot	TJP2	10.36	10.28	10.39
209555_s_at	CD36 molecule (th	CD36	3.51	3.44	3.41
210664_s_at	tissue factor path	TFPI	9.47	9.40	9.59
214701_s_at	fibronectin 1	FN1	7.85	7.79	7.93
214164_x_at	carbonic anhydras	CA12	10.75	10.80	10.68
179_at	postmeiotic segre	PMS2L11	8.69	8.69	8.73
207396_s_at	asparagine-linked	ALG3	8.70	8.59	8.54
203568_s_at	tripartite motif-co	TRIM38	8.05	8.18	8.11
201319_at	myosin, light chai	MYL12A	11.56	11.59	11.70
221552_at	abhydrolase doma	ABHD6	7.76	7.81	7.84
222159_at	---	---	5.82	5.88	5.76
204900_x_at	Sin3A-associated	SAP30	10.66	10.66	10.84
204389_at	monoamine oxida	MAOA	7.70	7.58	7.67
207592_s_at	hyperpolarization	HCN2	4.88	4.86	4.71
208109_s_at	chromosome 15 c	C15orf5	4.47	4.48	4.47
209725_at	UTP20, small subu	UTP20	9.73	9.80	9.77
218521_s_at	ubiquitin-conjugat	UBE2W	8.30	8.30	8.34
209261_s_at	nuclear receptor s	NR2F6	6.15	6.10	6.08
204162_at	NDC80 homolog, I	NDC80	10.29	10.29	10.54
202323_s_at	acyl-CoA binding c	ACBD3	7.69	7.92	7.82
202257_s_at	CD2 (cytoplasmic	CD2BP2	8.89	8.80	8.89
203082_at	BMS1 homolog, ri	BMS1	9.78	9.75	9.77
216262_s_at	TGFB-induced fact	TGIF2	8.80	8.68	8.77
221212_x_at	polybromo 1	PBRM1	3.06	3.02	3.10
209470_s_at	glycoprotein M6A	GPM6A	5.27	5.00	5.34
219148_at	PDZ binding kinas	PBK	11.78	11.93	11.88
212624_s_at	chimerin (chimaer	CHN1	10.72	10.81	10.78
213461_at	nudix (nucleoside	NUDT21	7.74	7.74	7.88
203581_at	RAB4A, member R	RAB4A	8.74	8.82	8.85
218081_at	chromosome 20 c	C20orf27	7.99	7.88	7.85
212823_s_at	pleckstrin homolo	PLEKHG3	7.71	7.67	7.68
215407_s_at	astrotactin 2	ASTN2	6.26	6.15	6.23
205404_at	hydroxysteroid (1	HSD11B1	5.77	5.54	5.57
201353_s_at	bromodomain adj	BAZ2A	6.85	7.05	6.95

203528_at	sema domain, imr	SEMA4D	5.64	5.77	5.61
214912_at	---	---	3.52	3.60	3.64
216122_at	---	---	5.23	5.30	5.36
217682_at	---	---	7.07	7.01	7.19
213511_s_at	myotubularin rela	MTMR1	9.87	9.94	10.00
202365_at	unc-119 homolog	UNC119B	9.80	9.84	9.83
215109_at	hypothetical prot	LOC100288007	5.02	5.20	5.14
203287_at	ladinin 1	LAD1	6.12	6.22	6.18
211792_s_at	cyclin-dependent	CDKN2C	8.49	8.46	8.48
218140_x_at	signal recognition	SRPRB	11.03	11.11	11.10
203570_at	lysyl oxidase-like 1	LOXL1	9.08	9.02	9.02
204899_s_at	Sin3A-associated	SAP30	8.77	8.80	8.79
216232_s_at	GCN1 general con	GCN1L1	8.47	8.43	8.46
217969_at	chromosome 11 c	C11orf2	9.87	9.71	9.74
206170_at	adrenergic, beta-2	ADRB2	9.18	9.13	9.33
202357_s_at	complement facto	CFB	6.63	6.45	6.08
215165_x_at	uridine monophos	UMPS	10.20	10.23	10.23
203797_at	visinin-like 1	VSNL1	6.38	6.28	6.46
201398_s_at	translocation asso	TRAM1	11.61	11.75	11.83
208243_s_at	cannabinoid recep	CNR1	5.94	6.08	6.09
219957_at	RUN and FYVE do	RUFY2	6.77	6.82	6.91
208810_at	DnaJ (Hsp40) hom	DNAJB6 /// TMEM	9.51	9.64	10.00
220771_at	melanoma antige	LOC51152	4.57	4.59	4.65
213107_at	TRAF2 and NCK in	TNIK	6.31	6.55	6.31
203659_s_at	tripartite motif-co	TRIM13	8.58	8.65	8.60
209094_at	dimethylarginine	DDAH1	11.03	11.15	11.16
201783_s_at	v-rel reticuloendo	RELA	8.92	8.84	8.94
207554_x_at	thromboxane A2 r	TBXA2R	6.73	6.78	6.64
218866_s_at	polymerase (RNA)	POLR3K	10.29	10.16	10.24
205315_s_at	syntrophin, beta 2	SNTB2	9.22	9.25	9.14
201456_s_at	budding uninhibit	BUB3	11.73	11.69	11.77
201578_at	podocalyxin-like	PODXL	7.13	7.31	7.28
212944_at	solute carrier fam	SLC5A3	10.15	10.38	10.27
209395_at	chitinase 3-like 1 (	CHI3L1	3.75	3.89	3.99
213357_at	general transcript	GTF2H5	10.85	10.89	11.00
214170_x_at	fumarate hydratas	FH	10.66	10.70	10.68
211769_x_at	serine incorporato	SERINC3	10.08	10.03	10.17
202177_at	growth arrest-spe	GAS6	8.37	8.42	8.35
202512_s_at	ATG5 autophagy r	ATG5	8.47	8.50	8.63
218013_x_at	dynactin 4 (p62)	DCTN4	8.77	8.68	8.78
211241_at	annexin A2 pseud	ANXA2P3	7.97	7.91	7.96
215767_at	zinc finger protein	ZNF804A	6.90	7.08	7.07
206993_at	ATP synthase, H+	ATP5S	7.15	7.28	7.19
219622_at	RAB20, member R	RAB20	7.39	7.47	7.42
205264_at	CD3e molecule, e	CD3EAP	9.14	8.95	9.20
221685_s_at	coiled-coil domain	CCDC99	10.79	10.76	10.93
212771_at	family with seque	FAM171A1	9.78	9.84	9.87

217898_at	chromosome 15 c	C15orf24	11.13	11.07	11.14
210247_at	synapsin II	SYN2	4.51	4.38	4.50
214056_at	---	---	6.89	7.04	7.08
200819_s_at	ribosomal protein	RPS15	14.61	14.59	14.62
211719_x_at	fibronectin 1	FN1	13.41	13.41	13.51
202936_s_at	SRY (sex determin	SOX9	5.42	5.32	5.48
213189_at	MYC induced nucl	MINA	9.69	9.62	9.76
204362_at	src kinase associa	SKAP2	10.25	10.27	10.36
209692_at	eyes absent homo	EYA2	7.72	7.59	7.56
204567_s_at	ATP-binding casse	ABCG1	8.07	7.59	8.21
216626_at	---	---	4.75	4.74	4.66
203673_at	thyroglobulin	TG	6.09	6.19	6.13
213772_s_at	golgi-associated, g	GGA2	6.63	6.64	6.76
212642_s_at	human immunode	HIVEP2	9.24	9.30	9.34
217125_at	---	---	4.78	4.79	4.80
202820_at	transcription elon	TCEB3	10.10	9.99	10.36
204675_at	steroid-5-alpha-re	SRD5A1	8.55	8.54	8.60
203647_s_at	ferredoxin 1	FDX1	9.51	9.52	9.70
212634_at	KIAA0776	KIAA0776	9.28	9.40	9.43
209769_s_at	glycoprotein Ib (p	GP1BB	7.00	6.79	6.87
209444_at	RAP1, GTP-GDP di	RAP1GDS1	9.72	9.64	9.70
213776_at	hypothetical prot	LOC157562	7.02	6.95	6.94
221619_s_at	mitochondrial car	MTCH1	11.92	11.85	11.86
220169_at	transmembrane p	TMEM156	7.97	8.17	8.27
222347_at	hypothetical prot	LOC644450	6.62	6.50	6.60
208764_s_at	ATP synthase, H+	ATP5G2	12.35	12.25	12.29
203454_s_at	ATX1 antioxidant	ATOX1	10.13	10.13	10.12
200665_s_at	secreted protein,	SPARC	11.17	11.25	11.24
203212_s_at	myotubularin rela	MTMR2	7.85	7.98	7.74
206756_at	carbohydrate (N-a	CHST7	7.55	7.56	7.54
203909_at	solute carrier fam	SLC9A6	9.72	9.72	9.71
205546_s_at	tyrosine kinase 2	TYK2	8.59	8.69	8.63
210021_s_at	cyclin O	CCNO	7.77	7.79	7.74
217956_s_at	enolase-phosphat	ENOPH1	11.17	11.14	11.21
209646_x_at	aldehyde dehydro	ALDH1B1	7.55	7.46	7.54
201924_at	AF4/FMR2 family,	AFF1	10.32	10.07	10.34
210951_x_at	RAB27A, member	RAB27A	8.52	8.64	8.62
202252_at	RAB13, member R	RAB13	11.77	11.77	11.77
213306_at	multiple PDZ dom	MPDZ	7.74	7.88	8.05
212466_at	sprouty-related, E	SPRED2	5.49	5.25	5.28
205262_at	potassium voltage	KCNH2	7.59	7.43	7.55
203245_s_at	non-protein codin	NCRNA00094	7.97	8.05	8.05
207237_at	potassium voltage	KCNA3	4.11	3.97	4.03
213757_at	Eukaryotic transla	EIF5A	9.30	9.28	9.23
204881_s_at	UDP-glucose cera	UGCG	10.37	10.52	10.45
204778_x_at	homeobox B7	HOXB7	6.76	6.80	6.67
209355_s_at	phosphatidic acid	PPAP2B	8.43	8.49	8.61

205057_s_at	iduronidase, alpha	IDUA	4.98	4.90	4.91
212280_x_at	ATG4 autophagy r	ATG4B	7.46	7.62	7.66
219735_s_at	transcription facto	TFCP2L1	5.02	4.93	4.95
200947_s_at	glutamate dehydr	GLUD1	10.69	10.60	10.67
218781_at	structural mainter	SMC6	9.65	9.70	9.84
212302_at	Rtf1, Paf1/RNA pc	RTF1	8.18	8.18	8.21
201095_at	death-associated	DAP	10.26	10.24	10.21
207827_x_at	synuclein, alpha (r	SNCA	7.93	8.12	8.15
203296_s_at	ATPase, Na+/K+ tr	ATP1A2	6.88	6.71	6.72
215652_at	succinate dehydrc	SDHD	4.90	4.87	4.77
218051_s_at	5'-nucleotidase dc	NT5DC2	7.54	7.60	7.54
201724_s_at	UDP-N-acetyl-alph	GALNT1	9.77	9.87	9.95
208913_at	golgi-associated, g	GGA2	5.99	6.14	6.24
214725_at	chromosome 8 op	C8orf84	3.14	3.04	3.06
204548_at	steroidogenic acu	STAR	4.67	4.65	4.57
203031_s_at	uroporphyrinoger	UROS	8.36	8.47	8.48
210256_s_at	phosphatidylinosi	PIP5K1A	6.06	5.91	5.96
202023_at	ephrin-A1	EFNA1	8.06	8.20	7.91
218888_s_at	neuropilin (NRP) a	NETO2	10.70	10.84	10.79
209474_s_at	ectonucleoside tri	ENTPD1	3.59	3.81	3.64
218511_s_at	pyridoxamine 5'-p	PNPO	9.05	9.01	9.09
208709_s_at	nardilysin (N-argir	NRD1	10.11	10.09	10.09
206811_at	adenylate cyclase	ADCY8	4.48	4.34	4.34
219863_at	hect domain and I	HERC5	4.39	4.46	4.23
205326_at	receptor (G protei	RAMP3	5.71	5.74	5.66
201280_s_at	disabled homolog	DAB2	9.01	9.04	9.28
214797_s_at	cyclin-dependent	CDK18	5.61	5.48	5.62
202645_s_at	multiple endocrin	MEN1	8.16	8.17	8.24
207723_s_at	killer cell lectin-lik	KLRC3	5.62	5.46	5.68
210731_s_at	lectin, galactoside	LGALS8	5.45	5.58	5.41
201085_s_at	SON DNA binding	SON	9.77	9.80	9.71
209629_s_at	nuclear transport	NXT2	6.24	6.39	6.13
204037_at	lysophosphatidic a	LPAR1	7.64	7.53	7.75
221956_at	leucine-rich repea	LRCH4	6.80	6.80	6.82
207688_s_at	---	---	8.21	8.28	8.31
211329_x_at	hemochromatosis	HFE	6.46	6.43	6.31
215919_s_at	mitochondrial ribo	MRPS11	5.64	5.53	5.65
203312_x_at	ADP-ribosylation f	ARF6	10.01	10.04	10.07
201506_at	transforming grov	TGFBI	13.65	13.57	13.62
205087_at	RWD domain cont	RWDD3	8.61	8.70	8.76
221780_s_at	DEAD (Asp-Glu-Ala	DDX27	9.69	9.75	9.65
205338_s_at	dopachrome taute	DCT	4.45	4.52	4.52
205402_x_at	protease, serine, 2	PRSS2	7.67	7.52	7.66
204212_at	acyl-CoA thioester	ACOT8	7.65	7.62	7.55
212518_at	phosphatidylinosi	PIP5K1C	7.36	7.38	7.32
204132_s_at	forkhead box O3 /	FOXO3 /// FOXO3I	8.86	8.89	8.89
201900_s_at	aldo-keto reducta	AKR1A1	10.11	10.12	10.18

207980_s_at	Cbp/p300-interac	CITED2	7.41	7.48	7.74
201675_at	A kinase (PRKA) a	AKAP1	10.23	10.20	10.32
204087_s_at	solute carrier fam	SLC5A6	8.24	8.33	8.36
217716_s_at	Sec61 alpha 1 sub	SEC61A1	10.21	10.11	10.17
213155_at	WSC domain cont	WSCD1	6.46	6.60	6.72
205141_at	angiogenin, ribon	ANG	6.42	6.37	6.19
207001_x_at	TSC22 domain fan	TSC22D3	10.58	10.30	10.59
218809_at	pantothenate kin	PANK2	9.83	9.83	9.76
214596_at	cholinergic recept	CHRM3	7.03	7.06	6.89
216873_s_at	ATPase, class I, ty	ATP8B2	8.80	8.76	8.78
211573_x_at	transglutaminase	TGM2	7.14	7.14	6.99
213421_x_at	protease, serine,	PRSS3	9.19	9.04	9.04
201753_s_at	adducin 3 (gamma	ADD3	6.84	6.89	7.18
206571_s_at	mitogen-activatec	MAP4K4	8.90	8.90	8.89
32502_at	glycerophosphodi	GDPD5	7.65	7.73	7.66
207180_s_at	HIV-1 Tat interact	HTATIP2	8.97	8.92	9.08
211589_at	promyelocytic leu	PML	5.01	4.95	4.98
202656_s_at	SERTA domain co	SERTAD2	10.01	9.97	10.06
203799_at	CD302 molecule	CD302	3.45	3.49	3.46
206138_s_at	phosphatidylinosi	PI4KB	9.15	9.13	9.09
219392_x_at	proline rich 11	PRR11	11.48	11.63	11.62
205040_at	orosomucoid 1	ORM1	6.13	5.94	5.78
56256_at	SID1 transmembr	SIDT2	8.40	8.36	8.48
208315_x_at	TNF receptor-assc	TRAF3	7.87	7.89	8.03
218451_at	CUB domain cont	CDCP1	10.25	10.16	10.13
210907_s_at	programmed cell	PDCD10	11.97	12.02	12.10
212975_at	DENN/MADD don	DENND3	7.53	7.49	7.52
208625_s_at	eukaryotic transla	EIF4G1	10.33	10.28	10.36
211981_at	collagen, type IV,	COL4A1	6.26	6.35	6.47
212703_at	talin 2	TLN2	7.34	7.18	7.32
212216_at	prolyl endopeptid	PREPL	8.11	8.29	8.38
217811_at	selenoprotein T	SELT	10.58	10.64	10.60
205198_s_at	ATPase, Cu++ trar	ATP7A	6.33	6.34	6.32
219810_at	valosin containing	VCPIP1	5.36	5.33	5.40
215661_at	microtubule assoc	MAST2	6.06	5.92	5.88
205933_at	SET binding prote	SETBP1	6.68	6.77	7.00
207382_at	tumor protein p6	TP63	6.63	6.20	6.32
201399_s_at	translocation assc	TRAM1	9.59	9.75	9.65
205732_s_at	nuclear receptor c	NCOA2	3.68	3.58	3.65
204015_s_at	dual specificity ph	DUSP4	7.76	7.72	7.66
221573_at	chromosome 7 op	C7orf25 /// PSMA	7.08	7.23	7.11
54632_at	thyroid adenoma	THADA	9.06	9.00	8.97
211468_s_at	RecQ protein-like	RECQL5	6.03	6.02	6.06
218810_at	zinc finger CCCH-t	ZC3H12A	6.88	7.23	6.92
213804_at	inositol polyphos	INPP5B	8.21	8.10	8.20
220675_s_at	patatin-like phosp	PNPLA3	6.55	6.77	6.58
206555_s_at	THUMP domain c	THUMPD1	9.97	10.03	10.20

212422_at	programmed cell (	PDCD11	8.67	8.73	8.77
201291_s_at	topoisomerase (D	TOP2A	12.84	12.88	13.04
215008_at	tolloid-like 2	TLL2	3.49	3.53	3.47
221245_s_at	frizzled homolog 5	FZD5	7.16	7.18	7.15
216624_s_at	myeloid/lymphoic	MLL	5.25	5.11	5.18
203627_at	insulin-like growtl	IGF1R	7.80	7.79	7.79
215485_s_at	intercellular adhe:	ICAM1	6.88	6.74	7.00
201878_at	ariadne homolog,	ARIH1	8.00	8.01	7.99
217232_x_at	hemoglobin, beta	HBB	7.29	7.54	7.45
221221_s_at	kelch-like 3 (Droso	KLHL3	6.02	6.17	6.17
203066_at	carbohydrate (N-a	CHST15	7.16	7.37	7.47
212093_s_at	microtubule assoc	MTUS1	3.95	3.95	4.23
208843_s_at	golgi reassembly s	GORASP2	10.28	10.40	10.34
207724_s_at	spastin	SPAST	6.29	6.11	6.25
206720_at	mannosyl (alpha-1	MGAT5	4.96	5.18	4.99
215836_s_at	protocadherin gar	PCDHGA1 /// PCDI	7.18	7.27	7.13
217529_at	ORAI calcium rele	ORAI2	7.24	7.29	7.32
213867_x_at	actin, beta	ACTB	14.26	14.32	14.34
218805_at	GTPase, IMAP fan	GIMAP5	6.35	6.29	6.18
203091_at	far upstream elen	FUBP1	10.26	10.33	10.34
219503_s_at	transmembrane p	TMEM40	6.46	6.19	6.38
218834_s_at	transmembrane p	TMEM132A	7.60	7.49	7.57
218436_at	SIL1 homolog, enc	SIL1	8.83	8.70	8.83
207467_x_at	calpastatin	CAST	10.66	10.69	10.69
216281_at	dicer 1, ribonucle	DICER1	4.31	4.33	4.21
201623_s_at	aspartyl-tRNA syn	DARS	12.18	12.17	12.22
209024_s_at	synaptotagmin bi	SYNCRIP	11.17	11.16	11.13
207702_s_at	membrane associ	MAGI2	3.65	3.61	3.61
221879_at	calmodulin-like 4	CALML4	7.91	8.08	8.12
214082_at	carbonic anhydras	CA5B	5.60	5.66	5.59
201969_at	nuclear autoantig	NASP	8.67	8.71	8.53
220254_at	low density lipopr	LRP12	6.26	6.50	6.50
203963_at	carbonic anhydras	CA12	10.62	10.76	10.74
219886_at	centrosomal prot	CEP97	4.98	4.82	4.89
217392_at	bromodomain adj	BAZ2B	3.41	3.32	3.42
212487_at	G patch domain c	GPATCH8	8.85	8.76	8.79
203282_at	glucan (1,4-alpha-	GBE1	12.29	12.38	12.38
221776_s_at	bromodomain cor	BRD7	9.54	9.58	9.55
210117_at	sperm associated	SPAG1	5.61	5.87	6.06
205829_at	hydroxysteroid (1	HSD17B1	5.73	5.92	5.87
221176_x_at	---	---	7.69	7.65	7.60
38069_at	chloride channel 7	CLCN7	8.68	8.62	8.61
220596_at	G patch domain c	GPATCH4	4.58	4.74	4.66
221986_s_at	kelch-like 24 (Dro	KLHL24	5.95	5.98	6.00
204596_s_at	stanniocalcin 1	STC1	5.16	4.98	5.03
219877_at	zinc finger, matrin	ZMAT4	5.10	5.07	4.96
203732_at	thyroid hormone	TRIP4	9.00	9.14	9.02

204493_at	BH3 interacting do	BID	9.00	8.96	9.11
203802_x_at	NOP2/Sun domair	NSUN5	10.24	10.15	10.21
203147_s_at	tripartite motif-co	TRIM14	8.59	8.67	8.64
59631_at	thioredoxin reduc	TXNRD3	8.06	8.13	8.05
204386_s_at	mitochondrial ribo	MRP63	12.13	12.09	12.14
217536_x_at	---	---	4.10	4.22	4.12
214647_s_at	hemochromatosis	HFE	4.24	4.19	4.24
212547_at	bromodomain cor	BRD3	9.12	9.08	9.10
208120_x_at	FKSG49	FKSG49	8.96	8.88	8.71
206744_s_at	zinc finger, MYM-1	ZMYM5	3.93	3.84	3.93
200921_s_at	B-cell translocatio	BTG1	12.12	12.04	12.30
204090_at	serine/threonine I	STK19	8.29	8.23	8.34
218264_at	BRCA2 and CDKN1	BCCIP	8.75	8.83	8.85
205238_at	TRM2 tRNA methy	TRMT2B	6.46	6.56	6.57
219679_s_at	WW domain conta	WAC	8.26	8.12	8.28
206074_s_at	high mobility grou	HMGA1	10.48	10.46	10.60
218069_at	dCTP pyrophosph	DCTPP1	10.91	10.90	10.88
206572_x_at	zinc finger proteir	ZNF85	6.55	6.75	6.64
222010_at	t-complex 1	TCP1	9.35	9.46	9.38
221046_s_at	GTP-binding prote	GTPBP8	10.44	10.55	10.44
213826_s_at	hypothetical LOC1	LOC100133109	7.30	7.46	7.60
209368_at	epoxide hydrolase	EPHX2	6.62	6.55	6.63
206769_at	thymosin beta 4, Y	TMSB4Y	7.52	7.44	7.50
218829_s_at	chromodomain he	CHD7	8.53	8.55	8.65
202510_s_at	tumor necrosis fac	TNFAIP2	7.96	8.03	8.17
208335_s_at	Duffy blood group	DARC	6.34	6.08	6.35
203906_at	IQ motif and Sec7	IQSEC1	6.91	7.18	6.96
214061_at	WD repeat domai	WDR67	8.43	8.35	8.54
202998_s_at	lysyl oxidase-like 2	LOXL2	8.39	8.36	8.45
208867_s_at	casein kinase 1, al	CSNK1A1	9.33	9.24	9.32
222139_at	KIAA1466 gene	KIAA1466	4.30	4.45	4.23
222043_at	clusterin	CLU	8.75	8.69	8.86
217995_at	sulfide quinone re	SQRDL	9.91	9.97	9.90
211389_x_at	killer cell immuno	KIR3DS1	5.36	5.48	5.34
207291_at	proline rich Gla (G	PRRG4	5.46	5.29	5.45
221742_at	CUGBP, Elav-like f	CELF1	9.29	9.26	9.24
215111_s_at	TSC22 domain fan	TSC22D1	12.13	12.12	12.19
200594_x_at	heterogeneous nu	HNRNPU	12.44	12.42	12.42
200835_s_at	microtubule-asso	MAP4	9.92	9.87	9.92
218640_s_at	pleckstrin homolo	PLEKHF2	8.62	8.70	8.86
200933_x_at	ribosomal protein	RPS4X	14.79	14.77	14.80
209760_at	KIAA0922	KIAA0922	7.14	7.34	7.44
209721_s_at	intermediate filan	IFFO1	8.78	8.90	8.77
209192_x_at	K(lysine) acetyltra	KAT5	7.96	7.86	7.95
204867_at	GTP cyclohydrolas	GCHFR	7.60	7.65	7.58
37117_at	Rho GTPase activa	ARHGAP8 /// PRR5	5.03	5.15	4.94
202378_s_at	leptin receptor ov	LEPROT	10.49	10.46	10.52

213116_at	NIMA (never in m	NEK3	7.74	7.81	7.77
200728_at	ARP2 actin-relate	ACTR2	12.62	12.69	12.64
200011_s_at	ADP-ribosylation f	ARF3	9.48	9.57	9.53
201257_x_at	ribosomal protein	RPS3A	15.07	15.04	15.07
204765_at	Rho guanine nucl	ARHGEF5	7.26	7.31	7.35
217708_x_at	---	---	3.46	3.55	3.40
213763_at	homeodomain int	HIPK2	9.06	9.19	9.07
220092_s_at	anthrax toxin rece	ANTXR1	6.74	6.82	6.77
218324_s_at	spermatogenesis a	SPATS2	8.09	7.96	8.15
217797_at	ubiquitin-fold mo	UFC1	10.39	10.50	10.49
212701_at	talin 2	TLN2	6.69	6.76	6.82
205271_s_at	cyclin-dependent	CDK20	6.63	6.59	6.46
203855_at	WD repeat domai	WDR47	8.44	8.67	8.53
221270_s_at	queuine tRNA-rib	QTRT1	7.60	7.56	7.42
201325_s_at	epithelial membra	EMP1	10.25	10.37	10.55
220297_at	BTB (POZ) domair	BTBD7	5.68	5.71	5.52
203142_s_at	adaptor-related p	AP3B1	9.86	9.91	9.96
206290_s_at	regulator of G-pro	RGS7	3.90	3.71	3.83
211503_s_at	RAB14, member R	RAB14	9.90	9.86	9.95
213452_at	zinc finger protein	ZNF184	7.84	7.80	7.98
217008_s_at	glutamate recepto	GRM7	3.64	3.58	3.52
217653_x_at	---	---	7.43	7.41	7.50
219024_at	pleckstrin homolo	PLEKHA1	9.84	9.83	10.02
216346_at	SEC14-like 3 (S. ce	SEC14L3	4.14	4.11	4.16
200761_s_at	ADP-ribosylation-l	ARL6IP5	11.78	11.82	11.93
213876_x_at	zinc finger (CCCH	ZRSR2	7.60	7.73	7.75
213010_at	protein kinase C, d	PRKCDBP	9.71	9.74	9.77
221960_s_at	RAB2A, member R	RAB2A	6.79	6.86	6.99
203232_s_at	ataxin 1	ATXN1	7.26	7.24	7.47
208923_at	cytoplasmic FMR1	CYFIP1	11.18	11.17	11.18
212063_at	CD44 molecule (In	CD44	11.73	11.73	11.89
208029_s_at	lysosomal protein	LAPTM4B	11.78	11.78	11.83
210255_at	RAD51-like 1 (S. c	RAD51L1	5.90	6.11	6.16
215572_at	Similar to golgi au	LOC646934	5.71	5.71	5.55
215253_s_at	regulator of calcin	RCAN1	7.85	7.73	7.48
211400_at	fibroblast growth	FGFR2	4.28	4.22	4.22
203598_s_at	WW domain bindi	WBP4	7.87	8.01	8.07
203303_at	dynein, light chair	DYNLT3	10.37	10.50	10.48
222025_s_at	5-oxoprolinase (A	OPLAH	6.95	7.00	6.84
215781_s_at	topoisomerase (D	TOP3B	6.01	5.97	5.85
208257_x_at	pregnancy specifi	PSG1	8.23	8.12	8.06
209281_s_at	ATPase, Ca++ tran	ATP2B1	10.45	10.49	10.49
212801_at	citron (rho-intera	CIT	9.29	9.33	9.15
220607_x_at	TH1-like (Drosoph	TH1L	9.34	9.36	9.38
203839_s_at	tyrosine kinase, n	TNK2	7.46	7.60	7.51
205129_at	nucleophosmin/n	NPM3	9.74	9.62	9.75
219738_s_at	protocadherin 9	PCDH9	7.22	7.17	7.30

204197_s_at	runt-related trans	RUNX3	4.19	4.23	4.04
209770_at	butyrophilin, subf	BTN3A1	6.76	6.72	6.54
202070_s_at	isocitrate dehydro	IDH3A	7.74	7.83	7.79
205345_at	BRCA1 associated	BARD1	9.38	9.35	9.47
222219_s_at	transducin-like en	TLE6	6.56	6.52	6.58
212856_at	GRAM domain co	GRAMD4	6.73	6.92	6.91
201023_at	TAF7 RNA polyme	TAF7	10.01	10.01	10.13
210736_x_at	dystrobrevin, alph	DTNA	6.75	6.64	6.85
212148_at	pre-B-cell leukemi	PBX1	7.68	7.77	7.67
209011_at	triple functional d	TRIO	8.47	8.50	8.41
219751_at	SET domain conta	SETD6	8.07	8.05	8.22
220659_s_at	chromosome 7 op	C7orf43	7.52	7.44	7.46
220608_s_at	zinc finger protein	ZNF770	4.55	4.73	4.62
222301_at	Chromosome 1 op	C1orf61	6.34	6.35	6.34
218269_at	ribonuclease type	RNASEN	10.58	10.51	10.55
206548_at	---	---	6.33	6.36	6.30
207266_x_at	RNA binding moti	RBMS1	9.77	9.77	9.82
213014_at	mitogen-activated	MAPK8IP1	5.43	5.41	5.32
221516_s_at	Smith-Magenis sy	SMCR7L	9.43	9.43	9.48
213892_s_at	adenine phosphor	APRT	10.82	10.69	10.73
200969_at	stress-associated	SERP1	10.45	10.49	10.59
219383_at	proline rich 5 like	PRR5L	6.79	7.01	6.80
33304_at	interferon stimula	ISG20	6.30	5.94	5.92
205721_at	GDNF family recep	GFRA2	5.69	5.69	5.58
210601_at	cadherin 6, type 2	CDH6	7.02	6.74	6.99
220873_at	---	---	4.19	4.04	4.09
204631_at	myosin, heavy cha	MYH2	3.95	3.90	3.72
208763_s_at	TSC22 domain fan	TSC22D3	14.10	13.99	14.16
205245_at	par-6 partitioning	PARD6A	7.59	7.42	7.33
222152_at	Programmed cell	PDCD6	7.69	7.69	7.74
214001_x_at	---	---	8.91	9.08	9.01
200701_at	Niemann-Pick dise	NPC2	11.74	11.77	11.82
208703_s_at	amyloid beta (A4)	APLP2	7.93	8.03	8.11
200876_s_at	proteasome (pros	PSMB1	12.89	12.95	12.90
203227_s_at	tetraspanin 31	TSPAN31	8.98	9.06	9.11
222086_s_at	Wingless-type MN	WNT6	4.20	4.20	4.29
220661_s_at	zinc finger protein	ZNF692	8.47	8.52	8.37
32094_at	solute carrier fam	SLC25A44	8.55	8.47	8.43
204105_s_at	neuronal cell adhe	NRCAM	9.27	9.22	9.44
211536_x_at	mitogen-activated	MAP3K7	7.98	8.00	8.01
221996_s_at	Clathrin, light chai	CLTB	4.88	5.01	4.92
210267_at	NIPA-like domain	NIPAL3	4.11	4.15	4.14
204588_s_at	solute carrier fam	SLC7A7	6.00	6.02	6.20
221473_x_at	serine incorporato	SERINC3	10.14	10.06	10.15
202424_at	mitogen-activated	MAP2K2	8.54	8.45	8.57
222242_s_at	kallikrein-related	KLK5	6.32	6.29	6.33
217153_at	Rho GTPase activa	ARHGAP1	6.39	6.35	6.57

203324_s_at	caveolin 2	CAV2	10.15	10.22	10.34
221299_at	G protein-couplec	GPR173	5.37	5.30	5.31
213467_at	Rho family GTPase	RND2	4.85	4.98	4.99
203845_at	K(lysine) acetyltra	KAT2B	7.80	7.74	8.01
200862_at	24-dehydrocholes	DHCR24	10.49	10.60	10.50
206052_s_at	stem-loop binding	SLBP	10.97	10.96	11.08
201239_s_at	signal peptidase c	LOC653566 /// SP(	11.51	11.70	11.59
208908_s_at	calpastatin	CAST	9.90	9.84	9.92
213187_x_at	ferritin, light poly	FTL	14.77	14.72	14.73
212387_at	transcription facto	TCF4	8.40	8.48	8.53
211522_s_at	gonadotropin-rele	GNRHR	3.30	3.24	3.26
204314_s_at	cAMP responsive	CREB1	8.34	8.45	8.54
218823_s_at	potassium channe	KCTD9	9.60	9.61	9.82
220241_at	transmembrane a	TMCO3	5.06	4.99	5.57
205027_s_at	mitogen-activatec	MAP3K8	6.87	6.95	6.93
AFFX-HSAC07/X(	actin, beta	ACTB	14.02	14.09	14.12
219998_at	galectin-related p	HSPC159	7.33	7.46	7.48
210854_x_at	solute carrier fam	SLC6A8	9.54	9.45	9.38
213455_at	family with seque	FAM114A1	8.44	8.79	8.46
204492_at	Rho GTPase activa	ARHGAP11A	7.36	7.36	7.45
201278_at	disabled homolog	DAB2	9.84	9.72	10.00
213159_at	pecanex homolog	PCNX	6.99	6.93	6.92
202184_s_at	nucleoporin 133kl	NUP133	9.64	9.59	9.61
207196_s_at	TNFAIP3 interacti	TNIP1	11.02	10.85	10.93
204417_at	galactosylceramid	GALC	6.60	6.58	6.63
218826_at	solute carrier fam	SLC35F2	9.11	8.92	9.11
211546_x_at	synuclein, alpha (r	SNCA	7.93	7.95	7.89
200636_s_at	protein tyrosine p	PTPRF	10.86	10.81	10.92
214739_at	leucine-rich repea	LRCH3	7.97	7.94	8.09
219493_at	SHC SH2-domain b	SHCBP1	9.45	9.74	9.66
206090_s_at	disrupted in schizo	DISC1 /// TSNAX-D	6.95	6.92	6.89
217884_at	N-acetyltransferas	NAT10	9.49	9.48	9.63
210776_x_at	transcription facto	TCF3	9.95	9.98	10.07
202003_s_at	acetyl-CoA acyltra	ACAA2	10.20	10.28	10.33
219363_s_at	MTERF domain co	MTERFD1	10.11	10.16	10.20
204200_s_at	platelet-derived g	PDGFB	6.18	6.09	5.98
38158_at	extra spindle pole	ESPL1	9.47	9.48	9.48
209550_at	necdin homolog (r	NDN	5.97	5.86	5.82
202092_s_at	ADP-ribosylation f	ARL2BP	9.87	9.83	9.79
217649_at	zinc finger, AN1-ty	ZFAND5	3.82	4.01	4.12
218664_at	mitochondrial tra	MECR	8.12	8.09	8.17
217905_at	chromosome 10 c	C10orf119	8.77	8.88	8.82
206508_at	CD70 molecule	CD70	6.82	6.90	6.70
214816_x_at	chromosome 19 c	C19orf40	6.76	6.78	6.77
217979_at	tetraspanin 13	TSPAN13	8.66	8.70	8.70
203130_s_at	kinesin family me	KIF5C	5.60	5.53	5.42
218610_s_at	calcineurin-like ph	CPPED1	3.94	4.36	4.13

200015_s_at	septin 2		2-Sep	12.37	12.48	12.47
208989_s_at	lysine (K)-specific	KDM2A		8.19	8.16	8.02
209015_s_at	DnaJ (Hsp40) hom	DNAJB6		7.71	7.76	7.85
204664_at	alkaline phosphat	ALPP		4.58	4.61	4.54
203632_s_at	G protein-couplec	GPRC5B		6.00	5.84	6.24
202630_at	amyloid beta prec	APPBP2		8.29	8.16	8.40
219635_at	zinc finger proteir	ZNF606		4.64	4.62	4.71
218226_s_at	NADH dehydroger	NDUFB4		12.45	12.49	12.50
219648_at	melanoregulin	MREG		6.95	7.27	7.24
222077_s_at	Rac GTPase activa	RACGAP1		11.66	11.72	11.83
213888_s_at	TRAF3 interacting	TRAF3IP3		4.30	4.09	4.26
219386_s_at	SLAM family mem	SLAMF8		5.81	5.63	5.67
219209_at	interferon induce	IFIH1		7.31	7.43	7.59
204298_s_at	lysyl oxidase	LOX		5.33	5.31	4.84
220890_s_at	DEAD (Asp-Glu-Al	DDX47		10.89	10.90	10.91
211682_x_at	UDP glucuronosyl	UGT2B28		6.85	7.04	7.03
216047_x_at	seizure related 6 l	SEZ6L		6.50	6.47	6.30
220448_at	potassium channe	KCNK12		4.11	4.35	4.07
212942_s_at	KIAA1199	KIAA1199		5.03	5.02	4.81
210125_s_at	barrier to autointe	BANF1		8.69	8.55	8.81
219525_at	solute carrier fam	SLC47A1		5.11	5.00	4.54
214913_at	ADAM metallopep	ADAMTS3		4.50	4.66	4.84
213325_at	poliovirus recepto	PVRL3		8.61	8.83	8.93
205796_at	t-complex 11 (mo	TCP11L1		7.13	7.00	7.18
215794_x_at	glutamate dehydr	GLUD2		8.61	8.57	8.73
216373_at	transmembrane a	TAPT1		3.35	3.34	3.35
218832_x_at	arrestin, beta 1	ARRB1		5.30	5.55	5.21
209304_x_at	growth arrest and	GADD45B		10.84	10.79	10.76
219258_at	TIMELESS interact	TIPIN		9.29	9.33	9.41
208978_at	cysteine-rich prot	CRIP2		6.49	6.46	6.39
210493_s_at	microfibrillar-asso	MFAP3L		4.82	4.99	4.86
201631_s_at	immediate early r	IER3		11.73	11.80	11.65
206866_at	cadherin 4, type 1	CDH4		5.38	5.44	5.31
203721_s_at	UTP18, small subu	UTP18		11.53	11.55	11.63
220047_at	sirtuin (silent mat	SIRT4		3.65	3.46	3.65
215597_x_at	---	---		4.47	4.44	4.33
204569_at	intestinal cell (MA	ICK		4.65	4.79	4.98
208938_at	papillary renal cel	PRCC		7.22	7.11	7.21
217706_at	---	---		4.20	4.13	4.15
212622_at	transmembrane p	TMEM41B		9.64	9.72	9.67
205922_at	vanin 2	VNN2		4.11	4.03	3.84
216740_at	---	---		3.52	3.38	3.54
210314_x_at	tumor necrosis fa	TNFSF13		4.89	4.65	4.79
201781_s_at	aryl hydrocarbon	AIP		8.45	8.40	8.40
208239_at	forkhead box E1 (	FOXE1		4.77	4.69	4.61
206500_s_at	chromosome 14 c	C14orf106		8.38	8.57	8.51
209818_s_at	hyaluronan bindir	HABP4		7.42	7.46	7.64

206544_x_at	SWI/SNF related,	SMARCA2	8.03	8.09	8.18
206057_x_at	sialophorin	SPN	6.31	6.21	6.37
211851_x_at	breast cancer 1, e	BRCA1	7.07	7.18	7.13
213857_s_at	CD47 molecule	CD47	10.32	10.27	10.29
209551_at	Yip1 domain famil	YIPF4	7.68	7.75	7.88
203075_at	SMAD family men	SMAD2	9.88	9.73	9.88
205041_s_at	orosomucoid 1 //,	ORM1 /// ORM2	4.54	4.34	4.42
208891_at	dual specificity ph	DUSP6	7.73	7.86	7.82
205709_s_at	CDP-diacylglycero	CDS1	6.55	6.66	6.67
205519_at	WD repeat domai	WDR76	6.69	6.78	6.77
219388_at	grainyhead-like 2	GRHL2	4.36	4.46	4.39
202973_x_at	family with seque	FAM13A	9.13	9.16	9.32
207676_at	one cut homeobo	ONECUT2	5.74	5.40	5.49
222226_at	serum amyloid A3	SAA3P	6.00	5.86	5.80
222114_x_at	WD repeat domai	WDR55	7.39	7.26	7.19
207198_s_at	LIM and senescen	LIMS1	11.03	10.96	10.95
208679_s_at	actin related prot	ARPC2	13.09	13.24	13.25
214321_at	nephroblastoma c	NOV	7.40	7.38	7.55
217661_x_at	SIX homeobox 5	SIX5	6.38	6.26	6.32
206447_at	chymotrypsin-like	CELA2A	4.61	4.58	4.51
212186_at	acetyl-CoA carbox	ACACA	10.16	10.18	10.21
209330_s_at	heterogeneous nu	HNRNPD	10.72	10.88	10.95
202864_s_at	SP100 nuclear ant	SP100	10.12	10.02	10.11
213264_at	poly(rC) binding p	PCBP2	7.84	7.73	7.68
203226_s_at	tetraspanin 31	TSPAN31	8.24	8.16	8.16
206932_at	cholesterol 25-hy	CH25H	3.93	4.24	4.10
209424_s_at	alpha-methylacyl-	AMACR /// C1QTN	8.09	8.17	8.29
210147_at	ADP-ribosyltransf	ART3	3.43	3.44	3.39
219168_s_at	proline rich 5 (ren	PRR5	4.95	4.86	4.55
212181_s_at	nudix (nucleoside	NUDT4 /// NUDT4	10.64	10.57	10.70
213056_at	FERM domain con	FRMD4B	8.00	8.09	8.30
201559_s_at	chloride intracellu	CLIC4	8.92	8.98	9.12
203534_at	LSM1 homolog, U	LSM1	11.30	11.36	11.38
217115_at	makorin ring finge	LOC100240726	6.26	6.14	6.08
220233_at	F-box protein 17 /	FBXO17 /// SARS2	8.46	8.41	8.48
201660_at	Acyl-CoA syntheta	ACSL3	11.01	11.17	11.06
207265_s_at	KDEL (Lys-Asp-Glu	KDELR3	8.62	8.58	8.59
206899_at	neurotensin recep	NTSR2	4.41	4.35	4.48
218208_at	PQ loop repeat co	PQLC1	8.38	8.36	8.43
205565_s_at	frataxin	FXN	8.16	8.24	8.44
219565_at	cytochrome P450,	CYP20A1	7.88	7.85	8.00
220688_s_at	mRNA turnover 4	MRTO4	11.07	10.96	11.03
205870_at	bradykinin recept	BDKRB2	7.10	6.71	6.55
214964_at	---	---	6.16	6.05	6.13
221927_s_at	abhydrolase doma	ABHD11	8.47	8.43	8.29
201485_s_at	reticulocalbin 2, E	RCN2	10.52	10.51	10.50
213405_at	RAB22A, member	RAB22A	8.83	8.89	9.07

213882_at	TM2 domain cont	TM2D1	6.12	6.16	6.35
210263_at	potassium voltage	KCNF1	6.61	6.53	6.45
203541_s_at	Kruppel-like facto	KLF9	6.47	6.38	6.49
201677_at	Chromosome 3 op	C3orf37	6.95	6.96	6.78
210319_x_at	msh homeobox 2	MSX2	4.84	4.75	4.81
204774_at	ecotropic viral int	EVI2A	7.68	7.71	7.92
202937_x_at	ribosomal RNA pr	RRP7A	8.79	8.78	8.77
212203_x_at	interferon induce	IFITM3	13.00	12.89	12.95
217867_x_at	beta-site APP-clea	BACE2	6.90	6.96	6.91
211064_at	zinc finger protein	ZNF493	4.16	4.23	4.09
207213_s_at	ubiquitin specific	USP2	5.47	5.54	5.40
209074_s_at	family with seque	FAM107A	6.47	6.74	6.70
206530_at	RAB30, member F	RAB30	4.89	4.92	4.79
211090_s_at	PRP4 pre-mRNA p	PRPF4B	7.30	7.47	7.52
201553_s_at	lysosomal-associa	LAMP1	12.56	12.49	12.60
203462_x_at	eukaryotic transla	EIF3B	11.84	11.77	11.78
200026_at	ribosomal protein	RPL34	14.09	14.02	14.11
202414_at	excision repair cro	ERCC5	8.86	8.95	8.93
221228_s_at	---	---	3.67	3.50	3.55
200650_s_at	lactate dehydroge	LDHA	14.33	14.38	14.39
206536_s_at	X-linked inhibitor	XIAP	6.13	6.23	6.35
219570_at	kinesin family me	KIF16B	6.70	6.72	6.77
202564_x_at	ADP-ribosylation f	ARL2	9.20	9.22	9.30
210658_s_at	golgi-associated, g	GGA2	5.82	5.87	6.03
201498_at	ubiquitin specific	USP7	10.57	10.65	10.66
204219_s_at	proteasome (pros	PSMC1	11.37	11.39	11.41
212350_at	TBC1 (tre-2/USP6,	TBC1D1	8.35	8.40	8.45
216361_s_at	MYST histone ace	MYST3	5.36	5.12	5.24
205980_s_at	Rho GTPase activa	ARHGAP8 /// PRR5	4.55	4.43	4.30
208873_s_at	receptor accessor	REEP5	10.23	10.50	10.52
214681_at	glycerol kinase	GK	5.99	6.12	6.06
212742_at	ring finger protein	RNF115	10.10	10.02	10.05
202545_at	protein kinase C, c	PRKCD	6.26	6.31	6.22
212789_at	non-SMC condens	NCAPD3	9.60	9.67	9.62
221272_s_at	chromosome 1 op	C1orf21	5.88	5.93	6.04
206079_at	choroideremia-lik	CHML	6.41	6.41	6.38
217912_at	dihydrouridine sy	DUS1L	9.63	9.56	9.64
201187_s_at	inositol 1,4,5-trip	ITPR3	6.37	6.35	6.33
212899_at	cyclin-dependent	CDK19	8.34	8.51	8.37
203219_s_at	adenine phosphor	APRT	10.37	10.21	10.38
202793_at	lysophosphatidylc	LPCAT3	7.58	7.44	7.45
220388_at	fer-1-like 4 (C. ele	FER1L4	4.60	4.53	4.40
213316_at	KIAA1462	KIAA1462	6.20	6.21	6.51
216091_s_at	beta-transducin re	BTRC	5.71	5.72	5.51
219976_at	hook homolog 1 (	HOOK1	3.89	3.72	3.75
213313_at	RAB GTPase activa	RABGAP1	8.93	8.85	8.89
213895_at	epithelial membra	EMP1	5.56	5.63	5.62

218722_s_at	coiled-coil domair	CCDC51	9.26	9.14	9.25
202621_at	interferon regulat	IRF3	7.11	7.23	7.23
220310_at	tubulin, alpha-like	TUBAL3	5.49	5.75	5.62
202107_s_at	minichromosome	MCM2	9.81	9.77	9.80
203339_at	solute carrier fam	SLC25A12	7.64	7.90	7.85
219174_at	intraflagellar trans	IFT74	7.50	7.58	7.78
221718_s_at	A kinase (PRKA) ar	AKAP13	9.06	9.01	9.10
210056_at	Rho family GTPase	RND1	6.97	6.85	6.80
215469_at	---	---	3.35	3.37	3.28
201404_x_at	proteasome (pros	PSMB2	7.26	7.33	7.53
205266_at	leukemia inhibitor	LIF	6.98	6.98	7.12
202948_at	interleukin 1 rece	IL1R1	8.66	8.86	8.77
201682_at	peptidase (mitoch	PMPCB	11.05	11.00	11.03
210764_s_at	cysteine-rich, angi	CYR61	9.89	9.96	10.03
217420_s_at	polymerase (RNA)	POLR2A	4.62	4.51	4.32
213792_s_at	insulin receptor	INSR	8.83	8.67	8.80
219956_at	UDP-N-acetyl-alph	GALNT6	4.09	4.04	3.91
208974_x_at	karyopherin (impo	KPNB1	12.81	12.76	12.83
206352_s_at	peroxisomal bioge	PEX10	7.50	7.59	7.60
210592_s_at	spermidine/sperm	SAT1	9.29	9.29	9.43
212744_at	Bardet-Biedl synd	BBS4	7.55	7.70	7.69
208688_x_at	eukaryotic transla	EIF3B	11.79	11.78	11.78
215071_s_at	histone cluster 1,	HIST1H2AC	8.53	8.57	8.70
213561_at	ASF1 anti-silencin	ASF1A	5.58	5.75	6.08
201810_s_at	SH3-domain bindi	SH3BP5	8.07	8.02	8.03
221617_at	TAF9B RNA polym	TAF9B	6.39	6.46	6.44
203875_at	SWI/SNF related,	SMARCA1	7.02	7.48	7.20
212400_at	family with seque	FAM102A	7.97	7.93	7.91
207139_at	ATPase, H+/K+ exc	ATP4A	6.18	6.02	5.99
209055_s_at	CDC5 cell division	CDC5L	8.36	8.46	8.49
206046_at	ADAM metallopep	ADAM23	5.65	5.42	5.60
202905_x_at	nibrin	NBN	8.77	8.94	8.85
213718_at	RNA binding moti	RBM4	7.05	7.22	7.34
219000_s_at	defective in sister	DSCC1	8.84	8.85	8.85
212424_at	programmed cell	PDCD11	9.14	9.03	9.20
214630_at	cytochrome P450,	CYP11B2	6.93	6.64	6.69
213339_at	KIAA0495	KIAA0495	5.81	5.57	5.58
204159_at	cyclin-dependent	CDKN2C	10.17	10.31	10.63
213688_at	calmodulin 1 (pho	CALM1	6.66	6.68	6.67
210678_s_at	1-acylglycerol-3-p	AGPAT2	7.92	7.87	7.77
208012_x_at	SP110 nuclear bo	SP110	8.33	8.40	8.39
213409_s_at	Ras homolog enri	RHEB	6.09	6.24	6.09
204818_at	hydroxysteroid (1	HSD17B2	3.41	3.50	3.38
216465_at	---	---	4.88	4.92	4.90
222124_at	hypoxia inducible	HIF3A	3.38	3.36	3.32
214202_at	---	---	6.35	6.44	6.46
212332_at	retinoblastoma-lik	RBL2	7.75	7.75	7.61

205364_at	acyl-CoA oxidase :	ACOX2	6.12	6.46	6.10
215530_at	Fanconi anemia, c	FANCA	6.58	6.52	6.29
203046_s_at	timeless homolog	TIMELESS	9.16	9.21	9.18
209537_at	exostoses (multip	EXTL2	8.79	8.98	9.03
214149_s_at	ATPase, H+ transp	ATP6V0E1	9.04	9.17	9.17
218100_s_at	intraflagellar trans	IFT57	9.73	9.88	9.92
203288_at	KIAA0355	KIAA0355	7.73	7.79	7.85
210162_s_at	nuclear factor of a	NFATC1	6.31	6.13	6.02
212252_at	calcium/calmodul	CAMKK2	7.91	8.01	7.97
205897_at	nuclear factor of a	NFATC4	6.54	6.52	6.36
221078_s_at	coiled-coil domair	CCDC88A	6.05	6.29	6.16
210461_s_at	actin binding LIM	ABLIM1	7.56	7.37	7.10
212217_at	prolyl endopeptid	PREPL	8.79	8.96	8.92
33148_at	zinc finger RNA bi	ZFR	6.35	6.36	6.45
212672_at	ataxia telangiecta	ATM	7.92	7.87	7.97
219279_at	dedicator of cytok	DOCK10	8.29	8.34	8.44
210453_x_at	ATP synthase, H+	ATP5L	13.70	13.67	13.69
211100_x_at	leukocyte immun	LILRA2	6.26	6.31	6.21
206272_at	RAB4A, member F	RAB4A /// SPHAR	7.64	7.71	7.78
206589_at	growth factor ind	GFI1	4.19	4.33	4.18
218016_s_at	polymerase (RNA)	POLR3E	10.13	10.09	10.18
213101_s_at	ARP3 actin-relate	ACTR3	13.28	13.32	13.37
202426_s_at	retinoid X recepto	RXRA	6.77	6.84	6.71
217478_s_at	major histocompa	HLA-DMA	4.90	4.96	4.90
200695_at	protein phosphat	PPP2R1A	7.93	7.85	7.97
205669_at	neural cell adhesi	NCAM2	5.72	5.68	5.71
208530_s_at	retinoic acid rece	RARB	4.53	4.67	4.66
205479_s_at	plasminogen activ	PLAU	7.39	7.52	7.52
215867_x_at	carbonic anhydras	CA12	10.83	10.88	10.69
215290_at	---	---	3.39	3.40	3.33
219240_s_at	chromosome 10 c	C10orf88	7.56	7.71	7.86
210355_at	parathyroid horm	PTHLH	3.16	3.28	3.04
207919_at	ADP-ribosyltransf	ART1	6.58	6.59	6.68
212446_s_at	LAG1 homolog, ce	LASS6	9.72	9.63	9.97
217051_s_at	---	---	5.85	5.82	5.90
215645_at	folliculin	FLCN	6.21	6.22	6.32
213784_at	intraflagellar trans	IFT27	6.03	6.08	6.03
217878_s_at	cell division cycle	CDC27	8.36	8.63	8.67
205888_s_at	janus kinase and r	JAKMIP2	5.67	5.82	5.81
210257_x_at	cullin 4B	CUL4B	6.88	6.97	6.76
221981_s_at	WD repeat domai	WDR59	5.91	6.13	6.13
212740_at	phosphoinositide-	PIK3R4	8.73	8.77	8.84
218463_s_at	MUS81 endonucle	MUS81	9.48	9.43	9.51
212262_at	quaking homolog,	QKI	9.06	9.02	9.10
217752_s_at	CNDP dipeptidase	CNDP2	9.30	9.33	9.52
213035_at	ankyrin repeat do	ANKRD28	9.70	9.71	9.79
221535_at	large subunit GTP	LSG1	9.19	9.19	9.36

211945_s_at	integrin, beta 1 (fi	ITGB1	13.26	13.37	13.36
207190_at	zinc finger, ZZ-typ	ZZEF1	5.62	5.64	5.67
202511_s_at	ATG5 autophagy r	ATG5	10.35	10.30	10.38
218597_s_at	CDGSH iron sulfur	CISD1	11.44	11.44	11.46
43977_at	transmembrane p	TMEM161A	9.17	9.07	9.03
202636_at	ring finger proteir	RNF103	9.33	9.38	9.42
208714_at	NADH dehydroger	NDUFV1	10.66	10.53	10.66
40225_at	cyclin G associate	GAK	9.41	9.42	9.41
217749_at	coatomer protein	COPG	8.63	8.58	8.64
203395_s_at	hairy and enhance	HES1	8.25	7.94	8.26
213120_at	UHRF1 binding pr	UHRF1BP1L	3.90	4.06	3.85
202246_s_at	cyclin-dependent	CDK4	11.16	11.10	11.18
221011_s_at	limb bud and hea	LBH	7.38	7.38	7.19
212996_s_at	URB1 ribosome bi	URB1	8.27	8.13	8.37
213664_at	solute carrier fam	SLC1A1	7.46	7.87	7.54
218262_at	required for meio	RMND5B	7.69	7.84	7.84
219563_at	chromosome 14 c	C14orf139	7.49	7.60	7.50
204560_at	FK506 binding pro	FKBP5	7.93	7.88	8.00
202657_s_at	SERTA domain co	SERTAD2	10.17	10.08	10.20
210395_x_at	myosin, light chai	MYL4	6.54	6.64	6.63
206472_s_at	transducin-like en	TLE3	6.68	6.55	6.53
200073_s_at	heterogeneous nu	HNRNPD	12.21	12.26	12.34
217677_at	pleckstrin homolo	PLEKHA2	7.18	7.16	7.41
201169_s_at	basic helix-loop-h	BHLHE40	5.41	5.50	5.33
212083_at	testis expressed 2	TEX261	8.91	8.93	8.93
215631_s_at	breast cancer met	BRMS1	9.17	9.19	9.01
221219_s_at	kelch domain con	KLHDC4	8.33	8.21	8.10
205190_at	plastin 1	PLS1	9.40	9.42	9.57
202051_s_at	zinc finger, MYM-	ZMYM4	9.12	9.26	9.23
211495_x_at	TNFSF12-TNFSF13	TNFSF12-TNFSF13	7.02	7.17	6.89
211701_s_at	trophinin	TRO	4.39	4.31	4.23
212979_s_at	family with seque	FAM115A	8.15	8.48	8.37
200598_s_at	heat shock protei	HSP90B1	12.36	12.44	12.43
211930_at	heterogeneous nu	HNRNPA3	10.18	10.23	10.24
209392_at	ectonucleotide py	ENPP2	11.53	11.49	11.73
212397_at	radixin	RDX	10.61	10.82	10.88
201680_x_at	serrate RNA effec	SRRT	9.88	9.95	9.89
206268_at	left-right determi	LEFTY1	4.88	5.03	5.13
220625_s_at	E74-like factor 5 (	ELF5	3.47	3.58	3.56
221207_s_at	neurobeachin	NBEA	7.23	7.02	7.30
207674_at	Fc fragment of IgA	FCAR	5.42	5.27	5.54
65521_at	ubiquitin-conjuga	UBE2D4	8.07	8.01	7.97
211080_s_at	NIMA (never in m	NEK2	8.04	8.13	8.21
201601_x_at	interferon induce	IFITM1	11.48	11.43	11.48
64371_at	splicing factor, ar	SFRS14	7.81	7.85	7.69
221192_x_at	major facilitator s	MFSD11	7.84	8.01	7.88
220924_s_at	solute carrier fam	SLC38A2	13.46	13.58	13.64

205842_s_at	Janus kinase 2	JAK2	5.49	5.46	5.45
202523_s_at	sparc/osteonectir	SPOCK2	6.76	6.86	6.63
222088_s_at	solute carrier fam	SLC2A14 /// SLC2A	7.38	7.54	7.32
219569_s_at	transmembrane p	TMEM22	8.58	8.54	8.65
208179_x_at	killer cell immuno	KIR2DL3	6.23	6.51	6.27
213927_at	mitogen-activatec	MAP3K9	6.51	6.61	6.56
217590_s_at	transient receptor	TRPA1	4.11	3.95	4.05
206918_s_at	copine I	CPNE1	8.56	8.46	8.50
200875_s_at	NOP56 ribonuclec	NOP56	12.51	12.54	12.59
206022_at	Norrie disease (ps	NDP	3.23	3.27	3.16
212739_s_at	non-metastatic ce	NME4	9.06	8.97	9.02
219972_s_at	chromosome 14 c	C14orf135	9.59	9.49	9.80
201420_s_at	WD repeat domai	WDR77	9.24	9.18	9.16
203518_at	lysosomal trafficki	LYST	6.82	6.98	6.78
215480_at	hypothetical LOC5	KIAA0509	4.16	4.14	4.20
209090_s_at	SH3-domain GRB2	SH3GLB1	10.09	10.15	10.13
219145_at	latrophilin 1	LPHN1	6.88	6.82	7.12
205308_at	family with seque	FAM164A	8.57	8.64	8.78
209795_at	CD69 molecule	CD69	3.36	3.24	3.35
218060_s_at	chromosome 16 c	C16orf57	8.28	8.16	8.25
220480_at	heart and neural c	HAND2	5.26	4.99	5.16
218379_at	RNA binding moti	RBM7	10.01	9.93	10.02
204794_at	dual specificity ph	DUSP2	7.34	7.62	7.73
201433_s_at	phosphatidylserin	PTDSS1	11.14	11.09	11.18
204092_s_at	aurora kinase A	AURKA	11.00	11.01	10.99
203450_at	chibby homolog 1	CBY1	9.15	9.18	9.11
204732_s_at	tripartite motif-co	TRIM23	7.37	7.47	7.60
205207_at	interleukin 6 (inte	IL6	6.89	7.04	7.02
203317_at	pleckstrin and Sec	PSD4	6.22	6.13	6.08
206704_at	chloride channel 5	CLCN5	5.70	5.71	5.46
210958_s_at	microtubule assoc	MAST4	7.35	7.56	7.24
212469_at	Nipped-B homolog	NIPBL	9.68	9.69	9.64
218170_at	isochorismatase d	ISOC1	9.17	9.33	9.42
217459_at	---	---	4.33	4.35	4.46
212597_s_at	HMG box domain	HMGXB4	9.02	9.04	9.06
201625_s_at	insulin induced ge	INSIG1	8.54	9.08	8.71
217873_at	calcium binding pr	CAB39	11.26	11.31	11.31
207279_s_at	nebulette	NEBL	6.80	6.88	6.66
202659_at	proteasome (pros	PSMB10	8.69	8.52	8.69
200896_x_at	hepatoma-derivec	HDGF	12.30	12.29	12.28
220646_s_at	killer cell lectin-lik	KLRF1	3.46	3.50	3.52
211565_at	SH3-domain GRB2	SH3GL3	4.58	4.63	4.42
209589_s_at	EPH receptor B2	EPHB2	7.39	6.87	6.99
214280_x_at	heterogeneous nu	HNRNPA1	12.17	12.17	12.18
210980_s_at	N-acylsphingosine	ASAH1	8.42	8.61	8.49
200748_s_at	ferritin, heavy pol	FTH1	14.79	14.76	14.85
213360_s_at	POM121 membra	POM121 /// POM1	10.52	10.55	10.53

203148_s_at	tripartite motif-co	TRIM14	9.91	9.83	9.91
209584_x_at	apolipoprotein B r	APOBEC3C	7.06	7.05	7.17
207678_s_at	SRY (sex determin	SOX30	3.52	3.41	3.29
211252_x_at	pre T-cell antigen	PTCRA	7.82	7.74	7.80
211115_x_at	survival of motor	SIP1	9.88	9.86	9.93
219196_at	secretogranin III	SCG3	3.40	3.50	3.37
204023_at	replication factor	RFC4	10.68	10.72	10.88
222035_s_at	poly(A) polymeras	PAPOLA	11.02	10.98	11.10
213278_at	myotubularin rela	MTMR9	8.44	8.52	8.43
217963_s_at	nerve growth fact	NGFRAP1	10.14	10.07	10.10
222230_s_at	actin-related prot	ACTR10	11.33	11.37	11.48
219628_at	zinc finger, matrin	ZMAT3	7.93	7.75	7.97
201130_s_at	cadherin 1, type 1	CDH1	4.67	4.72	4.62
211053_at	potassium voltage	KCNG1	7.10	6.91	7.14
219162_s_at	mitochondrial ribo	MRPL11	10.31	10.21	10.34
204367_at	Sp2 transcription	SP2	7.40	7.36	7.39
210036_s_at	potassium voltage	KCNH2	8.64	8.52	8.61
219304_s_at	platelet derived g	PDGFD	4.27	4.28	4.21
53991_at	DENN/MADD dom	DENND2A	6.02	5.90	6.01
222224_at	nascent polypepti	NACA2	3.94	4.04	3.98
202929_s_at	D-dopachrome ta	DDT	10.29	10.19	10.28
202697_at	nudix (nucleoside	NUDT21	9.98	9.80	10.00
222087_at	Pvt1 oncogene (n	PVT1	9.44	9.54	9.50
212175_s_at	adenylate kinase 2	AK2	10.13	10.13	10.21
201563_at	sorbitol dehydrog	SORD	10.32	10.37	10.43
215375_x_at	Leucine rich repea	LRRFIP1	4.52	4.59	4.45
206365_at	chemokine (C mot	XCL1	3.69	3.72	3.62
217791_s_at	aldehyde dehydro	ALDH18A1	9.10	9.03	9.01
201940_at	carboxypeptidase	CPD	8.96	9.02	9.07
40569_at	myeloid zinc finge	MZF1	8.46	8.44	8.49
217435_x_at	---	---	5.04	4.73	5.10
204643_s_at	ecto-NOX disulfide	ENOX2	8.01	7.99	8.14
207952_at	interleukin 5 (colo	IL5	3.86	3.55	3.84
200929_at	transmembrane e	TMED10	10.34	10.42	10.43
219942_at	myosin, light chai	MYL7	6.46	6.49	6.36
222336_at	chromosome 4 op	C4orf34	3.48	3.39	3.33
39817_s_at	chromosome 6 op	C6orf108	9.99	9.94	10.00
204066_s_at	ArfGAP with GTPa	AGAP1	8.73	8.68	8.81
209598_at	paraneoplastic an	PNMA2	5.60	6.07	6.30
43427_at	acetyl-CoA carbox	ACACB	5.55	5.66	5.68
208787_at	mitochondrial ribo	MRPL3	12.97	13.05	13.14
205945_at	interleukin 6 rece	IL6R	6.91	7.02	6.97
222036_s_at	minichromosome	MCM4	9.49	9.67	9.61
207436_x_at	KIAA0894 protein	KIAA0894	10.24	10.12	10.15
206565_x_at	glucuronidase, be	SMA4	7.41	7.52	7.61
207538_at	interleukin 4	IL4	3.75	3.81	3.71
215975_x_at	---	---	6.08	6.02	5.97

200708_at	glutamic-oxaloace	GOT2	10.86	10.82	10.84
217056_at	T cell receptor del	TRD@	3.95	4.00	4.04
218234_at	inhibitor of growt	ING4	7.61	7.66	7.70
209255_at	kelch domain con	KLHDC10	7.33	7.32	7.31
205647_at	RAD52 homolog (!	RAD52	6.30	6.19	6.33
210653_s_at	branched chain ke	BCKDHB	7.46	7.39	7.54
216758_at	---	---	3.31	3.19	3.24
206941_x_at	sema domain, imr	SEMA3E	4.38	4.37	4.58
205111_s_at	phospholipase C, (	PLCE1	6.96	6.79	6.98
216456_at	---	---	4.18	4.28	4.43
204731_at	transforming grov	TGFBR3	9.26	9.30	9.25
206699_x_at	neuronal PAS don	NPAS1	6.59	6.42	6.33
215473_at	---	---	3.36	3.44	3.47
215291_at	megakaryoblastic	MKL1	6.17	5.97	5.86
200978_at	malate dehydroge	MDH1	12.35	12.36	12.36
201186_at	low density lipopr	LRPAP1	8.92	8.82	8.87
220520_s_at	nucleoporin 62kD	NUP62CL	5.01	5.46	5.93
215693_x_at	DEAD (Asp-Glu-Al	DDX27	9.82	9.90	9.87
203970_s_at	peroxisomal bioge	PEX3	8.24	8.43	8.45
203285_s_at	heparan sulfate 2-	HS2ST1	7.94	7.90	8.09
211902_x_at	T cell receptor alp	TRA@	4.44	4.62	4.63
202551_s_at	cysteine rich trans	CRIM1	11.47	11.43	11.44
201285_at	makorin ring finge	MKRN1	9.72	9.72	9.71
201432_at	catalase	CAT	10.51	10.54	10.57
218725_at	solute carrier fam	SLC25A22	8.07	8.08	8.09
212472_at	microtubule assoc	MICAL2	9.46	9.41	9.59
206474_at	cyclin-dependent	CDK17	4.86	4.64	4.84
221792_at	RAB6B, member F	RAB6B	7.74	7.75	7.57
209724_s_at	zinc finger proteir	ZFP161	7.85	7.80	7.73
48531_at	TNFAIP3 interacti	TNIP2	9.03	9.03	9.19
213343_s_at	glycerophosphodi	GDPD5	7.18	7.01	7.08
217804_s_at	interleukin enhan	ILF3	9.45	9.33	9.31
207815_at	platelet factor 4 v	PF4V1	3.20	3.21	3.24
220551_at	solute carrier fam	SLC17A6	3.84	3.88	3.68
211458_s_at	GABA(A) receptor	GABARAPL1 /// GA	6.63	6.34	6.50
207303_at	phosphodiesteras	PDE1C	4.52	4.46	4.50
213945_s_at	---	---	5.54	5.20	5.20
213463_s_at	family with seque	FAM149B1	7.06	7.06	7.14
200090_at	farnesyltransferas	FNTA	11.98	11.96	12.02
221229_s_at	tRNA methyltrans	TRMT61B	9.58	9.54	9.67
217881_s_at	cell division cycle	CDC27	8.03	8.06	8.19
220767_at	---	---	3.63	3.80	3.83
210762_s_at	deleted in liver ca	DLC1	10.40	10.20	10.50
203422_at	polymerase (DNA	POLD1	8.78	8.75	8.71
202418_at	Yip1 interacting fa	YIF1A	10.62	10.56	10.77
215497_s_at	WD and tetratrico	WDTC1	7.20	7.14	7.18
203657_s_at	cathepsin F	CTSF	8.05	7.76	7.72

217413_s_at	tenascin XB	TNXB	7.12	7.00	7.09
215493_x_at	butyrophilin, subf	BTN2A1	8.39	8.45	8.43
216339_s_at	tenascin XA pseuc	TNXA /// TNXB	4.21	4.41	4.33
214543_x_at	quaking homolog,	QKI	8.99	9.04	9.10
34206_at	ArfGAP with RhoG	ARAP1	7.50	7.18	7.44
206393_at	troponin I type 2 (	TNNI2	6.43	6.15	6.22
214720_x_at	septin 10	10-Sep	8.27	8.23	8.39
208644_at	poly (ADP-ribose)	PARP1	10.68	10.64	10.70
211626_x_at	v-ets erythroblast	ERG	4.73	4.57	4.63
215043_s_at	glucuronidase, be	SMA4 /// SMA5	6.84	6.96	7.02
221280_s_at	par-3 partitioning	PARD3	6.45	6.32	6.45
203230_at	dishevelled, dsh h	DVL1	9.31	9.19	9.23
213625_at	zinc finger with KI	ZKSCAN4	7.14	7.29	7.35
212946_at	KIAA0564	KIAA0564	8.76	8.72	8.80
211965_at	zinc finger protein	ZFP36L1	7.55	7.77	7.67
212593_s_at	programmed cell	PDCD4	10.94	10.84	11.05
219269_at	homeobox contai	HMBOX1	8.68	8.61	8.65
209347_s_at	v-maf musculoap	MAF	3.95	3.96	4.00
210603_at	N(alpha)-acetyltra	NAA11	4.49	4.43	4.24
201600_at	prohibitin 2	PHB2	12.33	12.32	12.30
200791_s_at	IQ motif containir	IQGAP1	10.88	10.89	10.96
202836_s_at	thioredoxin-like 4	TXNL4A	12.33	12.27	12.34
210968_s_at	reticulon 4	RTN4	12.49	12.43	12.65
221511_x_at	cell cycle progress	CCPG1	7.77	7.80	7.97
205694_at	tyrosinase-relatec	TYRP1	6.03	5.97	6.00
215495_s_at	sterile alpha moti	SAMD4A	7.15	7.02	7.02
206788_s_at	core-binding facto	CBFB	7.34	7.52	7.52
218757_s_at	UPF3 regulator of	UPF3B	10.19	10.25	10.32
206976_s_at	heat shock 105kD	HSPH1	10.43	10.54	10.62
208294_x_at	chorionic somator	CSHL1	5.28	5.21	5.14
212635_at	transportin 1	TNPO1	12.15	12.14	12.39
213358_at	KIAA0802	KIAA0802	8.11	8.16	8.08
201350_at	flotillin 2	FLOT2	8.85	8.82	8.74
213667_at	Snf2-related CREB	SRCAP	7.65	7.32	7.33
214931_s_at	SFRS protein kinas	SRPK2	7.31	7.31	7.28
221195_at	ring finger protein	RNFT1	3.38	3.43	3.44
216978_x_at	---	---	3.52	3.49	3.49
202294_at	stromal antigen 1	STAG1	9.29	9.27	9.36
201696_at	splicing factor, arg	SFRS4	10.63	10.62	10.73
219398_at	cell death-inducin	CIDEC	7.11	6.99	7.10
203880_at	COX17 cytochrom	COX17	10.84	10.89	11.01
201516_at	spermidine syntha	SRM	10.10	10.04	10.13
203322_at	ADNP homeobox	ADNP2	9.93	9.82	9.95
204476_s_at	pyruvate carboxyl	PC	7.51	7.65	7.76
204026_s_at	ZW10 interactor	ZWINT	11.87	11.87	11.83
210290_at	zinc finger protein	ZNF174	6.44	6.41	6.33
215359_x_at	zinc finger protein	ZNF44	7.22	7.41	7.47

207757_at	zinc finger protein	ZFP2		3.62	3.71	3.55
205569_at	lysosomal-associa	LAMP3		4.23	4.34	4.21
219225_at	piggyBac transpos	PGBD5		5.19	5.13	5.11
215117_at	recombination act	RAG2		3.11	2.99	3.05
202918_s_at	MOB1, Mps One E	MOBKL3		10.29	10.36	10.40
202955_s_at	ADP-ribosylation f	ARFGEF1		7.95	7.96	7.89
203166_at	craniofacial devel	CFDP1		10.17	10.14	10.18
204747_at	interferon-induce	IFIT3		6.67	6.79	6.28
205052_at	AU RNA binding p	AUH		7.64	7.61	7.85
205268_s_at	adducin 2 (beta)	ADD2		7.93	7.69	7.96
209118_s_at	tubulin, alpha 1a	TUBA1A		12.11	12.27	12.14
212818_s_at	ankyrin repeat an	ASB1		9.25	9.32	9.22
213666_at	septin 6		6-Sep	5.57	5.60	5.59
208430_s_at	dystrobrevin, alph	DTNA		5.91	5.85	6.13
207773_x_at	cytochrome P450,	CYP3A43		3.87	3.84	3.92
214434_at	heat shock 70kDa	HSPA12A		6.86	6.82	6.74
213714_at	calcium channel, v	CACNB2		3.66	3.51	3.54
211655_at	Hypothetical prot	LOC100287927		3.83	3.75	3.65
208853_s_at	calnexin	CANX		9.60	9.53	9.64
201429_s_at	ribosomal protein	RPL37A		15.28	15.27	15.31
218575_at	anaphase promot	ANAPC1		10.03	9.99	9.98
201617_x_at	caldesmon 1	CALD1		7.60	7.82	8.19
211598_x_at	vasoactive intesti	VIPR2		5.86	5.81	5.91
205538_at	coronin, actin bin	CORO2A		5.03	4.91	4.77
210888_s_at	inter-alpha (globu	ITIH1		6.47	6.35	6.14
216899_s_at	src kinase associa	SKAP2		8.06	8.26	8.31
204525_at	PHD finger protein	PHF14		5.25	5.46	5.71
210937_s_at	pancreatic and du	PDX1		4.28	4.55	4.35
218645_at	zinc finger protein	ZNF277		9.46	9.51	9.51
219215_s_at	solute carrier fam	SLC39A4		8.62	8.53	8.52
218428_s_at	REV1 homolog (S.	REV1		9.30	9.28	9.39
219247_s_at	zinc finger, DHHC-	ZDHHC14		8.18	8.23	8.21
210367_s_at	prostaglandin E sy	PTGES		7.39	7.34	7.40
206943_at	transforming grov	TGFBR1		5.47	5.61	5.54
35666_at	sema domain, imr	SEMA3F		8.93	8.92	8.79
215287_at	Similar to hCG198	LOC100288939		5.58	5.85	5.71
216264_s_at	laminin, beta 2 (la	LAMB2		8.24	8.23	8.18
220672_at	protein phosphata	PPP4R4		5.52	5.52	5.59
200946_x_at	glutamate dehydr	GLUD1		9.66	9.69	9.68
221058_s_at	chemokine-like fa	CKLF		9.97	9.91	9.97
215018_at	KIAA1731	KIAA1731		4.99	4.87	5.02
201639_s_at	cleavage and poly	CPSF1		7.77	7.63	7.67
202169_s_at	aminoadipate-sen	AASDHPPT		10.72	10.82	10.83
204025_s_at	programmed cell	PDCD2		8.47	8.31	8.64
221159_at	---	---		5.10	4.82	5.02
214344_at	hypothetical LOC9	LOC92973		4.30	4.25	4.18
210301_at	xanthine dehydro	XDH		6.26	6.59	6.29

211324_s_at	RANBP2-like and (	RGPD5 /// RGPD6	3.65	3.57	3.64
202754_at	R3H domain conta	R3HDM1	10.09	10.05	10.12
202329_at	c-src tyrosine kina	CSK	8.83	8.75	8.81
208851_s_at	Thy-1 cell surface	THY1	6.04	5.96	6.06
212880_at	WD repeat domai	WDR7	8.26	8.22	8.30
201148_s_at	TIMP metallopept	TIMP3	6.74	6.76	6.59
219044_at	threonine synthas	THNSL2	5.48	5.53	5.34
213579_s_at	E1A binding prote	EP300	7.98	7.93	7.94
203321_s_at	ADNP homeobox	ADNP2	9.33	9.14	9.23
215013_s_at	ubiquitin specific	USP34	6.29	6.24	6.32
221016_s_at	transcription facto	TCF7L1	5.56	5.61	5.40
210779_x_at	survival of motor	SIP1	9.17	9.18	9.19
211665_s_at	son of sevenless h	SOS2	5.92	5.88	6.08
222292_at	CD40 molecule, TI	CD40	4.31	4.10	4.10
215197_at	---	---	3.60	3.64	3.54
221324_at	taste receptor, typ	TAS2R1	4.56	4.44	4.48
218425_at	ring finger proteir	RNF216	7.24	7.28	7.25
206053_at	zinc finger proteir	ZNF510	5.72	5.89	5.76
212778_at	phosphofurin acid	PACS2	7.32	7.11	7.08
200729_s_at	ARP2 actin-relate	ACTR2	11.80	11.88	11.98
202534_x_at	dihydrofolate redu	DHFR	10.57	10.61	10.71
209844_at	homeobox B13	HOXB13	4.05	4.12	4.15
201554_x_at	glycogenin 1	GYG1	10.45	10.44	10.53
208717_at	oxidase (cytochro	OXA1L	10.13	10.08	10.01
216202_s_at	serine palmitoyltr	SPTLC2	6.69	6.56	6.71
54970_at	zinc finger, MIZ-ty	ZMIZ2	10.10	10.11	10.06
214182_at	---	---	8.44	8.49	8.82
202609_at	epidermal growth	EPS8	12.34	12.27	12.41
201077_s_at	NHP2 non-histone	NHP2L1	12.24	12.23	12.23
213449_at	processing of prec	POP1	8.07	8.24	8.16
219690_at	transmembrane p	TMEM149	7.57	7.63	7.62
201134_x_at	cytochrome c oxic	COX7C	13.70	13.73	13.81
217839_at	TRK-fused gene	TFG	11.15	11.17	11.29
203973_s_at	CCAAT/enhancer	CEBPD	11.71	11.55	11.74
211791_s_at	potassium voltage	KCNAB2	6.80	6.79	6.84
206522_at	maltase-glucoamy	MGAM	4.32	4.63	4.61
219387_at	coiled-coil domair	CCDC88A	8.04	8.25	8.36
206956_at	bone gamma-carb	BGLAP	6.27	6.13	6.10
206855_s_at	hyaluronoglucosa	HYAL2	8.40	8.40	8.37
211056_s_at	steroid-5-alpha-re	SRD5A1	8.47	8.41	8.38
204308_s_at	tectonin beta-pro	TECPR2	7.54	7.56	7.49
219488_at	alpha 1,4-galactos	A4GALT	7.33	7.15	7.16
210638_s_at	F-box protein 9	FBXO9	10.81	10.78	10.82
202233_s_at	ubiquinol-cytochr	UQCRH	13.44	13.51	13.55
212015_x_at	polypyrimidine tra	PTBP1	10.53	10.56	10.53
210361_s_at	E74-like factor 2 (	ELF2	7.02	7.05	7.21
203948_s_at	myeloperoxidase	MPO	4.31	4.23	4.26

206538_at	muscle RAS oncog	MRAS	7.05	6.85	6.70
216824_at	---	---	5.67	5.74	5.72
214160_at	---	---	4.82	4.66	4.62
218271_s_at	presenilin associa	PARL	10.31	10.40	10.38
203740_at	M-phase phospho	MPHOSPH6	10.18	10.22	10.16
209579_s_at	methyl-CpG bindi	MBD4	10.40	10.41	10.55
209903_s_at	ataxia telangiecta	ATR	10.21	10.38	10.37
220870_at	---	---	5.53	5.51	5.49
221649_s_at	peter pan homolo	PPAN	10.08	9.89	10.13
211414_at	glutaminase	GLS	4.74	4.63	4.71
209492_x_at	ATP synthase, H+	ATP5I	11.60	11.76	11.77
203741_s_at	adenylate cyclase	ADCY7	7.74	7.95	7.96
214670_at	zinc finger with KI	ZKSCAN1	8.26	8.50	8.40
201799_s_at	oxysterol binding	OSBP	8.00	8.03	8.01
213637_at	DEAD (Asp-Glu-Al	DDX52	9.00	9.08	9.21
202976_s_at	Rho-related BTB d	RHOBTB3	7.98	7.84	8.17
221743_at	CUGBP, Elav-like f	CELF1	11.99	11.94	11.99
37590_g_at	---	---	5.75	5.76	5.69
200767_s_at	family with seque	FAM120A	8.33	8.36	8.37
217157_x_at	immunoglobulin k	IGK@ /// IGKC ///	5.54	5.73	5.56
221766_s_at	family with seque	FAM46A	7.05	7.04	6.91
44065_at	chromosome 12 c	C12orf52	8.14	8.19	8.27
218815_s_at	transmembrane p	TMEM51	7.85	8.02	7.85
212917_x_at	RecQ protein-like	RECQL	9.92	10.05	10.30
32042_at	DiGeorge syndron	DGCR14	7.05	7.10	7.25
209714_s_at	cyclin-dependent	CDKN3	11.56	11.63	11.63
203891_s_at	death-associated	DAPK3	7.36	7.24	7.24
221571_at	TNF receptor-assc	TRAF3	10.07	9.89	10.00
201745_at	twinfilin, actin-bin	TWF1	10.68	10.82	10.91
201829_at	neuroepithelial ce	NET1	9.56	9.60	9.64
200860_s_at	CCR4-NOT transcr	CNOT1	10.55	10.47	10.61
220791_x_at	sodium channel, v	SCN11A	3.22	3.36	3.32
204866_at	PHD finger protei	PHF16	8.48	8.47	8.63
219180_s_at	peroxisomal bioge	PEX26	7.80	7.69	7.77
204759_at	regulator of chror	RCBTB2	7.04	7.15	7.04
208892_s_at	dual specificity ph	DUSP6	7.03	7.30	7.47
203150_at	Rab9 effector pro	RABEPK	9.63	9.56	9.66
203328_x_at	insulin-degrading	IDE	8.03	8.13	8.09
205349_at	guanine nucleotid	GNA15	9.20	9.17	9.21
206734_at	jerky homolog-like	JRKL	8.27	8.49	8.40
209402_s_at	solute carrier fam	SLC12A4	8.25	8.07	8.13
220939_s_at	dipeptidyl-peptida	DPP8	10.23	10.29	10.24
215160_x_at	similar to FRG1 pr	LOC642236	9.47	9.48	9.62
209614_at	alcohol dehydroge	ADH1B	3.88	3.90	3.88
211935_at	ADP-ribosylation f	ARL6IP1	12.03	12.16	12.26
216470_x_at	protease, serine, 1	PRSS1 /// PRSS2 //	7.27	7.40	7.18
218124_at	retinol saturase (a	RETSAT	9.52	9.47	9.51

210057_at	SMG1 homolog, p	SMG1	5.90	5.73	6.03
219764_at	frizzled homolog 1	FZD10	3.66	3.74	3.65
213388_at	phosphodiesteras	PDE4DIP	8.17	8.45	8.44
202733_at	prolyl 4-hydroxyla	P4HA2	9.49	9.46	9.57
218055_s_at	WD repeat domai	WDR41	9.52	9.56	9.60
221549_at	glutamate-rich WI	GRWD1	8.86	8.65	8.72
216463_at	---	---	3.44	3.43	3.37
201859_at	serglycin	SRGN	12.26	12.51	12.56
222235_s_at	chondroitin sulfat	CSGALNACT2 /// L	8.92	8.76	8.95
207328_at	arachidonate 15-l	ALOX15	3.74	3.54	3.54
203335_at	phytanoyl-CoA 2-l	PHYH	10.60	10.52	10.68
204149_s_at	glutathione S-tran	GSTM4	4.98	5.15	4.98
203244_at	peroxisomal bioge	PEX5	10.59	10.44	10.55
219796_s_at	cadherin-related f	CDHR5	6.39	6.32	6.32
204982_at	G protein-couplec	GIT2	6.95	6.95	7.01
221009_s_at	angiopoietin-like 4	ANGPTL4	7.61	7.32	7.42
219882_at	tubulin tyrosine li	TTLL7	6.99	6.82	6.87
216450_x_at	heat shock protei	HSP90B1	8.75	8.77	8.79
205061_s_at	exosome compon	EXOSC9	9.44	9.45	9.43
202665_s_at	WAS/WASL intera	WIPF1	7.46	7.33	7.37
207268_x_at	abl-interactor 2	ABI2	8.35	8.40	8.33
202719_s_at	testis derived trar	TES	5.82	5.81	5.66
221852_at	---	---	4.55	4.55	4.60
207333_at	neuromedin B rec	NMBR	3.46	3.64	3.50
201992_s_at	kinesin family mei	KIF5B	8.51	8.64	8.67
200655_s_at	calmodulin 1 (pho	CALM1	12.05	12.14	12.08
203020_at	RAB GTPase activa	RABGAP1L	7.87	7.98	7.92
211643_x_at	immunoglobulin k	IGK@ /// IGKC ///	5.48	5.49	5.53
216725_at	Doublecortin-like	DCLK2	5.67	5.48	5.39
204481_at	bromodomain and	BRPF1	7.64	7.62	7.62
204254_s_at	vitamin D (1,25- d	VDR	8.82	8.63	8.76
219459_at	polymerase (RNA)	POLR3B	9.34	9.34	9.41
207318_s_at	cyclin-dependent	CDK13	8.78	8.78	8.74
213117_at	kelch-like 9 (Dros	KLHL9	5.93	6.46	6.32
214331_at	Ts translation elor	TSFM	6.86	7.01	6.93
215644_at	zinc finger proteir	ZNF518A	4.76	4.86	4.89
218485_s_at	solute carrier fam	SLC35C1	7.69	7.67	7.75
208358_s_at	UDP glycosyltrans	UGT8	5.06	5.03	5.38
206757_at	phosphodiesteras	PDE5A	3.66	3.68	3.77
206703_at	cholinergic recept	CHRNB1	5.23	5.35	5.40
212528_at	---	---	8.80	8.89	8.80
202081_at	immediate early r	IER2	10.46	10.57	10.45
205943_at	tryptophan 2,3-di	TDO2	6.18	6.40	6.31
219770_at	glycosyltransferas	GTDC1	8.02	7.96	7.97
217356_s_at	phosphoglycerate	PGK1	12.25	12.23	12.28
200631_s_at	SET nuclear oncog	SET	13.27	13.25	13.20
209687_at	chemokine (C-X-C	CXCL12	4.31	4.60	4.29

206016_at	coiled-coil domair	CCDC22	7.19	7.23	7.34
217795_s_at	transmembrane p	TMEM43	10.93	10.85	10.84
217499_x_at	olfactory receptor	OR7E37P	8.57	8.47	8.59
219083_at	SHQ1 homolog (S.	SHQ1	10.32	10.37	10.32
218862_at	ankyrin repeat an	ASB13	10.10	9.96	9.86
220759_at	epididymal protei	EDDM3B	3.29	3.05	2.98
213904_at	---	---	3.45	3.52	3.59
219451_at	methionine sulfox	MSRB2	8.00	8.15	8.03
208924_at	ring finger proteir	RNF11	10.51	10.61	10.69
202671_s_at	pyridoxal (pyridox	PDXK	9.05	8.96	9.10
213218_at	zinc finger proteir	ZNF187	6.99	7.08	6.96
205627_at	cytidine deaminas	CDA	6.55	6.63	6.80
206459_s_at	wingless-type MM	WNT2B	5.68	5.61	5.53
206558_at	single-minded hor	SIM2	8.33	8.27	8.36
213499_at	chloride channel 2	CLCN2	7.34	7.28	6.94
211233_x_at	estrogen receptor	ESR1	5.85	5.91	5.90
219067_s_at	non-SMC element	NSMCE4A	10.58	10.64	10.75
219807_x_at	RAB4B, member F	RAB4B	7.45	7.42	7.38
206256_at	carboxypeptidase	CPN1	5.29	5.21	5.37
207604_s_at	solute carrier fam	SLC4A7	8.36	8.25	8.34
204736_s_at	chondroitin sulfat	CSPG4	7.08	7.00	6.94
219825_at	cytochrome P450,	CYP26B1	5.47	5.32	5.17
219722_s_at	glycerophosphodi	GDPD3	5.49	5.45	5.32
217052_x_at	---	---	7.18	7.17	7.22
211707_s_at	IQ motif containir	IQCB1	8.57	8.50	8.66
207524_at	suppression of tur	ST7	6.12	6.40	6.16
203501_at	plasma glutamate	PGCP	6.69	6.72	6.78
207334_s_at	transforming grov	TGFBR2	6.71	6.56	6.59
201508_at	insulin-like growtl	IGFBP4	8.52	8.47	8.48
203392_s_at	C-terminal binding	CTBP1	10.04	10.03	10.01
207229_at	killer cell lectin-lik	KLRA1	4.07	3.96	3.86
218110_at	XPA binding prote	XAB2	6.87	6.65	6.75
219919_s_at	slingshot homolog	SSH3	7.78	7.90	7.85
202017_at	epoxide hydrolase	EPHX1	6.22	6.24	6.29
219345_at	bolA homolog 1 (E	BOLA1	7.42	7.51	7.55
209588_at	EPH receptor B2	EPHB2	6.70	7.30	7.09
213329_at	SLIT-ROBO Rho GT	SRGAP2	7.04	7.00	6.87
208718_at	DEAD (Asp-Glu-Ala	DDX17	11.82	12.00	11.82
204610_s_at	coiled-coil domair	CCDC85B	10.06	9.86	10.01
218905_at	integrator comple	INTS8	10.12	10.14	10.11
208254_at	---	---	4.88	4.82	4.68
218450_at	heme binding pro	HEBP1	10.19	10.12	10.25
201612_at	aldehyde dehydro	ALDH9A1	11.07	11.03	11.16
219725_at	triggering recepto	TREM2	6.78	7.06	6.61
201372_s_at	cullin 3	CUL3	5.49	5.59	5.55
217190_x_at	estrogen receptor	ESR1	4.21	4.27	4.28
212032_s_at	prostate tumor ov	PTOV1	8.35	8.36	8.39

216289_at	G protein-couplec	GPR144	6.23	5.97	6.16
202330_s_at	uracil-DNA glycos	UNG	7.84	7.83	7.99
213088_s_at	DnaJ (Hsp40) hom	DNAJC9	11.14	11.15	11.17
200717_x_at	ribosomal protein	RPL7	14.78	14.82	14.81
222167_at	---	---	6.71	6.80	6.75
214020_x_at	Integrin, beta 5	ITGB5	8.80	9.00	8.98
214499_s_at	BCL2-associated t	BCLAF1	9.10	9.30	9.21
222360_at	DPH5 homolog (S.	DPH5	7.25	7.45	7.47
209995_s_at	T-cell leukemia/ly	TCL1A	5.18	5.17	4.99
217046_s_at	advanced glycosyl	AGER	6.75	6.76	6.51
215733_x_at	cancer/testis anti	CTAG2	5.75	5.65	5.63
219056_at	ribonuclease H2, s	RNASEH2B	7.88	7.95	8.02
221628_s_at	glyoxylate reducta	GLYR1	5.45	5.48	5.38
211673_s_at	molybdenum cofa	MOCS1	6.61	6.32	6.45
208874_x_at	protein phosphata	PPP2R4	7.45	7.35	7.37
201045_s_at	RAB6A, member F	RAB6A	6.82	6.68	6.61
221520_s_at	cell division cycle	CDCA8	9.89	9.92	9.93
214395_x_at	eukaryotic transla	EEF1D	7.22	7.25	7.31
91617_at	DiGeorge syndron	DGCR8	6.91	6.90	6.87
216827_at	---	---	4.48	4.41	4.38
212825_at	PAX interacting (w	PAXIP1	9.13	9.30	9.34
214535_s_at	ADAM metallopep	ADAMTS2	5.50	5.24	5.17
218736_s_at	palmdelphin	PALMD	3.70	3.51	3.48
215581_s_at	minichromosome	MCM3AP	4.92	4.61	4.79
221542_s_at	ER lipid raft associ	ERLIN2	7.81	7.96	7.94
204709_s_at	kinesin family me	KIF23	9.78	9.87	9.99
217497_at	thymidine phosph	TYMP	7.11	6.90	7.08
204641_at	NIMA (never in m	NEK2	10.43	10.52	10.70
218092_s_at	ArfGAP with FG re	AGFG1	9.61	9.54	9.68
200807_s_at	heat shock 60kDa	HSPD1	14.04	14.08	14.03
220972_s_at	keratin associatec	KRTAP9-9	3.18	3.30	3.39
217602_at	peptidylprolyl isor	PPIA	4.40	4.32	4.51
208643_s_at	X-ray repair comp	XRCC5	10.53	10.62	10.61
213797_at	radical S-adenosyl	RSAD2	3.96	3.95	4.06
202430_s_at	phospholipid scra	PLSCR1	8.89	8.74	8.94
201744_s_at	lumican	LUM	3.08	3.08	3.12
218766_s_at	tryptophanyl tRN/	WARS2	8.30	8.27	8.28
217135_x_at	---	---	7.17	7.11	6.95
218586_at	chromosome 20 c	C20orf20	8.39	8.42	8.41
212676_at	neurofibromin 1	NF1	8.55	8.61	8.59
215660_s_at	microtubule assoc	MAST2	6.41	6.25	6.23
206084_at	protein tyrosine p	PTPRR	4.32	4.31	4.39
219937_at	thyrotropin-releas	TRHDE	6.86	6.67	6.91
205925_s_at	RAB3B, member F	RAB3B	5.61	5.54	5.55
212878_s_at	kinesin light chain	KLC1	9.21	9.12	9.27
216320_x_at	macrophage stimu	MST1	7.46	7.56	7.62
202806_at	ATP-binding casse	ABCC1	8.68	8.67	8.86

211569_s_at	hydroxyacyl-CoA d	HADH	9.10	9.09	9.26
201465_s_at	jun oncogene	JUN	8.22	8.19	8.14
209500_x_at	TNFSF12-TNFSF13	TNFSF12-TNFSF13	6.15	6.02	6.21
200697_at	hexokinase 1	HK1	11.77	11.77	11.63
211137_s_at	ATPase, Ca++ tran	ATP2C1	8.86	9.07	9.07
207060_at	engrailed homeob	EN2	6.11	6.06	6.13
222047_s_at	serrate RNA effec	SRRT	10.54	10.64	10.64
205110_s_at	fibroblast growth	FGF13	5.80	5.66	5.89
217106_x_at	DIM1 dimethylade	DIMT1L	11.10	11.22	11.23
202358_s_at	sorting nexin 19	SNX19	7.76	7.80	7.86
202071_at	syndecan 4	SDC4	10.61	10.45	10.63
203865_s_at	adenosine deamir	ADARB1	9.87	9.94	9.92
214080_x_at	protein kinase C s	PRKCSH	9.06	9.04	9.10
222220_s_at	translin-associate	TSNAXIP1	6.67	6.56	6.62
210187_at	FK506 binding pro	FKBP1A	7.87	7.94	7.94
205673_s_at	ankyrin repeat an	ASB9	5.86	5.87	5.76
221765_at	UDP-glucose cerar	UGCG	8.73	8.92	8.83
221486_at	endosulfine alpha	ENSA	9.17	8.94	9.09
211670_x_at	synovial sarcoma,	SSX3	5.74	5.77	5.75
210869_s_at	melanoma cell ad	MCAM	7.85	7.94	7.74
217914_at	two pore segment	TPCN1	7.98	8.06	7.95
217862_at	protein inhibitor c	PIAS1	8.84	8.88	8.81
213281_at	similar to c-jun	LOC100288387	6.26	6.45	6.33
219210_s_at	RAB8B, member R	RAB8B	5.99	6.17	6.10
203179_at	galactose-1-phosp	GALT	7.39	7.19	7.32
208707_at	eukaryotic transla	EIF5	7.48	7.45	7.63
220723_s_at	cell wall biogenesi	CWH43	4.49	4.29	4.32
202159_at	phenylalanyl-tRNA	FARSA	9.28	9.29	9.32
213243_at	vacuolar protein s	VPS13B	8.66	8.62	8.78
218356_at	FtsJ homolog 2 (E.	FTSJ2	9.79	9.85	9.83
65718_at	G protein-couplec	GPR124	6.48	6.31	6.54
205900_at	keratin 1	KRT1	4.70	4.57	4.56
204113_at	CUGBP, Elav-like f	CELF1	7.75	7.77	7.74
222339_x_at	---	---	7.03	7.14	7.08
212461_at	antizyme inhibitor	AZIN1	11.35	11.32	11.45
208770_s_at	eukaryotic transla	EIF4EBP2	7.87	8.01	7.82
201848_s_at	BCL2/adenovirus I	BNIP3	9.89	9.87	9.91
205270_s_at	lymphocyte cytos	LCP2	3.92	3.91	3.84
203867_s_at	notchless homolo	NLE1	8.87	8.84	8.87
210514_x_at	major histocompa	HLA-G	8.96	8.83	9.00
207786_at	cytochrome P450,	CYP2R1	7.20	7.04	7.21
205180_s_at	ADAM metallopep	ADAM8	6.70	6.83	6.76
202676_x_at	Fas-activated serii	FASTK	8.20	8.08	8.05
216579_at	gap junction prote	GJB4	5.26	5.23	5.23
217251_x_at	---	---	6.16	6.26	6.17
210651_s_at	EPH receptor B2	EPHB2	6.93	7.08	6.71
211110_s_at	androgen recepto	AR	6.98	6.77	6.91

203406_at	microfibrillar-asso	MFAP1	10.17	10.26	10.27
51226_at	RNA binding moti	RBM12B	4.36	4.19	4.33
214585_s_at	vacuolar protein s	VPS52	9.33	9.34	9.31
203592_s_at	follistatin-like 3 (s	FSTL3	8.58	8.63	8.53
218592_s_at	cat eye syndrome	CECR5	9.89	9.83	9.94
222034_at	Guanine nucleotic	GNB2L1	8.17	8.17	8.18
216576_x_at	immunoglobulin k	IGK@ /// IGKC ///	5.10	5.12	5.02
216404_at	ATXN8 opposite s	ATXN8OS	4.51	4.35	4.49
204828_at	RAD9 homolog A (	RAD9A	6.58	6.87	6.62
221498_at	sorting nexin fami	SNX27	8.71	8.70	8.57
221095_s_at	potassium voltage	KCNE2	6.16	6.27	6.17
218027_at	mitochondrial ribo	MRPL15	11.58	11.59	11.69
216222_s_at	myosin X	MYO10	8.86	8.81	8.88
209654_at	KIAA0947	KIAA0947	10.69	10.79	10.80
216121_at	---	---	3.69	3.85	3.68
202497_x_at	solute carrier fam	SLC2A3	7.52	7.50	7.67
203868_s_at	vascular cell adhe	VCAM1	6.01	5.52	5.80
214922_at	zinc finger proteir	ZNF484	4.66	4.50	4.52
216310_at	TAO kinase 1	TAOK1	7.78	7.80	7.63
208955_at	deoxyuridine tripł	DUT	9.71	9.72	9.73
215939_at	---	---	4.42	4.41	4.21
219284_at	HSPB (heat shock	HSPBAP1	7.91	7.81	8.20
214120_at	RFPL1 antisense R	RFPL1S	3.46	3.31	3.24
202028_s_at	ribosomal protein	RPL38	11.54	11.53	11.50
218295_s_at	nucleoporin 50kD	NUP50	7.70	7.78	7.78
208250_s_at	deleted in maligna	DMBT1	8.60	8.32	8.45
213366_x_at	ATP synthase, H+	ATP5C1	12.80	12.79	12.74
218983_at	complement com	C1RL	6.62	6.89	6.99
201473_at	jun B proto-oncog	JUNB	8.22	7.90	8.08
215434_x_at	neuroblastoma br	LOC100288142 ///	8.05	7.90	8.12
206445_s_at	protein arginine m	PRMT1	11.72	11.63	11.64
219154_at	transmembrane p	TMEM120B	8.27	8.21	8.23
218662_s_at	non-SMC condens	NCAPG	10.69	10.81	10.77
212401_s_at	cyclin-dependent	CDK11A	9.62	9.66	9.69
213805_at	abhydrolase doma	ABHD5	7.40	7.56	7.40
214354_x_at	surfactant proteir	SFTPB	6.40	6.25	6.55
214073_at	cortactin	CTTN	7.61	7.48	7.52
215885_at	synovial sarcoma,	SSX7	4.16	4.10	4.02
205832_at	carboxypeptidase	CPA4	7.01	7.27	7.18
206974_at	chemokine (C-X-C	CXCR6	5.37	5.28	5.20
219615_s_at	potassium channe	KCNK5	5.97	5.76	5.82
44617_at	2-oxoglutarate an	OGFOD2	7.94	7.94	7.92
210905_x_at	POU class 5 home	POU5F1P4	6.35	6.35	6.25
209628_at	nuclear transport	NXT2	9.31	9.56	9.43
208455_at	poliovirus recepto	PVRL1	5.20	5.32	5.33
215220_s_at	translocated pron	TPR	5.01	5.01	5.04
205868_s_at	protein tyrosine p	PTPN11	6.33	6.39	6.35

213870_at	collagen, type XI, a	COL11A2	6.60	6.49	6.52
203432_at	thymopoietin	TMPO	10.97	10.87	11.08
213377_x_at	ribosomal protein	RPS12	14.95	14.94	14.94
219202_at	rhomboid 5 homo	RHBDF2	7.65	7.52	7.65
213002_at	myristoylated alar	MARCKS	8.99	9.06	9.05
210153_s_at	malic enzyme 2, N	ME2	9.86	9.98	9.92
204974_at	RAB3A, member F	RAB3A	6.59	6.49	6.49
218335_x_at	TNFAIP3 interactir	TNIP2	8.79	8.81	8.74
218328_at	coenzyme Q4 hon	COQ4	7.88	7.90	7.96
202552_s_at	cysteine rich trans	CRIM1	10.41	10.41	10.50
217837_s_at	vacuolar protein s	VPS24	10.04	10.14	10.31
219374_s_at	asparagine-linked	ALG9	7.18	7.24	7.28
204351_at	S100 calcium bind	S100P	7.69	7.39	7.51
214308_s_at	homogentisate 1,2	HGD	5.77	5.80	5.74
214306_at	optic atrophy 1 (a	OPA1	8.61	8.77	8.78
203736_s_at	PTPRF interacting	PPFIBP1	6.52	6.38	6.42
215627_at	---	---	3.88	3.81	3.80
201819_at	scavenger recepto	SCARB1	8.54	8.43	8.47
209249_s_at	growth hormone i	GHITM	12.31	12.27	12.36
221164_x_at	carbohydrate (N-a	CHST5	6.71	6.62	6.89
206081_at	solute carrier fam	SLC24A1	8.07	7.92	8.03
216905_s_at	suppression of tur	ST14	3.80	3.70	4.00
202690_s_at	small nuclear ribo	SNRPD1	10.95	11.06	11.16
209433_s_at	phosphoribosyl py	PPAT	9.86	9.84	9.99
204142_at	enolase superfam	ENOSF1	9.78	9.85	9.81
215386_at	---	---	4.96	5.00	4.81
221277_s_at	pseudouridylate s	PUS3	8.22	8.28	8.37
221584_s_at	potassium large c	KCNMA1	10.82	10.67	10.85
214295_at	hypothetical LOC5	KIAA0485	6.26	6.13	6.29
212997_s_at	tousled-like kinase	TLK2	8.76	8.84	8.88
213053_at	HAUS augmin-like	HAUS5	7.04	7.12	7.13
219218_at	BAH domain and c	BAHCC1	7.40	7.20	7.31
35776_at	intersectin 1 (SH3	ITSN1	9.20	9.33	9.28
219324_at	nucleolar protein	NOL12 /// TRIOBP	7.82	7.83	7.98
208449_s_at	fibroblast growth	FGF8	5.18	5.08	4.97
214004_s_at	vestigial like 4 (Dr	VGLL4	8.07	8.18	8.07
204161_s_at	ectonucleotide py	ENPP4	5.06	5.08	5.43
205435_s_at	AP2 associated kir	AAK1	6.38	6.29	6.46
219631_at	low density lipopr	LRP12	8.33	8.22	8.49
218017_s_at	heparan-alpha-glu	HGSNAT	8.31	8.42	8.48
214702_at	fibronectin 1	FN1	5.62	5.58	5.80
210826_x_at	RAD17 homolog (S	RAD17	9.51	9.61	9.63
203998_s_at	synaptotagmin I	SYT1	6.34	6.28	6.72
205279_s_at	glycine receptor, b	GLRB	7.35	7.27	7.55
201030_x_at	lactate dehydroge	LDHB	13.81	13.84	13.80
221620_s_at	apolipoprotein O	APOO	8.83	8.83	8.85
201179_s_at	guanine nucleotid	GNAI3	9.38	9.38	9.38

202080_s_at	trafficking protein	TRAK1	8.81	8.68	8.65
203850_s_at	kinesin family mei	KIF1A	3.24	3.35	3.25
214556_at	somatostatin rece	SSTR4	5.11	4.88	5.04
204642_at	sphingosine-1-pho	S1PR1	4.71	5.16	4.97
204515_at	hydroxy-delta-5-s	HSD3B1	4.65	4.69	4.42
205062_x_at	AT rich interactive	ARID4A	7.44	7.80	7.68
218840_s_at	NAD synthetase 1	NADSYN1	8.86	8.74	8.73
201662_s_at	acyl-CoA syntheta	ACSL3	11.12	11.22	11.42
209114_at	tetraspanin 1	TSPAN1	7.00	6.90	7.03
218427_at	serologically defin	SDCCAG3	10.28	10.10	10.32
217521_at	---	---	4.79	4.78	4.78
214145_s_at	spectrin, beta, ery	SPTB	6.85	6.68	6.59
210513_s_at	vascular endothel	VEGFA	7.32	7.30	7.32
222160_at	A kinase (PRKA) ai	AKAP8L	6.84	7.02	6.80
207608_x_at	cytochrome P450,	CYP1A2	6.10	6.25	6.27
214007_s_at	twinfilin, actin-bin	TWF1	7.26	7.42	7.35
218574_s_at	LIM and cysteine-	LMCD1	8.11	8.25	8.16
204253_s_at	vitamin D (1,25- d	VDR	7.33	7.28	7.25
211774_s_at	methylmalonic ac	MMACHC	8.30	8.13	8.28
203246_s_at	nitrogen permeas	NPRL2	8.87	8.85	8.90
212429_s_at	general transcript	GTF3C2	10.28	10.34	10.28
202763_at	caspase 3, apopto	CASP3	9.10	9.16	9.16
222001_x_at	hypothetical LOC7	LOC728855	10.40	10.19	10.35
220060_s_at	chromosome 12 c	C12orf48	7.07	7.15	7.11
205631_at	KIAA0586	KIAA0586	8.24	8.36	8.31
213285_at	transmembrane p	TMEM30B	3.46	3.32	3.53
212154_at	syndecan 2	SDC2	8.31	8.32	8.26
218303_x_at	lysine-rich coiled-	KRCC1	9.87	9.77	10.08
219854_at	zinc finger protein	ZNF14	7.14	7.31	7.17
205844_at	vanin 1	VNN1	5.24	5.49	5.57
213659_at	zinc finger protein	ZNF75D	6.26	6.28	6.39
202439_s_at	iduronate 2-sulfat	IDS	7.09	7.19	7.18
206858_s_at	homeobox C6	HOXC6	9.86	9.97	9.86
203428_s_at	ASF1 anti-silencin	ASF1A	9.14	9.06	9.27
211320_s_at	protein tyrosine p	PTPRU	8.44	8.37	8.31
217853_at	tensin 3	TNS3	11.11	11.06	11.10
214039_s_at	lysosomal protein	LAPTM4B	12.86	12.91	12.96
201458_s_at	budding uninhibit	BUB3	11.74	11.74	11.90
211988_at	SWI/SNF related,	SMARCE1	10.97	11.06	11.11
208456_s_at	related RAS viral (	RRAS2	9.52	9.44	9.59
207469_s_at	pirin (iron-binding	PIR	9.84	9.83	10.02
213188_s_at	MYC induced nucl	MINA	10.37	10.31	10.50
206221_at	RAS p21 protein a	RASA3	4.19	4.36	4.48
205239_at	amphiregulin	AREG	10.99	10.97	11.17
218371_s_at	paraspeckle comp	PSPC1	8.23	8.28	8.20
202546_at	vesicle-associated	VAMP8	5.71	5.77	5.62
215711_s_at	WEE1 homolog (S	WEE1	8.06	8.13	8.11

214895_s_at	ADAM metallopep	ADAM10	6.97	7.12	7.05
210266_s_at	tripartite motif-co	TRIM33	9.66	9.75	9.74
202887_s_at	protein phosphata	PPP2R1B	13.93	13.87	13.94
207638_at	transmembrane p	TMPRSS15	11.02	11.09	11.35
212069_s_at	HLA-B associated	BAT2L1	7.09	7.06	6.91
213142_x_at	pigeon homolog (	PION	4.46	4.35	4.43
200912_s_at	eukaryotic transla	EIF4A2	13.67	13.70	13.74
203220_s_at	transducin-like en	TLE1	5.41	5.21	5.10
203759_at	ST3 beta-galactos	ST3GAL4	6.03	5.76	5.81
202913_at	Rho guanine nucle	ARHGEF11	7.99	7.78	7.77
201580_s_at	thioredoxin-relate	TMX4	10.19	10.17	10.21
219553_at	non-metastatic ce	NME7	9.91	9.96	10.04
212062_at	ATPase, class II, ty	ATP9A	8.56	8.48	8.53
208093_s_at	nudE nuclear distr	NDEL1	8.79	8.79	8.85
211256_x_at	butyrophilin, subf	BTN2A1	7.21	7.17	7.35
202663_at	WAS/WASL intera	WIPF1	6.76	6.89	6.92
207607_at	achaete-scute cor	ASCL2	3.87	3.88	3.69
217500_at	TIA1 cytotoxic gra	TIAL1	4.19	4.03	4.08
212347_x_at	MAX dimerization	MXD4	8.64	8.73	8.54
203857_s_at	protein disulfide is	PDIA5	9.48	9.60	9.55
201053_s_at	proteasome (pros	PSMF1	10.05	10.09	10.11
210995_s_at	tripartite motif-co	TRIM23	6.45	6.37	6.18
206945_at	lactase	LCT	5.05	5.14	5.37
213025_at	THUMP domain co	THUMPD1	8.99	9.14	9.29
203478_at	NADH dehydroger	NDUFC1	11.76	11.74	11.70
217492_s_at	phosphatase and	PTEN /// PTENP1	9.84	9.78	9.87
209669_s_at	SERPINE1 mRNA b	SERBP1	12.53	12.56	12.54
208331_at	basic charge, Y-lin	BPY2	3.94	3.75	3.78
65585_at	family with seque	FAM86B1	7.72	7.66	7.77
201845_s_at	RING1 and YY1 bi	RYBP	10.60	10.47	10.66
202000_at	NADH dehydroger	NDUFA6	9.19	9.22	9.24
221815_at	abhydrolase doma	ABHD2	8.81	8.54	8.81
207347_at	excision repair crc	ERCC6	6.93	7.03	6.96
205501_at	phosphodiesteras	PDE10A	3.27	3.28	3.02
214861_at	lysine (K)-specific	KDM4C	6.19	6.27	6.10
214085_x_at	GLI pathogenesis-	GLIPR1	9.36	9.55	9.55
202011_at	tight junction prot	TJP1	10.96	10.90	10.95
200804_at	transmembrane B	TMBIM6	11.59	11.52	11.73
206526_at	RIB43A domain w	RIBC2	5.70	5.73	5.76
209606_at	cytohesin 1 intera	CYTIP	8.46	8.35	8.77
201936_s_at	eukaryotic transla	EIF4G3	8.60	8.62	8.65
219100_at	oligonucleotide/o	OBFC1	9.04	9.09	9.18
212222_at	proteasome (pros	PSME4	8.51	8.57	8.48
201964_at	senataxin	SETX	8.23	8.32	8.31
210249_s_at	nuclear receptor c	NCOA1	8.03	7.97	8.07
204175_at	zinc finger proteir	ZNF593	10.18	10.05	10.15
207492_at	N-glycanase 1	NGLY1	6.94	7.21	6.99

218653_at	solute carrier fam	SLC25A15	10.32	10.12	10.17
221958_s_at	wntless homolog	WLS	10.33	10.30	10.51
204063_s_at	unc-51-like kinase	ULK2	5.98	5.87	5.86
204802_at	Ras-related associ	RRAD	4.74	5.10	4.90
219489_s_at	nucleoredoxin	NXN	11.06	10.91	10.98
205768_s_at	solute carrier fam	SLC27A2	7.89	7.53	8.02
212863_x_at	C-terminal binding	CTBP1	10.41	10.44	10.40
201948_at	guanine nucleotid	GNL2	9.59	9.67	9.75
219322_s_at	WD repeat domai	WDR8	8.52	8.55	8.50
201413_at	hydroxysteroid (1	HSD17B4	9.95	9.95	10.02
216819_at	---	---	3.66	3.62	3.53
218253_s_at	ligatin	LGTN	10.00	9.95	10.09
218202_x_at	mitochondrial ribo	MRPL44	6.17	6.24	6.29
205130_at	renal tumor antig	RAGE	9.68	9.70	9.87
213011_s_at	triosephosphate i	TPI1	12.76	12.74	12.76
219470_x_at	cyclin J	CCNJ	6.89	6.84	6.85
212600_s_at	ubiquinol-cytochr	UQCRC2	11.93	12.01	11.99
214199_at	surfactant protein	SFTPD	5.49	5.35	5.29
218314_s_at	chromosome 11 c	C11orf57	7.81	7.68	7.84
211003_x_at	transglutaminase	TGM2	7.61	7.68	7.37
200749_at	---	---	11.39	11.44	11.56
210975_x_at	Fas-activated seri	FASTK	8.18	8.18	8.02
212316_at	nucleoporin 210kl	NUP210	6.93	7.06	6.71
218705_s_at	sorting nexin 24	SNX24	8.16	8.09	8.14
205158_at	ribonuclease, RNa	RNASE4	5.89	6.03	6.04
215019_x_at	zinc finger protein	ZNF528	5.96	5.72	5.88
204270_at	v-ski sarcoma vira	SKI	8.39	8.24	8.42
200920_s_at	B-cell translocatio	BTG1	11.70	11.41	11.72
209018_s_at	PTEN induced put	PINK1	9.68	9.61	9.65
208338_at	purinergic recepto	P2RX3	5.07	4.86	4.63
203419_at	myeloid/lymphoic	MLL4	7.04	7.22	6.96
203962_s_at	nebulette	NEBL	9.82	9.76	9.98
216033_s_at	FYN oncogene rel	FYN	6.90	6.99	7.03
213793_s_at	homer homolog 1	HOMER1	8.36	8.47	8.68
210616_s_at	SEC31 homolog A	SEC31A	11.24	11.24	11.18
212575_at	chromosome 19 c	C19orf6	6.12	6.24	6.25
218199_s_at	nucleolar protein	NOL6	7.86	7.71	7.96
215381_at	Mechanistic targe	MTOR	5.44	5.50	5.51
203816_at	deoxyguanosine k	DGUOK	8.82	8.97	8.89
202178_at	protein kinase C, z	PRKCZ	5.65	5.54	5.47
219051_x_at	meteorin, glial cel	METRN	9.40	9.23	9.30
212442_s_at	LAG1 homolog, ce	LASS6	11.33	11.23	11.44
205710_at	low density lipopr	LRP2	3.99	4.09	4.15
34225_at	Wolf-Hirschhorn s	WHSC2	8.78	8.73	8.87
216274_s_at	SEC11 homolog A	SEC11A	12.08	12.10	12.14
201534_s_at	ubiquitin-like 3	UBL3	8.56	8.60	8.69
202530_at	mitogen-activatec	MAPK14	9.98	9.97	9.82

210511_s_at	inhibin, beta A	INHBA	5.69	5.34	5.50
203442_x_at	echinoderm micro	EML3	7.37	7.37	7.25
210530_s_at	nuclear receptor s	NR2C1	6.84	7.01	6.95
213603_s_at	ras-related C3 bot	RAC2	10.42	10.50	10.32
203811_s_at	DnaJ (Hsp40) hom	DNAJB4	7.04	7.59	7.28
201842_s_at	EGF-containing fib	EFEMP1	11.63	11.67	11.72
220655_at	TNFAIP3 interacti	TNIP3	5.83	5.81	5.96
211912_at	c-mer proto-onco	MERTK	3.41	3.45	3.28
219032_x_at	opsin 3	OPN3	10.69	10.84	10.63
209234_at	kinesin family me	KIF1B	7.99	8.06	8.05
203711_s_at	3-hydroxyisobutyr	HIBCH	7.64	7.68	7.69
218688_at	dihydroxyacetone	DAK	6.04	6.02	6.31
203622_s_at	partner of NOB1 h	PNO1	10.72	10.65	10.90
217468_at	Cytochrome P450	CYP2D6	7.05	6.94	6.98
210568_s_at	RecQ protein-like	RECQL	7.87	8.12	8.06
217208_s_at	discs, large homol	DLG1	7.21	7.27	7.12
216955_at	TAF1 RNA polyme	TAF1	4.90	4.83	4.71
34764_at	leucyl-tRNA synth	LARS2	9.43	9.43	9.49
214246_x_at	misshapen-like kir	MINK1	7.99	8.07	8.12
210521_s_at	fetuin B	FETUB	3.24	3.37	3.19
220387_s_at	HERV-H LTR-assoc	HHLA3	5.01	4.96	4.73
221334_s_at	forkhead box P3	FOXP3	6.08	5.83	5.97
201066_at	cytochrome c-1	CYC1	11.20	11.14	11.10
222121_at	Src homology 3 do	SGEF	4.03	3.88	3.81
222106_at	prion protein 2 (d	PRND	4.30	4.38	4.19
210881_s_at	insulin-like growth	IGF2 /// INS-IGF2	5.68	5.73	5.51
214902_x_at	---	---	7.79	7.79	7.87
219523_s_at	odz, odd Oz/ten-m	ODZ3	8.04	8.08	7.98
211937_at	eukaryotic transla	EIF4B	11.14	10.97	11.04
200687_s_at	splicing factor 3b,	SF3B3	9.70	9.72	9.76
218844_at	acyl-CoA syntheta	ACSF2	5.85	5.66	5.61
205593_s_at	phosphodiesteras	PDE9A	4.39	4.29	4.13
205953_at	leucine-rich repea	LRIG2	7.01	7.13	7.21
205871_at	plasminogen-like	PLGLA /// PLGLB1	3.65	3.67	3.74
204048_s_at	phosphatase and	PHACTR2	10.30	10.47	10.52
203455_s_at	spermidine/sperm	SAT1	9.22	9.26	9.58
213930_at	ATG12 autophagy	ATG12	5.65	5.57	5.71
218474_s_at	potassium channe	KCTD5	8.82	8.87	9.03
202883_s_at	nucleolar protein	NOL7	8.26	8.49	8.23
213522_s_at	Solute carrier fam	SLC16A3	4.53	4.68	4.38
216255_s_at	glutamate recepto	GRM8	3.60	3.46	3.56
202460_s_at	lipin 2	LPIN2	8.67	8.71	8.83
212055_at	chromosome 18 o	C18orf10	8.65	8.68	8.72
203556_at	zinc fingers and ho	ZHX2	8.07	7.98	8.08
204328_at	transmembrane c	TMC6	4.89	4.94	5.06
216306_x_at	polypyrimidine tra	PTBP1	11.09	10.96	11.07
219749_at	SH2 domain conta	SH2D4A	7.50	7.16	7.11

200705_s_at	eukaryotic transla	EEF1B2	13.52	13.46	13.49
212259_s_at	pre-B-cell leukemi	PBXIP1	6.98	6.89	6.97
210630_s_at	RAD52 homolog (!	RAD52	5.75	5.92	5.85
212788_x_at	ferritin, light poly	FTL	15.11	15.09	15.08
215051_x_at	allograft inflamma	AIF1	6.99	6.85	6.82
208584_at	synuclein, gamma	SNCG	5.86	5.75	5.58
207010_at	gamma-aminobut	GABRB1	3.71	3.74	3.88
219881_s_at	---	---	6.51	6.58	6.60
221082_s_at	NDRG family mem	NDRG3	7.19	7.22	7.04
200076_s_at	chromosome 19 c	C19orf50	8.83	8.81	8.86
218210_at	fructosamine 3 ki	FN3KRP	9.49	9.60	9.54
217870_s_at	cytidine monopho	CMPK1	11.97	12.03	12.02
205312_at	spleen focus form	SPI1	4.97	5.04	4.94
213487_at	Mitogen-activatec	MAP2K2	5.78	5.71	5.69
201159_s_at	N-myristoyltransf	NMT1	6.53	6.46	6.49
215830_at	SH3 and multiple	SHANK2	4.24	4.11	3.94
212741_at	monoamine oxida	MAOA	10.11	10.02	10.04
215722_s_at	small nuclear ribo	SNRPA1	9.74	9.79	9.81
202528_at	UDP-galactose-4-	GALE	9.59	9.61	9.61
206139_at	phosphatidylinosi	PI4KB	7.03	7.06	6.96
202381_at	ADAM metallopep	ADAM9	10.73	10.65	10.84
201440_at	DEAD (Asp-Glu-Al	DDX23	8.08	8.01	8.03
203597_s_at	WW domain bindi	WBP4	8.41	8.41	8.40
212003_at	chromosome 1 op	C1orf144	8.16	8.29	8.15
218375_at	nudix (nucleoside	NUDT9	9.12	9.23	9.20
221595_at	chromosome 7 op	C7orf64	6.47	6.40	6.47
211077_s_at	tousled-like kinase	TLK1	4.61	4.45	4.62
202548_s_at	Rho guanine nucle	ARHGEF7	9.50	9.40	9.45
213835_x_at	GTP binding prote	GTPBP3	8.92	8.88	8.94
218021_at	dehydrogenase/re	DHRS4 /// DHRS4L	9.11	8.96	9.00
213094_at	G protein-couplec	GPR126	7.75	7.71	7.96
218732_at	peptidyl-tRNA hyc	PTRH2	10.21	10.22	10.20
214551_s_at	CD7 molecule	CD7	5.15	4.80	4.79
203522_at	copper chaperone	CCS	8.22	8.30	8.11
202076_at	baculoviral IAP re	BIRC2	11.73	11.73	11.86
208712_at	cyclin D1	CCND1	9.89	9.86	9.95
203228_at	platelet-activating	PAFAH1B3	8.89	8.79	8.76
201332_s_at	signal transducer	STAT6	6.09	6.15	6.27
220694_at	ASAP1 intronic tra	ASAP1IT1	6.36	6.53	6.20
216366_x_at	---	---	7.81	7.59	7.57
201393_s_at	insulin-like growtl	IGF2R	9.87	9.75	9.82
209088_s_at	ubinuclein 1	UBN1	10.15	10.12	10.08
211121_s_at	docking protein 1,	DOK1	5.95	5.82	5.83
203159_at	glutaminase	GLS	8.46	8.43	8.63
210448_s_at	purinergic recepto	P2RX5	8.29	8.33	8.31
208308_s_at	glucose-6-phosph	GPI	10.85	10.74	10.87
203058_s_at	3'-phosphoadeno:	PAPSS2	4.54	4.35	4.28

213559_s_at	Zinc finger proteir	ZNF467	6.05	6.02	5.75
205091_x_at	RecQ protein-like	RECQL	8.45	8.70	8.74
203769_s_at	steroid sulfatase (	STS	4.22	4.40	4.26
201560_at	chloride intracellu	CLIC4	12.66	12.61	12.76
208249_s_at	TDP-glucose 4,6-d	TGDS	8.93	8.88	8.89
219356_s_at	chromatin modify	CHMP5	11.02	11.02	11.07
218961_s_at	polynucleotide kir	PNKP	7.27	7.21	7.33
213995_at	ATP synthase, H+	ATP5S	7.66	7.73	7.59
216308_x_at	glyoxylate reducta	GRHPR	9.61	9.59	9.47
207233_s_at	microphthalmia-a	MITF	8.42	8.30	8.43
208792_s_at	clusterin	CLU	9.50	9.32	9.57
202893_at	cell division cycle	CDC23	8.16	8.32	8.16
200763_s_at	ribosomal protein	RPLP1	15.00	15.00	15.05
200022_at	ribosomal protein	RPL18	14.51	14.45	14.50
204980_at	clock homolog (m	CLOCK	7.84	7.87	7.84
203753_at	transcription facto	TCF4	8.77	8.93	8.94
210559_s_at	cyclin-dependent	CDK1	11.27	11.32	11.31
211612_s_at	interleukin 13 rec	IL13RA1	7.17	7.27	7.33
218607_s_at	SDA1 domain con	SDAD1	7.81	7.85	7.82
215706_x_at	zyxin	ZYX	7.13	7.18	7.16
219002_at	FAST kinase doma	FASTKD1	9.88	10.01	9.91
200635_s_at	protein tyrosine p	PTPRF	9.13	9.06	9.22
219630_at	PDZK1 interacting	PDZK1IP1	5.62	5.55	5.36
204497_at	adenylate cyclase	ADCY9	10.21	10.03	10.05
212020_s_at	antigen identified	MKI67	8.40	8.27	8.27
213883_s_at	TM2 domain cont	TM2D1	10.18	10.16	10.37
201377_at	ubiquitin associat	UBAP2L	9.27	9.26	9.08
214743_at	cut-like homeobo	CUX1	9.33	9.29	9.28
209902_at	ataxia telangiecta	ATR	8.88	8.92	9.06
201598_s_at	inositol polyphosp	INPPL1	8.44	8.25	8.33
211024_s_at	NK2 homeobox 1	NKX2-1	5.97	5.93	6.18
203114_at	Sjogren syndrome	SSSCA1	9.61	9.43	9.65
37170_at	BMP2 inducible ki	BMP2K	5.35	5.57	5.59
214900_at	zinc finger with KF	ZKSCAN1	5.33	5.51	5.36
203485_at	reticulon 1	RTN1	9.63	9.70	9.78
204168_at	microsomal glutat	MGST2	8.91	8.87	8.82
219101_x_at	abhydrolase doma	ABHD8	4.95	4.87	4.74
46256_at	splA/ryanodine re	SPSB3	9.86	9.90	9.94
213305_s_at	protein phosphata	PPP2R5C	9.58	9.63	9.80
209366_x_at	cytochrome b5 ty	CYB5A	11.90	11.88	11.89
207700_s_at	nuclear receptor c	NCOA3	9.56	9.62	9.64
222351_at	protein phosphata	PPP2R1B	5.19	5.05	4.93
218344_s_at	REST corepressor	RCOR3	8.02	8.07	8.27
205935_at	forkhead box F1	FOXF1	6.39	6.55	6.43
40612_at	dopey family men	DOPEY1	5.88	5.88	5.70
213624_at	sphingomyelin ph	SMPDL3A	7.45	7.52	7.55
205612_at	multimerin 1	MMRN1	3.70	3.52	3.49

213244_at	secretory carrier r	SCAMP4	6.84	6.94	6.77
204863_s_at	interleukin 6 signa	IL6ST	9.33	9.15	9.29
204500_s_at	ATP/GTP binding	AGTPBP1	7.70	7.66	7.80
208063_s_at	calpain 9	CAPN9	4.09	3.99	3.88
AFFX-HUMISGF3	signal transducer	STAT1	7.13	7.04	6.97
214079_at	dehydrogenase/re	DHRS2	4.51	4.58	4.56
211708_s_at	stearoyl-CoA desa	SCD	8.44	8.57	8.48
201162_at	insulin-like growth	IGFBP7	9.56	9.57	9.52
202739_s_at	phosphorylase kin	PHKB	7.91	8.03	8.13
203616_at	polymerase (DNA	POLB	10.35	10.44	10.38
222291_at	family with seque	FAM149A	3.60	3.53	3.50
212432_at	GrpE-like 1, mitoc	GRPEL1	10.26	10.12	10.32
208991_at	signal transducer	STAT3	10.04	9.97	10.17
219048_at	phosphatidylinosi	PIGN	8.89	8.95	8.96
221213_s_at	zinc finger protein	ZNF280D	7.46	7.52	7.53
202792_s_at	SAPS domain fami	SAPS2	6.44	6.54	6.41
207640_x_at	netrin 3	NTN3	5.68	5.37	5.40
218583_s_at	DCN1, defective in	DCUN1D1	9.15	9.28	9.36
222310_at	splicing factor, arg	SFRS15	6.07	6.33	6.15
214855_s_at	Ral GTPase activat	RALGAPA1	8.23	8.27	8.44
204148_s_at	POM (POM121 ho	POMZP3 /// ZP3	7.16	7.32	7.18
219529_at	chloride intracellu	CLIC3	4.10	4.27	4.25
221733_s_at	G patch domain co	GPATCH4	5.69	5.69	5.81
201142_at	eukaryotic transla	EIF2S1	10.38	10.41	10.43
216411_s_at	galactokinase 2	GALK2	7.42	7.42	7.50
200637_s_at	protein tyrosine p	PTPRF	7.47	7.42	7.42
214581_x_at	tumor necrosis fac	TNFRSF21	6.25	6.12	6.50
201064_s_at	poly(A) binding pr	PABPC4	11.51	11.51	11.58
217654_at	CASP8 and FADD-l	CFLAR	4.04	4.16	4.14
219243_at	GTPase, IMAP fam	GIMAP4	3.53	3.34	3.48
218307_at	radical S-adenosyl	RSAD1	9.80	9.68	9.75
219093_at	phosphotyrosine i	PID1	9.63	9.56	9.78
208319_s_at	RNA binding motif	RBM3	10.79	10.91	11.09
215540_at	T cell receptor alp	TRA@	5.99	5.87	5.91
220568_at	---	---	4.12	4.13	4.13
219498_s_at	B-cell CLL/lympho	BCL11A	4.68	4.67	4.67
216608_at	hypothetical LOC2	LOC26102	6.71	6.66	6.55
221898_at	podoplanin	PDPN	9.75	9.76	9.94
220501_at	actin-like 7A	ACTL7A	6.40	6.43	6.19
204344_s_at	Sec23 homolog A	SEC23A	8.00	8.20	8.12
206542_s_at	SWI/SNF related,	SMARCA2	8.71	8.68	8.87
220223_at	ATPase family, AA	ATAD5	7.54	7.57	7.47
210612_s_at	synaptojanin 2	SYNJ2	9.36	9.33	9.45
215214_at	Immunoglobulin la	IGL@	5.39	5.44	5.17
222225_at	ribosomal protein	RPL23AP53	5.43	5.50	5.37
217047_s_at	family with seque	FAM13A	8.24	8.23	8.37
208659_at	chloride intracellu	CLIC1	12.53	12.60	12.59

214032_at	zeta-chain (TCR) a	ZAP70	4.47	4.38	4.33
211601_at	CATR tumorigenic	CATR1	3.38	3.32	3.28
213300_at	ATG2 autophagy r	ATG2A	9.25	9.17	9.16
208650_s_at	CD24 molecule	CD24	4.48	4.18	4.38
201604_s_at	protein phosphata	PPP1R12A	8.78	9.07	9.03
212531_at	lipocalin 2	LCN2	6.75	6.59	6.45
209509_s_at	dolichyl-phosphat	DPAGT1	9.29	9.25	9.34
206105_at	AF4/FMR2 family,	AFF2	5.78	6.26	5.96
209479_at	coiled-coil domair	CCDC28A	9.14	9.11	9.13
201492_s_at	ribosomal protein	RPL41	15.16	15.18	15.16
221783_at	widely interspace	WIZ	7.37	7.43	7.37
208779_x_at	discoidin domain	DDR1	7.84	7.93	7.97
204671_s_at	ankyrin repeat do	ANKRD6	3.27	3.45	3.28
202574_s_at	casein kinase 1, ga	CSNK1G2	9.04	8.98	8.94
213225_at	protein phosphata	PPM1B	8.80	8.56	8.84
218010_x_at	pancreatic progen	PPDPF	7.17	7.05	7.23
212987_at	F-box protein 9	FBXO9	10.85	10.87	10.91
210127_at	RAB6B, member F	RAB6B	5.63	5.85	5.58
210386_s_at	metaxin 1	MTX1	10.13	10.04	10.15
218310_at	RAB guanine nucle	RABGEF1	8.84	8.95	9.07
201444_s_at	ATPase, H+ transp	ATP6AP2	9.79	9.80	9.90
200649_at	nucleobindin 1	NUCB1	6.64	6.66	6.51
210114_at	inversin	INVS	8.08	8.05	8.09
217079_at	Hypothetical prot	LOC100288679	4.83	4.74	4.89
212986_s_at	tousled-like kinas	TLK2	8.32	8.42	8.32
218889_at	nucleolar comple	NOC3L	10.91	10.86	10.93
213538_at	SON DNA binding	SON	7.80	7.92	7.99
216607_s_at	cytochrome P450,	CYP51A1	9.09	9.19	9.14
36936_at	tissue specific trar	TSTA3	9.03	9.07	8.99
203826_s_at	phosphatidylinosi	PITPNM1	7.27	7.06	7.13
203131_at	platelet-derived g	PDGFRA	6.80	7.08	7.20
206668_s_at	secretory carrier r	SCAMP1	7.99	7.90	8.06
205191_at	retinitis pigmento	RP2	6.86	7.18	7.32
216808_at	microRNA 622	MIR622	4.03	3.92	4.05
202706_s_at	uridine monophos	UMPS	9.41	9.46	9.61
213182_x_at	cyclin-dependent	CDKN1C	6.55	6.67	6.57
206082_at	HLA complex P5	HCP5	4.83	4.42	4.26
218440_at	methylcrotonoyl-(	MCCC1	9.18	9.22	9.30
205994_at	ELK4, ETS-domain	ELK4	4.60	4.49	4.42
213957_s_at	centrosomal prot	CEP350	6.85	6.86	6.76
209465_x_at	pleiotrophin	PTN	7.03	7.09	7.36
213548_s_at	CDV3 homolog (m	CDV3	7.32	7.32	7.21
205677_s_at	deleted in lymphc	DLEU1	9.64	9.46	9.61
206746_at	beaded filament s	BFSP1	4.56	4.37	4.33
208350_at	casein alpha s1	CSN1S1	4.35	4.35	4.37
214507_s_at	exosome compon	EXOSC2	9.03	9.06	9.09
209468_at	low density lipopr	LRP5	6.53	6.44	6.39

203310_at	syntaxin binding p	STXBP3	9.72	9.92	9.95
212633_at	KIAA0776	KIAA0776	9.05	9.13	9.31
221680_s_at	ets variant 7	ETV7	5.81	5.61	5.54
213934_s_at	zinc finger protein	ZNF23	8.30	8.46	8.52
218591_s_at	nucleolar protein	NOL10	5.67	5.59	5.56
200055_at	TAF10 RNA polym	TAF10	10.91	10.90	11.05
205698_s_at	mitogen-activated	MAP2K6	5.44	5.41	5.31
207025_at	gap junction prote	GJC2	7.22	7.02	7.20
209908_s_at	transforming grov	TGFB2	3.46	3.40	3.52
207908_at	keratin 2	KRT2	3.91	4.19	4.13
221983_at	family with seque	FAM134A	9.54	9.55	9.60
220270_at	ring finger protein	RNF17	3.53	3.43	3.38
210423_s_at	solute carrier fam	SLC11A1	5.59	5.52	5.52
201637_s_at	fragile X mental re	FXR1	10.97	11.07	11.10
204112_s_at	histamine N-meth	HNMT	3.78	3.77	3.92
205354_at	guanidinoacetate	GAMT	8.02	8.02	8.08
209057_x_at	CDC5 cell division	CDC5L	8.67	8.75	8.62
211502_s_at	cyclin-dependent	CDK14	5.91	5.92	5.93
217179_x_at	---	---	5.05	5.17	4.99
222303_at	---	---	8.38	8.51	8.41
212240_s_at	phosphoinositide-	PIK3R1	11.52	11.53	11.66
209967_s_at	cAMP responsive	CREM	8.34	8.41	8.47
209336_at	PWP2 periodic try	PWP2	9.44	9.39	9.31
209039_x_at	EH-domain contai	EHD1	7.94	8.05	8.03
219098_at	MYB binding prote	MYBBP1A	8.20	8.22	8.41
222091_at	hippocalcin like 4	HPCAL4	6.42	6.49	6.53
212253_x_at	dystonin	DST	7.74	7.94	7.98
201283_s_at	trafficking protein	TRAK1	5.07	5.07	5.05
202637_s_at	intercellular adhe	ICAM1	8.61	8.36	8.61
202164_s_at	CCR4-NOT transcr	CNOT8	9.13	9.15	9.13
214668_at	chromosome 13 o	C13orf1	5.48	5.85	5.80
215658_at	---	---	4.55	4.45	4.47
203242_s_at	PDZ and LIM dom	PDLIM5	6.83	6.93	6.85
214948_s_at	TATA element mo	TMF1	10.55	10.60	10.60
213081_at	zinc finger and BT	ZBTB22	7.16	7.17	7.01
201505_at	laminin, beta 1	LAMB1	8.91	8.94	9.05
203311_s_at	ADP-ribosylation f	ARF6	9.56	9.49	9.72
208473_s_at	glycoprotein 2 (zy	GP2	4.94	5.05	4.96
214657_s_at	nuclear paraspeck	NEAT1	7.33	7.52	7.40
205876_at	leukemia inhibitor	LIFR	6.96	6.98	7.02
213443_at	TNFRSF1A-associa	TRADD	3.95	3.90	3.87
201674_s_at	A kinase (PRKA) ar	AKAP1	10.28	10.11	10.16
218892_at	dachsous 1 (Drosc	DCHS1	5.31	5.21	5.23
209297_at	intersectin 1 (SH3	ITSN1	9.23	9.29	9.27
205602_x_at	pregnancy specific	PSG7	8.14	8.22	8.05
201245_s_at	OTU domain, ubic	OTUB1	9.21	9.25	9.40
205324_s_at	FtsJ homolog 1 (E.	FTSJ1	10.17	10.13	10.30

207501_s_at	fibroblast growth	FGF12	4.48	4.38	4.48
208159_x_at	DEAD/H (Asp-Glu-	DDX11	8.71	8.82	8.74
212197_x_at	myosin phosphata	MPRIP	10.62	10.55	10.62
202041_s_at	fibroblast growth	FIBP	10.35	10.27	10.37
217808_s_at	mitogen-activatec	MAPKAP1	8.71	8.69	8.63
210744_s_at	interleukin 5 rece	IL5RA	5.23	5.21	5.00
213586_at	hypothetical prot	LOC100287600	5.27	5.05	5.10
208921_s_at	sorcin	SRI	11.94	11.86	11.94
202925_s_at	pleiomorphic ade	PLAGL2	7.06	7.20	7.31
216738_at	---	---	4.85	4.91	4.69
218071_s_at	makorin ring finge	MKRN2	9.80	9.70	9.81
221290_s_at	melanoma associa	MUM1	6.56	6.82	6.58
202798_at	SEC24 family, mer	SEC24B	10.23	10.10	10.21
35974_at	lymphoid-restricte	LRMP	3.73	3.72	3.88
212468_at	sperm associated	SPAG9	8.45	8.48	8.51
216167_at	leucine rich repea	LRRN2	3.62	3.67	3.62
210625_s_at	A kinase (PRKA) a	AKAP1	7.58	7.56	7.79
210697_at	zinc finger protein	ZNF257	3.03	3.11	3.24
207916_at	---	---	4.30	4.31	4.13
213199_at	C2 calcium-depen	C2CD3	7.52	7.63	7.67
216497_at	heterogeneous nu	HNRNPA1 /// HNR	5.83	6.03	5.98
211999_at	H3 histone, family	H3F3B	12.27	12.33	12.35
218104_at	testis expressed 1	TEX10	10.29	10.22	10.19
204069_at	Meis homeobox 1	MEIS1	8.35	8.26	8.34
210971_s_at	aryl hydrocarbon	ARNTL	7.76	7.72	7.93
215696_s_at	SEC16 homolog A	SEC16A	9.20	9.27	9.24
211556_at	microtubule-asso	MAPRE2	3.34	3.44	3.60
218929_at	CDKN2A interactir	CDKN2AIP	7.94	8.06	8.10
204099_at	SWI/SNF related,	SMARCD3	7.09	7.11	7.19
214864_s_at	glyoxylate reducta	GRHPR	10.12	10.03	9.98
218638_s_at	spondin 2, extrace	SPON2	7.57	7.84	7.52
202100_at	v-ral simian leuke	RALB	9.11	9.27	9.31
210847_x_at	tumor necrosis fa	TNFRSF25	5.62	5.40	5.52
219019_at	leucine-rich repea	LRDD	7.40	7.48	7.47
208117_s_at	LAS1-like (S. cerev	LAS1L	9.59	9.54	9.50
210720_s_at	N-terminal EF-har	NECAB3	7.27	7.39	7.12
202784_s_at	nicotinamide nucl	NNT	9.50	9.52	9.64
205841_at	Janus kinase 2	JAK2	5.10	4.82	5.20
210570_x_at	mitogen-activatec	MAPK9	8.38	8.36	8.42
219663_s_at	transmembrane p	TMEM121	5.89	6.00	5.98
200793_s_at	aconitase 2, mito	ACO2	8.63	8.62	8.54
212838_at	dynamin binding p	DNMBP	8.05	8.16	8.20
208758_at	5-aminoimidazole	ATIC	11.89	11.79	11.83
209256_s_at	PNAS-119	LOC100287552	9.00	9.09	9.13
221917_s_at	G-rich RNA seque	GRSF1	5.75	5.93	5.83
210747_at	major histocompa	HLA-DQB1	3.64	3.60	3.41
202384_s_at	Treacher Collins-F	TCOF1	8.53	8.45	8.58

220485_s_at	signal-regulatory p	SIRPG	4.11	4.22	4.06
216507_at	transmembrane a	TAPT1	4.25	4.57	4.40
209592_s_at	DDB1 and CUL4 as	DCAF7	7.83	7.79	7.77
218751_s_at	F-box and WD rep	FBXW7	7.72	7.48	7.40
216026_s_at	polymerase (DNA	POLE	7.76	7.80	7.63
214750_at	placenta-specific 4	PLAC4	5.74	6.03	5.78
210714_at	R3H domain conta	R3HDM1	5.08	4.80	4.74
212577_at	structural mainter	SMCHD1	8.30	8.44	8.38
201182_s_at	chromodomain he	CHD4	9.72	9.68	9.70
203739_at	zinc finger proteir	ZNF217	9.68	9.75	10.02
219367_s_at	---	---	6.38	6.45	6.10
213034_at	SIK family kinase 3	SIK3	8.35	8.13	8.31
219055_at	S1 RNA binding do	SRBD1	8.87	8.87	8.95
201480_s_at	suppressor of Ty 5	SUPT5H	8.58	8.68	8.58
218692_at	syntabulin (syntax	SYBU	7.68	7.29	7.80
211205_x_at	phosphatidylinosi	PIP5K1A	5.86	5.73	5.77
220365_at	allantoicase	ALLC	5.16	5.25	5.03
203625_x_at	S-phase kinase-as	SKP2	10.63	10.84	10.71
217641_at	G protein-couplec	GPR135	5.40	5.28	5.36
203594_at	RNA terminal pho	RTCD1	10.25	10.32	10.36
202857_at	solute carrier fam	SLC16A3	11.24	11.26	11.25
216063_at	hemoglobin, beta	HBBP1	4.87	4.71	4.77
218897_at	transmembrane p	TMEM177	8.76	8.88	8.94
221732_at	calcium activated	CANT1	9.37	9.36	9.29
218182_s_at	claudin 1	CLDN1	5.17	5.15	4.91
202212_at	pescadillo homolo	PES1	9.08	9.03	9.01
209502_s_at	BAI1-associated p	BAIAP2	6.39	6.61	6.39
220030_at	serine/threonine/	STYK1	3.37	3.38	3.32
212551_at	CAP, adenylate cy	CAP2	8.66	8.52	8.55
45572_s_at	golgi-associated, g	GGA1	9.53	9.64	9.50
203105_s_at	dynamin 1-like	DNM1L	9.67	9.75	9.72
208310_s_at	chromosome 7 op	C7orf28A /// C7or	9.73	9.84	9.88
207505_at	protein kinase, cG	PRKG2	4.61	4.72	4.54
211623_s_at	fibrillarin	FBL	12.16	12.11	12.15
221673_s_at	casein kinase 1, ga	CSNK1G1	6.54	6.39	6.37
217902_s_at	hect domain and I	HERC2	9.22	9.21	9.27
217893_s_at	akirin 1	AKIRIN1	9.29	9.25	9.32
214602_at	collagen, type IV,	COL4A4	4.03	3.84	4.04
218543_s_at	poly (ADP-ribose)	PARP12	9.46	9.37	9.30
214066_x_at	natriuretic peptid	NPR2	4.02	3.99	4.07
201367_s_at	zinc finger proteir	ZFP36L2	4.90	5.06	4.97
218711_s_at	serum deprivatior	SDPR	7.66	7.66	7.70
203775_at	solute carrier fam	SLC25A13	8.67	8.71	8.89
214150_x_at	ATPase, H+ transp	ATP6V0E1	11.02	11.01	11.06
202794_at	inositol polyphosp	INPP1	9.55	9.45	9.66
202506_at	sperm specific ant	SSFA2	10.21	10.30	10.46
201477_s_at	ribonucleotide red	RRM1	12.00	12.06	12.02

212090_at	glutamate recepto	GRINA	8.06	7.87	7.90
220051_at	protease, serine, 2	PRSS21	5.39	5.12	4.95
203354_s_at	pleckstrin and Sec	PSD3	6.86	7.04	7.02
205462_s_at	hippocalcin-like 1	HPCAL1	8.85	8.84	8.91
219997_s_at	COP9 constitutive	COPS7B	7.77	7.75	7.72
212795_at	KIAA1033	KIAA1033	9.56	9.73	9.82
208126_s_at	cytochrome P450,	CYP2C18	4.21	4.17	4.15
210879_s_at	RAB11 family inte	RAB11FIP5	8.76	8.73	8.81
213662_at	similar to hCG198	LOC100288944	3.37	3.38	3.47
201811_x_at	SH3-domain bindi	SH3BP5	8.83	8.67	8.80
203646_at	ferredoxin 1	FDX1	8.26	8.30	8.32
205743_at	SH3 and cysteine	STAC	9.25	9.04	9.15
209210_s_at	fermitin family ho	FERMT2	11.61	11.67	11.77
204756_at	mitogen-activatec	MAP2K5	8.16	8.10	8.40
202738_s_at	phosphorylase kir	PHKB	8.75	8.88	8.93
202816_s_at	hexamethylene bi	HEXIM1	8.57	8.68	8.76
205882_x_at	adducin 3 (gamma	ADD3	6.82	7.02	7.01
207009_at	paired-like homec	PHOX2B	5.27	5.07	4.97
204946_s_at	topoisomerase (D	TOP3A	6.39	6.22	6.22
209974_s_at	budding uninhibit	BUB3	12.32	12.38	12.50
208864_s_at	thioredoxin	TXN	12.96	13.04	13.12
221484_at	UDP-Gal:betaGlcN	B4GALT5	9.95	9.91	9.97
216238_s_at	fibrinogen beta ch	FGB	3.70	3.77	3.89
201472_at	von Hippel-Lindau	VBP1	11.80	11.81	11.82
204334_at	Kruppel-like facto	KLF7	8.91	8.79	8.97
212215_at	prolyl endopeptid	PREPL	10.20	10.32	10.36
209177_at	NADH dehydrogen	NDUFAF3	9.71	9.61	9.71
220749_at	chromosome 10 c	C10orf68	5.73	5.53	5.48
205859_at	lymphocyte antige	LY86	4.97	4.85	4.73
209113_s_at	high-mobility grou	HMG20B	7.28	7.47	7.40
206893_at	sal-like 1 (Drosoph	SALL1	3.27	3.23	3.23
201892_s_at	IMP (inosine 5'-m	IMPDH2	13.08	13.01	13.04
209678_s_at	protein kinase C, i	PRKCI	10.69	10.77	10.74
207857_at	leukocyte immun	LILRA2	4.39	4.49	4.30
213621_s_at	Guanylate kinase	GUK1	6.10	5.84	5.99
203645_s_at	CD163 molecule	CD163	5.19	5.12	5.08
220783_at	matrix metallope	MMP27	4.77	4.66	4.71
215653_at	---	---	3.24	3.22	3.10
209076_s_at	WDR45-like	WDR45L	10.71	10.67	10.68
205319_at	prostate stem cell	PSCA	5.81	5.87	5.71
204315_s_at	G-2 and S-phase e	GTSE1	9.20	9.26	9.14
218256_s_at	nucleoporin 54kD	NUP54	10.27	10.29	10.30
201735_s_at	chloride channel 3	CLCN3	9.03	9.11	9.15
209966_x_at	estrogen-related r	ESRRG	4.03	4.01	4.00
209417_s_at	interferon-induce	IFI35	8.35	8.23	8.38
218483_s_at	intraflagellar trans	IFT46	9.17	9.11	9.20
207068_at	zinc finger protein	ZFP37	6.51	6.57	6.61

221377_s_at	recombination sig	RBPJL	5.92	5.94	5.94
208842_s_at	golgi reassembly s	GORASP2	9.89	9.93	9.95
210270_at	regulator of G-prc	RGS6	4.70	4.82	4.72
209834_at	carbohydrate (chc	CHST3	8.19	8.27	8.37
217741_s_at	zinc finger, AN1-ty	ZFAND5	8.83	8.88	8.85
206371_at	folate receptor 3 (	FOLR3	5.65	5.49	5.30
206098_at	zinc finger and BT	ZBTB6	4.39	4.12	4.18
201022_s_at	destrin (actin dep	DSTN	13.06	13.12	13.19
210112_at	Hermansky-Pudla	HPS1	8.36	8.24	8.32
200671_s_at	spectrin, beta, no	SPTBN1	7.66	7.74	7.96
206724_at	chromobox homo	CBX4	4.44	4.42	4.38
213037_x_at	staufen, RNA bind	STAU1	11.56	11.56	11.47
201357_s_at	splicing factor 3a,	SF3A1	7.52	7.54	7.49
209556_at	neurochondrin	NCDN	7.27	7.12	7.07
206048_at	ovo-like 2 (Drosop	OVOL2	3.38	3.30	3.35
214141_x_at	splicing factor, arg	SFRS7	12.60	12.66	12.64
210447_at	glutamate dehydr	GLUD2	3.51	3.69	3.43
211627_x_at	estrogen receptor	ESR1	3.49	3.31	3.32
204918_s_at	myeloid/lymphoic	MLLT3	3.39	3.41	3.34
202462_s_at	DEAD (Asp-Glu-Al	DDX46	11.21	11.34	11.29
209624_s_at	methylcrotonoyl-(	MCCC2	8.21	8.20	8.07
212839_s_at	TROVE domain fai	TROVE2	4.64	4.97	4.94
220532_s_at	transmembrane p	TMEM176B	4.48	4.31	4.44
208100_x_at	sema domain, trai	SEMA6C	7.31	7.14	7.12
205490_x_at	gap junction prote	GJB3	6.40	6.33	5.96
204247_s_at	cyclin-dependent	CDK5	8.33	8.27	8.32
213302_at	phosphoribosylfoi	PFAS	9.43	9.58	9.60
208058_s_at	mannosyl (beta-1,	MGAT3	6.96	6.83	6.89
203377_s_at	cell division cycle	CDC40	6.52	6.38	6.57
220414_at	calmodulin-like 5	CALML5	5.18	5.17	5.02
203600_s_at	family with seque	FAM193A	7.89	7.81	7.89
210276_s_at	nucleolar protein	NOL12 /// TRIOBP	8.78	8.68	8.65
203204_s_at	lysine (K)-specific	KDM4A	9.17	9.06	9.07
207092_at	leptin	LEP	3.99	3.84	3.59
216129_at	ATPase, class II, ty	ATP9A	5.84	5.53	5.68
200747_s_at	nuclear mitotic ap	NUMA1	9.13	9.12	9.16
212912_at	ribosomal protein	RPS6KA2	8.77	8.79	9.00
219576_at	MAP7 domain cor	MAP7D3	8.79	8.80	8.84
210676_x_at	RANBP2-like and (	RGPD5 /// RGPD6	7.42	7.48	7.64
208501_at	growth factor ind	GFI1B	3.93	4.00	4.08
217215_s_at	---	---	3.66	3.65	3.51
200783_s_at	stathmin 1	STMN1	10.96	11.01	11.05
207161_at	KIAA0087	KIAA0087	3.36	3.36	3.45
214687_x_at	aldolase A, fructo	ALDOA	13.20	13.11	13.14
200639_s_at	tyrosine 3-monoo	YWHAZ	11.85	11.92	12.04
205881_at	zinc finger proteir	ZNF74	5.79	5.60	5.70
211340_s_at	melanoma cell ad	MCAM	8.99	9.05	9.03

216011_at	Solute carrier fam	SLC39A9	4.79	4.87	4.79
201544_x_at	poly(A) binding pr	PABPN1	11.62	11.71	11.70
201391_at	TNF receptor-assc	TRAP1	10.85	10.68	10.77
211423_s_at	sterol-C5-desatur	SC5DL	9.96	10.24	10.16
201324_at	epithelial membra	EMP1	11.28	11.41	11.67
222364_at	solute carrier fam	SLC44A1	6.53	6.75	6.61
209426_s_at	alpha-methylacyl-	AMACR /// C1QTN	7.67	7.68	7.77
211794_at	FYN binding prote	FYB	4.62	4.56	4.60
211749_s_at	vesicle-associated	VAMP3	9.06	9.19	9.21
205784_x_at	armadillo repeat g	ARVCF	7.76	7.76	7.61
200638_s_at	tyrosine 3-monoo	YWHAZ	12.12	12.23	12.26
221074_at	Natural cytotoxici	NCR2	6.15	6.08	5.94
215063_x_at	leucine rich repea	LRRC40	8.83	8.92	8.86
219799_s_at	dehydrogenase/re	DHRS9	7.46	7.59	7.53
206897_at	P antigen family, r	PAGE1	4.00	3.88	3.93
208914_at	golgi-associated, g	GGA2	6.48	6.38	5.96
221937_at	synergin, gamma	SYNRG	8.82	8.91	8.81
210823_s_at	protein tyrosine p	PTPRS	5.73	5.80	5.57
41469_at	peptidase inhibito	PI3	4.87	4.84	4.35
204906_at	ribosomal protein	RPS6KA2	8.21	8.04	8.08
212450_at	SECIS binding prot	SECISBP2L	7.57	7.44	7.70
221834_at	Lon peptidase 2, p	LONP2	7.23	7.40	7.39
203006_at	inositol polyphosp	INPP5A	9.34	9.33	9.34
220753_s_at	crystallin, lambda	CRYL1	7.89	7.76	7.77
202043_s_at	spermine synthas	SMS	10.40	10.48	10.66
202696_at	oxidative-stress re	OXSR1	10.96	11.02	11.04
208081_s_at	zinc finger protein	ZNF442	3.83	3.85	3.73
215155_at	Hexosaminidase A	HEXA	6.02	6.14	6.18
221760_at	Mannosidase, alpl	MAN1A1	7.85	8.13	7.92
208540_x_at	S100 calcium bind	S100A11	8.62	8.59	8.43
201160_s_at	cold shock domair	CSDA	12.92	12.82	13.03
220936_s_at	H2A histone famil	H2AFJ	6.13	6.22	6.39
52164_at	chromosome 11 c	C11orf24	10.07	10.10	10.10
220558_x_at	tetraspanin 32	TSPAN32	7.36	7.53	7.40
211399_at	fibroblast growth	FGFR2	5.23	5.29	5.10
202181_at	KIAA0247	KIAA0247	9.71	9.38	9.73
208097_s_at	thioredoxin-relate	TMX1	9.00	8.98	9.09
204259_at	matrix metallopep	MMP7	6.05	5.83	6.09
218305_at	importin 4	IPO4	8.75	8.75	8.98
209471_s_at	farnesyltransferas	FNTA	10.46	10.51	10.59
208170_s_at	tripartite motif-co	TRIM31	4.24	4.24	4.14
216107_at	hypothetical prote	LOC100129503	5.39	5.56	5.47
201939_at	polo-like kinase 2	PLK2	8.44	8.73	8.71
213063_at	zinc finger CCCH-t	ZC3H14	8.60	8.56	8.50
218633_x_at	abhydrolase doma	ABHD10	9.85	9.76	9.87
202544_at	glia maturation fa	GMFB	11.83	11.91	11.91
204552_at	inositol polyphosp	INPP4A	5.96	5.86	5.89

202875_s_at	ATPase, H+ transp	ATP6V1C1	8.00	7.85	7.95
208668_x_at	high-mobility grou	HMGN2	13.87	13.87	13.84
215054_at	erythropoietin rec	EPOR	8.38	8.35	8.25
222060_at	keratin 8 pseudog	KRT8P12	6.70	6.61	6.73
214203_s_at	proline dehydroge	PRODH	6.91	6.72	6.62
203726_s_at	laminin, alpha 3	LAMA3	7.49	7.53	7.65
218296_x_at	misato homolog 1	MSTO1 /// MSTO2	8.05	8.03	8.02
201603_at	protein phosphata	PPP1R12A	9.70	9.93	10.03
218114_at	golgi-associated, g	GGA1	7.68	7.69	7.71
202748_at	guanylate binding	GBP2	7.67	7.60	7.79
212627_s_at	exosome compon	EXOSC7	10.72	10.64	10.69
207668_x_at	protein disulfide i:	PDIA6	12.31	12.37	12.29
214814_at	YTH domain conta	YTHDC1	6.06	6.06	6.12
210923_at	solute carrier fam	SLC1A7	4.24	4.37	4.32
221558_s_at	lymphoid enhance	LEF1	8.98	9.04	8.96
215348_at	adenylate cyclase	ADCY1	5.70	5.41	5.54
202436_s_at	cytochrome P450,	CYP1B1	4.72	5.11	4.92
205559_s_at	proprotein conve	PCSK5	3.69	3.67	3.57
203426_s_at	insulin-like growth	IGFBP5	6.43	6.10	6.22
220639_at	transmembrane 4	TM4SF20	3.88	3.80	3.81
48808_at	dihydrofolate red	DHFR	10.36	10.45	10.46
220569_at	---	---	3.73	3.85	3.72
212732_at	maternally expres	MEG3	4.59	4.53	4.67
215739_s_at	tubulin, gamma co	TUBGCP3	6.38	6.29	6.74
212766_s_at	interferon stimula	ISG20L2	9.39	9.46	9.42
219772_s_at	small muscle prot	SMPX	4.12	4.06	3.94
216636_at	---	---	3.45	3.47	3.39
210427_x_at	annexin A2	ANXA2	14.40	14.42	14.39
201970_s_at	nuclear autoantig	NASP	10.38	10.48	10.45
212816_s_at	cystathionine-beta	CBS	7.23	7.08	7.18
208765_s_at	heterogeneous nu	HNRNPR	11.43	11.52	11.51
216837_at	EPH receptor A5	EPHA5	5.00	5.03	4.88
202560_s_at	chromosome 1 op	C1orf77	10.29	10.17	10.27
215664_s_at	EPH receptor A5	EPHA5	4.05	3.81	3.80
218565_at	chromosome 9 op	C9orf114	6.18	6.31	6.36
204220_at	glia maturation fa	GMFG	6.18	6.41	6.36
203633_at	carnitine palmitoy	CPT1A	8.01	8.05	7.99
201295_s_at	WD repeat and SC	WSB1	4.41	5.01	4.62
217539_at	chromosome 18 c	C18orf25	4.60	4.47	4.68
37012_at	capping protein (a	CAPZB	10.70	10.75	10.82
205133_s_at	heat shock 10kDa	HSPE1	11.93	12.01	11.97
214297_at	chondroitin sulfat	CSPG4	6.56	6.59	6.61
221833_at	Lon peptidase 2, p	LONP2	6.77	7.03	6.83
204944_at	protein tyrosine p	PTPRG	8.29	8.37	8.39
208445_s_at	bromodomain adj	BAZ1B	9.76	9.73	9.74
201346_at	adiponectin recep	ADIPOR2	10.22	10.18	10.31
208521_at	olfactory receptor	OR5I1	4.14	4.03	4.18

219922_s_at	latent transformir	LTBP3	7.11	7.02	7.33
204165_at	WAS protein fami	WASF1	9.71	9.68	9.88
221565_s_at	calcium homeosta	CALHM2	5.49	5.44	5.31
207950_s_at	ankyrin 3, node of	ANK3	5.25	5.53	5.49
206106_at	mitogen-activatec	MAPK12	8.12	8.02	8.10
216681_at	---	---	3.62	3.62	3.73
210623_at	UBX domain prote	UBXN1	6.30	6.06	6.16
204381_at	low density lipopr	LRP3	8.26	8.15	8.19
221521_s_at	GINS complex sub	GINS2	9.19	9.25	9.36
208751_at	N-ethylmaleimide	NAPA	5.42	5.38	5.33
204214_s_at	RAB32, member F	RAB32	11.31	11.19	11.27
218214_at	chromosome 12 c	C12orf44	9.28	9.23	9.28
220998_s_at	unc-93 homolog E	UNC93B1	7.30	7.26	7.13
219900_s_at	zinc finger proteir	ZNF446	7.64	7.48	7.61
211993_at	WNK lysine deficie	WNK1	8.39	8.16	8.14
219179_at	dapper, antagonis	DACT1	3.66	3.65	3.75
213253_at	structural mainter	SMC2	8.08	8.18	8.21
217896_s_at	family with seque	FAM192A	8.94	9.07	8.97
205551_at	synaptic vesicle gl	SV2B	4.59	4.35	4.51
219621_at	claspin homolog (	CLSPN	4.44	4.43	4.40
202778_s_at	zinc finger, MYM-	ZMYM2	8.77	8.83	8.86
211915_s_at	tubulin, beta poly	TUBB4Q	5.76	5.37	5.57
211800_s_at	ubiquitin specific	USP4	9.54	9.56	9.66
210090_at	activity-regulated	ARC	5.46	5.28	5.72
201222_s_at	RAD23 homolog E	RAD23B	11.42	11.42	11.51
221493_at	TSPY-like 1	TSPYL1	11.22	11.16	11.22
212864_at	CDP-diacylglycero	CDS2	7.68	7.72	7.62
200662_s_at	translocase of out	TOMM20	12.55	12.54	12.53
219300_s_at	contactin associat	CNTNAP2	6.06	6.31	6.02
205639_at	acyloxyacyl hydro	AOAH	6.05	6.08	6.20
209626_s_at	oxysterol binding	OSBPL3	8.35	8.37	8.44
210168_at	complement com	C6	4.51	4.38	4.68
207798_s_at	ataxin 2-like	ATXN2L	5.86	5.80	5.75
200696_s_at	gelsolin	GSN	8.62	8.72	8.78
202301_s_at	arginine/serine-ric	RSRC2	10.54	10.45	10.42
202057_at	karyopherin alpha	KPNA1	7.35	7.31	7.30
218954_s_at	BRF2, subunit of F	BRF2	6.91	6.76	6.99
213838_at	nucleolar protein	NOL7	9.24	9.27	9.19
219161_s_at	chemokine-like fa	CKLF	10.55	10.66	10.66
219110_at	GAR1 ribonucleop	GAR1	10.62	10.47	10.57
205620_at	coagulation factor	F10	5.54	5.68	5.23
217236_x_at	immunoglobulin h	IGH@ /// IGHA1 //	4.41	4.20	4.16
202354_s_at	general transcript	GTF2F1	8.68	8.59	8.53
204803_s_at	Ras-related associ	RRAD	5.92	5.79	6.04
204775_at	chromatin assemb	CHAF1B	7.43	7.51	7.43
203117_s_at	PAN2 poly(A) spec	PAN2	8.46	8.49	8.43
210218_s_at	SP100 nuclear ant	SP100	7.51	7.68	7.55

51774_s_at	hypothetical prote	LOC222070	8.32	8.50	8.39
32099_at	carbohydrate (chc	CHST3	9.05	9.08	8.97
218890_x_at	mitochondrial ribc	MRPL35	11.11	11.20	11.24
217721_at	---	---	7.36	7.57	7.52
201067_at	proteasome (pros	PSMC2	8.44	8.68	8.57
217301_x_at	retinoblastoma bi	RBBP4	10.13	10.09	10.02
206562_s_at	casein kinase 1, al	CSNK1A1	10.53	10.50	10.62
203080_s_at	bromodomain adj	BAZ2B	8.32	8.52	8.40
210156_s_at	protein-L-isoaspar	PCMT1	11.29	11.35	11.31
49329_at	kelch-like 22 (Dros	KLHL22	7.30	7.40	7.29
214428_x_at	complement com	C4A /// C4B /// LO	7.63	7.48	7.38
205445_at	prolactin	PRL	4.28	4.53	4.58
203551_s_at	COX11 cytochrom	COX11	8.65	8.72	8.60
203093_s_at	translocase of inn	TIMM44	9.01	8.95	9.00
218400_at	2'-5'-oligoadenyla	OAS3	7.43	7.49	7.51
219108_x_at	DEAD (Asp-Glu-Ala	DDX27 /// SS18	9.67	9.66	9.70
208204_s_at	caveolin 3	CAV3	6.85	6.93	6.90
219200_at	FAST kinase doma	FASTKD3	10.15	10.01	10.13
200901_s_at	mannose-6-phosp	M6PR	9.85	9.86	9.86
214997_at	suppressor of can	SCAI	4.36	4.21	4.22
215714_s_at	SWI/SNF related,	SMARCA4	9.25	9.17	9.22
209271_at	bromodomain PH	BPTF	8.62	8.70	8.74
203115_at	ferrochelatase	FECH	7.50	7.40	7.58
206306_at	ryanodine recepto	RYR3	3.19	3.12	3.15
200974_at	actin, alpha 2, smo	ACTA2	8.04	7.96	8.09
203650_at	protein C receptor	PROCR	10.43	10.32	10.52
212030_at	RNA binding motif	RBM25	9.57	9.64	9.74
211813_x_at	decorin	DCN	8.05	8.15	8.25
217685_at	Solute carrier fam	SLC16A3	4.96	4.74	4.76
218765_at	SID1 transmembra	SIDT2	7.71	7.64	7.55
206156_at	gap junction prote	GJB5	4.95	4.70	4.55
218133_s_at	NIF3 NGG1 interac	NIF3L1	9.80	9.91	9.91
201582_at	Sec23 homolog B	SEC23B	7.58	7.83	7.70
200698_at	KDEL (Lys-Asp-Glu	KDELR2	11.55	11.64	11.77
212054_x_at	TBC1 domain fam	TBC1D9B	7.49	7.66	7.59
221530_s_at	basic helix-loop-he	BHLHE41	7.57	7.12	7.64
208014_x_at	---	---	5.55	5.71	5.62
215268_at	KIAA0754	KIAA0754	4.96	5.00	4.97
209052_s_at	Wolf-Hirschhorn s	WHSC1	7.20	7.21	7.14
202319_at	SUMO1/sentrin sp	SENP6	7.48	7.58	7.65
204300_at	PET112-like (yeast	PET112L	8.52	8.48	8.37
201927_s_at	plakophilin 4	PKP4	9.13	9.02	8.92
203177_x_at	transcription facto	TFAM	9.98	10.07	10.16
214330_at	ATP synthase mito	ATPAF2	4.65	4.92	4.87
208876_s_at	p21 protein (Cdc4	PAK2	5.60	5.60	5.55
212685_s_at	transducin (beta)-	TBL2	9.69	9.71	9.77
204405_x_at	DIM1 dimethylade	DIMT1L	11.22	11.26	11.38

214843_s_at	ubiquitin specific	USP33	8.74	8.84	8.77
219251_s_at	WD repeat domai	WDR60	7.95	7.98	7.97
203193_at	estrogen-related r	ESRRA	8.24	8.06	8.12
215326_at	p21 protein (Cdc4	PAK4	5.32	5.47	5.29
221964_at	tubby like protein	TULP3	7.36	7.30	7.31
220296_at	hypothetical LOC3	FLJ38109	7.74	7.55	7.72
205442_at	microfibrillar-asso	MFAP3L	5.65	5.90	5.92
218024_at	brain protein 44-li	BRP44L	10.58	10.66	10.78
202605_at	glucuronidase, be	GUSB	9.73	9.64	9.67
217719_at	eukaryotic transla	EIF3L	13.58	13.58	13.70
202503_s_at	KIAA0101	KIAA0101	13.27	13.30	13.34
201007_at	hydroxyacyl-CoA (	HADHB	11.48	11.43	11.42
47069_at	proline rich 5 (ren	PRR5	6.53	6.74	6.44
221397_at	taste receptor, ty	TAS2R10	3.46	3.43	3.33
203280_at	scaffold attachme	SAFB2	7.85	7.87	7.73
204920_at	carbamoyl-phospl	CPS1	9.28	9.13	9.25
217472_at	---	---	6.33	6.42	6.32
207430_s_at	microseminoprote	MSMB	3.40	3.37	3.24
216703_at	---	---	3.30	3.00	3.05
218563_at	NADH dehydroger	NDUFA3	10.64	10.60	10.62
217229_at	ankyrin repeat an	ASB4	3.12	2.99	3.12
202275_at	glucose-6-phosph	G6PD	7.61	7.59	7.57
202129_s_at	RIO kinase 3 (yeas	RIOK3	7.90	7.86	7.94
211072_x_at	tubulin, alpha 1b	TUBA1B	14.51	14.54	14.50
201354_s_at	bromodomain adj	BAZ2A	8.35	8.32	8.19
209135_at	aspartate beta-hy	ASPH	11.48	11.53	11.67
219182_at	transmembrane p	TMEM231	8.10	8.03	8.08
207480_s_at	Meis homeobox 2	MEIS2	9.23	9.36	9.35
205076_s_at	myotubularin rela	MTMR11	6.01	6.15	6.30
211574_s_at	CD46 molecule, co	CD46	7.65	7.79	7.68
219757_s_at	chromosome 14 o	C14orf101	7.48	7.73	7.65
220741_s_at	pyrophosphatase	PPA2	10.88	10.84	10.90
212386_at	transcription facto	TCF4	9.51	9.52	9.52
204399_s_at	echinoderm micro	EML2	6.80	6.56	6.58
208231_at	neuregulin 1	NRG1	4.50	4.18	4.17
202213_s_at	cullin 4B	CUL4B	7.84	8.08	8.03
214425_at	Alpha-1-microglol	AMBP	6.06	5.76	5.79
207751_at	---	---	3.74	3.74	3.63
202631_s_at	amyloid beta prec	APPBP2	7.38	7.66	7.65
215776_at	insulin receptor-re	INSRR	5.97	5.90	5.77
217670_at	Ribosomal proteir	RPLP2	4.20	4.10	4.21
219138_at	ribosomal protein	RPL14	8.44	8.56	8.68
212430_at	RNA binding moti	RBM38	6.55	6.92	6.49
216877_at	Hypothetical gene	DKFZp686O1327	3.98	3.91	3.71
218686_s_at	rhomboid 5 homo	RHBDF1	8.02	8.15	7.99
214358_at	acetyl-CoA carbox	ACACA	7.42	7.53	7.41
220165_at	INO80 complex su	INO80D	6.84	6.68	6.54

212918_at	RecQ protein-like	RECQL	10.25	10.55	10.59
206948_at	sialidase 3 (memb	NEU3	4.70	4.66	4.66
201752_s_at	adducin 3 (gamma	ADD3	7.15	7.44	7.22
205497_at	zinc finger protein	ZNF175	5.28	5.40	5.33
203170_at	ribosomal RNA pr	RRP8	4.32	4.35	4.23
210200_at	WW domain conta	WWP2	5.75	5.64	5.57
206568_at	transition protein	TNP1	5.71	5.59	5.43
1316_at	thyroid hormone	THRA	7.28	7.23	7.07
203897_at	LYR motif contain	LYRM1	10.93	10.91	11.00
209360_s_at	runt-related trans	RUNX1	9.59	9.29	9.53
221089_at	NADPH oxidase 3	NOX3	4.03	4.20	4.05
204343_at	ATP-binding casse	ABCA3	8.01	7.76	7.65
204748_at	prostaglandin-enc	PTGS2	4.38	4.23	4.03
214817_at	unc-13 homolog A	UNC13A	4.87	4.92	4.76
222189_at	---	---	4.10	4.18	4.32
219658_at	pentatricopeptide	PTCD2	8.90	8.89	8.94
220356_at	corin, serine pepti	CORIN	3.19	3.33	3.10
216493_s_at	insulin-like growth	IGF2BP3	3.98	3.86	3.81
216970_at	regulator of G-prc	RGS7	4.69	4.58	4.55
204779_s_at	homeobox B7	HOXB7	7.43	7.49	7.37
202620_s_at	procollagen-lysine	PLOD2	8.54	8.85	9.05
215914_at	---	---	4.32	4.10	3.97
220651_s_at	minichromosome	MCM10	8.50	8.50	8.55
201741_x_at	splicing factor, arg	SFRS1	6.26	6.10	6.35
208786_s_at	microtubule-asso	MAP1LC3B	10.73	10.58	10.82
206067_s_at	Wilms tumor 1	WT1	3.40	3.42	3.35
201149_s_at	TIMP metallopept	TIMP3	6.08	6.00	5.80
221503_s_at	karyopherin alpha	KPNA3	10.07	10.18	10.19
201555_at	minichromosome	MCM3	10.40	10.45	10.49
215156_at	WD repeat domai	WDR61	7.45	7.44	7.36
204633_s_at	ribosomal protein	RPS6KA5	6.70	6.68	6.90
212225_at	eukaryotic transla	EIF1	5.77	5.78	5.85
202732_at	protein kinase (cA	PKIG	9.38	9.30	9.51
218041_x_at	solute carrier fam	SLC38A2	13.35	13.44	13.53
221943_x_at	Ribosomal protein	RPL38	10.91	11.06	11.19
218270_at	mitochondrial ribo	MRPL24	10.35	10.27	10.19
216199_s_at	mitogen-activated	MAP3K4	9.78	9.69	9.85
221139_s_at	cysteine sulfinic a	CSAD	6.42	6.61	6.65
221645_s_at	zinc finger protein	ZNF83	6.88	7.19	7.27
201256_at	cytochrome c oxid	COX7A2L	12.23	12.12	12.23
209621_s_at	PDZ and LIM dom	PDLIM3	7.62	7.69	7.59
212601_at	zinc finger, ZZ-typ	ZZEF1	7.92	7.78	7.85
214144_at	polymerase (RNA)	POLR2D	7.73	7.69	7.71
215345_x_at	---	---	5.48	5.55	5.34
202038_at	ubiquitination fac	UBE4A	10.81	10.93	10.96
206113_s_at	RAB5A, member R	RAB5A	7.74	7.87	8.05
203931_s_at	mitochondrial ribo	MRPL12	10.99	10.88	10.89

218737_at	strawberry notch	SBNO1	8.42	8.63	8.57
215997_s_at	cullin 4B	CUL4B	6.20	6.04	6.18
200706_s_at	lipopolysaccharide	LITAF	9.11	8.89	9.07
206440_at	lin-7 homolog A (C	LIN7A	4.59	4.38	4.42
214200_s_at	Collagen, type VI,	COL6A1	4.53	4.53	4.35
203559_s_at	amiloride binding	ABP1	4.11	4.22	4.43
201995_at	exostosin 1	EXT1	9.00	9.05	9.01
219229_at	solute carrier orga	SLCO3A1	6.72	6.67	6.94
217043_s_at	mitofusin 1	MFN1	8.77	8.97	9.07
201572_x_at	dCMP deaminase	DCTD	9.65	9.72	9.80
200762_at	dihydropyrimidina	DPYSL2	12.41	12.34	12.46
202737_s_at	LSM4 homolog, U	LSM4	10.77	10.74	10.87
211462_s_at	transducin (beta)-	TBL1Y	6.32	6.22	6.19
202941_at	NADH dehydrogen	NDUFV2	11.09	11.14	11.24
215274_at	solute carrier fam	SLC12A3	5.97	6.06	6.08
207730_x_at	---	---	8.74	8.75	8.71
210371_s_at	retinoblastoma bi	RBBP4	10.80	10.84	10.97
206312_at	guanylate cyclase	GUCY2C	4.40	4.28	4.28
220160_s_at	kaptin (actin bindi	KPTN	7.79	7.93	8.03
215113_s_at	SUMO1/sentrin/S	SENP3	6.97	6.90	7.02
209407_s_at	deformed epiderm	DEAF1	7.41	7.60	7.42
200878_at	endothelial PAS d	EPAS1	11.42	11.40	11.45
202094_at	baculoviral IAP rep	BIRC5	9.77	9.79	9.92
214747_at	zinc finger, BED-ty	ZBED4	5.28	5.34	5.45
203051_at	bromo adjacent h	BAHD1	8.35	8.30	8.21
208840_s_at	GTPase activating	G3BP2	8.61	8.69	8.70
211684_s_at	dynein, cytoplasm	DYNC1I2	11.62	11.71	11.73
205622_at	sphingomyelin ph	SMPD2	7.22	7.00	7.02
219953_s_at	chromosome 11 c	C11orf17	9.80	9.77	9.84
208654_s_at	CD164 molecule, s	CD164	10.61	10.59	10.82
206593_s_at	mediator complex	MED22	7.50	7.63	7.69
216602_s_at	phenylalanyl-tRNA	FARSA	8.63	8.51	8.70
203033_x_at	fumarate hydratas	FH	10.93	10.96	10.93
203684_s_at	B-cell CLL/lympho	BCL2	6.06	5.85	6.15
200923_at	lectin, galactoside	LGALS3BP	8.58	8.55	8.50
205429_s_at	membrane protei	MPP6	7.42	7.46	7.38
220009_at	LON peptidase N-t	LONRF3	4.34	4.35	4.43
200001_at	calpain, small sub	CAPNS1	9.84	9.80	9.85
59999_at	hypoxia inducible	HIF1AN	7.87	7.76	7.79
204238_s_at	chromosome 6 op	C6orf108	9.87	9.83	9.90
218392_x_at	sideroflexin 1	SFXN1	9.46	9.53	9.69
204221_x_at	GLI pathogenesis-	GLIPR1	8.15	8.25	8.25
208270_s_at	arginyl aminopept	RNPEP	9.87	9.85	9.90
201903_at	ubiquinol-cytochr	UQCRC1	10.09	10.15	10.20
210336_x_at	myeloid zinc finge	MZF1	8.62	8.66	8.57
222131_x_at	ras homolog gene	RHOT2	8.68	8.79	8.86
214229_at	dynein, axonemal	DNAH17	4.30	4.49	4.46

38521_at	CD22 molecule	CD22	7.41	7.23	7.25
208972_s_at	ATP synthase, H+	ATP5G1	11.29	11.25	11.15
204454_at	leucine zipper, do	LDOC1	5.07	4.93	4.76
213236_at	SAM and SH3 dom	SASH1	7.50	7.53	7.57
201488_x_at	KH domain contai	KHDRBS1	12.30	12.33	12.34
207695_s_at	immunoglobulin s	IGSF1	6.12	6.10	6.04
218288_s_at	coiled-coil domair	CCDC90B	10.11	9.95	10.15
218561_s_at	LYR motif contain	LYRM4	10.56	10.56	10.54
215014_at	potassium voltage	KCND3	3.02	3.03	2.92
220920_at	ATPase, class V, ty	ATP10B	4.34	4.45	4.24
209420_s_at	sphingomyelin ph	SMPD1	7.49	7.38	7.41
209605_at	thiosulfate sulfurt	TST	9.56	9.70	9.71
202366_at	acyl-CoA dehydro	ACADS	7.56	7.62	7.37
210475_at	POU class 3 home	POU3F1	4.97	5.05	4.97
209043_at	3'-phosphoadeno:	PAPSS1	10.51	10.52	10.51
216211_at	---	---	6.74	6.88	6.74
220872_at	hypothetical prote	PRO2964	3.65	3.68	3.72
210640_s_at	G protein-couplec	GPER	6.39	6.04	6.13
205043_at	cystic fibrosis tran	CFTR	4.97	4.56	4.63
217310_s_at	forkhead box J3	FOXJ3	5.28	4.61	4.87
208080_at	aurora kinase A	AURKA	6.12	6.24	6.20
208104_s_at	TSC22 domain fan	TSC22D4	7.08	7.07	7.08
203538_at	calcium modulatir	CAMLG	10.45	10.47	10.46
201290_at	SEC11 homolog A	SEC11A	12.64	12.70	12.75
210470_x_at	non-POU domain	NONO	9.80	9.70	9.79
35179_at	beta-1,3-glucuron	B3GAT3	6.94	6.97	6.93
209046_s_at	GABA(A) receptor	GABARAPL2	11.21	11.32	11.33
214131_at	chromosome Y op	CYorf15B	3.72	3.73	3.69
207557_s_at	ryanodine recepto	RYR2	5.25	5.21	4.98
219118_at	FK506 binding pro	FKBP11	10.35	10.36	10.45
216152_at	---	---	6.36	6.44	6.37
221453_at	glucose-6-phosph	G6PC2	5.48	5.54	5.64
221126_at	dickkopf homolog	DKK3	3.43	3.43	3.43
214393_at	Rho family GTPase	RND2	5.35	5.25	5.30
63305_at	PBX/knotted 1 ho	PKNOX2	10.24	10.10	10.24
220076_at	ankylosis, progres	ANKH	6.29	6.42	6.52
220967_s_at	zinc finger proteir	ZNF696	7.68	7.60	7.65
214260_at	COP9 constitutive	COPS8	6.86	6.92	6.98
220842_at	Abelson helper int	AHI1	5.16	5.02	5.06
204457_s_at	growth arrest-spe	GAS1	8.55	8.81	8.75
210347_s_at	B-cell CLL/lympho	BCL11A	4.22	4.37	4.40
203920_at	nuclear receptor s	NR1H3	7.15	7.15	7.18
219491_at	leucine rich repea	LRFN4	7.65	7.59	7.58
216163_at	---	---	5.42	5.49	5.38
210656_at	embryonic ectode	EED	3.81	3.97	4.15
202119_s_at	copine III	CPNE3	11.00	11.07	11.11
216921_s_at	keratin 35	KRT35	3.75	3.77	3.63

212890_at	solute carrier fam	SLC38A10	7.64	7.44	7.58
216924_s_at	dopamine recepto	DRD2	6.37	6.46	6.34
207105_s_at	phosphoinositide-	PIK3R2	6.80	6.55	6.62
216975_x_at	neuronal PAS dom	NPAS1	6.16	5.93	6.14
215092_s_at	nuclear factor of a	NFAT5	5.84	6.14	5.82
216077_s_at	l(3)mbt-like (Dros	L3MBTL	5.99	6.17	5.85
221135_s_at	asteroid homolog	ASTE1	7.92	7.87	7.81
217807_s_at	glioma tumor sup	GLTSCR2	13.58	13.42	13.52
214022_s_at	interferon induce	IFITM1	11.45	11.38	11.18
213312_at	chromosome 6 op	C6orf162	7.97	7.88	7.88
206366_x_at	chemokine (C mo	XCL1	4.79	4.95	4.73
200020_at	TAR DNA binding	TARDBP	10.82	10.89	10.78
201694_s_at	early growth resp	EGR1	8.22	8.79	8.39
203103_s_at	PRP19/PSO4 pre-r	PRPF19	10.25	10.29	10.31
203565_s_at	menage a trois ho	MNAT1	9.22	9.27	9.38
213707_s_at	distal-less homeo	DLX5	4.20	4.17	4.01
206354_at	solute carrier orga	SLCO1B3	6.64	6.76	7.08
216885_s_at	DDB1 and CUL4 as	DCAF8	7.22	7.25	7.11
218682_s_at	solute carrier fam	SLC4A1AP	9.02	8.92	9.08
218122_s_at	SUMO1/sentrin/S	SENP2	7.91	8.07	8.10
220432_s_at	cytochrome P450,	CYP39A1	3.76	3.95	4.19
207223_s_at	ROD1 regulator of	ROD1	6.65	6.62	6.76
205541_s_at	G1 to S phase tran	GSPT2	5.09	5.23	5.05
214390_s_at	branched chain an	BCAT1	7.70	7.52	7.88
214881_s_at	upstream binding	UBTF	6.67	6.65	6.51
201347_x_at	glyoxylate reducta	GRHPR	9.83	9.77	9.77
218361_at	golgi phosphoprot	GOLPH3L	9.13	9.09	9.09
203955_at	KIAA0649	KIAA0649	7.99	7.97	8.14
205058_at	solute carrier fam	SLC26A1	5.90	5.98	6.13
222113_s_at	epidermal growth	EPS15L1	6.68	6.78	6.59
203574_at	nuclear factor, int	NFIL3	9.96	9.66	10.00
215991_s_at	KIAA0090	KIAA0090	6.26	6.10	6.30
216797_at	---	---	4.51	4.60	4.71
200653_s_at	calmodulin 1 (pho	CALM1	11.64	11.71	11.69
218203_at	asparagine-linked	ALG5	10.98	11.00	11.04
200846_s_at	protein phosphata	PPP1CA	10.17	10.12	10.08
212411_at	IMP4, U3 small nu	IMP4	10.55	10.50	10.56
205427_at	zinc finger protein	ZNF354A	7.31	7.43	7.69
217268_at	RAB7A, member R	RAB7A	3.16	3.17	3.17
219433_at	BCL6 co-repressor	BCOR	7.06	7.21	7.34
220984_s_at	solute carrier orga	SLCO5A1	6.13	5.90	5.95
216068_at	---	---	3.08	3.26	3.26
212892_at	zinc finger protein	ZNF282	7.27	7.20	7.14
201957_at	protein phosphata	PPP1R12B	7.06	6.97	7.02
215667_x_at	PMS2 postmeiotic	LOC441259 /// PM	8.48	8.54	8.75
213912_at	TBC1 domain fam	TBC1D30	3.76	3.55	3.84
209222_s_at	oxysterol binding	OSBPL2	7.33	7.30	7.15

207310_s_at	nitric oxide syntha	NOS1	6.04	6.08	5.74
213242_x_at	KIAA0284	KIAA0284	8.80	8.81	8.77
204508_s_at	carbonic anhydras	CA12	9.53	9.50	9.56
215926_x_at	small nuclear RNA	SNAPC4	9.18	9.12	9.04
217293_at	---	---	5.50	5.50	5.44
205674_x_at	FXYD domain cont	FXYD2	5.88	5.66	5.60
219323_s_at	interleukin 18 bin	IL18BP	6.64	6.55	6.68
201642_at	interferon gamma	IFNGR2	10.02	9.89	9.82
207594_s_at	synaptojanin 1	SYNJ1	4.52	4.21	4.19
213489_at	Microtubule-asso	MAPRE2	7.71	7.69	7.56
208158_s_at	oxysterol binding	OSBPL1A	9.51	9.57	9.68
201850_at	capping protein (a	CAPG	7.44	7.25	7.45
207096_at	serum amyloid A4	SAA4	6.66	6.46	6.21
212147_at	Smg-5 homolog, r	SMG5	8.32	8.10	8.16
213940_s_at	formin binding pr	FNBP1	8.76	8.75	8.76
221672_s_at	trafficking protein	TRAPPC9	7.24	7.22	7.23
202692_s_at	upstream binding	UBTF	8.06	7.95	7.96
214380_at	PRP31 pre-mRNA	PRPF31	5.76	5.53	5.59
211986_at	AHNAK nucleopro	AHNAK	11.26	11.34	11.23
210483_at	hypothetical prote	MGC31957	6.78	6.55	6.63
201759_at	tubulin folding co	TBCD	6.61	6.83	6.60
206300_s_at	parathyroid horm	PTHLH	3.49	3.63	3.55
215062_at	---	---	5.85	6.02	5.64
209006_s_at	chromosome 1 op	C1orf63	8.05	8.59	8.24
221634_at	ribosomal protein	RPL23AP7	7.47	7.54	7.66
214516_at	histone cluster 1,	HIST1H4B	5.55	5.40	5.58
209038_s_at	EH-domain contai	EHD1	6.12	5.78	6.08
210190_at	syntaxin 11	STX11	5.17	5.23	5.17
218079_s_at	gametogenetin bi	GGNBP2	10.11	10.17	10.20
212803_at	NGFI-A binding pr	NAB2	7.87	7.71	7.78
208141_s_at	deoxyhypusine hy	DOHH	7.91	7.63	7.83
219469_at	dynein, cytoplasm	DYNC2H1	8.01	8.03	8.07
213642_at	---	---	8.81	8.89	8.88
219337_at	chromosome 1 op	C1orf159	6.83	6.63	6.73
215522_at	sortilin-related VP	SORCS3	3.55	3.57	3.58
201985_at	KIAA0196	KIAA0196	9.06	9.16	9.19
218300_at	chromosome 16 o	C16orf53	9.05	9.04	9.07
219528_s_at	B-cell CLL/lympho	BCL11B	3.66	3.51	3.88
203941_at	integrator comple	INTS9	7.80	7.69	7.82
221782_at	DnaJ (Hsp40) hom	DNAJC10	8.59	8.59	8.69
213042_s_at	ATPase, Ca++ tran	ATP2A3	4.64	4.78	4.68
208731_at	RAB2A, member R	RAB2A	11.05	11.11	11.24
207985_at	---	---	5.68	5.51	5.40
220572_at	hypothetical LOC5	DKFZp547G183	5.96	5.86	6.06
213650_at	golgin A8 family, r	GOLGA8A /// GOL	6.98	7.03	6.98
210375_at	prostaglandin E re	PTGER3	3.59	3.47	3.44
206979_at	complement com	C8B	5.84	5.81	6.00

200852_x_at	guanine nucleotid	GNB2	9.76	9.70	9.80
202124_s_at	trafficking protein	TRAK2	9.36	9.48	9.55
202598_at	S100 calcium bind	S100A13	10.34	10.31	10.24
205382_s_at	complement facto	CFD	7.97	8.12	8.01
211618_s_at	alkaline phosphat	ALPI	5.79	5.79	5.83
215310_at	adenomatous pol	APC	3.94	3.94	3.75
218668_s_at	RAP2C, member c	RAP2C	7.99	7.94	8.23
215544_s_at	U-box domain cor	UBOX5	7.45	7.35	7.40
216125_s_at	RAN binding prote	RANBP9	7.02	7.24	7.05
221930_at	---	---	6.31	6.32	6.26
209766_at	---	---	6.00	6.23	6.00
217998_at	hypothetical prote	LOC100289208 ///	8.29	8.32	8.22
218948_at	glutaminyl-tRNA s	QRSL1	7.20	7.13	7.22
204178_s_at	RNA binding moti	RBM14	9.22	9.14	9.11
205031_at	ephrin-B3	EFNB3	6.04	5.85	5.69
202919_at	MOB1, Mps One E	MOBKL3	9.69	9.78	9.76
206675_s_at	SKI-like oncogene	SKIL	5.24	5.03	5.09
208567_s_at	potassium inward	KCNJ12 /// KCNJ18	3.71	3.85	3.94
200789_at	enoyl CoA hydrata	ECH1	9.34	9.27	9.30
218470_at	tyrosyl-tRNA synt	YARS2	8.49	8.60	8.63
61732_r_at	intraflagellar tran	IFT74	4.89	4.72	4.89
217827_s_at	spastic paraplegia	SPG21	11.53	11.41	11.53
205064_at	small proline-rich	SPRR1B	4.24	4.05	3.97
206011_at	caspase 1, apopto	CASP1	6.40	6.89	6.92
203827_at	WD repeat domai	WIPI1	8.60	8.60	8.80
211944_at	HLA-B associated	BAT2L2	9.72	9.69	9.72
201190_s_at	phosphatidylinosi	PITPNA	9.40	9.34	9.32
203278_s_at	PHD finger protei	PHF21A	9.67	9.67	9.64
215635_at	---	---	5.56	5.77	5.76
218299_at	chromosome 11 c	C11orf24	8.71	8.60	8.75
206881_s_at	leukocyte immun	LILRA3	5.36	5.24	5.27
221215_s_at	receptor-interacti	RIPK4	10.52	10.43	10.44
222172_at	neuronal PAS dom	NPAS3	3.84	3.81	3.91
204746_s_at	protein interactin	PICK1	7.88	7.85	7.60
214110_s_at	---	---	10.04	9.93	10.10
206513_at	absent in melano	AIM2	5.05	5.34	5.26
204879_at	podoplanin	PDPN	9.91	9.74	9.78
218735_s_at	zinc finger protei	ZNF544	8.33	8.48	8.46
206830_at	solute carrier fam	SLC4A10	3.24	3.28	3.28
202429_s_at	protein phosphat	PPP3CA	10.68	10.68	10.81
208157_at	single-minded ho	SIM2	5.65	5.60	5.43
211509_s_at	reticulon 4	RTN4	13.03	13.06	13.23
217437_s_at	transforming, acic	TACC1	8.82	8.80	9.10
215559_at	ATP-binding casse	ABCC6 /// LOC100	3.80	3.67	3.80
219343_at	cell division cycle	CDC37L1	8.24	8.27	8.42
222064_s_at	alanyl-tRNA synth	AARSD1	9.20	9.06	9.16
200985_s_at	CD59 molecule, c	CD59	11.96	12.05	12.04

200898_s_at	meningioma expr	MGEA5	8.16	7.99	8.17
201063_at	reticulocalbin 1, E	RCN1	11.89	11.93	11.97
211322_s_at	sarcosine dehydro	SARDH	6.57	6.34	6.40
216660_at	myosin VIIB	MYO7B	3.51	3.49	3.74
220326_s_at	protein SOLO	FLJ10357	6.39	6.36	6.35
217903_at	striatin, calmoduli	STRN4	7.07	6.82	6.99
210584_s_at	polymerase (DNA-	POLDIP3 /// RRP7E	3.88	4.10	3.87
220964_s_at	RAB1B, member R	RAB1B	9.57	9.52	9.37
210047_at	solute carrier fam	SLC11A2	5.58	5.43	5.59
217286_s_at	NDRG family mem	NDRG3	9.99	9.80	9.64
213768_s_at	achaete-scute cor	ASCL1	5.56	5.59	5.63
216246_at	---	---	9.36	9.28	9.37
219934_s_at	sulfotransferase fa	SULT1E1	4.41	4.90	4.67
215865_at	Synaptotagmin XI	SYT12	7.30	7.21	7.06
201092_at	retinoblastoma bi	RBBP7	12.52	12.58	12.55
205652_s_at	tubulin tyrosine li	TTLL1	6.80	6.75	6.61
210359_at	metastasis suppre	MTSS1	4.03	3.92	4.02
212923_s_at	chromosome 6 op	C6orf145	8.76	8.55	8.92
218707_at	zinc finger protein	ZNF444	7.31	7.29	7.15
202542_s_at	aminoacyl tRNA sy	AIMP1	10.89	10.95	10.98
221919_at	heterogeneous nu	HNRNPA1	6.82	6.98	7.08
212692_s_at	LPS-responsive ve	LRBA	9.71	9.64	9.73
221746_at	ubiquitin-like 4A	UBL4A	9.20	9.25	9.18
211963_s_at	actin related prot	ARPC5	11.63	11.73	11.61
213685_at	---	---	7.71	7.73	7.91
202479_s_at	tribbles homolog	TRIB2	4.74	4.57	4.53
212174_at	adenylate kinase	AK2	9.47	9.60	9.42
219232_s_at	egl nine homolog	EGLN3	6.04	6.12	5.93
220471_s_at	myc target 1	MYCT1	4.64	4.51	4.55
202972_s_at	family with seque	FAM13A	7.77	7.69	7.67
205010_at	guanine nucleotid	GNL3L	7.70	7.71	7.86
39729_at	peroxiredoxin 2	PRDX2	12.45	12.35	12.45
210582_s_at	LIM domain kinas	LIMK2	7.65	7.39	7.27
220927_s_at	heparanase 2	HPSE2	4.64	4.61	4.58
221432_s_at	solute carrier fam	SLC25A28	7.85	7.87	7.99
205366_s_at	homeobox B6	HOXB6	5.24	5.36	5.24
221368_at	sialidase 2 (cytoso	NEU2	6.34	5.90	6.03
220337_at	neuroglobin	NGB	6.46	6.41	6.31
217729_s_at	amino-terminal er	AES	8.34	8.33	8.25
202447_at	2,4-dienoyl CoA re	DECR1	10.00	9.98	10.13
207356_at	defensin, beta 4A	DEFB4A	5.69	5.52	5.76
218341_at	phosphopantothe	PPCS	9.49	9.40	9.53
207920_x_at	zinc finger protein	ZFX	7.00	6.92	6.96
206188_at	zinc finger protein	ZNF623	7.00	7.09	7.19
207145_at	myostatin	MSTN	3.30	3.28	3.26
208116_s_at	mannosidase, alph	MAN1A1	4.28	3.99	4.13
218896_s_at	chromosome 17 o	C17orf85	7.46	7.48	7.41

214793_at	dual specificity ph	DUSP7	6.79	6.75	6.79
215878_at	integrin, beta 1 (fi	ITGB1	3.46	3.43	3.47
204676_at	transmembrane p	TMEM186	8.87	8.68	8.79
222171_s_at	PBX/knotted 1 ho	PKNOX2	9.24	9.14	9.42
204592_at	discs, large homol	DLG4	5.12	5.12	5.05
205100_at	glutamine-fructos	GFPT2	8.71	8.66	8.84
213956_at	centrosomal prote	CEP350	6.71	6.88	6.70
204452_s_at	frizzled homolog 1	FZD1	4.14	4.32	4.13
213636_at	KIAA1045	KIAA1045	5.74	5.76	5.69
210146_x_at	leukocyte immuno	LILRB2	3.55	3.46	3.60
217027_x_at	karyopherin (impo	KPNB1	9.25	9.12	9.14
202535_at	Fas (TNFRSF6)-ass	FADD	8.85	8.89	8.96
57540_at	ribokinase	RBKS	7.00	6.93	7.06
201065_s_at	general transcript	GTF2I /// GTF2IP1	9.71	9.80	9.71
218783_at	integrator comple	INTS7	7.88	7.71	7.83
212421_at	chromosome 22 c	C22orf9	7.27	7.30	7.35
221784_at	widely interspace	WIZ	7.01	6.81	6.87
220229_s_at	adaptor-related p	AP4E1	3.63	3.86	3.78
216642_at	---	---	3.80	4.05	3.86
203445_s_at	CTD (carboxy-term	CTDSP2	9.97	9.90	9.97
208045_at	---	---	3.37	3.44	3.42
201135_at	enoyl CoA hydrata	ECHS1	10.61	10.52	10.65
200613_at	adaptor-related p	AP2M1	9.43	9.37	9.51
209940_at	poly (ADP-ribose)	PARP3	6.68	6.59	6.58
219634_at	carbohydrate (cho	CHST11	6.48	6.28	6.64
206235_at	ligase IV, DNA, AT	LIG4	6.59	6.57	6.60
203275_at	interferon regulat	IRF2	7.89	7.69	7.73
205413_at	metallophosphoe	MPPED2	3.29	3.14	3.06
213620_s_at	intercellular adhe	ICAM2	6.56	6.43	6.43
216396_s_at	etoposide induced	EI24	10.70	10.73	10.72
200738_s_at	phosphoglycerate	PGK1	13.70	13.65	13.70
213908_at	WAS protein hom	WHAMML1 /// WH	6.88	7.00	7.23
200048_s_at	jumping transloca	JTB	12.77	12.70	12.74
211228_s_at	RAD17 homolog (S	RAD17	8.96	9.12	9.11
206975_at	lymphotoxin alpha	LTA	6.12	5.96	5.89
218481_at	exosome compon	EXOSC5	9.83	9.56	9.69
200822_x_at	triosephosphate is	TPI1	12.80	12.74	12.76
213279_at	dehydrogenase/re	DHRS1	7.34	7.42	7.51
209269_s_at	spleen tyrosine ki	SYK	4.95	5.21	4.96
216149_at	leucine rich repea	LRRC37B2	4.18	4.27	4.25
221103_s_at	WD repeat domai	WDR52	4.04	4.14	3.70
206766_at	integrin, alpha 10	ITGA10	8.08	8.26	8.05
221675_s_at	choline phosphotr	CHPT1	11.14	11.09	11.13
207943_x_at	pleiomorphic ade	PLAGL1	7.37	7.59	7.56
210617_at	phosphate regulat	PHEX	5.59	5.36	5.34
212202_s_at	transmembrane p	TMEM87A	10.04	9.95	10.17
212367_at	fem-1 homolog b	FEM1B	9.83	9.81	9.81

216887_s_at	LIM domain bindi	LDB3	4.76	4.80	4.76
221559_s_at	MIS12, MIND kine	MIS12	8.49	8.66	8.65
217424_at	---	---	4.58	4.60	4.51
209566_at	insulin induced ge	INSIG2	8.56	8.71	8.76
204730_at	regulating synapti	RIMS3	5.28	4.97	5.15
205760_s_at	8-oxoguanine DN/	OGG1	8.04	8.05	7.92
212921_at	SET and MYND do	SMYD2	4.84	4.92	4.60
214987_at	GRB2-associated I	GAB1	6.35	6.42	6.39
220001_at	peptidyl arginine (	PADI4	4.13	4.32	4.15
204252_at	cyclin-dependent	CDK2	10.10	10.19	10.05
200982_s_at	annexin A6	ANXA6	7.71	7.90	7.86
210102_at	von Willebrand fa	VWA5A	5.66	5.67	5.60
212150_at	EFR3 homolog A (	EFR3A	9.47	9.58	9.68
204390_at	---	---	5.95	5.70	5.65
217802_s_at	nuclear casein kin	NUCKS1	12.38	12.35	12.36
204428_s_at	lecithin-cholester	LCAT	6.28	6.11	6.23
204342_at	solute carrier fam	SLC25A24	10.84	10.97	11.02
212973_at	ribose 5-phosphat	RPIA	10.55	10.47	10.71
216003_at	COX10 homolog, (	COX10	3.74	3.76	3.91
203145_at	sperm associated	SPAG5	9.56	9.58	9.52
215947_s_at	family with seque	FAM136A	10.11	10.18	10.19
203390_s_at	kinesin family mei	KIF3C	6.44	6.39	6.28
216920_s_at	TCR gamma altern	TARP /// TRGC2	4.90	4.59	4.93
204997_at	glycerol-3-phosph	GPD1	6.13	5.95	6.01
215789_s_at	adherens junction	AJAP1	7.04	7.00	7.07
201424_s_at	cullin 4A	CUL4A	9.98	10.03	10.11
216256_at	glutamate recepto	GRM8	6.27	6.14	6.24
208918_s_at	NAD kinase	NADK	9.52	9.45	9.64
202664_at	WAS/WASL intera	WIPF1	9.53	9.60	9.92
214322_at	calcium/calmodul	CAMK2G	4.63	4.71	4.51
213481_at	S100 calcium bind	S100A13	6.28	6.38	6.17
216954_x_at	ATP synthase, H+	ATP5O	9.73	9.75	9.77
210334_x_at	baculoviral IAP re	BIRC5	9.71	9.74	9.64
206018_at	forkhead box G1	FOXG1	3.67	3.98	3.61
206663_at	Sp4 transcription	SP4	4.15	4.25	4.04
216080_s_at	fatty acid desatur	FADS3	9.14	9.15	9.22
213191_at	toll-like receptor a	TICAM1	8.43	8.52	8.57
206007_at	proteoglycan 4	PRG4	3.27	3.38	3.51
218095_s_at	transmembrane p	TMEM165	9.91	9.85	10.04
202995_s_at	fibulin 1	FBLN1	10.26	10.15	10.35
212485_at	G patch domain c	GPATCH8	8.29	8.36	8.37
220108_at	guanine nucleotid	GNA14	3.60	3.87	3.67
202783_at	nicotinamide nucl	NNT	10.43	10.36	10.57
215525_at	---	---	5.05	4.99	4.88
211801_x_at	mitofusin 1	MFN1	7.07	7.35	7.33
214247_s_at	dickkopf homolog	DKK3	12.32	12.29	12.42
214848_at	---	---	7.61	7.56	7.77

214174_s_at	PDZ and LIM dom	PDLIM4	7.30	7.22	7.24
206778_at	crystallin, beta B2	CRYBB2	7.25	7.20	7.24
220272_at	basonuclin 2	BNC2	4.96	5.21	5.16
202990_at	phosphorylase, gl	PYGL	4.51	4.27	4.48
210404_x_at	calcium/calmodul	CAMK2B	4.78	4.81	4.69
218501_at	Rho guanine nucle	ARHGEF3	5.73	5.75	6.25
202580_x_at	forkhead box M1	FOXM1	9.61	9.52	9.59
220958_at	unc-51-like kinase	ULK4	4.30	4.30	4.41
206873_at	carbonic anhydras	CA6	4.14	4.05	3.91
213402_at	zinc finger protein	ZNF787	7.79	7.53	7.59
206775_at	cubilin (intrinsic fa	CUBN	5.43	5.57	5.36
202610_s_at	mediator complex	MED14	7.99	7.99	8.07
220370_s_at	ubiquitin specific		USP36	6.19	6.00	6.16
203427_at	ASF1 anti-silencing	ASF1A	8.85	8.98	9.19
218195_at	chromosome 6 op	C6orf211	8.81	8.95	9.09
204084_s_at	ceroid-lipofuscino	CLN5	8.61	8.75	8.78
204578_at	diphosphoinositol	PPIP5K1	7.33	7.11	7.23
203043_at	zinc finger, BED-ty	ZBED1	8.59	8.76	8.80
216966_at	integrin, alpha 2b	ITGA2B	3.49	3.39	3.43
216565_x_at	---	---	9.88	9.81	9.72
216858_x_at	---	---	8.90	8.97	8.85
222175_s_at	mediator complex	MED15	8.50	8.47	8.42
209736_at	SRY (sex determin	SOX13	7.41	7.36	7.16
211355_x_at	leptin receptor	LEPR	4.41	4.28	4.43
215710_at	ST3 beta-galactos	ST3GAL4	4.59	4.58	4.53
221745_at	DDB1 and CUL4 as	DCAF7	8.79	8.79	8.80
218308_at	transforming, acic	TACC3	8.76	8.82	8.76
205284_at	URB2 ribosome bi	URB2	8.38	8.29	8.50
216185_at	Fucosyltransferase	FUT9	4.17	3.87	3.75
218910_at	anoctamin 10	ANO10	7.17	7.16	7.23
200782_at	annexin A5	ANXA5	12.07	11.99	12.09
208664_s_at	tetratricopeptide	TTC3	7.70	7.76	7.65
214937_x_at	pericentriolar mat	PCM1	9.26	9.33	9.47
203264_s_at	Cdc42 guanine nu	ARHGEF9	7.60	7.45	7.54
211540_s_at	retinoblastoma 1	RB1	5.57	5.42	5.53
210420_at	solute carrier fam	SLC24A1	6.25	6.26	6.22
207593_at	ATP-binding casse	ABCG4	5.59	5.51	5.31
212026_s_at	exocyst complex c	EXOC7	6.67	6.66	6.74
206969_at	keratin 34	KRT34	7.96	7.89	8.04
207178_s_at	fyn-related kinase	FRK	3.71	3.76	3.87
220665_at	leucine zipper pro	LUZP4	4.58	4.66	4.79
212527_at	PPPDE peptidase c	PPPDE2	8.93	8.82	8.84
205942_s_at	acyl-CoA syntheta	ACSM3	6.26	6.08	6.06
206885_x_at	growth hormone 1	GH1	5.74	6.03	5.80
202468_s_at	catenin (cadherin-	CTNNAL1	11.23	11.22	11.25
220243_at	zinc finger and BT	ZBTB44	5.62	5.62	5.66
203610_s_at	tripartite motif-co	TRIM38	6.71	6.94	7.03

219264_s_at	protein phosphata	PPP2R3B	6.72	6.56	6.57
204361_s_at	src kinase associat	SKAP2	7.98	8.05	8.31
207813_s_at	ferredoxin reducta	FDXR	7.15	7.24	7.03
215440_s_at	brain expressed, X	BEX4	3.60	3.31	3.46
202996_at	polymerase (DNA-	POLD4	7.90	7.72	7.78
209447_at	spectrin repeat cc	SYNE1	6.68	6.88	6.93
210885_s_at	tripartite motif-cc	TRIM15	5.27	5.23	5.19
210285_x_at	Wilms tumor 1 ass	WTAP	9.85	9.78	9.91
215005_at	N-terminal EF-har	NECAB2	6.84	6.71	6.72
207375_s_at	interleukin 15 rec	IL15RA	9.01	8.73	8.76
209059_s_at	endothelial differe	EDF1	11.52	11.43	11.46
210014_x_at	isocitrate dehydrc	IDH3B	10.15	10.15	10.22
222376_at	---	---	8.25	8.27	8.31
217838_s_at	Enah/Vasp-like	EVL	8.02	8.00	8.10
221773_at	ELK3, ETS-domain	ELK3	10.52	10.66	10.74
206676_at	carcinoembryonic	CEACAM8	3.05	3.24	3.20
214703_s_at	mannosidase, alpl	MAN2B2	7.47	7.40	7.37
217722_s_at	neugrin, neurite o	NGRN	11.59	11.60	11.57
221087_s_at	apolipoprotein L,	APOL3	6.89	6.75	6.73
205512_s_at	apoptosis-inducin	AIFM1	9.04	9.13	9.22
201588_at	thioredoxin-like 1	TXNL1	11.74	11.70	11.70
208256_at	ephrin-A2	EFNA2	4.72	4.56	4.48
216726_at	VENT homeobox (	VENTXP1	5.04	4.96	4.98
215173_at	leucine rich repea	LRRC50	5.52	5.63	5.35
204650_s_at	amyloid beta (A4)	APBB3	7.01	6.93	6.72
202712_s_at	creatine kinase, m	CKMT1A /// CKMT	6.31	6.28	6.10
212001_at	splicing factor, arg	SFRS14	8.26	8.23	8.22
213470_s_at	heterogeneous nu	HNRNPH1	8.78	8.73	8.81
202423_at	MYST histone ace	MYST3	9.23	9.23	9.25
220232_at	stearoyl-CoA desa	SCD5	6.13	6.32	6.17
204017_at	KDEL (Lys-Asp-Glu	KDELR3	9.08	9.39	9.36
217075_x_at	myeloid/lymphoic	MLL4	3.13	3.10	3.26
203630_s_at	component of olig	COG5	9.56	9.27	9.36
216412_x_at	similar to hCG919	LOC100290557	6.72	6.81	6.61
204656_at	Src homology 2 dc	SHB	7.45	7.00	7.26
63009_at	SHQ1 homolog (S.	SHQ1	8.67	8.82	8.79
215812_s_at	similar to solute c	LOC653562 /// SLC	9.61	9.53	9.27
219891_at	pyroglutamyl-pep	PGPEP1	6.74	6.67	6.57
208897_s_at	DEAD (Asp-Glu-Ala	DDX18	10.23	10.28	10.31
215517_at	pygopus homolog	PYGO1	5.94	5.54	5.90
215224_at	small nucleolar RN	SNORA21	7.98	8.13	8.23
213988_s_at	spermidine/sperm	SAT1	8.15	8.12	8.29
201181_at	guanine nucleotid	GNAI3	9.05	9.11	9.23
207934_at	ret finger protein-	RFPL1	3.33	3.27	3.19
218522_s_at	microtubule-asso	MAP1S	6.34	6.34	6.21
203856_at	vaccinia related ki	VRK1	10.09	10.19	10.24
200634_at	profilin 1	PFN1	10.29	10.26	10.29

33323_r_at	stratifin	SFN	11.42	11.76	11.50
201418_s_at	SRY (sex determin	SOX4	7.92	7.89	7.80
58780_s_at	protein SOLO	FLJ10357	7.83	7.70	7.74
211006_s_at	potassium voltage	KCNB1	5.70	5.67	5.96
205711_x_at	ATP synthase, H+	ATP5C1	12.61	12.61	12.58
216161_at	strawberry notch	SBNO1	4.71	4.80	4.97
220085_at	helicase, lymphoi	HELLS	8.71	8.77	8.84
206771_at	uroplakin 3A	UPK3A	6.18	6.10	6.07
207410_s_at	T-cell leukemia ho	TLX2	3.75	3.98	3.68
202351_at	integrin, alpha V (	ITGAV	10.64	10.69	10.82
209853_s_at	proteasome (pros	PSME3	10.26	10.13	10.28
217554_at	---	---	4.39	4.43	4.59
202348_s_at	torsin family 1, m	TOR1A	9.44	9.40	9.39
209866_s_at	latrophilin 3	LPHN3	8.80	8.81	8.90
216215_s_at	RNA binding moti	RBM9	10.19	10.14	10.28
216438_s_at	thymosin beta 4,)	TMSB4X /// TMSL3	13.47	13.46	13.60
202072_at	heterogeneous nu	HNRNPL	10.79	10.69	10.73
219012_s_at	chromosome 11 o	C11orf30	4.98	5.06	5.15
202332_at	casein kinase 1, e	CSNK1E	8.86	8.72	8.82
219408_at	protein arginine n	PRMT7	7.34	7.41	7.29
212719_at	PH domain and le	PHLPP1	6.31	6.18	6.33
39835_at	SET binding factor	SBF1	7.90	7.93	7.86
220933_s_at	zinc finger, CCHC	ZCCHC6	8.76	9.04	8.86
205907_s_at	osteomodulin	OMD	3.65	3.45	3.50
219837_s_at	cytokine-like 1	CYTL1	3.49	3.55	3.53
215235_at	spectrin, alpha, no	SPTAN1	8.83	8.67	8.71
213062_at	N-terminal aspara	NTAN1	8.45	8.54	8.51
216718_at	chromosome 1 op	C1orf46	4.88	4.55	4.48
220237_at	ATG3 autophagy r	ATG3	5.02	5.06	5.15
208636_at	actinin, alpha 1	ACTN1	12.36	12.31	12.39
209927_s_at	chromosome 1 op	C1orf77	8.61	8.42	8.56
221470_s_at	interleukin 1 fami	IL1F7	3.43	3.47	3.60
201471_s_at	sequestosome 1	SQSTM1	10.89	10.70	10.92
218364_at	leucine rich repea	LRRFIP2	7.91	7.77	8.16
209405_s_at	family with seque	FAM3A	7.54	7.22	7.22
204450_x_at	apolipoprotein A-I	APOA1	5.15	5.22	4.94
204831_at	cyclin-dependent	CDK8	8.90	8.83	8.97
218330_s_at	neuron navigator	NAV2	10.97	10.96	11.04
37424_at	coiled-coil alpha-h	CCHCR1	7.21	7.27	7.24
208372_s_at	LIM domain kinas	LIMK1	5.75	5.67	5.59
203825_at	bromodomain cor	BRD3	9.20	9.07	9.12
214829_at	aminoadipate-sen	AASS	6.18	6.60	6.47
213502_x_at	glucuronidase, be	LOC91316	7.50	7.59	7.54
212360_at	adenosine monop	AMPD2	8.96	8.93	8.97
37232_at	KIAA0586	KIAA0586	7.50	7.70	7.63
205571_at	lipoyltransferase 1	LIPT1	7.76	7.83	7.87
204016_at	leucyl-tRNA synth	LARS2	9.00	8.98	9.02

213860_x_at	casein kinase 1, al	CSNK1A1	12.25	12.24	12.21
202999_s_at	lysyl oxidase-like 2	LOXL2	4.99	4.90	4.75
215646_s_at	versican	VCAN	5.10	5.27	5.47
207378_at	trehalase (brush-b	TREH	5.37	5.25	4.97
219806_s_at	chromosome 11 c	C11orf75	7.41	7.62	7.55
213608_s_at	SRR1 domain cont	SRRD	8.98	8.96	9.00
201074_at	SWI/SNF related,	SMARCC1	10.69	10.63	10.70
216413_at	---	---	4.94	4.93	4.79
215388_s_at	complement facto	CFH /// CFHR1	7.17	7.42	7.28
217318_x_at	killer cell immuno	KIR2DL1 /// KIR2D	6.11	6.09	6.10
219193_at	WD repeat domai	WDR70	8.48	8.50	8.60
201087_at	paxillin	PXN	9.76	9.71	9.80
216693_x_at	hepatoma-derived	HDGFRP3	9.81	9.81	9.87
210684_s_at	discs, large homol	DLG4	6.05	6.12	6.12
212404_s_at	ubiquitin protein l	UBE3B	4.37	4.22	4.12
215615_x_at	---	---	6.07	6.08	6.03
210445_at	fatty acid binding	FABP6	6.72	6.76	6.67
204177_s_at	kelch-like 20 (Dros	KLHL20	8.38	8.44	8.40
207828_s_at	centromere prote	CENPF	10.62	10.82	10.79
202186_x_at	protein phosphata	PPP2R5A	5.40	5.37	5.51
212238_at	additional sex con	ASXL1	7.39	7.38	7.35
202876_s_at	pre-B-cell leukemi	PBX2	8.56	8.59	8.40
209567_at	RRS1 ribosome bi	RRS1	10.66	10.47	10.57
212131_at	LSM14A, SCD6 ho	LSM14A	10.18	10.13	10.30
208734_x_at	RAB2A, member R	RAB2A	10.26	10.34	10.44
218160_at	NADH dehydroger	NDUFA8	11.54	11.57	11.48
39650_s_at	erythropoietin rec	EPOR	6.41	6.29	6.09
200856_x_at	nuclear receptor c	C20orf191 /// LOC	7.04	6.82	7.00
210852_s_at	aminoadipate-sen	AASS	5.88	5.82	6.07
214825_at	family with seque	FAM155A	6.12	6.15	5.97
212720_at	poly(A) polymeras	PAPOLA	7.98	8.07	7.98
205392_s_at	chemokine (C-C m	CCL14 /// CCL14-C	5.74	5.75	5.54
204669_s_at	ring finger protein	RNF24	7.49	7.28	7.68
208614_s_at	filamin B, beta	FLNB	9.53	9.38	9.40
212229_s_at	F-box protein 21	FBXO21	9.94	10.00	9.95
217880_at	cell division cycle	CDC27	10.01	10.16	10.12
201908_at	dishevelled, dsh h	DVL3	8.53	8.64	8.48
209239_at	nuclear factor of k	NFKB1	9.78	9.42	9.66
203283_s_at	heparan sulfate 2-	HS2ST1	7.78	7.85	7.99
202074_s_at	optineurin	OPTN	9.83	9.83	9.90
201640_x_at	cleft lip and palate	CLPTM1	7.83	7.82	7.74
204134_at	phosphodiesteras	PDE2A	5.80	6.12	5.60
208182_x_at	interferon, alpha 1	IFNA14	4.15	4.45	4.45
221375_at	olfactory receptor	OR1G1	4.89	5.11	4.80
213335_s_at	ST3 beta-galactos	ST3GAL6	5.17	4.91	5.17
204637_at	glycoprotein horm	CGA	4.20	4.25	4.25
214513_s_at	cAMP responsive	CREB1	7.07	7.02	7.05

213024_at	TATA element mo	TMF1	6.50	6.88	6.90
218005_at	zinc finger protein	ZNF22	10.07	10.04	10.25
201844_s_at	RING1 and YY1 bi	RYBP	10.47	10.48	10.56
206039_at	RAB33A, member	RAB33A	4.26	4.31	4.21
210004_at	oxidized low dens	OLR1	3.60	3.45	3.58
222183_x_at	SNW domain cont	SNW1	8.29	8.32	8.04
217991_x_at	single stranded D	SSBP3	6.14	6.06	5.94
212023_s_at	antigen identified	MKI67	9.09	9.07	9.22
221581_s_at	linker for activatio	LAT2	6.71	6.66	6.63
214713_at	YLP motif contain	YLPM1	7.05	7.13	6.95
202446_s_at	phospholipid scra	PLSCR1	10.94	10.81	10.99
222102_at	glutathione S-tran	GSTA3	5.11	4.74	5.07
220818_s_at	transient recepto	TRPC4	8.00	7.96	8.00
203459_s_at	vacuolar protein s	VPS16	8.75	8.78	8.76
205014_at	fibroblast growth	FGFBP1	5.77	5.92	5.79
207812_s_at	golgi reassembly s	GORASP2	10.24	10.24	10.19
201292_at	topoisomerase (D	TOP2A	11.89	11.90	12.09
220275_at	CUB and zona pell	CUZD1	7.69	7.79	7.78
204297_at	phosphoinositide-	PIK3C3	9.38	9.40	9.54
210911_at	inhibitor of DNA b	ID2B	5.40	5.20	5.29
221755_at	EH domain bindin	EHBP1L1	6.82	6.93	6.83
211755_s_at	ATP synthase, H+	ATP5F1	13.37	13.43	13.46
214151_s_at	cell cycle progress	CCPG1	7.48	7.52	7.61
219543_at	phenazine biosynt	PBLD	6.14	6.15	6.32
211194_s_at	tumor protein p6	TP63	5.86	5.62	5.83
200057_s_at	non-POU domain	NONO	12.61	12.62	12.58
209915_s_at	neurexin 1	NRXN1	3.13	3.29	3.13
218893_at	isochorismatase d	ISOC2	8.67	8.80	8.81
219430_at	G protein-couplec	GPR137	5.47	5.34	5.17
218724_s_at	TGFB-induced fac	TGIF2	8.31	8.20	8.29
215531_s_at	gamma-aminobut	GABRA5	3.32	3.39	3.45
200895_s_at	FK506 binding pro	FKBP4	8.51	8.59	8.51
213837_at	l(3)mbt-like (Dros	L3MBTL	4.69	4.86	5.01
200066_at	IK cytokine, down	IK	10.55	10.53	10.53
217927_at	signal peptidase c	SPCS1	12.67	12.71	12.69
205215_at	ring finger protein	RNF2	7.01	6.97	7.16
202582_s_at	RAN binding prote	RANBP9	11.10	11.07	11.29
214496_x_at	MYST histone ace	MYST4	7.70	7.71	7.67
221788_at	---	---	7.27	7.63	7.52
211404_s_at	amyloid beta (A4)	APLP2	8.77	8.78	8.79
222341_x_at	---	---	6.15	6.03	5.83
208000_at	glycosylphosphati	GML	4.01	4.13	4.03
214394_x_at	eukaryotic transla	EEF1D	13.46	13.37	13.46
212615_at	chromodomain he	CHD9	8.87	8.97	8.93
220306_at	family with seque	FAM46C	4.53	4.57	4.40
207335_x_at	ATP synthase, H+	ATP5I	11.17	11.23	11.40
212375_at	E1A binding prote	EP400	8.99	9.02	8.98

204255_s_at	vitamin D (1,25- d	VDR	7.94	8.03	8.00
46142_at	lipase maturation	LMF1	3.71	3.52	3.58
204825_at	maternal embryo	MELK	11.76	11.74	11.86
212040_at	trans-golgi netwo	TGOLN2	9.43	9.33	9.56
203372_s_at	suppressor of cyto	SOCS2	6.68	6.41	6.92
221045_s_at	period homolog 3	PER3	5.76	5.70	5.53
202507_s_at	synaptosomal-ass	SNAP25	6.71	6.84	6.74
203542_s_at	Kruppel-like facto	KLF9	11.81	11.61	11.87
213752_at	kazrin	KAZ	5.23	4.94	5.02
204639_at	adenosine deamir	ADA	9.25	9.33	9.21
203259_s_at	HD domain contai	HDDC2	11.57	11.59	11.68
205894_at	arylsulfatase E (ch	ARSE	5.21	5.24	4.89
203054_s_at	T-cell leukemia tra	TCTA	7.95	7.90	8.01
215821_x_at	pregnancy specifi	PSG3	6.53	6.67	6.91
212204_at	transmembrane p	TMEM87A	10.48	10.37	10.60
209672_s_at	missing oocyte, m	MIOS	8.19	8.40	8.58
213135_at	T-cell lymphoma i	TIAM1	5.09	4.56	4.92
219046_s_at	PBX/knotted 1 ho	PKNOX2	9.52	9.43	9.55
213049_at	Ral GTPase activa	RALGAPA1	9.07	9.02	9.11
218740_s_at	CDK5 regulatory s	CDK5RAP3	9.27	9.26	9.16
214471_x_at	luteinizing hormo	LHB	7.36	7.34	7.19
204242_s_at	acyl-CoA oxidase 3	ACOX3	7.36	7.24	7.06
221487_s_at	endosulfine alpha	ENSA	7.07	6.99	7.00
206639_x_at	histatin 1	HTN1	3.28	3.50	3.37
202255_s_at	signal-induced pro	SIPA1L1	8.36	8.20	8.26
207417_s_at	zinc finger protein	ZNF177	5.87	6.22	6.04
209632_at	protein phosphata	PPP2R3A	6.73	6.83	6.76
202860_at	interleukin 8	IL8	8.75	8.74	8.76
212687_at	LIM and senescen	LIMS1	10.88	10.91	11.05
200713_s_at	microtubule-asso	MAPRE1	11.99	12.09	11.97
222243_s_at	transducer of ERB	TOB2	9.20	9.36	9.29
213303_x_at	zinc finger and BT	ZBTB7A	3.73	3.70	3.88
221241_s_at	BCL2-like 14 (apo	BCL2L14	5.38	5.63	5.60
205679_x_at	aggrecan	ACAN	6.37	6.44	6.49
206451_at	TBCC domain cont	TBCCD1	7.70	7.70	7.73
222297_x_at	---	---	7.44	7.43	7.55
219296_at	zinc finger, DHHC-	ZDHHC13	7.60	7.67	7.62
200970_s_at	stress-associated	SERP1	10.12	10.27	10.31
210316_at	fms-related tyrosi	FLT4	4.94	4.64	4.88
213193_x_at	T cell receptor be	TRBC1	5.50	5.65	5.53
205127_at	prostaglandin-enc	PTGS1	5.47	5.54	5.61
204851_s_at	doublecortin	DCX	3.46	3.50	3.34
202469_s_at	cleavage and poly	CPSF6	9.57	9.79	9.68
204209_at	phosphate cytidyl	PCYT1A	6.90	7.07	6.89
200690_at	heat shock 70kDa	HSPA9	9.21	9.24	9.42
216506_x_at	myeloid/lymphoi	MLLT10	7.96	7.94	7.92
208683_at	calpain 2, (m/II) la	CAPN2	12.77	12.83	12.82

211054_at	inversin	INVS	3.29	3.19	3.28
203416_at	CD53 molecule	CD53	6.33	6.37	6.23
220878_at	---	---	3.26	3.07	3.16
202825_at	transcription elon	TCEB1	9.63	9.60	9.62
208562_s_at	ATP-binding casse	ABCC9	5.08	4.75	4.79
204817_at	extra spindle pole	ESPL1	9.65	9.71	9.57
215676_at	BRF1 homolog, su	BRF1	7.18	7.25	7.04
203795_s_at	B-cell CLL/lympho	BCL7A	6.31	6.05	6.10
213600_at	signal-induced prc	SIPA1L3	8.21	8.16	8.15
205400_at	Wiskott-Aldrich sy	WAS	6.04	5.97	6.05
210683_at	neurturin	NRTN	6.67	6.22	6.56
212819_at	ankyrin repeat an	ASB1	6.96	6.96	7.37
266_s_at	CD24 molecule	CD24	4.33	4.55	4.27
212521_s_at	phosphodiesteras	PDE8A	8.49	8.49	8.50
205131_x_at	C-type lectin dom	CLEC11A	6.38	6.36	6.45
207165_at	hyaluronan-media	HMMR	11.95	12.12	12.25
219557_s_at	nuclear receptor i	NRIP3	8.68	8.65	8.70
207044_at	thyroid hormone	THRB	4.72	4.70	4.75
215490_at	chromosome 1 op	C1orf69	5.99	5.91	5.83
204172_at	coproporphyrinog	CPOX	9.84	9.85	10.20
213634_s_at	tRNA 5-methylam	TRMU	8.17	8.12	8.16
207626_s_at	solute carrier fam	SLC7A2	5.82	5.44	5.71
210464_at	chromosome 8 op	C8orf71	3.47	3.51	3.46
211508_s_at	growth hormone 2	GH2	3.59	3.53	3.37
217156_at	---	---	5.81	6.00	5.83
218121_at	heme oxygenase (	HMOX2	7.30	7.39	7.16
211490_at	adrenergic, alpha-	ADRA1A	5.31	5.21	5.13
211491_at	adrenergic, alpha-	ADRA1A	5.16	5.02	4.94
201314_at	serine/threonine l	STK25	8.72	8.76	8.70
205298_s_at	butyrophilin, subf	BTN2A2	7.45	7.46	7.51
218424_s_at	STEAP family men	STEAP3	9.92	10.02	10.04
221579_s_at	nudix (nucleoside	NUDT3	9.01	8.94	9.03
200869_at	ribosomal protein	RPL18A /// RPL18A	14.68	14.62	14.62
218747_s_at	TAP binding prote	TAPBPL	6.18	6.36	6.01
220368_s_at	SMEK homolog 1,	SMEK1	7.58	7.50	7.47
221335_x_at	chromosome 19 o	C19orf61	7.07	7.17	7.17
202812_at	STAM binding pro	STAMBP	7.91	7.78	7.76
204354_at	protection of telo	POT1	9.38	9.31	9.45
219989_s_at	ankyrin repeat an	ANKS1B	4.25	4.21	4.23
213307_at	SH3 and multiple a	SHANK2	3.88	3.90	3.80
213567_at	---	---	8.57	8.61	8.82
201121_s_at	progesterone rece	PGRMC1	11.92	12.01	11.96
201812_s_at	chromosome 4 op	C4orf46 /// TOMM	13.44	13.42	13.46
220865_s_at	prenyl (decapreny	PDSS1	8.51	8.72	8.78
212640_at	protein tyrosine p	PTPLB	11.19	11.26	11.29
216548_x_at	high-mobility grou	HMGB3L1	7.79	8.00	7.87
221120_at	---	---	4.33	4.40	4.43

212237_at	additional sex con	ASXL1	8.84	9.02	8.93
202480_s_at	death effector do	DEDD	6.05	5.87	6.11
200709_at	FK506 binding pro	FKBP1A	9.81	9.83	9.82
50965_at	RAB26, member R	RAB26	6.80	7.12	6.95
221791_s_at	coiled-coil domair	CCDC72	13.84	13.86	13.84
215747_s_at	regulator of chror	RCC1 /// SNHG3-R	7.63	7.53	7.57
208413_at	hypothetical prote	LOC100130354	4.34	4.29	4.14
217277_at	---	---	5.03	5.27	5.03
204679_at	potassium channe	KCNK1	9.11	9.31	9.33
213331_s_at	NIMA (never in m	NEK1	8.63	8.57	8.58
205655_at	Mdm4 p53 bindin	MDM4	4.58	4.37	4.45
215486_at	phosphoribosyl py	PRPS1L1	3.68	3.58	3.83
213546_at	hypothetical prote	DKFZP586I1420	7.09	7.18	7.22
218646_at	chromosome 4 op	C4orf27	10.01	10.15	10.14
208943_s_at	SEC62 homolog (S	SEC62	10.27	10.47	10.41
209142_s_at	ubiquitin-conjugat	UBE2G1	7.99	8.05	8.16
218849_s_at	protein phosphata	PPP1R13L	7.93	7.65	7.82
208816_x_at	annexin A2 pseud	ANXA2P2	13.05	13.12	12.95
211991_s_at	major histocompa	HLA-DPA1	4.54	4.71	4.66
220305_at	mitochondrial ant	MAVS	8.59	8.56	8.59
214945_at	family with seque	FAM153A /// FAM	4.24	4.24	3.98
219089_s_at	zinc finger proteir	ZNF576	8.31	8.37	8.33
202654_x_at	membrane-associ	7-Mar	8.24	8.20	8.53
204695_at	cell division cycle	CDC25A	7.98	7.95	8.05
203946_s_at	arginase, type II	ARG2	9.35	9.03	9.51
213774_s_at	---	---	5.38	5.40	5.31
220285_at	family with seque	FAM108B1	5.53	5.48	5.29
204447_at	ProSAPiP1 proteir	ProSAPiP1	7.44	7.67	7.60
212467_at	DnaJ (Hsp40) hom	DNAJC13	8.85	8.99	8.90
201802_at	solute carrier fam	SLC29A1	7.59	7.62	7.54
212409_s_at	torsin A interactin	TOR1AIP1	7.40	7.49	7.30
203737_s_at	peroxisome prolif	PPRC1	9.61	9.50	9.61
204602_at	dickkopf homolog	DKK1	7.80	8.46	8.00
208107_s_at	exonuclease NEF-s	LOC81691	8.77	8.59	8.69
217345_at	---	---	3.32	3.28	3.42
218529_at	CD320 molecule	CD320	9.40	9.26	9.35
218778_x_at	EPS8-like 1	EPS8L1	6.64	6.52	6.41
205807_s_at	tuftelin 1	TUFT1	9.70	9.53	9.73
207018_s_at	RAB27B, member	RAB27B	3.88	4.31	3.80
211027_s_at	inhibitor of kappa	IKBKB	5.39	5.27	5.35
211226_at	galanin receptor 2	GALR2	4.35	4.21	4.28
216162_at	---	---	3.37	3.61	3.51
217032_at	forkhead box D4 /	FOXD4 /// FOXD4L	5.76	5.64	5.56
218003_s_at	FK506 binding pro	FKBP3	11.45	11.43	11.45
213649_at	splicing factor, arg	SFRS7	9.39	9.45	9.44
213850_s_at	splicing factor, arg	SFRS2IP	10.42	10.59	10.50
220330_s_at	SAM domain, SH3	SAMSN1	9.90	9.99	10.15

219707_at	copine VII	CPNE7	7.73	7.69	7.79
203606_at	NADH dehydroger	NDUFS6	13.06	13.03	12.99
215807_s_at	plexin B1	PLXNB1	7.13	7.09	6.73
41577_at	protein phosphata	PPP1R16B	4.25	4.10	4.13
210250_x_at	adenylosuccinate	ADSL	11.67	11.63	11.69
211529_x_at	major histocompa	HLA-G	10.83	10.68	10.83
205407_at	reversion-inducing	RECK	6.71	6.86	6.64
33307_at	ribosomal RNA pr	RRP7A	9.25	9.11	9.07
204388_s_at	monoamine oxida	MAOA	8.95	9.02	9.04
214600_at	TEA domain famil	TEAD1	8.25	8.29	8.34
221002_s_at	tetraspanin 14	TSPAN14	8.77	8.96	8.72
202849_x_at	G protein-couplec	GRK6	8.62	8.60	8.41
213786_at	Tax1 (human T-ce	TAX1BP1	8.12	8.05	8.26
208937_s_at	inhibitor of DNA b	ID1	10.92	10.77	10.92
208365_s_at	G protein-couplec	GRK4	5.01	5.02	5.07
222369_at	N(alpha)-acetyltra	NAA40	8.22	8.49	8.42
208910_s_at	complement com	C1QBP	11.93	11.85	11.91
221876_at	zinc finger family	ZNF783	7.61	7.61	7.86
210463_x_at	TRM1 tRNA meth	TRMT1	7.89	7.93	7.88
204472_at	GTP binding prote	GEM	5.98	6.30	6.10
212190_at	serpin peptidase i	SERPINE2	9.61	9.44	9.63
205123_s_at	transmembrane p	TMEFF1	4.83	4.84	5.05
202499_s_at	solute carrier fam	SLC2A3	7.56	7.66	7.83
209075_s_at	iron-sulfur cluster	ISCU	11.32	11.37	11.35
213523_at	cyclin E1	CCNE1	7.80	7.74	7.37
216137_s_at	Mitogen-activatec	MAPK8IP3	6.41	6.48	6.30
220764_at	hypothetical LOC1	LOC100129064 ///	5.02	4.97	5.01
209303_at	NADH dehydroger	NDUFS4	10.73	10.66	10.78
217854_s_at	polymerase (RNA)	POLR2E	10.65	10.51	10.53
211148_s_at	angiopoietin 2	ANGPT2	5.34	5.21	5.03
210716_s_at	CAP-GLY domain c	CLIP1	7.59	7.62	7.90
201904_s_at	CTD (carboxy-tern	CTDSPL	8.34	8.22	8.32
211042_x_at	melanoma cell ad	MCAM	9.81	10.09	9.86
209165_at	apoptosis antagor	AATF	9.92	9.85	9.94
201197_at	adenosylmethioni	AMD1	10.39	10.42	10.55
206707_x_at	family with seque	FAM65B	8.82	8.63	8.80
203216_s_at	myosin VI	MYO6	9.50	9.48	9.58
209308_s_at	BCL2/adenovirus	BNIP2	9.89	9.99	10.14
211274_at	T-box 1	TBX1	4.82	4.80	4.63
36865_at	angel homolog 1 (	ANGEL1	7.75	7.67	7.82
38671_at	plexin D1	PLXND1	7.28	7.10	6.93
213676_at	transmembrane p	TMEM151B	6.28	6.28	6.45
206270_at	protein kinase C, g	PRKCG	6.52	6.57	6.65
215000_s_at	fasciculation and	FEZ2	11.12	11.11	11.23
203231_s_at	ataxin 1	ATXN1	6.10	6.11	6.17
221474_at	myosin, light chai	MYL12B	12.76	12.87	12.93
213414_s_at	ribosomal protein	RPS19	15.01	14.98	14.98

222299_x_at	---	---	3.26	3.17	3.09
211119_at	estrogen receptor	ESR2	3.76	3.70	3.76
203468_at	cyclin-dependent	CDK10	7.99	7.97	8.06
221994_at	PDZ and LIM dom	PDLIM5	5.43	5.27	5.35
219065_s_at	dpy-30 homolog (	DPY30 /// MEMO1	10.85	11.02	10.96
217705_at	protein kinase D1	PRKD1	3.82	3.91	3.71
206950_at	sodium channel, v	SCN9A	4.62	4.41	4.29
219758_at	tetratricopeptide	TTC26	7.35	7.57	7.50
215623_x_at	structural mainter	SMC4	7.71	7.64	7.65
210940_s_at	glutamate recepto	GRM1	5.14	4.96	4.82
208190_s_at	lipolysis stimulate	LSR	6.67	6.59	6.73
200935_at	calreticulin	CALR	7.26	7.34	7.34
211711_s_at	phosphatase and	PTEN	8.18	8.17	8.51
213831_at	major histocompa	HLA-DQA1	3.81	3.85	3.91
205457_at	chromosome 6 op	C6orf106	7.90	7.85	7.88
221017_s_at	leucine rich repea	LRRC3	4.42	4.53	4.57
55616_at	post-GPI attachm	PGAP3	7.55	7.57	7.56
206698_at	X-linked Kx blood	XK	5.01	5.28	5.29
212434_at	GrpE-like 1, mitoc	GRPEL1	9.96	9.82	9.90
205320_at	adenomatosis pol	APC2	6.41	6.36	6.29
204430_s_at	solute carrier fam	SLC2A5	5.97	6.18	6.06
213485_s_at	ATP-binding casse	ABCC10	8.64	8.73	8.76
220022_at	zinc finger protein	ZNF334	3.70	3.69	3.55
201260_s_at	synaptophysin-lik	SYPL1	10.34	10.37	10.54
216406_at	---	---	3.41	3.24	3.21
211982_x_at	exportin 6	XPO6	10.21	10.13	10.09
209602_s_at	GATA binding pro	GATA3	6.67	6.68	6.69
AFFX-r2-Bs-dap-	---	---	8.29	8.24	8.37
219239_s_at	zinc finger protein	ZNF654	6.34	6.49	6.17
216398_at	glycophorin B (MN	GYPB	5.42	5.47	5.56
211279_at	nuclear respirator	NRF1	6.88	7.00	6.63
210579_s_at	tripartite motif-co	TRIM10	5.42	5.34	5.21
211967_at	transmembrane p	TMEM123	12.67	12.75	12.88
218700_s_at	RAB7, member RA	RAB7L1	8.25	8.38	8.54
212052_s_at	TBC1 domain fam	TBC1D9B	8.10	8.30	8.30
211091_s_at	neurofibromin 2 (	NF2	6.23	6.17	6.14
207449_s_at	protein O-fucosylt	POFUT2	5.88	5.62	5.72
205398_s_at	SMAD family mem	SMAD3	8.16	8.25	8.13
206033_s_at	desmocollin 3	DSC3	3.37	3.41	3.42
221816_s_at	PHD finger protein	PHF11	9.52	9.48	9.52
211058_x_at	tubulin, alpha 1b	TUBA1B	14.50	14.55	14.49
217841_s_at	protein phosphata	PPME1	8.21	8.26	8.31
201531_at	zinc finger protein	ZFP36	8.55	8.62	8.61
207138_at	PHD finger protein	PHF2	5.34	5.15	5.22
214142_at	zymogen granule	ZG16	6.57	6.48	6.27
206005_s_at	KIAA1009	KIAA1009	6.30	6.58	6.46
201777_s_at	KIAA0494	KIAA0494	7.74	7.84	7.72

211704_s_at	spindlin family, m	SPIN2A /// SPIN2B	7.53	7.47	7.49
218475_at	TRM2 tRNA meth	TRMT2A	7.78	7.73	7.81
210628_x_at	latent transformir	LTBP4	6.46	6.55	6.51
205199_at	carbonic anhydras	CA9	6.24	5.90	5.92
218154_at	gasdermin D	GSDMD	8.15	7.97	7.97
203727_at	superkiller viralici	SKIV2L	8.53	8.46	8.51
215708_s_at	primase, DNA, pol	PRIM2	6.58	6.83	6.70
218703_at	SEC22 vesicle traf	SEC22A	6.94	6.99	7.10
201949_x_at	capping protein (a	CAPZB	10.05	10.02	10.06
206929_s_at	nuclear factor I/C	NFIC	8.84	8.80	8.76
201457_x_at	budding uninhibit	BUB3	11.77	11.81	11.87
204320_at	collagen, type XI,	COL11A1	4.00	4.11	4.14
205741_s_at	dystrobrevin, alph	DTNA	7.26	7.35	7.23
217137_x_at	hypothetical prote	LOC100289563	6.29	6.23	6.28
207822_at	fibroblast growth	FGFR1	5.83	5.67	5.57
209410_s_at	growth factor rec	GRB10	8.46	8.40	8.62
215470_at	general transcript	GTF2H2B	9.14	9.28	9.25
212291_at	homeodomain int	HIPK1	6.35	6.18	6.36
206000_at	meprin A, alpha (I	MEP1A	4.28	4.13	3.79
222164_at	fibroblast growth	FGFR1	6.91	6.79	7.13
208198_x_at	killer cell immuno	KIR2DS1	5.93	6.02	5.83
202195_s_at	transmembrane e	TMED5	7.74	7.82	7.87
212963_at	TM2 domain cont	TM2D1	5.04	4.84	5.10
205122_at	transmembrane p	TMEFF1	6.04	6.31	6.51
218439_s_at	COMM domain cc	COMMD10	9.69	9.56	9.68
208689_s_at	ribophorin II	RPN2	10.93	10.94	10.97
201596_x_at	keratin 18	KRT18	11.82	11.68	11.69
206533_at	cholinergic recept	CHRNA5	6.33	6.43	6.55
219826_at	zinc finger proteir	ZNF419	7.72	7.66	7.77
201872_s_at	ATP-binding casse	ABCE1	11.08	11.16	11.18
205022_s_at	forkhead box N3	FOXN3	10.29	10.16	10.36
200036_s_at	ribosomal protein	RPL10A	14.10	14.07	14.12
217889_s_at	cytochrome b red	CYBRD1	8.42	8.28	8.43
207549_x_at	CD46 molecule, cc	CD46	7.61	7.71	7.72
212219_at	proteasome (pros	PSME4	10.06	10.08	10.10
219073_s_at	oxysterol binding	OSBPL10	8.87	9.00	9.32
207199_at	telomerase revers	TERT	6.22	6.25	6.14
212683_at	solute carrier fam	SLC25A44	7.81	7.73	7.65
207986_x_at	cytochrome b-561	CYB561	9.24	9.25	9.18
221495_s_at	transcription facto	TCF25	9.25	9.20	9.20
210179_at	potassium inward	KCNJ13	4.48	4.29	4.33
202478_at	tribbles homolog	TRIB2	6.80	6.86	6.73
209275_s_at	ceroid-lipofuscino	CLN3	8.40	8.48	8.34
207972_at	glycine receptor,	GLRA1	4.51	4.39	4.38
209354_at	tumor necrosis fa	TNFRSF14	6.23	6.21	6.12
52255_s_at	collagen, type V,	COL5A3	6.80	6.75	6.68
214751_at	zinc finger proteir	ZNF468	6.54	6.83	6.71

201592_at	eukaryotic transla	EIF3H	12.93	12.89	12.94
204670_x_at	major histocompa	HLA-DRB1 /// HLA-	6.35	6.23	6.03
215491_at	v-myc myelocytor	MYCL1	5.60	5.56	5.28
218064_s_at	A kinase (PRKA) ar	AKAP8L	7.22	7.26	7.26
217473_x_at	solute carrier fam	SLC11A1	7.40	7.41	7.23
212234_at	additional sex con	ASXL1	9.34	9.20	9.26
204482_at	claudin 5	CLDN5	6.95	6.68	6.60
32541_at	protein phosphata	PPP3CC	8.29	8.17	8.41
214223_at	---	---	7.17	7.22	7.29
203441_s_at	cadherin 2, type 1	CDH2	8.05	8.07	8.28
220390_at	ATP/GTP binding p	AGBL2	4.55	4.47	4.40
201040_at	guanine nucleotid	GNAI2	8.85	8.72	8.68
220930_s_at	hypothetical prote	MGC5590	3.56	3.56	3.60
203524_s_at	mercaptopyruvate	MPST	9.02	8.99	9.05
207794_at	chemokine (C-C m	CCR2	3.60	3.57	3.57
219865_at	hypothetical LOC2	HSPC157	9.39	9.31	9.47
200765_x_at	catenin (cadherin-	CTNNA1	11.12	11.06	11.11
208375_at	interferon, alpha 1	IFNA1	3.43	3.48	3.47
203257_s_at	chromosome 11 o	C11orf49	8.76	8.58	8.63
214041_x_at	Ribosomal proteir	RPL37A	9.09	9.24	9.43
208701_at	Amyloid beta (A4)	APLP2	4.43	4.35	4.40
216237_s_at	minichromosome	MCM5	8.84	8.95	8.94
217613_at	transmembrane p	TMEM144	3.43	3.44	3.63
213032_at	nuclear factor I/B	NFIB	9.21	9.13	9.25
1294_at	ubiquitin-like moc	UBA7	6.58	6.49	6.66
208028_s_at	glutathione perox	GPX5	3.86	3.79	3.64
216355_at	protocadherin bet	PCDHB17	4.88	4.99	4.83
203503_s_at	peroxisomal bioge	PEX14	7.23	7.39	7.09
210553_x_at	proprotein conver	PCSK6	6.26	6.35	6.25
204341_at	tripartite motif-co	TRIM16	7.60	7.54	7.67
207982_at	histone cluster 1,	HIST1H1T	6.35	6.24	6.15
206992_s_at	ATP synthase, H+	ATP5S	8.11	8.07	7.93
202185_at	procollagen-lysine	PLOD3	9.87	9.73	9.76
210151_s_at	dual-specificity ty	DYRK3	7.84	8.01	7.85
212361_s_at	ATPase, Ca++ tran	ATP2A2	10.13	10.17	10.11
201338_x_at	general transcript	GTF3A	12.76	12.74	12.86
202758_s_at	regulatory factor X	RFXANK	7.87	7.91	8.03
204882_at	Rho GTPase activa	ARHGAP25	5.32	4.94	4.91
208727_s_at	cell division cycle	CDC42	10.58	10.58	10.52
220773_s_at	gephyrin	GPHN	6.95	6.86	7.07
203516_at	syntrophin, alpha	SNTA1	7.73	7.69	7.66
201201_at	cystatin B (stefin B	CSTB	12.31	12.30	12.26
217569_x_at	---	---	3.72	3.99	3.95
205339_at	SCL/TAL1 interrup	STIL	9.47	9.50	9.68
212655_at	zinc finger, CCHC c	ZCCHC14	8.60	8.76	8.65
218683_at	polypyrimidine tra	PTBP2	7.82	8.07	8.02
206732_at	SLIT and NTRK-like	SLITRK3	3.81	3.79	4.00

221367_at	v-mos Moloney m	MOS	5.02	4.90	4.81
212688_at	phosphoinositide-	PIK3CB	9.58	9.49	9.63
213872_at	Chromosome 6 op	C6orf62	11.15	11.14	11.21
206276_at	lymphocyte antige	LY6D	5.53	5.55	5.51
215637_at	testis specific, 14	TSGA14	4.03	4.33	4.13
221508_at	TAO kinase 3	TAOK3	6.94	6.82	6.93
201072_s_at	SWI/SNF related,	SMARCC1	10.88	10.89	10.81
202320_at	general transcript	GTF3C1	8.08	8.19	8.13
215598_at	Tetratricopeptide	TTC12	5.62	5.49	5.51
213503_x_at	annexin A2	ANXA2	14.30	14.33	14.30
204029_at	cadherin, EGF LAG	CELSR2	7.03	7.04	6.92
206351_s_at	peroxisomal bioge	PEX10	6.13	6.10	6.30
212913_at	chromosome 6 op	C6orf26	8.02	8.29	8.06
208795_s_at	minichromosome	MCM7	10.00	10.06	10.04
219603_s_at	zinc finger protein	ZNF226	7.06	7.38	7.46
212113_at	ataxin 7-like 3B	ATXN7L3B	8.36	8.44	8.42
216613_at	---	---	3.14	3.22	3.14
214869_x_at	GTPase activating	GAPVD1	8.19	8.42	8.36
213454_at	apoptosis-inducin	APITD1	9.45	9.56	9.52
219442_at	KIAA0664 pseudo	KIAA0664P3	7.14	7.20	7.00
209665_at	cytochrome b-561	CYB561D2	8.83	8.85	8.82
217295_at	mucin 8	MUC8	5.02	4.93	4.95
205707_at	interleukin 17 rec	IL17RA	8.54	8.52	8.26
207735_at	ring finger protein	RNF125	5.84	5.69	5.57
211124_s_at	KIT ligand	KITLG	3.84	3.92	3.88
203838_s_at	tyrosine kinase, no	TNK2	4.70	4.70	4.61
212235_at	plexin D1	PLXND1	5.93	5.23	5.74
213361_at	tudor domain con	TDRD7	8.09	8.24	8.25
208314_at	retinal pigment ep	RRH	4.36	4.44	4.32
200766_at	cathepsin D	CTSD	7.95	7.72	7.74
216880_at	RAD51-like 1 (S. ce	RAD51L1	5.27	5.44	5.55
218137_s_at	small ArfGAP 1	SMAP1	9.43	9.47	9.61
201861_s_at	leucine rich repea	LRRFIP1	9.52	9.59	9.66
209408_at	kinesin family mer	KIF2C	10.46	10.44	10.48
202720_at	testis derived tran	TES	3.51	3.59	4.11
215938_s_at	phospholipase A2	PLA2G6	6.69	6.61	6.54
218414_s_at	nudE nuclear distr	NDE1	9.29	9.13	9.20
214196_s_at	tripeptidyl peptida	TPP1	5.15	4.84	5.15
203629_s_at	component of olig	COG5	7.05	6.92	7.17
201620_at	membrane-bound	MBTPS1	9.08	9.11	9.14
218131_s_at	GATA zinc finger d	GATAD2A	9.36	9.34	9.40
216058_s_at	cytochrome P450,	CYP2C19	3.77	3.57	3.84
209241_x_at	misshapen-like kin	MINK1	7.12	6.97	7.14
212414_s_at	glyoxylate reducta	GLYR1 /// SEPT6	6.19	6.39	6.19
217325_at	keratin 3	KRT3	5.62	5.51	5.40
205088_at	mastermind-like d	MAMLD1	7.53	7.57	7.66
209391_at	dolichyl-phosphat	DPM2	8.83	8.80	8.83

203496_s_at	mediator comple›	MED1	9.46	9.49	9.58
200797_s_at	myeloid cell leuke	MCL1	12.41	12.46	12.42
201886_at	DDB1 and CUL4 a:	DCAF11	7.89	7.89	7.92
200640_at	tyrosine 3-monoo	YWHAZ	12.13	12.25	12.19
AFFX-DapX-5_at	---	---	7.32	7.21	7.22
218397_at	Fanconi anemia, c	FANCL	8.94	9.14	9.19
221497_x_at	egl nine homolog	EGLN1	8.37	8.34	8.47
205575_at	complement com	C1QL1	7.90	7.87	7.62
215770_at	olfactory recepto	OR7E87P	3.52	3.35	3.24
210561_s_at	WD repeat and SC	WSB1	9.61	9.84	9.88
214763_at	acyl-CoA thioeste	ACOT11	7.23	7.12	7.11
214304_x_at	synemin, interme	SYNM	4.15	3.95	4.10
219909_at	matrix metallope	MMP28	4.48	4.27	4.41
212208_at	mediator comple›	MED13L	9.85	9.69	9.80
201237_at	capping protein (a	CAPZA2	10.57	10.66	10.75
203206_at	family with seque	FAM53B	7.96	7.93	7.83
52169_at	STE20-related kin	STRADA	9.39	9.36	9.35
207754_at	Ras association (R	RASSF8	5.44	5.35	5.51
221445_at	olfactory recepto	OR1A2	3.66	3.58	3.36
201081_s_at	phosphatidylinosi	PIP4K2B	7.29	7.42	7.28
218348_s_at	zinc finger CCCH-t	ZC3H7A	9.70	9.61	9.67
202533_s_at	dihydrofolate red	DHFR	8.22	8.05	8.15
221485_at	UDP-Gal:betaGlcN	B4GALT5	9.71	9.67	9.74
203008_x_at	thioredoxin doma	TXNDC9	9.95	9.99	10.13
203469_s_at	cyclin-dependent	CDK10	7.59	7.64	7.59
218988_at	solute carrier fam	SLC35E3	8.90	8.77	8.79
211505_s_at	staufen, RNA bind	STAU1	8.57	8.62	8.75
203899_s_at	CGRP receptor co	CRCP	8.40	8.17	8.09
210815_s_at	calcitonin recepto	CALCRL	4.57	4.44	4.48
217799_x_at	ubiquitin-conjuga	UBE2H	9.06	8.97	8.96
203281_s_at	ubiquitin-like moc	UBA7	6.34	6.17	6.33
221226_s_at	amiloride-sensitiv	ACCN4	6.75	6.78	6.82
213480_at	vesicle-associated	VAMP4	8.86	8.94	9.10
216931_at	---	---	4.20	4.18	4.30
218761_at	ring finger proteir	RNF111	9.15	9.14	9.27
201396_s_at	small glutamine-ri	SGTA	8.50	8.45	8.26
200752_s_at	calpain 1, (mu/I) l	CAPN1	7.75	7.70	7.52
218780_at	hook homolog 2 (	HOOK2	7.53	7.65	7.45
219147_s_at	chromosome 9 op	C9orf95	7.34	7.63	7.68
202058_s_at	karyopherin alpha	KPNA1	7.47	7.51	7.43
219076_s_at	peroxisomal mem	PXMP2	9.71	9.71	9.71
206324_s_at	death-associated	DAPK2	6.17	6.01	6.05
203794_at	CDC42 binding pr	CDC42BPA	9.55	9.37	9.50
218730_s_at	osteoglycin	OGN	3.46	3.36	3.43
210931_at	ring finger proteir	RNF6	3.15	3.30	3.38
202951_at	serine/threonine I	STK38	9.12	9.03	9.12
202079_s_at	trafficking protein	TRAK1	6.42	6.46	6.37

206283_s_at	T-cell acute lymph	TAL1		4.10	3.97	4.08
204855_at	serpin peptidase i	SERPINB5		8.99	9.22	9.10
202346_at	ubiquitin-conjugat	UBE2K		9.28	9.32	9.35
207476_at	---	---		6.65	6.46	6.40
203910_at	Rho GTPase activa	ARHGAP29		7.79	8.25	8.03
216491_x_at	immunoglobulin h	IGHM		5.33	5.03	4.93
206164_at	chloride channel a	CLCA2		3.61	3.63	3.50
202265_at	BMI1 polycomb ri	BMI1		11.35	11.39	11.61
204966_at	brain-specific angi	BAI2		6.01	5.59	5.80
203035_s_at	protein inhibitor c	PIAS3		8.43	8.58	8.54
215258_at	cell adhesion mol	CADM4		5.63	5.39	5.38
204427_s_at	transmembrane e	TMED2		9.93	10.01	10.17
201055_s_at	heterogeneous nu	HNRNPA0		8.80	8.73	8.86
201323_at	EBNA1 binding pr	EBNA1BP2		12.23	12.20	12.29
216352_x_at	protocadherin gar	PCDHGA3		4.84	4.88	4.67
222042_x_at	Mex-3 homolog D	MEX3D		6.52	6.22	6.39
216038_x_at	death-domain ass	DAXX		7.92	7.86	7.86
221533_at	family with seque	FAM162A		5.18	5.07	5.01
211168_s_at	UPF1 regulator of	UPF1		8.83	8.75	8.81
201942_s_at	carboxypeptidase	CPD		6.07	5.82	5.90
204619_s_at	versican	VCAN		6.20	6.39	6.50
219867_at	chondrolectin	CHODL		5.08	5.16	5.32
219443_at	taspase, threonin	TASP1		8.07	8.21	8.17
221282_x_at	runt-related trans	RUNX2		5.96	6.02	6.10
217774_s_at	tRNA methyltrans	TRMT112		12.60	12.67	12.70
210449_x_at	mitogen-activatec	MAPK14		7.04	6.86	6.85
216153_x_at	reversion-inducing	RECK		6.93	7.04	7.00
213231_at	dystrophia myoto	DMWD		5.35	5.27	5.31
215029_at	---	---		5.12	5.32	5.15
219696_at	DENN/MADD dom	DENND1B		8.11	8.14	8.41
214051_at	thymosin beta 15l	TMSB15B		8.53	8.57	8.59
201307_at	septin 11		11-Sep	10.44	10.42	10.46
200087_s_at	transmembrane e	TMED2		12.49	12.58	12.64
220874_at	---	---		4.17	4.10	3.92
213387_at	ATPase family, AA	ATAD2B		8.01	8.17	8.23
213322_at	chromosome 6 op	C6orf130		9.60	9.61	9.76
207921_x_at	paired box 8	PAX8		6.20	6.10	6.22
216331_at	integrin, alpha 7	ITGA7		6.60	6.61	6.72
221540_x_at	general transcript	GTF2H2 /// GTF2H		9.05	9.23	9.14
219786_at	metallothionein-li	MTL5		6.42	6.33	5.92
207398_at	homeobox D13	HOXD13		5.53	5.50	5.54
208278_s_at	---	---		6.39	6.15	6.18
202083_s_at	SEC14-like 1 (S. ce	SEC14L1		9.39	9.37	9.61
208537_at	sphingosine-1-pho	S1PR2		6.00	5.94	5.90
201466_s_at	jun oncogene	JUN		8.81	8.84	8.79
212465_at	SET domain conta	SETD3		10.32	10.17	10.25
201155_s_at	mitofusin 2	MFN2		9.68	9.72	9.58

AFFX-hum_alu_a	---	---	15.23	15.25	15.20
208423_s_at	macrophage scave	MSR1	4.14	3.90	3.80
202762_at	Rho-associated, co	ROCK2	9.49	9.68	9.65
207958_at	UDP glucuronosyl	UGT2A1 /// UGT2A	4.60	4.60	4.41
219567_s_at	defects in morpho	DEM1	3.56	3.30	3.53
218411_s_at	MAP3K12 binding	MBIP	7.71	7.91	8.45
217793_at	RAB11B, member	RAB11B	6.19	6.06	6.25
202970_at	dual-specificity ty	DYRK2	7.21	7.24	7.15
209119_x_at	nuclear receptor s	NR2F2	6.61	6.55	6.36
221674_s_at	chordin	CHRD	6.29	6.02	6.10
204995_at	cyclin-dependent	CDK5R1	6.83	6.87	7.22
201678_s_at	chromosome 3 op	C3orf37	9.63	9.77	9.68
220342_x_at	ER degradation er	EDEM3	8.02	8.22	8.02
221206_at	PMS2 postmeiotic	PMS2 /// PMS2CL	8.45	8.41	8.49
204164_at	signal-induced pro	SIPA1	7.60	7.28	7.21
217121_at	tankyrase, TRF1-in	TNKS	4.91	4.72	4.67
208548_at	interferon, alpha (	IFNA6	4.37	4.35	4.14
201446_s_at	TIA1 cytotoxic gra	TIA1	8.42	8.61	8.61
202361_at	SEC24 family, mer	SEC24C	8.28	8.40	8.38
202800_at	E2F transcription f	E2F1	9.80	9.65	9.77
221323_at	UL16 binding prot	ULBP1	4.74	4.58	4.60
208145_at	---	---	5.86	5.63	5.60
215240_at	integrin, beta 3 (p	ITGB3	3.71	3.52	3.56
207346_at	syntaxin 2	STX2	7.50	7.33	7.33
200029_at	ribosomal protein	RPL19	14.33	14.30	14.36
219325_s_at	elaC homolog 1 (E	ELAC1	4.97	5.08	5.35
217892_s_at	LIM domain and a	LIMA1	11.20	11.30	11.30
204621_s_at	nuclear receptor s	NR4A2	6.71	6.54	6.64
208930_s_at	interleukin enhan	ILF3	10.18	10.12	10.14
203500_at	glutaryl-CoA dehy	GCDH	8.47	8.38	8.46
212136_at	ATPase, Ca++ tran	ATP2B4	9.28	9.36	9.36
208789_at	polymerase I and	PTRF	11.37	11.35	11.29
201635_s_at	fragile X mental re	FXR1	9.68	9.63	9.67
201976_s_at	myosin X	MYO10	9.98	10.00	10.07
219985_at	heparan sulfate (g	HS3ST3A1	8.31	7.99	8.39
212828_at	synaptojanin 2	SYNJ2	9.04	9.10	9.19
203919_at	transcription elon	TCEA2	7.16	7.08	7.25
212498_at	---	---	10.79	10.71	10.70
217926_at	chromosome 19 o	C19orf53	10.10	10.08	10.02
217974_at	transmembrane 7	TM7SF3	7.77	7.79	7.83
202960_s_at	methylmalonyl Co	MUT	10.19	10.32	10.38
221797_at	chromosome 17 o	C17orf90	9.20	9.02	9.07
221727_at	SUB1 homolog (S.	SUB1	10.77	10.67	10.81
51228_at	RNA binding moti	RBM12B	6.98	7.07	7.06
209286_at	CDC42 effector pr	CDC42EP3	8.69	8.84	9.05
214276_at	Kruppel-like facto	KLF12	6.99	6.88	7.01
208947_s_at	UPF1 regulator of	UPF1	7.57	7.64	7.33

204975_at	epithelial membra	EMP2	5.52	5.88	5.24
205449_at	SAC3 domain cont	SAC3D1	10.10	10.03	10.16
218035_s_at	RNA binding moti	RBM47	5.00	5.37	4.91
209821_at	interleukin 33	IL33	3.30	3.28	3.22
219692_at	kringle containing	KREMEN2	7.63	7.44	7.20
203704_s_at	ras responsive ele	RREB1	9.01	8.87	9.11
39548_at	neuronal PAS dom	NPAS2	3.96	4.07	3.92
202170_s_at	aminoadipate-sen	AASDHPPT	8.60	8.68	8.82
201862_s_at	leucine rich repea	LRRFIP1	9.32	9.43	9.49
210655_s_at	forkhead box O3 /	FOXO3 /// FOXO3I	7.68	7.69	7.77
209091_s_at	SH3-domain GRB2	SH3GLB1	10.32	10.33	10.36
207936_x_at	ret finger protein-	RFPL3	5.17	5.44	5.32
202438_x_at	iduronate 2-sulfat	IDS	4.71	4.67	4.53
217118_s_at	chromosome 22 c	C22orf9	9.29	9.18	9.17
209009_at	esterase D	ESD	11.99	11.99	12.01
216023_at	lysine (K)-specific	KDM4B	6.79	6.89	6.62
205441_at	occludin/ELL dom	OCEL1	8.61	8.48	8.48
211715_s_at	3-hydroxybutyrat	BDH1	8.36	8.47	8.40
210025_s_at	caspase recruitme	CARD10	7.32	7.12	7.02
204310_s_at	natriuretic peptid	NPR2	4.16	3.98	3.88
204680_s_at	Rap guanine nucle	RAPGEF5	4.19	4.15	3.93
203873_at	SWI/SNF related,	SMARCA1	3.56	3.65	3.56
213198_at	activin A receptor	ACVR1B	8.15	8.35	8.23
219185_at	sirtuin (silent mat	SIRT5	8.40	8.38	8.50
211397_x_at	killer cell immuno	KIR2DL2	5.31	5.45	5.20
205682_x_at	apolipoprotein M	APOM	6.40	6.42	6.54
221019_s_at	collectin sub-fami	COLEC12	4.46	4.28	4.22
201667_at	gap junction prote	GJA1	11.35	11.36	11.53
215686_x_at	transcription facto	TFAP2B	3.69	3.86	3.60
205456_at	CD3e molecule, e	CD3E	6.31	6.13	6.41
205813_s_at	methionine adeno	MAT1A	6.10	5.92	6.24
204871_at	mitochondrial tra	MTERF	7.17	7.29	7.11
220052_s_at	TERF1 (TRF1)-inte	TINF2	7.29	7.24	7.35
219652_s_at	chromosome X op	CXorf36	5.70	5.77	5.67
221163_s_at	MLX interacting p	MLXIPL	7.42	7.01	7.01
202189_x_at	polypyrimidine tra	PTBP1	11.31	11.27	11.28
212098_at	hypothetical LOC1	LOC151162 /// MC	9.81	9.79	9.67
208749_x_at	flotillin 1	FLOT1	9.63	9.57	9.52
201016_at	eukaryotic transla	EIF1AX	10.02	10.20	10.41
202923_s_at	glutamate-cystein	GCLC	8.62	8.62	8.82
58696_at	exosome compon	EXOSC4	9.67	9.55	9.66
218409_s_at	DnaJ (Hsp40) hom	DNAJC1	9.44	9.30	9.50
209712_at	solute carrier fam	SLC35D1	7.37	7.34	7.49
218505_at	WD repeat domai	WDR59	8.75	8.90	8.71
201059_at	cortactin	CTTN	11.29	11.31	11.29
200668_s_at	ubiquitin-conjuga	UBE2D3	12.17	12.28	12.31
204556_s_at	DAZ interacting pr	DZIP1	8.93	8.94	8.92

204846_at	ceruloplasmin (fe	CP	3.20	3.45	3.55
203945_at	arginase, type II	ARG2	6.63	6.44	6.39
203765_at	grancalcin, EF-han	GCA	6.69	7.02	7.08
32032_at	3-phosphoinositid	PDPK1	8.90	8.86	8.81
215859_at	nicalin homolog (z	NCLN	5.12	5.14	4.91
205481_at	adenosine A1 rece	ADORA1	4.88	4.55	4.72
213134_x_at	BTG family, memb	BTG3	10.69	10.62	10.73
204007_at	Fc fragment of IgG	FCGR3B	3.91	3.90	4.11
208699_x_at	transketolase	TKT	11.14	11.00	11.14
201923_at	peroxiredoxin 4	PRDX4	12.46	12.45	12.51
209906_at	complement com	C3AR1	5.43	5.03	4.77
219586_at	WD repeat domai	WDR59	4.56	4.31	4.70
215399_s_at	osteosarcoma am	OS9	7.85	7.90	7.86
213631_x_at	dihydroorotate de	DHODH	5.57	5.74	5.57
217944_at	protein O-linked n	POMGNT1	8.26	8.18	8.14
212495_at	lysine (K)-specific	KDM4B	7.38	7.21	7.30
217775_s_at	retinol dehydroge	RDH11	10.75	10.84	10.89
205880_at	protein kinase D1	PRKD1	8.40	8.40	8.56
215856_at	sialic acid binding	SIGLEC15	5.62	5.83	5.84
212263_at	quaking homolog,	QKI	11.42	11.28	11.45
214837_at	---	---	4.36	4.18	4.12
220212_s_at	thyroid adenoma	THADA	8.51	8.55	8.63
208191_x_at	pregnancy specific	PSG4	8.52	8.46	8.41
212258_s_at	SWI/SNF related,	SMARCA2	6.38	6.18	6.26
215217_at	Immunoglobulin k	IGKC	6.05	5.96	5.87
216000_at	---	---	4.35	4.18	4.10
219986_s_at	acyl-CoA dehydro	ACAD10	7.64	7.48	7.52
209768_s_at	glycoprotein Ib (p	GP1BB /// SEPT5	6.83	6.61	6.67
219774_at	coiled-coil domair	CCDC93	6.97	6.89	6.90
214272_at	cylindromatosis (t	CYLD	6.45	6.20	6.24
206900_x_at	zinc finger proteir	ZNF253	4.68	4.69	4.58
205500_at	complement com	C5	6.35	6.50	6.50
202216_x_at	nuclear transcript	NFYC	8.12	8.15	8.10
210457_x_at	high mobility grou	HMGA1	7.25	7.08	7.18
220164_s_at	F-box protein 40	FBXO40	4.71	4.57	4.66
215670_s_at	SCAN domain con	SCAND2	7.11	6.96	7.24
207693_at	calcium channel, v	CACNB4	4.61	4.82	4.30
217397_at	T cell receptor del	TRD@	4.29	4.32	4.11
212243_at	GRINL1A complex	GCOM1 /// GRINL1	9.56	9.57	9.73
210379_s_at	tousled-like kinase	TLK1	6.43	6.45	6.74
217647_at	---	---	7.03	6.76	6.78
201887_at	interleukin 13 rec	IL13RA1	9.02	9.02	9.11
219783_at	chromosome 2 op	C2orf18	7.48	7.51	7.59
200681_at	glyoxalase I	GLO1	12.30	12.37	12.38
212312_at	BCL2-like 1	BCL2L1	9.88	9.72	9.77
204208_at	RNA guanylyltrans	RNGTT	8.94	8.97	8.96
203042_at	lysosomal-associa	LAMP2	8.73	8.78	8.84

203599_s_at	WW domain bindi	WBP4	8.62	8.73	8.87
211270_x_at	polypyrimidine tra	PTBP1	11.36	11.25	11.31
209594_x_at	pregnancy specific	PSG9	5.38	5.49	5.46
219533_at	cyclin-dependent	CDKN1C	5.23	5.24	5.25
221809_at	RAN binding prote	RANBP10	7.18	7.31	7.14
213192_at	THAP domain con	THAP3	8.16	8.04	7.97
218319_at	pellino homolog 1	PELI1	7.33	7.24	7.51
214388_at	---	---	4.22	4.41	4.33
201577_at	non-metastatic ce	NME1	13.30	13.24	13.26
220295_x_at	DEP domain conta	DEPDC1	10.65	10.79	10.68
219321_at	membrane protei	MPP5	8.17	8.38	8.29
220459_at	MCM3AP antisens	MCM3APAS	7.83	8.06	8.05
214249_at	tripartite motif-co	TRIM2	4.32	4.47	4.26
218539_at	F-box protein 34	FBXO34	9.51	9.49	9.60
211417_x_at	gamma-glutamylt	GGT1	7.80	7.59	7.46
208003_s_at	nuclear factor of a	NFAT5	8.28	8.22	8.31
204429_s_at	solute carrier fam	SLC2A5	5.68	5.88	5.94
208433_s_at	low density lipopr	LRP8	9.08	9.30	9.23
210302_s_at	mab-21-like 2 (C. e	MAB21L2	3.93	3.87	3.83
212797_at	sortilin 1	SORT1	4.28	4.31	4.64
204527_at	myosin VA (heavy	MYO5A	4.51	4.52	4.24
221265_s_at	chromosome 15 o	C15orf44	9.34	9.38	9.46
218395_at	ARP6 actin-relate	ACTR6	9.48	9.43	9.65
204823_at	neuron navigator	NAV3	9.52	9.50	9.57
201692_at	sigma non-opioid	SIGMAR1	9.25	9.12	9.20
219657_s_at	Kruppel-like facto	KLF3	7.06	6.70	6.81
212694_s_at	propionyl CoA car	PCCB	10.00	10.01	10.05
209086_x_at	melanoma cell ad	MCAM	7.57	7.53	7.59
200904_at	major histocompa	HLA-E	7.97	8.09	8.11
202661_at	inositol 1,4,5-trip	ITPR2	4.94	4.97	4.87
221631_at	calcium channel, v	CACNA1I	4.67	4.58	4.81
213887_s_at	polymerase (RNA)	POLR2E	10.16	10.04	10.06
212009_s_at	stress-induced-ph	STIP1	7.28	7.34	7.35
214583_at	DNA-damage indu	DDI2 /// RSC1A1	6.41	6.42	6.50
219812_at	poliovirus recepto	PVRIG	4.78	4.80	4.78
203363_s_at	KIAA0652	KIAA0652	7.16	7.24	7.30
220173_at	chromosome 14 o	C14orf45	5.85	6.04	5.83
215593_at	---	---	4.62	4.45	4.35
200945_s_at	SEC31 homolog A	SEC31A	11.24	11.18	11.34
220744_s_at	intraflagellar tran	IFT122	7.43	7.44	7.30
210999_s_at	growth factor rec	GRB10	9.07	8.90	9.06
218498_s_at	ERO1-like (S. cere	ERO1L	7.91	8.10	8.10
213104_at	chromosome 16 o	C16orf42	8.82	8.79	8.93
221084_at	5-hydroxytryptam	HTR3B	4.85	4.78	4.66
201542_at	SAR1 homolog A (	SAR1A	11.21	11.28	11.21
212590_at	related RAS viral (	RRAS2	10.58	10.59	10.71
216712_at	solute carrier fam	SLC25A30	3.25	3.08	3.26

53076_at	xylosylprotein bet	B4GALT7	7.50	7.51	7.57
218036_x_at	NMD3 homolog (S	NMD3	9.14	9.21	9.23
209615_s_at	p21 protein (Cdc4	PAK1	5.78	6.10	5.98
212759_s_at	transcription facto	TCF7L2	7.28	7.11	7.22
221242_at	---	---	5.95	5.92	5.95
219755_at	chromobox homo	CBX8	5.95	6.06	5.99
201693_s_at	early growth resp	EGR1	6.60	6.90	6.40
218259_at	MKL/myocardin-li	MKL2	9.38	9.32	9.44
217083_at	---	---	4.51	4.40	4.35
219993_at	SRY (sex determin	SOX17	3.48	3.47	3.38
209983_s_at	neurexin 2	NRXN2	5.01	4.95	4.76
219596_at	THAP domain con	THAP10	8.63	8.66	8.87
212716_s_at	eukaryotic transla	EIF3K	12.09	12.12	12.17
208027_s_at	tolloid-like 2	TLL2	4.30	4.17	4.08
222000_at	chromosome 1 op	C1orf174	10.35	10.30	10.22
215966_x_at	glycerol kinase 3 p	GK3P	5.36	5.25	5.07
212419_at	zinc finger, CCHC d	ZCCHC24	8.58	8.60	8.41
219476_at	chromosome 1 op	C1orf116	4.60	4.58	4.65
201161_s_at	cold shock domain	CSDA	12.27	12.05	12.05
201225_s_at	serine/arginine re	SRRM1	11.15	11.22	11.24
207387_s_at	glycerol kinase	GK	4.89	4.66	4.83
200816_s_at	platelet-activating	PAFAH1B1	9.59	9.61	9.70
336_at	GM2 ganglioside a	GM2A	6.23	6.05	5.94
220944_at	peptidoglycan rec	PGLYRP4	5.85	5.66	5.48
204426_at	transmembrane e	TMED2	8.30	8.38	8.62
214526_x_at	postmeiotic segre	PMS2L1	8.01	8.13	8.22
221950_at	empty spiracles h	EMX2	7.61	7.43	7.87
220589_s_at	integrin alpha FG-	ITFG2	6.27	6.15	6.19
217773_s_at	NADH dehydrogen	NDUFA4	13.09	13.13	13.11
202813_at	glucosidase, alpha	GAA	9.07	9.15	9.06
217619_x_at	---	---	7.74	7.75	7.63
204107_at	nuclear transcript	NFYA	7.08	7.08	6.99
211784_s_at	splicing factor, arg	SFRS1	12.54	12.56	12.62
207045_at	coiled-coil domain	CCDC132	4.42	4.49	4.29
218384_at	calcium regulated	CARHSP1	9.71	9.61	9.53
201386_s_at	DEAH (Asp-Glu-Ala	DHX15	10.92	11.05	10.95
212478_at	required for meio	RMND5A	7.83	7.80	7.80
201331_s_at	signal transducer	STAT6	9.53	9.57	9.49
202240_at	polo-like kinase 1	PLK1	7.84	7.90	7.62
202254_at	Signal-induced pro	SIPA1L1	7.07	6.95	7.08
203188_at	UDP-GlcNAc:beta	B3GNT1	8.67	8.87	8.75
221921_s_at	cell adhesion mol	CADM3	6.64	6.42	6.70
201849_at	BCL2/adenovirus	BNIP3	11.48	11.51	11.55
208464_at	glutamate recepto	GRIA4	4.62	4.50	4.33
215539_at	---	---	3.52	3.61	3.49
219257_s_at	sphingosine kinas	SPHK1	7.15	7.30	7.32
214723_x_at	ankyrin repeat do	ANKRD36	6.59	6.92	6.72

210201_x_at	bridging integrato	BIN1	8.74	8.72	8.60
218118_s_at	translocase of inn	LOC10431 /// TIM	10.53	10.64	10.67
203842_s_at	microtubule-asso	MAPRE3	7.63	7.42	7.61
204291_at	zinc finger protein	ZNF518A	7.46	7.65	8.00
33760_at	peroxisomal bioge	PEX14	8.31	8.31	8.30
201143_s_at	eukaryotic transla	EIF2S1	10.81	10.84	10.78
203076_s_at	SMAD family men	SMAD2	9.86	9.76	9.92
218674_at	chromosome 5 op	C5orf44	8.04	8.07	8.41
212445_s_at	neural precursor c	NEDD4L	8.18	7.97	8.12
203767_s_at	steroid sulfatase (	STS	3.74	3.98	3.94
202253_s_at	dynamin 2	DNM2	7.51	7.46	7.41
200593_s_at	heterogeneous nu	HNRNPU	11.18	11.23	11.27
216715_at	---	---	6.93	6.83	6.92
213206_at	golgi SNAP recept	GOSR2	6.80	6.63	7.00
217266_at	ribosomal protein	RPL15	10.44	10.29	10.45
212546_s_at	FRY-like	FRYL	7.95	8.09	8.20
210859_x_at	ceroid-lipofuscino	CLN3	7.43	7.57	7.48
34868_at	Smg-5 homolog, r	SMG5	8.51	8.33	8.48
200834_s_at	similar to ribosom	LOC100291837 ///	14.96	14.94	14.94
201062_at	stomatin	STOM	3.03	3.15	3.14
220839_at	methyltransferase	METTL5	5.10	5.09	5.11
203893_at	TAF9 RNA polyme	TAF9	11.56	11.50	11.74
209034_at	proline-rich nucle	PNRC1	10.15	9.89	10.26
213060_s_at	chitinase 3-like 2	CHI3L2	5.39	4.89	5.06
209744_x_at	itchy E3 ubiquitin	ITCH	8.90	8.87	8.79
211700_s_at	trophinin	TRO	5.83	5.93	5.68
201776_s_at	KIAA0494	KIAA0494	8.09	8.16	8.18
213284_at	zinc finger protein	ZFP36L1	4.90	4.84	4.74
203762_s_at	dynein, cytoplasm	DYNC2LI1	7.14	7.29	7.53
35201_at	heterogeneous nu	HNRNPL	11.16	11.14	11.14
201564_s_at	fascin homolog 1,	FSCN1	9.35	9.40	9.26
221394_at	trace amine assoc	TAAR2	3.84	3.93	3.91
209136_s_at	ubiquitin specific		USP10	8.50	8.59	8.59
220512_at	deleted in liver ca	DLC1	6.46	6.47	6.47
202615_at	Guanine nucleotic	GNAQ	10.80	10.75	10.94
222239_s_at	integrator comple	INTS6	8.94	8.86	9.11
202498_s_at	solute carrier fam	SLC2A3	6.03	5.88	6.23
202434_s_at	cytochrome P450,	CYP1B1	5.03	5.15	4.90
210326_at	alanine-glyoxylate	AGXT	5.16	4.97	5.00
216154_at	---	---	4.69	4.56	4.61
208526_at	olfactory receptor	OR2F1	5.05	4.87	4.94
216761_at	---	---	4.22	4.41	4.20
210547_x_at	islet cell autoantig	ICA1	6.59	6.56	6.45
203803_at	prenylcysteine oxi	PCYOX1	7.36	7.65	7.73
211258_s_at	transforming grov	TGFA	5.18	5.37	5.05
212458_at	sprouty-related, E	SPRED2	9.23	9.17	9.36
211725_s_at	BH3 interacting do	BID	10.06	10.05	10.07

220266_s_at	Kruppel-like facto	KLF4		8.20	8.13	8.36
214491_at	somatostatin rece	SSTR3		7.21	7.20	7.32
210778_s_at	MAX dimerization	MXD4		8.19	8.08	8.10
215398_at	---	---		6.08	6.07	6.07
220845_at	acyl-CoA oxidase-l	ACOXL		3.28	3.26	3.31
200772_x_at	prothymosin, alph	PTMA		14.07	14.14	14.14
202935_s_at	SRY (sex determin	SOX9		4.18	3.81	3.77
212698_s_at	septin 10		10-Sep	11.25	11.29	11.54
205309_at	sphingomyelin ph	SMPDL3B		3.90	4.39	4.23
216645_at	---	---		7.80	7.54	7.54
209036_s_at	malate dehydroge	MDH2		12.21	12.14	12.16
221612_at	leucine-rich repea	LRTM1		5.24	5.34	5.40
37462_i_at	splicing factor 3a,	SF3A2		7.87	7.90	7.95
221874_at	KIAA1324	KIAA1324		3.84	3.67	3.64
221276_s_at	syncoilin, interme	SYNC		5.23	5.27	4.60
219050_s_at	zinc finger, HIT typ	ZNHIT2		7.63	7.51	7.40
57539_at	zinc finger, CCCH-t	ZGPAT		7.22	7.15	7.12
209399_at	holocarboxylase s	HLCS		7.39	7.40	7.46
202002_at	acetyl-CoA acyltra	ACAA2		7.62	7.26	7.26
205995_x_at	IQ motif containir	IQCB1		8.69	8.63	8.87
209924_at	chemokine (C-C m	CCL18		5.97	5.93	5.96
204062_s_at	unc-51-like kinase	ULK2		5.79	5.77	6.18
218767_at	REX4, RNA exonuc	REXO4		8.99	8.76	9.00
220761_s_at	TAO kinase 3	TAOK3		8.47	8.55	8.61
212039_x_at	ribosomal protein	RPL3		14.60	14.58	14.63
209298_s_at	intersectin 1 (SH3	ITSN1		7.58	7.78	7.79
221124_s_at	visual system hon	VSX1		6.06	6.05	5.87
208871_at	atrophin 1	ATN1		6.73	6.66	6.56
218383_at	HAUS augmin-like	HAUS4		9.99	9.91	9.92
213982_s_at	RAB GTPase activa	RABGAP1L		7.53	7.68	7.71
205928_at	zinc finger proteir	ZNF443		6.83	7.18	7.15
214831_at	Mediator complex	MED28		6.80	6.54	6.56
202915_s_at	family with seque	FAM20B		8.69	8.70	8.79
201522_x_at	small nuclear ribo	SNRPN /// SNURF		10.08	10.04	10.10
209227_at	tumor suppressor	TUSC3		6.54	6.60	6.82
209642_at	budding uninhibit	BUB1		9.89	9.96	10.01
204326_x_at	metallothionein 1	MT1X		13.34	13.52	13.49
216988_s_at	protein tyrosine p	PTP4A2		11.25	11.23	11.36
220466_at	coiled-coil domair	CCDC15		7.50	7.55	7.45
204284_at	protein phosphata	PPP1R3C		10.75	10.43	10.78
208815_x_at	heat shock 70kDa	HSPA4		11.69	11.68	11.64
202133_at	WW domain conta	WWTR1		8.75	8.94	8.98
217205_at	---	---		7.26	7.42	7.35
206672_at	aquaporin 2 (colle	AQP2		5.97	5.97	5.82
216942_s_at	CD58 molecule	CD58		8.88	9.05	9.05
205648_at	wingless-type MM	WNT2		5.25	5.09	5.10
217604_at	---	---		6.91	6.96	6.91

213258_at	tissue factor path	TFPI	10.25	10.22	10.57
216282_x_at	hypothetical prot	LOC100289122 ///	6.66	6.88	6.79
220799_at	glial cells missing	GCM2	3.08	3.00	3.15
203253_s_at	diphosphoinositol	PPIP5K2	8.36	8.52	8.65
217734_s_at	WD repeat domai	WDR6	8.93	8.97	8.88
208997_s_at	uncoupling protei	UCP2	5.25	5.38	5.27
208050_s_at	caspase 2, apopto	CASP2	6.66	6.77	6.68
203914_x_at	hydroxyprostaglar	HPGD	4.85	5.18	5.01
203313_s_at	TGFB-induced fact	TGIF1	9.63	9.46	9.65
207721_x_at	histidine triad nuc	HINT1	13.54	13.48	13.62
214021_x_at	Integrin, beta 5	ITGB5	4.64	4.75	4.73
215294_s_at	SWI/SNF related,	SMARCA1	3.98	4.36	4.29
210307_s_at	kelch-like 25 (Dro:	KLHL25	6.58	6.81	6.65
211269_s_at	interleukin 2 rece	IL2RA	4.30	4.38	4.66
216101_at	---	---	6.47	6.36	6.41
33322_i_at	stratifin	SFN	11.57	11.89	11.65
207289_at	matrix metallopep	MMP25	6.71	6.50	6.56
206250_x_at	arginine vasopres:	AVPR1A	6.62	6.69	6.93
201259_s_at	synaptophysin-like	SYPL1	10.00	9.94	10.03
211334_at	MRE11 meiotic re	MRE11A	4.00	3.66	3.60
210749_x_at	discoidin domain	DDR1	8.55	8.63	8.38
219926_at	popeye domain co	POPDC3	8.05	7.98	8.06
214665_s_at	calcium binding pi	CHP	10.79	10.80	10.84
216519_s_at	proline synthetase	PROSC	4.16	4.04	4.06
215411_s_at	TRAF3 interacting	TRAF3IP2	9.94	9.79	9.92
201722_s_at	UDP-N-acetyl-alpł	GALNT1	9.93	10.08	10.19
209901_x_at	allograft inflamma	AIF1	7.05	7.21	6.83
204049_s_at	phosphatase and	PHACTR2	9.59	9.69	9.83
209662_at	centrin, EF-hand p	CETN3	10.68	10.72	10.83
213640_s_at	lysyl oxidase	LOX	4.07	4.11	4.28
204837_at	myotubularin rela	MTMR9	9.37	9.49	9.35
220968_s_at	tetraspanin 9	TSPAN9	7.99	8.09	7.92
219555_s_at	centromere prote	CENPN	10.99	10.95	11.07
218429_s_at	chromosome 19 c	C19orf66	7.38	7.66	7.49
208227_x_at	ADAM metallopep	ADAM22	5.79	5.60	5.54
219331_s_at	kelch domain con	KLHDC8A	5.49	5.07	5.32
201809_s_at	endoglin	ENG	8.48	8.39	8.42
213055_at	CD47 molecule	CD47	7.07	7.12	7.20
220396_at	---	---	5.69	5.75	5.57
218551_at	migration and inv	MIIP	7.64	7.52	7.53
220486_x_at	transmembrane p	TMEM164	7.78	7.78	7.81
209833_at	CASP2 and RIPK1	CRADD	11.22	11.17	11.38
218225_at	ECSIT homolog (D	ECSIT	8.11	8.06	8.20
213734_at	WD repeat and SC	WSB2	9.34	9.48	9.51
220484_at	mucolipin 3	MCOLN3	3.60	3.62	3.51
212906_at	GRAM domain co	GRAMD1B	6.94	7.10	6.98
219724_s_at	KIAA0748	KIAA0748	3.60	3.66	3.56

201771_at	secretory carrier r	SCAMP3	9.68	9.63	9.61
222068_s_at	leucine rich repea	LRRC50	5.37	5.40	5.40
201229_s_at	ariadne homolog :	ARIH2	8.00	7.94	8.17
218385_at	mitochondrial ribo	MRPS18A	9.27	9.10	9.18
209726_at	carbonic anhydras	CA11	6.27	6.13	6.01
210480_s_at	myosin VI	MYO6	7.10	7.24	6.99
207088_s_at	solute carrier fam	SLC25A11	8.03	8.03	8.06
221047_s_at	MAP/microtubule	MARK1	7.37	7.40	7.65
204976_s_at	Alport syndrome,	AMMECR1	10.67	10.66	10.74
207429_at	solute carrier fam	SLC22A2	4.61	4.45	4.44
217790_s_at	signal sequence re	SSR3	8.00	7.99	8.21
211323_s_at	inositol 1,4,5-tripl	ITPR1	7.18	7.08	7.11
207135_at	5-hydroxytryptam	HTR2A	4.69	4.62	4.65
216697_at	Triple functional c	TRIO	6.52	6.71	6.44
212945_s_at	MAX gene associa	MGA	7.24	7.12	7.36
221654_s_at	ubiquitin specific		USP3	10.08	10.03	10.09
206482_at	PTK6 protein tyro:	PTK6	3.67	3.58	3.62
200622_x_at	calmodulin 3 (pho	CALM3	8.39	8.31	8.29
206412_at	fer (fps/fes relate	FER	8.02	7.90	7.96
209527_at	exosome compon	EXOSC2	9.07	9.08	8.97
202188_at	nucleoporin 93kD	NUP93	10.51	10.51	10.47
213589_s_at	UDP-GlcNAc:beta	B3GNTL1	8.43	8.37	8.45
202934_at	hexokinase 2	HK2	10.32	10.12	10.37
211454_x_at	FKSG49	FKSG49	8.67	8.69	8.64
200823_x_at	ribosomal protein	RPL29	14.36	14.32	14.38
208139_s_at	---	---	3.67	3.46	3.52
208690_s_at	PDZ and LIM dom	PDLIM1	11.40	11.34	11.38
217061_s_at	ets variant 1	ETV1	5.50	5.15	5.46
216713_at	KRIT1, ankyrin rep	KRIT1	8.23	8.46	8.56
208056_s_at	core-binding facto	CBFA2T3	4.39	4.28	4.38
203440_at	cadherin 2, type 1	CDH2	10.48	10.45	10.65
220681_at	chromosome 22 c	C22orf26	6.69	6.57	6.53
220386_s_at	echinoderm micro	EML4	6.73	6.75	6.87
204872_at	transducin-like en	TLE4	6.09	6.05	6.50
205070_at	inhibitor of growt	ING3	9.81	9.76	9.86
211501_s_at	eukaryotic transla	EIF3B	9.77	9.66	9.83
55093_at	chondroitin polym	CHPF2	8.27	8.36	8.10
210849_s_at	vacuolar protein s	VPS41	8.38	8.37	8.43
215716_s_at	ATPase, Ca++ tran	ATP2B1	9.49	9.58	9.79
212231_at	F-box protein 21	FBXO21	9.20	9.30	9.25
219029_at	chromosome 5 op	C5orf28	9.63	9.54	10.04
207369_at	bombesin-like rec	BRS3	4.73	4.70	4.53
214541_s_at	quaking homolog,	QKI	3.82	3.80	3.68
202600_s_at	nuclear receptor i	NRIP1	7.50	7.59	7.79
213582_at	ATPase, class VI, t	ATP11A	4.35	4.57	4.77
214887_at	NEDD4 binding pr	N4BP2L1	3.25	3.22	3.28
213133_s_at	glycine cleavage s	GCSH /// LOC1003	10.74	10.89	10.94

200809_x_at	ribosomal protein	RPL12	14.83	14.81	14.87
218241_at	golgin A5	GOLGA5	10.40	10.41	10.42
202874_s_at	ATPase, H+ transp	ATP6V1C1	9.19	9.25	9.27
210430_x_at	Rh blood group, D	RHD	6.02	5.93	5.86
207586_at	sonic hedgehog h	SHH	4.73	4.73	4.62
205581_s_at	nitric oxide syntha	NOS3	5.12	5.04	4.98
214003_x_at	ribosomal protein	RPS20	14.73	14.74	14.79
210821_x_at	centromere prote	CENPA	10.28	10.14	10.32
211242_x_at	killer cell immuno	KIR2DL4	5.72	5.97	5.63
211656_x_at	major histocompa	HLA-DQB1 /// LOC	7.18	6.99	6.85
216013_at	zinc finger, X-linke	ZXDB	4.75	4.72	4.80
206070_s_at	EPH receptor A3	EPHA3	6.75	6.38	6.98
210994_x_at	tripartite motif-co	TRIM23	6.58	6.37	6.33
217141_at	BTB (POZ) domain	BTBD7	4.43	4.65	4.34
220557_s_at	phosphofurin acid	PACS1	5.37	5.16	5.36
212029_s_at	---	---	6.32	6.09	6.25
201035_s_at	hydroxyacyl-CoA c	HADH	9.08	9.00	9.10
205762_s_at	dihydrouridine sy	DUS4L	6.46	6.59	6.62
204749_at	nucleosome asser	NAP1L3	7.49	7.67	7.58
201008_s_at	thioredoxin intera	TXNIP	12.16	11.90	12.10
209519_at	nuclear cap bindir	NCBP1	4.78	5.08	4.95
217620_s_at	phosphoinositide-	PIK3CB	4.57	4.82	4.93
203101_s_at	mannosyl (alpha-1	MGAT2	6.41	6.25	6.42
221734_at	proline-rich coiled	PRRC1	8.76	8.79	8.65
220533_at	---	---	4.91	5.08	5.02
221606_s_at	high-mobility grou	HMGN5	8.93	9.05	9.27
211444_at	---	---	5.91	6.24	5.97
205448_s_at	mitogen-activated	MAP3K12	7.32	7.20	7.08
205205_at	v-rel reticuloendo	RELB	8.11	7.77	7.87
212330_at	transcription facto	TFDP1	11.47	11.43	11.41
212678_at	neurofibromin 1	NF1	7.13	7.24	7.20
220687_at	---	---	5.02	4.79	4.89
205975_s_at	homeobox D1	HOXD1	5.54	5.45	5.49
205024_s_at	RAD51 homolog (	RAD51	9.65	9.63	9.71
219198_at	general transcript	GTF3C4	7.28	7.39	7.17
215610_at	---	---	5.53	5.28	5.53
221039_s_at	ArfGAP with SH3 c	ASAP1	8.34	8.39	8.64
213259_s_at	sterile alpha and T	SARM1	7.50	7.36	7.42
214420_s_at	Cytochrome P450	CYP2C9	4.20	3.92	4.09
201518_at	chromobox homo	CBX1	10.76	10.89	10.84
209007_s_at	chromosome 1 op	C1orf63	9.44	9.72	9.65
208024_s_at	DiGeorge syndron	DGCR6 /// DGCR6I	8.70	8.66	8.67
209220_at	glypican 3	GPC3	5.07	5.16	4.99
209836_x_at	bolA homolog 2 (E	BOLA2 /// BOLA2B	10.91	10.83	10.77
221405_at	---	---	4.10	4.27	4.28
208053_at	guanylate cyclase	GUCY2F	5.74	5.44	5.44
205007_s_at	calcium and integ	CIB2	7.37	7.15	7.06

212496_s_at	lysine (K)-specific	KDM4B	8.95	8.89	8.89
215458_s_at	SMAD specific E3	SMURF1	6.26	6.08	5.94
201626_at	insulin induced ge	INSIG1	10.72	11.44	11.05
204962_s_at	centromere prote	CENPA	11.70	11.55	11.69
203163_at	katanin p80 (WD r	KATNB1	6.87	6.71	6.50
203203_s_at	KRR1, small subur	KRR1	8.67	8.74	8.95
208700_s_at	transketolase	TKT	12.77	12.60	12.69
207633_s_at	muscle, skeletal, r	MUSK	3.43	3.52	3.26
209486_at	UTP3, small subur	UTP3	9.66	9.85	9.77
206506_s_at	suppressor of Ty 3	SUPT3H	8.14	8.05	8.17
206541_at	kallikrein B, plasm	KLKB1	5.31	5.19	4.96
201450_s_at	TIA1 cytotoxic gra	TIA1	8.38	8.59	8.65
203772_at	biliverdin reducta:	BLVRA	4.30	4.08	4.05
219061_s_at	L antigen family, r	LAGE3	9.58	9.66	9.73
203926_x_at	ATP synthase, H+	ATP5D	8.65	8.51	8.42
209884_s_at	solute carrier fam	SLC4A7	9.77	9.72	9.95
214037_s_at	coiled-coil domair	CCDC22	6.49	6.16	6.47
214288_s_at	proteasome (pros	PSMB1	13.16	13.24	13.14
212043_at	trans-golgi netwo	TGOLN2	11.71	11.74	11.89
220435_at	solute carrier fam	SLC30A10	3.52	3.31	3.43
211073_x_at	ribosomal protein	RPL3	14.61	14.59	14.65
206982_at	crystallin, beta A1	CRYBA1	3.35	3.48	3.43
207325_x_at	melanoma antiger	MAGEA1	7.82	7.72	7.71
216402_at	SEC14-like 4 (S. ce	SEC14L4	4.17	4.04	4.22
202908_at	Wolfram syndrom	WFS1	8.02	8.00	7.90
208579_x_at	H2B histone famil	H2BFS	8.25	8.27	8.35
207599_at	matrix metallopep	MMP20	3.63	3.61	3.54
203044_at	chondroitin sulfat	CHSY1	9.90	9.96	10.09
219011_at	pleckstrin homolo	PLEKHA4	6.12	5.83	5.85
201719_s_at	erythrocyte meml	EPB41L2	9.23	9.26	9.31
203851_at	insulin-like growtl	IGFBP6	10.35	10.42	10.30
206422_at	glucagon	GCG	3.54	3.55	3.51
212228_s_at	coenzyme Q9 hon	COQ9	10.48	10.42	10.46
202371_at	transcription elon	TCEAL4	11.08	11.19	11.36
203927_at	nuclear factor of k	NFKBIE	8.75	8.61	8.79
207219_at	zinc finger proteir	ZNF643	7.04	7.18	7.54
209361_s_at	poly(rC) binding p	PCBP4	8.35	8.48	8.49
217547_x_at	zinc finger proteir	ZNF675	3.60	3.56	3.56
213721_at	SRY (sex determin	SOX2	5.88	5.54	5.86
214273_x_at	nitrogen permeas	NPRL3	7.89	7.75	7.49
208794_s_at	SWI/SNF related,	SMARCA4	10.61	10.61	10.66
221180_at	YSK4 Sps1/Ste20-r	YSK4	6.64	6.48	6.45
208831_x_at	suppressor of Ty 6	SUPT6H	7.07	7.07	7.02
202880_s_at	cytohesin 1	CYTH1	8.16	8.15	8.38
205578_at	receptor tyrosine	ROR2	4.13	4.04	3.95
221216_s_at	sex comb on midl	SCMH1	7.58	7.40	7.63
220124_at	gigaxonin	GAN	6.27	6.20	6.12

222099_s_at	LSM14A, SCD6 ho	LSM14A	8.55	8.50	8.51
218733_at	male-specific leth	MSL2	9.12	9.01	9.26
212158_at	syndecan 2	SDC2	8.93	9.04	9.26
207747_s_at	docking protein 4	DOK4	5.54	5.54	5.48
221832_s_at	leucine zipper pro	LUZP1	8.56	8.50	8.50
206761_at	CD96 molecule	CD96	3.58	3.49	3.43
201728_s_at	KIAA0100	KIAA0100	8.05	7.86	7.91
201973_s_at	chromosome 7 op	C7orf28A /// C7or	12.22	12.25	12.31
200088_x_at	ribosomal protein	RPL12	14.89	14.85	14.87
221044_s_at	tripartite motif-co	TRIM34 /// TRIM6	5.80	6.14	5.95
211544_s_at	growth hormone	GHRHR	5.56	5.55	5.43
206763_at	FK506 binding pro	FKBP6	6.54	6.27	6.27
217230_at	ezrin	EZR	6.90	7.14	6.91
201253_s_at	CDP-diacylglycero	CDIPT	11.18	11.10	11.10
204821_at	butyrophilin, subf	BTN3A3	5.91	5.98	5.97
202699_s_at	transmembrane p	TMEM63A	6.97	6.84	6.74
220846_s_at	hypothetical prote	LOC100294391	6.54	6.50	6.27
203544_s_at	signal transducing	STAM	9.84	9.85	9.99
205750_at	biphenyl hydrolas	BPHL	7.91	7.96	7.92
209951_s_at	mitogen-activated	MAP2K7	5.56	5.58	5.40
221802_s_at	KIAA1598	KIAA1598	8.88	9.09	8.89
218077_s_at	zinc finger, DHHC-	ZDHHC3	7.55	7.54	7.33
201033_x_at	ribosomal protein	RPLP0	15.06	15.03	15.03
218084_x_at	FXYD domain cont	FXYD5	9.13	9.02	9.03
215354_s_at	proline, glutamate	PELP1	7.57	7.51	7.71
202375_at	SEC24 family, mer	SEC24D	7.74	7.88	8.24
211754_s_at	solute carrier fam	SLC25A17	7.88	7.94	7.98
209723_at	serpin peptidase i	SERPINB9	3.60	3.68	3.69
209199_s_at	myocyte enhance	MEF2C	6.33	6.05	5.97
203113_s_at	eukaryotic transla	EEF1D	13.21	13.08	13.10
222039_at	kinesin family mer	KIF18B	9.78	9.84	9.80
204402_at	rhomboid domain	RHBDD3	8.27	8.27	8.13
203614_at	UTP14, U3 small r	UTP14C	8.69	8.78	8.72
211493_x_at	dystrobrevin, alph	DTNA	6.22	6.59	6.35
221331_x_at	cytotoxic T-lymph	CTLA4	4.62	4.67	4.53
222073_at	collagen, type IV,	COL4A3	3.30	3.12	3.16
202586_at	polymerase (RNA)	POLR2L	7.96	7.95	8.04
220889_s_at	carbonic anhydras	CA10	3.29	3.24	3.14
219544_at	chromosome 13 o	C13orf34	8.45	8.44	8.58
205982_x_at	surfactant protein	SFTPC	6.54	6.41	6.42
208639_x_at	protein disulfide is	PDIA6	12.27	12.33	12.22
206055_s_at	small nuclear ribo	SNRPA1	11.77	11.89	11.96
205181_at	zinc finger protein	ZNF193	7.54	7.44	7.38
211114_x_at	survival of motor	SIP1	9.25	9.22	9.26
201934_at	WD repeat domai	WDR82	11.49	11.47	11.52
206424_at	cytochrome P450,	CYP26A1	4.79	4.79	4.69
218763_at	syntaxin 18	STX18	8.80	8.85	8.79

218590_at	chromosome 10 c	C10orf2	9.58	9.39	9.53
207849_at	interleukin 2	IL2	3.41	3.34	3.38
216517_at	immunoglobulin k	IGKC /// IGKV1D-8	4.37	4.00	4.34
203682_s_at	isovaleryl-CoA del	IVD	8.40	8.46	8.35
204072_s_at	furry homolog (Dr	FRY	6.19	6.12	6.19
209380_s_at	ATP-binding casse	ABCC5	7.67	8.00	7.77
207131_x_at	gamma-glutamylt	GGT1	7.09	6.96	7.01
204478_s_at	RAB interacting fa	RABIF	8.31	8.36	8.41
201192_s_at	phosphatidylinosi	PITPNA	7.94	8.06	8.00
209187_at	down-regulator of	DR1	11.67	11.77	11.86
201194_at	selenoprotein W,	SEPW1	9.11	9.08	9.07
221419_s_at	---	---	8.70	8.65	8.74
212285_s_at	agrin	AGRN	8.20	8.11	8.25
217346_at	similar to TRIMCy	LOC100293160 ///	7.15	7.11	7.16
212289_at	ankyrin repeat do	ANKRD12	6.61	6.79	6.76
200779_at	activating transcri	ATF4	13.51	13.50	13.55
221041_s_at	solute carrier fam	SLC17A5	7.47	7.75	7.66
206986_at	fibroblast growth	FGF18	7.01	6.93	6.82
211929_at	heterogeneous nu	HNRNPA3	11.42	11.41	11.66
203957_at	E2F transcription	E2F6	8.25	8.27	8.21
204230_s_at	solute carrier fam	SLC17A7	6.82	6.67	6.56
210094_s_at	par-3 partitioning	PARD3	9.89	9.66	9.84
203418_at	cyclin A2	CCNA2	10.07	10.03	10.16
202039_at	myosin XVIIIA ///	MYO18A /// TIAF1	6.11	6.12	6.07
204984_at	glypican 4	GPC4	3.66	3.75	3.75
209200_at	myocyte enhance	MEF2C	5.76	5.46	5.58
207836_s_at	RNA binding prote	RBPMS	7.55	7.50	7.51
202056_at	karyopherin alpha	KPNA1	9.12	9.08	9.29
214305_s_at	splicing factor 3b,	SF3B1	9.76	9.79	9.62
202917_s_at	S100 calcium bind	S100A8	6.09	6.12	6.02
209439_s_at	phosphorylase kir	PHKA2	7.32	7.55	7.47
204077_x_at	ectonucleoside tri	ENTPD4	6.56	6.68	6.81
216357_at	sorting nexin 1	SNX1	4.19	4.13	4.52
201060_x_at	stomatin	STOM	4.26	4.17	4.44
211273_s_at	T-box 1	TBX1	3.48	3.57	3.29
220253_s_at	low density lipopr	LRP12	7.30	7.46	7.66
220332_at	claudin 16	CLDN16	6.51	6.44	6.67
208176_at	double homeobox	DUX1	8.20	8.08	8.06
205511_at	hypothetical prote	FLJ10038	5.70	6.07	6.14
201105_at	lectin, galactoside	LGALS1	14.11	14.09	14.04
207924_x_at	paired box 8	PAX8	3.94	3.99	3.94
218615_s_at	transmembrane p	TMEM39A	7.86	8.04	8.06
216597_at	---	---	5.61	5.46	5.32
212511_at	phosphatidylinosi	PICALM	9.31	9.31	9.44
216818_s_at	olfactory receptor	OR2J2	4.29	4.27	4.30
216209_at	hypothetical gene	LOC400084	5.12	4.76	4.62
208240_s_at	fibroblast growth	FGF1	4.75	4.81	4.54

211459_at	---	---	3.60	3.60	3.58
207468_s_at	secreted frizzled-r	SFRP5	6.25	6.04	6.01
214853_s_at	SHC (Src homolog	SHC1	12.53	12.59	12.59
204689_at	hematopoietically	HHEX	5.03	4.76	4.72
204662_at	CP110 protein	CP110	9.14	9.13	9.25
220073_s_at	pleckstrin homolo	PLEKHG6	6.44	6.29	6.12
218002_s_at	chemokine (C-X-C	CXCL14	4.41	4.47	4.49
213427_at	ribonuclease P/M	RPP40	10.13	10.07	10.13
205476_at	chemokine (C-C m	CCL20	6.69	6.76	6.88
211319_at	RAE1 RNA export	RAE1	5.83	5.57	5.70
201666_at	TIMP metallopept	TIMP1	10.68	10.61	10.55
220640_at	casein kinase 1, ga	CSNK1G1	4.29	4.11	4.32
217152_at	---	---	7.48	7.56	7.72
205594_at	zinc finger protein	ZNF652	6.19	6.07	5.80
218871_x_at	chondroitin sulfat	CSGALNACT2	9.27	9.37	9.60
214523_at	CCAAT/enhancer I	CEBPE	6.42	6.02	5.89
216895_at	gamma-aminobut	GABRG3	3.37	3.33	3.40
203764_at	discs, large (Drosc	DLGAP5	10.33	10.58	10.48
41037_at	TEA domain famil	TEAD4	8.11	7.98	8.08
209203_s_at	bicaudal D homol	BICD2	7.96	7.95	7.94
203041_s_at	lysosomal-associa	LAMP2	9.59	9.62	9.71
219057_at	rabaptin, RAB GTF	RABEP2	7.52	7.29	7.34
200734_s_at	ADP-ribosylation f	ARF3	9.10	9.17	9.15
212872_s_at	mediator complex	MED20	9.63	9.51	9.69
210459_at	proteasome (pros	PSMD4	6.59	6.43	6.18
201803_at	polymerase (RNA)	POLR2B	11.32	11.42	11.36
217378_x_at	similar to hCG266	LOC100130100 ///	5.53	5.30	5.17
220516_at	zinc finger and SC	ZSCAN2	6.51	6.22	6.40
221758_at	armadillo repeat c	ARMC6	8.37	8.12	8.27
202829_s_at	matrix metallope	MMP14	10.68	10.83	10.89
210934_at	B lymphoid tyrosi	BLK	6.10	5.69	5.68
217508_s_at	chromosome 18 c	C18orf25	6.24	6.31	6.43
214123_s_at	chromosome 4 op	C4orf10	5.95	6.21	5.95
220876_at	---	---	3.47	3.62	3.65
203587_at	ADP-ribosylation f	ARL4D	3.83	3.79	3.88
207854_at	glycophorin E (MN	GYPE	3.69	3.57	3.75
214623_at	F-box and WD rep	FBXW4P1	6.66	6.39	6.33
205824_at	heat shock 27kDa	HSPB2	6.54	6.22	6.35
206364_at	kinesin family me	KIF14	10.24	10.56	10.54
211261_at	nucleoporin 214kl	NUP214	8.00	7.97	7.82
205488_at	granzyme A (gran	GZMA	3.47	3.59	3.63
208322_s_at	ST3 beta-galactos	ST3GAL1	7.26	7.22	7.50
213574_s_at	---	---	12.25	12.24	12.28
221959_at	family with seque	FAM110B	5.72	5.55	5.69
216801_at	---	---	6.05	5.87	6.13
217934_x_at	STIP1 homology a	STUB1	10.61	10.67	10.63
213931_at	inhibitor of DNA b	ID2 /// ID2B	5.04	5.20	5.01

208773_s_at	ankyrin repeat an	ANKHD1 /// ANKH	9.09	9.07	9.23
217690_at	---	---	6.93	7.03	7.07
207703_at	neuroligin 4, Y-linl	NLGN4Y	3.50	3.45	3.44
213030_s_at	plexin A2	PLXNA2	8.01	7.88	8.04
208253_at	sialic acid binding	SIGLEC8	5.69	5.34	5.44
212212_s_at	integrator comple	INTS1	8.88	8.78	8.82
39249_at	aquaporin 3 (Gill k	AQP3	6.99	7.11	6.92
214036_at	ephrin-A5	EFNA5	8.99	8.81	8.93
204122_at	TYRO protein tyro	TYROBP	4.78	4.66	4.71
218812_s_at	ORAI calcium rele	ORAI2	5.36	5.64	5.41
202406_s_at	TIA1 cytotoxic gra	TIAL1	10.67	10.61	10.71
219756_s_at	premature ovariai	POF1B	5.72	5.53	5.76
208121_s_at	protein tyrosine p	PTPRO	5.02	4.81	4.84
219856_at	chromosome 1 op	C1orf116	5.51	5.70	5.53
203429_s_at	chromosome 1 op	C1orf9	8.66	8.81	8.97
203344_s_at	retinoblastoma bi	RBBP8	10.81	10.83	11.02
208010_s_at	protein tyrosine p	PTPN22	4.10	4.12	4.50
208368_s_at	breast cancer 2, e	BRCA2	5.16	5.25	5.40
214567_s_at	chemokine (C mot	XCL1 /// XCL2	3.75	3.87	3.80
206311_s_at	phospholipase A2	PLA2G1B	3.52	3.61	3.55
211720_x_at	ribosomal protein	RPLP0	15.06	15.04	15.07
218311_at	mitogen-activatec	MAP4K3	7.72	7.87	8.16
204532_x_at	UDP glucuronosyl	UGT1A1 /// UGT1A	5.58	5.77	5.80
213332_at	pappalysin 2	PAPPA2	6.64	6.26	6.60
201627_s_at	insulin induced ge	INSIG1	10.06	10.70	10.13
214960_at	apoptosis inhibito	API5	7.74	7.88	7.83
202494_at	peptidylprolyl isor	PPIE	8.45	8.41	8.53
204083_s_at	tropomyosin 2 (be	TPM2	12.00	11.94	12.05
210855_at	growth regulation	GREB1	5.86	5.91	5.71
219436_s_at	endomucin	EMCN	5.00	5.11	5.26
215083_at	---	---	6.15	6.10	6.08
220619_at	chromodomain he	CHD7	3.76	3.68	3.51
220856_x_at	---	---	10.87	10.86	10.81
206073_at	collagen-like tail s	COLQ	5.63	5.82	5.77
217457_s_at	RAP1, GTP-GDP di	RAP1GDS1	8.55	8.46	8.67
218786_at	5'-nucleotidase dc	NT5DC3	7.24	7.28	7.27
220267_at	keratin 24	KRT24	3.92	3.91	3.69
221304_at	UDP glucuronosyl	UGT1A10 /// UGT1	3.94	4.10	4.07
202772_at	3-hydroxymethyl-	HMGCL	9.03	8.91	9.01
211648_at	immunoglobulin h	IGHG1 /// IGHM //	4.69	4.48	4.29
205173_x_at	CD58 molecule	CD58	9.84	10.00	10.05
200751_s_at	heterogeneous nu	HNRNPC	11.87	11.95	12.03
203094_at	MAD2L1 binding p	MAD2L1BP	9.30	9.27	9.30
208228_s_at	fibroblast growth	FGFR2	4.09	4.17	4.24
217337_at	---	---	5.47	5.52	5.35
211823_s_at	paxillin	PXN	5.75	5.78	5.69
208638_at	protein disulfide is	PDIA6	12.15	12.20	12.25

204782_at	---	---	5.41	5.43	5.15
221908_at	ring finger protein	RNFT2	6.91	6.81	6.74
204024_at	oxidative stress in	OSGIN2	5.82	6.21	6.07
209487_at	RNA binding prote	RBPMS	8.60	8.62	8.73
215358_x_at	zinc finger protein	ZNF37B	6.38	6.09	6.11
215352_at	---	---	4.31	4.18	4.04
218097_s_at	CUE domain conta	CUEDC2	10.91	10.82	10.92
201359_at	coatomer protein	COPB1	10.81	10.95	10.88
213292_s_at	sorting nexin 13	SNX13	8.11	8.34	8.39
59705_at	selenocysteine lya	SCLY	7.05	7.04	7.14
214477_at	myeloid/lymphoic	MLLT1	5.07	4.74	4.50
220401_at	hypothetical prote	FLJ21369	3.42	3.24	3.16
210662_at	kynureninase (L-k	KYNU	3.66	3.62	3.58
216615_s_at	5-hydroxytryptam	HTR3A	5.29	5.21	5.31
217631_at	GTP binding prote	GTPBP4	7.50	7.64	7.84
204798_at	v-myb myeloblast	MYB	5.49	5.33	5.26
216108_at	---	---	3.54	3.59	3.46
214449_s_at	ras homolog gene	RHOQ	8.58	8.55	8.71
219393_s_at	v-akt murine thym	AKT3	5.85	6.01	5.78
212822_at	HEG homolog 1 (z	HEG1	8.17	8.17	8.16
212632_at	syntaxin 7	STX7	9.08	8.98	9.06
201296_s_at	WD repeat and SC	WSB1	9.53	9.68	9.82
214476_at	trefoil factor 2	TFF2	6.76	6.60	6.54
213569_at	---	---	6.31	6.40	6.11
203724_s_at	RUN and FYVE dor	RUFY3	5.98	5.98	5.96
202261_at	vacuolar protein s	VPS72	8.97	8.93	9.04
205798_at	interleukin 7 rece	IL7R	6.61	6.89	6.75
205805_s_at	receptor tyrosine	ROR1	7.68	7.56	7.87
202702_at	tripartite motif-co	TRIM26	7.84	7.71	7.81
214640_at	unc-93 homolog A	UNC93A	4.30	4.21	4.01
202280_at	---	---	3.55	3.19	3.19
206922_at	variable charge, Y	VCY /// VCY1B	6.66	6.43	6.60
208804_s_at	splicing factor, arg	SFRS6	10.95	11.01	10.93
202377_at	leptin receptor ov	LEPROT	9.79	9.76	9.89
35846_at	thyroid hormone	THRA	7.08	7.18	7.07
219771_at	TBC1 domain fam	TBC1D8B	6.99	7.22	7.22
204249_s_at	LIM domain only 2	LMO2	6.26	6.16	5.98
206768_at	ribosomal protein	RPL3L	4.77	4.74	4.49
221737_at	guanine nucleotid	GNA12	8.37	8.21	8.32
221754_s_at	coronin, actin bin	CORO1B	7.78	7.40	7.52
205645_at	RALBP1 associate	REPS2	5.34	5.11	5.13
210557_x_at	colony stimulating	CSF1	6.26	6.24	6.01
209489_at	CUGBP, Elav-like f	CELF1	9.64	9.55	9.48
211949_s_at	nucleolar and coil	NOLC1	8.29	8.18	8.24
206051_at	ELAV (embryonic l	ELAVL4	5.14	5.18	4.85
216426_at	---	---	4.34	4.39	4.44
218453_s_at	chromosome 6 op	C6orf35	7.78	7.60	7.80

202166_s_at	protein phosphata	PPP1R2	9.19	9.43	9.50
201702_s_at	protein phosphata	PPP1R10	7.52	7.61	7.27
209732_at	C-type lectin dom	CLEC2B	3.96	3.66	3.85
200847_s_at	transmembrane p	TMEM66	11.81	11.87	11.99
218641_at	chromosome 11 c	C11orf95	8.49	8.30	8.41
208790_s_at	polymerase I and	PTRF	8.94	9.05	9.03
206215_at	opioid binding pro	OPCML	4.50	4.55	4.50
214008_at	Twinfilin, actin-bir	TWF1	4.18	4.35	4.63
201856_s_at	zinc finger RNA bi	ZFR	8.22	8.34	8.23
220020_at	X-prolyl aminopep	XPNPEP3	5.12	5.11	5.17
204659_s_at	growth factor, aug	GFER	8.56	8.40	8.57
220666_at	---	---	3.63	3.59	3.69
203843_at	ribosomal protein	RPS6KA3	7.79	7.76	7.84
218261_at	adaptor-related p	AP1M2	7.03	7.12	6.82
202590_s_at	pyruvate dehydro	PDK2	6.62	6.47	6.33
215110_at	mannose-binding	MBL1P	4.45	4.42	4.37
203025_at	N(alpha)-acetyltra	NAA10	9.97	9.83	9.84
222319_at	---	---	5.93	5.99	5.71
215700_x_at	copine VI (neuron	CPNE6	6.62	6.76	6.58
208011_at	protein tyrosine p	PTPN22	3.37	3.35	3.32
211339_s_at	IL2-inducible T-cel	ITK	3.69	3.55	3.74
211271_x_at	polypyrimidine tra	PTBP1	10.71	10.58	10.62
220040_x_at	zinc finger, C4H2 c	ZC4H2	7.51	7.36	7.18
200983_x_at	CD59 molecule, co	CD59	11.39	11.43	11.46
215188_at	serine/threonine k	STK24	4.60	4.94	4.85
218772_x_at	transmembrane p	TMEM38B	8.47	8.73	8.85
203421_at	tumor protein p53	TP53I11	6.41	6.32	6.25
221866_at	transcription facto	TFEB	7.89	7.71	7.86
211845_at	poliovirus recepto	PVRL1	6.36	6.06	6.13
216190_x_at	integrin, beta 1 (fi	ITGB1	3.99	3.71	3.65
201421_s_at	WD repeat domai	WDR77	9.37	9.40	9.29
212559_at	protein kinase, cA	PRKAR1B	6.15	6.04	6.04
207938_at	peptidase inhibito	PI15	4.92	4.97	4.97
208400_at	glucagon-like pep	GLP1R	4.15	3.97	3.97
213510_x_at	TL132 protein	LOC220594	8.54	8.59	8.67
207878_at	keratin 76	KRT76	4.36	4.47	4.26
205192_at	mitogen-activated	MAP3K14	6.44	6.58	6.42
200660_at	S100 calcium bind	S100A11	9.40	9.37	9.50
203864_s_at	actinin, alpha 2	ACTN2	5.00	4.91	4.72
204406_at	fms-related tyrosi	FLT1	3.62	3.81	4.00
206557_at	zinc finger protein	ZNF702P	3.45	3.73	3.69
AFFX-TrpnX-5_a	---	---	4.22	4.06	4.12
212144_at	Sad1 and UNC84 c	SUN2	8.95	8.79	8.87
221850_x_at	ankyrin repeat an	AGAP11 /// AGAP4	8.82	8.82	8.73
201051_at	acidic (leucine-ricl	ANP32A	11.38	11.47	11.40
213974_at	ADAMTS-like 3	ADAMTSL3	3.62	3.55	3.69
214811_at	RIMS binding prot	RIMBP2	3.58	3.70	3.35

218999_at	transmembrane p	TMEM140	5.75	5.64	5.88
202183_s_at	kinesin family mei	KIF22	8.87	8.77	8.80
219876_s_at	golgin A2 family, r	GOLGA2B	5.70	5.51	5.37
213506_at	coagulation factoı	F2RL1	9.83	9.88	10.09
204350_s_at	mediator complex	MED7	8.58	8.65	8.68
211872_s_at	regulator of G-prc	RGS11	6.10	6.09	6.03
204958_at	polo-like kinase 3	PLK3	7.34	7.29	7.28
201641_at	bone marrow strc	BST2	9.98	9.74	10.01
212507_at	transmembrane p	TMEM131	9.11	9.13	9.28
205561_at	potassium channe	KCTD17	7.24	7.10	6.94
203607_at	inositol polyphosp	INPP5F	8.27	8.38	8.45
207482_at	TP53 target 5	TP53TG5	6.41	6.63	6.50
207101_at	vesicle-associated	VAMP1	5.04	5.16	5.64
217066_s_at	dystrophia myoto	DMPK	6.84	6.83	6.70
201548_s_at	lysine (K)-specific	KDM5B	7.77	7.78	7.79
217626_at	aldo-keto reducta	AKR1C1	6.19	5.79	5.46
203989_x_at	coagulation factoı	F2R	8.88	9.03	9.35
221052_at	tudor and KH dom	TDRKH	4.15	4.15	3.91
208931_s_at	interleukin enhan	ILF3	9.48	9.38	9.43
201930_at	minichromosome	MCM6	10.69	11.06	10.89
218156_s_at	TSR1, 20S rRNA ac	TSR1	9.58	9.61	9.76
204510_at	cell division cycle	CDC7	9.65	9.73	9.77
201846_s_at	RING1 and YY1 bir	RYBP	10.40	10.23	10.35
204514_at	DPH2 homolog (S.	DPH2	8.94	8.71	8.83
221191_at	stromal antigen 3-	STAG3L1	6.46	6.44	6.22
221488_s_at	cutA divalent catic	CUTA	12.17	12.17	12.16
215556_at	---	---	5.43	5.45	5.51
213347_x_at	ribosomal protein	RPS4X	14.64	14.64	14.66
203271_s_at	unc-119 homolog	UNC119	7.97	7.95	7.68
215193_x_at	major histocompa	HLA-DRB1 /// HLA-	4.77	4.86	4.83
213536_s_at	Ubiquitin-conjuga	UBE2I	5.74	5.81	5.49
200601_at	actinin, alpha 4	ACTN4	8.93	9.02	8.87
209145_s_at	core-binding facto	CBFA2T2	5.32	5.32	5.12
216231_s_at	beta-2-microglobu	B2M	14.37	14.39	14.39
210733_at	translocation assc	TRAM1	8.26	8.39	8.27
221316_at	cation channel, sp	CATSPERG	4.31	4.15	4.31
213632_at	dihydroorotate de	DHODH	7.75	7.69	7.89
221902_at	G protein-couplec	GPR153	7.12	7.01	6.84
207595_s_at	bone morphogene	BMP1	6.76	6.58	6.58
204316_at	regulator of G-prc	RGS10	7.81	7.99	7.63
214071_at	guanine nucleotid	GNAL	3.20	3.31	3.28
212623_at	transmembrane p	TMEM41B	9.55	9.62	9.67
212088_at	peptidase (mitoch	PMPCA	9.37	9.30	9.31
200627_at	prostaglandin E sy	PTGES3	13.31	13.35	13.37
204720_s_at	DnaJ (Hsp40) hom	DNAJC6	9.16	8.97	9.17
203304_at	BMP and activin n	BAMBI	9.05	8.45	9.10
215694_at	spermatogenesis a	SPATA5L1	6.16	6.49	6.26

219899_x_at	NADPH dependen	NDOR1	6.90	6.87	6.74
203504_s_at	ATP-binding casse	ABCA1	5.53	5.41	5.90
46947_at	guanine nucleotid	GNL3L	7.31	7.22	7.39
209627_s_at	oxysterol binding	OSBPL3	8.52	8.50	8.69
205281_s_at	phosphatidylinosi	PIGA	7.70	7.79	7.86
220168_at	cancer susceptibil	CASC1	3.62	3.74	3.74
218719_s_at	GINS complex sub	GINS3	8.29	8.23	8.38
203119_at	coiled-coil domair	CCDC86	9.64	9.46	9.65
201454_s_at	aminopeptidase p	NPEPPS	10.52	10.61	10.53
203438_at	stanniocalcin 2	STC2	9.31	9.25	9.37
208218_s_at	activin A receptor	ACVR1B	5.26	5.17	5.23
215297_at	hypothetical locus	LOC441204	4.21	4.47	4.27
208581_x_at	metallothionein 1	MT1X	13.67	13.81	13.73
209082_s_at	collagen, type XVI	COL18A1	7.99	7.87	7.87
214760_at	zinc finger proteir	ZNF337	8.09	8.17	7.84
208791_at	clusterin	CLU	9.41	9.21	9.31
213488_at	sushi, nidogen anc	SNED1	4.63	4.75	4.72
222293_at	cell adhesion mol	CADM4	6.57	6.24	6.19
218875_s_at	F-box protein 5	FBXO5	10.02	10.00	10.32
211637_x_at	immunoglobulin h	IGH@ /// IGHA1 //	5.02	5.12	5.05
200019_s_at	Finkel-Biskis-Reilly	FAU	14.56	14.50	14.46
206980_s_at	fms-related tyrosi	FLT3LG	5.18	5.37	4.93
221841_s_at	Kruppel-like facto	KLF4	11.37	11.18	11.45
206413_s_at	T-cell leukemia/ly	TCL1B	6.75	6.46	6.81
201009_s_at	thioredoxin intera	TXNIP	12.98	12.74	12.89
219364_at	DEXH (Asp-Glu-X-I	DHX58	7.12	6.97	6.96
219123_at	zinc finger proteir	ZNF232	6.53	6.45	6.40
45653_at	potassium channe	KCTD13	5.55	5.68	5.75
216186_at	---	---	7.01	6.95	7.15
213998_s_at	DEAD (Asp-Glu-Al	DDX17	8.07	8.26	8.01
217214_s_at	solute carrier fam	SLC6A2	3.81	3.90	3.95
205389_s_at	ankyrin 1, erythro	ANK1	5.14	4.74	5.01
221551_x_at	ST6 (alpha-N-acet	ST6GALNAC4	7.36	7.19	7.36
220158_at	lectin, galactoside	LGALS14	4.36	4.56	4.48
208550_x_at	potassium voltage	KCNG2	5.83	5.85	5.63
202153_s_at	nucleoporin 62kD	NUP62	9.75	9.65	9.73
203139_at	death-associated	DAPK1	4.03	3.95	3.88
218836_at	ribonuclease P/M	RPP21 /// TRIM39	10.20	10.12	10.06
221186_at	hypothetical LOC1	LOC100131532	5.82	5.76	5.68
208187_s_at	---	---	4.84	4.62	4.79
202649_x_at	ribosomal protein	RPS19	14.90	14.90	14.99
215768_at	---	---	4.20	4.53	3.93
205883_at	zinc finger and BT	ZBTB16	9.81	9.64	9.72
219990_at	E2F transcription	E2F8	6.84	6.72	6.94
209581_at	phospholipase A2	PLA2G16	9.17	9.15	9.21
202110_at	cytochrome c oxic	COX7B	12.52	12.54	12.59
209938_at	transcriptional ad	TADA2A	7.41	7.19	7.22

203189_s_at	NADH dehydroger	NDUFS8	9.78	9.58	9.80
212447_at	kelch repeat and I	KBTBD2	9.58	9.47	9.46
213005_s_at	KN motif and anky	KANK1	8.73	8.67	8.81
203467_at	phosphomannom	PMM1	8.87	8.68	8.87
215731_s_at	M-phase phospho	MPHOSPH9	7.71	7.83	7.80
212675_s_at	centrosomal prote	CEP68	9.97	10.01	10.08
219221_at	zinc finger and BT	ZBTB38	10.69	10.72	10.80
209798_at	nuclear protein, a	NPAT	7.86	7.98	8.05
215719_x_at	Fas (TNF receptor	FAS	7.35	7.33	7.66
219348_at	unconventional SI	USE1	8.84	8.72	8.67
201713_s_at	RAN binding prote	RANBP2	10.03	10.20	10.24
212199_at	Morf4 family asso	MRFAP1L1	10.10	10.12	10.30
204363_at	coagulation factor	F3	8.67	8.97	8.86
209216_at	WD repeat domai	WDR45	8.62	8.30	8.69
AFFX-HSAC07/X(	actin, beta	ACTB	11.90	11.95	11.91
217018_at	---	---	4.90	4.74	4.78
218549_s_at	family with seque	FAM82B	9.90	9.86	10.01
220581_at	chromosome 6 op	C6orf97	3.78	3.85	3.80
214454_at	ADAM metallopep	ADAMTS2	5.90	6.03	5.83
210283_x_at	similar to poly(A)	LOC645139 /// PA	10.47	10.44	10.75
218774_at	decapping enzyme	DCPS	8.30	8.30	8.27
216871_at	---	---	3.79	3.82	3.80
218624_s_at	hypothetical LOC6	MGC2752	8.92	8.96	9.00
214849_at	potassium channe	KCTD20	6.95	6.88	6.88
220976_s_at	keratin associatec	KRTAP1-1	4.34	4.39	4.42
216027_at	thioredoxin-relate	TMX4	4.16	3.85	4.03
204791_at	nuclear receptor s	NR2C1	7.71	7.81	7.84
217419_x_at	agrin	AGRN	8.40	8.23	8.34
202062_s_at	sel-1 suppressor c	SEL1L	8.00	7.87	7.92
209926_at	hypothetical prote	LOC729991	6.16	6.16	6.12
202862_at	period homolog 1	PER1	8.56	8.36	8.49
207922_s_at	macrophage erytl	MAEA	9.78	9.95	9.90
216169_at	---	---	3.32	3.63	3.84
212844_at	ribosomal RNA pr	RRP1B	7.18	7.02	7.04
209266_s_at	solute carrier fam	SLC39A8	5.88	5.58	5.87
214641_at	collagen, type IV,	COL4A3	4.50	4.46	4.56
204438_at	mannose receptor	MRC1 /// MRC1L1	3.93	4.35	4.03
206607_at	Cas-Br-M (murine	CBL	5.24	5.12	5.27
209037_s_at	EH-domain contai	EHD1	6.18	5.92	5.92
214103_s_at	RAP2A, member c	RAP2A	4.94	4.76	4.70
204549_at	inhibitor of kappa	IKBKE	7.35	7.30	7.34
200956_s_at	structure specific	SSRP1	10.41	10.33	10.34
219656_at	protocadherin 12	PCDH12	7.06	7.13	6.77
215665_at	hydroxy-delta-5-s	HSD3B1 /// LOC39	3.98	3.90	4.12
218909_at	ribosomal protein	RPS6KC1	7.67	7.90	7.85
218443_s_at	DAZ associated pr	DAZAP1	9.89	9.88	10.04
202509_s_at	tumor necrosis fa	TNFAIP2	5.99	6.25	5.83

202570_s_at	discs, large (Drosc	DLGAP4		8.09	8.10	8.04
202289_s_at	transforming, acic	TACC2		9.11	8.87	9.04
212413_at	septin 6		6-Sep	5.47	5.45	5.69
212586_at	calpastatin	CAST		11.42	11.53	11.48
212773_s_at	translocase of out	TOMM20		11.50	11.36	11.51
220117_at	zinc finger proteir	ZNF385D		7.09	6.97	7.22
201974_s_at	chromosome 7 op	C7orf28A		6.17	6.32	6.27
200040_at	KH domain contai	KHDRBS1		11.32	11.28	11.37
211189_x_at	CD84 molecule	CD84		5.02	5.46	5.18
212665_at	TCDD-inducible pc	TIPARP		11.52	11.50	11.58
217835_x_at	chromosome 20 c	C20orf24		12.83	12.79	12.75
219940_s_at	PCI domain contai	PCID2		10.94	10.95	10.93
211985_s_at	calmodulin 1 (pho	CALM1		9.40	9.48	9.35
219715_s_at	tyrosyl-DNA phos	TDP1		8.20	8.38	8.31
56197_at	phospholipid scra	PLSCR3		9.97	10.10	10.00
220526_s_at	mitochondrial ribo	MRPL20		10.98	11.08	11.13
202734_at	thyroid hormone	TRIP10		7.62	7.45	7.42
220624_s_at	E74-like factor 5 (	ELF5		3.88	3.93	4.02
201402_at	adrenergic, beta,	ADRBK1		8.05	8.02	7.92
203027_s_at	mevalonate (diph	MVD		6.79	6.61	6.57
221965_at	M-phase phospho	MPHOSPH9		8.71	8.89	8.77
215261_at	---	---		5.60	5.77	5.76
205684_s_at	DENN/MADD dom	DENND4C		6.63	6.85	6.95
220159_at	ATP-binding casse	ABCA11P		7.01	7.22	7.34
217759_at	tripartite motif-co	TRIM44		10.99	10.91	11.11
216210_x_at	TRIO and F-actin b	TRIOBP		10.48	10.36	10.43
203652_at	mitogen-activatec	MAP3K11		7.38	7.65	7.50
201299_s_at	MOB1, Mps One B	MOBKL1B		8.92	9.18	9.18
204897_at	prostaglandin E re	PTGER4		10.52	10.58	10.61
216843_x_at	postmeiotic segre	PMS2L1		8.10	8.20	8.29
218746_at	TAP binding prote	TAPBPL		6.41	6.21	6.11
201115_at	polymerase (DNA	POLD2		9.85	9.67	9.80
206551_x_at	kelch-like 24 (Dros	KLHL24		9.13	9.01	9.32
206936_x_at	---	---		8.17	8.15	8.12
214115_at	Vesicle-associatec	VAMP5		5.41	5.15	5.29
207761_s_at	methyltransferase	METTL7A		9.44	9.50	9.33
209153_s_at	transcription facto	TCF3		9.65	9.63	9.75
212780_at	son of sevenless h	SOS1		7.73	7.85	7.80
205823_at	regulator of G-pro	RGS12		6.42	6.46	6.44
206160_at	apolipoprotein B r	APOBEC2		5.43	5.20	5.47
203024_s_at	chromosome 5 op	C5orf15		10.30	10.39	10.38
209467_s_at	MAP kinase intera	MKNK1		8.36	8.43	8.29
213087_s_at	eukaryotic transla	EEF1D		7.21	7.17	7.08
219400_at	contactin associat	CNTNAP1		8.45	8.47	8.39
207899_at	gastric inhibitory	GIP		4.91	4.83	4.82
220385_at	junctophilin 2	JPH2		5.99	6.23	5.87
200801_x_at	actin, beta	ACTB		14.26	14.40	14.41

200952_s_at	cyclin D2	CCND2	4.14	4.05	3.96
202328_s_at	polycystic kidney d	PKD1	8.37	8.00	8.32
207531_at	crystallin, gamma	CRYGC	6.07	6.02	6.17
213661_at	peptidase domain	PAMR1	5.13	5.03	5.07
209421_at	mutS homolog 2, c	MSH2	7.59	8.00	7.82
202931_x_at	bridging integrato	BIN1	8.28	8.17	8.15
208502_s_at	paired-like homec	PITX1	6.39	6.49	6.42
213205_s_at	RAD54-like 2 (S. ce	RAD54L2	7.78	7.70	7.70
218788_s_at	SET and MYND do	SMYD3	9.07	9.04	9.16
206049_at	selectin P (granule	SELP	4.52	4.84	4.81
209934_s_at	ATPase, Ca++ tran	ATP2C1	7.40	7.43	7.52
210254_at	membrane-spann	MS4A3	4.06	3.83	3.89
218924_s_at	chitobiase, di-N-ac	CTBS	7.41	7.24	7.49
212909_at	LY6/PLAUR domai	LYPD1	5.59	5.42	5.32
205998_x_at	cytochrome P450,	CYP3A4	8.72	8.45	8.64
210121_at	UDP-Gal:betaGlcN	B3GALT2	3.56	3.55	3.42
221455_s_at	wingless-type MM	WNT3	5.64	6.04	5.63
209060_x_at	nuclear receptor c	NCOA3	8.52	8.51	8.44
207716_at	keratin 38	KRT38	4.83	4.59	4.89
206266_at	glycosylphosphati	GPLD1	4.78	4.76	4.50
219661_at	RAN binding prote	RANBP17	7.25	7.42	7.24
211658_at	peroxiredoxin 2	PRDX2	9.91	9.79	9.78
221708_s_at	unc-45 homolog A	UNC45A	8.66	8.59	8.53
213578_at	bone morphogene	BMPR1A	8.87	8.88	9.00
213419_at	amyloid beta (A4)	APBB2	8.35	8.14	8.32
215442_s_at	thyroid stimulatin	TSHR	3.51	3.47	3.55
215518_at	syntaxin binding p	STXBP5L	3.05	2.93	3.18
208788_at	ELOVL family men	ELOVL5	12.03	12.07	12.10
205322_s_at	metal-regulatory t	MTF1	7.91	7.94	7.91
204559_s_at	LSM7 homolog, U	LSM7	11.61	11.62	11.62
215897_at	mediator complex	MED25	3.47	3.43	3.36
203701_s_at	TRM1 tRNA methy	TRMT1	7.93	8.00	7.94
208746_x_at	ATP synthase, H+	ATP5L	13.65	13.64	13.67
211235_s_at	estrogen receptor	ESR1	6.29	6.36	6.33
208161_s_at	ATP-binding casse	ABCC3	10.15	10.27	10.17
207114_at	lymphocyte antige	LY6G6C	6.56	6.28	6.36
219981_x_at	zinc finger protein	ZNF587	8.80	8.79	8.86
209526_s_at	hepatoma-derived	HDGFRP3	9.80	9.74	9.90
214429_at	myotubularin rela	MTMR6	10.42	10.37	10.64
212070_at	G protein-couplec	GPR56	5.69	5.28	5.37
215520_at	pygopus homolog	PYGO1	3.67	3.74	3.49
205053_at	primase, DNA, pol	PRIM1	9.97	9.94	10.09
221740_x_at	Leucine rich repea	LRRC37A2	7.60	7.64	7.66
218947_s_at	mitochondrial pol	MTPAP	9.52	9.45	9.55
202149_at	neural precursor c	NEDD9	6.58	6.76	6.79
218377_s_at	RWD domain cont	RWDD2B	9.78	9.69	9.75
214976_at	ribosomal protein	RPL13	6.86	6.91	7.03

209341_s_at	inhibitor of kappa	IKBKB	9.05	9.04	9.20
216635_at	---	---	4.34	4.71	4.59
205019_s_at	vasoactive intestin	VIPR1	7.74	7.74	7.56
206171_at	adenosine A3 rece	ADORA3	5.95	5.82	5.90
218188_s_at	translocase of inn	TIMM13	11.63	11.50	11.67
1320_at	protein tyrosine p	PTPN21	5.29	5.30	5.20
219096_at	armadillo repeat c	ARMC7	8.18	8.24	8.13
220518_at	ABI family, memb	ABI3BP	8.01	8.24	8.22
221469_at	G protein-couplec	GPR32	5.10	5.00	5.08
205981_s_at	inhibitor of growt	ING2	8.43	8.40	8.59
216661_x_at	cytochrome P450,	CYP2C9	3.92	3.74	4.23
215048_at	zinc finger protein	ZNF280B	5.67	5.90	5.74
36475_at	glycine C-acetyltra	GCAT	8.96	8.82	8.88
216302_at	heterogeneous nu	HNRNPC /// HNRN	5.68	5.75	5.69
205474_at	cytokine receptor	CRLF3	9.76	9.75	9.68
203048_s_at	tetratricopeptide	TTC37	9.74	9.75	9.75
217501_at	cytosolic iron-sulf	CIAO1	8.10	8.11	8.06
206618_at	interleukin 18 rec	IL18R1	7.78	7.78	8.19
219845_at	BARX homeobox 1	BARX1	5.47	5.39	5.43
219500_at	cardiotrophin-like	CLCF1	7.62	7.46	7.39
220329_s_at	required for meio	RMND1	8.81	8.69	9.01
214439_x_at	bridging integrato	BIN1	8.33	8.29	8.10
204718_at	EPH receptor B6	EPHB6	5.75	5.71	5.72
205272_s_at	proline-rich prote	PRH1 /// PRH2	6.87	6.96	7.00
212348_s_at	lysine (K)-specific	KDM1A	10.30	10.30	10.26
207529_at	defensin, alpha 5,	DEFA5	5.27	5.16	5.07
49485_at	PR domain contai	PRDM4	8.70	8.71	8.84
215831_at	---	---	3.32	3.25	3.29
215468_at	hypothetical LOC6	LOC647070	3.49	3.35	3.33
212865_s_at	collagen, type XIV	COL14A1	3.15	3.31	2.98
206674_at	fms-related tyrosi	FLT3	5.72	5.66	5.39
207738_s_at	NCK-associated pr	NCKAP1	11.40	11.53	11.64
209089_at	RAB5A, member R	RAB5A	11.67	11.65	11.69
45288_at	abhydrolase doma	ABHD6	7.52	7.59	7.77
206163_at	mab-21-like 1 (C. e	MAB21L1	3.72	3.80	3.46
210964_s_at	glycogenin 2	GYG2	4.80	4.90	4.98
213796_at	small proline-rich	SPRR1A	4.10	4.10	3.98
212517_at	attractin	ATRN	8.52	8.48	8.57
200821_at	lysosomal-associa	LAMP2	9.88	10.02	10.19
206245_s_at	influenza virus NS	IVNS1ABP	9.13	8.98	9.24
213009_s_at	tripartite motif-co	TRIM37	10.98	10.93	11.00
200620_at	transmembrane p	TMEM59	11.39	11.41	11.54
216305_s_at	chromosome 2 op	C2orf3	7.79	7.86	7.89
206481_s_at	LIM domain bindi	LDB2	4.74	4.72	4.86
216012_at	---	---	4.57	4.44	4.96
207900_at	chemokine (C-C m	CCL17	6.32	5.83	6.07
210595_at	zinc finger protein	ZNF235	3.90	3.82	3.75

210927_x_at	jumping transloca	JTB		12.63	12.56	12.49
222255_at	periaxin	PRX		3.94	4.06	3.94
212017_at	family with seque	FAM168B		9.97	9.96	10.07
218139_s_at	MU-2/AP1M2 dor	MUDENG		9.83	10.00	9.92
200778_s_at	septin 2		2-Sep	9.61	9.77	9.83
209515_s_at	RAB27A, member	RAB27A		8.93	8.95	9.13
215592_at	---	---		5.09	5.22	5.24
220565_at	chemokine (C-C m	CCR10		6.23	5.80	5.95
220350_at	zinc finger proteir	ZNF235		5.08	5.14	4.95
209287_s_at	CDC42 effector pr	CDC42EP3		7.25	7.39	7.28
203654_s_at	coilin	COIL		9.35	9.39	9.36
211947_s_at	HLA-B associated	BAT2L2		8.16	8.09	8.18
220786_s_at	solute carrier fam	SLC38A4		5.13	5.78	5.34
202322_s_at	geranylgeranyl dip	GGPS1		10.14	10.15	10.19
210417_s_at	phosphatidylinosi	PI4KB		7.56	7.72	7.47
214952_at	neural cell adhesi	NCAM1		4.43	4.41	4.36
203860_at	propionyl CoA car	PCCA		7.41	7.33	7.66
212535_at	myocyte enhance	MEF2A		9.67	9.68	9.79
215157_x_at	poly(A) binding pr	PABPC1		14.48	14.49	14.48
218189_s_at	N-acetylneuramin	NANS		10.64	10.57	10.63
213529_at	zinc finger proteir	ZNF688		6.10	6.24	6.11
1007_s_at	discoidin domain	DDR1		9.34	9.44	9.22
209236_at	solute carrier fam	SLC23A2		7.63	7.55	7.66
216630_at	---	---		6.06	5.93	6.04
208670_s_at	EP300 interacting	EID1		10.30	10.42	10.29
221699_s_at	DEAD (Asp-Glu-Al	DDX50		10.89	10.90	10.99
219516_at	transient receptor	TRPV4		6.59	6.47	6.62
201509_at	isocitrate dehydro	IDH3B		9.09	8.96	9.04
213111_at	phosphoinositide	PIKFYVE		9.47	9.60	9.63
220816_at	lysophosphatidic a	LPAR3		5.67	5.62	5.62
203340_s_at	solute carrier fam	SLC25A12		8.52	8.78	8.58
210536_s_at	sperm adhesion m	SPAM1		3.72	3.78	3.90
219484_at	host cell factor C2	HCFC2		7.48	7.72	7.47
210548_at	chemokine (C-C m	CCL23		3.41	3.62	3.35
215909_x_at	misshapen-like kir	MINK1		6.90	6.72	6.98
214868_at	piwi-like 1 (Droso	PIWIL1		4.03	3.99	3.88
202281_at	cyclin G associate	GAK		8.13	8.18	8.01
211083_s_at	mitogen-activatec	MAP3K13		3.86	4.08	4.11
2028_s_at	splicing factor, arg	SFRS3		6.98	7.01	6.84
215509_s_at	budding uninhibit	BUB1		6.66	6.79	6.52
206002_at	G protein-couplec	GPR64		10.22	10.20	10.32
209992_at	6-phosphofructo-2	PFKFB2		4.42	4.29	4.48
213391_at	dpy-19-like 4 (C. e	DPY19L4		8.95	9.18	9.08
220462_at	cysteine-serine-ric	CSRNP3		4.19	4.37	4.11
201959_s_at	MYC binding prote	MYCBP2		10.23	10.20	10.38
207682_s_at	kinesin family me	KIF25		4.82	4.67	4.53
207601_at	sulfotransferase fa	SULT1B1		4.98	5.44	5.53

208276_at	---	---	4.61	4.45	4.45
216782_at	---	---	3.97	3.66	3.87
201991_s_at	kinesin family mer	KIF5B	11.34	11.40	11.52
203669_s_at	diacylglycerol O-a	DGAT1	7.51	7.67	7.44
210908_s_at	prefoldin subunit	PFDN5	12.28	12.20	12.29
56919_at	WD repeat domai	WDR48	7.77	7.68	7.96
212760_at	ubiquitin protein l	UBR2	8.65	8.88	8.84
209885_at	ras homolog gene	RHOD	6.05	5.80	6.02
217566_s_at	transglutaminase	TGM4	4.13	4.03	3.83
200891_s_at	signal sequence re	SSR1	12.19	12.29	12.28
213702_x_at	N-acylsphingosine	ASAH1	9.60	9.77	9.71
216972_at	sperm adhesion m	SPAM1	3.68	3.49	3.47
201188_s_at	inositol 1,4,5-tripl	ITPR3	7.50	7.49	7.48
210802_s_at	DIM1 dimethylade	DIMT1L	10.13	10.13	10.27
218001_at	mitochondrial ribo	MRPS2	9.43	9.29	9.24
213475_s_at	integrin, alpha L (a	ITGAL	6.94	6.86	6.91
201469_s_at	SHC (Src homolog	SHC1	7.91	7.84	7.72
214721_x_at	CDC42 effector pr	CDC42EP4	8.60	8.59	8.79
217260_x_at	Immunoglobulin h	IGHG1 /// LOC100	5.01	4.99	4.90
205012_s_at	hydroxyacylglutat	HAGH	9.37	9.38	9.35
213330_s_at	stress-induced-ph	STIP1	8.76	8.83	8.87
206959_s_at	UPF3 regulator of	UPF3A	7.52	7.55	7.55
216040_x_at	---	---	4.69	4.76	4.94
216449_x_at	heat shock protein	HSP90B1	10.83	10.83	10.66
212189_s_at	component of olig	COG4	8.97	8.87	8.87
205453_at	homeobox B2	HOXB2	5.06	4.97	4.96
210154_at	malic enzyme 2, N	ME2	8.22	8.45	8.44
215489_x_at	homer homolog 3	HOMER3	8.04	8.00	8.03
202472_at	mannose phospha	MPI	6.94	7.15	7.07
221643_s_at	arginine-glutamic	RERE	5.89	5.72	6.09
200005_at	eukaryotic transla	EIF3D	12.28	12.25	12.35
201083_s_at	BCL2-associated t	BCLAF1	8.17	8.41	8.72
217414_x_at	hemoglobin, alpha	HBA1 /// HBA2	6.30	6.36	6.34
218927_s_at	carbohydrate (cho	CHST12	8.11	8.20	8.09
215799_at	---	---	6.83	6.51	6.69
219352_at	hect domain and l	HERC6	6.64	7.07	7.06
218235_s_at	UTP11-like, U3 sm	UTP11L	10.25	10.43	10.38
207454_at	glutamate recepto	GRIK3	4.56	4.42	4.47
213771_at	interferon regulat	IRF2BP1	6.92	6.74	7.03
52651_at	collagen, type VIII	COL8A2	5.00	5.04	4.83
200010_at	ribosomal protein	RPL11	14.14	14.14	14.19
203127_s_at	serine palmitoyltr	SPTLC2	8.20	8.14	7.97
219336_s_at	activating signal c	ASCC1	8.40	8.34	8.35
209689_at	coiled-coil domain	CCDC93	7.22	7.16	7.16
206342_x_at	iduronate 2-sulfat	IDS	7.82	7.92	7.91
200058_s_at	small nuclear ribo	SNRNP200	10.49	10.40	10.50
213228_at	phosphodiesteras	PDE8B	6.75	6.71	6.69

209343_at	EF-hand domain f	EFHD1	5.15	5.07	4.97
213212_x_at	golgin A6 family-li	GOLGA6L5 /// GOI	7.05	7.33	7.36
216111_x_at	postmeiotic segre	PMS2L3	7.40	7.14	7.25
216644_at	---	---	4.89	4.88	4.66
203190_at	NADH dehydroger	NDUFS8	11.11	11.00	11.07
218252_at	cytoskeleton asso	CKAP2	10.41	10.49	10.78
207808_s_at	protein S (alpha)	PROS1	10.52	10.54	10.72
213711_at	keratin 81	KRT81	6.66	6.66	6.45
213557_at	cyclin-dependent	CDK12	6.15	6.35	6.57
205350_at	cellular retinoic ac	CRABP1	5.22	5.26	5.25
222215_at	solute carrier fam	SLC38A7	6.03	5.85	5.88
209087_x_at	melanoma cell ad	MCAM	7.40	7.63	7.56
208568_at	melanocortin 2 re	MC2R	4.68	4.83	4.68
205552_s_at	2',5'-oligoadenyla	OAS1	4.88	4.67	4.41
206372_at	myogenic factor 6	MYF6	5.03	5.12	5.04
204912_at	interleukin 10 rec	IL10RA	4.79	4.73	4.77
219910_at	FIC domain contai	FICD	6.40	6.42	6.19
205795_at	neurexin 3	NRXN3	3.99	4.13	3.90
202963_at	regulatory factor X	RFX5	9.91	9.76	9.92
202304_at	fibronectin type II	FNDC3A	9.91	9.96	10.02
215730_at	---	---	4.98	4.86	4.98
216784_at	---	---	5.19	4.83	4.99
215655_at	Glutamate recept	GRIK2	3.19	3.24	3.31
205208_at	aldehyde dehydro	ALDH1L1	7.36	7.40	7.40
204703_at	intraflagellar trans	IFT88	7.60	7.74	7.54
203480_s_at	OTU domain conta	OTUD4	10.00	9.93	10.09
221479_s_at	BCL2/adenovirus I	BNIP3L	10.43	10.36	10.44
201247_at	sterol regulatory e	SREBF2	7.98	7.92	8.11
220702_at	---	---	4.92	4.67	4.77
208428_at	transporter 2, ATF	TAP2	5.33	5.20	5.28
221671_x_at	immunoglobulin k	IGK@ /// IGKC	7.14	7.13	7.03
218007_s_at	ribosomal protein	RPS27L	12.43	12.51	12.55
210215_at	transferrin recept	TFR2	6.48	6.47	6.17
216127_at	protein disulfide is	PDIA2	5.52	5.18	5.18
213749_at	mannan-binding le	MASP1	6.51	6.62	6.58
204325_s_at	neurofibromin 1	NF1	4.89	5.08	4.99
221388_at	olfactory receptor	OR1A1	3.94	3.96	3.85
210009_s_at	golgi SNAP recept	GOSR2	7.94	7.92	7.96
206420_at	immunoglobulin s	IGSF6	4.55	4.53	4.44
222286_at	small nuclear RNA	SNAPC3	4.79	5.12	5.23
211407_at	NADH dehydroger	NDUFB7	5.43	5.41	5.41
200844_s_at	peroxiredoxin 6	PRDX6	12.69	12.59	12.65
212192_at	potassium channe	KCTD12	9.34	9.35	9.60
215792_s_at	DnaJ (Hsp40) hom	DNAJC11	8.27	8.32	8.12
221253_s_at	muted homolog (r	MUTED /// TXNDC	11.85	11.88	11.98
215068_s_at	F-box and leucine	FBXL18	7.11	7.15	7.10
204880_at	O-6-methylguanin	MGMT	5.59	5.16	5.33

214173_x_at	chromosome 19 c	C19orf2	10.32	10.40	10.40
210574_s_at	nuclear distributic	NUDC	11.08	10.95	10.99
215179_x_at	Placental growth f	PGF	9.20	9.27	9.17
212239_at	phosphoinositide-	PIK3R1	12.17	12.12	12.41
200987_x_at	proteasome (pros	PSME3	10.36	10.29	10.45
211855_s_at	solute carrier fam	SLC25A14	7.83	7.93	7.88
212499_s_at	FCF1 small subuni	FCF1 /// MAPK1IP1	9.50	9.62	9.91
215004_s_at	splicing factor 4	SF4	7.53	7.62	7.65
219423_x_at	tumor necrosis fa	TNFRSF25	5.49	4.95	5.02
213265_at	pepsinogen 3, gro	PGA3 /// PGA4 ///	4.97	4.96	4.90
203133_at	Sec61 beta subun	SEC61B	12.18	12.26	12.22
218405_at	activator of basal	ABT1	8.22	8.27	8.15
200908_s_at	ribosomal protein	RPLP2	7.01	7.03	7.24
219892_at	transmembrane 6	TM6SF1	4.58	4.91	4.67
214105_at	---	---	5.92	5.95	5.83
217779_s_at	proline-rich nucle	PNRC2	11.24	11.32	11.39
214608_s_at	eyes absent homo	EYA1	5.75	5.68	5.62
221775_x_at	ribosomal protein	RPL22	14.13	14.15	14.24
206655_s_at	glycoprotein Ib (p	GP1BB /// SEPT5	5.75	5.39	5.71
215502_at	---	---	7.08	6.80	6.86
219419_at	chromosome 18 c	C18orf22	8.34	8.38	8.50
211029_x_at	fibroblast growth	FGF18	4.78	4.90	4.67
203844_at	von Hippel-Lindau	VHL	3.51	3.46	3.51
206066_s_at	RAD51 homolog C	RAD51C	8.98	8.88	9.03
218304_s_at	oxysterol binding	OSBPL11	9.19	9.19	9.20
201599_at	ornithine aminotr	OAT	11.41	11.58	11.53
202994_s_at	fibulin 1	FBLN1	10.63	10.42	10.63
65517_at	adaptor-related p	AP1M2	6.77	6.93	6.65
211596_s_at	leucine-rich repea	LRIG1	9.29	9.10	9.35
220389_at	coiled-coil domair	CCDC81	4.51	4.21	4.16
215241_at	anoctamin 3	ANO3	3.62	3.47	3.42
208043_at	---	---	4.86	4.72	4.69
216785_at	---	---	3.67	3.71	3.77
214161_at	Oxidative stress ir	OSGIN2	3.78	4.05	3.72
207299_s_at	glutamate recepto	GRM1	3.54	3.28	3.53
217755_at	hematological anc	HN1	11.57	11.62	11.67
220363_s_at	engulfment and c	ELMO2	4.85	4.88	5.25
205976_at	FAST kinase doma	FASTKD2	5.88	6.28	6.11
219584_at	phospholipase A1	PLA1A	3.84	4.00	3.74
213585_s_at	Programmed cell	PDCD2	6.23	6.02	6.19
209936_at	RNA binding moti	RBM5	5.66	5.93	5.91
202452_at	zer-1 homolog (C.	ZER1	6.82	6.81	6.80
207461_at	---	---	3.10	3.21	3.14
207971_s_at	centrosomal prote	CEP68	7.23	7.25	7.14
219241_x_at	slingshot homolog	SSH3	7.44	7.57	7.36
208619_at	damage-specific D	DDB1	10.88	10.88	10.88
203898_at	CGRP receptor co	CRCP	8.16	8.33	8.54

215811_at	---	---	5.03	5.13	4.80
207733_x_at	pregnancy specific	PSG9	5.35	5.59	5.35
211019_s_at	lanosterol synthas	LSS	5.95	6.13	6.18
215187_at	hypothetical prote	FLJ11292	4.22	4.16	4.11
201873_s_at	ATP-binding casse	ABCE1	10.55	10.59	10.59
217352_at	---	---	6.78	6.68	6.90
201871_s_at	UBX domain prote	UBXN1	10.57	10.49	10.56
207498_s_at	cytochrome P450,	CYP2D6	6.10	5.78	6.14
207183_at	G protein-couplec	GPR19	3.78	4.14	4.13
91684_g_at	exosome compon	EXOSC4	9.07	8.91	8.96
213839_at	calmin (calponin-l	CLMN	6.60	6.31	6.26
217371_s_at	interleukin 15	IL15	4.70	4.80	4.96
204068_at	serine/threonine l	STK3	9.55	9.18	9.49
206512_at	zinc finger (CCCH	ZRSR1	5.54	5.85	5.69
220102_at	forkhead box L2	FOXL2	4.48	4.44	4.17
204053_x_at	phosphatase and	PTEN	9.81	9.71	9.77
220481_at	G protein-couplec	GPR75	4.49	4.39	4.34
207302_at	sarcoglycan, gamr	SGCG	4.13	4.16	4.26
209676_at	tissue factor path	TFPI	6.81	6.58	6.99
210349_at	calcium/calmodul	CAMK4	4.96	4.50	4.82
202907_s_at	nibrin	NBN	9.99	10.09	10.13
210434_x_at	jumping transloca	JTB	12.79	12.73	12.70
201832_s_at	USO1 vesicle dock	USO1	10.65	10.73	10.78
209032_s_at	cell adhesion mol	CADM1	8.78	8.69	9.01
204139_x_at	myeloid zinc finge	MZF1	7.43	7.32	7.34
220635_at	psoriasis susceptil	PSORS1C2	5.90	6.16	6.00
208518_s_at	period homolog 2	PER2	4.13	4.13	4.18
210896_s_at	aspartate beta-hy	ASPH	10.25	10.54	10.57
217084_at	immunoglobulin h	IGHA1 /// IGHG1 /	3.29	3.56	3.41
209080_x_at	glutaredoxin 3	GLRX3	11.75	11.72	11.77
214684_at	myocyte enhance	MEF2A	8.87	8.95	9.09
204864_s_at	interleukin 6 signa	IL6ST	6.67	6.57	6.24
222038_s_at	UTP18, small subu	UTP18	4.57	4.62	4.15
212927_at	structural mainter	SMC5	7.52	7.84	7.84
204044_at	quinolinate phosp	QPRT	5.11	5.14	4.93
207412_x_at	carboxyl ester lipa	CELP	5.49	5.46	5.20
205765_at	cytochrome P450,	CYP3A5	3.68	3.52	3.49
212724_at	Rho family GTPase	RND3	10.56	10.49	10.72
213769_at	kinase suppressor	KSR1	6.45	6.38	6.34
215774_s_at	---	---	3.85	4.14	4.15
214108_at	MYC associated fa	MAX	6.01	6.10	6.08
222024_s_at	A kinase (PRKA) a	AKAP13	8.78	8.65	8.89
221646_s_at	zinc finger, DHHC-	ZDHHC11	6.12	6.09	5.76
200710_at	acyl-CoA dehydro	ACADVL	7.99	8.07	8.04
205310_at	F-box protein 46	FBXO46	8.55	8.34	8.30
218663_at	non-SMC condens	NCAPG	9.62	9.77	9.81
209418_s_at	THO complex 5	THOC5	8.40	8.43	8.62

41644_at	SAM and SH3 don	SASH1	8.05	7.93	7.92
217941_s_at	erbb2 interacting	ERBB2IP	11.13	11.26	11.47
218951_s_at	phosphatidylinosi	PLCXD1	7.43	7.68	7.52
208174_x_at	zinc finger (CCCH	ZRSR2	7.36	7.59	7.65
221934_s_at	DALR anticodon b	DALRD3	8.83	8.79	8.97
207005_s_at	B-cell CLL/lympho	BCL2	5.78	5.29	5.66
214954_at	sushi domain cont	SUSD5	8.99	8.89	9.26
202114_at	sorting nexin 2	SNX2	9.18	9.24	9.20
212602_at	WD repeat and FY	WDFY3	7.43	7.51	7.58
217178_at	retinoic acid recep	RARG	5.53	5.50	5.28
220866_at	ADAM metallope	ADAMTS6	4.83	5.37	5.00
205587_at	FGFR1 oncogene	FGFR1OP	4.85	4.92	4.80
221770_at	ribulose-5-phosph	RPE	8.80	8.87	9.10
160020_at	matrix metallope	MMP14	6.93	7.04	6.81
216257_at	serpin peptidase i	SERPINB13	4.46	4.15	4.48
201723_s_at	UDP-N-acetyl-alph	GALNT1	10.29	10.43	10.24
201565_s_at	inhibitor of DNA b	ID2	8.54	8.64	8.84
207211_at	ubiquitin specific	USP2	5.45	5.65	5.49
212096_s_at	microtubule assoc	MTUS1	4.31	4.77	4.33
214828_s_at	ribosomal RNA pr	RRP7A /// RRP7B	7.36	7.22	7.17
221630_s_at	DEAD (Asp-Glu-Al	DDX4	3.82	4.02	3.80
214512_s_at	SUB1 homolog (S.	SUB1	12.81	12.83	12.85
212440_at	small nuclear ribo	SNRNP27	9.60	9.57	9.73
209381_x_at	splicing factor 3a,	SF3A2	7.47	7.19	7.41
200673_at	lysosomal protein	LAPTM4A	12.02	12.08	12.13
205170_at	signal transducer	STAT2	5.44	5.74	5.37
206669_at	glutamate decarb	GAD1	4.15	4.06	4.18
212668_at	SMAD specific E3	SMURF1	4.59	4.45	4.44
204279_at	proteasome (pros	PSMB9	10.26	10.18	10.09
211728_s_at	hyaluronoglucosa	HYAL3	6.17	6.19	5.63
202394_s_at	ATP-binding casse	ABCF3	8.39	8.33	8.16
214163_at	heat shock protei	HSPB11	4.30	4.92	4.48
218508_at	DCP1 decapping e	DCP1A	8.13	8.23	8.25
201136_at	proteolipid protei	PLP2	12.41	12.32	12.33
203665_at	heme oxygenase (	HMOX1	7.29	7.01	6.93
212790_x_at	ribosomal protein	RPL13A	15.03	15.01	15.02
213572_s_at	serpin peptidase i	SERPINB1	9.12	9.02	9.10
207940_x_at	cannabinoid recep	CNR1	6.38	6.58	6.34
214728_x_at	SWI/SNF related,	SMARCA4	10.01	9.92	9.97
214376_at	---	---	4.03	4.03	3.88
201679_at	serrate RNA effec	SRRT	6.45	6.13	6.20
205342_s_at	sulfotransferase f	SULT1C2	3.37	3.67	3.44
209828_s_at	interleukin 16 (lyn	IL16	4.31	4.23	4.34
204651_at	nuclear respirator	NRF1	7.21	7.27	7.31
209910_at	solute carrier fam	SLC25A16	4.43	4.24	4.52
201994_at	mortality factor 4	MORF4L2	13.04	13.19	13.21
201234_at	integrin-linked kin	ILK	9.25	9.21	9.24

214332_s_at	Ts translation elor	TSFM	6.09	5.98	6.08
209056_s_at	CDC5 cell division	CDC5L	9.74	9.90	9.91
221658_s_at	interleukin 21 rec	IL21R	6.10	6.32	6.09
207426_s_at	tumor necrosis fa	TNFSF4	4.79	4.35	4.65
213748_at	tripartite motif-co	TRIM66	6.60	6.74	6.50
206780_at	glutamate decarb	GAD2	4.85	4.73	4.65
207718_x_at	cytochrome P450,	CYP2A7	5.37	5.54	5.15
215948_x_at	zinc finger, MYM-1	ZMYM5	5.98	6.10	6.20
200081_s_at	ribosomal protein	RPS6	14.74	14.70	14.71
210008_s_at	mitochondrial ribo	MRPS12	7.87	7.81	7.80
216567_at	---	---	5.67	5.44	5.55
202802_at	protein kinase, cA	PRKACA	8.11	8.06	8.01
219775_s_at	complexin 3 /// le	CPLX3 /// LMAN1L	5.80	5.53	5.43
211972_x_at	ribosomal protein	RPLP0	14.94	14.94	14.96
204947_at	E2F transcription f	E2F1	8.57	8.61	8.40
201708_s_at	nipsnap homolog	NIPSNAP1	7.91	8.10	7.87
220186_s_at	cadherin-related f	CDHR2	6.40	6.34	6.17
208323_s_at	annexin A13	ANXA13	4.48	4.13	3.96
221337_s_at	ADAM metallopep	ADAM29	3.45	3.60	3.48
218912_at	GRIP and coiled-co	GCC1	9.31	9.23	9.16
209053_s_at	Wolf-Hirschhorn s	WHSC1	9.79	9.90	9.93
207438_s_at	snurportin 1	SNUPN	9.92	9.91	9.84
208836_at	ATPase, Na+/K+ tr	ATP1B3	12.57	12.61	12.80
209796_s_at	canopy 2 homolog	CNPY2	10.54	10.66	10.56
205026_at	signal transducer	STAT5B	5.64	5.53	5.64
215969_at	---	---	3.82	3.97	3.79
219262_at	suppressor of vari	SUV39H2	6.01	6.09	5.94
203756_at	Rho guanine nucle	ARHGEF17	8.15	8.13	8.06
213091_at	CREB regulated tra	CRTC1	8.61	8.52	8.13
212073_at	casein kinase 2, al	CSNK2A1 /// CSNK	9.43	9.37	9.50
212188_at	potassium channe	KCTD12	8.97	9.07	9.18
209377_s_at	high mobility grou	HMGN3	12.09	12.17	12.13
220321_s_at	coiled-coil domair	CCDC121	3.77	3.70	3.80
207666_x_at	synovial sarcoma,	SSX3	3.67	3.95	3.85
217644_s_at	son of sevenless h	SOS2	4.84	5.01	4.68
212919_at	DCP2 decapping e	DCP2	8.94	9.04	9.15
201896_s_at	proline/serine-ricl	PSRC1	9.90	9.79	9.89
216386_at	docking protein 1-	LOC220077	4.19	3.97	3.87
219966_x_at	BTG3 associated n	BANP	9.06	9.19	9.19
214426_x_at	chromatin assemb	CHAF1A	9.19	9.13	9.17
215704_at	filaggrin	FLG	3.49	3.37	3.47
221436_s_at	cell division cycle	CDCA3	10.03	10.00	10.00
217981_s_at	fracture callus 1 h	FXC1	8.49	8.53	8.51
220280_s_at	ankyrin repeat an	ANKMY1	5.24	5.19	5.44
216834_at	regulator of G-pro	RGS1	3.28	3.10	3.14
206021_at	SCAN domain con	SCAND2	4.94	4.46	4.34
200753_x_at	splicing factor, arg	SFRS2	10.10	10.26	10.25

206024_at	4-hydroxyphenylp	HPD	4.32	4.23	4.30
214823_at	zinc finger protein	ZNF204P	5.04	5.00	5.03
209164_s_at	cytochrome b-561	CYB561	9.11	9.17	9.12
218280_x_at	histone cluster 2,	HIST2H2AA3 /// HI	8.63	8.45	8.57
208966_x_at	interferon, gamm	IFI16	10.59	10.52	10.83
210834_s_at	prostaglandin E re	PTGER3	4.14	3.91	3.77
AFFX-BioB-5_at	---	---	9.39	9.33	9.24
222353_at	LIM domains cont	LIMD1	3.62	3.79	3.51
214289_at	Proteasome (pros	PSMB1	4.75	4.64	4.75
220804_s_at	tumor protein p73	TP73	5.79	5.71	5.60
204359_at	fibronectin leucin	FLRT2	8.86	8.68	8.83
207709_at	protein kinase, AM	PRKAA2	4.05	3.84	4.19
203112_s_at	Wolf-Hirschhorn s	WHSC2	8.39	8.18	8.36
208768_x_at	ribosomal protein	RPL22	14.13	14.13	14.14
221591_s_at	family with seque	FAM64A	9.46	9.45	9.50
210948_s_at	lymphoid enhance	LEF1	5.44	5.03	5.01
201397_at	phosphoglycerate	PHGDH	10.32	9.95	10.07
214806_at	bicaudal D homol	BICD1	7.56	7.43	7.45
204201_s_at	protein tyrosine p	PTPN13	7.60	7.47	7.73
205415_s_at	ataxin 3	ATXN3	7.34	7.47	7.47
210809_s_at	periostin, osteobl	POSTN	3.61	3.63	3.58
207834_at	fibulin 1	FBLN1	5.63	5.63	5.60
210446_at	GATA binding pro	GATA1	6.44	6.51	6.32
205278_at	glutamate decarb	GAD1	3.92	3.76	3.68
209367_at	syntaxin binding p	STXBP2	6.33	6.23	6.25
203157_s_at	glutaminase	GLS	7.19	7.10	7.21
220180_at	coiled-coil domair	CCDC68	7.26	7.24	7.54
206426_at	melan-A	MLANA	3.69	3.91	3.73
216299_s_at	X-ray repair comp	XRCC3	5.66	5.89	5.77
202559_x_at	chromosome 1 op	C1orf77	10.76	10.69	10.57
204169_at	IMP (inosine 5'-m	IMPDH1	9.29	9.28	9.16
205819_at	macrophage recep	MARCO	7.25	6.84	7.04
209112_at	cyclin-dependent	CDKN1B	9.67	9.61	9.87
218033_s_at	stannin	SNN	4.75	4.79	4.92
203981_s_at	ATP-binding casse	ABCD4	7.21	7.27	7.31
201636_at	fragile X mental re	FXR1	9.98	10.02	10.11
204001_at	small nuclear RNA	SNAPC3	7.97	8.03	8.00
222372_at	---	---	5.37	5.26	5.36
201011_at	ribophorin I	RPN1	11.32	11.27	11.39
201805_at	protein kinase, AM	PRKAG1	10.12	10.06	10.08
220940_at	ankyrin repeat do	ANKRD36B	7.57	8.04	7.75
209652_s_at	placental growth f	PGF	5.16	5.15	5.04
220057_at	X antigen family, r	XAGE1A /// XAGE1	11.46	11.27	11.42
219608_s_at	F-box protein 38	FBXO38	4.17	4.17	4.23
203800_s_at	mitochondrial ribo	MRPS14	11.02	11.02	11.01
206738_at	apolipoprotein C-I	APOC4	5.30	5.18	5.22
216732_at	---	---	4.00	4.15	3.76

204860_s_at	NLR family, apopt	NAIP	6.01	6.32	6.36
201385_at	DEAH (Asp-Glu-Al	DHX15	12.31	12.44	12.35
212180_at	v-crk sarcoma viru	CRKL	10.34	10.20	10.25
210171_s_at	cAMP responsive	CREM	4.64	4.55	4.63
202049_s_at	zinc finger, MYM-	ZMYM4	8.48	8.58	8.62
208112_x_at	EH-domain contai	EHD1	8.07	7.96	8.05
219115_s_at	interleukin 20 rec	IL20RA	3.47	3.01	3.37
216314_at	cysteine-rich secr	CRISP1	3.11	3.16	3.18
204951_at	ras homolog gene	RHOH	5.51	5.63	5.39
210959_s_at	steroid-5-alpha-re	SRD5A1	8.13	8.37	8.45
208259_x_at	interferon, alpha	IFNA7	4.46	4.69	4.84
222016_s_at	zinc finger protein	ZNF323	6.57	6.78	6.65
201768_s_at	clathrin interactor	CLINT1	8.70	8.59	8.61
211222_s_at	huntingtin-associa	HAP1	6.97	7.14	7.10
206310_at	serine peptidase i	SPINK2	4.71	4.82	4.72
216544_at	RNA binding moti	RBMY2FP	4.59	4.49	4.63
201464_x_at	jun oncogene	JUN	10.56	10.61	10.64
205747_at	cerebellin 1 precu	CBLN1	4.13	4.25	4.11
207250_at	SIX homeobox 6	SIX6	4.00	3.85	4.00
204292_x_at	serine/threonine	STK11	7.26	7.06	7.19
205300_s_at	small nuclear ribo	SNRNP35	7.62	7.44	7.43
212693_at	MDN1, midasin ho	MDN1	9.04	9.07	9.09
203207_s_at	mitochondrial fiss	MTFR1	9.22	9.25	9.35
219490_s_at	DNA cross-link rep	DCLRE1B	7.36	7.50	7.34
220408_x_at	family with seque	FAM48A	9.11	9.05	9.05
215565_at	hypothetical prot	LOC100289053	3.50	3.49	3.38
214963_at	nucleoporin 160kl	NUP160	6.25	6.42	6.34
216913_s_at	ribosomal RNA pr	RRP12	9.18	9.00	9.05
206303_s_at	nudix (nucleoside	NUDT4	9.00	9.03	8.99
201407_s_at	protein phosphata	PPP1CB	10.58	10.67	11.14
207108_s_at	Nipped-B homolog	NIPBL	7.91	7.91	7.94
207795_s_at	killer cell lectin-lik	KLRD1	4.78	4.88	4.94
217709_at	---	---	3.21	3.15	3.09
220125_at	dynein, axonemal	DNAI1	6.02	5.90	5.65
202440_s_at	suppression of tur	ST5	7.51	7.61	7.45
217586_x_at	---	---	7.91	8.14	8.07
216228_s_at	WD repeat and HI	WDHD1	7.19	7.23	7.57
218287_s_at	eukaryotic transla	EIF2C1	8.83	8.82	8.63
220183_s_at	nudix (nucleoside	NUDT6	7.85	7.70	7.98
204668_at	ring finger protein	RNF24	5.69	5.44	5.53
220167_s_at	TP53 target 3 ///	TP53TG3 /// TP53	6.95	7.01	6.76
207639_at	frizzled homolog 9	FZD9	5.29	5.45	5.25
221659_s_at	myosin, light chai	MYL10	5.46	5.58	5.37
36552_at	C2 calcium-depen	C2CD3	7.83	7.77	7.75
210155_at	myocilin, trabecul	MYOC	4.58	4.60	4.38
219351_at	trafficking protein	TRAPPC2	8.89	8.93	9.13
213090_s_at	TAF4 RNA polyme	TAF4	8.03	8.18	8.26

221197_s_at	choline O-acetyltr	CHAT	4.96	4.97	5.00
209288_s_at	CDC42 effector pr	CDC42EP3	9.74	9.63	9.80
217885_at	importin 9	IPO9	9.85	9.92	9.92
218548_x_at	testis expressed 2	TEX264	8.58	8.53	8.65
212477_at	---	---	4.15	3.77	3.99
218880_at	FOS-like antigen 2	FOSL2	6.97	7.00	6.99
211608_at	T cell receptor bet	TRBV10-2	5.63	5.68	5.34
206260_at	transglutaminase	TGM4	4.32	4.50	4.65
207749_s_at	protein phosphata	PPP2R3A	6.82	7.06	6.90
201439_at	golgi brefeldin A r	GBF1	7.67	7.62	7.79
206637_at	purinergic recepto	P2RY14	3.40	3.37	3.26
202992_at	complement com	C7	3.57	3.79	3.69
208956_x_at	deoxyuridine tripl	DUT	13.19	13.26	13.27
212157_at	syndecan 2	SDC2	7.95	7.93	8.13
202077_at	NADH dehydroger	NDUFAB1	13.26	13.27	13.25
217465_at	NCK-associated pr	NCKAP1	4.61	4.64	4.62
37079_at	nuclear undecapr	NUS1P3	5.44	5.65	6.04
206238_s_at	YY1 associated fac	YAF2	8.08	8.06	8.08
201514_s_at	GTPase activating	G3BP1	10.11	10.17	10.14
214214_s_at	complement com	C1QBP	11.36	11.34	11.43
201928_at	plakophilin 4	PKP4	9.97	9.87	10.01
212557_at	zinc finger proteir	ZNF451	9.53	9.57	9.78
206096_at	zinc finger proteir	ZNF35	6.84	6.91	7.07
221400_at	myosin IIIA	MYO3A	3.92	3.92	4.00
214197_s_at	SET domain, bifur	SETDB1	7.02	7.01	6.95
207123_s_at	matrilin 4	MATN4	6.31	6.17	5.95
221537_at	plexin A1	PLXNA1	8.55	8.24	8.25
209523_at	TAF2 RNA polyme	TAF2	9.89	9.92	10.04
203451_at	LIM domain bindi	LDB1	7.65	7.57	7.53
213232_at	KIAA1467	KIAA1467	4.20	4.09	4.04
201202_at	proliferating cell r	PCNA	11.58	11.68	11.66
221000_s_at	Kazal-type serine	KAZALD1	7.47	7.55	7.29
204145_at	FSHD region gene	FRG1	10.45	10.40	10.47
211718_at	hypothetical prot	MGC2889	3.64	3.88	3.88
209524_at	hepatoma-derivec	HDGFRP3	9.89	9.84	10.01
212349_at	protein O-fucosylt	POFUT1	8.17	8.22	8.24
48612_at	NEDD4 binding pr	N4BP1	9.03	9.14	8.98
205613_at	synaptotagmin XV	SYT17	5.51	5.76	5.42
213246_at	chromosome 14 c	C14orf109	8.90	8.98	9.12
209128_s_at	squamous cell car	SART3	8.72	8.80	8.78
202745_at	ubiquitin specific	USP8	9.05	9.14	9.21
218537_at	host cell factor C1	HCFC1R1	8.62	8.53	8.61
221924_at	zinc finger, MIZ-ty	ZMIZ2	6.89	6.99	6.74
206594_at	PAS domain conta	PASK	5.96	6.02	6.23
219027_s_at	myosin IXA	MYO9A	9.09	9.03	9.17
221303_at	protocadherin bet	PCDHB1	4.20	4.37	4.10
211566_x_at	brain and reprodu	BRE	8.68	8.53	8.62

206691_s_at	protein disulfide is	PDIA2	5.97	5.74	5.79
217347_at	---	---	8.59	8.54	8.76
218702_at	seryl-tRNA synthe	SARS2	7.34	7.18	7.34
212423_at	zinc finger, CCHC (	ZCCHC24	8.12	8.35	8.18
220946_s_at	SET domain conta	SETD2	6.43	6.49	6.58
217957_at	chromosome 16 c	C16orf80	10.35	10.40	10.31
217359_s_at	neural cell adhesi	NCAM1	3.82	3.79	3.86
206017_at	KIAA0319	KIAA0319	5.54	5.41	5.54
200864_s_at	RAB11A, member	RAB11A	9.66	9.87	9.98
209994_s_at	ATP-binding casse	ABCB1 /// ABCB4	5.18	5.14	5.36
207662_at	T-box 1	TBX1	5.29	5.18	5.15
216656_at	---	---	6.03	6.21	5.90
201794_s_at	Smg-7 homolog, r	SMG7	9.16	9.11	9.13
211950_at	ubiquitin protein l	UBR4	9.03	9.02	9.06
204085_s_at	ceroid-lipofuscino	CLN5	7.69	7.84	7.90
209767_s_at	glycoprotein Ib (p	GP1BB /// SEPT5	6.26	5.86	5.93
221568_s_at	lin-7 homolog C (C	LIN7C	10.75	10.86	11.03
209517_s_at	ash2 (absent, sma	ASH2L	9.76	9.63	9.72
210607_at	fms-related tyrosi	FLT3LG	5.02	5.32	5.22
209842_at	SRY (sex determin	SOX10	5.10	4.99	4.94
209101_at	connective tissue	CTGF	8.54	8.79	8.70
203343_at	UDP-glucose 6-de	UGDH	9.10	9.41	9.47
210853_at	sodium channel, v	SCN11A	3.64	3.95	3.66
209469_at	glycoprotein M6A	GPM6A	6.15	6.05	6.39
216233_at	CD163 molecule	CD163	3.80	3.64	3.65
221145_at	---	---	3.10	3.20	2.96
220621_at	forkhead box E3	FOXE3	4.83	4.75	4.70
214682_at	GPS, PLAT and tra	LOC399491	7.31	7.21	7.05
220208_at	ADAM metallope	ADAMTS13	6.65	6.55	6.53
207404_s_at	5-hydroxytryptam	HTR1E	5.64	5.91	5.65
203005_at	lymphotoxin beta	LTBR	9.32	9.23	9.38
221462_x_at	kallikrein-related	KLK15	5.21	5.20	5.05
208671_at	serine incorporat	SERINC1	10.65	10.69	10.86
33778_at	TBC1 domain fam	TBC1D22A	9.06	9.11	9.20
218433_at	pantothenate kin	PANK3	7.69	7.71	7.87
216542_x_at	immunoglobulin h	IGHA1 /// IGHG1 /	6.04	5.96	6.10
213277_at	zinc finger protein	ZFP36L1	3.81	3.87	3.90
206846_s_at	histone deacetyla	HDAC6	7.93	8.12	8.15
218294_s_at	nucleoporin 50kD	NUP50	9.70	9.78	9.87
213333_at	malate dehydroge	MDH2	8.54	8.75	8.67
220916_at	---	---	4.56	4.51	4.31
206295_at	interleukin 18 (int	IL18	9.08	9.25	9.48
221895_at	motile sperm dom	MOSPD2	8.65	8.78	8.84
203846_at	tripartite motif-co	TRIM32	8.86	8.81	8.89
209563_x_at	calmodulin 1 (pho	CALM1	12.14	12.22	12.18
207168_s_at	H2A histone famil	H2AFY	12.47	12.52	12.55
210418_s_at	isocitrate dehydro	IDH3B	9.72	9.63	9.78

208801_at	signal recognition	SRP72	12.09	12.02	12.10
204075_s_at	KIAA0562	KIAA0562	5.49	5.43	5.56
208752_x_at	nucleosome asser	NAP1L1	13.49	13.48	13.51
215219_at	dopey family men	DOPEY2	4.46	4.58	4.12
220993_s_at	G protein-couplec	GPR63	6.41	6.56	6.27
215813_s_at	prostaglandin-enc	PTGS1	6.84	6.77	6.90
209040_s_at	proteasome (pros	PSMB8	10.38	10.25	10.40
216803_at	PDZ and LIM dom	PDLIM5	3.69	3.59	3.52
217453_at	---	---	5.65	5.43	5.65
202607_at	N-deacetylase/N-s	NDST1	7.84	7.76	7.66
220033_at	---	---	6.97	7.15	7.21
220069_at	tubulin, alpha 8	TUBA8	6.17	6.00	5.75
214190_x_at	golgi-associated, g	GGA2	5.41	5.51	5.51
205527_s_at	gem (nuclear orga	GEMIN4	8.26	8.04	8.20
212539_at	chromodomain he	CHD1L	9.97	9.96	10.03
AFFX-DapX-M_a	---	---	10.37	10.21	10.22
219273_at	cyclin K	CCNK	6.10	5.83	5.99
207345_at	follistatin	FST	3.85	3.91	3.69
205984_at	corticotropin rele	CRHBP	4.31	4.11	3.99
208909_at	ubiquinol-cytochr	UQCRFS1	12.51	12.59	12.58
206201_s_at	mesenchyme hom	MEOX2	3.29	3.12	3.13
201090_x_at	tubulin, alpha 1b	TUBA1B	14.47	14.50	14.45
208208_at	myosin, heavy cha	MYH13	5.29	4.74	4.89
203601_s_at	zinc finger and BT	ZBTB17	5.94	5.86	6.07
217908_s_at	DDB1 and CUL4 as	DCAF6	9.88	9.95	10.06
64418_at	synergin, gamma	SYNRG	8.71	8.59	8.72
221256_s_at	haloacid dehaloge	HDHD3	6.03	6.21	6.27
221970_s_at	nucleolar protein	NOL11	11.02	11.11	11.19
212835_at	family with seque	FAM175B	8.54	8.70	8.60
221396_at	taste receptor, typ	TAS2R7	3.95	3.74	3.70
217697_at	---	---	3.60	3.49	3.51
207609_s_at	cytochrome P450,	CYP1A2	6.02	5.82	5.70
214492_at	sarcoglycan, delta	SGCD	4.40	4.24	4.20
AFFX-r2-P1-cre-!	---	---	14.46	14.46	14.47
209625_at	phosphatidylinosi	PIGH	7.87	7.80	8.09
204260_at	chromogranin B (s	CHGB	4.40	4.31	4.23
220352_x_at	hypothetical LOC6	FLJ42627	7.16	7.36	7.35
221518_s_at	ubiquitin specific		USP47	10.68	10.71	10.76
208293_x_at	chorionic somato	CSHL1	4.05	3.85	3.83
203431_s_at	Rho GTPase activa	ARHGAP32	7.46	7.49	7.64
221423_s_at	Yip1 domain famil	YIPF5	8.95	9.09	9.20
218589_at	lysophosphatidic a	LPAR6	6.13	6.26	6.39
201606_s_at	PWP1 homolog (S	PWP1	11.34	11.40	11.39
205836_s_at	YTH domain conta	YTHDC2	6.48	6.77	6.75
220345_at	leucine rich repea	LRRTM4	3.40	3.37	3.28
217247_at	---	---	5.13	4.89	4.89
213799_s_at	protein tyrosine p	PTPRA	6.75	6.72	6.92

213654_at	TAF5-like RNA pol	TAF5L	7.58	7.48	7.55
205463_s_at	platelet-derived g	PDGFA	9.62	9.60	9.69
217924_at	chromosome 6 op	C6orf106	7.68	7.80	7.68
215137_at	---	---	7.17	7.09	7.14
221519_at	F-box and WD rep	FBXW4	8.18	8.05	8.23
200732_s_at	protein tyrosine p	PTP4A1	10.86	10.90	10.97
216126_at	---	---	5.04	5.11	5.03
217612_at	translocase of inn	TIMM50	6.47	6.27	6.36
207560_at	solute carrier fam	SLC28A1	5.62	5.28	5.42
215612_at	---	---	4.17	4.47	4.53
219043_s_at	phosducin-like 3 p	LOC285359 /// PD	9.23	9.23	9.27
205887_x_at	mutS homolog 3 (	MSH3	6.13	6.29	6.20
202568_s_at	MAP/microtubule	MARK3	9.62	9.40	9.63
202777_at	soc-2 suppressor c	SHOC2	10.53	10.52	10.58
210760_x_at	thyroid hormone	TRIP11	5.51	5.54	5.70
209107_x_at	nuclear receptor c	NCOA1	7.69	7.60	7.89
211301_at	potassium voltage	KCND3	5.82	5.58	5.44
205857_at	solute carrier fam	SLC18A2	4.23	4.55	4.12
220880_at	---	---	4.00	3.92	3.83
202729_s_at	latent transformir	LTBP1	5.57	5.63	5.55
207154_at	deiodinase, iodotl	DIO3	8.01	7.87	7.57
210576_at	cytochrome P450,	CYP4F8	3.75	3.81	3.62
209332_s_at	MYC associated fa	MAX	10.98	10.89	11.00
220328_at	polyhomeotic hor	PHC3	5.30	4.99	5.15
207231_at	DAZ interacting pr	DZIP3	6.55	7.14	7.12
216098_s_at	5-hydroxytryptam	HTR7 /// HTR7P1	3.71	3.47	3.80
207559_s_at	zinc finger, MYM-1	ZMYM3	7.28	7.22	6.98
204841_s_at	early endosome a	EEA1	6.34	6.28	6.40
219340_s_at	ceroid-lipofuscino	CLN8	7.13	7.13	7.01
215292_s_at	megakaryoblastic	MKL1	6.76	6.50	6.50
203038_at	protein tyrosine p	PTPRK	9.84	9.65	9.85
214622_at	cytochrome P450,	CYP21A2	6.46	6.35	6.43
201853_s_at	cell division cycle	CDC25B	11.32	11.27	11.22
208399_s_at	endothelin 3	EDN3	5.35	5.43	5.26
208085_s_at	Rho GTPase activa	ARHGAP6	4.22	3.81	4.23
213644_at	coiled-coil domair	CCDC46	6.84	6.95	7.00
205830_at	calmegin	CLGN	5.66	5.63	5.91
221184_at	---	---	3.97	4.33	4.03
217265_at	plasma membrane	PLLP	4.33	4.17	4.24
221153_s_at	---	---	3.44	3.32	3.38
221269_s_at	SH3 domain bindi	SH3BGRL3	9.80	9.81	9.79
207040_s_at	suppression of tur	ST13	11.96	11.93	12.04
218934_s_at	heat shock 27kDa	HSPB7	5.81	5.71	5.47
208848_at	alcohol dehydroge	ADH5	9.90	9.94	9.87
221381_s_at	mortality factor 4	MORF4 /// MORF4	11.29	11.45	11.65
211692_s_at	BCL2 binding com	BBC3	7.81	7.41	7.72
213518_at	protein kinase C, i	PRKCI	10.11	10.14	10.24

211689_s_at	transmembrane p	TMPRSS2	3.74	3.61	3.59
211076_x_at	Atrophin 1	ATN1	5.22	5.31	4.90
215207_x_at	nuclear undecapr	NUS1 /// NUS1P3	7.48	7.59	7.66
211548_s_at	hydroxyprostaglar	HPGD	4.16	4.63	4.48
203908_at	solute carrier fam	SLC4A4	3.73	3.70	3.59
216578_at	---	---	5.63	5.60	5.67
205809_s_at	Wiskott-Aldrich sy	WASL	9.28	9.26	9.57
215046_at	chromosome 2 op	C2orf67	3.67	3.96	3.84
216878_x_at	B1 for mucin	HAB1	5.86	5.95	5.92
204568_at	KIAA0831	KIAA0831	8.21	8.35	8.33
207660_at	dystrophin	DMD	3.63	3.85	3.69
212502_at	2-aminoethanethi	ADO	9.64	9.61	9.77
214890_s_at	family with seque	FAM149A	3.96	3.94	3.61
204227_s_at	thymidine kinase :	TK2	7.53	7.55	7.52
206832_s_at	sema domain, imr	SEMA3F	5.47	5.39	5.42
200868_s_at	ring finger proteir	RNF114	8.48	8.41	8.60
220019_s_at	zinc finger proteir	ZNF224	4.74	4.62	4.56
203841_x_at	microtubule-asso	MAPRE3	4.34	4.43	4.53
212143_s_at	insulin-like growtl	IGFBP3	6.40	6.03	6.22
210710_at	angiogenic factor	AGGF1	6.03	5.98	5.94
203904_x_at	CD82 molecule	CD82	9.04	8.66	8.83
215771_x_at	ret proto-oncoger	RET	6.03	6.23	5.99
208865_at	casein kinase 1, al	CSNK1A1	12.06	12.03	12.12
209218_at	squalene epoxida:	SQLE	11.49	11.64	11.55
220371_s_at	solute carrier fam	SLC12A9	7.77	7.78	7.76
208431_s_at	tubby homolog (n	TUB	4.43	4.46	4.25
209121_x_at	nuclear receptor s	NR2F2	8.81	8.86	8.94
211773_s_at	zinc finger with KF	ZKSCAN3	6.51	6.43	6.44
204355_at	DEAH (Asp-Glu-Al	DHX30	8.80	8.74	8.82
207621_s_at	phosphatidylethar	PEMT	7.71	7.70	7.57
207884_at	guanylate cyclase	GUCY2D	6.25	5.91	6.11
216840_s_at	laminin, alpha 2	LAMA2	5.44	5.30	5.38
219299_at	tRNA methyltrans	TRMT12	8.67	8.82	8.73
202639_s_at	RAN binding prote	RANBP3	8.09	7.97	7.79
219021_at	ring finger proteir	RNF121	7.82	7.51	7.62
200743_s_at	tripeptidyl peptid	TPP1	9.34	9.34	9.25
203092_at	translocase of inn	TIMM44	9.26	9.07	9.29
201133_s_at	praja ring finger 2	PJA2	9.35	9.42	9.55
202200_s_at	SFRS protein kinas	SRPK1	10.78	10.85	10.95
214349_at	---	---	6.47	6.39	6.24
218050_at	ubiquitin-fold mo	UFM1	10.65	10.65	10.87
204278_s_at	estrogen receptor	EBAG9	9.72	9.66	9.70
214627_at	eosinophil peroxi	EPX	4.81	4.78	4.80
209663_s_at	integrin, alpha 7	ITGA7	5.07	5.17	4.77
203002_at	angiomotin like 2	AMOTL2	10.91	10.75	10.93
221915_s_at	RAN binding prote	RANBP1	7.56	7.49	7.20
219815_at	galactose-3-O-sulf	GAL3ST4	6.67	6.50	6.64

218367_x_at	ubiquitin specific	USP21	7.07	7.10	7.18
209247_s_at	ATP-binding casse	ABCF2	8.65	8.72	8.54
205779_at	receptor (G protei	RAMP2	5.97	6.08	6.09
202954_at	ubiquitin-conjuga	UBE2C	11.80	11.69	11.81
215443_at	thyroid stimulatin	TSHR	3.79	3.80	3.74
218185_s_at	armadillo repeat c	ARMC1	10.15	10.24	10.43
207835_at	fibulin 1	FBLN1	7.07	7.26	7.06
210619_s_at	hyaluronoglucosa	HYAL1	4.96	5.06	4.88
205004_at	NFKB repressing fa	NKRF	9.51	9.54	9.58
AFFX-r2-Bs-dap-	---	---	11.37	11.27	11.29
220733_at	solute carrier fam	SLC26A1	6.16	5.80	6.10
220837_at	---	---	3.17	3.03	3.08
205548_s_at	BTG family, memb	BTG3	9.65	9.68	9.88
214741_at	zinc finger protein	ZNF131	9.82	9.83	9.83
204180_s_at	zinc finger and BT	ZBTB43	8.18	8.14	8.18
219947_at	C-type lectin dom	CLEC4A	4.40	4.66	4.21
202726_at	ligase I, DNA, ATP-	LIG1	7.93	7.94	7.81
218958_at	chromosome 19 c	C19orf60	8.76	8.59	8.64
200605_s_at	protein kinase, cA	PRKAR1A	11.41	11.41	11.50
215116_s_at	dynamin 1	DNM1	5.70	5.28	5.32
210992_x_at	Fc fragment of IgG	FCGR2C	5.74	5.66	6.01
205531_s_at	glutaminase 2 (liv	GLS2	5.26	5.22	5.18
204495_s_at	chromosome 15 c	C15orf39	8.08	8.00	7.95
202314_at	cytochrome P450,	CYP51A1	11.00	11.10	11.44
221312_at	glucagon-like pep	GLP2R	3.72	3.84	3.80
214175_x_at	PDZ and LIM dom	PDLIM4	9.09	9.15	9.10
219896_at	calcyon neuron-sp	CALY	4.20	4.22	4.00
203675_at	nucleobindin 2	NUCB2	9.05	9.10	9.27
201779_s_at	ring finger protein	RNF13	10.17	10.16	10.14
202228_s_at	neuroplastin	NPTN	11.68	11.82	11.85
214993_at	aspartate beta-hy	ASPHD1	8.75	8.60	8.63
212255_s_at	ATPase, Ca++ tran	ATP2C1	9.39	9.27	9.35
213389_at	zinc finger protein	ZNF592	7.66	7.74	7.63
34187_at	RNA binding moti	RBMS2	7.66	7.66	7.47
205657_at	3-hydroxyanthran	HAAO	6.69	6.76	6.57
205607_s_at	SCY1-like 3 (S. cer	SCYL3	7.60	7.74	7.61
221214_s_at	nasal embryonic L	NELF	8.64	8.58	8.78
207394_at	zinc finger protein	ZNF137	4.46	4.66	4.41
208702_x_at	amyloid beta (A4)	APLP2	8.73	8.75	8.83
213272_s_at	transmembrane p	TMEM159	8.59	8.40	8.48
64408_s_at	calmodulin-like 4	CALML4	7.73	7.47	7.70
211514_at	dual serine/threo	DSTYK	5.22	4.98	4.85
218279_s_at	histone cluster 2,	HIST2H2AA3	5.62	5.36	5.24
213534_s_at	PAS domain conta	PASK	6.64	6.57	6.34
214368_at	RAS guanyl releas	RASGRP2	4.12	3.88	4.12
218525_s_at	hypoxia inducible	HIF1AN	7.81	7.69	7.75
210262_at	cysteine-rich secr	CRISP2	4.91	4.89	4.56

212815_at	activating signal c	ASCC3	9.30	9.36	9.44
215853_at	---	---	3.31	3.18	3.14
218249_at	zinc finger, DHHC-	ZDHHC6	10.38	10.45	10.49
214259_s_at	aldo-keto reducta	AKR7A2	10.06	10.09	10.08
205896_at	solute carrier fam	SLC22A4	6.47	6.48	6.69
216689_x_at	Rho GTPase activa	ARHGAP1	7.62	7.52	7.35
206065_s_at	dihydropyrimidina	DPYS	3.70	3.83	3.56
214033_at	ATP-binding casse	ABCC6	4.73	4.70	4.57
213645_at	enolase superfam	ENOSF1	8.08	8.14	8.24
201415_at	glutathione synth	GSS	9.04	8.86	9.00
201079_at	synaptogyrin 2	SYNGR2	9.52	9.41	9.45
202675_at	succinate dehydro	SDHB	10.17	10.16	10.12
204954_s_at	dual-specificity ty	DYRK1B	6.47	6.41	6.25
218855_at	transmembrane p	TPRA1	8.31	8.05	8.19
34210_at	CD52 molecule	CD52	3.76	3.60	3.48
217776_at	retinol dehydroge	RDH11	11.55	11.74	11.77
221028_s_at	glucose-fructose c	GFOD2	7.68	7.59	7.46
217765_at	nuclear receptor b	NRBP1	8.06	8.12	8.08
209752_at	regenerating islet-	REG1A	5.97	5.94	5.94
201774_s_at	non-SMC condens	NCAPD2	9.47	9.58	9.58
218306_s_at	hect (homologous	HERC1	8.26	8.23	8.36
208743_s_at	tyrosine 3-monoo	YWHAB	12.14	12.07	12.25
AFFX-M27830_5	---	---	14.17	14.15	14.09
219015_s_at	asparagine-linked	ALG13	7.86	7.99	8.06
204916_at	receptor (G protei	RAMP1	8.90	8.89	8.80
206499_s_at	regulator of chror	RCC1 /// SNHG3-R	9.87	9.77	9.96
203180_at	aldehyde dehydro	ALDH1A3	5.86	5.97	5.64
221690_s_at	NLR family, pyrin	NLRP2	3.75	3.66	3.76
121_at	paired box 8	PAX8	8.26	8.22	8.33
205042_at	glucosamine (UDF	GNE	8.57	8.67	8.57
216032_s_at	ERGIC and golgi 3	ERGIC3	9.19	9.08	9.13
200944_s_at	high-mobility grou	HMGN1	12.46	12.53	12.59
204822_at	TTK protein kinase	TTK	9.78	10.05	10.11
216061_x_at	platelet-derived g	PDGFB	7.04	6.80	6.92
213665_at	SRY (sex determin	SOX4	6.07	5.68	5.71
206345_s_at	paraoxonase 1	PON1	3.73	3.54	3.58
213071_at	dermatopontin	DPT	4.76	4.83	4.62
213842_x_at	NOP2/Sun domain	NSUN5P2	10.08	10.12	10.12
217874_at	succinate-CoA liga	SUCLG1	11.78	11.82	11.76
217736_s_at	eukaryotic transla	EIF2AK1	10.59	10.52	10.63
220002_at	kinesin family me	KIF26B	5.60	5.52	5.42
202400_s_at	serum response fa	SRF	5.66	5.66	5.47
214950_at	---	---	5.66	5.87	5.58
201551_s_at	lysosomal-associa	LAMP1	7.70	7.52	7.62
210654_at	tumor necrosis fa	TNFRSF10D	5.16	5.07	5.04
219673_at	minichromosome	MCM9	7.39	7.43	7.70
204648_at	natriuretic peptid	NPR1	7.59	7.40	7.53

201537_s_at	dual specificity ph	DUSP3	7.20	7.28	7.33
216136_at	---	---	3.80	3.60	3.70
212051_at	WAS/WASL intera	WIPF2	8.03	8.09	8.08
201920_at	solute carrier fam	SLC20A1	11.15	11.26	11.25
206595_at	cystatin E/M	CST6	5.71	5.52	5.37
206989_s_at	splicing factor, arg	SFRS2IP	9.49	9.69	9.69
220534_at	tripartite motif-co	TRIM48	4.18	3.90	3.97
205874_at	inositol 1,4,5-trisp	ITPKA	5.38	5.34	5.54
212207_at	mediator complex	MED13L	8.48	8.22	8.58
220623_s_at	testis specific, 10	TSGA10	4.21	4.07	3.78
216791_at	---	---	7.87	7.71	7.53
215059_at	---	---	3.37	3.06	3.41
212786_at	C-type lectin dom	CLEC16A	7.37	7.11	7.37
210768_x_at	transmembrane a	TMCO1	10.00	10.04	10.10
216688_at	---	---	4.66	4.76	4.62
215364_s_at	KIAA0467	KIAA0467	6.74	6.63	6.52
57588_at	solute carrier fam	SLC24A3	4.09	3.84	3.88
217652_at	KIAA0892	KIAA0892	6.09	6.09	5.93
221720_s_at	---	---	5.19	4.99	4.98
214303_x_at	mucin 5AC, oligon	MUC5AC	5.30	5.12	5.09
202108_at	peptidase D	PEPD	8.68	8.65	8.46
206202_at	mesenchyme hom	MEOX2	3.17	3.19	3.02
203400_s_at	transferrin	TF	4.17	4.07	3.89
202790_at	claudin 7	CLDN7	5.87	5.91	5.91
91580_at	leucine-rich repea	LRTM1	3.36	3.33	3.29
215702_s_at	cystic fibrosis tran	CFTR	3.40	3.35	3.18
214204_at	PARK2 co-regulate	PACRG	3.86	3.81	3.83
205069_s_at	Rho GTPase activa	ARHGAP26	3.92	3.69	3.73
214473_x_at	postmeiotic segre	PMS2L3	8.35	8.43	8.29
213880_at	leucine-rich repea	LGR5	3.46	3.55	3.40
201369_s_at	zinc finger proteir	ZFP36L2	9.16	9.07	8.97
217028_at	chemokine (C-X-C	CXCR4	3.63	3.48	3.30
202601_s_at	HIV-1 Tat specific	HTATSF1	6.79	7.00	6.88
204008_at	dynein, axonemal	DNAL4	8.00	7.75	7.88
207901_at	interleukin 12B (n	IL12B	3.23	3.58	3.43
204233_s_at	choline kinase alp	CHKA	7.74	7.65	7.78
201929_s_at	plakophilin 4	PKP4	8.85	8.85	8.99
207653_at	forkhead box D2	FOXD2	4.11	4.27	4.21
215798_at	aldehyde dehydro	ALDH1L1	6.85	6.87	6.76
200870_at	serine/threonine	STRAP	12.68	12.69	12.70
209186_at	ATPase, Ca++ tran	ATP2A2	10.15	10.07	10.17
222118_at	centromere prote	CENPN	7.70	7.72	7.80
206181_at	signaling lymphoc	SLAMF1	3.78	3.71	3.88
206545_at	CD28 molecule	CD28	3.35	3.12	3.39
213926_s_at	ArfGAP with FG re	AGFG1	6.46	5.95	6.35
216445_at	---	---	3.77	3.87	3.95
208857_s_at	protein-L-isoaspar	PCMT1	10.53	10.65	10.83

221637_s_at	chromosome 11 c	C11orf48	10.67	10.66	10.71
211438_at	thyrotropin-releas	TRHR	4.64	4.61	4.46
221847_at	similar to mCG115	LOC100129361	9.91	9.85	10.06
216034_at	zinc finger protein	ZNF280A	3.52	3.63	3.51
220404_at	G protein-couplec	GPR97	6.63	6.50	6.27
215775_at	---	---	5.25	5.36	5.00
201507_at	prefoldin subunit	PFDN1	9.48	9.38	9.54
215860_at	Synaptotagmin XI	SYT12	5.33	4.88	5.18
221365_at	motilin receptor	MLNR	4.54	4.29	4.34
217564_s_at	carbamoyl-phospl	CPS1	7.34	7.30	7.82
202513_s_at	protein phosphata	PPP2R5D	9.00	8.92	8.92
222188_at	chromosome 9 op	C9orf156	4.05	3.92	3.70
219281_at	methionine sulfox	MSRA	9.21	9.13	8.98
206228_at	paired box 2	PAX2	5.10	4.88	4.80
217390_x_at	---	---	6.80	6.80	6.74
208911_s_at	pyruvate dehydro	PDHB	9.44	9.52	9.44
209825_s_at	uridine-cytidine ki	UCK2	10.49	10.32	10.51
49077_at	protein phosphata	PPME1	8.80	8.90	8.66
213729_at	PRP40 pre-mRNA	PRPF40A	10.54	10.69	10.64
222156_x_at	cell cycle progress	CCPG1	6.37	6.58	6.89
217166_at	---	---	6.72	6.53	6.43
218695_at	exosome compon	EXOSC4	9.44	9.41	9.35
213913_s_at	TBC1 domain fam	TBC1D30	4.92	4.98	4.78
202121_s_at	chromatin modify	CHMP2A	11.04	10.99	11.07
206287_s_at	inter-alpha (globu	ITIH4	5.93	5.71	5.86
216150_at	---	---	4.51	4.58	4.42
214669_x_at	Immunoglobulin k	IGKC	6.62	6.62	6.47
209501_at	cerebellar degene	CDR2	9.30	9.06	9.37
216090_x_at	---	---	3.73	3.55	3.68
219479_at	KDEL (Lys-Asp-Glu	KDELC1	8.89	8.78	8.96
206020_at	suppressor of cyto	SOCS6	6.10	6.38	6.40
205030_at	fatty acid binding	FABP7	3.90	3.94	3.98
222204_s_at	RRN3 RNA polyme	RRN3	8.86	8.88	8.90
212930_at	ATPase, Ca++ tran	ATP2B1	8.09	8.19	8.53
206848_at	family with seque	FAM36A /// HOXA	7.10	7.26	7.28
220760_x_at	zinc finger protein	ZNF665	4.97	5.08	5.06
214909_s_at	dimethylarginine (	DDAH2	7.24	7.33	7.06
221904_at	family with seque	FAM131A	9.07	8.90	8.99
215504_x_at	---	---	8.48	8.54	8.46
214709_s_at	kinectin 1 (kinesin	KTN1	11.34	11.42	11.67
217610_at	---	---	5.78	5.71	5.92
220299_at	spermatogenesis a	SPATA6	5.29	4.99	4.79
201890_at	ribonucleotide red	RRM2	12.38	12.39	12.64
90610_at	leucine-rich repea	LRCH4 /// SAP25	9.20	9.16	9.09
210195_s_at	pregnancy specific	PSG1	4.62	4.76	4.78
205050_s_at	mitogen-activated	MAPK8IP2	6.41	6.25	6.40
210876_at	annexin A2 pseud	ANXA2P1	5.82	5.59	5.91

211045_s_at	potassium voltage	KCNH6	4.22	3.99	3.81
213970_at	RAB, member of R	RABL3	7.84	7.96	8.05
213051_at	zinc finger CCCH-t	ZC3HAV1	8.39	8.69	8.59
202980_s_at	seven in absentia	SIAH1	7.98	7.93	8.23
200777_s_at	basic leucine zipp	BZW1	12.05	12.20	12.31
220301_at	coiled-coil domair	CCDC102B	6.04	6.23	6.47
219249_s_at	FK506 binding prc	FKBP10	8.13	8.05	8.11
202026_at	succinate dehydrc	SDHD	10.22	10.31	10.37
201958_s_at	protein phosphata	PPP1R12B	4.21	4.05	4.32
203433_at	5,10-methenyltetı	MTHFS	8.67	8.50	8.51
213819_s_at	Flotillin 1	FLOT1	5.70	5.89	5.76
205781_at	chromosome 16 c	C16orf7	6.56	6.56	6.52
217162_at	testis specific prot	TSPY1	5.93	6.04	5.96
205576_at	serpin peptidase i	SERPIND1	4.55	4.33	4.30
207004_at	B-cell CLL/lympho	BCL2	7.30	7.08	7.01
205630_at	corticotropin rele	CRH	4.23	4.32	4.29
201740_at	NADH dehydroger	NDUFS3	10.96	10.95	10.90
217574_at	cadherin 8, type 2	CDH8	5.98	6.20	6.24
210606_x_at	killer cell lectin-lik	KLRD1	4.70	5.02	4.66
205658_s_at	small nuclear RNA	SNAPC4	7.13	7.32	7.13
214756_x_at	postmeiotic segre	PMS2L1	8.06	8.30	8.20
222245_s_at	fer-1-like 4 (C. ele	FER1L4	5.52	5.57	5.42
202412_s_at	ubiquitin specific	USP1	10.16	10.13	10.35
211182_x_at	runt-related trans	RUNX1	5.96	5.92	6.09
214802_at	exocyst complex c	EXOC7	6.65	6.70	6.53
212359_s_at	KIAA0913	KIAA0913	7.78	7.80	7.78
208954_s_at	La ribonucleoprot	LARP4B	9.13	8.99	9.12
203435_s_at	membrane metall	MME	9.07	9.03	9.13
222074_at	uroporphyrinoger	UROD	4.60	4.53	4.64
212082_s_at	myosin, light chai	MYL6	13.61	13.63	13.63
213124_at	zinc finger proteir	ZNF473	8.09	8.07	8.09
209337_at	PC4 and SFRS1 int	PSIP1	10.53	10.65	10.60
214994_at	apolipoprotein B r	APOBEC3F	5.84	5.53	5.85
203262_s_at	family with seque	FAM50A	10.53	10.51	10.53
215016_x_at	dystonin	DST	11.27	11.50	11.53
213594_x_at	splicing factor, arg	SFRS13A	9.86	9.98	10.04
203603_s_at	zinc finger E-box b	ZEB2	7.14	6.88	7.45
207357_s_at	UDP-N-acetyl-alph	GALNT10	7.79	7.80	7.85
210914_at	---	---	3.93	3.76	3.79
208635_x_at	nascent polypepti	NACA	14.35	14.32	14.35
221251_x_at	INO80 complex su	INO80B	6.89	6.78	6.85
221848_at	zinc finger, CCCH-	ZGPAT	7.38	7.43	7.37
203612_at	bystin-like	BYSL	9.60	9.42	9.53
213646_x_at	tubulin, alpha 1b	TUBA1B	14.48	14.55	14.49
201204_s_at	ribosome binding	RRBP1	9.12	9.19	9.03
205489_at	crystallin, mu	CRYM	7.14	6.99	7.10
203892_at	WAP four-disulfid	WFDC2	6.89	6.89	6.86

212266_s_at	splicing factor, arg	SFRS5	10.30	10.39	10.34
203373_at	suppressor of cyto	SOCS2	9.46	9.20	9.61
217946_s_at	SUMO1 activating	SAE1	10.95	10.98	10.93
215764_x_at	adaptor-related p	AP2A2	8.06	7.96	7.93
222304_x_at	olfactory receptor	OR7E47P	5.86	5.95	6.06
211336_x_at	leukocyte immuno	LILRB1	6.10	5.93	6.08
220349_s_at	endo-beta-N-acet	ENGASE	7.64	7.75	7.40
207954_at	GATA binding pro	GATA2	6.56	6.43	6.47
219539_at	gem (nuclear orga	GEMIN6	9.52	9.62	9.61
210131_x_at	succinate dehydro	SDHC	9.83	9.82	9.74
202689_at	RNA binding moti	RBM15B	8.69	8.67	8.82
210421_s_at	solute carrier fam	SLC24A1	6.58	6.55	6.41
212769_at	transducin-like en	TLE3	6.99	6.73	6.84
212612_at	REST corepressor	RCOR1	9.62	9.55	9.57
222169_x_at	SH2 domain conta	SH2D3A	7.02	7.00	7.02
215481_s_at	peroxisomal bioge	PEX5	5.66	5.57	5.41
212224_at	aldehyde dehydro	ALDH1A1	7.79	7.84	7.93
210458_s_at	TRAF family meml	TANK	5.16	5.02	5.16
217720_at	coiled-coil-helix-co	CHCHD2	13.34	13.31	13.26
215797_at	T cell receptor alp	TRAV8-3	4.80	4.68	4.53
215447_at	---	---	5.42	5.82	5.69
219967_at	mitochondrial rRN	MRM1	7.84	7.77	7.72
202698_x_at	cytochrome c oxid	COX4I1	13.61	13.57	13.56
213202_at	SET domain conta	SETD1A	7.12	7.09	7.28
214773_x_at	TIP41, TOR signali	TIPRL	9.01	9.11	9.26
222192_s_at	chromosome 2 op	C2orf43	8.75	8.74	8.73
217890_s_at	parvin, alpha	PARVA	7.38	7.44	7.52
203674_at	helicase with zinc	HELZ	7.89	7.87	7.87
221697_at	microtubule-asso	MAP1LC3C	5.40	5.36	5.21
200603_at	protein kinase, cA	PRKAR1A	11.95	11.99	12.29
216750_at	amyloid beta (A4)	APBB2	5.59	5.59	5.45
201231_s_at	enolase 1, (alpha)	ENO1	13.21	13.24	13.22
215403_at	---	---	5.29	5.32	5.42
220255_at	Fanconi anemia, c	FANCE	8.91	8.77	9.00
208036_at	opsin 1 (cone pigr	OPN1SW	4.60	4.74	4.40
209534_x_at	A kinase (PRKA) a	AKAP13	8.84	8.78	8.77
213429_at	---	---	4.40	4.70	4.25
216503_s_at	myeloid/lymphoi	MLLT10	8.03	8.19	7.97
203755_at	budding uninhibit	BUB1B	11.17	11.26	11.19
207543_s_at	prolyl 4-hydroxyla	P4HA1	9.81	9.87	9.98
213928_s_at	ArfGAP with FG re	AGFG1	5.03	4.79	5.12
220784_s_at	urotensin 2	UTS2	3.26	3.13	3.21
222251_s_at	glucocorticoid mo	GMEB2	8.89	8.67	8.86
209152_s_at	transcription facto	TCF3	7.77	7.90	7.64
219316_s_at	feline leukemia vi	FLVCR2	8.37	8.66	8.45
204154_at	cysteine dioxygen	CDO1	3.76	3.88	3.63
217351_at	---	---	4.34	4.34	4.46

201070_x_at	splicing factor 3b,	SF3B1	8.93	8.96	8.73
213678_at	transmembrane p	TMEM151B	5.03	4.90	4.96
209731_at	nth endonuclease	NTHL1	8.74	8.76	8.73
218975_at	collagen, type V, a	COL5A3	6.04	5.94	5.99
218250_s_at	CCR4-NOT transcr	CNOT7	11.58	11.66	11.67
202444_s_at	ER lipid raft associ	ERLIN1	6.49	6.57	6.78
206337_at	chemokine (C-C m	CCR7	3.90	3.83	3.87
215231_at	Protein kinase, AM	PRKAG2	5.27	5.28	5.26
220823_at	HCG1732469	LOC729164	7.26	7.26	7.21
215946_x_at	immunoglobulin la	IGLL3	5.51	5.48	5.46
211179_at	runt-related trans	RUNX1	3.36	3.51	3.28
211412_at	peptidyl arginine (	PADI4	3.35	3.35	3.37
203823_at	regulator of G-pro	RGS3	9.44	9.44	9.48
219578_s_at	cytoplasmic polya	CPEB1	4.75	5.28	5.23
208066_s_at	general transcript	GTF2B	8.94	9.18	9.09
216609_at	Thioredoxin	TXN	7.69	7.86	7.90
222148_s_at	ras homolog gene	RHOT1	10.26	10.28	10.32
220221_at	vacuolar protein s	VPS13D	6.09	6.31	5.67
213240_s_at	keratin 4	KRT4	4.54	4.62	4.31
207037_at	tumor necrosis fa	TNFRSF11A	7.11	7.10	7.38
219086_at	zinc finger protein	ZNF839	6.75	6.78	6.57
220077_at	coiled-coil domain	CCDC134	6.60	6.72	6.76
210646_x_at	ribosomal protein	RPL13A	15.09	15.08	15.10
206546_at	synaptonemal cor	SYCP2	3.47	3.17	3.30
206909_at	---	---	3.34	3.47	3.24
214410_at	Transient recepto	TRPM1	3.55	3.22	3.39
217055_x_at	---	---	7.60	7.45	7.24
212874_at	hypothetical LOC1	LOC100129500	5.37	4.99	5.11
214104_at	G protein-couplec	GPR161	7.65	7.76	7.57
203952_at	activating transcri	ATF6	9.03	9.06	9.05
221415_s_at	gap junction prote	GJA9	6.51	6.54	6.40
212774_at	zinc finger protein	ZNF238	8.10	8.12	8.25
216561_x_at	---	---	4.05	3.82	3.90
207620_s_at	calcium/calmodul	CASK	6.52	6.52	6.49
203881_s_at	dystrophin	DMD	5.22	5.32	5.88
217442_at	Hypothetical prot	LOC100131825	4.25	4.27	4.28
218251_at	MID1 interacting	MID1IP1	5.64	5.72	5.51
203399_x_at	pregnancy specifi	PSG3	4.05	4.40	4.14
209027_s_at	abl-interactor 1	ABI1	9.15	9.19	9.43
1431_at	cytochrome P450,	CYP2E1	3.99	4.18	4.04
210528_at	major histocompa	MR1	4.98	4.81	4.89
206013_s_at	actin-like 6B	ACTL6B	4.43	4.37	4.29
51200_at	chromosome 19 c	C19orf60	9.44	9.38	9.53
221194_s_at	ring finger protein	RNFT1	5.50	5.83	5.68
206492_at	fragile histidine tr	FHIT	6.55	6.51	6.47
216159_s_at	---	---	5.53	5.68	5.52
212868_x_at	chromosome 12 c	C12orf47	7.50	7.30	7.28

201137_s_at	major histocompa	HLA-DPB1	4.22	4.32	4.48
215363_x_at	folate hydrolase (|	FOLH1	3.68	3.71	3.69
214271_x_at	ribosomal protein	RPL12	14.34	14.30	14.35
205583_s_at	asparagine-linked	ALG13	8.97	8.98	9.22
201590_x_at	annexin A2	ANXA2	14.35	14.39	14.39
222349_x_at	ring finger proteir	RNF126P1	7.41	7.25	7.23
221543_s_at	ER lipid raft associ	ERLIN2	10.16	10.24	10.18
221211_s_at	chromosome 21 c	C21orf7	4.58	5.05	5.16
213517_at	poly(rC) binding p	PCBP2	8.40	8.53	8.38
48580_at	CXXC finger 1 (PHI	CXXC1	9.37	9.34	9.37
218213_s_at	chromosome 11 c	C11orf10	12.06	12.04	12.10
220817_at	transient receptoı	TRPC4	7.97	8.18	8.15
217023_x_at	tryptase alpha/be	TPSAB1 /// TPSB2	6.50	6.45	6.45
217636_at	polymerase (DNA	POLG	5.84	5.87	5.77
207545_s_at	numb homolog (D	NUMB	7.73	7.54	7.90
210551_s_at	acetylserotonin O	ASMT	5.23	5.01	4.93
201481_s_at	phosphorylase, gl	PYGB	9.13	9.11	8.88
218887_at	mitochondrial ribo	MRPL2	8.04	8.01	7.96
214152_at	cell cycle progress	CCPG1	7.59	7.52	7.68
218158_s_at	adaptor protein, p	APPL1	5.50	5.66	5.66
216853_x_at	immunoglobulin la	IGLV3-19	5.75	5.49	5.73
214232_at	---	---	7.79	7.95	7.95
218390_s_at	chromosome 10 c	C10orf84	6.02	6.12	5.97
204451_at	frizzled homolog 1	FZD1	7.26	7.27	7.26
215952_s_at	ornithine decarbo	OAZ1	13.39	13.36	13.46
215977_x_at	glycerol kinase	GK	6.00	6.06	5.77
205729_at	oncostatin M rece	OSMR	6.47	6.40	6.53
212368_at	zinc finger proteir	ZNF292	7.80	7.90	7.98
209510_at	ring finger proteir	RNF139	10.40	10.33	10.33
207247_s_at	zinc finger proteir	ZFX /// ZFY	5.70	5.67	5.83
213061_s_at	N-terminal aspara	NTAN1	8.95	9.06	9.17
209989_at	zinc finger proteir	ZNF268	7.14	7.38	7.33
213157_s_at	WSC domain cont	WSCD1	6.53	6.27	6.35
204403_x_at	family with seque	FAM115A	8.42	8.78	8.67
220827_at	---	---	6.14	6.11	5.95
209307_at	SWAP switching B	SWAP70	9.16	9.43	9.45
204834_at	fibrinogen-like 2	FGL2	4.05	3.96	3.91
205174_s_at	glutaminyl-peptid	QPCT	3.56	3.43	3.38
208471_at	haptoglobin-relate	HPR	5.19	5.13	4.94
220947_s_at	TBC1 domain fam	TBC1D10B	7.80	7.86	7.76
208555_x_at	cystatin SA	CST2	6.26	6.11	6.35
214674_at	ubiquitin specific		USP19	7.15	7.08	7.00
219514_at	angiopoietin-like 2	ANGPTL2	5.19	5.10	5.03
209636_at	nuclear factor of k	NFKB2	6.09	5.96	6.27
220227_at	cadherin 4, type 1	CDH4	5.48	4.95	4.68
203912_s_at	deoxyribonucleas	DNASE1L1	8.86	8.72	8.81
216985_s_at	syntaxin 3	STX3	5.91	5.63	5.90

206856_at	leukocyte immuno	LILRB5	4.65	4.30	4.36
205416_s_at	ataxin 3	ATXN3	6.39	6.54	6.58
207999_s_at	adenosine deamir	ADARB1	6.86	6.79	6.58
201416_at	SRY (sex determin	SOX4	8.20	8.23	8.26
207569_at	c-ros oncogene 1	ROS1	6.37	6.68	6.38
215020_at	neurexin 3	NRXN3	4.30	4.10	4.08
218046_s_at	mitochondrial ribo	MRPS16	11.16	11.11	11.11
221881_s_at	chloride intracellu	CLIC4	8.26	8.06	8.19
210917_at	v-yes-1 Yamaguch	YES1	3.83	3.79	3.82
202477_s_at	tubulin, gamma co	TUBGCP2	8.58	8.57	8.49
222029_x_at	prefoldin subunit	PFDN6	8.75	8.65	8.68
202785_at	NADH dehydroger	NDUFA7	10.86	10.83	10.91
210627_s_at	mannosyl-oligosa	MOGS	8.56	8.59	8.50
220281_at	solute carrier fam	SLC12A1	3.33	3.30	3.11
221098_x_at	UTP14, U3 small r	UTP14A	4.77	4.84	4.90
221472_at	serine incorporato	SERINC3	9.91	9.79	9.76
203532_x_at	cullin 5	CUL5	6.75	6.78	6.91
204391_x_at	tripartite motif-co	TRIM24	8.84	8.91	8.83
206141_at	molybdenum cofa	MOCS3	6.32	6.35	6.42
38043_at	family with seque	FAM3A	7.53	7.62	7.57
216204_at	Catechol-O-methy	COMT	7.03	7.10	7.04
222355_at	---	---	4.05	4.01	4.05
201069_at	matrix metallopep	MMP2	8.37	8.50	8.46
221871_s_at	TRK-fused gene	TFG	6.07	6.26	6.00
221494_x_at	eukaryotic transla	EIF3K	12.06	11.96	12.05
217920_at	mannosidase, alph	MAN1A2	7.41	7.56	7.53
32088_at	NEDD4 binding pr	N4BP1	5.35	5.53	5.74
206332_s_at	interferon, gamm	IFI16	10.51	10.54	10.85
204133_at	ribosomal RNA pr	RRP9	7.28	7.00	7.23
212799_at	syntaxin 6	STX6	9.51	9.47	9.59
207715_at	crystallin, gamma	CRYGB	4.61	4.90	4.81
214387_x_at	surfactant protein	SFTPC	6.65	6.46	6.28
211212_s_at	origin recognition	ORC5L	8.02	8.16	8.57
211633_x_at	---	---	5.57	5.33	5.38
222379_at	potassium voltage	KCNE4	8.04	7.99	8.32
216529_at	---	---	3.42	3.47	3.26
211227_s_at	protocadherin 11	PCDH11Y	3.14	3.56	3.54
203298_s_at	jumonji, AT rich in	JARID2	8.90	8.87	8.86
214198_s_at	DiGeorge syndron	DGCR2	8.44	8.48	8.47
202635_s_at	polymerase (RNA)	POLR2K	10.62	10.61	10.64
216251_s_at	tubulin tyrosine li	TTLL12	10.08	9.94	10.06
201683_x_at	TOX high mobility	TOX4	8.54	8.61	8.58
57082_at	low density lipopr	LDLRAP1	7.34	7.47	7.34
210681_s_at	ubiquitin specific	USP15	9.02	9.04	9.28
213852_at	RNA binding moti	RBM8A	9.26	9.49	9.36
213986_s_at	chromosome 19 c	C19orf6	6.20	6.02	6.02
217134_at	methylthioadenos	MTAP	4.75	4.68	4.84

207137_at	nuclear factor of k	NFKBIL2	6.11	5.99	5.65
201867_s_at	transducin (beta)-	TBL1X	9.79	9.58	9.72
221916_at	neurofilament, lig	NEFL	3.60	3.59	3.61
209248_at	growth hormone i	GHITM	12.51	12.51	12.65
37145_at	granulysin	GNLY	4.71	4.77	4.61
213469_at	post-GPI attachm	PGAP1	6.76	6.94	7.15
212438_at	small nuclear ribo	SNRNP27	9.38	9.35	9.48
205099_s_at	chemokine (C-C m	CCR1	4.70	4.40	4.37
218579_s_at	DEAH (Asp-Glu-Al	DHX35	7.98	7.90	7.93
209578_s_at	protein O-fucosylt	POFUT2	7.58	7.54	7.53
213137_s_at	protein tyrosine p	PTPN2	9.22	9.21	9.27
210505_at	alcohol dehydrog	ADH7	4.38	4.52	4.27
208506_at	histone cluster 1,	HIST1H3F	4.40	4.40	4.75
208808_s_at	high-mobility grou	HMGB2	13.11	13.14	13.38
214755_at	UDP-N-acteylgluc	UAP1L1	4.94	5.07	5.14
221319_at	protocadherin bet	PCDHB8	4.35	4.21	4.15
203241_at	UV radiation resis	UVRAG	7.72	7.65	7.79
217662_x_at	---	---	3.90	4.14	4.13
214901_at	zinc finger protein	ZNF8	7.98	8.10	8.08
209044_x_at	splicing factor 3b,	SF3B4	7.96	7.96	8.03
211424_x_at	methyltransferase	METTL7A	3.96	4.01	3.93
204167_at	biotinidase	BTD	7.07	7.09	7.26
206748_s_at	sperm associated	SPAG9	6.44	6.45	6.34
220026_at	chloride channel a	CLCA4	3.04	3.00	3.11
209092_s_at	glyoxalase domain	GLOD4	8.59	8.54	8.60
212981_s_at	family with seque	FAM115A	7.00	7.26	7.31
208695_s_at	ribosomal protein	RPL39	14.86	14.82	14.83
218272_at	tetratricopeptide	TTC38	7.75	7.79	7.72
209622_at	serine/threonine l	STK16	8.16	8.06	8.34
209742_s_at	myosin, light chai	MYL2	5.69	5.59	5.63
219726_at	neuroligin 3	NLGN3	5.76	5.66	5.38
209950_s_at	villin-like	VILL	5.68	5.78	5.61
207805_s_at	proteasome (pros	PSMD9	10.50	10.49	10.55
212754_s_at	MON2 homolog (S	MON2	8.75	8.78	8.85
217335_at	hypothetical prot	FLJ11292	4.88	4.80	4.61
208397_x_at	potassium inward	KCNJ5	7.27	7.45	7.27
200903_s_at	adenosylhomocys	AHCY	11.57	11.49	11.59
203651_at	zinc finger, FYVE c	ZFYVE16	8.00	8.26	8.37
222259_s_at	SPO11 meiotic pro	SPO11	3.28	3.33	3.60
214159_at	Phospholipase C, e	PLCE1	3.43	3.80	3.52
203699_s_at	deiodinase, iodotl	DIO2	6.93	7.27	7.08
214880_x_at	caldesmon 1	CALD1	8.19	8.12	7.78
206679_at	amyloid beta (A4)	APBA1	4.92	4.96	5.06
213103_at	StAR-related lipid	STARD13	8.98	9.26	9.17
202481_at	dehydrogenase/re	DHRS3	7.68	7.58	7.79
212456_at	KIAA0664	KIAA0664	9.30	9.25	9.24
44654_at	glucose 6 phospha	G6PC3	9.63	9.47	9.61

219903_s_at	cytochrome P450,	CYP2C8	3.71	3.42	3.56
219642_s_at	peroxisomal bioge	PEX5L	3.03	3.35	3.37
202487_s_at	H2A histone famil	H2AFV	11.41	11.45	11.39
215132_at	---	---	3.95	3.92	3.61
217375_at	---	---	3.89	3.74	3.88
201015_s_at	junction plakoglol	JUP	8.18	7.74	7.92
214392_at	immunity-related	IRGC	5.94	5.51	5.71
210444_at	neuropeptide Y re	NPY6R	5.45	5.57	5.74
34471_at	myosin, heavy cha	MYH8	3.70	3.89	3.66
217054_at	---	---	6.79	6.42	6.33
215906_at	---	---	5.40	5.11	5.19
222191_s_at	xylosylprotein bet	B4GALT7	7.19	7.17	7.23
218083_at	prostaglandin E sy	PTGES2	8.26	8.15	8.08
202826_at	solute carrier fam	SLC25A4	5.60	5.35	5.61
208302_at	histocompatibility	HMHB1	4.38	4.32	3.96
200827_at	procollagen-lysine	PLOD1	9.35	9.25	9.39
221763_at	jumonji domain co	JMJD1C	9.44	9.52	9.77
211537_x_at	mitogen-activatec	MAP3K7	8.66	8.83	8.71
219559_at	solute carrier fam	SLC17A9	7.40	7.51	7.57
218977_s_at	tRNA selenocystei	TRNAU1AP	8.07	8.05	8.11
215849_x_at	tetratricopeptide	TTC18	6.71	6.84	6.81
209604_s_at	GATA binding pro	GATA3	9.61	9.41	9.69
208783_s_at	CD46 molecule, co	CD46	10.96	11.06	11.13
211805_s_at	solute carrier fam	SLC8A1	5.52	5.67	5.66
40472_at	lysophosphatidylc	LPCAT4	7.62	7.62	7.73
207710_at	late cornified env	LCE2B	4.55	4.33	4.21
208410_x_at	amelogenin, X-linl	AMELX	3.63	3.67	3.48
215907_at	---	---	6.73	6.66	6.95
207021_at	zona pellucida bin	ZPBP	4.07	4.19	3.95
209656_s_at	transmembrane p	TMEM47	9.89	9.97	10.16
218026_at	coiled-coil domair	CCDC56	11.00	11.05	10.91
221279_at	ganglioside-induc	GDAP1	5.35	5.36	5.25
203507_at	CD68 molecule	CD68	6.70	6.54	6.57
203717_at	dipeptidyl-peptida	DPP4	4.02	4.28	3.87
206965_at	Kruppel-like facto	KLF12	3.59	3.58	3.50
209409_at	growth factor rec	GRB10	10.27	10.06	10.25
211197_s_at	inducible T-cell co	ICOSLG	6.38	6.04	5.99
213048_s_at	---	---	10.86	10.82	10.95
221842_s_at	zinc finger proteir	ZNF131	10.16	10.13	10.22
218085_at	chromatin modify	CHMP5	10.03	10.10	10.18
222133_s_at	PHD finger protei	PHF20L1	7.79	7.84	7.96
216221_s_at	pumilio homolog	PUM2	10.26	10.31	10.35
215471_s_at	microtubule-asso	MAP7	5.02	5.44	5.25
213554_s_at	CDV3 homolog (m	CDV3	9.44	9.26	9.31
208995_s_at	peptidylprolyl isor	PPIG	8.39	8.63	8.77
215566_x_at	lysophospholipase	LYPLA2	8.66	8.81	8.75
204723_at	sodium channel, v	SCN3B	3.89	4.05	3.74

205614_x_at	macrophage stimu	MST1	7.63	7.64	7.72
200948_at	myeloid leukemia	MLF2	7.95	8.09	8.10
215322_at	---	---	7.14	7.42	7.42
201215_at	plastin 3	PLS3	11.94	12.02	12.00
201476_s_at	ribonucleotide re	RRM1	8.59	8.69	8.64
209198_s_at	synaptotagmin XI	SYT11	6.11	6.11	6.19
219653_at	LSM14B, SCD6 ho	LSM14B	7.40	7.36	7.42
211842_s_at	solute carrier fam	SLC24A1	4.77	4.73	4.95
221974_at	imprinted in Prad	IPW	8.27	8.32	8.72
218194_at	REX2, RNA exonu	REXO2	11.33	11.32	11.44
221111_at	interleukin 26	IL26	3.20	3.07	3.14
210600_s_at	G protein-couplec	GRK4	3.66	3.68	3.61
221492_s_at	ATG3 autophagy r	ATG3	11.53	11.56	11.66
212396_s_at	KIAA0090	KIAA0090	9.26	9.19	9.16
211150_s_at	dihydrolipoamide	DLAT	7.85	7.93	8.02
212265_at	quaking homolog,	QKI	11.93	11.87	12.01
212541_at	FAD1 flavin adeni	FLAD1	8.18	8.01	8.12
207519_at	solute carrier fam	SLC6A4	5.30	5.12	5.26
221843_s_at	KIAA1609	KIAA1609	7.72	8.03	7.85
202879_s_at	CD93 molecule	CD93	5.50	5.39	5.46
217872_at	PIH1 domain cont	PIH1D1	10.15	10.13	10.09
220498_at	actin-like 7B	ACTL7B	5.67	5.56	5.70
216667_at	similar to Nonsec	LOC643332 /// RN	5.12	5.18	5.13
210035_s_at	ribosomal protein	RPL5 /// SNORA66	3.81	3.72	3.71
205411_at	serine/threonine	STK4	5.01	4.72	4.87
202667_s_at	solute carrier fam	SLC39A7	8.67	8.61	8.57
222298_at	---	---	4.23	4.20	4.09
203239_s_at	CCR4-NOT transcr	CNOT3	5.88	5.97	5.81
206731_at	connector enhanc	CNKSR2	3.68	3.81	3.49
210012_s_at	Ewing sarcoma br	EWSR1	5.38	5.18	5.37
221117_at	---	---	3.34	3.51	3.24
218868_at	ARP3 actin-relate	ACTR3B	7.87	7.81	7.90
210076_x_at	SERPINE1 mRNA b	SERBP1	10.18	10.23	10.34
200089_s_at	ribosomal protein	RPL4	14.27	14.24	14.35
204800_s_at	dehydrogenase/re	DHRS12	6.04	6.06	5.76
218162_at	olfactomedin-like	OLFML3	4.93	4.90	4.88
208106_x_at	pregnancy specifi	PSG6	6.68	6.55	6.56
210000_s_at	---	---	4.09	4.03	3.82
212281_s_at	transmembrane p	TMEM97	10.93	11.03	11.02
212522_at	phosphodiesteras	PDE8A	9.21	9.26	9.39
218902_at	Notch homolog 1,	NOTCH1	9.04	8.70	8.73
220078_at	ubiquitin specific	USP48	6.88	6.60	6.71
211078_s_at	serine/threonine	STK3	3.75	3.71	3.57
206851_at	ribonuclease, RNa	RNASE3	6.53	6.31	6.20
202822_at	LIM domain conta	LPP	11.37	11.35	11.32
210465_s_at	small nuclear RNA	SNAPC3	7.73	7.85	7.89
206718_at	LIM domain only 1	LMO1	5.21	5.06	5.24

211676_s_at	interferon gamma	IFNGR1	10.19	10.16	10.33
201441_at	cytochrome c oxic	COX6B1	11.94	11.93	11.88
204849_at	transcription facto	TCFL5	8.85	8.80	8.86
209758_s_at	microfibrillar asso	MFAP5	4.79	5.08	4.66
208778_s_at	t-complex 1	TCP1	12.46	12.55	12.57
216182_at	Synaptojanin 2	SYNJ2	6.08	5.99	5.99
219518_s_at	elongation factor	ELL3 /// SERINC4	6.12	6.20	6.12
213384_x_at	phospholipase C, I	PLCB3	8.13	8.17	7.94
209016_s_at	keratin 7	KRT7	12.08	12.00	12.10
202897_at	signal-regulatory p	SIRPA	8.70	8.84	8.96
AFFX-r2-Bs-thr-M	---	---	8.25	8.20	8.17
203481_at	family with seque	FAM178A	7.26	7.20	7.42
209817_at	protein phosphata	PPP3CB	9.51	9.37	9.49
220190_s_at	general transcript	GTF2A1L /// STON	3.27	3.19	3.35
218132_s_at	tRNA splicing endo	TSEN34	9.82	9.74	9.73
219804_at	synaptopodin 2-lil	SYNPO2L	3.18	3.00	3.13
203132_at	retinoblastoma 1	RB1	9.54	9.61	9.85
203936_s_at	matrix metallopep	MMP9	6.98	7.01	6.95
201834_at	protein kinase, AM	PRKAB1	7.55	7.54	7.74
206754_s_at	cytochrome P450,	CYP2B6 /// CYP2B7	4.80	4.85	4.71
212887_at	Sec23 homolog A	SEC23A	10.60	10.75	10.92
202916_s_at	family with seque	FAM20B	9.25	9.21	9.29
220483_s_at	ring finger protein	RNF19A	5.99	6.01	5.90
203635_at	Down syndrome c	DSCR3	8.91	8.94	9.01
214656_x_at	myosin IC	MYO1C	7.81	7.99	8.08
213747_at	---	---	5.21	5.01	5.05
209223_at	NADH dehydrogen	NDUFA2	5.52	5.28	5.26
218243_at	RUN and FYVE dom	RUFY1	9.17	9.28	9.13
216253_s_at	parvin, beta	PARVB	6.09	5.97	5.92
222313_at	---	---	6.28	6.60	6.50
207124_s_at	guanine nucleotid	GNB5	8.35	8.20	8.33
206960_at	lysophosphatidic a	LPAR4	3.62	3.63	3.61
221731_x_at	versican	VCAN	7.05	7.35	7.38
209026_x_at	tubulin, beta	TUBB	12.77	12.76	12.74
217694_at	---	---	2.93	3.18	3.04
207464_at	adenosylhomocys	AHCYL1	4.20	4.00	4.05
204028_s_at	RAB GTPase activa	RABGAP1	9.48	9.44	9.41
220071_x_at	HAUS augmin-like	HAUS2	9.22	9.38	9.41
202234_s_at	solute carrier fam	SLC16A1	9.47	9.58	9.67
209081_s_at	collagen, type XVI	COL18A1	7.01	6.79	6.80
209097_s_at	jagged 1 (Alagille s	JAG1	5.44	5.12	5.32
211382_s_at	transforming, acic	TACC2	7.36	7.20	7.44
215993_at	---	---	3.53	3.15	3.24
207366_at	potassium voltage	KCNS1	6.21	6.07	6.17
215923_s_at	pleckstrin and Sec	PSD4	3.85	4.20	4.05
214819_at	IQ motif and Sec7	IQSEC2	7.60	7.63	7.45
204394_at	solute carrier fam	SLC43A1	7.91	7.81	7.91

202454_s_at	v-erb-b2 erythrob	ERBB3	4.14	4.03	4.16
211470_s_at	sulfotransferase fa	SULT1C2	3.33	3.35	3.13
201562_s_at	sorbitol dehydrog	SORD	8.49	8.46	8.61
205068_s_at	Rho GTPase activa	ARHGAP26	7.51	7.43	7.48
208357_x_at	chorionic somatom	CSH1	4.69	4.60	4.63
203619_s_at	Fas apoptotic inhi	FAIM2	5.36	5.21	5.22
201883_s_at	UDP-Gal:betaGlcN	B4GALT1	10.72	10.58	10.70
215041_s_at	dedicator of cytok	DOCK9	4.12	4.02	3.94
215066_at	protein tyrosine p	PTPRF	6.22	6.23	5.84
201021_s_at	destrin (actin dep	DSTN	11.46	11.55	11.78
212074_at	Sad1 and UNC84 c	SUN1	10.86	10.88	10.94
201827_at	SWI/SNF related,	SMARCD2	9.10	9.12	9.07
206177_s_at	arginase, liver	ARG1	3.79	3.47	3.65
207692_s_at	aggrecan	ACAN	6.69	6.92	6.60
206400_at	lectin, galactoside	LGALS7 /// LGALS7	5.15	5.26	4.93
201235_s_at	BTG family, memb	BTG2	5.84	5.65	5.48
214794_at	proliferation-asso	PA2G4	9.95	10.00	10.10
217733_s_at	thymosin beta 10	TMSB10	14.89	14.91	14.90
203961_at	nebulette	NEBL	9.93	9.98	10.12
213593_s_at	transformer 2 alph	TRA2A	8.18	8.39	8.21
211763_s_at	ubiquitin-conjugat	UBE2B	9.85	9.86	9.86
218393_s_at	smu-1 suppressor	SMU1	8.93	8.88	8.86
213918_s_at	Nipped-B homolog	NIPBL	9.44	9.43	9.53
200899_s_at	meningioma expre	MGEA5	10.53	10.64	10.74
201173_x_at	nuclear distributio	NUDC	11.44	11.34	11.42
220024_s_at	periaxin	PRX	7.73	7.42	7.57
201610_at	isoprenylcysteine	ICMT	4.99	4.77	5.18
217520_x_at	similar to hCG200	LOC100288671	7.19	7.39	7.13
212318_at	transportin 3	TNPO3	9.82	9.82	9.81
212329_at	SREBF chaperone	SCAP	9.29	9.12	9.20
211111_at	similar to HGC6.3	HGC6.3	6.40	6.00	6.14
200017_at	ribosomal protein	RPS27A	14.69	14.71	14.82
219626_at	MAP7 domain con	MAP7D3	8.16	8.19	8.33
210680_s_at	mannan-binding le	MASP1	6.09	6.15	6.07
204173_at	myosin, light chain	MYL6B	9.74	9.76	9.72
209653_at	karyopherin alpha	KPNA4	7.63	7.80	7.87
220626_at	serpin peptidase i	SERPINA10	6.32	6.19	5.97
213693_s_at	mucin 1, cell surfa	MUC1	7.88	7.84	7.63
200764_s_at	catenin (cadherin-	CTNNA1	9.46	9.47	9.45
215055_at	UDP-GlcNAc:betaG	B3GNTL1	6.79	6.73	6.73
207006_s_at	coiled-coil domain	CCDC106	6.32	5.98	5.77
211401_s_at	fibroblast growth	FGFR2	5.15	5.32	5.19
212964_at	hypermethylated	HIC2	6.43	6.49	6.30
205157_s_at	keratin 17	KRT17	4.45	4.62	4.64
215010_s_at	BR serine/threoni	BRSK2	5.11	5.08	4.91
203692_s_at	E2F transcription f	E2F3	7.99	7.95	8.00
215618_at	Ras suppressor pr	RSU1	6.35	6.35	6.51

220346_at	methylenetetrahy	MTHFD2L	5.94	5.99	6.22
218734_at	N(alpha)-acetyltra	NAA40	7.08	7.38	7.39
217461_x_at	basic transcriptior	BTF3L2	6.63	6.55	6.64
202205_at	vasodilator-stimul	VASP	9.34	9.27	9.26
221668_s_at	dynein, axonemal	DNAI2	5.11	5.10	4.94
218382_s_at	U2 small nuclear I	U2AF2	7.82	7.74	7.81
203523_at	lymphocyte-speci	LSP1	5.86	5.77	5.66
202742_s_at	protein kinase, cA	PRKACB	5.59	5.66	5.65
1494_f_at	cytochrome P450,	CYP2A6	6.94	6.95	6.72
215144_at	---	---	5.26	5.18	5.26
216054_x_at	myosin, light chai	MYL4	6.16	6.31	6.22
206285_at	nephronophthisis	NPHP1	4.61	4.52	4.42
214365_at	tropomyosin 3	TPM3	5.25	4.94	5.17
203491_s_at	centrosomal prote	CEP57	9.93	9.94	10.30
203863_at	actinin, alpha 2	ACTN2	3.80	3.56	3.48
213144_at	golgi SNAP recept	GOSR2	7.46	7.25	7.37
203349_s_at	ets variant 5	ETV5	9.76	9.52	9.72
204012_s_at	leucine carboxyl n	LCMT2	8.56	8.74	8.64
213047_x_at	SET nuclear oncog	SET	13.10	13.15	13.17
213438_at	neurofascin homo	NFASC	3.89	3.68	3.58
203815_at	glutathione S-tran	GSTT1	7.03	7.00	6.93
201533_at	catenin (cadherin-	CTNNB1	12.33	12.34	12.37
201335_s_at	Rho guanine nucle	ARHGEF12	6.83	6.81	6.82
203370_s_at	PDZ and LIM dom	PDLIM7	5.74	5.64	5.37
213743_at	cyclin T2	CCNT2	8.76	8.76	8.94
211377_x_at	v-myc myelocytor	MYCN	6.28	5.92	6.06
216974_at	---	---	3.20	2.92	3.08
218517_at	PHD finger protei	PHF17	10.25	10.23	10.45
216471_x_at	synovial sarcoma,	SSX2 /// SSX2B	4.57	4.37	4.20
212955_s_at	polymerase (RNA)	POLR2I	10.82	10.77	10.81
218600_at	LIM domain conta	LIMD2	6.30	6.12	6.04
208771_s_at	leukotriene A4 hy	LTA4H	11.77	11.72	11.82
204640_s_at	speckle-type POZ	SPOP	10.57	10.49	10.63
222135_at	zinc finger proteir	ZNF814	3.79	3.73	3.65
203751_x_at	jun D proto-oncog	JUND	6.12	5.87	5.96
220907_at	G protein-couplec	GPR110	6.12	5.67	5.66
202567_at	small nuclear ribo	SNRPD3	11.82	11.77	11.82
206431_x_at	TBC1 domain fam	TBC1D9B	7.72	7.84	7.65
215979_s_at	---	---	6.80	6.68	6.44
212369_at	zinc finger proteir	ZNF384	8.77	8.71	8.66
201208_s_at	tumor necrosis fa	TNFAIP1	7.65	7.51	7.61
205055_at	integrin, alpha E (a	ITGAE	10.24	10.25	10.30
218238_at	GTP binding prote	GTPBP4	10.61	10.60	10.77
221239_s_at	Fc receptor-like 2	FCRL2	4.74	4.35	4.33
221134_at	angiopoietin 4	ANGPT4	6.95	7.06	6.87
210335_at	Ras association (R	RASSF9	4.07	3.96	4.31
205202_at	protein-L-isoaspa	PCMT1	11.13	11.12	11.23

215461_at	zinc and ring finge	ZNRF4	6.81	6.87	6.61
222055_at	fumarylacetoacet	FAHD2A /// FAHD2	5.26	5.26	4.98
210861_s_at	WNT1 inducible si	WISP3	3.63	3.62	3.51
221499_s_at	syntaxin 16	STX16	9.61	9.58	9.65
203563_at	actin filament ass	AFAP1	4.42	4.25	4.39
215133_s_at	family with seque	FAM153A /// FAM	4.31	4.12	4.08
214753_at	NEDD4 binding pr	N4BP2L2	7.98	8.28	8.16
78495_at	zinc finger family	ZNF783	8.14	7.84	7.96
203293_s_at	lectin, mannose-b	LMAN1	6.28	5.99	6.33
218119_at	translocase of inn	LOC10431 /// TIM	10.53	10.46	10.50
218847_at	insulin-like growth	IGF2BP2	11.19	11.19	11.22
206951_at	histone cluster 1,	HIST1H4E	7.07	7.18	6.93
204553_x_at	inositol polyphos	INPP4A	6.18	6.10	5.87
215616_s_at	lysine (K)-specific	KDM4B	7.39	7.41	7.22
210594_x_at	myelin protein ze	MPZL1	8.54	8.43	8.53
221618_s_at	TAF9B RNA polym	TAF9B	5.22	5.56	5.56
214329_x_at	tumor necrosis fa	TNFSF10	3.28	3.33	3.24
215895_x_at	Perilipin 2	PLIN2	5.94	5.79	5.76
213289_at	Apolipoprotein O-	APOOL	8.49	8.53	8.55
217058_at	GNAS complex loc	GNAS	6.92	6.98	7.00
214850_at	glucuronidase, be	LOC100170939	6.87	7.14	7.02
216717_at	Family with seque	FAM48A	4.36	4.62	4.36
214342_at	ataxin 7-like 1	ATXN7L1	4.98	4.69	4.61
202881_x_at	cytohesin 1	CYTH1	7.19	7.08	6.97
207500_at	caspase 5, apopto	CASP5	5.53	5.51	5.57
217299_s_at	nibrin	NBN	6.83	6.96	7.30
222283_at	zinc finger protein	ZNF480	4.18	4.27	4.31
212145_at	mitochondrial ribo	MRPS27	11.44	11.45	11.49
211760_s_at	vesicle-associated	VAMP4	6.67	6.74	7.03
217821_s_at	WW domain bindi	WBP11	8.61	8.61	8.49
211439_at	splicing factor, ar	SFRS7	6.80	6.92	6.88
201944_at	hexosaminidase B	HEXB	11.79	11.74	11.74
208437_at	chloride channel 1	CLCN1	6.23	6.37	6.20
201002_s_at	transmembrane p	TMEM189 /// TME	10.34	10.29	10.27
208508_s_at	olfactory receptor	OR2J2	3.39	3.39	3.35
209897_s_at	slit homolog 2 (Dr	SLIT2	8.99	8.96	8.95
207571_x_at	chromosome 1 op	C1orf38	5.56	5.42	5.37
211184_s_at	Usher syndrome 1	USH1C	5.94	5.64	5.35
213737_x_at	Golgin subfamily A	LOC728498	8.16	8.31	8.30
200943_at	high-mobility grou	HMGN1	12.26	12.33	12.39
215808_at	kallikrein-related	KLK10	4.03	3.77	4.21
213052_at	Protein kinase, cA	PRKAR2A	10.37	10.31	10.25
208641_s_at	ras-related C3 bot	RAC1	13.26	13.24	13.22
218381_s_at	U2 small nuclear R	U2AF2	8.72	8.75	8.81
206758_at	endothelin 2	EDN2	7.63	8.05	7.97
205626_s_at	calbindin 1, 28kDa	CALB1	3.76	3.74	3.47
218791_s_at	chromosome 15 o	C15orf29	8.41	8.34	8.59

220067_at	spectrin, beta, noı	SPTBN5	5.17	5.21	5.47
202116_at	D4, zinc and doub	DPF2	9.46	9.17	9.36
209388_at	poly(A) polymeras	PAPOLA	9.72	9.68	9.97
205423_at	adaptor-related p	AP1B1	8.25	8.20	8.13
205152_at	solute carrier fam	SLC6A1	3.64	3.80	3.56
201004_at	signal sequence re	SSR4	12.23	12.15	12.23
208086_s_at	dystrophin	DMD	4.39	4.25	4.28
36830_at	mitochondrial inte	MIPEP	8.02	8.10	8.21
220018_at	Cas-Br-M (murine	CBLL1	5.90	5.91	5.87
204441_s_at	polymerase (DNA	POLA2	8.75	8.85	8.54
213996_at	yippee-like 1 (Dro	YPEL1	5.38	5.33	5.40
203861_s_at	actinin, alpha 2	ACTN2	5.49	5.48	6.02
202959_at	methylmalonyl Cc	MUT	8.36	8.61	8.48
206335_at	galactosamine (N-	GALNS	7.89	7.91	8.05
221048_x_at	chromosome 17 c	C17orf80	6.21	6.21	6.11
216139_s_at	mitogen-activatec	MAPK8IP3	5.97	6.15	6.20
211061_s_at	mannosyl (alpha-1	MGAT2	9.65	9.73	9.63
206652_at	zinc finger, MYM-1	ZMYM5	6.19	6.32	6.22
222348_at	---	---	5.07	5.68	5.36
217803_at	golgi phosphoprot	GOLPH3	10.94	10.87	11.22
205232_s_at	platelet-activating	PAFAH2	7.05	6.81	7.06
208337_s_at	nuclear receptor s	NR5A2	4.67	4.71	4.60
202172_at	vascular endothel	VEZF1	10.52	10.30	10.59
210735_s_at	carbonic anhydras	CA12	8.72	8.75	8.72
218825_at	EGF-like-domain,	EGFL7	6.93	6.77	6.62
220685_at	family with seque	FAM120C	7.45	7.64	7.34
210581_x_at	POZ (BTB) and AT	PATZ1	5.22	4.98	5.24
212533_at	WEE1 homolog (S	WEE1	10.85	10.94	11.09
201380_at	cartilage associate	CRTAP	9.92	9.85	9.91
215508_at	budding uninhibit	BUB1	4.32	4.45	4.42
200991_s_at	sorting nexin 17	SNX17	9.74	9.53	9.61
218163_at	malignant T cell ar	MCTS1	11.34	11.34	11.34
206587_at	chaperonin contai	CCT6B	4.97	4.79	4.84
207566_at	major histocompa	MR1	6.39	6.39	6.24
222309_at	---	---	7.01	7.17	7.39
204335_at	coiled-coil domair	CCDC94	8.44	8.29	8.41
210501_x_at	eukaryotic transla	EIF3K	12.09	11.94	12.01
208615_s_at	protein tyrosine p	PTP4A2	11.17	11.20	11.22
204150_at	stabilin 1	STAB1	6.16	6.00	6.05
217598_at	---	---	7.58	7.64	7.53
209907_s_at	intersectin 2	ITSN2	7.29	7.31	7.40
217143_s_at	T cell receptor alp	TRA@ /// TRD@	4.17	4.33	4.37
213483_at	peptidylprolyl isoı	PPWD1	9.46	9.66	9.65
211998_at	H3 histone, family	H3F3B	8.16	8.20	8.16
211761_s_at	calcyclin binding p	CACYBP	11.78	11.75	11.73
216981_x_at	sialophorin	SPN	6.53	6.64	6.73
203362_s_at	MAD2 mitotic arr	MAD2L1	11.08	11.05	11.14

219315_s_at	transmembrane p	TMEM204	8.82	8.69	8.80
217142_at	---	---	7.08	6.98	7.01
219931_s_at	kelch-like 12 (Dro:	KLHL12	7.04	7.10	7.18
202284_s_at	cyclin-dependent	CDKN1A	9.60	9.45	9.41
203655_at	X-ray repair comp	XRCC1	8.05	7.90	8.02
1487_at	estrogen-related r	ESRRA	9.02	9.04	9.09
206627_s_at	synovial sarcoma,	SSX1	3.28	3.36	3.34
201613_s_at	adaptor-related p	AP1G2	6.82	6.83	6.66
209461_x_at	WD repeat domai	WDR18	8.14	8.07	8.15
221062_at	heparan sulfate (g	HS3ST3B1	3.42	3.40	3.43
205282_at	low density lipopr	LRP8	9.42	9.57	9.55
208895_s_at	DEAD (Asp-Glu-Al	DDX18	11.34	11.42	11.44
210298_x_at	four and a half LIN	FHL1	4.09	4.07	4.18
212536_at	ATPase, class VI, t	ATP11B	8.77	8.77	9.07
209396_s_at	chitinase 3-like 1 (	CHI3L1	4.04	3.92	3.89
213868_s_at	Dehydrogenase/r	DHRS7	5.33	5.15	5.05
209612_s_at	alcohol dehydroge	ADH1B	3.75	3.34	3.59
205466_s_at	heparan sulfate (g	HS3ST1	6.23	6.59	6.30
217078_s_at	CD300a molecule	CD300A	3.73	3.66	3.82
211284_s_at	granulin	GRN	8.99	8.84	8.98
222130_s_at	FtsJ homolog 2 (E.	FTSJ2	8.98	9.19	9.20
211371_at	mitogen-activatec	MAP2K5	4.71	4.87	4.54
208999_at	septin 8	8-Sep	9.37	9.52	9.36
213057_at	ATP synthase mitc	ATPAF2	6.19	5.85	6.05
211300_s_at	tumor protein p5:	TP53	4.58	4.64	4.67
210810_s_at	solute carrier fam	SLC6A5	5.69	5.60	5.42
206144_at	membrane associ	MAGI1	4.58	4.70	4.72
210424_s_at	golgin A8 family, r	GOLGA8A /// GOL	5.93	5.94	5.83
205187_at	SMAD family men	SMAD5	7.23	7.16	7.48
218502_s_at	trichorhinophalan	TRPS1	7.99	7.83	8.13
211485_s_at	fibroblast growth	FGF18	6.00	6.09	5.78
213584_s_at	CREB/ATF bZIP tra	CREBZF	6.17	6.02	6.09
207867_at	paired box 4	PAX4	3.30	3.27	3.20
206990_at	tenascin R (restric	TNR	4.02	4.16	3.79
35820_at	GM2 ganglioside a	GM2A	7.51	7.55	7.51
214353_at	---	---	5.72	5.42	5.09
203108_at	G protein-couplec	GPRC5A	5.04	5.02	4.93
205054_at	nebulin	NEB	4.00	4.19	4.00
203869_at	ubiquitin specific		USP46	7.33	7.67	7.65
220853_at	glycosyltransferas	GTDC1	4.06	4.52	4.68
212380_at	FtsJ methyltransfe	FTSJD2	9.06	9.11	9.12
205396_at	SMAD family men	SMAD3	5.28	5.06	5.24
216666_at	maltase-glucoamy	LOC100124692 ///	4.03	3.95	3.91
212646_at	raftlin, lipid raft lir	RFTN1	9.84	9.68	9.75
218669_at	RAP2C, member c	RAP2C	9.15	9.19	9.32
205808_at	aspartate beta-hy	ASPH	5.92	5.61	5.52
202004_x_at	succinate dehydrc	SDHC	10.00	9.94	9.79

204571_x_at	protein (peptidylp	PIN4		8.92	9.07	9.03
201364_s_at	ornithine decarbo	OAZ2		8.94	8.99	8.74
201583_s_at	Sec23 homolog B	SEC23B		10.01	10.04	10.04
218201_at	NADH dehydroger	NDUFB2		11.10	11.14	11.17
206686_at	pyruvate dehydro	PDK1		6.22	6.32	6.19
221944_at	hypothetical LOC6	FLJ42627		6.02	6.04	5.79
217422_s_at	CD22 molecule	CD22		3.88	3.84	3.66
213946_s_at	obscurin-like 1	OBSL1		7.65	7.59	7.46
213263_s_at	poly(rC) binding p	PCBP2		8.11	7.89	8.11
219008_at	chromosome 2 op	C2orf43		9.06	9.10	9.11
222090_at	hypothetical prote	LOC100134713		7.30	7.15	6.90
209599_s_at	prune homolog (D	PRUNE		6.05	6.26	5.93
206438_x_at	tectonic family me	TCTN2		8.33	8.38	8.26
221575_at	selenocysteine lya	SCLY		8.02	8.13	7.96
214691_x_at	family with seque	FAM63B		5.43	5.60	5.66
202743_at	phosphoinositide-	PIK3R3		8.26	8.14	8.10
218758_s_at	ribosomal RNA pr	RRP1		8.50	8.31	8.44
220751_s_at	chromosome 5 op	C5orf4		5.28	5.15	5.07
213564_x_at	lactate dehydroge	LDHB		13.93	13.98	13.93
216569_at	---	---		5.04	5.04	4.89
219858_s_at	major facilitator s	MFSD6		5.72	5.70	5.83
208657_s_at	septin 9		9-Sep	8.61	8.55	8.55
218146_at	glycosyltransferas	GLT8D1		10.63	10.63	10.65
200813_s_at	platelet-activating	PAFAH1B1		7.65	7.69	7.68
201743_at	CD14 molecule	CD14		5.54	5.50	5.47
206088_at	leucine rich repea	LRRC37A3		4.69	4.37	4.63
221932_s_at	glutaredoxin 5	GLRX5		11.08	11.16	11.14
206560_s_at	melanoma inhibit	MIA		6.83	6.63	6.57
208177_at	solute carrier fam	SLC34A1		6.03	6.09	5.89
208523_x_at	histone cluster 1,	HIST1H2BI		6.92	6.90	7.02
209144_s_at	core-binding facto	CBFA2T2		6.37	6.54	6.34
214545_s_at	proline synthetase	PROSC		8.08	8.20	8.15
208232_x_at	---	---		7.82	7.57	7.40
221258_s_at	kinesin family mer	KIF18A		7.77	7.88	8.05
201721_s_at	lysosomal protein	LAPTM5		10.51	10.41	10.42
221208_s_at	chromosome 11 o	C11orf61		7.42	7.60	7.59
211363_s_at	methylthioadenos	MTAP		4.38	4.21	4.28
205586_x_at	VGF nerve growth	VGF		7.33	7.28	7.35
219266_at	zinc finger proteir	ZNF350		5.92	5.73	5.79
216572_at	forkhead box L1	FOXL1		4.40	4.13	4.15
203649_s_at	phospholipase A2	PLA2G2A		5.44	5.39	5.33
221550_at	COX15 homolog, c	COX15		7.10	6.96	7.23
203356_at	calpain 7	CAPN7		8.88	8.82	9.21
214916_x_at	immunoglobulin h	IGH@ /// IGHA1 //		5.62	5.51	5.34
210718_s_at	ADP-ribosylation f	ARL17A /// LOC10		5.73	5.88	5.94
201648_at	Janus kinase 1	JAK1		10.89	10.92	11.05
209730_at	sema domain, imr	SEMA3F		7.81	7.68	7.49

220973_s_at	SHANK-associatec	SHARPIN	8.17	8.10	8.21
206281_at	adenylate cyclase	ADCYAP1	3.81	3.84	3.66
214414_x_at	hemoglobin, alpha	HBA1 /// HBA2	3.84	3.95	3.83
201644_at	tissue specific tra	TSTA3	8.95	8.85	8.75
212436_at	tripartite motif-co	TRIM33	7.50	7.76	7.60
200962_at	ribosomal protein	RPL31	9.50	9.39	9.53
212504_at	DIP2 disco-interac	DIP2C	8.96	8.86	8.79
218794_s_at	thioredoxin-like 4	TXNL4B	8.61	8.60	8.58
208341_x_at	chorionic somato	CSH2	4.28	4.39	4.36
213820_s_at	StAR-related lipid	STARD5	6.75	6.76	6.90
219132_at	pellino homolog 2	PELI2	5.80	5.44	5.48
37793_r_at	RAD51-like 3 (S. c	RAD51L3	5.05	5.10	5.04
219727_at	dual oxidase 2	DUOX2	3.90	3.88	3.73
213668_s_at	SRY (sex determin	SOX4	6.62	6.70	6.57
208541_x_at	transcription facto	TFAM	9.08	9.02	9.00
201096_s_at	ADP-ribosylation f	ARF4	11.05	11.04	10.99
AFFX-HSAC07/X	actin, beta	ACTB	13.58	13.67	13.54
214317_x_at	ribosomal protein	RPS9	12.58	12.53	12.71
205517_at	GATA binding pro	GATA4	3.96	3.87	3.91
210891_s_at	general transcript	GTF2I /// GTF2IP1	11.08	11.17	11.09
206752_s_at	DNA fragmentatic	DFFB	7.00	7.13	6.96
220960_x_at	ribosomal protein	RPL22	14.00	14.06	14.10
210652_s_at	tetratricopeptide	TTC39A	4.27	4.17	4.34
207143_at	cyclin-dependent	CDK6	6.14	6.38	5.90
217834_s_at	synaptotagmin bi	SYNCRIP	10.34	10.35	10.44
210920_x_at	---	---	4.85	4.81	4.99
202618_s_at	methyl CpG bindi	MECP2	7.81	7.56	7.69
216118_at	---	---	5.11	5.09	4.99
210572_at	protocadherin alp	PCDHA2	3.55	3.47	3.46
207896_s_at	deleted in lung an	DLEC1	4.96	4.66	5.04
215536_at	major histocompa	HLA-DQB2	4.08	4.27	4.45
213238_at	ATPase, class V, ty	ATP10D	8.33	8.32	8.52
208767_s_at	lysosomal protein	LAPTM4B	10.85	10.87	10.83
211238_at	ADAM metallope	ADAM7	5.05	5.09	5.00
209079_x_at	protocadherin gai	PCDHGA1 /// PCDI	7.51	7.49	7.36
212588_at	protein tyrosine p	PTPRC	3.57	3.68	3.43
207098_s_at	mitofusin 1	MFN1	9.06	9.25	9.40
205381_at	leucine rich repea	LRRC17	3.71	3.90	3.88
221887_s_at	deafness, autoso	DFNB31	7.59	7.57	7.44
221142_s_at	peroxisomal trans	PECR	7.33	7.24	7.29
210745_at	one cut homeobo	ONECUT1	4.20	4.20	4.29
204039_at	CCAAT/enhancer	CEBPA	6.03	5.37	5.68
209688_s_at	coiled-coil domair	CCDC93	8.48	8.42	8.62
202910_s_at	CD97 molecule	CD97	10.23	10.02	10.25
203738_at	chromosome 5 op	C5orf22	9.65	9.69	9.75
207036_x_at	glutamate recept	GRIN2D	4.11	4.03	3.95
218161_s_at	ceroid-lipofuscino	CLN6	7.58	7.56	7.51

211365_s_at	protocadherin alp	PCDHA2	4.30	4.68	4.39
204060_s_at	protein kinase, X-l	PRKX /// PRKY	8.24	8.23	8.08
202751_at	tuftelin interacting	TFIP11	4.49	4.65	4.94
215553_x_at	---	---	7.88	8.07	7.78
212385_at	transcription facto	TCF4	7.71	7.76	7.84
36129_at	small G protein sig	SGSM2	8.68	8.74	8.67
217780_at	chromosome 19 c	C19orf56	10.67	10.59	10.68
215027_at	Rap guanine nucle	RAPGEF3	5.55	5.60	5.42
205095_s_at	ATPase, H+ transp	ATP6V0A1	6.23	6.10	5.91
200771_at	laminin, gamma 1	LAMC1	10.28	10.19	10.31
202920_at	ankyrin 2, neuron	ANK2	7.41	7.27	7.60
214679_x_at	guanine nucleotid	GNA11	7.99	7.98	8.10
212254_s_at	dystonin	DST	10.94	11.15	11.05
203686_at	N-methylpurine-D	MPG	9.33	9.16	9.32
202226_s_at	v-crk sarcoma viru	CRK	8.39	8.41	8.22
210277_at	adaptor-related p	AP4S1	6.50	6.55	6.36
202805_s_at	ATP-binding casse	ABCC1	6.64	6.61	6.55
214940_s_at	Smg-6 homolog, n	SMG6	6.40	6.34	6.20
217276_x_at	serine hydrolase-l	SERHL2	5.48	5.39	5.46
212247_at	nucleoporin 205kl	NUP205	9.98	9.98	10.04
201671_x_at	ubiquitin specific		USP14	10.27	10.39	10.35
215591_at	SATB homeobox 2	SATB2	3.95	4.18	3.92
201027_s_at	eukaryotic transla	EIF5B	9.39	9.53	9.39
220692_at	similar to HSPC04	LOC100286937	5.98	5.77	5.98
217484_at	complement com	CR1	4.30	4.36	4.13
218848_at	THO complex 6 hc	THOC6	8.72	8.73	8.82
209506_s_at	nuclear receptor s	NR2F1	4.76	4.57	4.47
203184_at	fibrillin 2	FBN2	3.18	3.18	3.55
209980_s_at	serine hydroxyme	SHMT1	7.06	7.05	7.08
210786_s_at	Friend leukemia v	FLI1	3.75	4.10	3.66
208870_x_at	ATP synthase, H+	ATP5C1	12.69	12.65	12.65
204295_at	surfeit 1	SURF1	10.87	10.81	10.78
203943_at	kinesin family me	KIF3B	8.03	8.19	8.21
205056_s_at	G protein-couplec	GPR162	7.12	6.72	6.71
211878_at	Immunoglobulin h	IGH@	4.53	4.28	4.32
65438_at	KIAA1609	KIAA1609	5.39	5.59	5.57
215715_at	solute carrier fam	SLC6A2	6.25	6.15	6.17
211441_x_at	cytochrome P450,	CYP3A43	3.88	3.90	3.80
212002_at	chromosome 1 op	C1orf144	8.01	8.33	8.16
220737_at	ribosomal protein	RPS6KA6	4.26	4.12	3.97
220919_s_at	chromosome 10 c	C10orf79	3.24	3.31	3.20
204097_s_at	RNA binding moti	RBMX2	7.92	7.88	7.90
200659_s_at	prohibitin	PHB	9.70	9.66	9.65
221110_x_at	phosphodiesteras	PDE11A	5.23	5.21	5.19
211866_x_at	hemochromatosis	HFE	6.43	6.37	6.12
201971_s_at	ATPase, H+ transp	ATP6V1A	7.24	7.48	7.74
205499_at	sushi-repeat-cont	SRPX2	5.69	5.71	5.42

202199_s_at	SFRS protein kinas	SRPK1	7.88	7.85	7.65
210454_s_at	potassium inward	KCNJ6	5.99	6.04	5.57
220410_s_at	calmodulin regula	CAMSAP1	4.20	4.39	4.28
203017_s_at	synovial sarcoma,	SSX2IP	8.53	8.69	8.71
222105_s_at	NFKB inhibitor int	NKIRAS2	7.66	7.74	7.66
216911_s_at	hypermethylated	HIC2	6.80	6.89	6.83
210960_at	adrenergic, alpha-	ADRA1D	4.51	4.55	4.76
205305_at	fibrinogen-like 1	FGL1	6.21	6.03	6.42
207376_at	VENT homeobox h	VENTX	5.44	5.37	5.00
212382_at	transcription facto	TCF4	7.99	8.05	7.97
214631_at	zinc finger and BT	ZBTB33	3.43	3.31	3.20
216670_at	kallikrein-related	KLK13	6.14	5.94	6.01
212960_at	TBC1 domain fam	TBC1D9	5.71	5.64	5.76
210957_s_at	AF4/FMR2 family,	AFF2	4.91	4.66	4.73
216659_at	dihydrofolate redu	LOC1720	3.48	3.36	3.59
213173_at	pecanex homolog	PCNX	5.71	5.63	5.93
209157_at	DnaJ (Hsp40) hom	DNAJA2	10.41	10.55	10.53
210633_x_at	keratin 10	KRT10	10.79	10.81	10.84
206586_at	cannabinoid recep	CNR2	5.36	5.42	5.03
214995_s_at	apolipoprotein B r	APOBEC3F /// APC	5.01	4.78	4.93
212714_at	La ribonucleoprot	LARP4	10.66	10.72	10.75
221858_at	TBC1 domain fam	TBC1D12	8.18	8.06	8.29
209820_s_at	transducin (beta)-	TBL3	8.65	8.60	8.45
203967_at	cell division cycle	CDC6	7.94	8.14	8.24
221374_at	fibroblast growth	FGF16	4.61	4.71	4.57
213849_s_at	protein phosphata	PPP2R2B	5.31	5.34	5.18
203890_s_at	death-associated	DAPK3	4.52	4.72	4.56
204957_at	origin recognition	ORC5L	10.29	10.31	10.37
207937_x_at	fibroblast growth	FGFR1	7.71	7.84	7.48
210924_at	olfactomedin 1	OLFM1	6.58	6.45	6.41
204811_s_at	calcium channel, v	CACNA2D2	5.75	5.72	5.39
211325_x_at	destrin-2 pseudog	LOC171220	7.18	7.21	7.57
216288_at	cysteinyl leukotrie	CYSLTR1	3.78	3.55	3.76
201981_at	pregnancy-associa	PAPPA	5.19	4.94	5.10
218738_s_at	ring finger protein	RNF138	10.39	10.37	10.55
217172_at	---	---	4.53	4.42	4.28
210194_at	phospholipase A2	PLA2R1	3.57	3.55	3.85
202095_s_at	baculoviral IAP re	BIRC5	11.00	10.97	11.02
205972_at	solute carrier fam	SLC38A3	6.50	6.14	6.54
217948_at	family with seque	FAM127B	8.72	8.70	8.73
202305_s_at	fasciculation and	FEZ2	10.08	10.06	10.14
216287_at	---	---	3.93	3.78	3.65
221589_s_at	aldehyde dehydro	ALDH6A1	7.83	7.80	8.02
219234_x_at	secernin 3	SCRN3	4.88	4.81	4.89
222095_s_at	family with seque	FAM163A	5.59	5.50	5.28
214433_s_at	selenium binding	SELENBP1	7.28	7.19	7.27
212613_at	butyrophilin, subf	BTN3A2	8.28	8.29	8.08

204940_at	phospholamban	PLN	2.97	3.05	3.03
214465_at	orosomucoid 1 //,	ORM1 /// ORM2	4.47	4.36	4.46
210816_s_at	cytochrome b-561	CYB561	5.86	5.82	6.06
214798_at	ATPase, Ca++ tran	ATP2C2	5.79	5.57	5.69
217916_s_at	family with seque	FAM49B	9.08	9.20	9.24
207623_at	ATP-binding casse	ABCF2	5.53	5.43	5.44
204244_s_at	DBF4 homolog (S.	DBF4	10.33	10.47	10.62
207435_s_at	serine/arginine re	SRRM2	8.92	9.12	8.95
208992_s_at	signal transducer	STAT3	9.66	9.54	9.60
204883_s_at	HUS1 checkpoint	HUS1	8.70	8.76	8.80
213327_s_at	ubiquitin specific	USP12	7.50	7.49	7.45
213724_s_at	pyruvate dehydro	PDK2	6.80	6.78	6.87
36566_at	cystinosis, nephro	CTNS	6.52	6.46	6.43
221295_at	cell death-inducin	CIDEA	4.89	4.76	5.57
203286_at	ring finger proteir	RNF44	8.22	8.06	8.27
215855_s_at	TATA element mo	TMF1	5.76	5.87	5.82
221722_x_at	leucine zipper, pu	LZTS1	5.41	5.19	4.82
205248_at	dopey family men	DOPEY2	5.49	5.54	5.77
208622_s_at	ezrin	EZR	9.89	9.84	9.84
204185_x_at	peptidylprolyl isor	PPID	10.32	10.53	10.54
40489_at	atrophin 1	ATN1	7.15	7.19	7.16
200936_at	ribosomal protein	RPL8	14.29	14.24	14.33
208631_s_at	hydroxyacyl-CoA	HADHA	10.18	10.06	10.10
219718_at	FGGY carbohydrat	FGGY	7.61	7.50	7.54
209873_s_at	plakophilin 3	PKP3	8.19	8.28	8.09
217824_at	ubiquitin-conjugat	UBE2J1	7.72	7.66	7.71
205956_x_at	PSMC3 interacting	PSMC3IP	8.98	8.86	8.95
214002_at	myosin, light chai	MYL6	7.54	7.31	7.33
207852_at	chemokine (C-X-C	CXCL5	3.36	3.28	3.22
243_g_at	microtubule-asso	MAP4	10.82	10.81	10.89
202160_at	CREB binding prot	CREBBP	9.67	9.38	9.84
203716_s_at	dipeptidyl-peptida	DPP4	4.84	4.66	4.59
211884_s_at	class II, major hist	CIITA	5.01	5.11	5.21
212812_at	serine incorporatc	SERINC5	7.60	7.72	7.96
215022_x_at	zinc finger proteir	ZNF33B	7.09	7.10	7.05
209260_at	stratifin	SFN	7.75	7.97	7.84
210011_s_at	Ewing sarcoma br	EWSR1	8.91	8.87	8.94
216945_x_at	PAS domain conta	PASK	6.00	6.64	6.61
220674_at	CD22 molecule	CD22	4.42	4.39	4.41
205228_at	RNA binding moti	RBMS2	7.21	7.36	7.25
220560_at	chromosome 11 c	C11orf21	4.10	4.03	3.97
218581_at	abhydrolase dom	ABHD4	9.12	8.93	8.76
210543_s_at	protein kinase, DN	PRKDC	9.92	9.79	9.83
40020_at	cadherin, EGF LAG	CELSR3	8.35	8.47	8.38
213980_s_at	C-terminal binding	CTBP1	9.66	9.57	9.72
221193_s_at	zinc finger, CCHC	ZCCHC10	8.62	8.64	8.73
211410_x_at	killer cell immuno	KIR2DL5A	4.85	4.85	4.73

219898_at	G protein-couplec	GPR85	3.87	3.71	3.77
220178_at	chromosome 19 c	C19orf28	7.79	7.69	7.65
203464_s_at	epsin 2	EPN2	7.66	7.73	7.57
210287_s_at	fms-related tyrosi	FLT1	3.83	3.96	3.64
202670_at	mitogen-activatec	MAP2K1	10.37	10.41	10.45
210030_at	---	---	6.50	6.49	6.28
206596_s_at	neural retina leuci	NRL	5.24	5.16	5.36
202065_s_at	protein tyrosine p	PPFIA1	8.15	8.24	8.01
202001_s_at	NADH dehydroger	NDUFA6	11.75	11.79	11.83
206857_s_at	FK506 binding prc	FKBP1B	6.30	6.55	6.54
217970_s_at	CCR4-NOT transcr	CNOT6	8.71	8.97	9.01
207960_at	---	---	4.82	4.53	4.71
208919_s_at	NAD kinase	NADK	9.85	9.76	9.83
212333_at	family with seque	FAM98A	10.35	10.43	10.39
209217_s_at	WD repeat domai	WDR45	7.97	7.60	7.93
204479_at	osteoclast stimula	OSTF1	8.06	8.22	8.20
201698_s_at	splicing factor, arg	SFRS9	11.99	11.95	11.98
203161_s_at	ring finger protein	RNF8	4.24	4.45	4.21
211906_s_at	serpin peptidase i	SERPINB4	3.40	3.35	3.05
220119_at	erythrocyte meml	EPB41L4A	4.18	3.93	3.90
221364_at	glutamate recepto	GRID2	4.49	4.48	4.34
210272_at	cytochrome P450,	CYP2B7P1	5.64	5.66	5.43
210176_at	toll-like receptor 1	TLR1	4.17	3.96	3.89
219314_s_at	zinc finger protein	ZNF219	7.77	7.71	7.57
204076_at	ectonucleoside tri	ENTPD4	9.43	9.55	9.59
217909_s_at	MAX-like protein X	MLX	8.28	8.29	8.20
219672_at	alpha hemoglobin	AHSP	6.27	6.19	6.29
212910_at	THAP domain con	THAP11	8.29	8.22	8.29
210265_x_at	POU class 5 home	POU5F1P3	6.54	6.44	6.55
220150_s_at	family with seque	FAM184A	4.99	5.60	5.15
217750_s_at	ubiquitin-conjugat	UBE2Z	10.51	10.51	10.62
214158_s_at	PR domain contai	PRDM10	6.51	6.56	6.28
38766_at	Snf2-related CREB	SRCAP	7.47	7.69	7.38
210180_s_at	transformer 2 bet	TRA2B	9.03	8.98	9.08
209655_s_at	transmembrane p	TMEM47	8.12	8.13	8.22
220523_at	EF-hand domain ((	EFHC2	3.13	3.17	3.00
207390_s_at	smoothelin	SMTN	5.78	5.69	5.59
205862_at	growth regulation	GREB1	3.14	3.19	3.09
202673_at	dolichyl-phosphat	DPM1	11.79	11.78	11.95
216240_at	---	---	5.78	5.81	5.77
204605_at	cell growth regula	CGRRF1	8.99	8.98	9.16
39891_at	zinc finger protein	ZNF710	7.07	6.97	6.99
217664_at	---	---	5.95	5.81	5.77
213105_s_at	chromosome 16 c	C16orf42	8.60	8.60	8.72
208244_at	bone morphogene	BMP3	4.69	4.44	4.70
47105_at	dihydrouridine syı	DUS2L	7.94	7.87	7.81
210138_at	regulator of G-prc	RGS20	4.20	4.92	4.65

214222_at	dynein, axonemal	DNAH7	5.10	4.87	4.95
217567_at	transglutaminase	TGM4	5.60	5.48	5.20
208658_at	protein disulfide i:	PDIA4	9.24	9.16	9.02
215621_s_at	immunoglobulin h	IGHD	6.34	6.14	6.14
219290_x_at	dual adaptor of pl	DAPP1	5.40	5.38	5.26
218921_at	single immunoglo	SIGIRR	6.61	6.72	6.58
217680_x_at	ribosomal protein	RPL10	4.27	4.22	4.23
200887_s_at	signal transducer	STAT1	10.63	10.74	10.92
220261_s_at	zinc finger, DHHC-	ZDHHC4	10.16	10.12	10.06
37512_at	hydroxysteroid (1	HSD17B6	4.30	4.03	4.16
64900_at	transmembrane p	TMEM231	7.63	7.70	7.74
200922_at	KDEL (Lys-Asp-Glu	KDELR1	8.37	8.36	8.38
202493_x_at	chorionic somatoı	CSH1	3.60	3.34	3.40
208561_at	ATP-binding casse	ABCC9	4.27	4.10	4.35
211699_x_at	hemoglobin, alpha	HBA1 /// HBA2	6.38	6.35	6.52
58916_at	potassium channe	KCTD14	7.81	7.81	7.81
215529_x_at	DIP2 disco-interac	DIP2A	8.72	8.98	8.98
201650_at	keratin 19	KRT19	4.14	3.84	3.51
216142_at	---	---	6.59	6.21	6.37
52975_at	family with seque	FAM125B	6.52	6.41	6.40
207487_at	---	---	3.71	4.04	3.79
216998_s_at	ADAM metallopep	ADAM5P	4.27	4.17	4.18
202392_s_at	phosphatidylserin	PISD	8.23	8.26	8.22
1053_at	replication factor	RFC2	9.01	9.02	9.08
219701_at	tropomodulin 2 (r	TMOD2	3.23	3.19	3.14
218764_at	protein kinase C, e	PRKCH	9.85	9.55	9.90
220587_s_at	MTOR associated	MLST8	8.19	8.11	8.11
221174_at	---	---	3.84	3.87	3.91
216421_at	---	---	8.16	8.02	8.23
209762_x_at	SP110 nuclear boc	SP110	8.17	8.17	8.33
217683_at	Hemoglobin, epsil	HBE1	4.40	4.20	4.14
222321_at	angiotensin II rece	AGTR2	4.54	4.73	4.95
217474_at	---	---	5.79	5.60	5.67
219632_s_at	transient receptoı	TRPV1	4.65	4.46	4.38
204432_at	SRY (sex determin	SOX12	7.27	7.13	7.14
217932_at	mitochondrial ribo	MRPS7	10.46	10.45	10.49
206436_at	metallophosphoe:	MPPED1	6.32	6.04	6.30
206574_s_at	protein tyrosine p	PTP4A3	5.37	5.31	5.31
218417_s_at	solute carrier fam	SLC48A1	6.14	6.24	6.15
211173_at	cholecystokinin A	CCKAR	6.32	6.33	6.26
213597_s_at	CTD (carboxy-tern	CTDSPL	5.44	5.33	5.13
220201_at	ring finger and CC	RC3H2	7.77	7.88	7.87
217001_x_at	major histocompa	HLA-DOA	6.35	6.53	6.39
214486_x_at	CASP8 and FADD-l	CFLAR	8.05	8.19	8.09
208334_at	N-deacetylase/N-:	NDST4	4.80	4.62	4.82
203100_s_at	chromodomain pr	CDYL	8.97	8.93	9.00
202437_s_at	cytochrome P450,	CYP1B1	3.58	3.63	3.88

219485_s_at	proteasome (pros	PSMD10	10.49	10.62	10.59
219918_s_at	asp (abnormal spi	ASPM	10.65	11.11	10.85
220442_at	UDP-N-acetyl-alpl	GALNT4 /// POC1E	4.79	4.88	4.62
210055_at	thyroid stimulatin	TSHR	3.11	3.47	3.22
213023_at	utrophin	UTRN	5.68	5.65	5.70
208959_s_at	endoplasmic retic	ERP44	10.17	10.10	10.22
215479_at	---	---	4.79	5.24	4.92
214697_s_at	ROD1 regulator of	ROD1	8.09	8.07	8.19
214974_x_at	chemokine (C-X-C	CXCL5	3.25	3.38	3.20
207731_at	---	---	3.98	4.00	3.95
217167_x_at	glycerol kinase	GK	5.94	5.93	5.77
208651_x_at	CD24 molecule	CD24	3.82	4.03	3.84
213007_at	Fanconi anemia, c	FANCI	10.11	10.08	10.23
222085_at	Family with seque	FAM174B	5.18	5.28	5.24
215167_at	mediator complex	MED14	4.50	4.81	4.47
219927_at	FCF1 small subuni	FCF1	6.87	6.89	7.04
218939_at	leucine zipper-EF-	LETM1	7.62	7.40	7.52
218106_s_at	mitochondrial ribo	MRPS10	10.63	10.69	10.79
200079_s_at	lysyl-tRNA synthet	KARS	11.35	11.30	11.40
211780_x_at	dynactin 1 (p150,	DCTN1	8.50	8.52	8.47
203172_at	fragile X mental re	FXR2	8.40	8.43	8.41
200647_x_at	eukaryotic transla	EIF3C /// EIF3CL	12.14	12.01	12.06
36829_at	period homolog 1	PER1	8.50	8.39	8.52
218509_at	lipid phosphate pl	LPPR2	7.10	6.74	7.09
204055_s_at	CTAGE family, me	CTAGE5	6.24	6.33	6.45
205543_at	heat shock 70kDa	HSPA4L	8.00	8.49	8.54
215721_at	immunoglobulin h	IGHG1	5.64	5.29	5.54
209998_at	phosphatidylinosi	PIGO	7.81	7.73	7.93
57703_at	SUMO1/sentrin sp	SENP5	7.17	7.25	7.23
204272_at	lectin, galactoside	LGALS4	5.33	5.28	5.45
214258_x_at	K(lysine) acetyltra	KAT5	8.30	8.30	8.42
203859_s_at	paralemmin	PALM	6.66	6.73	6.59
208390_s_at	glucagon-like pep	GLP1R	6.13	6.13	6.02
213392_at	IQ motif containir	IQCK	7.14	6.95	7.14
200003_s_at	ribosomal protein	RPL28	14.77	14.75	14.80
201704_at	ectonucleoside tri	ENTPD6	8.39	8.42	8.53
203383_s_at	golgin A1	GOLGA1	7.49	7.55	7.35
215168_at	translocase of inn	TIMM17A	3.02	3.08	3.00
206800_at	methylenetetrahy	MTHFR	5.20	5.45	5.42
202721_s_at	glutamine--fructo	GFPT1	8.25	8.00	8.26
210342_s_at	thyroid peroxidas	TPO	5.26	5.14	5.02
207162_s_at	calcium channel, v	CACNA1B	4.91	5.16	5.01
215282_at	anaphase promot	ANAPC13	3.90	3.91	3.88
217913_at	vacuolar protein s	VPS4A	9.42	9.31	9.39
216806_at	---	---	8.49	8.48	8.43
211903_s_at	myeloproliferative	MPL	5.12	5.02	5.34
215911_x_at	ATPase, Ca++ tran	ATP2B3	4.44	4.34	4.39

206411_s_at	v-abl Abelson mur	ABL2	6.05	6.02	6.07
207518_at	diacylglycerol kina	DGKE	3.89	3.99	3.87
209196_at	WD repeat domai	WDR46	8.71	8.68	8.83
202214_s_at	cullin 4B	CUL4B	9.55	9.71	9.65
214189_s_at	hypothetical prote	LOC100288178	4.30	4.07	4.12
220276_at	RERG/RAS-like	RERGL	3.49	3.26	3.25
220213_at	teashirt zinc finge	TSHZ2	6.47	6.18	6.25
220260_at	TBC1 domain fam	TBC1D19	7.69	7.61	7.79
212305_s_at	melanoma inhibit	MIA3	7.54	7.55	7.73
206573_at	potassium voltage	KCNQ3	5.82	5.78	5.92
206495_s_at	histone H4 transc	HINFP	8.17	8.23	8.41
209140_x_at	major histocompa	HLA-B	12.37	12.24	12.31
221526_x_at	par-3 partitioning	PARD3	9.81	9.63	9.77
209449_at	LSM2 homolog, U	LSM2	9.66	9.66	9.74
205036_at	LSM6 homolog, U	LSM6	10.30	10.45	10.63
205725_at	secretoglobin, fan	SCGB1A1	4.33	4.13	3.95
209253_at	sorbin and SH3 dc	SORBS3	8.39	8.30	8.26
215742_at	---	---	4.09	4.43	4.12
218769_s_at	ankyrin repeat, fa	ANKRA2	9.35	9.48	9.80
206403_at	zinc finger protein	ZNF536	5.53	5.21	5.33
220716_at	guanine nucleotid	GNL3LP1	6.69	6.75	6.44
202680_at	general transcript	GTF2E2	10.17	10.13	10.29
201737_s_at	membrane-associ	6-Mar	10.05	10.10	10.36
219354_at	kelch-like 26 (Dros	KLHL26	7.01	6.94	6.95
209115_at	ubiquitin-like moc	UBA3	9.86	10.04	10.07
216737_at	---	---	3.27	3.29	3.12
206054_at	kininogen 1	KNG1	4.43	4.24	4.20
211916_s_at	myosin IA	MYO1A	6.01	5.90	5.50
203590_at	dynein, cytoplasm	DYNC1LI2	10.19	10.18	10.18
216767_at	---	---	3.50	3.69	3.37
209130_at	synaptosomal-ass	SNAP23	9.49	9.49	9.50
209931_s_at	FK506 binding pro	FKBP1B /// MFSD2	6.12	6.02	6.00
211130_x_at	ectodysplasin A	EDA	6.11	5.95	6.07
203430_at	heme binding pro	HEBP2	10.36	10.40	10.40
215870_s_at	phospholipase A2	PLA2G5	3.52	3.24	3.34
220941_s_at	chromosome 21 c	C21orf91	5.37	5.62	5.45
209720_s_at	serpin peptidase i	SERPINB3	2.93	3.16	3.09
215512_at	membrane-associ	6-Mar	4.12	4.05	4.06
53202_at	chromosome 7 op	C7orf25 /// PSMA2	5.57	5.74	5.76
211663_x_at	prostaglandin D2 :	PTGDS	6.26	6.27	6.19
202470_s_at	cleavage and poly	CPSF6	6.61	6.92	6.84
204126_s_at	cell division cycle	CDC45	9.47	9.38	9.46
201905_s_at	CTD (carboxy-term	CTDSPL	6.92	7.07	6.87
201657_at	ADP-ribosylation f	ARL1	8.44	8.54	8.52
217291_at	carcinoembryonic	CEACAM5	7.08	6.99	6.74
211079_s_at	dual-specificity ty	DYRK1A	5.20	5.31	5.64
215270_at	LFNG O-fucosylpe	LFNG	6.30	5.93	6.10

218032_at	stannin	SNN	6.36	6.62	6.38
202837_at	thioredoxin-like 4.	TXNL4A	7.69	7.63	7.59
210622_x_at	cyclin-dependent	CDK10	6.47	6.42	6.51
217197_x_at	NEDD4 binding pr	N4BP2L1	4.40	4.27	4.47
208251_at	potassium voltage	KCNC4	6.72	6.46	6.38
AFFX-HUMISGF3	signal transducer	STAT1	4.79	4.65	4.74
213830_at	T cell receptor del	TRD@	5.43	5.55	5.16
219831_at	cyclin-dependent	CDKL3	7.18	7.20	7.13
212846_at	ribosomal RNA pr	RRP1B	10.68	10.71	10.90
204661_at	CD52 molecule	CD52	5.09	4.76	4.82
212972_x_at	amyloid beta (A4)	APBB2	7.51	7.51	7.23
204530_s_at	thymocyte selecti	TOX	4.83	4.90	4.86
220740_s_at	solute carrier fam	SLC12A6	4.51	4.70	4.58
202823_at	LIM domain conta	LPP	8.49	8.48	8.85
204471_at	growth associatec	GAP43	3.52	3.35	3.36
218616_at	integrator comple	INTS12	9.76	9.68	9.90
203365_s_at	matrix metallopep	MMP15	6.97	7.14	6.98
210593_at	KIAA0913 /// sper	KIAA0913 /// SAT1	5.12	5.07	4.94
205444_at	ATPase, Ca++ tran	ATP2A1	4.27	4.29	4.43
215330_at	---	---	3.97	4.31	3.97
220138_at	heart and neural c	HAND1	5.95	5.83	5.86
207059_at	paired box 9	PAX9	5.52	5.35	5.66
210346_s_at	CDC-like kinase 4	CLK4	8.88	8.95	9.12
217542_at	Mdm2 p53 bindin	MDM2	6.80	6.69	6.74
203781_at	mitochondrial ribo	MRPL33	12.43	12.40	12.44
217348_x_at	Rho guanine nucle	ARHGEF15	7.59	7.49	7.49
220424_at	nephrosis 2, idiop	NPHS2	5.77	5.69	5.34
209257_s_at	structural mainter	SMC3	10.20	10.25	10.22
208673_s_at	splicing factor, arg	SFRS3	11.00	11.16	11.15
206939_at	deleted in colorec	DCC	3.93	3.59	3.68
200776_s_at	basic leucine zipp	BZW1 /// BZW1L1	10.45	10.64	10.56
212722_s_at	jumonji domain cc	JMJD6	7.16	7.24	7.04
218842_at	RNA polymerase I	RPAP3	8.54	8.71	9.02
221787_at	chromosome 6 op	C6orf120	9.01	8.95	9.05
214253_s_at	dystrobrevin, beta	DTNB	7.14	6.48	6.81
36019_at	serine/threonine I	STK19	8.22	8.13	8.30
208964_s_at	fatty acid desatura	FADS1	10.91	10.82	10.94
217132_at	---	---	3.25	3.15	3.13
217514_at	---	---	6.15	6.04	6.08
207111_at	egf-like module cc	EMR1	6.04	6.43	6.50
205046_at	centromere prote	CENPE	9.67	9.91	9.88
216741_at	---	---	5.49	5.39	5.33
213633_at	SH3-domain bindi	SH3BP1	7.98	7.98	7.72
AFFX-r2-Ec-bioC	---	---	11.36	11.27	11.26
206152_at	ArfGAP with GTPa	AGAP2	7.05	6.69	6.78
216166_at	---	---	3.78	3.76	3.67
214162_at	hypothetical prote	LOC284244	3.92	3.79	3.75

205008_s_at	calcium and integ	CIB2	6.38	6.30	6.27
214489_at	follicle stimulating	FSHB	5.38	5.37	4.98
216528_at	---	---	4.40	4.48	4.56
202632_at	DPH1 homolog (S.	DPH1 /// OVCA2	8.03	8.12	8.15
201219_at	C-terminal binding	CTBP2	9.00	8.90	8.91
220231_at	chromosome 7 op	C7orf16	5.32	5.28	5.05
214472_at	histone cluster 1,	HIST1H2AD /// HIS	4.69	4.48	4.68
205340_at	zinc finger and BT	ZBTB24	9.05	9.19	9.07
205901_at	prepronociceptin	PNOC	5.07	5.24	4.78
216236_s_at	solute carrier fam	SLC2A14 /// SLC2A	7.42	7.43	7.54
217155_at	---	---	4.06	4.17	4.21
207360_s_at	neurotensin recep	NTSR1	6.29	6.25	5.83
222170_at	---	---	4.68	4.75	4.38
209852_x_at	proteasome (pros	PSME3	8.52	8.60	8.61
208060_at	paired box 7	PAX7	6.09	5.96	6.07
217304_at	serine hydroxyme	SHMT1	4.76	4.54	4.58
205789_at	CD1d molecule	CD1D	3.44	3.42	3.46
204496_at	striatin, calmoduli	STRN3	8.88	9.21	9.04
212335_at	glucosamine (N-ac	GNS	8.13	8.15	8.40
214707_x_at	Alstrom syndrome	ALMS1	7.60	7.85	7.81
200872_at	S100 calcium bind	S100A10	13.24	13.23	13.28
202180_s_at	major vault protei	MVP	9.38	9.45	9.43
200961_at	selenophosphate	SEPHS2	10.32	10.30	10.36
218074_at	family with seque	FAM96B	11.51	11.48	11.47
203174_s_at	ADP-ribosylation f	ARFRP1	7.86	7.78	7.82
219045_at	ras homolog gene	RHOF	6.93	6.87	6.80
218102_at	2-deoxyribose-5-p	DERA	10.18	10.13	10.30
216776_at	---	---	5.34	5.29	4.96
222263_at	solute carrier fam	SLC35E1	8.04	7.93	8.04
203580_s_at	solute carrier fam	SLC7A6	9.57	9.61	9.41
202532_s_at	dihydrofolate red	DHFR	9.26	9.34	9.54
218363_at	exonuclease 3'-5'	EXD2	7.86	7.86	7.84
206398_s_at	CD19 molecule	CD19	4.48	4.46	4.24
208509_s_at	olfactory receptor	OR7A17	5.64	5.34	5.39
32209_at	family with seque	FAM89B	8.56	8.56	8.51
200099_s_at	ribosomal protein	RPS3A	14.78	14.80	14.89
217894_at	potassium channe	KCTD3	9.00	9.20	9.58
221259_s_at	testis expressed 1	TEX11	3.93	3.70	3.97
38487_at	stabilin 1	STAB1	6.30	6.47	6.40
221471_at	serine incorporato	SERINC3	11.13	11.08	11.10
222206_s_at	nicalin homolog (z	NCLN	8.53	8.28	8.39
212670_at	elastin	ELN	5.82	6.02	6.01
213121_at	small nuclear ribo	SNRNP70	6.56	6.38	5.94
222260_at	---	---	5.91	6.12	6.03
210137_s_at	dCMP deaminase	DCTD	9.52	9.44	9.60
210539_at	tubulin tyrosine lig	TTLL5	5.89	5.84	5.78
204369_at	phosphoinositide-	PIK3CA	8.62	8.84	8.97

208074_s_at	adaptor-related p	AP2S1	11.35	11.32	11.41
207596_at	solute carrier orga	SLCO4C1	3.66	3.26	3.19
220132_s_at	C-type lectin dom	CLEC2D	3.57	3.54	3.56
44040_at	F-box protein 41	FBXO41	6.41	6.38	6.47
221149_at	G protein-couplec	GPR77	5.10	5.16	5.16
208022_s_at	CDC14 cell divisio	CDC14B	8.49	8.49	8.63
204483_at	enolase 3 (beta, n	ENO3	6.58	6.71	6.60
209764_at	mannosyl (beta-1,	MGAT3	6.48	6.35	6.04
221844_x_at	signal peptidase c	SPCS3	10.10	10.22	10.31
221315_s_at	fibroblast growth	FGF22	5.66	5.55	5.61
212915_at	PDZ domain conta	PDZRN3	3.62	3.33	3.63
220841_s_at	Abelson helper int	AHI1	7.07	7.16	7.34
41856_at	Unc-5 homolog B	UNC5B	9.13	8.70	8.91
206109_at	fucosyltransferase	FUT1	7.28	6.79	7.23
207765_s_at	KIAA1539	KIAA1539	7.04	6.92	6.72
217427_s_at	HIR histone cell cy	HIRA	7.73	7.75	7.81
205297_s_at	CD79b molecule, i	CD79B	5.92	5.47	5.33
215972_at	prostate androger	PART1	4.24	4.21	4.23
208284_x_at	gamma-glutamylt	GGT1	7.11	7.26	6.94
213241_at	plexin C1	PLXNC1	5.30	5.08	5.25
211837_s_at	pre T-cell antigen	PTCRA	6.89	6.76	6.41
209419_at	THO complex 5	THOC5	4.89	4.82	4.87
209434_s_at	phosphoribosyl py	PPAT	9.10	9.25	9.42
206302_s_at	nudix (nucleoside	NUDT4 /// NUDT4	9.84	9.90	9.98
208007_at	---	---	4.30	4.44	4.31
212264_s_at	wings apart-like h	WAPAL	9.66	9.72	9.77
200035_at	dullard homolog (	DULLARD	8.46	8.31	8.33
216205_s_at	mitofusin 2	MFN2	7.55	7.53	7.55
220099_s_at	LUC7-like 2 (S. cer	LUC7L2	10.43	10.41	10.55
211092_s_at	neurofibromin 2 (	NF2	4.91	5.00	5.15
200806_s_at	heat shock 60kDa	HSPD1	12.71	12.71	12.66
215309_at	zinc finger family	ZNF674	3.41	3.24	3.24
204915_s_at	SRY (sex determin	SOX11	4.86	4.96	4.48
213237_at	chromosome 16 c	C16orf88	8.27	8.38	8.33
221355_at	cholinergic recept	CHRNG	6.00	5.99	5.96
217532_x_at	---	---	4.10	4.20	3.82
201265_at	---	---	5.63	5.42	5.38
204313_s_at	cAMP responsive	CREB1	8.96	8.93	9.19
214416_at	---	---	3.43	3.50	3.26
219175_s_at	solute carrier fam	SLC41A3	9.08	8.87	9.07
208975_s_at	karyopherin (impo	KPNB1	10.95	11.00	11.24
56829_at	trafficking protein	TRAPPC9	7.84	7.94	7.77
44702_at	synapse defective	SYDE1	9.58	9.46	9.53
213955_at	myozenin 3	MYOZ3	6.01	6.35	6.24
211962_s_at	zinc finger protein	ZFP36L1	11.66	11.68	11.90
217777_s_at	protein tyrosine p	PTPLAD1	8.54	8.64	8.63
208513_at	forkhead box B1	FOXB1	5.07	5.15	4.90

45828_at	ATP5S-like	ATP5SL	10.04	10.07	10.02
214538_x_at	regulator of G-prc	RGS6	6.65	6.59	6.59
213869_x_at	Thy-1 cell surface	THY1	5.35	4.96	4.95
201587_s_at	interleukin-1 rece	IRAK1	11.87	11.79	11.85
204262_s_at	presenilin 2 (Alzhe	PSEN2	7.65	7.70	7.62
221564_at	protein arginine m	PRMT2	8.35	8.50	8.58
206995_x_at	scavenger recepto	SCARF1	3.30	3.35	3.29
208069_x_at	chorionic somatoi	CSH1 /// CSH2 ///	6.15	6.17	5.95
215869_at	---	---	5.90	5.82	5.52
207200_at	ornithine carbamo	OTC	3.58	3.54	3.54
220200_s_at	SET domain conta	SETD8	7.38	7.13	7.24
209991_x_at	gamma-aminobut	GABBR2	5.84	5.58	5.60
202854_at	RYK receptor-like	RYK	12.02	12.00	12.09
200041_s_at	HLA-B associated	BAT1	9.79	9.85	9.86
218858_at	DEP domain conta	DEPDC6	7.27	7.26	7.31
219068_x_at	ATPase family, AA	ATAD3A	9.08	9.10	8.96
217865_at	ring finger proteir	RNF130	9.14	9.18	9.13
203939_at	5'-nucleotidase, e	NT5E	8.92	9.17	9.26
221449_s_at	integrin alpha FG-	ITFG1	9.88	9.83	9.85
205526_s_at	katanin p60 (ATPa	KATNA1	9.78	9.67	9.88
214677_x_at	cyclosporin A tran	CYAT1 /// IGLV1-4	6.00	6.44	6.06
201742_x_at	splicing factor, arg	SFRS1	9.69	9.73	9.67
219461_at	p21 protein (Cdc4	PAK6	4.75	5.12	4.87
208415_x_at	inhibitor of growt	ING1	9.03	8.96	8.97
217608_at	SFRS12-interactin	SFRS12IP1	8.21	8.50	8.43
217010_s_at	cell division cycle	CDC25C	6.07	5.84	6.15
209100_at	interferon-related	IFRD2	10.84	10.78	10.88
221615_at	bone morphogene	BMP8B	3.98	4.21	4.24
203975_s_at	chromatin assemb	CHAF1A	8.79	8.91	8.93
220443_s_at	ventral anterior h	VAX2	6.07	6.07	6.36
215409_at	lysophosphatidylc	LPCAT4	5.68	5.80	5.49
221005_s_at	phosphatidylserin	PTDSS2	8.22	8.02	8.15
206045_s_at	nucleolar protein	NOL4	3.56	3.60	3.82
205280_at	glycine receptor, b	GLRB	7.24	7.40	7.81
201417_at	SRY (sex determin	SOX4	9.65	9.71	9.81
210796_x_at	sialic acid binding	SIGLEC6	4.38	4.59	4.49
217305_s_at	adenylate cyclase	ADCY10	4.00	4.06	3.98
211378_x_at	peptidylprolyl isor	PPIA	14.42	14.41	14.41
212664_at	tubulin, beta 4	TUBB4	4.40	4.56	4.59
211488_s_at	integrin, beta 8	ITGB8	3.70	3.70	3.71
201309_x_at	chromosome 5 op	C5orf13	8.92	9.17	9.10
213818_x_at	---	---	5.83	6.00	6.10
218670_at	pseudouridylate s	PUS1	8.70	8.60	8.71
202155_s_at	nucleoporin 214kI	NUP214	7.76	7.73	7.86
204970_s_at	v-maf musculoapo	MAFG	8.88	8.82	8.98
208640_at	ras-related C3 bot	RAC1	13.37	13.40	13.40
220945_x_at	MANSC domain co	MANSC1	6.94	7.09	7.01

207801_s_at	ring finger protein	RNF10	10.21	10.18	10.32
213840_s_at	Mitochondrial ribo	MRPS12	6.83	6.89	6.74
215757_at	Protein kinase, DN	PRKDC	4.57	4.88	4.65
218113_at	transmembrane p	TMEM2	7.01	7.27	7.13
204683_at	intercellular adhe	ICAM2	5.81	6.05	5.76
208135_at	HNF1 homeobox I	HNF1B	4.71	4.58	4.30
204131_s_at	forkhead box O3	FOXO3	10.89	10.72	10.86
212142_at	minichromosome	MCM4	7.02	7.16	6.97
221662_s_at	solute carrier fam	SLC22A7	7.20	7.20	7.01
212168_at	RNA binding moti	RBM12	8.70	9.00	8.81
220954_s_at	paired immunoglo	PILRB	7.39	7.56	7.64
210178_x_at	splicing factor, arg	SFRS13A	9.63	9.79	9.79
202165_at	protein phosphata	PPP1R2	8.61	8.72	8.94
220734_s_at	glycolipid transfer	GLTPD1	7.82	7.76	7.87
217868_s_at	methyltransferase	METTL9	10.37	10.44	10.42
202102_s_at	bromodomain con	BRD4	10.59	10.38	10.38
216230_x_at	sphingomyelin ph	SMPD1	5.51	5.52	5.23
201951_at	activated leukocyt	ALCAM	8.51	8.82	8.58
212847_at	Far upstream elem	FUBP1	8.47	8.77	8.86
210846_x_at	tripartite motif-co	TRIM14	6.67	6.59	6.57
212755_at	MON2 homolog (S	MON2	5.43	5.24	5.22
207762_at	lipoprotein, Lp(a)-	LPAL2	4.57	4.41	4.45
220025_at	T-box, brain, 1	TBR1	3.59	3.30	3.27
218493_at	small nuclear ribo	SNRNP25	10.01	10.05	9.92
214699_x_at	WD repeat domai	WIPI2	6.27	6.23	6.09
217479_at	---	---	3.70	3.55	3.64
221257_x_at	F-box protein 38	FBXO38	8.51	8.61	8.77
215695_s_at	glycogenin 2	GYG2	6.27	6.03	5.75
206779_s_at	acetylserotonin O	ASMT	4.39	4.03	4.17
216029_at	---	---	4.78	4.88	4.78
222082_at	zinc finger and BT	ZBTB7A	5.20	5.51	5.42
222092_at	Protein tyrosine p	PTPN21	6.51	6.16	6.05
216300_x_at	retinoic acid recep	RARA	7.66	7.61	7.75
211112_at	solute carrier fam	SLC12A4	7.56	7.54	7.35
205478_at	protein phosphata	PPP1R1A	4.49	4.41	4.73
219291_at	DTW domain cont	DTWD1	8.09	8.01	8.19
214356_s_at	KIAA0368	KIAA0368	10.67	10.55	10.66
218180_s_at	EPS8-like 2	EPS8L2	5.88	5.66	5.44
201244_s_at	v-raf-1 murine leu	RAF1	10.58	10.60	10.57
209289_at	nuclear factor I/B	NFIB	9.70	9.67	9.91
221313_at	G protein-coupled	GPR52	4.96	4.87	4.75
215756_at	hypothetical prote	LOC730227	6.44	6.49	6.39
208396_s_at	phosphodiesteras	PDE1A	3.90	3.95	3.73
212907_at	Solute carrier fam	SLC30A1	11.23	11.17	11.32
212871_at	mitogen-activated	MAPKAPK5	8.52	8.38	8.66
203397_s_at	UDP-N-acetyl-alph	GALNT3	3.27	3.55	3.69
210771_at	peroxisome prolif	PPARA	4.06	3.98	4.04

210695_s_at	WW domain conta	WWOX	6.47	6.63	6.34
210990_s_at	laminin, alpha 4	LAMA4	7.33	7.14	7.13
216030_s_at	semenogelin II	SEMG2	3.78	3.92	3.85
209321_s_at	adenylate cyclase	ADCY3	8.27	8.37	8.47
209272_at	NGFI-A binding pr	NAB1	10.33	10.38	10.49
206809_s_at	heterogeneous nu	HNRNPA3 /// HNR	10.88	10.85	10.87
215425_at	BTG family, memb	BTG3	4.45	4.70	4.59
212307_s_at	O-linked N-acetylg	OGT	8.81	8.88	8.90
202146_at	interferon-related	IFRD1	10.65	10.45	10.73
217580_x_at	---	---	7.28	7.35	7.19
216242_x_at	polymerase (RNA)	POLR2J2	7.30	7.36	7.34
213788_s_at	non-protein codin	NCRNA00094	7.90	7.63	7.77
210524_x_at	---	---	6.73	6.91	6.75
203049_s_at	tetratricopeptide	TTC37	10.09	10.31	10.43
210216_x_at	RAD1 homolog (S.	RAD1	10.63	10.57	10.67
205450_at	phosphorylase kin	PHKA1	8.30	8.42	8.48
212733_at	KIAA0226	KIAA0226	9.11	9.11	9.08
203458_at	sepiapterin reduct	SPR	9.22	9.07	8.96
202088_at	solute carrier fam	SLC39A6	12.03	12.09	12.13
211361_s_at	serpin peptidase i	SERPINB13	3.13	3.29	3.06
221771_s_at	M-phase phospho	MPHOSPH8	7.31	7.62	7.67
212776_s_at	obscurin-like 1	OBSL1	6.44	6.82	6.67
206085_s_at	cystathionase (cys	CTH	9.39	8.87	9.41
211467_s_at	nuclear factor I/B	NFIB	6.30	6.17	6.26
214876_s_at	tubulin, gamma co	TUBGCP5	7.33	7.48	7.50
221911_at	ets variant 1	ETV1	9.22	9.19	9.46
215837_x_at	---	---	3.96	4.09	4.06
201778_s_at	KIAA0494	KIAA0494	9.91	9.93	10.04
210667_s_at	chromosome 21 o	C21orf33	8.11	8.09	7.94
202653_s_at	membrane-associ	7-Mar	9.62	9.91	9.91
200833_s_at	RAP1B, member o	RAP1B	11.99	12.03	12.13
200093_s_at	histidine triad nuc	HINT1	13.14	13.13	13.28
208262_x_at	Mediterranean fe	MEFV	5.73	5.57	5.66
215630_at	---	---	6.43	5.99	6.12
220529_at	hypothetical prote	FLJ11710	6.52	6.43	6.08
218472_s_at	pelota homolog (D	PELO	10.19	10.07	10.15
205148_s_at	chloride channel 4	CLCN4	4.53	4.50	4.53
214333_x_at	isocitrate dehydro	IDH3G	8.58	8.49	8.52
210912_x_at	glutathione S-tran	GSTM4	6.16	6.01	6.05
220135_s_at	solute carrier fam	SLC7A9	4.58	4.65	4.65
200715_x_at	ribosomal protein	RPL13A	13.60	13.54	13.56
209525_at	Hepatoma-derived	HDGFRP3	5.71	5.80	6.12
221384_at	uncoupling protei	UCP1	4.64	4.57	4.25
211353_at	leucine-rich repea	LRIT1	4.20	4.03	3.85
209694_at	6-pyruvoyltetrahy	PTS	9.47	9.63	9.58
202865_at	SP100 nuclear ant	SP100	5.06	4.96	4.94
204945_at	protein tyrosine p	PTPRN	4.56	4.44	4.66

201877_s_at	protein phosphata	PPP2R5C	10.28	10.43	10.49
200842_s_at	glutamyl-prolyl-tR	EPRS	12.38	12.31	12.36
221644_s_at	solute carrier fam	SLC45A2	4.25	4.23	4.03
219885_at	schlafen family m	SLFN12	7.85	7.93	8.28
220146_at	toll-like receptor 7	TLR7	3.95	3.66	3.62
218673_s_at	ATG7 autophagy r	ATG7	7.79	7.70	7.79
204330_s_at	mitochondrial ribo	MRPS12	6.68	6.74	6.66
211636_at	immunoglobulin h	IGH@ /// IGHA1 //	5.00	4.85	4.81
216566_at	---	---	3.61	3.77	3.71
220141_at	chromosome 11 o	C11orf63	4.63	4.39	4.80
202235_at	solute carrier fam	SLC16A1	8.73	8.66	8.86
213516_at	A kinase (PRKA) a	AKAP13	3.66	3.71	3.66
205470_s_at	kallikrein-related	KLK11	5.21	4.97	4.95
208075_s_at	chemokine (C-C m	CCL7	3.94	4.11	4.13
202677_at	RAS p21 protein a	RASA1	10.76	10.75	10.85
209394_at	acetylserotonin O	ASMTL	8.63	8.68	8.53
212705_x_at	similar to patatin-	LOC100293124 ///	7.61	7.42	7.61
216617_s_at	myelin associated	MAG	4.74	4.48	4.49
202224_at	v-crk sarcoma viru	CRK	8.88	8.99	8.84
201098_at	coatomer protein	COPB2	11.33	11.49	11.48
218617_at	tRNA isopentenylt	TRIT1	9.21	9.24	9.39
215649_s_at	mevalonate kinase	MVK	6.80	6.50	6.54
214683_s_at	CDC-like kinase 1	CLK1	9.31	9.33	9.60
202144_s_at	adenylosuccinate	ADSL	12.22	12.15	12.12
204146_at	RAD51 associated	RAD51AP1	10.57	10.57	10.94
213723_s_at	Iduronidase, alpha	IDUA	6.41	5.88	6.27
220195_at	methyl-CpG bindin	MBD5	6.91	7.07	6.87
203414_at	monocyte to mac	MMD	7.85	7.96	7.90
209020_at	chromosome 20 o	C20orf111	9.39	9.17	9.52
203088_at	fibulin 5	FBLN5	7.71	7.80	7.95
200992_at	importin 7	IPO7	11.68	11.68	11.81
202442_at	adaptor-related p	AP3S1	11.74	11.84	11.80
47530_at	chromosome 9 op	C9orf156	7.41	7.32	7.44
209753_s_at	thymopoietin	TMPO	7.37	7.65	7.53
219941_at	transmembrane p	TMEM19	4.18	4.41	4.18
221371_at	tumor necrosis fa	TNFSF18	4.55	4.47	4.31
203129_s_at	kinesin family me	KIF5C	5.62	5.65	5.50
209106_at	nuclear receptor c	NCOA1	7.31	7.25	7.44
65770_at	ras homolog gene	RHOT2	9.22	9.33	9.15
219754_at	RNA binding moti	RBM41	6.94	7.20	7.34
217656_at	SWI/SNF related,	SMARCA4	6.37	6.59	6.32
202084_s_at	SEC14-like 1 (S. ce	SEC14L1	10.87	10.89	11.11
210091_s_at	dystrobrevin, alph	DTNA	6.27	5.98	6.06
221823_at	chromosome 5 op	C5orf30	8.11	8.04	8.17
210484_s_at	hypothetical prote	MGC31957 /// TN	6.43	6.21	6.17
206396_at	solute carrier fam	SLC1A1	5.31	5.14	5.33
219457_s_at	Ras and Rab intera	RIN3	6.47	6.33	6.29

210163_at	chemokine (C-X-C	CXCL11	3.46	3.59	3.04
201251_at	pyruvate kinase, r	PKM2	9.56	9.34	9.46
202898_at	signal-regulatory	SIRPA	8.38	8.41	8.27
220234_at	carbonic anhydras	CA8	4.28	4.38	4.12
35617_at	mitogen-activatec	MAPK7	6.40	6.24	6.44
217272_s_at	serpin peptidase i	SERPINB13	3.70	3.56	3.44
209175_at	SEC23 interacting	SEC23IP	9.32	9.47	9.42
50277_at	golgi-associated, g	GGA1	9.65	9.65	9.53
218720_x_at	seizure related 6 h	SEZ6L2	6.62	6.43	6.61
210363_s_at	sodium channel, v	SCN2B	5.39	5.30	5.57
201167_x_at	Rho GDP dissociat	ARHGDIA	7.25	7.29	7.19
204234_s_at	zinc finger proteir	ZNF195	7.78	8.13	7.91
203929_s_at	microtubule-asso	MAPT	7.07	6.93	7.10
219889_at	frequently rearrar	FRAT1	6.63	6.92	6.79
219226_at	cyclin-dependent	CDK12	8.53	8.58	8.57
213261_at	tetratricopeptide	TRANK1	6.15	6.29	5.92
214767_s_at	heat shock proteir	HSPB6	4.59	4.63	4.64
201843_s_at	EGF-containing fib	EFEMP1	9.56	9.72	9.86
216915_s_at	protein tyrosine p	PTPN12	5.16	5.48	5.12
220758_s_at	roundabout homo	ROBO4	6.57	6.43	6.52
220726_at	---	---	2.88	2.98	3.11
207745_at	calcium binding pr	CABP2	5.53	5.46	5.28
201348_at	glutathione perox	GPX3	4.05	4.00	4.35
217857_s_at	RNA binding moti	RBM8A	7.63	7.71	7.64
203966_s_at	protein phosphata	PPM1A	9.11	9.14	9.21
205863_at	S100 calcium bind	S100A12	4.75	4.70	4.59
222037_at	minichromosome	MCM4	8.71	8.78	8.83
219199_at	AF4/FMR2 family,	AFF4	5.56	5.59	5.42
210769_at	cyclic nucleotide g	CNGB1	6.98	6.80	7.02
218342_s_at	endoplasmic retic	ERMP1	8.34	8.43	8.28
218290_at	pleckstrin homolo	PLEKHJ1	9.71	9.51	9.58
209680_s_at	kinesin family mer	KIFC1	9.14	9.05	9.10
207278_s_at	CD209 molecule	CD209	6.54	6.46	6.31
209254_at	kelch domain con	KLHDC10	7.55	7.53	7.53
208009_s_at	Rho guanine nucle	ARHGEF16	7.12	6.99	6.76
203084_at	transforming grov	TGFB1	6.31	6.48	6.46
219069_at	ankyrin repeat do	ANKRD49	8.36	8.70	8.64
221588_x_at	aldehyde dehydro	ALDH6A1	7.47	7.55	7.63
208479_at	potassium voltage	KCNA1	5.06	5.29	4.94
214690_at	TATA box binding	TAF1B	6.10	6.36	6.31
218689_at	Fanconi anemia, c	FANCF	8.41	8.41	8.47
217022_s_at	immunoglobulin h	IGH@ /// IGHA1 //	4.75	4.91	4.50
217485_x_at	postmeiotic segre	PMS2L1	8.36	8.53	8.34
220802_at	potassium voltage	KCNH4	6.02	5.85	6.08
210659_at	chemokine-like re	CMKLR1	4.85	4.86	4.87
212112_s_at	syntaxin 12	STX12	8.91	9.06	8.89
215622_x_at	PHD finger proteir	PHF7	6.35	6.21	6.25

213692_s_at	Vitamin D (1,25- d	VDR	5.81	5.71	5.62
217393_x_at	ubiquitin-conjuga	UBE2NL	10.50	10.51	10.37
216081_at	laminin, alpha 4	LAMA4	4.37	4.34	4.26
211666_x_at	ribosomal protein	RPL3	14.20	14.13	14.21
217984_at	ribonuclease T2	RNASET2	8.17	8.22	8.24
221294_at	G protein-couplec	GPR21	5.56	5.62	5.51
34260_at	TEL2, telomere m	TELO2	7.95	7.89	7.96
207109_at	POU class 2 home	POU2F3	3.26	3.02	3.04
209969_s_at	signal transducer	STAT1	8.60	8.60	8.70
208825_x_at	ribosomal protein	RPL23A	15.15	15.15	15.18
211576_s_at	solute carrier fam	SLC19A1	9.24	9.04	9.39
221629_x_at	chromosome 8 op	C8orf30A	7.06	6.90	7.00
204475_at	matrix metallopep	MMP1	3.65	3.46	3.38
214852_x_at	vacuolar protein s	VPS13A	4.52	4.80	4.54
201236_s_at	BTG family, meml	BTG2	7.29	7.40	7.08
211145_x_at	interferon, alpha	IFNA21	3.86	3.83	3.67
215202_at	glucuronidase, be	LOC91316	7.79	7.73	7.60
211135_x_at	leukocyte immun	LILRB3	6.71	6.54	6.58
202264_s_at	translocase of out	TOMM40	8.54	8.46	8.63
215456_at	---	---	4.48	4.35	4.27
208855_s_at	serine/threonine	STK24	12.30	12.21	12.32
212016_s_at	polypyrimidine tra	PTBP1	7.18	7.02	7.19
202969_at	dual-specificity ty	DYRK2	8.24	8.25	8.31
222233_s_at	DNA cross-link rep	DCLRE1C	7.97	8.01	8.18
45687_at	proline rich 14	PRR14	9.94	9.88	9.84
208216_at	distal-less homeol	DLX4	5.14	4.71	4.67
215984_s_at	ADP-ribosylation f	ARFRP1	7.52	7.23	7.38
212953_x_at	calreticulin	CALR	10.40	10.38	10.46
201097_s_at	ADP-ribosylation f	ARF4	11.76	11.89	11.81
214191_at	islet cell autoantig	ICA1	6.47	6.36	6.00
202230_s_at	calcium homeosta	CHERP	10.07	10.14	10.31
219592_at	microcephalin 1	MCPH1	6.01	6.10	5.81
212223_at	iduronate 2-sulfat	IDS	8.24	8.22	8.28
215427_s_at	zinc finger, CCHC	ZCCHC14	5.99	5.77	5.47
212346_s_at	MAX dimerization	MXD4	8.18	8.01	8.01
218822_s_at	aminopeptidase-li	NPEPL1	6.44	6.38	6.34
217131_at	---	---	3.61	3.71	3.59
220929_at	UDP-N-acetyl-alpl	GALNT8	5.88	5.80	5.80
219532_at	elongation of very	ELOVL4	5.99	5.69	5.95
201238_s_at	capping protein (a	CAPZA2	11.24	11.27	11.43
206901_at	chromosome 19 c	C19orf57	6.55	6.66	6.62
206991_s_at	chemokine (C-C m	CCR5	6.00	6.08	6.00
210080_x_at	chymotrypsin-like	CELA3A	5.56	5.62	5.31
221452_s_at	transmembrane p	TMEM14B	11.96	12.03	12.10
214148_at	---	---	6.48	6.60	6.49
206874_s_at	STE20-like kinase	SLK	9.37	9.46	9.51
212313_at	CHMP family, mer	CHMP7	8.83	8.92	9.09

222367_at	WAS protein hom	WHAMM /// WHA	5.10	5.14	5.22
213301_x_at	tripartite motif-co	TRIM24	8.74	8.90	9.00
213656_s_at	kinesin light chain	KLC1	10.52	10.34	10.37
218291_at	roadblock domain	ROBLD3	8.65	8.65	8.80
209788_s_at	endoplasmic retic	ERAP1	8.54	8.61	8.65
218710_at	tetratricopeptide	TTC27	9.44	9.36	9.45
202571_s_at	discs, large (Drosc	DLGAP4	7.00	6.84	6.73
220678_at	hypothetical FLJ2(	FLJ20712	5.84	5.61	5.38
213079_at	TSR2, 20S rRNA ac	TSR2	8.01	7.92	7.92
209376_x_at	splicing factor, arg	SFRS2IP	9.74	9.88	9.72
211517_s_at	interleukin 5 rece	IL5RA	4.18	4.14	4.02
217381_s_at	T cell receptor gar	TRGV5	6.51	6.41	6.48
217606_at	---	---	5.23	5.11	5.23
222153_at	myelin expression	MYEF2	4.32	3.71	3.95
202015_x_at	---	---	3.29	3.40	3.33
217113_at	alpha-methylacyl-	AMACR	5.73	5.51	5.49
213280_at	RAP1 GTPase acti	RAP1GAP2	8.63	8.25	8.32
211298_s_at	albumin	ALB	3.44	3.44	3.69
216290_x_at	---	---	4.75	5.06	4.96
208274_at	oculomedin	OCLM	5.52	5.28	5.21
216326_s_at	histone deacetyla	HDAC3	9.26	9.38	9.30
206967_at	cyclin T1	CCNT1	6.48	6.51	6.33
221779_at	MICAL-like 1	MICALL1	9.13	9.03	9.22
217350_at	keratin 19 pseudo	KRT19P2	4.30	4.39	4.03
204835_at	polymerase (DNA	POLA1	8.44	8.65	8.47
221179_at	---	---	4.87	4.66	4.66
203353_s_at	methyl-CpG bindi	MBD1	10.02	9.82	9.91
215587_x_at	---	---	7.99	7.98	8.04
217004_s_at	MCF.2 cell line de	MCF2	4.31	4.33	4.16
201351_s_at	YME1-like 1 (S. ce	YME1L1	11.04	11.18	11.15
205231_s_at	epilepsy, progress	EPM2A	6.10	6.03	5.98
205834_s_at	prostate androger	PART1	4.21	4.19	4.26
220591_s_at	EF-hand domain ((	EFHC2	3.95	4.01	3.85
203526_s_at	adenomatous poly	APC	5.28	5.46	5.47
216417_x_at	homeobox B9	HOXB9	6.77	6.65	6.55
215421_at	hypothetical LOC1	LOC100131510	4.53	4.48	4.32
212177_at	splicing factor, arg	SFRS18	8.28	8.43	8.36
215679_at	---	---	3.95	4.15	4.07
213102_at	ARP3 actin-relate	ACTR3	11.63	11.83	11.82
219282_s_at	transient receptor	TRPV2	5.90	5.82	5.58
211084_x_at	protein kinase D3	PRKD3	7.53	7.76	7.64
213086_s_at	casein kinase 1, al	CSNK1A1	11.43	11.35	11.58
217186_at	zinc finger proteir	ZNF259P1	4.45	4.28	4.01
209066_x_at	ubiquinol-cytochr	UQCRB	12.36	12.27	12.40
202266_at	tyrosyl-DNA phos	TDP2	10.45	10.45	10.42
206036_s_at	v-rel reticuloendo	REL	5.40	5.65	5.64
219346_at	leucine rich repea	LRFN3	7.31	7.18	6.94

216509_x_at	myeloid/lymphoic	MLLT10	5.97	5.85	5.93
212514_x_at	DEAD (Asp-Glu-Ala	DDX3X	8.02	8.28	8.10
201700_at	cyclin D3	CCND3	9.24	9.18	9.08
203110_at	PTK2B protein tyr	PTK2B	7.33	7.30	7.19
204786_s_at	interferon (alpha,	IFNAR2	7.88	7.75	7.99
215353_at	---	---	5.83	5.73	5.48
210953_at	TSC22 domain fan	TSC22D2	4.35	4.35	4.27
218197_s_at	oxidation resistan	OXR1	9.59	9.72	9.77
209508_x_at	CASP8 and FADD-l	CFLAR	7.71	7.76	7.95
218813_s_at	SH3-domain GRB2	SH3GLB2	5.80	6.02	5.96
210219_at	SP100 nuclear ant	SP100	5.35	5.36	5.08
206688_s_at	cleavage and poly	CPSF4	10.13	9.94	9.92
213019_at	RAN binding prote	RANBP6	8.62	8.90	8.90
214716_at	BMP2 inducible ki	BMP2K	4.57	4.67	4.88
218171_at	vacuolar protein s	VPS4B	9.81	9.93	10.05
219713_at	sedoheptulokinas	SHPK	6.16	6.25	6.12
212452_x_at	MYST histone ace	MYST4	7.95	8.14	7.99
203374_s_at	tripeptidyl peptid	TPP2	9.34	9.40	9.33
204396_s_at	G protein-couplec	GRK5	7.21	7.23	7.37
218628_at	coiled-coil domair	CCDC53	9.72	9.79	9.92
211152_s_at	HtrA serine peptic	HTRA2	8.53	8.51	8.67
208160_at	---	---	6.38	6.45	6.14
221030_s_at	Rho GTPase activa	ARHGAP24	5.42	5.22	5.50
202175_at	chondroitin polym	CHPF	6.81	6.75	6.81
220807_at	hemoglobin, theta	HBQ1	6.54	6.17	6.29
211846_s_at	poliovirus recepto	PVRL1	6.39	6.24	6.08
213204_at	cullin 9	CUL9	6.68	6.78	6.65
205351_at	gamma-glutamyl c	GGCX	8.50	8.45	8.49
209028_s_at	abl-interactor 1	ABI1	8.13	8.26	8.46
203487_s_at	armadillo repeat c	ARMC8	8.02	8.21	8.27
206370_at	phosphoinositide-	PIK3CG	3.33	3.39	3.33
214757_at	postmeiotic segre	PMS2L2	5.23	4.93	4.93
219139_s_at	ciliary rootlet coil	CROCCL2	4.19	4.22	4.20
220629_at	KCNQ1 downstrea	KCNQ1DN	5.88	5.71	5.83
218090_s_at	WD repeat domai	WDR11	9.34	9.56	9.71
211159_s_at	protein phosphata	PPP2R5D	7.65	7.78	7.84
212241_at	GRINL1A complex	GCOM1 /// GRINL1	7.78	7.69	7.86
210388_at	phospholipase C, I	PLCB2	5.13	5.07	4.77
202906_s_at	nibrin	NBN	8.31	8.50	8.57
213092_x_at	DnaJ (Hsp40) hom	DNAJC9	9.11	9.21	9.26
218168_s_at	chaperone, ABC1	CABC1	9.36	8.98	9.10
203744_at	high-mobility grou	HMGB3	10.64	10.76	10.62
203104_at	colony stimulating	CSF1R	5.76	5.84	5.78
213736_at	Cytochrome c oxic	COX5B	8.05	7.84	7.98
202431_s_at	v-myc myelocytor	MYC	13.99	13.88	13.97
217090_at	---	---	4.20	4.19	4.07
201262_s_at	biglycan	BGN	6.45	6.37	6.37

210926_at	POTE ankyrin dom	POTEKP	7.05	7.20	6.97
218159_at	DDRGK domain cc	DDRGK1	9.03	8.94	9.01
217099_s_at	gem (nuclear orga	GEMIN4	7.89	7.81	7.61
207132_x_at	prefoldin subunit	PFDN5	12.25	12.14	12.20
207611_at	histone cluster 1,	HIST1H2BL	5.16	5.00	4.94
204517_at	peptidylprolyl isor	PPIC	10.12	10.34	10.31
217856_at	RNA binding moti	RBM8A	8.02	8.18	8.07
212389_at	SET binding factor	SBF1	6.39	6.26	6.48
209641_s_at	ATP-binding casse	ABCC3	7.94	8.03	8.13
213866_at	sterile alpha moti	SAMD14	6.69	6.62	6.41
202475_at	transmembrane p	TMEM147	11.41	11.28	11.37
215937_at	prostaglandin D2	PTGDR	4.35	4.00	4.02
204612_at	protein kinase (cA	PKIA	6.71	7.05	6.75
203289_s_at	nitrogen permeas	NPRL3	6.59	6.62	6.43
215028_at	sema domain, trai	SEMA6A	3.41	3.46	3.45
204852_s_at	protein tyrosine p	PTPN7	5.94	5.82	5.64
220547_s_at	family with seque	FAM35A	11.72	11.75	11.81
202173_s_at	vascular endothel	VEZF1	9.52	9.46	9.74
210801_at	DIM1 dimethylad	DIMT1L	6.18	6.17	6.20
215159_s_at	NAD kinase	NADK	6.57	6.52	6.57
39248_at	aquaporin 3 (Gill b	AQP3	4.53	4.36	4.10
209895_at	protein tyrosine p	PTPN11	7.81	7.84	7.90
201317_s_at	proteasome (pros	PSMA2	12.08	12.11	12.18
211338_at	interferon, alpha 2	IFNA2	3.22	3.33	3.44
201120_s_at	progesterone rece	PGRMC1	9.82	9.84	9.83
219425_at	sulfotransferase fa	SULT4A1	6.15	5.98	6.21
212141_at	minichromosome	MCM4	9.54	9.67	9.59
208676_s_at	proliferation-asso	PA2G4	10.39	10.29	10.32
210436_at	chaperonin contai	CCT8	4.28	4.31	4.33
205038_at	IKAROS family zin	IKZF1	4.36	4.40	4.33
201836_s_at	suppressor of Ty 7	SUPT7L	7.56	7.69	7.61
203471_s_at	pleckstrin	PLEK	4.57	4.41	4.41
208242_at	retina and anterio	RAX	8.01	7.74	7.47
220505_at	chromosome 9 op	C9orf53	5.64	5.45	5.41
219205_at	serine racemase	SRR	6.88	6.86	6.88
204280_at	regulator of G-prc	RGS14	6.18	6.04	5.90
205085_at	origin recognition	ORC1L	6.81	7.01	6.72
217747_s_at	ribosomal protein	RPS9	14.05	14.00	14.07
202174_s_at	pericentriolar mat	PCM1	9.89	10.03	10.20
205195_at	adaptor-related p	AP1S1	8.79	8.80	8.74
208215_x_at	dopamine recepto	DRD4	6.14	6.00	6.00
204364_s_at	receptor accessor	REEP1	3.77	3.61	3.70
218366_x_at	similar to methylt	LOC731602 /// ME	10.55	10.52	10.62
205579_at	histamine recepto	HRH1	8.22	8.27	8.46
219541_at	Lck interacting tra	LIME1	6.99	6.77	6.72
219659_at	ATPase, aminopho	ATP8A2	5.36	5.32	5.15
210119_at	potassium inward	KCNJ15	4.08	3.98	3.78

218246_at	mitochondrial E3	MUL1	8.22	8.22	7.97
201955_at	cyclin C	CCNC	11.53	11.59	11.65
214712_at	RUN domain cont	RUNDC2C	3.63	3.59	3.48
215279_at	---	---	6.54	6.82	6.57
204269_at	pim-2 oncogene	PIM2	6.94	7.10	6.95
209042_s_at	ubiquitin-conjuga	UBE2G2	10.51	10.58	10.60
212245_at	multiple coagulati	MCFD2	10.79	10.81	10.85
211858_x_at	GNAS complex loc	GNAS	11.94	11.91	11.89
35671_at	general transcript	GTF3C1	8.82	8.94	8.83
222138_s_at	WD repeat domai	WDR13	9.23	9.06	9.14
214587_at	collagen, type VIII	COL8A1	7.35	7.31	7.57
216777_at	---	---	4.22	3.79	3.68
209017_s_at	lon peptidase 1, n	LONP1	9.98	9.86	9.96
207049_at	sodium channel, v	SCN8A	5.88	5.87	5.64
220634_at	T-box 4	TBX4	6.09	5.99	5.88
219183_s_at	cytohesin 4	CYTH4	6.58	6.61	6.54
220793_at	sarcoma antigen 1	SAGE1	3.52	3.51	3.42
214789_x_at	splicing factor, arg	SFRS2B	10.73	10.71	10.77
215928_at	---	---	3.79	3.88	3.85
209004_s_at	F-box and leucine-	FBXL5	9.54	9.65	9.78
218495_at	ubiquitously-expr	UXT	11.22	11.17	11.27
211713_x_at	KIAA0101	KIAA0101	9.11	9.21	9.22
207632_at	muscle, skeletal, r	MUSK	4.61	4.63	4.62
209213_at	carbonyl reductas	CBR1	9.86	9.94	9.80
221855_at	succinate dehydro	SDHAF1	8.51	8.60	8.54
214985_at	exostosin 1	EXT1	5.96	6.14	6.31
218408_at	translocase of inn	TIMM10	10.45	10.33	10.28
218535_s_at	RIO kinase 2 (yeas	RIOK2	9.52	9.55	9.57
208926_at	sialidase 1 (lysoso	NEU1	7.76	7.86	7.69
201336_at	vesicle-associated	VAMP3	11.58	11.66	11.54
206801_at	natriuretic peptid	NPPB	5.91	5.53	5.82
216100_s_at	torsin A interactin	TOR1AIP1	6.76	6.89	6.98
220458_at	---	---	5.73	5.79	5.54
219342_at	CAS1 domain cont	CASD1	7.59	7.91	8.12
210183_x_at	pinin, desmosome	PNN	12.47	12.49	12.46
210385_s_at	endoplasmic retic	ERAP1	5.65	5.57	5.80
35148_at	tight junction prot	TJP3	6.08	5.85	5.66
220755_s_at	chromosome 6 op	C6orf48	12.67	12.43	12.64
220105_at	rhabdoid tumor d	RTDR1	4.69	4.80	4.63
204091_at	phosphodiesteras	PDE6D	9.08	9.26	9.35
214784_x_at	exportin 6	XPO6	9.82	9.84	9.84
209993_at	ATP-binding casse	ABCB1	4.24	4.48	4.26
203164_at	solute carrier fam	SLC33A1	8.74	8.92	9.02
214057_at	---	---	7.44	7.51	7.41
207917_at	---	---	6.21	6.06	6.07
218339_at	mitochondrial ribo	MRPL22	10.45	10.43	10.55
214639_s_at	homeobox A1	HOXA1	6.43	6.61	6.64

203829_at	elongation protei	ELP4	8.83	8.81	8.78
53968_at	integrator comple	INTS5	8.74	8.76	8.72
204969_s_at	radixin	RDX	6.21	6.34	6.30
204192_at	CD37 molecule	CD37	3.85	3.78	3.75
44673_at	sialic acid binding	SIGLEC1	6.51	6.55	6.28
220620_at	cysteine-rich C-te	CRCT1	4.15	3.84	3.65
221768_at	Splicing factor pro	SFPQ	8.33	8.75	8.63
216093_at	---	---	4.27	4.11	4.16
207910_at	secretoglobin, fan	SCGB1D1	3.47	3.50	3.55
220013_at	epoxide hydrolase	EPHX3	5.18	5.34	5.27
213893_x_at	postmeiotic segre	PMS2L5	8.43	8.44	8.21
202353_s_at	proteasome (pros	PSMD12	9.98	10.15	10.12
206711_at	chromosome X op	CXorf1	4.09	4.01	4.00
211160_x_at	actinin, alpha 1	ACTN1	10.18	10.17	10.16
203223_at	rabaptin, RAB GTF	RABEP1	6.57	6.53	6.51
211703_s_at	TM2 domain cont	TM2D1	9.24	9.27	9.34
218177_at	chromatin modify	CHMP1B	7.42	7.14	7.42
201099_at	ubiquitin specific	USP9X	9.72	9.75	9.76
213575_at	transformer 2 alpl	TRA2A	6.77	6.86	6.53
212552_at	hippocalcin-like 1	HPCAL1	11.74	11.68	11.71
206159_at	growth differentia	GDF10	4.45	4.31	4.33
217447_at	myelin associated	MAG	6.27	6.12	6.07
217596_at	UPF3 regulator of	UPF3A	6.37	6.57	6.22
201760_s_at	WD repeat and SC	WSB2	11.43	11.55	11.55
219312_s_at	zinc finger and BT	ZBTB10	7.32	7.29	7.69
212066_s_at	ubiquitin specific	USP34	9.77	9.84	9.89
214735_at	interaction protei	IPCEF1	4.34	4.09	4.24
207504_at	carbonic anhydras	CA7	6.92	6.81	6.87
217888_s_at	ADP-ribosylation f	ARFGAP1	7.95	7.94	7.82
213765_at	microfibrillar asso	MFAP5	3.95	3.80	3.81
209512_at	hydroxysteroid de	HSDL2	7.59	7.89	8.01
207509_s_at	leukocyte-associa	LAIR2	4.22	4.38	4.13
209190_s_at	diaphanous homo	DIAPH1	10.31	10.27	10.21
221234_s_at	BTB and CNC hom	BACH2	3.61	3.67	3.72
219712_s_at	sedoheptulokinas	SHPK /// TRPV1	5.12	4.99	4.76
211928_at	dynein, cytoplasm	DYNC1H1	10.56	10.46	10.64
215994_x_at	TBC1 domain fam	TBC1D9B	8.20	8.19	8.10
206750_at	v-maf musculoapo	MAFK	5.86	5.71	5.50
213295_at	cylindromatosis (t	CYLD	7.82	7.76	7.86
214254_at	melanoma antigen	MAGEA4	4.12	4.03	3.73
209214_s_at	Ewing sarcoma br	EWSR1	10.22	10.25	10.28
200651_at	guanine nucleotid	GNB2L1	14.30	14.20	14.33
204701_s_at	stomatin (EPB72)-	STOML1	8.48	8.41	8.36
214918_at	heterogeneous nu	HNRNPM	4.79	4.74	4.74
219629_at	family with seque	FAM118A	6.01	5.60	5.80
214935_at	interleukin 4 indu	IL4I1 /// NUP62 //	6.13	5.84	5.96
220101_x_at	chromosome 17 c	C17orf59	5.62	5.64	5.51

218318_s_at	nemo-like kinase	NLK	7.64	7.93	7.73
218598_at	RAD50 interactor	RINT1	8.10	8.37	8.51
210950_s_at	farnesyl-diphosph	FDFT1	11.14	11.31	11.27
217315_s_at	kallikrein-related	KLK13	5.46	5.32	5.17
208475_at	FERM domain con	FRMD4A	6.58	6.45	6.45
214911_s_at	bromodomain cor	BRD2	10.44	10.34	10.43
206810_at	zinc finger protein	ZNF44	3.42	3.59	3.07
221859_at	synaptotagmin XII	SYT13	4.18	4.23	4.23
220043_s_at	antigen p97 (mela	MFI2	3.92	3.78	3.55
205601_s_at	homeobox B5	HOXB5	7.00	7.13	6.75
218374_s_at	chromosome 12 c	C12orf4	7.72	7.71	7.90
219506_at	chromosome 1 op	C1orf54	5.47	5.21	5.46
212483_at	Nipped-B homolog	NIPBL	8.87	8.99	9.02
203208_s_at	mitochondrial fiss	MTFR1	10.84	10.84	11.07
212139_at	GCN1 general con	GCN1L1	10.09	9.98	10.04
208826_x_at	histidine triad nuc	HINT1	13.51	13.51	13.67
213401_s_at	---	---	6.18	6.28	6.43
205686_s_at	CD86 molecule	CD86	4.46	4.40	4.32
220994_s_at	syntaxin binding p	STXBP6	4.23	3.97	3.95
221586_s_at	E2F transcription	E2F5	8.39	8.26	8.40
222211_x_at	SCAN domain con	SCAND2	5.90	5.64	5.80
61297_at	CASK interacting p	CASKIN2	6.28	6.35	6.40
204555_s_at	protein phosphata	PPP1R3D	5.75	5.52	5.54
205753_at	C-reactive protein	CRP	5.19	4.72	4.91
221072_at	chromosome 9 op	C9orf31	5.91	6.09	5.98
209580_s_at	methyl-CpG bindi	MBD4	8.58	8.65	8.81
209633_at	protein phosphata	PPP2R3A	8.43	8.59	8.59
216894_x_at	cyclin-dependent	CDKN1C	6.73	6.77	6.43
201028_s_at	CD99 molecule	CD99	9.28	9.24	9.45
219192_at	ubiquitin associat	UBAP2	10.08	10.04	10.02
206864_s_at	harakiri, BCL2 inte	HRK	6.52	6.24	6.40
203878_s_at	matrix metallopep	MMP11	7.24	7.12	6.67
212080_at	Myeloid/lymphoid	MLL	7.79	7.83	7.65
216729_at	Similar to Kruppel	LOC100133321	6.39	6.25	6.21
214744_s_at	ribosomal protein	RPL23	7.37	7.57	7.50
221155_x_at	---	---	6.43	6.56	6.41
212939_at	collagen, type VI,	COL6A1	4.59	4.69	4.71
220430_at	glycine/arginine ri	GRRP1	4.49	4.44	4.47
208266_at	chromosome 8 op	C8orf17	5.59	5.51	5.21
222320_at	---	---	3.47	3.38	3.57
64883_at	motile sperm dom	MOSPD2	7.11	7.15	7.26
212344_at	sulfatase 1	SULF1	3.95	4.05	3.92
202404_s_at	collagen, type I, al	COL1A2	3.95	4.13	4.04
214225_at	protein (peptidylp	PIN4	4.87	4.60	4.65
202971_s_at	dual-specificity ty	DYRK2	8.27	8.32	8.56
207758_at	---	---	6.30	6.24	6.56
212800_at	syntaxin 6	STX6	6.73	6.89	6.93

214060_at	single-stranded DI	SSBP1	7.16	7.48	7.34
211142_x_at	major histocompa	HLA-DOA	6.62	6.58	6.57
215012_at	zinc finger protein	ZNF451	6.94	7.00	7.03
200795_at	SPARC-like 1 (hevi	SPARCL1	3.55	3.66	3.63
208281_x_at	deleted in azoosp	DAZ1 /// DAZ2 ///	4.54	4.17	4.20
209516_at	SMYD family mem	SMYD5	7.06	7.09	7.11
215339_at	natural killer-tum	NKTR	3.44	3.50	3.62
212406_s_at	protein-L-isoaspa	PCMTD2	9.22	9.26	9.27
206239_s_at	serine peptidase i	SPINK1	3.59	3.81	3.80
217443_at	---	---	3.28	3.37	3.13
217181_at	---	---	3.93	3.90	3.89
203697_at	frizzled-related pr	FRZB	3.90	3.57	3.65
220704_at	IKAROS family zin	IKZF1	3.84	3.73	3.58
206288_at	protein geranylge	PGGT1B	6.72	6.77	6.90
202209_at	LSM3 homolog, U	LSM3	11.44	11.46	11.42
203307_at	guanine nucleotid	GNL1	7.78	7.77	7.75
209786_at	high mobility grou	HMGN4	10.05	10.10	10.31
218108_at	ubiquitin protein l	UBR7	9.76	9.81	9.94
204983_s_at	glypican 4	GPC4	5.24	5.05	5.11
209772_s_at	CD24 molecule	CD24	4.44	4.48	4.60
209700_x_at	phosphodiesteras	PDE4DIP	3.28	3.19	3.44
212256_at	UDP-N-acetyl-alpl	GALNT10	8.96	8.91	9.03
207644_at	forkhead box H1	FOXH1	5.87	6.07	5.76
205610_at	myomesin 1, 185k	MYOM1	5.47	5.27	5.39
222257_s_at	angiotensin I conv	ACE2	3.86	4.12	4.11
205996_s_at	adenylate kinase 2	AK2	8.01	8.21	8.14
206099_at	protein kinase C, e	PRKCH	7.72	7.72	7.67
220825_s_at	kin of IRRE like (D	KIRREL	6.46	6.28	6.49
214614_at	motor neuron and	MNX1	6.69	6.56	6.30
216598_s_at	chemokine (C-C m	CCL2	4.96	4.74	4.74
202139_at	aldo-keto reducta	AKR7A2	9.93	9.77	9.76
203695_s_at	deafness, autosor	DFNA5	10.63	10.54	10.80
207402_at	zinc finger protein	ZNF132	3.65	3.53	3.36
210965_x_at	cyclin-dependent	CDK13	4.85	5.07	4.81
201312_s_at	SH3 domain bindi	SH3BGRL	9.98	10.10	10.31
201532_at	proteasome (pros	PSMA3	12.35	12.40	12.39
214097_at	ribosomal protein	RPS21	10.32	10.34	10.30
206689_x_at	K(lysine) acetyltra	KAT5	8.17	8.08	8.28
201755_at	minichromosome	MCM5	7.42	7.47	7.44
205197_s_at	ATPase, Cu++ trar	ATP7A	7.23	7.37	7.42
221227_x_at	coenzyme Q3 hon	COQ3	9.39	9.40	9.15
201643_x_at	lysine (K)-specific	KDM3B	9.79	9.82	9.81
203327_at	insulin-degrading	IDE	9.34	9.35	9.39
208162_s_at	---	---	4.30	3.95	4.19
203796_s_at	B-cell CLL/lympho	BCL7A	6.40	6.31	6.06
203158_s_at	glutaminase	GLS	7.01	6.82	7.06
217876_at	general transcript	GTF3C5	6.78	6.84	6.80

206612_at	calcium channel, v	CACNG1	6.65	6.74	6.91
205860_x_at	folate hydrolase (p	FOLH1	3.67	3.76	3.89
220743_at	---	---	7.56	7.53	7.26
206552_s_at	tachykinin, precur	TAC1	3.23	3.16	2.98
209171_at	inosine triphospha	ITPA	10.83	10.71	10.70
220548_at	polycystic kidney (	PKDREJ	5.91	5.92	5.89
215426_at	zinc finger, CCHC (	ZCCHC14	5.37	5.46	5.32
40446_at	PHD finger protein	PHF1	8.96	8.93	9.00
219921_s_at	dedicator of cytok	DOCK5	5.58	5.67	5.41
208732_at	RAB2A, member R	RAB2A	9.00	9.04	9.08
44790_s_at	chromosome 13 o	C13orf18	3.20	3.30	3.15
213921_at	somatostatin	SST	4.52	4.34	4.40
212196_at	interleukin 6 signa	IL6ST	9.03	9.12	9.27
211594_s_at	mitochondrial ribo	MRPL9	9.57	9.54	9.63
204922_at	chromosome 11 o	C11orf80	7.07	7.02	7.26
208663_s_at	tetratricopeptide	TTC3	10.23	10.37	10.32
206553_at	2'-5'-oligoadenyla	OAS2	4.26	3.87	4.35
212737_at	GM2 ganglioside a	GM2A	8.36	8.47	8.40
219811_at	DiGeorge syndrom	DGCR8	7.71	7.83	7.41
210675_s_at	protein tyrosine p	PTPRR	4.60	4.44	4.77
203536_s_at	cytosolic iron-sulf	CIAO1	9.26	9.23	9.19
200794_x_at	DAZ associated pr	DAZAP2	12.09	12.13	12.08
220543_at	chromosome 21 o	C21orf62	4.48	4.46	4.35
220417_s_at	THAP domain con	THAP4	8.59	8.44	8.63
205385_at	---	---	5.95	5.83	5.74
217329_x_at	---	---	10.00	10.16	10.26
216070_at	---	---	3.84	3.71	3.55
201681_s_at	discs, large homol	DLG5	11.76	11.73	11.74
222221_x_at	EH-domain contai	EHD1	7.68	7.77	7.80
210672_s_at	nitrogen permeas	NPRL3	8.08	8.20	8.37
215225_s_at	G protein-coupled	GPR17	4.56	4.28	4.33
53720_at	chromosome 19 o	C19orf66	7.00	7.17	7.22
218402_s_at	Hermansky-Pudla	HPS4	8.14	7.97	8.19
203331_s_at	inositol polyphosp	INPP5D	4.57	4.41	4.53
209618_at	catenin (cadherin-	CTNND2	3.29	3.43	3.03
206684_s_at	activating transcri	ATF7	4.85	4.78	4.76
212311_at	sel-1 suppressor o	SEL1L3	6.83	6.77	6.78
220742_s_at	N-glycanase 1	NGLY1	9.99	10.01	10.01
207759_s_at	disrupted in schizo	DISC1 /// TSNAX /,	3.82	3.90	3.75
213595_s_at	CDC42 binding pro	CDC42BPA	7.94	7.81	7.88
220788_s_at	ring finger protein	RNF31	7.03	7.01	7.02
219540_at	zinc finger protein	ZNF267	6.37	6.76	7.10
220719_at	---	---	4.96	4.91	5.05
218478_s_at	zinc finger, CCHC (	ZCCHC8	10.48	10.32	10.56
210919_at	---	---	3.70	3.30	3.34
40560_at	T-box 2	TBX2	6.70	6.62	6.32
200889_s_at	signal sequence re	SSR1	9.36	9.37	9.47

207598_x_at	X-ray repair comp	XRCC2	6.97	7.05	6.84
203745_at	holocytochrome c	HCCS	7.98	8.14	8.18
210621_s_at	RAS p21 protein a	RASA1	8.98	9.01	9.15
208469_s_at	EGF-like-domain,	EGFL8 /// PPT2	6.35	6.18	6.35
203318_s_at	zinc finger protein	ZNF148	9.39	9.39	9.53
204541_at	SEC14-like 2 (S. ce	SEC14L2	6.81	6.88	6.74
204789_at	formin-like 1	FMNL1	7.42	7.24	7.08
202844_s_at	DnaJ (Hsp40) hom	DNAJB9	9.77	9.79	9.92
202967_at	glutathione S-tran	GSTA4	10.07	10.18	10.04
220497_at	zinc finger protein	ZNF214	3.51	3.64	3.70
201403_s_at	microsomal glutat	MGST3	11.63	11.67	11.76
208517_x_at	basic transcription	BTF3	14.17	14.16	14.18
218242_s_at	suppressor of vari	SUV420H1	7.97	8.04	8.07
212631_at	syntaxin 7	STX7	8.21	8.15	8.20
210573_s_at	polymerase (RNA)	POLR3C	8.07	7.82	8.16
211427_s_at	potassium inward	KCNJ13	3.11	3.15	3.21
208667_s_at	suppression of tur	ST13	10.70	10.79	10.80
208425_s_at	tetratricopeptide	TANC2	6.90	6.71	6.96
218748_s_at	exocyst complex c	EXOC5	5.95	6.29	6.33
213069_at	HEG homolog 1 (z	HEG1	9.90	9.83	9.88
220517_at	vacuolar protein s	VPS13C	3.61	3.95	3.55
206233_at	UDP-Gal:betaGlcN	B4GALT6	6.43	6.39	6.61
218982_s_at	mitochondrial ribo	MRPS17 /// ZNF71	10.36	10.36	10.34
219286_s_at	RNA binding moti	RBM15	10.12	9.86	10.13
216103_at	acyl-CoA thioester	ACOT11	4.84	4.77	4.81
206363_at	v-maf musculoapo	MAF	3.94	4.07	4.26
221576_at	hypothetical prote	LOC100287132	5.25	5.14	4.97
220965_s_at	radial spoke head	RSPH6A	5.77	5.74	5.68
222157_s_at	WD repeat domai	WDR48	7.06	7.16	7.25
216762_at	KN motif and anky	KANK1	5.52	5.51	5.51
217317_s_at	hect domain and I	HERC2P2 /// HERC	9.32	9.49	9.27
216105_x_at	protein phosphata	PPP2R4	7.18	7.03	7.29
216889_s_at	hepatocyte nuclea	HNF4A	6.61	6.49	6.57
219948_x_at	UDP glucuronosyl	UGT2A3	3.27	3.26	3.07
214126_at	---	---	8.13	8.02	8.16
221640_s_at	leucine-rich repea	LRDD	6.83	6.74	6.70
204796_at	echinoderm micro	EML1	6.05	6.06	6.16
221243_s_at	---	---	4.16	4.01	4.33
212982_at	zinc finger, DHHC-	ZDHHC17	8.40	8.53	8.53
217550_at	Activating transcri	ATF6	8.08	8.04	8.27
215585_at	KIAA0174	KIAA0174	3.67	3.91	3.51
210383_at	sodium channel, v	SCN1A	3.18	3.16	3.12
206791_s_at	phosphodiesteras	PDE4C	6.90	7.10	6.80
218553_s_at	potassium channe	KCTD15	5.26	5.31	5.07
212220_at	proteasome (pros	PSME4	7.20	7.43	7.26
208863_s_at	splicing factor, arg	SFRS1	11.20	11.24	11.35
214042_s_at	ribosomal protein	RPL22	11.96	11.95	12.00

221322_at	neuropeptide VF p	NPVF	3.61	3.63	3.46
205911_at	parathyroid horm	PTH1R	6.19	6.28	6.00
209252_at	histidyl-tRNA synt	HARS2	9.70	9.82	9.71
215927_at	ADP-ribosylation f	ARFGEF2	5.65	5.74	5.40
210504_at	Kruppel-like facto	KLF1	4.40	4.49	4.49
212947_at	solute carrier fam	SLC9A8	7.17	7.12	7.18
211103_at	myosin VIIA	MYO7A	4.28	4.60	4.31
216547_at	---	---	7.99	7.87	8.17
209860_s_at	annexin A7	ANXA7	11.49	11.42	11.51
222218_s_at	paired immunoglo	PILRA	5.04	5.18	5.18
55872_at	zinc finger protein	ZNF512B	7.71	7.69	7.69
206442_at	semenogelin I	SEMG1	3.34	3.18	3.26
221489_s_at	sprouty homolog 4	SPRY4	8.49	8.38	8.56
205292_s_at	heterogeneous nu	HNRNPA2B1	13.23	13.23	13.24
201797_s_at	valyl-tRNA synthe	VARS	9.40	9.20	9.27
215305_at	platelet-derived g	PDGFRA	4.87	5.10	4.86
204554_at	protein phosphata	PPP1R3D	5.68	5.86	6.14
206879_s_at	neuregulin 2	NRG2	5.23	5.30	4.89
216672_s_at	myelin transcripti	MYT1L	3.30	3.12	3.20
214227_at	---	---	4.81	4.92	4.84
202389_s_at	huntingtin	HTT	8.23	8.11	8.12
209014_at	melanoma antigen	MAGED1	9.92	9.99	9.88
217319_x_at	cytochrome P450,	CYP4A22	3.27	3.22	3.26
202333_s_at	ubiquitin-conjugat	UBE2B	10.93	10.94	11.21
208180_s_at	histone cluster 1,	HIST1H4H	5.33	5.49	5.47
220154_at	dystonin	DST	5.00	5.00	4.51
212956_at	TBC1 domain fam	TBC1D9	9.88	9.98	10.11
206603_at	solute carrier fam	SLC2A4	4.48	4.46	4.47
206193_s_at	corneodesmosin	CDSN	4.99	5.13	5.02
206742_at	c-fos induced grov	FIGF	3.91	4.18	3.92
209650_s_at	TBC1 domain fam	TBC1D22A	8.17	8.20	7.98
209215_at	major facilitator s	MFSD10	8.75	8.76	8.60
219546_at	BMP2 inducible ki	BMP2K	8.10	7.76	8.08
216668_at	---	---	6.44	6.24	6.05
222033_s_at	fms-related tyrosi	FLT1	5.43	5.23	5.19
210358_x_at	GATA binding pro	GATA2	7.23	6.79	7.07
216145_at	---	---	5.86	5.79	5.53
212297_at	ATPase type 13A3	ATP13A3	8.91	8.95	9.17
210142_x_at	flotillin 1	FLOT1	9.64	9.56	9.52
50374_at	chromosome 17 o	C17orf90	10.07	9.84	9.95
204453_at	zinc finger protein	ZNF84	7.65	7.77	7.89
36553_at	acetylserotonin O	ASMTL	10.30	10.34	10.24
215759_at	ankyrin repeat do	ANKRD53	5.02	4.90	4.99
212520_s_at	SWI/SNF related,	SMARCA4	9.76	9.62	9.73
215835_at	hypothetical prote	LOC100288270 ///	3.59	3.53	3.67
207307_at	5-hydroxytryptam	HTR2C	5.40	5.24	5.62
209161_at	PRP4 pre-mRNA p	PRPF4	10.18	10.19	10.19

213433_at	ADP-ribosylation f	ARL3	5.90	5.99	6.04
219554_at	Rh family, C glycop	RHCG	6.85	6.43	6.59
205761_s_at	dihydrouridine syn	DUS4L	8.67	8.70	8.75
214597_at	somatostatin rece	SSTR2	3.99	4.06	3.96
203211_s_at	myotubularin rela	MTMR2	10.36	10.25	10.48
201997_s_at	spen homolog, tra	SPEN	9.61	9.56	9.63
213544_at	Inhibitor of growt	ING2	3.98	4.20	4.18
215175_at	pecanex homolog	PCNX	5.12	5.33	5.06
209414_at	fizzy/cell division	FZR1	6.97	6.82	6.71
218603_at	headcase homolo	HECA	8.82	8.82	9.00
210887_s_at	Ellis van Creveld s	EVC	7.21	7.21	7.19
215335_at	---	---	5.49	5.06	5.53
217923_at	penta-EF-hand do	PEF1	9.74	9.59	9.69
216756_at	---	---	5.00	5.12	5.00
212674_s_at	DEAH (Asp-Glu-Ala	DHX30	9.58	9.55	9.58
206598_at	insulin	INS	5.02	5.03	5.09
210403_s_at	potassium inward	KCNJ1	4.36	4.35	4.17
221713_s_at	MAP6 domain cor	MAP6D1	5.93	6.01	5.58
210469_at	discs, large homol	DLG5	7.96	8.18	7.91
207777_s_at	SP140 nuclear bod	SP140	4.12	4.05	4.03
215396_at	G protein-couplec	GPR98	3.30	3.22	3.44
211782_at	iduronate 2-sulfat	IDS	4.28	3.94	3.97
201726_at	ELAV (embryonic l	ELAVL1	12.21	12.25	12.24
218731_s_at	von Willebrand fa	VWA1	5.88	5.98	5.84
209546_s_at	apolipoprotein L,	APOL1	7.34	7.22	7.18
215104_at	nuclear receptor i	NRIP2	5.50	5.39	5.36
202832_at	glutathione perox	GPX2	8.00	8.36	8.25
204522_at	dom-3 homolog Z	DOM3Z	7.96	7.51	7.66
202962_at	kinesin family mer	KIF13B	6.92	6.80	6.84
208295_x_at	chorionic somatom	CSHL1	3.95	4.19	4.01
211619_s_at	alkaline phosphat	ALPP /// ALPPL2	6.22	6.36	6.15
212115_at	hematological and	HN1L	11.64	11.56	11.64
202147_s_at	interferon-related	IFRD1	10.23	10.08	10.40
211732_x_at	histamine N-meth	HNMT	4.11	4.58	4.32
217702_at	interleukin 27 rec	IL27RA	5.69	5.37	5.17
205564_at	P antigen family, r	PAGE4	4.68	4.81	4.66
209172_s_at	centromere prote	CENPF	8.89	9.16	9.42
200996_at	ARP3 actin-relatec	ACTR3	11.77	11.88	12.05
202843_at	DnaJ (Hsp40) hom	DNAJB9	6.75	6.72	7.24
218520_at	TANK-binding kina	TBK1	9.06	9.20	9.24
218394_at	rogdi homolog (Dr	ROGDI	6.04	5.80	5.89
204865_at	carbonic anhydras	CA3	3.83	3.75	3.83
218312_s_at	zinc finger and SCA	ZSCAN18	4.28	4.18	4.13
219432_at	Ellis van Creveld s	EVC	7.37	7.45	7.28
204921_at	growth arrest-spe	GAS8	8.30	8.26	7.94
212745_s_at	Bardet-Biedl synd	BBS4	7.92	8.06	7.88
211106_at	---	---	5.08	5.12	5.34

203099_s_at	chromodomain pr	CDYL	7.99	7.70	7.81
206916_x_at	tyrosine aminotra	TAT	6.47	6.33	6.28
210491_at	---	---	4.51	4.40	4.25
201366_at	annexin A7	ANXA7	10.93	10.94	11.31
220474_at	solute carrier fam	SLC25A21	4.59	4.54	4.79
204790_at	SMAD family men	SMAD7	6.76	6.63	6.85
220895_at	ubiquitin specific	USP29	4.73	4.49	4.53
202531_at	interferon regulat	IRF1	8.02	7.77	7.98
207563_s_at	O-linked N-acetylg	OGT	8.36	8.39	8.41
213099_at	angel homolog 1 (	ANGEL1	8.72	8.57	8.79
219494_at	RAD54 homolog B	RAD54B	8.30	8.45	8.45
204740_at	connector enhanc	CNKSR1	6.53	6.62	6.43
58367_s_at	zinc finger proteir	ZNF419	8.40	8.43	8.39
202926_at	neuroblastoma ar	NBAS	8.82	8.64	8.77
212405_s_at	methyltransferase	METTL13	8.94	8.82	8.89
219875_s_at	PPPDE peptidase	PPPDE1	4.47	4.20	4.22
213751_at	leucine rich repea	LRRC68	5.63	5.74	5.70
217491_x_at	cytochrome c oxic	COX7C	13.35	13.39	13.42
211605_s_at	retinoic acid recep	RARA	7.25	6.99	7.01
201360_at	cystatin C	CST3	9.62	9.51	9.47
200918_s_at	signal recognition	SRPR	8.78	8.77	8.65
33132_at	cleavage and poly	CPSF1	7.83	7.83	7.85
201356_at	splicing factor 3a,	SF3A1	9.82	9.80	9.70
208944_at	transforming grov	TGFBR2	11.87	11.81	11.96
204725_s_at	NCK adaptor prot	NCK1	9.76	9.70	9.81
219641_at	de-etiolated hom	DET1	8.22	8.15	8.21
216760_at	HRAS-like suppres	HRASLS2	5.24	5.68	5.45
212165_at	transmembrane p	TMEM183A /// TM	9.45	9.37	9.46
214059_at	Interferon-induce	IFI44	4.53	4.67	4.33
219575_s_at	component of olig	COG8 /// PDF	9.57	9.52	9.67
204141_at	tubulin, beta 2A	TUBB2A	11.14	11.39	11.31
220803_at	STAM binding pro	STAMBPL1	3.97	3.91	3.82
211345_x_at	eukaryotic transla	EEF1G /// TUT1	14.50	14.40	14.41
212787_at	YLP motif contain	YLPM1	9.18	9.42	9.34
210087_s_at	myelin protein zer	MPZL1	8.04	7.94	8.03
215432_at	acyl-CoA syntheta	ACSM1	4.99	4.68	4.49
218061_at	male-enhanced ar	MEA1	9.91	10.04	10.05
219278_at	mitogen-activated	MAP3K6	7.81	7.91	7.85
219902_at	betaine--homocys	BHMT2	3.81	3.57	3.37
215321_at	RUN domain cont	RUNDC3B	5.21	5.44	5.65
203351_s_at	origin recognition	ORC4L	10.70	10.54	10.66
220578_at	ADAMTS-like 4	ADAMTSL4	4.96	4.94	4.76
200831_s_at	stearoyl-CoA desa	SCD	9.90	9.87	9.72
212967_x_at	nucleosome asser	NAP1L1	13.32	13.31	13.37
211384_s_at	calcium-sensing re	CASR	3.87	3.61	3.63
37425_g_at	coiled-coil alpha-h	CCHCR1	7.23	7.19	7.21
210306_at	l(3)mbt-like (Dros	L3MBTL	5.25	5.31	5.20

200688_at	splicing factor 3b,	SF3B3	7.73	7.67	7.74
212303_x_at	KH-type splicing re	KHSRP	7.01	7.03	7.08
202112_at	von Willebrand fa	VWF	6.02	6.18	6.06
209837_at	adaptor-related p	AP4M1	7.16	7.16	6.95
201091_s_at	chromobox homo	CBX3	10.73	10.82	11.03
205899_at	cyclin A1	CCNA1	3.90	3.88	3.72
201339_s_at	sterol carrier prot	SCP2	10.20	10.32	10.43
219901_at	FYVE, RhoGEF anc	FGD6	6.29	6.22	6.45
215074_at	myosin IB	MYO1B	4.74	4.66	4.57
217454_at	similar to hCG164	LOC203510	5.19	5.09	5.06
219769_at	inner centromere	INCENP	6.99	6.97	6.80
207224_s_at	sialic acid binding	SIGLEC7	4.61	4.56	4.43
213315_x_at	chromosome X op	CXorf40A	9.50	9.49	9.48
207650_x_at	prostaglandin E re	PTGER1	7.08	7.13	6.94
214053_at	v-erb-a erythrobla	ERBB4	3.77	3.94	3.78
204917_s_at	myeloid/lymphoic	MLLT3	4.43	4.74	4.45
214776_x_at	xylulokinase homo	XYLB	3.60	3.42	3.58
206524_at	T, brachyury homo	T	6.66	6.83	6.43
203301_s_at	cyclin D binding m	DMTF1	9.06	9.18	9.32
209333_at	unc-51-like kinase	ULK1	7.36	7.22	7.44
206743_s_at	asialoglycoprotein	ASGR1	6.13	6.04	5.69
215487_x_at	family with seque	FAM182B	4.93	5.02	4.71
201075_s_at	SWI/SNF related,	SMARCC1	9.27	9.13	9.27
207405_s_at	RAD17 homolog (S	RAD17	10.29	10.41	10.46
219082_at	amidohydrolase d	AMDHD2	6.87	6.99	6.55
213148_at	chromosome 2 op	C2orf72	7.23	6.98	6.79
205771_s_at	A kinase (PRKA) a	AKAP7	8.21	8.00	8.46
207746_at	polymerase (DNA	POLQ	6.31	6.24	6.45
210585_s_at	transportin 2	TNPO2	5.37	5.46	5.03
216848_at	---	---	5.30	5.57	5.27
204706_at	inositol polyphosp	INPP5E	8.05	8.02	7.93
220644_at	---	---	5.81	5.81	5.87
208653_s_at	CD164 molecule, s	CD164	8.29	8.29	8.48
215752_at	salt-inducible kina	SIK2	4.12	4.26	4.00
217761_at	acireductone diox	ADI1	12.41	12.44	12.54
202911_at	mutS homolog 6 (	MSH6	9.64	9.93	9.85
220899_at	---	---	3.65	3.42	3.11
208006_at	forkhead box I1	FOXI1	4.84	5.21	5.00
203162_s_at	katanin p80 (WD r	KATNB1	9.21	9.13	9.30
214783_s_at	annexin A11	ANXA11	8.43	8.38	8.39
218899_s_at	brain and acute le	BAALC	3.56	3.72	3.75
203456_at	PRA1 domain fam	PRAF2	7.13	7.24	7.00
201304_at	NADH dehydroger	NDUFA5	10.97	11.02	11.27
213210_at	TAF6-like RNA pol	TAF6L	6.31	6.14	6.06
210063_at	sarcosine dehydro	SARDH	6.46	6.12	5.96
221025_x_at	pseudouridylate s	PUS7L	7.33	7.60	7.43
221376_at	fibroblast growth	FGF17	5.40	5.03	5.13

207149_at	cadherin 12, type	CDH12	8.37	8.42	8.56
215790_at	adherens junction	AJAP1	4.35	4.53	4.62
205145_s_at	myosin, light chai	MYL5	5.90	5.83	5.58
220394_at	fibroblast growth	FGF20	3.34	3.16	3.16
35436_at	golgin A2	GOLGA2	8.26	8.17	8.19
215806_x_at	TCR gamma alter	TARP /// TRGC2	4.10	3.78	3.86
214919_s_at	ANKHD1-EIF4EBP:	ANKHD1-EIF4EBP3	7.42	7.49	7.44
220519_s_at	lens intrinsic mem	LIM2	5.53	5.46	5.42
206349_at	leucine-rich, gliom	LGI1	3.29	3.41	3.23
216002_at	---	---	3.67	3.79	3.83
219594_at	ninjurin 2	NINJ2	4.25	4.40	4.41
215929_at	hypothetical prot	LOC283079	3.38	3.36	3.31
207201_s_at	solute carrier fam	SLC22A1	5.25	4.96	5.19
220282_at	resistance to inhil	RIC3	4.36	4.27	4.20
206821_x_at	ArfGAP with FG re	AGFG2	7.16	6.94	6.74
219595_at	similar to zinc fing	LOC100287515 ///	8.54	8.64	8.65
221661_at	solute carrier fam	SLC22A7	4.30	4.02	4.06
217698_at	---	---	3.03	3.02	2.97
209372_x_at	tubulin, beta 2A /,	TUBB2A /// TUBB2	8.21	8.41	8.15
211039_at	cholinergic recept	CHRNA1	3.77	3.64	3.64
207184_at	solute carrier fam	SLC6A13	5.51	5.53	5.51
213283_s_at	sal-like 2 (Drosopl	SALL2	6.50	6.41	6.24
209711_at	solute carrier fam	SLC35D1	8.28	8.47	8.56
219288_at	chromosome 3 op	C3orf14	10.34	10.48	10.45
217688_at	---	---	4.94	5.20	5.01
219583_s_at	spermatogenesis	SPATA7	6.83	7.00	7.10
202045_s_at	glucocorticoid rec	GRLF1	7.14	7.12	7.17
218863_s_at	tensin 1	TNS1	5.20	4.85	5.01
216780_at	---	---	3.66	3.43	3.45
218676_s_at	phosphatidylcholi	PCTP	8.54	8.56	8.67
202583_s_at	RAN binding prote	RANBP9	8.08	7.92	8.22
215284_at	---	---	4.22	4.02	3.76
208708_x_at	eukaryotic transla	EIF5	11.50	11.35	11.58
211919_s_at	chemokine (C-X-C	CXCR4	4.49	4.48	4.43
220549_at	RAD54 homolog E	RAD54B	5.02	4.99	5.03
218321_x_at	serine/threonine/	STYXL1	8.53	8.52	8.64
205399_at	doublecortin-like	DCLK1	4.03	3.98	4.09
209258_s_at	structural mainte	SMC3	9.71	9.86	9.95
210516_at	family with seque	FAM120A	3.25	3.20	3.46
217036_at	hypothetical prot	LOC100293679	4.51	4.11	3.98
204332_s_at	aspartylglucosami	AGA	8.26	8.17	8.23
213972_at	---	---	6.92	6.79	6.92
214372_x_at	endoplasmic retic	ERN2	8.02	8.21	8.19
201139_s_at	Sjogren syndrome	SSB	11.16	11.32	11.44
208020_s_at	calcium channel, \	CACNA1C	6.42	6.32	6.49
209205_s_at	LIM domain only 4	LMO4	10.77	10.57	10.76
201838_s_at	suppressor of Ty 7	SUPT7L	6.35	6.48	6.48

216525_x_at	postmeiotic segre	PMS2L3	8.84	9.07	9.06
221430_s_at	ring finger protein	RNF146	6.29	6.48	6.80
211714_x_at	tubulin, beta	TUBB	13.40	13.39	13.33
217845_x_at	HIG1 hypoxia indu	HIGD1A	11.78	11.84	11.92
205800_at	---	---	4.19	4.04	4.00
215259_s_at	cell adhesion mol	CADM4	5.13	4.80	4.82
215037_s_at	BCL2-like 1	BCL2L1	6.47	6.38	6.60
212269_s_at	minichromosome	MCM3AP	9.40	9.33	9.44
205078_at	phosphatidylinosi	PIGF	9.53	9.44	9.67
221587_s_at	chromosome 19 c	C19orf24	8.39	8.22	8.42
210058_at	mitogen-activated	MAPK13	5.50	5.22	5.12
213706_at	glycerol-3-phosph	GPD1	6.22	6.27	6.13
213078_x_at	lysophosphatidylc	LPCAT4	6.57	6.50	6.53
220444_at	zinc finger protein	ZNF557	6.17	6.26	5.98
209197_at	synaptotagmin XI	SYT11	7.11	7.26	7.10
205471_s_at	dachshund homol	DACH1	4.70	4.80	4.57
200735_x_at	nascent polypepti	NACA	14.19	14.19	14.22
211200_s_at	EF-hand calcium b	EFCAB2	7.46	7.42	7.36
207547_s_at	family with seque	FAM107A	7.28	7.36	7.27
219643_at	low density lipopr	LRP1B	3.21	3.19	3.23
209120_at	nuclear receptor s	NR2F2	8.60	8.68	8.79
202495_at	tubulin folding co	TBCC	8.72	8.79	8.78
210352_at	bromodomain con	BRD8	3.60	3.55	3.62
222032_s_at	ubiquitin specific	USP7	7.25	7.14	6.99
211687_x_at	killer cell immuno	KIR3DL1	4.44	4.69	4.86
210942_s_at	ST3 beta-galactos	ST3GAL6	8.85	8.94	9.08
216059_at	paired box 3	PAX3	5.56	5.71	5.54
221826_at	angel homolog 2 (	ANGEL2	7.55	7.66	7.81
204153_s_at	MFNG O-fucosylp	MFNG	4.34	4.50	4.27
215171_s_at	translocase of inn	TIMM17A	11.83	12.05	12.01
213591_at	aldehyde dehydro	ALDH7A1	5.57	5.07	5.33
217211_at	---	---	8.24	8.39	8.42
210596_at	---	---	8.43	8.53	8.43
218801_at	UDP-glucose glyco	UGGT2	8.64	8.65	8.77
211156_at	cyclin-dependent	CDKN2A	5.31	5.46	5.01
220479_at	hypothetical LOC2	LOC29034	4.55	4.51	4.22
203506_s_at	mediator complex	MED12	6.52	6.81	6.67
213084_x_at	ribosomal protein	RPL23A	15.15	15.15	15.19
216826_at	---	---	3.79	3.76	3.92
208748_s_at	flotillin 1	FLOT1	7.01	6.88	6.79
214800_x_at	basic transcription	BTF3	12.41	12.46	12.46
211568_at	brain-specific angi	BAI3	3.40	3.19	3.32
220127_s_at	F-box and leucine-	FBXL12	9.18	9.05	9.00
208866_at	casein kinase 1, al	CSNK1A1	9.22	9.19	9.47
201748_s_at	scaffold attachme	SAFB	9.21	9.08	8.98
219832_s_at	homeobox C13	HOXC13	8.23	8.21	8.13
218057_x_at	COX4 neighbor	COX4NB	10.63	10.66	10.59

214606_at	tetraspanin 2	TSPAN2	3.00	3.07	3.14
213326_at	vesicle-associated	VAMP1	7.86	7.88	7.88
215701_at	---	---	6.93	6.92	6.74
216960_s_at	zinc finger protein	ZNF133	7.40	7.46	7.50
210048_at	N-ethylmaleimide	NAPG	7.79	7.91	7.85
204443_at	arylsulfatase A	ARSA	6.58	6.53	6.55
211289_x_at	cyclin-dependent	CDK11A /// CDK11	7.79	7.91	7.98
216665_s_at	testis-specific tran	TTTY2	3.38	3.19	3.24
214754_at	tet oncogene fam	TET3	6.07	5.97	5.86
216842_x_at	similar to RNA bin	RBM /// RBMY1A1	7.01	6.62	6.72
201632_at	eukaryotic transla	EIF2B1	10.58	10.60	10.64
209928_s_at	musculin	MSC	6.33	6.04	6.15
207441_at	submaxillary gland	SMR3B	3.51	3.72	3.63
215492_x_at	pre T-cell antigen	PTCRA	7.81	7.89	7.96
222165_x_at	chromosome 9 op	C9orf16	7.28	7.39	7.20
212829_at	phosphatidylinosi	PIP4K2A	9.92	9.81	9.95
210096_at	cytochrome P450,	CYP4B1	11.38	11.24	11.42
209617_s_at	catenin (cadherin-	CTNND2	5.34	5.03	5.07
218739_at	abhydrolase doma	ABHD5	7.84	7.96	7.89
221496_s_at	transducer of ERB	TOB2	7.30	7.29	7.16
200925_at	cytochrome c oxic	COX6A1	12.90	12.90	12.94
217446_x_at	---	---	7.04	7.23	7.16
213985_s_at	Chromosome 19 c	C19orf6	5.39	5.49	5.39
220357_s_at	serum/glucocortic	SGK2	7.07	7.10	6.93
212849_at	axin 1	AXIN1	7.85	7.82	7.85
208414_s_at	homeobox B3	HOXB3	7.13	6.90	6.82
210253_at	HIV-1 Tat interact	HTATIP2	6.51	6.75	6.65
202433_at	solute carrier fam	SLC35B1	10.14	10.10	10.24
215805_at	---	---	4.35	4.21	4.51
202501_at	microtubule-assoc	MAPRE2	9.41	9.44	9.47
203935_at	activin A receptor	ACVR1	9.41	9.32	9.58
219455_at	chromosome 7 op	C7orf63	6.48	6.41	6.32
219313_at	GRAM domain con	GRAMD1C	3.25	3.39	3.34
217009_at	phosphoglycerate	PGK2	6.53	6.37	6.50
202769_at	cyclin G2	CCNG2	8.76	8.60	8.79
218275_at	solute carrier fam	SLC25A10	8.09	8.15	8.02
216025_x_at	cytochrome P450,	CYP2C9	4.05	4.19	3.92
206454_s_at	rhodopsin	RHO	5.30	5.26	4.76
37966_at	parvin, beta	PARVB	8.18	8.14	8.14
202818_s_at	synovial sarcoma	SS18	9.13	9.19	8.99
218357_s_at	translocase of inn	TIMM8B	11.22	11.26	11.26
202581_at	heat shock 70kDa	HSPA1A /// HSPA1	7.36	7.62	7.47
204690_at	syntaxin 8	STX8	8.89	8.90	8.96
204261_s_at	presenilin 2 (Alzhe	PSEN2	5.13	4.97	5.15
214310_s_at	zinc finger protein	ZFPL1	6.59	6.16	6.15
221036_s_at	anterior pharynx c	APH1B	6.31	6.09	5.84
208904_s_at	ribosomal protein	RPS28	14.71	14.67	14.69

218599_at	REC8 homolog (ye	REC8	6.19	6.44	6.05
211582_x_at	leukocyte specific	LST1	5.36	5.32	5.42
218165_at	MYST/Esa1-associ	MEAF6	9.62	9.73	9.84
206632_s_at	apolipoprotein B r	APOBEC3B	10.11	10.24	10.18
207128_s_at	zinc finger protein	ZNF223	3.93	3.94	4.27
216191_s_at	T cell receptor alp	TRA@ /// TRD@	3.27	3.23	3.09
219223_at	chromosome 9 op	C9orf7	7.22	6.84	7.21
212661_x_at	peptidylprolyl isor	PPIA	14.61	14.63	14.63
213545_x_at	sorting nexin 3	SNX3	10.76	10.82	10.97
222231_s_at	leucine rich repea	LRRC59	12.01	11.98	12.00
212407_at	methyltransferase	METTL13	8.47	8.54	8.50
208493_at	homeobox A11	HOXA11	5.12	5.02	5.03
203533_s_at	cullin 5	CUL5	7.58	7.66	7.83
202596_at	endosulfine alpha	ENSA	11.31	11.19	11.30
211939_x_at	basic transcription	BTF3	14.14	14.13	14.18
204780_s_at	Fas (TNF receptor	FAS	8.79	8.91	9.15
213592_at	apelin receptor	APLNR	4.46	4.55	4.38
214820_at	bromodomain and	BRWD1	6.53	6.70	6.63
219480_at	snail homolog 1 (D	SNAI1	6.64	6.22	6.33
206888_s_at	Rho GDP dissociat	ARHGDIG	6.23	6.40	5.95
216285_at	DiGeorge syndrom	DGCR14	5.08	4.87	5.06
204833_at	ATG12 autophagy	ATG12	8.73	8.81	8.83
209280_at	mannose receptor	MRC2	7.67	7.44	7.60
211445_x_at	nascent-polypepti	NACAP1	11.91	11.82	11.78
218550_s_at	leucine rich repea	LRRC20	6.75	6.94	7.06
213514_s_at	diaphanous homo	DIAPH1	8.37	8.32	8.39
222104_x_at	general transcript	GTF2H3	9.26	9.52	9.69
214482_at	zinc finger and BT	ZBTB25	7.22	7.42	7.46
220006_at	coiled-coil domain	CCDC48	5.00	5.28	5.32
214888_at	calpain 2, (m/II) la	CAPN2	6.03	6.21	6.03
209691_s_at	docking protein 4	DOK4	6.14	6.16	6.37
206582_s_at	G protein-coupled	GPR56	6.69	6.79	6.76
209148_at	retinoid X recepto	RXRB	9.07	9.00	9.15
212609_s_at	V-akt murine thym	AKT3	7.11	6.98	7.13
213915_at	natural killer cell g	NKG7	5.01	5.34	5.24
209824_s_at	aryl hydrocarbon	ARNTL	10.07	9.99	10.23
205306_x_at	kynurenine 3-mor	KMO	4.00	4.27	4.23
219827_at	uncoupling protei	UCP3	6.62	6.70	6.60
201960_s_at	MYC binding prot	MYCBP2	11.38	11.35	11.47
202279_at	chromosome 14 o	C14orf2	10.05	10.03	10.12
219394_at	phosphatidylglyce	PGS1	8.07	8.25	8.11
208137_x_at	zinc finger protein	ZNF611	7.67	7.88	7.67
211975_at	ADP-ribosylation f	ARFGAP2	8.65	8.66	8.55
210770_s_at	calcium channel, v	CACNA1A	4.66	4.47	4.30
214132_at	ATP synthase, H+	ATP5C1	7.76	8.06	7.98
221231_s_at	chromosome 14 o	C14orf102	5.01	5.07	4.85
201961_s_at	ring finger protein	RNF41	7.86	7.72	7.85

212201_at	ankyrin repeat an	ANKLE2	7.41	7.38	7.27
206547_s_at	protein phosphata	PPEF1	5.38	5.51	4.92
218174_s_at	chromosome 10 c	C10orf57	6.54	6.46	6.56
202525_at	protease, serine, 8	PRSS8	4.87	4.95	4.98
200894_s_at	FK506 binding pro	FKBP4	8.49	8.46	8.50
203185_at	Ras association (R	RASSF2	4.71	4.41	4.55
201952_at	activated leukocyt	ALCAM	9.75	9.96	9.82
217756_x_at	small EDRK-rich fa	SERF2	12.59	12.52	12.53
217668_at	Chromosome 22 c	C22orf36	4.70	5.09	5.07
215513_at	hydatidiform mol	HYMAI	5.50	5.80	5.56
205267_at	POU class 2 associ	POU2AF1	3.82	3.53	3.30
206104_at	ISL LIM homeobox	ISL1	3.68	3.68	3.65
205458_at	melanocortin 1 re	MC1R	7.24	7.26	7.17
206505_at	UDP glucuronosyl	UGT2B4	3.09	2.93	2.98
221348_at	natriuretic peptid	NPPC	4.56	4.48	4.59
209894_at	leptin receptor	LEPR	6.94	7.00	7.30
210794_s_at	maternally expres	MEG3	6.83	6.61	6.78
213854_at	synaptogyrin 1	SYNGR1	6.23	6.24	5.99
206353_at	cytochrome c oxic	COX6A2	5.32	5.03	5.01
219651_at	developmental pl	DPPA4	3.17	3.23	3.06
222005_s_at	guanine nucleotid	GNG3	5.66	5.71	5.70
208887_at	eukaryotic transla	EIF3G	11.65	11.56	11.62
214736_s_at	adducin 1 (alpha)	ADD1	8.92	8.78	8.82
205075_at	serpin peptidase i	SERPINF2	6.46	6.66	6.16
206670_s_at	glutamate decarb	GAD1	4.10	4.37	4.16
219467_at	gypsy retrotransp	GIN1	8.26	8.41	8.62
218625_at	neuritin 1	NRN1	6.23	6.57	6.52
218327_s_at	synaptosomal-ass	SNAP29	9.61	9.47	9.55
211694_at	testis-specific seri	TSSK1B	4.77	4.47	4.33
208422_at	macrophage scave	MSR1	5.17	4.93	5.14
213810_s_at	hypothetical prote	LOC100286972	5.50	5.20	5.41
202556_s_at	microspherule pro	MCRS1	7.87	7.93	8.00
222015_at	casein kinase 1, ep	CSNK1E	8.94	8.70	8.80
208536_s_at	BCL2-like 11 (apop	BCL2L11	3.93	3.90	3.99
220779_at	peptidyl arginine d	PADI3	4.99	5.27	5.28
202651_at	lysophosphatidylg	LPGAT1	9.38	9.40	9.48
213299_at	zinc finger and BT	ZBTB7A	5.80	5.88	5.86
219103_at	ArfGAP with SH3 d	ASAP3	5.92	5.91	5.79
216252_x_at	Fas (TNF receptor	FAS	7.02	6.92	7.23
211742_s_at	ecotropic viral int	EVI2B	4.76	4.98	5.20
215754_at	scavenger recepto	SCARB2	6.51	6.62	6.56
215683_at	Chromosome 18 c	C18orf22	6.07	6.32	6.36
204163_at	elastin microfibril	EMILIN1	5.51	5.14	5.25
214296_x_at	chromosome 19 c	C19orf36	6.97	6.73	6.65
206350_at	amyloid P compor	APCS	5.61	5.61	5.14
209191_at	tubulin, beta 6	TUBB6	12.23	12.34	12.15
209146_at	sterol-C4-methyl c	SC4MOL	11.15	11.59	11.38

206528_at	transient receptor	TRPC6	4.65	4.41	4.36
213353_at	ATP-binding casse	ABCA5	3.59	3.36	3.47
215548_s_at	sec1 family domai	SCFD1	10.60	10.71	10.94
203885_at	RAB21, member F	RAB21	9.53	9.60	9.62
203976_s_at	chromatin assemk	CHAF1A	7.55	7.57	7.47
216940_x_at	Y box binding prot	YBX1 /// YBX1P2	5.68	5.47	5.55
221553_at	magnesium transp	MAGT1	8.23	8.37	9.01
219486_at	dihydrouridine syı	DUS2L	8.45	8.48	8.42
209888_s_at	myosin, light chai	MYL1	4.62	5.05	4.92
219134_at	EGF, latrophilin ar	ELTD1	3.02	3.37	3.25
211822_s_at	NLR family, pyrin (	NLRP1	5.62	5.73	5.52
215877_at	chromosome 14 c	C14orf56	6.27	5.98	5.97
205484_at	signaling threshol	SIT1	6.14	6.15	5.97
214672_at	tubulin tyrosine li	TTLL5	7.95	7.89	7.96
212356_at	KH and NYN doma	KHNYN	8.17	8.26	8.21
214375_at	similar to PTPRF ir	LOC729222 /// PPI	7.22	7.41	7.21
214795_at	zinc finger, MYND	ZMYND8	7.57	7.58	7.75
207488_at	---	---	6.33	6.29	5.98
212272_at	lipin 1	LPIN1	6.11	6.46	6.07
221426_s_at	olfactory receptor	OR3A3	5.70	5.54	5.42
213969_x_at	ribosomal protein	RPL29	14.50	14.51	14.56
206678_at	gamma-aminobut	GABRA1	3.63	3.85	3.69
206271_at	toll-like receptor 3	TLR3	6.39	6.21	6.26
43544_at	mediator complex	MED16	8.75	8.73	8.72
210122_at	protamine 2	PRM2	4.56	4.42	4.23
219624_at	BCL2-associated a	BAG4	4.71	5.26	4.93
215325_x_at	chromosome 19 c	C19orf26	5.32	5.15	5.18
208889_s_at	nuclear receptor c	NCOR2	6.16	6.13	5.90
215760_s_at	strawberry notch	SBNO2	6.14	6.03	5.74
215726_s_at	cytochrome b5 ty	CYB5A	11.65	11.68	11.78
209739_s_at	patatin-like phosp	PNPLA4	4.62	4.63	4.69
217353_at	heterogeneous nu	HNRNPA1 /// HNR	5.26	5.47	5.46
214579_at	NIPA-like domain	NIPAL3	8.99	8.91	9.09
219752_at	RAS protein activa	RASAL1	6.68	6.59	6.53
207273_at	---	---	3.64	3.42	3.55
213178_s_at	mitogen-activatec	MAPK8IP3	7.44	7.56	7.41
208225_at	fibroblast growth	FGFR2	5.12	5.13	5.22
202237_at	nicotinamide N-m	NNMT	12.46	12.35	12.41
212862_at	CDP-diacylglycero	CDS2	7.97	8.03	8.01
216009_at	Solute carrier fam	SLC39A9	5.33	5.37	5.46
208519_x_at	gonadotropin-rele	GNRH2	7.01	7.09	6.80
216832_at	runt-related trans	RUNX1T1	5.24	5.28	5.10
208246_x_at	---	---	10.95	10.89	10.71
201010_s_at	thioredoxin intera	TXNIP	13.43	13.26	13.39
37892_at	collagen, type XI, a	COL11A1	3.90	3.85	3.56
203102_s_at	mannosyl (alpha-1	MGAT2	11.06	11.15	11.27
213223_at	ribosomal protein	RPL28	8.37	8.45	8.49

202053_s_at	aldehyde dehydro	ALDH3A2	9.17	9.25	9.41
203596_s_at	interferon-induce	IFIT5	7.90	7.80	7.96
220575_at	family with seque	FAM106A	5.05	5.28	5.11
218479_s_at	exportin 4	XPO4	6.99	7.00	7.06
211586_s_at	ATPase, Ca++ tran	ATP2B2	3.60	3.77	3.54
220118_at	zinc finger and BT	ZBTB32	5.86	5.68	5.83
219667_s_at	B-cell scaffold pro	BANK1	3.27	3.28	3.11
210918_at	---	---	4.14	4.02	3.78
217805_at	interleukin enhan	ILF3	9.15	9.04	9.19
216488_s_at	ATPase, class VI, t	ATP11A	4.95	5.31	5.17
219178_at	queuine tRNA-ribo	QTRTD1	8.18	8.24	8.45
215488_at	---	---	6.26	5.83	5.88
206192_at	corneodesmosin	CDSN	5.60	5.83	5.66
210104_at	mediator complex	MED6	8.59	8.77	8.85
206876_at	single-minded hor	SIM1	3.75	4.03	3.85
222163_s_at	spermatogenesis a	SPATA5L1	9.49	9.60	9.52
203670_at	tubulin tyrosine li	TTLL3	6.23	5.99	6.27
214835_s_at	succinate-CoA liga	SUCLG2	10.18	10.27	10.71
215362_at	---	---	4.65	4.82	4.78
200788_s_at	phosphoprotein e	PEA15	10.67	10.47	10.75
214381_at	septin 7 pseudoge	LOC441601	4.57	4.59	4.47
207490_at	tubulin, alpha 4b (	TUBA4B	7.52	7.58	7.69
205780_at	BCL2-interacting k	BIK	4.99	4.88	4.51
214734_at	exophilin 5	EXPH5	7.04	6.85	7.06
201313_at	enolase 2 (gamma	ENO2	7.17	6.98	6.92
201373_at	plectin	PLEC	8.19	8.27	8.30
216317_x_at	Rh blood group, C	RHCE	5.92	5.99	6.05
201729_s_at	KIAA0100	KIAA0100	9.94	9.79	9.72
209645_s_at	aldehyde dehydro	ALDH1B1	7.30	6.95	7.20
211086_x_at	NIMA (never in m	NEK1	7.18	7.18	7.28
200861_at	CCR4-NOT transcr	CNOT1	7.85	7.62	7.66
219932_at	solute carrier fam	SLC27A6	3.69	3.42	3.34
207181_s_at	caspase 7, apopto	CASP7	8.69	8.89	8.97
214268_s_at	myotubularin rela	MTMR4	8.88	9.06	9.06
200979_at	pyruvate dehydro	PDHA1	8.33	8.61	8.59
220189_s_at	mannosyl (alpha-1	MGAT4B	8.73	8.64	8.65
58900_at	hypothetical prote	LOC222070	8.51	8.59	8.45
210280_at	myelin protein zer	MPZ	5.24	5.13	5.08
210492_at	microfibrillar-asso	MFAP3L	4.54	4.09	4.26
205164_at	glycine C-acetyltra	GCAT	9.42	9.33	9.40
206839_at	chromosome 22 c	C22orf31	5.99	5.74	5.83
222254_at	---	---	4.00	4.11	4.08
214311_at	zinc finger protein	ZFPL1	7.40	7.24	7.08
200927_s_at	RAB14, member R	RAB14	9.25	9.20	9.31
209202_s_at	exostoses (multip	EXTL3	7.31	7.06	7.22
221988_at	chromosome 19 c	C19orf42	7.55	7.51	7.51
207264_at	KDEL (Lys-Asp-Glu	KDELR3	5.73	5.43	5.71

204190_at	ubiquitin specific	USPL1	8.44	8.43	8.48
200618_at	LIM and SH3 prot	LASP1	11.16	11.28	11.25
211722_s_at	histone deacetyla	HDAC6	5.41	5.33	5.27
219074_at	transmembrane p	TMEM184C	9.56	9.48	9.54
214943_s_at	RNA binding moti	RBM34	7.34	7.50	7.53
200069_at	squamous cell car	SART3	9.34	9.27	9.41
211449_at	mutS homolog 6 (	MSH6	3.63	3.60	3.65
214076_at	glucose-fructose c	GFOD2	6.87	6.76	7.01
212696_s_at	ring finger protei	RNF4	10.59	10.68	10.65
38918_at	SRY (sex determin	SOX13	6.06	6.05	5.98
222295_x_at	---	---	6.32	6.21	5.99
208742_s_at	Sin3A-associated	SAP18	12.09	12.13	12.27
216692_at	---	---	5.04	4.94	4.89
217798_at	CCR4-NOT transcr	CNOT2	9.63	9.60	9.66
202327_s_at	polycystic kidney	PKD1	6.43	6.18	6.29
216508_x_at	CCCTC-binding fac	CTCFL /// HMGB1	10.86	11.02	11.31
220858_at	sorbin and SH3 dc	SORBS2	4.82	4.56	4.78
218944_at	pyrroline-5-carbo	PYCRL	7.39	7.18	7.06
207877_s_at	nuclear VCP-like	NVL	8.50	8.53	8.61
216676_x_at	killer cell immuno	KIR3DL3	5.26	5.60	5.26
207179_at	T-cell leukemia hc	TLX1	5.39	4.91	4.91
220230_s_at	cytochrome b5 re	CYB5R2	4.20	4.20	4.17
219244_s_at	mitochondrial ribo	MRPL46	9.75	9.63	9.81
212568_s_at	dihydrolipoamide	DLAT	8.50	8.81	8.83
221359_at	glial cell derived n	GDNF	5.48	5.73	5.50
202145_at	lymphocyte antige	LY6E	7.15	7.07	7.10
219211_at	ubiquitin specific	USP18	6.92	6.78	7.00
217754_at	DEAD (Asp-Glu-Al	DDX56	8.86	8.86	8.88
204771_s_at	transcription term	TTF1	8.88	9.10	9.01
209536_s_at	EH-domain contai	EHD4	10.25	10.16	10.21
218995_s_at	endothelin 1	EDN1	9.85	9.84	9.90
212460_at	chromosome 14 c	C14orf147	9.39	9.50	9.72
220340_at	growth regulation	GREB1L	4.70	4.62	4.66
219390_at	FK506 binding pro	FKBP14	7.80	8.09	8.03
204836_at	glycine dehydroge	GLDC	5.47	5.47	5.20
217400_at	---	---	3.33	3.23	3.29
203561_at	Fc fragment of IgG	FCGR2A	4.87	4.64	4.57
216804_s_at	PDZ and LIM dom	PDLIM5	7.86	7.89	7.74
211316_x_at	CASP8 and FADD-	CFLAR	8.33	8.53	8.48
203801_at	mitochondrial ribo	MRPS14	6.65	6.75	7.07
216603_at	solute carrier fam	SLC7A8	5.44	5.47	5.16
207495_at	RAB28, member F	RAB28	6.57	6.53	6.70
216389_s_at	DDB1 and CUL4 as	DCAF11	7.33	7.11	7.29
217743_s_at	transmembrane p	TMEM30A	9.61	9.86	9.98
216292_at	---	---	4.63	4.83	4.64
213806_at	Purine-rich eleme	PURA	5.86	5.67	5.98
218576_s_at	dual specificity ph	DUSP12	10.39	10.39	10.57

213989_x_at	SET domain conta	SETD4	8.21	8.05	8.31
212585_at	oxysterol binding	OSBPL8	10.15	10.24	10.26
206318_at	serine peptidase i	SPINLW1	3.72	3.75	3.43
218360_at	RAB22A, member	RAB22A	8.77	8.65	8.78
219531_at	centrosomal prot	CEP72	8.92	9.00	9.07
204956_at	methylthioadenos	MTAP	3.60	3.27	3.37
211047_x_at	adaptor-related p	AP2S1	11.65	11.54	11.61
207517_at	laminin, gamma 2	LAMC2	4.51	4.51	4.23
210586_x_at	Rh blood group, D	RHD	6.08	5.82	6.06
200672_x_at	spectrin, beta, no	SPTBN1	9.60	9.57	9.47
218877_s_at	tRNA methyltrans	TRMT11	9.61	9.84	9.94
206786_at	histatin 3	HTN3	3.51	3.46	3.28
213889_at	---	---	8.20	8.18	8.26
220780_at	phospholipase A2	PLA2G3	5.28	4.86	5.12
201497_x_at	myosin, heavy cha	MYH11	5.64	5.57	5.55
202163_s_at	CCR4-NOT transcr	CNOT8	8.82	8.96	9.25
210202_s_at	bridging integrato	BIN1	7.42	7.39	7.29
216307_at	diacylglycerol kina	DGKB	3.68	3.48	3.51
214506_at	G protein-couplec	GPR182	6.49	6.66	6.58
216440_at	ELKS/RAB6-intera	ERC1	3.54	3.76	3.66
200084_at	chromosome 11 c	C11orf58	11.86	11.91	11.97
219246_s_at	2-oxoglutarate an	OGFOD2	7.23	7.30	7.01
203960_s_at	heat shock protei	HSPB11	8.68	8.87	9.01
200674_s_at	ribosomal protein	RPL32	14.99	14.95	14.95
217552_x_at	complement com	CR1	3.51	3.33	3.22
206891_at	actinin, alpha 3	ACTN3	6.16	6.48	6.12
220754_at	armadillo repeat c	ARMC9	4.31	4.08	4.22
210192_at	ATPase, aminoph	ATP8A1	4.76	4.36	4.84
210663_s_at	kynureninase (L-k	KYNU	5.98	5.62	5.40
214130_s_at	phosphodiesteras	PDE4DIP	6.94	6.85	6.82
212870_at	son of sevenless h	SOS2	8.81	8.79	8.99
209611_s_at	solute carrier fam	SLC1A4	6.10	5.99	5.88
221060_s_at	toll-like receptor 4	TLR4	5.30	5.32	5.52
205966_at	TAF13 RNA polym	TAF13	5.57	5.72	5.61
201104_x_at	neuroblastoma br	LOC200030 /// NB	10.09	10.12	10.03
217307_at	aconitase 2, mito	ACO2	5.35	5.61	5.73
218811_at	ORAI calcium rele	ORAI2	5.84	5.91	5.84
207117_at	zinc finger protein	ZNF117	3.20	3.17	3.21
222048_at	crystallin, beta B2	CRYBB2P1	7.80	7.54	7.70
220697_at	---	---	3.42	3.54	3.45
221314_at	growth differentia	GDF9	5.89	6.26	6.11
214710_s_at	cyclin B1	CCNB1	12.38	12.44	12.48
201447_at	TIA1 cytotoxic gra	TIA1	8.28	8.37	8.47
221907_at	tRNA methyltrans	TRMT61A	8.34	8.19	8.38
210721_s_at	p21 protein (Cdc4	PAK7	3.30	3.31	3.27
217418_x_at	membrane-spann	MS4A1	4.11	4.22	4.05
217094_s_at	itchy E3 ubiquitin	ITCH	8.19	8.08	8.08

221608_at	---	---	5.64	5.85	5.72
208624_s_at	eukaryotic transla	EIF4G1	9.20	9.23	9.19
210243_s_at	UDP-Gal:betaGlcN	B4GALT3	8.38	8.18	8.38
221260_s_at	cysteine-serine-ric	CSRNP2	7.58	7.59	7.89
210845_s_at	plasminogen activ	PLAUR	6.98	6.99	6.79
204491_at	phosphodiesteras	PDE4D	7.71	7.50	7.85
217737_x_at	chromosome 20 c	C20orf43	9.34	9.37	9.45
51158_at	family with seque	FAM174B	3.74	3.82	3.82
209141_at	ubiquitin-conjugat	UBE2G1	9.53	9.59	9.55
213832_at	potassium voltage	KCND3	5.02	5.23	4.62
214121_x_at	PDZ and LIM dom	PDLIM7	5.50	5.70	5.32
212257_s_at	SWI/SNF related,	SMARCA2	6.17	6.07	6.11
206645_s_at	nuclear receptor s	NR0B1	4.25	4.31	4.15
202211_at	ADP-ribosylation f	ARFGAP3	9.54	9.60	9.74
216769_x_at	---	---	4.47	4.49	4.57
202848_s_at	phosphoenolpyru	PCK2	8.21	8.17	8.24
204031_s_at	poly(rC) binding p	PCBP2	12.28	12.16	12.22
216303_s_at	myotubularin rela	MTMR1	5.00	5.05	4.98
203520_s_at	zinc finger protein	ZNF318	6.71	6.68	6.95
221857_s_at	tight junction asso	TJAP1	8.08	7.86	7.77
220747_at	hypothetical LOC2	HSPC072	6.14	6.44	6.09
206406_at	sperm mitochond	SMCP	5.06	5.16	5.35
209878_s_at	v-rel reticuloendo	RELA	7.62	7.74	7.44
212706_at	similar to HSPC04	LOC100286937 ///	4.14	4.22	4.14
203381_s_at	apolipoprotein E	APOE	6.39	6.19	6.24
202939_at	zinc metallopeptic	ZMPSTE24	10.89	11.01	11.06
208566_at	similar to inward r	LOC100131509 ///	3.86	3.79	3.81
217404_s_at	collagen, type II, a	COL2A1	4.86	4.70	4.71
218143_s_at	secretory carrier r	SCAMP2	8.83	8.72	8.70
207000_s_at	protein phosphata	PPP3CC	8.03	8.17	8.28
207912_s_at	deleted in azoosp	DAZ1 /// DAZ2 ///	3.28	3.19	3.24
221984_s_at	family with seque	FAM134A	10.08	10.05	10.09
206861_s_at	CGG triplet repeat	CGGBP1	8.00	7.86	8.08
201618_x_at	glycosylphosphati	GPAA1	8.78	8.89	8.91
216261_at	integrin, beta 3 (p	ITGB3	5.00	5.18	5.06
208402_at	interleukin 17A	IL17A	3.55	3.83	3.50
216178_x_at	integrin, beta 1 (fi	ITGB1	4.07	4.15	4.36
208188_at	keratin 9	KRT9	6.90	6.80	6.45
214807_at	---	---	4.06	4.38	4.38
205243_at	solute carrier fam	SLC13A3	6.45	6.38	6.10
51146_at	phosphatidylinosi	PIGV	8.51	8.30	8.62
205872_x_at	phosphodiesteras	PDE4DIP	5.30	5.50	5.09
219136_s_at	lipase maturation	LMF1	4.61	4.57	4.54
216110_x_at	---	---	5.52	5.55	5.36
219448_at	transmembrane p	TMEM70	7.49	7.48	7.37
222128_at	NOP2/Sun domain	NSUN6	5.72	5.98	5.63
207023_x_at	keratin 10	KRT10	10.73	10.72	11.01

213525_at	---	---	6.88	6.41	6.43
201670_s_at	myristoylated alar	MARCKS	9.86	9.74	10.03
202536_at	chromatin modify	CHMP2B	9.42	9.42	9.76
207536_s_at	tumor necrosis fa	TNFRSF9	6.51	5.90	6.44
64486_at	coronin, actin bin	CORO1B	8.76	8.80	8.87
204562_at	interferon regulat	IRF4	5.65	5.75	5.61
211632_at	immunoglobulin h	IGH@ /// IGHD ///	5.44	5.07	5.06
206341_at	interleukin 2 rece	IL2RA	5.22	5.20	5.03
220902_at	---	---	5.30	4.81	5.29
216875_x_at	B1 for mucin	HAB1	7.06	6.94	6.78
220251_at	chromosome 1 op	C1orf107	7.82	7.86	7.78
218940_at	chromosome 14 c	C14orf138	8.60	8.91	8.78
220814_at	---	---	3.57	3.67	3.51
214527_s_at	polyglutamine bin	PQBP1	9.53	9.35	9.56
203930_s_at	microtubule-asso	MAPT	5.52	5.44	5.35
202061_s_at	sel-1 suppressor c	SEL1L	9.78	9.67	10.01
213215_at	adaptor-related p	AP3S2	7.35	7.35	7.05
203694_s_at	DEAH (Asp-Glu-Al	DHX16	9.17	9.23	9.27
204724_s_at	collagen, type IX,	COL9A3	5.41	5.50	5.61
217762_s_at	RAB31, member R	RAB31	10.58	10.64	10.66
219301_s_at	contactin associat	CNTNAP2	5.98	5.82	6.06
221411_at	homeobox D12	HOXD12	3.85	3.81	3.76
218891_at	chromosome 10 c	C10orf76	7.42	7.50	7.44
216498_at	---	---	4.17	4.09	4.24
212474_at	AVL9 homolog (S.	AVL9	9.30	9.27	9.37
221724_s_at	C-type lectin dom	CLEC4A	4.53	4.51	4.36
37831_at	signal-induced pro	SIPA1L3	8.28	8.43	8.49
219186_at	zinc finger and BT	ZBTB7A	7.81	7.88	7.99
205254_x_at	transcription facto	TCF7	4.88	4.95	4.82
203248_at	zinc finger protein	ZNF24	7.33	7.25	7.22
218792_s_at	B-box and SPRY do	BSPRY	4.95	4.86	4.67
208593_x_at	corticotropin rele	CRHR1	5.24	5.06	5.00
205572_at	angiopoietin 2	ANGPT2	4.00	3.73	3.69
206774_at	FERM and PDZ do	FRMPD1	4.96	4.70	4.92
64942_at	G protein-couplec	GPR153	8.44	8.34	8.19
209031_at	cell adhesion mol	CADM1	9.22	9.04	9.21
206790_s_at	NADH dehydroger	NDUFB1	12.42	12.45	12.42
203221_at	transducin-like en	TLE1	9.71	9.70	9.72
207142_at	potassium inward	KCNJ3	3.76	3.40	3.49
203672_x_at	thiopurine S-meth	TPMT	8.13	8.31	8.42
218153_at	cysteinyl-tRNA syr	CARS2	9.56	9.49	9.51
209104_s_at	NHP2 ribonucleop	NHP2	13.31	13.23	13.26
205676_at	cytochrome P450,	CYP27B1	6.49	6.56	6.67
216123_x_at	---	---	4.36	4.51	4.47
214484_s_at	sigma non-opioid	SIGMAR1	8.93	8.99	9.15
211127_x_at	ectodysplasin A	EDA	3.54	3.78	3.60
211333_s_at	Fas ligand (TNF su	FASLG	4.07	4.02	3.95

207844_at	interleukin 13	IL13	6.10	5.97	5.89
215905_s_at	small nuclear ribo	SNRNP40	10.57	10.49	10.59
210858_x_at	ataxia telangiecta	ATM	6.96	6.94	7.02
201901_s_at	YY1 transcription	YY1	11.58	11.50	11.58
213181_s_at	molybdenum cofa	MOCS1	6.81	6.58	6.37
215931_s_at	ADP-ribosylation f	ARFGEF2	5.87	5.80	5.60
213773_x_at	NOP2/Sun domain	NSUN5	10.66	10.53	10.59
214435_x_at	v-ral simian leuke	RALA	9.00	9.09	9.25
215780_s_at	SET translocation	LOC642869 /// SET	11.84	11.88	11.94
207481_at	---	---	3.65	3.47	3.55
200840_at	lysyl-tRNA synthet	KARS	13.69	13.64	13.65
213530_at	RAB3 GTPase activ	RAB3GAP1	8.36	8.41	8.76
204057_at	interferon regulat	IRF8	6.85	6.86	6.63
202736_s_at	LSM4 homolog, U	LSM4	11.08	10.96	11.14
208544_at	adrenergic, alpha-	ADRA2B	6.83	6.72	6.51
215876_at	---	---	5.13	5.17	5.02
204870_s_at	proprotein conver	PCSK2	5.93	5.85	5.50
208275_x_at	undifferentiated e	UTF1	5.50	5.33	5.37
209856_x_at	abl-interactor 2	ABI2	7.95	7.80	7.96
207556_s_at	diacylglycerol kina	DGKZ	7.69	7.56	7.45
210193_at	myelin-associated	MOBP	4.10	4.01	3.72
208587_s_at	olfactory receptor	OR1E1 /// OR1E2	5.76	5.74	5.57
211221_at	transcriptional ad	TADA3	5.28	5.04	4.85
207306_at	transcription facto	TCF15	5.77	5.73	5.79
221088_s_at	protein phosphata	PPP1R9A	6.95	6.97	6.78
215573_at	Catalase	CAT	3.98	3.60	3.86
214515_at	olfactory receptor	OR1E1	3.44	3.42	3.34
208363_s_at	inositol polyphosp	INPP4A	5.70	6.00	5.78
222333_at	ALS2 C-terminal li	ALS2CL	6.93	6.86	6.58
211125_x_at	glutamate recepto	GRIN1	7.47	7.44	6.92
205139_s_at	uronyl-2-sulfotrar	UST	7.87	7.84	7.98
215528_at	---	---	6.21	6.28	6.11
201031_s_at	heterogeneous nu	HNRNPH1	12.43	12.55	12.60
216958_s_at	isovaleryl-CoA del	IVD	8.15	8.32	8.23
216243_s_at	interleukin 1 rece	IL1RN	5.45	5.18	5.07
220287_at	ADAM metallopep	ADAMTS9	6.57	6.71	6.67
203292_s_at	vacuolar protein s	VPS11	8.90	8.79	8.66
220364_at	hypothetical FLJ1	FLJ11235	3.94	3.93	3.75
211808_s_at	CREB binding prot	CREBBP	6.59	6.46	6.41
212767_at	mitochondrial GTI	MTG1	8.96	8.97	8.95
204582_s_at	kallikrein-related	KLK3	4.94	4.40	4.54
45749_at	family with seque	FAM65A	10.00	9.91	9.98
213426_s_at	Caveolin 2	CAV2	5.32	5.27	5.39
218675_at	solute carrier fam	SLC22A17	5.76	5.92	5.92
214660_at	Pelota homolog (I	PELO	3.37	3.33	3.49
208087_s_at	Z-DNA binding pro	ZBP1	5.39	5.20	5.19
217789_at	sorting nexin 6	SNX6	9.64	9.65	9.62

219778_at	zinc finger protein	ZFPM2	7.78	7.72	7.95
201343_at	ubiquitin-conjugat	UBE2D2	10.16	10.30	10.24
32091_at	basic leucine zippe	BLZF1	8.57	8.68	8.68
204250_s_at	centrosomal prote	CEP164	6.69	6.48	6.57
209667_at	carboxylesterase 2	CES2	7.67	7.71	7.78
212641_at	human immunode	HIVEP2	8.00	8.02	8.00
205510_s_at	hypothetical prote	FLJ10038	8.04	8.32	8.12
203678_at	myotubularin rela	MTMR15	8.76	8.86	8.84
211087_x_at	mitogen-activated	MAPK14	6.04	5.83	5.77
210734_x_at	MYC associated fa	MAX	6.94	6.87	6.76
220211_at	hypothetical prote	FLJ13224	4.09	4.13	3.67
211017_s_at	neurofibromin 2 (	NF2	5.89	5.88	5.98
207802_at	cysteine-rich secre	CRISP3	3.81	3.60	3.65
202131_s_at	RIO kinase 3 (yeas	RIOK3	8.74	8.73	8.97
208205_at	protocadherin alp	PCDHA9	4.30	4.27	4.21
217788_s_at	UDP-N-acetyl-alph	GALNT2	10.78	10.79	10.72
207872_s_at	leukocyte immuno	LILRA1	4.21	4.03	4.19
215532_x_at	zinc finger protein	ZNF492	3.29	3.31	3.26
212273_x_at	GNAS complex loc	GNAS	12.97	12.89	12.85
218263_s_at	zinc finger, BED-ty	ZBED5	9.81	9.91	9.90
204088_at	purinergic recepto	P2RX4	4.76	4.96	4.87
222375_at	---	---	4.66	4.62	4.44
215462_at	polo-like kinase 3	PLK3	6.94	6.99	6.97
210357_s_at	spermine oxidase	SMOX	7.85	7.66	7.38
203573_s_at	Rab geranylgerany	RABGGTA	7.44	7.56	7.40
208079_s_at	aurora kinase A	AURKA	11.24	11.19	11.26
204558_at	RAD54-like (S. cer	RAD54L	8.09	8.24	8.23
219793_at	sorting nexin 16	SNX16	5.31	5.14	5.66
217354_s_at	Hermansky-Pudlak	HPS1	7.89	7.75	7.68
202334_s_at	ubiquitin-conjugat	UBE2B	9.83	9.82	10.01
221308_at	fibroblast growth	FRS2	5.66	5.38	5.61
203537_at	phosphoribosyl py	PRPSAP2	8.79	8.81	8.77
211010_s_at	natural cytotoxicit	NCR3	5.82	5.63	5.54
214009_at	male-specific leth	MSL3	7.85	7.69	7.69
212751_at	ubiquitin-conjugat	UBE2N	9.45	9.66	9.65
219733_s_at	solute carrier fam	SLC27A5	8.99	8.74	8.90
209063_x_at	poly(A) binding pr	PAIP1	10.57	10.49	10.73
208970_s_at	uroporphyrinogen	UROD	10.97	10.96	11.01
203236_s_at	lectin, galactoside	LGALS9	6.56	6.54	6.52
210565_at	glucagon receptor	GCGR	6.96	7.11	6.87
211009_s_at	zinc finger protein	ZNF271	8.53	8.64	8.64
204857_at	MAD1 mitotic arre	MAD1L1	7.59	7.61	7.68
219289_at	HEAT repeat conta	HEATR3	8.87	8.87	8.97
217624_at	PDGFA associated	PDAP1	4.43	4.50	4.76
218596_at	TBC1 domain fam	TBC1D13	6.87	6.95	6.74
219973_at	arylsulfatase fami	ARSJ	9.03	8.94	9.21
207646_s_at	chromodomain pr	CDY1 /// CDY1B //	4.75	4.55	4.75

218742_at	nuclear prelamin A	NARFL	7.62	7.45	7.48
201526_at	ADP-ribosylation f	ARF5	10.36	10.25	10.14
204449_at	phosducin-like	PDCL	7.99	8.24	8.13
55662_at	chromosome 10 c	C10orf76	6.60	6.52	6.74
210727_at	calcitonin-related	CALCA	5.29	5.38	5.35
219411_at	engulfment and c	ELMO3	6.34	6.44	6.35
218987_at	activating transcri	ATF7IP	8.59	8.70	8.64
221291_at	UL16 binding prot	ULBP2	5.65	5.91	5.55
202417_at	kelch-like ECH-ass	KEAP1	9.20	9.28	9.18
216039_at	similar to PMS2 p	LOC100289811 ///	3.40	3.26	3.13
210384_at	protein arginine m	PRMT2	4.16	4.06	4.29
214607_at	p21 protein (Cdc4	PAK3	5.83	6.04	5.86
215389_s_at	troponin T type 2	TNNT2	6.41	6.41	6.10
210880_s_at	embryonal Fyn-as	EFS	5.61	5.58	5.44
215578_at	---	---	3.90	4.23	4.08
205802_at	transient receptor	TRPC1	8.60	8.68	8.81
203270_at	deoxythymidylate	DTYMK	9.81	9.73	9.80
201321_s_at	SWI/SNF related,	SMARCC2	7.98	8.15	8.10
217158_at	similar to hCG203	LOC728683	5.72	5.82	5.64
202930_s_at	succinate-CoA liga	SUCLA2	10.39	10.46	10.59
213862_at	Patatin-like phosp	PNPLA2	4.39	4.39	4.42
214017_s_at	DEAH (Asp-Glu-Ala	DHX34	6.71	6.63	6.62
56748_at	tripartite motif-co	TRIM10	4.05	4.06	4.17
217252_at	sequestosome 1	SQSTM1	3.86	3.79	3.99
211671_s_at	nuclear receptor s	NR3C1	11.59	11.42	11.69
220302_at	male germ cell-as	MAK	4.37	4.28	4.54
205233_s_at	platelet-activating	PAFAH2	6.51	6.52	6.66
208729_x_at	major histocompa	HLA-B	11.03	10.89	10.92
210394_x_at	synovial sarcoma,	SSX4 /// SSX4B	4.04	3.99	3.97
201593_s_at	zinc finger CCCH-t	ZC3H15	11.99	11.98	12.05
202167_s_at	MMS19 nucleotid	MMS19	8.85	8.81	8.82
207324_s_at	desmocollin 1	DSC1	3.17	3.18	3.44
214592_s_at	small nuclear RNA	SNAPC5	5.80	5.60	5.68
208071_s_at	leukocyte-associa	LAIR1	6.54	6.59	6.40
219600_s_at	transmembrane p	TMEM50B	9.40	9.49	9.42
205704_s_at	ATPase, H+ transp	ATP6V0A2	7.61	7.49	7.65
215057_at	hypothetical LOC1	LOC100272228	5.77	5.53	5.56
214081_at	plexin domain cor	PLXDC1	3.84	3.47	3.84
208383_s_at	phosphoenolpyru	PCK1	3.42	3.05	3.08
206418_at	NADPH oxidase 1	NOX1	3.61	3.51	3.62
206792_x_at	phosphodiesteras	PDE4C	10.25	10.23	10.20
205117_at	fibroblast growth	FGF1	6.63	6.88	6.80
211139_s_at	NGFI-A binding pr	NAB1	7.78	7.78	7.67
210842_at	neuropilin 2	NRP2	4.74	4.20	4.27
219638_at	F-box protein 22	FBXO22	5.90	5.91	5.98
221653_x_at	apolipoprotein L,	APOL2	7.60	7.68	7.56
210751_s_at	regucalcin (senesc	RGN	4.44	3.96	3.80

219113_x_at	hydroxysteroid (1	HSD17B14	6.00	6.17	6.02
207290_at	plexin A2	PLXNA2	4.06	4.19	3.87
212336_at	erythrocyte meml	EPB41L1	5.03	4.72	4.85
203901_at	TGF-beta activate	TAB1	6.18	6.25	6.17
205592_at	solute carrier fam	SLC4A1	5.93	5.89	5.63
217611_at	Glutamate-rich 1	ERICH1	4.80	4.53	4.66
210181_s_at	calcium binding pı	CABP1	4.69	4.34	4.64
210198_s_at	proteolipid protei	PLP1	3.34	3.34	3.45
204073_s_at	chromosome 11 c	C11orf9	6.46	6.32	5.88
204110_at	histamine N-meth	HNMT	4.34	4.24	4.17
216041_x_at	granulin	GRN	9.10	9.06	9.00
213072_at	cysteine/histidine	CYHR1	6.82	6.58	6.54
210410_s_at	chromosome 6 op	C6orf26 /// MSH5	7.75	7.73	7.79
222014_x_at	mitochondrial trai	MTO1	9.54	9.53	9.74
214256_at	ATPase, class V, ty	ATP10A	5.05	5.23	5.05
201305_x_at	acidic (leucine-ricl	ANP32B	13.09	13.03	13.13
205115_s_at	RNA binding moti	RBM19	7.57	7.51	7.78
216555_at	chromosome 22 c	C22orf30	6.45	6.28	6.30
218368_s_at	tumor necrosis fa	TNFRSF12A	11.47	11.40	11.51
207636_at	serpin peptidase i	SERPINI2	3.57	3.55	3.65
217289_s_at	solute carrier fam	SLC37A4	7.73	7.68	7.61
214461_at	lipopolysaccharide	LBP	4.11	3.96	4.16
219930_at	Kruppel-like facto	KLF8	4.79	5.03	4.82
202828_s_at	matrix metallopep	MMP14	7.79	7.55	7.28
205903_s_at	potassium interm	KCNN3	4.24	4.50	4.55
214906_x_at	NEDD4 binding pr	N4BP2L1	4.99	5.16	4.95
212484_at	family with seque	FAM89B	7.94	7.73	7.70
217534_at	family with seque	FAM49B	3.35	3.73	3.56
211876_x_at	protocadherin gar	PCDHGA10 /// PCI	4.89	4.85	4.62
214178_s_at	SRY (sex determin	SOX2	4.72	4.45	4.29
221236_s_at	stathmin-like 4	STMN4	5.21	5.07	4.99
209373_at	mal, T-cell differe	MALL	5.65	5.52	5.69
204910_s_at	tripartite motif-co	TRIM3	5.32	5.05	5.11
220323_at	cyclin N-terminal	CNTD2	7.62	7.67	7.38
206962_x_at	---	---	3.60	3.67	3.32
207804_s_at	ficolin (collagen/fi	FCN2	5.07	4.76	4.76
220320_at	docking protein 3	DOK3	6.10	6.18	5.83
202587_s_at	adenylate kinase :	AK1	8.54	8.67	8.68
206847_s_at	homeobox A7	HOXA7	4.64	4.41	4.56
204890_s_at	lymphocyte-speci	LCK	4.81	4.95	4.78
206850_at	RAS-like, family 1(	RASL10A	6.10	5.97	5.62
217267_s_at	RAB7A, member F	RAB7A	3.21	3.25	3.26
218254_s_at	SAR1 homolog B (	SAR1B	9.71	9.71	9.83
219508_at	glucosaminyl (N-a	GCNT3	6.02	5.91	5.81
212006_at	UBX domain prote	UBXN4	10.22	10.38	10.29
218152_at	high-mobility grou	HMG20A	9.68	9.67	9.86
214879_x_at	upstream transcri	USF2	8.35	8.22	8.10

216590_at	guanine nucleotid	GNAT3	3.65	3.50	3.35
205090_s_at	N-acetylglucosam	NAGPA	7.48	7.69	7.55
210741_at	Rho guanine nucle	ARHGEF12	4.17	4.02	3.93
220903_at	G elongation facto	GFM1	4.92	4.83	4.67
219670_at	BEN domain conta	BEND5	4.34	4.53	3.86
221205_at	---	---	3.41	3.70	3.79
204191_at	interferon (alpha,	IFNAR1	6.74	6.65	6.68
202764_at	stromal interactio	STIM1	7.45	7.22	7.24
202896_s_at	signal-regulatory	SIRPA	8.04	7.84	7.69
209464_at	aurora kinase B	AURKB	8.38	8.29	8.44
204963_at	sarcospan (Kras or	SSPN	3.74	3.46	3.28
202380_s_at	natural killer-tum	NKTR	7.98	8.15	8.06
206473_at	membrane-bound	MBTPS2	5.85	6.19	5.69
209318_x_at	pleiomorphic ade	PLAGL1	8.58	8.77	8.83
32062_at	ecto-NOX disulfid	ENOX2	8.08	8.13	8.33
207953_at	---	---	6.40	6.15	6.24
220245_at	solute carrier fam	SLC45A2	6.43	6.33	6.25
212648_at	DEAH (Asp-Glu-Ala	DHX29	9.94	10.16	10.10
201482_at	quiescin Q6 sulfhy	QSOX1	8.91	8.79	8.89
207380_x_at	NADPH oxidase 1	NOX1	5.32	5.22	4.98
205895_s_at	nucleolar and coil	NOLC1	9.07	9.05	9.05
205766_at	titin-cap (telethor	TCAP	6.82	6.64	6.42
204140_at	tyrosylprotein sul	TPST1	6.30	6.61	6.93
215871_at	phospholipase A2	PLA2G5	4.45	4.52	4.50
202099_s_at	DiGeorge syndron	DGCR2	7.49	7.66	7.42
33197_at	myosin VIIA	MYO7A	5.01	4.83	4.80
213207_s_at	golgi SNAP recept	GOSR2	7.36	7.18	7.28
219560_at	chromosome 22 c	C22orf29	8.11	7.89	8.14
208072_s_at	diacylglycerol kina	DGKD	8.80	8.78	8.70
201888_s_at	interleukin 13 rec	IL13RA1	6.56	6.67	6.66
221652_s_at	chromosome 12 c	C12orf11	11.31	11.29	11.42
213652_at	Proprotein conve	PCSK5	3.18	3.30	3.31
218494_s_at	SLC2A4 regulator	SLC2A4RG	8.55	8.52	8.52
200865_at	eukaryotic transla	EIF3F	3.60	3.47	3.44
204445_s_at	arachidonate 5-lip	ALOX5	4.27	4.07	4.29
214732_at	Sp1 transcription	SP1	4.40	4.37	4.55
208329_at	prostate and brea	PBOV1	3.30	3.26	3.33
200716_x_at	ribosomal protein	RPL13A	14.90	14.89	14.92
217960_s_at	translocase of out	TOMM22	10.43	10.40	10.38
211052_s_at	tubulin folding co	TBCD	8.76	8.69	8.75
201689_s_at	tumor protein D5	TPD52	9.59	9.57	9.73
203064_s_at	forkhead box K2	FOXK2	8.70	8.60	8.54
217622_at	---	---	7.59	7.62	7.41
220546_at	myeloid/lymphoi	MLL	4.76	4.76	4.57
211406_at	immediate early r	IER3IP1	7.98	8.02	8.00
210481_s_at	C-type lectin dom	CLEC4M	5.22	5.26	5.15
221901_at	KIAA1644	KIAA1644	8.32	8.34	8.44

206705_at	tubby like protein	TULP1		6.20	5.91	5.87
217666_at	---	---		4.02	4.03	3.97
216348_at	ribosomal protein	RPS17P5		10.72	10.66	10.81
210844_x_at	catenin (cadherin-	CTNNA1		11.81	11.78	11.75
208533_at	SRY (sex determin	SOX1		6.11	5.66	5.60
213999_at	Yip1 domain famil	YIPF4		6.11	5.86	5.83
212416_at	secretory carrier r	SCAMP1		10.38	10.43	10.57
201646_at	scavenger recepto	SCARB2		7.56	7.47	7.52
211089_s_at	NIMA (never in m	NEK3		6.54	6.34	6.62
221289_at	distal-less homeol	DLX6		4.35	4.30	4.32
206330_s_at	SHC (Src homolog	SHC3		5.33	5.34	5.41
210578_at	tripartite motif-co	TRIM10		4.14	4.19	4.13
212393_at	SET binding factor	SBF1		6.93	6.93	6.86
222097_at	---	---		5.24	5.44	5.40
213151_s_at	septin 7		7-Sep	11.99	12.13	12.15
213219_at	adenylate cyclase	ADCY2		3.72	3.68	3.93
216318_at	Immunoglobulin h	IGHA1		6.60	6.58	6.65
211260_at	bone morphogene	BMP7		7.52	7.43	7.44
214951_at	solute carrier fam	SLC26A10		4.91	4.78	4.57
220724_at	cell wall biogenes	CWH43		3.45	3.39	3.40
212998_x_at	major histocompa	HLA-DQB1 /// LOC		4.66	4.53	4.35
200796_s_at	myeloid cell leuke	MCL1		6.23	6.24	6.25
215777_at	immunoglobulin la	IGLV4-60		5.60	5.77	5.49
207494_s_at	zinc finger protein	ZNF76		8.32	8.30	8.11
211680_at	PDZ and LIM dom	PDLIM5		3.40	3.25	3.32
212580_at	Endoplasmic retic	ERAP1		5.69	5.93	5.87
212089_at	lamin A/C	LMNA		9.14	9.13	9.07
217971_at	MAPK scaffold pro	MAPKSP1		9.90	10.06	10.09
201157_s_at	N-myristoyltransfe	NMT1		9.47	9.39	9.46
208941_s_at	selenophosphate	SEPHS1		9.62	9.50	9.57
221051_s_at	integrin beta 1 bir	ITGB1BP3		6.25	6.31	6.38
214689_at	pappalysin 2	PAPPA2		6.91	6.71	6.50
217703_x_at	---	---		8.53	8.64	8.58
203052_at	complement com	C2		6.52	6.52	6.73
218421_at	ceramide kinase	CERK		8.37	8.60	8.56
201730_s_at	translocated pron	TPR		10.35	10.54	10.59
209346_s_at	phosphatidylinosi	PI4K2A		7.17	7.01	6.91
215833_s_at	signal peptide pep	SPPL2B		5.98	6.15	6.10
200926_at	ribosomal protein	RPS23		15.08	15.08	15.08
217033_x_at	neurotrophic tyro	NTRK3		5.88	5.67	5.73
214559_at	dopamine recepto	DRD3		3.91	3.96	3.81
209808_x_at	inhibitor of growt	ING1		7.35	7.29	7.28
203125_x_at	solute carrier fam	SLC11A2		7.58	7.40	7.11
212572_at	serine/threonine k	STK38L		8.81	8.95	9.06
204763_s_at	guanine nucleotid	GNAO1		5.41	5.09	5.25
208901_s_at	topoisomerase (D	TOP1		11.24	11.33	11.35
205855_at	zinc finger protein	ZNF197		6.27	6.21	6.29

214275_at	Mediator comple›	MED12	4.77	4.73	4.60
215852_x_at	chromosome 20 c	C20orf117	5.81	6.03	5.67
214309_s_at	---	---	4.92	5.06	5.02
208478_s_at	BCL2-associated X	BAX	7.83	7.65	7.74
212277_at	myotubularin rela	MTMR4	9.43	9.52	9.54
205167_s_at	cell division cycle	CDC25C	8.47	8.53	8.46
212621_at	transmembrane p	TMEM194A	9.52	9.36	9.47
219971_at	interleukin 21 rec	IL21R	5.43	5.27	5.41
221627_at	tripartite motif-co	TRIM10	5.55	5.46	5.09
211132_at	integrator comple	INTS3	4.98	4.83	4.80
212211_at	ankyrin repeat do	ANKRD17	8.30	8.35	8.66
209315_at	HBS1-like (S. cere	HBS1L	5.79	5.90	5.72
218697_at	NCK interacting p	NCKIPSD	7.74	7.74	7.89
203388_at	arrestin, beta 2	ARRB2	6.51	6.52	6.43
204211_x_at	eukaryotic transla	EIF2AK2	8.60	8.61	8.74
206726_at	hematopoietic pr	HPGDS	4.58	4.48	4.07
200614_at	clathrin, heavy ch	CLTC	13.31	13.34	13.24
205175_s_at	ketohexokinase (f	KHK	5.89	6.01	5.68
221664_s_at	F11 receptor	F11R	5.89	5.89	5.73
221119_at	Rho guanine nucle	ARHGEF38	4.45	4.48	4.13
200016_x_at	heterogeneous nu	HNRNPA1	14.28	14.27	14.30
202731_at	programmed cell	PDCD4	9.11	8.81	9.28
216727_at	Serine/threonine	STK38	4.65	4.76	4.72
203543_s_at	Kruppel-like facto	KLF9	10.70	10.64	10.97
220461_at	pecanex-like 2 (Dr	PCNXL2	3.84	3.91	4.03
214898_x_at	mucin 3B, cell sur	MUC3B	7.41	7.21	7.31
217940_s_at	carbohydrate kina	CARKD	9.87	9.97	9.92
214481_at	histone cluster 1,	HIST1H2AM	5.43	5.40	5.29
216573_at	immunoglobulin l	IGLV1-44 /// LOC1	3.43	3.52	3.21
208103_s_at	acidic (leucine-ricl	ANP32E	8.87	8.94	9.07
203571_s_at	chromosome 10 c	C10orf116	5.18	5.15	5.02
215079_at	---	---	3.62	3.81	3.67
207235_s_at	glutamate recept	GRM5	4.93	4.93	4.87
217676_at	---	---	5.86	5.48	5.52
201897_s_at	CDC28 protein kin	CKS1B	12.37	12.35	12.45
216266_s_at	ADP-ribosylation f	ARFGEF1	6.85	6.91	6.91
216325_x_at	regulator of telom	RTEL1	6.23	6.14	6.29
216045_at	coiled-coil domair	CCDC144A	4.30	4.43	4.23
222181_at	CCR4-NOT transcr	CNOT2	5.07	5.00	5.14
208229_at	fibroblast growth	FGFR2	5.25	5.47	5.52
215056_at	---	---	4.31	4.41	4.11
219968_at	zinc finger protein	ZNF589	7.40	7.48	7.34
208869_s_at	GABA(A) receptor	GABARAPL1	5.65	5.35	5.58
201733_at	chloride channel 3	CLCN3	5.96	5.87	6.02
215600_x_at	F-box and WD rep	FBXW12	8.31	8.32	8.41
217555_at	structural mainter	SMC1A	5.50	5.74	5.65
215943_at	KIAA1661 protein	KIAA1661	4.07	3.96	3.76

220782_x_at	kallikrein-related	KLK12	6.74	6.70	6.62
205025_at	zinc finger and BT	ZBTB48	6.78	6.58	6.64
207722_s_at	BTB (POZ) domair	BTBD2	5.86	5.70	5.78
218435_at	DnaJ (Hsp40) hom	DNAJC15	11.70	11.78	11.81
210360_s_at	metastasis suppre	MTSS1	4.05	4.04	3.75
217221_x_at	RNA binding moti	RBM10	9.03	8.91	9.02
200023_s_at	eukaryotic transla	EIF3F	13.50	13.41	13.45
36612_at	family with seque	FAM168A	6.81	6.82	6.83
220409_at	calmodulin regula	CAMSAP1	4.33	4.16	4.39
205252_at	zinc finger proteir	ZNF174	6.42	6.62	6.41
222327_x_at	olfactory receptor	OR7E156P	5.93	5.99	6.04
216686_at	hypothetical LOC6	FLJ40330	3.59	3.45	3.46
211028_s_at	ketohexokinase (f	KHK	7.78	7.64	7.59
201217_x_at	ribosomal protein	RPL3	14.62	14.61	14.68
202863_at	fumarylacetoacet	FAH	9.68	9.66	9.97
39582_at	cylindromatosis (t	CYLD	7.89	7.89	7.86
213129_s_at	glycine cleavage s	GCSH /// LOC1003	11.18	11.23	11.24
214715_x_at	zinc finger proteir	ZNF160	9.46	9.57	9.75
222045_s_at	PDX1 C-terminal i	PCIF1	6.16	6.52	6.19
205217_at	translocase of inn	TIMM8A	8.88	8.69	8.97
211208_s_at	calcium/calmodul	CASK	8.66	8.57	8.63
203109_at	ubiquitin-conjuga	UBE2M	10.09	10.05	10.12
208394_x_at	endothelial cell-sp	ESM1	3.69	3.40	3.50
204504_s_at	HIRA interacting p	HIRIP3	9.14	9.01	9.14
202774_s_at	splicing factor, arg	SFRS8	9.31	9.27	9.30
219237_s_at	DnaJ (Hsp40) hom	DNAJB14	8.74	9.00	9.03
209134_s_at	ribosomal protein	RPS6	14.74	14.72	14.74
212078_s_at	myeloid/lymphoic	MLL	8.65	8.62	8.49
219505_at	cat eye syndrome	CECR1	5.03	5.05	5.17
202853_s_at	alpha- and gamma	AAGAB	10.28	10.29	10.52
217770_at	phosphatidylinosi	PIGT	9.83	9.77	9.85
216492_at	killer cell immuno	KIR3DX1	5.86	5.77	5.69
205692_s_at	CD38 molecule	CD38	6.09	6.12	5.93
216980_s_at	sialophorin	SPN	6.61	6.13	6.56
205792_at	WNT1 inducible si	WISP2	7.56	7.49	7.76
205201_at	GLI family zinc fin	GLI3	8.94	8.78	8.77
219619_at	DIRAS family, GTP	DIRAS2	3.62	3.56	3.18
202492_at	ATG9 autophagy r	ATG9A	8.83	8.76	8.69
220974_x_at	sideroflexin 3	SFXN3	8.90	8.95	8.88
215099_s_at	retinoid X recepto	RXRB	5.29	5.39	5.26
220953_s_at	myotubularin rela	MTMR12	5.78	5.84	6.00
214824_at	---	---	6.37	6.22	5.94
220729_at	---	---	3.48	3.47	3.59
218519_at	solute carrier fam	SLC35A5	8.92	9.05	9.13
219349_s_at	exocyst complex c	EXOC2	7.61	7.48	7.65
212598_at	WD repeat and FY	WDFY3	5.36	5.07	5.15
220303_at	PDZ domain conta	PDZD3	6.93	6.67	6.44

210756_s_at	Notch homolog 2	NOTCH2	7.85	7.83	8.08
201907_x_at	dishevelled, dsh h	DVL3	6.55	6.47	6.17
215846_at	---	---	4.01	4.03	3.84
204157_s_at	SIK family kinase 3	SIK3	6.80	6.89	7.08
202276_at	split hand/foot ma	SHFM1	10.97	11.15	11.05
209425_at	alpha-methylacyl-	AMACR /// C1QTN	6.17	6.46	6.45
219382_at	SERTA domain cor	SERTAD3	6.74	6.87	6.89
205716_at	solute carrier fam	SLC25A40	5.45	5.17	5.41
217466_x_at	ribosomal protein	RPS2	12.45	12.40	12.46
41329_at	SCY1-like 3 (S. cer	SCYL3	7.49	7.59	7.55
213619_at	Heterogeneous nu	HNRNPH1	12.06	12.18	12.20
213021_at	golgi SNAP recept	GOSR1	8.68	8.79	8.75
207622_s_at	ATP-binding casse	ABCF2	8.01	8.06	8.06
221979_at	similar to hCG181	LOC100129250	8.17	8.43	8.31
215983_s_at	UBX domain prote	UBXN8	9.08	9.19	9.28
219822_at	mitochondrial trai	MTRF1	6.59	6.75	6.64
215011_at	small nucleolar RN	SNHG3	7.76	8.03	7.99
217029_at	---	---	4.99	5.14	5.04
203197_s_at	chromosome 1 op	C1orf123	9.35	9.40	9.42
211541_s_at	dual-specificity ty	DYRK1A	5.88	6.01	5.69
209282_at	protein kinase D2	PRKD2	7.18	7.00	7.05
65635_at	endo-beta-N-acet	ENGASE	8.07	8.11	8.01
214101_s_at	Aminopeptidase p	NPEPPS	6.67	6.87	7.05
208407_s_at	catenin (cadherin-	CTNND1	9.10	9.17	9.20
205893_at	neuroligin 1	NLGN1	3.17	2.99	3.06
204628_s_at	integrin, beta 3 (p	ITGB3	6.21	6.15	6.33
216362_at	---	---	4.84	4.47	4.63
219678_x_at	DNA cross-link rep	DCLRE1C	8.03	8.24	8.30
208852_s_at	calnexin	CANX	10.97	11.03	11.15
215245_x_at	fragile X mental re	FMR1	10.32	10.47	10.60
209162_s_at	PRP4 pre-mRNA p	PRPF4	9.53	9.57	9.62
32811_at	myosin IC	MYO1C	7.48	7.67	7.41
210486_at	ankyrin repeat an	ANKMY1	7.40	7.34	7.16
217449_at	---	---	4.57	4.73	4.53
212408_at	torsin A interactin	TOR1AIP1	10.26	10.27	10.32
215784_at	CD1e molecule	CD1E	3.55	3.74	3.50
202005_at	suppression of tur	ST14	6.53	6.22	5.97
220800_s_at	tropomodulin 3 (u	TMOD3	6.81	7.04	7.13
210766_s_at	CSE1 chromosome	CSE1L	12.21	12.30	12.26
209322_s_at	SH2B adaptor pro	SH2B1	7.43	7.17	7.09
205356_at	ubiquitin specific	USP13	9.42	9.51	9.54
200004_at	eukaryotic transla	EIF4G2	13.28	13.37	13.37
205118_at	formyl peptide re	FPR1	3.89	3.82	3.84
213064_at	zinc finger CCCH-t	ZC3H14	9.34	9.38	9.42
204186_s_at	peptidylprolyl isor	PPID	9.57	9.79	9.75
214658_at	transmembrane e	TMED7	9.28	9.41	9.55
202885_s_at	protein phosphata	PPP2R1B	4.67	4.89	4.83

212214_at	optic atrophy 1 (a	OPA1	8.92	9.02	9.03
220531_at	---	---	4.98	5.16	4.92
205794_s_at	neuro-oncological	NOVA1	6.31	6.29	6.33
219188_s_at	MACRO domain c	MACROD1	8.09	7.83	8.01
206797_at	N-acetyltransferas	NAT2	4.19	4.35	4.28
214055_x_at	HLA-B associated	BAT2L2	10.47	10.50	10.50
210231_x_at	SET nuclear oncog	SET	12.73	12.80	12.77
216499_at	---	---	5.50	5.74	5.35
208813_at	glutamic-oxaloace	GOT1	9.25	9.26	9.34
217326_x_at	Interleukin 23, alp	IL23A /// TRBV19	6.12	6.10	6.10
206173_x_at	GA binding protei	GABPB1	8.36	8.21	8.36
221525_at	zinc finger, MIZ-ty	ZMIZ2	6.75	6.85	6.94
215656_at	Lectin, mannose-b	LMAN2	3.94	3.76	3.72
201866_s_at	nuclear receptor s	NR3C1	9.02	8.84	9.15
216833_x_at	glycophorin B (MN	GYPB	6.05	5.68	5.59
200039_s_at	proteasome (pros	PSMB2	12.13	12.14	12.15
201284_s_at	N-acylaminoacyl-p	APEH	9.38	9.31	9.41
205961_s_at	PC4 and SFRS1 int	PSIP1	9.31	9.51	9.51
220492_s_at	otoferlin	OTOF	4.04	4.10	3.98
201026_at	eukaryotic transla	EIF5B	7.62	8.03	7.84
218773_s_at	methionine sulfox	MSRB2	9.86	9.86	9.76
209956_s_at	calcium/calmodul	CAMK2B	3.71	3.33	3.41
205845_at	calcium channel, v	CACNA1H	7.22	7.05	6.82
216094_at	---	---	5.68	5.82	5.75
202282_at	hydroxysteroid (1	HSD17B10	11.18	11.14	11.26
209979_at	adenosine deamir	ADARB1	7.49	7.20	7.22
207421_at	carbonic anhydras	CA5A	5.21	5.06	5.16
212736_at	chromosome 16 c	C16orf45	7.56	7.37	7.11
201933_at	chromatin modify	CHMP1A	8.61	8.57	8.42
217250_s_at	chromodomain he	CHD5	3.85	3.61	3.74
201756_at	replication protei	RPA2	8.23	8.29	8.26
204199_at	Ral GEF with PH d	RALGPS1	4.16	4.30	3.89
208443_x_at	short stature hom	SHOX2	6.85	7.08	6.83
219062_s_at	zinc finger, CCHC	ZCCHC2	8.11	8.05	8.04
214809_at	---	---	5.32	5.46	5.36
205171_at	protein tyrosine p	PTPN4	7.57	7.76	7.81
220893_at	uncharacterized g	LOC57399	3.87	3.79	3.61
221656_s_at	Rho guanine nucle	ARHGEF10L	8.26	8.21	8.11
210906_x_at	aquaporin 4	AQP4	3.47	3.30	3.24
217145_at	immunoglobulin k	IGK@ /// IGKC ///	3.61	3.43	3.25
207907_at	tumor necrosis fa	TNFSF14	5.44	5.20	5.22
203876_s_at	matrix metallopep	MMP11	5.63	5.48	5.66
222031_at	hypothetical prote	LOC286434 /// LO	3.34	3.54	3.60
202052_s_at	retinoic acid induc	RAI14	10.29	10.32	10.40
212394_at	KIAA0090	KIAA0090	7.05	6.96	6.96
216953_s_at	Wilms tumor 1	WT1	5.10	4.67	4.50
208710_s_at	adaptor-related p	AP3D1	10.65	10.58	10.53

220262_s_at	delta-like 2 homol	DLK2	8.60	8.63	8.46
220653_at	zinc finger, imprin	ZIM2	6.07	5.99	5.79
221641_s_at	acyl-CoA thioester	ACOT9	9.63	9.75	9.83
210943_s_at	lysosomal trafficki	LYST	6.78	6.82	6.47
212564_at	potassium channe	KCTD2	8.02	8.02	7.94
217659_at	---	---	5.77	5.67	5.43
208476_s_at	FERM domain con	FRMD4A	7.17	7.14	7.08
206458_s_at	wingless-type MM	WNT2B	4.00	3.73	3.69
213903_s_at	RCD1 required for	RQCD1	5.15	4.96	5.16
213973_at	ribosome binding	RRBP1	5.49	5.42	5.37
213118_at	UHRF1 binding pr	UHRF1BP1L	8.10	8.14	8.55
222184_at	---	---	4.27	4.53	4.18
219445_at	glioma tumor sup	GLTSCR1	7.48	7.37	7.15
211543_s_at	G protein-couplec	GRK6	7.43	7.45	7.49
216582_at	POM121 membra	POM121L2	5.51	5.54	5.58
212293_at	homeodomain int	HIPK1	9.93	9.90	10.20
215874_at	---	---	5.15	5.03	4.97
212209_at	mediator complex	MED13L	9.39	9.10	9.45
215344_at	---	---	4.65	4.57	4.43
217021_at	cytochrome b5 ty	CYB5A	4.12	4.01	4.18
203089_s_at	HtrA serine peptic	HTRA2	9.48	9.44	9.54
205168_at	discoidin domain	DDR2	5.50	5.16	5.41
213344_s_at	H2A histone famil	H2AFX	7.94	8.26	8.15
201293_x_at	peptidylprolyl isor	PPIA	14.41	14.41	14.43
204694_at	alpha-fetoprotein	AFP	6.28	6.48	6.57
219536_s_at	zinc finger protein	ZFP64	6.96	6.91	6.64
209463_s_at	TAF12 RNA polym	TAF12	9.22	9.29	9.27
205177_at	troponin I type 1 (	TNNI1	5.91	5.48	5.74
206823_at	l(3)mbt-like (Dros	L3MBTL	7.32	6.95	7.06
212354_at	sulfatase 1	SULF1	5.64	5.60	5.48
211911_x_at	major histocompa	HLA-B	11.03	10.87	10.99
204501_at	nephroblastoma c	NOV	5.88	5.87	6.24
219006_at	NADH dehydroger	NDUFAF4	10.38	10.34	10.48
207053_at	solute carrier fam	SLC8A1	4.14	4.03	3.73
201826_s_at	saccharopine deh	SCCPDH	8.28	8.29	8.41
205380_at	PDZ domain conta	PDZK1	3.85	3.85	3.85
209571_at	corepressor intera	CIR1	7.36	7.50	7.58
207365_x_at	ubiquitin specific	USP34	8.88	9.02	8.75
201392_s_at	insulin-like growth	IGF2R	9.27	9.27	9.34
206264_at	glycosylphosphati	GPLD1	3.95	4.20	3.78
209797_at	canopy 2 homolog	CNPY2	9.63	9.78	9.78
41386_i_at	lysine (K)-specific	KDM6B	7.30	7.26	7.40
213403_at	---	---	5.10	5.61	5.18
221175_at	chromosome 3 op	C3orf36	5.06	4.90	4.88
217136_at	peptidylprolyl isor	PPIAL4A	4.23	4.11	4.43
60815_at	polymerase (RNA)	POLR2J4	4.71	4.67	4.39
219306_at	kinesin family mei	KIF15	8.74	8.92	9.22

202292_x_at	lysophospholipase	LYPLA2	8.51	8.67	8.56
219970_at	GIPC PDZ domain	GIPC2	3.26	3.32	3.30
206540_at	galactosidase, bet	GLB1L	5.24	5.09	4.95
207537_at	6-phosphofructo-2	PFKFB1	5.54	5.79	5.67
201199_s_at	proteasome (pros	PSMD1	11.70	11.73	11.64
216283_s_at	poliovirus recepto	PVR	6.87	7.01	6.79
218799_at	GPN-loop GTPase	GPN2	8.00	8.12	8.02
215123_at	similar to acyl-CoA	LOC100288332 ///	8.07	8.14	7.92
218762_at	zinc finger protein	ZNF574	7.48	7.33	7.39
203696_s_at	replication factor	RFC2	9.42	9.45	9.60
221225_at	dephospho-CoA k	DCAKD	4.64	4.65	4.65
221346_at	olfactory receptor	OR10J1	6.04	6.22	6.11
215190_at	eukaryotic transla	EIF3M	7.19	7.41	7.32
206236_at	G protein-coupled	GPR4	6.33	6.03	5.66
201395_at	RNA binding moti	RBM5	10.41	10.43	10.29
220504_at	keratocan	KERA	3.26	3.52	3.20
209221_s_at	oxysterol binding	OSBPL2	6.75	6.92	6.77
206626_x_at	synovial sarcoma,	SSX1	3.58	3.87	3.85
216526_x_at	major histocompa	HLA-C	11.28	11.16	11.27
210894_s_at	centrosomal prote	CEP250	6.65	6.32	6.48
200737_at	phosphoglycerate	PGK1	11.82	11.68	11.87
205585_at	ets variant 6	ETV6	6.03	5.77	5.36
217144_at	ubiquitin B	UBB	7.01	7.09	7.22
218012_at	TSPY-like 2	TSPYL2	7.97	8.24	8.03
211500_at	mitogen-activated	MAPK11	7.74	7.55	7.52
218419_s_at	transmembrane a	TMUB2	7.31	7.26	7.44
219496_at	ankyrin repeat do	ANKRD57	9.00	8.97	9.18
205374_at	sarcolipin	SLN	5.28	5.60	5.21
206727_at	complement com	C9	3.61	3.44	3.46
200886_s_at	phosphoglycerate	PGAM1	12.95	13.01	13.05
203832_at	small nuclear ribo	SNRPF	11.93	12.01	11.96
218403_at	TP53 regulated inl	TRIAP1	10.79	10.71	10.78
218787_x_at	CWF19-like 1, cell	CWF19L1	7.96	8.01	8.07
200009_at	GDP dissociation i	GDI2	12.85	12.94	12.96
208515_at	histone cluster 1,	HIST1H2BM	4.42	4.26	4.27
206304_at	myosin binding pr	MYBPH	6.60	6.63	6.43
216695_s_at	tankyrase, TRF1-ir	TNKS	5.13	4.83	4.74
206258_at	ST8 alpha-N-acety	ST8SIA5	5.18	5.25	5.37
210343_s_at	solute carrier fam	SLC22A6	6.22	6.09	6.26
214285_at	fatty acid binding	FABP3	4.15	4.26	4.37
218380_at	hypothetical prote	LOC728392 /// NL	5.21	5.12	5.13
208510_s_at	peroxisome prolif	PPARG	8.81	8.76	8.84
207776_s_at	calcium channel, v	CACNB2	5.82	5.96	5.70
219105_x_at	origin recognition	ORC6L	10.61	10.63	10.71
206008_at	transglutaminase	TGM1	6.61	6.47	6.48
200612_s_at	adaptor-related p	AP2B1	9.51	9.47	9.49
214000_s_at	Regulator of G-pro	RGS10	5.30	5.55	5.35

208403_x_at	MYC associated fa	MAX	7.54	7.53	7.36
201571_s_at	dCMP deaminase	DCTD	8.68	8.63	8.75
220522_at	crumbs homolog 1	CRB1	4.35	4.66	4.25
214643_x_at	bridging integrato	BIN1	6.91	6.98	6.83
207071_s_at	aconitase 1, solub	ACO1	10.09	10.06	10.06
212035_s_at	exocyst complex c	EXOC7	6.84	6.72	6.50
215989_at	chromobox homo	CBX2	6.23	6.31	6.19
201268_at	NME1-NME2 read	NME1-NME2 /// N	13.68	13.60	13.68
218096_at	1-acylglycerol-3-p	AGPAT5	11.35	11.35	11.46
220595_at	PDZ domain conta	PDZRN4	3.56	3.33	3.24
208144_s_at	---	---	4.81	4.92	4.78
221562_s_at	sirtuin (silent mat	SIRT3	7.05	6.94	7.09
215239_x_at	zinc finger protein	ZNF273	4.28	4.33	4.14
217255_at	sequestosome 1	SQSTM1	5.19	5.24	5.18
217314_at	---	---	6.27	6.27	6.11
219558_at	ATPase type 13A3	ATP13A3	7.05	7.12	7.00
209030_s_at	cell adhesion mol	CADM1	9.62	9.29	9.71
209835_x_at	CD44 molecule (Ir	CD44	10.18	10.08	10.24
220219_s_at	similar to c114 SL	LOC100294335 ///	7.09	7.10	6.90
209431_s_at	POZ (BTB) and AT	PATZ1	8.05	7.81	7.86
41397_at	zinc finger protein	ZNF821	6.64	6.64	6.49
215331_at	myosin, heavy cha	MYH15	3.68	3.50	3.53
219124_at	chromosome 8 op	C8orf41	8.12	8.13	8.23
209528_s_at	TEL2, telomere m	TELO2	7.93	7.79	7.85
217968_at	tumor suppressing	TSSC1	9.36	9.39	9.46
205523_at	hyaluronan and pr	HAPLN1	3.57	3.29	3.45
216774_at	---	---	4.83	5.01	4.58
204893_s_at	zinc finger, FYVE c	ZFYVE9	7.57	7.27	7.29
218527_at	aprataxin	APTX	9.99	10.14	10.08
219510_at	polymerase (DNA	POLQ	9.02	9.29	9.25
209047_at	aquaporin 1 (Colto	AQP1	5.00	4.90	4.71
213297_at	required for meio	RMND5B	8.04	8.06	7.89
202815_s_at	hexamethylene bi	HEXIM1	7.47	7.73	7.60
214859_at	follistatin-like 4	FSTL4	4.66	4.88	4.76
201868_s_at	transducin (beta)-	TBL1X	7.16	7.00	7.07
209917_s_at	TP53 target 1 (nor	TP53TG1	7.06	7.12	7.47
215336_at	A kinase (PRKA) a	AKAP11	5.97	5.96	5.87
208494_at	solute carrier fam	SLC6A7	6.86	6.77	6.67
220482_s_at	secretion regulati	SERGEF	6.63	6.47	6.43
214775_at	Nedd4 binding pro	N4BP3	4.05	4.01	4.26
204393_s_at	acid phosphatase,	ACPP	3.65	3.82	3.67
214619_at	corticotropin rele	CRHR1	6.70	6.58	6.43
202988_s_at	regulator of G-pro	RGS1	4.10	4.04	4.02
200746_s_at	guanine nucleotid	GNB1	11.00	10.97	10.97
216457_s_at	splicing factor 3a,	SF3A1	9.77	9.84	9.85
221165_s_at	interleukin 22	IL22	4.78	4.72	4.42
216053_x_at	Family with seque	FAM182A	5.95	6.10	5.77

205549_at	Purkinje cell prote	PCP4	4.50	4.28	4.09
215130_s_at	IQ motif containir	IQCK	5.52	5.48	5.39
217101_at	tetratricopeptide	TTC39A	6.29	6.12	6.36
216193_at	---	---	5.15	5.06	5.01
221721_s_at	leucine zipper, pu	LZTS1	5.21	4.97	5.03
206086_x_at	hemochromatosis	HFE	6.14	6.14	6.09
221846_s_at	CASK interacting p	CASKIN2	7.69	7.67	7.54
220775_s_at	UEV and lactate/n	UEVLD	7.64	7.91	8.06
38037_at	heparin-binding E	HBEGF	7.32	7.50	7.48
44783_s_at	hairy/enhancer-of	HEY1	7.53	7.50	7.69
209810_at	surfactant protein	SFTPB	6.41	6.14	6.05
207156_at	histone cluster 1,	HIST1H2AG	5.83	5.83	5.94
40850_at	FK506 binding pro	FKBP8	5.98	5.87	5.85
203996_s_at	chromosome 21 c	C21orf2	7.91	7.74	7.66
209914_s_at	neurexin 1	NRXN1	6.48	6.01	6.14
202981_x_at	seven in absentia	SIAH1	10.23	10.17	10.28
210439_at	inducible T-cell co	ICOS	4.89	5.01	4.69
215588_x_at	RIO kinase 3 (yeas	RIOK3	8.93	9.00	8.83
219106_s_at	kelch repeat and E	KBTBD10	3.12	3.24	3.01
215085_x_at	deleted in lung an	DLEC1	5.57	5.61	5.54
218315_s_at	CDK5 regulatory s	CDK5RAP1	9.22	9.08	9.20
209771_x_at	CD24 molecule	CD24	6.79	6.76	6.71
202455_at	histone deacetyla	HDAC5	8.20	8.11	8.15
208883_at	ubiquitin protein l	UBR5	7.96	7.93	8.10
209363_s_at	mediator complex	MED21	7.88	8.03	7.95
208564_at	potassium voltage	KCNA2	4.69	4.59	4.99
221781_s_at	DnaJ (Hsp40) hom	DNAJC10	8.13	8.23	8.38
206019_at	RNA binding moti	RBM19	7.31	7.30	7.22
202496_at	enhancer of mRNA	EDC4	8.63	8.62	8.61
205081_at	cysteine-rich prot	CRIP1	5.28	4.94	4.82
220451_s_at	baculoviral IAP rep	BIRC7	6.42	6.04	6.17
211370_s_at	mitogen-activated	MAP2K5	7.61	7.74	7.84
215521_at	polyhomeotic hor	PHC3	6.42	6.05	6.13
217573_at	glutamate recepto	GRIN2C	7.35	7.18	7.08
200608_s_at	RAD21 homolog (S	RAD21	11.58	11.65	11.93
214279_s_at	NDRG family mem	NDRG2	3.93	4.07	4.19
212091_s_at	collagen, type VI,	COL6A1	5.31	5.22	5.22
203550_s_at	family with seque	FAM189B	9.74	9.68	9.69
214221_at	Alstrom syndrome	ALMS1	7.99	8.03	8.08
213337_s_at	---	---	6.42	6.46	6.31
208448_x_at	interferon, alpha 1	IFNA16	3.91	4.04	3.98
219788_at	paired immunoglo	PILRA	4.38	4.09	4.07
205125_at	phospholipase C, d	PLCD1	7.07	6.86	7.01
207354_at	chemokine (C-C m	CCL16	4.05	4.10	4.31
220900_at	---	---	3.43	3.12	3.58
209661_at	kinesin family mei	KIFC3	7.57	7.22	7.47
221267_s_at	family with seque	FAM108A1	9.39	9.32	9.38

214118_x_at	pericentriolar mat	PCM1	8.74	8.89	8.79
221532_s_at	WD repeat domai	WDR61	11.26	11.40	11.37
208998_at	uncoupling protei	UCP2	5.13	5.16	4.89
204513_s_at	engulfment and c	ELMO1	5.39	5.18	5.06
218192_at	inositol hexakisph	IP6K2	7.86	8.13	7.88
211171_s_at	phosphodiesteras	PDE10A	5.10	5.11	5.12
211264_at	glutamate decarb	GAD2	3.11	3.08	3.14
221570_s_at	methyltransferase	METTL5	12.33	12.39	12.45
212134_at	pleckstrin homolo	PHLDB1	7.99	7.79	7.70
212384_at	HLA-B associated	BAT1	6.61	6.40	6.76
205352_at	serpin peptidase i	SERPINI1	7.02	7.13	6.92
221975_s_at	Chromosome 21 c	C21orf2	6.06	5.81	5.98
221263_s_at	splicing factor 3b,	SF3B5	12.12	12.15	12.05
213396_s_at	A kinase (PRKA) a	AKAP10	7.47	7.63	7.59
204489_s_at	CD44 molecule (Ir	CD44	10.05	9.99	10.26
220766_at	B-cell translocatio	BTG4	4.72	4.81	4.52
37433_at	protein inhibitor c	PIAS2	5.45	5.64	5.64
216753_at	---	---	3.88	3.70	3.67
202398_at	adaptor-related p	AP3S2	6.41	6.15	6.54
212753_at	polycomb group r	PCGF3	7.85	8.11	7.99
204094_s_at	TSC22 domain fan	TSC22D2	9.83	9.89	10.14
205734_s_at	AF4/FMR2 family,	AFF3	5.45	5.54	5.37
65472_at	chromosome 2 op	C2orf68	6.99	7.08	7.33
216051_x_at	---	---	7.52	7.60	7.48
213318_s_at	HLA-B associated	BAT3	9.77	9.74	9.76
206784_at	aquaporin 8	AQP8	5.98	5.94	5.99
212123_at	tectonic family me	TCTN3	9.42	9.49	9.44
203249_at	enhancer of zeste	EZH1	7.44	7.42	7.51
201780_s_at	ring finger proteir	RNF13	9.15	9.20	9.50
208630_at	hydroxyacyl-CoA c	HADHA	10.96	10.83	10.90
217707_x_at	SWI/SNF related,	SMARCA2	8.77	8.92	9.12
204309_at	cytochrome P450,	CYP11A1	4.67	4.51	4.73
208868_s_at	GABA(A) receptor	GABARAPL1	7.39	7.07	7.15
207344_at	A kinase (PRKA) a	AKAP3	5.26	5.26	4.94
213698_at	zinc finger, MYM-	ZMYM6	9.51	9.49	9.53
219908_at	dickkopf homolog	DKK2	3.08	3.20	3.41
201528_at	replication proteir	RPA1	10.72	10.76	10.79
205455_at	macrophage stimu	MST1R	5.63	5.83	5.48
206136_at	frizzled homolog 5	FZD5	5.79	5.96	5.76
215673_at	thyrotrophic emb	TEF	3.29	3.54	3.40
220116_at	potassium interm	KCNN2	3.27	3.34	3.10
210564_x_at	CASP8 and FADD-	CFLAR	8.49	8.39	8.56
219311_at	centrosomal prote	CEP76	8.33	8.56	8.50
218693_at	tetraspanin 15	TSPAN15	4.34	4.13	4.29
200006_at	Parkinson disease	PARK7	13.16	13.16	13.28
220216_at	chromosome 8 op	C8orf44	5.61	5.35	5.26
215569_at	GTF2I repeat dom	GTF2IRD2 /// GTF2	6.05	5.75	5.95

213109_at	TRAF2 and NCK in	TNIK	4.34	4.22	3.90
221261_x_at	melanoma antiger	MAGED4 /// MAG	4.48	4.50	4.50
212969_x_at	echinoderm micro	EML3	6.78	6.76	6.48
214711_at	glutamyl-tRNA(Gl	GATC	7.57	7.50	7.89
216881_x_at	proline-rich prote	PRB4	6.37	6.11	5.85
203754_s_at	BRF1 homolog, su	BRF1	6.53	6.71	6.73
208906_at	Berardinelli-Seip c	BSCL2	8.31	8.13	8.24
210708_x_at	caspase 10, apopt	CASP10	6.19	6.57	6.30
211925_s_at	phospholipase C, l	PLCB1	4.43	4.35	4.23
219385_at	SLAM family mem	SLAMF8	5.84	5.87	6.02
204288_s_at	sorbin and SH3 dc	SORBS2	8.32	8.21	8.42
216901_s_at	IKAROS family zin	IKZF1	3.65	3.66	3.47
218709_s_at	intraflagellar trans	IFT52	8.71	8.82	8.80
218407_x_at	neuron derived ne	NENF	10.74	10.71	10.63
217578_at	---	---	3.76	3.79	3.66
AFFX-HUMISGF3	glyceraldehyde-3-	GAPDH	9.81	9.84	10.01
213577_at	squalene epoxidas	SQLE	9.98	10.15	10.07
221092_at	IKAROS family zin	IKZF3	3.37	3.38	3.16
221947_at	hypothetical prote	LOC100288640	6.88	7.02	6.63
212721_at	splicing factor, arg	SFRS12	10.90	10.97	11.13
218946_at	NFU1 iron-sulfur c	NFU1	9.91	9.99	10.02
210437_at	melanoma antiger	MAGEA9 /// MAG	4.41	4.18	4.08
212378_at	phosphoribosylgly	GART	10.34	10.17	10.42
205371_s_at	dihydrolipoamide	DBT	6.49	6.90	6.88
220550_at	F-box protein 4	FBXO4	4.20	3.86	4.10
220739_s_at	cyclin M3	CNNM3	7.23	7.28	7.07
214655_at	G protein-couplec	GPR6	4.79	4.95	4.63
214094_at	far upstream elem	FUBP1	5.50	5.76	5.51
203247_s_at	zinc finger protein	ZNF24	9.33	9.32	9.34
221569_at	Abelson helper int	AHI1	7.53	7.69	7.58
201962_s_at	ring finger protein	RNF41	7.59	7.62	7.72
207295_at	sodium channel, r	SCNN1G	5.09	5.16	4.83
207170_s_at	LETM1 domain co	LETMD1	9.78	9.80	9.91
212649_at	DEAH (Asp-Glu-Ala	DHX29	7.27	7.47	7.29
214169_at	---	---	6.52	6.76	7.06
207470_at	---	---	4.27	4.30	4.22
202553_s_at	SYF2 homolog, RN	SYF2	9.95	10.00	10.01
217524_x_at	---	---	3.11	3.15	3.21
217331_at	---	---	5.46	5.69	5.61
212643_at	mitogen-activatec	MAPK1IP1L	11.92	11.83	11.80
206083_at	brain-specific angi	BAI1	6.93	6.60	6.35
213260_at	forkhead box C1	FOXC1	8.60	8.43	8.37
205482_x_at	sorting nexin 15	SNX15	8.47	8.44	8.40
201732_s_at	chloride channel 3	CLCN3	8.16	8.13	8.27
205169_at	retinoblastoma bi	RBBP5	8.15	7.99	8.06
206871_at	elastase, neutropl	ELANE	4.52	4.23	4.11
210173_at	protein tyrosine p	PTPRJ	3.77	3.56	3.59

215604_x_at	---	---	8.54	8.61	8.59
219409_at	Smad nuclear inte	SNIP1	7.92	7.79	7.99
211868_x_at	immunoglobulin h	IGH@ /// IGHA1 //	5.35	5.32	5.48
215951_at	---	---	6.09	5.98	5.95
207353_s_at	H6 family homeob	HMX1	7.06	6.86	7.04
216218_s_at	phospholipase C-l	PLCL2	5.75	6.18	5.83
203733_at	Dexi homolog (mc	DEXI	8.76	8.60	8.65
211826_s_at	AF4/FMR2 family,	AFF1	5.96	6.05	5.91
214111_at	opioid binding pro	OPCML	3.60	3.92	3.83
212109_at	hematological and	HN1L	7.65	7.79	7.52
208647_at	farnesyl-diphosph	FDFT1	12.58	12.72	12.68
211835_at	immunoglobulin k	IGKV1-5	5.90	6.00	5.85
207100_s_at	vesicle-associated	VAMP1	5.81	6.36	5.94
209666_s_at	conserved helix-lo	CHUK	8.98	9.15	9.26
206401_s_at	microtubule-asso	MAPT	5.89	6.02	5.92
221113_s_at	wingless-type MM	WNT16	6.96	7.04	6.67
210496_at	NAG18 mRNA	NAG18	3.49	3.60	3.47
221527_s_at	par-3 partitioning	PARD3	7.97	7.93	8.05
204964_s_at	sarcospan (Kras or	SSPN	4.74	4.91	4.45
220199_s_at	axin interactor, do	AIDA	9.59	9.70	9.89
219709_x_at	family with seque	FAM173A	8.87	8.65	8.72
214882_s_at	splicing factor, arg	SFRS2	11.76	11.97	11.75
217891_at	chromosome 16 o	C16orf58	6.27	6.22	6.24
200881_s_at	DnaJ (Hsp40) hom	DNAJA1	10.01	10.40	10.10
204516_at	ataxin 7	ATXN7	8.22	8.22	8.56
203553_s_at	mitogen-activated	MAP4K5	7.68	7.73	7.97
202287_s_at	tumor-associated	TACSTD2	3.92	3.63	3.61
216904_at	collagen, type VI,	COL6A1	4.78	4.68	4.91
202270_at	guanylate binding	GBP1	7.26	7.45	7.77
215166_at	immunoglobulin s	IGSF9B	4.20	4.46	4.08
201267_s_at	proteasome (pros	PSMC3	9.96	9.99	10.00
216728_at	---	---	3.68	3.57	3.33
221614_s_at	rabphilin 3A-like (	RPH3AL	6.82	6.89	6.68
200725_x_at	ribosomal protein	RPL10	14.49	14.46	14.53
203925_at	glutamate-cystein	GCLM	9.66	9.73	9.90
81737_at	---	---	5.30	5.26	5.20
204884_s_at	HUS1 checkpoint	HUS1	3.91	3.97	4.26
206919_at	ELK4, ETS-domain	ELK4	4.58	4.31	4.48
208837_at	transmembrane e	TMED3	10.29	10.21	10.09
217127_at	cystathionase (cys	CTH	10.72	10.30	10.98
203771_s_at	biliverdin reducta	BLVRA	8.82	8.78	8.84
216749_at	---	---	6.56	6.23	6.11
211136_s_at	cleft lip and palate	CLPTM1	7.18	7.25	6.88
222018_at	nascent polypepti	NACA	8.19	8.54	8.26
220115_s_at	cadherin 10, type	CDH10	3.74	3.83	3.52
221803_s_at	nuclear receptor b	NRBF2	8.87	8.76	9.08
207051_at	solute carrier fam	SLC17A4	4.98	5.18	5.15

201252_at	proteasome (pros	PSMC4	9.64	9.70	9.77
219216_at	Ewing tumor-asso	ETAA1	7.85	8.39	8.24
211146_at	---	---	4.92	4.69	4.55
213216_at	OTU domain conta	OTUD3	7.16	7.31	7.09
202196_s_at	dickkopf homolog	DKK3	9.71	9.43	9.75
205938_at	protein phosphata	PPM1E	6.42	6.10	6.51
207153_s_at	glomulin, FKBP as	GLMN	8.04	8.15	8.11
222079_at	v-ets erythroblast	ERG	4.74	4.78	4.70
219152_at	podocalyxin-like 2	PODXL2	6.20	6.17	5.88
206665_s_at	BCL2-like 1	BCL2L1	6.33	6.05	6.48
204750_s_at	desmocollin 2	DSC2	3.80	3.72	3.74
217751_at	glutathione S-tran	GSTK1	9.23	9.23	9.12
221980_at	Elastin microfibril	EMILIN2	4.13	4.30	4.12
216486_x_at	zinc finger protein	ZNF79	6.54	6.63	6.44
216870_x_at	deleted in lympho	DLEU2	7.49	7.92	7.96
204195_s_at	PBX/knotted 1 ho	PKNOX1	7.90	7.87	7.86
210229_s_at	colony stimulating	CSF2	6.44	6.31	6.26
202259_s_at	NEDD4 binding pr	N4BP2L2	8.57	8.40	8.56
203096_s_at	Rap guanine nucle	RAPGEF2	8.06	7.99	8.18
220428_at	CD207 molecule, l	CD207	3.85	3.73	3.92
218130_at	chromosome 17 o	C17orf62	7.95	7.92	7.74
216405_at	---	---	6.04	6.03	6.26
211578_s_at	ribosomal protein	RPS6KB1	7.15	7.22	7.38
205617_at	proline rich Gla (G	PRRG2	7.05	6.78	6.93
202856_s_at	solute carrier fam	SLC16A3	7.53	7.72	7.65
217618_x_at	HUS1 checkpoint	HUS1	7.34	7.35	7.47
207615_s_at	chromosome 16 o	C16orf3	4.92	4.75	5.04
222050_at	---	---	5.92	5.93	5.68
205166_at	calpain 5	CAPN5	4.64	4.43	4.38
207528_s_at	solute carrier fam	SLC7A11	7.65	7.51	7.60
201255_x_at	HLA-B associated	BAT3	9.92	9.84	9.85
219214_s_at	5', 3'-nucleotidase	NT5C	8.88	8.67	8.80
219131_at	UbiA prenyltransfe	UBIAD1	8.02	7.69	7.93
AFFX-r2-Bs-dap-	---	---	10.61	10.59	10.46
216891_at	---	---	3.93	3.97	4.03
213251_at	SWI/SNF related,	SMARCA5	9.48	9.87	9.85
211765_x_at	peptidylprolyl isor	PPIA	14.43	14.44	14.46
215283_at	hypothetical LOC3	LOC339290	7.28	7.33	7.05
216147_at	---	---	5.63	5.72	5.57
220054_at	interleukin 23, alp	IL23A	6.06	5.90	5.86
207973_x_at	acrosomal vesicle	ACRV1	3.93	3.93	3.88
205430_at	bone morphogene	BMP5	3.15	3.24	3.22
201766_at	elaC homolog 2 (E	ELAC2	4.21	4.35	4.33
210742_at	CDC14 cell divisio	CDC14A	4.78	4.09	4.42
217589_at	RAB40A, member	RAB40A	4.41	4.21	4.34
207720_at	loricrin	LOR	5.85	5.67	5.68
210313_at	leukocyte immuno	LILRA4	6.38	6.33	6.19

208226_x_at	ADAM metallopep	ADAM22	4.41	4.15	4.16
209763_at	chordin-like 1	CHRDL1	4.76	4.49	4.57
200977_s_at	Tax1 (human T-ce	TAX1BP1	10.93	10.90	11.02
218343_s_at	general transcript	GTF3C3	9.57	9.66	9.60
218378_s_at	PRKR interacting p	PRKRIP1	8.25	8.23	8.38
209554_at	CD36 molecule (th	CD36	5.06	4.67	4.75
218014_at	nucleoporin 85kD	NUP85	10.11	10.28	10.18
211250_s_at	SH3-domain bindi	SH3BP2	7.03	6.92	7.02
206605_at	endonuclease, po	ENDOU	4.65	4.62	4.35
221262_s_at	solute carrier fam	SLC2A11	5.96	5.73	5.78
211997_x_at	H3 histone, family	H3F3B	12.68	12.67	12.72
206714_at	arachidonate 15-li	ALOX15B	4.05	4.29	4.06
214403_x_at	SAM pointed dom	SPDEF	7.95	7.86	7.73
220915_s_at	---	---	6.64	6.90	6.87
212762_s_at	transcription facto	TCF7L2	6.94	6.79	7.03
221096_s_at	transmembrane a	TMCO6	8.27	8.31	8.27
201355_s_at	bromodomain adj	BAZ2A	7.23	7.31	7.27
214774_x_at	TOX high mobility	TOX3	3.16	3.24	2.90
218570_at	kelch repeat and B	KBTBD4 /// PTPMT	7.78	7.90	7.80
217579_x_at	---	---	6.57	6.74	6.87
214591_at	kelch-like 4 (Droso	KLHL4	3.60	3.53	3.58
201792_at	AE binding protein	AEBP1	6.44	6.45	6.27
201337_s_at	vesicle-associated	VAMP3	7.56	8.04	7.93
220808_at	Theg homolog (mo	THEG	5.34	5.52	5.28
212236_x_at	keratin 17	KRT17	7.92	7.87	7.74
201389_at	integrin, alpha 5 (f	ITGA5	8.22	8.22	8.06
216338_s_at	Yip1 domain famil	YIPF3	7.31	7.19	7.34
209985_s_at	achaete-scute cor	ASCL1	5.67	5.92	5.95
210188_at	GA binding protein	GABPA	5.95	6.30	6.48
221715_at	---	---	8.00	7.77	7.39
212371_at	PPPDE peptidase c	PPPDE1	10.99	10.94	11.05
205550_s_at	brain and reprodu	BRE	9.56	9.45	9.47
202617_s_at	methyl CpG bindir	MECP2	6.65	6.82	6.86
209498_at	carcinoembryonic	CEACAM1	3.97	3.85	3.75
219674_s_at	high density lipop	HDLBP	6.13	6.10	5.97
219640_at	claudin 15	CLDN15	9.13	9.26	8.96
215360_at	---	---	4.02	3.90	3.87
208258_s_at	growth arrest-spe	GAS2L1	6.49	6.15	6.01
217607_x_at	eukaryotic transla	EIF4G2	3.64	3.60	3.60
204692_at	leucine-rich repea	LRCH4 /// SAP25	7.39	7.30	7.30
201116_s_at	carboxypeptidase	CPE	6.77	6.60	6.38
221794_at	dedicator of cytok	DOCK6	6.54	6.47	6.38
59375_at	myosin XVB pseud	MYO15B	6.35	6.56	6.28
213791_at	proenkephalin	PENK	4.16	3.97	3.89
214334_x_at	DAZ associated pr	DAZAP2	11.90	12.00	12.07
207927_at	5-hydroxytryptam	HTR7	6.99	6.82	6.60
207284_s_at	aspartate beta-hy	ASPH	4.47	4.79	4.76

208678_at	ATPase, H+ transp	ATP6V1E1	10.74	10.89	10.73
216570_x_at	ribosomal protein	RPL29	12.28	12.26	12.36
202660_at	inositol 1,4,5-tripl	ITPR2	5.65	5.88	5.84
207544_s_at	alcohol dehydroge	ADH6	3.78	3.57	3.78
217655_at	hypothetical LOC1	LOC100127972	6.20	5.98	5.95
221706_s_at	unconventional SI	USE1	7.95	7.81	7.93
205063_at	survival of motor	SIP1	8.65	8.73	9.10
206816_s_at	sperm associated	SPAG8	7.09	6.76	6.73
205778_at	kallikrein-related	KLK7	5.80	5.78	5.80
217663_at	zinc finger protein	ZNF234	5.36	4.98	5.19
208233_at	podoplanin	PDPN	3.87	4.08	3.81
200744_s_at	guanine nucleotid	GNB1	10.23	10.15	10.21
205717_x_at	protocadherin gar	PCDHGA1 /// PCDI	7.29	7.24	6.94
211012_s_at	golgin A6 family-li	GOLGA6L4 /// PMI	7.94	7.80	7.83
205358_at	glutamate recepto	GRIA2	3.86	3.96	3.56
209416_s_at	fizzy/cell division	FZR1	6.27	6.30	5.98
212127_at	Ran GTPase activa	RANGAP1	8.57	8.39	8.47
221022_s_at	polyamine modula	PMFBP1	5.61	5.54	5.17
208962_s_at	fatty acid desatur	FADS1	9.74	9.81	9.94
209375_at	xeroderma pigme	XPC	9.35	9.30	9.43
202945_at	folylpolyglutamat	FPGS	7.80	7.86	7.94
208168_s_at	chitinase 1 (chitot	CHIT1	5.24	5.24	5.10
200798_x_at	myeloid cell leuke	MCL1	10.81	10.96	11.01
218967_s_at	phosphotriesteras	PTER	8.06	8.01	8.48
220677_s_at	ADAM metallopep	ADAMTS8	4.39	4.18	4.13
215183_at	---	---	3.81	4.18	4.14
221861_at	---	---	6.76	6.78	6.60
215567_at	FCF1 small subuni	FCF1	5.99	6.13	6.05
214550_s_at	transportin 3	TNPO3	8.52	8.58	8.58
203591_s_at	colony stimulating	CSF3R	5.10	5.31	4.70
203918_at	protocadherin 1	PCDH1	7.05	7.02	6.92
217806_s_at	polymerase (DNA-	POLDIP2	9.99	10.02	9.97
211992_at	WNK lysine defici	WNK1	9.30	9.17	9.33
217951_s_at	PHD finger protei	PHF3	7.79	7.93	8.02
207705_s_at	ninein-like	NINL	5.91	5.88	6.11
221332_at	bone morphogene	BMP15	4.31	4.55	4.26
201712_s_at	RAN binding prote	RANBP2	8.17	8.40	8.20
210041_s_at	phosphoglucomut	PGM3	8.09	8.31	8.36
203511_s_at	trafficking protein	TRAPPC3	9.38	9.40	9.50
214098_at	KIAA1107	KIAA1107	4.97	5.23	5.25
214946_x_at	family with seque	FAM21A /// FAM2	9.10	9.12	9.09
202103_at	bromodomain cor	BRD4	8.30	8.06	8.31
211893_x_at	CD6 molecule	CD6	4.70	4.94	4.63
202765_s_at	fibrillin 1	FBN1	5.26	5.20	4.95
222287_at	triadin	TRDN	3.18	3.08	3.19
205764_at	---	---	3.36	3.61	3.22
211688_x_at	killer cell immuno	KIR3DL1 /// KIR3D	4.44	4.61	4.41

208491_s_at	phosphoglucomut	PGM5	5.28	5.24	5.07
208131_s_at	prostaglandin I2 (|	PTGIS	4.51	4.42	4.15
212152_x_at	AT rich interactive	ARID1A	8.42	8.48	8.43
213165_at	centrosomal prot	CEP350	9.27	9.36	9.46
207064_s_at	amine oxidase, co	AOC2	5.59	5.69	5.61
200957_s_at	structure specific	SSRP1	10.94	10.88	11.01
216730_at	---	---	6.13	6.19	6.24
201770_at	small nuclear ribo	SNRPA	10.17	10.04	10.08
218222_x_at	aryl hydrocarbon	ARNT	6.36	6.29	6.18
203036_s_at	metastasis suppre	MTSS1	6.36	6.72	6.29
222110_at	SUMO1/sentrin sp	SENP5	5.94	5.75	5.92
207082_at	colony stimulating	CSF1	5.15	5.21	5.00
217714_x_at	stathmin 1	STMN1	9.05	8.77	8.94
203969_at	peroxisomal bioge	PEX3	4.95	5.06	4.93
201344_at	ubiquitin-conjugat	UBE2D2	7.27	7.31	7.24
201688_s_at	tumor protein D5	TPD52	8.48	8.52	8.65
206868_at	StAR-related lipid	STARD8	6.87	6.84	6.74
218179_s_at	chromosome 4 op	C4orf41	7.79	8.01	7.91
213677_s_at	PMS1 postmeiotic	PMS1	9.86	9.85	9.94
216768_x_at	Tetratricopeptide	TTC38	6.10	5.70	6.05
207163_s_at	v-akt murine thym	AKT1	8.01	8.02	7.99
207118_s_at	matrix metallope	MMP23A /// MMF	5.52	5.41	5.28
210407_at	protein phosphata	PPM1A	8.02	8.00	8.43
219117_s_at	FK506 binding pro	FKBP11	11.18	11.16	11.26
206869_at	chondroadherin	CHAD	4.67	4.87	4.40
202770_s_at	cyclin G2	CCNG2	7.17	6.92	7.32
201775_s_at	KIAA0494	KIAA0494	6.93	7.11	6.77
217943_s_at	MAP7 domain cor	MAP7D1	9.96	9.93	9.98
201950_x_at	capping protein (a	CAPZB	9.35	9.37	9.46
221387_at	neuropeptide FF r	NPFFR1	6.87	6.69	6.44
210069_at	choline kinase-like	CHKB-CPT1B /// CI	7.76	7.83	7.73
206220_s_at	RAS p21 protein a	RASA3	7.51	7.26	6.93
205218_at	polymerase (RNA)	POLR3F	7.11	7.37	7.23
215981_at	---	---	3.49	3.52	3.59
220628_s_at	sidekick homolog	SDK2	6.72	6.58	6.36
211767_at	GINS complex sub	GINS4	8.03	7.96	7.84
206543_at	SWI/SNF related,	SMARCA2	3.60	3.80	3.62
216822_x_at	---	---	7.35	7.39	7.29
214917_at	protein kinase, AM	PRKAA1	5.75	6.37	6.09
220500_s_at	RAB, member of F	RABL2A /// RABL2	5.21	5.31	5.16
217366_at	catenin (cadherin-	CTNNA1	4.31	4.32	4.16
220106_at	NPC1 (Niemann-P	NPC1L1	5.55	5.46	5.10
220187_at	STEAP family men	STEAP4	3.28	3.42	3.67
209748_at	spastin	SPAST	8.25	8.25	8.45
214319_at	furry homolog (Dr	FRY	7.07	7.21	7.36
210086_at	hairless homolog	HR	5.89	5.80	5.71
217111_at	alpha-methylacyl-	AMACR	6.59	6.32	6.25

221049_s_at	polymerase (DNA	POLL	8.44	8.23	8.12
207372_s_at	ectonucleoside tri	ENTPD2	5.31	5.17	5.31
211486_s_at	potassium voltage	KCNQ2	6.47	6.05	6.27
208115_x_at	chromosome 10 c	C10orf137	4.80	5.08	5.14
201979_s_at	protein phosphata	PPP5C	7.93	7.88	7.84
204773_at	interleukin 11 rec	IL11RA	6.86	6.69	6.47
216860_s_at	growth differentia	GDF11	6.80	6.62	6.85
220072_at	centrosome and s	CSPP1	8.24	8.09	8.24
204382_at	N-acetyltransfera	NAT9	8.37	8.35	8.49
218572_at	chromatin modify	CHMP4A	8.80	8.72	8.89
220228_at	adaptor-related p	AP4E1	3.66	3.79	3.54
214501_s_at	H2A histone famil	H2AFY	10.98	10.99	11.01
215962_at	---	---	3.28	3.31	3.17
213250_at	Coiled-coil domai	CCDC85B	6.77	6.48	6.65
218224_at	paraneoplastic an	PNMA1	9.47	9.31	9.60
212110_at	solute carrier fam	SLC39A14	11.74	11.62	11.72
211144_x_at	TCR gamma altern	TARP /// TRGC2	4.63	4.76	4.40
218426_s_at	ring finger protei	RNF216	7.98	7.91	8.22
205431_s_at	bone morphogen	BMP5	5.57	5.84	5.34
213234_at	KIAA1467	KIAA1467	7.06	6.87	7.03
221679_s_at	abhydrolase dom	ABHD6	6.38	6.34	6.44
206267_s_at	megakaryocyte-as	MATK	7.05	6.80	6.91
220603_s_at	multiple C2 doma	MCTP2	6.22	6.44	6.27
210705_s_at	tripartite motif-co	TRIM5	6.72	7.04	7.09
201898_s_at	ubiquitin-conjuga	UBE2A	9.88	9.95	10.01
213902_at	N-acylsphingosine	ASAH1	9.12	9.03	9.33
207210_at	gamma-aminobut	GABRA3	3.85	3.93	3.99
204534_at	vitronectin	VTN	5.65	5.47	5.21
208984_x_at	RNA binding moti	RBM10	9.61	9.45	9.54
221860_at	heterogeneous nu	HNRNPL	8.22	8.28	8.23
218048_at	COMM domain co	COMMD3	10.83	10.71	10.93
217820_s_at	enabled homolog	ENAH	11.06	11.14	11.38
200049_at	MYST histone ace	MYST2	9.02	8.94	9.12
211960_s_at	RAB7A, member R	RAB7A	9.58	9.83	9.87
216187_x_at	---	---	10.15	10.30	10.41
207617_at	DEAD (Asp-Glu-Al	DDX3X	4.43	4.41	4.30
214038_at	chemokine (C-C m	CCL8	3.62	3.63	3.84
216696_s_at	proline dehydroge	PRODH2	6.94	7.03	6.93
207944_at	oncomodulin 2	OCM2	4.73	4.59	4.51
206660_at	immunoglobulin l	IGLL1	4.70	4.44	5.01
213229_at	dicer 1, ribonucle	DICER1	8.72	8.78	8.78
207847_s_at	mucin 1, cell surfa	MUC1	6.23	6.14	6.05
215406_at	---	---	4.88	4.98	4.82
215247_at	LIM and senescen	LOC440895	5.86	5.65	5.73
212658_at	lipoma HMGIC fus	LHFPL2	8.26	8.04	8.27
220273_at	interleukin 17B	IL17B	4.68	4.97	4.77
210988_s_at	prune homolog (D	PRUNE	5.96	6.15	6.10

221692_s_at	mitochondrial ribo	MRPL34	10.87	10.83	10.86
215391_at	microtubule-asso	MAP1A	4.14	4.17	4.26
209899_s_at	poly-U binding spl	PUF60	10.59	10.47	10.46
221064_s_at	unkempt homolog	UNKL	8.00	7.89	8.02
216479_at	ribosomal protein	RPL21 /// RPL21P1	4.80	4.70	5.05
221896_s_at	HIG1 hypoxia indu	HIGD1A	11.28	11.38	11.49
219368_at	nucleosome asser	NAP1L2	3.86	4.31	4.00
215108_x_at	TOX high mobility	TOX3	3.08	3.08	3.18
216580_at	ribosomal protein	RPL7	3.36	3.51	3.49
203297_s_at	jumonji, AT rich in	JARID2	8.18	8.06	8.20
206988_at	chemokine (C-C m	CCL25	3.72	3.88	3.91
213026_at	ATG12 autophagy	ATG12	9.78	9.71	9.92
218459_at	torsin family 3, m	TOR3A	8.75	8.62	8.73
221689_s_at	phosphatidylinosi	PIGP	10.75	10.78	10.83
203515_s_at	phosphomevalona	PMVK	9.14	9.07	9.06
220955_x_at	RAB23, member R	RAB23	7.44	7.41	7.82
200679_x_at	high-mobility grou	HMGB1	12.36	12.51	12.57
219732_at	lipid phosphate pl	LPPR1	3.66	3.54	3.73
214959_s_at	apoptosis inhibito	API5	6.71	6.75	6.62
218658_s_at	ARP8 actin-relate	ACTR8	6.81	7.10	6.99
218661_at	N-acetyltransfera	NAT15	8.27	8.08	8.01
220191_at	gastrokine 1	GKN1	5.62	5.56	5.52
200769_s_at	methionine adeno	MAT2A	8.58	8.89	8.72
202903_at	LSM5 homolog, U	LSM5	8.40	8.52	8.49
212746_s_at	centrosomal prot	CEP170	8.90	8.87	9.06
201530_x_at	eukaryotic transla	EIF4A1	12.79	12.90	12.87
215734_at	chromosome 19 o	C19orf36	3.66	3.86	3.60
211193_at	tumor protein p6	TP63	4.27	4.56	4.52
208384_s_at	midline 2	MID2	5.77	5.97	5.73
218584_at	tectonic family m	TCTN1	7.88	7.83	7.86
204614_at	serpin peptidase i	SERPINB2	3.93	3.90	3.64
221311_x_at	LYR motif contain	LYRM2	8.58	8.72	8.76
200044_at	splicing factor, ar	SFRS9	11.63	11.70	11.77
201921_at	guanine nucleotid	GNG10	12.20	12.37	12.37
213728_at	lysosomal-associa	LAMP1	7.62	7.58	7.65
203680_at	protein kinase, cA	PRKAR2B	3.45	3.69	3.62
206435_at	beta-1,4-N-acetyl-	B4GALNT1	7.57	7.44	7.44
207914_x_at	even-skipped hom	EVX1	7.58	7.30	7.16
210332_at	hypothetical prot	LOC100287322	7.40	7.01	6.89
209761_s_at	SP110 nuclear bo	SP110	6.85	6.35	6.65
221190_s_at	chromosome 18 o	C18orf8	8.60	8.57	8.60
201699_at	proteasome (pros	PSMC6	11.25	11.34	11.26
211870_s_at	protocadherin alp	PCDHA3	5.79	5.90	5.45
209778_at	thyroid hormone	TRIP11	6.53	7.06	6.94
207355_at	solute carrier fam	SLC1A7	6.35	6.45	6.11
204246_s_at	dynactin 3 (p22)	DCTN3	10.07	10.03	9.92
218690_at	PDZ and LIM dom	PDLIM4	6.83	7.00	6.79

217183_at	low density lipopr	LDLR	5.95	6.11	6.01
33767_at	neurofilament, he	NEFH	3.27	3.26	3.37
212176_at	splicing factor, arg	SFRS18	9.49	9.75	9.72
209023_s_at	stromal antigen 2	STAG2	9.07	9.05	9.33
211736_at	Sp2 transcription	SP2	3.70	3.77	3.69
208677_s_at	basigin (Ok blood	BSG	9.00	8.81	8.76
213537_at	major histocompa	HLA-DPA1	3.96	4.24	4.00
202730_s_at	programmed cell	PDCD4	8.57	8.37	8.77
219405_at	tripartite motif-co	TRIM68	8.00	8.06	8.09
213175_s_at	small nuclear ribo	SNRPB	12.17	12.15	12.13
220789_s_at	transforming grov	TBRG4	9.04	9.00	9.12
201470_at	glutathione S-tran	GSTO1	12.58	12.55	12.57
220650_s_at	solute carrier fam	SLC9A5	7.11	6.94	7.02
213754_s_at	similar to poly(A)	LOC645139 /// PA	10.43	10.31	10.63
220037_s_at	lymphatic vessel e	LYVE1	3.39	3.42	3.36
212808_at	nuclear factor of a	NFATC2IP	7.04	7.21	7.26
211560_s_at	aminolevulinate, c	ALAS2	6.24	6.21	5.91
35254_at	TRAF-type zinc fin	TRAFD1	7.51	7.54	7.53
203336_s_at	integrin beta 1 bir	ITGB1BP1	11.44	11.48	11.49
206415_at	tolloid-like 1	TLL1	4.26	4.27	4.31
217097_s_at	putative homeodo	PHTF2	4.71	4.58	4.13
200703_at	dynein, light chair	DYNLL1	11.92	12.15	12.03
204065_at	carbohydrate sulfo	CHST10	6.52	6.84	6.80
206476_s_at	neuro-oncological	NOVA2	4.41	4.22	4.19
218860_at	nucleolar complex	NOC4L	8.34	8.26	8.31
202891_at	microtubule-assoc	MAP7	9.05	8.88	9.05
203894_at	tubulin, gamma 2	TUBG2	6.56	6.62	6.76
208733_at	RAB2A, member R	RAB2A	4.77	4.81	5.29
201806_s_at	ataxin 2-like	ATXN2L	6.94	6.71	6.80
211497_x_at	NK3 homeobox 1	NKX3-1	5.34	5.35	5.03
204536_s_at	---	---	5.75	5.70	5.37
214391_x_at	prostaglandin E re	PTGER1	7.78	8.05	7.70
216794_at	---	---	5.88	5.45	5.59
220966_x_at	actin related prote	ARPC5L	10.83	10.87	10.82
204930_s_at	BCL2/adenovirus	BNIP1	8.90	8.85	8.70
212095_s_at	microtubule assoc	MTUS1	3.98	4.00	4.05
215097_at	capping protein (a	CAPZB	4.79	5.01	4.66
220730_at	zinc finger proteir	ZNF778	5.86	5.86	5.79
209882_at	Ras-like without C	RIT1	7.65	7.77	7.91
220912_at	---	---	6.43	6.36	6.50
200787_s_at	phosphoprotein e	PEA15	7.65	7.62	7.90
212149_at	EFR3 homolog A (	EFR3A	10.51	10.60	10.77
207288_at	non-protein codin	NCRNA00105	4.74	4.92	4.95
218351_at	COMM domain co	COMMD8	10.77	10.87	10.87
205958_x_at	chorionic somato	CSHL1	3.47	3.54	3.45
208424_s_at	cytokine induced	CIAPIN1	9.62	9.67	9.81
207946_at	mastermind-like 3	MAML3	5.90	5.82	6.04

213494_s_at	YY1 transcription	YY1	7.21	7.21	7.10
203653_s_at	coilin	COIL	8.25	8.21	8.21
204538_x_at	nuclear pore com	NPIP	10.42	10.29	10.29
218943_s_at	DEAD (Asp-Glu-Al	DDX58	6.44	6.59	6.61
217048_at	---	---	4.72	4.77	4.73
203122_at	tetratricopeptide	TTC15	8.32	8.43	8.41
217481_x_at	---	---	3.87	3.56	3.62
210093_s_at	mago-nashi homo	MAGOH	8.89	8.84	9.12
217628_at	chloride intracellu	CLIC5	5.40	5.12	5.17
206804_at	CD3g molecule, ga	CD3G	5.02	4.67	4.75
214248_s_at	tripartite motif-co	TRIM2	5.37	5.62	5.37
216511_s_at	transcription facto	TCF7L2	8.51	8.28	8.74
216677_at	zinc finger protein	ZNF154	5.21	5.11	5.07
212065_s_at	ubiquitin specific	USP34	6.26	6.11	6.15
216821_at	keratin 8	KRT8	6.64	6.22	6.42
217012_at	---	---	3.68	3.70	3.72
219610_at	190 kDa guanine r	RGNEF	8.03	8.08	7.97
216775_at	ubiquitin specific	USP53	4.97	5.38	5.00
218677_at	S100 calcium bind	S100A14	5.89	5.92	5.90
212545_s_at	zinc fingers and ho	ZHX3	6.95	6.99	7.08
204729_s_at	syntaxin 1A (brain	STX1A	7.58	7.51	7.50
201111_at	CSE1 chromosome	CSE1L	11.38	11.54	11.49
217825_s_at	ubiquitin-conjugat	UBE2J1	8.13	8.12	8.30
209697_at	---	---	5.90	5.93	5.63
212558_at	sprouty homolog	SPRY1	6.42	6.41	6.59
210660_at	leukocyte immuno	LILRA1	3.39	3.31	3.24
213474_at	potassium channe	KCTD7	6.71	6.95	6.61
202416_at	DnaJ (Hsp40) hom	DNAJC7	10.37	10.28	10.32
210208_x_at	HLA-B associated	BAT3	9.98	9.91	9.99
205378_s_at	acetylcholinestera	ACHE	4.42	4.29	4.11
202786_at	serine threonine k	STK39	9.33	9.26	9.78
212582_at	oxysterol binding	OSBPL8	11.09	11.30	11.43
220333_at	progestin and adip	PAQR5	7.70	7.70	7.74
209005_at	F-box and leucine	FBXL5	8.27	8.36	8.48
202293_at	stromal antigen 1	STAG1	9.12	9.14	9.30
201945_at	furin (paired basic	FURIN	7.32	7.18	7.47
204744_s_at	isoleucyl-tRNA syr	IARS	12.88	12.78	12.93
202135_s_at	ARP1 actin-related	ACTR1B	9.27	9.21	9.20
202527_s_at	SMAD family men	SMAD4	9.30	9.27	9.46
208656_s_at	cyclin I	CCNI	12.19	12.10	12.22
220811_at	proteoglycan 3	PRG3	5.69	5.65	5.63
201541_s_at	zinc finger, HIT typ	ZNHIT1	10.31	10.25	10.31
AFFX-TrpnX-3_a	---	---	3.56	3.59	3.44
222158_s_at	PPPDE peptidase	PPPDE1	9.95	9.90	10.13
202019_s_at	LanC lantibiotic sy	LANCL1	7.56	7.59	7.46
201716_at	sorting nexin 1	SNX1	9.22	9.21	9.29
204942_s_at	aldehyde dehydro	ALDH3B2	4.06	3.89	3.94

211153_s_at	tumor necrosis fa	TNFSF11	4.82	4.74	4.58
207816_at	lactalbumin, alpha	LALBA	4.38	4.46	4.38
208900_s_at	topoisomerase (D	TOP1	10.15	10.19	10.21
207103_at	potassium voltage	KCND2	3.17	3.24	3.08
211693_at	Immunoglobulin h	IGHA1	7.39	7.15	7.17
206694_at	pancreatic lipase-	PNLIPRP1	6.71	6.39	6.07
208996_s_at	polymerase (RNA)	POLR2C	9.57	9.58	9.57
211672_s_at	actin related prot	ARPC4	8.88	8.80	8.88
214348_at	tachykinin recepto	TACR2	5.83	5.86	5.64
201676_x_at	proteasome (pros	PSMA1	12.45	12.43	12.49
206751_s_at	phosphate cytidyl	PCYT1B	3.54	3.35	3.34
207876_s_at	filamin C, gamma	FLNC	7.24	6.95	6.74
202795_x_at	TRIO and F-actin b	TRIOBP	10.02	9.88	9.95
205179_s_at	ADAM metallopep	ADAM8	5.28	5.47	5.37
220691_at	---	---	6.87	7.12	6.78
214415_at	plasminogen-like	PLGLB1 /// PLGLB2	3.76	3.38	3.46
209077_at	thioredoxin 2	TXN2	9.28	9.24	9.25
211900_x_at	CD6 molecule	CD6	4.10	4.54	4.25
211395_x_at	Fc fragment of IgG	FCGR2C	5.44	5.28	5.60
217103_at	low density lipopr	LDLR	4.59	4.13	3.97
206165_s_at	chloride channel a	CLCA2	3.69	3.65	3.45
208797_s_at	golgin A8 family, r	GOLGA8A	6.36	6.45	6.26
214740_at	polymerase (RNA)	POLR2J /// POLR2J	6.64	6.39	6.22
201144_s_at	eukaryotic transla	EIF2S1	11.47	11.50	11.56
200909_s_at	ribosomal protein	RPLP2	14.75	14.73	14.78
212500_at	2-aminoethanethi	ADO	8.94	8.92	9.18
219049_at	chondroitin sulfat	CSGALNACT1	3.62	3.69	3.88
210172_at	splicing factor 1	SF1	6.86	7.07	7.01
202549_at	VAMP (vesicle-ass	VAPB	6.39	6.14	6.22
206369_s_at	phosphoinositide-	PIK3CG	3.82	3.85	3.81
220904_at	chromosome 6 op	C6orf208	5.16	5.24	5.03
219366_at	apoptosis, caspas	AVEN	9.17	9.06	9.07
216227_at	HLA complex grou	HCG2P7	4.52	4.28	4.67
217871_s_at	macrophage migr	MIF	13.96	13.92	13.98
220840_s_at	chromosome 1 op	C1orf112	7.88	7.90	8.13
39313_at	WNK lysine deficie	WNK1	5.16	5.36	5.25
215800_at	dual oxidase 1	DUOX1	3.77	3.83	3.76
202302_s_at	arginine/serine-ri	RSRC2	11.45	11.47	11.58
201056_at	golgin B1	GOLGB1	7.46	7.55	7.61
217728_at	S100 calcium bind	S100A6	14.34	14.28	14.28
210078_s_at	potassium voltage	KCNAB1	5.11	5.09	5.24
216589_at	similar to hCG164	LOC390998	8.30	8.02	8.07
220373_at	dachsous 2 (Drosc	DCHS2	4.43	4.63	4.37
219475_at	oxidative stress in	OSGIN1	7.25	7.00	6.79
200722_s_at	cell cycle associat	CAPRIN1	8.99	8.89	8.98
207256_at	mannose-binding	MBL2	3.28	3.37	3.43
208982_at	platelet/endothel	PECAM1	5.37	5.34	5.22

47773_at	F-box protein 42	FBXO42	7.70	7.62	7.75
201874_at	myelin protein zer	MPZL1	9.23	9.16	9.13
44822_s_at	mesoderm induct	MIER2	6.93	7.17	7.05
211917_s_at	prolactin receptor	PRLR	3.82	3.48	3.50
219819_s_at	mitochondrial ribo	MRPS28	10.44	10.37	10.40
213527_s_at	zinc finger protein	ZNF688	6.66	6.47	6.79
217017_at	oxysterol binding	OSBPL10	5.96	5.82	5.83
220188_at	junctophilin 3	JPH3	4.01	4.06	4.24
205086_s_at	non-SMC condens	NCAPH2	8.30	8.25	8.26
214894_x_at	microtubule-actin	MACF1	8.72	8.86	9.12
215632_at	neurogenin 2	NEUROG2	3.62	3.57	3.30
204275_at	small optic lobes I	SOLH	6.43	6.43	6.57
221182_at	chromosome 1 op	C1orf129	3.85	3.63	3.70
216828_at	---	---	4.10	4.16	3.84
210925_at	class II, major hist	CIITA	5.44	5.61	5.22
210451_at	pyruvate kinase, li	PKLR	5.11	5.18	5.38
204914_s_at	SRY (sex determin	SOX11	3.98	3.91	3.66
209609_s_at	mitochondrial ribo	MRPL9	10.84	10.84	11.04
219571_s_at	zinc finger protein	ZNF12	8.62	8.79	8.87
212308_at	cytoplasmic linker	CLASP2	6.95	7.14	7.12
220437_at	hepatocellular car	LOC55908	5.30	5.20	4.75
219038_at	MORC family CW-	MORC4	8.07	8.19	8.23
219744_at	fructosamine 3 kin	FN3K	4.16	4.08	3.85
207368_at	5-hydroxytryptam	HTR1D	5.13	5.32	5.04
215552_s_at	estrogen receptor	ESR1	5.65	5.70	5.58
218659_at	additional sex con	ASXL2	8.91	9.07	9.07
216048_s_at	Rho-related BTB d	RHOBTB3	5.86	6.05	6.18
204649_at	trophinin associat	TROAP	8.01	8.25	7.95
203618_at	Fas apoptotic inhi	FAIM2	6.85	6.87	6.75
211538_s_at	heat shock 70kDa	HSPA2	5.04	4.64	4.48
203200_s_at	5-methyltetrahyd	MTRR	10.42	10.51	10.66
219590_x_at	DPH5 homolog (S.	DPH5	10.50	10.44	10.45
211471_s_at	RAB36, member F	RAB36	4.94	5.07	4.82
202681_at	ubiquitin specific		USP4	9.14	9.07	9.18
215829_at	SH3 and multiple	SHANK2	4.07	4.17	4.10
208055_s_at	hect domain and I	HERC4	6.87	6.96	6.72
220660_at	chromosome 9 op	C9orf27	3.72	3.78	3.72
209843_s_at	SRY (sex determin	SOX10	7.14	7.13	6.96
211405_x_at	interferon, alpha 1	IFNA17	5.43	5.65	5.79
215031_x_at	ring finger protein	RNF126	8.41	8.20	8.40
218530_at	formin homology	FHOD1	8.68	8.92	8.71
222070_at	dead end homolog	DND1	7.22	7.32	7.27
217368_at	---	---	6.94	6.84	6.96
220206_at	zinc finger, MYM-	ZMYM1	7.64	8.00	8.09
204776_at	thrombospondin 4	THBS4	6.14	6.30	6.06
202373_s_at	RAB3 GTPase acti	RAB3GAP2	9.77	9.85	9.88
214727_at	breast cancer 2, e	BRCA2	6.35	6.44	6.77

200850_s_at	adenosylhomocys	AHCYL1	11.31	11.38	11.49
222344_at	---	---	6.83	7.51	7.22
203030_s_at	protein tyrosine p	PTPRN2	4.85	4.51	4.56
215067_x_at	peroxiredoxin 2	PRDX2	8.23	8.40	8.23
207112_s_at	GRB2-associated l	GAB1	4.63	4.76	4.54
209658_at	cell division cycle	CDC16	10.37	10.50	10.56
206001_at	neuropeptide Y	NPY	4.74	5.14	5.05
215341_at	dynein, axonemal	DNAH6	6.31	6.53	6.32
64899_at	lipid phosphate pl	LPPR2	6.77	7.07	6.99
209657_s_at	heat shock transc	HSF2	8.28	8.24	8.41
219716_at	apolipoprotein L,	APOL6	7.54	7.26	7.36
211753_s_at	relaxin 1	RLN1	4.07	3.65	3.81
209521_s_at	angiomotin	AMOT	3.88	4.20	3.62
214077_x_at	Meis homeobox 3	MEIS3P1	5.57	5.24	5.57
216379_x_at	CD24 molecule	CD24	6.57	6.91	6.77
204685_s_at	ATPase, Ca++ tran	ATP2B2	6.25	6.17	5.92
211604_x_at	huntingtin-associa	HAP1	6.86	6.83	6.55
218166_s_at	remodeling and sp	RSF1	7.38	7.64	7.56
214270_s_at	microtubule-asso	MAPRE3	3.78	4.02	3.82
215889_at	SKI-like oncogene	SKIL	3.40	3.31	3.43
216192_at	fatty acid binding	FABP7	3.79	3.34	3.71
206712_at	growth hormone	GRTP1	5.77	5.85	5.93
210550_s_at	Ras protein-specif	RASGRF1	3.92	3.93	3.89
201520_s_at	G-rich RNA seque	GRSF1	11.01	11.03	11.16
220029_at	elongation of very	ELOVL2	4.25	4.38	4.37
217323_at	major histocompa	HLA-DRB6	4.43	4.43	4.44
214005_at	gamma-glutamyl	GGCX	8.28	8.24	8.35
215184_at	death-associated	DAPK2	5.17	4.90	4.93
221889_at	potassium channe	KCTD13	6.07	6.06	6.06
207386_at	cytochrome P450,	CYP7B1	3.71	3.53	3.39
204617_s_at	adrenocortical dy	ACD	8.69	8.53	8.70
202901_x_at	cathepsin S	CTSS	3.24	3.07	3.08
217147_s_at	T cell receptor ass	TRAT1	3.32	3.34	2.99
205892_s_at	fatty acid binding	FABP1	3.14	3.18	3.30
211201_at	follicle stimulating	FSHR	3.82	4.16	3.95
206465_at	acyl-CoA syntheta	ACSBG1	5.02	4.91	4.91
209982_s_at	neurexin 2	NRXN2	4.40	4.35	4.47
221132_at	claudin 18	CLDN18	5.48	5.68	5.56
215753_at	---	---	5.78	5.55	5.36
212851_at	DCN1, defective in	DCUN1D4	8.25	8.39	8.44
210637_at	tachykinin recept	TACR1	4.33	4.31	4.37
204672_s_at	ankyrin repeat do	ANKRD6	3.76	4.00	3.53
220864_s_at	NADH dehydroge	NDUFA13 /// YJEFI	11.54	11.49	11.55
204329_s_at	zinc finger protei	ZNF202	7.78	7.78	7.78
220834_at	membrane-spann	MS4A12	4.05	4.04	3.88
218759_at	dishevelled, dsh h	DVL2	7.76	7.52	7.44
208535_x_at	collagen, type XIII,	COL13A1	6.68	6.84	6.70

204011_at	sprouty homolog	SPRY2	9.48	9.38	9.58
218157_x_at	CDC42 small effec	CDC42SE1	9.17	9.23	9.27
211431_s_at	TYRO3 protein tyr	TYRO3	5.61	5.28	5.45
216019_x_at	pleckstrin homolo	PHLDB1	6.71	6.45	6.43
211217_s_at	potassium voltage	KCNQ1	7.13	7.04	6.94
204006_s_at	Fc fragment of IgG	FCGR3A /// FCGR3	3.94	3.83	3.63
200913_at	protein phosphata	PPM1G	10.47	10.51	10.46
217558_at	cytochrome P450,	CYP2C9	3.65	3.57	3.52
218030_at	G protein-couplec	GIT1	8.26	8.13	8.07
209207_s_at	SEC22 vesicle traf	SEC22B	8.97	9.00	9.04
202148_s_at	pyrroline-5-carbo:	PYCR1	9.49	9.35	9.26
216540_at	T cell receptor del	TRD@	4.31	4.28	4.19
200028_s_at	StAR-related lipid	STARD7	11.13	11.23	11.28
214291_at	similar to ribosom	LOC729046 /// RPI	7.78	7.95	7.98
208942_s_at	SEC62 homolog (S	SEC62	11.11	11.35	11.36
207618_s_at	BCS1-like (yeast)	BCS1L	10.10	10.04	10.03
209545_s_at	receptor-interacti	RIPK2	9.49	9.36	9.54
217809_at	basic leucine zipp	BZW2	13.27	13.23	13.27
207964_x_at	interferon, alpha 4	IFNA4	5.10	5.20	4.88
206947_at	UDP-Gal:betaGlcN	B3GALT5	4.47	4.23	4.62
204499_at	ATP/GTP binding p	AGTPBP1	5.54	5.51	5.33
221738_at	Ral GTPase activat	RALGAPB	7.53	7.53	7.59
221029_s_at	wingless-type MM	WNT5B	5.37	5.31	5.02
218900_at	cyclin M4	CNNM4	7.10	6.95	6.87
213507_s_at	karyopherin (impo	KPNB1	13.27	13.23	13.23
217271_at	guanine nucleotid	GNA11	4.44	4.12	4.53
221090_s_at	2-oxoglutarate an	OGFOD1	8.73	8.71	8.84
203191_at	ATP-binding casse	ABCB6	6.62	6.11	6.31
208785_s_at	microtubule-asso	MAP1LC3B	10.09	9.96	10.16
220686_s_at	piwi-like 2 (Droso	PIWIL2	6.18	6.00	5.95
207540_s_at	spleen tyrosine ki	SYK	5.10	4.82	4.65
213304_at	family with seque	FAM179B	8.60	8.53	8.65
209154_at	Tax1 (human T-ce	TAX1BP3	11.60	11.62	11.81
219355_at	chromosome X op	CXorf57	8.68	8.86	8.75
218931_at	RAB17, member F	RAB17	6.23	6.22	6.26
207930_at	lipocalin 1 (tear pi	LCN1	6.16	6.05	6.17
214346_at	Complement com	C1QL1	5.01	4.76	4.81
206996_x_at	calcium channel, v	CACNB1	5.46	5.30	5.13
213012_at	neural precursor c	NEDD4	10.22	10.24	10.45
213183_s_at	Cyclin-dependent	CDKN1C	6.17	5.86	5.76
219742_at	proline rich 7 (syn	PRR7	7.67	7.45	7.48
203315_at	NCK adaptor prote	NCK2	10.37	10.28	10.42
214546_s_at	purinergic recepto	P2RY11	6.51	6.45	6.56
208750_s_at	ADP-ribosylation f	ARF1	9.18	9.14	9.09
AFFX-PheX-3_at	---	---	8.69	8.56	8.56
215095_at	---	---	6.22	6.67	6.36
208030_s_at	adducin 1 (alpha)	ADD1	9.71	9.76	9.80

214018_at	glutamate recepto	GRIP1	4.41	3.97	4.31
217148_x_at	similar to Ig lamb	LOC100293440	7.11	7.28	6.80
201044_x_at	dual specificity ph	DUSP1	7.14	6.86	7.14
207506_at	glutaredoxin 3	GLRX3	6.29	6.00	5.81
220465_at	hypothetical LOC8	LOC80054	7.19	7.36	7.38
214528_s_at	paired box 8	PAX8	3.69	3.65	3.70
220857_at	---	---	4.03	3.93	3.85
222308_x_at	---	---	4.18	4.03	4.08
211520_s_at	glutamate recepto	GRIA1	3.76	3.35	3.42
205018_s_at	muscleblind-like 2	MBNL2	6.37	6.14	6.57
221107_at	cholinergic recept	CHRNA9	3.59	4.01	3.68
204378_at	breast carcinoma	BCAS1	6.71	6.53	6.34
215365_at	calcium channel, v	CACNB2	3.85	3.68	3.64
215106_at	tetratricopeptide	TTC22	5.07	4.82	4.73
210399_x_at	fucosyltransferase	FUT6	7.12	7.30	7.09
218655_s_at	CWC25 spliceosor	CWC25	7.94	8.05	7.92
216276_s_at	ADAM metallope	ADAM3A	3.90	4.21	3.84
205582_s_at	gamma-glutamylt	GGT5	6.97	7.09	6.76
222093_s_at	INO80 complex su	INO80B	7.57	7.49	7.47
204022_at	WW domain conta	WWP2	7.38	7.41	7.49
215682_at	proline dehydroge	LOC440792	6.87	6.89	6.55
201975_at	CAP-GLY domain c	CLIP1	9.04	8.97	9.36
211448_s_at	regulator of G-pro	RGS6	6.70	6.17	6.19
206708_at	forkhead box N2	FOXN2	5.06	5.21	5.20
214264_s_at	chromosome 14 c	C14orf143	8.91	8.95	8.93
200826_at	small nuclear ribo	SNRPD2	13.41	13.43	13.45
222168_at	---	---	3.35	3.39	3.48
206014_at	actin-like 6B	ACTL6B	6.27	6.39	6.18
220063_at	glutathione S-tran	GSTCD	6.85	6.83	6.68
214618_at	CASP8 and FADD-l	CFLAR	3.50	3.51	3.36
202006_at	protein tyrosine p	PTPN12	10.67	10.76	10.88
210286_s_at	solute carrier fam	SLC4A7	4.71	4.95	5.27
207262_at	apolipoprotein F	APOF	3.90	3.78	3.79
206196_s_at	RUN domain cont	RUNDC3A	4.74	4.86	4.45
205991_s_at	paired related hor	PRRX1	4.63	4.33	4.28
212187_x_at	prostaglandin D2 :	PTGDS	6.58	6.64	6.47
208306_x_at	Major histocompa	HLA-DRB1	6.19	6.33	5.99
217851_s_at	slowmo homolog	SLMO2	9.67	9.89	10.00
209978_s_at	lipoprotein, Lp(a)	LPA /// PLG	4.51	4.47	4.65
212654_at	tropomyosin 2 (be	TPM2	6.74	6.72	6.64
209098_s_at	jagged 1 (Alagille :	JAG1	6.83	6.62	6.78
218191_s_at	LMBR1 domain cc	LMBRD1	8.85	8.97	8.97
208894_at	major histocompa	HLA-DRA	4.68	4.50	4.34
220725_x_at	Dynein, axonemal	DNAH3	7.65	7.71	7.52
201622_at	staphylococcal nu	SND1	9.99	9.88	9.99
211641_x_at	immunoglobulin h	IGH@ /// IGHA1 //	5.23	5.43	5.06
207343_at	lysozyme-like 6	LYZL6	5.45	5.78	5.48

222199_s_at	bridging integrato	BIN3	7.85	7.97	7.82
218255_s_at	fibrosin	FBRS	7.96	7.73	7.80
219999_at	mannosidase, alpl	MAN2A2	6.68	6.68	6.52
213555_at	RWD domain cont	RWDD2A	6.84	7.03	7.06
215858_at	---	---	6.81	7.00	6.94
216382_s_at	myeloid/lymphoic	MLL2	7.12	7.20	7.34
206316_s_at	kinetochore assoc	KNTC1	8.25	8.38	8.36
213984_at	PDS5, regulator of	PDS5A	7.15	7.33	7.79
200800_s_at	heat shock 70kDa	HSPA1A /// HSPA1	6.83	6.93	7.09
205421_at	solute carrier fam	SLC22A3	3.24	3.26	3.10
212731_at	ankyrin repeat do	ANKRD46	8.39	8.43	8.60
210032_s_at	sperm associated	SPAG6	4.32	4.34	4.29
215880_at	N-acetylglucosam	NAGLU	6.03	5.90	5.68
206347_at	pyruvate dehydro	PDK3	5.19	4.78	4.84
210109_at	chromosome 7 op	C7orf54	6.04	6.39	6.00
219060_at	WDYHV motif con	WDYHV1	9.61	9.52	9.60
214863_at	hypothetical prote	LOC100287602	4.71	4.87	4.66
204843_s_at	protein kinase, cA	PRKAR2A	7.45	7.31	7.31
200630_x_at	SET nuclear oncog	SET	13.36	13.39	13.39
213115_at	ATG4 autophagy r	ATG4A	7.26	7.30	7.48
202241_at	tribbles homolog	TRIB1	9.70	9.82	9.70
216388_s_at	leukotriene B4 rec	LTB4R	6.54	6.76	6.41
207017_at	RAB27B, member	RAB27B	4.71	4.84	4.80
208450_at	lectin, galactoside	LGALS2	4.80	4.71	5.07
205108_s_at	apolipoprotein B (	APOB	4.43	4.40	4.54
218488_at	eukaryotic transla	EIF2B3	9.70	9.64	9.64
216213_at	NIMA (never in m	NEK1	3.66	3.68	3.56
208686_s_at	bromodomain cor	BRD2	8.79	8.76	8.64
211040_x_at	G-2 and S-phase e	GTSE1	10.01	10.13	9.99
201865_x_at	nuclear receptor s	NR3C1	12.07	11.95	12.10
213966_at	High-mobility grou	HMG20B	4.87	4.85	4.61
219700_at	plexin domain cor	PLXDC1	3.67	3.67	3.61
219849_at	zinc finger protein	ZNF671	6.59	6.50	6.46
218741_at	centromere prote	CENPM	10.25	10.05	10.05
214088_s_at	fucosyltransferase	FUT3	5.47	4.99	5.40
211603_s_at	ets variant 4	ETV4	7.88	7.61	7.61
219434_at	triggering recepto	TREM1	3.90	3.85	3.73
209779_at	LLP homolog, long	LLPH	6.06	6.50	6.33
201784_s_at	chromosome 11 o	C11orf58	11.60	11.65	11.82
202086_at	myxovirus (influer	MX1	5.68	5.79	5.73
215599_at	glucuronidase, be	GUSBP3	6.47	6.61	6.69
217922_at	---	---	9.45	9.56	9.50
208091_s_at	vesicular, overexp	VOPP1	10.62	10.55	10.63
207433_at	interleukin 10	IL10	4.07	4.00	3.77
220093_at	anthrax toxin rece	ANTXR1	6.08	6.09	5.86
201966_at	NADH dehydroger	NDUFS2	10.60	10.59	10.53
201362_at	influenza virus NS	IVNS1ABP	8.88	8.90	9.12

208799_at	proteasome (pros	PSMB5	11.42	11.57	11.55
219040_at	coronin 7	CORO7	6.27	6.10	6.22
221971_x_at	ArfGAP with GTPa	AGAP10 /// AGAP4	8.62	8.54	8.51
217711_at	TEK tyrosine kinas	TEK	5.05	5.01	5.08
217757_at	alpha-2-macroglo	A2M	4.05	3.71	3.80
212246_at	multiple coagulati	MCFD2	8.94	9.00	9.10
221356_x_at	purinergic recepto	P2RX2	5.53	5.37	5.62
215393_s_at	COBL-like 1	COBLL1	5.17	4.66	4.78
203269_at	neutral sphingom	NSMAF	10.99	11.02	11.16
209715_at	chromobox homo	CBX5	9.26	9.22	9.30
221458_at	5-hydroxytryptam	HTR1F	3.87	4.02	3.70
209619_at	CD74 molecule, m	CD74	5.42	5.33	5.41
203521_s_at	zinc finger protein	ZNF318	7.38	7.57	7.41
217003_s_at	ADAM metallopep	ADAM5P	3.81	3.64	3.97
214500_at	H2A histone famil	H2AFY	9.06	9.11	9.15
212579_at	structural mainte	SMCHD1	8.40	8.79	9.02
210224_at	major histocompa	MR1	7.00	7.04	6.99
216984_x_at	immunoglobulin l	IGLV2-23 /// LOC1	5.16	5.11	4.90
211422_at	transient recepto	TRPM3	4.72	4.74	4.32
219669_at	CD177 molecule	CD177	5.78	5.75	5.72
212980_at	ubiquitin specific	USP34	6.32	6.64	6.50
221114_at	ameloblastin (ena	AMBN	5.16	5.06	5.30
210479_s_at	RAR-related orpha	RORA	3.30	3.71	3.30
209339_at	seven in absentia	SIAH2	8.71	8.52	8.63
203439_s_at	stanniocalcin 2	STC2	10.33	9.97	10.26
201124_at	integrin, beta 5	ITGB5	7.09	7.27	7.16
219107_at	brevican	BCAN	5.89	5.57	5.70
214016_s_at	splicing factor pro	SFPQ	9.25	9.26	9.35
204196_x_at	PBX/knotted 1 ho	PKNOX1	5.55	5.38	5.16
220897_at	---	---	3.68	3.39	3.69
212599_at	autism susceptibil	AUTS2	4.14	3.91	3.81
211185_s_at	splicing factor 3b,	SF3B1	11.79	11.91	11.99
200082_s_at	ribosomal protein	RPS7	14.29	14.32	14.35
214417_s_at	Fetuin B	FETUB	4.61	4.82	4.42
206770_s_at	solute carrier fam	SLC35A3	7.77	8.14	8.33
208273_at	zinc finger protein	ZNF695	3.62	3.30	3.39
208915_s_at	golgi-associated, g	GGA2	7.04	7.19	7.18
208175_s_at	dentin matrix acid	DMP1	3.42	3.16	3.09
205196_s_at	adaptor-related p	AP1S1	9.05	9.16	9.11
205367_at	SH2B adaptor pro	SH2B2	6.70	6.86	6.50
205154_at	leucine rich repea	LRRN2	6.91	6.76	6.49
217257_at	SH3-domain bindi	SH3BP2	3.71	3.63	4.01
218578_at	cell division cycle	CDC73	8.77	8.71	9.04
204955_at	sushi-repeat-cont	SRPX	11.04	11.02	11.17
201170_s_at	basic helix-loop-h	BHLHE40	8.70	9.02	8.96
221828_s_at	family with seque	FAM125B	5.88	6.40	6.02
201037_at	phosphofructokin	PFKP	10.78	10.74	10.65

214210_at	solute carrier fam	SLC25A17	7.72	7.84	7.47
218807_at	vav 3 guanine nuc	VAV3	8.02	8.11	8.19
222190_s_at	chromosome 16 c	C16orf58	7.56	7.52	7.58
213100_at	Unc-5 homolog B	UNC5B	8.49	8.16	8.48
207866_at	bone morphogen	BMP8A	4.78	4.61	4.39
218492_s_at	THAP domain con	THAP7	8.55	8.42	8.35
216849_at	---	---	5.29	5.54	5.20
219675_s_at	UDP-glucuronate	UXS1	9.11	9.14	9.20
208805_at	proteasome (pros	PSMA6	13.11	13.10	13.10
212365_at	myosin IB	MYO1B	9.41	9.49	9.50
208711_s_at	cyclin D1	CCND1	9.58	9.75	9.68
220561_at	insulin-like growt	IGF2AS	6.68	6.40	6.65
205606_at	low density lipopr	LRP6	6.37	6.44	6.50
204722_at	sodium channel, v	SCN3B	3.57	3.98	3.65
208032_s_at	glutamate recept	GRIA3	5.58	5.58	5.41
219452_at	dipeptidase 2	DPEP2	4.71	4.88	4.27
217972_at	coiled-coil-helix-c	CHCHD3	10.40	10.44	10.54
218464_s_at	chromosome 17 c	C17orf63	8.85	8.87	8.80
218025_s_at	peroxisomal D3,D	PECI	10.69	10.72	10.80
207535_s_at	nuclear factor of k	NFKB2	7.14	7.29	7.11
202427_s_at	brain protein 44	BRP44	10.98	11.02	11.03
211492_s_at	adrenergic, alpha-	ADRA1A	3.66	3.72	3.76
208303_s_at	cytokine receptor	CRLF2	5.82	6.07	5.74
202679_at	Niemann-Pick dis	NPC1	10.29	10.23	10.38
201986_at	mediator complex	MED13	9.27	9.30	9.45
201751_at	Josephin domain	JOSD1	10.75	10.61	10.67
208133_at	replication factor	RFC1	4.48	4.72	4.50
213093_at	protein kinase C, a	PRKCA	10.32	10.28	10.41
205227_at	interleukin 1 rece	IL1RAP	8.26	8.04	8.30
220475_at	solute carrier fam	SLC28A3	4.49	4.44	4.34
214539_at	serpin peptidase i	SERPINB10	3.30	3.37	3.45
204127_at	replication factor	RFC3	10.47	10.50	10.67
212896_at	superkiller virali	SKIV2L2	11.27	11.29	11.30
203444_s_at	metastasis associa	MTA2	6.57	6.53	6.33
202013_s_at	exostosin 2	EXT2	8.78	8.83	8.98
220537_at	myotubularin rela	MTMR8	3.51	3.55	3.25
203824_at	tetraspanin 8	TSPAN8	3.97	3.97	3.84
208586_s_at	synovial sarcoma,	SSX4 /// SSX4B	4.07	4.01	3.77
216436_at	---	---	3.74	3.39	3.21
210961_s_at	adrenergic, alpha-	ADRA1D	4.14	3.93	3.87
AFFX-BioB-3_at	---	---	9.32	9.32	9.31
215494_at	---	---	3.72	4.08	3.78
200611_s_at	WD repeat domai	WDR1	10.47	10.47	10.31
202624_s_at	calcineurin bindin	CABIN1	7.18	7.47	7.13
220553_s_at	PRP39 pre-mRNA	PRPF39	8.58	8.82	9.01
208283_at	G antigen 1	GAGE1	4.05	3.82	3.96
204829_s_at	folate receptor 2 (	FOLR2	7.12	7.08	6.80

205580_s_at	histamine recepto	HRH1	8.08	8.24	8.19
202113_s_at	sorting nexin 2	SNX2	9.65	9.63	9.68
219683_at	frizzled homolog 3	FZD3	7.47	7.74	7.79
210311_at	fibroblast growth	FGF5	5.75	5.57	5.50
205397_x_at	SMAD family men	SMAD3	6.67	6.51	6.42
209008_x_at	keratin 8	KRT8	9.80	9.72	9.73
209149_s_at	transmembrane 9	TM9SF1	7.68	7.87	7.74
217225_x_at	NODAL modulator	NOMO1 /// NOMO	11.24	11.22	11.24
212279_at	transmembrane p	TMEM97	10.42	10.55	10.49
219992_at	tachykinin 3	TAC3	6.88	6.78	6.66
218898_at	family with seque	FAM57A	8.09	8.07	8.15
216545_at	---	---	5.15	4.88	5.05
216714_at	chemokine (C-C m	CCL13	3.85	3.76	3.87
217288_at	---	---	4.14	4.27	4.18
214594_x_at	ATPase, aminopho	ATP8B1	6.86	6.90	6.95
210556_at	nuclear factor of a	NFATC3	4.94	4.95	4.71
201902_s_at	YY1 transcription	YY1	6.33	6.66	6.68
203776_at	G patch domain a	GPKOW	8.18	8.08	8.29
220130_x_at	leukotriene B4 rec	LTB4R2	7.09	6.80	7.12
222119_s_at	F-box protein 11	FBXO11	9.37	9.32	9.56
207002_s_at	pleiomorphic ade	PLAGL1	8.22	8.38	8.56
212524_x_at	H2A histone famil	H2AFX	5.80	5.85	5.70
203337_x_at	integrin beta 1 bi	ITGB1BP1	6.60	6.71	6.83
216599_x_at	solute carrier fam	SLC22A6	5.79	5.41	5.45
210396_s_at	bolA homolog 2 (E	BOLA2 /// LOC440	9.86	9.81	10.05
218744_s_at	protein kinase C a	PACSIN3	7.76	7.46	7.27
206973_at	protein tyrosine p	PPFIA2	5.21	5.69	5.54
222075_s_at	ornithine decarbo	OAZ3	5.88	5.93	5.82
213898_at	RNA binding moti	RBM9	4.97	4.78	4.74
219320_at	myosin XIX	MYO19	7.88	7.91	7.95
211690_at	ribosomal protein	RPS6	4.48	4.38	4.48
205485_at	ryanodine recepto	RYR1	4.72	4.48	4.75
206890_at	interleukin 12 rec	IL12RB1	5.84	6.05	5.54
204240_s_at	structural mainte	SMC2	8.70	9.00	9.22
205120_s_at	sarcoglycan, beta	SGCB	9.39	9.40	9.37
211155_s_at	thrombopoietin	THPO	3.75	3.63	3.65
201765_s_at	hexosaminidase A	HEXA	9.46	9.44	9.52
218755_at	kinesin family me	KIF20A	10.79	11.03	10.86
210140_at	cystatin F (leukoc	CST7	5.81	5.78	5.28
204474_at	zinc finger protei	ZNF142	7.68	7.77	7.58
204319_s_at	regulator of G-pro	RGS10	10.18	10.24	10.21
220250_at	zinc finger protei	ZNF286A	4.31	4.00	3.97
209163_at	cytochrome b-561	CYB561	9.09	9.22	9.09
220194_at	NOP2/Sun domai	NSUN7	3.44	3.51	3.38
204739_at	centromere prote	CENPC1	6.99	7.30	7.32
216099_at	5-hydroxytryptam	HTR7P1	3.84	3.84	3.72
207577_at	5-hydroxytryptam	HTR4	3.52	3.74	3.66

213373_s_at	caspase 8, apopto	CASP8	9.52	9.64	9.91
214153_at	ELOVL family men	ELOVL5	5.80	5.74	5.72
200829_x_at	zinc finger protein	ZNF207	11.59	11.58	11.58
202130_at	RIO kinase 3 (yeas	RIOK3	10.42	10.30	10.46
210241_s_at	TP53 target 1 (nor	TP53TG1	7.24	7.06	7.28
213937_s_at	FtsJ homolog 1 (E.	FTSJ1	8.30	8.19	8.28
211484_s_at	Down syndrome c	DSCAM	5.62	5.64	5.61
205502_at	cytochrome P450,	CYP17A1	5.59	5.70	5.23
214096_s_at	serine hydroxyme	SHMT2	11.85	11.69	11.81
200098_s_at	anaphase promot	ANAPC5	11.66	11.67	11.65
207769_s_at	polyglutamine bin	PQBP1	9.64	9.44	9.55
204580_at	matrix metallopep	MMP12	3.37	3.32	3.41
217192_s_at	PR domain contai	PRDM1	6.82	6.72	6.64
222041_at	DPH1 homolog (S.	DPH1 /// OVCA2	7.51	7.55	7.40
209734_at	NCK-associated pr	NCKAP1L	5.63	5.71	5.63
205113_at	neurofilament, me	NEFM	3.76	3.39	3.56
220934_s_at	transmembrane p	TMEM223	9.44	9.38	9.51
206532_at	---	---	4.15	4.27	4.59
210412_at	glutamate recepto	GRIN2B	4.51	4.74	4.31
212821_at	pleckstrin homolo	PLEKHG3	7.14	7.23	7.00
218496_at	ribonuclease H1	RNASEH1	10.39	10.35	10.46
213471_at	nephronophthisis	NPHP4	6.83	6.70	6.76
219463_at	chromosome 20 c	C20orf103	4.81	4.55	4.47
206126_at	chemokine (C-X-C	CXCR5	4.71	4.54	4.60
213077_at	YTH domain conta	YTHDC2	9.14	9.41	9.35
202575_at	cellular retinoic ac	CRABP2	7.43	7.21	7.03
209830_s_at	solute carrier fam	SLC9A3R2	4.17	4.20	4.11
215413_at	exocyst complex c	EXOC7	6.76	6.86	6.73
208039_at	---	---	4.73	4.94	4.65
213950_s_at	---	---	4.75	4.35	4.64
206446_s_at	chymotrypsin-like	CELA2A /// CELA2B	4.36	4.31	4.46
211533_at	platelet-derived g	PDGFRA	3.61	3.15	3.00
209891_at	SPC25, NDC80 kin	SPC25	9.68	9.70	9.86
217627_at	zinc finger protein	ZNF573	6.39	6.54	6.62
217637_at	---	---	6.58	6.59	6.33
212060_at	U2-associated SR1	SR140	9.05	9.28	9.24
220317_at	lecithin retinol acy	LRAT	3.70	3.81	3.55
206108_s_at	splicing factor, arg	SFRS6	8.76	8.95	8.90
209806_at	histone cluster 1,	HIST1H2BK	9.66	9.63	9.55
213379_at	coenzyme Q2 hon	COQ2	10.12	10.13	10.28
214443_at	poliovirus recepto	PVR	6.78	6.72	6.78
212412_at	PDZ and LIM dom	PDLIM5	10.52	10.63	10.72
218970_s_at	cutC copper trans	CUTC	10.26	10.30	10.24
221429_x_at	testis expressed 1	TEX13A	4.43	4.39	4.16
212530_at	NIMA (never in m	NEK7	10.41	10.56	10.81
203792_x_at	polycomb group r	PCGF2	6.73	6.52	6.71
211796_s_at	T cell receptor bet	TRBC1	4.03	3.83	3.80

220515_at	dual specificity ph	DUSP21	4.14	4.01	4.09
208349_at	transient receptor	TRPA1	3.76	4.14	4.09
217455_s_at	somatostatin rece	SSTR2	4.89	4.78	4.78
206172_at	interleukin 13 rec	IL13RA2	4.03	4.15	4.05
217278_x_at	---	---	5.24	4.92	4.89
210196_s_at	pregnancy specific	PSG1	4.03	3.94	3.65
219969_at	taxilin gamma	TXLNG	7.36	7.18	7.26
204809_at	ClpX caseinolytic p	CLPX	9.40	9.51	9.79
203921_at	carbohydrate (N-a	CHST2	9.99	9.75	10.10
205337_at	dopachrome taut	DCT	3.57	3.15	3.26
217978_s_at	ubiquitin-conjugat	UBE2Q1	9.94	9.82	9.80
200770_s_at	laminin, gamma 1	LAMC1	8.51	8.32	8.60
215397_x_at	---	---	5.65	5.52	5.86
206523_at	cytohesin 3	CYTH3	6.45	6.39	6.64
201830_s_at	neuroepithelial ce	NET1	10.13	10.21	10.37
218446_s_at	family with seque	FAM18B	7.62	7.81	7.97
209335_at	decorin	DCN	6.16	6.21	6.42
202453_s_at	general transcript	GTF2H1	8.83	8.78	8.89
215582_x_at	minichromosome	MCM3AP	7.60	7.69	7.51
204459_at	cleavage stimulati	CSTF2	8.79	8.87	8.85
207665_at	ADAM metallopep	ADAM21 /// ADAM	4.64	4.87	4.62
218974_at	sine oculis binding	SOBP	4.11	3.87	3.80
213058_at	tetratricopeptide	TTC28	8.63	8.60	8.66
220708_at	---	---	5.22	5.39	5.45
218952_at	proprotein conver	PCSK1N	7.23	7.21	6.85
222269_at	apolipoprotein O-	APOOL	7.67	7.60	7.71
210989_at	laminin, alpha 4	LAMA4	3.54	3.48	3.39
216691_at	---	---	4.10	3.74	4.13
209073_s_at	numb homolog (D	NUMB	8.32	8.25	8.48
204490_s_at	CD44 molecule (In	CD44	10.06	9.90	9.98
216577_at	---	---	5.81	5.96	5.67
205408_at	myeloid/lymphoid	MLLT10	7.06	6.96	7.08
219924_s_at	zinc finger, MYM-t	ZMYM6	8.74	8.88	8.94
216844_at	zinc finger CCCH-t	ZC3H7B	3.84	3.71	3.72
220045_at	neurogenic differe	NEUROD6	4.64	4.70	4.54
208547_at	histone cluster 1,	HIST1H2BB	4.72	4.42	4.37
215304_at	---	---	5.86	5.83	6.00
206902_s_at	endo/exonuclease	EXOG	4.69	4.54	4.75
51192_at	slingshot homolog	SSH3	7.85	7.95	7.93
206269_at	glial cells missing	GCM1	3.11	3.34	3.34
201767_s_at	elaC homolog 2 (E	ELAC2	8.30	8.20	8.31
208570_at	wingless-type MM	WNT1	4.64	4.49	4.31
205736_at	phosphoglycerate	PGAM2	5.16	5.12	4.83
213739_at	---	---	6.69	6.69	6.70
213355_at	ST3 beta-galactos	ST3GAL6	9.07	9.17	9.35
220489_s_at	---	---	6.13	5.65	5.83
32029_at	peroxisomal bioge	PEX6	9.34	9.34	9.28

202318_s_at	SUMO1/sentrin sp	SENP6	9.56	9.66	9.76
218237_s_at	solute carrier fam	SLC38A1	12.38	12.29	12.37
220717_at	ADAM metallopep	ADAMTS20	3.53	3.47	3.38
209306_s_at	SWAP switching B	SWAP70	8.78	8.91	8.91
211098_x_at	transmembrane a	TMCO1	10.52	10.56	10.61
220499_at	fibronectin type II	FNDC8	5.96	5.73	5.91
208637_x_at	actinin, alpha 1	ACTN1	10.53	10.48	10.56
208728_s_at	cell division cycle	CDC42	10.54	10.54	10.56
210289_at	N-acetyltransfera	NAT8	5.07	4.90	4.81
203347_s_at	metal response el	MTF2	6.05	6.25	6.52
221435_x_at	hydroxypyruvate i	HYI	7.69	7.74	7.63
207791_s_at	RAB1A, member F	RAB1A	9.82	10.01	10.07
200675_at	CD81 molecule	CD81	11.59	11.55	11.59
212860_at	zinc finger, DHHC-	ZDHHC18	7.27	7.20	7.20
205873_at	phosphatidylinosi	PIGL	6.35	6.30	6.06
213732_at	---	---	5.07	5.30	5.09
205065_at	ectonucleotide py	ENPP1	4.00	3.66	3.61
208661_s_at	tetratricopeptide	TTC3	10.13	10.29	10.37
202040_s_at	lysine (K)-specific	KDM5A	9.55	9.45	9.48
209790_s_at	caspase 6, apopto	CASP6	8.09	8.32	8.30
220095_at	centlein, centroso	CNTLN	4.70	5.03	4.66
203465_at	mitochondrial ribo	MRPL19	10.01	10.10	10.11
220959_s_at	odorant binding p	OBP2A /// OBP2B	5.67	5.36	5.25
222150_s_at	pigeon homolog (	PION	4.09	3.90	4.03
219435_at	chromosome 17 o	C17orf68	5.95	6.09	6.08
208542_x_at	zinc finger protein	ZNF208	3.68	3.41	3.58
210028_s_at	origin recognition	ORC3L	9.67	9.61	9.70
220271_x_at	EF-hand calcium b	EFCAB6	6.24	5.92	6.24
206130_s_at	asialoglycoprotein	ASGR2	6.17	6.42	6.67
212669_at	calcium/calmodul	CAMK2G	6.94	7.05	6.91
214171_s_at	U2 small nuclear R	U2AF2	6.00	5.89	5.72
215745_at	---	---	4.70	4.73	4.56
207597_at	ADAM metallopep	ADAM18	3.63	3.82	3.60
209129_at	thyroid hormone	TRIP6	9.77	9.83	9.89
221884_at	MDS1 and EVI1 co	MECOM	9.34	9.26	9.27
211558_s_at	deoxyhypusine sy	DHPS	8.47	8.39	8.61
219465_at	apolipoprotein A-I	APOA2	4.31	4.27	4.00
217962_at	NOP10 ribonucleo	NOP10	12.46	12.54	12.46
207225_at	aralkylamine N-ac	AANAT	4.46	4.36	4.20
220265_at	G protein-couplec	GPR107	5.84	5.74	5.58
200091_s_at	ribosomal protein	RPS25	14.52	14.53	14.58
209229_s_at	SAPS domain fami	SAPS1	8.41	8.21	8.23
215498_s_at	mitogen-activatec	MAP2K3	7.70	7.92	7.83
221992_at	nuclear pore com	NPIPL2	7.24	7.36	7.34
212038_s_at	voltage-depender	VDAC1	12.28	12.30	12.28
200648_s_at	glutamate-ammo	GLUL	8.94	8.86	8.94
209313_at	GPN-loop GTPase	GPN1	10.29	10.39	10.36

207236_at	zinc finger protein	ZNF345	4.47	4.90	4.72
217087_at	chromosome 1 op	C1orf68	6.24	6.56	6.32
222129_at	family with seque	FAM134A	8.66	8.41	8.72
221531_at	WD repeat domai	WDR61	9.65	9.68	9.72
206416_at	zinc finger protein	ZNF205	8.37	8.19	8.18
203083_at	thrombospondin 2	THBS2	4.19	4.28	4.35
217879_at	cell division cycle	CDC27	9.47	9.59	9.55
219568_x_at	SRY (sex determin	SOX18	4.86	4.53	4.41
221547_at	PRP18 pre-mRNA	PRPF18	9.35	9.41	9.41
219172_at	ubiquitin domain	UBTD1	6.43	6.10	6.06
221146_at	---	---	4.20	3.99	3.66
211833_s_at	BCL2-associated X	BAX	8.72	8.64	8.74
216976_s_at	RYK receptor-like	RYK	8.83	8.82	8.97
200949_x_at	ribosomal protein	RPS20	14.42	14.39	14.44
215900_at	---	---	4.39	4.42	4.60
214177_s_at	pre-B-cell leukemi	PBXIP1	7.31	7.09	7.07
220023_at	apolipoprotein B4	APOB48R	5.15	5.14	5.24
221189_s_at	threonyl-tRNA syn	TARS2	7.87	7.94	7.94
213393_at	---	---	5.09	5.28	5.01
213897_s_at	mitochondrial ribo	MRPL23	10.55	10.57	10.64
218915_at	neurofibromin 2 (	NF2	9.67	9.70	9.65
211166_at	family with seque	FAM153A	6.28	6.27	6.66
208347_at	---	---	6.15	6.16	6.18
205941_s_at	collagen, type X, a	COL10A1	5.45	5.72	5.48
206486_at	lymphocyte-activa	LAG3	5.14	5.25	5.17
215463_at	olfactory receptor	OR7E24	4.23	3.92	4.03
204502_at	SAM domain and	SAMHD1	6.84	7.05	7.02
205530_at	electron-transferr	ETFDH	6.92	6.83	7.06
210567_s_at	S-phase kinase-as	SKP2	7.47	7.82	7.56
220898_at	---	---	3.95	3.85	4.24
213703_at	hypothetical prote	LOC150759	5.97	6.36	5.90
216043_x_at	---	---	6.57	6.47	6.69
219789_at	natriuretic peptide	NPR3	7.89	8.04	8.10
211440_x_at	cytochrome P450,	CYP3A43	3.46	3.58	3.55
203575_at	casein kinase 2, al	CSNK2A2	9.50	9.31	9.49
216372_at	---	---	5.66	5.11	5.31
200990_at	tripartite motif-co	TRIM28	9.23	9.29	9.23
209996_x_at	pericentriolar mat	PCM1	7.25	7.07	7.11
210864_x_at	hemochromatosis	HFE	6.83	6.76	6.36
212639_x_at	tubulin, alpha 1b	TUBA1B	14.67	14.69	14.60
213823_at	homeobox A11	HOXA11	6.37	6.28	6.48
219236_at	progestin and adip	PAQR6	6.18	6.48	6.00
208459_s_at	exportin 7	XPO7	7.21	6.93	6.73
221431_s_at	olfactory receptor	OR12D3	5.16	5.07	5.05
218884_s_at	GUF1 GTPase hom	GUF1	7.71	7.65	7.95
212671_s_at	major histocompa	HLA-DQA1 /// HLA	3.71	3.42	3.50
205126_at	vaccinia related ki	VRK2	9.91	9.82	9.96

215338_s_at	natural killer-tum	NKTR	8.67	8.82	8.58
211452_x_at	leucine rich repea	LRRFIP1	10.46	10.69	10.65
215206_at	---	---	6.40	6.87	6.30
213230_at	cerebellar degene	CDR2L	8.17	8.09	8.11
220495_s_at	thioredoxin doma	TXNDC15	9.67	9.73	9.71
220925_at	N(alpha)-acetyltra	NAA35	9.79	9.85	9.92
211787_s_at	eukaryotic transla	EIF4A1	12.98	13.06	13.05
213991_s_at	Heparan sulfate (g	HS3ST1	6.29	6.24	5.98
212338_at	myosin ID	MYO1D	4.96	4.73	4.85
212410_at	EF-hand domain fa	EFHA1	9.96	10.17	10.32
216286_at	---	---	4.30	4.44	4.23
218837_s_at	ubiquitin-conjugat	UBE2D4	7.92	8.11	8.01
217410_at	agrin	AGRN	6.83	6.67	6.56
202474_s_at	host cell factor C1	HCFC1	8.75	8.69	8.57
205094_at	peroxisomal bioge	PEX12	8.24	8.35	8.42
203734_at	forkhead box J2	FOXJ2	8.56	8.52	8.65
210763_x_at	natural cytotoxicit	NCR3	5.88	5.74	5.77
207261_at	cyclic nucleotide g	CNGA3	3.33	3.53	3.43
221869_at	zinc finger protein	ZNF512B	6.22	6.39	6.38
209792_s_at	kallikrein-related p	KLK10	4.80	4.47	4.86
221681_s_at	dentin sialophosp	DSPP	4.82	4.67	4.73
211162_x_at	stearoyl-CoA desa	SCD	8.35	8.47	8.39
220036_s_at	limb region 1 hom	LMBR1L	7.14	7.28	6.92
212320_at	tubulin, beta	TUBB	12.17	12.34	12.26
209849_s_at	RAD51 homolog C	RAD51C	9.84	9.83	9.84
215433_at	Dpy-19-like 1 (C. e	DPY19L1	5.47	5.25	4.99
215995_x_at	---	---	6.23	5.95	5.91
219542_at	NIMA (never in m	NEK11	6.75	6.84	6.40
218103_at	FtsJ homolog 3 (E.	FTSJ3	9.66	9.63	9.63
205685_at	CD86 molecule	CD86	7.14	7.22	6.61
214245_at	ribosomal protein	RPS14	5.02	4.84	4.96
210132_at	ephrin-A3	EFNA3	7.61	7.38	7.28
213740_s_at	hypothetical LOC3	LOC399904	6.46	5.99	5.95
208592_s_at	CD1e molecule	CD1E	6.44	6.11	6.35
217822_at	WW domain bindi	WBP11	10.79	10.86	11.01
211432_s_at	TYRO3 protein tyr	TYRO3	7.86	7.59	7.66
204826_at	cyclin F	CCNF	8.73	8.64	8.71
214696_at	chromosome 17 o	C17orf91	7.61	8.08	7.85
211977_at	G protein-couplec	GPR107	7.63	7.59	7.65
218604_at	LEM domain conta	LEMD3	9.48	9.56	9.47
216896_at	collagen, type IV,	COL4A3	3.91	3.39	3.65
222373_at	---	---	4.64	4.46	4.16
201574_at	eukaryotic transla	ETF1	12.09	12.13	12.16
208498_s_at	amylase, alpha 1A	AMY1A /// AMY1B	5.27	5.35	5.61
213607_x_at	NAD kinase	NADK	7.25	7.57	7.49
208646_at	ribosomal protein	RPS14P3	13.72	13.59	13.67
209483_s_at	---	---	5.34	5.07	5.10

210182_at	cortistatin	CORT	5.85	5.86	5.83
207530_s_at	cyclin-dependent	CDKN2B	5.50	5.27	5.21
208192_at	---	---	3.08	2.92	3.13
215162_at	Ral GTPase activat	RALGAPA1	4.68	4.88	5.44
213994_s_at	spondin 1, extrace	SPON1	3.34	3.40	3.33
203547_at	CD4 molecule	CD4	6.82	6.82	6.59
220121_at	lines homolog 1 (I	LINS1	6.33	6.06	6.25
212532_s_at	LSM12 homolog (S	LSM12	9.74	9.78	9.84
201246_s_at	OTU domain, ubic	OTUB1	6.68	6.55	6.42
212985_at	amyloid beta (A4)	APBB2	9.58	9.47	9.75
217505_at	kelch-like 23 (Dros	KLHL23	3.24	3.28	3.22
212644_s_at	mitogen-activated	MAPK1IP1L	9.29	9.31	9.35
216116_at	NCK interacting pr	NCKIPSD	5.41	5.31	5.45
212681_at	erythrocyte meml	EPB41L3	9.05	9.14	9.41
210073_at	ST8 alpha-N-acety	ST8SIA1	3.47	3.33	3.17
207218_at	coagulation factor	F9	3.64	3.80	3.55
203676_at	glucosamine (N-ac	GNS	6.31	6.34	6.12
201566_x_at	inhibitor of DNA b	ID2	6.44	6.48	6.46
211630_s_at	glutathione synth	GSS	8.29	8.13	8.26
200786_at	proteasome (pros	PSMB7	12.38	12.39	12.44
215832_x_at	phosphatidylinosi	PICALM	8.33	8.29	8.34
64438_at	chromosome 17 c	C17orf101	7.42	7.68	7.36
216082_at	sialidase 3 (memb	NEU3	5.94	5.42	5.67
222279_at	hypothetical LOC2	LOC285830	8.62	8.83	8.74
203798_s_at	visinin-like 1	VSNL1	4.72	4.34	4.65
214617_at	perforin 1 (pore fo	PRF1	7.02	7.22	6.93
211894_x_at	seizure related 6 h	SEZ6L	5.11	5.28	5.51
217199_s_at	signal transducer a	STAT2	7.07	6.85	6.82
206044_s_at	v-raf murine sarco	BRAF	6.06	5.83	6.02
214093_s_at	far upstream elem	FUBP1	8.56	8.77	8.72
214092_x_at	splicing factor, arg	SFRS14	9.13	9.26	9.32
213119_at	solute carrier fam	SLC36A1	8.92	8.85	8.62
221070_s_at	KIAA1967	KIAA1967	4.42	4.45	4.60
210282_at	zinc finger, MYM-t	ZMYM2	4.38	4.68	4.36
220949_s_at	chromosome 7 op	C7orf49	9.27	9.31	9.26
210186_s_at	FK506 binding pro	FKBP1A	9.11	9.13	9.06
216926_s_at	KIAA0892	KIAA0892	4.49	4.57	4.45
220421_at	butyrophilin-like 8	BTNL8	3.86	4.10	4.07
220110_s_at	nuclear RNA expo	NXF3	4.05	4.19	4.23
213413_at	stonin 1	STON1	4.41	4.66	4.48
213540_at	hydroxysteroid (1	HSD17B8	9.21	9.16	9.08
215736_at	---	---	4.50	4.37	4.45
204757_s_at	C2CD2-like	C2CD2L	6.94	6.88	7.23
220981_x_at	nuclear RNA expo	NXF2	5.67	5.64	5.51
204673_at	mucin 2, oligomer	MUC2	5.85	5.69	5.83
209644_x_at	cyclin-dependent	CDKN2A	6.49	6.70	6.68
221073_s_at	nucleotide-bindin	NOD1	7.14	7.30	7.53

205334_at	S100 calcium bind	S100A1	6.28	6.18	6.18
209316_s_at	HBS1-like (S. cere	HBS1L	10.07	10.14	10.13
210502_s_at	peptidylprolyl isor	PPIE	9.50	9.40	9.48
218052_s_at	ATPase type 13A1	ATP13A1	8.15	8.12	7.99
210589_s_at	glucosidase, beta,	GBAP1	8.64	8.60	8.54
214026_s_at	---	---	4.07	3.89	3.94
217093_at	ribonuclease, RNa	RNASE1	3.47	3.27	3.04
215436_at	Hydroxysteroid de	HSDL2	6.44	6.49	6.79
221057_at	spermatogenesis	SPATA1	3.17	3.30	3.29
216912_at	Rho guanine nucle	ARHGEF4	5.78	5.73	5.64
205496_at	KIAA0408	KIAA0408	3.27	3.20	3.32
207818_s_at	5-hydroxytryptam	HTR7	3.82	3.80	3.88
219887_at	coiled-coil domair	CCDC87	5.51	5.84	5.64
208299_at	calcium channel, v	CACNA1I	6.55	6.40	6.63
218221_at	aryl hydrocarbon	ARNT	8.02	8.09	8.14
222049_s_at	Retinol binding pr	RBP4	5.14	5.38	4.99
218196_at	osteopetrosis ass	OSTM1	8.47	8.46	8.66
217745_s_at	N(alpha)-acetyltra	NAA50	11.19	11.30	11.61
207061_at	endoplasmic retic	ERN1	5.41	5.17	5.11
216001_at	PRAME family me	PRAMEF12	4.96	4.83	4.58
220181_x_at	solute carrier fam	SLC30A5	8.54	8.62	8.57
213801_x_at	ribosomal protein	RPSA	15.12	15.07	15.06
212548_s_at	FRY-like	FRYL	8.86	8.91	8.98
212232_at	formin binding pr	FNBP4	9.52	9.52	9.47
215925_s_at	CD72 molecule	CD72	5.28	5.10	5.33
216950_s_at	Fc fragment of Ig	FCGR1A /// FCGR1	4.78	4.57	4.62
212373_at	fem-1 homolog b	FEM1B	8.32	8.31	8.41
218721_s_at	chromosome 1 op	C1orf27	8.93	9.08	9.28
217464_at	---	---	5.63	5.68	5.57
200820_at	proteasome (pros	PSMD8	11.74	11.69	11.85
218546_at	chromosome 1 op	C1orf115	7.39	7.55	7.38
216444_at	---	---	3.75	3.51	3.65
214520_at	forkhead box C2 (	FOXC2	6.56	6.48	6.37
215125_s_at	UDP glucuronosyl	UGT1A1 /// UGT1A	5.19	5.17	5.20
219294_at	centromere prote	CENPQ	7.11	7.26	7.63
219016_at	FAST kinase doma	FASTKD5	7.95	8.14	7.99
213543_at	sarcoglycan, delta	SGCD	4.06	4.02	4.33
216781_at	KIAA1751	KIAA1751	4.60	4.66	4.58
211865_s_at	fizzy/cell division	FZR1	5.78	5.56	5.87
221248_s_at	Wolf-Hirschhorn s	WHSC1L1	6.10	6.29	6.28
200616_s_at	malectin	MLEC	11.53	11.47	11.53
215891_s_at	GM2 ganglioside a	GM2A	4.67	5.06	4.80
217585_at	nebulette	NEBL	4.97	5.13	5.05
206095_s_at	splicing factor, arg	SFRS13A	10.37	10.50	10.55
217639_at	---	---	3.76	4.11	3.95
201980_s_at	Ras suppressor pr	RSU1	10.15	10.21	10.47
202944_at	N-acetylgalactosa	NAGA	7.83	8.03	7.91

210133_at	chemokine (C-C m	CCL11	3.76	3.70	3.72
219135_s_at	lipase maturation	LMF1	5.78	5.53	5.42
221899_at	NEDD4 binding pr	N4BP2L2	9.09	9.36	9.33
208859_s_at	alpha thalassemia	ATRX	8.02	8.35	8.36
206924_at	interleukin 11	IL11	4.59	4.31	4.26
205931_s_at	cAMP responsive	CREB5	8.06	8.04	8.34
207642_at	hypocretin (orexin	HCRT	5.11	5.02	4.97
217843_s_at	mediator complex	MED4	8.86	8.96	9.10
202162_s_at	CCR4-NOT transcr	CNOT8	7.98	8.11	8.10
201316_at	proteasome (pros	PSMA2	9.30	9.45	9.45
201750_s_at	endothelin conver	ECE1	7.82	7.76	7.72
209918_at	gamma-glutamylt	GGTLC2	5.93	5.81	6.04
201198_s_at	proteasome (pros	PSMD1	10.68	10.76	10.70
220313_at	G protein-couplec	GPR88	3.34	3.34	3.15
204213_at	polymeric immun	PIGR	6.85	6.85	6.50
213227_at	progesterone rece	PGRMC2	9.41	9.46	9.53
209869_at	adrenergic, alpha-	ADRA2A	3.87	3.62	3.42
206910_x_at	complement facto	CFHR2	4.77	4.57	4.67
205390_s_at	ankyrin 1, erythro	ANK1	5.45	5.59	5.35
207610_s_at	egf-like module co	EMR2	7.03	6.96	7.23
219664_s_at	2,4-dienoyl CoA re	DECR2	8.81	8.73	8.75
214517_at	keratin associated	KRTAP5-9	5.53	5.29	5.38
205708_s_at	transient receptor	TRPM2	6.37	6.26	6.12
205597_at	solute carrier fam	SLC44A4	4.59	4.53	4.82
210092_at	mago-nashi homo	MAGOH	8.70	8.65	8.81
221820_s_at	MYST histone ace	MYST1	9.33	9.27	9.29
220391_at	zinc finger and BT	ZBTB3	5.27	5.45	5.67
204708_at	mitogen-activated	MAPK4	4.99	4.69	4.99
214560_at	formyl peptide re	FPR3	4.18	4.00	4.09
207212_at	solute carrier fam	SLC9A3	4.96	4.94	5.02
212058_at	U2-associated SR1	SR140	10.85	11.02	11.02
202966_at	calpain 6	CAPN6	4.04	3.98	4.00
221181_at	---	---	5.61	5.29	5.16
215376_at	---	---	5.07	5.18	4.85
214553_s_at	cAMP-regulated p	ARPP19	8.57	8.66	8.59
221278_at	homeobox B8	HOXB8	4.22	4.48	4.19
214996_at	---	---	6.71	6.67	6.43
207389_at	glycoprotein Ib (p	GP1BA	5.73	5.80	5.62
200880_at	DnaJ (Hsp40) hom	DNAJA1	9.76	10.11	9.97
204949_at	intercellular adhe	ICAM3	8.29	8.34	8.37
210983_s_at	minichromosome	MCM7	9.79	9.85	9.69
211023_at	pyruvate dehydro	PDHB	11.29	11.34	11.38
215267_s_at	solute carrier fam	SLC8A2	5.19	5.11	4.95
206461_x_at	metallothionein 1	MT1H	13.04	13.11	13.06
65591_at	WD repeat domai	WDR48	7.86	8.01	8.02
205920_at	solute carrier fam	SLC6A6	4.97	4.94	4.52
203870_at	ubiquitin specific	USP46	7.69	7.90	8.05

210730_s_at	neuropeptide Y re	NPY2R	3.84	3.73	3.66
202127_at	PRP4 pre-mRNA p	PRPF4B	7.84	8.23	8.23
216313_at	protocadherin bet	PCDHB17	3.80	3.37	3.33
210364_at	sodium channel, v	SCN2B	6.57	6.12	6.13
202797_at	SAC1 suppressor c	SACM1L	10.24	10.40	10.35
206190_at	G protein-couplec	GPR17	4.24	4.25	4.00
208391_s_at	glucagon-like pep	GLP1R	4.44	4.15	4.06
217597_x_at	RAB40B, member	RAB40B	7.51	7.33	7.20
204714_s_at	coagulation factor	F5	4.35	3.96	4.29
204021_s_at	purine-rich eleme	PURA	9.10	9.13	9.01
212119_at	ras homolog gene	RHOQ	10.52	10.58	10.59
202244_at	proteasome (pros	PSMB4	13.12	13.07	13.07
216483_s_at	chromosome 19 c	C19orf10	9.69	9.48	9.62
216022_at	---	---	4.93	5.31	4.75
216816_at	---	---	5.29	5.14	5.37
212625_at	syntaxin 10	STX10	8.89	8.91	8.92
200839_s_at	cathepsin B	CTSB	10.30	10.23	10.22
201462_at	secernin 1	SCRN1	10.56	10.52	10.61
215073_s_at	nuclear receptor s	NR2F2	6.06	5.99	6.12
217126_at	---	---	3.36	3.52	3.30
213022_s_at	utrophin	UTRN	6.53	6.62	6.47
215779_s_at	histone cluster 1,	HIST1H2BG	5.56	5.33	5.61
202359_s_at	sorting nexin 19	SNX19	7.56	7.75	8.07
206964_at	N-acetyltransferas	NAT8B	5.07	4.96	5.19
218230_at	ADP-ribosylation f	ARFIP1	9.07	9.12	9.17
210259_s_at	distal-less homeol	DLX4	4.43	4.58	4.25
206132_at	mutated in colore	MCC	6.56	6.98	6.51
205605_at	homeobox D9	HOXD9	6.34	6.14	6.11
212019_at	ribosomal L1 dom	RSL1D1	6.60	6.56	6.69
210788_s_at	dehydrogenase/re	DHRS7	9.66	9.70	9.68
213217_at	adenylate cyclase	ADCY2	5.20	5.47	5.32
217283_at	---	---	6.03	5.80	5.90
214554_at	histone cluster 1,	HIST1H2AL	3.85	3.86	3.89
201546_at	thyroid hormone	TRIP12	11.07	11.09	11.04
214025_at	DEAD (Asp-Glu-Al	DDX28	6.20	5.97	5.92
205689_at	pecanex-like 2 (Dr	PCNXL2	6.46	6.23	6.40
201358_s_at	coatomer protein	COPB1	12.08	12.19	12.33
211526_s_at	regulator of telom	RTEL1	3.97	3.94	3.82
216610_at	solute carrier fam	SLC6A2	3.76	3.40	3.77
218532_s_at	family with seque	FAM134B	6.79	6.73	6.63
212606_at	WD repeat and FY	WDFY3	8.22	8.17	8.13
220667_at	---	---	4.12	4.24	4.47
201406_at	ribosomal protein	RPL36A	14.41	14.36	14.43
211744_s_at	CD58 molecule	CD58	8.87	9.04	9.24
201049_s_at	ribosomal protein	RPS18	15.16	15.18	15.22
201249_at	solute carrier fam	SLC2A1	4.91	4.80	4.66
211936_at	heat shock 70kDa	HSPA5	12.04	11.97	12.14

206549_at	insulin-like 4 (plac	INSL4	5.36	5.16	5.08
208741_at	Sin3A-associated	SAP18	7.08	7.24	7.20
211430_s_at	immunoglobulin h	IGH@ /// IGHG1 //	4.79	4.26	4.57
213448_at	---	---	5.90	6.33	6.26
201340_s_at	ectodermal-neura	ENC1	7.85	7.96	7.99
211649_x_at	immunoglobulin h	IGH@ /// IGHA1 //	5.14	5.05	4.80
222067_x_at	histone cluster 1,	HIST1H2BD	8.05	8.06	8.01
209703_x_at	methyltransferase	METTL7A	5.79	5.88	5.84
210152_at	leukocyte immuno	LILRB4	4.98	5.01	5.33
209211_at	Kruppel-like facto	KLF5	8.37	8.12	8.78
210748_at	---	---	5.78	5.59	5.81
216850_at	small nuclear ribo	SNRPN	4.98	5.11	4.90
217859_s_at	solute carrier fam	SLC39A9	6.33	6.22	6.09
213276_at	calcium/calmodul	CAMK2B	6.64	6.64	6.10
216321_s_at	nuclear receptor s	NR3C1	11.42	11.26	11.70
207755_at	---	---	4.53	4.55	4.13
205393_s_at	CHK1 checkpoint	CHEK1	7.80	7.81	7.92
205082_s_at	aldehyde oxidase	AOX1	9.27	9.58	9.62
203202_at	KRR1, small subur	KRR1	9.45	9.50	9.57
219319_at	hypoxia inducible	HIF3A	6.83	6.41	6.60
217071_s_at	methylenetetrahy	MTHFR	6.68	6.79	6.68
201210_at	DEAD (Asp-Glu-Al	DDX3X	11.90	11.92	12.01
215296_at	CDC42 binding pro	CDC42BPA	6.86	6.88	6.86
215980_s_at	immunoglobulin r	IGHMBP2	7.03	7.07	6.99
219235_s_at	phosphatase and	PHACTR4	8.40	8.34	8.55
31835_at	histidine-rich glyc	HRG	4.76	4.85	4.63
203410_at	adaptor-related p	AP3M2	9.17	9.23	9.17
212791_at	chromosome 1 op	C1orf216	8.28	8.36	8.19
212748_at	megakaryoblastic	MKL1	6.94	7.02	6.97
222052_at	chromosome 19 c	C19orf54	8.24	8.11	8.33
210402_at	potassium inward	KCNJ1	4.17	4.32	3.86
205989_s_at	myelin oligodendr	MOG	3.60	3.57	3.60
209064_x_at	poly(A) binding pr	PAIP1	9.98	10.04	10.11
204526_s_at	TBC1 domain fam	TBC1D8	9.53	9.55	9.62
218956_s_at	pentatricopeptide	PTCD1	8.45	8.26	8.22
213944_x_at	guanine nucleotid	GNA11	8.95	9.05	9.12
218911_at	YEATS domain cor	YEATS4	8.17	8.09	8.73
31845_at	E74-like factor 4 (	ELF4	8.46	8.44	8.49
219058_x_at	tubulointerstitial	TINAGL1	8.16	8.10	8.00
221083_at	potassium voltage	KCNQ4	6.72	6.32	6.57
214494_s_at	spastic paraplegia	SPG7	9.08	9.05	9.00
208342_x_at	chorionic somator	CSH2	5.00	5.19	4.86
220699_s_at	kinesin heavy chai	KIF2A	3.67	3.47	3.53
220810_at	chloride channel a	CLCA3P	3.02	3.05	3.06
208968_s_at	cytokine induced	CIAPIN1	10.84	10.79	10.76
216935_at	chromosome 1 op	C1orf46	5.07	4.83	4.81
206837_at	ALX homeobox 1	ALX1	5.98	6.02	5.96

204647_at	homer homolog 3	HOMER3	9.19	9.11	9.28
213382_at	macrophage stimu	MST1 /// MST1P2	3.32	3.24	3.24
204626_s_at	integrin, beta 3 (p	ITGB3	6.76	6.76	6.66
207339_s_at	lymphotoxin beta	LTB	6.81	6.55	6.47
207832_at	BAI1-associated p	BAIAP2	6.17	6.02	5.99
217439_at	---	---	5.91	5.62	5.43
211005_at	linker for activatic	LAT /// SPNS1	5.89	6.13	5.95
217081_at	olfactory receptor	OR2H2	5.92	5.58	5.52
222382_x_at	nucleoporin 205kI	NUP205	7.23	7.03	7.07
219753_at	stromal antigen 3	STAG3	6.91	6.86	6.81
222007_s_at	hypothetical prot	LOC100292534	6.22	6.11	5.78
203382_s_at	apolipoprotein E	APOE	7.69	7.39	7.33
222311_s_at	splicing factor, arg	SFRS15	6.46	6.45	6.32
213596_at	caspase 4, apopto	CASP4	6.64	6.51	6.60
37152_at	peroxisome prolif	PPARD	8.91	8.93	9.14
206498_at	oculocutaneous a	OCA2	4.06	4.20	3.82
212000_at	splicing factor, arg	SFRS14	7.21	7.18	7.05
221393_at	trace amine assoc	TAAR3	4.04	4.04	4.03
219095_at	JMJD7-PLA2G4B r	JMJD7-PLA2G4B //	7.31	7.21	7.03
221795_at	neurotrophic tyro	NTRK2	3.48	3.62	3.31
208582_s_at	double homeobox	DUX1 /// DUX3 ///	4.29	4.14	3.87
219424_at	Epstein-Barr virus	EBI3	5.68	5.12	5.45
200754_x_at	splicing factor, arg	SFRS2	12.23	12.43	12.17
218490_s_at	zinc finger protein	ZNF302	8.06	8.48	8.41
204035_at	secretogranin II	SCG2	4.34	4.06	4.21
219694_at	family with seque	FAM105A	3.95	4.03	4.17
215349_at	BTB (POZ) domain	BTBD18	4.53	4.88	4.53
214884_at	MCF.2 cell line de	MCF2	3.90	3.81	3.83
215717_s_at	fibrillin 2	FBN2	3.60	3.48	3.18
215815_at	BUD31 homolog (	BUD31	6.27	6.20	6.32
201017_at	eukaryotic transla	EIF1AX	10.14	10.14	10.19
203712_at	KIAA0020	KIAA0020	9.44	9.47	9.52
206444_at	phosphodiesteras	PDE1B	5.89	5.96	5.71
214355_x_at	CTAGE family, me	CTAGE4 /// CTAGE	7.06	7.24	7.00
217600_at	Signal peptide, CU	SCUBE3	5.08	5.66	5.14
206042_x_at	small nuclear ribo	SNRPN /// SNURF	8.66	8.57	8.74
212617_at	zinc finger protein	ZNF609	6.64	6.63	6.63
214938_x_at	high-mobility grou	HMGB1	13.96	14.03	13.98
215265_at	empty spiracles h	EMX1	6.80	6.74	6.90
200067_x_at	sorting nexin 3	SNX3	12.21	12.24	12.27
204400_at	embryonal Fyn-as	EFS	3.85	3.52	3.94
213291_s_at	ubiquitin protein l	UBE3A	9.99	9.96	10.03
218042_at	COP9 constitutive	COPS4	10.93	11.00	11.05
205506_at	villin 1	VIL1	5.03	4.47	4.51
204282_s_at	phenylalanyl-tRN/	FARS2	8.66	8.51	8.56
212738_at	Rho GTPase activa	ARHGAP19	9.18	9.14	9.16
218484_at	NADH dehydroger	NDUFA4L2	6.58	6.22	6.31

220440_at	lectin, galactoside	LGALS13	3.69	3.50	3.73
218416_s_at	solute carrier fam	SLC48A1	6.77	6.54	6.45
212879_x_at	protein inhibitor c	PIAS4	7.50	7.47	7.43
201224_s_at	serine/arginine re	SRRM1	8.98	8.94	8.82
213681_at	cysteine/histidine	CYHR1	7.01	7.00	6.91
217258_x_at	immunoglobulin la	IGLV1-44 /// LOC1	6.44	6.55	6.28
208756_at	eukaryotic transla	EIF3I	12.63	12.73	12.80
214787_at	DENN/MADD dom	DENND4A	7.08	7.13	7.29
209155_s_at	5'-nucleotidase, cy	NT5C2	9.35	9.24	9.35
211645_x_at	---	---	4.98	4.85	4.81
220528_at	vanin 3	VNN3	4.41	4.20	4.36
217907_at	mitochondrial ribo	MRPL18	11.33	11.37	11.40
205286_at	transcription facto	TFAP2C	7.94	8.17	8.08
221616_s_at	TAF9B RNA polym	TAF9B	5.66	5.75	5.81
204299_at	splicing factor, arg	SFRS13A	9.31	9.52	9.72
211611_s_at	activating transcri	ATF6B /// TNXB	6.35	6.26	6.15
202519_at	MLX interacting p	MLXIP	9.03	9.28	9.17
207496_at	membrane-spann	MS4A2	3.54	3.43	3.47
214360_at	SWI/SNF related,	SMARCA4	4.95	4.91	4.62
204345_at	collagen, type XVI	COL16A1	10.03	9.99	9.98
219399_at	lin-7 homolog C (C	LIN7C	8.51	8.54	8.73
220980_s_at	ADP-dependent g	ADPGK	9.94	10.03	9.91
219248_at	THUMP domain co	THUMPD2	8.52	8.76	8.66
216746_at	---	---	3.65	3.99	3.86
214370_at	S100 calcium bind	S100A8	3.49	3.61	3.42
205128_x_at	prostaglandin-end	PTGS1	7.30	7.08	7.35
210608_s_at	fucosyltransferase	FUT2	5.71	5.58	5.73
217034_at	---	---	6.43	6.14	6.20
206697_s_at	haptoglobin	HP	5.90	5.94	5.64
203393_at	hairy and enhance	HES1	4.85	4.67	4.51
220787_at	---	---	3.22	3.24	3.28
221053_s_at	tudor and KH dom	TDRKH	4.10	4.37	4.13
207384_at	peptidoglycan rec	PGLYRP1	6.44	6.63	6.25
217704_x_at	Suppressor of zest	SUZ12P	5.51	5.61	5.46
213981_at	catechol-O-methy	COMT	7.55	7.55	7.66
205409_at	FOS-like antigen 2	FOSL2	6.25	5.88	5.71
211033_s_at	peroxisomal bioge	PEX7	7.91	8.05	8.05
222365_at	---	---	6.57	6.81	6.69
203385_at	diacylglycerol kina	DGKA	6.60	6.87	6.89
211426_x_at	guanine nucleotid	GNAQ	7.13	7.12	6.91
200976_s_at	Tax1 (human T-ce	TAX1BP1	11.05	11.07	11.09
207965_at	neurogenin 3	NEUROG3	5.46	5.34	5.35
215729_s_at	vestigial like 1 (Dr	VGLL1	3.56	3.33	3.23
219698_s_at	methyltransferase	METTL4	8.36	8.34	8.49
206709_x_at	glutamic-pyruvate	GPT	7.45	7.55	7.26
208098_at	olfactory receptor	OR12D3 /// OR5V1	3.45	3.41	3.33
213363_at	Carbonic anhydra:	CA5BP	5.88	5.78	5.59

212544_at	zinc finger, HIT typ	ZNHIT3	10.01	9.97	10.12
214485_at	outer dense fiber	ODF1	4.98	5.02	4.81
220598_at	caspase recruitme	CARD14	5.98	5.52	5.51
201914_s_at	SEC63 homolog (S	SEC63	9.57	9.53	9.69
201647_s_at	scavenger recepto	SCARB2	5.57	5.50	5.55
220393_at	lengsin, lens prote	LGSN	3.13	3.39	3.05
213535_s_at	ubiquitin-conjugat	UBE2I	11.08	11.06	11.08
210717_at	---	---	4.38	4.56	4.23
202054_s_at	aldehyde dehydro	ALDH3A2	9.67	9.64	9.56
201298_s_at	MOB1, Mps One B	MOBKL1B	9.70	9.82	9.77
211581_x_at	leukocyte specific	LST1	5.34	5.57	5.53
200689_x_at	eukaryotic transla	EEF1G	14.48	14.35	14.39
203527_s_at	adenomatous pol	APC	6.23	6.36	6.28
208470_s_at	haptoglobin /// ha	HP /// HPR	5.51	5.58	5.58
208796_s_at	cyclin G1	CCNG1	11.81	11.80	11.88
204608_at	argininosuccinate	ASL	8.66	8.45	8.47
209812_x_at	caspase 2, apopto	CASP2	6.94	6.69	6.70
211074_at	folate receptor 1 (	FOLR1	10.21	10.20	10.42
211065_x_at	phosphofructokin	PFKL	8.55	8.35	8.32
212659_s_at	interleukin 1 rece	IL1RN	5.98	5.61	5.82
203152_at	mitochondrial ribo	MRPL40	11.20	11.17	11.25
215278_at	---	---	5.94	5.87	5.90
202773_s_at	splicing factor, arg	SFRS8	8.43	8.25	8.14
213406_at	WD repeat and SO	WSB1	4.00	4.08	4.02
213674_x_at	immunoglobulin h	IGHD	5.25	5.29	5.22
206906_at	intercellular adhe	ICAM5	6.37	6.36	6.24
215740_at	hypothetical prote	LOC100130741	5.32	4.89	4.97
208477_at	potassium voltage	KCNC1	3.76	3.92	3.67
219254_at	chromosome 17 o	C17orf101	7.67	7.51	7.61
204484_at	phosphoinositide-	PIK3C2B	7.39	7.54	7.38
219890_at	C-type lectin dom	CLEC5A	3.38	3.51	3.32
219268_at	ethanolamine kina	ETNK2	6.43	6.37	6.43
207933_at	zona pellucida gly	ZP2	5.22	4.84	4.96
205999_x_at	cytochrome P450,	CYP3A4	5.51	5.41	5.31
207327_at	eyes absent homo	EYA4	4.75	4.65	4.60
217452_s_at	UDP-Gal:betaGlcN	B3GALT2	3.14	3.25	3.11
215455_at	timeless homolog	TIMELESS	5.34	5.61	5.22
205156_s_at	amiloride-sensitiv	ACCN2	5.63	5.65	5.50
205950_s_at	carbonic anhydras	CA1	5.31	5.20	4.81
202376_at	serpin peptidase i	SERPINA3	7.15	6.65	6.98
203055_s_at	Rho guanine nucle	ARHGEF1	7.37	7.21	7.24
211883_x_at	carcinoembryonic	CEACAM1	3.93	4.01	3.82
215152_at	v-myb myeloblast	MYB	3.73	3.31	3.38
204745_x_at	metallothionein 1	MT1G	12.59	12.71	12.56
210560_at	gastrulation brain	GBX2	5.06	5.00	5.10
211830_s_at	calcium channel, v	CACNA1I	6.23	6.30	5.96
200691_s_at	heat shock 70kDa	HSPA9	12.24	12.10	12.22

210242_x_at	suppressor of tum	ST20	7.27	7.44	7.30
215319_at	ATPase, aminopho	ATP8B3	3.72	3.64	3.61
41553_at	Oxidative stress ir	OSGIN2	4.44	4.53	4.34
219668_at	ganglioside-induc	GDAP1L1	6.35	5.85	6.11
203068_at	kelch-like 21 (Dro:	KLHL21	7.32	7.29	7.59
204985_s_at	trafficking protein	TRAPPC6A	8.67	8.56	8.63
203828_s_at	interleukin 32	IL32	9.06	9.02	9.07
208488_s_at	complement com	CR1	3.88	3.79	3.99
208296_x_at	tumor necrosis fa	TNFAIP8	7.40	7.37	7.46
221741_s_at	YTH domain famil	YTHDF1	10.21	10.13	10.12
205376_at	inositol polyphosp	INPP4B	7.38	7.33	7.45
204033_at	thyroid hormone	TRIP13	11.41	11.50	11.42
218401_s_at	zinc finger proteir	ZNF281	9.01	9.10	9.10
203780_at	myelin protein zer	MPZL2	3.29	3.21	3.03
216829_at	immunoglobulin k	IGK@ /// IGKC ///	4.20	4.33	4.32
217936_at	Rho GTPase activa	ARHGAP5	9.11	9.25	9.32
221517_s_at	mediator complex	MED17	9.08	9.17	9.15
211299_s_at	flotillin 2	FLOT2	5.85	5.60	5.76
221027_s_at	phospholipase A2	PLA2G12A	7.41	7.21	7.60
218933_at	spermatogenesis a	SPATA5L1	8.12	8.16	8.25
219255_x_at	interleukin 17 rec	IL17RB	4.29	4.36	4.04
218322_s_at	acyl-CoA syntheta	ACSL5	7.46	7.16	7.57
213504_at	COP9 constitutive	COPS6	9.33	9.19	9.09
210377_at	acyl-CoA syntheta	ACSM3	4.60	4.45	4.52
209965_s_at	RAD51-like 3 (S. ce	RAD51L3	6.80	6.76	6.69
211306_s_at	Fc fragment of IgA	FCAR	4.74	4.77	4.73
59433_at	hypothetical prote	LOC286434	6.80	6.69	6.91
215818_at	nudix (nucleoside	NUDT7	5.33	5.21	5.61
205584_at	asparagine-linked	ALG13	6.89	7.06	6.98
204317_at	G-2 and S-phase e	GTSE1	7.14	7.09	6.91
218302_at	presenilin enhanc	PSENEN	8.67	8.76	8.65
205840_x_at	growth hormone 1	GH1	4.56	4.86	4.31
205185_at	serine peptidase i	SPINK5	4.77	4.71	4.34
211313_s_at	bromodomain adj	BAZ1B	6.60	6.64	6.52
212086_x_at	lamin A/C	LMNA	11.15	11.16	11.15
216067_at	---	---	5.04	4.94	4.78
207714_s_at	serpin peptidase i	SERPINH1	9.11	8.80	9.06
212439_at	inositol hexakisph	IP6K1	8.58	8.42	8.27
208704_x_at	amyloid beta (A4)	APLP2	9.99	9.94	9.96
201461_s_at	mitogen-activatec	MAPKAPK2	5.20	4.94	4.74
205226_at	platelet-derived g	PDGFRL	5.05	5.38	5.08
215819_s_at	Rh blood group, C	RHCE /// RHD	5.19	5.25	5.25
214904_at	zinc finger proteir	ZNF41	4.07	4.22	4.30
205329_s_at	sorting nexin 4	SNX4	7.30	7.64	7.64
213777_s_at	---	---	5.71	5.55	5.40
202968_s_at	dual-specificity tyı	DYRK2	7.70	7.59	7.72
211177_s_at	thioredoxin reduc	TXNRD2	9.71	9.48	9.65

203677_s_at	TAR (HIV-1) RNA b	TARBP2	8.41	8.43	8.43
220354_at	similar to hCG177	LOC100134018 ///	4.78	5.18	4.98
209291_at	inhibitor of DNA b	ID4	3.42	3.39	3.22
220765_s_at	LIM and senescen	LIMS2	5.99	6.31	5.82
214519_s_at	relaxin 2	RLN2	3.41	3.59	3.59
206080_at	phospholipase C,	PLCH2	7.38	6.87	6.77
219327_s_at	G protein-couplec	GPRC5C	6.10	6.02	5.64
211037_s_at	membrane bound	MBOAT7	7.55	7.63	7.36
208666_s_at	suppression of tu	ST13	8.67	8.83	8.77
213417_at	T-box 2	TBX2	6.32	6.19	6.15
36907_at	mevalonate kinas	MVK	8.91	9.11	8.81
220419_s_at	ubiquitin specific	USP25	10.09	9.98	10.13
220172_at	DDB1 and CUL4 as	DCAF17	6.91	6.95	7.07
201611_s_at	isoprenylcysteine	ICMT	9.92	9.95	10.00
221524_s_at	Ras-related GTP b	RRAGD	6.64	6.62	6.51
202577_s_at	DEAD (Asp-Glu-Al	DDX19A	8.39	8.54	8.59
207194_s_at	intercellular adhe	ICAM4	3.89	3.56	3.72
217778_at	solute carrier fam	SLC39A1	7.84	7.73	7.95
204967_at	shroom family me	SHROOM2	4.39	4.16	4.12
210866_s_at	CCR4-NOT transcr	CNOT4	4.87	5.17	5.36
216792_at	---	---	4.75	4.73	4.94
205654_at	complement com	C4BPA	6.19	6.20	5.90
217040_x_at	SRY (sex determin	SOX15	6.64	6.61	6.83
212785_s_at	La ribonucleoprot	LARP7	9.73	9.76	9.80
220055_at	zinc finger protein	ZNF287	3.85	3.52	3.65
208429_x_at	hepatocyte nucle	HNF4A	4.92	4.39	4.36
214678_x_at	zinc finger protein	ZFX	7.85	7.99	8.08
201297_s_at	MOB1, Mps One E	MOBKL1B	8.00	8.23	8.33
213352_at	transmembrane a	TMCC1	6.11	6.05	6.15
211419_s_at	chimerin (chimae	CHN2	3.68	3.68	3.55
215499_at	mitogen-activatec	MAP2K3	9.34	9.31	9.25
203273_s_at	tumor suppressor	TUSC2	8.09	7.96	7.92
217999_s_at	pleckstrin homolo	PHLDA1	6.36	6.34	6.66
207588_at	---	---	3.34	3.39	3.34
217262_s_at	cadherin, EGF LAG	CELSR1	4.23	4.22	4.08
214830_at	solute carrier fam	SLC38A6	7.78	8.19	8.10
219437_s_at	ankyrin repeat do	ANKRD11	9.64	9.51	9.72
220794_at	gremlin 2, cystein	GREM2	3.52	3.79	3.59
212182_at	nudix (nucleoside	NUDT4 /// NUDT4	4.10	3.69	3.95
211793_s_at	abl-interactor 2	ABI2	8.36	8.35	8.38
219593_at	solute carrier fam	SLC15A3	6.54	6.28	6.26
222132_s_at	acylglycerol kinas	AGK	9.09	9.02	9.06
218420_s_at	chromosome 13 c	C13orf23	8.00	7.81	7.99
AFFX-r2-Bs-lys-3	---	---	7.93	7.89	7.91
212025_s_at	flightless I homolo	FLII	7.74	7.88	7.91
214632_at	neuropilin 2	NRP2	6.16	5.58	6.00
218680_x_at	chromosome 15 c	C15orf63 /// SERF2	11.08	11.07	11.05

203959_s_at	zinc finger and BT	ZBTB40	8.35	8.28	8.11
209586_s_at	prune homolog (D	PRUNE	8.55	8.65	8.47
210329_s_at	sarcoglycan, delta	SGCD	6.18	5.99	6.20
216434_at	Tetratricopeptide	TTC38	7.33	7.40	7.32
208096_s_at	collagen, type XXI	COL21A1	3.99	4.14	4.17
222317_at	phosphodiesteras	PDE3B	4.01	4.09	3.92
203182_s_at	SFRS protein kinas	SRPK2	9.17	9.29	9.32
202895_s_at	EPH receptor B4	EPHB4	5.24	5.02	5.20
220566_at	phosphoinositide-	PIK3R5	6.28	6.02	5.84
215429_s_at	zinc finger protein	ZNF428	8.36	8.29	8.33
205705_at	ankyrin repeat do	ANKRD26	5.80	6.40	6.16
216431_at	---	---	5.58	5.37	5.09
211512_s_at	opioid growth fac	OGFR	8.51	8.10	8.23
218843_at	fibronectin type II	FNDC4	7.11	7.20	6.85
221918_at	cyclin-dependent	CDK17	11.07	10.90	11.11
208963_x_at	fatty acid desatur	FADS1	10.43	10.34	10.34
206391_at	retinoic acid recep	RARRES1	5.72	5.56	5.60
221863_at	mesoderm induct	MIER2	7.44	7.26	7.38
215992_s_at	Rap guanine nucle	RAPGEF2	6.28	6.78	6.67
208186_s_at	lipase, hormone-s	LIPE	5.82	5.65	5.64
221466_at	pyrimidinergic rec	P2RY4	4.55	4.64	4.44
213314_at	chromosome 6 op	C6orf162	5.78	5.99	6.01
212999_x_at	major histocompa	HLA-DQB1	3.48	3.42	3.39
209912_s_at	KIAA0415	KIAA0415	7.04	6.99	7.02
204176_at	kelch-like 20 (Dro	KLHL20	7.32	7.47	7.60
201938_at	cyclin-dependent	CDK2AP1	12.54	12.63	12.65
216494_at	insulin-like growth	IGF2BP3	4.53	4.67	4.42
221127_s_at	dickkopf homolog	DKK3	7.18	7.05	7.14
212578_x_at	ribosomal protein	RPS17	14.89	14.86	14.91
221491_x_at	major histocompa	HLA-DRB1 /// HLA-	4.40	4.13	3.68
221862_at	mesoderm induct	MIER2	4.85	5.12	5.02
206667_s_at	secretory carrier r	SCAMP1	6.46	6.57	6.73
217584_at	Niemann-Pick dise	NPC1	5.16	5.02	5.09
221514_at	UTP14, U3 small r	UTP14A	9.60	9.63	9.82
218797_s_at	sirtuin (silent mat	SIRT7	8.04	8.04	7.98
212637_s_at	WW domain conta	WWP1	6.47	6.54	6.90
202526_at	SMAD family mem	SMAD4	4.10	4.24	4.21
211862_x_at	CASP8 and FADD-l	CFLAR	7.44	7.52	7.50
206467_x_at	regulator of telom	RTEL1 /// TNFRSF6	6.81	7.02	6.66
204111_at	histamine N-meth	HNMT	3.26	3.43	3.17
201824_at	ring finger protein	RNF14	9.24	9.53	9.69
208196_x_at	nuclear factor of a	NFATC1	7.66	7.91	7.58
216475_at	---	---	6.16	6.43	6.02
214979_at	ATP-binding casse	ABCC3	4.91	4.80	4.82
211996_s_at	similar to Unchara	LOC100132247 ///	10.24	10.14	10.21
207923_x_at	paired box 8	PAX8	6.01	5.78	5.77
211285_s_at	ubiquitin protein l	UBE3A	10.23	10.28	10.35

222208_s_at	polymerase (RNA)	POLR2J4	6.05	6.14	5.83
207351_s_at	SH2 domain conta	SH2D2A	6.80	6.44	6.49
204751_x_at	desmocollin 2	DSC2	3.50	3.56	3.37
205225_at	estrogen receptor	ESR1	3.42	3.52	3.63
220594_at	O-linked N-acetylg	OGT	5.86	5.63	5.79
221456_at	taste receptor, typ	TAS2R3	4.64	4.63	4.18
214315_x_at	calreticulin	CALR	9.33	9.33	9.46
200712_s_at	microtubule-asso	MAPRE1	8.77	8.84	8.79
210084_x_at	tryptase alpha/be	TPSAB1	5.66	5.60	5.54
209507_at	replication protei	RPA3	11.62	11.67	11.69
208301_at	---	---	6.07	6.32	6.07
211528_x_at	major histocompa	HLA-G	10.55	10.41	10.60
207628_s_at	Williams Beuren s	WBSCR22	10.21	10.12	10.30
205422_s_at	integrin, beta-like	ITGBL1	4.54	4.71	4.30
216558_x_at	immunoglobulin h	IGHA1 /// IGHD //,	5.72	5.36	5.19
208599_at	HECT, UBA and W	HUWE1	5.14	5.26	5.03
205633_s_at	aminolevulinate, d	ALAS1	9.57	9.63	9.56
218510_x_at	family with seque	FAM134B	6.40	6.47	6.45
203693_s_at	E2F transcription f	E2F3	8.73	8.78	8.83
208806_at	chromodomain he	CHD3	7.09	6.70	6.59
208077_at	chromosome 9 op	C9orf38	4.52	4.55	4.53
217285_at	DiGeorge syndrom	DGCR14	6.72	6.93	6.93
220007_at	methyltransferase	METTL8	8.06	8.24	8.36
206520_x_at	sialic acid binding	SIGLEC6	6.50	6.58	6.66
207911_s_at	transglutaminase	TGM5	6.31	6.24	6.06
216606_x_at	lysophospholipase	LYPLA2P1	8.08	8.14	7.87
217695_x_at	---	---	4.25	3.60	3.81
207906_at	interleukin 3 (colo	IL3	3.76	3.77	3.64
203201_at	phosphomannom	PMM2	8.42	8.58	8.48
210525_x_at	chromosome 14 o	C14orf143	3.79	3.90	3.74
204266_s_at	choline kinase alp	CHKA	7.57	7.68	7.69
205097_at	solute carrier fam	SLC26A2	9.46	9.79	9.57
202335_s_at	ubiquitin-conjugat	UBE2B	4.09	3.73	4.11
217128_s_at	calcium/calmodul	CAMK1G	4.82	4.65	4.75
210177_at	tripartite motif-co	TRIM15	7.82	7.63	7.87
205109_s_at	Rho guanine nucle	ARHGEF4	5.34	4.90	5.00
208105_at	gastric inhibitory p	GIPR	6.75	6.83	6.93
216487_at	---	---	3.28	3.16	3.43
212567_s_at	microtubule-asso	MAP4	8.79	8.74	8.77
203579_s_at	solute carrier fam	SLC7A6	7.32	7.47	7.33
200917_s_at	signal recognition	SRPR	6.94	7.03	7.03
204098_at	RNA binding moti	RBMX2	8.11	8.33	8.31
209807_s_at	nuclear factor I/X	NFIX	6.68	6.33	6.31
213899_at	methionyl aminop	METAP2	9.02	9.01	9.21
207312_at	phosphorylase kin	PHKG1	5.48	5.61	5.55
206253_at	discs, large homol	DLG2	3.39	3.34	3.41
220090_at	cornulin	CRNN	5.47	5.40	5.61

208652_at	protein phosphata	PPP2CA	11.97	12.06	12.02
35685_at	ring finger protein	RING1	9.77	9.66	9.68
207635_s_at	potassium voltage	KCNH1	4.07	4.30	4.00
220830_at	interphotorecepto	IMPG2	4.46	4.15	4.13
206439_at	epiphycan	EPYC	3.38	3.26	3.27
208496_x_at	histone cluster 1,	HIST1H3G	7.48	7.54	7.44
208026_at	histone cluster 1,	HIST1H4F	4.13	3.92	3.70
218990_s_at	small proline-rich	SPRR3	4.67	4.58	4.93
207734_at	lymphocyte transı	LAX1	4.26	4.06	4.39
221201_s_at	zinc finger protein	ZNF155	4.02	4.10	4.02
206812_at	adrenergic, beta-3	ADRB3	5.65	5.62	5.47
216140_at	---	---	4.58	4.27	4.16
216092_s_at	solute carrier fam	SLC7A8	6.93	6.84	6.62
203658_at	solute carrier fam	SLC25A20	7.34	7.36	7.36
219137_s_at	mitochondrial fiss	MFF	10.22	10.38	10.47
211337_s_at	tubulin, gamma co	TUBGCP4	8.76	8.80	8.81
211456_x_at	metallothionein 1	MT1P2	13.29	13.28	13.17
201568_at	ubiquinol-cytochr	UQCRQ	12.66	12.75	12.84
205817_at	SIX homeobox 1	SIX1	7.98	7.66	7.81
215961_at	coagulation factor	F12	5.75	5.80	5.26
209451_at	TRAF family meml	TANK	8.99	8.97	9.17
210833_at	prostaglandin E re	PTGER3	3.67	3.39	3.57
220690_s_at	dehydrogenase/re	DHRS7B	7.78	7.87	7.68
204597_x_at	stanniocalcin 1	STC1	5.61	5.76	5.69
217831_s_at	NSFL1 (p97) cofac	NSFL1C	8.36	8.47	8.42
201515_s_at	translin	TSN	11.21	11.27	11.29
218015_s_at	Werner helicase in	WRNIP1	4.74	4.73	4.87
206394_at	myosin binding pr	MYBPC2	4.81	5.16	5.03
213226_at	cyclin A2	CCNA2	10.87	11.00	11.08
38241_at	butyrophilin, subf	BTN3A3	4.68	4.57	4.64
208344_x_at	interferon, alpha 1	IFNA1 /// IFNA13	4.47	4.60	4.27
214962_s_at	nucleoporin 160kl	NUP160	5.90	5.75	5.71
216711_s_at	TAF1 RNA polyme	TAF1	7.24	7.02	7.16
219359_at	ATH1, acid trehala	ATHL1	7.41	7.50	7.24
219384_s_at	adenosine deamir	ADAT1	8.71	8.68	8.88
218462_at	ribosome product	RPF1	11.44	11.41	11.48
1729_at	TNFRSF1A-associa	TRADD	8.34	8.40	8.36
221112_at	interleukin 1 rece	IL1RAPL2	4.44	4.56	4.47
205096_at	POM121 membra	POM121	5.17	5.52	5.28
202947_s_at	glycophorin C (Ge	GYPC	10.65	10.51	10.77
210144_at	hypothetical prot	LOC100289420	6.75	6.21	6.12
205514_at	zinc finger protein	ZNF415	3.36	3.15	3.31
205388_at	troponin C type 2	TNNC2	6.33	6.07	5.81
216478_at	---	---	3.67	3.56	3.52
201467_s_at	NAD(P)H dehydro	NQO1	10.79	10.73	10.78
215666_at	major histocompa	HLA-DRB4	3.59	3.60	3.33
216353_s_at	---	---	3.45	3.37	3.23

210605_s_at	milk fat globule-E(	MFGE8	8.33	8.19	8.08
220923_s_at	paraneoplastic an	PNMA3	6.79	6.72	6.51
219157_at	kelch-like 2, Mayv	KLHL2	9.59	9.53	9.74
206978_at	chemokine (C-C m	CCR2	4.49	4.82	4.26
210690_at	killer cell lectin-lik	KLRC4	3.51	3.36	3.34
201913_s_at	CoA synthase	COASY	9.25	9.18	9.21
204232_at	Fc fragment of IgE	FCER1G	4.33	4.49	4.12
214586_at	G protein-couplec	GPR37	3.17	3.29	3.30
208929_x_at	ribosomal protein	RPL13	14.48	14.44	14.50
215355_at	POU class 2 home	POU2F3	3.53	3.39	3.32
211460_at	testis-specific trar	TTTY9A /// TTTY9E	3.93	4.14	3.90
210874_s_at	N-acetyltransfera:	NAT6	8.33	8.22	8.50
215982_s_at	dom-3 homolog Z	DOM3Z	7.88	7.62	7.65
213114_at	---	---	7.05	6.81	7.01
201479_at	dyskeratosis cong	DKC1	12.25	12.17	12.38
213675_at	---	---	6.02	6.00	5.94
217239_x_at	---	---	4.63	4.50	4.19
221909_at	ring finger proteir	RNFT2	7.05	7.05	6.87
216685_s_at	methylthioadenos	MTAP	4.15	4.11	4.21
205244_s_at	solute carrier fam	SLC13A3	6.29	6.43	6.26
216461_at	---	---	5.89	5.92	5.81
222021_x_at	succinate dehydrc	SDHAP1	10.88	10.80	10.88
217092_x_at	ribosomal protein	RPL7	11.82	11.76	11.77
208184_s_at	trafficking protein	TRAPPC10	6.08	6.41	6.20
213201_s_at	troponin T type 1	TNNT1	8.69	8.80	8.60
207926_at	glycoprotein V (pl	GP5	3.63	3.62	3.73
218632_at	HECT domain con	HECTD3	6.51	6.58	6.49
213113_s_at	solute carrier fam	SLC43A3	5.41	5.32	5.12
206567_s_at	PHD finger proteir	PHF20	8.76	8.78	8.93
32259_at	enhancer of zeste	EZH1	8.36	8.42	8.42
37652_at	calcineurin bindin	CABIN1	7.04	7.21	7.10
214778_at	multiple EGF-like-	MEGF8	6.34	6.09	6.10
214805_at	Eukaryotic transla	EIF4A1	7.90	8.08	8.01
206904_at	matrilin 1, cartilag	MATN1	5.55	5.92	5.46
59437_at	chromosome 9 op	C9orf116	7.12	7.18	7.05
202788_at	mitogen-activatec	MAPKAPK3	10.10	10.22	10.09
216297_at	---	---	3.80	3.70	3.72
208557_at	homeobox A6	HOXA6	5.31	5.34	5.31
209359_x_at	runt-related trans	RUNX1	6.94	6.56	6.77
211483_x_at	calcium/calmodul	CAMK2B	5.04	5.19	5.18
222359_x_at	3-ketodihydrosph	KDSR	3.55	3.72	3.67
212553_at	regulation of nucl	RPRD2	7.71	7.65	7.97
204563_at	selectin L	SELL	3.64	3.69	3.56
211403_x_at	variable charge, X	VCX2	6.47	6.39	6.21
219625_s_at	collagen, type IV,	COL4A3BP	8.63	8.74	8.80
210583_at	polymerase (DNA-	POLDIP3	5.78	5.48	5.73
222103_at	activating transcri	ATF1	9.99	9.90	10.13

213424_at	KIAA0895	KIAA0895	4.32	4.42	4.44
209799_at	protein kinase, AN	PRKAA1	6.99	7.14	7.07
221204_s_at	cartilage acidic pro	CRTAC1	6.50	6.35	6.18
211154_at	thrombopoietin	THPO	3.26	3.25	3.06
205853_at	zinc finger and BT	ZBTB7B	6.26	6.13	6.13
216757_at	---	---	4.10	3.97	3.72
220089_at	L-2-hydroxyglutar	L2HGDH	5.75	5.87	5.94
206747_at	G protein regulate	GPRIN2	6.97	6.52	6.56
212699_at	secretory carrier r	SCAMP5	6.38	6.53	6.43
216427_at	---	---	6.45	6.26	6.06
220871_at	---	---	4.12	4.40	3.98
215127_s_at	RNA binding moti	RBMS1	11.04	11.12	11.22
203073_at	component of olig	COG2	8.80	8.94	8.96
207889_at	sarcosine dehydro	SARDH	3.84	3.74	3.65
211592_s_at	calcium channel, v	CACNA1C	3.43	3.39	3.34
215356_at	tudor domain con	TDRD12	4.56	4.24	4.66
205703_at	---	---	3.74	4.07	3.97
212937_s_at	collagen, type VI,	COL6A1	6.90	6.96	6.64
201383_s_at	neighbor of BRCA	NBR1	7.68	7.72	7.90
207462_at	glycine receptor, a	GLRA2	5.16	5.04	5.23
216771_at	---	---	3.98	4.09	3.70
209972_s_at	aminoacyl tRNA sy	AIMP2	7.89	7.81	7.79
218093_s_at	ankyrin repeat do	ANKRD10	9.29	9.31	9.20
202861_at	DENN/MADD dom	DENND4B	8.50	8.28	8.40
202562_s_at	chromosome 14 o	C14orf1	10.26	10.37	10.30
220696_at	---	---	3.98	3.94	3.75
220114_s_at	stabilin 2	STAB2	4.95	4.85	4.78
216910_at	X-prolyl aminopep	XPNPEP2	5.57	5.57	5.40
216268_s_at	jagged 1 (Alagille s	JAG1	10.86	10.66	11.00
217308_at	olfactory receptor	OR1F2P	5.21	5.48	5.23
205370_x_at	dihydrolipoamide	DBT	10.86	10.96	10.79
215814_at	Dystonin	DST	3.42	3.36	3.53
216650_at	---	---	7.23	6.90	6.92
211897_s_at	corticotropin rele	CRHR1	5.48	5.48	5.14
221254_s_at	PITPNM family me	PITPNM3	4.15	3.82	3.86
202337_at	polyamine-modul	PMF1	7.42	7.29	7.25
201828_x_at	family with seque	FAM127A	10.08	10.13	10.03
204215_at	chromosome 7 op	C7orf23	9.76	9.61	9.81
209180_at	Rab geranylgeran	RABGGTB	10.83	10.84	10.87
216401_x_at	similar to Ig kappa	LOC652493	4.66	4.56	4.45
212965_at	hypermethylated	HIC2	6.27	6.27	5.75
202541_at	aminoacyl tRNA sy	AIMP1	9.91	10.11	10.25
210611_s_at	dystrobrevin, alph	DTNA	5.58	5.13	5.61
211920_at	complement facto	CFB	5.83	5.54	5.81
211789_s_at	MLX interacting p	MLXIP	4.21	4.22	3.75
211706_s_at	cyclin-dependent	CDK19	4.91	5.04	4.58
208559_at	pancreatic and du	PDX1	5.75	6.01	5.65

214557_at	pituitary tumor-tr	PTTG2	4.12	4.66	4.48
211265_at	prostaglandin E re	PTGER3	3.89	3.83	3.74
205403_at	interleukin 1 rece	IL1R2	5.50	5.12	5.40
38340_at	huntingtin interac	HIP1R /// LOC1002	8.97	8.99	8.95
213082_s_at	solute carrier fam	SLC35D2	4.50	4.22	4.20
202978_s_at	CREB/ATF bZIP tra	CREBZF	8.27	8.12	8.21
220511_s_at	deleted in liver ca	DLC1	4.56	4.44	4.45
219280_at	bromodomain and	BRWD1	6.70	6.72	6.86
50314_i_at	chromosome 20 c	C20orf27	7.40	7.34	7.46
205688_at	transcription facto	TFAP4	7.72	7.42	7.30
214209_s_at	ATP-binding casse	ABCB9	5.51	5.58	5.11
212561_at	DENN/MADD dom	DENND5A	10.27	10.19	10.24
208288_at	ATP-binding casse	ABCB11	3.80	3.90	3.47
213807_x_at	met proto-oncoge	MET	6.75	6.73	6.73
215445_x_at	---	---	4.97	4.91	4.95
204204_at	solute carrier fam	SLC31A2	6.62	6.75	6.84
221686_s_at	RecQ protein-like	RECQL5	8.47	8.32	8.26
211610_at	Kruppel-like facto	KLF6	6.76	6.84	6.34
218830_at	ribosomal protein	RPL26L1	11.38	11.40	11.41
221230_s_at	AT rich interactive	ARID4B	8.76	8.98	9.07
221817_at	dolichyl pyrophos	DOLPP1	7.94	7.94	7.77
211204_at	malic enzyme 1, N	ME1	4.61	4.32	4.52
200600_at	moesin	MSN	12.14	12.21	12.17
206628_at	solute carrier fam	SLC5A1	5.68	5.42	5.51
215899_at	---	---	6.17	6.19	6.02
212936_at	family with seque	FAM172A	8.36	8.33	8.39
208143_s_at	epididymal protei	EDDM3A	3.36	3.31	3.31
214598_at	claudin 8	CLDN8	3.52	3.48	3.39
219228_at	zinc finger protein	ZNF331	7.31	7.27	7.20
219260_s_at	chromosome 17 c	C17orf81	7.95	7.88	8.16
208333_at	LIM homeobox 5	LHX5	7.51	7.25	7.18
208261_x_at	interferon, alpha 1	IFNA10	3.82	3.86	4.08
220705_s_at	ADAM metallopep	ADAMTS7	7.28	7.08	7.10
203222_s_at	transducin-like en	TLE1	8.00	8.00	8.13
210477_x_at	mitogen-activated	MAPK8	5.58	5.58	5.40
220068_at	pre-B lymphocyte	VPREB3	5.15	5.18	5.26
220269_at	zinc finger, B-box	ZBBX	3.45	3.32	3.46
216961_s_at	RPA interacting pr	RPAIN	6.04	6.26	6.20
215044_s_at	signal transducing	STAM2	6.70	6.57	6.53
219797_at	mannosyl (alpha-1	MGAT4A	3.47	3.47	3.46
215554_at	glycosylphosphati	GPLD1	3.34	3.21	3.37
215850_s_at	NADH dehydrogen	NDUFA5	3.97	3.85	3.78
207843_x_at	cytochrome b5 ty	CYB5A	11.74	11.74	11.86
221544_s_at	mediator complex	MED16	6.87	6.55	6.55
221548_s_at	integrin-linked kin	ILKAP	9.21	8.95	9.10
216903_s_at	calcium binding at	CBARA1	8.57	8.55	8.35
203007_x_at	lysophospholipase	LYPLA1	10.59	10.79	10.76

203272_s_at	tumor suppressor	TUSC2	9.08	8.98	8.97
210497_x_at	synovial sarcoma,	SSX2 /// SSX2B	4.10	4.13	3.80
214364_at	MTERF domain co	MTERFD2	6.60	6.67	6.39
203078_at	cullin 2	CUL2	6.74	6.69	6.78
210340_s_at	colony stimulating	CSF2RA	5.89	5.63	5.63
217494_s_at	hypothetical prot	LOC100291860	4.02	3.92	4.00
210467_x_at	melanoma antiger	MAGEA12	11.70	11.76	11.88
202756_s_at	glypican 1	GPC1	10.04	9.83	9.72
216707_at	---	---	6.77	6.49	6.33
220539_at	chromosome 10 c	C10orf92	4.45	4.41	4.33
217710_x_at	inositol 1,3,4-tripl	ITPK1	7.14	6.99	6.95
220129_at	spermatogenesis a	SOHLH2	7.65	7.43	7.84
201917_s_at	solute carrier fam	SLC25A36	11.13	11.05	11.13
207234_at	regulatory factor)	RFX3	3.66	3.73	3.50
32723_at	cleavage stimulati	CSTF1	7.23	7.41	7.40
207283_at	ribosomal protein	RPL23AP32	7.80	7.95	7.93
209035_at	midkine (neurite g	MDK	7.89	7.73	7.39
222161_at	N-acetylated alph	NAALAD2	3.34	3.24	3.20
210996_s_at	tyrosine 3-monoo	YWHAE	9.73	9.70	9.67
214772_at	chromosome 11 c	C11orf41	4.47	4.17	4.35
218034_at	fission 1 (mitocho	FIS1	10.49	10.35	10.52
213683_at	acyl-CoA syntheta	ACSL6	4.66	4.61	4.55
217672_x_at	eukaryotic transla	EIF1	8.87	8.73	9.00
215533_s_at	ubiquitination fac	UBE4B	5.65	5.67	5.56
203888_at	thrombomodulin	THBD	7.72	7.58	7.87
203267_s_at	developmentally r	DRG2	6.86	6.66	6.78
202432_at	protein phosphata	PPP3CB	9.97	10.10	9.96
202482_x_at	RAN binding prote	RANBP1	7.01	7.05	7.06
220564_at	renalase, FAD-dep	RNLS	5.70	5.25	5.43
215638_at	v-erb-b2 erythrob	ERBB3	5.26	4.98	4.91
219894_at	MAGE-like 2	MAGEL2	3.75	3.52	3.58
211752_s_at	NADH dehydroger	NDUFS7	10.50	10.31	10.37
205301_s_at	8-oxoguanine DN/	OGG1	7.85	7.72	7.65
219111_s_at	DEAD (Asp-Glu-Ala	DDX54	8.11	7.99	8.05
209679_s_at	small cell adhesio	SMAGP	8.34	8.34	8.38
213248_at	hypothetical LOC7	LOC730101	6.56	6.88	6.67
202198_s_at	myotubularin rela	MTMR3	5.11	5.13	4.69
215527_at	KH domain contai	KHDRBS2	5.28	5.20	5.03
212990_at	synaptojanin 1	SYNJ1	7.68	7.85	7.79
205540_s_at	Ras-related GTP b	RRAGB	7.01	7.17	7.00
212135_s_at	ATPase, Ca++ tran	ATP2B4	8.50	8.72	8.49
219372_at	intraflagellar trans	IFT81	8.19	8.48	8.25
212120_at	ras homolog gene	RHOQ	9.49	9.70	9.72
209413_at	UDP-Gal:betaGlcN	B4GALT2	8.47	8.31	8.37
202602_s_at	HIV-1 Tat specific	HTATSF1	10.16	10.21	10.28
214028_x_at	tudor domain con	TDRD3	5.53	5.42	5.47
215415_s_at	lysosomal trafficki	LYST	4.37	4.34	4.26

212310_at	melanoma inhibit	MIA3	6.87	7.01	7.35
201193_at	isocitrate dehydrc	IDH1	11.02	11.18	11.08
213672_at	methionyl-tRNA s	MARS	6.91	7.25	7.04
217701_x_at	---	---	4.69	4.31	4.62
205782_at	fibroblast growth	FGF7	4.00	4.08	4.12
220820_at	---	---	4.73	4.62	4.62
212618_at	zinc finger proteir	ZNF609	7.03	6.98	6.70
214812_s_at	MOB1, Mps One E	MOBKL1B	10.02	10.20	10.25
202457_s_at	protein phosphata	PPP3CA	10.95	10.90	10.93
204507_s_at	protein phosphata	PPP3R1	7.51	7.56	7.75
203982_s_at	ATP-binding casse	ABCD4	4.97	5.17	4.99
201041_s_at	dual specificity ph	DUSP1	12.34	12.23	12.44
206968_s_at	nuclear factor rela	NFRKB	5.33	5.45	5.08
212153_at	pogo transposable	POGZ	8.53	8.56	8.54
220176_at	nucleotide binding	NUBPL	7.48	7.47	7.68
38703_at	aspartyl aminope	DNPEP	10.07	10.08	9.97
210829_s_at	single-stranded D	SSBP2	9.87	9.67	9.84
211004_s_at	aldehyde dehydrc	ALDH3B1	7.27	7.24	7.24
210626_at	A kinase (PRKA) a	AKAP1	5.36	5.50	4.97
202789_at	phospholipase C,	PLCG1	8.54	8.52	8.41
212543_at	absent in melanor	AIM1	3.72	3.75	3.85
208382_s_at	DMC1 dosage sup	DMC1	4.04	4.32	4.14
45526_g_at	N-acetyltransfera	NAT15	9.92	9.88	9.84
219195_at	peroxisome prolif	PPARGC1A	4.73	4.71	4.41
219397_at	coenzyme Q10 ho	COQ10B	8.59	8.64	8.65
215826_x_at	zinc finger proteir	ZNF835	5.20	5.58	5.25
204986_s_at	TAO kinase 2	TAOK2	5.14	5.31	5.05
222207_x_at	---	---	10.20	10.30	10.27
216847_at	---	---	5.60	5.54	5.39
218770_s_at	transmembrane p	TMEM39B	8.07	8.07	8.19
201118_at	phosphogluconate	PGD	9.32	9.41	9.37
218020_s_at	zinc finger, AN1-ty	ZFAND3	8.75	8.50	8.70
208885_at	lymphocyte cytos	LCP1	8.67	8.73	8.70
202201_at	biliverdin reducta	BLVRB	11.03	10.80	10.77
214652_at	dopamine recepto	DRD1	3.74	3.78	3.52
216595_at	family with seque	FAM186A	3.34	3.41	3.34
208327_at	cytochrome P450,	CYP2A13	5.98	5.73	5.62
220535_at	family with seque	FAM90A1	5.71	5.55	5.39
219599_at	eukaryotic transla	EIF4B	8.66	8.71	8.85
212783_at	retinoblastoma bi	RBBP6	8.30	8.47	8.46
209765_at	ADAM metallope	ADAM19	5.30	5.25	4.90
212047_s_at	ring finger proteir	RNF167	8.33	8.30	8.27
204277_s_at	thymidine kinase	TK2	7.09	6.93	7.05
216349_at	fumarate hydrata	FH	5.47	5.52	5.56
209423_s_at	PHD finger protei	PHF20	6.22	6.36	6.27
212549_at	signal transducer	STAT5B	8.19	8.18	8.18
221326_s_at	tubulin, delta 1	TUBD1	6.53	6.46	6.21

217815_at	suppressor of Ty 1	SUPT16H		11.12	11.26	11.33
213445_at	zinc finger CCCH-t	ZC3H3		6.91	7.02	6.81
219121_s_at	epithelial splicing	ESRP1		5.09	4.81	4.55
204079_at	tyrosylprotein sul	TPST2		9.08	9.22	9.31
222284_at	---	---		7.46	7.55	7.26
207419_s_at	ras-related C3 bot	RAC2		6.99	7.11	6.86
220881_at	---	---		4.29	4.09	3.79
219146_at	chromosome 17 c	C17orf42		8.94	9.07	9.05
220707_s_at	FAD-dependent o	FOXRED2		5.58	5.49	5.33
215743_at	N-myristoyltransf	NMT2		6.94	7.42	7.47
202286_s_at	tumor-associated	TACSTD2		3.85	3.54	3.50
210956_at	pancreatic polype	PPYR1		5.48	5.73	5.21
220004_at	DEAD (Asp-Glu-Al	DDX43		7.42	7.49	7.59
219708_at	5',3'-nucleotidase	NT5M		6.47	6.24	6.54
206865_at	harakiri, BCL2 inte	HRK		4.84	5.02	4.67
AFFX-HUMGAPD	glyceraldehyde-3-	GAPDH		14.48	14.46	14.44
210916_s_at	CD44 molecule (In	CD44		9.26	9.19	9.19
213298_at	nuclear factor I/C	NFIC		7.86	7.76	7.96
218803_at	checkpoint with f	CHFR		7.33	7.11	7.32
210949_s_at	eukaryotic transla	EIF3C /// EIF3CL		12.29	12.09	12.18
204705_x_at	aldolase B, fructo	ALDOB		6.18	6.04	5.83
202641_at	ADP-ribosylation f	ARL3		9.89	9.87	9.96
213351_s_at	transmembrane a	TMCC1		7.37	7.23	7.29
216959_x_at	neuronal cell adhe	NRCAM		5.77	5.73	5.47
220028_at	activin A receptor	ACVR2B		7.37	7.18	7.02
205865_at	AT rich interactive	ARID3A		6.58	6.71	6.58
213917_at	paired box 8	PAX8		5.21	5.05	5.07
207078_at	mediator complex	MED6		5.40	5.57	5.62
205959_at	matrix metallopep	MMP13		4.07	3.80	3.77
206355_at	guanine nucleotid	GNAL		3.36	3.72	3.83
213345_at	nuclear factor of a	NFATC4		6.99	6.97	6.79
219373_at	dolichyl-phosphat	DPM3		9.34	9.31	9.36
91703_at	EH domain bindin	EHBP1L1		6.85	6.57	6.93
207744_at	---	---		3.14	3.38	3.14
213756_s_at	heat shock transc	HSF1		6.25	6.06	6.04
44111_at	vacuolar protein s	VPS33B		8.66	8.69	8.71
202502_at	acyl-CoA dehydro	ACADM		9.46	9.78	9.80
212569_at	structural mainte	SMCHD1		8.59	8.96	8.81
211210_x_at	SH2 domain conta	SH2D1A		6.24	6.21	6.12
207525_s_at	GIPC PDZ domain	GIPC1		8.27	8.16	8.12
205850_s_at	gamma-aminobut	GABRB3		4.79	4.64	4.53
221824_s_at	membrane-associ		8-Mar	7.69	7.76	7.76
217067_s_at	dentin matrix acid	DMP1		3.84	3.91	3.87
204565_at	acyl-CoA thioeste	ACOT13		10.58	10.58	10.60
207187_at	Janus kinase 3	JAK3		6.40	6.21	6.14
215464_s_at	Tax1 (human T-ce	TAX1BP3		9.09	9.10	9.06
201594_s_at	protein phosphata	PPP4R1		10.81	10.78	10.84

216004_s_at	PBX/knotted 1 ho	PKNOX1	4.30	4.05	3.95
203840_at	basic leucine zipp	BLZF1	6.79	6.71	7.16
203557_s_at	pterin-4 alpha-car	PCBD1	7.79	7.64	7.73
206217_at	ectodysplasin A	EDA	6.69	6.29	6.27
206894_at	apolipoprotein A-I	APOA4	6.35	6.11	6.01
33579_i_at	galanin receptor 3	GALR3	7.50	7.62	7.50
204601_at	NEDD4 binding pr	N4BP1	7.06	7.08	7.10
214834_at	Prader-Willi/Ange	PAR5	3.75	3.67	3.91
221158_at	GC-rich sequence	GCFC1	8.17	8.50	8.54
205759_s_at	sulfotransferase fa	SULT2B1	7.19	7.13	7.10
222262_s_at	ethanolamine kina	ETNK1	7.54	7.65	7.55
220831_at	glucosaminyl (N-a	GCNT4	3.74	3.47	3.48
216993_s_at	collagen, type XI, a	COL11A2	7.15	6.86	6.76
213865_at	discoidin, CUB and	DCBLD2	7.68	7.98	7.84
215299_x_at	sulfotransferase fa	SULT1A1	6.97	7.07	6.74
210893_at	---	---	3.04	3.05	2.95
219499_at	Sec61 alpha 2 sub	SEC61A2	6.98	6.94	6.67
213802_at	---	---	5.03	4.87	4.98
202516_s_at	discs, large homol	DLG1	6.35	6.44	6.55
206124_s_at	lethal giant larvae	LLGL1	6.47	6.59	6.77
213762_x_at	RNA binding moti	RBMX	11.68	11.66	11.86
208307_at	RNA binding moti	RBMY1A1 /// RBM	4.24	4.41	4.18
218552_at	enoyl CoA hydrata	ECHDC2	6.88	7.09	6.83
216075_at	---	---	4.69	4.62	4.91
218072_at	COMM domain co	COMMD9	8.27	8.36	8.34
211059_s_at	golgin A2	GOLGA2	8.15	8.11	7.88
215728_s_at	acyl-CoA thioeste	ACOT7	8.51	8.45	8.48
216165_at	GTPase activating	GARNL3	3.77	3.76	4.16
217910_x_at	MAX-like protein X	MLX	8.86	8.89	8.91
214544_s_at	synaptosomal-ass	SNAP23	6.55	6.71	6.38
210026_s_at	caspase recruitme	CARD10	7.85	8.04	8.01
206475_x_at	chorionic somato	CSH1	4.47	4.19	4.41
213328_at	NIMA (never in m	NEK1	8.28	8.45	8.44
209362_at	mediator complex	MED21	9.77	9.79	9.89
208858_s_at	extended synapto	ESYT1	9.75	9.60	9.75
211899_s_at	TNF receptor-asso	TRAF4	6.01	6.16	6.06
211095_at	neurofibromin 1	NF1	3.62	3.51	3.49
221330_at	cholinergic recept	CHRM2	3.86	4.07	3.93
203266_s_at	mitogen-activated	MAP2K4	7.55	7.60	7.82
203791_at	Dmx-like 1	DMXL1	7.32	7.33	7.41
200694_s_at	DEAD (Asp-Glu-Ala	DDX24	10.27	10.36	10.30
202490_at	inhibitor of kappa	IKBKAP	5.86	5.85	5.74
217717_s_at	tyrosine 3-monoo	YWHAB	12.45	12.43	12.47
201455_s_at	aminopeptidase p	NPEPPS	9.20	9.23	9.32
219276_x_at	chromosome 9 op	C9orf82	10.13	10.20	10.37
217848_s_at	pyrophosphatase	PPA1	13.48	13.48	13.54
214860_at	solute carrier fam	SLC9A7	4.77	4.31	4.31

214505_s_at	four and a half LIN	FHL1	5.12	5.03	4.97
219961_s_at	polo-like kinase 1	PLK1S1	9.07	8.93	9.14
211516_at	interleukin 5 rece	IL5RA	3.54	3.43	3.57
209324_s_at	regulator of G-prc	RGS16	7.53	7.24	7.49
206785_s_at	killer cell lectin-lik	KLRC1 /// KLRC2	7.36	7.37	7.49
212914_at	chromobox homo	CBX7	7.18	7.15	7.04
204385_at	kynureninase (L-k	KYNU	6.04	6.29	6.01
216363_at	---	---	6.54	6.41	6.17
212690_at	DDHD domain cor	DDHD2	9.19	9.28	9.37
214224_s_at	protein (peptidylp	PIN4	8.80	8.88	8.79
209050_s_at	ral guanine nucle	RALGDS	8.16	8.21	8.10
216451_at	Serine/threonine	STK38	5.66	5.88	5.78
206317_s_at	ATP-binding casse	ABCB8	7.07	7.16	6.91
201052_s_at	proteasome (pros	PSMF1	8.44	8.40	8.47
205902_at	potassium interm	KCNN3	3.92	4.01	3.89
221063_x_at	ring finger protei	RNF123	6.91	6.63	6.60
221875_x_at	major histocompa	HLA-F	9.74	9.57	9.70
221059_s_at	coactosin-like 1 (D	COTL1	11.54	11.42	11.47
213925_at	Chromosome 1 o	C1orf95	4.92	5.06	4.88
221700_s_at	ubiquitin A-52 res	UBA52	13.93	13.95	14.00
210970_s_at	inhibitor of Bruto	IBTK	9.17	9.26	9.49
211473_s_at	collagen, type IV,	COL4A6	7.31	7.24	7.01
204767_s_at	flap structure-spe	FEN1	10.08	10.31	10.16
214035_x_at	GPS, PLAT and tra	LOC399491	9.49	9.33	9.36
216892_at	Immunoglobulin h	IGHG1	6.29	6.43	6.27
215450_at	---	---	8.83	9.08	9.01
204901_at	beta-transducin re	BTRC	6.35	6.46	6.58
217518_at	myoferlin	MYOF	7.58	7.59	7.40
202851_at	ATP-binding casse	ABCD3	7.69	7.62	7.68
204436_at	pleckstrin homolo	PLEKHO2	8.22	8.25	8.05
201697_s_at	DNA (cytosine-5-)	DNMT1	11.05	11.01	10.97
210113_s_at	NLR family, pyrin	NLRP1	6.62	6.49	6.50
203713_s_at	lethal giant larvae	LLGL2	5.51	5.66	5.37
209454_s_at	TEA domain famil	TEAD3	6.72	6.80	7.01
209429_x_at	eukaryotic transla	EIF2B4	9.84	9.77	9.77
210711_at	chromosome 1 op	C1orf217	5.13	5.37	5.20
222019_at	prefoldin subunit	PFDN6	6.50	6.60	6.43
201999_s_at	dynein, light chair	DYNLT1	12.65	12.64	12.63
205944_s_at	clathrin, heavy ch	CLTCL1	7.35	7.26	7.21
222363_at	---	---	5.74	5.77	5.60
216944_s_at	inositol 1,4,5-tripl	ITPR1	7.74	7.69	7.95
221853_s_at	NODAL modulato	NOMO1 /// NOMC	10.08	9.90	9.95
219616_at	acyl-CoA syntheta	ACSS3	4.58	4.22	4.64
222328_x_at	Maternally expres	MEG3	4.56	4.70	4.52
211014_s_at	golgin A6 family-li	GOLGA6L4 /// PM	7.23	7.00	6.89
214430_at	galactosidase, alp	GLA	9.57	9.71	9.60
204727_at	WD repeat and HI	WDHD1	7.07	7.14	7.10

209722_s_at	serpin peptidase i	SERPINB9	4.69	4.74	4.92
209438_at	phosphorylase kir	PHKA2	6.15	6.32	6.40
220353_at	family with seque	FAM86C	7.11	7.18	7.01
210836_x_at	phosphodiesteras	PDE4D	3.76	3.90	3.74
221882_s_at	transmembrane p	TMEM8A	7.39	7.14	7.48
204461_x_at	RAD1 homolog (S.	RAD1	10.45	10.45	10.62
204422_s_at	fibroblast growth	FGF2	8.78	8.60	9.17
202125_s_at	trafficking protein	TRAK2	9.86	9.95	9.94
221094_s_at	elongation protei	ELP3	8.52	8.42	8.42
201715_s_at	apoptotic chroma	ACIN1	8.22	8.40	8.25
203938_s_at	TATA box binding	TAF1C	8.35	8.38	8.22
220882_at	---	---	5.16	5.03	4.66
220815_at	catenin (cadherin-	CTNNA3	3.91	4.04	3.80
214839_at	hypothetical LOC1	LOC157627	5.86	5.92	5.84
207579_at	melanoma antigei	MAGEB3	4.28	4.23	3.96
206038_s_at	nuclear receptor s	NR2C2	5.92	5.88	6.07
219895_at	family with seque	FAM70A	3.29	2.88	3.21
211031_s_at	CAP-GLY domain c	CLIP2	5.98	5.95	6.07
216639_at	sushi-repeat-cont	SRPX2	3.11	3.10	3.14
217560_at	golgi-associated, g	GGA1	7.48	7.32	7.14
205590_at	RAS guanyl releas	RASGRP1	4.06	3.64	3.80
207992_s_at	adenosine monop	AMPD3	6.93	6.77	7.09
201880_at	ariadne homolog,	ARIH1	8.57	8.63	8.80
220509_at	RNA binding moti	RBM26	3.70	3.80	3.66
217444_at	---	---	6.21	6.33	6.05
203199_s_at	5-methyltetrahyd	MTRR	8.26	8.43	8.60
212417_at	secretory carrier r	SCAMP1	9.24	9.38	9.49
208446_s_at	zinc finger, FYVE c	ZFYVE9	5.49	5.49	5.14
218353_at	regulator of G-prc	RGS5	5.03	5.08	4.83
212712_at	calmodulin regula	CAMSAP1	8.04	8.02	8.15
215246_at	La ribonucleoprot	LARP7	5.51	5.27	5.42
207133_x_at	alpha-kinase 1	ALPK1	7.65	7.63	7.55
216206_x_at	mitogen-activatec	MAP2K7	6.32	6.30	6.52
212084_at	testis expressed 2	TEX261	7.80	7.53	7.66
208260_at	arginine vasopres:	AVPR1B	7.97	7.95	7.77
218120_s_at	heme oxygenase (	HMOX2	8.51	8.52	8.52
204972_at	2'-5'-oligoadenyla	OAS2	4.17	4.35	4.48
205317_s_at	solute carrier fam	SLC15A2	5.10	5.14	4.99
208611_s_at	spectrin, alpha, nc	SPTAN1	7.37	7.45	7.30
207317_s_at	calsequestrin 2 (ca	CASQ2	5.46	5.52	5.12
222384_at	DKFZP564C196 pr	DKFZP564C196	4.57	4.07	4.26
222187_x_at	GTPase activating	G3BP1	6.41	6.54	6.43
220318_at	epsin 3	EPN3	3.81	4.16	4.06
221351_at	5-hydroxytryptam	HTR1A	4.88	5.20	4.99
215503_at	serine peptidase i	SPINT3	3.39	3.76	3.55
214944_at	PH domain and le	PHLPP2	4.36	4.78	4.26
217150_s_at	neurofibromin 2 (	NF2	6.38	6.65	6.38

209832_s_at	chromatin licensir	CDT1	7.91	7.69	7.49
202841_x_at	TAF15 RNA polym	TAF15	8.45	8.21	8.21
212861_at	major facilitator s	MFSD5	7.94	8.24	7.99
214610_at	cytochrome P450,	CYP11B1	4.04	3.88	3.82
221936_x_at	Mitochondrial rib	MRPL41	6.32	6.44	6.33
208472_at	IKAROS family zin	IKZF4	6.56	6.55	6.27
221825_at	angel homolog 2 (	ANGEL2	9.68	9.65	9.77
209230_s_at	nuclear protein, tı	NUPR1	10.55	9.98	10.52
204950_at	caspase recruitme	CARD8	6.56	6.62	6.35
201483_s_at	suppressor of Ty 4	SUPT4H1	9.59	9.56	9.64
213951_s_at	PSMC3 interacting	PSMC3IP	8.56	8.56	8.71
211989_at	SWI/SNF related,	SMARCE1	10.52	10.54	10.54
206616_s_at	ADAM metallopep	ADAM22	5.33	5.20	5.22
218155_x_at	TSR1, 20S rRNA ac	TSR1	8.58	8.51	8.50
213324_at	v-src sarcoma (Sch	SRC	7.76	7.76	7.68
207342_at	cyclic nucleotide g	CNGB1	4.11	4.16	4.17
211117_x_at	estrogen receptor	ESR2	4.76	4.41	4.51
201209_at	histone deacetyla	HDAC1	10.11	10.26	10.13
217840_at	DEAD (Asp-Glu-Ala	DDX41	9.22	9.30	9.14
210425_x_at	golgin A8 family, r	GOLGA8B	7.78	8.10	7.83
207809_s_at	ATPase, H+ transp	ATP6AP1	8.92	8.85	8.85
214243_s_at	serine hydrolase-l	SERHL /// SERHL2	6.11	6.13	5.69
215441_at	---	---	3.15	3.32	3.29
201103_x_at	neuroblastoma br	LOC200030 /// NB	11.84	11.91	11.93
212275_s_at	Snf2-related CREB	SRCAP	7.92	7.43	7.76
204595_s_at	stanniocalcin 1	STC1	7.68	7.90	7.88
222244_s_at	taurine upregulate	TUG1	9.79	9.95	10.11
217395_at	metallothionein 4	MT4	5.72	5.93	5.75
52731_at	autophagy/beclin-	AMBRA1	7.41	7.33	7.44
214947_at	family with seque	FAM105A	3.69	3.92	3.62
218340_s_at	ubiquitin-like moc	UBA6	8.01	8.32	8.36
208353_x_at	ankyrin 1, erythro	ANK1	7.08	6.95	6.68
213630_at	NAC alpha domair	NACAD	5.73	5.59	5.74
201227_s_at	NADH dehydroger	NDUFB8	11.73	11.71	11.81
202360_at	mastermind-like 1	MAML1	9.76	9.72	9.88
219766_at	B9 protein domaiı	B9D2	7.28	7.14	7.11
205898_at	chemokine (C-X3-	CX3CR1	4.00	3.64	3.88
207038_at	solute carrier fam	SLC16A6	6.14	6.00	6.13
221342_at	chromosome 6 op	C6orf25	6.06	5.70	5.67
207141_s_at	potassium inward	KCNJ3	4.71	4.63	4.18
211496_s_at	phosducin	PDC	3.77	3.72	3.55
205384_at	FXYD domain cont	FXYD1	6.98	6.94	6.84
209122_at	perilipin 2	PLIN2	4.95	4.98	4.91
209802_at	---	---	6.22	6.10	6.05
202520_s_at	mutL homolog 1,	MLH1	10.90	10.94	10.85
207824_s_at	MYC-associated zi	MAZ	4.99	5.02	4.95
200877_at	chaperonin contai	CCT4	13.03	13.03	13.02

210792_x_at	SIVA1, apoptosis-i	SIVA1	10.47	10.45	10.43
220452_x_at	---	---	7.29	7.55	7.72
202922_at	glutamate-cystein	GCLC	8.49	8.40	8.72
206289_at	homeobox A4	HOXA4	6.71	6.54	6.36
211877_s_at	protocadherin gar	PCDHGA11	4.38	4.64	4.29
213845_at	glutamate recepto	GRIK2	5.41	5.24	5.37
221390_s_at	myotubularin rela	MTMR8	6.66	6.77	6.40
206089_at	NEL-like 1 (chicke	NELL1	5.21	5.29	5.18
218240_at	NFKB inhibitor int	NKIRAS2	8.17	8.26	8.21
201937_s_at	aspartyl aminope	DNPEP	8.02	8.03	7.97
204171_at	ribosomal protein	RPS6KB1	8.17	8.14	8.37
217562_at	family with seque	FAM5C	3.18	3.21	3.10
213964_x_at	---	---	3.52	3.97	3.90
219341_at	ceroid-lipofuscino	CLN8	5.35	4.92	4.95
205424_at	TBK1 binding prot	TBKBP1	6.86	6.41	6.39
207855_s_at	chloride channel (	CLCC1	8.22	8.20	8.45
208981_at	platelet/endothel	PECAM1	5.31	5.03	4.98
209158_s_at	cytohesin 2	CYTH2	7.79	7.65	7.59
202192_s_at	growth arrest-spe	GAS7	6.62	6.49	6.33
207582_at	peptidylprolyl cis/	PIN1L	7.23	6.93	6.83
207713_s_at	RanBP-type and C	RBCK1	7.21	7.30	7.55
202695_s_at	serine/threonine	STK17A	6.90	7.04	7.11
209693_at	astrotactin 2	ASTN2	6.39	6.28	6.21
203530_s_at	syntaxin 4	STX4	8.94	8.91	8.87
202573_at	casein kinase 1, g	CSNK1G2	9.80	9.77	9.97
203022_at	ribonuclease H2, s	RNASEH2A	10.09	10.06	10.25
204599_s_at	mitochondrial rib	MRPL28	9.63	9.50	9.63
212399_s_at	vestigial like 4 (Dr	VGLL4	8.19	8.21	8.25
206240_s_at	zinc finger protein	ZNF136	6.68	6.66	6.41
220861_at	---	---	4.52	4.46	4.65
220875_at	---	---	5.13	5.11	4.80
214099_s_at	phosphodiesteras	PDE4DIP	5.87	5.80	5.85
204657_s_at	Src homology 2 do	SHB	6.31	6.46	6.03
211735_x_at	surfactant protein	SFTPC	6.57	6.43	6.26
215787_at	Actin, alpha 2, sm	ACTA2	3.51	3.71	3.49
222198_at	---	---	6.39	6.26	6.10
207502_at	guanylate cyclase	GUCA2B	5.12	5.36	4.96
204324_s_at	golgi integral men	GOLIM4	8.36	8.46	8.48
222147_s_at	ARP5 actin-relate	ACTR5	6.41	6.58	6.40
207263_x_at	vezatin, adherens	VEZT	5.63	5.49	5.43
214448_x_at	nuclear factor of k	NFKBIB	8.01	8.01	8.05
220563_s_at	SH3 and multiple	SHANK1	6.16	6.04	6.01
203586_s_at	ADP-ribosylation f	ARL4D	8.31	8.23	8.31
210936_at	WD repeat domai	WDR1	5.67	5.20	5.32
211838_x_at	protocadherin alp	PCDHA5	5.79	5.70	5.62
222201_s_at	caspase 8 associat	CASP8AP2	8.18	8.45	8.54
200981_x_at	GNAS complex loc	GNAS	12.14	12.13	12.06

214781_at	---	---	5.43	5.30	5.21
215280_s_at	protein tyrosine p	PPFIA3	7.52	7.19	7.14
216965_x_at	spastic paraplegia	SPG20	6.36	6.56	6.25
211938_at	eukaryotic transla	EIF4B	11.54	11.52	11.66
219302_s_at	contactin associat	CNTNAP2	3.71	3.75	4.02
214872_at	RAP1 interacting f	RIF1	5.32	5.07	4.99
219415_at	tweety homolog 1	TTYH1	3.78	4.12	3.85
204902_s_at	ATG4 autophagy r	ATG4B	7.00	6.79	6.81
213627_at	melanoma antiger	MAGED2	8.44	8.51	8.48
218955_at	BRF2, subunit of F	BRF2	8.73	8.63	8.69
202942_at	electron-transfer-	ETFB	9.65	9.69	9.62
203720_s_at	excision repair crc	ERCC1	9.89	9.72	9.85
206677_at	keratin 31	KRT31	4.43	4.58	4.05
204052_s_at	secreted frizzled-r	SFRP4	3.53	3.57	3.36
221105_at	---	---	4.70	4.81	4.61
206254_at	epidermal growth	EGF	4.04	4.09	3.92
217015_at	retinoblastoma bi	RBBP4	3.60	3.79	3.71
207245_at	UDP glucuronosyl	UGT2B17	3.23	3.32	3.10
219860_at	lymphocyte antige	LY6G5C	4.83	4.51	4.21
214969_at	mitogen-activatec	MAP3K9	5.82	5.82	5.85
219166_at	chromosome 14 c	C14orf104	9.67	9.72	9.75
206125_s_at	kallikrein-related		KLK8	6.75	6.82	6.43
205634_x_at	zinc finger, DHHC-	ZDHHC24	7.86	7.67	7.59
219996_at	ankyrin repeat an	ASB7	6.40	6.45	6.71
218178_s_at	chromatin modify	CHMP1B	9.62	9.58	9.66
211686_s_at	MAK16 homolog (	MAK16	10.57	10.46	10.59
208298_at	ecotropic viral int	EVI5	4.03	4.04	3.69
201556_s_at	vesicle-associated	VAMP2	5.24	5.28	4.94
213568_at	odd-skipped relat	OSR2	5.22	5.07	5.20
210422_x_at	solute carrier fam	SLC11A1	7.07	6.96	6.87
206068_s_at	acyl-CoA dehydro	ACADL	4.51	4.35	4.52
220263_at	SMAD family men	SMAD5OS	4.63	4.73	4.68
200950_at	actin related prot	ARPC1A	10.76	10.83	10.78
200062_s_at	ribosomal protein	RPL30	14.82	14.75	14.78
204520_x_at	bromodomain cor	BRD1	7.68	7.69	7.83
216311_at	similar to OVO ho	LOC728361	5.82	5.59	5.69
215372_x_at	---	---	7.58	7.53	7.31
202563_at	chromosome 14 c	C14orf1	5.59	5.37	5.67
205649_s_at	fibrinogen alpha c	FGA	3.11	3.14	3.07
207842_s_at	cancer susceptibil	CASC3	9.19	9.07	9.23
205304_s_at	potassium inward	KCNJ8	4.49	4.65	4.77
216102_at	pleckstrin homolo	PHLDB1	5.67	5.61	5.45
208971_at	uroporphyrinoger	UROD	9.65	9.63	9.75
207673_at	nephrosis 1, cong	NPHS1	4.42	4.44	4.19
211478_s_at	dipeptidyl-peptida	DPP4	3.74	3.78	3.69
214508_x_at	cAMP responsive	CREM	7.97	8.18	7.84
220812_s_at	HERV-H LTR-assoc	HHLA2	4.90	4.36	4.44

204138_s_at	myeloid zinc finge	MZF1	5.69	5.62	5.49
205826_at	myomesin (M-pro	MYOM2	5.71	5.68	5.62
36888_at	HAUS augmin-like	HAUS5	8.03	8.30	8.24
211062_s_at	carboxypeptidase	CPZ	7.15	7.11	6.91
209206_at	SEC22 vesicle traf	SEC22B	8.78	8.85	8.86
213853_at	DnaJ (Hsp40) hom	DNAJC24	8.44	8.58	8.56
210074_at	cathepsin L2	CTSL2	7.49	7.52	7.33
215783_s_at	alkaline phosphat	ALPL	4.57	4.57	4.45
219501_at	ecto-NOX disulfid	ENOX1	7.29	7.35	7.27
208973_at	ERI1 exoribonucle	ERI3	6.76	6.80	6.75
204768_s_at	flap structure-spe	FEN1	8.28	8.75	8.47
201749_at	endothelin conve	ECE1	8.69	8.45	8.45
204368_at	solute carrier org	SLCO2A1	6.94	7.00	6.79
207493_x_at	synovial sarcoma,	SSX2 /// SSX2B	4.53	4.75	4.81
206700_s_at	lysine (K)-specific	KDM5D	4.17	4.27	3.84
215401_at	---	---	7.35	7.25	7.13
209946_at	vascular endothel	VEGFC	6.67	6.70	6.87
216235_s_at	endothelin recept	EDNRA	5.63	5.70	5.36
213953_at	keratin 20	KRT20	3.44	3.21	3.17
220309_at	tetratricopeptide	TTC22	4.40	4.59	4.14
208089_s_at	tudor domain con	TDRD3	6.91	7.11	7.16
209962_at	erythropoietin rec	EPOR	4.66	4.37	4.83
210210_at	myelin protein ze	MPZL1	7.23	7.30	7.16
212770_at	transducin-like en	TLE3	7.48	7.40	7.58
203062_s_at	mediator of DNA-	MDC1	9.49	9.55	9.57
221954_at	Chromosome 20 c	C20orf111	9.01	8.73	8.96
217384_x_at	Immunoglobulin h	IGHV3-48 /// IGKV	5.78	5.54	5.66
212519_at	ubiquitin-conjuga	UBE2E1	12.87	12.95	13.06
215574_at	---	---	5.91	5.64	5.80
211469_s_at	chemokine (C-X-C	CXCR6	4.90	4.87	4.62
207821_s_at	PTK2 protein tyro	PTK2	8.63	8.64	8.66
204806_x_at	major histocompa	HLA-F	9.71	9.64	9.70
216407_at	Vac14 homolog (S	VAC14	6.37	6.18	5.90
214941_s_at	PRP40 pre-mRNA	PRPF40A	10.20	10.34	10.07
216759_at	HRAS-like suppres	HRASLS2	6.36	6.11	6.04
209263_x_at	tetraspanin 4	TSPAN4	10.12	9.90	9.95
208838_at	cullin-associated a	CAND1	9.85	9.92	10.08
203279_at	ER degradation er	EDEM1	9.25	9.14	9.21
217013_at	---	---	4.38	4.24	4.01
215169_at	solute carrier fam	SLC35E2	7.35	7.42	7.41
213038_at	ring finger proteir	RNF19B	7.11	6.95	6.94
218631_at	arginine vasopres	AVPI1	9.18	8.91	9.16
221125_s_at	potassium large c	KCNMB3	4.84	5.11	5.14
213847_at	peripherin	PRPH	6.91	7.04	6.54
204570_at	cytochrome c oxic	COX7A1	4.65	4.31	4.47
211542_x_at	ribosomal protein	RPS10	15.03	15.02	15.03
204285_s_at	phorbol-12-myrist	PMAIP1	10.07	9.81	9.95

208193_at	interleukin 9	IL9	3.46	3.41	3.11
217817_at	actin related prot	ARPC4	9.22	9.21	9.03
208405_s_at	CD164 molecule, s	CD164	11.24	11.25	11.50
215275_at	TRAF3 interacting	TRAF3IP3	4.41	4.76	4.52
213074_at	pleckstrin homolo	PHIP	10.15	10.20	10.48
207272_at	zinc finger protein	ZNF80	3.24	3.17	3.21
216375_s_at	ets variant 5	ETV5	7.17	6.94	7.06
207499_x_at	unc-45 homolog A	UNC45A	6.65	6.84	6.90
218358_at	cysteine-rich with	CRELD2	10.55	10.65	10.62
206569_at	interleukin 24	IL24	5.01	5.02	4.96
221545_x_at	mediator complex	MED16	7.79	7.73	7.81
222066_at	Erythrocyte meml	EPB41L1	5.42	5.29	5.22
208834_x_at	ribosomal protein	RPL23A	15.13	15.13	15.15
206808_at	heterogeneous nu	HNRNPA3P1	3.43	3.47	3.49
216249_at	Pvt1 oncogene (n	PVT1	5.82	5.51	5.53
216557_x_at	immunoglobulin h	IGHA1 /// IGHD //,	4.87	5.26	4.92
204938_s_at	phospholamban	PLN	3.05	3.27	3.05
214889_at	family with seque	FAM149A	4.37	4.10	4.15
202067_s_at	low density lipopr	LDLR	7.67	8.08	7.85
220538_at	adrenomedullin 2	ADM2	7.90	7.88	7.90
212583_at	aquarius homolog	AQR	7.39	7.27	7.28
215514_at	---	---	3.27	3.27	2.97
208769_at	eukaryotic transla	EIF4EBP2	5.54	5.75	5.73
204537_s_at	gamma-aminobut	GABRE	6.38	6.50	6.27
216201_at	---	---	4.19	4.46	4.18
213128_s_at	ubiquitin protein l	UBE3A	8.51	8.56	8.91
211807_x_at	protocadherin gar	PCDHGB5	7.03	7.08	6.99
211730_s_at	polymerase (RNA)	POLR2L	12.26	12.28	12.28
210945_at	collagen, type IV,	COL4A6	6.49	6.42	6.71
210765_at	CSE1 chromosome	CSE1L	5.99	6.09	5.95
218086_at	neural proliferatic	NPDC1	8.57	8.66	8.67
210271_at	neurogenic differe	NEUROD2	5.48	5.50	5.33
203415_at	programmed cell	PDCD6	12.10	12.13	12.00
221806_s_at	SET domain conta	SETD5	9.52	9.54	9.57
207205_at	carcinoembryonic	CEACAM4	7.65	7.56	7.13
203729_at	epithelial membra	EMP3	9.04	9.04	9.04
220377_at	family with seque	FAM30A	5.90	5.80	5.64
206340_at	nuclear receptor s	NR1H4	3.75	3.80	3.61
208435_s_at	aquaporin 6, kidn	AQP6	6.00	5.67	5.39
215230_x_at	eukaryotic transla	EIF3C /// EIF3CL	12.43	12.25	12.36
207883_s_at	transferrin recept	TFR2	6.54	6.34	6.38
219534_x_at	cyclin-dependent	CDKN1C	6.78	6.55	6.53
214427_at	NOP2 nucleolar pı	NOP2	8.93	8.85	9.06
207685_at	crystallin, beta B3	CRYBB3	7.05	6.80	6.66
204827_s_at	cyclin F	CCNF	6.83	6.77	6.83
213941_x_at	ribosomal protein	RPS7	14.54	14.53	14.58
206064_s_at	peptidylprolyl isor	PPIL2	6.37	6.44	6.48

214075_at	neuron derived ne	NENF	9.03	9.01	9.04
213172_at	tetratricopeptide	TTC9	3.20	3.30	3.15
200721_s_at	ARP1 actin-relatec	ACTR1A	8.37	8.35	8.39
203359_s_at	c-myc binding pro	MYCBP	9.75	9.68	9.84
222126_at	ArfGAP with FG re	AGFG2	8.08	8.03	8.28
201720_s_at	lysosomal protein	LAPTM5	8.69	8.73	8.93
216951_at	Fc fragment of IgG	FCGR1A	3.83	3.91	3.87
214408_s_at	RFPL1 antisense R	RFPL1S /// RFPL3S	4.86	4.76	4.81
212124_at	zinc finger, MIZ-ty	ZMIZ1	10.04	9.98	10.17
214378_at	tissue factor path	TFPI	5.28	5.21	5.29
208051_s_at	poly(A) binding pr	PAIP1	10.37	10.33	10.54
204873_at	peroxisomal bioge	PEX1	6.49	6.83	6.77
203821_at	heparin-binding E	HBEGF	7.63	7.62	7.79
215896_at	---	---	4.20	4.27	4.09
216329_at	---	---	6.10	6.17	5.96
219654_at	protein tyrosine p	PTPLA	9.80	9.84	9.80
221952_x_at	TRM5 tRNA meth	TRMT5	12.96	12.98	13.09
205825_at	proprotein conver	PCSK1	5.42	5.41	5.38
209505_at	Nuclear receptor s	NR2F1	3.83	3.74	3.71
203968_s_at	cell division cycle	CDC6	8.36	8.53	8.46
209948_at	potassium large co	KCNMB1	4.49	4.11	4.33
204862_s_at	non-metastatic ce	NME3	9.50	9.34	9.40
207514_s_at	guanine nucleotid	GNAT1	4.99	4.74	4.92
48825_at	inhibitor of growt	ING4	7.61	7.66	7.65
221510_s_at	glutaminase	GLS	10.77	10.89	11.01
222314_x_at	eosinophil granule	EGOT	4.50	4.80	4.45
208317_at	xylulokinase homo	XYLB	3.65	3.40	3.26
221811_at	post-GPI attachme	PGAP3	7.45	7.05	7.21
34726_at	calcium channel, v	CACNB3	6.79	6.71	6.84
220884_at	---	---	3.28	3.09	3.10
208163_s_at	oxysterol binding	OSBPL7	5.89	5.67	5.74
216596_at	hypothetical LOC2	DKFZP434L187	5.53	5.47	5.47
41160_at	methyl-CpG bindi	MBD3	9.38	9.32	9.43
205767_at	epiregulin	EREG	10.48	10.71	10.93
209251_x_at	tubulin, alpha 1c	TUBA1C	14.57	14.62	14.53
201736_s_at	membrane-associ	6-Mar	9.25	9.46	9.40
216241_s_at	transcription elon	TCEA1	12.14	11.98	12.17
213590_at	solute carrier fam	SLC16A5	5.43	5.40	5.22
201478_s_at	dyskeratosis cong	DKC1	11.84	11.70	11.82
206284_x_at	clathrin, light chai	CLTB	8.98	9.13	9.11
220359_s_at	cyclic AMP-regula	ARPP21	3.32	3.59	3.48
201250_s_at	solute carrier fam	SLC2A1	9.00	8.78	8.99
220772_at	blepharophimosis	BPESC1	3.38	3.30	3.34
205224_at	surfeit 2	SURF2	8.49	8.44	8.48
207293_s_at	angiotensin II rece	AGTR2	3.97	4.23	4.17
214667_s_at	tumor protein p53	TP53I11	6.21	6.16	5.93
213477_x_at	eukaryotic transla	EEF1A1	14.88	14.89	14.90

207298_at	solute carrier fam	SLC17A3	4.28	4.14	4.32
213885_at	tripartite motif-cc	TRIM3	6.87	6.93	6.69
220648_at	adenosine deamir	ADARB2	5.00	5.01	4.87
204226_at	staufen, RNA bind	STAU2	7.95	7.99	8.12
217947_at	CKLF-like MARVEL	CMTM6	10.93	11.01	11.02
214045_at	lipoic acid synthet	LIAS	9.69	9.66	9.77
216234_s_at	protein kinase, cA	PRKACA	5.75	5.85	5.56
203391_at	FK506 binding prc	FKBP2	9.20	9.03	9.25
211555_s_at	guanylate cyclase	GUCY1B3	3.94	3.69	3.60
220131_at	FXYD domain cont	FXYD7	4.81	4.45	4.98
37022_at	proline/arginine-r	PRELP	6.65	6.59	6.85
204820_s_at	butyrophilin, subf	BTN3A2 /// BTN3A	9.19	9.44	9.45
219224_x_at	zinc finger proteir	ZNF408	8.55	8.55	8.38
202356_s_at	general transcript	GTF2F1	8.19	8.30	8.28
201884_at	carcinoembryonic	CEACAM5	4.09	3.94	4.22
216341_s_at	gonadotropin-rele	GNRHR	4.21	4.16	4.12
206035_at	v-rel reticuloendo	REL	4.34	4.35	4.13
211762_s_at	karyopherin alpha	KPNA2	12.76	12.91	12.82
206898_at	cadherin 19, type	CDH19	3.92	3.81	4.21
217041_at	neuronal pentraxi	NPTXR	7.65	7.65	7.37
215320_at	hypothetical prote	LOC645961	4.03	4.01	3.86
208380_at	ladybird homeobc	LBX1	5.81	5.44	5.34
217462_at	chromosome 11 c	C11orf9	6.97	6.48	6.72
206226_at	histidine-rich glyc	HRG	4.08	4.43	4.30
206921_at	GLE1 RNA export	GLE1	4.61	4.38	4.40
212784_at	capicua homolog	CIC	7.79	7.67	7.54
222368_at	---	---	6.68	6.80	6.33
206507_at	zinc finger and SC	ZSCAN12	7.84	7.94	8.13
207796_x_at	killer cell lectin-lik	KLRD1	4.30	4.04	4.03
206828_at	TXK tyrosine kinas	TXK	4.07	4.25	3.89
213877_x_at	transcription elon	TCEB2	8.47	8.37	8.32
201437_s_at	eukaryotic transla	EIF4E	9.00	9.13	9.23
205675_at	microsomal triglyc	MTTP	3.74	3.85	3.50
209484_s_at	NSL1, MIND kinet	NSL1	10.12	10.18	10.20
210365_at	runt-related trans	RUNX1	6.05	5.97	5.55
37004_at	surfactant proteir	SFTPB	6.30	5.85	5.98
207107_at	retinal pigment ep	RPE65	3.05	3.02	3.15
221624_at	T-cell leukemia/ly	TCL6	4.42	4.27	4.23
209404_s_at	transmembrane e	TMED7	9.17	9.28	9.51
219197_s_at	signal peptide, CU	SCUBE2	3.95	3.84	3.86
205875_s_at	three prime repai	TREX1	7.43	7.40	7.42
206685_at	HLA complex grou	HCG4	4.89	5.20	4.91
217249_x_at	---	---	10.49	10.52	10.60
219748_at	triggering recepto	TREML2	6.80	6.65	6.43
219880_at	---	---	8.99	9.13	9.05
201707_at	peroxisomal bioge	PEX19	9.00	8.96	8.87
215467_x_at	hypothetical LOC6	LOC647070	6.22	6.38	6.26

202599_s_at	nuclear receptor i	NRIP1	8.64	8.58	8.75
205737_at	potassium voltage	KCNQ2	5.73	5.47	5.28
202203_s_at	autocrine motility	AMFR	9.72	9.67	9.87
217470_at	---	---	3.33	3.27	3.39
220225_at	iroquois homeobo	IRX4	5.50	5.19	5.24
209959_at	nuclear receptor s	NR4A3	4.80	4.76	4.75
200720_s_at	ARP1 actin-relatec	ACTR1A	9.34	9.31	9.38
221621_at	chromosome 17 o	C17orf86	7.87	8.05	8.17
201158_at	N-myristoyltransfe	NMT1	8.14	8.14	8.12
220554_at	solute carrier fam	SLC22A7	6.04	5.79	6.01
211567_at	---	---	3.51	3.40	3.20
207727_s_at	mutY homolog (E.	MUTYH	8.02	7.87	7.92
202603_at	---	---	10.57	10.75	10.72
202547_s_at	Rho guanine nucle	ARHGEF7	6.52	6.64	6.54
222004_s_at	dedicator of cytok	DOCK6	6.02	6.14	5.63
202569_s_at	MAP/microtubule	MARK3	9.16	9.10	9.37
207052_at	hepatitis A virus c	HAVCR1	3.53	3.48	3.69
59644_at	BMP2 inducible ki	BMP2K	7.82	7.84	8.14
205563_at	KiSS-1 metastasis-	KISS1	5.07	4.95	5.21
216377_x_at	alkaline phosphat	ALPPL2	7.23	7.00	7.20
210107_at	chloride channel a	CLCA1	4.30	4.33	4.53
207361_at	HMG-box transcri	HBP1	4.21	4.09	3.92
205136_s_at	nuclear fragile X n	NUFIP1	7.88	7.88	8.02
218828_at	phospholipid scra	PLSCR3	7.54	7.57	7.67
207450_s_at	POU class 6 home	POU6F2	4.57	4.92	4.71
205247_at	Notch homolog 4	NOTCH4	6.06	6.08	5.80
210309_at	RecQ protein-like	RECQL5	5.77	5.26	5.41
202714_s_at	KIAA0391	KIAA0391	6.26	6.54	6.59
212166_at	exportin 7	XPO7	8.69	8.67	8.74
205265_s_at	SPEG complex loc	SPEG	7.40	7.03	6.98
206328_at	cadherin 15, type	CDH15	6.42	6.06	6.39
210966_x_at	La ribonucleoprot	LARP1	8.54	8.47	8.46
211971_s_at	leucine-rich PPR-r	LRPPRC	11.20	11.31	11.31
213136_at	protein tyrosine p	PTPN2	10.00	10.07	10.14
216994_s_at	runt-related trans	RUNX2	4.40	4.24	4.17
217679_x_at	---	---	9.75	9.76	9.92
208480_s_at	ATP-binding casse	ABCC6 /// LOC100	4.99	4.68	5.04
202713_s_at	KIAA0391	KIAA0391	9.14	9.13	9.26
219963_at	dual specificity ph	DUSP13	5.79	5.64	5.72
208660_at	citrate synthase	CS	11.22	11.17	11.34
208590_x_at	gap junction prote	GJA3	7.17	7.12	6.96
208078_s_at	salt-inducible kina	SIK1	8.96	8.75	8.93
57739_at	dead end homolo	DND1	7.61	7.90	7.64
206478_at	KIAA0125	KIAA0125	4.08	4.01	4.01
209904_at	troponin C type 1	TNNC1	4.51	4.65	4.64
211118_x_at	estrogen receptor	ESR2	5.21	4.89	4.88
210070_s_at	choline kinase-like	CHKB-CPT1B /// CI	6.48	6.79	6.69

201511_at	angio-associated,	AAMP	9.91	9.84	9.75
210921_at	---	---	4.30	4.20	4.24
202403_s_at	collagen, type I, al	COL1A2	5.10	5.12	5.01
221463_at	chemokine (C-C m	CCL24	6.22	5.98	6.09
221762_s_at	PDX1 C-terminal i	PCIF1	7.13	7.09	6.94
215944_at	---	---	3.79	3.71	3.53
205477_s_at	alpha-1-microglob	AMBP	5.15	5.50	5.11
210933_s_at	fascin homolog 1,	FSCN1	7.12	7.19	7.37
204170_s_at	CDC28 protein kin	CKS2	12.84	12.91	12.99
206278_at	platelet-activating	PTAFR	6.22	5.92	6.09
206204_at	growth factor rece	GRB14	3.81	3.63	3.67
215538_at	like-glycosyltransf	LARGE	5.52	5.25	5.13
220559_at	engrailed homeob	EN1	4.42	4.43	4.34
219194_at	sema domain, imr	SEMA4G	5.58	5.40	5.40
209988_s_at	achaete-scute cor	ASCL1	5.44	5.30	5.52
210441_at	CDC14 cell divisio	CDC14A	4.86	4.67	4.67
204327_s_at	zinc finger protein	ZNF202	9.06	8.87	8.97
217282_at	mannosidase, alpl	MAN1A2	4.16	3.83	3.75
218965_s_at	terminal uridylyl t	TUT1	7.50	7.65	7.31
201122_x_at	eukaryotic transla	EIF5A	10.21	10.17	10.09
201895_at	v-raf murine sarco	ARAF	7.63	7.64	7.63
208452_x_at	myosin IXB	MYO9B	6.67	6.58	6.66
214063_s_at	transferrin	TF	5.89	6.06	5.80
218333_at	Der1-like domain	DERL2	8.75	8.73	8.83
205332_at	RCE1 homolog, pr	RCE1	6.31	6.25	6.00
209224_s_at	NADH dehydroger	NDUFA2	11.18	11.31	11.28
216278_at	---	---	4.10	4.12	4.10
210904_s_at	interleukin 13 rec	IL13RA1	7.31	7.19	7.18
214455_at	histone cluster 1,	HIST1H2BC	4.87	4.53	4.62
217700_at	Canopy 4 homolo	CNPY4	6.93	6.35	6.64
211795_s_at	FYN binding prote	FYB	4.27	4.21	4.18
211131_s_at	ectodysplasin A	EDA	3.53	3.62	3.56
216605_s_at	carcinoembryonic	CEACAM21	4.82	4.51	4.39
218236_s_at	protein kinase D3	PRKD3	9.66	9.76	9.92
90265_at	ArfGAP with dual	ADAP1	7.79	7.82	7.60
218528_s_at	ring finger protein	RNF38	8.27	8.39	8.46
205775_at	family with seque	FAM50B	7.15	7.07	6.92
208890_s_at	plexin B2	PLXNB2	9.52	9.41	9.44
216184_s_at	regulating synapti	RIMS1	4.63	4.32	4.51
208199_s_at	zinc finger protein	ZFP161	5.94	5.85	6.04
203348_s_at	ets variant 5	ETV5	9.05	8.77	8.99
214645_at	---	---	3.74	3.58	3.77
207767_s_at	early growth resp	EGR4	6.34	5.99	5.60
217073_x_at	apolipoprotein A-I	APOA1	4.78	4.83	4.38
201491_at	AHA1, activator o	AHSA1	10.63	10.54	10.67
AFFX-r2-Ec-bioB	---	---	9.61	9.44	9.47
222061_at	CD58 molecule	CD58	5.67	5.55	5.69

206529_x_at	solute carrier fam	SLC26A4	3.57	3.65	3.58
208798_x_at	golgin A8 family, r	GOLGA8A	7.21	7.48	7.30
218577_at	leucine rich repea	LRRC40	10.40	10.67	10.70
40687_at	gap junction prote	GJA4	3.96	4.09	3.83
208687_x_at	heat shock 70kDa	HSPA8	12.55	12.76	12.59
220711_at	---	---	4.72	4.85	5.11
201854_s_at	ATM interactor	ATMIN	8.97	8.95	9.02
212218_s_at	fatty acid synthas	FASN	8.82	8.94	8.80
220205_at	transmembrane p	TPTE	3.28	3.31	3.32
208680_at	peroxiredoxin 1	PRDX1	13.51	13.55	13.49
201763_s_at	death-domain ass	DAXX	8.86	8.92	9.02
209597_s_at	paraneoplastic an	PNMA2	5.48	5.05	4.77
212508_at	modulator of apo	MOAP1	9.44	9.34	9.53
216050_at	---	---	3.47	3.63	3.36
201200_at	cellular repressor	CREG1	10.74	10.71	10.79
208682_s_at	melanoma antige	MAGED2	8.20	8.25	8.24
210598_at	---	---	5.52	5.84	5.55
217088_s_at	natural cytotoxici	NCR1	5.38	5.13	4.79
213684_s_at	PDZ and LIM dom	PDLIM5	6.32	6.52	6.31
214287_s_at	Cyclin-dependent	CDK13	4.74	4.51	4.35
203984_s_at	caspase 9, apopto	CASP9	8.23	8.34	8.29
211513_s_at	opioid growth fac	OGFR	6.98	6.67	6.82
212677_s_at	centrosomal prot	CEP68	8.87	8.82	8.95
206575_at	cyclin-dependent	CDKL5	5.90	5.74	5.54
218413_s_at	zinc finger protein	ZNF639	6.51	6.35	6.36
217210_at	---	---	4.70	5.01	4.73
220992_s_at	chromosome 1 op	C1orf25	8.50	8.47	8.47
218107_at	WD repeat domai	WDR26	10.69	10.60	10.68
212943_at	KIAA0528	KIAA0528	9.75	9.82	9.96
210657_s_at	septin 4	4-Sep	5.48	5.22	5.08
219078_at	G patch domain c	GPATCH2	7.23	7.10	7.05
215930_s_at	CTAGE family, me	CTAGE5	6.85	6.76	6.93
212842_x_at	RANBP2-like and	RGPD4 /// RGPD5	8.96	9.13	9.02
208524_at	G protein-couplec	GPR15	5.63	5.54	5.35
202950_at	crystallin, zeta (qu	CRYZ	10.08	10.29	10.22
218928_s_at	solute carrier fam	SLC37A1	5.85	6.06	5.85
210558_at	aldo-keto reducta	AKR1C4	3.38	3.63	3.50
207941_s_at	RNA binding moti	RBM39	11.80	11.83	11.91
220334_at	regulator of G-pro	RGS17	6.78	7.31	7.05
214281_s_at	ring finger and CH	RCHY1	9.93	10.00	10.12
220769_s_at	WD repeat domai	WDR78	3.30	3.78	3.69
210542_s_at	solute carrier orga	SLCO3A1	6.35	6.50	6.29
222267_at	transmembrane p	TMEM209	6.63	6.37	6.24
202524_s_at	sparc/osteonectin	SPOCK2	5.21	5.01	4.95
205915_x_at	glutamate recept	GRIN1	5.90	6.12	6.02
217042_at	retinol dehydroge	RDH11	5.40	5.78	5.09
221928_at	acetyl-CoA carbox	ACACB	6.06	5.99	6.17

48659_at	migration and inva	MIIP	7.80	7.90	7.81
209690_s_at	docking protein 4	DOK4	5.95	5.99	5.72
209596_at	matrix-remodellin	MXRA5	3.97	3.75	3.72
207352_s_at	gamma-aminobut	GABRB2	5.90	5.82	5.76
220957_at	cutaneous T-cell ly	CTAGE1	3.80	3.68	3.78
200836_s_at	microtubule-asso	MAP4	8.85	8.83	8.79
221683_s_at	centrosomal prote	CEP290	7.66	7.92	7.63
205939_at	cytochrome P450,	CYP3A7	4.17	4.20	4.03
204136_at	collagen, type VII,	COL7A1	8.22	8.40	8.35
212395_s_at	KIAA0090	KIAA0090	7.75	7.61	7.69
218045_x_at	parathymosin	PTMS	6.94	6.91	6.79
203345_s_at	metal response el	MTF2	8.76	9.07	8.81
204000_at	guanine nucleotid	GNB5	8.28	8.17	8.34
202397_at	nuclear transport	NUTF2	12.03	12.01	12.00
215384_s_at	microtubule-asso	MAP1A	5.26	5.18	4.78
201915_at	SEC63 homolog (S	SEC63	7.62	7.59	7.55
216786_at	non-protein codin	NCRNA00230A	3.97	4.09	3.68
211741_x_at	pregnancy specific	PSG3	3.84	3.94	3.68
216364_s_at	AF4/FMR2 family,	AFF2	4.54	4.44	4.55
202179_at	bleomycin hydrola	BLMH	8.09	8.03	8.23
220573_at	kallikrein-related	KLK14	6.74	6.51	6.41
207576_x_at	oxytocin, preprop	OXT	6.29	6.18	5.46
210535_at	B9 protein domai	B9D1	6.25	6.15	6.15
200617_at	malectin	MLEC	8.90	8.73	8.69
206829_x_at	zinc finger protein	ZNF430	6.20	6.70	6.00
216006_at	---	---	4.90	5.14	5.06
220147_s_at	family with seque	FAM60A	11.12	10.85	11.17
205299_s_at	butyrophilin, subf	BTN2A2	5.22	5.52	5.24
203783_x_at	polymerase (RNA)	POLRMT	6.41	6.44	6.17
212743_at	ring finger and CH	RCHY1	6.45	6.26	6.49
219109_at	sperm associated	SPAG16	8.38	8.57	8.70
211599_x_at	met proto-oncoge	MET	7.43	7.25	6.95
220601_at	chromosome 16 c	C16orf70	4.09	4.42	4.03
208238_x_at	---	---	8.65	8.78	8.62
215380_s_at	gamma-glutamylc	GGCT	11.61	11.60	11.78
211011_at	collagen, type XIX	COL19A1	4.78	4.70	4.78
201119_s_at	cytochrome c oxic	COX8A	12.95	12.91	12.89
209582_s_at	CD200 molecule	CD200	3.94	3.93	3.91
210442_at	interleukin 1 rece	IL1RL1	4.81	4.81	4.48
217193_x_at	---	---	5.96	6.02	6.02
214451_at	transcription facto	TFAP2B	4.40	4.40	4.34
219601_s_at	chromosome 10 c	C10orf12	4.68	4.30	4.40
214413_at	Tyrosine aminotra	TAT	4.75	4.84	4.41
215675_at	hypothetical LOC1	LOC100129648	5.52	5.29	5.47
217280_x_at	gamma-aminobut	GABRA5	3.86	3.72	3.65
202782_s_at	inositol polyphosp	INPP5K	7.53	7.54	7.57
211347_at	CDC14 cell divisio	CDC14B	5.07	5.41	5.41

222142_at	cylindromatosis (t	CYLD	5.11	4.88	5.07
206615_s_at	ADAM metallopep	ADAM22	3.34	3.37	3.42
201857_at	zinc finger RNA bi	ZFR	11.54	11.58	11.61
219005_at	transmembrane p	TMEM59L	7.38	7.48	7.25
210017_at	mucosa associate	MALT1	7.19	7.47	7.40
210108_at	calcium channel, v	CACNA1D	3.82	3.75	3.60
217327_at	transcription elon	LOC100287483	4.32	4.40	4.28
216546_s_at	chitinase 3-like 1 (	CHI3L1	4.05	4.09	4.05
214675_at	nucleoporin 188kl	NUP188	5.64	5.49	5.42
220622_at	leucine rich repea	LRRC31	3.31	3.28	3.08
56821_at	solute carrier fam	SLC38A7	6.86	6.87	6.95
AFFX-r2-Ec-bioB	---	---	9.58	9.49	9.63
201128_s_at	ATP citrate lyase	ACLY	12.15	12.27	12.38
219795_at	solute carrier fam	SLC6A14	3.36	3.56	3.21
221467_at	melanocortin 4 re	MC4R	3.90	3.64	3.91
208048_at	tachykinin recepto	TACR1	4.00	3.86	3.69
206207_at	Charcot-Leyden cr	CLC	4.45	4.30	4.04
215254_at	regulator of calcin	RCAN1	4.76	4.83	4.50
215718_s_at	PHD finger protei	PHF3	7.78	7.85	7.85
216864_at	---	---	4.04	4.20	4.06
204042_at	WAS protein fami	WASF3	4.14	4.42	4.25
200741_s_at	ribosomal protein	RPS27	14.82	14.83	14.83
214286_at	guanine nucleotid	GNAT1	5.29	5.48	5.57
209274_s_at	iron-sulfur cluster	ISCA1	10.99	11.07	11.17
218606_at	zinc finger, DHHC-	ZDHHC7	10.23	10.36	10.16
218649_x_at	serologically defin	SDCCAG1	8.86	9.15	9.31
214571_at	fibroblast growth	FGF3	5.51	5.28	5.25
215146_s_at	tetratricopeptide	TTC28	7.89	7.69	7.83
206935_at	protocadherin 8	PCDH8	3.14	3.09	2.99
221013_s_at	apolipoprotein L,	APOL2	8.05	7.81	7.81
221071_at	---	---	7.59	7.55	7.36
211799_x_at	major histocompa	HLA-C	9.91	9.72	9.65
207949_s_at	islet cell autoantig	ICA1	6.93	6.62	6.49
220642_x_at	G protein-couplec	GPR89A /// GPR89	9.34	9.42	9.54
204845_s_at	glutamyl aminope	ENPEP	3.37	3.64	3.17
204412_s_at	neurofilament, he	NEFH	3.80	4.01	3.85
216614_at	---	---	4.06	4.18	3.93
216778_s_at	cylicin, basic prote	CYLC1	3.33	3.49	3.41
210533_at	mutS homolog 4 (	MSH4	3.50	3.80	3.73
218605_at	transcription facto	TFB2M	9.88	9.69	9.93
212650_at	EH domain bindin	EHBP1	7.42	7.56	7.52
204682_at	latent transformir	LTBP2	6.96	6.76	6.56
216365_x_at	immunoglobulin l	IGLV3-19	4.07	3.84	4.10
201839_s_at	epithelial cell adh	EPCAM	4.21	3.93	4.08
201408_at	protein phosphata	PPP1CB	9.32	9.38	9.40
212691_at	nucleoporin 188kl	NUP188	8.99	8.93	8.70
206787_at	bromodomain, tes	BRDT	3.58	3.81	3.51

203198_at	cyclin-dependent	CDK9	7.93	8.08	8.06
204054_at	phosphatase and	PTEN	7.48	7.55	7.62
216701_at	chromosome 1 op	C1orf68	6.06	5.70	5.90
216585_at	---	---	4.99	5.03	5.13
207415_at	phospholipase A2	PLA2R1	3.99	3.95	3.58
219923_at	tripartite motif-co	TRIM45	5.85	5.94	5.92
206987_x_at	fibroblast growth	FGF18	4.63	4.81	4.83
217403_s_at	zinc finger protein	ZNF227	6.73	6.79	6.55
204666_s_at	suppressor of IKBI	SIKE1	4.69	4.67	4.54
213741_s_at	karyopherin alpha	KPNA1	7.42	7.60	7.61
221900_at	collagen, type VIII	COL8A2	3.92	4.00	3.78
212427_at	KIAA0368	KIAA0368	7.26	7.24	7.60
217541_x_at	zinc finger protein	ZNF816A	5.10	5.05	5.33
212328_at	LIM and calponin	LIMCH1	10.57	10.54	10.71
200077_s_at	ornithine decarbo	OAZ1	13.92	13.94	13.94
211695_x_at	mucin 1, cell surfa	MUC1	5.38	5.10	5.18
209613_s_at	alcohol dehydroge	ADH1B	3.25	3.17	3.28
210963_s_at	glycogenin 2	GYG2	5.16	5.12	5.04
218654_s_at	mitochondrial ribo	MRPS33	11.41	11.29	11.38
200790_at	ornithine decarbo	ODC1	13.34	13.30	13.37
221273_s_at	ring finger protein	RNF208	6.04	5.82	5.53
216865_at	collagen, type XIV	COL14A1	4.01	4.12	3.77
204815_s_at	DEAH (Asp-Glu-Al	DHX34	5.40	5.69	5.70
217240_at	signal-regulatory p	SIRPB1	3.76	3.94	3.86
219371_s_at	Kruppel-like facto	KLF2	6.83	6.79	6.81
203818_s_at	splicing factor 3a,	SF3A3	9.55	9.48	9.51
212221_x_at	iduronate 2-sulfat	IDS	9.99	9.99	10.03
206581_at	basonuclin 1	BNC1	6.50	6.30	6.48
204681_s_at	Rap guanine nucle	RAPGEF5	3.60	3.81	3.63
200811_at	cold inducible RN/	CIRBP	9.62	9.52	9.75
217163_at	estrogen receptor	ESR1	4.64	4.93	4.86
222109_at	guanine nucleotid	GNL3L	7.67	7.40	7.87
206189_at	unc-5 homolog C (	UNC5C	4.83	4.54	4.37
206642_at	desmoglein 1	DSG1	3.65	3.51	3.36
220292_at	zinc finger protein	ZNF434	6.65	6.68	6.42
209696_at	fructose-1,6-bisph	FBP1	7.24	6.69	6.76
53987_at	RAN binding prote	RANBP10	6.82	6.99	7.08
206338_at	ELAV (embryonic	ELAVL3	7.31	6.83	7.16
208628_s_at	Y box binding prot	YBX1	13.78	13.77	13.85
202152_x_at	upstream transcri	USF2	8.27	8.29	7.96
208856_x_at	ribosomal protein	RPLP0	15.04	15.02	15.03
202655_at	mesencephalic as	MANF	11.42	11.51	11.42
213611_at	aquaporin 5	AQP5	5.95	5.66	5.59
210944_s_at	calpain 3, (p94)	CAPN3	6.44	6.54	6.66
209954_x_at	synovial sarcoma	SS18	7.05	7.03	6.78
216112_at	---	---	5.61	5.85	5.75
215366_at	sorting nexin 13	SNX13	4.34	4.22	4.71

216521_s_at	BRCA1/BRCA2-coı	BRCC3	7.13	7.20	7.10
203237_s_at	Notch homolog 3	NOTCH3	5.82	5.76	5.56
220446_s_at	carbohydrate (N-a	CHST4	5.33	5.24	5.07
214119_s_at	FK506 binding prc	FKBP1A	8.99	8.98	8.99
221756_at	phosphoinositide-	PIK3IP1	7.91	7.72	7.49
200684_s_at	ubiquitin-conjuga	UBE2L3	8.72	8.84	8.84
213166_x_at	family with seque	FAM128A	11.57	11.51	11.60
200002_at	ribosomal protein	RPL35	14.43	14.39	14.38
211764_s_at	ubiquitin-conjuga	UBE2D1	8.95	9.25	9.38
222195_s_at	chromosome 9 op	C9orf156	7.88	7.82	7.87
207680_x_at	paired box 3	PAX3	5.98	6.10	5.86
209986_at	achaete-scute cor	ASCL1	5.78	5.60	5.30
202405_at	TIA1 cytotoxic gra	TIAL1	8.02	8.04	7.99
213715_s_at	KN motif and ank	KANK3	5.05	5.52	4.93
209738_x_at	pregnancy specifi	PSG6	5.05	5.51	5.16
216423_at	---	---	6.75	6.63	6.51
213186_at	DAZ interacting pı	DZIP3	8.21	8.41	8.63
201772_at	antizyme inhibitoı	AZIN1	10.37	10.48	10.64
213825_at	oligodendrocyte li	OLIG2	4.24	4.19	4.01
215890_at	GM2 ganglioside a	GM2A	5.49	5.34	5.11
213213_at	death inducer-obl	DIDO1	7.41	7.66	7.17
215785_s_at	cytoplasmic FMR1	CYFIP2	7.58	7.57	7.63
222013_x_at	family with seque	FAM86A	8.62	8.38	8.52
208572_at	histone cluster 3,	HIST3H3	6.51	6.20	6.23
AFFX-ThrX-M_at	---	---	7.31	7.26	7.34
215198_s_at	caldesmon 1	CALD1	7.53	7.41	7.21
201869_s_at	transducin (beta)-	TBL1X	8.71	8.69	8.86
220727_at	potassium channe	KCNK10	6.86	6.54	6.57
209070_s_at	regulator of G-prc	RGS5	5.73	5.78	5.51
221131_at	alpha-1,4-N-acety	A4GNT	6.09	6.04	6.03
201512_s_at	translocase of out	TOMM70A	10.06	10.09	10.22
217823_s_at	ubiquitin-conjuga	UBE2J1	9.00	9.00	9.19
217699_at	---	---	3.86	3.77	4.16
204981_at	solute carrier fam	SLC22A18	8.26	8.24	8.25
208386_x_at	DMC1 dosage sup	DMC1	4.17	4.40	4.20
203749_s_at	retinoic acid rece	RARA	7.09	7.03	7.11
206294_at	hydroxy-delta-5-s	HSD3B2	4.01	3.53	3.55
215511_at	transcription facto	TCF20	4.45	4.55	4.19
AFFX-DapX-3_at	---	---	11.31	11.23	11.25
210020_x_at	calmodulin-like 3	CALML3	4.59	4.53	4.74
205269_at	lymphocyte cytos	LCP2	3.01	3.05	3.11
218630_at	Meckel syndrome	MKS1	7.15	7.17	7.02
207058_s_at	Parkinson disease	PARK2	3.43	3.47	3.81
207413_s_at	sodium channel, v	SCN5A	5.68	5.80	5.64
203160_s_at	ring finger proteir	RNF8	8.77	9.00	8.80
208138_at	gastrin	GAST	6.34	6.36	6.17
200047_s_at	YY1 transcription	YY1	12.34	12.35	12.49

219487_at	Bardet-Biedl synd	BBS10	7.84	7.91	8.22
213647_at	DNA replication h	DNA2	7.51	7.79	7.79
219201_s_at	twisted gastrulati	TWSG1	7.03	7.07	7.10
202393_s_at	Kruppel-like facto	KLF10	10.51	10.25	10.60
209403_at	TBC1 domain fam	TBC1D3 /// TBC1D	7.74	7.92	7.63
216515_x_at	prothymosin, alph	PTMA	12.59	12.72	12.84
218771_at	pantothenate kin	PANK4	8.53	8.35	8.11
219023_at	adaptor-related p	AP1AR	8.58	8.54	9.00
204194_at	BTB and CNC hom	BACH1	7.47	7.85	7.69
216085_at	DKFZP434C153 pr	DKFZP434C153	4.24	4.03	4.12
220175_s_at	COBW domain co	CBWD1 /// CBWD	8.24	8.27	8.65
207015_s_at	aldehyde dehydro	ALDH1A2	6.38	6.24	6.29
208247_at	chromosome 3 op	C3orf51	3.95	3.95	3.88
206509_at	prolactin-induced	PIP	6.56	6.27	6.19
217261_at	testis-specific tra	TTTY2 /// TTTY2B	4.73	4.52	4.50
214357_at	chromosome 1 op	C1orf105	3.98	4.05	3.91
217658_at	THAP domain con	THAP3	6.35	6.04	6.10
214488_at	RAP2B, member c	RAP2B	4.01	3.84	3.81
211172_x_at	A kinase (PRKA) a	AKAP7	7.54	7.49	7.31
202550_s_at	VAMP (vesicle-ass	VAPB	9.69	9.65	9.84
219988_s_at	ring finger protei	RNF220	8.79	8.73	8.80
203369_x_at	PDZ and LIM dom	PDLIM7	6.15	5.85	6.02
216836_s_at	v-erb-b2 erythrob	ERBB2	8.51	8.54	8.45
205986_at	apoptosis-associa	AATK	7.56	7.60	7.39
209923_s_at	BRCA1 associated	BRAP	8.45	8.23	8.21
200885_at	ras homolog gene	RHOC	11.19	11.21	11.13
214708_at	syntrophin, beta 1	SNTB1	4.57	4.64	4.63
221457_s_at	butyrophilin-like 2	BTNL2	6.09	5.82	5.92
220290_at	absent in melano	AIM1L	6.59	6.68	6.39
215570_s_at	zinc finger protei	ZNF780A /// ZNF7	3.45	3.32	3.44
216611_s_at	solute carrier fam	SLC6A2	6.53	6.23	6.22
216591_s_at	succinate dehydro	SDHC	9.02	9.20	9.11
215387_x_at	---	---	6.66	6.65	6.55
215921_at	nuclear pore com	NPIPL3	4.49	4.35	4.58
213855_s_at	lipase, hormone-s	LIPE	4.91	5.02	5.07
206657_s_at	myogenic differen	MYOD1	7.00	6.97	7.05
200941_at	heat shock factor	HSBP1	10.93	10.94	10.92
202171_at	vascular endothel	VEZF1	10.69	10.52	10.76
211169_s_at	protein phosphat	PPP1R3A	3.33	3.41	3.09
220080_at	F-box and leucine	FBXL8	5.90	5.61	5.63
203302_at	deoxycytidine kin	DCK	9.14	9.14	9.27
202343_x_at	cytochrome c oxi	COX5B	12.03	11.99	12.07
208102_s_at	pleckstrin and Sec	PSD	6.56	6.08	6.13
44696_at	TBC1 domain fam	TBC1D13	7.57	7.68	7.53
200658_s_at	prohibitin	PHB	11.32	11.28	11.41
217091_at	---	---	3.27	3.24	3.31
49051_g_at	deltex homolog 3	DTX3	4.37	4.09	4.39

202441_at	ER lipid raft associ	ERLIN1		11.48	11.38	11.48
203862_s_at	actinin, alpha 2	ACTN2		3.86	3.74	3.71
203346_s_at	metal response el	MTF2		7.15	7.44	7.23
213386_at	chromosome 9 op	C9orf125		4.32	4.12	4.47
208088_s_at	complement facto	CFHR5		3.20	3.27	3.11
201221_s_at	small nuclear ribo	SNRNP70		9.85	9.79	9.70
209530_at	calcium channel, v	CACNB3		7.29	7.23	7.32
201303_at	eukaryotic transla	EIF4A3		10.42	10.67	10.49
214509_at	histone cluster 1,	HIST1H3I		3.77	3.82	3.73
201061_s_at	stomatin	STOM		4.82	4.81	4.63
213565_s_at	SMAD family men	SMAD6		6.09	5.89	6.07
219866_at	chloride intracellu	CLIC5		5.55	5.53	5.40
213160_at	dedicator of cytok	DOCK2		4.30	3.87	3.66
213616_at	chromosome 18 o	C18orf10		8.44	8.36	8.48
207399_at	beaded filament s	BFSP2		5.85	5.82	5.75
200838_at	cathepsin B	CTSB		9.69	9.67	9.74
215750_at	KIAA1659 protein	KIAA1659		4.56	4.95	4.64
221501_x_at	hypothetical prote	LOC339047		10.48	10.38	10.33
206878_at	D-amino-acid oxic	DAO		4.91	4.90	4.40
210240_s_at	cyclin-dependent	CDKN2D		6.55	6.44	6.45
204193_at	choline kinase bet	CHKB		8.50	8.58	8.35
204281_at	TEA domain famil	TEAD4		9.32	9.38	9.26
201410_at	pleckstrin homolo	PLEKHB2		10.72	10.75	10.84
219094_at	armadillo repeat c	ARMC8		7.01	6.98	7.18
215651_at	---	---		6.04	5.70	5.65
212184_s_at	TGF-beta activate	TAB2		9.73	9.56	9.83
214012_at	endoplasmic retic	ERAP1		4.95	5.35	5.36
217997_at	pleckstrin homolo	PHLDA1		9.84	9.79	10.12
210913_at	cadherin 20, type	CDH20		4.60	4.83	4.41
221726_at	ribosomal protein	RPL22		12.41	12.40	12.41
206755_at	cytochrome P450,	CYP2B6		4.39	4.45	3.87
215848_at	S-phase cyclin A-a	SCAPER		5.80	6.19	5.80
215576_at	---	---		5.30	5.30	5.21
221183_at	---	---		4.53	4.26	4.47
212415_at	septin 6		6-Sep	4.18	4.37	4.39
202390_s_at	huntingtin	HTT		7.40	7.57	7.43
221162_at	HERV-H LTR-assoc	HHLA1		7.41	7.56	7.25
221444_at	taste receptor, typ	TAS2R16		3.63	3.73	3.45
218098_at	ADP-ribosylation f	ARFGEF2		9.93	9.89	10.02
216395_at	F-box and leucine-	FBXL18		4.59	4.93	4.88
217184_s_at	leukocyte recepto	LTK		5.13	5.13	4.90
216949_s_at	polycystic kidney	PKD1		6.77	6.66	6.23
211798_x_at	immunoglobulin la	IGLJ3		4.90	4.76	4.92
214054_at	docking protein 2,	DOK2		6.50	6.41	6.32
214361_s_at	regulator of G-pro	RGS12		6.34	6.26	5.54
204611_s_at	protein phosphata	PPP2R5B		8.31	8.17	8.19
221033_s_at	ring finger protein	RNF17		3.91	3.77	3.73

208715_at	transmembrane a	TMCO1	9.07	9.08	9.03
213722_at	SRY (sex determin	SOX2	5.72	5.92	5.72
206031_s_at	ubiquitin specific	USP5	7.39	7.21	7.25
201102_s_at	phosphofructokin	PFKL	7.58	7.38	7.52
201151_s_at	muscleblind-like (	MBNL1	9.78	9.80	10.17
209352_s_at	SIN3 homolog B, t	SIN3B	6.55	6.45	6.21
217370_x_at	fused in sarcoma	FUS	9.09	9.07	9.05
204630_s_at	golgi SNAP recept	GOSR1	9.60	9.59	9.55
209273_s_at	iron-sulfur cluster	ISCA1	9.40	9.49	9.53
220795_s_at	brain-enriched gu	BEGAIN	6.44	6.41	6.58
210782_x_at	glutamate recepto	GRIN1	8.21	8.09	8.07
203446_s_at	oculocerebrorena	OCRL	8.22	8.23	8.45
208123_at	potassium voltage	KCNB2	3.69	3.62	3.56
208099_x_at	tubulin tyrosine li	TTLL5	5.46	5.64	5.51
205049_s_at	CD79a molecule, i	CD79A	6.46	6.05	6.08
214411_x_at	chymotrypsinoger	CTRB2	6.04	6.11	5.61
213952_s_at	Arachidonate 5-li	ALOX5	4.36	4.26	4.31
213287_s_at	keratin 10	KRT10	10.54	10.56	10.84
221623_at	brevican	BCAN	4.29	4.34	4.24
221892_at	hexose-6-phospha	H6PD	6.99	6.85	6.40
215045_at	CUGBP, Elav-like f	CELF3	7.63	7.19	7.26
210967_x_at	calcium channel, v	CACNB1	6.15	6.00	5.60
217506_at	Hypothetical LOC3	LOC339290	6.47	6.71	6.65
220977_x_at	erythrocyte meml	EPB41L5	5.97	6.08	6.07
215237_at	dedicator of cytok	DOCK9	4.89	4.99	4.72
47550_at	leucine zipper, pu	LZTS1	4.45	4.43	3.99
220083_x_at	ubiquitin carboxyl	UCHL5	7.91	8.14	8.31
206111_at	ribonuclease, RNa	RNASE2	3.93	3.79	3.64
217587_at	---	---	4.49	4.47	4.53
210382_at	secretin receptor	SCTR	5.88	5.46	5.70
210929_s_at	alpha-2-HS-glycop	AHSG /// LOC1001	4.13	4.21	3.89
209942_x_at	melanoma antiger	MAGEA3	11.89	11.94	11.87
217522_at	potassium channe	KCNV2	3.46	3.68	3.53
216562_at	---	---	6.65	6.61	6.47
204824_at	endonuclease G	ENDOG	8.70	8.60	8.71
201171_at	ATPase, H+ transp	ATP6V0E1	6.40	6.34	6.39
221504_s_at	ATPase, H+ transp	ATP6V1H	9.71	9.81	9.89
218514_at	chromosome 17 o	C17orf71	8.91	8.98	9.05
219950_s_at	T-cell lymphoma i	TIAM2	5.13	4.92	4.77
219617_at	chromosome 2 op	C2orf34	7.26	7.46	7.15
216754_at	Similar to Kruppel	LOC100133321	5.90	5.97	5.80
210397_at	defensin, beta 1	DEFB1	6.10	5.91	5.75
211875_x_at	protocadherin gar	PCDHGA10	5.01	5.34	5.09
201965_s_at	senataxin	SETX	7.88	7.91	7.88
214100_x_at	NOP2/Sun domair	NSUN5P1	9.99	9.89	9.89
216799_at	---	---	5.23	5.12	5.09
218389_s_at	anterior pharynx o	APH1A	9.67	9.69	9.79

211786_at	tumor necrosis fac	TNFRSF9	6.23	6.04	6.29
201281_at	adhesion regulatin	ADRM1	9.01	8.98	9.00
221969_at	paired box 5	PAX5	4.73	4.59	4.46
203990_s_at	lysine (K)-specific	KDM6A	4.35	4.61	4.42
222240_s_at	inositol-3-phospha	ISYNA1	5.18	4.97	4.95
215940_at	---	---	3.90	3.79	3.68
219009_at	chromosome 14 o	C14orf93	6.76	6.68	6.76
202808_at	target of myb1 (ch	TOM1	8.40	8.42	8.36
220510_at	Rh family, B glyco	RHBG	8.83	8.80	8.48
204691_x_at	phospholipase A2	PLA2G6	7.87	7.91	7.70
218614_at	chromosome 12 o	C12orf35	8.91	9.01	8.95
218518_at	family with seque	FAM13B	8.15	8.34	8.44
206384_at	calcium channel, v	CACNG3	4.37	4.31	4.35
202298_at	NADH dehydroger	NDUFA1	12.06	12.05	12.16
219309_at	chromosome 22 o	C22orf46	5.72	6.06	5.65
217917_s_at	dynein, light chair	DYNLRB1	10.48	10.59	10.52
208483_x_at	keratin 33A	KRT33A	5.91	5.87	6.20
205714_s_at	zinc finger, MYND	ZMYND10	6.34	6.03	6.17
216015_s_at	NLR family, pyrin	NLRP3	3.95	3.90	3.76
200024_at	ribosomal protein	RPS5	14.64	14.60	14.64
209750_at	nuclear receptor s	NR1D2	8.83	8.72	8.99
208613_s_at	filamin B, beta	FLNB	8.56	8.46	8.48
219418_at	nonhomologous e	NHEJ1	7.48	7.48	7.54
205738_s_at	fatty acid binding	FABP3	5.29	5.34	4.92
205773_at	cytoplasmic polya	CPEB3	5.04	4.85	5.36
205443_at	small nuclear RNA	SNAPC1	11.49	11.32	11.60
208181_at	histone cluster 1,	HIST1H4H	3.38	3.44	3.55
204505_s_at	erythrocyte meml	EPB49	6.47	6.44	6.50
220918_at	chromosome 21 o	C21orf96	6.42	6.69	6.36
219759_at	endoplasmic retic	ERAP2	8.74	8.81	8.85
212282_at	transmembrane p	TMEM97	11.27	11.33	11.22
217563_at	clock homolog (m	CLOCK	5.16	5.20	5.00
216176_at	hepatocellular car	HCRP1	7.41	7.63	7.30
212179_at	splicing factor, arg	SFRS18	8.47	8.83	8.71
216263_s_at	neuroguidin, EIF4I	NGDN	5.24	5.04	4.99
210798_x_at	mannan-binding le	MASP2	4.91	5.00	4.85
212443_at	neurobeachin-like	NBEAL2	7.21	7.15	6.96
216151_at	---	---	3.04	3.05	3.28
211628_x_at	ferritin, heavy pol	FTHL5	14.63	14.62	14.67
219450_at	chromosome 4 op	C4orf19	4.04	3.68	4.20
203072_at	myosin IE	MYO1E	7.78	7.73	7.98
211266_s_at	G protein-couplec	GPR4	5.48	5.72	5.35
214167_s_at	ribosomal protein	RPLP0 /// RPLP0P6	14.45	14.43	14.45
215629_s_at	deleted in lympho	DLEU2 /// DLEU2L	7.47	7.43	7.61
204939_s_at	phospholamban	PLN	6.02	5.56	5.73
222116_s_at	TBC1 domain fam	TBC1D16	9.13	8.99	9.03
205440_s_at	neuropeptide Y re	NPY1R	3.65	3.92	3.91

202831_at	solute carrier fam	SLC37A4	5.71	5.71	5.53
209452_s_at	vesicle transport t	VTI1B	9.98	10.00	9.99
207945_s_at	casein kinase 1, d	CSNK1D	8.29	8.59	8.65
217401_at	---	---	3.41	3.63	3.35
218323_at	ras homolog gene	RHOT1	10.14	10.24	10.23
221167_s_at	coiled-coil domair	CCDC70	5.20	5.20	5.19
212437_at	centromere prote	CENPB	7.56	7.39	7.28
213532_at	ADAM metallope	ADAM17	9.32	9.13	9.32
207863_at	ADP-ribosylarginir	ADPRH	5.56	5.54	5.38
203605_at	signal recognition	SRP54	9.69	9.71	9.85
214642_x_at	melanoma antiger	MAGEA5	8.39	8.33	8.64
200629_at	tryptophanyl-tRN,	WARS	10.81	10.54	10.72
217900_at	isoleucyl-tRNA syr	IARS2	11.37	11.37	11.50
200678_x_at	granulin	GRN	9.46	9.30	9.32
216260_at	dicer 1, ribonucle	DICER1	3.61	4.02	3.86
206142_at	zinc finger proteir	ZNF135	3.43	3.08	3.22
203787_at	single-stranded D	SSBP2	10.41	10.15	10.53
218220_at	chromosome 12 c	C12orf10	8.84	8.90	8.85
201277_s_at	heterogeneous nu	HNRNPAB	12.99	12.93	13.02
200731_s_at	protein tyrosine p	PTP4A1	9.38	9.31	9.56
220341_s_at	chromosome 5 op	C5orf45	7.54	7.45	7.61
209558_s_at	huntingtin interac	HIP1R /// LOC100	8.66	8.71	8.77
216643_at	---	---	4.12	4.08	3.85
205678_at	adaptor-related p	AP3B2	4.58	4.42	4.38
203808_at	---	---	4.69	4.48	4.73
213520_at	RecQ protein-like	RECQL4	8.45	8.39	8.18
205636_at	SH3-domain GRB2	SH3GL3	5.53	5.74	5.44
200054_at	zinc finger proteir	ZNF259	9.37	9.31	9.42
207766_at	cyclin-dependent	CDKL1	4.83	5.06	4.47
206408_at	leucine rich repea	LRRTM2	3.81	4.05	3.84
221404_at	interleukin 1 fami	IL1F6	5.59	5.72	5.65
221478_at	BCL2/adenovirus	BNIP3L	10.74	10.80	10.97
216637_at	---	---	5.44	6.00	5.25
208793_x_at	SWI/SNF related,	SMARCA4	7.63	7.51	7.63
219171_s_at	zinc finger proteir	ZNF236	5.57	5.38	5.41
205132_at	actin, alpha, cardi	ACTC1	6.05	5.89	6.15
219353_at	NHL repeat contai	NHLRC2	7.93	7.93	7.93
216164_at	leucine rich repea	LRRN2	6.82	6.73	6.53
217744_s_at	PERP, TP53 apopt	PERP	8.77	8.86	9.04
203576_at	branched chain ar	BCAT2	8.08	8.05	8.40
214185_at	KH domain contai	KHDRBS1	5.39	5.47	5.19
217496_s_at	insulin-degrading	IDE	8.28	8.29	8.28
208723_at	ubiquitin specific	USP11	9.85	9.70	9.71
203656_at	FIG4 homolog, SA	FIG4	8.66	8.78	8.73
214440_at	N-acetyltransfera	NAT1	6.76	7.34	7.01
219859_at	C-type lectin dom	CLEC4E	3.85	3.88	3.69
200939_s_at	arginine-glutamic	RERE	4.34	4.40	4.11

216687_x_at	UDP glucuronosyl	UGT2B15	4.05	4.18	3.85
219609_at	WD repeat domai	WDR25	7.22	6.86	7.22
211881_x_at	immunoglobulin la	IGLJ3	6.39	6.15	6.18
211821_x_at	glycophorin A (MN	GYPA	3.85	3.51	3.24
200642_at	superoxide dismu	SOD1	12.97	12.98	13.05
212342_at	Yip1 domain famil	YIPF6	7.69	7.58	7.77
219974_x_at	enoyl CoA hydrata	ECHDC1	9.24	9.28	9.43
213004_at	angiopoietin-like 2	ANGPTL2	4.73	4.66	4.70
207508_at	ATP synthase, H+	ATP5G3	12.59	12.73	12.69
209131_s_at	synaptosomal-ass	SNAP23	6.06	6.19	6.05
200955_at	inner membrane p	IMMT	11.61	11.58	11.56
210170_at	PDZ and LIM dom	PDLIM3	4.12	4.19	4.32
216845_x_at	myeloid/lymphoic	MLL2	7.38	7.60	7.37
206009_at	integrin, alpha 9	ITGA9	4.37	4.07	4.02
203986_at	starch binding dor	STBD1	6.28	5.96	6.23
211984_at	calmodulin 1 (pho	CALM1	9.65	9.99	9.93
211750_x_at	tubulin, alpha 1c	TUBA1C	14.47	14.51	14.44
201282_at	oxoglutarate (alph	OGDH	7.75	7.95	7.71
203098_at	chromodomain pr	CDYL	10.15	9.91	10.12
207780_at	cylicin, basic prote	CYLC2	3.28	3.68	3.46
220556_at	ATPase, Na+/K+ tr	ATP1B4	3.23	3.54	3.27
206681_x_at	glycoprotein 2 (zy	GP2	5.63	5.57	5.55
204273_at	endothelin recept	EDNRB	8.85	8.97	9.22
213252_at	SH3 and PX doma	SH3PXD2A	4.70	4.69	5.04
202190_at	cleavage stimulati	CSTF1	7.81	7.95	7.94
201586_s_at	splicing factor pro	SFPQ	12.39	12.45	12.39
216014_s_at	zinc finger, X-linke	ZXDA /// ZXDB	6.21	5.77	6.07
213563_s_at	Tubulin, gamma c	TUBGCP2	6.44	6.57	6.49
201493_s_at	pumilio homolog 2	PUM2	10.19	10.26	10.31
220088_at	complement com	C5AR1	3.34	3.33	3.38
216258_s_at	serpin peptidase i	SERPINB13	3.55	3.51	3.48
205426_s_at	huntingtin interac	HIP1	4.55	4.85	4.71
205134_s_at	nuclear fragile X n	NUFIP1	9.12	9.01	9.12
201673_s_at	glycogen synthase	GYS1	7.45	7.66	7.41
217011_at	gastrulation brain	GBX1	6.14	6.15	5.89
34846_at	calcium/calmodul	CAMK2B	7.79	7.73	7.64
212995_x_at	family with seque	FAM128B	11.46	11.42	11.44
212628_at	protein kinase N2	PKN2	9.92	10.00	10.07
218623_at	HMP19 protein	HMP19	4.26	3.91	3.97
208195_at	titin	TTN	4.27	4.36	4.10
218850_s_at	LIM domains cont	LIMD1	6.34	6.49	6.22
203106_s_at	vacuolar protein s	VPS41	4.68	4.63	4.98
209847_at	cadherin 17, LI cad	CDH17	4.48	4.43	4.19
213639_s_at	zinc finger protein	ZNF500	7.55	7.46	7.63
217724_at	SERPINE1 mRNA b	SERBP1	12.89	12.89	13.01
221997_s_at	Mitochondrial ribo	MRPL52	6.13	6.19	6.53
208404_x_at	potassium inward	KCNJ5	7.14	6.94	6.91

201269_s_at	NudC domain con	NUDCD3	4.96	5.10	4.78
206853_s_at	Mitogen-activatec	MAP3K7	8.67	8.80	8.86
214010_s_at	ATPase, class II, ty	ATP9B	7.09	7.36	7.20
218126_at	family with seque	FAM82A2	9.21	9.29	9.14
213821_s_at	Iduronate 2-sulfat	IDS	5.96	5.70	5.80
202727_s_at	interferon gamma	IFNGR1	11.52	11.53	11.62
221716_s_at	acyl-CoA syntheta	ACSBG2	3.89	3.88	4.08
212858_at	progestin and adi	PAQR4	7.96	8.06	7.90
207932_at	interferon, alpha 8	IFNA8	3.67	3.62	3.46
211310_at	enhancer of zeste	EZH1	4.40	4.79	4.96
204338_s_at	regulator of G-prc	RGS4	4.31	4.03	4.52
211195_s_at	tumor protein p63	TP63	5.30	4.98	4.89
201701_s_at	progesterone rece	PGRMC2	9.06	9.17	9.41
205916_at	S100 calcium bind	S100A7	4.25	4.00	3.65
209719_x_at	serpin peptidase i	SERPINB3	4.88	4.80	4.61
218867_s_at	chromosome 12 c	C12orf49	7.53	7.68	7.68
203300_x_at	adaptor-related p	AP1S2	10.38	10.44	10.40
212102_s_at	karyopherin alpha	KPNA6	7.33	7.52	7.42
209911_x_at	histone cluster 1,	HIST1H2BD	7.97	8.10	8.13
213508_at	chromosome 14 c	C14orf147	8.77	8.75	8.84
204511_at	FERM, RhoGEF an	FARP2	5.96	5.89	5.54
206620_at	GRB2-related ada	GRAP	5.73	5.42	5.79
214241_at	NADH dehydroger	NDUFB8	6.66	6.86	6.52
212513_s_at	ubiquitin specific	USP33	10.03	10.22	10.21
208416_s_at	spectrin, beta, ery	SPTB	7.04	6.68	6.80
207760_s_at	nuclear receptor c	NCOR2	10.53	10.54	10.43
216938_x_at	dopamine recepto	DRD2	6.60	6.48	6.24
221512_at	transmembrane p	TMEM222	7.62	7.47	7.48
213147_at	homeobox A10	HOXA10	8.24	8.15	8.20
220849_at	hypothetical LOC7	LOC79999	6.49	6.44	6.19
206279_at	protein kinase, Y-l	PRKY	5.49	5.45	5.31
220838_at	exonuclease 3'-5'	EXD3	7.11	7.05	7.27
215709_at	primase, DNA, pol	PRIM2	5.96	5.62	5.78
211595_s_at	mitochondrial ribo	MRPS11	9.98	10.04	10.11
204283_at	phenylalanyl-tRNA	FARS2	8.55	8.70	8.82
206063_x_at	peptidylprolyl isor	PPIL2	8.07	8.04	7.93
211829_s_at	G protein-couplec	GPER	5.31	4.98	4.76
201833_at	histone deacetyla	HDAC2	11.54	11.50	11.54
201535_at	ubiquitin-like 3	UBL3	8.66	8.90	8.93
205355_at	acyl-CoA dehydro	ACADSB	5.65	5.62	5.68
213873_at	---	---	5.38	5.55	5.24
219502_at	nei endonuclease	NEIL3	8.88	8.87	9.05
212883_at	hypothetical LOC1	LOC100129500	3.77	3.72	3.64
216772_at	---	---	4.32	4.23	3.90
207254_at	solute carrier fam	SLC15A1	4.37	4.34	4.41
38710_at	OTU domain, ubic	OTUB1	8.91	9.06	8.97
206187_at	prostaglandin I2 (|	PTGIR	6.51	6.49	6.32

48031_r_at	chromosome 5 op	C5orf4	3.95	4.05	3.79
208928_at	P450 (cytochrome	POR	7.56	7.57	7.61
208839_s_at	cullin-associated a	CAND1	9.37	9.51	9.41
207062_at	islet amyloid poly	IAPP	4.41	4.38	4.40
205296_at	retinoblastoma-lik	RBL1	6.56	6.81	7.22
214698_at	ROD1 regulator of	ROD1	7.22	7.10	7.21
218907_s_at	leucine rich repea	LRRC61	6.86	6.73	6.41
205410_s_at	ATPase, Ca++ tran	ATP2B4	4.73	4.43	4.46
220835_s_at	zinc finger proteir	ZNF407	3.95	3.85	3.58
205977_s_at	EPH receptor A1	EPHA1	5.68	5.80	5.34
201818_at	lysophosphatidylc	LPCAT1	9.01	9.16	8.97
207856_s_at	sphingomyelin ph	LOC150776 /// SM	8.58	8.57	8.60
203014_x_at	small G protein sig	SGSM3	7.50	7.32	7.34
205242_at	chemokine (C-X-C	CXCL13	3.46	3.56	3.17
205960_at	pyruvate dehydro	PDK4	4.79	4.61	4.14
204583_x_at	kallikrein-related	KLK3	4.31	4.65	4.36
216096_s_at	neurexin 1	NRXN1	3.39	3.65	3.48
205486_at	testis-specific kina	TESK2	6.99	6.97	6.57
221333_at	forkhead box P3	FOXP3	5.40	5.26	5.21
217634_at	supervillin	SVIL	6.85	6.84	6.66
215418_at	parvin, alpha	PARVA	4.81	5.13	4.87
211330_s_at	hemochromatosis	HFE	5.39	5.24	5.06
208757_at	transmembrane e	TMED9	10.18	10.13	10.20
210738_s_at	solute carrier fam	SLC4A4	3.35	3.33	3.05
204239_s_at	neuronatin	NNAT	4.92	5.21	4.83
209127_s_at	squamous cell car	SART3	9.62	9.66	9.65
204374_s_at	galactokinase 1	GALK1	6.97	6.88	6.84
207445_s_at	chemokine (C-C m	CCR9	3.85	3.55	3.53
217786_at	protein arginine m	PRMT5	10.82	10.81	10.70
213626_at	carbonyl reductas	CBR4	9.11	9.11	9.25
210819_x_at	deiodinase, iodotl	DIO2	6.79	7.08	6.68
213638_at	phosphatase and	PHACTR1	4.80	4.78	4.38
52741_at	tRNA methyltrans	TRMT61A	8.18	7.96	8.20
216600_x_at	Aldolase B, fructo	ALDOB	6.78	6.92	6.90
217386_at	---	---	4.38	4.12	3.86
204005_s_at	PRKC, apoptosis, \	PAWR	8.97	8.83	9.14
210184_at	integrin, alpha X (	ITGAX	6.38	6.45	6.33
204129_at	B-cell CLL/lympho	BCL9	7.01	7.09	6.85
203671_at	thiopurine S-meth	TPMT	7.27	7.54	7.65
214877_at	CDK5 regulatory s	CDKAL1	6.90	6.87	7.04
217382_at	---	---	4.42	4.31	4.18
211739_x_at	chorionic somato	CSH1	4.31	3.82	3.82
221837_at	kelch-like 22 (Dro	KLHL22	4.96	4.97	4.42
210016_at	myelin transcripti	MYT1L	3.90	3.55	3.50
215147_at	---	---	5.03	4.81	4.87
217528_at	chloride channel a	CLCA2	3.14	2.93	3.22
216683_at	---	---	4.67	5.05	4.47

213459_at	ribosomal protein	RPL37A	8.37	8.28	8.31
212881_at	protein inhibitor c	PIAS4	6.92	6.75	7.04
218412_s_at	GTF2I repeat dom	GTF2IRD1	9.83	9.63	9.83
202268_s_at	NEDD8 activating	NAE1	11.75	11.81	11.76
220593_s_at	coiled-coil domair	CCDC40	7.19	7.13	6.63
213168_at	Sp3 transcription	SP3	10.54	10.58	10.73
205406_s_at	sperm autoantige	SPA17	8.57	8.77	8.71
211852_s_at	attractin	ATRN	6.85	6.88	7.07
208067_x_at	ubiquitously trans	UTY	6.08	6.13	5.71
220137_at	V-set and immunc	VSIG10	7.19	7.06	7.06
214967_at	---	---	6.69	7.10	6.86
216062_at	---	---	3.28	3.68	3.38
213623_at	kinesin family mei	KIF3A	7.19	7.51	7.27
202877_s_at	pre-B-cell leukemi	PBX2	5.69	5.76	5.46
204850_s_at	doublecortin	DCX	4.07	4.05	3.88
209561_at	thrombospondin 3	THBS3	6.83	6.93	6.63
203225_s_at	riboflavin kinase	RFK	9.62	9.65	9.72
202613_at	CTP synthase	CTPS	9.85	9.63	9.86
210350_x_at	inhibitor of growt	ING1	7.18	7.10	6.98
220584_at	hypothetical prote	FLJ22184	6.74	6.52	6.27
220684_at	T-box 21	TBX21	5.43	5.48	5.56
217760_at	tripartite motif-co	TRIM44	10.23	10.19	10.11
201038_s_at	acidic (leucine-ricl	ANP32A	9.11	9.24	9.35
208035_at	glutamate recepto	GRM6	5.09	5.08	5.25
211151_x_at	growth hormone	GH1	4.90	4.86	4.84
201177_s_at	ubiquitin-like moc	UBA2	11.72	11.75	11.92
207788_s_at	sorbin and SH3 dc	SORBS3	6.72	6.63	6.62
218266_s_at	neuronal calcium	NCS1	7.74	7.71	7.58
213170_at	glutathione perox	GPX7	4.11	4.21	4.07
209664_x_at	nuclear factor of a	NFATC1	5.26	5.26	5.03
210719_s_at	high-mobility grou	HMG20B	8.80	8.87	8.92
211634_x_at	immunoglobulin h	IGHM /// LOC1001	6.96	6.96	6.53
206577_at	vasoactive intestir	VIP	3.30	3.18	3.36
220126_at	protease, serine, 5	PRSS50	6.59	6.43	6.42
205777_at	dual specificity ph	DUSP9	5.89	5.62	5.71
219213_at	junctional adhesic	JAM2	5.31	5.61	5.37
214718_at	GATA zinc finger c	GATAD1	9.32	9.33	9.43
221769_at	splA/ryanodine re	SPSB3	7.52	7.35	7.67
207260_at	FEV (ETS oncogen	FEV	4.71	4.64	4.63
207248_at	potassium voltage	KCNA4	4.25	4.22	4.08
208114_s_at	interferon stimula	ISG20L2	9.01	9.19	9.21
207991_x_at	acrosomal vesicle	ACRV1	5.17	5.13	4.81
216066_at	ATP-binding casse	ABCA1	5.73	5.46	5.56
212185_x_at	metallothionein 2	MT2A	14.55	14.64	14.58
209283_at	crystallin, alpha B	CRYAB	7.20	7.00	7.12
214208_at	Kelch-like 35 (Dro	KLHL35	5.65	5.51	5.49
221667_s_at	heat shock 22kDa	HSPB8	9.73	9.64	9.92

214694_at	similar to Myosin	LOC729143 /// MF	6.94	6.72	6.38
217533_x_at	---	---	5.04	4.60	4.79
205671_s_at	major histocompa	HLA-DOB	5.61	5.97	5.67
212453_at	KIAA1279	KIAA1279	8.77	9.02	8.97
208529_at	basic transcriptior	BTF3L1	3.35	3.55	3.46
218373_at	AKT interacting pr	AKTIP	9.26	9.11	9.16
208985_s_at	eukaryotic transla	EIF3J	11.04	11.04	11.11
215685_s_at	distal-less homeol	DLX2	5.12	5.15	4.98
216748_at	pyrin and HIN dor	PYHIN1	4.08	4.38	4.22
205425_at	huntingtin interac	HIP1	7.81	7.82	7.79
207125_at	zinc finger proteir	ZNF225	4.29	4.32	4.12
207164_s_at	zinc finger proteir	ZNF238	3.40	3.47	3.47
212081_x_at	HLA-B associated	BAT2	8.39	8.13	8.29
211051_s_at	exostoses (multip	EXTL3	6.63	6.28	6.21
215659_at	Gasdermin B	GSDMB	4.85	4.52	4.53
207465_at	---	---	3.28	3.17	3.39
204349_at	mediator complex	MED7	8.34	8.31	8.35
201327_s_at	chaperonin contai	CCT6A	11.73	11.79	11.82
201801_s_at	solute carrier fam	SLC29A1	6.06	6.05	5.92
207072_at	interleukin 18 rec	IL18RAP	5.97	5.58	5.51
206464_at	BMX non-recepto	BMX	3.94	3.96	3.75
208948_s_at	staufen, RNA bind	STAU1	10.97	10.99	11.04
216481_at	glutamate recepto	GRIP2	6.61	6.37	6.08
211259_s_at	bone morphogene	BMP7	7.11	6.84	6.90
214548_x_at	GNAS complex loc	GNAS	12.08	12.01	11.98
214107_x_at	hypothetical prote	LOC440434	8.70	8.71	8.71
AFFX-r2-Bs-lys-M	---	---	7.58	7.44	7.57
212777_at	son of sevenless h	SOS1	6.13	6.56	6.06
34689_at	three prime repai	TREX1	8.04	8.27	8.10
208812_x_at	major histocompa	HLA-C	11.97	11.81	11.90
205659_at	histone deacetyla	HDAC9	7.94	7.86	8.32
217758_s_at	transmembrane 9	TM9SF3	10.38	10.37	10.58
206892_at	anti-Mullerian hor	AMHR2	7.33	7.11	6.93
213500_at	---	---	7.99	8.39	8.23
217303_s_at	adrenergic, beta-3	ADRB3	6.22	6.28	5.86
206617_s_at	renin binding prot	RENBP	4.84	4.77	4.46
200733_s_at	protein tyrosine p	PTP4A1	10.10	10.13	10.29
209577_at	phosphate cytidyl	PCYT2	7.81	7.80	7.48
206927_s_at	guanylate cyclase	GUCY1A2	4.92	5.01	4.81
215255_at	immunoglobulin s	IGSF9B	6.27	6.13	5.87
201943_s_at	carboxypeptidase	CPD	7.52	7.63	7.71
217469_at	Immunoglobulin h	IGHA1	5.43	4.95	5.30
211959_at	insulin-like growtl	IGFBP5	3.52	3.24	3.28
219471_at	chromosome 13 c	C13orf18	4.98	5.11	5.14
208272_at	RAN binding prote	RANBP3	6.39	6.20	6.12
204243_at	rearranged L-myc	RLF	7.95	7.85	7.93
217388_s_at	kynureninase (L-k	KYNU	5.17	5.35	5.07

202352_s_at	proteasome (pros	PSMD12	11.07	11.13	11.09
203404_at	armadillo repeat c	ARMCX2	3.79	4.04	3.98
221054_s_at	T-cell leukemia/ly	TCL6	3.72	3.64	3.58
201978_s_at	KIAA0141	KIAA0141	8.97	8.90	8.93
205788_s_at	zinc finger CCCH-t	ZC3H11A	11.11	11.05	11.11
209383_at	DNA-damage-indu	DDIT3	10.95	10.57	10.95
213336_at	bromodomain adj	BAZ1B	7.96	7.82	7.86
203366_at	polymerase (DNA	POLG	7.78	7.72	7.85
211450_s_at	mutS homolog 6 (	MSH6	7.40	7.64	7.59
202461_at	eukaryotic transla	EIF2B2	9.31	9.26	9.40
209590_at	bone morphogene	BMP7	4.35	4.35	4.30
205475_at	stimulator of chor	SCRG1	4.86	4.77	4.75
206129_s_at	arylsulfatase B	ARSB	5.19	5.39	5.23
215392_at	---	---	3.90	4.15	4.05
207097_s_at	solute carrier fam	SLC17A2	4.21	3.94	3.80
201747_s_at	scaffold attachme	SAFB	6.84	6.68	6.96
213015_at	bobby sox homolo	BBX	10.88	10.91	10.95
208210_at	MAS1 oncogene	MAS1	4.74	4.79	4.78
204772_s_at	transcription term	TTF1	8.03	8.34	8.20
209174_s_at	glutamine-rich 1	QRICH1	9.03	9.01	9.09
203371_s_at	NADH dehydroger	NDUFB3	11.47	11.64	11.56
205929_at	glycoprotein A33 (	GPA33	6.12	6.23	5.82
221101_at	---	---	5.14	4.98	5.04
212034_s_at	exocyst complex c	EXOC7	8.49	8.59	8.49
214234_s_at	cytochrome P450,	CYP3A5	3.63	3.96	3.95
213296_at	RER1 retention in	RER1	7.75	7.79	7.73
217313_at	---	---	8.23	8.29	8.40
213864_s_at	nucleosome asser	NAP1L1	13.19	13.21	13.25
207019_s_at	A kinase (PRKA) ar	AKAP4	5.69	5.51	5.35
201746_at	tumor protein p53	TP53	6.75	6.60	6.68
36499_at	cadherin, EGF LAG	CELSR2	6.83	6.98	6.89
215015_at	coiled-coil domair	CCDC64	7.76	7.29	7.23
202529_at	phosphoribosyl py	PRPSAP1	9.74	9.90	9.74
208122_x_at	killer cell immuno	KIR2DS3	5.59	5.81	5.63
206133_at	XIAP associated fa	XAF1	5.79	5.74	5.35
206151_x_at	chymotrypsin-like	CELA3B	6.14	5.85	5.80
220978_at	keratin associated	KRTAP1-3	3.38	3.46	3.44
202584_at	nuclear transcript	NFX1	8.62	8.67	8.59
202505_at	small nuclear ribo	SNRPB2	11.74	11.82	11.99
202467_s_at	COP9 constitutive	COPS2	11.68	11.88	11.81
204477_at	RAB interacting fa	RABIF	6.22	6.52	6.61
217723_x_at	---	---	7.96	8.16	7.83
208321_s_at	calcium binding pr	CABP1	5.92	5.67	5.75
207534_at	melanoma antiger	MAGEB1	3.59	3.63	3.29
200914_x_at	kinectin 1 (kinesin	KTN1	10.40	10.55	10.94
221187_s_at	fuzzy homolog (Dr	FUZ	7.60	7.38	7.16
218876_at	tubulin polymeriza	TPPP3	6.22	6.11	6.05

205072_s_at	X-ray repair comp	XRCC4	5.20	5.25	5.41
201990_s_at	cAMP responsive	CREBL2	7.75	7.76	8.03
203847_s_at	A kinase (PRKA) a	AKAP8	8.42	8.42	8.39
214621_at	glycogen synthase	GYS2	3.36	3.23	3.42
218369_s_at	exosome compon	EXOSC1	4.78	4.25	4.52
208059_at	chemokine (C-C m	CCR8	3.96	4.31	3.89
207819_s_at	ATP-binding casse	ABCB4	4.41	4.32	4.32
211143_x_at	nuclear receptor s	NR4A1	7.49	7.07	7.09
206386_at	serpin peptidase i	SERPINA7	4.73	4.31	4.51
217906_at	kelch domain con	KLHDC2	10.94	10.79	10.95
209371_s_at	SH3-domain bindi	SH3BP2	5.98	6.00	5.85
216462_at	---	---	3.46	3.45	3.30
219741_x_at	zinc finger protein	ZNF552	7.22	6.96	6.94
201089_at	ATPase, H+ transp	ATP6V1B2	9.35	9.29	9.32
217665_at	zinc finger protein	ZNF813	5.80	5.68	5.59
210546_x_at	cancer/testis anti	CTAG1A /// CTAG1	5.07	4.93	4.87
211030_s_at	solute carrier fam	SLC6A6	6.03	5.67	5.57
217572_at	---	---	5.16	5.19	4.85
48030_i_at	chromosome 5 op	C5orf4	8.82	8.99	8.81
221024_s_at	solute carrier fam	SLC2A10	3.53	3.50	3.34
201967_at	RNA binding moti	RBM6	8.02	8.21	8.30
212005_at	chromosome 1 op	C1orf144	8.32	8.30	8.22
218870_at	Rho GTPase activa	ARHGAP15	4.08	3.99	4.31
210366_at	solute carrier orga	SLCO1B1	4.66	4.61	4.35
204923_at	SAM and SH3 dom	SASH3	5.08	5.43	5.13
206692_at	potassium inward	KCNJ10	3.85	4.09	3.80
218704_at	ring finger protein	RNF43	6.89	6.73	6.72
221506_s_at	transportin 2	TNPO2	10.69	10.47	10.73
203023_at	NOP16 nucleolar	NOP16	9.77	9.84	10.10
206563_s_at	opiate receptor-lil	OPRL1	5.59	5.42	5.40
217504_at	ATP-binding casse	ABCA6	5.62	5.66	6.16
216553_x_at	---	---	3.81	3.93	3.86
202708_s_at	histone cluster 2,	HIST2H2BE	6.69	6.81	6.60
208439_s_at	ficolin (collagen/fi	FCN2	6.66	6.35	6.49
203685_at	B-cell CLL/lympho	BCL2	7.30	7.08	7.62
214983_at	testis-specific trar	TTTY15	3.59	3.56	3.66
209428_s_at	zinc finger protein	ZFPL1	7.58	7.55	7.37
211104_s_at	myosin VIIA	MYO7A	6.76	6.52	6.67
217601_at	nucleoporin 188kl	NUP188	6.77	6.57	6.49
210534_s_at	B9 protein domai	B9D1	7.50	7.24	7.48
210532_s_at	chromosome 14 c	C14orf2	12.46	12.41	12.40
213442_x_at	SAM pointed dom	SPDEF	7.63	7.51	7.49
220599_s_at	caspase recruitme	CARD14	4.91	4.88	4.98
208290_s_at	eukaryotic transla	EIF5	10.63	10.47	10.70
206831_s_at	arylsulfatase D	ARSD	5.29	5.52	5.31
221712_s_at	WD repeat domai	WDR74	9.83	9.63	9.75
214250_at	nuclear mitotic ap	NUMA1	5.39	5.72	5.40

202420_s_at	DEAH (Asp-Glu-Al	DHX9	9.73	9.93	9.77
214673_s_at	HECT, UBA and W	HUWE1	5.77	6.06	5.82
202089_s_at	solute carrier fam	SLC39A6	9.94	9.94	10.00
208220_x_at	amelogenin, Y-linl	AMELY	4.14	4.13	4.19
221446_at	ADAM metallopep	ADAM30	6.03	6.23	6.10
200680_x_at	high-mobility grou	HMGB1	13.89	14.01	13.95
204905_s_at	eukaryotic transla	EEF1E1	10.99	10.94	11.06
221075_s_at	natural cytotoxicit	NCR2	3.76	3.68	3.76
32069_at	leucine rich repea	LRRC14	8.66	8.84	8.68
205820_s_at	apolipoprotein C-I	APOC3	4.87	4.59	4.35
212630_at	exocyst complex c	EXOC3	8.67	8.63	8.39
212374_at	fem-1 homolog b	FEM1B	6.78	6.96	6.83
202579_x_at	high mobility grou	HMGN4	11.13	11.18	11.15
219665_at	nudix (nucleoside	NUDT18	8.10	8.20	8.11
214116_at	---	---	6.15	6.19	5.96
212749_s_at	ring finger and CH	RCHY1	9.60	9.58	9.74
217510_at	cone-rod homeob	CRX	4.69	4.66	4.42
215329_s_at	cyclin-dependent	CDK11A /// CDK11	7.27	7.25	7.26
205544_s_at	complement com	CR2	3.47	3.46	3.33
217718_s_at	tyrosine 3-monoo	YWHAB	13.31	13.33	13.36
221286_s_at	plasma cell-induce	MGC29506	5.18	5.01	4.99
212616_at	chromodomain he	CHD9	10.25	10.36	10.44
203584_at	tetratricopeptide	TTC35	9.61	9.85	9.85
207768_at	early growth resp	EGR4	3.93	3.55	3.51
207026_s_at	ATPase, Ca++ tran	ATP2B3	4.95	5.08	4.58
217509_x_at	glutamate recepto	GRIK5	8.04	7.76	7.72
220457_at	sterile alpha moti	SAMD4B	6.18	6.24	6.28
202463_s_at	methyl-CpG bindi	MBD3	8.19	7.99	8.03
209743_s_at	itchy E3 ubiquitin	ITCH	6.17	6.46	6.59
213878_at	pyridine nucleotid	PYROXD1	9.59	9.69	9.83
218820_at	chromosome 14 c	C14orf132	10.86	10.80	10.79
208879_x_at	PRP6 pre-mRNA p	PRPF6	9.50	9.50	9.43
218660_at	dysferlin, limb gir	DYSF	5.81	5.54	5.46
219018_s_at	coiled-coil domair	CCDC85C	6.66	6.65	6.47
212042_x_at	ribosomal protein	RPL7	14.09	14.14	14.20
212403_at	ubiquitin protein l	UBE3B	8.90	8.85	8.78
205234_at	solute carrier fam	SLC16A4	3.30	3.27	3.20
219868_s_at	ankyrin repeat an	ANKFY1	6.08	5.97	5.83
202456_s_at	zer-1 homolog (C.	ZER1	6.70	6.72	6.47
202791_s_at	SAPS domain fami	SAPS2	7.67	7.68	7.76
211781_x_at	---	---	6.21	6.21	6.01
206564_at	opiate receptor-lil	OPRL1	5.45	5.38	5.51
205629_s_at	corticotropin rele	CRH	6.90	7.04	6.42
201963_at	acyl-CoA syntheta	ACSL1	10.48	10.41	10.83
202317_s_at	ubiquitination fac	UBE4B	8.29	8.39	8.20
215368_at	nebulin	NEB	4.89	5.03	4.75
213385_at	chimerin (chimaer	CHN2	4.08	4.12	4.17

217364_x_at	---	---	7.25	7.13	7.22
48106_at	solute carrier fam	SLC48A1	8.47	8.38	8.34
204271_s_at	endothelin recept	EDNRB	9.05	9.03	9.31
210100_s_at	ATP-binding casse	ABCA2	6.34	5.89	5.84
219581_at	tRNA splicing end	TSEN2	9.56	9.61	9.63
212319_at	small G protein sig	SGSM2	6.32	6.44	6.39
217911_s_at	BCL2-associated a	BAG3	10.96	10.86	10.84
206305_s_at	complement com	C8A	4.31	4.13	3.98
207047_s_at	chloride channel K	CLCNKA /// CLCNK	4.17	4.08	3.80
216514_at	---	---	4.40	4.31	4.53
213274_s_at	cathepsin B	CTSB	8.08	7.99	7.96
204241_at	acyl-CoA oxidase 3	ACOX3	7.40	7.56	7.72
217986_s_at	bromodomain adj	BAZ1A	9.35	9.52	9.54
217882_at	transmembrane p	TMEM111	10.72	10.70	10.61
222030_at	SIVA1, apoptosis-i	SIVA1	6.56	6.50	6.58
220367_s_at	Sin3A-associated	SAP130	8.32	8.53	8.38
204041_at	monoamine oxida	MAOB	5.10	5.07	5.19
216705_s_at	adenosine deamir	ADA	8.29	8.08	8.13
202711_at	ephrin-B1	EFNB1	6.99	6.76	6.71
219142_at	RAS-like, family 1	RASL11B	7.84	7.81	7.82
206388_at	phosphodiesteras	PDE3A	5.73	5.52	5.52
211814_s_at	cyclin E2	CCNE2	4.48	4.94	4.62
203790_s_at	heat-responsive p	HRSP12	8.97	9.23	9.22
203473_at	solute carrier orga	SLCO2B1	6.98	6.75	6.87
215842_s_at	ATPase, class VI, t	ATP11A	6.08	6.01	5.43
216991_at	zinc finger proteir	ZNF224	4.03	4.07	4.21
216510_x_at	immunoglobulin h	IGHA1 /// IGHG1 /	5.26	5.19	5.09
203922_s_at	cytochrome b-245	CYBB	5.13	4.53	4.51
207990_x_at	acrosomal vesicle	ACRV1	4.94	4.91	4.55
216992_s_at	glutamate recepto	GRM8	3.49	3.69	3.42
219853_at	fukutin related pr	FKRP	6.13	6.36	6.40
213262_at	spastic ataxia of C	SACS	9.69	9.99	9.89
205146_x_at	amyloid beta (A4)	APBA3	6.87	6.95	6.75
207338_s_at	zinc finger proteir	ZNF200	7.19	7.29	7.17
211547_s_at	platelet-activating	PAFAH1B1	5.73	6.04	5.68
39763_at	hemopexin	HPX	6.61	6.61	6.43
209960_at	hepatocyte growt	HGF	3.27	3.07	3.14
219277_s_at	oxoglutarate dehy	OGDHL	5.03	5.14	4.95
212966_at	hypermethylated	HIC2	6.18	6.40	6.27
213744_at	attractin-like 1	ATRNL1	3.92	4.13	3.88
207174_at	glypican 5	GPC5	3.66	3.34	3.43
215822_x_at	myelin transcripti	MYT1	6.55	6.67	6.64
206666_at	granzyme K (gran	GZMK	4.94	4.83	4.69
213029_at	nuclear factor I/B	NFIB	9.24	9.07	9.35
221799_at	chondroitin polym	CHPF2	7.34	7.42	7.23
212793_at	dishevelled associ	DAAM2	5.73	5.87	5.58
206309_at	leukocyte cell der	LECT1	4.07	4.30	4.17

207048_at	solute carrier fam	SLC6A11	6.11	5.88	6.02
209476_at	thioredoxin-relate	TMX1	11.26	11.41	11.62
213356_x_at	heterogeneous nu	HNRNPA1 /// HNR	14.14	14.12	14.19
213599_at	Opa interacting pr	OIP5	10.48	10.40	10.41
206896_s_at	guanine nucleotid	GNG7	6.51	6.10	5.97
207507_s_at	ATP synthase, H+	ATP5G3	12.96	13.02	12.98
203784_s_at	DEAD (Asp-Glu-Al	DDX28	6.21	5.86	5.63
206717_at	myosin, heavy cha	MYH8	3.49	3.35	3.48
207675_x_at	artemin	ARTN	5.10	5.17	5.01
219338_s_at	leucine rich repea	LRRC49	8.39	8.32	8.53
217460_at	transition protein	TNP2	3.44	3.28	3.43
207630_s_at	cAMP responsive	CREM	8.54	8.63	8.44
213209_at	TAF6-like RNA pol	TAF6L	4.80	4.74	5.26
214129_at	phosphodiesteras	PDE4DIP	7.77	7.84	7.90
214616_at	histone cluster 1,	HIST1H3E	5.95	5.97	5.80
219646_at	differentially expr	DEF8	10.20	10.08	10.07
202306_at	polymerase (RNA)	POLR2G	11.76	11.63	11.60
210555_s_at	nuclear factor of a	NFATC3	8.41	8.31	8.49
208726_s_at	eukaryotic transla	EIF2S2	12.91	12.85	12.93
202123_s_at	c-abl oncogene 1,	ABL1	9.79	9.57	9.72
217332_at	CTAGE family, me	LOC647288	4.40	4.38	4.36
220631_at	O-sialoglycoprote	OSGEPL1	8.41	8.46	8.59
206789_s_at	POU class 2 home	POU2F1	7.51	7.60	7.50
222326_at	---	---	3.70	3.46	3.69
221200_at	---	---	3.96	4.08	4.00
40829_at	WD and tetratrico	WDTC1	7.70	7.61	7.58
202540_s_at	3-hydroxy-3-meth	HMGCR	10.22	10.74	10.38
208356_x_at	chorionic somato	CSH1	4.02	3.80	4.06
205908_s_at	osteomodulin	OMD	3.52	3.57	3.29
217174_s_at	adenomatosis pol	APC2	6.49	6.36	6.36
208818_s_at	catechol-O-methy	COMT	10.04	9.99	10.12
216351_x_at	deleted in azoosp	DAZ1 /// DAZ2 ///	3.71	3.66	3.94
210317_s_at	tyrosine 3-monoo	YWHAE	6.49	6.49	6.41
222217_s_at	solute carrier fam	SLC27A3	4.97	5.17	5.24
208754_s_at	nucleosome asser	NAP1L1	11.48	11.67	11.94
221714_s_at	RNA polymerase I	RRN3P1	6.71	6.42	6.49
203029_s_at	protein tyrosine p	PTPRN2	3.29	3.63	3.71
212505_s_at	KIAA0892	KIAA0892	8.52	8.47	8.51
218445_at	H2A histone famil	H2AFY2	6.32	6.59	6.50
213017_at	abhydrolase doma	ABHD3	7.82	7.87	7.73
201434_at	tetratricopeptide	TTC1	9.64	9.72	9.68
205666_at	flavin containing r	FMO1	4.35	3.85	4.03
222252_x_at	ubiquilin 4	UBQLN4	8.04	8.20	8.26
208021_s_at	replication factor	RFC1	10.05	10.06	10.09
213556_at	similar to R28379_	LOC390940	5.21	5.65	5.47
217217_at	immunoglobulin h	IGH@ /// IGHA1 //	4.86	5.18	5.04
204737_s_at	myosin, heavy cha	MYH6 /// MYH7	4.39	4.26	4.29

221347_at	cholinergic recept	CHRM5	3.90	4.03	4.00
201638_s_at	cleavage and poly	CPSF1	6.56	6.65	6.32
210693_at	signal peptide pep	SPPL2B	6.15	6.21	5.86
205311_at	dopa decarboxyla	DDC	4.92	4.73	4.67
208108_s_at	arginine vasopres	AVPR2	4.94	4.88	4.69
207192_at	deoxyribonucleas	DNASE1L2	6.60	6.47	6.53
201058_s_at	myosin, light chai	MYL9	7.25	7.06	6.94
209099_x_at	jagged 1 (Alagille	JAG1	11.13	10.91	11.14
215080_s_at	ArfGAP with GTPa	AGAP2	4.83	4.36	4.45
217640_x_at	spindle and kinetc	SKA1	7.57	7.62	7.82
218410_s_at	phosphoglycolate	PGP	7.21	7.12	7.04
209455_at	F-box and WD rep	FBXW11	9.49	9.44	9.63
217416_x_at	---	---	6.29	6.25	6.35
222256_s_at	jumonji domain c	JMJD7 /// JMJD7-F	7.41	7.35	7.35
210321_at	granzyme H (cath	GZMH	4.45	4.18	4.12
210613_s_at	synaptogyrin 1	SYNGR1	6.61	6.78	6.80
217391_x_at	---	---	6.51	6.38	6.42
200000_s_at	PRP8 pre-mRNA p	PRPF8	9.46	9.33	9.47
209695_at	protein tyrosine p	PTP4A3	6.61	6.39	6.44
218752_at	zinc finger, matrin	ZMAT5	7.81	7.64	7.65
204408_at	APEX nuclease (ap	APEX2	8.06	8.00	7.79
205553_s_at	cysteine and glyci	CSRP3	4.63	5.05	4.76
209176_at	SEC23 interacting	SEC23IP	6.82	6.97	7.25
209913_x_at	KIAA0415	KIAA0415	7.15	7.38	7.51
57532_at	dishevelled, dsh h	DVL2	8.64	8.64	8.71
220081_x_at	hydroxysteroid (1	HSD17B7	9.65	9.88	9.73
215199_at	caldesmon 1	CALD1	5.32	5.35	5.13
208369_s_at	glutaryl-CoA dehy	GCDH	8.08	8.26	8.42
218442_at	tetratricopeptide	TTC4	9.22	9.15	9.20
215050_x_at	mitogen-activatec	MAPKAPK2	5.78	6.01	5.96
205391_x_at	ankyrin 1, erythro	ANK1	6.58	6.49	6.49
208497_x_at	neurogenin 1	NEUROG1	5.03	5.26	5.05
216214_at	---	---	3.53	3.53	3.62
204404_at	solute carrier fam	SLC12A2	6.85	6.81	6.82
206175_x_at	zinc finger proteir	ZNF222	6.39	6.35	6.47
221434_s_at	chromosome 14 c	C14orf156	12.40	12.48	12.40
214068_at	brain expressed, a	BEAN	4.10	4.62	4.53
220612_at	---	---	6.12	6.33	6.27
207860_at	natural cytotoxicit	NCR1	4.68	4.26	4.28
203994_s_at	chromosome 21 c	C21orf2	7.23	7.05	6.68
203835_at	leucine rich repea	LRRC32	6.55	6.46	6.07
221810_at	RAB15, member F	RAB15	6.09	6.28	6.16
205346_at	ST3 beta-galactos	ST3GAL2	5.48	4.96	5.21
213822_s_at	ubiquitin protein l	UBE3B	7.94	7.92	7.97
212300_at	taxilin alpha	TXLNA	8.25	8.15	8.36
203140_at	B-cell CLL/lympho	BCL6	11.56	11.52	11.60
201956_s_at	glyceronephospha	GNPAT	10.59	10.64	10.67

216967_at	hypothetical prote	LOC100288551	3.48	3.09	3.55
209327_s_at	NOP16 nucleolar	NOP16	6.10	5.87	5.59
220056_at	interleukin 22 rec	IL22RA1	5.78	6.02	5.49
206911_at	tripartite motif-co	TRIM25	7.69	7.41	7.79
203663_s_at	cytochrome c oxic	COX5A	12.79	12.81	12.90
215369_at	---	---	6.77	6.57	6.29
218936_s_at	coiled-coil domair	CCDC59	9.90	10.00	10.19
210882_s_at	trophinin	TRO	4.15	4.17	3.97
208148_at	myosin, heavy cha	MYH4	3.72	3.72	3.85
209150_s_at	transmembrane 9	TM9SF1	9.86	9.87	9.97
216069_at	---	---	3.66	3.72	3.38
204793_at	G protein-couplec	GPRASP1	5.03	5.22	5.15
221014_s_at	RAB33B, member	RAB33B	7.90	7.87	8.12
214457_at	homeobox A2	HOXA2	3.67	3.72	3.67
206050_s_at	ribonuclease/angi	RNH1	9.81	9.64	9.75
203034_s_at	ribosomal protein	RPL27A	14.89	14.91	14.94
207195_at	contactin 6	CNTN6	3.37	3.43	3.50
214112_s_at	chromosome X op	CXorf40A /// CXor	9.21	9.19	9.18
214503_x_at	G protein-couplec	GPR135	3.99	3.86	3.64
203786_s_at	tumor protein D5	TPD52L1	8.73	8.65	8.64
200669_s_at	ubiquitin-conjugat	UBE2D3	11.09	11.25	11.35
214044_at	ryanodine recepto	RYR2	3.54	3.57	3.53
221968_s_at	Zinc finger proteir	ZNF771	7.08	6.84	6.86
216089_at	multiple coagulati	MCFD2	3.33	3.43	3.19
209021_x_at	KIAA0652	KIAA0652	7.28	7.57	7.40
204677_at	cadherin 5, type 2	CDH5	4.78	4.72	4.48
206032_at	desmocollin 3	DSC3	3.69	3.96	3.54
205035_at	CTD (carboxy-tern	CTDP1	7.53	7.60	7.69
218386_x_at	ubiquitin specific	USP16	10.01	10.17	10.26
218749_s_at	solute carrier fam	SLC24A6	7.31	7.26	7.18
216882_s_at	nebulette	NEBL	6.57	6.34	6.61
204331_s_at	mitochondrial ribo	MRPS12	11.45	11.36	11.32
204373_s_at	centrosomal prote	CEP350	8.75	8.85	8.81
207983_s_at	stromal antigen 2	STAG2	9.86	9.73	9.80
209793_at	glutamate recepto	GRIA1	3.82	3.79	3.84
203951_at	calponin 1, basic,	CNN1	5.74	5.69	5.51
55081_at	MICAL-like 1	MICALL1	10.30	10.14	10.37
218142_s_at	cereblon	CRBN	9.08	9.13	9.10
221592_at	TBC1 domain fam	TBC1D8	6.41	6.50	6.34
209782_s_at	D site of albumin	DBP	5.70	5.50	5.39
202047_s_at	chromobox homo	CBX6	9.66	9.72	9.75
219915_s_at	solute carrier fam	SLC16A10	3.17	3.24	3.34
217575_s_at	son of sevenless h	SOS2	3.50	3.20	3.39
217476_at	nuclear receptor s	NR1D1 /// THRA	6.28	5.82	5.69
205021_s_at	forkhead box N3	FOXN3	7.10	7.26	7.05
91816_f_at	mex-3 homolog D	MEX3D	8.62	8.73	8.84
211577_s_at	insulin-like growtl	IGF1	5.92	5.72	5.72

206323_x_at	oligophrenin 1	OPHN1	10.09	10.34	10.35
205539_at	advillin	AVIL	5.59	5.58	5.40
204632_at	ribosomal protein	RPS6KA4	9.06	8.93	8.66
212352_s_at	transmembrane e	TMED10	11.46	11.62	11.58
214851_at	hepatocyte nucle	HNF4A	5.64	5.61	5.86
216568_x_at	---	---	6.80	6.50	6.57
217519_at	---	---	4.44	4.50	4.32
200602_at	amyloid beta (A4)	APP	10.41	10.43	10.54
208200_at	interleukin 1, alph	IL1A	5.13	4.85	4.80
221040_at	calpain 10	CAPN10	3.30	3.28	3.50
202710_at	blocked early in tr	BET1	9.87	9.96	10.10
221003_s_at	calcium binding pr	CAB39L	6.32	6.26	5.84
211034_s_at	chromosome 12 c	C12orf51	7.51	7.51	7.45
204187_at	guanosine monop	GMPR	7.93	8.00	7.92
221020_s_at	solute carrier fam	SLC25A32	9.96	9.84	10.16
214977_at	---	---	6.13	6.02	5.83
215841_at	guanylate cyclase	GUCA1B	7.06	7.13	6.73
218117_at	ring-box 1	RBX1	11.44	11.54	11.54
207043_s_at	solute carrier fam	SLC6A9	4.81	4.31	4.10
210166_at	toll-like receptor 5	TLR5	4.82	4.89	4.62
221301_at	chromosome 6 op	C6orf27	6.59	6.58	6.31
215608_at	---	---	4.33	4.58	4.46
221604_s_at	peroxisomal bioge	PEX16	8.40	8.37	8.34
215038_s_at	SET domain conta	SETD2	7.23	7.24	7.37
204907_s_at	B-cell CLL/lympho	BCL3	5.94	6.12	5.71
219816_s_at	RNA binding moti	RBM23	8.59	8.77	8.80
216831_s_at	runt-related trans	RUNX1T1	5.48	5.12	4.99
222040_at	heterogeneous nu	HNRNPA1	7.79	8.09	7.94
207699_at	zinc finger homeo	ZFHX2	6.00	6.02	5.78
211481_at	solute carrier orga	SLCO1A2	3.55	3.59	3.39
209648_x_at	suppressor of cyto	SOCS5	8.59	8.71	8.94
219548_at	zinc finger protein	ZNF16	7.53	7.46	7.61
205200_at	C-type lectin dom	CLEC3B	5.85	5.89	6.02
203884_s_at	RAB11 family inte	RAB11FIP2	9.95	9.92	10.09
222238_s_at	polymerase (DNA	POLM	6.14	5.92	6.12
216721_at	Solute carrier fam	SLC25A30	4.45	4.40	4.42
208554_at	POU class 4 home	POU4F3	4.25	4.41	4.31
219079_at	cytochrome b5 re	CYB5R4	8.47	8.51	8.56
209025_s_at	synaptotagmin bi	SYNCRIP	10.88	11.06	11.12
206880_at	purinergic recepto	P2RX6	6.49	6.37	6.39
214047_s_at	methyl-CpG bindi	MBD4	9.68	9.66	9.95
207792_at	opioid receptor, d	OPRD1	6.53	6.17	6.41
219873_at	collectin sub-fami	COLEC11	5.54	5.36	5.36
215121_x_at	cyclosporin A tran	CYAT1 /// IGLV1-4	6.14	6.22	5.83
214062_x_at	nuclear factor of k	NFKBIB	7.95	7.88	7.68
220785_at	urotensin 2	UTS2	3.30	3.31	3.43
210354_at	interferon, gamm	IFNG	3.69	3.97	3.64

204523_at	zinc finger protein	ZNF140	7.56	7.63	7.82
208832_at	ataxin 10	ATXN10	8.53	8.55	8.43
208183_at	tachykinin recepto	TACR3	4.67	4.47	4.67
219164_s_at	ATG2 autophagy r	ATG2B	7.35	7.31	7.42
210817_s_at	calcium binding ar	CALCOCO2	9.94	9.89	9.97
205948_at	protein tyrosine p	PTPRT	4.96	5.32	4.94
219326_s_at	UDP-GlcNAc:beta	B3GNT2	5.47	5.67	5.61
201050_at	phospholipase D f	PLD3	6.78	6.66	6.63
221995_s_at	---	---	6.86	6.87	7.00
203793_x_at	polycomb group r	PCGF2	7.10	7.11	6.89
201825_s_at	saccharopine deh	SCCPDH	10.42	10.43	10.45
210368_at	protocadherin gar	PCDHGA8	3.81	3.77	3.72
208854_s_at	serine/threonine I	STK24	9.63	9.38	9.53
213602_s_at	---	---	6.14	6.21	5.92
201046_s_at	RAD23 homolog A	RAD23A	10.44	10.35	10.44
204754_at	hepatic leukemia	HLF	4.87	4.47	4.36
221657_s_at	ankyrin repeat an	ASB6	8.19	8.10	8.18
37943_at	zinc finger, FYVE c	ZFYVE26	6.77	7.01	7.06
217638_at	---	---	5.65	5.50	6.04
207800_at	A kinase (PRKA) ar	AKAP5	4.05	3.80	3.98
211457_at	GABA(A) receptor	GABARAPL3	4.03	3.89	3.94
203252_at	cyclin-dependent	CDK2AP2	8.73	8.72	8.62
222345_at	---	---	6.65	6.25	6.40
207671_s_at	bestrophin 1	BEST1	6.16	5.87	5.94
214777_at	immunoglobulin k	IGKV4-1	5.10	4.84	5.03
200815_s_at	platelet-activating	PAFAH1B1	9.06	9.01	9.01
202364_at	MAX interactor 1	MXI1	11.76	11.71	11.79
203505_at	ATP-binding casse	ABCA1	5.72	6.18	6.23
212933_x_at	ribosomal protein	RPL13	14.40	14.33	14.37
204027_s_at	methyltransferase	METTL1	8.46	8.37	8.42
217730_at	transmembrane B	TMBIM1	10.37	10.30	10.19
74694_s_at	rabaptin, RAB GTF	RABEP2	7.61	7.59	7.65
222193_at	chromosome 2 op	C2orf43	7.92	7.96	7.99
210083_at	semaphorin 7A, G	SEMA7A	6.64	6.71	6.35
215272_at	8-oxoguanine DN/	OGG1	6.42	6.39	5.94
217545_at	myosin, heavy cha	MYH14	7.34	7.19	7.14
217975_at	WW domain bindi	WBP5	9.17	9.47	9.41
215888_at	PDS5, regulator of	PDS5B	6.33	6.37	6.37
220259_at	pleckstrin homolo	PLEKHH3	4.71	4.55	4.35
206344_at	paraoxonase 1	PON1	4.05	4.06	3.72
221109_at	---	---	7.32	7.09	6.95
216914_at	cell division cycle	CDC25C	5.33	5.26	5.50
219426_at	eukaryotic transla	EIF2C3	8.39	8.38	8.37
214201_x_at	HLA-B associated	BAT2	9.30	8.92	9.01
219743_at	hairy/enhancer-of	HEY2	3.74	3.83	3.65
202622_s_at	ataxin 2	ATXN2	9.22	9.05	9.10
214307_at	homogentisate 1,2	HGD	3.91	3.90	3.55

220249_at	hyaluronoglucosa	HYAL4	4.45	4.31	4.22
208952_s_at	La ribonucleoprot	LARP4B	8.17	8.15	8.17
203175_at	ras homolog gene	RHOG	8.30	8.38	8.36
216593_s_at	similar to phospha	LOC100289666 ///	7.31	7.24	7.21
205520_at	striatin, calmoduli	STRN	5.30	5.20	5.29
221274_s_at	lectin, mannose-b	LMAN2L	9.19	9.10	9.14
201705_at	proteasome (pros	PSMD7	10.22	10.40	10.43
218656_s_at	lipoma HMGIC fus	LHFP	9.74	9.82	10.02
221688_s_at	IMP3, U3 small nu	IMP3	10.67	10.59	10.62
200645_at	GABA(A) receptor	GABARAP	10.75	10.72	10.71
202725_at	polymerase (RNA)	POLR2A	6.66	6.52	6.64
218904_s_at	chromosome 9 op	C9orf40	7.39	7.62	7.68
201918_at	solute carrier fam	SLC25A36	11.29	11.35	11.31
217209_at	carcinoembryonic	CEACAM3	4.25	4.39	4.14
211815_s_at	golgi-associated, g	GGA3	6.38	6.33	6.20
203940_s_at	vasohibin 1	VASH1	5.63	6.20	5.82
220120_s_at	erythrocyte meml	EPB41L4A	3.46	3.47	3.54
214846_s_at	alpha-kinase 3	ALPK3	5.77	5.79	5.49
221362_at	5-hydroxytryptam	HTR5A	7.21	7.13	7.02
205624_at	carboxypeptidase	CPA3	4.06	4.27	3.91
202413_s_at	ubiquitin specific		USP1	12.08	12.13	12.24
208209_s_at	complement com	C4BPB	6.44	6.35	6.43
219344_at	solute carrier fam	SLC29A3	7.34	7.41	7.33
215751_at	RNA binding moti	RBM26	3.51	3.43	3.43
220762_s_at	guanine nucleotid	GNB1L	7.67	7.68	7.58
214267_s_at	Cell adhesion mol	CADM4	6.24	5.94	5.86
218949_s_at	glutaminyl-tRNA s	QRSL1	8.89	8.69	9.04
37278_at	tafazzin	TAZ	8.61	8.64	8.71
208201_at	double homeobox	DUX2	5.49	5.36	5.39
218906_x_at	kinesin light chain	KLC2	9.22	9.30	9.01
210393_at	leucine-rich repea	LGR5	4.17	4.00	4.11
210886_x_at	TP53 target 1 (nor	TP53TG1	7.54	7.68	7.63
211435_at	---	---	4.68	4.85	4.70
215174_at	flavin containing r	FMO6P	4.16	4.17	3.99
209003_at	solute carrier fam	SLC25A11	7.62	7.80	7.59
201080_at	phosphatidylinosi	PIP4K2B	7.67	7.71	7.76
200064_at	heat shock protei	HSP90AB1	12.72	12.73	12.63
219556_at	chromosome 16 o	C16orf59	8.26	8.10	8.32
213394_at	mitogen-activated	MAPKBP1	7.63	7.73	7.57
220757_s_at	UBX domain prote	UBXN6	7.06	7.03	7.00
217220_at	Hypothetical prot	LOC100287387	5.62	5.35	5.81
207358_x_at	microtubule-actin	MACF1	9.23	9.20	9.41
207393_at	hypocretin (orexin	HCRTR2	3.99	4.07	4.04
201138_s_at	Sjogren syndrome	SSB	11.18	11.43	11.44
214014_at	CDC42 effector pr	CDC42EP2	8.42	8.31	8.39
201163_s_at	insulin-like growth	IGFBP7	8.99	9.39	9.50
220149_at	chromosome 2 op	C2orf54	5.86	5.81	5.81

214524_at	growth hormone r	GHRH	5.12	4.66	4.67
216533_at	Propionyl Coenzyr	PCCA	3.07	3.23	3.18
206972_s_at	G protein-couplec	GPR161	4.96	4.94	5.09
212105_s_at	DEAH (Asp-Glu-Ala	DHX9	6.54	7.01	6.55
204758_s_at	C2CD2-like	C2CD2L	4.76	4.51	4.65
201261_x_at	biglycan	BGN	5.89	5.83	5.89
215564_at	Amphiregulin	AREG	3.50	3.56	3.41
216430_x_at	immunoglobulin la	IGLV1-44 /// LOC1	5.07	4.93	4.88
210338_s_at	heat shock 70kDa	HSPA8	12.29	12.57	12.32
64064_at	GTPase, IMAP fam	GIMAP5	6.89	6.94	6.72
207987_s_at	gonadotropin-rele	GNRH1	4.12	4.18	3.94
221097_s_at	potassium large co	KCNMB2	3.76	3.97	3.91
214578_s_at	Rho-associated, co	ROCK1	8.12	8.21	8.09
200595_s_at	eukaryotic transla	EIF3A	11.69	11.74	11.86
217426_at	---	---	4.38	4.23	4.33
213294_at	---	---	9.38	9.52	9.56
215149_at	---	---	4.68	4.32	4.54
202938_x_at	ribosomal RNA pro	RRP7A /// RRP7B	6.78	6.90	6.82
206359_at	suppressor of cyto	SOCS3	5.58	5.36	5.43
55692_at	engulfment and ce	ELMO2	7.83	7.84	7.88
217060_at	similar to hCG200	LOC100293532	4.44	4.28	4.19
208775_at	exportin 1 (CRM1	XPO1	12.55	12.49	12.48
206870_at	peroxisome prolif	PPARA	4.58	4.74	4.23
202324_s_at	acyl-CoA binding c	ACBD3	9.54	9.68	9.73
208042_at	angiogenic factor	AGGF1	7.20	7.37	7.33
214866_at	plasminogen activ	PLAUR	7.28	7.25	7.12
202303_x_at	SWI/SNF related,	SMARCA5	9.03	9.22	9.08
217253_at	SH3-domain bindi	SH3BP2	6.31	6.25	6.39
221515_s_at	leucine carboxyl n	LCMT1	10.15	10.10	10.15
207209_at	centrin, EF-hand p	CETN1	5.25	5.18	5.52
200984_s_at	CD59 molecule, co	CD59	11.00	11.12	11.17
209859_at	tripartite motif-co	TRIM9	3.74	3.70	3.40
203578_s_at	solute carrier fam	SLC7A6	6.80	7.00	6.99
211387_x_at	RNA guanylyltrans	RNGTT	5.88	5.88	5.75
202715_at	carbamoyl-phospl	CAD	9.62	9.31	9.37
219693_at	1-acylglycerol-3-p	AGPAT4	7.97	8.04	7.85
212012_at	peroxidasin homo	PXDN	6.62	6.53	6.41
215932_at	similar to melanor	LOC100292575 ///	4.87	4.85	4.34
222236_s_at	ArfGAP with SH3 c	ASAP3	7.14	7.31	7.31
217669_s_at	A kinase (PRKA) an	AKAP6	5.41	5.11	5.28
204859_s_at	apoptotic peptida	APAF1	7.31	7.66	7.45
213031_s_at	WD repeat domai	WDR73	8.78	8.83	8.82
211625_s_at	dopamine recepto	DRD3	4.47	4.52	4.57
215142_at	chromosome X op	CXorf27	4.81	5.09	4.66
211642_at	Immunoglobulin h	IGHG1 /// ZCWPW	3.56	3.25	3.42
216277_at	budding uninhibit	BUB1	4.09	4.27	4.25
222177_s_at	SCAN domain con	SCAND2	3.79	4.19	3.98

207305_s_at	KIAA1012	KIAA1012	9.88	10.05	10.09
212471_at	AVL9 homolog (S.	AVL9	6.87	6.72	6.83
217692_at	mago-nashi homo	MAGOH2	4.06	3.97	3.99
207998_s_at	calcium channel, v	CACNA1D	5.57	5.60	5.27
219031_s_at	nuclear import 7 h	NIP7	9.88	9.83	10.26
208620_at	poly(rC) binding p	PCBP1	11.48	11.55	11.47
205011_at	von Willebrand fa	VWA5A	6.03	6.32	6.34
217836_s_at	YY1 associated pr	YY1AP1	10.33	10.13	10.21
216226_at	TAF4b RNA polym	TAF4B	6.85	6.69	6.76
222123_s_at	hypoxia inducible	HIF3A	4.99	4.94	4.89
205745_x_at	ADAM metallopep	ADAM17	8.34	8.36	8.71
206644_at	nuclear receptor s	NR0B1	3.27	3.63	3.28
217537_x_at	---	---	3.60	3.26	3.44
212893_at	zinc finger, ZZ-typ	ZZZ3	9.97	9.96	10.16
202350_s_at	matrilin 2	MATN2	3.61	3.66	3.60
207533_at	chemokine (C-C m	CCL1	4.22	4.09	3.96
214274_s_at	acetyl-CoA acyltra	ACAA1	9.58	9.62	9.55
218790_s_at	trimethyllysine hy	TMLHE	4.61	5.16	4.68
204446_s_at	arachidonate 5-lip	ALOX5	3.38	3.43	3.42
222062_at	interleukin 27 rec	IL27RA	6.75	6.77	6.80
203813_s_at	slit homolog 3 (Dr	SLIT3	4.40	4.42	4.37
215176_x_at	immunoglobulin k	IGK@ /// IGKC ///	5.13	5.16	4.85
AFFX-r2-P1-cre-3	---	---	14.73	14.74	14.71
218044_x_at	parathymosin	PTMS	7.29	7.16	6.95
218205_s_at	MAP kinase intera	MKNK2	12.31	12.12	12.37
216399_s_at	S-phase cyclin A-a	SCAPER	5.93	5.83	6.03
209379_s_at	family with seque	FAM190B	8.88	9.05	9.31
212565_at	serine/threonine k	STK38L	7.78	7.98	8.11
32837_at	1-acylglycerol-3-p	AGPAT2	7.88	8.00	8.00
209065_at	ubiquinol-cytochr	UQCRB	8.62	8.60	8.64
207781_s_at	zinc finger protein	ZNF711	3.05	3.06	3.16
212589_at	related RAS viral (	RRAS2	11.25	11.05	11.40
208219_at	activin A receptor	ACVR1B	5.51	5.07	5.40
218388_at	6-phosphoglucono	PGLS	9.26	9.16	9.20
213083_at	solute carrier fam	SLC35D2	4.41	4.24	4.22
209415_at	fizzy/cell division c	FZR1	8.21	8.12	8.02
209139_s_at	protein kinase, int	PRKRA	10.09	10.09	10.23
216684_s_at	synovial sarcoma	SS18	6.63	6.24	6.42
207669_at	keratin 83	KRT83	7.34	6.92	6.96
218562_s_at	transmembrane p	TMEM57	7.54	7.50	7.31
216485_s_at	tryptase alpha/be	TPSAB1	6.88	7.04	6.83
214233_at	hypothetical prote	LOC100288178	6.32	6.18	6.39
204998_s_at	activating transcri	ATF5	8.03	7.54	7.84
214626_s_at	glucosidase, alpha	GANAB	11.02	10.98	10.91
203375_s_at	tripeptidyl peptida	TPP2	9.28	9.44	9.49
219261_at	chromosome 7 op	C7orf26	8.47	8.37	8.18
217248_s_at	solute carrier fam	SLC7A8	4.43	4.44	4.44

213381_at	chromosome 10 c	C10orf72	6.67	6.55	6.54
211733_x_at	sterol carrier prot	SCP2	10.15	10.43	10.54
220662_s_at	hairy/enhancer-of	HEYL	7.42	6.94	6.91
207424_at	myogenic factor 5	MYF5	5.10	4.73	4.85
207726_at	estrogen-related r	ESRRB	5.16	5.31	5.24
204646_at	dihydropyrimidine	DPYD	9.15	9.12	9.27
211248_s_at	chordin	CHRD	5.64	5.54	5.83
205223_at	DEP domain conta	DEPDC5	7.12	7.20	6.97
206026_s_at	tumor necrosis fac	TNFAIP6	7.91	7.73	7.97
222096_x_at	---	---	6.49	6.33	6.51
222008_at	collagen, type IX, a	COL9A1	5.58	5.83	5.61
204130_at	hydroxysteroid (1	HSD11B2	5.46	5.56	5.21
210723_x_at	---	---	5.45	5.31	5.37
208004_at	proline rich, lacrin	PROL1	3.44	3.72	3.42
217812_at	YTH domain famil	YTHDF2	10.87	10.89	10.91
208781_x_at	sorting nexin 3	SNX3	11.61	11.73	11.70
202277_at	serine palmitoyltr	SPTLC1	10.80	11.01	11.09
201088_at	karyopherin alpha	KPNA2	12.12	12.43	12.46
211600_at	protein tyrosine p	PTPRO	11.05	11.05	11.12
211413_s_at	peptidyl arginine d	PADI4	5.18	5.04	4.80
213371_at	LIM domain bindi	LDB3	4.91	4.82	5.00
201889_at	family with seque	FAM3C	9.80	9.97	10.08
202958_at	protein tyrosine p	PTPN9	7.55	7.70	7.68
220809_at	---	---	4.67	4.68	4.57
221172_at	chromosome 7 op	C7orf69	4.40	3.85	4.06
211240_x_at	catenin (cadherin-	CTNND1	7.83	7.98	7.87
40149_at	SH2B adaptor pro	SH2B1	7.64	7.53	7.37
34408_at	reticulon 2	RTN2	7.25	7.27	7.13
201731_s_at	translocated prom	TPR	10.26	10.53	10.52
200096_s_at	ATPase, H+ transp	ATP6V0E1	11.98	12.04	12.06
220928_s_at	PR domain contai	PRDM16	5.42	5.12	5.51
209204_at	LIM domain only 4	LMO4	8.24	8.00	8.26
206243_at	TIMP metallopept	TIMP4	4.78	4.46	4.46
219760_at	lin-7 homolog B (C	LIN7B	6.78	6.57	6.55
211662_s_at	voltage-depender	VDAC2	12.83	12.77	12.87
215094_at	coiled-coil domair	CCDC52	3.76	3.86	3.43
213519_s_at	laminin, alpha 2	LAMA2	6.44	6.05	6.19
205818_at	deleted in bladder	DBC1	4.62	4.34	4.25
207431_s_at	degenerative sper	DEGS1	9.21	9.37	9.45
210527_x_at	tubulin, alpha 3c	TUBA3C	7.83	7.72	7.68
218612_s_at	tumor suppressing	TSSC4	8.40	8.37	8.20
211129_x_at	ectodysplasin A	EDA	4.66	4.46	4.50
203411_s_at	lamin A/C	LMNA	11.22	11.11	11.21
219064_at	inter-alpha (globu	ITIH5	5.84	5.55	5.61
221961_at	chloride channel 7	CLCN7	7.14	6.81	6.64
177_at	farnesyltransferas	FNTB	4.13	4.24	4.17
208500_x_at	forkhead box D3	FOXD3	3.73	3.90	3.84

208538_at	acidic (leucine-ricl	ANP32C	4.48	4.51	4.78
49327_at	sirtuin (silent mat	SIRT3	8.15	8.37	8.11
204620_s_at	versican	VCAN	6.87	7.22	7.02
216779_at	cylicin, basic prote	CYLC1	4.43	4.11	4.25
219929_s_at	zinc finger, FYVE c	ZFYVE21	7.49	7.51	7.63
31837_at	lipase maturation	LMF2	8.29	8.44	8.33
211402_x_at	nuclear receptor s	NR6A1	6.20	6.50	6.39
208847_s_at	alcohol dehydroge	ADH5	11.32	11.36	11.39
221670_s_at	LIM homeobox 3	LHX3	4.54	4.70	4.58
209735_at	ATP-binding casse	ABCG2	7.94	7.97	7.99
210843_s_at	microfibrillar-asso	MFAP3L	4.03	3.85	3.74
211557_x_at	solute carrier orga	SLCO2B1	6.24	6.25	6.50
210149_s_at	ATP synthase, H+	ATP5H	12.74	12.76	12.74
212278_x_at	ubiquitin protein l	UBE3A	6.15	5.74	5.94
206154_at	retinaldehyde bin	RLBP1	6.52	6.27	6.23
211088_s_at	polo-like kinase 4	PLK4	3.36	3.23	3.70
210411_s_at	glutamate recepto	GRIN2B	5.86	5.74	5.60
214320_x_at	cytochrome P450,	CYP2A6	7.21	6.93	6.70
210380_s_at	calcium channel, v	CACNA1G	6.35	6.08	5.94
218819_at	integrator comple	INTS6	10.04	10.08	10.03
206027_at	S100 calcium bind	S100A3	7.40	7.28	7.35
212992_at	AHNAK nucleopro	AHNAK2	10.21	10.34	10.24
221507_at	transportin 2	TNPO2	9.32	9.15	9.50
208672_s_at	splicing factor, arg	SFRS3	12.74	12.88	12.83
208835_s_at	LUC7-like 3 (S. cer	LUC7L3	11.39	11.59	11.48
218806_s_at	vav 3 guanine nuc	VAV3	6.97	7.03	6.96
212717_at	pleckstrin homolo	PLEKHM1	7.92	7.95	7.74
222246_at	---	---	3.69	3.45	3.44
204627_s_at	integrin, beta 3 (p	ITGB3	5.13	5.28	5.02
222202_at	---	---	3.43	3.25	3.22
202688_at	tumor necrosis fa	TNFSF10	2.94	3.22	3.16
217423_at	---	---	4.21	4.29	4.08
216279_at	zinc finger protein	ZNF460	3.53	3.48	3.46
216143_at	---	---	6.77	6.72	6.40
220041_at	phosphatidylinosi	PIGZ	6.63	6.84	6.63
209083_at	coronin, actin bin	CORO1A	5.68	5.75	5.81
207370_at	integrin-binding si	IBSP	6.48	6.37	6.25
204293_at	N-sulfoglucosamir	SGSH	6.97	7.04	6.92
211280_s_at	nuclear respirator	NRF1	6.25	6.35	6.36
210248_at	wingless-type MM	WNT7A	5.60	5.20	5.32
218972_at	tetratricopeptide	TTC17	8.29	8.26	8.27
209916_at	dehydrogenase E1	DHTKD1	7.93	8.04	7.88
221785_at	widely interspace	WIZ	5.90	5.66	5.61
203785_s_at	DEAD (Asp-Glu-Al	DDX28	7.73	7.83	7.81
214052_x_at	HLA-B associated	BAT2L2	6.87	7.05	6.85
205951_at	myosin, heavy cha	MYH1	3.98	3.65	3.53
205589_at	myosin, light chai	MYL3	6.89	6.84	6.76

212357_at	family with seque	FAM168A	7.36	7.46	7.52
220701_at	non-protein codin	NCRNA00216	3.84	3.85	3.84
214604_at	homeobox D11	HOXD11	5.10	4.81	5.06
207066_at	histidine rich calci	HRC	5.08	4.90	4.68
204968_at	chromosome 6 op	C6orf47	8.05	7.74	7.83
205467_at	caspase 10, apopt	CASP10	6.67	6.47	6.48
222179_at	---	---	6.41	6.26	6.12
216574_s_at	rcRPE /// ribulose	LOC729020 /// RPI	7.09	7.29	7.27
216428_x_at	killer cell immuno	KIR3DX1	3.79	3.97	3.72
206600_s_at	solute carrier fam	SLC16A5	5.87	5.68	5.49
208441_at	insulin-like growth	IGF1R	5.28	5.23	5.11
215212_at	---	---	4.91	5.36	4.81
202846_s_at	ralA binding prote	RALBP1	9.12	9.14	9.09
214858_at	similar to hCG177	PP14571	3.71	4.03	3.60
217781_s_at	zinc finger protein	ZFP106	9.28	9.33	9.49
212128_s_at	dystroglycan 1 (dy	DAG1	6.86	6.71	6.97
218370_s_at	S100P binding pro	S100PBP	9.05	9.04	9.16
214648_at	glutathione perox	GPX5	4.06	3.94	3.83
208047_s_at	NGFI-A binding pr	NAB1	5.41	5.07	5.63
200714_x_at	osteosarcoma am	OS9	9.17	9.31	9.14
220854_at	---	---	3.85	3.55	3.65
214437_s_at	serine hydroxyme	SHMT2	10.68	10.45	10.61
212114_at	ataxin 7-like 3B	ATXN7L3B	9.81	9.81	9.80
211788_s_at	three prime repai	TREX2	5.00	5.07	4.88
219809_at	WD repeat domai	WDR55	7.45	7.62	7.70
207736_s_at	transition protein	TNP2	4.40	4.36	4.24
206776_x_at	acrosomal vesicle	ACRV1	3.93	4.29	3.79
213894_at	thrombospondin,	THSD7A	3.55	3.80	3.31
215402_at	amyloid beta prec	APPBP2	3.72	3.33	3.48
210143_at	annexin A10	ANXA10	4.23	3.72	4.07
203498_at	regulator of calcin	RCAN2	4.02	3.99	3.79
204797_s_at	echinoderm micro	EML1	7.39	7.60	7.50
202402_s_at	cysteinyl-tRNA syn	CARS	10.34	10.06	10.48
207085_x_at	colony stimulating	CSF2RA	3.61	3.63	3.60
207864_at	sodium channel, v	SCN7A	3.59	3.31	3.52
211170_s_at	phosphodiesteras	PDE10A	4.02	3.95	3.75
216155_at	---	---	4.50	4.54	4.38
222144_at	kinesin family mer	KIF17	5.72	5.86	5.79
209147_s_at	phosphatidic acid	PPAP2A	10.03	9.99	10.04
205137_x_at	Usher syndrome 1	USH1C	5.39	5.60	5.49
203396_at	proteasome (pros	PSMA4	12.10	12.15	12.21
205210_at	transforming grov	TGFBRAP1	7.81	7.80	7.68
219004_s_at	chromosome 21 o	C21orf45	8.78	8.75	8.82
216919_at	tumor protein p53	TP53I11	5.62	5.90	5.67
218865_at	MOCO sulphurase	MOSC1	6.11	6.35	6.27
206358_at	protamine 1	PRM1	5.49	5.35	5.39
203782_s_at	polymerase (RNA)	POLRMT	8.50	8.34	8.49

216044_x_at	family with seque	FAM69A	8.20	8.47	8.38
218105_s_at	mitochondrial ribo	MRPL4	9.42	9.27	9.11
207391_s_at	phosphatidylinosi	PIP5K1A	9.06	8.75	9.03
217334_at	olfactory receptor	OR2J3	3.92	3.72	3.46
203626_s_at	S-phase kinase-as	SKP2	4.86	5.21	4.79
205009_at	trefoil factor 1	TFF1	5.44	5.60	4.99
213439_x_at	RUN domain cont	RUNDC3A	7.51	7.28	7.15
216700_at	Triple functional c	TRIO	6.24	6.57	5.93
201875_s_at	myelin protein zer	MPZL1	8.39	8.33	8.32
205194_at	phosphoserine ph	PSPH	8.95	8.70	9.07
217993_s_at	methionine adenc	MAT2B	11.20	11.30	11.35
202248_at	E2F transcription	E2F4	7.42	7.27	7.24
209235_at	chloride channel 7	CLCN7	8.62	8.66	8.44
206466_at	acyl-CoA syntheta	ACSBG1	3.63	3.24	3.28
213456_at	sclerostin domain	SOSTDC1	3.74	3.44	3.66
217227_x_at	immunoglobulin la	IGLV1-44 /// LOC1	6.03	5.98	5.70
203983_at	translin-associate	TSNAX	9.17	9.27	9.23
208041_at	G protein-couplec	GRK1	5.45	5.28	5.34
218782_s_at	ATPase family, AA	ATAD2	8.72	8.94	8.78
215893_x_at	---	---	4.49	4.40	4.31
206183_s_at	hect domain and I	HERC3	6.31	6.35	6.15
205651_x_at	Rap guanine nucle	RAPGEF4	3.25	3.27	3.09
219574_at	membrane-associ	1-Mar	3.48	3.74	3.51
217535_at	Family with seque	FAM49B	5.02	4.88	4.91
205706_s_at	ankyrin repeat do	ANKRD26	6.41	6.41	6.44
217031_at	keratin 84	KRT84	4.26	4.09	4.06
207853_s_at	synuclein, beta	SNCB	5.86	5.81	5.75
220214_at	zinc finger protein	ZNF215	8.02	7.97	8.04
214135_at	claudin 18	CLDN18	4.43	4.43	4.34
204762_s_at	guanine nucleotid	GNAO1	4.09	3.85	4.15
207453_s_at	DnaJ (Hsp40) hom	DNAJB5	4.51	4.58	4.48
207011_s_at	PTK7 protein tyro	PTK7	8.11	8.00	8.10
211494_s_at	solute carrier fam	SLC4A4	4.96	5.05	4.74
219422_at	espin	ESPN	7.21	7.08	6.85
204202_at	IQ motif containin	IQCE	6.37	6.47	6.48
208546_x_at	histone cluster 1,	HIST1H2BH	7.22	7.01	7.30
203146_s_at	gamma-aminobut	GABBR1	6.41	6.61	6.39
214764_at	ribosomal RNA pr	RRP15	7.22	7.19	7.41
214989_x_at	---	---	7.55	7.81	7.61
206983_at	chemokine (C-C m	CCR6	3.78	3.82	3.71
211721_s_at	zinc finger protein	ZNF551	5.90	5.72	5.94
207961_x_at	myosin, heavy cha	MYH11	5.94	5.96	5.86
209635_at	adaptor-related p	AP1S1	10.22	10.28	10.20
212493_s_at	SET domain conta	SETD2	9.47	9.51	9.46
210515_at	HNF1 homeobox A	HNF1A	7.74	7.76	7.35
214847_s_at	G-protein signalin	GPSM3	7.05	7.06	6.57
216708_x_at	---	---	5.83	5.79	5.69

212901_s_at	cleavage stimulati	CSTF2T	8.75	8.82	8.79
204781_s_at	Fas (TNF receptor	FAS	7.97	7.94	8.12
216535_at	cell adhesion mol	CADM3	5.17	5.05	4.80
215668_s_at	plexin B1	PLXNB1	6.51	6.32	6.26
216454_at	TRM1 tRNA meth	TRMT1	5.27	5.41	4.93
204480_s_at	chromosome 9 op	C9orf16	7.66	7.62	7.79
202977_s_at	CREB/ATF bZIP tra	CREBZF	5.97	5.80	5.78
204535_s_at	RE1-silencing tran	REST	7.27	7.42	7.68
219835_at	PR domain contai	PRDM8	6.58	6.17	6.23
212702_s_at	bicaudal D homol	BICD2	9.33	9.31	9.30
220400_at	vacuolar protein s	VPS13B	4.13	4.04	4.02
217463_s_at	chromosome 11 c	C11orf9	4.55	4.44	4.13
203703_s_at	tubulin tyrosine li	TTLL4	5.45	5.59	5.42
205964_at	zinc finger protein	ZNF426	6.88	6.81	6.91
214300_s_at	topoisomerase (D	TOP3A	4.79	4.68	4.87
217045_x_at	natural cytotoxicit	NCR2	7.01	7.12	7.16
207841_at	spindlin family, m	SPIN2A	4.86	4.67	4.49
214928_at	obscurin-like 1	OBSL1	5.28	5.57	5.21
201569_s_at	sorting and assem	SAMM50	10.47	10.36	10.43
201400_at	proteasome (pros	PSMB3	12.28	12.26	12.21
216131_at	FERM domain con	FRMD4B	3.77	3.62	3.68
37201_at	inter-alpha (globu	ITIH4	5.26	5.17	5.11
219017_at	ethanolamine kin	ETNK1	8.23	8.29	8.42
222151_s_at	centrosomal prot	CEP63	7.72	7.96	8.23
201573_s_at	eukaryotic transla	ETF1	10.94	10.83	10.96
218716_x_at	mitochondrial tra	MTO1	9.52	9.52	9.56
216888_at	LIM domain bindi	LDB3	7.27	6.99	6.92
205114_s_at	chemokine (C-C m	CCL3 /// CCL3L1 //	5.03	5.02	4.68
220926_s_at	ER degradation er	EDEM3	7.92	7.98	8.10
214949_at	---	---	9.65	9.71	9.77
213040_s_at	neuronal pentraxi	NPTXR	4.67	4.94	4.41
213588_x_at	ribosomal protein	RPL14	14.52	14.55	14.63
209513_s_at	hydroxysteroid de	HSDL2	9.37	9.40	9.55
215286_s_at	putative homeod	PHTF2	5.35	5.59	5.84
213001_at	angiopoietin-like	ANGPTL2	5.00	5.16	4.84
216604_s_at	solute carrier fam	SLC7A8	3.89	4.31	3.96
218063_s_at	CDC42 effector pr	CDC42EP4	5.86	6.23	5.70
218458_at	germ cell-less hon	GMCL1	8.29	8.32	8.51
219830_at	retinoic acid indu	RAI1	6.43	6.53	6.20
222089_s_at	chromosome 16 c	C16orf71	6.56	6.43	6.20
221417_x_at	sphingosine-1-ph	S1PR5	6.15	6.29	5.59
215148_s_at	amyloid beta (A4)	APBA3	7.62	7.58	7.78
210369_at	SWAP switching B	SWAP70	4.81	5.38	5.17
215724_at	phospholipase D1	PLD1	3.75	3.83	3.67
211968_s_at	heat shock protei	HSP90AA1	12.92	13.20	13.11
202921_s_at	ankyrin 2, neuron	ANK2	4.38	4.24	4.25
209345_s_at	phosphatidylinosi	PI4K2A	8.46	8.51	8.55

210615_at	neuropilin 1	NRP1	3.89	3.95	3.75
206297_at	chymotrypsin C (c	CTRC	4.91	4.88	4.79
211836_s_at	myelin oligodendr	MOG	4.05	3.85	4.03
213526_s_at	lin-37 homolog (C	LIN37	8.48	8.01	8.52
211948_x_at	HLA-B associated	BAT2L2	10.55	10.53	10.68
211198_s_at	inducible T-cell co	ICOSLG	6.98	6.79	6.74
217297_s_at	myosin IXB	MYO9B	6.01	6.05	5.81
218512_at	WD repeat domai	WDR12	11.41	11.48	11.48
208720_s_at	RNA binding moti	RBM39	11.78	11.82	11.82
216429_at	---	---	5.08	5.09	5.09
203238_s_at	Notch homolog 3	NOTCH3	7.05	6.66	6.86
206322_at	synapsin III	SYN3	4.98	5.06	5.03
206843_at	crystallin, beta A4	CRYBA4	4.91	4.86	4.84
205249_at	early growth resp	EGR2	4.49	4.68	4.52
215746_at	---	---	3.74	3.77	3.80
216620_s_at	Rho guanine nucle	ARHGEF10	10.13	10.13	10.07
210225_x_at	leukocyte immuno	LILRB3	6.62	6.67	6.49
209538_at	zinc finger protein	ZNF32	8.35	8.34	8.42
212111_at	syntaxin 12	STX12	8.97	9.25	9.13
220109_at	transferrin	TF	3.15	3.11	3.08
201691_s_at	tumor protein D5	TPD52	5.75	5.40	5.69
207613_s_at	calcium/calmodul	CAMK2A	6.46	6.38	6.41
217438_at	---	---	5.33	5.30	5.32
203964_at	N-myc (and STAT)	NMI	9.03	8.92	9.11
206093_x_at	tenascin XA pseuc	TNXA /// TNXB	7.18	7.10	7.08
210299_s_at	four and a half LIM	FHL1	3.48	3.70	3.41
215863_at	transferrin recept	TFR2	5.95	5.79	5.89
217849_s_at	CDC42 binding pro	CDC42BPB	7.58	7.57	7.68
209608_s_at	acetyl-CoA acetylt	ACAT2	10.42	10.41	10.52
211591_s_at	phosphodiesteras	PDE4A	4.56	4.42	4.58
204509_at	carbonic anhydras	CA12	6.55	6.58	6.39
214514_at	minichromosome	MCM3AP	3.86	3.51	3.67
201687_s_at	apoptosis inhibito	API5	10.02	10.20	10.18
202313_at	protein phosphata	PPP2R2A	10.96	10.90	10.98
207590_s_at	centromere prote	CENPI	7.00	7.45	6.91
208802_at	signal recognition	SRP72	9.78	9.83	10.13
220447_at	histamine recepto	HRH3	5.52	5.23	5.12
211677_x_at	cell adhesion mol	CADM3	6.96	7.06	6.85
217398_x_at	glyceraldehyde-3-	GAPDH	14.51	14.45	14.49
210089_s_at	laminin, alpha 4	LAMA4	5.20	4.74	5.01
221676_s_at	coronin, actin bin	CORO1C	10.71	10.89	10.71
220798_x_at	lipid phosphate p	LPPR3	6.69	6.59	6.50
215112_x_at	MCF.2 cell line de	MCF2L2	3.15	3.30	2.96
209290_s_at	nuclear factor I/B	NFIB	9.65	9.59	9.71
217784_at	YKT6 v-SNARE hor	YKT6	8.75	8.69	8.88
203490_at	E74-like factor 4 (	ELF4	7.63	7.52	7.62
221425_s_at	iron-sulfur cluster	ISCA1	8.43	8.40	8.54

215252_at	---	---	5.25	5.39	4.89
218555_at	anaphase promot	ANAPC2	6.36	6.30	6.11
209919_x_at	gamma-glutamylt	GGT1	7.21	7.11	6.71
211678_s_at	ring finger protein	RNF114	10.47	10.45	10.50
219130_at	coiled-coil domain	CCDC76	8.96	9.09	9.26
217955_at	BCL2-like 13 (apo	BCL2L13	9.22	9.06	9.33
202761_s_at	spectrin repeat cc	SYNE2	11.27	11.22	11.34
220133_at	odontogenic, ame	ODAM	4.03	3.61	3.66
205537_s_at	vav 2 guanine nuc	VAV2	5.09	4.81	4.54
215692_s_at	metallophosphoe:	MPPED2	3.74	3.79	3.78
220050_at	chromosome 9 op	C9orf9	6.07	6.38	6.20
207083_s_at	coiled-coil and C2	CC2D1A	5.59	5.48	5.31
220721_at	zinc finger protein	ZNF614	4.80	5.21	5.33
207696_at	fucosyltransferase	FUT9	3.18	3.05	3.14
204117_at	prolyl endopeptid	PREP	9.86	9.75	9.78
214529_at	thyroid stimulatin	TSHB	4.22	4.12	4.03
215327_at	---	---	5.75	5.53	5.40
213552_at	glucuronic acid ep	GLCE	7.45	7.78	7.69
214299_at	topoisomerase (D	TOP3A	6.92	6.79	6.88
218455_at	NFS1 nitrogen fixa	NFS1	8.11	8.26	8.10
212449_s_at	lysophospholipase	LYPLA1	11.09	11.15	11.47
212515_s_at	DEAD (Asp-Glu-Ala	DDX3X	9.26	9.32	9.29
214589_at	fibroblast growth	FGF12	4.01	3.70	3.82
222306_at	---	---	5.65	5.78	5.63
221877_at	immunity-related	IRGQ	8.45	8.35	8.08
211990_at	major histocompa	HLA-DPA1	4.40	4.35	4.29
203417_at	microfibrillar-asso	MFAP2	6.14	6.06	5.85
202755_s_at	glypican 1	GPC1	6.46	6.65	6.59
214290_s_at	histone cluster 2,	HIST2H2AA3 /// HI	9.31	9.01	9.32
213440_at	RAB1A, member F	RAB1A	8.84	8.95	8.92
220828_s_at	hypothetical prote	FLJ11292	3.62	3.66	3.85
221539_at	eukaryotic transla	EIF4EBP1	11.03	10.63	10.93
207737_at	---	---	3.34	3.50	3.17
202042_at	histidyl-tRNA synt	HARS	9.92	10.05	9.88
33736_at	stomatin (EPB72)-	STOML1	9.58	9.34	9.46
204216_s_at	zinc finger CCCH-t	ZC3H14	9.06	9.05	9.01
211038_s_at	ciliary rootlet coil	CROCCL1	9.89	9.94	10.03
AFFX-r2-Bs-phe-	---	---	9.23	9.04	8.96
207475_at	fatty acid binding	FABP2	5.14	4.91	5.12
220246_at	calcium/calmodul	CAMK1D	3.93	4.06	4.18
61874_at	chromosome 9 op	C9orf7	8.32	8.41	8.21
203761_at	Src-like-adaptor	SLA	6.03	6.00	5.87
212244_at	GRINL1A complex	GCOM1 /// GRINL1	10.41	10.40	10.58
202983_at	helicase-like trans	HLTF	9.62	9.77	9.87
222146_s_at	transcription facto	TCF4	7.33	7.46	7.57
202249_s_at	DDB1 and CUL4 as	DCAF8	7.84	7.62	7.70
211436_at	PRO1095	LOC100131298	3.15	3.14	3.13

208934_s_at	lectin, galactoside	LGALS8	8.43	8.48	8.49
206844_at	fructose-1,6-bispl	FBP2	5.89	5.82	5.25
208685_x_at	bromodomain cor	BRD2	10.82	10.79	10.84
32402_s_at	symplekin	SYMPK	7.41	7.29	7.27
208173_at	interferon, beta 1	IFNB1	4.22	3.85	4.16
215081_at	KIAA1024	KIAA1024	4.36	4.45	4.27
217929_s_at	KIAA0319-like	KIAA0319L	8.09	8.18	7.89
207652_s_at	chemokine-like re	CMKLR1	4.99	5.26	5.13
222009_at	cementum protei	CEMP1	6.21	5.73	6.10
221344_at	olfactory receptor	OR12D2	6.09	5.90	5.74
214827_at	par-6 partitioning	PARD6B	4.09	3.58	3.79
213267_at	dopey family men	DOPEY1	3.33	3.35	3.52
219035_s_at	ring finger protein	RNF34	10.06	10.00	9.95
216584_at	---	---	6.38	6.37	6.27
205375_at	MyoD family inhib	MDFI	7.18	6.98	6.77
218265_at	SECIS binding prot	SECISBP2	7.41	7.36	7.57
209659_s_at	cell division cycle	CDC16	11.02	10.99	10.99
214998_at	AP2 associated ki	AAK1	4.18	3.83	4.04
220455_at	solute carrier fam	SLC16A8	5.85	5.54	5.58
209868_s_at	RNA binding moti	RBMS1	9.38	9.50	9.50
206602_s_at	homeobox D3	HOXD3	4.79	4.66	4.69
205357_s_at	angiotensin II rece	AGTR1	3.59	3.52	3.54
215476_at	---	---	6.24	5.88	5.85
207511_s_at	chromosome 2 op	C2orf24	6.83	6.63	6.79
215918_s_at	spectrin, beta, no	SPTBN1	5.35	5.24	5.19
201725_at	cell division cycle	CDC123	11.09	11.14	11.19
221156_x_at	cell cycle progress	CCPG1	6.57	6.58	7.03
218560_s_at	jumonji domain co	JMJD4	7.87	7.62	7.59
205891_at	adenosine A2b re	ADORA2B	7.12	7.27	7.27
214603_at	melanoma antiger	MAGEA2 /// MAGI	10.82	10.88	11.01
37384_at	protein phosphata	PPM1F	8.32	8.51	8.39
216200_at	pleckstrin homolo	PLEKHM1	4.48	4.37	4.07
219140_s_at	retinol binding pr	RBP4	5.92	5.63	5.70
214748_at	NEDD4 binding pr	N4BP2L2	6.22	6.94	6.65
201271_s_at	RNA binding prote	RALY	9.20	9.09	8.98
207840_at	CD160 molecule	CD160	4.44	4.32	4.00
212538_at	dedicator of cytok	DOCK9	8.87	8.98	9.03
217189_s_at	Smg-7 homolog, r	SMG7	5.74	6.06	6.32
215766_at	Glutathione S-trar	GSTA1	6.01	6.07	5.61
221460_at	olfactory receptor	OR2C1	5.82	6.16	5.78
210431_at	alkaline phosphat	ALPPL2	6.46	6.30	6.47
220347_at	Smg-6 homolog, r	SMG6	4.74	4.86	4.44
203512_at	trafficking protein	TRAPPC3	9.50	9.60	9.54
220304_s_at	cyclic nucleotide g	CNGB3	3.93	4.23	4.12
201263_at	threonyl-tRNA syr	TARS	12.59	12.50	12.81
221926_s_at	interleukin 17 rec	IL17RC	6.10	6.21	5.85
216334_s_at	cytochrome P450	CYP2A7P1	5.15	5.05	4.78

213140_s_at	synovial sarcoma	SS18L1	7.81	8.08	8.19
212170_at	RNA binding moti	RBM12	7.54	7.78	7.56
204716_at	coiled-coil domair	CCDC6	8.45	8.32	8.42
221963_x_at	---	---	7.02	7.16	7.33
212130_x_at	eukaryotic transla	EIF1	13.61	13.34	13.56
205918_at	solute carrier fam	SLC4A3	5.97	6.21	5.87
218881_s_at	FOS-like antigen 2	FOSL2	6.89	6.88	6.92
205263_at	B-cell CLL/lympho	BCL10	9.88	9.94	10.07
221651_x_at	immunoglobulin k	IGK@ /// IGKC	6.94	7.22	6.75
204741_at	bicaudal D homol	BICD1	6.61	6.82	6.58
207654_x_at	down-regulator o	DR1	9.34	9.26	9.40
204812_at	ZW10, kinetochor	ZW10	8.52	8.70	8.48
207471_at	---	---	3.37	3.37	3.28
214634_at	---	---	6.83	6.61	6.81
213528_at	chromosome 1 op	C1orf156	8.34	8.32	8.52
205318_at	kinesin family me	KIF5A	4.12	4.00	3.97
215580_at	minichromosome	MCM3AP	6.24	6.25	5.70
214530_x_at	erythrocyte meml	EPB41	4.92	4.90	4.99
207629_s_at	Rho/Rac guanine	ARHGEF2	8.55	8.18	8.58
214927_at	integrin, beta-like	ITGBL1	4.84	4.84	4.96
215143_at	dpy-19-like 2 pseu	DPY19L2P2	4.54	4.41	4.49
203662_s_at	tropomodulin 1	TMOD1	6.12	6.18	6.12
200068_s_at	calnexin	CANX	12.79	12.77	12.81
71933_at	wingless-type MN	WNT6	7.60	7.56	7.43
218873_at	gon-4-like (C. eleg	GON4L	8.31	8.31	8.31
206292_s_at	sulfotransferase f	SULT2A1	4.30	4.44	4.26
221577_x_at	growth differentia	GDF15 /// LOC100	8.59	8.02	8.71
209755_at	nicotinamide nucl	NMNAT2	4.21	4.36	4.24
201365_at	ornithine decarbo	OAZ2	9.12	9.07	9.05
207564_x_at	O-linked N-acetyl	OGT	7.48	7.54	7.58
208573_s_at	olfactory receptor	OR2H2	4.98	4.35	4.53
211775_x_at	hypothetical MGC	MGC13053	3.06	3.02	3.29
214230_at	cell division cycle	CDC42	4.46	4.28	4.35
211650_x_at	immunoglobulin h	IGHA1 /// IGHD //	6.76	6.43	6.26
220801_s_at	hydroxyacid oxida	HAO2	6.03	6.15	5.95
219792_at	agmatine ureohyc	AGMAT	4.91	5.18	4.83
205560_at	proprotein conve	PCSK5	5.28	4.74	5.08
210291_s_at	zinc finger protein	ZNF174	6.90	6.93	6.65
219767_s_at	crystallin, zeta (q	CRYZL1	8.48	8.58	8.77
221427_s_at	cyclin L2	CCNL2	8.13	8.30	8.05
221835_at	deltex homolog 3	DTX3	5.37	5.27	5.14
203900_at	KIAA0467	KIAA0467	5.50	5.46	5.56
208482_at	somatostatin rece	SSTR1	5.35	5.26	5.11
213434_at	syntaxin 2	STX2	8.78	8.84	9.03
200032_s_at	ribosomal protein	RPL9	14.75	14.77	14.89
219914_at	endothelin conve	ECEL1	6.39	6.55	5.93
215200_x_at	---	---	7.17	7.17	7.11

212286_at	ankyrin repeat do	ANKRD12	6.78	7.26	7.26
213390_at	zinc finger CCCH-t	ZC3H4	10.54	10.47	10.57
211640_x_at	immunoglobulin h	IGHG1 /// IGHM //	4.32	4.10	4.13
1773_at	phospholipase D1	PLD1	5.74	5.77	5.85
214231_s_at	KIAA0564	KIAA0564	6.79	6.58	6.75
205235_s_at	kinesin family me	KIF20B	8.30	8.66	8.57
215953_at	DKFZP564C196 pr	DKFZP564C196	4.85	4.38	4.24
212857_x_at	SUB1 homolog (S.	SUB1	13.29	13.35	13.44
35626_at	N-sulfoglucosamir	SGSH	8.42	8.39	8.38
212326_at	vacuolar protein s	VPS13D	6.81	6.99	6.85
218175_at	coiled-coil domair	CCDC92	6.81	6.63	6.64
204296_at	dynactin 1 (p150,	DCTN1	3.96	3.94	3.92
213441_x_at	SAM pointed dom	SPDEF	7.34	7.49	7.39
214875_x_at	amyloid beta (A4)	APLP2	8.22	8.09	8.17
206905_s_at	matrilin 1, cartila	MATN1	6.55	6.39	5.92
206390_x_at	platelet factor 4	PF4	5.97	5.77	5.55
213983_s_at	PDS5, regulator o	PDS5A	8.36	8.27	8.32
213562_s_at	squalene epoxida	SQLE	7.88	7.95	8.00
206491_s_at	N-ethylmaleimide	NAPA	7.31	7.44	7.38
212993_at	NACC family mem	NACC2	8.54	8.74	8.61
207122_x_at	sulfotransferase f	SULT1A2	7.10	7.07	6.95
206822_s_at	l(3)mbt-like (Dros	L3MBTL	4.61	5.01	4.76
208457_at	gamma-aminobut	GABRD	5.46	5.77	5.81
217006_x_at	fatty acid synthas	FASN	7.75	7.54	7.51
218444_at	asparagine-linked	ALG12	6.24	6.42	6.06
202260_s_at	syntaxin binding p	STXBP1	8.73	9.00	8.94
221596_s_at	chromosome 7 op	C7orf64	6.72	6.73	6.99
210310_s_at	fibroblast growth	FGF5	5.56	5.78	5.49
213851_at	transmembrane p	TMEM110	7.34	7.37	7.30
217992_s_at	EF-hand domain f	EFHD2	9.38	9.58	9.47
211867_s_at	protocadherin alp	PCDHA10	7.47	7.09	7.17
206578_at	NK2 transcription	NKX2-5	8.73	8.57	8.37
220715_at	---	---	5.48	5.51	5.45
204733_at	kallikrein-related	KLK6	4.63	4.78	4.78
207995_s_at	C-type lectin dom	CLEC4M	4.62	4.75	4.39
213778_x_at	zinc finger proteir	ZNF276	7.48	7.16	7.43
204638_at	acid phosphatase	ACP5	5.86	5.90	5.81
216839_at	Laminin, alpha 2	LAMA2	3.31	3.29	3.26
220379_at	fascin homolog 3,	FSCN3	4.90	5.10	4.97
204622_x_at	nuclear receptor s	NR4A2	6.53	6.77	6.69
202578_s_at	DEAD (Asp-Glu-Al	DDX19A	7.55	7.82	7.35
220952_s_at	pleckstrin homolo	PLEKHA5	6.51	6.81	6.86
217819_at	golgin A7	GOLGA7	10.97	10.98	11.06
206246_at	6-phosphofructo-	PFKFB4	6.02	5.85	5.70
213785_at	importin 9	IPO9	7.53	7.74	7.53
215103_at	cytochrome P450	CYP2C18	3.79	3.54	3.50
211806_s_at	potassium inward	KCNJ15	4.93	4.98	4.83

218058_at	CXXC finger 1 (PHI	CXXC1	7.52	7.45	7.75
207034_s_at	GLI family zinc fing	GLI2	5.92	5.79	5.74
212938_at	collagen, type VI,	COL6A1	7.24	6.95	6.71
202684_s_at	RNA (guanine-7-)	RNMT	7.35	7.28	7.49
202014_at	protein phosphata	PPP1R15A	7.77	7.67	7.84
220397_at	Mdm1 nuclear pro	MDM1	5.98	5.92	6.17
212723_at	jumonji domain co	JMJD6	8.22	8.39	8.30
220527_at	mitochondrial ribo	MRPL20	4.39	4.02	4.01
220163_s_at	hairless homolog	HR	7.01	6.78	6.67
218916_at	zinc finger protein	ZNF768	7.86	7.39	7.66
210077_s_at	splicing factor, arg	SFRS5	4.28	3.97	3.86
210984_x_at	epidermal growth	EGFR	6.83	6.38	6.47
216751_at	---	---	5.43	5.46	5.49
217020_at	retinoic acid recep	RARB	5.03	4.74	4.55
214312_at	forkhead box A2	FOXA2	5.59	5.34	5.30
214463_x_at	histone cluster 1,	HIST1H4J	7.74	7.64	7.68
217513_at	chromosome 17 o	C17orf60	5.70	5.39	5.16
218636_s_at	mannosidase, alph	MAN1B1	8.50	8.62	8.54
201891_s_at	beta-2-microglobu	B2M	14.31	14.37	14.33
201785_at	ribonuclease, RNa	RNASE1	6.19	6.07	5.82
208090_s_at	autoimmune regu	AIRE	6.02	5.72	5.95
202251_at	PRP3 pre-mRNA p	PRPF3	7.76	7.81	7.89
206383_s_at	GTPase activating	G3BP2	7.02	7.15	6.96
219666_at	membrane-spann	MS4A6A	5.14	5.44	5.19
207548_at	glutamate recepto	GRM7	3.27	3.37	3.13
203151_at	microtubule-asso	MAP1A	6.17	6.47	6.05
212435_at	tripartite motif-co	TRIM33	8.62	8.89	8.97
215913_s_at	GULP, engulfment	GULP1	3.90	3.96	3.55
208374_s_at	capping protein (a	CAPZA1	12.22	12.28	12.23
208958_at	endoplasmic retic	ERP44	4.55	4.46	4.74
204566_at	protein phosphata	PPM1D	8.40	8.44	8.61
210758_at	PC4 and SFRS1 int	PSIP1	8.22	8.35	8.48
207888_at	---	---	3.47	3.85	3.82
210052_s_at	TPX2, microtubule	TPX2	10.90	10.89	10.95
212462_at	MYST histone ace	MYST4	9.79	9.85	9.93
206212_at	carboxypeptidase	CPA2	5.31	5.70	5.04
206490_at	discs, large (Drosc	DLGAP1	3.65	3.91	3.59
215033_at	transmembrane 4	TM4SF1	3.37	3.27	3.46
219042_at	leucine zipper, pu	LZTS1	5.14	5.23	4.81
215367_at	KIAA1614	KIAA1614	6.51	6.51	6.31
213976_at	CDKN1A interactir	CIZ1	4.50	4.16	4.24
213495_s_at	---	---	5.69	5.68	5.60
214418_at	cancer/testis anti	CT62	4.03	3.69	3.69
220434_at	aarF domain conta	ADCK4	4.39	4.48	4.43
213962_s_at	Ankyrin repeat an	ANKLE2	4.39	4.87	4.67
206556_at	clusterin-like 1 (re	CLUL1	3.89	3.73	3.74
210244_at	cathelicidin antim	CAMP	6.30	6.13	5.96

214768_x_at	---	---	5.56	5.56	5.42
211255_x_at	death effector do	DEDD	6.46	6.30	6.14
214479_at	GDNF family rece	GFRA3	6.00	5.69	5.51
201552_at	lysosomal-associa	LAMP1	11.43	11.37	11.50
214015_at	suppressor of cyt	SOCS7	5.47	5.39	5.75
216010_x_at	fucosyltransferase	FUT3	4.73	4.66	4.25
220182_at	solute carrier fam	SLC25A23	7.50	7.37	7.59
204928_s_at	solute carrier fam	SLC10A3	8.72	8.56	8.42
207081_s_at	phosphatidylinosi	PI4KA	8.41	8.34	8.38
220713_at	Family with seque	FAM116B	6.39	6.53	6.50
202557_at	heat shock protei	HSPA13	10.83	10.72	10.98
214316_x_at	---	---	8.74	8.59	8.78
211357_s_at	aldolase B, fructo	ALDOB	4.73	4.92	4.81
205469_s_at	interferon regulat	IRF5	7.30	7.41	7.18
218349_s_at	Zwilch, kinetocho	ZWILCH	9.39	9.52	9.54
219369_s_at	OTU domain, ubic	OTUB2	5.30	5.19	4.86
216520_s_at	tumor protein, tra	TPT1	14.84	14.82	14.87
201048_x_at	RAB6A, member R	RAB6A	6.11	6.11	5.82
205434_s_at	AP2 associated ki	AAK1	9.94	9.67	9.63
203183_s_at	SWI/SNF related,	SMARCD1	4.95	5.08	5.04
210103_s_at	forkhead box A2	FOXA2	6.58	6.59	6.35
217344_at	farnesyl diphosph	FDPS	5.70	5.61	5.79
206580_s_at	EGF-containing fib	EFEMP2	5.68	5.47	5.81
212491_s_at	DnaJ (Hsp40) hom	DNAJC8	9.73	9.78	9.87
200888_s_at	ribosomal protein	RPL23	13.91	13.93	14.06
219158_s_at	N(alpha)-acetyltra	NAA15	10.80	10.87	10.94
220288_at	myosin XVA	MYO15A	5.47	5.10	5.33
216076_at	l(3)mbt-like (Dros	L3MBTL	7.53	7.00	7.15
217343_at	pancreatic lipase-	PNLIPRP2	3.42	3.60	3.23
213152_s_at	splicing factor, arg	SFRS2B	8.76	8.75	8.87
219958_at	chromosome 20 o	C20orf46	4.15	4.26	3.85
216866_s_at	collagen, type XIV	COL14A1	5.32	5.26	5.13
215663_at	muscleblind-like (	MBNL1	3.62	3.62	3.31
209829_at	family with seque	FAM65B	8.62	8.68	8.80
218500_at	chromosome 8 op	C8orf55	7.09	7.12	7.03
201527_at	ATPase, H+ transp	ATP6V1F	10.10	10.13	10.19
206150_at	CD27 molecule	CD27	5.82	5.89	5.34
216952_s_at	lamin B2	LMNB2	10.70	10.68	10.68
207274_at	cholinergic recept	CHRNE	4.57	4.74	4.74
210832_x_at	prostaglandin E re	PTGER3	4.57	4.76	4.45
219587_at	tetratricopeptide	TTC12	6.79	7.06	7.03
218289_s_at	ubiquitin-like mod	UBA5	9.11	9.49	9.32
219644_at	coiled-coil domair	CCDC41	7.98	8.29	8.28
221406_s_at	chromosome 6 op	C6orf26	5.86	6.06	5.70
210689_at	claudin 14	CLDN14	6.06	5.92	5.80
201203_s_at	ribosome binding	RRBP1	6.16	5.85	5.92
213695_at	paraoxonase 3	PON3	4.66	5.06	4.52

202833_s_at	GRIP and coiled-c	GCC2	3.83	3.61	3.65
215082_at	ELOVL family men	ELOVL5	8.40	8.26	8.37
219507_at	arginine/serine-ri	RSRC1	9.28	9.28	9.35
208609_s_at	tenascin XB	TNXB	6.78	6.55	6.46
203405_at	proteasome (pros	PSMG1	10.73	10.75	10.90
201273_s_at	signal recognition	SRP9	13.24	13.36	13.46
215546_at	proteasome (pros	PSMD5	4.34	4.05	3.99
201176_s_at	archain 1	ARCN1	9.89	10.08	10.00
210686_x_at	solute carrier fam	SLC25A16	8.97	9.12	9.05
213803_at	Karyopherin (imp	KPNB1	10.93	11.00	11.06
222275_at	mitochondrial rib	MRPS30	9.17	9.10	9.24
220493_at	doublesex and ma	DMRT1	3.81	3.53	3.56
217284_x_at	serine hydrolase-l	SERHL2	5.33	5.24	5.00
211369_at	---	---	3.80	3.64	3.66
221338_at	lymphocyte antig	LY6G6E	4.27	4.51	4.47
218080_x_at	Fas (TNFRSF6) ass	FAF1	10.49	10.31	10.36
213290_at	collagen, type VI,	COL6A2	6.17	6.09	6.23
205852_at	cyclin-dependent	CDK5R2	5.75	5.75	5.36
222084_s_at	SET binding factor	SBF1	5.55	5.45	5.44
211698_at	EP300 interacting	EID1	8.21	8.42	8.47
218040_at	PRP38 pre-mRNA	PRPF38B	8.76	8.81	8.98
205557_at	bactericidal/perm	BPI	4.35	4.24	4.20
214067_at	chromosome 16 c	C16orf42	6.12	5.64	5.79
200932_s_at	dynactin 2 (p50)	DCTN2	10.64	10.53	10.77
212213_x_at	optic atrophy 1 (a	OPA1	9.48	9.60	9.60
201411_s_at	pleckstrin homolo	PLEKHB2	9.76	9.73	9.83
218713_at	NMDA receptor re	NARG2	8.77	8.93	9.19
210531_at	nuclear receptor s	NR2C1	4.98	5.28	5.32
200092_s_at	ribosomal protein	RPL37	14.98	14.96	15.03
222312_s_at	---	---	7.70	7.67	7.86
217170_at	---	---	4.99	4.93	4.78
214236_at	Similar to cell divi	LOC100286895	4.50	4.87	4.63
219623_at	ARP5 actin-relate	ACTR5	7.76	8.02	7.86
215134_at	phosphatidylinosi	PI4K2A	6.51	6.73	6.58
208940_at	selenophosphate	SEPHS1	9.85	9.80	9.78
220962_s_at	peptidyl arginine	PADI1	7.94	7.79	7.50
218461_at	GPN-loop GTPase	GPN3	9.98	9.97	10.02
217866_at	cleavage and poly	CPSF7	9.95	10.01	10.11
203944_x_at	butyrophilin, subf	BTN2A1	8.31	8.47	8.55
217292_at	myotubularin rela	MTMR7	3.48	3.51	3.28
203820_s_at	insulin-like growt	IGF2BP3	4.16	4.09	3.80
219878_s_at	Kruppel-like facto	KLF13	7.77	7.57	7.42
214335_at	ribosomal protein	RPL18	7.85	7.71	7.63
209325_s_at	regulator of G-pro	RGS16	6.36	5.84	6.22
207341_at	proteinase 3	PRTN3	3.77	3.92	3.97
212711_at	calmodulin regula	CAMSAP1	8.72	8.66	8.62
209975_at	cytochrome P450,	CYP2E1	4.67	4.68	4.70

210552_s_at	Ral GEF with PH d	RALGPS1	6.10	5.87	5.66
222253_s_at	POM121 membra	POM121L9P	4.74	4.64	4.25
211909_x_at	prostaglandin E re	PTGER3	3.78	3.82	3.99
208898_at	ATPase, H+ transp	ATP6V1D	10.95	11.06	11.10
206794_at	v-erb-a erythrobla	ERBB4	4.93	4.52	4.44
214841_at	cornichon homolo	CNIH3	4.79	4.44	4.50
212525_s_at	H2A histone famil	H2AFX	7.03	6.99	6.99
219370_at	reprimo, TP53 dep	RPRM	5.81	5.69	5.76
213909_at	leucine rich repea	LRRC15	4.51	4.69	4.44
207473_at	motilin	MLN	6.06	6.05	5.99
213321_at	branched chain ke	BCKDHB	7.92	8.05	7.87
214522_x_at	histone cluster 1,	HIST1H2AD /// HIS	6.18	5.80	6.05
220938_s_at	glucocorticoid mo	GMEB1	7.98	7.97	8.06
208571_at	acidic (leucine-ricl	ANP32A /// ANP32	3.54	3.61	3.25
210537_s_at	transcriptional ad	TADA2A	6.51	6.40	6.62
214745_at	phospholipase C,	PLCH1	4.05	3.98	3.73
221099_at	---	---	7.03	6.91	6.44
213320_at	protein arginine n	PRMT3	10.01	10.00	10.24
221173_at	Usher syndrome 1	USH1C	4.68	4.26	4.32
202683_s_at	RNA (guanine-7-)	RNMT	9.30	9.31	9.39
211584_s_at	nuclear protein, a	NPAT	6.64	6.60	6.46
217883_at	methylmalonic ac	MMADHC	11.86	11.96	12.03
209259_s_at	structural mainter	SMC3	10.27	10.30	10.61
204869_at	proprotein conver	PCSK2	3.57	3.53	3.14
203819_s_at	insulin-like growth	IGF2BP3	4.75	4.87	5.02
217007_s_at	ADAM metallopep	ADAM15	7.31	6.99	7.05
213731_s_at	---	---	6.34	6.47	6.11
200995_at	importin 7	IPO7	10.62	10.75	10.89
212814_at	adenosylhomocys	AHCYL2	7.03	7.05	7.15
219222_at	ribokinase	RBKS	7.52	7.42	7.33
213180_s_at	golgi SNAP recept	GOSR2	7.80	7.82	7.79
212028_at	RNA binding moti	RBM25	9.80	9.90	10.02
205932_s_at	msh homeobox 1	MSX1	7.58	7.64	7.80
211905_s_at	integrin, beta 4	ITGB4	4.99	4.69	4.79
202924_s_at	pleiomorphic ade	PLAGL2	5.92	5.97	5.83
214921_at	potassium large c	KCNMA1	4.11	3.60	3.49
208426_x_at	killer cell immuno	KIR2DL4 /// KIR2D	4.19	4.21	4.11
218913_s_at	GEM interacting p	GMIP	6.46	6.35	5.98
217571_at	---	---	3.65	3.37	3.53
217515_s_at	calcium channel, v	CACNA1S	6.16	5.91	5.45
207789_s_at	dipeptidyl-peptida	DPP6	4.72	4.54	4.38
220806_x_at	guanine nucleotid	GNG13	3.77	3.88	3.54
205420_at	peroxisomal bioge	PEX7	7.22	7.31	7.29
201665_x_at	ribosomal protein	RPS17	14.94	14.95	15.05
208762_at	SMT3 suppressor	SUMO1	7.59	7.73	7.98
36994_at	ATPase, H+ transp	ATP6V0C	10.39	10.44	10.28
215628_x_at	---	---	9.02	9.21	9.14

208588_at	apoptosis inhibito	FKSG2	5.03	4.76	5.11
213309_at	phospholipase C-l	PLCL2	6.88	6.92	6.79
204494_s_at	chromosome 15 c	C15orf39	7.02	6.94	6.92
204674_at	lymphoid-restricte	LRMP	6.63	6.62	6.04
221157_s_at	F-box protein 24	FBXO24	5.39	5.19	5.19
221891_x_at	heat shock 70kDa	HSPA8	12.66	12.87	12.80
201341_at	ectodermal-neura	ENC1	9.15	9.11	9.09
207833_s_at	holocarboxylase s	HLCS	6.59	6.48	6.55
222330_at	---	---	3.11	2.97	3.12
210649_s_at	AT rich interactive	ARID1A	6.24	6.40	6.32
210245_at	ATP-binding casse	ABCC8	5.92	6.07	6.02
214726_x_at	adducin 1 (alpha)	ADD1	7.18	7.12	7.04
218879_s_at	methenyltetrahyd	MTHFSD	6.23	6.23	6.46
205746_s_at	ADAM metallopep	ADAM17	7.35	7.17	7.33
219133_at	3-oxoacyl-ACP syr	OXSM	8.89	9.10	9.29
206072_at	urocortin	UCN	8.17	7.97	8.02
221287_at	ribonuclease L (2',	RNASEL	7.02	6.79	6.50
220209_at	peptide YY, 2 (sem	PYY2	6.37	6.11	5.85
200080_s_at	H3 histone, family	H3F3A /// LOC440	13.63	13.67	13.71
220380_at	deoxyribonucleas	DNASE2B	3.73	3.80	3.55
207087_x_at	ankyrin 1, erythro	ANK1	6.59	6.71	6.22
222120_at	zinc finger protein	ZNF764	6.60	6.48	6.67
217516_x_at	armadillo repeat g	ARVCF	8.05	8.14	8.11
213196_at	zinc finger protein	ZNF629	8.33	8.32	8.21
215072_x_at	chromosome 10 c	C10orf137	4.37	4.51	4.63
220796_x_at	solute carrier fam	SLC35E1	10.18	10.44	10.39
213663_s_at	similar to hCG198	LOC100288944	3.28	3.16	3.12
211116_at	solute carrier fam	SLC9A2	5.25	5.29	5.10
205437_at	zinc finger protein	ZNF211	6.34	6.49	6.50
210432_s_at	sodium channel, v	SCN3A	3.34	3.55	3.41
210620_s_at	general transcript	GTF3C2	6.67	6.79	6.74
216989_at	sperm adhesion m	SPAM1	3.72	3.83	3.72
204543_at	Rap guanine nucle	RAPGEF1	6.04	5.82	5.82
221990_at	paired box 8	PAX8	6.11	6.02	5.92
206817_x_at	CUGBP, Elav-like f	CELF3	7.52	7.42	7.69
214840_at	target of myb1-lik	TOM1L2	6.97	6.83	6.49
210982_s_at	major histocompa	HLA-DRA	5.49	5.22	5.21
37028_at	protein phosphata	PPP1R15A	8.16	8.05	8.18
206115_at	early growth resp	EGR3	3.62	3.74	3.65
217487_x_at	folate hydrolase (|	FOLH1	6.16	6.09	6.15
204229_at	solute carrier fam	SLC17A7	6.89	6.76	6.54
219522_at	four jointed box 1	FJX1	10.09	10.01	10.12
210562_at	growth regulation	GREB1	3.38	3.31	3.12
211163_s_at	tumor necrosis fa	TNFRSF10C	5.34	4.91	5.25
215191_at	---	---	5.34	5.37	5.17
205336_at	parvalbumin	PVALB	3.96	4.28	4.11
222322_at	---	---	3.74	3.51	3.71

220819_at	FERM domain con	FRMD1	6.48	6.71	6.76
220468_at	ADP-ribosylation f	ARL14	4.37	4.42	4.30
203848_at	A kinase (PRKA) ar	AKAP8	8.65	8.53	8.66
210474_s_at	cyclin-dependent	CDK11A /// CDK11	7.21	7.45	7.30
78383_at	similar to hCG181	LOC100129250	7.61	7.46	7.56
202401_s_at	serum response fa	SRF	8.33	8.25	8.49
208300_at	protein tyrosine p	PTPRH	7.18	6.85	7.18
210390_s_at	CCL14-CCL15 reac	CCL14-CCL15 /// C	3.26	3.35	3.36
216856_s_at	---	---	4.31	4.54	4.31
211768_at	linker for activatic	LAT2	6.32	6.47	6.11
219537_x_at	delta-like 3 (Drosc	DLL3	7.18	7.18	7.17
208277_at	paired-like homec	PITX3	7.22	6.94	6.73
41512_at	BRCA1 associated	BRAP	8.80	8.79	8.71
214194_at	DIS3 mitotic contr	DIS3	6.79	7.06	7.24
219429_at	fatty acid 2-hydro	FA2H	4.15	3.92	3.65
211898_s_at	EPH receptor B1	EPHB1	4.69	4.80	4.52
213911_s_at	H2A histone famil	H2AFZ	13.75	13.84	13.86
217877_s_at	GC-rich promoter	GPBP1L1	9.54	9.37	9.43
213580_at	---	---	3.60	3.31	3.07
217935_s_at	ubiquinol-cytochr	UQCC	8.97	8.98	8.92
206961_s_at	mediator complex	MED20	6.39	5.99	5.81
204512_at	human immunode	HIVEP1	8.13	8.07	8.21
206503_x_at	promyelocytic leu	PML	7.56	7.52	7.58
210212_x_at	mature T-cell prol	MTCP1NB	9.36	9.37	9.45
214283_at	transmembrane p	TMEM97	8.69	8.77	8.67
218404_at	sorting nexin 10	SNX10	7.77	8.08	8.11
204372_s_at	KH-type splicing re	KHSRP	10.03	10.05	10.06
217095_x_at	natural cytotoxicit	NCR1	6.54	6.33	6.52
207981_s_at	estrogen-related r	ESRRG	3.88	3.70	3.97
207913_at	cytochrome P450,	CYP2F1	3.68	3.73	3.37
217050_at	early lymphoid ac	EPAG	3.69	3.86	3.61
205947_s_at	vasoactive intestin	VIPR2	4.54	5.02	4.77
208694_at	protein kinase, DN	PRKDC	10.75	10.77	10.96
217772_s_at	mitochondrial car	MTCH2	11.11	11.00	11.13
219253_at	transmembrane p	TMEM185B	7.89	8.11	8.15
217376_at	similar to hCG201	LOC100289473 ///	5.74	5.71	5.70
212820_at	Dmx-like 2	DMXL2	7.79	7.89	8.12
210789_x_at	carcinoembryonic	CEACAM3	7.10	6.72	6.75
202187_s_at	protein phosphata	PPP2R5A	9.49	9.57	9.48
213270_at	membrane protei	MPP2	6.59	6.40	6.40
219365_s_at	CaM kinase-like ve	CAMKV	6.56	6.35	6.25
202115_s_at	nucleolar complex	NOC2L	8.82	8.89	8.95
201813_s_at	TBC1 domain fam	TBC1D5	9.46	9.43	9.45
214504_at	ABO blood group	ABO	6.06	6.25	5.77
210580_x_at	sulfotransferase fa	SULT1A3 /// SULT1	7.47	7.65	7.53
213465_s_at	protein phosphata	PPP1R7	10.49	10.39	10.44
220398_at	N-acetylglucosam	GNPTAB	6.67	6.47	6.27

213067_at	myosin, heavy cha	MYH10	7.41	7.57	7.52
209183_s_at	chromosome 10 c	C10orf10	7.24	7.09	7.33
218992_at	chromosome 9 op	C9orf46	8.52	8.48	8.57
207329_at	matrix metallopep	MMP8	3.78	3.99	3.75
205048_s_at	phosphoserine ph	PSPH	7.45	7.41	7.70
210085_s_at	annexin A9	ANXA9	5.05	4.94	5.16
31799_at	---	---	6.68	6.94	6.77
35150_at	CD40 molecule, TI	CD40	7.80	8.04	7.95
219699_at	leucine-rich repea	LGI2	6.19	6.16	5.86
212772_s_at	ATP-binding casse	ABCA2	7.79	7.49	7.52
222248_s_at	sirtuin (silent mat	SIRT4	4.09	4.21	4.10
207300_s_at	coagulation facto	F7	4.94	4.88	4.92
210372_s_at	tumor protein D5	TPD52L1	7.30	7.25	7.34
211810_s_at	galactosylceramid	GALC	4.94	4.55	4.72
216855_s_at	heterogeneous nu	HNRNPU	6.89	6.93	7.00
216316_x_at	glycerol kinase ///	GK /// GK3P	4.79	4.65	4.83
208015_at	SMAD family men	SMAD1	5.80	6.15	5.92
206725_x_at	bone morphogene	BMP1	7.94	7.83	7.58
211644_x_at	immunoglobulin k	IGK@ /// IGKC ///	5.73	5.60	5.69
220752_at	erythrocyte trans	LOC51145	3.47	3.74	3.43
221856_s_at	family with seque	FAM63A	7.78	7.75	7.58
214480_at	ets variant 3	ETV3	4.92	4.60	4.78
205877_s_at	zinc finger CCCH-t	ZC3H7B	9.20	8.95	8.98
216648_s_at	hypothetical prote	LOC100288758	4.51	4.30	4.23
203631_s_at	G protein-couplec	GPRC5B	5.84	5.59	5.61
212728_at	discs, large homol	DLG3	6.57	6.57	6.35
202194_at	transmembrane e	TMED5	10.58	10.63	10.77
219828_at	chromosome 9 op	C9orf86	8.15	8.18	8.34
211465_x_at	fucosyltransferase	FUT6	7.42	7.43	7.30
205815_at	regenerating islet-	REG3A	4.01	4.10	4.28
210206_s_at	DEAD/H (Asp-Glu-	DDX11	5.21	5.25	5.23
36004_at	inhibitor of kappa	IKBKG	8.11	7.93	8.08
202932_at	v-yes-1 Yamaguch	YES1	9.44	9.60	9.71
220700_at	---	---	3.20	2.98	3.04
202471_s_at	isocitrate dehydro	IDH3G	8.94	8.95	8.93
203235_at	thimet oligopeptio	THOP1	7.97	7.89	7.94
211922_s_at	catalase	CAT	8.02	8.28	8.18
204217_s_at	reticulon 2	RTN2	5.81	5.80	5.73
205878_at	POU class 6 home	POU6F1	6.77	6.58	6.83
218998_at	chromosome 9 op	C9orf6	8.81	8.89	8.95
206875_s_at	STE20-like kinase	SLK	9.75	9.99	10.01
65588_at	hypothetical LOC3	LOC388796	10.42	10.11	10.39
211844_s_at	neuropilin 2	NRP2	4.50	4.67	4.70
200780_x_at	GNAS complex loc	GNAS	13.27	13.20	13.17
208336_s_at	trans-2,3-enoyl-Co	TECR	9.26	9.23	9.19
220360_at	THAP domain con	THAP9	4.01	3.97	3.72
214294_at	hypothetical LOC5	KIAA0485	6.19	5.99	6.02

208119_s_at	zinc finger protein	ZNF93	5.11	5.07	5.03
216669_at	---	---	3.77	3.40	3.70
217581_at	---	---	5.55	5.76	5.44
213622_at	collagen, type IX, a	COL9A2	6.78	6.69	6.31
203731_s_at	zinc finger with KR	ZKSCAN5	7.67	7.79	7.32
209733_at	midline 2	MID2	4.21	4.35	3.93
208739_x_at	SMT3 suppressor	SUMO2	12.01	12.05	12.07
204179_at	myoglobin	MB	5.36	5.28	5.12
201073_s_at	SWI/SNF related,	SMARCC1	8.51	8.65	8.53
215735_s_at	tuberous sclerosis	TSC2	7.01	6.94	6.77
206926_s_at	interleukin 11	IL11	5.56	5.45	5.57
208165_s_at	protease, serine, 1	PRSS16	5.16	5.31	4.92
209544_at	receptor-interacti	RIPK2	6.94	6.96	7.18
221076_at	---	---	3.56	3.43	3.35
216035_x_at	transcription facto	TCF7L2	8.38	8.35	8.81
205360_at	---	---	6.74	6.77	6.37
218228_s_at	tankyrase, TRF1-in	TNKS2	7.98	7.80	8.03
206119_at	betaine--homocys	BHMT	4.89	4.88	4.98
209747_at	transforming grov	TGFB3	6.27	6.31	6.20
221767_x_at	high density lipop	HDLBP	10.53	10.46	10.40
209932_s_at	deoxyuridine tripl	DUT	12.11	12.19	12.13
216995_x_at	makorin ring finge	MKRN2	6.12	5.93	5.65
205758_at	CD8a molecule	CD8A	4.78	4.68	5.08
214564_s_at	protocadherin gar	PCDHGC3	4.08	3.90	3.63
221800_s_at	chromosome 17 o	C17orf70	8.30	8.08	8.14
221079_s_at	methyltransferase	METTL2A /// MET	8.24	8.38	8.46
210456_at	phosphate cytidyl	PCYT1B	6.29	5.96	5.78
209704_at	metal response el	MTF2	6.56	6.83	7.02
205702_at	putative homeodo	PHTF1	7.21	7.55	7.50
200837_at	B-cell receptor-as	BCAP31	10.20	10.01	10.34
218043_s_at	5-azacytidine indu	AZI2	7.36	7.36	7.69
202982_s_at	acyl-CoA thioester	ACOT1 /// ACOT2	8.80	8.67	8.89
218376_s_at	microtubule assoc	MICAL1	7.50	7.56	7.74
214339_s_at	mitogen-activated	MAP4K1	6.37	6.62	6.19
219960_s_at	ubiquitin carboxyl	UCHL5	10.33	10.44	10.52
221038_at	---	---	5.15	5.33	5.22
205142_x_at	ATP-binding casse	ABCD1	6.71	6.46	6.53
211421_s_at	ret proto-oncogen	RET	3.45	3.66	3.59
211015_s_at	heat shock 70kDa	HSPA4	10.58	10.62	10.76
219836_at	zinc finger, BED-ty	ZBED2	3.80	4.47	3.91
221601_s_at	Fas apoptotic inhi	FAIM3	6.33	6.28	5.87
203937_s_at	TATA box binding	TAF1C	7.03	6.69	6.67
200083_at	ubiquitin specific	USP22	10.24	10.19	10.26
210634_at	kelch-like 20 (Dros	KLHL20	4.59	4.44	4.21
215762_at	---	---	3.91	3.93	4.27
213131_at	olfactomedin 1	OLFM1	4.37	4.35	3.79
218260_at	DET1 and DDB1 as	DDA1	7.52	7.35	7.47

214563_at	protocadherin gar	PCDHGC3	4.67	4.95	4.60
209630_s_at	F-box and WD rep	FBXW2	9.22	9.32	9.47
203725_at	growth arrest and	GADD45A	13.06	12.94	13.33
221010_s_at	sirtuin (silent mat	SIRT5	6.30	6.68	6.38
209686_at	S100 calcium bind	S100B	3.82	3.43	3.90
219633_at	tocopherol (alpha	TTPAL	8.54	8.52	8.52
215758_x_at	zinc finger proteir	ZNF93	4.42	4.49	4.43
212007_at	UBX domain prote	UBXN4	10.81	10.91	10.82
216343_at	protocadherin gar	PCDHGA3	5.76	6.01	5.21
202700_s_at	transmembrane p	TMEM63A	7.37	7.20	6.99
201322_at	ATP synthase, H+	ATP5B	13.04	12.99	13.04
214326_x_at	jun D proto-oncog	JUND	4.99	4.88	4.71
215609_at	---	---	6.22	6.32	6.30
218639_s_at	ZXD family zinc fir	ZXDC	7.96	8.00	7.90
216217_at	phospholipase C-l	PLCL2	5.00	4.84	4.56
212486_s_at	FYN oncogene rel	FYN	7.04	6.80	7.01
203707_at	zinc finger proteir	ZNF263	8.95	8.88	8.93
218147_s_at	glycosyltransferas	GLT8D1	9.02	9.13	9.41
215555_at	---	---	4.09	4.27	4.52
218151_x_at	G protein-couplec	GPR172A	8.98	8.87	8.89
206248_at	protein kinase C, e	PRKCE	5.63	5.75	5.72
217950_at	nitric oxide syntha	NOSIP	10.20	10.03	10.14
221437_s_at	mitochondrial ribo	MRPS15	10.08	10.18	10.24
220294_at	potassium channe	KCNV1	3.43	3.25	3.22
65133_i_at	INO80 complex su	INO80B	5.08	4.80	5.04
221893_s_at	aarF domain cont	ADCK2	7.89	7.92	8.09
206862_at	zinc finger proteir	ZNF254	3.51	3.23	3.36
203085_s_at	transforming grov	TGFB1	7.48	7.30	7.27
202722_s_at	glutamine--fructo	GFPT1	9.19	8.78	9.36
209193_at	pim-1 oncogene	PIM1	7.86	7.82	7.68
215431_at	syntrophin, beta 1	SNTB1	4.98	4.92	4.79
210761_s_at	growth factor rec	GRB7	5.28	5.44	5.21
207799_x_at	armadillo repeat c	ARMCX4	5.05	4.65	4.65
216337_at	---	---	5.00	4.93	4.94
210079_x_at	potassium voltage	KCNAB1	5.67	5.72	5.54
203376_at	cell division cycle	CDC40	9.65	9.80	9.86
206527_at	4-aminobutyrate a	ABAT	4.88	4.78	4.59
201629_s_at	acid phosphatase	ACP1	10.03	10.06	10.19
203648_at	TatD DNase doma	TATDN2	9.25	9.31	9.28
219883_at	chromosome 11 c	C11orf20	6.29	6.33	5.98
210002_at	GATA binding pro	GATA6	9.46	9.28	9.68
210687_at	carnitine palmitoy	CPT1A	3.72	3.72	3.72
209649_at	signal transducing	STAM2	8.46	8.60	8.81
218885_s_at	UDP-N-acetyl-alpl	GALNT12	3.51	3.56	3.62
220145_at	microtubule-asso	MAP9	6.72	6.92	7.27
203329_at	protein tyrosine p	PTPRM	9.18	9.09	9.15
219791_s_at	Nbla00301	NBLA00301	4.75	4.57	4.35

214345_at	---	---	5.30	5.33	5.51
208434_at	MDS1 and EVI1 cc	MECOM	3.88	3.91	3.76
209384_at	proline synthetase	PROSC	9.70	9.86	9.89
210709_at	---	---	3.21	3.29	3.36
216923_at	cyclin-dependent	CDKL5	5.10	5.06	4.95
221648_s_at	---	---	3.75	3.70	3.47
211069_s_at	SMT3 suppressor	SUMO1	11.33	11.45	11.47
208591_s_at	phosphodiesteras	PDE3B	4.47	4.90	4.76
202120_x_at	adaptor-related p	AP2S1	10.56	10.53	10.58
211873_s_at	protocadherin gar	PCDHGA9	7.28	7.05	6.63
203258_at	DR1-associated pr	DRAP1	8.23	8.25	8.35
206375_s_at	heat shock 27kDa	HSPB3	5.05	4.93	4.74
214314_s_at	eukaryotic transla	EIF5B	8.38	8.58	8.56
222346_at	laminin, alpha 1	LAMA1	5.77	5.43	5.45
211588_s_at	similar to promye	LOC652346 /// PM	5.95	6.06	5.85
220693_at	coiled-coil domair	CCDC82	5.04	5.15	4.87
217133_x_at	cytochrome P450,	CYP2B6	4.20	3.98	3.74
222329_x_at	---	---	6.64	6.81	6.83
208398_s_at	TBP-like 1	TBPL1	9.47	9.39	9.64
216482_x_at	zinc finger proteir	ZNF79	4.17	4.01	4.27
217847_s_at	thyroid hormone	THRAP3	6.84	7.43	7.17
203555_at	protein tyrosine p	PTPN18	6.75	7.05	6.78
204702_s_at	nuclear factor (ery	NFE2L3	4.11	3.95	4.38
201005_at	CD9 molecule	CD9	10.60	10.74	10.62
53912_at	sorting nexin 11	SNX11	8.27	8.18	8.35
210895_s_at	CD86 molecule	CD86	3.88	4.18	3.91
205687_at	ubiquitin family do	UBFD1	7.70	7.74	7.82
208318_s_at	xylulokinase homo	XYLB	4.80	4.89	4.68
216473_x_at	double homeobox	DUX4 /// LOC4410	8.42	8.05	8.23
213615_at	Lysophosphatidylc	LPCAT3	6.76	6.76	6.82
216079_at	epilepsy, progress	EPM2A	5.17	5.35	5.37
206087_x_at	hemochromatosis	HFE	6.67	6.64	6.72
204407_at	transcription term	TTF2	8.46	8.51	8.63
214930_at	SLIT and NTRK-like	SLITRK5	4.14	4.01	4.18
206510_at	SIX homeobox 2	SIX2	6.32	6.62	6.20
209749_s_at	angiotensin I conv	ACE	4.65	4.13	4.12
214293_at	Septin 11	11-Sep	7.11	7.16	7.40
215737_x_at	upstream transcri	USF2	8.91	8.67	8.57
222017_x_at	leucine-rich repea	LRCH4	7.29	7.16	7.02
219333_s_at	calpain 10	CAPN10	6.71	6.78	6.60
209819_at	hyaluronan bindir	HABP4	4.83	4.89	4.62
216618_at	---	---	3.25	3.33	3.23
203118_at	proprotein conver	PCSK7	7.22	7.51	7.34
221454_at	BCL2-related ovar	BOK	5.88	5.41	5.43
37872_at	jerky homolog (m	JRK	7.22	7.45	7.10
202830_s_at	vesicle-associated	VAMP7	9.74	9.52	9.61
207309_at	nitric oxide syntha	NOS1	6.50	6.77	6.48

214412_at	H2A histone famil	H2AFB1 /// H2AFB	6.61	6.57	6.36
203852_s_at	survival of motor	SMN1 /// SMN2	9.14	9.16	9.32
221007_s_at	FIP1 like 1 (S. cere	FIP1L1	9.12	8.98	9.24
220038_at	chromosome 8 op	C8orf44 /// SGK3	5.09	5.26	5.47
208305_at	progesterone rece	PGR	3.63	3.70	3.55
215595_x_at	---	---	6.49	6.62	6.53
212383_at	ATPase, H+ transp	ATP6V0A1	8.14	8.20	8.01
209791_at	peptidyl arginine	PADI2	6.45	6.22	6.12
215021_s_at	neurexin 3	NRXN3	6.35	6.31	6.08
207684_at	T-box 6	TBX6	7.37	7.06	6.90
203305_at	coagulation factor	F13A1	3.87	4.26	3.81
209857_s_at	sphingosine kinas	SPHK2	7.15	7.03	6.87
217614_at	---	---	4.69	4.27	4.19
202882_x_at	---	---	11.59	11.64	11.75
208744_x_at	heat shock 105kD	HSPH1	10.00	10.05	10.04
213910_at	insulin-like growth	IGFBP7	3.24	3.56	3.35
215347_at	---	---	4.58	4.23	4.33
221665_s_at	EPS8-like 1	EPS8L1	7.16	6.97	6.85
220426_at	chromosome 20 c	C20orf195	5.51	5.56	5.28
217828_at	SAFB-like, transcri	SLTM	9.86	9.97	10.04
210037_s_at	nitric oxide syntha	NOS2	5.78	5.42	5.46
202025_x_at	acetyl-CoA acyltra	ACAA1	8.40	8.45	8.45
210440_s_at	CDC14 cell divisio	CDC14A	4.04	4.09	4.08
216575_at	---	---	3.34	3.54	3.48
209268_at	vacuolar protein s	VPS45	8.56	8.82	8.81
208927_at	speckle-type POZ	SPOP	10.03	9.93	10.07
218186_at	RAB25, member R	RAB25	5.06	5.07	4.82
206966_s_at	Kruppel-like facto	KLF12	5.54	6.02	5.86
213288_at	membrane bound	MBOAT2	7.73	7.82	7.75
215003_at	DiGeorge syndron	DGCR9	4.90	5.03	4.76
206643_at	histidine ammonia	HAL	4.03	3.93	3.91
204678_s_at	potassium channe	KCNK1	7.40	7.50	7.48
216460_at	FLJ00049 protein	FLJ00049	6.86	6.58	6.33
60084_at	cylindromatosis (t	CYLD	4.64	4.27	4.28
212765_at	calmodulin regula	CAMSAP1L1	10.87	10.80	10.87
220836_at	zinc finger protein	ZNF407	3.45	3.15	3.27
208803_s_at	signal recognition	SRP72	9.04	9.01	9.19
219612_s_at	fibrinogen gamma	FGG	5.38	5.19	5.09
201146_at	nuclear factor (er	NFE2L2	11.69	11.63	11.99
215845_x_at	---	---	5.76	5.86	5.51
76897_s_at	FK506 binding pro	FKBP15	6.33	6.02	6.04
206471_s_at	plexin C1	PLXNC1	3.59	3.70	3.36
208528_x_at	synovial sarcoma,	SSX5	6.25	6.12	5.60
210784_x_at	leukocyte immuno	LILRB3	6.92	6.46	6.38
219420_s_at	chromosome 1 op	C1orf163	9.01	9.11	9.02
207080_s_at	peptide YY	PYY	4.24	4.18	3.89
217467_at	---	---	5.68	5.60	5.93

217559_at	ribosomal protein	RPL10L	7.08	6.99	6.93
216400_at	glucosidase, beta,	GBA /// GBAP1	5.41	5.51	4.89
216851_at	hypothetical prote	LOC100287927	5.74	5.56	5.59
207127_s_at	heterogeneous nu	HNRNPH3	10.00	10.05	10.11
208503_s_at	GATA zinc finger c	GATAD1	9.21	9.17	9.23
214746_s_at	zinc finger protein	ZNF467	5.22	5.21	4.91
211359_s_at	opioid receptor, n	OPRM1	3.78	4.16	4.04
210042_s_at	cathepsin Z	CTSZ	9.20	9.03	9.06
212994_at	THO complex 2	THOC2	9.25	9.48	9.23
205295_at	creatine kinase, m	CKMT2	5.26	4.94	4.71
200014_s_at	heterogeneous nu	HNRNPC	12.07	12.12	12.14
208202_s_at	PHD finger protein	PHF15	6.91	6.67	6.93
202063_s_at	sel-1 suppressor c	SEL1L	4.69	4.29	4.47
200808_s_at	zyxin	ZYX	7.61	7.51	7.50
210915_x_at	T cell receptor bet	TRBC1	5.02	4.96	4.81
212433_x_at	ribosomal protein	RPS2	15.01	14.98	14.97
211140_s_at	caspase 2, apopto	CASP2	6.40	6.66	6.33
216049_at	Rho-related BTB d	RHOBTB3	3.90	3.71	3.67
220913_at	---	---	4.04	4.09	3.74
204785_x_at	interferon (alpha,	IFNAR2	8.42	8.52	8.30
210571_s_at	cytidine monopho	CMAH	3.93	3.45	3.94
209208_at	mannose-P-dolich	MPDU1	8.38	8.39	8.45
207706_at	Usher syndrome 2	USH2A	3.49	3.45	3.41
217394_at	---	---	3.26	3.30	3.58
204856_at	UDP-GlcNAc:beta	B3GNT3	7.68	7.35	7.37
211043_s_at	clathrin, light chai	CLTB	9.90	9.90	9.78
208979_at	nuclear receptor c	NCOA6	9.87	9.87	10.01
212859_x_at	metallothionein 1	MT1E	13.30	13.34	13.17
220905_at	---	---	7.26	7.15	7.13
218167_at	archaelysin family	AMZ2	10.80	10.78	10.81
221701_s_at	stimulated by reti	STRA6	5.70	5.87	5.54
208366_at	protocadherin 11	PCDH11X	4.25	4.00	4.04
222200_s_at	BSD domain conta	BSDC1	7.56	7.30	7.30
217642_at	ring finger protein	RNF40	5.72	5.74	5.67
203424_s_at	insulin-like growth	IGFBP5	5.16	5.35	4.88
34406_at	phosphofurin acid	PACS2	7.66	7.50	7.47
202097_at	nucleoporin 153kD	NUP153	10.71	10.60	10.84
221998_s_at	vaccinia related ki	VRK3	8.33	8.33	8.30
211885_x_at	fucosyltransferase	FUT6	4.30	4.71	3.96
205619_s_at	mesenchyme hom	MEOX1	5.12	4.60	4.86
211175_at	G protein-couplec	GPR45	5.69	5.44	5.55
212898_at	KIAA0406	KIAA0406	8.03	7.95	8.18
220307_at	CD244 molecule, r	CD244	4.90	5.02	4.76
219639_x_at	poly (ADP-ribose)	PARP6	9.01	8.98	9.06
216629_at	serine/arginine re	SRRM2	4.51	4.11	4.53
200664_s_at	DnaJ (Hsp40) hom	DNAJB1	7.72	7.65	7.73
210831_s_at	prostaglandin E re	PTGER3	4.02	4.27	4.01

220887_at	chromosome 14 c	C14orf162	4.63	4.79	4.34
210928_at	PRO1454	LOC100131613	3.28	3.15	3.29
221764_at	chromosome 19 c	C19orf22	7.69	7.62	7.69
215307_at	zinc finger proteir	ZNF529	3.69	3.81	3.47
209520_s_at	nuclear cap bindir	NCBP1	9.42	9.47	9.65
208378_x_at	fibroblast growth	FGF5	4.27	4.72	4.34
215311_at	neurotrophic tyro	NTRK3	4.17	3.77	3.61
219169_s_at	transcription facto	TFB1M	9.62	9.58	9.75
221232_s_at	ankyrin repeat do	ANKRD2	5.99	5.56	5.74
209504_s_at	pleckstrin homolo	PLEKHB1	4.09	3.66	3.93
214184_at	neuropeptide FF-a	NPFF	5.01	5.18	5.00
216543_at	unknown transcrip	LOC100093698	3.34	3.71	3.33
219872_at	family with seque	FAM198B	3.45	3.58	3.33
210635_s_at	kelch-like 20 (Dro:	KLHL20	6.05	5.92	5.87
213704_at	Rab geranylgeranу	RABGGTB	9.23	9.30	9.46
203872_at	actin, alpha 1, ske	ACTA1	3.99	4.32	4.43
208005_at	netrin 1	NTN1	4.23	4.19	4.00
214257_s_at	SEC22 vesicle traf	SEC22B	8.72	8.75	8.96
205106_at	mature T-cell prol	MTCP1	5.97	5.98	5.94
205186_at	dynein, axonemal	DNALI1	5.29	4.86	4.88
200666_s_at	DnaJ (Hsp40) hom	DNAJB1	8.61	8.80	8.75
200599_s_at	heat shock proteir	HSP90B1	12.63	12.74	12.75
207447_s_at	mannosyl (alpha-1	MGAT4C	3.31	3.25	3.28
211521_s_at	cytohesin 4	CYTH4	6.46	6.38	5.85
216229_x_at	HLA complex grou	HCG2P7	5.27	5.36	5.45
221710_x_at	family with seque	FAM176B	7.31	7.35	7.15
214027_x_at	desmin /// family	DES /// FAM48A	8.10	7.65	7.77
213549_at	PDZ domain conta	PDZD8	8.89	9.00	9.22
216291_at	Zinc finger proteir	ZNF440	3.93	3.74	3.87
203332_s_at	inositol polyphosp	INPP5D	5.31	5.38	5.07
208601_s_at	tubulin, beta 1	TUBB1	4.63	4.53	4.46
209194_at	centrin, EF-hand p	CETN2	9.83	9.79	9.77
215070_x_at	RAB GTPase activa	RABGAP1	4.53	4.51	4.53
219719_at	HIG1 hypoxia indu	HIGD1B	6.79	6.56	6.50
209789_at	coronin, actin bin	CORO2B	3.56	3.48	3.48
47608_at	tight junction asso	TJAP1	9.72	9.60	9.67
213041_s_at	ATP synthase, H+	ATP5D	10.39	10.32	10.34
216820_at	---	---	4.96	5.05	5.07
215158_s_at	death effector do	DEDD	8.46	8.60	8.54
218076_s_at	Rho GTPase activa	ARHGAP17	9.16	9.15	9.01
206334_at	lipase, gastric	LIPF	3.95	3.99	3.77
205926_at	interleukin 27 rec	IL27RA	6.41	6.68	6.68
202694_at	serine/threonine k	STK17A	5.83	6.04	6.16
218556_at	ORM1-like 2 (S. ce	ORMDL2	10.36	10.40	10.36
211071_s_at	myeloid/lymphoid	MLLT11	10.25	10.39	10.29
208169_s_at	prostaglandin E re	PTGER3	3.95	4.12	3.83
209958_s_at	Bardet-Biedl synd	BBS9	6.61	6.65	6.83

205545_x_at	DnaJ (Hsp40) hom	DNAJC8	11.11	11.13	11.00
217146_at	jerky homolog (m	JRK	4.39	4.37	4.08
218396_at	vacuolar protein s	VPS13C	9.65	9.68	9.90
207587_at	crystallin, gamma	CRYGA	4.76	4.76	4.52
220718_at	chromosome 15 c	C15orf34	3.76	3.65	3.52
206604_at	ovo-like 1(Drosop	OVOL1	6.33	6.35	6.04
212854_x_at	neuroblastoma br	NBPF10	11.25	11.28	11.27
214582_at	phosphodiesteras	PDE3B	6.39	6.44	6.43
214398_s_at	inhibitor of kappa	IKBKE	6.71	6.53	6.35
200085_s_at	transcription elon	TCEB2	12.84	12.77	12.75
216706_x_at	Immunoglobulin h	IGHG1 /// LOC100	6.02	6.11	5.68
208705_s_at	eukaryotic transla	EIF5	12.98	12.91	13.05
220236_at	pyruvate dehydro	PDPR	4.95	4.81	4.55
211356_x_at	leptin receptor	LEPR	4.57	4.30	4.32
206076_at	leucine rich repea	LRRC23	6.52	6.21	6.56
207350_s_at	vesicle-associated	VAMP4	7.04	6.98	7.25
203210_s_at	replication factor	RFC5	8.20	8.14	8.23
209922_at	BRCA1 associated	BRAP	6.68	6.46	6.77
204629_at	parvin, beta	PARVB	6.93	6.92	6.99
AFFX-r2-Bs-phe-	---	---	8.07	8.00	8.20
210691_s_at	calcyclin binding p	CACYBP	10.28	10.33	10.61
204397_at	echinoderm micro	EML2	7.04	7.06	7.03
214706_at	zinc finger protein	ZNF200	7.71	7.72	7.89
207541_s_at	exosome compon	EXOSC10	9.77	9.84	9.80
205261_at	progastricsin (pep	PGC	6.47	6.59	6.38
218966_at	myosin VC	MYO5C	4.82	4.56	4.45
205285_s_at	FYN binding prote	FYB	4.58	4.25	4.33
207707_s_at	SEC13 homolog (S	SEC13	11.59	11.59	11.58
204155_s_at	SIK family kinase 3	SIK3	6.62	6.49	6.66
212682_s_at	lipase maturation	LMF2	8.94	8.86	8.44
217994_x_at	cleavage and poly	CPSF3L	8.03	8.09	8.02
202803_s_at	deoxyhypusine sy	DHPS	5.19	5.23	5.07
221382_at	---	---	5.70	5.87	5.41
221513_s_at	UTP14, U3 small r	UTP14A /// UTP14	8.08	8.22	8.19
203932_at	major histocompa	HLA-DMB	6.94	6.86	6.59
217930_s_at	toll interacting pro	TOLLIP	7.40	7.42	7.18
216410_at	---	---	4.63	4.31	4.50
214662_at	WD repeat domai	WDR43	10.81	10.65	10.97
216559_x_at	heterogeneous nu	HNRNPA1 /// HNR	10.81	10.87	11.02
207106_s_at	leukocyte recepto	LTK	4.89	4.22	4.75
211393_at	POZ (BTB) and AT	PATZ1	5.22	5.23	5.06
203513_at	spastic paraplegia	SPG11	9.74	9.71	9.83
218779_x_at	EPS8-like 1	EPS8L1	7.12	7.00	6.87
201094_at	ribosomal protein	RPS29	14.78	14.82	14.83
202647_s_at	neuroblastoma RA	NRAS	8.99	9.06	9.17
217138_x_at	---	---	6.12	5.93	6.05
211647_x_at	immunoglobulin h	IGHG1 /// IGHM //	4.75	4.60	4.34

208556_at	G protein-couplec	GPR31	5.69	5.82	5.99
208552_at	glutamate recepto	GRIK4	5.51	5.34	5.19
220425_x_at	ropporin, rhophili	ROPN1B	5.46	5.25	5.12
208881_x_at	isopentenyl-diphc	IDI1	11.26	11.61	11.40
206887_at	chemokine bindin	CCBP2	6.00	6.00	6.06
222294_s_at	---	---	8.44	8.51	8.93
206006_s_at	KIAA1009	KIAA1009	5.85	5.90	5.92
209984_at	lysine (K)-specific	KDM4C	7.79	7.74	7.91
203683_s_at	vascular endothel	VEGFB	7.83	7.50	7.43
211524_at	nuclear factor of k	NFKB2	3.92	4.31	4.04
212132_at	LSM14A, SCD6 ho	LSM14A	10.21	10.11	10.32
203423_at	retinol binding pro	RBP1	5.31	5.30	5.31
216158_at	---	---	3.29	3.11	3.10
202515_at	discs, large homol	DLG1	10.32	10.34	10.42
202064_s_at	sel-1 suppressor c	SEL1L	6.90	7.04	6.80
200792_at	X-ray repair comp	XRCC6	12.50	12.46	12.46
205260_s_at	acylphosphatase 1	ACYP1	8.73	8.78	8.85
210312_s_at	intraflagellar trans	IFT20	10.63	10.64	10.72
206736_x_at	cholinergic recept	CHRNA4	6.38	5.92	5.77
216934_at	---	---	5.40	5.32	5.28
202387_at	BCL2-associated a	BAG1	9.38	9.31	9.31
219492_at	cysteine-rich hydr	CHIC2	9.71	9.58	9.69
206923_at	protein kinase C, a	PRKCA	5.72	5.55	5.43
204009_s_at	v-Ki-ras2 Kirsten r	KRAS	10.73	10.86	10.75
217434_at	melanocortin 2 re	MC2R	3.47	3.81	3.56
212116_at	tripartite motif-co	TRIM27	9.67	9.60	9.70
212167_s_at	SWI/SNF related,	SMARCB1	7.69	7.61	7.59
203295_s_at	ATPase, Na+/K+ tr	ATP1A2	5.34	5.02	5.02
214469_at	histone cluster 1,	HIST1H2AE	6.50	6.09	6.23
209450_at	O-sialoglycoprote	OSGEP	8.80	8.77	8.80
201652_at	COP9 constitutive	COPS5	10.70	10.78	10.84
220192_x_at	SAM pointed dom	SPDEF	7.17	7.05	6.99
218942_at	phosphatidylinosi	PIP4K2C	8.86	8.84	8.81
212595_s_at	DAZ associated pr	DAZAP2	8.72	8.66	8.56
201428_at	claudin 4	CLDN4	5.47	5.20	5.22
214733_s_at	Yip1 domain famil	YIPF1	8.07	8.07	7.88
205914_s_at	glutamate recepto	GRIN1	5.65	5.53	6.37
206166_s_at	chloride channel a	CLCA2	4.27	4.06	3.90
206301_at	tec protein tyrosir	TEC	6.57	6.17	5.85
203916_at	N-deacetylase/N-s	NDST2	7.92	7.98	7.94
212151_at	pre-B-cell leukemi	PBX1	9.70	9.65	9.59
204952_at	LY6/PLAUR domai	LYPD3	6.91	6.69	6.64
212811_x_at	solute carrier fam	SLC1A4	8.36	8.17	8.38
207311_at	double C2-like dor	DOC2B	6.68	6.92	6.66
217246_s_at	diaphanous homo	DIAPH2	3.86	3.46	3.36
204014_at	dual specificity ph	DUSP4	6.28	6.00	6.09
212195_at	interleukin 6 signa	IL6ST	12.18	12.27	12.46

212845_at	sterile alpha moti	SAMD4A	10.14	10.09	10.17
220413_at	solute carrier fam	SLC39A2	5.58	5.64	5.51
202961_s_at	ATP synthase, H+	ATP5J2	11.87	11.97	11.98
211372_s_at	interleukin 1 rece	IL1R2	5.09	5.22	4.82
216897_s_at	family with seque	FAM76A	6.66	6.72	6.60
217339_x_at	cancer/testis anti	CTAG1A /// CTAG1	4.71	5.04	4.67
208886_at	H1 histone family,	H1F0	11.19	11.02	11.30
210213_s_at	eukaryotic transla	EIF6	10.85	10.84	10.83
219871_at	hypothetical FLJ1	FLJ13197	4.69	4.78	4.75
207996_s_at	chromosome 18 c	C18orf1	3.76	3.55	3.73
205241_at	SCO cytochrome	SCO2	10.13	9.95	10.05
200854_at	nuclear receptor	NCOR1	7.83	7.94	8.04
215894_at	prostaglandin D2	PTGDR	3.35	3.36	3.40
219238_at	phosphatidylinosi	PIGV	9.17	8.95	9.18
208922_s_at	nuclear RNA expo	NXF1	8.27	8.39	8.20
219097_x_at	chromosome 19 c	C19orf42	10.19	10.13	10.25
200034_s_at	ribosomal protein	RPL6	14.06	14.06	14.14
200968_s_at	peptidylprolyl iso	PPIB	12.04	11.96	12.01
207428_x_at	cyclin-dependent	CDK11A /// CDK11	7.51	7.69	7.48
215689_s_at	sex hormone-binc	SHBG	6.99	6.85	6.89
204287_at	synaptogyrin 1	SYNGR1	7.29	7.29	6.96
213977_s_at	CDKN1A interacti	CIZ1	7.23	7.24	7.12
202419_at	3-ketodihydrosph	KDSR	10.68	10.73	10.84
217432_s_at	iduronate 2-sulfat	IDS	6.84	6.68	6.52
207871_s_at	suppression of tu	ST7	6.95	6.67	6.93
209117_at	WW domain bindi	WBP2	7.92	7.88	7.61
202985_s_at	BCL2-associated a	BAG5	9.94	9.95	9.96
215671_at	phosphodiesteras	PDE4B	3.24	3.17	3.15
205701_at	importin 8	IPO8	6.14	6.10	5.92
207612_at	wingless-type MM	WNT8B	7.17	6.98	6.38
211532_x_at	killer cell immuno	KIR2DS2	3.96	3.90	4.16
206601_s_at	homeobox D3	HOXD3	3.81	3.58	3.47
208736_at	actin related prot	ARPC3	8.85	8.92	9.05
208351_s_at	mitogen-activate	MAPK1	7.50	7.45	7.45
205446_s_at	activating transcri	ATF2	6.93	6.97	6.97
211392_s_at	POZ (BTB) and AT	PATZ1	7.04	6.91	6.70
221736_at	Ral GTPase activa	RALGAPB	8.80	8.99	8.98
218526_s_at	RAN guanine nucl	RANGRF	8.78	8.84	8.94
221598_s_at	mediator comple	MED27	8.85	8.74	8.88
218482_at	enhancer of yello	ENY2	11.18	11.25	11.25
217973_at	dicarbonyl/L-xylul	DCXR	9.48	9.38	9.40
201585_s_at	splicing factor pro	SFPQ	11.66	11.69	11.72
203514_at	mitogen-activate	MAP3K3	7.63	7.79	7.64
208549_x_at	Prothymosin, alph	PTMA	12.44	12.49	12.56
220011_at	chromosome 1 op	C1orf135	8.41	8.38	8.53
216925_s_at	T-cell acute lymp	TAL1	6.46	6.17	6.11
215561_s_at	interleukin 1 rece	IL1R1	4.77	4.57	4.46

208468_at	SRY (sex determin	SOX21	5.54	5.54	5.55
210985_s_at	SP100 nuclear ant	SP100	5.47	5.66	5.62
216534_at	---	---	4.12	4.35	4.43
217068_at	---	---	4.32	3.86	4.00
201540_at	four and a half LIM	FHL1	5.17	5.24	5.05
200609_s_at	WD repeat domai	WDR1	11.67	11.78	11.78
215337_at	---	---	5.01	4.86	4.80
206634_at	SIX homeobox 3	SIX3	3.62	3.96	3.60
220488_s_at	breast carcinoma	BCAS3	7.46	7.37	7.51
203988_s_at	fucosyltransferase	FUT8	7.62	7.81	7.87
213960_at	neurotrophic tyro	NTRK3	3.66	3.57	3.85
206884_s_at	sciellin	SCEL	3.40	3.56	3.66
203517_at	metaxin 2	MTX2	10.49	10.59	10.69
212566_at	microtubule-asso	MAP4	10.49	10.45	10.42
204503_at	envoplakin	EVPL	4.78	4.87	4.68
215098_at	retinoid X recepto	RXRB	5.51	5.58	5.29
209610_s_at	solute carrier fam	SLC1A4	8.12	7.71	8.15
201714_at	tubulin, gamma 1	TUBG1	10.31	10.31	10.23
200711_s_at	S-phase kinase-as	SKP1	10.75	10.87	10.84
214821_at	solute carrier fam	SLC25A4	5.42	5.60	5.27
200915_x_at	kinectin 1 (kinesin	KTN1	11.58	11.73	11.77
213000_at	MORC family CW-	MORC3	8.21	8.38	8.41
210435_at	ADP-ribosylation f	ARL17A /// ARL17I	4.47	4.24	4.23
210413_x_at	serpin peptidase i	SERPINB3 /// SERP	4.81	4.42	4.21
207008_at	chemokine (C-X-C	CXCR2	3.36	3.45	3.45
215614_at	---	---	4.48	4.56	4.27
206721_at	chromosome 1 op	C1orf114	3.64	3.60	3.23
218336_at	prefoldin subunit	PFDN2	11.77	11.63	11.76
217105_at	Similar to maligna	LOC100287076	3.82	3.64	3.67
219746_at	D4, zinc and doub	DPF3	6.70	6.44	6.54
211907_s_at	par-6 partitioning	PARD6B	3.81	3.88	4.11
211515_s_at	dual serine/threo	DSTYK	5.26	5.13	4.89
207683_at	forkhead box N1	FOXN1	5.01	4.66	4.83
220989_s_at	amnionless homo	AMN	7.26	6.86	7.23
202771_at	family with seque	FAM38A	9.86	9.68	9.80
202894_at	unc-13 homolog B	UNC13B	8.57	8.55	8.46
222270_at	SMEK homolog 2,	SMEK2	6.55	6.27	6.57
217270_s_at	dual-specificity ty	DYRK1B	5.05	4.87	5.14
208545_x_at	TAF4 RNA polyme	TAF4	5.73	5.69	5.79
219650_at	excision repair cro	ERCC6L	7.75	7.92	7.86
204761_at	USP6 N-terminal l	USP6NL	6.82	6.80	7.14
211085_s_at	serine/threonine l	STK4	7.70	7.60	7.79
213097_s_at	DnaJ (Hsp40) hom	DNAJC2	10.58	10.56	10.70
206224_at	cystatin SN	CST1	5.77	5.77	5.60
203268_s_at	developmentally r	DRG2	8.32	8.19	8.11
202118_s_at	copine III	CPNE3	8.09	8.05	8.09
216633_s_at	phospholipase C, e	PLCH1	5.39	4.98	5.03

215285_s_at	putative homeod	PHTF1	7.83	7.94	7.90
201184_s_at	chromodomain he	CHD4	10.25	10.22	10.20
204415_at	interferon, alpha-	IFI6	8.06	7.85	7.86
220331_at	cytochrome P450,	CYP46A1	4.68	4.69	4.69
203039_s_at	NADH dehydroger	NDUFS1	10.29	10.33	10.33
209000_s_at	septin 8	8-Sep	7.71	7.67	7.42
220445_s_at	CSAG family, mem	CSAG2 /// CSAG3	9.84	9.92	9.89
206387_at	caudal type home	CDX2	6.16	5.89	5.66
205869_at	protease, serine, 1	PRSS1	4.09	4.24	4.01
200061_s_at	ribosomal protein	RPS24	14.95	14.94	14.99
203715_at	tubulin folding co	TBCE	8.25	8.26	8.39
219808_at	selenocysteine lya	SCLY	6.81	6.88	6.69
221704_s_at	vacuolar protein s	VPS37B	7.79	7.74	7.92
211545_at	growth hormone r	GHRHR	5.66	5.40	5.07
217080_s_at	homer homolog 2	HOMER2	6.13	6.17	6.09
213123_at	microfibrillar-asso	MFAP3	9.51	9.48	9.42
204875_s_at	GDP-mannose 4,6	GMDS	7.21	7.32	7.46
217543_s_at	membrane-bounc	MBTPS1	7.35	7.49	7.57
206934_at	signal-regulatory p	SIRPB1	4.00	4.21	4.03
218019_s_at	pyridoxal (pyridox	PDXK	9.14	9.12	9.04
216225_at	---	---	5.29	5.31	5.23
202339_at	symplekin	SYMPK	7.40	7.35	7.39
202592_at	biogenesis of lyso	BLOC1S1	8.67	8.69	8.60
219122_s_at	tRNA-histidine gu	THG1L	8.50	8.34	8.64
210452_x_at	cytochrome P450,	CYP4F2	5.85	5.71	5.83
213340_s_at	KIAA0495	KIAA0495	4.74	4.69	4.78
219245_s_at	2-oxoglutarate an	OGFOD2	6.84	6.69	6.76
216174_at	hepatocellular car	HCRP1	6.83	7.01	6.39
200998_s_at	cytoskeleton-asso	CKAP4	9.94	9.84	9.89
214926_at	spectrin, alpha, no	SPTAN1	4.53	4.71	4.85
217038_at	---	---	4.68	4.62	4.64
217379_at	---	---	12.22	12.07	12.19
216371_at	---	---	7.09	7.00	6.57
202855_s_at	hypoxanthine pho	HPRT1	6.87	6.94	6.70
208268_at	ADAM metallopep	ADAM28	3.19	3.39	3.12
201214_s_at	protein phosphata	PPP1R7	9.80	9.79	9.80
216323_x_at	tubulin, alpha 3d	TUBA3D	8.51	8.45	8.34
209411_s_at	golgi-associated, g	GGA3	7.33	7.44	7.34
37586_at	zinc finger protein	ZNF142	7.26	6.96	7.06
202781_s_at	inositol polyphosp	INPP5K	7.13	6.94	7.33
217177_s_at	protein tyrosine p	PTPRB	4.53	4.26	4.15
58308_at	tripartite motif-co	TRIM62	6.56	6.25	6.36
206749_at	CD1b molecule	CD1B	4.32	4.66	4.62
209981_at	cold shock domain	CSDC2	6.85	6.59	6.70
207160_at	interleukin 12A (n	IL12A	6.17	5.98	6.26
201764_at	transmembrane p	TMEM106C	10.89	10.79	10.83
217689_at	Protein tyrosine p	PTPN1	4.46	4.26	4.53

217651_at	---	---	3.89	3.89	3.62
210013_at	hemopexin	HPX	6.55	6.62	6.40
218022_at	vaccinia related ki	VRK3	7.99	7.96	7.87
204943_at	ADAM metallopep	ADAM12	6.03	6.17	5.80
205518_s_at	cytidine monopho	CMAH	3.26	3.65	3.36
212260_at	GRB10 interacting	GIGYF2	7.93	8.03	7.99
215222_x_at	microtubule-actin	MACF1	9.03	9.00	9.06
220886_at	gamma-aminobut	GABRQ	4.03	4.05	4.12
213641_at	zinc finger protein	ZNF500	5.97	6.10	5.69
203053_at	breast carcinoma	BCAS2	10.30	10.35	10.53
216731_s_at	---	---	3.54	3.35	3.39
210679_x_at	---	---	8.19	8.18	8.09
214521_at	hairy and enhance	HES2	3.75	3.71	3.68
204813_at	mitogen-activated	MAPK10	6.71	6.85	6.63
220969_s_at	---	---	6.43	6.62	6.16
213764_s_at	microfibrillar asso	MFAP5	3.08	2.95	2.88
203176_s_at	transcription facto	TFAM	10.36	10.48	10.56
216160_at	---	---	5.07	4.95	5.02
207407_x_at	cytochrome P450,	CYP4A11	4.13	4.15	3.98
206455_s_at	rhodopsin	RHO	6.16	6.20	6.00
219208_at	F-box protein 11	FBXO11	5.10	5.14	5.45
204019_s_at	SH3 domain conta	SH3YL1	7.70	7.92	7.88
213745_at	attractin-like 1	ATRNL1	3.20	3.36	3.22
203240_at	Fc fragment of IgG	FCGBP	4.42	4.32	4.47
208824_x_at	cyclin-dependent	CDK16	7.45	7.22	7.17
213269_at	zinc finger protein	ZNF248	7.27	7.35	7.08
216422_at	proliferation-asso	PA2G4 /// PA2G4P	5.90	5.67	5.83
209284_s_at	chromosome 3 op	C3orf63	8.89	9.02	9.26
221108_at	chromosome 22 o	C22orf43	7.41	7.22	6.94
204561_x_at	apolipoprotein C-I	APOC2	5.92	5.83	5.75
222266_at	Chromosome 19 o	C19orf2	8.63	8.90	8.84
216124_at	---	---	3.43	3.47	3.32
217431_x_at	cytochrome b-245	CYBB	3.57	3.12	3.31
216634_at	phospholipase C,	PLCH1	3.20	3.36	3.27
206912_at	forkhead box E1 (	FOXE1	3.16	3.42	3.39
204120_s_at	adenosine kinase	ADK	6.64	6.63	6.98
203470_s_at	pleckstrin	PLEK	7.05	7.05	6.96
204456_s_at	growth arrest-spe	GAS1	3.58	3.74	3.67
221065_s_at	carbohydrate (N-a	CHST8	5.62	5.98	5.84
221747_at	tensin 1	TNS1	5.91	5.57	5.75
212636_at	quaking homolog,	QKI	9.05	9.01	9.21
217242_at	zinc finger protein	ZNF154	3.76	3.39	3.57
202156_s_at	CUGBP, Elav-like f	CELF2	3.17	3.17	3.22
208019_at	zinc finger protein	ZNF157	3.28	3.37	3.42
205005_s_at	N-myristoyltransfe	NMT2	9.61	9.55	9.85
218917_s_at	AT rich interactive	ARID1A	10.22	10.20	10.35
220867_s_at	solute carrier fam	SLC24A2	4.92	4.85	4.84

214192_at	Nucleoporin 88kD	NUP88	5.08	4.82	4.67
209569_x_at	DNA segment on (	D4S234E /// FOXP	4.28	4.41	4.08
208969_at	NADH dehydroger	NDUFA9	10.73	10.60	10.72
207222_at	phospholipase A2	PLA2G10	6.20	6.13	6.05
222237_s_at	zinc finger proteir	ZFP112	6.48	6.83	6.51
215390_at	---	---	4.34	4.41	3.99
218049_s_at	mitochondrial rib	MRPL13	10.58	10.65	10.77
201663_s_at	structural mainter	SMC4	10.85	11.09	11.08
214547_at	adenylate cyclase	ADCY10	4.20	3.92	3.98
200999_s_at	cytoskeleton-asso	CKAP4	10.84	10.83	10.89
213046_at	poly(A) binding pr	PABPN1	7.57	7.80	7.49
215419_at	zinc finger RNA bi	ZFR2	5.67	5.52	5.75
218365_s_at	aspartyl-tRNA syn	DARS2	8.27	8.57	8.45
212475_at	AVL9 homolog (S.	AVL9	7.86	7.79	7.62
207151_at	adenylate cyclase	ADCYAP1R1	3.51	3.82	3.79
210226_at	nuclear receptor s	NR4A1	7.76	7.57	7.55
219033_at	poly (ADP-ribose)	PARP8	8.13	8.09	8.25
214490_at	arylsulfatase F	ARSF	5.70	5.66	5.43
214089_at	ribosomal protein	RPS8 /// RPS8P8	4.62	4.88	5.04
207572_at	---	---	4.37	4.37	4.24
207280_at	Ring finger proteir	RNF185	6.15	6.25	5.96
216384_x_at	prothymosin, alph	LOC441454 /// LO	12.48	12.51	12.57
218346_s_at	sestrin 1	SESN1	8.48	8.45	8.15
202634_at	polymerase (RNA)	POLR2K	9.30	9.43	9.55
210252_s_at	MAP-kinase activa	MADD	8.29	8.33	8.32
207379_at	EGF-like repeats a	EDIL3	4.41	4.64	4.68
208309_s_at	mucosa associate	MALT1	8.38	8.58	8.66
217530_at	solute carrier fam	SLC34A1	5.06	4.63	4.62
221018_s_at	tudor domain con	TDRD1	3.51	3.51	3.53
211223_at	PROP paired-like	PROP1	6.31	6.10	6.02
202408_s_at	PRP31 pre-mRNA	PRPF31	10.09	10.04	10.07
216280_s_at	dicer 1, ribonucle	DICER1	3.83	4.06	3.84
215506_s_at	DIRAS family, GTP	DIRAS3	4.09	4.12	4.11
205039_s_at	IKAROS family zin	IKZF1	8.61	8.55	8.21
221399_at	ectodysplasin A2 r	EDA2R	6.02	6.08	5.47
205140_at	fucose-1-phospha	FPGT	7.18	7.40	7.60
211504_x_at	Rho-associated, c	ROCK2	5.97	6.29	6.19
216787_at	---	---	4.91	5.08	5.11
215965_at	---	---	4.50	4.51	4.18
220374_at	kelch-like 28 (Dro	KLHL28	5.62	5.83	5.68
205473_at	ATPase, H+ transp	ATP6V1B1	5.84	6.06	5.66
216796_s_at	---	---	3.79	3.58	3.49
220453_at	PQ loop repeat co	PQLC2	7.58	7.51	7.22
221870_at	EH-domain contai	EHD2	9.76	9.81	9.78
54037_at	Hermansky-Pudla	HPS4	7.96	7.82	8.17
211253_x_at	peptide YY	PYY	6.65	6.84	6.34
202899_s_at	syndecan 3	SDC3	11.94	12.09	12.07

219453_at	kelch-like 36 (Dro:	KLHL36	7.20	7.20	7.32
215526_at	---	---	5.20	5.29	5.30
205162_at	excision repair crc	ERCC8	8.92	9.06	9.12
219814_at	muscleblind-like 3	MBNL3	3.30	3.36	3.26
214423_x_at	Aldolase B, fructo:	ALDOB	6.65	6.69	6.65
209382_at	polymerase (RNA)	POLR3C	9.40	9.17	9.38
210010_s_at	solute carrier fam	SLC25A1	8.54	8.50	8.63
213158_at	---	---	9.48	9.50	9.61
204294_at	aminomethyltran:	AMT	7.03	7.43	6.77
211913_s_at	c-mer proto-onco	MERTK	5.10	5.09	4.64
214893_x_at	hyperpolarization	HCN2	7.55	7.43	7.30
211203_s_at	contactin 1	CNTN1	5.03	5.43	5.21
207397_s_at	homeobox D13	HOXD13	3.73	3.80	3.44
214769_at	chloride channel 4	CLCN4	4.56	4.42	4.20
204654_s_at	transcription facto	TFAP2A	4.15	4.23	4.08
217228_s_at	ankyrin repeat an	ASB4	4.36	4.13	4.09
213145_at	F-box and leucine-	FBXL14	8.95	8.97	9.09
207627_s_at	transcription facto	TFCP2	7.21	7.17	7.21
210293_s_at	Sec23 homolog B	SEC23B	10.10	10.10	10.11
201695_s_at	purine nucleoside	PNP	9.40	9.59	9.51
205628_at	primase, DNA, pol	PRIM2	8.07	8.12	8.40
210023_s_at	polycomb group r	PCGF1	5.81	6.06	6.16
208054_at	hect domain and I	HERC4	4.37	4.92	4.14
214908_s_at	transformation/tr	TRRAP	5.65	5.66	5.61
203443_at	echinoderm micro	EML3	6.37	6.69	6.26
200848_at	adenosylhomocys	AHCYL1	10.72	10.70	10.75
210022_at	polycomb group r	PCGF1	8.60	8.60	8.28
216720_at	cytochrome P450,	CYP2U1	4.00	3.47	3.72
208761_s_at	SMT3 suppressor	SUMO1	11.33	11.44	11.55
216086_at	synaptic vesicle gl	SV2C	3.61	3.53	3.41
213462_at	neuronal PAS dom	NPAS2	5.65	5.66	5.56
200012_x_at	ribosomal protein	RPL21	14.82	14.83	14.89
214458_at	TNF receptor-assc	TRAF3IP1	5.81	5.87	5.90
212962_at	synapse defective	SYDE1	5.85	5.86	5.51
221392_at	taste receptor, ty	TAS2R4	4.37	4.22	4.66
204225_at	histone deacetyla	HDAC4	8.33	8.15	8.17
212596_s_at	HMG box domain	HMGXB4	9.55	9.59	9.56
32699_s_at	poliovirus recepto	PVR	6.91	6.97	7.04
215765_at	leucine rich repea	LRRC41	4.72	4.48	4.14
219983_at	HRAS-like suppres	HRASLS	4.62	4.16	4.31
203167_at	TIMP metallopept	TIMP2	7.78	7.60	7.71
215009_s_at	SEC31 homolog A	SEC31A	10.27	10.18	10.71
218506_x_at	glyoxylate reducta	GLYR1	8.59	8.69	8.60
210800_at	translocase of inn	TIMM8A	3.49	3.68	3.76
210115_at	ribosomal protein	RPL39L	5.58	5.17	5.22
213354_s_at	hypothetical prote	LOC100287051	4.95	5.01	4.43
205804_s_at	TRAF3 interacting	TRAF3IP3	4.36	3.89	3.89

200910_at	chaperonin contai	CCT3	12.12	12.13	12.17
214836_x_at	immunoglobulin k	IGK@ /// IGKC	4.38	4.56	4.70
217850_at	guanine nucleotid	GNL3	11.56	11.57	11.76
220140_s_at	sorting nexin 11	SNX11	7.34	7.33	7.54
220128_s_at	NIPA-like domain	NIPAL2	5.81	6.03	5.70
210772_at	formyl peptide re	FPR2	3.98	3.84	3.87
213359_at	Heterogeneous nu	HNRNPD	8.84	9.04	9.02
207308_at	solute carrier orga	SLCO1A2	3.97	3.88	4.06
214251_s_at	nuclear mitotic ap	NUMA1	6.12	6.24	6.12
205751_at	SH3-domain GRB2	SH3GL2	3.24	3.18	3.25
221886_at	DENN/MADD dom	DENND2A	3.39	3.10	3.27
218398_at	mitochondrial ribo	MRPS30	10.85	10.64	10.75
215416_s_at	stomatin (EPB72)-	STOML2	10.63	10.56	10.64
213174_at	tetratricopeptide	TTC9	6.00	5.82	5.90
218989_x_at	solute carrier fam	SLC30A5	9.95	10.15	10.28
203611_at	telomeric repeat b	TERF2	6.62	6.79	6.83
216854_at	growth differentia	GDF11	5.02	4.76	4.56
207787_at	keratin 33B	KRT33B	5.21	4.97	4.92
206825_at	oxytocin receptor	OXTR	6.01	6.21	5.93
206583_at	zinc finger family	ZNF673	7.78	7.87	7.77
204593_s_at	Smith-Magenis sy	SMCR7L	8.83	8.98	9.09
221224_s_at	dephospho-CoA k	DCAKD	5.56	5.56	5.76
204586_at	bassoon (presyna	BSN	4.48	4.78	4.22
215244_at	DiGeorge syndrom	DGCR5	5.54	5.29	5.22
212810_s_at	solute carrier fam	SLC1A4	7.96	7.76	7.97
218666_s_at	syntaxin 17	STX17	7.09	7.15	7.19
221500_s_at	syntaxin 16	STX16	9.68	9.79	9.83
205643_s_at	protein phosphata	PPP2R2B	4.19	3.63	3.87
204237_at	GULP, engulfment	GULP1	3.31	3.28	3.21
201503_at	GTPase activating	G3BP1	12.05	12.04	12.20
204174_at	arachidonate 5-lip	ALOX5AP	5.53	5.48	5.44
201595_s_at	zinc finger CCCH-t	ZC3H15	11.18	11.22	11.44
207918_s_at	testis specific prot	TSPY1 /// TSPY4 //	4.03	3.92	3.76
219620_x_at	chromosome 9 op	C9orf167	7.18	7.14	7.17
214040_s_at	gelsolin	GSN	5.61	5.56	5.20
204034_at	ethylmalonic ence	ETHE1	9.99	9.87	9.96
205074_at	solute carrier fam	SLC22A5	7.40	7.63	7.71
217259_at	---	---	3.96	4.11	3.81
205742_at	troponin I type 3 (	TNNI3	5.06	5.21	4.76
203719_at	excision repair cro	ERCC1	8.66	8.62	8.74
214065_s_at	calcium and integ	CIB2	5.95	5.57	5.66
39705_at	SIN3 homolog B, t	SIN3B	7.28	7.40	7.26
200851_s_at	KIAA0174	KIAA0174	10.79	10.77	10.80
202984_s_at	BCL2-associated a	BAG5	9.95	10.05	10.22
211779_x_at	adaptor-related p	AP2A2	9.14	9.06	9.03
222249_at	---	---	3.48	3.39	3.54
203107_x_at	ribosomal protein	RPS2	15.17	15.16	15.20

220198_s_at	eukaryotic transla	EIF5A2	7.49	7.50	7.77
209827_s_at	interleukin 16 (lyn	IL16	6.26	5.93	5.78
213775_x_at	zinc finger proteir	ZNF638	9.58	9.79	9.74
215634_at	---	---	3.70	3.65	3.55
211843_x_at	cytochrome P450,	CYP3A7	3.82	4.11	3.77
216104_at	---	---	3.34	3.36	3.35
219439_at	core 1 synthase, g	C1GALT1	8.85	8.91	9.04
220312_at	family with seque	FAM83E	5.91	5.47	5.49
216322_at	CD58 molecule	CD58	3.52	3.43	3.36
214966_at	glutamate recepto	GRIK5	5.64	5.38	5.48
205921_s_at	solute carrier fam	SLC6A6	4.37	4.05	4.09
200656_s_at	prolyl 4-hydroxyla	P4HB	9.41	9.44	9.53
218903_s_at	oligonucleotide/o	OBFC2B	7.36	7.32	7.69
214833_at	transmembrane p	TMEM63A	5.04	5.04	5.12
203805_s_at	Fanconi anemia, c	FANCA	6.81	6.97	6.95
207287_at	hypothetical LOC8	FLJ14107	5.73	5.65	5.63
216671_x_at	mucin 8	MUC8	8.82	8.61	8.23
200916_at	transgelin 2	TAGLN2	12.21	12.24	12.20
212164_at	transmembrane p	TMEM183A	7.55	7.57	7.53
217119_s_at	chemokine (C-X-C	CXCR3	7.13	6.64	6.93
209045_at	X-prolyl aminopep	XPNPEP1	10.07	10.08	10.14
207989_at	opioid receptor, n	OPRM1	4.11	3.89	3.90
215180_at	---	---	5.45	5.48	4.94
201189_s_at	inositol 1,4,5-tripl	ITPR3	8.88	8.96	8.98
216874_at	Hypothetical gene	DKFZp686O1327	3.26	3.50	3.02
203291_at	CCR4-NOT transcr	CNOT4	8.43	8.37	8.65
217523_at	CD44 molecule (Ir	CD44	7.22	7.59	7.39
203942_s_at	MAP/microtubule	MARK2	7.17	7.08	7.01
221666_s_at	PYD and CARD do	PYCARD	5.73	5.19	5.17
210234_at	glutamate recepto	GRM4	7.23	7.05	6.82
207890_s_at	matrix metallopep	MMP25	6.75	6.79	6.81
220473_s_at	zinc finger, CCHC (	ZCCHC4	3.97	3.91	3.93
214804_at	centromere prote	CENPI	7.57	7.68	7.45
215185_at	---	---	6.21	6.30	6.03
215213_at	nucleoporin 54kD	NUP54	3.35	3.31	3.25
218923_at	chitobiase, di-N-a	CTBS	6.83	7.01	7.13
204276_at	thymidine kinase :	TK2	8.87	8.60	8.82
219395_at	epithelial splicing	ESRP2	4.96	4.78	4.82
202341_s_at	tripartite motif-co	TRIM2	8.55	8.60	8.78
200615_s_at	adaptor-related p	AP2B1	9.61	9.46	9.62
218833_at	sterile alpha motif	ZAK	6.90	6.64	6.68
215624_at	tuberous sclerosis	TSC2	5.71	5.93	5.94
221144_at	---	---	3.13	3.32	3.16
208520_at	olfactory receptor	OR10H3	4.21	4.43	3.76
217044_s_at	pleckstrin homolo	PLEKHG3	5.90	5.85	5.86
215194_at	protein kinase C, a	PRKCA	4.93	4.85	4.78
220406_at	transforming grov	TGFB2	4.03	4.24	4.35

221728_x_at	X (inactive)-specif	XIST	10.42	10.60	10.55
214729_at	TWIST neighbor	TWISTNB	6.16	6.29	6.63
202316_x_at	ubiquitination fac	UBE4B	5.94	6.04	5.82
206361_at	G protein-couplec	GPR44	7.80	7.67	7.56
200863_s_at	RAB11A, member	RAB11A	11.24	11.27	11.24
205644_s_at	small nuclear ribo	SNRPG	12.76	12.87	12.92
216501_at	Vac14 homolog (S	VAC14	5.09	5.36	5.27
202349_at	torsin family 1, m	TOR1A	8.71	8.82	8.73
215250_at	Transmembrane p	TMEM111	6.89	6.99	7.04
208460_at	gap junction prote	GJC1	6.46	6.58	6.51
215934_at	---	---	3.72	3.93	3.97
216663_s_at	zinc finger, MYND	ZMYND10	4.90	5.17	4.70
207089_at	nebulin-related ar	NRAP	3.93	3.82	3.67
219446_at	resistance to inhib	RIC8B	7.27	7.33	7.35
202021_x_at	eukaryotic transla	EIF1	13.66	13.37	13.58
217918_at	dynein, light chair	DYNLRB1	10.41	10.53	10.50
221854_at	plakophilin 1 (ecto	PKP1	5.79	5.80	5.46
221885_at	DENN/MADD dom	DENND2A	6.03	5.94	5.83
220324_at	chromosome 6 op	C6orf155	4.25	4.44	4.32
204433_s_at	spermatogenesis a	SPATA2	7.12	7.11	6.90
208474_at	claudin 6	CLDN6	7.97	7.73	7.81
206958_s_at	UPF3 regulator of	UPF3A	9.22	9.45	9.43
209977_at	plasminogen	PLG	4.43	4.49	4.47
212804_s_at	GTPase activating	GAPVD1	8.49	8.55	8.56
204414_at	like-glycosyltransf	LARGE	3.66	3.40	3.31
218320_s_at	NADH dehydroger	NDUFB11	11.20	11.05	11.10
207550_at	myeloproliferative	MPL	4.13	3.80	3.65
217616_at	---	---	4.01	3.80	3.81
220494_s_at	---	---	6.51	6.30	6.58
206914_at	cytotoxic and regu	CRTAM	3.35	3.27	3.20
216441_at	---	---	3.05	3.20	3.36
200018_at	ribosomal protein	RPS13	14.66	14.64	14.66
219552_at	sushi, von Willebr	SVEP1	3.41	3.35	3.25
212198_s_at	transmembrane 9	TM9SF4	8.30	8.25	8.40
208189_s_at	myosin VIIA	MYO7A	5.81	5.62	5.53
216805_at	---	---	4.71	4.71	4.54
210890_x_at	killer cell immuno	KIR2DL1	5.70	5.90	5.54
212503_s_at	DIP2 disco-interac	DIP2C	8.13	8.22	8.26
214549_x_at	small proline-rich	SPRR1A	6.18	6.08	5.86
218301_at	arginyl aminopept	RNPEPL1	7.44	7.29	7.19
218980_at	formin homology	FHOD3	6.10	5.98	6.38
211523_at	gonadotropin-rele	GNRHR	3.66	3.59	3.75
216788_at	---	---	4.62	4.47	4.40
205283_at	fukutin	FKTN	8.29	8.32	8.29
203224_at	riboflavin kinase	RFK	9.49	9.51	9.77
208920_at	sorcin	SRI	8.54	8.61	8.68
200959_at	fused in sarcoma	FUS	11.09	11.05	11.12

200699_at	KDEL (Lys-Asp-Glu	KDELR2	11.17	11.18	11.09
220244_at	loss of heterozygo	LOH3CR2A	5.81	6.06	5.91
206327_s_at	cadherin 15, type	CDH15	6.42	6.58	6.21
205886_at	regenerating islet-	REG1B	3.94	3.84	3.63
221613_s_at	zinc finger, AN1-ty	ZFAND6	9.99	9.98	10.20
216739_at	---	---	6.75	6.60	6.37
205153_s_at	CD40 molecule, TI	CD40	6.36	6.61	6.29
213863_s_at	ornithine decarbo	OAZ3	6.67	6.46	6.18
221750_at	3-hydroxy-3-meth	HMGCS1	9.41	10.34	9.79
222361_at	tubulin, beta pseu	TUBBP5	4.16	3.85	4.03
206715_at	transcription facto	TFEC	3.60	3.53	3.66
205846_at	protein tyrosine p	PTPRB	4.43	4.24	4.02
213200_at	synaptophysin	SYP	5.94	5.90	5.64
209406_at	BCL2-associated a	BAG2	10.10	10.14	10.32
207014_at	gamma-aminobut	GABRA2	3.34	3.40	3.19
213884_s_at	tripartite motif-co	TRIM3	5.02	5.23	4.89
216328_at	sialic acid binding	SIGLEC8	4.38	4.20	4.38
215998_at	---	---	4.92	4.84	4.69
214091_s_at	glutathione perox	GPX3	4.96	4.80	4.81
201605_x_at	calponin 2	CNN2	8.16	8.14	8.09
209557_s_at	neurochondrin	NCDN	7.04	6.99	6.85
215346_at	CD40 molecule, TI	CD40	5.56	5.95	5.92
218776_s_at	transmembrane p	TMEM62	7.82	7.86	7.67
213760_s_at	zinc finger protein	ZNF330	9.11	9.03	9.18
204894_s_at	amine oxidase, co	AOC3	6.06	5.96	5.91
221696_s_at	serine/threonine/	STYK1	3.68	3.77	3.40
200596_s_at	eukaryotic transla	EIF3A	10.90	10.87	10.96
218138_at	McKusick-Kaufma	MKKS	10.14	10.11	10.15
208134_x_at	pregnancy specific	PSG2	4.76	4.34	4.31
204764_at	farnesyltransferas	FNTB	5.96	6.00	5.99
211181_x_at	runt-related trans	RUNX1	6.61	6.66	6.38
201387_s_at	ubiquitin carboxyl	UCHL1	5.67	5.76	5.91
213978_at	metastasis suppre	MTSS1L	6.44	6.46	6.36
216419_at	ciliary rootlet coil	CROCC	5.19	5.01	4.79
201624_at	aspartyl-tRNA syn	DARS	9.58	9.67	9.91
221461_at	taste receptor, typ	TAS2R9	3.61	3.51	3.62
216447_at	---	---	3.89	3.92	4.08
206502_s_at	insulinoma-associ	INSM1	3.43	3.65	3.41
212570_at	endonuclease dor	ENDOD1	6.62	6.71	6.62
214897_at	---	---	3.67	3.60	3.65
212194_s_at	transmembrane 9	TM9SF4	7.97	8.05	8.11
218953_s_at	prenylcysteine oxi	PCYOX1L	8.70	8.85	8.86
213990_s_at	p21 protein (Cdc4	PAK7	5.46	5.49	5.41
203067_at	pyruvate dehydro	PDHX	10.00	9.95	10.17
203641_s_at	COBL-like 1	COBLL1	6.04	6.18	6.02
211887_x_at	macrophage scave	MSR1	4.14	4.00	4.37
202290_at	PDGFA associated	PDAP1	9.18	9.05	9.14

205910_s_at	carboxyl ester lipa	CEL	3.77	3.76	3.41
205221_at	homogentisate 1,2	HGD	4.01	4.12	3.95
201847_at	lipase A, lysosoma	LIPA	9.76	9.73	9.77
214758_at	DDB1 and CUL4 as	DCAF4	6.00	5.96	5.90
222331_at	---	---	5.91	6.12	6.01
220066_at	nucleotide-binding	NOD2	3.94	4.27	3.98
221561_at	sterol O-acyltrans	SOAT1	7.42	7.37	7.71
209349_at	RAD50 homolog (S	RAD50	8.35	8.45	8.53
205372_at	pleiomorphic ader	PLAG1	7.40	7.69	7.73
216267_s_at	transmembrane p	TMEM115	7.73	7.61	7.47
208980_s_at	ubiquitin C	UBC	12.38	12.51	12.41
205255_x_at	transcription facto	TCF7	8.05	8.13	7.95
207091_at	purinergic recepto	P2RX7	4.94	5.12	4.76
219047_s_at	zinc finger protein	ZNF668	6.18	5.90	6.00
218469_at	gremlin 1, cystein	GREM1	3.12	3.31	3.28
203308_x_at	Hermansky-Pudlak	HPS1	7.93	7.93	7.86
214347_s_at	dopa decarboxyla	DDC	5.01	4.92	4.77
215725_at	DiGeorge syndrom	DGCR11	6.79	6.91	7.05
207258_at	Down syndrome c	DSCR4	5.70	5.67	5.28
217486_s_at	zinc finger, DHHC-	ZDHHC17	4.33	4.41	4.81
207779_at	---	---	3.82	3.61	3.47
221774_x_at	family with seque	FAM48A	8.91	8.99	8.94
202445_s_at	Notch homolog 2	NOTCH2	6.30	6.16	6.19
220710_at	chromosome 15 o	C15orf28	4.19	4.20	4.22
210260_s_at	tumor necrosis fac	TNFAIP8	7.91	7.96	8.27
210837_s_at	phosphodiesteras	PDE4D	3.78	3.38	3.70
206210_s_at	cholesteryl ester t	CETP	4.22	4.23	4.18
212325_at	LIM and calponin	LIMCH1	8.90	8.70	9.03
221939_at	Yip1 domain famil	YIPF2	8.00	7.98	7.78
34449_at	caspase 2, apopto	CASP2	5.08	5.19	4.92
207275_s_at	acyl-CoA syntheta	ACSL1	9.46	9.43	9.56
202336_s_at	peptidylglycine al	PAM	10.33	10.39	10.52
215373_x_at	---	---	8.63	8.78	8.76
215002_at	similar to Unchara	LOC100132247 ///	4.44	4.09	4.14
218620_s_at	HemK methyltran	HEMK1	8.36	8.29	8.15
203057_s_at	PR domain contai	PRDM2	8.68	8.85	8.76
218480_at	ATP/GTP binding	AGBL5	7.75	7.95	7.73
219481_at	tetratricopeptide	TTC13	8.61	8.68	8.48
216586_at	---	---	6.06	5.95	5.83
210838_s_at	activin A receptor	ACVRL1	5.43	5.62	5.11
219606_at	PHD finger protei	PHF20L1	5.13	5.13	4.94
207110_at	potassium inward	KCNJ12	4.03	3.66	3.82
216007_at	---	---	3.36	3.51	3.14
214468_at	myosin, heavy cha	MYH6	4.36	4.45	4.20
211685_s_at	neurocalcin delta	NCALD	4.79	4.79	4.67
216024_at	dynamin 2	DNM2	5.35	5.39	5.35
209575_at	interleukin 10 rec	IL10RB	7.82	7.79	7.93

214815_at	tripartite motif-co	TRIM33	5.37	5.71	5.45
210780_at	estrogen receptor	ESR2	6.34	6.56	6.20
203352_at	origin recognition	ORC4L	7.71	7.75	7.83
207460_at	granzyme M (lymp	GZMM	5.04	5.00	4.78
216424_at	CD4 molecule	CD4	4.88	4.64	4.52
208693_s_at	glycyl-tRNA synth	GARS	13.27	12.97	13.15
215006_at	---	---	6.94	7.04	6.71
211776_s_at	erythrocyte meml	EPB41L3	8.95	9.00	9.04
202744_at	solute carrier fam	SLC20A2	5.97	5.93	6.10
210812_at	X-ray repair comp	XRCC4	5.14	5.32	5.23
220427_at	obscurin, cytoskel	OBSCN	7.34	7.24	6.68
210827_s_at	E74-like factor 3 (	ELF3	7.04	7.16	6.64
210508_s_at	potassium voltage	KCNQ2	5.73	5.52	5.63
216272_x_at	synapse defective	SYDE1	8.16	8.21	8.09
204913_s_at	SRY (sex determin	SOX11	3.37	3.64	3.55
213653_at	methyltransferase	METTL3	7.94	8.14	8.05
206716_at	uromodulin	UMOD	4.71	4.47	4.80
209511_at	polymerase (RNA)	POLR2F	10.13	10.07	10.07
211942_x_at	ribosomal protein	RPL13A /// RPL13A	14.67	14.62	14.56
221327_s_at	opsin 1 (cone pigr	OPN1LW /// OPN1	4.93	5.04	4.89
208092_s_at	family with seque	FAM49A	5.86	5.66	5.51
214533_at	chymase 1, mast c	CMA1	5.00	4.99	4.75
205504_at	Bruton agammagl	BTK	5.17	5.07	4.76
207515_s_at	polymerase (RNA)	POLR1C	9.35	9.21	9.26
219357_at	GTP binding prote	GTPBP1	9.08	8.98	9.11
202987_at	TRAF3 interacting	TRAF3IP2	5.37	5.53	5.35
216948_at	---	---	3.57	3.42	3.20
78047_s_at	hypothetical LOC7	LOC729580	9.00	9.04	8.90
213939_s_at	RUN and FYVE dor	RUFY3	8.05	8.25	8.19
219842_at	ADP-ribosylation f	ARL15	4.43	4.45	4.50
38892_at	KIAA0240	KIAA0240	8.15	7.96	8.23
220525_s_at	ancient ubiquitou	AUP1	10.21	10.16	10.10
204032_at	breast cancer anti	BCAR3	8.72	8.79	8.98
201132_at	heterogeneous nu	HNRNPH2	10.68	10.71	10.78
217408_at	mitochondrial ribo	MRPS18B	11.06	11.03	11.26
209244_s_at	kinesin family mer	KIF1C	7.68	7.67	7.74
204183_s_at	adrenergic, beta, r	ADRBK2	7.22	7.03	7.04
216425_at	---	---	5.95	5.93	5.49
214920_at	thrombospondin,	THSD7A	3.76	3.54	3.45
209540_at	insulin-like growth	IGF1	3.90	3.82	3.88
219307_at	prenyl (decapreny	PDSS2	8.37	8.41	8.59
208495_at	T-cell leukemia ho	TLX3	5.08	5.12	4.85
212288_at	formin binding pro	FNBP1	8.46	8.46	8.55
208583_x_at	histone cluster 1,	HIST1H2AJ	7.96	7.80	7.81
209156_s_at	collagen, type VI,	COL6A2	6.47	6.42	5.82
215417_at	exocyst complex c	EXOC6B	4.19	4.03	4.35
217182_at	mucin 5AC, oligon	MUC5AC	6.36	6.25	6.01

204356_at	LIM domain kinas	LIMK1		6.50	6.58	6.29
212852_s_at	TROVE domain fa	TROVE2		10.68	10.74	10.80
204004_at	PRKC, apoptosis, \	PAWR		9.62	9.64	9.82
216459_x_at	tigger transposab	TIGD1L		4.21	4.47	4.46
205598_at	TRAF interacting p	TRAIP		7.08	6.99	7.05
203403_s_at	ring finger protein	RNF6		10.01	10.12	10.28
216088_s_at	proteasome (pros	PSMA7		10.47	10.38	10.65
221418_s_at	mediator complex	MED16		6.25	6.33	6.18
214730_s_at	golgi glycoprotein	GLG1		9.02	8.90	9.06
211446_at	regulator of G-pro	RGSL1		4.71	4.43	4.42
220403_s_at	tumor protein p53	TP53AIP1		4.25	3.92	3.75
212911_at	DnaJ (Hsp40) hom	DNAJC16		7.24	7.50	7.61
210392_x_at	nuclear receptor s	NR6A1		6.45	6.74	6.56
208230_s_at	neuregulin 1	NRG1		6.42	6.17	6.04
214615_at	purinergic recepto	P2RY10		3.27	3.27	3.25
211416_x_at	gamma-glutamylt	GGTLC1		6.78	6.76	6.69
218183_at	chromosome 16 o	C16orf5		5.97	6.31	6.26
207772_s_at	protein arginine m	PRMT8		4.61	4.44	4.47
208265_at	chromosome 2 op	C2orf83		4.29	3.86	3.93
219473_at	ganglioside induce	GDAP2		6.48	6.12	6.22
219513_s_at	SH2 domain conta	SH2D3A		5.55	5.36	5.02
206326_at	gastrin-releasing p	GRP		3.70	3.47	3.49
204469_at	protein tyrosine p	PTPRZ1		3.12	3.05	3.30
218818_at	four and a half LIN	FHL3		8.53	8.40	8.69
214738_s_at	NIMA (never in m	NEK9		6.74	6.91	6.87
217869_at	hydroxysteroid (1	HSD17B12		11.78	11.82	11.86
220781_at	deleted in esopha		1-Dec	4.12	3.79	3.72
41220_at	septin 9		9-Sep	12.26	12.26	12.24
217937_s_at	histone deacetyla	HDAC7		7.52	7.30	7.63
207458_at	chromosome 8 op	C8orf51		6.49	6.52	6.45
211934_x_at	glucosidase, alpha	GANAB		9.46	9.35	9.43
206807_s_at	adducin 2 (beta)	ADD2		7.01	6.99	6.53
216800_at	---	---		5.99	5.95	5.83
211305_x_at	Fc fragment of IgA	FCAR		5.94	5.63	5.45
210101_x_at	SH3-domain GRB2	SH3GLB1		10.45	10.32	10.32
210884_s_at	sperm associated	SPAG11A		5.35	4.96	4.92
222241_at	hypothetical prote	LOC100291367		5.97	6.00	5.88
208371_s_at	ring finger protein	RING1		9.09	8.94	8.96
222071_s_at	solute carrier orga	SLCO4C1		3.65	3.83	3.38
218789_s_at	chromosome 11 o	C11orf71		5.97	5.60	5.51
213512_at	chromosome 14 o	C14orf79		7.31	7.04	7.20
210120_s_at	RAN binding prote	RANBP3		7.36	7.12	7.27
201946_s_at	chaperonin contai	CCT2		10.74	10.88	10.86
206176_at	bone morphogene	BMP6		5.13	5.24	5.00
206805_at	sema domain, imr	SEMA3A		5.20	4.88	5.00
218447_at	chromosome 16 o	C16orf61		11.27	11.13	11.34
213497_at	ankyrin repeat an	ABTB2		9.56	9.43	9.40

205970_at	metallothionein 3	MT3	7.28	7.25	6.97
218533_s_at	uridine-cytidine ki	UCKL1	8.40	8.47	8.36
206077_at	Kell blood group,	KEL	5.60	5.67	5.91
221534_at	chromosome 11 c	C11orf68	9.14	9.12	9.02
203234_at	uridine phosphory	UPP1	9.75	9.51	9.61
203081_at	catenin, beta inte	CTNNBIP1	8.14	8.26	7.83
220441_at	DnaJ (Hsp40) hom	DNAJC22	5.50	5.63	5.49
212046_x_at	mitogen-activatec	MAPK3	7.59	7.33	7.41
202997_s_at	lysyl oxidase-like 2	LOXL2	6.42	6.38	6.19
204590_x_at	vacuolar protein s	VPS33A	7.43	7.38	7.41
221808_at	RAB9A, member F	RAB9A	11.22	11.04	11.29
216890_at	---	---	5.22	5.22	4.86
210862_s_at	sarcosine dehydrc	SARDH	5.15	4.79	4.69
211563_s_at	chromosome 19 c	C19orf2	8.83	8.88	9.05
221878_at	chromosome 2 op	C2orf68	7.69	7.73	7.86
210892_s_at	general transcript	GTF2I	5.54	5.75	5.45
206346_at	prolactin receptor	PRLR	3.21	3.31	3.11
216490_x_at	---	---	5.79	5.87	6.03
205940_at	myosin, heavy cha	MYH3	3.94	3.83	3.85
202912_at	adrenomedullin	ADM	8.18	8.24	8.22
212248_at	metadherin	MTDH	10.54	10.51	10.58
213254_at	trinucleotide repe	TNRC6B	7.46	7.50	7.48
215039_at	heparan sulfate 2-	HS2ST1 /// LOC33	3.49	3.40	3.36
218144_s_at	inverted formin, F	INF2	7.78	7.73	7.70
210889_s_at	Fc fragment of IgG	FCGR2B	4.30	4.45	4.33
201379_s_at	tumor protein D52	TPD52L2	10.63	10.60	10.71
204693_at	CDC42 effector pr	CDC42EP1	7.53	7.39	7.26
60528_at	JMJD7-PLA2G4B r	JMJD7-PLA2G4B //	7.70	7.96	7.80
214262_at	thyroid hormone	TRIP6	5.65	5.54	5.73
215385_at	---	---	5.65	6.06	5.71
208389_s_at	solute carrier fam	SLC1A2	5.86	5.98	5.80
201140_s_at	RAB5C, member F	RAB5C	8.94	8.99	8.95
206671_at	S-antigen; retina a	SAG	5.46	5.53	5.35
216455_at	---	---	6.26	6.19	6.08
213959_s_at	RPGRIP1-like	RPGRIP1L	5.95	6.51	6.35
208237_x_at	ADAM metallopep	ADAM22	4.07	4.09	3.77
210702_s_at	prostaglandin I2 (	PTGIS	3.53	3.39	3.89
202325_s_at	ATP synthase, H+	ATP5J	12.23	12.30	12.53
213334_x_at	HAUS augmin-like	HAUS7	8.71	8.70	8.73
220277_at	CXXC finger 4	CXXC4	6.85	6.39	6.19
220615_s_at	fatty acyl CoA red	FAR2	7.12	6.96	7.06
211956_s_at	eukaryotic transla	EIF1	14.08	14.07	14.14
213929_at	exophilin 5	EXPH5	6.69	6.55	6.66
215088_s_at	succinate dehydrc	SDHC	9.44	9.46	9.61
217149_x_at	tyrosine kinase, n	TNK1	6.36	6.09	6.66
214128_at	diacylglycerol lipa	DAGLA	7.09	6.88	7.15
213208_at	KIAA0240	KIAA0240	7.59	7.61	7.79

219456_s_at	Ras and Rab intera	RIN3	5.54	5.23	5.27
214048_at	methyl-CpG bindi	MBD4	6.09	6.08	6.04
210068_s_at	aquaporin 4	AQP4	3.94	3.95	3.83
206877_at	MAX dimerization	MXD1	5.44	5.22	4.85
206392_s_at	retinoic acid recep	RARRES1	3.73	3.69	3.66
215025_at	neurotrophic tyro	NTRK3	4.16	4.15	4.65
211758_x_at	thioredoxin doma	TXNDC9	10.54	10.57	10.54
203661_s_at	tropomodulin 1	TMOD1	6.73	6.98	6.58
204401_at	potassium interm	KCNN4	6.25	6.17	6.18
205791_x_at	zinc finger protein	ZNF230	6.06	5.78	5.93
202642_s_at	transformation/tr	TRRAP	8.84	8.97	8.84
213018_at	GATA zinc finger c	GATAD1	8.29	8.41	8.57
207074_s_at	solute carrier fam	SLC18A1	3.80	4.05	3.65
215153_at	nitric oxide syntha	NOS1AP	5.73	5.80	5.69
34031_i_at	KRIT1, ankyrin rep	KRIT1	8.41	8.80	8.97
207374_at	phospholipid scra	PLSCR2	4.19	3.95	3.67
216208_s_at	activating transcri	ATF6B	6.71	6.77	6.47
216968_at	mannan-binding le	MASP2	5.74	5.75	5.74
217399_s_at	forkhead box O3 /	FOXO3 /// FOXO3E	6.26	6.23	6.03
209813_x_at	TCR gamma altern	TARP	3.72	3.88	3.60
204777_s_at	mal, T-cell differe	MAL	5.64	5.38	5.42
215941_at	---	---	4.53	4.49	4.35
220378_at	t-complex 11 hom	TCP11	4.59	4.46	4.58
206782_s_at	DnaJ (Hsp40) hom	DNAJC4	7.48	7.58	7.38
207193_at	agouti related pro	AGRP	6.73	6.59	6.69
213583_x_at	eukaryotic transla	EEF1A1 /// EEF1A1	14.91	14.88	14.87
216675_at	---	---	5.21	5.19	5.23
206059_at	zinc finger protein	ZNF91	3.93	3.50	3.41
213979_s_at	---	---	8.26	8.37	8.40
214518_at	pyruvate dehydro	PDHA2	4.67	4.90	4.62
203003_at	myocyte enhance	MEF2D	6.97	6.59	6.78
210737_at	tubby homolog (m	TUB	7.59	7.41	7.31
204431_at	transducin-like en	TLE2	7.50	7.32	7.47
221999_at	vaccinia related ki	VRK3	6.84	6.88	6.71
203013_at	ecdysoneless hom	ECD	8.67	8.69	8.83
210476_s_at	prolactin receptor	PRLR	5.18	5.28	5.22
213464_at	similar to hCG174	LOC100291393 ///	6.60	6.45	6.12
212904_at	leucine rich repea	LRRC47	10.47	10.42	10.49
216387_x_at	---	---	6.83	6.79	6.82
211661_x_at	platelet-activating	PTAFR	5.27	5.31	5.58
212341_at	Yip1 domain famil	YIPF6	9.79	9.76	9.91
221177_at	melanoma inhibit	MIA2	3.53	3.22	3.16
213735_s_at	cytochrome c oxic	COX5B	12.60	12.50	12.55
207216_at	tumor necrosis fa	TNFSF8	5.66	5.44	5.56
AFFX-BioB-M_at	---	---	9.42	9.48	9.50
201754_at	cytochrome c oxic	COX6C	12.82	12.91	12.88
208675_s_at	dolichyl-diphosph	DDOST	10.73	10.55	10.73

220863_at	major intrinsic prc	MIP	6.18	6.14	5.95
215333_x_at	glutathione S-tran	GSTM1	7.49	7.41	7.34
201276_at	RAB5B, member F	RAB5B	7.62	7.82	7.92
205386_s_at	Mdm2 p53 bindin	MDM2	4.02	4.21	4.23
206723_s_at	lysophosphatidic a	LPAR2	6.73	6.61	6.43
208287_at	HLA complex grou	HCG9	6.08	6.01	5.99
220470_at	blocked early in tr	BET1L	7.32	7.07	6.97
220859_at	---	---	4.41	4.40	4.26
219265_at	MOB1, Mps One E	MOBKL2B	7.21	7.31	6.97
220325_at	TAF7-like RNA pol	TAF7L	4.50	4.37	4.25
204286_s_at	phorbol-12-myrist	PMAIP1	8.49	8.48	8.91
212292_at	solute carrier fam	SLC7A1	7.93	7.75	7.74
201710_at	v-myb myeloblast	MYBL2	8.78	8.70	8.55
206664_at	sucrase-isomaltas	SI	3.27	3.28	3.08
211354_s_at	leptin receptor	LEPR	4.12	4.65	4.19
206907_at	tumor necrosis fa	TNFSF9	5.40	5.88	5.75
214487_s_at	RAP2A, member c	RAP2A /// RAP2B	7.26	6.96	7.05
214139_at	AT rich interactive	ARID4B	4.40	4.35	4.09
212782_x_at	polymerase (RNA)	POLR2J	11.27	11.21	11.21
220917_s_at	WD repeat domai	WDR19	8.26	8.43	8.33
210694_s_at	midline 1 (Opitz/E	MID1	4.69	5.02	4.98
205161_s_at	peroxisomal bioge	PEX11A	6.89	7.11	6.89
205621_at	alkB, alkylation re	ALKBH1	8.28	8.29	8.44
220383_at	ATP-binding casse	ABCG5	3.73	3.80	3.85
211262_at	proprotein conver	PCSK6	4.13	4.03	4.33
221128_at	ADAM metallopep	ADAM19	5.89	5.74	5.89
218831_s_at	Fc fragment of IgG	FCGRT	6.49	6.41	6.16
202048_s_at	chromobox homo	CBX6	9.72	9.67	9.66
215306_at	---	---	3.56	3.52	3.34
218354_at	trafficking protein	TRAPPC2L	10.71	10.64	10.71
203026_at	zinc finger and BT	ZBTB5	8.58	8.43	8.54
201443_s_at	ATPase, H+ transp	ATP6AP2	11.23	11.25	11.37
221557_s_at	lymphoid enhance	LEF1	6.09	6.07	6.00
204888_s_at	neuralized homolo	NEURL	4.10	4.06	4.04
209800_at	keratin 16	KRT16	5.47	5.51	5.49
208129_x_at	runt-related trans	RUNX1	6.68	6.27	6.39
200086_s_at	cytochrome c oxic	COX4I1	12.78	12.77	12.80
220943_s_at	chromosome 2 op	C2orf56	6.35	6.49	6.47
222358_x_at	---	---	8.11	8.17	8.05
211668_s_at	plasminogen activ	PLAU	5.47	5.72	5.65
203367_at	dual specificity ph	DUSP14	10.30	10.08	10.33
201557_at	vesicle-associated	VAMP2	6.87	6.66	6.79
205000_at	DEAD (Asp-Glu-Ala	DDX3Y	3.20	3.19	3.02
209987_s_at	achaete-scute cor	ASCL1	5.47	5.26	5.30
220963_s_at	chromosome 1 op	C1orf89	7.01	6.96	6.73
211726_s_at	flavin containing r	FMO2	5.02	5.30	4.79
207984_s_at	membrane protei	MPP2	5.90	6.09	5.40

206624_at	ubiquitin specific	USP9Y	3.68	3.46	3.38
209093_s_at	glucosidase, beta,	GBA /// GBAP1	7.81	7.74	7.77
209541_at	insulin-like growth	IGF1	3.64	3.41	3.42
205206_at	Kallmann syndron	KAL1	4.45	4.00	4.10
216722_at	VENT homeobox (	VENTXP1	3.46	3.45	3.49
210059_s_at	mitogen-activatec	MAPK13	5.68	5.44	5.56
207472_at	arginyl-tRNA synt	RARS2	3.30	3.43	3.26
207741_x_at	tryptase alpha/be	TPSAB1	6.25	5.82	5.94
202307_s_at	transporter 1, ATF	TAP1	9.66	9.54	9.57
219416_at	scavenger recepto	SCARA3	7.97	7.91	7.79
220279_at	tripartite motif-co	TRIM17	6.14	5.82	5.47
201734_at	Chloride channel 3	CLCN3	10.18	10.22	10.35
213705_at	---	---	7.32	7.55	7.55
217512_at	kininogen 1	KNG1	4.21	4.10	4.15
205460_at	neuronal PAS don	NPAS2	5.33	5.24	5.46
209262_s_at	nuclear receptor s	NR2F6	10.18	9.99	10.11
213787_s_at	emopamil binding	EBP	10.55	10.66	10.67
210618_at	RAP1 GTPase acti	RAP1GAP	6.75	6.75	6.45
209930_s_at	nuclear factor (er	NFE2	4.18	4.44	4.27
215315_at	zinc finger proteir	ZNF549	3.68	3.45	3.65
220770_s_at	chromosome 5 op	C5orf54	6.56	6.74	6.60
211249_at	G protein-couplec	GPR68	5.74	6.03	5.59
211315_s_at	calcium channel, v	CACNA1G	5.87	5.34	5.49
210610_at	carcinoembryonic	CEACAM1	6.78	6.34	6.37
211213_at	origin recognition	ORC5L	4.22	4.17	3.95
203883_s_at	RAB11 family inte	RAB11FIP2	10.70	10.65	10.80
215318_at	hypothetical gene	CG012	5.89	5.63	5.51
207050_at	calcium channel, v	CACNA2D1	4.58	4.86	4.51
214384_s_at	Dynactin 2 (p50)	DCTN2	5.64	5.29	5.38
220990_s_at	microRNA 21 /// t	MIR21 /// TMEM4	10.39	10.56	10.53
217218_at	wings apart-like h	WAPAL	4.25	4.13	3.78
218497_s_at	ribonuclease H1	RNASEH1	10.92	10.87	11.01
214327_x_at	tumor protein, tra	TPT1	14.19	14.19	14.21
201621_at	neuroblastoma, su	NBL1	8.25	8.30	8.25
211396_at	Fc fragment of IgG	FCGR2C	6.53	6.71	6.55
219163_at	zinc finger proteir	ZNF562	8.15	8.18	8.26
219464_at	carbonic anhydras	CA14	5.59	5.63	5.49
213789_at	similar to hCG204	LOC100292959	7.74	7.79	7.80
204121_at	growth arrest and	GADD45G	6.47	6.22	6.08
218190_s_at	ubiquinol-cytochr	UQCR10	12.35	12.37	12.29
206249_at	mitogen-activatec	MAP3K13	4.35	4.33	4.26
212958_x_at	peptidylglycine al	PAM	9.98	10.01	9.98
216654_at	tenascin XB	TNXB	5.55	5.49	5.20
207055_at	G protein-couplec	GPR37L1	5.74	6.00	6.00
204993_at	guanine nucleotid	GNAZ	6.51	6.93	6.45
217160_at	testis specific prot	TSPY1	5.59	5.45	5.45
207513_s_at	zinc finger proteir	ZNF189	8.38	8.54	8.58

217140_s_at	voltage-depender	VDAC1	11.12	11.14	11.18
206946_at	hyperpolarization	HCN4	6.13	6.19	5.98
221441_at	goosecoid homeo	GSC2	3.56	3.38	3.41
217667_at	SEC14-like 1 pseu	LOC729799	5.95	6.39	6.23
203770_s_at	steroid sulfatase (	STS	4.63	4.22	4.32
222186_at	Zinc finger, AN1-ty	ZFAND6	6.45	6.67	6.65
208149_x_at	DEAD/H (Asp-Glu-	DDX11	7.88	7.95	7.80
217373_x_at	Mdm2 p53 bindin	MDM2	5.06	4.94	5.07
215955_x_at	Rho GTPase activa	ARHGAP26	5.46	5.47	5.37
38707_r_at	E2F transcription	E2F4	8.70	8.86	8.80
216416_at	---	---	5.57	5.73	5.16
220336_s_at	glycoprotein VI (p	GP6	3.90	3.52	3.77
207581_s_at	melanoma antigei	MAGEB4	4.27	4.12	4.19
216212_s_at	dyskeratosis cong	DKC1	10.56	10.42	10.54
204306_s_at	CD151 molecule (	CD151	9.79	9.62	9.57
208917_x_at	NAD kinase	NADK	6.59	6.76	6.31
205727_at	telomerase-associ	TEP1	4.05	4.05	4.00
211580_s_at	phosphoinositide-	PIK3R3	5.02	5.03	4.61
214402_s_at	Sfi1 homolog, spir	SFI1	6.16	6.35	6.17
205670_at	galactose-3-O-sul	GAL3ST1	6.52	6.64	6.15
218415_at	vacuolar protein s	VPS33B	8.12	8.19	8.14
217886_at	epidermal growth	EPS15	9.02	8.97	9.19
215647_at	---	---	3.12	2.98	3.02
202953_at	complement com	C1QB	4.56	4.65	4.70
215596_s_at	ring finger proteir	RNF160	9.35	9.38	9.60
212656_at	Ts translation elor	TSFM	9.68	9.71	9.62
207885_at	S100 calcium bind	S100G	4.49	4.61	4.34
37566_at	KIAA1045	KIAA1045	4.68	4.56	4.54
217436_x_at	major histocompa	HLA-J	9.54	9.30	9.30
215590_x_at	hypothetical LOC1	LOC100128640	5.15	5.18	5.11
220777_at	kinesin family mei	KIF13A	3.68	3.73	3.71
AFFX-r2-Bs-lys-5	---	---	7.33	7.31	7.27
208935_s_at	lectin, galactoside	LGALS8	7.21	7.24	7.42
210066_s_at	aquaporin 4	AQP4	3.20	3.33	3.33
205368_at	family with seque	FAM131B	5.40	5.16	5.12
40189_at	SET nuclear oncog	SET	12.55	12.53	12.69
206695_x_at	zinc finger proteir	ZNF43	4.28	4.12	4.04
215324_at	sema domain, imr	SEMA3D	4.04	3.83	3.85
220950_s_at	KIAA1310	KIAA1310	6.67	6.49	6.40
206102_at	GINS complex sub	GINS1	10.29	10.41	10.47
214498_at	agouti signaling pi	ASIP	5.13	4.92	4.66
202034_x_at	RB1-inducible coil	RB1CC1	8.44	8.61	8.81
214705_at	InaD-like (Drosopl	INADL	3.20	3.24	3.29
218487_at	aminolevulinate d	ALAD	8.22	8.15	8.22
216979_at	nuclear receptor s	NR4A3	4.58	4.51	4.65
202008_s_at	nidogen 1	NID1	4.40	4.63	4.47
216344_at	nephronophthisis	NPHP4	6.56	6.35	6.11

219605_at	zinc finger protein	ZNF3	4.03	4.16	3.98
207959_s_at	dynein, axonemal	DNAH9	5.62	5.64	5.37
217925_s_at	chromosome 6 op	C6orf106	8.49	8.27	8.40
204929_s_at	vesicle-associated	VAMP5	5.80	5.91	5.86
210715_s_at	serine peptidase i	SPINT2	5.13	4.99	4.87
221804_s_at	family with seque	FAM45A /// FAM4	9.97	10.04	10.00
214874_at	plakophilin 4	PKP4	5.25	5.04	4.94
216868_s_at	hypothetical LOC1	LOC100129656	3.57	3.68	3.63
204891_s_at	lymphocyte-speci	LCK	5.09	5.03	4.98
31826_at	FK506 binding pro	FKBP15	8.72	8.83	8.80
211186_s_at	AP2 associated ki	AAK1	5.04	5.05	5.25
204203_at	CCAAT/enhancer	CEBPG	10.13	9.81	10.19
217769_s_at	proteasome matu	POMP	12.67	12.73	12.78
209390_at	tuberous sclerosis	TSC1	8.27	8.21	8.03
220988_s_at	C1q and tumor ne	C1QTNF3	5.85	5.84	6.04
210339_s_at	kallikrein-related	KLK2	5.04	5.33	5.11
221778_at	jumonji C domain	JHDM1D	8.29	8.14	8.34
215300_s_at	flavin containing r	FMO5	3.40	3.28	3.25
218945_at	chromosome 16 c	C16orf68	8.23	8.33	8.41
202407_s_at	PRP31 pre-mRNA	PRPF31	8.03	8.08	7.98
211766_s_at	pancreatic lipase-r	PNLIPRP2	3.70	3.52	3.61
211263_s_at	proprotein conver	PCSK6	7.76	7.27	7.27
207485_x_at	butyrophilin, subf	BTN3A1	6.89	7.23	7.13
203169_at	RGP1 retrograde g	RGP1	8.82	8.82	8.63
220997_s_at	diaphanous homo	DIAPH3	4.17	4.72	4.38
207605_x_at	zinc finger protein	ZNF117	3.98	4.06	3.82
214752_x_at	filamin A, alpha	FLNA	9.29	9.05	9.21
207120_at	zinc finger protein	ZNF667	3.56	3.60	3.42
217887_s_at	epidermal growth	EPS15	9.84	9.77	10.04
204804_at	tripartite motif-co	TRIM21	6.59	6.48	6.39
203718_at	patatin-like phosp	PNPLA6	6.64	6.75	6.52
218173_s_at	Wolf-Hirschhorn s	WHSC1L1	5.60	5.30	5.11
217740_x_at	ribosomal protein	RPL7A	14.98	14.97	14.94
221751_at	pantothenate kina	PANK3	10.47	10.58	10.78
203895_at	phospholipase C,	PLCB4	3.77	3.63	3.50
209374_s_at	immunoglobulin h	IGHM	6.66	6.52	6.75
211267_at	HESX homeobox 1	HESX1	3.98	3.98	4.14
202965_s_at	calpain 6	CAPN6	3.84	4.00	4.03
214664_at	phosphoribosylam	PAICS	5.94	6.27	5.89
221684_s_at	nyctalopin	NYX	4.81	4.72	4.94
213798_s_at	CAP, adenylate cy	CAP1	12.80	12.86	12.78
209441_at	Rho-related BTB d	RHOBTB2	7.42	7.45	7.36
208111_at	arginine vasopres	AVPR2	5.37	4.97	4.91
217025_s_at	drebrin 1	DBN1	7.62	7.47	7.54
206468_s_at	methyltransferase	METTL13	7.71	7.94	7.77
213609_s_at	seizure related 6 h	SEZ6L	3.45	3.65	3.36
210438_x_at	TROVE domain fa	TROVE2	10.42	10.45	10.49

208827_at	proteasome (pros	PSMB6	11.18	11.07	11.06
212366_at	zinc finger protein	ZNF292	6.56	6.89	7.00
209595_at	general transcript	GTF2F2	8.17	8.19	8.57
216446_at	---	---	7.52	7.51	7.22
209559_at	huntingtin interac	HIP1R /// LOC1002	7.06	6.93	6.78
220197_at	ATPase, H+ transp	ATP6V0A4	3.86	3.96	3.72
208861_s_at	alpha thalassemia	ATRX	8.39	8.61	8.59
212454_x_at	heterogeneous nu	HNRPDL	11.03	11.17	11.24
202128_at	KIAA0317	KIAA0317	9.07	9.14	9.07
220411_x_at	podocan-like 1	PODNL1	7.27	7.60	7.20
204989_s_at	integrin, beta 4	ITGB4	5.92	5.86	5.58
220676_at	ADAM metallopep	ADAMTS8	5.22	4.90	5.15
204370_at	CLP1, cleavage an	CLP1	9.05	9.21	9.19
221154_at	tripartite motif-co	TRIM49	3.55	3.08	3.23
218745_x_at	transmembrane p	TMEM161A	9.15	9.04	8.76
221744_at	DDB1 and CUL4 as	DCAF7	9.24	9.00	9.10
214611_at	glutamate recepto	GRIK1	4.17	3.89	3.69
206493_at	integrin, alpha 2b	ITGA2B	6.08	5.93	5.97
204886_at	polo-like kinase 4	PLK4	7.40	7.67	7.69
207637_at	WSC domain cont	WSCD2	4.49	4.67	4.37
207204_at	fascin homolog 2,	FSCN2	5.99	5.79	5.72
40093_at	basal cell adhesion	BCAM	6.33	6.48	6.43
200045_at	ATP-binding casse	ABCF1	9.39	9.40	9.34
208632_at	ring finger protein	RNF10	8.09	8.15	8.15
209495_at	centrosomal prote	CEP250	7.23	7.37	7.47
37226_at	BCL2/adenovirus	BNIP1	7.96	7.88	8.04
217525_at	olfactomedin-like	OLFML1	3.32	3.45	2.98
214292_at	integrin, beta 4	ITGB4	6.27	5.81	5.77
220822_at	---	---	4.09	3.98	4.43
215549_x_at	CTAGE family, me	CTAGE9	6.24	6.34	6.57
200046_at	defender against	DAD1	12.34	12.34	12.28
203138_at	histone acetyltran	HAT1	10.98	11.01	11.14
206539_s_at	cytochrome P450,	CYP4F12	4.91	4.53	4.49
200879_s_at	endothelial PAS d	EPAS1	4.95	4.77	4.74
220894_x_at	PR domain contai	PRDM12	7.48	7.22	6.89
207442_at	colony stimulating	CSF3	7.82	7.55	7.34
212045_at	golgi glycoprotein	GLG1	7.93	7.95	7.81
208213_s_at	potassium voltage	KCNAB1	4.70	4.60	4.68
204223_at	proline/arginine-r	PRELP	5.26	5.45	5.31
215227_x_at	acid phosphatase	ACP1	11.09	11.10	11.14
214801_at	---	---	9.47	9.52	9.51
214576_at	keratin 36	KRT36	4.70	4.46	4.52
214695_at	ubiquitin associat	UBAP2L	7.15	7.38	7.16
221872_at	retinoic acid recep	RARRES1	3.67	3.80	3.86
206764_x_at	metallophosphoe	MPPE1	7.41	7.27	7.50
216811_at	---	---	6.89	6.93	6.60
206920_s_at	GLE1 RNA export	GLE1	7.00	7.31	7.00

221440_s_at	retinoblastoma bi	RBBP9	5.02	5.19	5.08
204644_at	ecto-NOX disulfide	ENOX2	6.04	6.39	6.05
201019_s_at	eukaryotic transla	EIF1AX /// EIF1AXP	11.72	11.84	12.00
216883_x_at	phosphodiesteras	PDE6D	8.37	8.47	8.44
204965_at	group-specific cor	GC	3.38	3.23	3.52
203992_s_at	lysine (K)-specific	KDM6A	6.95	7.25	7.31
37547_at	Bardet-Biedl synd	BBS9	4.69	4.60	4.56
214625_s_at	misshapen-like kir	MINK1	7.23	7.02	6.84
216893_s_at	collagen, type IV,	COL4A3	3.01	3.32	3.41
209329_x_at	HIG1 hypoxia indu	HIGD2A	11.20	11.10	11.08
219361_s_at	apoptosis enhanci	AEN	8.79	8.67	8.84
37950_at	prolyl endopeptid	PREP	8.94	8.74	8.96
221910_at	ets variant 1	ETV1	5.07	5.07	4.70
203217_s_at	ST3 beta-galactos	ST3GAL5	6.49	6.20	6.46
214127_s_at	Serrate RNA effec	SRRT	4.97	4.66	4.97
202483_s_at	RAN binding prote	RANBP1	12.60	12.60	12.58
212122_at	ras homolog gene	RHOQ	5.61	5.75	5.97
219081_at	ankyrin repeat an	ANKHD1	8.92	8.89	8.91
219861_at	DnaJ (Hsp40) hom	DNAJC17	8.15	8.26	8.19
202884_s_at	protein phosphata	PPP2R1B	6.96	7.12	7.05
203134_at	phosphatidylinosi	PICALM	6.86	6.50	6.62
206174_s_at	protein phosphata	PPP6C	8.74	8.84	8.88
210275_s_at	zinc finger, AN1-ty	ZFAND5	11.08	11.10	11.18
216964_at	Ubiquitin specific	USP22	5.37	5.59	5.23
206012_at	left-right determi	LEFTY2	6.33	6.30	6.31
219440_at	retinoic acid induc	RAI2	3.90	4.01	3.77
207046_at	histone cluster 2,	HIST2H4A /// HIST	6.69	6.45	6.39
208167_s_at	matrix metallopep	MMP16	4.31	4.39	4.05
203187_at	dedicator of cytok	DOCK1	7.74	7.74	7.77
218569_s_at	kelch repeat and E	KBTBD4	6.93	7.02	7.09
211097_s_at	pre-B-cell leukemi	PBX2	5.14	5.11	4.69
200007_at	signal recognition	SRP14	13.22	13.34	13.37
213268_at	calmodulin bindin	CAMTA1	4.04	4.62	4.28
214688_at	transducin-like en	TLE4	3.47	3.56	3.27
203660_s_at	pericentrin	PCNT	8.97	9.10	8.91
215454_x_at	surfactant protein	SFTPC	7.03	7.05	7.07
204591_at	cell adhesion mol	CHL1	3.94	3.47	3.42
200625_s_at	CAP, adenylate cy	CAP1	11.50	11.57	11.56
206296_x_at	mitogen-activatec	MAP4K1	5.88	5.90	5.81
207567_at	solute carrier fam	SLC13A2	6.17	5.75	5.77
221993_s_at	Chromosome 16 c	C16orf42	5.97	5.72	5.59
212106_at	Fas associated fac	FAF2	8.69	8.69	8.61
213942_at	multiple EGF-like-	MEGF6	6.75	6.69	6.48
215809_at	cytochrome P450,	CYP2D6	7.41	7.57	7.48
217245_at	early lymphoid ac	EPAG	3.46	3.73	3.80
45297_at	EH-domain contai	EHD2	8.92	8.85	9.00
202538_s_at	chromatin modify	CHMP2B	9.38	9.44	9.44

215090_x_at	hypothetical prote	LOC440434	8.99	9.01	8.92
51176_at	mediator complex	MED27	9.99	9.87	9.93
218477_at	transmembrane p	TMEM14A	11.62	11.77	11.70
211068_x_at	family with seque	FAM21C /// FAM2	9.11	9.17	9.36
207244_x_at	cytochrome P450,	CYP2A6 /// CYP2A	8.56	8.30	8.50
215505_s_at	striatin, calmoduli	STRN3	7.52	7.66	7.67
212726_at	PHD finger protei	PHF2	7.88	7.95	7.84
211018_at	lanosterol synthas	LSS	4.96	5.34	4.81
217330_at	disrupted in schiz	DISC1	4.37	4.15	4.14
212376_s_at	E1A binding prote	EP400	8.41	8.31	8.43
218554_s_at	ash1 (absent, sma	ASH1L	6.08	5.98	5.77
222176_at	phosphatase and	PTEN	3.74	3.83	3.43
215248_at	growth factor rec	GRB10	7.84	7.83	7.77
211941_s_at	phosphatidylethai	PEBP1	12.56	12.51	12.54
207113_s_at	tumor necrosis fa	TNF	4.52	4.56	4.18
217407_x_at	peptidylprolyl isor	PPIL2	8.05	8.07	7.94
220418_at	ubiquitin associat	UBASH3A	4.86	4.48	4.24
217063_x_at	T cell receptor del	TRD@	4.46	4.22	4.32
202443_x_at	Notch homolog 2	NOTCH2	11.26	11.17	11.25
202101_s_at	v-ral simian leuke	RALB	7.15	6.98	7.32
213420_at	DEAH (Asp-Glu-Al	DHX57	7.09	7.44	7.45
210538_s_at	baculoviral IAP re	BIRC3	11.79	11.99	12.13
222216_s_at	mitochondrial ribo	MRPL17	10.63	10.54	10.49
204663_at	malic enzyme 3, N	ME3	4.42	4.36	4.65
46167_at	chromosome 1 op	C1orf175 /// TTC4	9.38	9.47	9.58
221284_s_at	v-src sarcoma (Sch	SRC	5.95	5.97	5.44
217406_at	---	---	6.68	6.39	6.28
210303_at	mab-21-like 2 (C.	MAB21L2	3.41	3.20	3.29
212562_s_at	Cathepsin Z	CTSZ	7.55	7.40	7.31
215136_s_at	exosome compon	EXOSC8	10.99	10.98	11.26
219454_at	EGF-like-domain,	EGFL6	4.08	4.13	4.01
214988_s_at	SON DNA binding	SON	11.17	11.21	11.18
215884_s_at	ubiquilin 2	UBQLN2	10.07	9.97	10.14
635_s_at	protein phosphata	PPP2R5B	6.77	6.79	6.80
210333_at	nuclear receptor s	NR5A1	5.57	5.40	5.17
218827_s_at	centrosomal prote	CEP192	8.46	8.72	8.47
221838_at	kelch-like 22 (Dro	KLHL22	6.54	6.67	6.58
215477_at	---	---	3.65	3.71	3.45
216646_at	defective in sister	DSCC1	6.98	6.98	6.88
39854_r_at	similar to patatin-	LOC100293124 ///	9.25	9.54	9.56
203255_at	F-box protein 11	FBXO11	8.32	8.35	8.57
206818_s_at	cyclin M2	CNNM2	6.24	5.92	5.90
206182_at	zinc finger protein	ZNF134	7.06	7.04	7.04
219862_s_at	nuclear prelamin A	NARF	9.09	9.11	9.11
216224_s_at	histone deacetyla	HDAC6	7.02	7.10	6.86
221905_at	cylindromatosis (t	CYLD	6.95	7.16	7.44
203709_at	phosphorylase kin	PHKG2	7.34	7.42	7.30

214034_at	endoplasmic retic	ERAP1	3.41	3.46	3.11
210281_s_at	zinc finger, MYM-t	ZMYM2	6.00	6.26	5.79
207056_s_at	solute carrier fam	SLC4A8	4.04	3.91	3.89
208849_at	---	---	4.57	4.26	4.00
202595_s_at	leptin receptor ov	LEPROTL1	9.44	9.42	9.55
207232_s_at	DAZ interacting pr	DZIP3	7.26	7.47	7.65
207330_at	pregnancy-zone p	PZP	4.41	4.75	4.74
212556_at	scribbled homolog	SCRIB	10.42	10.34	10.45
209085_x_at	replication factor	RFC1	9.21	9.25	9.31
209478_at	stimulated by reti	STRA13	11.05	11.12	11.12
217844_at	CTD (carboxy-term	CTDSP1	8.89	8.76	8.74
204210_s_at	phosphate cytidyl	PCYT1A	4.91	5.12	5.14
219379_x_at	zinc finger protein	ZNF358	7.30	7.05	7.06
212048_s_at	tyrosyl-tRNA syntl	YARS	11.50	11.24	11.39
204726_at	cadherin 13, H-cad	CDH13	9.85	9.76	9.90
211022_s_at	alpha thalassemia	ATRX	5.01	5.07	4.83
216702_x_at	---	---	7.07	6.91	6.91
202140_s_at	CDC-like kinase 3	CLK3	8.35	8.44	8.38
216581_at	---	---	5.05	4.97	4.90
202379_s_at	natural killer-tumo	NKTR	9.45	9.71	9.51
208454_s_at	plasma glutamate	PGCP	7.14	7.09	7.04
215114_at	SUMO1/sentrin/S	SENP3	5.88	6.11	6.03
209814_at	zinc finger protein	ZNF330	10.93	10.80	10.97
222342_at	---	---	3.18	2.95	3.13
212210_at	integrator comple	INTS1	5.62	5.82	5.78
221264_s_at	TAR DNA binding	TARDBP	8.22	8.39	8.26
216936_at	---	---	5.25	5.05	4.61
220268_at	chromosome 11 o	C11orf67	3.42	3.36	3.47
210400_at	glutamate recepto	GRIN2C	6.69	6.83	6.43
201266_at	thioredoxin reduc	TXNRD1	12.94	12.90	12.92
211527_x_at	vascular endothel	VEGFA	6.53	6.32	6.98
207719_x_at	centrosomal prote	CEP170	9.64	9.77	9.89
34858_at	potassium channe	KCTD2	7.99	7.97	7.93
221812_at	F-box protein 42	FBXO42	7.45	7.47	7.48
204264_at	carnitine palmitoy	CPT2	7.76	7.97	7.96
210806_at	tubulin tyrosine lig	TTLL5	4.43	4.51	4.44
207862_at	uroplakin 2	UPK2	5.63	5.50	5.35
207296_at	zinc finger protein	ZNF343	5.20	5.16	5.26
216016_at	NLR family, pyrin c	NLRP3	3.53	3.64	3.29
209531_at	glutathione transf	GSTZ1	8.17	8.28	8.45
203079_s_at	cullin 2	CUL2	9.59	9.65	9.64
209078_s_at	thioredoxin 2	TXN2	9.57	9.65	9.70
221593_s_at	ribosomal protein	RPL31	9.75	9.83	9.93
205596_s_at	SMAD specific E3	SMURF2	8.58	8.63	8.71
201274_at	proteasome (pros	PSMA5	11.38	11.43	11.42
215703_at	cystic fibrosis tran	CFTR	3.64	3.83	3.52
202588_at	adenylate kinase 1	AK1	7.42	7.50	7.04

204911_s_at	tripartite motif-cc	TRIM3	4.13	4.20	3.87
216197_at	activating transcri	ATF7IP	5.62	5.82	5.78
202902_s_at	cathepsin S	CTSS	4.73	4.75	4.74
206485_at	CD5 molecule	CD5	6.86	7.04	6.83
213767_at	kinase suppressor	KSR1	5.31	5.22	5.22
216724_at	Doublecortin-like	DCLK2	4.08	4.20	3.85
215102_at	dpy-19-like 1 pseu	DPY19L1P1	3.99	4.26	3.72
204081_at	neurogranin (prot	NRGN	6.97	7.12	6.94
214873_at	low density lipopr	LRP5L	6.85	7.00	6.91
215761_at	Dmx-like 2	DMXL2	3.93	4.50	4.09
218978_s_at	solute carrier fam	SLC25A37	6.15	5.93	6.22
215828_at	---	---	7.32	7.46	7.25
201486_at	reticulocalbin 2, E	RCN2	10.58	10.72	10.86
208156_x_at	epiplakin 1	EPPK1	5.19	4.99	4.77
200803_s_at	transmembrane B	TMBIM6	11.52	11.34	11.47
200964_at	ubiquitin-like moc	UBA1	8.66	8.52	8.59
206378_at	secretoglobin, fan	SCGB2A2	3.28	3.34	3.19
201233_at	proteasome (pros	PSMD13	8.88	8.61	8.72
206379_at	eyes absent homc	EYA3	4.62	4.79	4.65
209456_s_at	F-box and WD rep	FBXW11	5.64	5.19	5.63
208280_at	CMT1A duplicatec	CDRT1	4.71	4.54	4.50
217311_at	---	---	4.06	4.11	3.93
217415_at	polymerase (RNA)	POLR2A	4.51	4.62	4.26
210871_x_at	synovial sarcoma,	SSX2IP	7.99	8.03	8.12
204080_at	target of EGR1, m	TOE1	8.69	8.71	8.69
205212_s_at	ArfGAP with coile	ACAP1	7.28	6.87	7.14
200652_at	signal sequence re	SSR2	11.53	11.58	11.50
214870_x_at	hypothetical LOC1	LOC100288442 ///	9.72	9.54	9.58
217372_at	---	---	3.77	3.56	3.28
210909_x_at	lipoprotein, Lp(a)-	LPAL2	4.85	4.79	4.73
207764_s_at	homeodomain int	HIPK3	3.91	3.95	3.89
204909_at	DEAD (Asp-Glu-Al	DDX6	5.29	5.25	5.45
217175_at	UDP glucuronosyl	UGT2B15	3.40	3.40	3.47
202473_x_at	host cell factor C1	HCFC1	7.16	6.77	6.99
206121_at	adenosine monop	AMPD1	3.94	3.80	3.59
212657_s_at	interleukin 1 rece	IL1RN	4.44	4.43	4.16
215438_x_at	G1 to S phase trar	GSPT1	11.69	11.61	11.67
204506_at	protein phosphata	PPP3R1	8.81	8.56	8.78
220412_x_at	potassium channe	KCNK7	6.82	6.92	6.59
216522_at	olfactory receptor	OR2B6	4.04	4.35	4.18
221034_s_at	testis expressed 1	TEX13B	7.21	7.26	6.65
209518_at	SWI/SNF related,	SMARCD1	8.09	8.02	8.08
39549_at	neuronal PAS dom	NPAS2	3.71	3.71	3.73
207655_s_at	B-cell linker	BLNK	3.77	3.90	3.51
218127_at	nuclear transcript	NFYB	9.45	9.51	9.66
206719_at	synaptogyrin 4	SYNGR4	4.02	4.04	4.04
209293_x_at	inhibitor of DNA b	ID4	6.79	6.68	6.62

205303_at	potassium inward	KCNJ8	5.53	5.59	5.48
220862_s_at	ubiquitin specific		USP22	6.05	6.25	6.16
203730_s_at	zinc finger with KF	ZKSCAN5	7.21	7.45	7.58
215023_s_at	peroxisomal bioge	PEX1	6.37	6.67	6.65
207591_s_at	AT rich interactive	ARID1A	5.32	4.98	5.05
206043_s_at	ATPase, Ca++ tran	ATP2C2	4.39	4.35	4.12
217899_at	transmembrane p	TMEM214	7.90	7.77	7.95
221203_s_at	YEATS domain cor	YEATS2	9.05	8.90	9.08
206650_at	IQ motif containir	IQCC	6.60	6.16	6.49
201212_at	legumain	LGMN	9.39	9.50	9.58
215748_at	kelch-like 35 (Dro:	KLHL35	5.64	5.80	5.42
205472_s_at	dachshund homol	DACH1	3.96	4.12	4.02
211624_s_at	dopamine recepto	DRD2	6.75	6.79	6.34
221578_at	Ras association (R	RASSF4	6.26	6.08	5.63
216072_at	---	---	4.23	4.74	4.38
217333_at	---	---	4.08	4.35	4.01
200937_s_at	ribosomal protein	RPL5	14.26	14.25	14.34
220082_at	protein phosphata	PPP1R14D	5.75	6.03	5.60
220805_at	histamine recepto	HRH2	6.93	6.67	6.57
205600_x_at	homeobox B5	HOXB5	4.85	4.97	4.66
216071_x_at	mediator comple›	MED12	7.09	7.10	6.81
208465_at	glutamate recepto	GRM2	6.05	5.87	6.19
206954_at	Wilms tumor upst	WIT1	4.43	4.50	4.20
202775_s_at	splicing factor, arg	SFRS8	8.60	8.47	8.64
117_at	heat shock 70kDa	HSPA6	4.87	4.87	5.11
204908_s_at	B-cell CLL/lympho	BCL3	7.43	7.11	7.43
201804_x_at	tubulin folding co	TBCB	10.71	10.58	10.61
219705_at	glutamine and ser	QSER1	6.30	6.57	6.26
208846_s_at	voltage-depender	VDAC3	11.27	11.36	11.36
221709_s_at	zinc finger proteir	ZNF839	7.38	7.19	7.33
214181_x_at	leukocyte specific	LST1	5.39	5.30	5.28
212666_at	SMAD specific E3	SMURF1	7.44	7.32	7.50
206112_at	ankyrin repeat do	ANKRD7	3.60	3.39	3.18
206273_at	slowmo homolog	SLMO1	7.05	6.93	7.08
207215_at	glutathione S-tran	GSTTP1	6.18	6.13	5.92
218994_s_at	stromal antigen 3-	STAG3L4	6.09	5.90	6.06
215271_at	tenascin N	TNN	5.10	5.05	5.02
204933_s_at	tumor necrosis fa	TNFRSF11B	4.03	3.91	4.15
206515_at	cytochrome P450,	CYP4F3	3.61	3.55	3.42
209400_at	Solute carrier fam	SLC12A4	4.91	4.88	4.73
216649_at	hypothetical prote	LOC100288758	5.42	4.91	5.31
206184_at	v-crk sarcoma viru	CRKL	6.61	7.07	6.69
211207_s_at	acyl-CoA syntheta	ACSL6	3.37	3.52	3.49
212494_at	tensin like C1 dor	TENC1	8.58	8.36	8.14
205720_at	proopiomelanoco	POMC	5.86	6.26	5.90
201114_x_at	proteasome (pros	PSMA7	12.60	12.58	12.57
206244_at	complement com	CR1	4.69	4.61	4.53

204807_at	transmembrane p	TMEM5		8.11	8.24	8.53
211352_s_at	nuclear receptor c	NCOA3		7.72	7.76	7.71
215791_at	---	---		3.89	4.32	4.47
221940_at	RNA pseudouridyl	RPUSD2		8.24	8.22	8.16
215251_at	---	---		5.29	5.40	5.33
204410_at	eukaryotic transla	EIF1AY		3.86	3.30	3.63
215478_at	regulating synapti	RIMS2		3.65	3.92	3.49
219817_at	chromosome 12 c	C12orf47		8.43	8.56	8.43
211809_x_at	collagen, type XIII,	COL13A1		6.47	6.38	6.24
219888_at	sperm associated	SPAG4		7.25	7.32	7.27
207503_at	t-complex 10 hom	TCP10		4.40	4.92	4.80
204721_s_at	DnaJ (Hsp40) hom	DNAJC6		3.56	3.65	3.41
218516_s_at	inositol monopho	IMPAD1		8.77	8.88	8.82
222354_at	F11 receptor	F11R		4.12	3.98	4.00
205216_s_at	apolipoprotein H (	APOH		5.00	4.84	4.99
214714_at	zinc finger proteir	ZNF394		7.30	7.33	7.37
218932_at	zinc finger, HIT typ	ZNHIT6		9.84	9.77	9.85
203977_at	tafazzin	TAZ		7.94	7.87	7.55
218187_s_at	chromosome 8 op	C8orf33		10.00	9.88	10.16
32128_at	chemokine (C-C m	CCL18		3.86	4.08	3.80
219879_s_at	chromosome 17 c	C17orf53		6.33	6.20	6.15
201401_s_at	adrenergic, beta, r	ADRBK1		6.22	5.98	5.78
209871_s_at	amyloid beta (A4)	APBA2		8.18	8.32	8.30
202869_at	processing of prec	POP4		3.99	4.41	4.38
212734_x_at	ribosomal protein	RPL13		14.56	14.52	14.65
201093_x_at	succinate dehydrc	SDHA		11.54	11.50	11.45
218223_s_at	pleckstrin homolo	PLEKHO1		6.90	7.08	6.90
AFFX-HUMGAPD	glyceraldehyde-3-	GAPDH		14.10	14.07	14.14
208560_at	potassium voltage	KCNA10		5.91	5.97	5.55
203585_at	zinc finger proteir	ZNF185		6.03	5.92	6.06
91682_at	---	---		5.91	6.17	5.88
205623_at	aldehyde dehydrc	ALDH3A1		6.17	5.98	6.02
201816_s_at	glioblastoma amp	GBAS		10.28	10.36	10.49
219604_s_at	zinc finger proteir	ZNF3		4.26	4.63	4.54
37860_at	zinc finger proteir	ZNF337		7.43	7.46	7.21
202845_s_at	ralA binding prote	RALBP1		9.08	9.17	9.19
210348_at	septin 4		4-Sep	5.05	5.21	5.01
217202_s_at	glutamate-ammoı	GLUL		8.30	8.18	8.24
221309_at	RNA binding moti	RBM17		7.17	6.97	6.42
220256_s_at	3-oxoacid CoA tra	OXCT2		5.65	5.52	5.62
204713_s_at	coagulation factoı	F5		6.10	5.70	5.45
212064_x_at	MYC-associated zi	MAZ		7.49	7.42	7.39
207820_at	alcohol dehydroge	ADH1A		3.54	3.39	3.41
203978_at	nucleotide binding	NUBP1		8.04	8.05	8.13
211304_x_at	potassium inward	KCNJ5		5.30	5.08	5.16
201174_s_at	telomeric repeat l	TERF2IP		10.10	10.16	10.25
217987_at	asparagine synthe	ASNSD1		10.51	10.58	10.61

64474_g_at	DiGeorge syndron	DGCR8	7.37	7.45	7.21
208110_x_at	mediator complex	MED25	7.39	7.44	7.44
65086_at	Yip1 domain famil	YIPF2	7.68	7.71	7.59
AFFX-HUMGAPD	---	---	14.44	14.44	14.46
216144_at	---	---	4.40	4.55	4.34
217603_at	ATPase, H+ transp	ATP6V0A2	5.69	5.81	5.55
210753_s_at	EPH receptor B1	EPHB1	4.72	4.76	4.66
207027_at	HGF activator	HGFAC	5.51	5.10	5.14
206293_at	sulfotransferase fa	SULT2A1	3.65	3.35	3.49
210938_at	pancreatic and du	PDX1	4.88	4.69	4.34
214049_x_at	CD7 molecule	CD7	5.92	5.83	5.62
212834_at	DEAD (Asp-Glu-Ala	DDX52	7.74	7.96	7.86
212949_at	non-SMC condens	NCAPH	9.12	9.11	9.15
209054_s_at	Wolf-Hirschhorn s	WHSC1	10.12	10.04	10.16
222028_at	zinc finger proteir	ZNF45	7.70	7.80	7.81
205562_at	ribonuclease P/M	RPP38	8.80	8.88	8.92
219184_x_at	translocase of inn	TIMM22	7.42	7.45	7.38
215163_at	---	---	6.37	6.40	6.30
218681_s_at	stromal cell-deriv	SDF2L1	10.03	9.92	9.86
213780_at	trichohyalin	TCHH	3.77	3.78	3.61
215065_at	PHD finger protei	PHF8	3.46	3.61	3.51
217493_x_at	natural cytotoxicit	NCR2	7.37	7.17	7.18
206223_at	lemur tyrosine kir	LMTK2	4.91	5.33	5.04
216900_s_at	cholinergic recept	CHRNA4	6.44	6.14	6.11
200670_at	X-box binding pro	XBP1	10.33	9.97	10.50
206999_at	interleukin 12 rec	IL12RB2	3.68	3.60	3.72
218853_s_at	motile sperm dom	MOSPD1	8.51	8.72	8.74
205222_at	enoyl-CoA, hydrat	EHHADH	6.58	6.95	6.99
203195_s_at	nucleoporin 98kD	NUP98	9.22	9.23	9.31
216571_at	hypothetical prote	LOC100289043	6.63	6.55	6.37
206110_at	histone cluster 1,	HIST1H3H	6.42	6.42	6.24
214385_s_at	similar to mucin /,	LOC100293983 ///	5.67	5.91	5.57
214786_at	mitogen-activated	MAP3K1	4.26	4.40	4.33
205776_at	flavin containing r	FMO5	4.25	4.04	3.58
203047_at	serine/threonine	STK10	7.47	7.43	7.35
212178_s_at	POM121 membra	POM121 /// POM1	9.30	9.44	9.43
210318_at	retinol binding pro	RBP3	6.63	6.72	6.40
220235_s_at	chromosome 1 op	C1orf103	9.50	9.61	9.70
222127_s_at	exocyst complex c	EXOC1	10.07	10.18	10.21
212895_s_at	active BCR-relatec	ABR	7.84	7.92	7.69
209826_at	EGF-like-domain,	EGFL8 /// PPT2	6.88	6.97	6.74
217216_x_at	mutL homolog 3 (	MLH3	6.29	6.15	6.17
208505_s_at	fucosyltransferase	FUT2	5.95	5.78	5.98
221414_s_at	defensin, beta 12(	DEFB126	4.07	4.16	3.68
206933_s_at	hexose-6-phospha	H6PD	6.10	5.89	5.85
222356_at	---	---	5.42	5.42	4.97
214369_s_at	RAS guanyl releas	RASGRP2	4.99	5.03	4.70

216943_at	---	---	6.27	6.10	5.91
215221_at	---	---	7.19	7.04	7.03
215170_s_at	centrosomal prote	CEP152	6.03	6.37	6.12
220405_at	syntrophin, gamm	SNTG1	3.18	3.37	3.24
220720_x_at	family with seque	FAM128B	8.35	8.38	8.35
216403_at	Sp3 transcription	SP3P	4.99	4.76	4.52
216698_x_at	similar to hCG204	LOC100290714 ///	6.35	6.47	6.09
221650_s_at	mediator complex	MED18	7.97	8.00	7.83
218317_x_at	GIY-YIG domain co	GIYD1 /// GIYD2	8.80	8.95	8.77
210126_at	pregnancy specific	PSG9	6.27	5.90	6.24
221687_s_at	family with seque	FAM125B	6.43	6.41	6.43
221151_at	PR domain contai	PRDM9	4.67	4.27	4.40
209542_x_at	insulin-like growth	IGF1	6.02	5.94	6.08
204304_s_at	prominin 1	PROM1	3.60	3.55	3.26
217681_at	similar to wingless	LOC100289775 ///	6.84	6.78	6.46
200037_s_at	chromobox homo	CBX3	12.38	12.60	12.65
208730_x_at	RAB2A, member R	RAB2A	8.49	8.41	8.61
201894_s_at	signal sequence re	SSR1	10.56	10.59	10.65
211025_x_at	cytochrome c oxic	COX5B	12.00	11.99	12.19
221086_s_at	FEZ family zinc fin	FEZF2	3.64	3.41	3.08
221790_s_at	low density lipopr	LDLRAP1	6.88	6.84	6.86
206134_at	ADAM-like, decysi	ADAMDEC1	3.32	3.45	3.35
203319_s_at	zinc finger protein	ZNF148	8.41	8.48	8.56
213833_x_at	NOP16 nucleolar p	NOP16	6.63	6.63	6.70
204384_at	golgin A2	GOLGA2	6.85	6.79	6.93
208487_at	LIM homeobox tra	LMX1B	5.50	5.37	5.34
219711_at	zinc finger protein	ZNF586	7.03	7.32	7.25
211044_at	tripartite motif-co	TRIM14	4.18	4.07	4.07
219511_s_at	synuclein, alpha in	SNCAIP	5.10	4.98	4.84
220074_at	cadherin-related f	CDHR5	6.43	6.15	5.92
209754_s_at	thymopoietin	TMPO	7.07	7.02	7.34
211831_s_at	thrombopoietin	THPO	6.05	5.88	6.00
201788_at	DEAD (Asp-Glu-Ala	DDX42	9.94	9.94	9.98
220570_at	resistin	RETN	6.82	6.50	6.40
210665_at	tissue factor path	TFPI	6.73	6.79	6.81
207603_at	solute carrier fam	SLC4A7	3.98	3.86	3.85
218729_at	latexin	LXN	3.60	3.58	3.11
221753_at	slingshot homolog	SSH1	8.70	8.54	8.72
219920_s_at	GDP-mannose pyr	GMPPB	8.93	9.06	8.88
203902_at	hephaestin	HEPH	4.08	4.09	3.84
202537_s_at	chromatin modify	CHMP2B	9.59	9.62	9.72
204816_s_at	DEAH (Asp-Glu-Ala	DHX34	4.20	4.13	4.24
221451_s_at	olfactory receptor	OR2W1	3.59	3.45	3.69
208525_s_at	olfactory receptor	OR2F1 /// OR2F2	4.01	4.12	3.78
209201_x_at	chemokine (C-X-C	CXCR4	4.11	4.42	4.26
219740_at	vasohibin 2	VASH2	5.00	4.81	4.52
206071_s_at	EPH receptor A3	EPHA3	4.25	4.20	4.19

209638_x_at	regulator of G-prc	RGS12	4.26	4.01	4.20
220502_s_at	solute carrier fam	SLC13A1	3.48	3.43	3.39
217441_at	---	---	4.17	4.35	4.07
214570_x_at	hypothetical prote	LOC100294050 ///	6.94	6.67	6.46
207319_s_at	cyclin-dependent	CDK13	5.33	5.57	5.28
216466_at	neuron navigator	NAV3	4.40	4.27	4.62
221798_x_at	---	---	15.20	15.15	15.15
215987_at	Rap guanine nucle	RAPGEF2	4.43	4.14	3.86
207886_s_at	calcitonin recepto	CALCR	3.84	3.86	3.86
202204_s_at	autocrine motility	AMFR	9.32	9.24	9.34
209777_s_at	solute carrier fam	SLC19A1	9.11	9.03	8.76
39318_at	T-cell leukemia/ly	TCL1A	4.10	4.25	4.06
206561_s_at	aldo-keto reducta	AKR1B10	4.11	4.32	4.42
219787_s_at	epithelial cell tran	ECT2	10.71	11.04	11.21
206034_at	serpin peptidase i	SERPINB8	6.42	6.47	6.48
206908_s_at	claudin 11	CLDN11	7.41	7.33	6.75
215922_at	RALBP1 associate	REPS1	4.03	4.20	3.92
208882_s_at	ubiquitin protein l	UBR5	9.59	9.55	9.63
214195_at	tripeptidyl peptid	TPP1	6.99	7.08	6.49
217074_at	spermine oxidase	SMOX	6.30	6.03	6.05
210401_at	purinergic recepto	P2RX1	6.31	6.22	6.19
201931_at	electron-transfer-	ETFA	11.84	11.75	11.84
203338_at	protein phosphata	PPP2R5E	10.73	10.93	10.92
207679_at	paired box 3	PAX3	6.52	6.59	6.24
213958_at	CD6 molecule	CD6	8.17	7.99	7.72
220152_at	chromosome 10 c	C10orf95	6.42	6.34	6.14
219739_at	ring finger protein	RNF186	6.00	5.96	5.61
217593_at	zinc finger and SC	ZSCAN18	5.40	5.42	5.37
201180_s_at	guanine nucleotid	GNAI3	12.07	12.24	12.18
218325_s_at	death inducer-obl	DIDO1	7.56	7.41	7.33
212540_at	cell division cycle	CDC34	8.43	8.45	8.46
219686_at	serine/threonine l	STK32B	4.48	4.70	4.76
204874_x_at	BAI1-associated p	BAIAP3	7.14	7.15	6.85
220614_s_at	chromosome 6 op	C6orf103	3.54	3.57	3.39
214973_x_at	immunoglobulin h	IGHD /// LOC1002	5.86	5.86	5.40
212250_at	metadherin	MTDH	11.27	11.32	11.57
218621_at	HemK methyltran	HEMK1	5.51	5.73	5.52
219360_s_at	transient receptor	TRPM4	6.66	6.26	6.14
202889_x_at	alanyl (membrane	ANPEP	5.73	5.47	5.18
217450_at	ArfGAP with FG re	AGFG2	5.55	5.85	5.26
213766_x_at	guanine nucleotid	GNA11	9.36	9.38	9.43
203535_at	S100 calcium bind	S100A9	5.42	5.39	5.13
220790_s_at	membrane-spann	MS4A5	4.27	4.15	4.23
207304_at	zinc finger protein	ZNF45	6.24	6.21	6.04
215234_at	---	---	4.52	4.52	3.99
201815_s_at	TBC1 domain fam	TBC1D5	7.02	7.19	7.24
206062_at	guanylate cyclase	GUCA1A	5.07	4.85	4.56

207783_x_at	HECT, UBA and W	HUWE1	14.83	14.82	14.82
216327_s_at	sialic acid binding	SIGLEC8	4.74	4.53	4.53
211910_at	---	---	4.31	4.42	4.62
202940_at	WNK lysine deficie	WNK1	6.54	6.57	6.57
211927_x_at	eukaryotic transla	EEF1G /// TUT1	14.71	14.62	14.63
200021_at	cofilin 1 (non-mus	CFL1	13.56	13.56	13.46
214968_at	DEAD (Asp-Glu-Ala	DDX51	6.02	5.90	5.97
212952_at	---	---	11.01	11.00	11.03
200781_s_at	ribosomal protein	RPS15A	15.07	15.04	15.09
204106_at	testis-specific kina	TESK1	8.02	7.95	7.64
219155_at	phosphatidylinosi	PITPNC1	6.05	6.22	6.23
208576_s_at	histone cluster 1,	HIST1H3B	5.60	5.72	5.48
214936_at	leucine-rich repea	LRCH1	5.20	5.25	4.93
211841_s_at	tumor necrosis fac	TNFRSF25	5.73	5.79	5.49
209741_x_at	S-phase cyclin A-a	SCAPER	7.02	7.08	7.38
220576_at	post-GPI attachme	PGAP1	5.67	5.86	5.82
205650_s_at	fibrinogen alpha c	FGA	4.95	4.71	4.53
211825_s_at	Ewing sarcoma br	EWSR1 /// FLI1	5.36	5.26	4.86
214923_at	ATPase, H+ transp	ATP6V1D	4.53	4.75	4.39
203209_at	replication factor	RFC5	9.90	9.79	10.04
202256_at	CD2 (cytoplasmic	CD2BP2	7.88	7.94	7.80
210648_x_at	sorting nexin 3	SNX3	12.46	12.52	12.47
220061_at	acyl-CoA syntheta	ACSM5	4.99	4.88	4.55
216420_at	---	---	4.04	3.80	3.88
214624_at	uroplakin 1A	UPK1A	4.09	4.30	4.22
203583_at	unc-50 homolog (	UNC50	9.42	9.49	9.61
206728_at	endothelin conver	ECE2	6.58	6.16	6.37
204792_s_at	intraflagellar trans	IFT140	5.63	5.84	5.34
212916_at	PHD finger protein	PHF8	7.45	7.27	7.32
218062_x_at	CDC42 effector pr	CDC42EP4	8.50	8.28	8.43
208766_s_at	heterogeneous nu	HNRNPR	12.66	12.76	12.71
219449_s_at	transmembrane p	TMEM70	10.47	10.44	10.45
205277_at	PR domain contai	PRDM2	6.26	6.49	5.81
222173_s_at	TBC1 domain fam	TBC1D2	7.04	7.04	6.98
208820_at	PTK2 protein tyro	PTK2	10.54	10.57	10.78
215803_at	chromosome X op	CXorf57	3.24	3.20	3.24
215691_x_at	heat shock protein	HSPB11	10.41	10.53	10.63
215697_at	---	---	6.97	7.08	6.67
37796_at	similar to FLJ0024	LOC100290658 ///	7.25	7.38	7.17
207477_at	---	---	3.57	3.42	3.38
200785_s_at	low density lipopr	LRP1	7.12	6.83	6.87
208037_s_at	mucosal vascular	MADCAM1	4.95	5.21	4.91
212779_at	KIAA1109	KIAA1109	8.99	9.26	9.21
216354_at	---	---	6.82	6.78	6.81
209682_at	Cas-Br-M (murine	CBLB	9.14	8.90	9.19
217630_at	angel homolog 2 (	ANGEL2	5.36	5.59	5.44
218150_at	ADP-ribosylation f	ARL5A	10.05	10.26	10.35

209385_s_at	proline synthetase	PROSC	9.82	9.93	9.91
221217_s_at	ataxin 2-binding p	A2BP1	6.00	6.01	5.47
209124_at	myeloid differenti	MYD88	9.12	9.45	9.33
221410_x_at	protocadherin bet	PCDHB3	6.08	6.31	6.10
206058_at	solute carrier fam	SLC6A12	4.86	4.92	4.71
201390_s_at	casein kinase 2, b	CSNK2B	11.24	11.28	11.33
218558_s_at	mitochondrial rib	MRPL39	10.05	10.11	10.09
221476_s_at	ribosomal protein	RPL15	13.03	13.05	13.08
220134_x_at	family with seque	FAM176B	6.92	6.73	6.72
204235_s_at	GULP, engulfment	GULP1	3.27	3.50	3.13
216133_at	T cell receptor del	TRD@	5.13	5.06	5.24
206261_at	zinc finger protein	ZNF239	3.78	3.45	3.88
219063_at	chromosome 1 op	C1orf35	8.26	8.20	8.20
210506_at	fucosyltransferase	FUT7	4.71	4.67	4.43
218708_at	NTF2-like export f	NXT1	10.86	10.66	10.79
209138_x_at	Immunoglobulin l	IGL@	6.08	6.04	5.95
211466_at	nuclear factor I/B	NFIB	5.00	5.01	4.83
208784_s_at	kelch domain con	KLHDC3	7.49	7.42	7.18
207363_at	retinoschisin 1	RS1	3.72	3.54	3.50
220571_at	PR domain contai	PRDM11	6.79	6.71	6.63
203595_s_at	interferon-induce	IFIT5	8.51	8.49	8.75
211705_s_at	sorbin and SH3 dc	SORBS1	7.13	6.74	6.55
206963_s_at	N-acetyltransfera	NAT8 /// NAT8B	5.99	5.77	5.61
209968_s_at	neural cell adhesi	NCAM1	6.46	6.56	5.95
209740_s_at	patatin-like phosp	PNPLA4	3.56	3.61	3.50
210941_at	protocadherin 7	PCDH7	3.36	3.38	3.31
213524_s_at	G0/G1switch 2	G0S2	5.21	5.18	5.02
215589_at	---	---	3.88	4.26	3.78
214459_x_at	major histocompa	HLA-C	11.55	11.39	11.44
203760_s_at	Src-like-adaptor	SLA	7.03	6.77	6.62
205864_at	solute carrier fam	SLC7A4	5.92	6.06	5.79
211188_at	CD84 molecule	CD84	5.30	4.99	4.89
222362_at	ArfGAP with FG re	AGFG2	6.30	6.29	6.31
201218_at	C-terminal binding	CTBP2	11.65	11.67	11.78
204071_s_at	topoisomerase I b	TOPORS	8.88	9.18	9.26
207322_at	intersectin 1 (SH3	ITSN1	4.43	4.47	4.57
208136_s_at	hypothetical LOC8	MGC3771	5.55	5.23	5.08
206128_at	adrenergic, alpha-	ADRA2C	6.79	6.43	6.27
206449_s_at	mannan-binding le	MASP1	3.93	3.76	3.63
202591_s_at	single-stranded D	SSBP1	12.30	12.40	12.42
200972_at	tetraspanin 3	TSPAN3	11.23	11.29	11.34
208447_s_at	phosphoribosyl py	PRPS1	9.12	9.25	9.25
201607_at	PWP1 homolog (S	PWP1	9.04	9.14	9.09
220643_s_at	Fas apoptotic inhi	FAIM	9.41	9.44	9.70
219007_at	nucleoporin 43kD	NUP43	11.10	11.15	11.28
207881_at	---	---	3.48	3.28	3.30
214584_x_at	acetyl-CoA carbox	ACACB	3.91	3.95	3.59

219702_at	placenta-specific :	PLAC1	6.19	6.18	6.30
211616_s_at	5-hydroxytryptam	HTR2A	4.48	4.25	4.13
210707_x_at	postmeiotic segre	PMS2L11	7.82	7.80	7.86
203476_at	trophoblast glyco	TPBG	11.14	11.02	11.09
212512_s_at	coactivator-associ	CARM1	7.87	7.93	7.98
206208_at	carbonic anhydras	CA4	5.93	5.82	5.97
221703_at	BRCA1 interacting	BRIP1	4.76	5.15	4.93
212355_at	KH and NYN doma	KHNYN	8.51	8.50	8.60
216408_at	olfactory receptor	OR2B2	3.36	3.37	3.39
823_at	chemokine (C-X3-	CX3CL1	5.12	5.12	5.10
213154_s_at	bicaudal D homol	BICD2	9.35	9.42	9.49
208164_s_at	interleukin 9 rece	IL9R	4.86	4.75	4.71
200719_at	S-phase kinase-as	SKP1	8.28	8.37	8.34
204978_at	splicing factor, arg	SFRS16	8.72	8.69	8.51
205419_at	G protein-couplec	GPR183	3.25	3.44	3.08
208986_at	transcription facto	TCF12	9.94	9.85	10.08
217863_at	protein inhibitor c	PIAS1	8.31	8.17	8.19
202035_s_at	secreted frizzled-r	SFRP1	3.93	4.23	3.94
202362_at	RAP1A, member c	RAP1A	10.55	10.61	10.71
204123_at	ligase III, DNA, ATI	LIG3	7.56	7.37	7.46
204842_x_at	protein kinase, cA	PRKAR2A	10.68	10.69	10.58
204734_at	keratin 15	KRT15	5.15	5.03	4.94
200074_s_at	ribosomal protein	RPL14	14.01	13.98	14.02
217748_at	adiponectin recep	ADIPOR1	8.79	8.86	8.98
55705_at	chromosome 19 c	C19orf22	10.01	10.01	9.98
201919_at	solute carrier fam	SLC25A36	10.85	10.80	10.86
215542_at	---	---	3.43	3.60	3.42
216802_at	---	---	3.95	4.00	3.65
207839_s_at	transmembrane p	TMEM8B	7.29	7.05	7.10
205253_at	pre-B-cell leukemi	PBX1	5.64	6.19	5.41
205002_at	AT hook, DNA bin	AHDC1	6.55	7.01	6.70
208147_s_at	cytochrome P450,	CYP2C8	3.60	3.51	3.40
214043_at	protein tyrosine p	PTPRD	6.48	6.49	7.00
213975_s_at	lysozyme	LYZ	3.31	3.28	3.65
206456_at	gamma-aminobut	GABRA5	3.17	2.82	3.07
209181_s_at	Rab geranylgeran	RABGGTB	12.22	12.34	12.40
219318_x_at	mediator complex	MED31	4.94	5.59	5.07
216699_s_at	kallikrein 1	KLK1	7.27	6.97	7.02
200882_s_at	proteasome (pros	PSMD4	10.98	10.90	11.09
214155_s_at	La ribonucleoprot	LARP4	7.36	7.34	7.56
221310_at	fibroblast growth	FGF14	4.54	4.47	4.34
215620_at	ras responsive ele	RREB1	5.67	5.90	5.50
203499_at	EPH receptor A2	EPHA2	9.64	9.57	9.66
221977_at	T-box 2	TBX2	4.84	4.84	4.77
217425_at	---	---	3.53	3.51	3.76
207600_at	potassium voltage	KCNC3	4.77	5.01	4.50
215968_at	---	---	4.45	4.57	4.57

204618_s_at	GA binding protei	GABPB1	8.77	8.80	8.94
218028_at	elongation of very	ELOVL1	9.87	9.73	9.78
201822_at	translocase of inn	TIMM17A	6.09	6.21	6.04
203854_at	complement facto	CFI	3.60	3.65	3.75
206651_s_at	carboxypeptidase	CPB2	3.55	3.38	3.42
203477_at	collagen, type XV,	COL15A1	3.38	3.44	3.28
212420_at	E74-like factor 1 (	ELF1	10.35	10.38	10.29
215035_at	immunoglobulin la	IGLV6-57	4.74	4.25	4.31
205051_s_at	v-kit Hardy-Zucker	KIT	4.03	4.26	4.18
216678_at	intraflagellar trans	IFT122	6.58	6.79	6.72
207641_at	tumor necrosis fa	TNFRSF13B	6.91	6.96	6.96
217086_at	cholinergic recept	CHRNB3	3.90	3.75	3.98
213245_at	adenylate cyclase	ADCY1	5.30	4.83	4.80
215882_at	Centrosomal prot	CEP152	5.90	5.67	5.55
210549_s_at	chemokine (C-C m	CCL23	3.35	3.39	3.29
206511_s_at	SIX homeobox 2	SIX2	4.60	4.35	4.07
211067_s_at	growth arrest-spe	GAS7	4.90	4.88	4.32
219472_at	centromere prote	CENPO	7.28	6.95	7.20
202191_s_at	growth arrest-spe	GAS7	5.84	5.44	5.64
206633_at	cholinergic recept	CHRNA1	4.07	3.73	3.61
209727_at	GM2 ganglioside a	GM2A	6.30	6.67	6.23
204696_s_at	cell division cycle	CDC25A	6.43	6.37	6.42
214219_x_at	mitogen-activatec	MAP4K1	6.39	6.17	6.06
202238_s_at	nicotinamide N-m	NNMT	11.90	11.71	11.83
209845_at	makorin ring finge	MKRN1	8.62	8.55	8.77
220970_s_at	keratin associatec	KRTAP2-4	3.64	3.37	3.91
216294_s_at	KIAA1109	KIAA1109	4.75	4.83	4.55
216195_at	Ankyrin 2, neuron	ANK2	4.78	4.73	5.15
204603_at	exonuclease 1	EXO1	8.08	8.06	8.29
215677_s_at	BRF1 homolog, su	BRF1	6.69	6.52	6.59
202514_at	discs, large homol	DLG1	9.13	9.19	9.34
220203_at	bone morphogene	BMP8A	7.30	7.33	6.94
202012_s_at	exostosin 2	EXT2	9.92	9.84	9.88
219233_s_at	gasdermin B	GSDMB	6.41	6.07	6.06
220632_s_at	protein-O-manno:	POMT2	8.01	7.80	7.60
218248_at	family with seque	FAM111A	9.94	9.89	10.06
201205_at	hypothetical prot	LOC100287025	6.63	7.17	6.92
212459_x_at	succinate-CoA liga	SUCLG2	10.42	10.37	10.62
207790_at	leucine rich repea	LRRC1	5.38	5.46	5.39
213431_x_at	Sfi1 homolog, spir	SFI1	7.43	7.79	7.47
220869_at	ubiquitin-like moc	UBA6	4.22	4.44	4.55
206186_at	membrane protei	MPP3	6.10	6.43	6.03
217570_x_at	---	---	6.17	6.07	5.97
AFFX-CreX-5_at	---	---	14.00	14.04	13.99
220151_at	chromosome 19 c	C19orf73	6.95	6.82	7.06
218585_s_at	denticleless homc	DTL	10.22	10.14	10.48
220664_at	small proline-rich	SPRR2C	3.85	4.29	3.95

207785_s_at	recombination sig	RBPJ	10.31	10.41	10.56
212306_at	cytoplasmic linker	CLASP2	8.78	8.82	8.91
219551_at	ELL associated fac	EAF2	5.51	5.92	5.58
222380_s_at	Programmed cell	PDCD6	8.37	8.54	8.36
219189_at	F-box and leucine	FBXL6	6.70	6.95	6.88
215839_at	leukocyte immun	LILRA5	4.67	4.94	4.88
208064_s_at	ST8 alpha-N-acety	ST8SIA3	4.01	3.87	4.07
211729_x_at	biliverdin reducta	BLVRA	11.06	11.07	11.03
31874_at	growth arrest-spe	GAS2L1	9.17	9.26	9.24
205250_s_at	centrosomal prot	CEP290	7.76	7.97	8.12
216496_s_at	---	---	4.36	3.96	4.40
220454_s_at	sema domain, tra	SEMA6A	4.94	4.92	4.99
207643_s_at	tumor necrosis fa	TNFRSF1A	10.24	10.20	10.15
220613_s_at	synaptotagmin-lik	SYTL2	3.64	3.65	3.69
216414_at	---	---	4.11	3.72	3.49
220579_at	hypothetical prot	FLJ14100	4.98	5.29	4.90
204615_x_at	isopentenyl-diphc	IDI1	11.33	11.69	11.42
218618_s_at	fibronectin type II	FNDC3B	10.80	10.56	10.64
219022_at	chromosome 12 c	C12orf43	7.58	7.72	7.89
203095_at	mitochondrial tra	MTIF2	11.18	11.24	11.26
213965_s_at	chromodomain he	CHD5	6.23	6.03	5.79
203743_s_at	thymine-DNA glyc	TDG	9.77	9.82	9.85
207182_at	gamma-aminobut	GABRA6	3.23	3.33	3.06
205155_s_at	spectrin, beta, no	SPTBN2	7.10	6.94	6.85
221178_at	BAI1-associated p	BAIAP2L2	3.51	3.51	3.18
221594_at	chromosome 7 op	C7orf64	3.49	3.67	3.61
218714_at	proline rich 14	PRR14	8.52	8.48	8.48
207282_s_at	myogenin (myoge	MYOG	4.69	4.54	4.47
211759_x_at	tubulin folding co	TBCB	10.77	10.70	10.78
209480_at	major histocompa	HLA-DQB1	3.29	3.46	3.27
216789_at	---	---	5.74	5.70	5.58
220084_at	chromosome 14 c	C14orf105	3.04	3.34	3.09
221130_s_at	---	---	4.77	4.48	4.34
204267_x_at	protein kinase, m	PKMYT1	7.89	7.93	7.90
216846_at	cyclosporin A tran	CYAT1 /// IGLV2-1	5.47	4.95	5.20
203342_at	translocase of inn	TIMM17B	8.79	8.78	8.89
215886_x_at	ubiquitin specific	USP12	5.40	5.03	4.99
217324_at	---	---	5.19	5.16	4.95
218650_at	DiGeorge syndron	DGCR8	6.64	6.76	6.66
211220_s_at	heat shock transc	HSF2	5.44	5.09	5.23
204103_at	chemokine (C-C m	CCL4	4.54	4.49	4.53
204839_at	processing of pre	POP5	10.81	10.90	10.84
206772_at	parathyroid horm	PTH2R	3.35	3.39	3.34
218068_s_at	zinc finger protei	ZNF672	8.58	8.55	8.65
210232_at	cell division cycle	CDC42	4.25	4.87	4.31
215312_at	---	---	3.47	3.52	3.11
215459_at	cystinosis, nephro	CTNS	4.58	4.48	4.38

209238_at	syntaxin 3	STX3	8.66	8.93	8.83
212715_s_at	microtubule assoc	MICAL3	7.96	7.87	8.01
46270_at	ubiquitin associat	UBAP1	8.65	8.54	8.81
211066_x_at	protocadherin gar	PCDHGA1 /// PCDI	7.79	7.81	7.61
211519_s_at	kinesin family mei	KIF2C	8.54	8.55	8.61
206463_s_at	dehydrogenase/re	DHRS2	7.13	6.93	6.77
205361_s_at	prefoldin subunit	PFDN4	10.04	10.17	10.35
203137_at	Wilms tumor 1 as:	WTAP	11.79	11.68	11.94
215357_s_at	polymerase (DNA-	POLDIP3	6.83	6.91	6.73
215825_at	---	---	5.44	5.64	5.41
202154_x_at	tubulin, beta 3	TUBB3	10.99	11.08	11.02
207931_s_at	6-phosphofructo-:	PFKFB2	7.40	7.30	6.93
214965_at	spermatogenesis a	SPATA2L	8.20	7.94	8.15
205695_at	serine dehydratas	SDS	7.14	7.24	6.74
200828_s_at	zinc finger proteir	ZNF207	11.17	11.15	11.13
209941_at	receptor (TNFRSF)	RIPK1	6.30	6.48	6.02
210773_s_at	formyl peptide re	FPR2	5.26	5.44	5.28
207909_x_at	deleted in azoosp	DAZ1 /// DAZ2 ///	3.44	3.11	3.28
202122_s_at	perilipin 3	PLIN3	10.82	10.95	10.83
213491_x_at	ribophorin II	RPN2	12.00	11.93	12.04
219597_s_at	dual oxidase 1	DUOX1	5.32	4.82	4.93
217237_at	---	---	4.13	3.87	3.87
202227_s_at	bromodomain cor	BRD8	10.41	10.54	10.36
205149_s_at	chloride channel 4	CLCN4	4.61	4.59	4.44
209488_s_at	RNA binding prote	RBPMS	7.71	7.79	7.90
202716_at	protein tyrosine p	PTPN1	9.97	10.00	9.96
214363_s_at	matrin 3	MATR3	12.77	12.84	12.87
208738_x_at	similar to SMT3 su	LOC728825 /// SU	11.70	11.87	11.91
219092_s_at	inositol 1,3,4,5,6-p	IPPK	8.34	8.33	8.18
204607_at	3-hydroxy-3-meth	HMGCS2	4.83	4.71	4.45
58994_at	coiled-coil and C2	CC2D1A	6.11	5.96	5.89
219798_s_at	methylphosphate	MEPCE	9.57	9.53	9.48
208721_s_at	anaphase promot	ANAPC5	8.69	8.85	8.83
222296_at	---	---	5.03	4.83	5.26
206259_at	protein C (inactiva	PROC	5.34	5.17	5.36
208606_s_at	wingless-type MM	WNT4	4.23	3.92	4.25
216084_at	chromosome 9 op	C9orf144	3.74	3.48	3.28
200989_at	hypoxia inducible	HIF1A	13.65	13.66	13.82
220439_at	Ras and Rab intera	RIN3	6.09	6.06	5.96
211609_x_at	proteasome (pros	PSMD4	11.50	11.43	11.49
207649_at	keratin 37	KRT37	4.80	4.72	4.79
214379_at	heterogeneous nu	HNRPDL	5.80	5.95	5.93
217309_s_at	Down syndrome c	DSCR3	6.91	6.81	6.70
205997_at	ADAM metallopep	ADAM28	4.12	3.78	3.98
206597_at	neural retina leuci	NRL	5.54	5.04	5.40
211101_x_at	leukocyte immuno	LILRA2	6.21	6.16	6.13
216531_at	YY2 transcription	YY2	6.79	6.92	6.48

216008_s_at	ariadne homolog :	ARIH2	7.63	7.49	7.61
214956_at	AP2 associated kir	AAK1	4.14	4.12	3.75
215036_at	---	---	6.08	5.90	5.68
222282_at	---	---	7.47	7.79	7.51
219573_at	leucine rich repea	LRRC16A	6.09	5.69	5.82
215412_x_at	postmeiotic segre	PMS2L2	7.41	7.40	7.31
214467_at	G protein-couplec	GPR65	3.44	3.35	2.93
218115_at	ASF1 anti-silencin	ASF1B	8.20	8.47	8.14
211036_x_at	anaphase promot	ANAPC5	11.36	11.32	11.29
213521_at	protein tyrosine p	PTPN18	7.46	7.47	7.44
205847_at	protease, serine, :	PRSS22	7.36	7.11	7.15
216556_x_at	---	---	5.06	4.93	4.88
219389_at	sushi domain cont	SUSD4	4.12	4.48	4.08
208713_at	heterogeneous nu	HNRNPUL1	9.95	9.99	9.97
214046_at	fucosyltransferase	FUT9	3.43	3.52	3.33
221482_s_at	cAMP-regulated p	ARPP19	10.28	10.40	10.45
49878_at	peroxisomal bioge	PEX16	9.15	9.11	9.20
217213_at	solute carrier fam	SLC6A2	3.50	3.71	3.47
214838_at	SFT2 domain cont	SFT2D2	6.92	6.87	6.87
210647_x_at	phospholipase A2	PLA2G6	7.78	7.72	7.48
206955_at	aquaporin 7	AQP7	6.17	6.13	5.95
216743_at	---	---	4.12	3.93	3.84
203987_at	frizzled homolog 6	FZD6	9.58	9.78	9.86
221412_at	vomeronasal 1 rec	VN1R1	3.81	3.61	3.74
209058_at	endothelial differe	EDF1	11.25	11.13	11.40
205401_at	alkylglycerone ph	AGPS	8.39	8.46	8.38
205509_at	carboxypeptidase	CPB1	4.25	4.01	3.97
202288_at	mechanistic targe	MTOR	7.25	7.34	7.53
215026_x_at	sodium channel, r	SCNN1A	8.06	7.79	7.79
215773_x_at	poly (ADP-ribose)	PARP2	10.23	10.14	10.17
210398_x_at	fucosyltransferase	FUT6	4.65	4.47	4.31
211364_at	methylthioadenos	MTAP	5.38	5.68	5.28
206143_at	solute carrier fam	SLC26A3	3.36	3.32	3.38
204100_at	thyroid hormone	THRA	6.77	6.57	6.61
211123_at	solute carrier fam	SLC5A5	8.21	8.10	7.73
219084_at	nuclear receptor b	NSD1	6.44	6.42	6.46
212428_at	KIAA0368	KIAA0368	10.52	10.41	10.58
201807_at	vacuolar protein s	VPS26A	11.67	11.78	11.80
203554_x_at	pituitary tumor-tr	PTTG1	12.95	13.00	13.02
222100_at	Cytochrome P450	CYP2E1	4.66	4.41	4.47
208127_s_at	suppressor of cyto	SOCS5	8.70	8.89	8.95
205927_s_at	cathepsin E	CTSE	6.10	5.77	5.53
216551_x_at	phospholipase C, g	PLCG1	6.71	6.36	6.19
211614_at	---	---	4.05	4.06	4.07
40465_at	DEAD (Asp-Glu-Ala	DDX23	7.84	8.01	7.85
215824_at	nudix (nucleoside	NUDT7	4.43	4.58	3.90
219784_at	F-box protein 31	FBXO31	7.35	7.48	7.28

213400_s_at	transducin (beta)-	TBL1X	11.01	10.89	11.07
217355_at	---	---	4.89	4.64	4.65
220671_at	CCR4 carbon catal	CCRN4L	6.36	6.40	6.21
215571_at	---	---	5.95	6.60	6.21
220951_s_at	APOBEC1 complem	A1CF	6.32	6.66	5.85
214896_at	---	---	5.43	5.78	5.43
202878_s_at	CD93 molecule	CD93	4.17	4.16	4.11
218432_at	F-box protein 3	FBXO3	8.38	8.57	8.58
220877_at	---	---	4.36	4.44	4.25
206576_s_at	carcinoembryonic	CEACAM1	5.12	5.03	5.19
203817_at	guanylate cyclase	GUCY1B3	3.67	3.57	3.43
207129_at	carbonic anhydras	CA5B	6.25	6.14	5.90
219141_s_at	autophagy/beclin-	AMBRA1	8.01	7.94	8.01
210575_at	nuclear distributic	NUDC	4.49	4.53	4.64
204067_at	sulfite oxidase	SUOX	5.91	6.15	6.09
211824_x_at	NLR family, pyrin (	NLRP1	4.82	4.73	4.35
207439_s_at	solute carrier fam	SLC35A2	5.49	5.44	5.32
221725_at	WAS protein fami	WASF2	9.05	8.91	9.05
219380_x_at	polymerase (DNA	POLH	6.86	6.77	6.87
219339_s_at	euchromatic histo	EHMT1	5.79	5.79	5.72
221143_at	replication protei	RPA4	5.00	4.58	5.05
215475_at	---	---	3.51	3.63	3.61
217016_x_at	transmembrane p	TMEM212	4.67	4.60	4.63
217852_s_at	ADP-ribosylation f	ARL8B	11.20	11.19	11.32
219335_at	armadillo repeat c	ARMCX5	7.98	7.64	7.99
217336_at	ribosomal protein	RPS10 /// RPS10P5	7.35	7.34	7.61
212118_at	tripartite motif-co	TRIM27	9.31	9.42	9.38
217107_at	---	---	6.44	6.42	6.36
217196_s_at	calmodulin regula	CAMSAP1L1	8.65	8.59	8.80
216928_at	T-cell acute lymph	TAL1	6.30	5.80	6.01
201513_at	translin	TSN	8.40	8.42	8.42
201382_at	calcyclin binding p	CACYBP	4.64	4.55	4.62
202872_at	TNF receptor-asso	TRAF4	7.89	8.08	8.19
222247_at	---	---	3.67	3.84	3.72
208263_at	signal recognition	SRP72	3.90	3.66	3.51
208633_s_at	microtubule-actin	MACF1	7.78	8.09	8.31
209565_at	ring finger protein	RNF113A	8.87	8.72	8.93
215643_at	---	---	3.11	3.03	3.09
52078_at	transmembrane p	TMEM222	8.20	8.53	8.36
211559_s_at	cyclin G2	CCNG2	6.03	5.79	5.99
209660_at	transthyretin	TTR	7.28	7.32	6.57
40284_at	forkhead box A2	FOXA2	6.63	6.48	6.63
211109_at	Janus kinase 3	JAK3	5.23	5.04	5.13
217275_at	testis-specific seri	TSSK2	7.03	6.93	7.03
33768_at	dystrophia myoto	DMWD	7.73	7.93	7.83
201773_at	activity-depender	ADNP	11.39	11.30	11.41
203638_s_at	fibroblast growth	FGFR2	4.30	4.28	4.60

219298_at	enoyl CoA hydrata	ECHDC3	8.84	8.84	8.62
216734_s_at	chemokine (C-X-C	CXCR5	5.90	6.02	6.02
214106_s_at	GDP-mannose 4,6	GMDS	8.06	8.05	8.12
215619_at	---	---	3.56	3.59	3.47
202285_s_at	tumor-associated	TACSTD2	6.21	6.04	6.27
221106_at	solute carrier fam	SLC22A17	5.13	4.75	5.28
200774_at	family with seque	FAM120A	12.57	12.61	12.78
219829_at	integrin beta 1 bir	ITGB1BP2	5.90	6.03	5.80
214367_at	RAS guanyl releasi	RASGRP2	6.13	6.02	6.14
203010_at	signal transducer	STAT5A	6.44	6.34	6.36
211951_at	nucleolar and coil	NOLC1	12.29	12.23	12.29
207756_at	---	---	5.74	5.44	5.69
218997_at	polymerase (RNA)	POLR1E	9.17	9.14	9.17
215064_at	sterol-C5-desatura	SC5DL	4.17	4.15	4.23
216909_at	ribosomal RNA pr	RRP12	5.29	5.43	5.24
212425_at	secretory carrier r	SCAMP1	6.30	6.58	6.76
203643_at	Ets2 repressor fac	ERF	7.98	7.80	7.91
220491_at	hepcidin antimicr	HAMP	4.95	5.18	5.17
208484_at	histone cluster 1,	HIST1H1A	5.03	5.42	4.97
206706_at	neurotrophin 3	NTF3	6.52	6.44	6.15
210540_s_at	UDP-Gal:betaGlcN	B4GALT4	7.36	7.59	7.60
209879_at	selectin P ligand	SELPLG	6.21	6.24	6.05
209855_s_at	kallikrein-related	KLK2	6.62	6.35	6.42
216134_at	FERM domain con	FRMD4B	4.21	4.21	4.18
205307_s_at	kynurenine 3-mor	KMO	4.32	4.19	4.14
213271_s_at	dopey family men	DOPEY1	7.13	7.43	7.09
201451_x_at	Ras homolog enri	RHEB	5.25	5.14	5.32
212284_x_at	tumor protein, tra	TPT1	14.91	14.92	14.92
200683_s_at	ubiquitin-conjuga	UBE2L3	9.46	9.60	9.44
207326_at	betacellulin	BTC	3.61	3.69	3.29
220222_at	chromosome 8 op	C8orf39	4.73	4.57	4.78
217165_x_at	metallothionein 1	MT1F	13.24	13.32	13.26
220204_s_at	bone morphogene	BMP8A	5.48	4.87	4.76
201926_s_at	CD55 molecule, d	CD55	9.91	9.86	10.09
218075_at	achalasia, adreno	AAAS	6.91	6.81	6.86
216742_at	---	---	4.17	4.17	3.82
57163_at	elongation of very	ELOVL1	10.08	10.04	10.08
214432_at	ATPase, Na+/K+ tr	ATP1A3	4.30	4.28	4.39
201983_s_at	epidermal growth	EGFR	10.08	9.98	10.21
215769_at	T cell receptor del	TRD@	4.54	4.51	4.46
217104_at	suppressor of tum	ST20	3.64	3.60	3.52
207616_s_at	TRAF family meml	TANK	9.28	9.20	9.36
215205_x_at	nuclear receptor c	NCOR2	6.48	6.65	6.65
220238_s_at	kelch-like 7 (Dros	KLHL7	6.25	6.41	6.62
52159_at	HemK methyltran	HEMK1	8.62	8.62	8.71
221793_at	dedicator of cytok	DOCK6	5.30	5.26	5.53
202448_s_at	zer-1 homolog (C.	ZER1	4.34	4.58	4.38

205258_at	inhibin, beta B	INHBB	5.92	6.17	5.48
205387_s_at	chorionic gonadot	CGB /// CGB5 /// (	7.27	6.71	6.58
212875_s_at	C2 calcium-depen	C2CD2	8.02	8.21	8.10
207267_s_at	Down syndrome c	DSCR6	3.58	3.77	3.42
205954_at	retinoid X recepto	RXRG	4.95	5.21	4.78
207583_at	ATP-binding casse	ABCD2	3.08	3.09	3.14
221252_s_at	germ cell associat	GSG1	5.45	5.31	5.12
206827_s_at	transient receptor	TRPV6	6.60	6.48	6.31
212463_at	CD59 molecule, co	CD59	9.16	9.39	9.30
215452_x_at	SMT3 suppressor	SUMO4	9.49	9.67	9.72
215823_x_at	poly(A) binding pr	PABPC1 /// RLIM	13.32	13.26	13.39
220609_at	hypothetical prote	LOC202181	5.73	5.56	5.43
222357_at	zinc finger and BT	ZBTB20	6.34	6.39	6.11
201328_at	v-ets erythroblast	ETS2	10.79	10.71	10.76
220756_s_at	G protein-couplec	GPR172B	4.62	4.70	4.36
221807_s_at	TraB domain cont	TRABD	6.61	6.59	6.33
221873_at	zinc finger proteir	ZNF143	8.10	8.43	8.27
202283_at	serpin peptidase i	SERPINF1	4.37	4.08	4.35
215698_at	lysine (K)-specific	KDM5A	6.42	6.59	6.44
211933_s_at	heterogeneous nu	HNRNPA3 /// HNR	11.99	11.98	12.04
213224_s_at	non-protein codin	NCRNA00081	8.57	8.74	8.74
211057_at	receptor tyrosine	ROR1	7.21	7.24	7.11
215361_at	dedicator of cytok	DOCK5	3.69	3.85	3.42
200867_at	ring finger proteir	RNF114	9.40	9.29	9.42
202989_at	regulator of G-prc	RGS1	3.21	3.00	3.26
204498_s_at	adenylate cyclase	ADCY9	5.45	5.45	5.49
219893_at	coiled-coil domair	CCDC71	8.07	7.47	7.60
212125_at	Ran GTPase activa	RANGAP1	7.86	7.93	8.01
203284_s_at	heparan sulfate 2-	HS2ST1	8.36	8.33	8.57
206795_at	coagulation factor	F2RL2	3.64	3.71	3.61
205006_s_at	N-myristoyltransfe	NMT2	9.64	9.77	9.93
201306_s_at	acidic (leucine-ricl	ANP32B	13.37	13.33	13.33
211660_at	POU class 2 home	POU2F2	8.04	7.93	7.76
202489_s_at	FXYD domain cont	FXYD3	6.11	6.19	5.96
207810_at	coagulation factor	F13B	3.66	3.44	3.39
204231_s_at	fatty acid amide h	FAAH	6.80	6.34	6.52
203993_x_at	Chromosome 21 c	C21orf2	7.10	6.89	7.00
220892_s_at	phosphoserine am	PSAT1	9.80	9.43	9.85
220086_at	IKAROS family zin	IKZF5	7.57	7.70	7.47
203933_at	RAB11 family inte	RAB11FIP3	7.24	7.32	7.36
200873_s_at	chaperonin contai	CCT8	13.27	13.30	13.38
206813_at	cardiotrophin 1	CTF1	6.46	6.43	6.15
220044_x_at	LUC7-like 3 (S. cer	LUC7L3	10.80	10.98	10.93
207136_at	arrestin 3, retinal	ARR3	6.50	6.50	6.24
203539_s_at	---	---	5.26	5.46	4.96
206949_s_at	RUN and SH3 dom	RUSC1	8.44	8.32	8.33
208264_s_at	eukaryotic transla	EIF3J	9.23	9.18	9.36

206333_at	musashi homolog	MSI1	4.64	4.19	4.62
214278_s_at	NDRG family mem	NDRG2	4.99	5.06	4.98
206037_at	cysteine conjugat	CCBL1	7.77	7.83	7.58
210296_s_at	peroxisomal bioge	PEX2	9.69	9.86	9.81
219768_at	V-set domain cont	VTCN1	3.82	3.94	3.56
214633_at	SRY (sex determin	SOX3	5.05	4.89	4.83
212954_at	dual-specificity ty	DYRK4	8.88	8.87	8.83
219864_s_at	RCAN family mem	RCAN3	4.07	3.81	3.45
204795_at	proline rich 3	PRR3	8.69	8.67	8.69
216347_s_at	protein phosphata	PPP1R13B	6.89	6.71	6.78
214187_x_at	CTD (carboxy-term	CTDSPL	5.74	5.61	5.24
222268_x_at	mucin 5B, oligome	MUC5B	4.08	4.18	3.72
204375_at	calsyntenin 3	CLSTN3	7.51	7.17	7.11
218723_s_at	chromosome 13 c	C13orf15	3.73	3.96	3.66
217989_at	hydroxysteroid (1	HSD17B11	8.95	9.04	9.30
211214_s_at	death-associated	DAPK1	3.81	4.06	3.84
219813_at	LATS, large tumor	LATS1	3.95	3.73	3.59
211931_s_at	heterogeneous nu	HNRNPA3 /// HNR	12.87	12.85	12.95
222143_s_at	myotubularin rela	MTMR14	8.78	8.76	8.68
207831_x_at	deoxyhypusine sy	DHPS	8.82	8.75	8.78
203486_s_at	armadillo repeat c	ARMC8	8.36	8.67	8.58
201993_x_at	heterogeneous nu	HNRPDL	12.05	12.00	12.09
214444_s_at	poliovirus recepto	PVR	7.11	6.95	7.00
205373_at	catenin (cadherin-	CTNNA2	3.80	3.65	3.86
203019_x_at	synovial sarcoma,	SSX2IP	7.66	7.88	7.93
217191_x_at	---	---	5.54	5.99	5.98
214109_at	LPS-responsive ve	LRBA	8.00	8.08	8.08
217696_at	fucosyltransferase	FUT7	7.33	6.87	7.02
221416_at	phospholipase A2	PLA2G2F	3.73	3.53	3.49
205755_at	inter-alpha (globu	ITIH3	5.00	4.56	4.64
211590_x_at	thromboxane A2 r	TBXA2R	6.72	6.86	6.50
210043_at	FERM domain con	FRMD8	7.65	7.60	7.67
217568_at	epididymal protei	EDDM3A	3.41	3.39	3.58
215642_at	---	---	3.86	3.74	3.56
218588_s_at	family with seque	FAM114A2	8.26	8.23	8.34
207427_at	acrosin /// similar	ACR /// LOC64552	5.38	5.03	5.29
206849_at	gamma-aminobut	GABRG2	5.63	5.92	5.18
201275_at	farnesyl diphosph	FDPS	11.58	11.66	11.68
202451_at	general transcript	GTF2H1	10.37	10.40	10.54
210136_at	myelin basic prote	MBP	4.33	4.30	4.22
204442_x_at	latent transformin	LTBP4	6.99	6.92	6.75
220641_at	NADPH oxidase, E	NOX5	6.00	5.82	5.89
216381_x_at	aldo-keto reducta	AKR7A3	7.63	7.60	7.57
62212_at	chromosome 1 op	C1orf50	8.08	8.13	8.07
217495_x_at	calcitonin-related	CALCA	6.14	6.00	6.20
211772_x_at	cholinergic recept	CHRNA3	5.69	5.90	5.50
213686_at	---	---	7.44	7.73	7.55

210674_s_at	protocadherin alp	PCDHA1 /// PCDH/	3.45	3.53	3.61
219010_at	chromosome 1 op	C1orf106	4.60	4.89	4.89
203858_s_at	COX10 homolog, c	COX10	7.72	7.84	7.49
206913_at	bile acid CoA: ami	BAAT	6.27	6.21	6.33
211575_s_at	ubiquitin protein l	UBE3A	8.46	8.57	8.62
213054_at	HAUS augmin-like	HAUS5	6.66	7.03	7.06
206740_x_at	synaptonemal cor	SYCP1	3.94	3.43	3.52
206842_at	potassium voltage	KCND1	5.67	5.64	5.68
213149_at	dihydrolipoamide	DLAT	7.55	8.07	8.22
201664_at	structural mainter	SMC4	12.32	12.58	12.55
210320_s_at	DEAD (Asp-Glu-Ala	DDX52	7.53	7.79	7.95
207286_at	centrosomal prote	CEP135	3.43	3.78	3.76
89977_at	acyl-CoA syntheta	ACSM5	4.73	4.77	4.69
214770_at	macrophage scave	MSR1	3.05	3.09	3.33
216859_x_at	---	---	5.77	5.99	6.22
205642_at	centrosomal prote	CEP110	7.13	7.36	7.26
217582_at	---	---	5.01	4.88	4.65
214265_at	integrin, alpha 8	ITGA8	3.70	3.73	3.78
205861_at	Spi-B transcriptior	SPIB	5.48	5.47	5.11
218247_s_at	mex-3 homolog C	MEX3C	9.60	9.45	9.94
210175_at	chromosome 2 op	C2orf3	8.21	8.08	8.33
219874_at	solute carrier fam	SLC12A8	5.18	5.08	5.17
221336_at	atonal homolog 1	ATOH1	5.60	5.61	5.46
213551_x_at	polycomb group r	PCGF2	8.89	8.79	8.79
215138_s_at	kazrin	KAZ	6.79	6.92	6.49
213700_s_at	---	---	7.15	7.34	7.29
201468_s_at	NAD(P)H dehydro	NQO1	11.42	11.48	11.42
206824_at	carboxylesterase 4	CES4	5.97	5.87	5.78
204050_s_at	clathrin, light chai	CLTA	12.02	12.02	12.02
203071_at	sema domain, imr	SEMA3B	7.04	6.90	6.75
216448_at	---	---	6.36	6.10	6.11
221395_at	taste receptor, typ	TAS2R13	3.76	4.08	3.69
216764_at	---	---	4.07	3.84	3.81
1438_at	EPH receptor B3	EPHB3	5.52	5.83	5.71
36554_at	acetylserotonin O	ASMTL	8.05	8.23	8.18
217675_at	zinc finger and BT	ZBTB7C	4.47	4.46	4.12
214146_s_at	pro-platelet basic	PPBP	3.38	3.49	3.39
205993_s_at	T-box 2	TBX2	6.74	6.65	6.18
216245_at	interleukin 1 rece	IL1RN	4.24	4.39	4.07
320_at	scaffold attachme	SAFB2	6.06	6.20	5.72
213096_at	transmembrane a	TMCC2	6.49	6.40	6.32
219112_at	Rap guanine nucle	RAPGEF6	8.45	8.59	8.73
211342_x_at	mediator complex	MED12	6.92	6.94	6.94
215883_at	Catenin (cadherin	CTNNA1	5.53	5.72	5.20
206015_s_at	forkhead box J3	FOXJ3	10.29	10.03	10.28
210161_at	---	---	7.28	7.16	7.15
220932_at	---	---	5.06	5.41	4.93

210979_at	N-acylsphingosine	ASAH1	3.63	3.50	3.70
204206_at	MAX binding prot	MNT	8.92	8.73	8.97
205646_s_at	paired box 6	PAX6	5.12	5.16	5.02
219803_at	angiopoietin-like 3	ANGPTL3	3.69	3.40	3.54
214651_s_at	homeobox A9	HOXA9	7.01	7.02	7.16
208735_s_at	CTD (carboxy-term	CTDSP2	8.58	8.63	8.66
212290_at	solute carrier fam	SLC7A1	8.86	8.52	8.68
201220_x_at	C-terminal binding	CTBP2	12.24	12.09	12.25
204323_x_at	neurofibromin 1	NF1	4.96	5.43	5.28
210429_at	Rh blood group, D	RHD	3.65	3.89	3.42
49452_at	acetyl-CoA carbox	ACACB	5.57	5.23	5.61
206023_at	neuromedin U	NMU	8.84	8.89	8.89
202271_at	F-box protein 28	FBXO28	9.12	9.34	9.45
206147_x_at	sex comb on midl	SCML2	10.00	9.88	9.91
207858_s_at	pyruvate kinase, l	PKLR	4.49	4.46	4.59
208507_at	olfactory receptor	OR7C2	6.34	6.39	6.12
217488_x_at	postmeiotic segre	PMS2L3	4.75	4.85	4.54
219303_at	ring finger protein	RNF219	9.09	9.11	9.18
207664_at	ADAM metallopep	ADAM2	3.30	3.23	3.26
216823_at	ribosomal protein	RPS3A	5.31	5.30	5.30
206841_at	phosphodiesteras	PDE6H	3.28	3.23	3.07
217200_x_at	cytochrome b-561	CYB561	9.53	9.47	9.30
219731_at	weakly similar to	FLJ34077	7.98	8.02	8.17
202594_at	leptin receptor ov	LEPROTL1	10.27	10.12	10.37
207115_x_at	mbt domain conta	MBTD1	5.68	5.83	5.72
214953_s_at	amyloid beta (A4)	APP	10.99	10.87	11.02
201371_s_at	cullin 3	CUL3	11.14	11.23	11.30
212757_s_at	calcium/calmodul	CAMK2G	8.24	8.31	8.21
218979_at	RMI1, RecQ media	RMI1	9.92	9.90	10.06
207551_s_at	male-specific leth	MSL3	9.12	9.15	9.11
205595_at	desmoglein 3 (per	DSG3	6.07	6.24	5.63
204755_x_at	hepatic leukemia	HLF	4.48	4.28	4.70
213028_at	nuclear factor rela	NFRKB	7.65	7.78	7.71
222213_x_at	---	---	4.51	4.49	4.13
222111_at	---	---	7.01	7.70	7.56
212299_at	NIMA (never in m	NEK9	9.67	9.76	9.68
204093_at	cyclin H	CCNH	10.16	10.16	10.24
215449_at	translocator prote	TSPO2	5.64	5.74	5.54
220983_s_at	sprouty homolog	SPRY4	4.64	4.69	4.46
211745_x_at	hemoglobin, alpha	HBA1 /// HBA2	6.71	6.44	6.51
213598_at	---	---	8.56	8.85	8.85
215942_s_at	G-2 and S-phase e	GTSE1	7.81	7.97	8.01
221102_s_at	transient receptor	TRPM6	5.12	5.01	4.88
213936_x_at	surfactant protein	SFTPB	7.26	7.57	7.47
204707_s_at	mitogen-activated	MAPK4	6.75	6.27	6.33
215973_at	HLA complex grou	HCG4P6	4.64	4.29	4.08
214083_at	protein phosphata	PPP2R5C	6.72	6.45	6.70

208458_at	sodium channel, r	SCNN1D	5.00	4.58	4.66
200740_s_at	SMT3 suppressor	SUMO3	11.47	11.47	11.50
210643_at	tumor necrosis fa	TNFSF11	3.77	3.54	3.36
204377_s_at	Vpr (HIV-1) bindin	VPRBP	5.85	6.06	5.99
218760_at	coenzyme Q6 hon	COQ6	8.28	8.32	8.32
217360_x_at	immunoglobulin h	IGHA1 /// IGHG1 /	5.08	4.60	4.66
217763_s_at	RAB31, member R	RAB31	10.47	10.47	10.58
202835_at	angiotensinogen (	AGT	6.85	6.70	7.09
216632_at	Neuron navigator	NAV3	3.71	3.51	3.43
222137_at	coiled-coil and C2	CC2D1A	4.70	4.96	4.26
209839_at	dynamin 3	DNM3	5.67	5.29	5.44
209491_s_at	adenosine monop	AMPD3	5.85	6.16	5.93
214140_at	solute carrier fam	SLC25A16	6.33	6.47	6.60
200626_s_at	matrin 3	MATR3	12.30	12.45	12.42
218136_s_at	solute carrier fam	SLC25A37	7.78	7.82	7.96
213691_at	---	---	7.56	7.43	7.35
206940_s_at	POU class 4 home	POU4F1	3.82	3.62	3.37
217338_at	keratin 19 pseudo	KRT19P2	5.92	5.94	5.92
211362_s_at	serpin peptidase i	SERPINB13	4.55	4.74	4.45
209576_at	guanine nucleotid	GNAI1	8.02	8.16	8.25
218362_s_at	DIS3 mitotic contr	DIS3	6.19	6.52	6.26
212727_at	discs, large homol	DLG3	6.66	6.58	6.63
201145_at	HCLS1 associated	HAX1	11.37	11.19	11.35
207672_at	---	---	3.50	3.32	3.34
205828_at	matrix metallopep	MMP3	3.50	3.75	3.86
213364_s_at	sorting nexin 1	SNX1	7.83	8.01	8.25
220075_s_at	cadherin-related f	CDHR5	3.44	3.43	3.32
201361_at	transmembrane p	TMEM109	10.42	10.48	10.43
207575_at	golgin A6 family, r	GOLGA6A	3.81	3.60	3.59
213905_x_at	biglycan	BGN	5.03	5.01	4.91
214213_x_at	Lamin A/C	LMNA	8.61	8.41	8.47
206263_at	flavin containing r	FMO4	5.32	5.19	4.96
202158_s_at	CUGBP, Elav-like f	CELF2	3.72	3.59	3.57
206944_at	5-hydroxytryptam	HTR6	4.95	4.95	5.15
216709_at	---	---	3.32	3.46	3.12
211475_s_at	BCL2-associated a	BAG1	9.17	9.14	9.20
219824_at	solute carrier fam	SLC13A4	6.25	6.06	6.07
206469_x_at	aldo-keto reducta	AKR7A3	7.49	7.47	7.49
221439_at	retinoblastoma bi	RBBP9	4.42	4.50	4.07
207316_at	hyaluronan synth	HAS1	6.28	6.04	5.75
204712_at	WNT inhibitory fa	WIF1	3.81	3.51	3.61
216623_x_at	TOX high mobility	TOX3	3.06	3.16	3.21
219159_s_at	SLAM family mem	SLAMF7	4.58	4.49	4.35
203886_s_at	fibulin 2	FBLN2	5.95	5.89	5.80
216583_x_at	---	---	8.07	7.88	7.94
205912_at	pancreatic lipase	PNLIP	4.21	4.07	4.18
205668_at	lymphocyte antig	LY75	3.08	3.06	2.97

206443_at	RAR-related orpha	RORB	3.91	3.60	3.60
217139_at	voltage-depender	VDAC1 /// VDAC1F	5.90	5.94	5.73
43934_at	G protein-couplec	GPR137	6.98	7.11	6.96
202589_at	thymidylate synth	TYMS	12.38	12.44	12.42
207217_s_at	NADPH oxidase 1	NOX1	4.30	4.32	4.25
220860_at	purine-rich eleme	PURG	3.78	3.41	3.32
209328_x_at	HIG1 hypoxia indu	HIGD2A	7.89	7.66	7.61
215654_at	branched chain ar	BCAT2	6.95	6.74	6.70
204058_at	malic enzyme 1, N	ME1	10.42	10.40	10.60
210221_at	cholinergic recept	CHRNA3	4.64	4.46	4.07
206231_at	potassium interm	KCNN1	6.68	6.55	6.24
213657_s_at	---	---	6.61	6.41	6.33
200940_s_at	arginine-glutamic	RERE	9.80	9.72	9.86
215699_x_at	Sfi1 homolog, spir	SFI1	7.49	7.68	7.54
215920_s_at	hypothetical LOC1	LOC100288442 ///	6.42	6.25	6.28
206025_s_at	tumor necrosis fa	TNFAIP6	8.76	8.44	8.80
211691_x_at	---	---	7.65	7.37	7.47
211318_s_at	RAE1 RNA export	RAE1	9.83	9.83	9.84
215420_at	Indian hedgehog I	IHH	5.74	5.93	5.36
222194_at	family with seque	FAM66D	4.47	4.61	4.30
219580_s_at	transmembrane c	TMC5	3.81	3.97	3.80
209243_s_at	paternally express	PEG3 /// ZIM2	3.71	3.62	3.53
219329_s_at	chromosome 2 op	C2orf28	11.93	11.79	11.87
217623_at	myosin light chain	MYLK3	4.25	3.88	3.93
220832_at	toll-like receptor 8	TLR8	4.07	3.91	4.04
212283_at	agrin	AGRN	7.26	7.21	7.08
202269_x_at	guanylate binding	GBP1	7.79	8.02	7.90
210246_s_at	ATP-binding casse	ABCC8	6.32	6.13	5.66
218056_at	bifunctional apop	BFAR	9.39	9.38	9.57
213362_at	protein tyrosine p	PTPRD	5.04	5.11	5.01
219598_s_at	RWD domain cont	RWDD1	11.14	11.24	11.29
209231_s_at	dynactin 5 (p25)	DCTN5	7.88	8.08	8.11
222371_at	---	---	6.46	7.04	6.71
211479_s_at	5-hydroxytryptam	HTR2C	4.39	4.25	4.16
201166_s_at	pumilio homolog	PUM1	11.55	11.46	11.61
204188_s_at	retinoic acid recep	RARG	5.56	5.48	5.70
AFFX-CreX-3_at	---	---	14.14	14.15	14.13
214301_s_at	Dihydropyrimidin	DPYSL4	7.16	7.07	6.85
218078_s_at	zinc finger, DHHC-	ZDHHC3	9.01	9.15	9.11
220487_at	syntrophin, gamm	SNTG2	4.88	4.75	4.34
215912_at	guanine nucleotid	GNAO1	4.30	4.19	4.18
201789_at	dehydrogenase/re	DHRS7	3.44	3.27	3.57
213961_s_at	---	---	5.89	5.30	5.37
201547_at	lysine (K)-specific	KDM5B	3.57	3.51	3.71
217783_s_at	yippee-like 5 (Dro	YPEL5	9.38	9.29	9.46
205934_at	phospholipase C-l	PLCL1	3.95	3.87	3.89
203378_at	PCF11, cleavage a	PCF11	7.93	8.16	8.11

210325_at	CD1a molecule	CD1A	4.26	4.18	4.22
214992_s_at	deoxyribonucleas	DNASE2	7.59	7.34	7.42
218573_at	melanoma antigei	MAGEH1	8.26	8.38	8.37
204844_at	glutamyl aminope	ENPEP	3.44	3.28	3.38
219080_s_at	CTP synthase II	CTPS2	7.68	7.75	7.82
219153_s_at	thrombospondin,	THSD4	4.41	4.29	4.16
209787_s_at	high mobility grou	HMGN4	10.88	10.91	11.05
206199_at	carcinoembryonic	CEACAM7	3.93	4.24	4.17
207478_at	---	---	3.51	3.94	3.69
214737_x_at	heterogeneous nu	HNRNPC	12.33	12.36	12.43
214899_at	zinc finger proteir	ZNF780B	3.13	3.18	3.17
216309_x_at	jerky homolog (m	JRK	7.98	8.13	8.00
203604_at	zinc finger proteir	ZNF516	7.66	7.60	7.74
218795_at	acid phosphatase	ACP6	7.87	7.79	7.99
201113_at	Tu translation elo	TUFM	11.65	11.66	11.72
222302_at	---	---	6.91	6.99	6.92
205744_at	double C2-like do	DOC2A	5.78	6.08	5.65
220094_s_at	coiled-coil domair	CCDC90A	10.43	10.43	10.42
205697_at	secretagogin, EF-h	SCGN	5.20	5.17	5.08
210061_at	zinc finger proteir	ZNF589	5.58	5.52	5.36
205588_s_at	FGFR1 oncogene p	FGFR1OP	8.02	8.07	8.28
202558_s_at	heat shock protei	HSPA13	8.31	8.27	8.59
219026_s_at	RAS protein activa	RASAL2	6.43	6.33	6.23
44146_at	glucocorticoid mo	GMEB2	9.39	9.31	9.41
217726_at	coatomer protein	COPZ1	10.04	10.04	10.08
216809_at	cylicin, basic prote	CYLC1	3.67	3.45	3.62
221972_s_at	stromal cell derive	SDF4	8.88	8.76	8.91
222334_at	---	---	5.54	5.77	5.39
221350_at	homeobox C8	HOXC8	7.30	7.51	7.55
40148_at	amyloid beta (A4)	APBB2	7.79	7.75	7.76
212756_s_at	ubiquitin protein l	UBR2	8.19	8.28	8.32
212271_at	mitogen-activatec	MAPK1	9.67	9.78	9.87
203173_s_at	chromosome 16 c	C16orf62	8.07	8.06	8.17
202331_at	branched chain ke	BCKDHA	8.71	8.58	8.43
212087_s_at	Era G-protein-like	ERAL1	7.88	7.82	7.68
211987_at	topoisomerase (D	TOP2B	10.61	10.73	10.60
203243_s_at	PDZ and LIM dom	PDLIM5	9.46	9.55	9.79
205229_s_at	coagulation factor	COCH	3.78	3.65	3.93
208629_s_at	hydroxyacyl-CoA c	HADHA	10.78	10.72	10.77
215229_at	hypothetical prote	LOC100129973	3.68	3.56	3.60
215260_s_at	transcription facto	TCF3	6.66	6.71	6.52
214886_s_at	NEDD4 binding pr	N4BP2L1	4.56	3.96	4.34
207175_at	adiponectin, C1Q	ADIPOQ	3.83	3.75	3.88
212107_s_at	DEAH (Asp-Glu-Al	DHX9	7.33	7.63	7.44
205803_s_at	transient receptor	TRPC1	8.64	8.76	8.66
205323_s_at	metal-regulatory t	MTF1	8.32	8.21	8.12
204152_s_at	MFNG O-fucosylp	MFNG	6.03	5.68	6.24

211848_s_at	carcinoembryonic	CEACAM7	3.97	4.06	3.85
215510_at	ets variant 2	ETV2	5.70	5.34	5.55
215605_at	Nuclear receptor (	NCOA2	5.99	6.43	6.15
209823_x_at	major histocompa	HLA-DQB1	6.55	6.65	6.53
208221_s_at	slit homolog 1 (Dr	SLIT1	5.02	5.18	5.33
217829_s_at	ubiquitin specific		USP39	10.49	10.39	10.54
207542_s_at	aquaporin 1 (Colt	AQP1	4.63	4.44	4.62
222166_at	chromosome 9 op	C9orf16	4.06	4.12	4.19
209583_s_at	CD200 molecule	CD200	3.52	3.53	4.22
209529_at	phosphatidic acid	PPAP2C	7.70	7.67	7.66
216148_at	---	---	4.03	3.86	3.91
222053_at	TAF6-like RNA pol	TAF6L	3.62	3.41	3.49
207915_at	similar to L-myc-2	LOC100293553	4.28	4.15	4.08
204573_at	carnitine O-octan	CROT	7.55	7.91	7.75
220142_at	hyaluronan and p	HAPLN2	5.67	5.44	5.06
218148_at	centromere prote	CENPT	6.30	6.32	6.14
217657_at	B-cell receptor-as	BCAP29	5.50	5.24	5.24
202220_at	KIAA0907	KIAA0907	10.22	10.38	10.28
210300_at	RAS (RAD and GEI	REM1	7.19	7.26	7.06
217231_s_at	microtubule asso	MAST1	5.78	5.49	5.87
215575_at	phosphodiesteras	PDE4DIP	3.89	3.58	3.81
209864_at	frequently rearra	FRAT2	8.25	8.17	8.29
202075_s_at	phospholipid tran	PLTP	4.66	4.77	4.66
216765_at	Mitogen-activate	MAP2K5	4.16	4.05	4.10
212827_at	immunoglobulin h	IGHM	4.18	4.34	4.00
201672_s_at	ubiquitin specific		USP14	12.05	12.15	12.03
214568_at	tryptase delta 1	TPSD1	5.31	4.92	5.08
215211_at	RNA polymerase I	RRN3P1	4.06	4.10	3.90
213108_at	calcium/calmodul	CAMK2A	6.14	5.86	5.94
222340_at	---	---	3.55	3.81	3.77
216971_s_at	plectin	PLEC	5.07	5.42	5.16
220300_at	regulator of G-pro	RGS3	4.88	4.67	4.52
216719_s_at	---	---	3.37	3.41	3.54
222335_at	---	---	6.80	7.21	7.02
206205_at	M-phase phospho	MPHOSPH9	8.86	8.90	9.02
205812_s_at	transmembrane e	TMED9	11.80	11.79	11.74
203265_s_at	mitogen-activate	MAP2K4	6.32	6.53	6.42
221967_at	neurexophilin 4	NXPH4	7.27	7.07	7.08
203097_s_at	Rap guanine nucl	RAPGEF2	10.94	10.76	11.00
212343_at	Yip1 domain famil	YIPF6	7.24	7.56	7.56
203178_at	glycine amidinotr	GATM	3.50	3.33	3.17
215096_s_at	esterase D	ESD	11.78	11.76	11.92
218219_s_at	LanC lantibiotic sy	LANCL2	8.57	8.57	8.55
201320_at	SWI/SNF related,	SMARCC2	8.23	8.02	7.74
214050_at	---	---	5.34	5.30	5.42
218869_at	malonyl-CoA deca	MLYCD	7.91	8.05	7.80
208655_at	---	---	12.08	11.99	12.14

206840_at	afamin	AFM	3.48	3.29	3.05
208597_at	ciliary neurotroph	CNTF /// ZFP91 ///	4.12	4.04	4.22
65630_at	transmembrane p	TMEM80	7.27	7.19	7.31
215086_at	inhibitor of Bruto	IBTK	3.60	3.57	3.43
209062_x_at	nuclear receptor c	NCOA3	7.21	7.32	7.50
207126_x_at	UDP glucuronosyl	UGT1A1 /// UGT1A	5.70	6.27	6.18
207647_at	chromodomain pr	CDY1	3.44	3.47	3.40
206277_at	purinergic recepto	P2RY2	4.93	4.91	4.68
207104_x_at	leukocyte immun	LILRB1	5.88	5.83	6.04
207976_at	kelch-like 18 (Dro	KLHL18	5.09	4.86	4.64
204624_at	ATPase, Cu++ trar	ATP7B	6.39	6.61	6.16
208649_s_at	valosin-containing	VCP	10.01	10.09	9.97
217037_at	---	---	3.68	3.49	3.38
215557_at	---	---	5.07	5.22	5.21
207294_at	angiotensin II rece	AGTR2	3.75	3.34	3.79
201820_at	keratin 5	KRT5	6.85	6.84	6.51
207775_at	hypothetical LOC7	MGC4859	3.51	3.25	3.13
201112_s_at	CSE1 chromosome	CSE1L	12.57	12.69	12.60
212971_at	cysteinyl-tRNA syr	CARS	12.63	12.46	12.63
205570_at	phosphatidylinosi	PIP4K2A	6.82	6.34	6.68
208612_at	protein disulfide is	PDIA3	11.53	11.46	11.65
217039_x_at	Immunoglobulin h	IGHG1	7.75	7.67	7.57
215607_x_at	---	---	7.02	7.21	6.97
218212_s_at	molybdenum cofa	MOCS2	8.92	9.06	9.12
215370_at	---	---	4.98	5.16	5.24
220366_at	epididymal sperm	ELSPBP1	6.84	6.84	6.37
214422_at	RAD23 homolog B	RAD23B	5.74	5.77	5.11
218073_s_at	transmembrane p	TMEM48	8.04	8.08	8.18
203735_x_at	PTPRF interacting	PPFIBP1	8.64	8.68	8.89
208417_at	fibroblast growth	FGF6	7.87	7.55	7.38
214599_at	involucrin	IVL	4.01	4.32	4.37
202652_at	amyloid beta (A4)	APBB1	6.10	6.33	5.95
215024_at	chromosome 7 op	C7orf28B	6.53	6.63	6.98
209482_at	processing of prec	POP7	9.61	9.81	9.60
205867_at	protein tyrosine p	PTPN11	4.15	3.84	3.89
213036_x_at	ATPase, Ca++ tran	ATP2A3	5.70	6.05	5.28
217814_at	coiled-coil domair	CCDC47	9.79	9.87	9.92
205001_s_at	DEAD (Asp-Glu-Ala	DDX3Y	4.62	4.36	4.50
216554_s_at	---	---	8.53	8.62	8.45
203566_s_at	amylo-alpha-1, 6-g	AGL	9.08	9.31	9.48
207645_s_at	chromodomain he	CHD1L	5.49	5.43	5.49
211049_at	T-cell leukemia ho	TLX2	5.90	5.97	5.86
206146_s_at	Rh-associated glyc	RHAG	6.34	6.17	6.52
220914_at	---	---	6.24	6.06	6.18
221220_s_at	SCY1-like 2 (S. cer	SCYL2	5.01	5.10	4.93
203156_at	A kinase (PRKA) ar	AKAP11	10.29	10.27	10.33
220768_s_at	casein kinase 1, ga	CSNK1G3	9.25	9.33	9.39

205831_at	CD2 molecule	CD2	4.20	4.73	4.34
206410_at	nuclear receptor s	NR0B2	6.78	6.80	6.72
215343_at	coiled-coil domair	CCDC88C	5.87	5.44	5.53
221929_at	RNA binding moti	RBM12B	7.11	7.27	6.90
218437_s_at	leucine zipper tra	LZTFL1	8.26	8.43	8.57
219689_at	sema domain, imr	SEMA3G	4.27	4.32	3.90
209858_x_at	metallophosphoe:	MPPE1	8.56	8.67	8.60
213881_x_at	SMT3 suppressor	SUMO2	12.98	13.09	13.13
220592_at	coiled-coil domair	CCDC40	4.95	4.60	4.78
219034_at	poly (ADP-ribose)	PARP16	7.45	7.29	7.48
209067_s_at	heterogeneous nu	HNRPDL	11.71	11.60	11.74
207119_at	protein kinase, cG	PRKG1	3.84	3.76	3.67
222290_at	olfactory receptor	OR2A20P /// OR2A	5.37	5.62	5.26
211375_s_at	interleukin enhan	ILF3	11.02	11.09	11.29
209458_x_at	hemoglobin, alph	HBA1 /// HBA2	6.69	6.66	6.63
203997_at	protein tyrosine p	PTPN3	6.28	6.53	6.36
204799_at	zinc finger, BED-ty	ZBED4	8.04	8.09	8.27
200784_s_at	low density lipopr	LRP1	7.45	7.27	7.07
215602_at	FYVE, RhoGEF anc	FGD2	4.51	4.46	4.60
215898_at	tubulin tyrosine li	TTLL5	4.43	5.06	4.58
205204_at	neuromedin B	NMB	7.16	6.90	6.53
210044_s_at	lymphoblastic leu	LYL1	7.14	7.16	6.91
214235_at	cytochrome P450,	CYP3A5	4.33	4.30	4.30
219535_at	hormonally up-reg	HUNK	5.59	5.59	5.63
203171_s_at	ribosomal RNA pr	RRP8	8.98	8.85	8.93
221459_at	trace amine assoc	TAAR5	5.66	5.17	4.95
203564_at	Fanconi anemia, c	FANCG	8.33	8.26	8.32
201100_s_at	ubiquitin specific	USP9X	11.34	11.41	11.40
209746_s_at	coenzyme Q7 hon	COQ7	8.46	8.36	8.27
210785_s_at	chromosome 1 op	C1orf38	5.38	5.36	5.01
203326_x_at	---	---	7.55	7.37	7.49
211138_s_at	kynurenine 3-mor	KMO	3.33	3.88	3.77
209773_s_at	ribonucleotide re	RRM2	12.40	12.45	12.39
206741_at	chromosome 3 op	C3orf32	5.92	6.06	5.99
204030_s_at	schwannomin inte	SCHIP1	9.60	9.58	9.79
211225_at	fucosyltransferase	FUT5	6.80	6.79	6.63
205447_s_at	mitogen-activatec	MAP3K12	6.88	6.61	6.28
212457_at	transcription facto	TFE3	10.30	10.06	10.15
213922_at	tau tubulin kinase	TTBK2	8.19	8.19	8.43
215720_s_at	nuclear transcript	NFYA	6.91	6.67	6.80
216922_x_at	deleted in azoosp	DAZ1 /// DAZ2 ///	4.02	4.00	3.90
205037_at	intraflagellar trans	IFT27	6.72	6.97	6.67
219818_s_at	G patch domain c	GPATCH1	7.79	7.71	7.85
219729_at	paired related hor	PRRX2	7.86	7.83	7.90
210518_at	cadherin 8, type 2	CDH8	5.71	5.83	5.88
221247_s_at	Williams-Beuren s	WBSCR16	8.07	8.04	8.13
207221_at	coagulation facto	F2RL3	4.39	4.03	4.21

219913_s_at	crooked neck pre-	CRNKL1	8.91	8.88	8.95
200859_x_at	filamin A, alpha	FLNA	9.23	8.97	9.06
220703_at	chromosome 10 c	C10orf110	5.93	5.85	5.65
87100_at	abhydrolase doma	ABHD2	4.95	5.09	4.98
208440_at	chromosome 3 op	C3orf27	5.65	5.73	5.57
202768_at	FBJ murine osteos	FOSB	4.65	4.86	4.81
204095_s_at	elongation factor	ELL	6.71	6.50	6.37
220580_at	bicaudal C homolo	BICC1	4.13	4.22	4.37
212108_at	Fas associated fac	FAF2	10.38	10.42	10.50
219030_at	TP53RK binding pr	TPRKB	10.41	10.62	10.64
31807_at	DEAD (Asp-Glu-Ala	DDX49	9.42	9.33	9.40
205843_x_at	carnitine O-acetyl	CRAT	8.38	8.23	7.96
211093_at	phosphodiesteras	PDE6C	3.11	3.00	3.08
204847_at	zinc finger and BT	ZBTB11	9.77	9.95	10.09
207440_at	solute carrier fam	SLC35A2	6.10	5.62	5.66
204465_s_at	internexin neuron	INA	5.71	5.66	5.35
215107_s_at	tetratricopeptide	TTC22	3.74	3.88	3.87
204182_s_at	zinc finger and BT	ZBTB43	7.64	7.89	7.88
203634_s_at	carnitine palmitoy	CPT1A	3.95	3.84	3.83
211602_s_at	transient receptor	TRPC1	5.14	5.41	5.02
204340_at	transmembrane p	TMEM187	7.51	7.51	7.57
203061_s_at	mediator of DNA-	MDC1	7.01	7.04	6.95
217302_at	olfactory receptor	OR2F2	4.11	4.18	3.90
209370_s_at	SH3-domain bindi	SH3BP2	7.85	7.75	7.44
216861_at	---	---	3.87	4.14	3.72
213923_at	RAP2B, member c	RAP2B	9.80	9.77	10.00
212059_s_at	transient receptor	TRPC4AP	7.09	6.95	7.22
221363_x_at	G protein-couplec	GPR25	4.65	4.20	4.50
203350_at	adaptor-related p	AP1G1	8.15	8.27	8.01
221196_x_at	BRCA1/BRCA2-cor	BRCC3	8.57	8.79	8.57
202728_s_at	latent transformir	LTBP1	6.31	6.24	5.97
212571_at	chromodomain he	CHD8	8.24	8.47	8.23
221920_s_at	solute carrier fam	SLC25A37	7.77	7.67	7.98
202157_s_at	CUGBP, Elav-like f	CELF2	5.71	5.16	5.60
216141_at	---	---	6.28	6.23	6.15
217609_at	leucine rich repea	LRRC23	5.99	6.09	5.95
211562_s_at	leiomodin 1 (smo	LMOD1	5.37	5.17	4.80
213800_at	complement facto	CFH	7.38	7.57	7.70
200052_s_at	interleukin enhan	ILF2	11.51	11.59	11.56
207270_x_at	CD300c molecule	CD300C	6.94	6.93	6.61
217921_at	mannosidase, alpl	MAN1A2	6.31	6.62	6.56
218793_s_at	sex comb on midl	SCML1	8.73	8.81	9.11
216065_at	---	---	4.42	4.36	4.19
43511_s_at	---	---	6.05	6.19	6.11
202210_x_at	glycogen synthase	GSK3A	8.09	7.98	7.81
201152_s_at	muscleblind-like (	MBNL1	11.90	11.99	12.03
221008_s_at	alanine-glyoxylate	AGXT2L1	3.71	3.39	3.29

201127_s_at	ATP citrate lyase	ACLY	9.41	9.54	9.49
207570_at	short stature hom	SHOX	5.05	4.64	4.78
215313_x_at	major histocompa	HLA-A	12.80	12.73	12.72
208760_at	Ubiquitin-conjuga	UBE2I	7.89	8.14	8.01
210498_at	clathrin, heavy ch	CLTC	3.70	3.61	3.50
206836_at	solute carrier fam	SLC6A3	7.52	7.40	7.05
220521_s_at	ATG16 autophagy	ATG16L1	6.56	6.39	6.56
216815_at	---	---	4.71	4.52	4.31
204542_at	ST6 (alpha-N-acet	ST6GALNAC2	4.39	4.72	4.39
213415_at	chloride intracellu	CLIC2	3.72	3.38	3.31
209677_at	protein kinase C, i	PRKCI	5.59	5.65	5.91
217321_x_at	Ataxin 3	ATXN3	4.51	4.33	4.42
217660_at	myosin, heavy cha	MYH14	6.95	6.72	6.82
219790_s_at	natriuretic peptid	NPR3	6.80	6.65	6.86
205464_at	sodium channel, r	SCNN1B	5.01	4.83	4.63
206028_s_at	c-mer proto-onco	MERTK	5.12	4.96	4.96
209668_x_at	carboxylesterase 2	CES2	7.62	7.83	7.68
47553_at	deafness, autosor	DFNB31	4.09	3.81	3.90
208912_s_at	2',3'-cyclic nucleo	CNP	9.51	9.50	9.59
207443_at	nuclear receptor s	NR2E1	4.49	4.27	4.56
201988_s_at	cAMP responsive	CREBL2	6.99	7.10	7.22
208697_s_at	eukaryotic transla	EIF3E	13.63	13.64	13.73
201570_at	sorting and assem	SAMM50	10.19	10.06	10.11
206997_s_at	heparan sulfate 6-	HS6ST1	6.77	6.54	6.70
207035_at	solute carrier fam	SLC30A3	7.58	7.81	7.72
220821_at	galanin receptor 1	GALR1	3.89	3.86	3.74
219745_at	transmembrane p	TMEM180	6.15	6.01	6.11
206882_at	solute carrier fam	SLC1A6	4.49	4.24	4.33
219945_at	DEAD (Asp-Glu-Al	DDX25	3.72	3.63	3.57
206687_s_at	protein tyrosine p	PTPN6	7.15	7.16	6.78
212830_at	multiple EGF-like-	MEGF9	8.44	8.38	8.44
207585_s_at	ribosomal protein	RPL36AL	12.68	12.72	12.75
204896_s_at	prostaglandin E re	PTGER4	5.42	5.53	5.43
210374_x_at	prostaglandin E re	PTGER3	4.33	4.40	4.08
204645_at	cyclin T2	CCNT2	7.47	7.43	7.53
202867_s_at	DnaJ (Hsp40) hom	DNAJB12	7.74	7.75	7.87
220416_at	ATPase, class I, ty	ATP8B4	3.63	3.81	3.61
209351_at	keratin 14	KRT14	4.24	4.23	4.17
213372_at	progestin and adi	PAQR3	8.81	9.03	9.33
215186_at	TBC1 domain fam	TBC1D30	5.72	5.60	5.47
212663_at	FK506 binding pro	FKBP15	8.76	8.65	8.47
210239_at	iroquois homeobo	IRX5	5.80	5.76	5.64
218088_s_at	Ras-related GTP b	RRAGC	9.62	9.68	9.62
215519_x_at	small G protein si	SGSM3	6.61	6.75	6.84
201126_s_at	mannosyl (alpha-1	MGAT1	8.53	8.47	8.48
218852_at	protein phosphata	PPP2R3C	9.72	9.74	9.87
210673_x_at	NK2 homeobox 1	NKX2-1	6.27	6.06	6.54

214982_at	small nuclear ribo	SNRNP200		4.51	4.58	4.52
221233_s_at	family with seque	FAM135A		3.21	3.11	3.24
217583_at	phenylalanine hyc	PAH		3.26	3.47	3.38
208740_at	Sin3A-associated		SAP18		7.93	7.90	8.18
211096_at	pre-B-cell leukemi	PBX2		7.02	6.83	7.09
65884_at	mannosidase, alpl	MAN1B1		7.96	7.82	7.75
205275_at	GTP binding prote	GTPBP1		6.43	6.18	6.44
215633_x_at	leukocyte specific	LST1		5.16	5.31	5.28
213727_x_at	metallophosphoe:	MPPE1		8.52	8.56	8.70
210703_at	---	---		3.51	3.25	3.40
217448_s_at	TOX high mobility	TOX4		6.39	6.23	6.31
38290_at	regulator of G-prc	RGS14		5.91	6.06	6.05
214336_s_at	coatomer protein	COPA		6.39	6.44	6.51
219906_at	endogenous Born	FLJ10213		7.68	7.70	7.78
203463_s_at	epsin 2	EPN2		7.62	7.73	7.63
201301_s_at	annexin A4	ANXA4		10.24	10.29	10.30
210545_at	intersectin 2	ITSN2		4.87	4.66	4.38
213697_at	homeodomain int	HIPK3		4.35	4.18	4.21
218816_at	leucine rich repea	LRRC1		7.96	8.05	8.30
218331_s_at	chromosome 10 c	C10orf18		10.65	10.58	10.95
214117_s_at	biotinidase	BTD		5.86	6.03	5.95
214466_at	gap junction prote	GJA5		4.98	4.53	4.60
211192_s_at	CD84 molecule	CD84		3.98	4.39	3.97
214019_at	---	---		5.83	5.64	5.52
218756_s_at	dehydrogenase/re	DHRS11		5.82	5.54	5.22
210922_at	---	---		4.19	4.17	3.89
214780_s_at	myosin IXB	MYO9B		7.50	7.53	7.50
213256_at	membrane-associ		3-Mar	6.73	6.89	6.63
221080_s_at	DENN/MADD don	DENND1C		5.64	5.52	5.12
215483_at	A kinase (PRKA) ai	AKAP9		4.12	4.31	4.14
206693_at	interleukin 7	IL7		5.93	5.89	6.02
220633_s_at	heterochromatin	HP1BP3		7.92	7.95	7.99
202839_s_at	fucosidase, alpha-	FUCA1		10.20	10.03	10.31
202539_s_at	3-hydroxy-3-meth	HMGCR		9.34	9.75	9.53
216472_at	---	---		5.86	5.97	5.59
208932_at	protein phosphata	PPP4C		9.39	9.39	9.25
202767_at	acid phosphatase	ACP2		7.95	7.76	8.08
35147_at	MCF.2 cell line de	MCF2L		6.16	6.30	6.38
216064_s_at	aspartylglucosami	AGA		6.91	7.01	7.10
215916_at	cholinergic recept	CHRNE		5.86	5.51	5.62
207935_s_at	keratin 13	KRT13		4.10	4.03	3.74
202132_at	WW domain cont:	WWTR1		7.79	7.75	7.74
211803_at	cyclin-dependent	CDK2		4.31	4.12	4.05
210268_at	nuclear transcript	NFX1		5.59	5.79	5.66
220422_at	ubiquilin 3	UBQLN3		4.03	3.94	3.79
219116_s_at	DCN1, defective ir	DCUN1D2		7.46	7.63	7.67
204156_at	SIK family kinase 3	SIK3		4.74	4.67	5.01

207437_at	neuro-oncological	NOVA1	3.65	3.39	3.50
216505_x_at	ribosomal protein	RPS10P5	11.74	11.71	11.75
215802_at	---	---	5.58	5.55	5.21
213618_at	ArfGAP with RhoG	ARAP2	6.65	6.44	7.00
202029_x_at	ribosomal protein	RPL38	14.35	14.32	14.40
210049_at	serpin peptidase i	SERPINC1	4.51	4.44	4.26
222125_s_at	prolyl 4-hydroxyla	P4HTM	7.50	7.40	7.59
209071_s_at	regulator of G-pro	RGS5	6.66	6.84	6.73
222020_s_at	neurotrimin	NTM	5.11	4.89	4.76
200871_s_at	prosaposin	PSAP	12.18	11.94	12.11
221818_at	integrator comple	INTS5	7.96	7.93	7.76
221752_at	slingshot homolog	SSH1	9.43	9.36	9.49
215181_at	cadherin 22, type	CDH22	7.24	7.23	7.26
206487_at	Sad1 and UNC84 (	SUN1	7.25	7.29	7.34
218696_at	eukaryotic transla	EIF2AK3	8.62	8.82	9.00
219662_at	chromosome 2 op	C2orf49	7.90	8.00	8.06
205344_at	chondroitin sulfat	CSPG5	6.43	6.37	6.02
220669_at	OTU domain conta	OTUD4	6.20	6.22	6.44
220728_at	---	---	5.98	6.38	6.25
221152_at	collagen, type VIII	COL8A1	3.48	3.28	3.29
207681_at	chemokine (C-X-C	CXCR3	5.15	5.18	5.13
204358_s_at	fibronectin leucin	FLRT2	6.52	6.29	6.49
218387_s_at	6-phosphoglucono	PGLS	9.06	8.96	9.13
221867_at	NEDD4 binding pr	N4BP1	6.57	6.62	6.38
209264_s_at	tetraspanin 4	TSPAN4	9.34	9.24	9.30
201459_at	RuvB-like 2 (E. col	RUVBL2	11.69	11.61	11.67
214166_at	succinate dehydro	SDHB	5.35	5.34	5.54
211861_x_at	CD28 molecule	CD28	3.61	3.45	3.21
220577_at	GTPase, very large	GVIN1	4.77	5.03	4.75
206646_at	GLI family zinc fin	GLI1	7.68	7.06	7.53
208061_at	---	---	3.95	4.08	3.89
211746_x_at	proteasome (pros	PSMA1	12.44	12.46	12.56
210345_s_at	dynein, axonemal	DNAH9	6.45	6.34	6.49
222210_at	KIAA0195	KIAA0195	6.85	6.47	6.47
221081_s_at	DENN/MADD dom	DENND2D	4.91	5.07	4.70
207373_at	homeobox D10	HOXD10	4.34	4.21	3.95
207130_at	zinc finger, MYND	ZMYND8	6.71	6.93	6.91
215131_at	IQ motif containir	IQCK	6.16	6.32	6.19
221735_at	WD repeat domai	WDR48	8.76	8.69	8.73
202986_at	aryl-hydrocarbon	ARNT2	5.91	5.54	5.75
201264_at	coatomer protein	COPE	7.81	7.85	7.91
210955_at	caspase 10, apopt	CASP10	4.63	4.32	4.21
221443_x_at	prolactin releasing	PRLH	6.95	7.27	6.93
220695_at	---	---	4.89	4.95	4.70
201191_at	phosphatidylinosi	PITPNA	7.78	7.77	7.84
213247_at	sushi, von Willebr	SVEP1	5.27	5.33	5.04
218859_s_at	ESF1, nucleolar pr	ESF1	9.86	10.00	10.11

202046_s_at	glucocorticoid rec	GRLF1	5.46	5.23	5.35
201769_at	clathrin interactor	CLINT1	10.85	10.69	11.03
213809_x_at	---	---	7.38	7.41	7.45
213814_s_at	Syntrophin, beta 2	SNTB2	3.85	3.93	3.95
214552_s_at	rabaptin, RAB GTF	RABEP1	6.93	6.96	7.04
201349_at	solute carrier fam	SLC9A3R1	6.98	6.98	6.98
220343_at	phosphodiesteras	PDE7B	4.94	4.67	4.71
221329_at	olfactory receptor	OR52A1	3.57	3.61	3.21
216704_at	---	---	4.53	4.29	4.58
209497_s_at	RNA binding moti	RBM4B	6.95	7.14	6.86
204895_x_at	mucin 4, cell surfa	MUC4	5.12	4.81	4.88
219160_s_at	poly(A) polymeras	PAPOLG	4.25	4.39	4.30
212268_at	serpin peptidase i	SERPINB1	9.67	9.56	9.62
205103_at	chromosome 1 op	C1orf61	6.97	7.16	7.02
215515_at	---	---	7.51	7.53	7.29
218642_s_at	coiled-coil-helix-c	CHCHD7	8.50	8.64	8.58
200730_s_at	protein tyrosine p	PTP4A1	8.58	8.47	8.68
204108_at	nuclear transcript	NFYA	8.57	8.66	8.70
220908_at	coiled-coil domair	CCDC33	5.78	5.60	5.61
203196_at	ATP-binding casse	ABCC4	9.53	9.52	9.76
219102_at	reticulocalbin 3, E	RCN3	7.21	7.05	6.97
214351_x_at	ribosomal protein	RPL13	14.51	14.44	14.43
215428_at	---	---	4.91	5.22	5.18
206313_at	major histocompa	HLA-DOA	7.18	6.95	6.87
220985_s_at	ring finger proteir	RNF170	7.41	7.61	7.76
207242_s_at	glutamate recepto	GRIK1	4.27	3.96	4.04
220999_s_at	cytoplasmic FMR1	CYFIP2	5.83	6.31	6.07
206319_s_at	serine peptidase i	SPINLW1	3.43	3.51	3.47
202245_at	lanosterol synthas	LSS	10.48	10.59	10.44
205811_at	polymerase (DNA	POLG2	8.87	8.92	8.87
219649_at	asparagine-linked	ALG6	8.62	8.59	8.63
215269_at	trafficking protein	TRAPPC10	6.49	6.70	6.33
217818_s_at	actin related prot	ARPC4	9.29	9.25	9.10
208354_s_at	solute carrier fam	SLC12A3	6.27	6.37	6.01
210645_s_at	tetratricopeptide	TTC3	10.65	10.80	10.75
206619_at	dickkopf homolog	DKK4	5.68	5.46	5.27
221077_at	armadillo repeat c	ARMC4	5.65	5.83	5.81
210406_s_at	RAB6A, member F	RAB6A /// RAB6C	10.63	10.66	10.80
207483_s_at	cullin-associated a	CAND1	10.03	10.12	10.07
214058_at	v-myc myelocytor	MYCL1	4.43	4.34	3.87
221340_at	caudal type home	CDX4	6.31	6.23	6.31
212948_at	calmodulin bindin	CAMTA2	8.42	8.18	8.32
204392_at	calcium/calmodul	CAMK1	6.61	6.72	6.57
221962_s_at	ubiquitin-conjuga	UBE2H	8.42	8.29	8.47
215881_x_at	synovial sarcoma,	SSX2 /// SSX2B ///	6.14	6.16	5.84
216180_s_at	synaptojanin 2	SYNJ2	7.82	7.77	7.89
210587_at	inhibin, beta E	INHBE	9.16	8.32	9.18

202779_s_at	ubiquitin-conjugat	UBE2S	12.29	12.18	12.18
206702_at	TEK tyrosine kinas	TEK	3.53	3.65	3.68
204953_at	synaptosomal-ass	SNAP91	3.85	3.41	3.45
209639_s_at	regulator of G-prc	RGS12	6.12	5.95	5.76
215641_at	SEC24 family, mer	SEC24D	4.39	4.51	4.25
211287_x_at	colony stimulating	CSF2RA	5.17	5.02	4.68
214134_at	Chromosome 2 op	C2orf55	6.00	5.83	5.76
214072_x_at	hypothetical prote	LOC100287008	6.71	6.86	6.35
217168_s_at	homocysteine-ind	HERPUD1	12.22	11.95	12.26
207667_s_at	mitogen-activatec	MAP2K3	7.73	7.67	7.64
216524_x_at	---	---	8.53	8.54	8.31
206377_at	forkhead box F2	FOXF2	10.05	9.87	10.03
209285_s_at	chromosome 3 op	C3orf63	9.53	9.53	9.63
222182_s_at	CCR4-NOT transcr	CNOT2	9.78	9.67	9.85
202914_s_at	Rho guanine nucle	ARHGEF11	5.98	6.26	5.67
202009_at	twinfilin, actin-bin	TWF2	9.03	8.85	8.78
221266_s_at	transmembrane 7	TM7SF4	4.02	4.00	3.99
222276_at	methyltransferase	METTL2B	6.83	6.79	6.89
222203_s_at	retinol dehydroge	RDH14	9.71	9.80	9.84
212626_x_at	heterogeneous nu	HNRNPC	11.53	11.56	11.65
212882_at	kelch-like 18 (Dros	KLHL18	9.01	9.13	9.03
202979_s_at	CREB/ATF bZIP tra	CREBZF	8.66	8.58	8.98
209880_s_at	selectin P ligand	SELPLG	8.01	7.78	7.66
214686_at	zinc finger proteir	ZNF266	8.23	8.32	8.12
201619_at	peroxiredoxin 3	PRDX3	11.38	11.50	11.67
204697_s_at	chromogranin A (p	CHGA	4.94	4.82	4.86
203254_s_at	talin 1	TLN1	6.76	6.77	6.74
220107_s_at	family with seque	FAM164C	5.22	5.39	4.93
220478_at	apolipoprotein L,	APOL5	4.82	4.75	4.38
203484_at	Sec61 gamma sub	SEC61G	11.78	11.76	11.79
207276_at	cerebellar degene	CDR1	3.76	3.81	3.95
219059_s_at	lymphatic vessel e	LYVE1	4.58	4.68	4.58
203111_s_at	PTK2B protein tyr	PTK2B	6.40	6.24	6.24
216766_at	---	---	3.80	3.81	3.85
219912_s_at	---	---	4.03	3.82	3.78
218456_at	caprin family men	CAPRIN2	8.64	8.80	8.79
206599_at	solute carrier fam	SLC16A5	7.26	7.15	7.03
209728_at	major histocompa	HLA-DRB4	4.30	4.58	3.79
209219_at	RD RNA binding p	RDBP	10.88	10.85	10.93
222307_at	hypothetical prote	LOC282997	4.91	5.13	5.01
200030_s_at	solute carrier fam	SLC25A3	13.72	13.69	13.69
207778_at	regenerating islet-	REG1P	4.90	4.48	4.45
215302_at	hypothetical prote	LOC257152	4.99	5.12	4.62
215796_at	---	---	3.46	3.54	3.39
216458_at	PQ loop repeat co	PQLC3	7.19	7.06	6.60
221288_at	G protein-couplec	GPR22	4.29	4.04	3.94
219334_s_at	oligonucleotide/o	OBFC2A	4.44	4.51	4.88

218204_s_at	FYVE and coiled-c	FYCO1	8.79	8.73	9.00
207552_at	ATP synthase, H+	ATP5G2	5.28	5.61	5.27
206069_s_at	acyl-CoA dehydro	ACADL	3.65	3.85	3.38
203004_s_at	myocyte enhance	MEF2D	7.27	7.15	7.00
204934_s_at	hepsin	HPN	6.36	6.24	6.02
213770_at	kinase suppressor	KSR1	7.08	6.73	6.70
214156_at	myosin VIIA and R	MYRIP	3.24	3.26	3.15
220852_at	PRO1768	PRO1768	4.41	4.05	3.94
214405_at	---	---	3.35	3.15	3.30
210134_x_at	short stature hom	SHOX2	6.12	5.56	5.69
206501_x_at	ets variant 1	ETV1	7.21	7.09	6.92
218728_s_at	cornichon homolo	CNIH4	10.41	10.42	10.45
210981_s_at	G protein-couplec	GRK6	7.64	7.76	7.47
205060_at	poly (ADP-ribose)	PARG	8.83	8.84	8.94
221757_at	phosphoinositide-	PIK3IP1	7.78	7.97	7.42
216170_at	---	---	4.70	5.02	4.52
208642_s_at	X-ray repair comp	XRCC5	12.59	12.63	12.54
212707_s_at	similar to calcium	LOC100132214 ///	7.46	6.77	6.89
217860_at	similar to NADH d	LOC732160 /// ND	10.38	10.33	10.39
205487_s_at	vestigial like 1 (Dr	VGLL1	6.37	6.09	5.97
212010_s_at	CDV3 homolog (m	CDV3	12.66	12.60	12.69
206242_at	transmembrane 4	TM4SF5	6.83	6.43	6.49
213658_at	---	---	4.92	4.48	4.62
216198_at	activating transcri	ATF7IP	4.13	4.35	4.12
206097_at	solute carrier fam	SLC22A18AS	6.99	7.02	7.02
210064_s_at	uroplakin 1B	UPK1B	4.44	4.58	4.48
216275_at	budding uninhibit	BUB1	5.96	5.82	5.62
216538_at	---	---	5.64	5.41	5.61
212885_at	M-phase phospho	MPHOSPH10	9.63	9.72	9.81
213871_s_at	Chromosome 6 op	C6orf108	6.25	6.24	6.04
218399_s_at	cell division cycle	CDCA4	9.71	9.56	9.76
209803_s_at	pleckstrin homolo	PHLDA2	11.42	11.35	11.44
215075_s_at	growth factor rec	GRB2	9.37	9.28	9.29
209874_x_at	cyclin M2	CNNM2	6.81	6.94	7.10
214495_at	calcium channel, v	CACNG2	4.43	4.24	4.40
214799_at	neurofascin homo	NFASC	4.05	3.90	4.00
220680_at	ribonucleoprotein	RAVER2	3.74	3.54	3.62
206357_at	optic atrophy 3 (a	OPA3	6.81	6.70	6.76
204594_s_at	Smith-Magenis sy	SMCR7L	8.81	8.75	8.83
219310_at	transmembrane p	TMEM90B	5.37	5.21	5.13
204101_at	myotubularin 1	MTM1	5.58	5.75	5.31
202176_at	excision repair cro	ERCC3	8.04	8.09	8.10
220746_s_at	ubiquitin interacti	UIMC1	9.64	9.55	9.64
219274_at	tetraspanin 12	TSPAN12	3.50	3.51	3.10
37577_at	Rho GTPase activa	ARHGAP19	8.04	8.17	8.25
201686_x_at	apoptosis inhibito	API5	6.66	6.92	6.96
216500_at	---	---	7.13	7.08	6.90

204606_at	chemokine (C-C m	CCL21	6.47	6.42	6.43
202834_at	serpin peptidase i	SERPINA1	6.15	5.96	5.67
217489_s_at	interleukin 6 rece	IL6R	3.66	3.50	3.24
210040_at	solute carrier fam	SLC12A5	3.74	3.94	3.53
200739_s_at	SMT3 suppressor	SUMO3	9.93	9.98	9.93
220429_at	N-deacetylase/N-s	NDST3	3.69	3.82	3.58
221849_s_at	DDB1 and CUL4 as	DCAF15	5.80	5.70	5.47
205230_at	rabphilin 3A homo	RPH3A	5.90	5.84	5.78
210088_x_at	myosin, light chai	MYL4	6.80	7.02	6.58
217062_at	hypothetical prote	LOC100287204	6.70	6.56	6.37
211530_x_at	major histocompa	HLA-G	10.20	10.04	10.02
212645_x_at	brain and reprodu	BRE	8.88	8.76	8.60
217985_s_at	bromodomain adj	BAZ1A	9.13	9.19	9.52
209713_s_at	solute carrier fam	SLC35D1	5.97	5.82	5.63
216906_at	suppression of tur	ST14	5.17	5.09	4.89
203475_at	cytochrome P450,	CYP19A1	4.76	4.98	4.96
200657_at	solute carrier fam	SLC25A5	13.30	13.29	13.26
218082_s_at	upstream binding	UBP1	10.51	10.48	10.65
200682_s_at	ubiquitin-conjugat	UBE2L3	11.52	11.64	11.68
212172_at	adenylate kinase 2	AK2	6.43	6.42	6.17
207423_s_at	ADAM metallopep	ADAM20	5.10	4.87	4.91
212482_at	required for meio	RMND5A	9.36	9.08	9.37
201953_at	calcium and integ	CIB1	9.84	9.96	9.93
220027_s_at	Ras interacting pr	RASIP1	8.97	8.78	8.84
42361_g_at	coiled-coil alpha-h	CCHCR1	6.55	6.38	6.57
41387_r_at	lysine (K)-specific	KDM6B	8.04	8.16	8.25
212327_at	LIM and calponin	LIMCH1	10.24	10.25	10.38
218938_at	F-box and leucine-	FBXL15	9.25	9.08	9.08
220166_at	cyclin M1	CNNM1	4.19	4.21	3.76
209976_s_at	cytochrome P450,	CYP2E1	4.18	4.26	3.83
200960_x_at	clathrin, light chai	CLTA	12.24	12.26	12.32
206029_at	ankyrin repeat do	ANKRD1	4.98	5.09	4.97
217421_at	piwi-like 2 (Droso	PIWIL2	5.97	6.16	5.60
219417_s_at	chromosome 17 o	C17orf59	7.32	7.26	7.05
213694_at	round spermatid b	RSBN1	8.43	8.41	8.51
207084_at	POU class 3 home	POU3F2	5.68	5.73	5.51
221238_at	high-mobility grou	HMGN5	4.78	4.71	4.85
219550_at	roundabout, axon	ROBO3	7.44	7.10	7.16
205459_s_at	neuronal PAS dom	NPAS2	7.17	7.12	6.83
203449_s_at	telomeric repeat b	TERF1	8.53	8.71	8.81
211954_s_at	importin 5	IPO5	11.45	11.42	11.52
214991_s_at	phosphatidylinosi	PIGO	6.39	6.30	6.37
214084_x_at	neutrophil cytoso	NCF1C	5.94	5.91	5.52
203510_at	met proto-oncoge	MET	11.96	11.91	12.10
202827_s_at	serine peptidase i	SPINT1	5.46	5.40	5.13
206585_at	makorin ring finge	MKRN3	4.95	4.74	4.80
208575_at	histone cluster 1,	HIST1H3A	4.17	3.82	3.73

209422_at	PHD finger protei	PHF20	9.37	9.42	9.60
222051_s_at	E2F transcription	E2F5	3.50	3.51	3.51
215216_at	vacuolar protein s	VPS16	4.23	4.12	4.01
212050_at	WAS/WASL intera	WIPF2	8.66	8.66	8.82
215201_at	RALBP1 associate	REPS1	4.88	4.81	4.89
221572_s_at	solute carrier fam	SLC26A6	6.84	6.94	6.76
203059_s_at	3'-phosphoadeno	PAPSS2	7.19	7.06	6.67
204861_s_at	NLR family, apopt	NAIP	6.58	6.85	6.32
221361_at	olfactory marker	OMP	5.72	5.55	5.06
212183_at	nudix (nucleoside	NUDT4	8.72	8.57	8.65
221136_at	growth differentia	GDF2	7.17	7.22	6.99
218070_s_at	GDP-mannose pyr	GMPPA	7.69	7.94	7.72
212309_at	cytoplasmic linker	CLASP2	8.37	8.40	8.38
210167_s_at	thyrotrophic emb	TEF	4.52	4.50	4.15
205257_s_at	amphiphysin	AMPH	4.62	4.22	4.19
215560_x_at	Mitochondrial tra	MTRF1L	5.76	5.88	5.60
201510_at	E74-like factor 3 (	ELF3	5.98	5.80	5.67
210835_s_at	C-terminal binding	CTBP2	11.78	11.66	11.78
213635_s_at	Scaffold attachme	SAFB	4.86	5.19	5.29
213311_s_at	transcription facto	TCF25	10.44	10.24	10.38
37408_at	mannose recepto	MRC2	9.84	9.76	9.77
218216_x_at	ADP-ribosylation-	ARL6IP4	10.53	10.51	10.39
214749_s_at	armadillo repeat	ARMCX6 /// LOC6	10.16	10.15	10.13
203519_s_at	UPF2 regulator of	UPF2	9.56	9.69	9.74
209002_s_at	calcium binding a	CALCOCO1	6.40	6.27	6.32
208245_at	RAB9B, member F	RAB9BP1	4.24	4.21	4.01
212072_s_at	casein kinase 2, al	CSNK2A1	10.89	10.75	10.95
215868_x_at	---	---	4.78	4.73	4.58
211094_s_at	neurofibromin 1	NF1	5.93	6.21	5.87
212935_at	MCF.2 cell line de	MCF2L	4.79	5.27	4.91
205143_at	neurocan	NCAN	5.85	5.53	5.27
206957_at	alanine-glyoxylate	AGXT	4.56	4.62	4.63
202197_at	myotubularin rela	MTMR3	8.15	8.22	8.01
215340_at	adenylate cyclase	ADCY1	5.66	5.38	5.36
211667_x_at	---	---	5.79	5.77	5.52
208574_at	SRY (sex determin	SOX14	4.61	4.57	4.23
220338_at	Ral GEF with PH d	RALGPS2	4.37	4.26	4.03
207830_s_at	protein phosphat	PPP1R8	10.62	10.73	10.59
221413_at	potassium voltage	KCNAB3	4.34	3.98	4.10
211748_x_at	prostaglandin D2	PTGDS	6.80	6.88	6.91
206537_at	X-linked inhibitor	XIAP	5.08	4.95	4.99
218824_at	PNMA-like 1	PNMAL1	4.07	3.87	3.85
201738_at	eukaryotic transla	EIF1B	10.89	10.78	11.05
214470_at	killer cell lectin-lik	KLRB1	5.24	5.28	5.20
206925_at	ST8 alpha-N-acety	ST8SIA4	3.98	3.76	3.99
221298_s_at	solute carrier fam	SLC22A8	6.98	6.71	6.51
210632_s_at	sarcoglycan, alpha	SGCA	4.70	4.33	4.81

221160_s_at	calcium binding p	CABP5	4.73	4.59	4.68
210237_at	artemin	ARTN	5.91	6.03	5.87
211239_s_at	ADAM metallope	ADAM7	3.26	3.06	2.82
201523_x_at	ubiquitin-conjuga	UBE2N	10.17	10.38	10.28
219191_s_at	bridging integrato	BIN2	4.60	4.28	4.10
62987_r_at	calcium channel, v	CACNG4	9.58	9.73	9.56
201310_s_at	chromosome 5 op	C5orf13	11.37	11.52	11.38
214790_at	SUMO1/sentrin sp	SENP6	7.82	7.63	8.00
207891_s_at	HAUS augmin-like	HAUS7 /// TREX2	7.45	7.42	7.60
216114_at	NCK interacting p	NCKIPSD	5.86	5.97	5.56
215990_s_at	B-cell CLL/lympho	BCL6	8.35	8.33	8.13
214218_s_at	X (inactive)-specif	XIST	8.35	8.65	8.51
218971_s_at	WD repeat domai	WDR91	6.44	6.64	6.14
204228_at	peptidylprolyl iso	PPIH	10.65	10.67	10.70
214124_x_at	FGFR1 oncogene	FGFR1OP	8.56	8.56	8.70
214534_at	histone cluster 1,	HIST1H1B	5.56	5.40	5.13
209398_at	histone cluster 1,	HIST1H1C	8.48	8.29	8.31
211631_x_at	UDP-Gal:betaGlcN	B4GALT1	5.43	5.19	5.41
216319_at	---	---	3.43	3.61	3.48
217864_s_at	protein inhibitor c	PIAS1	8.47	8.60	8.69
219645_at	calsequestrin 1 (fa	CASQ1	3.83	3.84	3.51
221325_at	potassium channe	KCNK13	4.27	4.49	4.11
218701_at	lactamase, beta 2	LACTB2	7.66	7.84	8.05
202807_s_at	drebrin 1	DBN1	6.89	6.71	6.77
212809_at	nuclear factor of a	NFATC2IP	8.37	8.46	8.48
207269_at	defensin, alpha 4,	DEFA4	4.99	5.15	5.12
204769_s_at	transporter 2, ATF	TAP2	7.98	7.96	7.97
222234_s_at	dysbindin (dystrol	DBNDD1	6.15	5.80	5.76
214122_at	PDZ and LIM dom	PDLIM7	7.71	7.44	7.75
221352_at	---	---	4.01	4.10	3.72
202852_s_at	alpha- and gamma	AAGAB	7.51	7.27	7.39
221987_s_at	TSR1, 20S rRNA ac	TSR1	8.40	8.28	8.58
214796_at	chromosome 8 op	C8orf79	4.03	3.81	3.87
205806_at	retinal outer segm	ROM1	7.15	6.62	6.74
217742_s_at	WW domain conta	WAC	10.43	10.35	10.49
212076_at	myeloid/lymphoic	MLL	8.18	8.19	8.04
212542_s_at	pleckstrin homolo	PHIP	9.46	9.60	9.67
217650_x_at	ST3 beta-galactos	ST3GAL2	8.54	8.39	8.28
217429_at	---	---	4.56	4.64	4.31
221626_at	zinc finger proteir	ZNF506	5.18	5.12	5.22
210939_s_at	glutamate recepto	GRM1	4.83	4.57	4.70
221297_at	G protein-couplec	GPRC5D	5.09	5.31	5.01
206180_x_at	zinc finger proteir	ZNF747	8.69	8.53	8.56
200685_at	splicing factor, arg	SFRS11	8.29	8.39	8.77
200988_s_at	proteasome (pros	PSME3	7.99	8.10	8.04
202521_at	CCCTC-binding fac	CTCF	9.73	9.65	9.79
201653_at	cornichon homolo	CNIH	12.12	12.11	12.34

202704_at	transducer of ERB	TOB1	9.11	9.08	9.22
222098_s_at	---	---	4.60	4.48	4.31
213138_at	AT rich interactive	ARID5A	7.04	6.98	6.93
211743_s_at	proteoglycan 2, bo	PRG2	5.39	4.99	5.29
210294_at	TAP binding prote	TAPBP	5.70	6.07	5.75
216907_x_at	killer cell immuno	KIR3DL1 /// KIR3D	5.61	5.36	5.45
202425_x_at	protein phosphata	PPP3CA	11.10	11.17	11.37
215904_at	myeloid/lymphoid	MLLT4	4.02	4.32	4.17
205752_s_at	glutathione S-tran	GSTM5	5.89	5.96	5.83
202223_at	STT3, subunit of th	STT3A	10.59	10.45	10.53
219521_at	beta-1,3-glucuron	B3GAT1	6.83	6.67	6.29
220384_at	thioredoxin doma	TXNDC3	3.51	3.63	3.64
206737_at	wingless-type MM	WNT11	5.37	5.39	5.15
220847_x_at	zinc finger protein	ZNF221	5.89	5.63	5.75
215586_at	---	---	5.05	5.02	4.78
206985_at	hydroxysteroid (1	HSD17B3	4.65	4.66	4.68
202342_s_at	tripartite motif-co	TRIM2	8.31	8.24	8.46
216798_at	hypothetical prote	FLJ23519 /// RNH1	4.68	4.71	4.28
202326_at	euchromatic histo	EHMT2	6.00	6.14	6.00
207158_at	apolipoprotein B r	APOBEC1	4.60	4.65	4.53
220153_at	ectonucleoside tri	ENTPD7	5.85	5.86	5.85
202866_at	DnaJ (Hsp40) hom	DNAJB12	8.46	8.41	8.50
219285_s_at	ninein (GSK3B inte	NIN	3.93	3.48	3.86
206214_at	phospholipase A2	PLA2G7	3.62	3.58	3.61
211553_x_at	apoptotic peptida	APAF1	4.87	4.99	5.22
213963_s_at	Sin3A-associated p	SAP30	6.72	6.82	6.62
217059_at	mucin 7, secreted	MUC7	3.44	3.61	3.31
222011_s_at	t-complex 1	TCP1	9.28	9.39	9.40
216810_at	keratin associated	KRTAP4-7	3.26	3.41	3.42
209684_at	Ras and Rab intera	RIN2	11.50	11.49	11.69
213673_x_at	hypothetical prote	LOC100287008	5.68	5.51	5.60
218874_s_at	chromosome 6 op	C6orf134	4.88	5.07	4.63
220585_at	hexokinase domai	HKDC1	5.80	5.75	5.32
207226_at	histone cluster 1,	HIST1H2BN	6.09	6.11	5.79
216189_at	hypothetical prote	LOC100289109	4.13	4.20	4.20
222272_x_at	scinderin	SCIN	5.15	5.17	5.00
214637_at	oncostatin M	OSM	6.94	6.91	6.73
201761_at	methylenetetrahy	MTHFD2	13.18	13.13	13.33
210783_x_at	C-type lectin dom	CLEC11A	7.09	6.92	6.69
205359_at	A kinase (PRKA) an	AKAP6	4.43	4.21	4.11
206075_s_at	casein kinase 2, al	CSNK2A1	8.95	8.90	9.02
205172_x_at	clathrin, light chai	CLTB	8.35	8.26	8.38
202814_s_at	TAR (HIV-1) RNA b	TARBP1	8.62	8.96	8.82
207167_at	CD101 molecule	CD101	4.84	4.63	4.39
203928_x_at	microtubule-asso	MAPT	7.36	7.69	7.46
204379_s_at	fibroblast growth	FGFR3	4.36	4.28	4.23
209971_x_at	aminoacyl tRNA sy	AIMP2	12.38	12.19	12.35

207904_s_at	leucyl/cystinyl am	LNPEP	5.45	5.38	5.29
211918_x_at	pappalysin 2	PAPPA2	6.26	6.34	6.30
215383_x_at	spastic paraplegia	SPG21	7.98	8.01	7.79
204854_at	G protein-couplec	GPR162 /// LEPREI	6.94	6.84	6.70
219187_at	FK506 binding prc	FKBPL	7.30	7.27	7.32
203949_at	myeloperoxidase	MPO	5.06	5.11	4.86
209717_at	ecotropic viral int	EVI5	9.77	10.00	10.21
214147_at	chromosome 1 op	C1orf175	6.19	6.11	6.19
212718_at	poly(A) polymeras	PAPOLA	11.58	11.75	11.63
218857_s_at	asparaginase like	ASRGL1	7.00	6.67	6.54
206610_s_at	coagulation factor	F11	6.46	6.16	6.32
215523_at	zinc finger protein	ZNF391	4.83	4.66	4.44
214613_at	G protein-couplec	GPR3	4.52	4.49	4.66
216647_at	Transcription fact	TCF3	7.39	7.13	7.20
216550_x_at	ankyrin repeat do	ANKRD12	6.58	6.81	6.79
205681_at	BCL2-related prot	BCL2A1	3.43	3.64	3.51
205341_at	EH-domain contai	EHD2	7.07	7.17	6.90
40273_at	sphingosine kinas	SPHK2	7.63	7.44	7.42
204337_at	regulator of G-prc	RGS4	3.62	4.32	3.87
212824_at	far upstream elem	FUBP3	8.66	9.00	8.96
207606_s_at	Rho GTPase activa	ARHGAP12	8.13	8.33	8.38
214277_at	COX11 cytochrom	COX11	5.22	5.18	5.28
205333_s_at	RCE1 homolog, pr	RCE1	6.96	6.66	6.83
218547_at	dehydrodolichyl d	DHDDS	8.25	8.30	8.29
207658_s_at	forkhead box G1	FOXG1	5.74	5.73	5.87
204424_s_at	LIM domain only 3	LMO3	3.65	3.82	3.54
205439_at	glutathione S-tran	GSTT2	7.64	7.82	7.73
210353_s_at	solute carrier fam	SLC6A2	4.56	4.48	4.67
214980_at	Ubiquitin protein	UBE3A	6.77	6.65	6.60
218029_at	family with seque	FAM65A	8.28	8.26	8.39
212900_at	SEC24 family, mer	SEC24A	8.96	9.07	9.11
214511_x_at	Fc fragment of IgG	FCGR1B	4.76	4.85	4.75
38964_r_at	Wiskott-Aldrich sy	WAS	7.37	7.39	7.38
220291_at	glycerophosphodi	GDPD2	6.64	6.61	6.26
221490_at	ubiquitin associat	UBAP1	8.50	8.37	8.54
220174_at	leucine rich repea	LRRC8E	6.31	6.45	6.39
212121_at	tectonic family me	TCTN3	9.47	9.46	9.46
208070_s_at	REV3-like, catalyti	REV3L	9.49	9.67	9.86
218712_at	chromosome 1 op	C1orf109	8.62	8.60	8.76
217445_s_at	phosphoribosylgly	GART	6.18	5.75	5.78
202104_s_at	spastic paraplegia	SPG7	7.97	8.33	8.16
221509_at	density-regulated	DENR	11.38	11.45	11.58
201345_s_at	ubiquitin-conjugat	UBE2D2	11.28	11.28	11.30
218973_at	elongation factor	EFTUD1	9.10	9.00	9.13
200775_s_at	heterogeneous nu	HNRNPK	13.08	13.18	13.18
212137_at	La ribonucleoprot	LARP1	12.15	12.10	12.23
215053_at	Snf2-related CREB	SRCAP	4.63	4.39	4.42

204832_s_at	bone morphogene	BMPR1A	7.80	7.92	8.13
208602_x_at	CD6 molecule	CD6	4.11	3.90	4.18
204371_s_at	KH-type splicing re	KHSRP	6.32	6.28	6.12
212171_x_at	vascular endothel	VEGFA	8.45	8.29	8.42
203642_s_at	COBL-like 1	COBLL1	7.13	6.99	7.24
220778_x_at	sema domain, tra	SEMA6B	8.27	8.02	7.90
209549_s_at	deoxyguanosine k	DGUOK	11.00	10.95	11.05
205093_at	pleckstrin homolo	PLEKHA6	5.24	5.47	4.88
201029_s_at	CD99 molecule	CD99	11.61	11.61	11.64
206123_at	lethal giant larvae	LLGL1	7.54	7.43	6.80
205969_at	arylacetamide dea	AADAC	6.97	7.07	6.96
219847_at	histone deacetyla	HDAC11	7.66	7.84	7.49
202340_x_at	nuclear receptor s	NR4A1	7.40	7.46	7.36
217949_s_at	vitamin K epoxide	VKORC1	11.01	10.92	10.97
205325_at	phytanoyl-CoA 2-h	PHYHIP	6.17	6.36	6.12
212891_s_at	growth arrest and	GADD45GIP1	10.07	9.95	10.01
211747_s_at	LSM5 homolog, U	LSM5	11.71	11.91	11.92
206611_at	chromosome 2 op	C2orf27A	6.91	7.00	6.82
217389_s_at	activating transcri	ATF5	8.15	7.78	7.72
215164_at	---	---	3.89	4.10	3.35
211349_at	solute carrier fam	SLC15A1	4.57	4.69	4.29
212680_x_at	protein phosphata	PPP1R14B	11.32	11.22	11.30
221949_at	hypothetical prote	LOC222070	6.77	6.80	6.49
202842_s_at	opioid growth fac	OGFR	8.93	8.68	8.92
204189_at	retinoic acid recep	RARG	7.31	7.27	6.93
202106_at	golgin A3	GOLGA3	9.77	9.70	9.69
213211_s_at	TAF6-like RNA pol	TAF6L	4.73	4.64	4.72
206397_x_at	growth differentia	GDF1 /// LASS1	6.61	6.43	6.26
200654_at	prolyl 4-hydroxyla	P4HB	12.02	11.91	11.91
205641_s_at	TNFRSF1A-associa	TRADD	8.46	8.39	8.54
204926_at	inhibin, beta A	INHBA	4.01	4.01	3.88
210157_at	chromosome 19 o	C19orf2	8.02	8.17	8.14
211331_x_at	hemochromatosis	HFE	6.83	6.89	6.74
202060_at	Ctr9, Paf1/RNA po	CTR9	9.57	9.68	9.71
200070_at	chromosome 2 op	C2orf24	8.50	8.47	8.57
203306_s_at	solute carrier fam	SLC35A1	10.23	10.27	10.30
218198_at	DEAH (Asp-Glu-Ala	DHX32	10.14	10.14	10.23
200027_at	asparaginyl-tRNA	NARS	12.85	12.82	12.95
215804_at	EPH receptor A1	EPHA1	5.60	5.63	5.51
206621_s_at	eukaryotic transla	EIF4H	11.38	11.37	11.50
208324_at	---	---	3.95	3.94	4.15
215209_at	SEC24 family, mer	SEC24D	4.77	4.87	4.73
204814_at	Ca++-dependent s	CADPS	3.90	4.06	3.88
218267_at	cyclin-dependent	CINP	6.99	6.90	6.97
202554_s_at	glutathione S-tran	GSTM3	4.20	4.09	3.91
212068_s_at	HLA-B associated	BAT2L1	9.25	9.13	9.25
220906_at	---	---	3.90	3.42	3.73

216109_at	Mediator complex	MED13L	4.17	4.63	4.93
211286_x_at	colony stimulating	CSF2RA	4.72	4.75	4.62
216046_at	---	---	5.07	4.87	4.97
221931_s_at	SEH1-like (S. cerev	SEH1L	11.45	11.43	11.59
215135_at	aspartyl aminopep	DNPEP	4.46	4.70	4.63
216969_s_at	kinesin family mer	KIF22	7.05	7.10	7.06
212555_at	protein kinase, cA	PRKAR1B	6.71	6.42	5.84
214013_s_at	TBC1 (tre-2/USP6,	TBC1D1	4.98	4.95	4.71
34478_at	RAB11B, member	RAB11B	3.61	3.73	3.83
210713_at	intersectin 1 (SH3	ITSN1	4.24	4.38	4.04
216254_at	parvin, beta	PARVB	4.92	4.99	4.65
214030_at	beta-gamma cryst	CRYBG3	7.50	7.65	7.82
212398_at	radixin	RDX	6.99	7.10	7.31
211107_s_at	aurora kinase C	AURKC	6.32	6.25	5.64
203896_s_at	phospholipase C, I	PLCB4	3.51	3.61	3.30
209132_s_at	COMM domain cc	COMMD4	10.17	10.19	10.09
207817_at	interferon, omega	IFNW1	5.03	4.93	4.96
218284_at	SMAD family men	SMAD3	9.82	9.87	9.98
201153_s_at	muscleblind-like (	MBNL1	11.68	11.77	11.86
AFFX-BioDn-5_a	---	---	11.67	11.70	11.76
206404_at	fibroblast growth	FGF9	6.04	6.07	5.85
221985_at	kelch-like 24 (Dros	KLHL24	7.42	7.33	7.23
214180_at	mannosidase, alpl	MAN1C1	5.21	5.25	5.27
216157_at	---	---	5.06	4.85	4.72
211099_s_at	cyclic nucleotide g	CNGB1	5.13	4.95	4.65
210820_x_at	coenzyme Q7 hon	COQ7	8.35	8.28	8.27
203448_s_at	telomeric repeat I	TERF1	8.26	8.34	8.40
203368_at	cysteine-rich with	CRELD1	6.75	6.88	6.55
211904_x_at	RAD52 homolog (S	RAD52	5.10	5.30	4.93
212013_at	peroxidasin homo	PXDN	4.69	4.37	4.49
212037_at	pinin, desmosome	PNN	9.84	10.09	9.99
220797_at	methyltransferase	METT10D	5.82	5.70	5.83
217219_at	hypothetical LOC2	LOC26102	6.88	6.46	6.44
218293_x_at	nucleoporin 50kD	NUP50	5.13	4.81	5.13
205144_at	major facilitator s	MFSD7	6.38	6.70	6.52
221611_s_at	PHD finger protei	PHF7	6.89	6.93	6.70
211849_s_at	RNA guanylyltrans	RNGTT	5.98	5.95	5.97
213935_at	abhydrolase doma	ABHD5	6.35	6.82	6.66
200773_x_at	prothymosin, alph	PTMA	13.77	13.87	13.87
201084_s_at	BCL2-associated t	BCLAF1	11.31	11.49	11.56
207656_s_at	acyl-CoA oxidase 1	ACOX1	5.78	5.91	5.73
203723_at	inositol 1,4,5-trisp	ITPKB	5.67	5.80	5.74
211617_at	aldolase A, fructo:	ALDOAP2	3.84	3.64	3.43
216755_at	oxysterol binding	OSBPL10	6.23	6.46	6.39
221121_at	chromosome X op	CXorf48	5.55	5.66	5.28
204524_at	3-phosphoinositid	PDPK1	9.11	8.98	9.21
202900_s_at	nucleoporin 88kD	NUP88	9.34	9.35	9.47

207134_x_at	tryptase beta 2 (g	TPSB2	6.32	6.33	6.19
208401_s_at	glucagon-like pep	GLP1R	5.29	5.45	5.35
220658_s_at	aryl hydrocarbon	ARNTL2	7.14	7.14	7.13
221660_at	myosin, light chai	MYL10	8.34	8.18	8.34
203758_at	cathepsin O	CTSO	5.36	6.03	5.66
204801_s_at	dehydrogenase/re	DHRS12	7.48	7.25	7.47
208725_at	eukaryotic transla	EIF2S2	8.67	8.57	8.71
201195_s_at	solute carrier fam	SLC7A5	11.53	11.15	11.46
221293_s_at	differentially expr	DEF6	7.45	7.21	7.05
208532_x_at	keratin associatec	KRTAP5-8	7.83	7.79	7.69
216468_s_at	zinc finger proteir	ZNF682	3.00	2.96	2.93
219305_x_at	F-box protein 2	FBXO2	6.59	6.53	6.15
209944_at	zinc finger proteir	ZNF410	11.33	11.14	11.40
203316_s_at	small nuclear ribo	SNRPE	12.13	12.24	12.28
216350_s_at	zinc finger proteir	ZNF10	3.89	3.90	3.83
206867_at	glucokinase (hexo	GCKR	5.15	5.27	4.98
215902_at	---	---	4.34	4.49	4.23
216541_x_at	immunoglobulin h	IGHG1 /// IGHM //	3.98	4.04	3.91
220314_at	heat shock transc	HSFX1 /// HSFX2	5.54	5.31	5.00
AFFX-ThrX-5_at	---	---	6.41	6.37	6.04
202780_at	3-oxoacid CoA tra	OXCT1	11.18	11.31	11.36
207963_at	chromosome 6 op	C6orf54	4.59	4.56	4.26
212448_at	neural precursor c	NEDD4L	7.99	7.78	7.73
203752_s_at	jun D proto-oncog	JUND	10.76	10.84	10.96
212961_x_at	chromosome X op	CXorf40B	9.67	9.64	9.67
214006_s_at	gamma-glutamyl c	GGCX	8.36	8.36	8.44
214510_at	G protein-couplec	GPR20	5.55	5.42	5.19
213755_s_at	---	---	7.31	7.35	7.21
217768_at	chromosome 14 c	C14orf166	13.05	13.05	13.06
220064_at	tetratricopeptide	TTC21B	4.65	4.49	4.17
211498_s_at	NK3 homeobox 1	NKX3-1	5.73	5.88	5.66
216392_s_at	SEC23 interacting	SEC23IP	9.03	9.11	9.11
204805_s_at	H1 histone family,	H1FX	10.79	10.52	10.69
206793_at	phenylethanolami	PNMT	7.35	6.79	6.63
213195_at	chromosome 17 c	C17orf108	7.14	6.93	6.92
205101_at	class II, major hist	CIITA	5.31	5.33	4.96
213430_at	RUN and FYVE dor	RUFY3	5.57	5.44	5.66
212506_at	phosphatidylinosi	PICALM	10.81	10.68	10.95
209805_at	PMS2 postmeiotic	PMS2 /// PMS2CL	8.81	8.64	9.01
206635_at	cholinergic recept	CHRNB2	9.00	8.57	8.42
206004_at	transglutaminase	TGM3	5.44	5.29	5.03
216879_at	Hr44 antigen	HR44	4.89	4.83	4.82
219149_x_at	debranching enzy	DBR1	7.94	8.08	7.84
203000_at	stathmin-like 2	STMN2	4.28	4.44	3.79
206970_at	contactin 2 (axona	CNTN2	6.41	6.44	6.24
204931_at	transcription facto	TCF21	5.38	4.84	4.99
208214_at	adrenergic, beta-1	ADRB1	4.13	3.78	3.67

211174_s_at	cholecystokinin A	CCKAR	4.76	4.76	4.50
207704_s_at	growth arrest-spe	GAS7	5.54	5.43	4.92
204937_s_at	zinc finger protein	ZNF274	8.81	8.91	8.99
214981_at	periostin, osteobl	POSTN	3.56	3.43	3.43
209620_s_at	ATP-binding casse	ABCB7	9.79	9.75	9.87
215954_s_at	chromosome 19 c	C19orf29	6.27	6.06	5.98
203154_s_at	p21 protein (Cdc4	PAK4	7.88	7.87	7.51
218886_at	PAK1 interacting p	PAK1IP1	10.08	9.91	10.28
209378_s_at	family with seque	FAM190B	7.16	7.29	7.49
208316_s_at	oculocerebrorena	OCRL	6.86	6.71	6.81
204380_s_at	fibroblast growth	FGFR3	3.68	3.83	3.76
209838_at	COP9 constitutive	COPS2	8.09	8.29	8.33
220574_at	sema domain, tra	SEMA6D	5.51	5.47	5.51
202450_s_at	cathepsin K	CTSK	5.16	5.32	5.27
222324_at	---	---	4.13	4.37	4.43
209862_s_at	centrosomal prot	CEP57	9.28	9.25	9.39
214401_at	paired box 1	PAX1	6.30	6.16	5.87
221006_s_at	sorting nexin fami	SNX27	7.08	6.86	6.76
46323_at	calcium activated	CANT1	8.67	8.76	8.61
204307_at	tectonin beta-pro	TECPR2	6.50	6.80	6.45
207228_at	protein kinase, cA	PRKACG	5.93	5.84	6.03
207077_at	chymotrypsin-like	CELA2B	4.67	4.52	4.46
203483_at	sema domain, imr	SEMA4G	5.13	4.97	4.84
204418_x_at	glutathione S-tran	GSTM2	7.43	7.62	7.34
203877_at	---	---	5.46	5.40	5.33
220103_s_at	mitochondrial rib	MRPS18C	4.68	4.82	4.65
207336_at	SRY (sex determin	SOX5	4.14	4.09	4.18
213498_at	cAMP responsive	CREB3L1	5.83	5.87	5.86
211871_x_at	guanine nucleotid	GNB5	7.90	7.57	7.61
216939_s_at	5-hydroxytryptam	HTR4	3.95	3.89	3.58
215334_at	EFR3 homolog B (!	EFR3B	5.33	5.72	5.13
207033_at	gastric intrinsic fa	GIF	4.06	4.08	3.93
208486_at	dopamine recepto	DRD5	5.57	5.51	5.25
216271_x_at	synapse defective	SYDE1	6.52	6.63	6.15
220049_s_at	programmed cell	PDCD1LG2	5.10	5.17	5.07
211880_x_at	protocadherin gar	PCDHGA1	4.46	4.55	4.36
216532_x_at	---	---	10.03	10.08	10.19
217024_x_at	signal-regulatory	SIRPA	5.63	5.44	5.40
219904_at	zinc finger and SC	ZSCAN5A	4.99	5.01	4.96
220258_s_at	WD repeat contai	WRAP53	7.56	7.63	7.32
203668_at	mannosidase, alpl	MAN2C1	6.49	6.45	6.08
220155_s_at	bromodomain cor	BRD9	9.76	9.80	9.79
220514_at	---	---	6.59	6.58	6.14
213395_at	megalencephalic l	MLC1	5.57	5.31	5.30
207748_at	---	---	5.65	5.89	5.92
209785_s_at	phospholipase A2	PLA2G4C	5.49	5.44	5.18
220606_s_at	chromosome 17 c	C17orf48	7.67	7.73	8.02

202606_s_at	tousled-like kinas	TLK1	9.96	10.04	10.08
211447_s_at	phosphodiesteras	PDE4A	5.51	5.75	5.44
215303_at	doublecortin-like	DCLK1	3.54	3.58	3.51
203460_s_at	presenilin 1	PSEN1	8.90	8.89	9.25
206570_s_at	pregnancy specifi	PSG11	4.30	4.62	4.24
204468_s_at	tyrosine kinase wi	TIE1	4.23	4.12	3.98
212056_at	KIAA0182	KIAA0182	8.09	8.10	8.25
220627_at	cystatin 8 (cystati	CST8	6.10	5.82	5.87
220885_s_at	centromere prote	CENPJ	5.66	5.65	5.79
205083_at	aldehyde oxidase	AOX1	11.21	11.32	11.34
203728_at	BCL2-antagonist/k	BAK1	7.84	7.83	7.77
215496_at	sterile alpha moti	SAMD4A	5.24	5.33	5.00
220113_x_at	polymerase (RNA)	POLR1B	8.43	8.51	8.58
202572_s_at	discs, large (Drosc	DLGAP4	7.30	7.26	7.27
37953_s_at	amiloride-sensitiv	ACCN2	4.58	4.77	4.38
208817_at	catechol-O-methy	COMT	9.49	9.42	9.57
209935_at	ATPase, Ca++ tran	ATP2C1	8.21	8.28	8.54
203314_at	GTP binding prote	GTPBP6	8.88	8.78	8.76
209061_at	nuclear receptor c	NCOA3	8.44	8.44	8.44
214628_at	nescient helix loo	NHLH1	4.93	5.43	5.36
208585_at	butyrophilin, subf	BTN2A3	5.56	5.35	4.85
204046_at	phospholipase C, I	PLCB2	7.48	7.26	7.11
214138_at	zinc finger protein	ZNF79	6.24	6.05	6.13
207589_at	adrenergic, alpha-	ADRA1B	4.24	4.22	4.03
207452_s_at	contactin 5	CNTN5	3.61	3.68	3.38
221237_s_at	oxysterol binding	OSBP2	7.76	7.48	7.16
210631_at	neurofibromin 1	NF1	4.27	4.18	4.23
201025_at	eukaryotic transla	EIF5B	8.28	8.48	8.84
205071_x_at	X-ray repair comp	XRCC4	8.34	8.54	8.59
214350_at	Syntrophin, beta 2	SNTB2	3.78	3.90	3.65
210116_at	SH2 domain conta	SH2D1A	4.21	4.21	3.97
207377_at	protein phosphata	PPP1R2P9	4.58	4.50	4.38
217746_s_at	programmed cell	PDCD6IP	10.26	10.27	10.31
212191_x_at	ribosomal protein	RPL13	14.47	14.39	14.57
205013_s_at	adenosine A2a re	ADORA2A /// CYTS	6.27	6.21	6.11
218962_s_at	transmembrane p	TMEM168	9.36	9.45	9.64
204903_x_at	ATG4 autophagy r	ATG4B	7.60	7.69	7.74
211892_s_at	prostaglandin I2 (|	PTGIS	4.77	4.57	4.51
206659_at	---	---	4.74	4.82	4.87
201561_s_at	calsyntenin 1	CLSTN1	8.65	8.61	8.77
209096_at	ubiquitin-conjuga	UBE2V2	10.98	11.08	11.08
52837_at	KIAA1644	KIAA1644	8.73	8.79	8.74
205451_at	forkhead box O4	FOXO4	6.26	6.37	6.46
201430_s_at	dihydropyrimidina	DPYSL3	3.65	3.57	3.80
216872_at	Hr44 antigen	HR44	3.41	3.38	3.41
213068_at	dermatopontin	DPT	3.81	3.58	3.49
219120_at	chromosome 2 op	C2orf44	7.25	7.56	7.05

219747_at	chromosome 4 op	C4orf31	4.31	4.37	4.56
212014_x_at	CD44 molecule (Ir	CD44	10.02	9.97	10.08
212227_x_at	eukaryotic transla	EIF1	13.59	13.33	13.56
217433_at	transforming, acic	TACC1	4.86	4.83	5.07
217361_at	---	---	4.31	4.28	4.10
205124_at	LOC729991-MEF2	LOC729991-MEF2I	5.70	5.74	5.49
221223_x_at	cytokine inducible	CISH	7.00	6.88	6.85
214649_s_at	myotubularin rela	MTMR2	9.10	9.04	9.35
208427_s_at	ELAV (embryonic l	ELAVL2	4.51	4.43	4.49
213660_s_at	topoisomerase (D	TOP3B	6.44	6.21	6.27
212894_at	suppressor of var1	SUPV3L1	9.53	9.28	9.53
221383_at	neuromedin U rec	NMUR1	5.81	5.85	5.49
208432_s_at	calcium channel, v	CACNA1E	4.74	4.39	4.79
207144_s_at	Cbp/p300-interac	CITED1	5.03	5.15	4.77
212388_at	ubiquitin specific p	USP24	8.47	8.60	8.62
216219_at	aquaporin 6, kidn	AQP6	6.52	6.40	6.85
218099_at	testis expressed 2	TEX2	9.13	9.09	9.25
207206_s_at	arachidonate 12-l	ALOX12	4.71	4.72	4.35
211717_at	ankyrin repeat do	ANKRD40	7.56	8.07	7.78
211840_s_at	phosphodiesteras	PDE4D	3.83	3.61	3.75
222155_s_at	G protein-couplec	GPR172A	7.11	7.36	7.37
208094_s_at	coiled-coil domair	CCDC130	8.21	8.21	8.12
205465_x_at	heparan sulfate (g	HS3ST1	6.00	6.36	6.13
206588_at	deleted in azoosp	DAZL	3.43	3.17	3.55
219614_s_at	solute carrier fam	SLC6A20	3.74	3.65	3.71
216130_at	---	---	5.36	4.90	4.97
201164_s_at	pumilio homolog 1	PUM1	10.00	10.02	10.05
214826_at	Phosphodiesteras	PDE12	3.98	3.94	3.98
219128_at	chromosome 2 op	C2orf42	7.31	7.47	7.48
205757_at	ectonucleoside tri	ENTPD5	5.50	5.81	5.63
209698_at	coiled-coil alpha-h	CCHCR1	8.09	8.04	8.03
211710_x_at	ribosomal protein	RPL4	14.75	14.71	14.80
217117_x_at	mucin 3A, cell sur	MUC3A	7.68	7.48	7.26
219039_at	sema domain, imr	SEMA4C	8.65	8.42	7.93
213746_s_at	filamin A, alpha	FLNA	8.63	8.41	8.60
221321_s_at	Kv channel interac	KCNIP2	5.39	4.86	5.12
216018_at	ring finger proteir	RNF5	4.62	4.71	5.02
221448_s_at	testis expressed 1	TEX15	4.37	4.65	4.59
207320_x_at	staufen, RNA bind	STAU1	11.23	11.28	11.30
203768_s_at	steroid sulfatase (	STS	5.20	4.99	4.68
216315_x_at	transmembrane p	TMEM189 /// TME	4.63	4.56	4.56
200094_s_at	eukaryotic transla	EEF2	13.33	13.28	13.38
221305_s_at	UDP glucuronosyl	UGT1A8 /// UGT1A	3.83	3.69	3.72
200025_s_at	ribosomal protein	RPL27	14.85	14.88	14.92
215551_at	---	---	5.22	5.27	4.82
200902_at	15 kDa selenoprot	15-Sep	10.63	10.68	10.91
220536_at	chromosome 14 o	C14orf115	6.95	6.95	6.90

214779_s_at	small G protein si§	SGSM3	7.51	7.38	7.48
213153_at	SET domain conta	SETD1B	10.04	9.95	9.88
203412_at	leucine-zipper-like	LZTR1	8.22	8.24	8.18
219823_at	lin-28 homolog A	LIN28A	3.72	3.91	3.63
215854_at	---	---	5.33	5.46	5.19
205436_s_at	H2A histone famil	H2AFX	11.44	11.51	11.41
209166_s_at	mannosidase, alpl	MAN2B1	8.63	8.50	8.60
218922_s_at	LAG1 homolog, ce	LASS4	6.77	6.67	6.27
221864_at	ORAI calcium rele	ORAI3	8.84	8.79	8.79
203779_s_at	myelin protein zer	MPZL2	3.37	3.27	3.42
214717_at	hypothetical prote	DKFZp434H1419	6.63	6.75	6.62
216042_at	tumor necrosis fa	TNFRSF25	6.36	6.44	6.22
213781_at	leucine rich repea	LRRC68	5.26	5.00	4.65
222122_s_at	THO complex 2	THOC2	9.73	9.83	10.01
200065_s_at	ADP-ribosylation f	ARF1	11.96	11.97	11.98
209887_at	SMAD family men	SMAD6	4.73	4.89	4.63
220355_s_at	polybromo 1	PBRM1	9.74	9.63	9.49
205524_s_at	hyaluronan and pr	HAPLN1	5.65	5.93	5.46
220829_s_at	UDP-Gal:betaGlcN	B3GALT1	4.84	4.78	4.58
208674_x_at	dolichyl-diphosph	DDOST	10.39	10.29	10.54
207364_at	testis expressed 2	TEX28	5.98	6.11	5.45
203497_at	mediator complex	MED1	10.07	10.06	10.16
209250_at	degenerative sper	DEGS1	10.70	10.85	10.87
216367_at	collagen, type IV,	COL4A3	4.25	4.04	3.83
207892_at	CD40 ligand	CD40LG	5.86	5.69	5.40
217224_at	---	---	5.80	5.82	5.63
203261_at	dynactin 6	DCTN6	10.97	10.94	10.97
220293_at	cation channel, sp	CATSPERB	3.81	4.11	3.95
209490_s_at	palmitoyl-protein	PPT2	8.54	8.25	8.36
210500_at	---	---	4.21	4.27	4.08
208340_at	---	---	3.70	3.47	3.58
203979_at	cytochrome P450,	CYP27A1	7.45	7.05	7.44
211914_x_at	neurofibromin 1	NF1	5.89	6.05	5.88
210204_s_at	CCR4-NOT transcr	CNOT4	6.21	5.93	5.86
222063_s_at	CDP-diacylglycero	CDS1	4.90	4.88	4.93
221341_s_at	olfactory receptor	OR1D4 /// OR1D5	5.17	5.09	5.14
203320_at	SH2B adaptor pro	SH2B3	8.40	8.23	8.48
201814_at	TBC1 domain fam	TBC1D5	10.26	10.21	10.24
217727_x_at	vacuolar protein s	VPS35	11.65	11.75	11.66
220136_s_at	crystallin, beta A2	CRYBA2	4.61	4.30	4.88
211391_s_at	POZ (BTB) and AT	PATZ1	4.54	4.60	4.97
218678_at	nestin	NES	4.93	4.99	4.67
216330_s_at	POU class 6 home	POU6F1	5.57	5.37	5.36
212638_s_at	WW domain conta	WWP1	8.87	9.05	9.16
222185_at	PBX/knotted 1 ho	PKNOX2	5.98	6.19	5.56
219482_at	SET domain conta	SETD4	7.17	7.30	7.43
200810_s_at	cold inducible RN/	CIRBP	10.79	10.57	10.93

37802_r_at	family with seque	FAM63B	4.04	4.23	4.32
219776_s_at	---	---	4.95	5.24	4.98
217895_at	Pentatricopeptide	PTCD3	10.83	10.87	10.87
212709_at	nucleoporin 160kl	NUP160	8.67	8.79	9.04
200610_s_at	nucleolin	NCL	13.17	13.29	13.41
214186_s_at	HLA complex grou	HCG26	7.28	6.94	7.02
201288_at	Rho GDP dissociat	ARHGDIB	7.96	8.14	8.12
AFFX-r2-Ec-bioD	---	---	13.26	13.21	13.26
205858_at	nerve growth fact	NGFR	4.05	3.92	4.28
214986_x_at	peptidylprolyl isor	PPIL2	8.05	8.06	7.98
214609_at	paired-like homec	PHOX2A	6.94	6.77	6.65
218359_at	neurensin 2	NRSN2	6.08	6.18	6.13
202956_at	ADP-ribosylation f	ARFGEF1	9.29	9.27	9.40
204684_at	neuronal pentraxi	NPTX1	8.36	8.49	8.27
213601_at	slit homolog 1 (Dr	SLIT1	6.54	6.51	6.24
217358_at	DnaJ (Hsp40) hom	DNAJC16	5.11	5.27	4.88
214450_at	cathepsin W	CTSW	6.75	6.82	6.53
206994_at	cystatin S	CST4	6.31	6.38	6.26
212704_at	zinc finger, CCHC (	ZCCHC11	8.47	8.67	8.67
211978_x_at	peptidylprolyl isor	PPIA	14.42	14.42	14.43
210159_s_at	tripartite motif-co	TRIM31	6.10	5.87	5.85
219090_at	solute carrier fam	SLC24A3	4.42	4.53	3.95
221104_s_at	nipsnap homolog	NIPSNAP3B	3.40	3.43	3.24
208194_s_at	signal transducing	STAM2	5.54	5.62	5.55
216673_at	testis-specific trar	TTTY1 /// TTTY1B	4.03	4.07	3.95
207479_at	---	---	3.42	3.39	3.27
204996_s_at	cyclin-dependent	CDK5R1	5.55	5.49	5.66
201935_s_at	eukaryotic transla	EIF4G3	9.36	9.30	9.41
222222_s_at	homer homolog 3	HOMER3	7.59	7.79	7.59
216369_at	---	---	3.95	4.18	3.89
218687_s_at	mucin 13, cell surf	MUC13	4.85	4.52	4.93
217019_at	ribosomal protein	RPS4X /// RPS4XP6	6.81	6.78	6.55
206122_at	SRY (sex determin	SOX15	6.70	6.66	6.44
44120_at	aarF domain conta	ADCK2	8.70	8.65	8.66
209109_s_at	tetraspanin 6	TSPAN6	8.12	8.18	8.10
215674_at	KIAA1659 protein	KIAA1659	2.99	3.20	3.12
218491_s_at	thymocyte nuclea	THYN1	9.13	9.21	9.25
215232_at	Rho-type GTPase-	RICH2	5.98	5.45	5.90
220148_at	aldehyde dehydro	ALDH8A1	5.21	4.86	4.91
217526_at	nuclear factor of a	NFATC2IP	8.91	8.93	9.09
213070_at	phosphoinositide-	PIK3C2A	10.87	11.21	11.25
209896_s_at	protein tyrosine p	PTPN11	9.77	9.80	9.78
220298_s_at	spermatogenesis a	SPATA6	4.07	4.11	4.07
216484_x_at	Hepatoma-derived	HDGF	10.59	10.64	10.59
205835_s_at	YTH domain conta	YTHDC2	5.33	5.24	5.74
213938_at	ELKS/RAB6-intera	ERC2	3.75	3.69	3.68
208800_at	signal recognition	SRP72	10.22	10.32	10.49

218274_s_at	ankyrin repeat an	ANKZF1	8.37	8.31	8.20
200980_s_at	pyruvate dehydro	PDHA1	11.32	11.28	11.31
220843_s_at	DDB1 and CUL4 a	DCAF13	6.22	6.40	6.10
212362_at	ATPase, Ca++ tran	ATP2A2	5.96	6.22	6.10
218838_s_at	tetratricopeptide	TTC31	7.62	7.72	7.86
219347_at	nudix (nucleoside	NUDT15	10.57	10.54	10.64
217362_x_at	major histocompa	HLA-DRB6	6.25	6.62	6.59
216563_at	Ankyrin repeat do	ANKRD12	6.02	6.58	6.39
221199_at	GDNF family rece	GFRA4	5.37	5.23	4.93
214113_s_at	RNA binding moti	RBM8A	9.87	9.88	10.04
206158_s_at	CCHC-type zinc fir	CNBP	12.96	12.94	13.10
200075_s_at	guanylate kinase	GUK1	10.15	10.08	10.29
220689_at	---	---	5.55	5.95	5.33
218172_s_at	Der1-like domain	DERL1	8.74	8.66	8.98
212024_x_at	flightless I homolo	FLII	9.31	9.25	9.11
203569_s_at	oral-facial-digital	OFD1	9.28	9.32	9.33
219834_at	amyotrophic later	ALS2CR8	4.62	4.61	4.39
207859_s_at	cholinergic recept	CHRNB3	4.07	3.98	4.00
219846_at	gon-4-like (C. eleg	GON4L	5.66	5.59	5.13
219519_s_at	sialic acid binding	SIGLEC1	5.07	5.18	4.97
215298_at	---	---	3.68	3.48	3.66
209681_at	solute carrier fam	SLC19A2	9.69	9.67	9.92
211908_x_at	Immunoglobulin k	IGKV3-20	6.22	5.91	5.59
207456_at	hepatocyte nucle	HNF4G	3.28	3.44	3.47
203853_s_at	GRB2-associated	GAB2	7.59	7.22	7.42
220156_at	EF-hand calcium b	EFCAB1	4.70	5.18	4.48
219293_s_at	Obg-like ATPase 1	OLA1	12.39	12.51	12.68
209905_at	homeobox A9	HOXA9	5.85	6.04	6.08
219935_at	ADAM metallope	ADAMTS5	3.49	3.48	3.54
212725_s_at	taurine upregulat	TUG1	9.67	9.70	9.68
218000_s_at	pleckstrin homolo	PHLDA1	7.11	6.96	6.75
219219_at	transmembrane p	TMEM160	9.25	9.25	9.32
210698_at	---	---	3.40	3.31	3.33
217632_at	guanine nucleotid	GNL3L	5.14	5.20	5.08
206091_at	matrilin 3	MATN3	3.14	3.18	3.17
214525_x_at	mutL homolog 3 (	MLH3	6.26	6.34	5.88
219332_at	MICAL-like 2	MICALL2	8.52	8.64	8.52
205015_s_at	transforming grov	TGFA	5.80	6.00	5.54
203012_x_at	ribosomal protein	RPL23A	15.14	15.14	15.17
202161_at	protein kinase N1	PKN1	7.04	6.62	6.84
208946_s_at	beclin 1, autopha	BECN1	9.81	9.91	10.15
217102_at	Similar to maligna	LOC100287076	5.22	5.48	5.23
210512_s_at	vascular endothel	VEGFA	10.89	10.70	11.05
202593_s_at	glycerophosphodi	GDE1	8.71	8.58	8.71
207362_at	solute carrier fam	SLC30A4	3.93	3.95	4.31
201870_at	translocase of out	TOMM34	9.62	9.67	9.59
206200_s_at	annexin A11	ANXA11	11.18	11.15	11.09

213547_at	cullin-associated a	CAND2	5.91	5.97	5.24
209126_x_at	keratin 6B	KRT6B	7.33	7.47	7.04
217945_at	BTB (POZ) domain	BTBD1	10.55	10.78	10.71
206137_at	regulating synapti	RIMS2	6.86	6.79	6.60
208130_s_at	thromboxane A sy	TBXAS1	6.39	6.56	6.41
207388_s_at	prostaglandin E sy	PTGES	7.06	6.90	6.95
214362_at	regulator of G-pro	RGS12	5.96	5.21	5.85
64432_at	chromosome 12 c	C12orf47	8.23	8.15	8.39
212480_at	cytospin A	CYTSA	9.64	9.50	9.69
209133_s_at	COMM domain co	COMMD4	6.46	6.42	6.29
37996_s_at	dystrophia myoto	DMPK	6.08	5.93	5.94
219075_at	Yip1 domain famil	YIPF2	6.95	6.78	6.67
201628_s_at	Ras-related GTP b	RRAGA	9.42	9.50	9.51
221555_x_at	CDC14 cell divisio	CDC14B	9.45	9.48	9.40
218798_at	KRI1 homolog (S.	KRI1	8.88	8.60	8.68
205713_s_at	cartilage oligomer	COMP	5.38	4.91	5.04
216536_at	olfactory receptor	OR7E19P	4.17	4.04	4.10
204868_at	immature colon c	ICT1	10.12	10.20	10.22
217771_at	golgi membrane p	GOLM1	9.69	9.68	9.87
206203_at	recoverin	RCVRN	4.36	4.34	4.04
201043_s_at	acidic (leucine-ricl	ANP32A	10.07	9.94	9.98
212587_s_at	protein tyrosine p	PTPRC	4.22	4.17	4.05
209600_s_at	acyl-CoA oxidase	ACOX1	7.98	8.04	7.99
214961_at	microtubule assoc	MTUS2	3.44	3.31	3.46
210251_s_at	RUN and FYVE dor	RUFY3	7.25	7.25	7.43
204448_s_at	phosducin-like	PDCL	7.58	7.67	7.52
209532_at	phospholipase A2	PLAA	6.06	6.16	5.96
221035_s_at	testis expressed 1	TEX14	4.85	4.81	4.05
219104_at	ring finger proteir	RNF141	7.77	7.69	7.81
32625_at	natriuretic peptid	NPR1	6.32	6.24	5.98
210688_s_at	carnitine palmitoy	CPT1A	5.96	5.73	5.73
202753_at	proteasome (pros	PSMD6	11.53	11.72	11.66
217212_s_at	interleukin 9 rece	IL9R	5.80	6.16	5.86
207418_s_at	D-aspartate oxida	DDO	6.58	6.17	6.49
211082_x_at	MAP/microtubule	MARK2	5.30	5.02	5.21
212133_at	Cytoplasmic FMR	CYFIP1	8.65	8.74	8.75
220123_at	solute carrier fam	SLC35F5	5.09	4.88	4.89
205988_at	CD84 molecule	CD84	3.93	4.15	3.54
40562_at	guanine nucleotid	GNA11	9.83	9.90	9.91
201837_s_at	suppressor of Ty 7	SUPT7L	9.25	9.23	9.26
203502_at	2,3-bisphosphogly	BPGM	5.72	6.16	6.11
207486_x_at	chimerin (chimaer	CHN2	5.37	5.24	5.19
206981_at	sodium channel, v	SCN4A	4.28	4.46	4.40
214377_s_at	chymotrypsin-like	CTRL	6.10	5.83	5.52
200053_at	sperm associated	SPAG7	10.02	9.90	9.78
209881_s_at	linker for activatic	LAT /// SPNS1	6.25	6.56	6.45
202693_s_at	serine/threonine	STK17A	10.29	10.29	10.49

202858_at	canopy 2 homolog	CNPY2	12.71	12.61	12.58
201591_s_at	nischarin	NISCH	8.65	8.84	8.81
210297_s_at	microseminoprote	MSMB	3.53	3.35	3.50
222343_at	BCL2-like 11 (apo	BCL2L11	5.39	5.21	5.43
221976_s_at	Hepatoma-derive	HDGFRP3	5.68	5.36	5.60
221407_at	gap junction prot	GJD2	6.26	6.31	6.02
212431_at	HMG box domain	HMGXB3	9.05	8.88	8.72
212652_s_at	sorting nexin 4	SNX4	9.14	9.28	9.45
214252_s_at	ceroid-lipofuscino	CLN5	7.52	7.77	7.67
222012_at	DDB1 and CUL4 a	DCAF15	6.73	6.47	6.23
210624_s_at	ilvB (bacterial ace	ILVBL	8.92	8.70	8.76
215801_at	---	---	3.30	3.18	3.16
212700_x_at	similar to hCG200	LOC100291232 ///	6.82	6.51	6.79
217691_x_at	solute carrier fam	SLC16A3	8.46	8.55	8.45
207484_s_at	euchromatic histo	EHMT2	7.39	7.41	7.19
209022_at	stromal antigen 2	STAG2	10.97	11.02	11.16
201536_at	dual specificity ph	DUSP3	8.94	8.97	8.84
207687_at	inhibin, beta C	INHBC	4.99	4.86	4.58
213110_s_at	collagen, type IV,	COL4A5	9.34	9.62	9.68
216031_x_at	hematological and	HN1L	7.32	7.54	7.44
210051_at	Rap guanine nucle	RAPGEF3	6.17	6.40	6.10
203472_s_at	solute carrier orga	SLCO2B1	5.33	5.18	4.86
220375_s_at	---	---	7.46	7.46	7.32
217169_at	immunoglobulin h	IGHA1 /// IGHG1 /	4.92	4.66	4.68
210873_x_at	apolipoprotein B r	APOBEC3A	4.33	4.09	4.02
209353_s_at	---	---	3.92	4.27	4.14
217030_at	---	---	4.90	4.88	4.58
207929_at	gastrin-releasing p	GRPR	5.95	5.95	5.83
213185_at	KIAA0556	KIAA0556	7.45	7.45	7.39
208234_x_at	fibroblast growth	FGFR2	6.82	6.92	6.58
205294_at	BAI1-associated p	BAIAP2	6.83	6.85	6.73
221556_at	CDC14 cell divisio	CDC14B	9.20	9.35	9.42
206745_at	homeobox C11	HOXC11	7.37	7.44	7.39
200853_at	H2A histone famil	H2AFZ	13.14	13.19	13.16
216852_x_at	Immunoglobulin l	IGL@	7.69	7.44	7.05
219779_at	zinc finger homeo	ZFHX4	4.38	4.62	4.23
206798_x_at	deleted in lung an	DLEC1	5.45	5.38	5.19
205833_s_at	prostate androgen	PART1	5.54	5.80	5.66
209189_at	FBJ murine osteos	FOS	5.10	4.83	4.52
205507_at	Rho guanine nucle	ARHGEF15	5.67	5.83	5.71
214462_at	suppressor of cyto	SOCS6	5.47	5.38	5.17
213323_s_at	zinc finger CCCH-t	ZC3H7B	7.78	7.64	7.49
201863_at	family with seque	FAM32A	9.37	9.40	9.23
222383_s_at	arachidonate lipo	ALOXE3	4.14	3.95	3.62
213816_s_at	met proto-oncoge	MET	6.27	6.22	6.27
211734_s_at	Fc fragment of IgE	FCER1A	3.25	3.11	3.15
213486_at	COPG2 imprinted	COPG2IT1	5.26	5.09	5.25

211531_x_at	proline-rich prote	PRB1	8.43	8.21	7.79
207255_at	leptin receptor	LEPR	4.46	4.50	4.35
218684_at	leucine rich repea	LRRC8D	11.30	10.94	11.33
220826_at	t-complex 10 (mo	TCP10L	5.50	5.29	5.15
205722_s_at	GDNF family recep	GFRA2	5.42	4.93	5.09
218566_s_at	cysteine and histic	CHORDC1	9.64	9.73	10.08
219267_at	glycolipid transfer	GLTP	8.39	8.49	8.47
216783_at	---	---	3.98	4.26	3.90
209892_at	fucosyltransferase	FUT4	5.99	5.78	5.89
204256_at	ELOVL family men	ELOVL6	10.62	10.79	10.91
205327_s_at	activin A receptor	ACVR2A	7.28	7.18	7.49
209683_at	family with seque	FAM49A	4.45	4.31	4.42
217238_s_at	aldolase B, fructos	ALDOB	3.55	3.47	3.62
217430_x_at	collagen, type I, al	COL1A1	7.75	7.60	7.44
209716_at	colony stimulating	CSF1	9.25	9.06	9.05
206517_at	cadherin 16, KSP-c	CDH16	5.62	5.79	5.43
218244_at	nucleolar protein	NOL8	9.82	10.01	10.03
218065_s_at	TMEM9 domain fa	TMEM9B	9.58	9.66	9.63
213993_at	spondin 1, extrace	SPON1	3.62	3.69	3.55
210160_at	platelet-activating	PAFAH1B2	5.99	6.13	6.23
216747_at	amyloid beta (A4)	APBB2	4.12	3.95	3.86
201912_s_at	G1 to S phase trar	GSPT1	10.23	10.23	10.44
203087_s_at	kinesin heavy chai	KIF2A	10.33	10.38	10.40
220202_s_at	ring finger and CC	RC3H2	8.68	8.66	8.71
206690_at	amiloride-sensitiv	ACCN1	4.65	4.56	4.48
211120_x_at	estrogen receptor	ESR2	4.98	5.17	4.52
204455_at	dystonin	DST	7.17	7.55	7.63
211191_at	CD84 molecule	CD84	6.11	6.05	5.79
205508_at	sodium channel, v	SCN1B	7.25	6.72	7.14
210472_at	Metallothionein 1	MT1G	4.15	4.15	4.29
217633_at	URB1 ribosome bi	URB1	5.78	5.85	5.76
217621_at	solute carrier fam	SLC6A2	3.57	3.14	2.90
207690_at	ALX homeobox 3	ALX3	6.70	6.53	6.68
220415_at	TNNI3 interacting	TNNI3K	3.99	3.89	3.57
204853_at	origin recognition	ORC2L	8.79	8.81	8.86
218993_at	RNA methyltransf	RNMTL1	8.19	8.15	8.10
222057_at	nucleolar protein	NOL12	6.81	7.22	6.84
209427_at	smoothelin	SMTN	6.04	6.48	6.28
206056_x_at	sialophorin	SPN	7.11	7.17	7.06
215861_at	---	---	5.18	4.84	5.05
202750_s_at	tuftelin interactin	TFIP11	8.31	8.34	8.30
209314_s_at	HBS1-like (S. cerev	HBS1L	8.49	8.66	9.06
202687_s_at	tumor necrosis fac	TNFSF10	3.68	3.20	3.35
203763_at	dynein, cytoplasm	DYNC2LI1	6.54	6.79	6.87
207271_x_at	sperm associated	SPAG11B	3.88	3.79	3.85
221100_at	chromosome 6 op	C6orf15	7.15	7.12	7.28
202117_at	Rho GTPase activa	ARHGAP1	9.65	9.66	9.61

205625_s_at	calbindin 1, 28kDa	CALB1	3.81	3.46	3.41
220991_s_at	ring finger protein	RNF32	3.61	3.80	3.54
214588_s_at	microfibrillar-asso	MFAP3	6.19	6.26	6.06
220736_at	solute carrier fam	SLC19A3	3.41	3.46	3.30
218926_at	myoneurin	MYNN	8.52	8.50	8.77
221465_at	olfactory receptor	OR6A2	4.27	4.12	4.09
207968_s_at	myocyte enhance	MEF2C	4.19	4.15	4.29
40837_at	transducin-like en	TLE2	8.55	8.45	8.59
214396_s_at	methyl-CpG bindi	MBD2	4.03	4.57	4.32
201405_s_at	COP9 constitutive	COPS6	11.04	11.04	11.05
215042_at	bone morphogen	BMP6	4.92	4.87	4.75
205236_x_at	superoxide dismu	SOD3	5.63	5.70	5.59
215970_at	actin, gamma-like	LOC100130331	4.01	4.04	4.06
216662_at	myosin VIIB	MYO7B	6.26	5.96	5.56
216435_at	Mitogen-activated	MAP2K5	3.07	3.06	3.17
220450_at	---	---	4.48	4.50	4.42
216908_x_at	RNA polymerase I	RRN3P1	4.70	4.99	4.98
205786_s_at	integrin, alpha M	ITGAM	4.16	3.85	3.90
210141_s_at	inhibin, alpha	INHA	5.52	5.45	5.68
211778_s_at	ovo-like 2 (Drosop	OVOL2	3.97	3.88	3.76
215712_s_at	insulin-like growth	IGFALS	6.21	6.02	6.20
217096_at	piccolo (presynap	PCLO	4.48	3.79	4.05
221682_s_at	protocadherin gar	PCDHGB6	4.00	3.83	3.69
213016_at	bobby sox homolo	BBX	9.89	10.07	10.02
219001_s_at	DDB1 and CUL4 as	DCAF10	8.06	7.98	8.17
208052_x_at	carcinoembryonic	CEACAM3	7.15	7.10	6.92
210006_at	abhydrolase doma	ABHD14A	7.50	7.51	7.56
218538_s_at	MRS2 magnesium	MRS2	8.42	8.56	8.64
206148_at	interleukin 3 rece	IL3RA	6.91	6.69	6.71
211350_s_at	chromosome 6 op	C6orf54	5.71	5.74	5.44
211487_x_at	ribosomal protein	RPS17	14.95	14.94	15.00
219515_at	PR domain contai	PRDM10	7.77	7.93	7.83
207770_x_at	chorionic somato	CSH2	6.63	6.40	6.35
214031_s_at	Keratin 7	KRT7	5.54	5.83	5.73
202036_s_at	secreted frizzled-r	SFRP1	4.32	3.97	4.22
215732_s_at	deltex homolog 2	DTX2	7.17	7.29	7.00
215816_at	glucuronidase, be	GUSB /// LOC1002	5.75	5.95	5.82
217263_x_at	runt-related trans	RUNX1	5.29	5.03	5.03
210060_at	phosphodiesteras	PDE6G	6.96	6.82	6.16
205715_at	bone marrow stro	BST1	4.75	4.81	4.18
205566_at	abhydrolase doma	ABHD2	7.83	7.78	7.86
218206_x_at	SCAN domain con	SCAND1	9.77	9.60	9.54
207455_at	purinergic recepto	P2RY1	3.47	3.56	3.37
208282_x_at	deleted in azoosp	DAZ1 /// DAZ2 ///	3.31	3.23	3.06
207337_at	cancer/testis anti	CTAG2	3.99	3.68	3.69
208412_s_at	retinoic acid recep	RARB	5.70	5.67	5.50
211461_at	chondroitin sulfat	CSPG4PY1 /// CSP	5.78	5.75	5.40

211063_s_at	NCK adaptor prot	NCK1	9.84	9.92	10.33
211472_at	Plexin B2	PLXNB2	5.31	5.03	5.25
215772_x_at	succinate-CoA liga	SUCLG2	10.45	10.45	10.61
222250_s_at	integrator comple	INTS7	9.11	8.88	9.24
206915_at	NK2 homeobox 2	NKX2-2	3.57	3.47	3.42
222378_at	---	---	3.92	4.15	3.82
209499_x_at	TNFSF12-TNFSF13	TNFSF12-TNFSF13	6.55	6.62	6.46
220670_at	---	---	3.53	3.54	3.17
217287_s_at	transient receptor	TRPC6	4.15	4.32	4.57
208352_x_at	ankyrin 1, erythro	ANK1	6.67	6.59	6.39
221021_s_at	catenin, beta like	CTNNBL1	8.41	8.57	8.60
210389_x_at	tubulin, delta 1	TUBD1	7.41	7.81	7.61
212934_at	UBX domain prote	UBXN2B	8.88	8.92	9.00
211654_x_at	major histocompa	HLA-DQB1	6.82	6.91	6.65
213437_at	RUN and FYVE do	RUFY3	7.16	7.24	7.16
201448_at	TIA1 cytotoxic gra	TIA1	9.30	9.54	9.52
213628_at	chloride channel (	CLCC1	8.33	8.37	8.44
210391_at	nuclear receptor s	NR6A1	5.32	5.64	5.28
219483_s_at	porcupine homolo	PORCN	7.26	7.59	7.43
201718_s_at	erythrocyte meml	EPB41L2	7.12	7.23	7.32
207079_s_at	mediator complex	MED6	8.59	8.85	8.86
209389_x_at	diazepam binding	DBI	12.13	12.20	12.13
220284_at	dickkopf-like 1 (sc	DKKL1	6.27	5.99	5.70
210972_x_at	T cell receptor alp	TRA@ /// TRAC ///	5.96	6.48	6.24
218448_at	chromosome 20 c	C20orf11	8.74	8.81	8.77
200072_s_at	heterogeneous nu	HNRNPM	11.92	11.98	11.98
213468_at	excision repair cro	ERCC2	7.56	7.59	7.61
211442_x_at	cytochrome P450,	CYP3A43	3.58	3.80	3.77
216489_at	transient receptor	TRPM3	4.98	4.83	4.51
220184_at	Nanog homeobox	NANOG	3.74	4.12	3.62
201226_at	NADH dehydroger	NDUFB8 /// SEC31	11.12	11.08	11.08
204636_at	collagen, type XVI	COL17A1	6.43	6.79	6.40
211901_s_at	phosphodiesteras	PDE4A	6.92	6.73	6.64
217171_at	sphingomyelin ph	SMPD1	6.73	6.65	6.34
216207_x_at	Immunoglobulin k	IGKV1D-13	6.38	6.41	6.23
205683_x_at	tryptase alpha/be	TPSAB1	5.88	6.03	5.63
202202_s_at	laminin, alpha 4	LAMA4	3.82	3.52	3.66
218619_s_at	suppressor of vari	SUV39H1	7.96	7.81	7.56
207448_at	protein O-fucosylt	POFUT2	5.32	5.17	4.89
210804_x_at	solute carrier fam	SLC8A1	5.10	5.03	4.86
221150_at	matrix extracellula	MEPE	5.77	6.01	6.00
212653_s_at	EH domain bindin	EHBP1	9.48	9.57	9.81
205577_at	phosphorylase, gl	PYGM	5.22	5.20	5.34
211607_x_at	epidermal growth	EGFR	6.71	6.55	6.58
57516_at	zinc finger protein	ZNF764	6.89	7.24	6.93
202870_s_at	2',5'-oligoadenyla	OAS1	12.17	12.31	12.22
215617_at	spermatogenesis a	SPATS2L	5.42	5.76	5.71

218337_at	family with seque	FAM160B2	8.25	8.29	8.22
219611_s_at	coiled-coil domair	CCDC21	8.00	8.17	7.90
216332_at	POU class 6 home	POU6F1	7.24	7.01	6.93
219720_s_at	chromosome 14 c	C14orf118	6.22	6.23	6.14
210677_at	sterol O-acyltrans	SOAT2	5.84	5.89	5.42
215484_at	---	---	6.32	6.43	6.20
220348_at	kelch-like 29 (Dros	KLHL29	5.62	5.77	5.63
207007_at	nuclear receptor s	NR1I3	5.21	5.07	4.86
200031_s_at	ribosomal protein	RPS11	14.79	14.76	14.79
218750_at	TATA box binding	TAF1D	9.57	9.57	9.63
212126_at	chromobox homo	CBX5	8.76	8.84	8.88
202111_at	solute carrier fam	SLC4A2	7.32	7.09	7.10
209964_s_at	ataxin 7	ATXN7	5.97	5.84	5.53
207520_at	TROVE domain fai	TROVE2	4.45	4.27	4.42
221796_at	neurotrophic tyro	NTRK2	3.40	3.49	3.43
212932_at	RAB3 GTPase acti	RAB3GAP1	8.19	8.23	8.28
208172_s_at	potassium voltage	KCNB2	5.64	5.71	5.28
209279_s_at	NAD(P) dependen	NSDHL	7.81	8.04	7.88
220463_at	transient recepto	TRPM3	5.18	5.08	4.91
209496_at	retinoic acid recep	RARRES2	4.93	5.09	4.85
212161_at	adaptor-related p	AP2A2	6.63	6.49	6.37
206092_x_at	regulator of telom	RTEL1	7.63	7.51	7.46
218091_at	ArfGAP with FG re	AGFG1	10.18	10.10	10.20
217712_at	---	---	3.87	3.97	4.21
211863_x_at	hemochromatosis	HFE	6.30	5.92	5.87
218846_at	mediator complex	MED23	9.07	9.24	9.24
213844_at	homeobox A5	HOXA5	5.52	5.56	5.16
205821_at	killer cell lectin-lik	KLRK1	5.08	5.38	5.33
201609_x_at	isoprenylcysteine	ICMT	10.39	10.43	10.28
203165_s_at	solute carrier fam	SLC33A1	7.50	7.47	7.62
218215_s_at	nuclear receptor s	NR1H2	7.25	7.17	6.97
221166_at	fibroblast growth	FGF23	4.79	4.86	4.54
211751_at	phosphodiesteras	PDE4DIP	3.69	3.42	3.21
215908_at	---	---	7.62	7.64	7.35
206696_at	G protein-couplec	GPR143	4.16	4.29	4.58
210775_x_at	caspase 9, apopto	CASP9	7.17	7.40	7.16
216898_s_at	collagen, type IV,	COL4A3	3.97	4.10	3.99
221739_at	chromosome 19 c	C19orf10	10.14	10.06	10.07
214478_at	secreted phospho	SPP2	5.36	5.60	5.18
38447_at	adrenergic, beta, r	ADRBK1	7.88	8.06	8.00
207383_s_at	rhomboid, veinlet	RHBDL1	4.79	4.91	4.70
222316_at	---	---	5.97	6.57	6.25
200718_s_at	S-phase kinase-as:	SKP1	13.55	13.69	13.66
213716_s_at	secreted and tran	SECTM1	8.85	8.80	8.70
221723_s_at	solute carrier fam	SLC4A5	4.64	4.15	4.29
221691_x_at	nucleophosmin (n	NPM1	13.80	13.84	13.87
200700_s_at	KDEL (Lys-Asp-Glu	KDELR2	12.02	12.09	12.18

204078_at	synaptonemal cor	SC65	9.03	8.98	8.99
216549_s_at	TBC1 domain fam	TBC1D22B	4.83	4.49	4.57
218835_at	surfactant protein	SFTPA2	4.81	5.44	5.23
218227_at	nucleotide binding	NUBP2	8.66	8.68	8.65
220911_s_at	NYN domain and r	NYNRIN	4.95	4.87	4.87
209393_s_at	eukaryotic transla	EIF4E2	9.72	9.76	9.62
215040_at	Ribonuclease H2,	RNASEH2B	7.03	7.38	6.93
222381_at	Programmed cell	PDCD6	6.52	6.23	6.23
218920_at	family with seque	FAM193B	8.24	8.14	8.03
206336_at	chemokine (C-X-C	CXCL6	3.41	3.01	3.18
206450_at	dopamine beta-hy	DBH	6.49	6.63	6.32
210031_at	CD247 molecule	CD247	4.66	4.88	4.41
200056_s_at	C1D nuclear recep	C1D	8.75	8.89	9.09
215122_at	T-box 6	TBX6	4.41	4.63	4.38
219170_at	fibronectin type II	FSD1	7.95	7.87	7.48
220597_s_at	ADP-ribosylation-l	ARL6IP4	9.02	8.85	8.85
203144_s_at	KIAA0040	KIAA0040	4.15	4.15	3.88
204248_at	guanine nucleotid	GNA11	9.30	9.05	9.22
209178_at	DEAH (Asp-Glu-Ala	DHX38	8.01	7.89	7.93
204598_at	U-box domain cor	UBOX5	7.77	7.43	7.54
221023_s_at	potassium voltage	KCNH6	5.64	5.87	5.70
202096_s_at	translocator prote	TSPO	11.95	11.92	12.03
204096_s_at	elongation factor	ELL	7.21	7.16	7.21
219091_s_at	multimerin 2	MMRN2	5.02	4.98	5.26
215967_s_at	lymphocyte antige	LY9	4.19	4.39	4.25
211696_x_at	hemoglobin, beta	HBB	6.03	6.19	6.21
220792_at	PR domain contai	PRDM5	5.14	4.93	5.27
208558_at	olfactory receptor	OR10H1	5.78	5.58	5.80
213696_s_at	mediator complex	MED8	8.93	8.84	8.95
206047_at	guanine nucleotid	GNB3	7.02	7.25	7.09
207894_s_at	T-cell leukemia/ly	TCL6	5.37	5.48	5.17
204557_s_at	DAZ interacting pr	DZIP1	8.06	8.12	8.43
207172_s_at	cadherin 11, type	CDH11	5.06	5.16	5.33
200938_s_at	arginine-glutamic	RERE	5.31	4.84	4.82
214460_at	limbic system-ass	LSAMP	4.89	4.80	4.63
206477_s_at	neuro-oncological	NOVA2	8.19	8.17	7.89
203457_at	syntaxin 7	STX7	5.98	6.09	5.98
211839_s_at	colony stimulating	CSF1	6.61	6.80	6.53
218112_at	mitochondrial ribo	MRPS34	10.49	10.25	10.17
218808_at	DALR anticodon b	DALRD3	5.88	5.80	6.27
212094_at	paternally express	PEG10	10.17	10.25	10.34
220264_s_at	G protein-couplec	GPR107	6.42	6.58	6.60
211587_x_at	cholinergic recept	CHRNA3	7.03	6.98	6.63
217069_at	myeloid/lymphoic	MLL4	5.59	5.39	5.17
210323_at	tektin 2 (testicular	TEKT2	6.25	6.24	6.19
213453_x_at	glyceraldehyde-3-	GAPDH	14.43	14.39	14.36
211013_x_at	promyelocytic leu	PML	7.92	7.82	7.58

204819_at	FYVE, RhoGEF anc	FGD1	7.20	7.07	6.80
206131_at	colipase, pancreat	CLPS	4.88	4.74	4.48
214620_x_at	peptidylglycine al	PAM	7.98	8.11	8.13
222300_at	---	---	3.77	3.79	3.84
213648_at	exosome compon	EXOSC7	8.28	8.28	8.21
222332_at	---	---	3.79	3.72	3.78
207240_s_at	luteinizing hormo	LHCGR	3.46	3.35	3.24
217830_s_at	NSFL1 (p97) cofac	NSFL1C	8.22	8.27	8.36
209573_s_at	chromosome 18 c	C18orf1	6.29	6.44	5.85
220100_at	solute carrier fam	SLC22A11	5.50	5.40	5.04
211388_s_at	---	---	5.29	5.34	5.27
220552_at	transient receptor	TRPC5	3.49	3.36	3.24
202760_s_at	A kinase (PRKA) a	AKAP2 /// PALM2-	10.44	10.28	10.43
202597_at	interferon regulat	IRF6	5.74	5.80	5.60
217322_x_at	---	---	7.80	7.78	7.92
201793_x_at	Smg-7 homolog, r	SMG7	6.91	6.73	6.45
206299_at	family with seque	FAM155B	5.85	5.63	5.73
205077_s_at	phosphatidylinosi	PIGF	10.28	10.28	10.27
215847_at	hect domain and I	HERC2P3	3.68	3.72	3.63
214531_s_at	sorting nexin 1	SNX1	8.77	8.66	8.89
212817_at	DnaJ (Hsp40) hom	DNAJB5	6.75	6.70	6.59
221240_s_at	UDP-GlcNAc:beta	B3GNT4	7.73	7.42	7.14
211955_at	importin 5	IPO5	10.76	10.64	10.67
211070_x_at	diazepam binding	DBI	12.55	12.62	12.57
206168_at	zinc finger CCCH-t	ZC3H7B	5.65	5.75	5.47
203436_at	ribonuclease P/M	RPP30	10.00	10.04	9.99
218647_s_at	yrdC domain cont	YRDC	9.44	9.42	9.57
211295_x_at	cytochrome P450,	CYP2A6	5.94	6.03	6.06
221749_at	YTH domain famil	YTHDF3	10.20	10.37	10.58
221590_s_at	Aldehyde dehydro	ALDH6A1	5.14	4.78	4.79
203613_s_at	NADH dehydroge	NDUFB6	12.30	12.40	12.32
211211_x_at	SH2 domain conta	SH2D1A	6.12	6.15	5.74
213098_at	RCD1 required for	RQCD1	8.33	8.24	8.31
214644_at	histone cluster 1,	HIST1H2AK	3.65	3.49	3.33
204623_at	trefoil factor 3 (in	TFF3	5.94	6.01	6.44
208065_at	ST8 alpha-N-acety	ST8SIA3	3.53	3.43	3.05
AFFX-r2-Bs-thr-5	---	---	5.84	5.83	5.91
205033_s_at	defensin, alpha 1 ,	DEFA1 /// DEFA1B	5.66	5.64	5.41
217901_at	desmoglein 2	DSG2	11.09	11.09	11.22
213065_at	zinc finger, C3H1-t	ZFC3H1	8.44	8.74	8.59
204585_s_at	L1 cell adhesion m	L1CAM	6.94	6.68	6.63
218283_at	synovial sarcoma	SS18L2	9.72	9.91	9.82
211953_s_at	importin 5	IPO5	9.94	10.01	10.24
216744_at	---	---	4.10	3.91	3.99
217328_at	hypothetical prote	LOC100134017	5.06	5.16	4.93
221669_s_at	acyl-CoA dehydro	ACAD8	6.89	7.29	6.89
213369_at	cadherin-related f	CDHR1	4.85	4.85	4.94

206101_at	extracellular matr	ECM2	8.94	8.60	8.99
203489_at	SIVA1, apoptosis-i	SIVA1	10.20	10.13	10.22
221647_s_at	resistance to inhik	RIC8A	10.08	10.19	10.08
213834_at	IQ motif and Sec7	IQSEC3	5.74	5.47	5.18
216188_at	v-myc myelocytor	MYCNOS	4.38	4.38	4.33
AFFX-HUMISGF3	signal transducer	STAT1	6.92	7.10	7.18
211817_s_at	potassium inward	KCNJ5	5.62	5.62	5.72
207401_at	prospero homeob	PROX1	4.96	4.85	4.65
207314_x_at	killer cell immuno	KIR3DL1 /// KIR3D	4.14	4.79	4.20
212695_at	cryptochrome 2 (p	CRY2	6.81	6.71	6.63
207075_at	NLR family, pyrin	NLRP3	5.37	5.20	5.04
219358_s_at	ArfGAP with dual	ADAP2	6.73	6.55	6.43
213141_at	protein serine kin	PSKH1	7.50	7.41	7.52
211453_s_at	v-akt murine thym	AKT2	5.34	5.65	5.72
203452_at	beta-1,3-glucuron	B3GAT3	7.02	7.13	7.01
220683_at	retinol dehydroge	RDH8	5.17	5.25	4.72
217548_at	Chromosome 15 c	C15orf38	5.93	5.16	5.46
218969_at	mitochondria-ass	TIMM16	8.30	8.15	8.47
203849_s_at	kinesin family mei	KIF1A	4.67	4.98	4.88
210337_s_at	ATP citrate lyase	ACLY	9.91	9.87	9.88
204544_at	Hermansky-Pudlak	HPS5	9.34	9.30	9.32
208312_s_at	PRAME family me	PRAMEF1 /// PRAI	4.91	5.05	4.70
202098_s_at	protein arginine n	PRMT2	8.01	8.19	8.00
210935_s_at	WD repeat domai	WDR1	7.62	7.70	7.74
217294_s_at	enolase 1, (alpha)	ENO1	12.76	12.66	12.77
219936_s_at	G protein-couplec	GPR87	9.19	9.33	9.32
203834_s_at	trans-golgi netwo	TGOLN2	7.86	7.78	8.02
204787_at	V-set and immunc	VSIG4	4.58	4.91	4.74
206209_s_at	carbonic anhydras	CA4	4.35	4.70	4.58
208755_x_at	H3 histone, family	H3F3A /// LOC440	13.61	13.59	13.57
221222_s_at	chromosome 1 op	C1orf56	6.36	6.41	6.20
210750_s_at	discs, large (Drosc	DLGAP1	6.30	6.42	5.87
64488_at	immunity-related	IRGQ	7.97	7.93	7.89
214216_s_at	La ribonucleoprot	LARP4B	5.56	5.68	5.15
217511_at	Kazal-type serine	KAZALD1	7.23	7.06	7.06
205917_at	zinc finger protein	ZNF264	6.18	6.13	6.18
202909_at	EPM2A (laforin) ir	EPM2AIP1	9.48	9.84	9.56
205740_s_at	RNA binding moti	RBM42	7.73	7.60	7.54
201168_x_at	Rho GDP dissociat	ARHGDIA	8.45	8.48	8.43
207416_s_at	nuclear factor of a	NFATC3	8.27	8.16	8.09
214282_at	---	---	3.55	3.47	3.38
213432_at	mucin 5B, oligome	MUC5B	6.58	6.48	6.57
215650_at	---	---	4.53	4.85	4.31
201831_s_at	USO1 vesicle dock	USO1	9.05	9.11	9.05
206559_x_at	eukaryotic transla	EEF1A1	15.21	15.20	15.17
208512_s_at	myeloid/lymphoic	MLLT4	3.99	3.56	3.84
212140_at	PDS5, regulator of	PDS5A	9.93	10.09	10.16

205102_at	transmembrane p	TMPRSS2	6.80	6.82	6.58
204927_at	Ras association (R	RASSF7	8.30	8.49	8.21
212976_at	leucine rich repea	LRRC8B	5.58	5.78	5.65
214914_at	family with seque	FAM13C	3.26	3.35	3.15
221894_at	aarF domain conta	ADCK2	7.94	7.98	8.02
203702_s_at	tubulin tyrosine lig	TTLL4	7.19	7.20	7.14
204040_at	ring finger protein	RNF144A	3.87	3.76	3.67
217625_x_at	---	---	6.68	6.71	6.88
203425_s_at	insulin-like growth	IGFBP5	6.41	6.44	6.27
205353_s_at	phosphatidylethan	PEBP1	11.39	11.33	11.52
202518_at	B-cell CLL/lympho	BCL7B	8.16	8.21	8.33
202952_s_at	ADAM metallopep	ADAM12	7.75	7.78	7.80
217072_at	CD300a molecule	CD300A	5.74	5.65	5.72
217996_at	pleckstrin homolo	PHLDA1	10.73	10.70	10.82
204411_at	kinesin family mer	KIF21B	4.96	5.11	5.05
212079_s_at	myeloid/lymphoid	MLL	6.81	6.92	6.75
220438_at	glutaminyl-peptid	QPCTL	4.50	4.50	4.60
221610_s_at	signal transducing	STAP2	8.44	8.37	8.53
206977_at	parathyroid horm	PTH	3.37	3.18	3.42
209850_s_at	CDC42 effector pr	CDC42EP2	8.21	8.18	8.40
207489_at	ribosomal protein	RPS2P45	3.98	4.00	3.98
202811_at	developmentally r	DRG1	9.72	9.63	9.72
201342_at	small nuclear ribo	SNRPC	11.04	11.09	11.14
217312_s_at	collagen, type VII,	COL7A1	7.67	7.93	7.86
222002_at	Chromosome 7 op	C7orf26	5.04	4.84	4.74
AFFX-r2-Ec-bioC	---	---	11.01	10.96	10.96
214635_at	claudin 9	CLDN9	6.20	5.91	6.17
213758_at	cytochrome c oxid	COX4I1	6.90	6.54	6.55
220467_at	---	---	6.14	6.43	6.55
208062_s_at	neuregulin 2	NRG2	3.66	3.45	3.31
205971_s_at	chymotrypsinogen	CTRB1 /// CTRB2	4.80	4.63	4.44
213020_at	golgi SNAP recept	GOSR1	8.19	8.24	8.41
206845_s_at	ring finger protein	RNF40	7.86	7.81	7.78
204539_s_at	cadherin, EGF LAG	CELSR1	5.64	5.34	5.11
209607_x_at	sulfotransferase fa	SULT1A3 /// SULT1	6.94	6.96	7.05
36920_at	myotubularin 1	MTM1	4.33	4.30	4.36
202678_at	general transcript	GTF2A2	11.07	11.15	11.24
209412_at	trafficking protein	TRAPPC10	7.54	7.65	7.63
205184_at	guanine nucleotid	GNG4	5.15	5.14	5.24
218981_at	ACN9 homolog (S.	ACN9	8.91	8.99	9.23
221717_at	---	---	6.42	6.46	6.10
212033_at	RNA binding motif	RBM25	10.37	10.47	10.49
204999_s_at	activating transcri	ATF5	7.11	6.74	6.90
219944_at	CAP-GLY domain c	CLIP4	7.08	6.81	6.97
204458_at	phospholipase A2	PLA2G15	7.13	6.82	6.60
221605_s_at	pipecolic acid oxid	PIPOX	5.15	4.86	5.21
205369_x_at	dihydrolipoamide	DBT	7.26	7.10	7.27

214206_at	peptidylprolyl isor	PPIL6	5.44	5.33	5.44
210670_at	pancreatic polype	PPY	5.16	5.27	4.69
209364_at	BCL2-associated a	BAD	7.98	7.95	8.05
208993_s_at	peptidylprolyl isor	PPIG	10.34	10.45	10.58
217483_at	folate hydrolase (|	FOLH1	4.56	4.44	4.33
205343_at	sulfotransferase f	SULT1C2	3.95	4.13	3.80
208551_at	histone cluster 1,	HIST1H4G	5.20	5.11	4.93
213472_at	heterogeneous nu	HNRNPH1	3.85	4.12	3.87
213614_x_at	eukaryotic transla	EEF1A1	14.88	14.86	14.89
215301_at	synaptonemal cor	SYCE1L	3.23	3.15	3.11
221161_at	achaete-scute cor	ASCL3	5.45	5.63	5.40
207573_x_at	ATP synthase, H+	ATP5L	13.60	13.48	13.52
211646_at	immunoglobulin h	IGH@ /// IGHA1 //	3.37	3.31	3.26
208669_s_at	EP300 interacting	EID1	10.10	10.29	10.33
221912_s_at	coiled-coil domair	CCDC28B	7.76	7.59	7.77
217592_at	Zinc finger, SWIM	ZSWIM1	4.57	4.68	4.17
222229_x_at	ribosomal protein	RPL26	12.70	12.71	12.84
207539_s_at	interleukin 4	IL4	3.87	3.99	3.89
206834_at	hemoglobin, delta	HBD	6.12	6.10	5.73
213130_at	zinc finger proteir	ZNF473	6.67	6.66	6.66
55065_at	MAP/microtubule	MARK4	8.52	8.49	8.23
212708_at	male-specific leth	MSL1	10.27	10.16	10.22
214125_s_at	hypothetical prote	LOC100287008	6.57	6.60	6.49
215150_at	YOD1 OTU deubiq	YOD1	4.69	4.68	5.19
204434_at	spermatogenesis	SPATA2	7.89	8.02	7.99
214188_at	Hexamethylene b	HEXIM1	6.69	6.72	6.64
205554_s_at	deoxyribonucleas	DNASE1L3	4.19	4.15	4.27
218141_at	ubiquitin-conjugat	UBE2O	7.58	7.71	7.34
52285_f_at	centrosomal prote	CEP76	7.99	8.04	8.22
206889_at	protein disulfide i	PDIA2	7.13	7.04	6.96
205219_s_at	galactokinase 2	GALK2	8.50	8.32	8.40
220935_s_at	CDK5 regulatory s	CDK5RAP2	8.40	8.42	8.42
205534_at	protocadherin 7	PCDH7	7.16	7.08	7.28
201213_at	protein phosphata	PPP1R7	7.45	7.42	7.18
216516_at	---	---	4.32	4.35	3.99
216138_at	---	---	6.85	7.03	6.69
205573_s_at	sorting nexin 7	SNX7	11.54	11.51	11.52
201330_at	arginyl-tRNA synth	RARS	11.06	11.16	11.08
215465_at	ATP-binding casse	ABCA12	3.35	3.71	3.37
220395_at	DnaJ (Hsp40) hom	DNAJA4	7.53	7.54	7.22
204546_at	KIAA0513	KIAA0513	7.39	7.55	6.93
211215_x_at	deiodinase, iodotl	DIO2	6.83	6.83	6.37
210767_at	neurofibromin 2 (	NF2	4.08	4.19	4.30
210824_at	---	---	4.84	5.18	4.81
206803_at	prodynorphin	PDYN	5.01	4.84	4.74
211021_s_at	regulator of G-prc	RGS14	6.04	5.78	5.34
217753_s_at	ribosomal protein	RPS26	13.93	13.95	14.00

207203_s_at	nuclear receptor s	NR1I2	6.95	6.63	6.51
213190_at	component of olig	COG7	6.54	6.58	6.71
216927_at	KIAA0892	KIAA0892	6.27	6.26	5.92
216793_x_at	---	---	4.30	4.00	3.96
208905_at	cytochrome c, sor	CYCS	13.33	13.42	13.51
208291_s_at	tyrosine hydroxyla	TH	4.13	4.29	4.42
213342_at	Yes-associated pro	YAP1	8.36	8.37	8.56
204104_at	small nuclear RNA	SNAPC2	7.59	7.23	7.47
221067_s_at	chromosome 12 o	C12orf39	6.18	5.97	6.05
219688_at	Bardet-Biedl synd	BBS7	7.56	7.61	7.85
217800_s_at	Nedd4 family inte	NDFIP1	9.49	9.68	9.75
206149_at	calcineurin B hom	CHP2	6.20	5.76	5.81
204948_s_at	follistatin	FST	6.17	6.39	5.92
212099_at	ras homolog gene	RHOB	8.54	8.63	8.53
201248_s_at	sterol regulatory e	SREBF2	8.17	7.94	8.04
200799_at	heat shock 70kDa	HSPA1A	6.83	6.87	6.76
216183_at	transglutaminase	TGM2	6.00	5.90	5.76
208361_s_at	polymerase (RNA)	POLR3D	7.90	7.84	7.91
210341_at	myelin transcripti	MYT1	4.76	4.65	4.81
205992_s_at	interleukin 15	IL15	7.20	7.06	7.70
208343_s_at	nuclear receptor s	NR5A2	3.66	3.45	3.55
217159_x_at	sialic acid binding	SIGLEC7	6.02	5.85	5.78
221137_at	---	---	4.63	4.35	4.25
216601_at	AOC3 pseudogene	LOC90586	6.76	6.53	6.49
212358_at	CAP-GLY domain c	CLIP3	6.61	6.48	6.25
201232_s_at	proteasome (pros	PSMD13	10.42	10.46	10.54
205493_s_at	dihydropyrimidina	DPYSL4	4.78	4.39	4.35
221827_at	RanBP-type and C	RBCK1	11.35	11.07	11.37
217082_at	Hypothetical prot	LOC100288679	3.60	3.87	4.15
202010_s_at	zinc finger protein	ZNF410	9.48	9.45	9.60
211296_x_at	ubiquitin C	UBC	13.86	13.89	13.74
217480_x_at	similar to Ig kappa	LOC100287723 ///	8.24	8.18	7.75
207708_at	arachidonate lipo	ALOXE3	5.56	5.32	5.43
200858_s_at	ribosomal protein	RPS8	14.16	14.15	14.27
206608_s_at	retinitis pigmento	RPGRIP1	5.42	5.30	5.18
201524_x_at	ubiquitin-conjugat	UBE2N	11.70	11.76	11.66
214532_x_at	POU class 5 home	POU5F1B	6.96	6.85	6.97
204634_at	NIMA (never in m	NEK4	8.62	8.78	8.88
206421_s_at	serpin peptidase i	SERPINB7	4.28	4.70	4.54
208271_at	poly(A) polymeras	PAPOLB	3.51	3.61	3.29
221502_at	karyopherin alpha	KPNA3	10.92	11.13	11.18
206195_x_at	growth hormone 2	GH2	4.25	4.05	3.83
200866_s_at	prosaposin	PSAP	10.70	10.45	10.51
218753_at	XK, Kell blood gro	XKR8	9.50	9.64	9.35
204274_at	estrogen receptor	EBAG9	10.61	10.64	10.84
210433_at	protein O-fucosylt	POFUT1	4.76	4.68	4.75
210865_at	Fas ligand (TNF su	FASLG	4.40	4.12	4.68

213125_at	olfactomedin-like	OLFML2B	4.95	4.65	4.70
209232_s_at	dynactin 5 (p25)	DCTN5	8.52	8.54	8.52
214666_x_at	iron-responsive el	IREB2	6.27	6.00	6.03
33850_at	microtubule-asso	MAP4	8.78	8.74	8.74
219270_at	ChaC, cation trans	CHAC1	9.20	9.07	9.12
221138_s_at	---	---	3.42	3.46	3.37
205452_at	phosphatidylinosi	PIGB	8.12	8.16	8.36
213378_s_at	DEAD/H (Asp-Glu-	DDX11 /// DDX12	5.78	6.01	6.04
214865_at	DOT1-like, histone	DOT1L	5.58	5.47	5.11
207150_at	solute carrier fam	SLC18A3	5.15	5.06	4.94
217223_s_at	breakpoint cluster	BCR	6.58	6.75	6.64
211332_x_at	hemochromatosis	HFE	6.50	6.88	6.35
210947_s_at	mutS homolog 3 (	MSH3	8.05	7.97	8.10
216530_at	---	---	5.38	5.34	5.06
220909_at	tripartite motif-co	TRIM46	3.80	3.73	3.73
218067_s_at	arginine and gluta	ARGLU1	10.49	10.50	10.53
211190_x_at	CD84 molecule	CD84	4.26	4.44	4.55
216983_s_at	zinc finger protein	ZNF224	5.82	5.60	5.74
201374_x_at	protein phosphata	PPP2CB	5.72	5.67	5.62
214263_x_at	polymerase (RNA)	POLR2C	9.98	10.06	9.92
203294_s_at	lectin, mannose-b	LMAN1	5.06	5.05	5.10
214238_at	---	---	4.87	4.86	4.26
212523_s_at	KIAA0146	KIAA0146	7.24	7.31	7.29
207239_s_at	cyclin-dependent	CDK16	6.39	6.54	6.28
208608_s_at	syntrophin, beta 1	SNTB1	5.39	5.14	5.14
219952_s_at	mucolipin 1	MCOLN1	6.44	6.61	6.72
206479_at	transient receptor	TRPM1	3.52	3.79	3.86
202091_at	ADP-ribosylation f	ARL2BP	4.31	4.21	4.34
212689_s_at	lysine (K)-specific	KDM3A	8.86	8.79	9.03
217938_s_at	potassium channe	KCMF1	12.20	12.06	12.19
211224_s_at	ATP-binding casse	ABCB11	4.76	4.63	4.68
207246_at	zinc finger protein	ZFY	3.43	3.60	3.45
208084_at	integrin, beta 6	ITGB6	4.70	4.58	4.61
215901_at	zinc finger protein	ZNF81	3.30	3.14	3.23
215093_at	NAD(P) dependen	NSDHL	8.01	8.33	8.17
202850_at	G protein-couplec	GRK6	10.07	10.33	10.23
218239_s_at	GTP binding prote	GTPBP4	11.21	11.21	11.42
211297_s_at	cyclin-dependent	CDK7	10.69	10.77	10.85
222366_at	---	---	6.58	6.87	6.48
217014_s_at	alpha-2-glycoprot	AZGP1	6.04	6.04	6.12
220218_at	chromosome 9 op	C9orf68	3.83	3.91	4.12
215690_x_at	glycosylphosphati	GPAA1	9.38	9.36	9.37
219994_at	amyloid beta (A4)	APBB1IP	5.28	5.40	4.84
201409_s_at	protein phosphata	PPP1CB	10.35	10.41	10.53
217544_at	similar to hCG172	LOC729806	4.35	4.14	4.12
201300_s_at	prion protein	PRNP	11.86	11.77	12.04
221880_s_at	family with seque	FAM174B	5.88	5.70	5.78

38398_at	MAP-kinase activa	MADD	8.22	8.30	8.32
212075_s_at	casein kinase 2, al	CSNK2A1	8.92	8.90	9.08
207851_s_at	insulin receptor	INSR	5.42	5.50	5.42
206683_at	zinc finger protein	ZNF165	4.30	4.67	4.39
215379_x_at	immunoglobulin la	IGLV1-44	7.15	7.05	7.06
210220_at	frizzled homolog 2	FZD2	6.83	6.90	6.67
209729_at	growth arrest-spe	GAS2L1	8.28	8.18	8.03
221148_at	---	---	5.98	5.80	5.45
210743_s_at	CDC14 cell divisio	CDC14A	3.91	3.78	3.65
220436_at	similar to cell reco	CNTNAP3B	4.42	4.47	4.18
203274_at	coagulation facto	F8A1 /// F8A2 ///	9.44	9.48	9.51
208367_x_at	cytochrome P450,	CYP3A4	4.43	4.39	4.24
212146_at	pleckstrin homolo	PLEKHM2	7.57	7.65	7.70
201078_at	transmembrane 9	TM9SF2	11.02	11.16	11.10
207584_at	lipoprotein, Lp(a)	LPA	3.68	3.62	3.49
211727_s_at	COX11 cytochrom	COX11	10.43	10.58	10.65
218184_at	tubby like protein	TULP4	8.55	8.45	8.69
210205_at	UDP-Gal:betaGlcN	B3GALT4	7.21	7.27	7.17
221292_at	patched homolog	PTCH2	6.13	6.23	5.83
215684_s_at	activating signal c	ASCC2	7.73	7.66	7.84
218515_at	GC-rich sequence	GCFC1	9.50	9.71	9.49
219685_at	transmembrane p	TMEM35	3.51	3.43	3.30
211167_s_at	glucokinase (hexo	GCK	5.74	5.22	5.01
204743_at	transgelin 3	TAGLN3	5.75	5.79	5.69
216394_x_at	---	---	6.26	6.00	5.89
206425_s_at	transient recepto	TRPC3	3.68	3.43	3.62
213651_at	inositol polyphos	INPP5J	7.78	7.63	7.51
201932_at	leucine rich repea	LRRC41	8.52	8.59	8.61
210811_s_at	DEAD (Asp-Glu-Al	DDX49	7.33	7.09	7.23
210870_s_at	epilepsy, progress	EPM2A	4.89	5.22	4.97
216997_x_at	transducin-like en	TLE4	3.34	3.25	3.52
217796_s_at	nuclear protein lo	NPLOC4	9.60	9.54	9.45
217035_at	Similar to hCG181	LOC100293559	5.85	6.09	6.09
218564_at	ring finger and WI	RFWD3	8.25	8.39	8.39
206078_at	kalirin, RhoGEF ki	KALRN	3.76	3.53	3.69
211343_s_at	collagen, type XIII,	COL13A1	4.34	4.71	4.57
220616_at	zinc finger protein	ZNF384	5.74	6.10	5.41
208031_s_at	regulatory factor X	RFX2	7.46	7.23	7.09
31637_s_at	nuclear receptor s	NR1D1 /// THRA	9.16	8.98	8.90
221955_at	---	---	5.80	5.62	5.14
210124_x_at	sema domain, imr	SEMA4F	7.29	7.36	7.31
201431_s_at	dihydropyrimidina	DPYSL3	6.55	6.51	6.48
214228_x_at	tumor necrosis fa	TNFRSF4	6.88	6.97	6.61
201394_s_at	RNA binding moti	RBM5	8.22	8.33	8.24
209494_s_at	POZ (BTB) and AT	PATZ1	7.23	7.31	7.05
221403_s_at	insulin-like 6	INSL6	5.25	5.27	5.22
202299_s_at	hepatitis B virus x	HBXIP	11.64	11.69	11.64

203290_at	major histocompa	HLA-DQA1	5.30	5.25	5.49
204318_s_at	G-2 and S-phase e	GTSE1	8.87	9.04	9.06
205763_s_at	DEAD (Asp-Glu-Al	DDX18	9.52	9.57	9.59
206852_at	EPH receptor A7	EPHA7	3.51	3.60	3.49
205291_at	interleukin 2 rece	IL2RB	6.01	5.72	5.68
212160_at	exportin, tRNA (nu	XPOT	12.18	12.12	12.36
213458_at	family with seque	FAM149B1	3.36	3.45	3.21
209436_at	spondin 1, extrace	SPON1	6.17	6.11	5.84
204311_at	ATPase, Na+/K+ tr	ATP1B2	5.31	5.40	5.18
220039_s_at	CDK5 regulatory s	CDKAL1	4.19	3.91	4.10
212018_s_at	ribosomal L1 dom	RSL1D1	11.16	11.15	11.28
202759_s_at	A kinase (PRKA) ai	AKAP2 /// PALM2-	10.55	10.46	10.60
219925_at	zinc finger, MYM-1	ZMYM6	4.84	5.03	4.93
218231_at	N-acetylglucosam	NAGK	8.73	8.85	8.63
220185_at	spectrin, beta, noi	SPTBN4	7.19	6.99	6.71
206489_s_at	discs, large (Drosc	DLGAP1	3.64	3.17	3.48
205555_s_at	msh homeobox 2	MSX2	5.56	5.44	5.07
208600_s_at	G protein-couplec	GPR39	5.31	5.13	4.98
205394_at	CHK1 checkpoint I	CHEK1	8.02	8.17	8.32
213539_at	CD3d molecule, d	CD3D	5.95	5.89	6.16
217482_at	---	---	7.10	7.21	7.00
205731_s_at	nuclear receptor c	NCOA2	4.90	4.62	4.62
216296_at	clathrin, light chai	CLTA	6.14	5.89	5.91
215636_at	ubiquitin protein I	UBR4	5.54	5.37	5.35
216469_at	similar to Putative	LOC100288765	3.29	3.19	3.15
213712_at	elongation of very	ELOVL2	3.63	3.47	3.69
200900_s_at	mannose-6-phosp	M6PR	9.79	9.95	10.00
219928_s_at	calcium binding ty	CABYR	5.41	5.97	5.61
207191_s_at	immunoglobulin s	ISLR	5.07	4.32	4.72
213671_s_at	methionyl-tRNA s	MARS	11.75	11.36	11.74
219943_s_at	ubiquitin specific		USP46	6.99	7.19	6.73
206680_at	CD5 molecule-like	CD5L	4.03	3.93	4.01
214165_s_at	Heparan sulfate 6	HS6ST1	6.47	6.07	6.21
201412_at	low density lipopr	LRP10	9.97	9.81	9.90
210991_s_at	regulating synapti	RIMS3	5.46	5.75	5.66
220961_s_at	transforming grov	TBRG4	5.33	5.71	5.62
220144_s_at	ankyrin repeat do	ANKRD5	5.94	5.99	5.73
204222_s_at	GLI pathogenesis-	GLIPR1	9.96	9.98	9.92
217076_s_at	homeobox D3	HOXD3	3.36	3.67	3.09
217206_at	---	---	4.32	4.38	4.26
220735_s_at	SUMO1/sentrin sp	SENP7	3.98	3.95	3.91
201228_s_at	ariadne homolog 2	ARIH2	6.99	6.83	7.03
207882_at	---	---	7.15	6.95	6.60
221370_at	similar to Zinc fing	LOC100287163 ///	3.38	3.65	3.34
215189_at	keratin 86	KRT86	5.24	5.11	5.24
217026_at	cystic fibrosis tran	CFTR	3.60	3.52	3.58
221366_at	NK6 homeobox 1	NKX6-1	4.82	4.44	4.40

221914_at	synapsin I	SYN1	4.39	4.57	4.50
203330_s_at	syntaxin 5	STX5	5.50	5.33	5.77
205080_at	retinoic acid recep	RARB	4.57	4.59	4.56
215741_x_at	A kinase (PRKA) ar	AKAP8L	7.88	7.73	7.57
218727_at	solute carrier fam	SLC38A7	6.43	6.69	6.44
215139_at	Rho guanine nucle	ARHGEF10	5.85	5.59	5.98
215218_s_at	WD repeat domai	WDR62	7.83	7.72	7.66
219173_at	myosin XVB pseuc	MYO15B	6.17	6.39	6.12
208095_s_at	signal recognition	SRP72	11.96	11.97	12.11
204528_s_at	nucleosome asser	NAP1L1	12.49	12.42	12.59
213374_x_at	3-hydroxyisobutyr	HIBCH	8.85	8.93	9.00
203009_at	basal cell adhesio	BCAM	6.65	6.54	6.24
213451_x_at	tenascin XA pseuc	TNXA /// TNXB	6.39	6.16	6.08
219350_s_at	diablo homolog (D	DIABLO	10.58	10.50	10.46
201808_s_at	endoglin	ENG	6.92	6.74	6.22
49679_s_at	hypothetical LOC7	LOC729580	8.28	8.30	8.33
220956_s_at	egl nine homolog	EGLN2	8.38	8.40	8.21
219227_at	cyclin J-like	CCNJL	6.71	6.62	6.65
213808_at	---	---	3.88	3.76	3.61
205017_s_at	muscleblind-like 2	MBNL2	5.62	5.31	5.44
216933_x_at	adenomatous pol	APC	3.69	3.60	3.44
221209_s_at	otoraplin	OTOR	4.67	4.71	4.67
215613_at	ADAM metallope	ADAM12	5.09	5.05	4.58
222065_s_at	flightless I homolo	FLII	8.34	8.33	8.39
202488_s_at	FXYD domain cont	FXYD3	7.08	7.23	6.96
219524_s_at	chromosome 20 c	C20orf7	5.51	5.51	5.37
212983_at	v-Ha-ras Harvey ra	HRAS	8.52	8.60	8.49
216418_at	ATP-binding casse	ABCD1	4.52	4.77	4.55
AFFX-M27830_3	---	---	5.47	5.72	5.63
218802_at	coiled-coil domair	CCDC109B	9.69	9.79	9.81
219962_at	angiotensin I conv	ACE2	3.70	3.66	3.52
221638_s_at	syntaxin 16	STX16	5.43	5.16	5.33
201384_s_at	neighbor of BRCA	NBR1	10.14	10.06	10.21
218622_at	nucleoporin 37kD	NUP37	11.22	11.32	11.37
205377_s_at	acetylcholinestera	ACHE	6.32	6.37	5.93
220433_at	proline rich Gla (G	PRRG3	5.33	5.66	5.24
219458_s_at	NOP2/Sun domair	NSUN3	8.13	8.05	8.12
215657_at	Solute carrier fam	SLC26A3	3.57	3.60	3.55
212684_at	zinc finger proteir	ZNF3	7.77	8.14	8.21
203812_at	---	---	6.71	6.80	6.94
204485_s_at	target of myb1 (cl	TOM1L1	9.34	9.43	9.58
217235_x_at	immunoglobulin la	IGLL5 /// IGLV2-23	5.78	5.85	5.46
205045_at	A kinase (PRKA) ar	AKAP10	6.15	6.16	5.85
221050_s_at	GTP binding prote	GTPBP2	8.60	7.96	8.35
217053_x_at	ets variant 1	ETV1	6.15	6.10	5.93
221402_at	olfactory receptor	OR1F1	4.98	5.17	4.94
219308_s_at	adenylate kinase 5	AK5	4.81	4.74	4.94

217561_at	calcitonin-related	CALCA	3.64	3.83	3.77
203809_s_at	v-akt murine thym	AKT2	4.83	4.44	4.88
207677_s_at	neutrophil cytoso	NCF4	4.79	4.64	4.57
213266_at	Tubulin, gamma c	TUBGCP4	6.35	6.61	6.30
202566_s_at	supervillin	SVIL	7.61	7.66	7.85
208833_s_at	ataxin 10	ATXN10	12.25	12.30	12.32
203143_s_at	KIAA0040	KIAA0040	5.56	5.46	5.21
89948_at	PDX1 C-terminal i	PCIF1	7.14	7.30	7.37
210499_s_at	polyglutamine bin	PQBP1	7.90	7.49	7.47
221966_at	G protein-couplec	GPR137	7.44	7.52	6.73
212761_at	transcription facto	TCF7L2	10.88	10.58	10.91
210381_s_at	cholecystokinin B	CCKBR	5.65	5.60	5.45
217100_s_at	UBX domain prote	UBXN7	8.75	8.62	8.62
220311_at	N-6 adenine-speci	N6AMT1	7.40	7.53	7.68
221935_s_at	chromosome 3 op	C3orf64	8.10	8.32	8.31
221037_s_at	solute carrier fam	SLC25A31	3.44	3.30	3.34
209794_at	SLIT-ROBO Rho GT	SRGAP3	6.12	6.23	5.90
200843_s_at	glutamyl-prolyl-tR	EPRS	11.70	11.58	11.69
206641_at	tumor necrosis fac	TNFRSF17	3.62	3.76	3.55
214337_at	coatomer protein	COPA	4.95	4.81	5.11
213398_s_at	short chain dehyd	SDR39U1	10.07	9.84	9.93
206275_s_at	microtubule assoc	MICAL2	6.56	6.26	6.31
218817_at	signal peptidase c	SPCS3	8.20	8.24	8.23
204289_at	aldehyde dehydro	ALDH6A1	5.59	5.49	5.34
202030_at	branched chain ke	BCKDK	7.97	7.87	7.89
201630_s_at	acid phosphatase	ACP1	10.69	10.74	10.81
207292_s_at	mitogen-activatec	MAPK7	6.21	6.65	6.06
200855_at	nuclear receptor c	C20orf191 /// LOC	7.37	7.17	7.15
210541_s_at	tripartite motif-co	TRIM27	8.36	8.23	8.33
213416_at	integrin, alpha 4 (a	ITGA4	3.57	3.93	3.64
40640_at	non-SMC condens	NCAPH2	7.57	7.46	7.31
215996_at	---	---	5.45	5.32	4.97
206733_at	tubby like protein	TULP2	4.18	4.27	3.97
210732_s_at	lectin, galactoside	LGALS8	6.64	6.82	7.01
219375_at	choline/ethanolar	CEPT1	10.54	10.39	10.57
210519_s_at	NAD(P)H dehydro	NQO1	11.87	11.95	11.91
218468_s_at	gremlin 1, cystein	GREM1	3.72	3.81	3.75
207951_at	casein beta	CSN2	3.84	3.81	3.77
203871_at	SUMO1/sentrin/S	SENP3	8.21	8.13	8.13
217915_s_at	ribosomal L24 dor	RSL24D1	11.88	11.94	12.10
208953_at	La ribonucleoprot	LARP4B	9.49	9.47	9.44
215204_at	---	---	6.09	6.71	6.32
215192_at	postmeiotic segre	PMS2L4	3.36	3.33	3.44
205256_at	zinc finger and BT	ZBTB39	6.92	7.06	6.99
217290_at	---	---	4.14	4.12	4.03
203602_s_at	zinc finger and BT	ZBTB17	8.25	8.24	8.16
216710_x_at	zinc finger protein	ZNF287	3.72	3.74	3.75

215782_at	RAB40A, member	RAB40AL	4.72	4.81	4.61
216946_at	major histocompa	HLA-DOA	4.07	4.09	4.04
210050_at	triosephosphate i:	TPI1	6.26	6.21	6.33
210781_x_at	glutamate recepto	GRIN1	6.81	7.01	6.62
37254_at	zinc finger proteir	ZNF133	7.86	7.87	7.77
59625_at	nucleolar protein	NOL3	8.23	8.16	8.23
201539_s_at	four and a half LIN	FHL1	4.85	4.88	5.69
216385_at	lysyl oxidase-like 3	LOXL3	7.55	7.48	7.11
214612_x_at	melanoma antigen	MAGEA6	11.77	11.80	11.67
206625_at	peripherin 2 (retir	PRPH2	5.07	5.02	5.05
216432_at	solute carrier fam	SLC28A2	3.88	4.23	4.33
207166_at	guanine nucleotid	GNGT1	4.33	3.98	4.08
212364_at	myosin IB	MYO1B	8.84	8.93	9.22
215541_s_at	diaphanous homo	DIAPH1	8.23	8.33	7.95
208698_s_at	non-POU domain	NONO	9.36	9.27	9.46
208152_s_at	DEAD (Asp-Glu-Al	DDX21	12.51	12.53	12.64
217976_s_at	dynein, cytoplasm	DYNC1LI1	8.85	9.05	9.15
216763_at	KN motif and ank	KANK1	4.87	4.71	4.72
220545_s_at	testis-specific seri	TSKS	3.83	3.92	3.79
219151_s_at	RAB, member of F	RABL2A /// RABL2	7.07	7.23	7.28
201495_x_at	myosin, heavy cha	MYH11	5.45	5.29	5.23
219396_s_at	nei endonuclease	NEIL1	6.75	6.78	6.66
217686_at	protein tyrosine p	PTPN1	6.28	6.43	6.17
207837_at	RNA binding prote	RBPMS	3.45	3.78	3.77
211890_x_at	calpain 3, (p94)	CAPN3	5.46	5.69	5.53
215680_at	KIAA1654 protein	KIAA1654	6.10	5.75	5.76
219438_at	Na+/K+ transporti	NKAIN1	5.90	5.99	5.89
218338_at	polyhomeotic hor	PHC1	7.31	7.35	7.45
208957_at	endoplasmic retic	ERP44	6.91	6.86	6.83
207042_at	E2F transcription	E2F2	5.82	6.03	5.42
203615_x_at	sulfotransferase fa	SULT1A1	7.07	7.34	6.75
214975_s_at	myotubularin rela	MTMR1	4.97	5.07	4.97
214266_s_at	PDZ and LIM dom	PDLIM7	5.39	5.40	5.03
201996_s_at	spen homolog, tra	SPEN	10.20	10.18	10.14
207742_s_at	nuclear receptor s	NR6A1	5.94	5.80	5.69
206308_at	tRNA aspartic acic	TRDMT1	7.79	7.88	8.03
205849_s_at	ubiquinol-cytochr	UQCRB	12.66	12.56	12.80
217194_at	---	---	2.88	2.90	3.19
214892_x_at	chromosome 19 c	C19orf29	7.31	7.50	7.19
202027_at	transmembrane p	TMEM184B	8.55	8.50	8.55
221801_x_at	neurofilament, lig	NEFL	3.89	3.64	3.85
200817_x_at	ribosomal protein	RPS10	14.92	14.90	14.93
200874_s_at	NOP56 ribonuclec	NOP56	9.80	9.56	9.76
AFFX-ThrX-3_at	---	---	9.36	9.27	9.23
212920_at	RE1-silencing tran	REST	8.41	8.47	8.62
210373_at	nitrogen permeas	NPRL2	7.38	7.39	7.35
218672_at	sodium channel m	SCNM1	9.52	9.46	9.42

213604_at	transcription elon	TCEB3	8.88	8.60	8.75
220490_at	---	---	5.16	5.42	5.20
206232_s_at	UDP-Gal:betaGlcN	B4GALT6	4.02	4.13	4.09
214086_s_at	poly (ADP-ribose)	PARP2	9.49	9.42	9.67
219763_at	DENN/MADD dom	DENND1A	8.21	8.14	8.43
209533_s_at	phospholipase A2	PLAA	8.58	8.48	8.58
216480_x_at	Myeloid/lymphoid	MLLT10	4.58	4.53	4.47
221357_at	cholinergic recept	CHRM4	5.95	5.87	6.06
209898_x_at	intersectin 2	ITSN2	9.27	9.38	9.49
213050_at	cordon-bleu homo	COBL	4.28	4.11	4.12
210199_at	crystallin, alpha A	CRYAA	6.26	6.09	6.02
219687_at	hedgehog acyltran	HHAT	4.79	4.39	4.58
206591_at	recombination act	RAG1	3.79	3.56	3.73
212908_at	DnaJ (Hsp40) hom	DNAJC16	9.45	9.47	9.40
204125_at	NADH dehydrogen	NDUFAF1	9.01	9.09	8.97
217490_at	---	---	5.43	5.74	5.55
219053_s_at	vacuolar protein s	VPS37C	9.53	9.22	9.45
218004_at	BSD domain conta	BSDC1	8.31	8.23	8.20
221566_s_at	nucleolar protein	NOL3	8.23	8.18	8.14
215738_at	---	---	5.84	5.74	5.59
215568_x_at	lysophospholipase	LYPLA2 /// LYPLA2	8.70	8.84	8.54
213043_s_at	mediator complex	MED24	7.75	7.78	7.72
219165_at	PDZ and LIM dom	PDLIM2	9.62	9.58	9.67
211187_at	---	---	3.66	3.48	3.33
218200_s_at	NADH dehydrogen	NDUFB2	12.25	12.25	12.20
206497_at	chromosome 7 op	C7orf44	4.84	5.12	5.16
34697_at	low density lipopr	LRP6	6.28	5.98	5.71
209643_s_at	phospholipase D2	PLD2	7.33	7.06	7.11
201352_at	YME1-like 1 (S. ce	YME1L1	11.82	11.90	11.86
205116_at	laminin, alpha 2	LAMA2	5.90	5.35	5.51
208345_s_at	POU class 3 home	POU3F1	4.05	3.81	3.76
203384_s_at	golgin A1	GOLGA1	7.57	7.68	7.60
211697_x_at	partner of NOB1 h	PNO1	10.13	10.10	10.25
207974_s_at	S-phase kinase-as:	SKP1	11.79	11.78	11.93
209861_s_at	methionyl aminop	METAP2	10.58	10.70	10.80
218534_s_at	angiogenic factor	AGGF1	8.89	9.04	9.09
213476_x_at	tubulin, beta 3	TUBB3	11.59	11.71	11.56
217417_at	myosin VA (heavy	MYO5A	3.31	3.55	3.17
208073_x_at	tetratricopeptide	TTC3	10.39	10.61	10.60
219695_at	sphingomyelin ph	SMPD3	4.30	4.32	3.99
201435_s_at	eukaryotic transla	EIF4E	9.90	10.07	10.15
211302_s_at	phosphodiesteras	PDE4B	4.43	4.38	4.24
211716_x_at	Rho GDP dissociat	ARHGDIA	9.12	9.20	9.11
202222_s_at	desmin	DES	6.73	6.57	6.45
206826_at	peripheral myelin	PMP2	3.31	3.05	3.17
205432_at	oviductal glycopro	OVGP1	6.36	6.67	6.40
220682_s_at	---	---	5.18	5.70	5.37

219504_s_at	RNA polymerase I	RPAP2	7.10	7.35	7.22
216987_at	interferon regulat	IRF4	3.46	3.36	3.16
221602_s_at	Fas apoptotic inhi	FAIM3	5.68	5.74	5.74
206423_at	angiopoietin-like 7	ANGPTL7	5.34	5.26	5.31
219412_at	RAB38, member R	RAB38	4.52	4.55	4.67
201608_s_at	PWP1 homolog (S	PWP1	11.00	11.00	11.06
216518_at	---	---	3.54	3.47	3.27
217507_at	solute carrier fam	SLC11A1	6.21	6.24	5.99
211554_s_at	apoptotic peptida	APAF1	6.25	6.37	6.02
204421_s_at	fibroblast growth	FGF2	9.19	9.01	9.35
212031_at	RNA binding moti	RBM25	9.72	9.79	9.87
216036_x_at	WD and tetratrico	WDTC1	8.21	8.11	7.83
207845_s_at	anaphase promot	ANAPC10	9.04	8.93	9.07
218316_at	translocase of inn	TIMM9	9.76	9.55	9.86
218937_at	zinc finger protein	ZNF434	7.28	7.59	7.56
221554_at	STE20-related kin	STRADA	8.28	8.13	8.13
205119_s_at	formyl peptide re	FPR1	5.70	5.58	5.48
212959_s_at	N-acetylglucosam	GNPTAB	8.73	8.97	9.14
219242_at	centrosomal prot	CEP63	9.42	9.49	9.53
209437_s_at	spondin 1, extrace	SPON1	5.96	5.66	5.89
212321_at	sphingosine-1-ph	SGPL1	8.70	8.50	8.84
218467_at	proteasome (pros	PSMG2	11.74	11.71	11.78
210279_at	G protein-couplec	GPR18	3.59	3.59	3.19
220649_at	ATP/GTP binding	AGBL3	3.33	3.13	3.33
217538_at	small G protein si	SGSM2	4.69	4.63	4.50
205890_s_at	gamma-aminobut	GABBR1 /// UBD	5.31	4.73	4.78
207155_at	T-box 5	TBX5	4.22	4.26	3.89
210974_s_at	adaptor-related p	AP3D1	7.83	7.85	7.82
217635_s_at	polymerase (DNA	POLG	7.77	7.62	7.73
210082_at	ATP-binding casse	ABCA4	4.92	4.99	4.55
209949_at	neutrophil cytoso	NCF2	4.14	3.82	3.91
206649_s_at	transcription fact	TFE3	7.55	7.74	7.54
218918_at	mannosidase, alpl	MAN1C1	4.31	4.24	4.18
216664_at	---	---	4.44	4.34	4.30
209872_s_at	plakophilin 3	PKP3	5.81	5.50	5.37
57715_at	calcium homeosta	CALHM2	4.41	4.64	4.22
217787_s_at	UDP-N-acetyl-alpl	GALNT2	7.59	7.60	7.78
209890_at	tetraspanin 5	TSPAN5	5.01	4.95	4.88
209952_s_at	mitogen-activatec	MAP2K7	6.04	6.12	5.57
209340_at	UDP-N-acteylgluc	UAP1	11.81	11.70	11.96
219946_x_at	myosin, heavy cha	MYH14	6.64	6.76	6.50
217365_at	PRAME family me	PRAMEF11	6.09	6.27	6.00
207797_s_at	LRP2 binding prot	LRP2BP	5.24	5.03	5.13
208759_at	nicastrin	NCSTN	7.61	7.78	7.84
203250_at	RNA binding moti	RBM16	10.72	10.65	10.73
204885_s_at	mesothelin	MSLN	4.70	4.60	4.56
201685_s_at	TOX high mobility	TOX4	8.19	8.10	8.33

219509_at	myozenin 1	MYOZ1	5.38	5.51	5.16
220361_at	IQ motif containir	IQCH	3.86	3.96	3.64
201086_x_at	SON DNA binding	SON	10.98	11.07	11.04
210039_s_at	protein kinase C, t	PRKCQ	4.93	5.33	4.94
220833_at	---	---	3.67	3.49	3.57
216380_x_at	---	---	11.41	11.16	11.39
207624_s_at	retinitis pigmento	RPGR	8.15	8.28	8.64
216619_at	GTP-binding prote	GTPBP10	3.55	3.53	3.30
208716_s_at	transmembrane a	TMCO1	11.43	11.49	11.44
215500_at	sorting nexin 29	SNX29	4.84	4.96	4.81
206928_at	zinc finger proteir	ZNF124	5.95	6.16	5.84
210284_s_at	TGF-beta activate	TAB2	8.73	8.78	8.78
218524_at	E4F transcription	E4F1	7.79	7.80	7.80
212831_at	multiple EGF-like-	MEGF9	4.96	5.19	4.92
217988_at	cyclin B1 interacti	CCNB1IP1	11.54	11.34	11.63
207349_s_at	uncoupling protei	UCP3	4.53	4.30	3.80
222370_x_at	---	---	6.17	6.16	6.22
217188_s_at	chromosome 14 c	C14orf1	9.35	9.55	9.37
221358_at	neuropeptides B/	NPBWR2	3.59	3.74	3.61
209955_s_at	fibroblast activati	FAP	4.00	3.74	4.00
221719_s_at	leucine zipper, pu	LZTS1	4.04	3.79	4.27
210604_at	guanine nucleotid	GNAT2	4.00	4.00	3.80
205822_s_at	3-hydroxy-3-meth	HMGCS1	7.05	7.67	7.28
209780_at	putative homeodo	PHTF2	9.31	9.49	9.43
207868_at	cholinergic recept	CHRNA2	7.32	7.21	7.05
221707_s_at	vacuolar protein s	VPS53	4.52	4.83	4.90
214537_at	histone cluster 1,	HIST1H1D	6.15	6.28	5.95
221271_at	interleukin 21	IL21	4.81	4.80	4.92
215414_at	Phenylalanyl-tRN/	FARS2	5.10	4.81	5.01
210850_s_at	ELK1, member of	ELK1	4.28	4.17	3.98
206376_at	solute carrier fam	SLC6A15	4.48	4.23	4.07
205957_at	plexin B3	PLXNB3	5.10	4.94	4.79
214179_s_at	nuclear factor (er	NFE2L1	9.69	9.23	9.62
220540_at	potassium channe	KCNK15	3.51	3.56	3.50
212418_at	E74-like factor 1 (	ELF1	9.44	9.45	9.54
221677_s_at	downstream neigl	DONSON	9.52	9.65	9.80
200686_s_at	splicing factor, arg	SFRS11	11.00	11.12	11.31
218593_at	RNA binding moti	RBM28	9.68	9.83	9.82
216612_x_at	---	---	4.12	3.98	4.01
205735_s_at	AF4/FMR2 family,	AFF3	4.01	3.91	3.83
208724_s_at	RAB1A, member F	RAB1A	12.48	12.65	12.57
212792_at	dpy-19-like 1 (C. e	DPY19L1	10.07	10.16	10.18
218418_s_at	KN motif and anky	KANK2	9.50	9.50	9.49
212603_at	mitochondrial ribo	MRPS31	10.10	10.15	10.15
210097_s_at	nucleolar protein	NOL7	11.35	11.32	11.53
200643_at	high density lipop	HDLBP	8.52	8.52	8.67
60794_f_at	zinc finger proteir	ZNF814	4.67	4.78	4.72

201057_s_at	golgin B1	GOLGB1	8.17	8.41	8.54
221813_at	F-box protein 42	FBXO42	8.52	8.30	8.26
210189_at	heat shock 70kDa	HSPA1L	4.88	4.86	4.66
215688_at	Ras protein-specif	RASGRF1	5.68	5.78	5.49
214932_at	kinase D-interacti	KIDINS220	3.47	3.31	3.28
206191_at	ectonucleoside tri	ENTPD3	4.47	4.70	4.16
209886_s_at	SMAD family mem	SMAD6	6.27	6.41	6.01
204376_at	Vpr (HIV-1) bindin	VPRBP	4.97	4.99	5.16
206118_at	signal transducer	STAT4	4.92	4.78	4.80
215999_at	CMT1A duplicated	CDRT1	4.52	4.60	4.11
220600_at	chromosome 3 op	C3orf75	6.58	6.74	6.24
219959_at	molybdenum cofa	MOCOS	9.21	8.89	9.31
214483_s_at	ADP-ribosylation f	ARFIP1	5.52	5.44	6.13
217440_at	---	---	3.37	3.38	3.27
221129_at	chromosome 17 o	C17orf88	5.57	5.52	5.19
209943_at	F-box and leucine	FBXL4	8.50	8.48	8.78
205574_x_at	bone morphogene	BMP1	6.73	6.59	6.37
206211_at	selectin E	SELE	3.39	3.37	3.21
201003_x_at	arginyl aminopept	RNPEP /// TMEM1	9.71	9.74	9.72
38691_s_at	surfactant protein	SFTPC	7.65	7.63	7.31
215949_x_at	immunoglobulin h	IGHM /// LOC6524	7.16	7.08	7.35
209631_s_at	G protein-coupled	GPR37	4.47	4.12	3.84
219025_at	CD248 molecule, e	CD248	6.91	6.87	7.03
203260_at	HD domain contai	HDDC2	7.49	7.63	7.72
203493_s_at	centrosomal prote	CEP57	9.80	9.74	9.94
221004_s_at	integral membran	ITM2C	8.15	8.04	7.99
210868_s_at	ELOVL family mem	ELOVL6	8.71	9.10	9.20
204738_s_at	KRIT1, ankyrin rep	KRIT1	7.90	7.72	7.78
215288_at	transient receptor	TRPC2	6.29	6.33	5.76
214374_s_at	PTPRF interacting	PPFIBP1	7.75	7.73	7.66
221283_at	runt-related trans	RUNX2	5.26	5.27	5.24
212968_at	RFNG O-fucosylpe	RFNG	7.48	7.50	7.50
216996_s_at	FAST kinase doma	FASTKD2	9.73	9.61	9.81
209443_at	serpin peptidase i	SERPINA5	6.01	6.19	6.43
218872_at	tescalcin	TESC	5.99	5.95	5.91
220335_x_at	carboxylesterase 3	CES3	7.57	7.60	7.46
217615_at	---	---	6.04	6.24	6.26
218634_at	pleckstrin homolo	PHLDA3	7.49	7.64	7.72
220714_at	PR domain contai	PRDM14	5.86	6.09	5.78
205723_at	ciliary neurotroph	CNTFR	7.06	7.00	6.92
217198_x_at	immunoglobulin h	IGH@ /// IGHA2 //	6.83	6.67	6.36
210739_x_at	solute carrier fam	SLC4A4	5.17	4.99	4.62
202946_s_at	BTB (POZ) domain	BTBD3	10.08	10.10	10.28
204575_s_at	matrix metallopep	MMP19	6.78	7.08	7.04
202033_s_at	RB1-inducible coil	RB1CC1	9.17	9.31	9.46
217363_x_at	---	---	9.34	9.14	9.25
215266_at	dynein, axonemal	DNAH3	7.03	6.66	6.64

203546_at	importin 13	IPO13	7.22	7.28	7.23
200692_s_at	heat shock 70kDa	HSPA9	11.62	11.53	11.85
203628_at	insulin-like growtl	IGF1R	7.47	7.40	7.61
218233_s_at	prickle homolog 4	PRICKLE4 /// TOM	12.48	12.57	12.54
209675_s_at	heterogeneous nu	HNRNPUL1	7.87	7.82	7.82
204935_at	protein tyrosine p	PTPN2	5.06	5.02	5.39
203309_s_at	Hermansky-Pudla	HPS1	6.90	7.01	6.80
220850_at	MORC family CW-	MORC1	3.17	3.34	3.14
208933_s_at	lectin, galactoside	LGALS8	8.04	8.27	8.17
206198_s_at	carcinoembryonic	CEACAM7	3.20	3.37	3.17
215061_at	methyltransferase	METTL10	6.84	6.79	6.28
203069_at	synaptic vesicle gl	SV2A	7.18	7.41	7.25
211570_s_at	receptor-associat	RAPSN	5.14	5.10	4.87
219821_s_at	glucose-fructose c	GFOD1	8.42	8.29	8.60
216259_at	---	---	4.88	4.50	4.61
217527_s_at	nuclear factor of a	NFATC2IP	8.88	8.87	8.92
219462_at	transmembrane p	TMEM53	6.94	6.90	6.53
210795_s_at	hypothetical prot	LOC100130955	7.54	7.26	7.12
201452_at	Ras homolog enri	RHEB	7.05	6.62	6.97
211254_x_at	Rh-associated gly	RHAG	5.39	5.28	5.22
209292_at	Inhibitor of DNA b	ID4	3.12	3.07	2.86
221923_s_at	nucleophosmin (n	NPM1	12.63	12.75	12.82
209876_at	G protein-couplec	GIT2	6.62	6.92	6.82
218454_at	phospholipase B c	PLBD1	4.56	4.61	4.28
201024_x_at	eukaryotic transla	EIF5B	11.16	11.33	11.22
203461_at	chromodomain he	CHD2	4.40	4.36	3.96
221409_at	olfactory recepto	OR2S2	4.30	4.45	4.31
202300_at	hepatitis B virus x	HBXIP	11.80	11.78	11.84
208411_x_at	protein phosphata	PPEF2	3.44	3.28	3.17
207152_at	neurotrophic tyro	NTRK2	4.74	4.79	4.23
219087_at	asporin	ASPN	3.70	3.77	3.68
214891_at	F-box protein 21	FBXO21	5.83	5.83	5.87
211659_at	G protein-couplec	GPR135	5.62	5.34	5.56
200849_s_at	adenosylhomocys	AHCYL1	10.98	10.79	10.90
213733_at	myosin IF	MYO1F	5.87	6.20	5.71
AFFX-BioC-5_at	---	---	10.36	10.29	10.27
206430_at	caudal type home	CDX1	7.71	7.48	6.96
206483_at	leucine rich repea	LRRC6	5.34	5.34	4.96
206886_x_at	growth hormone	GH1	4.38	4.58	4.21
220931_at	hypothetical prot	MGC5590	4.18	4.18	4.32
211924_s_at	plasminogen activ	PLAUR	6.77	6.97	6.77
AFFX-r2-Bs-phe-	---	---	7.91	7.82	8.03
203807_x_at	chorionic somato	CSH2	4.08	4.04	3.73
219287_at	potassium large c	KCNMB4	5.30	5.51	5.50
207659_s_at	myelin-associated	MOBP	3.82	3.63	3.47
219414_at	calsyntenin 2	CLSTN2	4.11	3.94	4.44
205034_at	cyclin E2	CCNE2	7.34	8.04	7.94

220986_s_at	tigger transposabl	TIGD6	4.10	4.00	4.29
221339_at	olfactory receptor	OR10C1	4.17	4.38	4.21
201302_at	annexin A4	ANXA4	10.07	10.10	10.19
36545_s_at	Sfi1 homolog, spir	SFI1	7.45	7.43	7.23
220008_at	NKF3 kinase famil	SGK269	5.52	5.48	5.59
213399_x_at	ribophorin II	RPN2	11.99	11.97	12.08
204531_s_at	breast cancer 1, e	BRCA1	9.50	9.41	9.45
203689_s_at	fragile X mental re	FMR1	9.36	9.54	9.61
205816_at	integrin, beta 8	ITGB8	3.61	3.41	3.48
216652_s_at	down-regulator of	DR1	8.99	8.91	9.24
216655_s_at	---	---	5.60	6.12	5.89
213907_at	Eukaryotic transla	EEF1E1	6.95	7.13	7.14
206739_at	homeobox C5	HOXC5	7.25	7.34	7.33
221450_x_at	protocadherin bet	PCDHB13	6.90	6.77	6.63
200604_s_at	protein kinase, cA	PRKAR1A	7.51	7.52	7.65
214323_s_at	UPF3 regulator of	UPF3A	10.26	10.48	10.50
215834_x_at	scavenger recepto	SCARB1	6.55	6.31	6.05
209048_s_at	zinc finger, MYND	ZMYND8	10.04	10.03	10.07
211428_at	serpin peptidase i	SERPINA1	3.78	3.77	3.65
220855_at	---	---	4.17	4.03	3.97
200905_x_at	major histocompa	HLA-E	10.79	10.64	10.68
204788_s_at	protoporphyrinog	PPOX	8.04	8.02	8.04
212988_x_at	actin, gamma 1	ACTG1	14.21	14.23	14.08
209846_s_at	butyrophilin, subf	BTN3A2	8.69	8.75	8.77
202296_s_at	RER1 retention in	RER1	11.41	11.38	11.38
215875_at	---	---	6.73	6.65	6.34
220513_at	KH homology dom	KHDC1L	4.90	4.67	4.53
201370_s_at	cullin 3	CUL3	7.89	8.06	8.26
216239_at	Bardet-Biedl synd	BBS9	3.74	3.87	3.77
218601_at	upregulator of cel	URGCP	8.79	8.78	8.70
205121_at	sarcoglycan, beta	SGCB	3.70	3.95	3.90
207689_at	T-box 10	TBX10	3.62	3.72	3.47
210324_at	complement com	C8G	5.17	5.24	4.88
206402_s_at	neuropeptide FF-a	NPFF	7.35	7.30	7.01
209240_at	O-linked N-acetylg	OGT	10.99	11.04	10.97
219143_s_at	ribonuclease P/M	RPP25	5.84	5.54	5.24
221560_at	MAP/microtubule	MARK4	8.41	8.29	8.19
214493_s_at	InaD-like (Drosopl	INADL	5.90	5.78	5.42
204460_s_at	RAD1 homolog (S.	RAD1	9.93	9.87	10.12
211360_s_at	inositol 1,4,5-tripl	ITPR2	5.86	5.94	5.70
211341_at	POU class 4 home	POU4F1	4.21	4.19	3.79
218800_at	steroid 5 alpha-re	SRD5A3	3.53	3.74	3.77
221831_at	leucine zipper pro	LUZP1	7.49	7.67	7.62
222352_at	---	---	7.47	7.29	7.45
220381_at	Rho GTPase activa	ARHGAP28	4.02	3.88	3.56
220224_at	hydroxyacid oxida	HAO1	4.37	4.39	4.05
214970_s_at	ST6 beta-galactos	ST6GAL1	4.78	4.60	4.63

203911_at	RAP1 GTPase activ	RAP1GAP	5.79	5.71	5.45
220698_at	hypothetical prote	MGC4294	6.13	6.12	5.99
AFFX-M27830_I	---	---	11.69	11.63	11.53
212085_at	solute carrier fam	SLC25A6	12.95	12.81	13.02
207238_s_at	protein tyrosine p	PTPRC	3.52	3.54	3.50
207230_at	Cdon homolog (m	CDON	5.47	5.51	5.62
216005_at	Tenascin C	TNC	5.19	5.76	5.61
206614_at	growth differentia	GDF5	5.83	5.82	5.85
218268_at	TBC1 domain fam	TBC1D15	9.22	9.25	9.35
204124_at	solute carrier fam	SLC34A2	3.98	3.93	3.92
207873_x_at	seizure related 6 l	SEZ6L	5.71	5.82	5.75
203830_at	chromosome 17 c	C17orf75	8.59	8.61	8.72
216956_s_at	integrin, alpha 2b	ITGA2B	5.68	5.69	5.29
201706_s_at	peroxisomal bioge	PEX19	7.21	7.18	7.40
211958_at	insulin-like growt	IGFBP5	5.59	5.33	5.15
36742_at	tripartite motif-co	TRIM15	6.17	6.33	5.90
209929_s_at	inhibitor of kappa	IKBKG	8.99	9.07	8.85
217005_at	---	---	5.69	5.66	5.23
209188_x_at	down-regulator of	DR1	9.30	9.26	9.29
217673_x_at	GNAS complex loc	GNAS	9.03	9.01	9.12
205348_s_at	dynein, cytoplasm	DYNC1I1	5.23	5.22	4.92
212869_x_at	tumor protein, tra	TPT1	15.07	15.06	15.05
214555_at	somatostatin rece	SSTR5	6.27	6.10	6.06
220021_at	transmembrane c	TMC7	3.80	3.79	3.97
205653_at	cathepsin G	CTSG	7.57	7.32	7.34
215195_at	protein kinase C, a	PRKCA	7.82	7.79	7.95
218452_at	SWI/SNF related,	SMARCAL1	8.96	9.00	9.06
214137_at	Protein tyrosine p	PTPRJ	4.15	3.98	4.38
217122_s_at	similar to solute c	SLC35E2	10.03	9.89	10.08
204413_at	TNF receptor-asso	TRAF2	7.58	7.20	7.30
217738_at	nicotinamide pho	NAMPT	9.84	9.90	10.11
203747_at	aquaporin 3 (Gill b	AQP3	6.81	6.78	6.79
31861_at	immunoglobulin r	IGHMBP2	8.50	8.77	8.71
213167_s_at	solute carrier fam	SLC5A3	4.33	4.70	4.78
203364_s_at	KIAA0652	KIAA0652	7.54	7.68	7.61
220382_s_at	Rho GTPase activa	ARHGAP28	4.13	4.01	4.19
219703_at	meiosis-specific n	MNS1	7.60	7.95	7.98
204810_s_at	creatine kinase, m	CKM	4.60	4.55	4.64
212526_at	spastic paraplegia	SPG20	8.83	8.99	9.16
211060_x_at	glycosylphosphati	GPAA1	9.46	9.30	9.43
212138_at	PDS5, regulator of	PDS5A	10.85	10.92	11.02
219530_at	partner and locali	PALB2	9.77	9.73	9.79
221302_at	Kruppel-like facto	KLF15	6.09	6.19	6.18
213861_s_at	family with seque	FAM119B	7.88	7.75	8.01
221821_s_at	chromosome 12 c	C12orf41	9.59	9.57	9.75
219526_at	chromosome 14 c	C14orf169	8.66	8.68	8.78
207561_s_at	amiloride-sensitiv	ACCN3	4.32	4.17	4.24

206262_at	alcohol dehydroge	ADH1C	4.02	4.32	4.14
206773_at	lymphocyte antige	LY6H	6.42	6.35	6.23
205985_x_at	chloride channel I	CLCNKB	5.54	5.57	5.35
206998_x_at	proline-rich prote	PRB3	6.49	6.51	5.90
202415_s_at	HSPA (heat shock	HSPBP1	8.29	8.40	8.30
209033_s_at	dual-specificity ty	DYRK1A	10.05	9.94	10.05
221761_at	adenylosuccinate	ADSS	9.06	8.96	9.15
213933_at	prostaglandin E re	PTGER3	4.81	4.41	4.12
203882_at	interferon regulat	IRF9	7.51	7.28	7.12
215535_s_at	1-acylglycerol-3-p	AGPAT1	8.87	8.91	8.74
206525_at	gamma-aminobut	GABRR1	3.40	3.71	3.66
212889_x_at	growth arrest and	GADD45GIP1	6.23	6.04	5.76
213841_at	---	---	3.88	3.78	3.73
206815_at	sperm associated	SPAG8	4.62	4.18	4.23
209051_s_at	ral guanine nuclec	RALGDS	6.95	7.00	6.86
210443_x_at	opioid growth fac	OGFR	8.07	8.18	7.98
215161_at	calcium/calmodul	CAMK1G	4.94	5.06	5.04
204010_s_at	v-Ki-ras2 Kirsten r	KRAS	3.53	3.78	3.45
34063_at	RecQ protein-like	RECQL5	6.43	6.34	6.01
200832_s_at	stearoyl-CoA desa	SCD	11.93	12.06	12.10
217990_at	guanosine monop	GMPR2	9.76	9.79	9.82
207185_at	solute carrier fam	SLC10A1	4.79	4.75	4.71
216293_at	Clathrin, light chai	CLTA	8.01	7.97	7.91
209477_at	emerin	EMD	9.91	9.91	9.77
213949_s_at	Deoxyhypusine hy	DOHH	4.81	4.50	4.68
215843_s_at	tolloid-like 2	TLL2	4.67	4.60	4.39
214244_s_at	ATPase, H+ transp	ATP6V0E1	5.56	6.21	5.73
37986_at	erythropoietin rec	EPOR	6.97	6.91	6.86
208539_x_at	small proline-rich	SPRR2B	4.96	4.74	4.63
201634_s_at	cytochrome b5 ty	CYB5B	9.33	9.52	9.64
215935_at	family with seque	FAM75A1 /// FAM	4.74	4.69	4.59
206854_s_at	mitogen-activatec	MAP3K7	9.52	9.62	9.70
219150_s_at	ArfGAP with dual	ADAP1	7.93	7.93	7.44
205313_at	HNF1 homeobox I	HNF1B	5.77	5.51	5.59
207459_x_at	glycophorin B (MN	GYPB	6.85	6.59	6.32
217964_at	tetratricopeptide	TTC19	9.68	9.83	9.84
208481_at	ankyrin repeat an	ASB4	3.39	3.52	3.28
201436_at	eukaryotic transla	EIF4E	8.19	8.40	8.57
219545_at	potassium channe	KCTD14	8.08	8.12	8.10
214502_at	histone cluster 1,	HIST1H2BJ	4.33	4.40	4.38
206453_s_at	NDRG family mem	NDRG2	7.54	7.40	7.10
217693_x_at	mago-nashi homo	MAGOH2	7.00	6.87	6.99
219256_s_at	SH3 domain and t	SH3TC1	8.14	8.15	8.21
217958_at	trafficking protein	TRAPPC4	11.42	11.45	11.40
201082_s_at	dynactin 1 (p150,	DCTN1	6.30	6.09	6.20
217810_x_at	leucyl-tRNA synth	LARS	10.10	10.16	10.23
208514_at	potassium voltage	KCNE1	4.41	4.57	4.57

204752_x_at	poly (ADP-ribose)	PARP2	10.21	10.25	10.20
210661_at	glycine receptor, a	GLRA3	3.31	3.10	3.22
210110_x_at	heterogeneous nu	HNRNPH3	9.49	9.59	9.60
32540_at	---	---	3.90	4.02	4.03
205522_at	homeobox D4 ///	HOXD4 /// MIR10E	4.68	4.45	4.68
211597_s_at	HOP homeobox	HOPX	4.66	4.66	4.29
206662_at	glutaredoxin (thio	GLRX	9.04	9.13	9.12
213044_at	Rho-associated, co	ROCK1	10.49	10.70	10.78
213214_x_at	actin, gamma 1	ACTG1	14.22	14.29	14.19
205885_s_at	integrin, alpha 4 (a	ITGA4	3.32	3.43	3.15
210053_at	TAF5 RNA polyme	TAF5	8.73	8.73	8.83
215424_s_at	SNW domain cont	SNW1	9.96	9.92	10.02
210825_s_at	phosphatidylethar	PEBP1	12.06	12.02	12.10
209111_at	ring finger proteir	RNF5	8.84	8.77	8.98
91920_at	brevican	BCAN	7.34	7.44	7.33
210029_at	indoleamine 2,3-c	IDO1	6.16	6.44	6.22
208146_s_at	carboxypeptidase,	CPVL	3.80	3.72	3.88
209601_at	acyl-CoA oxidase 1	ACOX1	4.81	4.31	4.34
211738_x_at	chymotrypsin-like	CELA3A	5.47	5.57	5.32
201651_s_at	protein kinase C a	PACSIN2	11.04	10.98	11.04
219907_at	fibroblast growth	FRS3	6.33	6.25	6.26
205089_at	zinc finger proteir	ZNF7	8.42	8.50	8.36
207186_s_at	bromodomain PH	BPTF	9.72	9.97	9.69
210978_s_at	transgelin 2	TAGLN2	10.37	10.34	10.43
201977_s_at	KIAA0141	KIAA0141	7.12	7.35	7.22
202312_s_at	collagen, type I, al	COL1A1	6.68	6.32	6.27
206257_at	coiled-coil domair	CCDC9	7.35	7.45	7.12
218169_at	Vac14 homolog (S	VAC14	6.99	6.98	7.27
217674_at	---	---	4.08	3.88	3.95
215547_at	TSC22 domain fan	TSC22D2	4.79	4.47	4.36
215129_at	phosphoinositide-	PIK3C2G	3.35	3.62	3.46
204013_s_at	leucine carboxyl n	LCMT2	5.02	5.08	4.87
207147_at	distal-less homeol	DLX2	5.40	5.44	5.41
221170_at	histamine recepto	HRH4	3.35	3.27	3.36
215323_at	leucine zipper pro	LUZP2	5.34	5.49	5.30
208332_at	PTPN13-like, Y-lin	PRY	6.17	6.16	5.98
214650_x_at	myelin oligodendr	MOG	4.12	3.90	3.87
205936_s_at	hexokinase 3 (whi	HK3	7.08	6.97	6.85
216694_at	---	---	6.96	6.87	7.02
211463_at	Zic family membe	ZIC4	4.83	4.70	4.53
213896_x_at	family with seque	FAM149B1	5.71	5.44	5.83
204577_s_at	clusterin associate	CLUAP1	7.02	7.07	7.06
220316_at	neuronal PAS dom	NPAS3	4.31	4.35	4.32
219125_s_at	recombination act	RAG1AP1	8.62	8.34	8.50
200805_at	lectin, mannose-b	LMAN2	9.78	9.70	9.77
209718_at	non-SMC condens	NCAPH2	7.63	7.49	7.30
222083_at	glycine-N-acyltran	GLYAT	5.86	5.95	5.56

211651_s_at	laminin, beta 1	LAMB1	8.62	8.90	8.77
216378_at	---	---	3.50	3.34	3.33
210629_x_at	leukocyte specific	LST1	4.86	5.13	4.85
213795_s_at	protein tyrosine p	PTPRA	7.59	7.78	7.59
210867_at	CCR4-NOT transcr	CNOT4	4.68	4.48	4.75
201326_at	chaperonin conta	CCT6A	11.32	11.36	11.36
205913_at	perilipin 1	PLIN1	5.32	5.34	5.03
218278_at	WD repeat domai	WDR74	9.78	9.36	9.71
216301_at	hypothetical prot	LOC100287927	5.00	4.89	4.73
396_f_at	pecanex-like 2 (Dr	PCNXL2	7.58	7.85	8.13
214069_at	acyl-CoA syntheta	ACSM2A /// ACSM	5.00	5.13	5.02
216342_x_at	---	---	13.89	13.86	13.88
213919_at	DnaJ (Hsp40) hom	DNAJC4	6.17	6.00	5.68
219602_s_at	family with seque	FAM38B	4.04	3.67	3.83
208884_s_at	ubiquitin protein l	UBR5	9.14	9.11	9.20
217792_at	sorting nexin 5	SNX5	10.98	10.93	11.05
221421_s_at	ADAM metallope	ADAMTS12	5.47	5.25	4.99
220423_at	phospholipase A2	PLA2G2D	4.53	4.82	4.27
221061_at	polycystic kidney	PKD2L1	8.02	7.98	7.73
208292_at	bone morphogen	BMP10	5.14	5.47	5.11
214595_at	potassium voltage	KCNG1	8.90	8.57	9.03
217645_at	COX16 cytochrom	COX16	7.03	7.41	7.30
207202_s_at	nuclear receptor s	NR1I2	4.22	3.85	3.55
205183_at	heterogeneous nu	HNRNPD	4.65	4.39	4.23
221772_s_at	protein phosphata	PPP2R2D	7.70	7.60	7.62
208463_at	gamma-aminobut	GABRA4	4.44	4.44	4.18
219750_at	transmembrane p	TMEM144	3.76	3.95	3.75
201175_at	thioredoxin-relate	TMX2	11.05	11.01	11.15
213606_s_at	Rho GDP dissociat	ARHGDIA	7.09	7.02	6.95
218218_at	adaptor protein, p	APPL2	4.87	5.33	5.25
203494_s_at	centrosomal prot	CEP57	10.21	10.14	10.45
216073_at	ankyrin repeat do	ANKRD34C	3.68	3.68	3.18
205251_at	period homolog 2	PER2	7.75	7.80	7.94
210466_s_at	SERPINE1 mRNA b	SERBP1	12.35	12.37	12.55
206590_x_at	dopamine recept	DRD2	6.85	6.80	6.39
214862_x_at	---	---	3.29	3.72	3.45
207725_at	POU class 4 home	POU4F2	3.73	3.51	3.57
217085_at	---	---	3.41	3.39	3.02
211464_x_at	caspase 6, apopto	CASP6	8.22	8.29	7.94
214328_s_at	heat shock protei	HSP90AA1	13.95	14.12	14.09
202974_at	membrane protei	MPP1	8.93	8.95	8.95
208902_s_at	ribosomal protein	RPS28	6.51	6.94	6.82
206167_s_at	Rho GTPase activ	ARHGAP6	3.60	3.47	3.23
206107_at	regulator of G-pro	RGS11	5.15	5.04	5.11
214818_at	coiled-coil domai	CCDC57	5.75	5.68	5.78
207020_at	heat shock transc	HSF2BP	5.63	5.57	5.58
212363_x_at	actin, gamma 1	ACTG1	14.07	14.13	13.95

200063_s_at	nucleophosmin (n	NPM1	14.21	14.26	14.37
212855_at	DCN1, defective ir	DCUN1D4	9.19	9.37	9.41
203774_at	5-methyltetrahyd	MTR	9.15	9.19	9.47
201460_at	mitogen-activatec	MAPKAPK2	9.49	9.29	9.39
215151_at	dedicator of cytok	DOCK10	4.83	5.08	4.97
213273_at	odz, odd Oz/ten-n	ODZ4	4.60	4.56	4.55
213346_at	chromosome 13 c	C13orf27	8.31	8.36	8.43
222059_at	zinc finger protein	ZNF335	6.46	6.14	5.99
206550_s_at	nucleoporin 155kl	NUP155	10.27	10.18	10.33
203773_x_at	biliverdin reducta:	BLVRA	11.03	11.06	11.03
209435_s_at	Rho/Rac guanine	ARHGEF2	10.96	10.40	10.87
217861_s_at	prolactin regulato	PREB	8.85	8.97	8.92
219156_at	synaptojanin 2 bir	SYNJ2BP	8.47	8.69	8.63
222350_at	---	---	4.07	3.80	3.94
216947_at	hypothetical prote	LOC100288941	3.22	3.01	3.08
AFFX-LysX-M_at	---	---	7.52	7.44	7.47
200078_s_at	ATPase, H+ transp	ATP6V0B	11.30	11.19	11.13
215154_at	unc-51-like kinase	ULK2	5.24	5.34	4.75
203804_s_at	LUC7-like 3 (S. cer	LUC7L3	10.37	10.59	10.62
207016_s_at	aldehyde dehydro	ALDH1A2	4.04	3.94	4.22
200883_at	ubiquinol-cytochr	UQCRC2	11.04	11.17	11.24
219710_at	SH3 domain and t	SH3TC2	4.37	4.35	4.43
216128_at	tubulin folding co	TBCD	6.95	6.84	6.92
210828_s_at	aryl hydrocarbon	ARNT	5.23	5.11	4.82
219547_at	COX15 homolog, (	COX15	9.13	8.99	9.01
207313_x_at	killer cell immuno	KIR3DL1 /// KIR3D	6.00	5.97	5.85
208823_s_at	cyclin-dependent	CDK16	8.25	8.03	8.13
216333_x_at	tenascin XA pseuc	TNXA /// TNXB	6.53	7.07	6.87
206781_at	DnaJ (Hsp40) hom	DNAJC4	7.40	7.24	7.19
217456_x_at	major histocompa	HLA-E	10.20	10.14	10.05
34221_at	HMG box domain	HMGXB3	7.96	8.08	7.98
632_at	glycogen synthase	GSK3A	8.04	8.13	7.87
220732_at	phosphatidylinosi	PREX2	4.17	4.08	4.06
221317_x_at	protocadherin bet	PCDHB6	5.78	5.56	5.58
201659_s_at	ADP-ribosylation f	ARL1	9.12	9.32	9.28
213759_at	---	---	5.97	5.93	5.81
220496_at	C-type lectin dom	CLEC1B	5.90	5.66	5.57
214590_s_at	ubiquitin-conjugat	UBE2D1	6.12	6.60	6.61
204357_s_at	LIM domain kinas	LIMK1	7.47	7.41	7.52
210362_x_at	promyelocytic leu	PML	6.04	6.13	5.89
207491_at	monoacylglycerol	MOGAT2	6.94	6.76	6.56
219003_s_at	mannosidase, enc	MANEA	7.04	7.17	7.26
220087_at	beta-carotene 15,	BCMO1	4.04	3.74	3.81
219984_s_at	HRAS-like suppres	HRASLS	3.44	3.43	3.41
202927_at	peptidylprolyl cis/	PIN1	9.11	9.04	9.07
208286_x_at	POU class 5 home	POU5F1 /// POU5I	7.23	7.16	7.28
202372_at	RAB3 GTPase acti	RAB3GAP2	8.82	9.02	9.18

AFFX-LysX-3_at	---	---	8.13	8.07	8.03
219176_at	chromosome 2 op	C2orf47	9.55	9.71	9.67
204020_at	purine-rich eleme	PURA	9.83	9.94	9.90
212750_at	protein phosphata	PPP1R16B	4.00	3.90	3.65
201211_s_at	DEAD (Asp-Glu-Ala	DDX3X	7.50	7.76	7.57
207408_at	solute carrier fam	SLC22A14	8.02	8.11	7.69
210822_at	ribosomal protein	RPL13P5	6.97	7.32	7.17
202500_at	DnaJ (Hsp40) hom	DNAJB2	6.97	7.11	6.97
213177_at	mitogen-activated	MAPK8IP3	7.16	6.76	7.16
212455_at	YTH domain conta	YTHDC1	10.70	10.66	10.70
205314_x_at	syntrophin, beta 2	SNTB2	6.85	6.44	6.41
200768_s_at	methionine adeno	MAT2A	11.70	11.87	11.66
212529_at	LSM12 homolog (S	LSM12	8.23	8.16	8.26
AFFX-LysX-5_at	---	---	6.91	6.96	7.01
210636_at	peroxisome prolif	PPARD	5.11	5.20	5.03
221275_s_at	---	---	5.35	5.75	5.33
215242_at	phosphatidylinosi	PIGC	4.04	3.77	4.03
218991_at	HEAT repeat conta	HEATR6	9.05	8.88	9.10
213095_x_at	allograft inflamma	AIF1	7.29	7.21	6.85
40420_at	serine/threonine k	STK10	7.75	7.76	7.76
206636_at	RAS p21 protein a	RASA2	5.15	5.59	4.92
216592_at	melanoma antigen	MAGEC3	4.42	4.01	4.16
202247_s_at	metastasis associa	MTA1	6.13	6.01	5.89
211489_at	adrenergic, alpha-	ADRA1A	3.76	3.51	3.62
216502_at	interferon stimula	ISG20L2	3.87	3.86	3.65
209029_at	COP9 constitutive	COPS7A	9.41	9.39	9.61
212576_at	mahogunin, ring f	MGRN1	9.26	9.18	8.93
202623_at	E2F-associated ph	EAPP	10.42	10.28	10.39
217961_at	solute carrier fam	SLC25A38	8.96	8.98	9.14
206255_at	B lymphoid tyrosi	BLK	5.67	5.76	5.56
203700_s_at	deiodinase, iodoth	DIO2	6.94	7.34	6.97
206535_at	solute carrier fam	SLC2A2	3.48	3.28	3.35
217551_at	similar to olfactor	LOC441453	3.24	3.21	3.47
215678_at	---	---	6.49	6.14	6.14
213150_at	homeobox A10	HOXA10	7.84	7.64	7.88
48117_at	coiled-coil domain	CCDC101	7.99	7.65	7.95
210356_x_at	membrane-spann	MS4A1	4.30	4.32	4.17
203540_at	glial fibrillary acidi	GFAP	4.70	4.45	4.59
202799_at	ClpP caseinolytic p	CLPP	10.41	10.28	10.42
220506_at	guanylate cyclase	GUCY1B2	4.87	4.74	4.71
207743_at	---	---	6.69	6.81	6.48
207403_at	insulin receptor su	IRS4	5.22	5.21	5.33
215172_at	protein tyrosine p	PTPN20A /// PTPN	3.24	3.41	3.28
202395_at	N-ethylmaleimide	NSF	10.20	10.28	10.10
218486_at	Kruppel-like facto	KLF11	7.95	7.58	7.92
206930_at	glycine-N-acyltran	GLYAT	3.66	3.65	3.41
208311_at	G protein-coupled	GPR50	4.17	3.90	3.95

205754_at	coagulation factor	F2	6.57	6.19	6.08
211358_s_at	CDKN1A interactin	CIZ1	6.81	6.87	6.71
202168_at	TAF9 RNA polyme	TAF9	11.29	11.48	11.53
211219_s_at	LIM homeobox 2	LHX2	4.86	5.21	4.93
221202_at	similar to PRO232	LOC100293871	3.75	3.74	3.43
220376_at	leucine rich repea	LRRC19	3.26	2.94	3.13
220456_at	serine palmitoyltr	SPTLC3	3.74	4.12	3.93
212242_at	tubulin, alpha 4a	TUBA4A	11.04	11.23	11.16
203015_s_at	synovial sarcoma,	SSX2IP	6.87	6.68	6.81
208777_s_at	proteasome (pros	PSMD11	11.23	11.21	11.17
205946_at	vasoactive intestin	VIPR2	7.74	7.51	7.29
213466_at	RAB40C, member	RAB40C	7.56	7.57	7.25
211147_s_at	purinergic recepto	P2RX6	5.28	5.36	5.16
217732_s_at	integral membran	ITM2B	10.68	10.68	10.88
221883_at	PBX/knotted 1 ho	PKNOX1	7.83	7.93	7.76
218864_at	tensin 1	TNS1	6.57	6.21	5.81
212117_at	ras homolog gene	RHOQ	10.07	10.10	10.07
206441_s_at	COMM domain co	COMMD4	10.49	10.45	10.26
209108_at	tetraspanin 6	TSPAN6	7.40	7.41	7.39
208916_at	solute carrier fam	SLC1A5	8.70	8.49	8.66
218431_at	VPS33B interactin	VIPAR	8.04	8.05	8.01
217396_at	---	---	4.99	4.92	4.94
208252_s_at	carbohydrate (cho	CHST3	5.57	5.76	5.31
208151_x_at	DEAD (Asp-Glu-Al	DDX17	6.41	6.38	6.18
AFFX-r2-Ec-bioD	---	---	12.35	12.33	12.42
217065_at	T cell receptor del	TRD@	3.72	3.75	3.52
202428_x_at	diazepam binding	DBI	12.61	12.71	12.67
201758_at	tumor susceptibili	TSG101	10.91	10.84	10.94
207409_at	leukocyte cell-der	LECT2	3.87	3.92	4.12
216374_at	---	---	4.27	4.32	4.17
53071_s_at	chromosome 17 o	C17orf101	9.03	8.88	9.18
211551_at	epidermal growth	EGFR	5.19	5.23	5.13
220196_at	mucin 16, cell surf	MUC16	3.99	4.08	4.01
216838_at	hypothetical LOC9	LOC92249	4.65	4.86	4.69
211652_s_at	lipopolysaccharide	LBP	5.47	5.41	5.27
205302_at	insulin-like growth	IGFBP1	3.52	3.46	3.27
217855_x_at	stromal cell derive	SDF4	9.52	9.48	9.40
210129_s_at	tubulin tyrosine lig	TTLL3	5.47	5.04	5.21
219730_at	mediator complex	MED18	5.86	5.98	5.71
202604_x_at	ADAM metallopep	ADAM10	7.11	7.11	7.05
212267_at	wings apart-like h	WAPAL	9.54	9.64	9.74
206265_s_at	glycosylphosphati	GPLD1	4.58	4.89	4.47
212353_at	sulfatase 1	SULF1	3.93	4.33	3.88
211314_at	calcium channel, v	CACNA1G	5.86	5.42	5.61
81811_at	---	---	7.82	7.95	7.88
211455_at	---	---	6.71	6.36	6.44
200967_at	peptidylprolyl isor	PPIB	12.89	12.90	12.87

217477_at	phosphatidylinosi	PIP5K1B	3.78	3.90	3.70
219252_s_at	gem (nuclear orga	GEMIN8	6.28	6.19	6.06
220034_at	interleukin-1 rece	IRAK3	3.83	3.65	3.45
207054_at	interphotorecept	IMPG1	3.08	3.22	3.12
206494_s_at	integrin, alpha 2b	ITGA2B	4.84	4.92	4.84
221085_at	tumor necrosis fa	TNFSF15	3.32	3.34	3.15
210997_at	hepatocyte growt	HGF	3.43	3.16	3.36
206984_s_at	Ras-like without C	RIT2	3.12	3.20	3.17
208038_at	interleukin 1 rece	IL1RL2	4.18	4.29	4.12
218671_s_at	ATPase inhibitory	ATPIF1	10.35	10.26	10.29
205259_at	nuclear receptor s	NR3C2	8.96	8.89	9.16
217591_at	---	---	5.99	5.81	5.84
219177_at	BRX1, biogenesis	BRIX1	8.53	8.52	8.66
210699_at	---	---	4.55	4.22	4.06
202297_s_at	RER1 retention in	RER1	10.68	10.65	10.76
204753_s_at	hepatic leukemia	HLF	5.28	4.49	4.77
219736_at	tripartite motif-co	TRIM36	5.83	5.90	6.07
220844_at	transcription elon	TCEB3B	3.87	3.73	3.73
209701_at	endoplasmic retic	ERAP1	7.07	6.95	7.09
221401_at	calcium channel, v	CACNG5	5.90	5.71	5.33
206274_s_at	ciliary rootlet coil	CROCC	6.97	6.78	6.45
52005_at	widely interspace	WIZ	8.49	8.45	8.54
221408_x_at	protocadherin bet	PCDHB12	5.84	5.95	5.78
203822_s_at	E74-like factor 2 (	ELF2	9.21	9.18	9.25
211756_at	parathyroid horm	PTHLH	3.61	3.34	3.25
211970_x_at	actin, gamma 1	ACTG1	14.32	14.35	14.19
216244_at	interleukin 1 rece	IL1RN	3.60	3.18	3.24
222080_s_at	Sirtuin (silent mat	SIRT5	7.03	6.95	6.99
205159_at	colony stimulating	CSF2RB	3.63	3.47	3.65
211731_x_at	synovial sarcoma,	SSX3	5.66	5.45	5.67
203447_at	proteasome (pros	PSMD5	10.00	10.12	10.16
220257_x_at	nuclear RNA expo	NXF2 /// NXF2B	5.31	5.47	5.70
215435_at	---	---	5.33	5.56	5.32
222141_at	kelch-like 22 (Dro	KLHL22	4.09	4.21	3.73
212679_at	transducin (beta)-	TBL2	5.07	5.24	4.26
210081_at	advanced glycosyl	AGER	5.58	5.58	5.27
220362_at	psoriasis susceptil	PSORS1C1	5.34	5.13	5.14
221285_at	ST8 alpha-N-acety	ST8SIA2	4.05	4.00	3.89
216037_x_at	transcription fact	TCF7L2	8.45	8.26	8.57
221639_x_at	hypothetical LOC1	LOC100129656	5.97	5.36	5.68
35160_at	LIM domain bindi	LDB1	7.13	7.02	6.87
206178_at	phospholipase A2	PLA2G5	4.82	4.65	4.65
215350_at	spectrin repeat co	SYNE1	3.61	3.43	3.39
219805_at	chromosome X op	CXorf56	6.99	7.04	7.21
208379_x_at	neuropeptide Y re	NPY2R	4.83	4.52	4.27
222112_at	epidermal growth	EPS15L1	4.70	4.34	4.22
201550_x_at	actin, gamma 1	ACTG1	14.34	14.36	14.23

213080_x_at	ribosomal protein	RPL5	14.70	14.69	14.70
205516_x_at	CDKN1A interactir	CIZ1	7.40	7.30	7.38
215964_at	---	---	3.72	3.50	3.14
205774_at	coagulation factor	F12	8.50	8.24	8.45
202703_at	dual specificity ph	DUSP11	10.51	10.44	10.66
216963_s_at	growth associatec	GAP43	6.18	5.84	5.50
211032_at	COBL-like 1	COBLL1	3.46	3.41	3.42
221140_s_at	G protein-couplec	GPR132	5.29	5.40	5.28
218276_s_at	salvador homolog	SAV1	7.79	7.86	8.01
205536_at	vav 2 guanine nuc	VAV2	7.00	7.08	7.01
220097_s_at	transmembrane p	TMEM104	7.21	7.03	7.14
208684_at	coatomer protein	COPA	10.16	10.28	10.31
203907_s_at	IQ motif and Sec7	IQSEC1	7.45	7.26	7.18
221953_s_at	hypothetical LOC7	LOC729580	8.82	8.67	8.76
205150_s_at	TLR4 interactor w	TRIL	4.07	3.96	3.60
214685_at	chromosome 4 op	C4orf10	3.94	3.49	3.53
206374_at	dual specificity ph	DUSP8	6.81	7.10	6.92
215786_at	---	---	5.03	5.23	4.99
210883_x_at	ephrin-B3	EFNB3	7.19	6.95	6.68
204760_s_at	nuclear receptor s	NR1D1 /// THRA	6.75	6.41	6.41
213473_at	BRCA1 associated	BRAP	8.71	8.65	8.81
201131_s_at	cadherin 1, type 1	CDH1	4.00	4.11	3.83
209103_s_at	ubiquitin fusion de	UFD1L	11.68	11.65	11.56
210414_at	fibronectin leucin	FLRT1	6.34	6.21	6.16
214183_s_at	transketolase-like	TKTL1	4.23	4.06	3.89
220179_at	dipeptidase 3	DPEP3	6.15	5.71	5.86
206729_at	tumor necrosis fa	TNFRSF8	6.20	6.13	6.16
205135_s_at	nuclear fragile X n	NUFIP1	9.25	9.14	9.34
204336_s_at	regulator of G-prc	RGS19	8.18	8.08	8.08
216723_at	---	---	4.03	3.53	3.22
220239_at	kelch-like 7 (Drosc	KLHL7	8.17	8.18	8.28
210045_at	isocitrate dehydrc	IDH2	7.00	7.19	6.80
219905_at	erythroblast mem	ERMAP	7.36	7.42	7.39
208101_s_at	ubiquitin related r	URM1	8.45	8.34	8.39
210706_s_at	ring finger proteir	RNF24	7.12	7.09	7.24
209854_s_at	kallikrein-related	KLK2	4.90	4.79	4.81
36564_at	ring finger proteir	RNF19B	6.81	6.99	6.77
210230_at	---	---	7.82	7.83	7.83
212837_at	family with seque	FAM175B	9.60	9.69	9.71
218134_s_at	RNA binding moti	RBM22	9.98	9.86	9.97
211889_x_at	carcinoembryonic	CEACAM1	4.30	4.69	4.35
202666_s_at	actin-like 6A	ACTL6A	10.00	10.07	10.37
212957_s_at	hypothetical LOC9	LOC92249	6.43	6.63	6.48
206103_at	ras-related C3 bot	RAC3	6.93	6.75	6.37
210724_at	egf-like module cc	EMR3	3.64	3.93	4.14
212370_x_at	family with seque	FAM21A /// FAM2	9.02	9.15	9.13
211381_x_at	sperm associated	SPAG11B	4.12	4.48	3.90

209889_at	SEC31 homolog B	SEC31B	6.51	6.44	6.21
202374_s_at	RAB3 GTPase acti	RAB3GAP2	8.26	8.42	8.28
209358_at	TAF11 RNA polym	TAF11	9.75	9.89	9.85
221582_at	histone cluster 3,	HIST3H2A	9.21	8.98	9.10
218406_x_at	hypothetical prot	LOC100287008	5.11	5.11	4.97
202810_at	integrator comple	INTS3	10.72	10.83	10.76
206730_at	glutamate recept	GRIA3	3.71	3.66	3.60
214157_at	GNAS complex lo	GNAS	4.91	4.83	4.78
220369_at	SMEK homolog 1,	SMEK1	4.33	4.15	4.37
207697_x_at	leukocyte immun	LILRB2	6.13	5.95	5.86
31846_at	ras homolog gene	RHOD	8.49	8.64	8.41
206227_at	cartilage interme	CILP	5.78	5.93	5.41
220058_at	chromosome 17 c	C17orf39	6.27	6.05	5.97
211946_s_at	HLA-B associated	BAT2L2	11.44	11.44	11.47
212004_at	chromosome 1 op	C1orf144	8.95	9.06	9.13
212826_s_at	solute carrier fam	SLC25A6	13.09	12.96	13.05
209317_at	polymerase (RNA)	POLR1C	9.30	9.19	9.30
220776_at	potassium inward	KCNJ14	6.18	6.35	6.21
203915_at	chemokine (C-X-C	CXCL9	4.79	4.74	4.59
216680_s_at	EPH receptor B4	EPHB4	7.59	7.56	7.16
220012_at	ERO1-like beta (S.	ERO1LB	5.09	5.39	4.92
216220_s_at	adenosine A1 rec	ADORA1	7.24	7.13	6.90
202964_s_at	regulatory factor	RFX5	7.71	7.80	7.96
211579_at	integrin, beta 3 (p	ITGB3	3.89	3.81	3.57
216057_at	RAB3 GTPase acti	RAB3GAP2	4.50	4.55	4.36
211771_s_at	POU class 2 home	POU2F2	5.68	5.43	5.14
204699_s_at	chromosome 1 op	C1orf107	9.24	9.10	9.20
208880_s_at	PRP6 pre-mRNA p	PRPF6	9.11	8.99	9.01
207752_x_at	proline-rich prote	PRB1	7.40	7.38	6.94
212193_s_at	La ribonucleoprot	LARP1	7.64	7.73	7.65
208267_at	transient recepto	TRPV5	6.53	6.04	6.50
218441_s_at	RNA polymerase I	RPAP1	8.25	8.38	8.21
217002_s_at	5-hydroxytryptam	HTR3A	5.11	4.95	5.00
204687_at	prostate androge	PARM1	6.92	6.87	6.38
207651_at	G protein-couple	GPR171	3.63	3.38	3.28
200043_at	enhancer of rudin	ERH	12.62	12.66	12.64
205699_at	mitogen-activate	MAP2K6	3.83	4.09	3.76
207869_s_at	calcium channel,	CACNA1G	3.29	3.35	3.17
215601_at	---	---	3.63	3.41	3.41
206566_at	solute carrier fam	SLC7A1	4.77	4.20	4.78
220171_x_at	KIAA1704	KIAA1704	8.09	8.19	8.35
221632_s_at	WD repeat domai	WDR4	7.91	7.94	7.73
205160_at	Peroxisomal biog	PEX11A	7.02	7.28	7.02
217114_at	similar to U5 snR	LOC652147	4.75	4.75	4.58
208774_at	casein kinase 1, d	CSNK1D	9.67	9.58	9.52
219381_at	chromosome 5 op	C5orf42	5.38	5.33	5.37
221702_s_at	TM2 domain cont	TM2D3	10.82	10.80	10.85

215437_x_at	bromodomain adj	BAZ2A	4.41	4.58	4.51
205321_at	eukaryotic transla	EIF2S3	11.25	11.11	11.22
210740_s_at	inositol 1,3,4-tripl	ITPK1	10.42	10.27	10.29
209182_s_at	chromosome 10 c	C10orf10	7.07	7.01	6.85
207711_at	chromosome 20 c	C20orf117	6.84	7.06	6.68
209331_s_at	MYC associated fa	MAX	8.31	8.32	8.44
216113_at	Abl-interactor 2	ABI2	3.77	3.78	3.44
221851_at	DDB1 and CUL4 as	DCAF15	5.91	5.58	5.55
211046_at	potassium voltage	KCNH6	3.56	3.56	3.54
208936_x_at	lectin, galactoside	LGALS8	7.92	8.05	8.01
205616_at	potassium voltage	KCNA6	5.90	5.80	5.67
214421_x_at	cytochrome P450,	CYP2C9	4.57	4.06	4.09
205906_at	forkhead box J1	FOXJ1	7.19	6.83	6.80
218135_at	ERGIC and golgi 2	ERGIC2	10.64	10.75	10.74
208018_s_at	hemopoietic cell k	HCK	4.98	5.07	4.81
215101_s_at	chemokine (C-X-C	CXCL5	3.80	3.60	3.47
211232_x_at	glucagon-like pep	GLP1R	5.88	5.96	5.90
213968_at	---	---	4.05	3.65	3.62
208304_at	chemokine (C-C m	CCR3	4.42	4.53	4.16
222228_s_at	alkB, alkylation re	ALKBH4	7.30	7.08	7.15
211819_s_at	sorbin and SH3 dc	SORBS1	5.46	5.65	5.24
201545_s_at	poly(A) binding pr	PABPN1	8.86	8.91	8.98
202068_s_at	low density lipopr	LDLR	10.05	10.49	10.07
220010_at	KCNE1-like	KCNE1L	4.79	4.88	5.23
209999_x_at	suppressor of cyto	SOCS1	6.03	5.83	5.64
218541_s_at	chromosome 8 op	C8orf4	3.04	3.08	3.10
207902_at	interleukin 5 rece	IL5RA	3.48	3.56	3.21
206452_x_at	protein phosphata	PPP2R4	7.33	7.34	7.33
216658_at	---	---	5.87	5.92	5.71
204587_at	solute carrier fam	SLC25A14	8.20	8.18	8.37
208211_s_at	anaplastic lymphc	ALK	5.77	5.55	5.14
211921_x_at	prothymosin, alph	PTMA	13.88	13.93	13.97
203149_at	poliovirus recepto	PVRL2	8.11	7.77	7.86
219028_at	homeodomain int	HIPK2	8.11	7.90	8.11
213383_at	SET binding factor	SBF1	7.07	7.10	7.09
206938_at	steroid-5-alpha-re	SRD5A2	5.46	5.20	5.21
217049_x_at	protocadherin 11	PCDH11Y	3.28	3.28	3.39
213493_at	sushi, nidogen and	SNED1	4.06	3.86	4.11
214409_at	RFPL3 antisense R	RFPL3S	3.59	3.57	3.49
203679_at	transmembrane e	TMED1	9.42	9.22	9.12
211712_s_at	annexin A9	ANXA9	3.86	3.94	3.87
217300_at	---	---	4.88	4.95	4.92
202218_s_at	fatty acid desatur	FADS2	7.77	7.86	7.68
210523_at	bone morphogene	BMPR1B	4.00	4.34	4.06
221693_s_at	mitochondrial ribo	MRPS18A	9.42	9.35	9.38
204959_at	myeloid cell nucle	MNDA	3.62	3.25	3.26
204409_s_at	eukaryotic transla	EIF1AY	3.58	3.25	3.39

222285_at	immunoglobulin h	IGHD	6.42	6.06	6.29
206381_at	sodium channel, v	SCN2A	4.53	4.55	4.46
221318_at	neurogenic differe	NEUROD4	5.53	5.37	4.93
210376_x_at	ELK1, member of	ELK1	6.24	6.20	6.24
215611_at	transcription facto	TCF12	4.97	5.22	4.83
213027_at	TROVE domain fa	TROVE2	9.16	9.25	9.40
211164_at	EPH receptor A3	EPHA3	3.44	3.52	3.37
200724_at	ribosomal protein	RPL10	5.75	5.95	5.81
215371_at	mediator complex	MED27	5.47	5.45	5.24
221585_at	calcium channel, v	CACNG4	6.05	5.90	5.41
89476_r_at	aminopeptidase-li	NPEPL1	7.65	7.61	7.41
208255_s_at	FK506 binding pro	FKBP8	7.05	6.85	6.94
209195_s_at	adenylate cyclase	ADCY6	7.78	7.55	7.64
214087_s_at	myosin binding pr	MYBPC1	4.52	4.44	4.30
220562_at	cytochrome P450,	CYP2W1	6.72	6.84	6.72
202868_s_at	DnaJ (Hsp40) hom	DNAJB12	10.39	10.50	10.43
216641_s_at	ladinin 1	LAD1	7.27	7.16	6.96
204579_at	fibroblast growth	FGFR4	5.41	5.21	5.44
207670_at	keratin 85	KRT85	6.38	6.40	6.50
220541_at	matrix metallopep	MMP26	4.47	4.62	4.31
203572_s_at	TAF6 RNA polyme	TAF6	9.43	9.26	9.27
204742_s_at	PDS5, regulator of	PDS5B	7.60	7.66	7.92
220472_at	zinc finger, CCHC	ZCCHC4	4.99	4.83	4.95
206606_at	lipase, hepatic	LIPC	4.76	4.62	4.23
220586_at	chromodomain he	CHD9	3.50	3.49	3.70
218334_at	THO complex 7 ho	THOC7	11.02	11.06	11.30
213066_at	RUN and SH3 dom	RUSC2	8.91	8.54	8.68
49049_at	deltex homolog 3	DTX3	6.34	6.14	6.20
202752_x_at	solute carrier fam	SLC7A8	7.49	7.60	7.54
208925_at	claudin domain co	CLDND1	9.27	9.22	9.30
210408_s_at	copine VI (neuron	CPNE6	5.04	4.99	4.97
215308_at	X-ray repair comp	XRCC6	4.77	4.77	4.56
216269_s_at	elastin	ELN	6.59	6.25	6.15
215917_at	RAD21-like 1 (S. p	RAD21L1	3.48	3.67	3.59
217842_at	LUC7-like 2 (S. cer	LUC7L2	9.34	9.21	9.39
217176_s_at	zinc finger protein	ZFX	5.67	6.10	5.84
210614_at	tocopherol (alpha	TTPA	3.71	3.49	3.59
209706_at	NK3 homeobox 1	NKX3-1	7.78	7.75	7.87
219468_s_at	CUE domain conta	CUEDC1	5.74	6.15	5.93
211180_x_at	runt-related trans	RUNX1	6.89	6.43	6.61
208907_s_at	mitochondrial ribo	MRPS18B	10.24	10.09	10.38
216588_at	ribosomal protein	RPL7	3.48	3.28	3.44
200623_s_at	calmodulin 3 (pho	CALM3	9.04	9.00	9.04
204665_at	suppressor of IKBI	SIKE1	4.67	4.91	4.79
205347_s_at	thymosin beta 15a	TMSB15A	3.59	3.72	3.49
210813_s_at	X-ray repair comp	XRCC4	7.48	7.48	7.65
209068_at	heterogeneous nu	HNRPDL	9.29	9.34	9.38

216340_s_at	cytochrome P450,	CYP2A7P1	6.98	6.92	6.58
208408_at	pleiotrophin	PTN	4.03	4.43	4.34
213824_at	oligodendrocyte li	OLIG2	4.80	5.21	4.54
209737_at	membrane associ	MAGI2	3.30	3.39	3.35
201529_s_at	replication protei	RPA1	9.35	9.48	9.47
205104_at	syntaphilin	SNPH	7.51	7.26	7.14
204245_s_at	ribonuclease P/M	RPP14	8.75	8.85	8.92
215669_at	major histocompa	HLA-DRB4	4.95	4.88	4.95
211105_s_at	nuclear factor of a	NFATC1	5.17	5.20	5.03
206140_at	LIM homeobox 2	LHX2	3.35	3.71	3.34
219916_s_at	ring finger protei	RNF39	5.14	4.99	4.82
221188_s_at	cell death-inducin	CIDEB	6.18	6.16	5.82
220096_at	---	---	7.73	7.43	7.29
219444_at	BCL6 co-represso	BCORL1	6.15	6.44	5.98
216132_at	---	---	4.54	4.66	4.27
219330_at	vang-like 1 (van g	VANGL1	7.99	8.16	8.12
208212_s_at	anaplastic lymph	ALK	5.39	5.32	5.23
207095_at	solute carrier fam	SLC10A2	3.90	4.00	3.62
221991_at	neurexophilin 3	NXPH3	7.02	6.93	6.54
217713_x_at	---	---	8.02	7.96	7.93
207771_at	solute carrier fam	SLC5A2	6.74	6.88	6.43
218089_at	chromosome 20 c	C20orf4	8.48	8.62	8.57
219447_s_at	solute carrier fam	SLC35C2	7.81	7.70	7.57
214341_at	Thiamine triphosp	THTPA	4.89	4.70	4.89
209535_s_at	---	---	6.81	7.03	6.66
211425_x_at	synovial sarcoma,	SSX4 /// SSX4B	3.72	4.18	3.77
216345_at	KIAA0913	KIAA0913	6.34	6.02	6.05
212619_at	transmembrane p	TMEM194A	6.01	5.62	5.88
206496_at	flavin containing r	FMO3	4.31	4.12	3.93
214955_at	transmembrane p	TMPRSS6	8.05	7.94	7.55
205866_at	ficolin (collagen/fi	FCN3	5.38	5.79	5.62
214220_s_at	Alstrom syndrome	ALMS1	8.16	7.95	8.02
204258_at	chromodomain he	CHD1	9.65	9.69	9.91
209947_at	ubiquitin associat	UBAP2L	7.65	7.57	7.57
216055_at	platelet-derived g	PDGFB	5.44	5.60	5.28
210135_s_at	short stature hom	SHOX2	6.57	6.60	6.55
202263_at	cytochrome b5 re	CYB5R1	8.39	8.27	8.26
222197_s_at	---	---	6.38	6.17	6.39
204994_at	myxovirus (influer	MX2	5.93	5.93	5.53
203710_at	inositol 1,4,5-tripl	ITPR1	8.56	8.23	8.50
201543_s_at	SAR1 homolog A (	SAR1A	9.30	9.33	9.45
208569_at	histone cluster 1,	HIST1H2AB	6.07	5.84	5.90
205827_at	cholecystokinin	CCK	6.23	6.05	5.88
202491_s_at	inhibitor of kappa	IKBKAP	10.05	10.21	10.23
222374_at	beta-transducin re	BTRC	5.73	6.06	5.62
206713_at	netrin G1	NTNG1	4.37	4.49	4.16
203492_x_at	centrosomal prot	CEP57	9.83	9.79	9.96

205092_x_at	zinc finger and BT	ZBTB1	3.81	3.65	3.59
218350_s_at	geminin, DNA rep	GMNN	11.32	11.32	11.43
214386_at	---	---	6.50	6.25	6.04
221464_at	olfactory receptor	OR1D2	4.55	4.17	4.16
203181_x_at	SFRS protein kinas	SRPK2	9.79	9.92	9.90
212978_at	leucine rich repea	LRRC8B	8.37	8.33	8.56
204263_s_at	carnitine palmitoy	CPT2	7.79	7.89	7.73
564_at	guanine nucleotid	GNA11	9.94	9.95	9.87
217959_s_at	trafficking protein	TRAPPC4	11.30	11.35	11.28
203070_at	sema domain, imr	SEMA3B	5.45	5.64	5.05
209756_s_at	v-myc myelocytor	MYCN	6.39	6.33	6.09
214700_x_at	RAP1 interacting f	RIF1	8.50	8.67	8.74
217296_at	Killer cell immuno	KIR2DL1	4.55	4.52	4.36
200095_x_at	ribosomal protein	RPS10	15.17	15.15	15.15
200890_s_at	signal sequence re	SSR1	9.19	9.25	9.34
215342_s_at	RAB GTPase activa	RABGAP1L	5.80	5.77	5.78
202138_x_at	aminoacyl tRNA sy	AIMP2	12.21	12.04	12.23
207146_at	keratin 32	KRT32	4.87	4.71	4.50
210752_s_at	MAX-like protein X	MLX	7.10	6.86	6.79
207116_s_at	glyceraldehyde-3-	GAPDHS	4.50	4.18	4.46
202640_s_at	RAN binding prote	RANBP3	8.87	8.80	8.73
213617_s_at	chromosome 18 c	C18orf10	8.81	8.87	8.83
222044_at	PDX1 C-terminal i	PCIF1	6.55	6.59	6.95
206656_s_at	chromosome 20 c	C20orf3	11.22	11.22	11.29
204439_at	interferon-induce	IFI44L	5.72	5.97	5.82
207099_s_at	choroideremia (Ra	CHM	4.40	4.74	4.08
200924_s_at	solute carrier fam	SLC3A2	10.17	9.92	10.30
206838_at	T-box 19	TBX19	6.31	6.39	6.32
221133_s_at	claudin 18	CLDN18	5.87	6.04	6.03
214389_at	solute carrier fam	SLC5A12	3.26	3.55	3.42
211149_at	ubiquitously trans	UTY	3.28	3.31	3.43
203090_at	stromal cell-deriv	SDF2	8.61	8.83	8.87
211994_at	WNK lysine defici	WNK1	9.95	9.80	9.77
201916_s_at	SEC63 homolog (S	SEC63	9.94	9.94	10.18
221748_s_at	tensin 1	TNS1	3.90	3.48	3.43
219841_at	activation-induce	AICDA	3.63	3.47	3.42
41047_at	chromosome 9 op	C9orf16	8.36	8.42	8.60
210426_x_at	RAR-related orpha	RORA	3.37	3.36	3.25
205454_at	hippocalcin	HPCA	7.53	7.24	7.48
202347_s_at	ubiquitin-conjuga	UBE2K	11.12	11.11	11.11
211008_s_at	ubiquitin-conjuga	UBE2I	7.98	7.95	7.50
218059_at	zinc finger protein	ZNF706	11.42	11.43	11.46
201376_s_at	heterogeneous nu	HNRNPF	10.02	10.04	10.11
217207_s_at	butyrophilin-like 3	BTNL3	6.99	6.89	6.91
219190_s_at	eukaryotic transla	EIF2C4	4.79	4.39	4.48
210128_s_at	leukotriene B4 rec	LTB4R	6.36	6.60	6.65
206219_s_at	vav 1 guanine nuc	VAV1	4.57	4.32	4.28

218476_at	protein-O-manno:	POMT1	6.85	6.99	6.95
221093_at	bromodomain cor	BRD7P3	4.52	4.18	4.90
203276_at	lamin B1	LMNB1	8.13	8.17	8.22
206504_at	cytochrome P450,	CYP24A1	3.43	3.35	3.13
209084_s_at	RAB28, member F	RAB28	8.32	8.28	8.44
220813_at	cysteinyl leukotrie	CYSLTR2	5.37	5.40	4.90
211828_s_at	TRAF2 and NCK in	TNIK	5.35	5.08	5.01
213039_at	Rho/Rac guanine	ARHGEF18	8.70	8.79	8.78
213146_at	lysine (K)-specific	KDM6B	5.57	5.41	5.32
202757_at	cofactor of BRCA1	COBRA1	9.37	9.34	9.47
217617_at	---	---	5.35	5.13	4.88
215958_at	---	---	3.32	3.43	3.39
210462_at	basic leucine zipp	BLZF1	3.91	3.83	3.59
202576_s_at	DEAD (Asp-Glu-Al	DDX19A /// DDX19	8.47	8.41	8.31
221633_at	non-SMC condens	NCAPH2	6.35	6.03	6.05
209848_s_at	silver homolog (m	SILV	5.82	5.57	5.67
212331_at	retinoblastoma-lik	RBL2	9.49	9.53	9.55
222072_at	X-prolyl aminopep	XPNPEP1	5.23	5.13	4.92
218914_at	chromosome 1 op	C1orf66	6.94	6.82	7.20
219036_at	centrosomal prot	CEP70	8.84	9.04	8.99
216807_at	KIAA1751	KIAA1751	4.25	4.21	4.03
200726_at	protein phosphata	PPP1CC	12.95	13.00	12.95
207814_at	defensin, alpha 6,	DEFA6	5.04	5.13	5.25
215978_x_at	zinc finger protein	ZNF721	10.46	10.76	10.53
218313_s_at	UDP-N-acetyl-alph	GALNT7	10.06	10.22	10.12
216835_s_at	docking protein 1,	DOK1	7.88	7.81	7.79
209939_x_at	CASP8 and FADD-	CFLAR	11.27	11.09	11.20
202069_s_at	isocitrate dehydro	IDH3A	9.16	9.29	9.39
217931_at	canopy 3 homolog	CNPY3	8.12	8.03	7.99
201463_s_at	transaldolase 1	TALDO1	12.23	12.16	12.18
33814_at	p21 protein (Cdc4	PAK4	8.10	8.00	8.00
209937_at	transmembrane 4	TM4SF4	3.47	3.51	3.36
218777_at	receptor accessor	REEP4	8.59	8.56	8.55
214722_at	Notch homolog 2	NOTCH2NL	9.66	9.68	9.73
33646_g_at	thromboxane A2 r	TBXA2R	4.98	5.08	4.97
209811_at	caspase 2, apopto	CASP2	6.19	6.34	6.43
204488_at	dolichol kinase	DOLK	7.50	7.71	7.44
214939_x_at	myeloid/lymphoic	MLLT4	5.43	5.51	5.51
210841_s_at	neuropilin 2	NRP2	5.52	5.55	5.51
201183_s_at	chromodomain he	CHD4	10.15	10.14	10.13
206799_at	secretoglobin, fan	SCGB1D2	3.79	3.76	3.67
40359_at	Ras association (R	RASSF7	8.97	9.17	8.89
205884_at	integrin, alpha 4 (	ITGA4	3.33	3.08	3.22
220065_at	tenomodulin	TNMD	3.97	4.04	4.01
214064_at	transferrin	TF	7.01	6.97	6.88
205638_at	brain-specific angi	BAI3	3.71	3.41	3.51
216117_at	exosome compon	EXOSC2	5.40	5.56	5.30

203077_s_at	SMAD family men	SMAD2	9.05	8.91	9.16
212735_at	KIAA0226	KIAA0226	6.35	6.00	6.55
207619_at	hypocretin (orexir	HCRTR1	5.56	5.45	5.35
220351_at	chemokine (C-C m	CCRL1	4.00	3.60	3.87
213900_at	family with seque	FAM189A2	5.40	5.28	5.08
210223_s_at	major histocompa	MR1	6.49	6.55	6.44
217349_s_at	prickle homolog 3	PRICKLE3	6.31	6.53	6.34
219495_s_at	zinc finger proteir	ZNF180	6.54	6.92	6.66
205558_at	TNF receptor-assc	TRAF6	7.90	7.78	7.85
215208_x_at	Ribosomal proteir	RPL35A	7.30	7.43	7.26
217164_at	---	---	7.10	7.17	7.15
215196_at	---	---	5.25	5.25	5.20
211231_x_at	cytochrome P450,	CYP4A11	5.79	5.77	5.42
210197_at	inositol 1,3,4-tripl	ITPK1	5.70	5.90	5.54
214540_at	histone cluster 1,	HIST1H2BO	3.99	3.96	3.97
218473_s_at	glycosyltransferas	GLT25D1	10.04	9.76	9.86
204118_at	CD48 molecule	CD48	5.57	5.59	5.78
212833_at	solute carrier fam	SLC25A46	10.35	10.39	10.50
207879_at	---	---	3.57	3.38	3.19
212924_s_at	LSM4 homolog, U	LSM4	5.56	5.38	5.38
210520_at	fetuin B	FETUB	4.53	4.49	4.17
204473_s_at	zinc finger proteir	ZNF592	7.37	7.10	6.96
AFFX-r2-Bs-thr-3	---	---	10.05	9.94	9.88
211374_x_at	---	---	6.64	6.67	6.75
214302_x_at	Gap junction prot	GJC2	6.81	6.81	6.53
220015_at	castor zinc finger	CASZ1	8.12	8.14	8.26
214978_s_at	protein tyrosine p	PPFIA4	6.59	6.21	6.02
201538_s_at	dual specificity ph	DUSP3	6.71	6.82	6.99
211383_s_at	WD repeat domai	WDR37	7.89	8.02	7.92
208577_at	histone cluster 1,	HIST1H3C	7.23	6.94	7.27
59697_at	RAB15, member F	RAB15	6.81	6.66	6.63
217498_at	---	---	4.00	3.98	3.74
218571_s_at	chromatin modify	CHMP4A	9.96	9.83	9.84
209543_s_at	CD34 molecule	CD34	7.20	7.06	6.83
207067_s_at	histidine decarbo	HDC	5.10	4.76	4.79
206360_s_at	suppressor of cyto	SOCS3	6.12	6.14	5.83
220283_at	HHIP-like 2	HHIPL2	5.53	5.20	4.99
212159_x_at	adaptor-related p	AP2A2	9.07	9.10	9.08
210671_x_at	mitogen-activatec	MAPK8	4.54	4.64	4.65
220851_at	---	---	3.37	3.44	3.17
208862_s_at	catenin (cadherin-	CTNND1	7.77	7.74	7.60
215892_at	Zinc finger proteir	ZNF440	3.99	3.88	3.93
204876_at	zinc finger proteir	ZNF646	6.31	5.85	6.18
201006_at	peroxiredoxin 2	PRDX2	7.06	6.79	7.02
211891_s_at	Rho guanine nucle	ARHGEF4	5.30	5.19	4.85
201258_at	ribosomal protein	RPS16	14.75	14.67	14.68
205680_at	matrix metallopep	MMP10	3.91	3.81	3.52

216690_at	olfactory receptor	OR7C1	4.39	4.08	4.34
214990_at	phosphatidylinosi	PIGO	6.06	6.06	5.55
204904_at	gap junction prote	GJA4	6.33	6.26	6.06
201703_s_at	protein phosphata	PPP1R10	8.02	8.09	7.89
212866_at	R3H domain and c	R3HCC1	8.05	8.11	8.10
216284_at	---	---	6.09	6.14	5.52
208553_at	histone cluster 1,	HIST1H1E	6.03	5.56	5.85
211411_at	---	---	6.18	6.05	5.96
205542_at	six transmembran	STEAP1	10.79	10.67	10.80
205213_at	ArfGAP with coile	ACAP1	5.67	5.87	5.58
200919_at	polyhomeotic hor	PHC2	11.80	11.59	11.72
213932_x_at	major histocompa	HLA-A	12.64	12.60	12.66
209151_x_at	transcription facto	TCF3	5.32	5.51	5.81
220344_at	chromosome 11 c	C11orf16	7.36	7.51	7.08
205591_at	olfactomedin 1	OLFM1	4.16	4.04	4.08
209173_at	anterior gradient	AGR2	4.20	4.12	3.93
205748_s_at	ring finger proteir	RNF126	7.86	7.81	7.86
206859_s_at	progestagen-asso	PAEP	5.76	5.91	5.72
210273_at	protocadherin 7	PCDH7	4.93	4.84	4.65
211534_x_at	protein tyrosine p	PTPRN2	5.27	5.36	5.24
213161_at	tropomodulin 1 //	TMOD1 /// TSTD2	9.39	9.14	9.29
215236_s_at	phosphatidylinosi	PICALM	7.17	7.00	7.15
217129_at	---	---	6.01	5.74	5.66
219676_at	zinc finger and SC	ZSCAN16	6.62	6.48	6.60
203748_x_at	RNA binding moti	RBMS1	9.72	9.70	9.75
222214_at	---	---	6.62	6.77	6.52
206917_at	guanine nucleotid	GNA13	4.42	4.35	4.18
210274_at	melanoma antiger	MAGEA8	4.43	4.51	4.46
202032_s_at	mannosidase, alpl	MAN2A2	8.56	8.48	8.43
219259_at	sema domain, imr	SEMA4A	7.18	7.25	6.78
219691_at	sterile alpha moti	SAMD9	5.28	5.37	5.48
216120_s_at	ATPase, Ca++ tran	ATP2B2	4.01	3.79	4.16
204089_x_at	mitogen-activatec	MAP3K4	10.01	10.01	10.05
217540_at	family with seque	FAM55C	6.81	6.93	7.00
205293_x_at	BAI1-associated p	BAIAP2	6.41	6.54	6.21
220605_s_at	sirtuin (silent mat	SIRT2	7.83	7.63	7.90
217256_x_at	---	---	13.17	13.16	13.27
222274_at	zinc finger, DHHC-	ZDHHC8P1	6.35	6.08	5.85
202239_at	poly (ADP-ribose)	PARP4	9.97	9.91	9.99
216735_x_at	---	---	4.02	4.26	4.25
209637_s_at	regulator of G-prc	RGS12	5.58	5.56	5.47
217471_at	---	---	4.53	4.31	4.10
220031_at	OTU domain cont	OTUD7B	4.41	4.12	4.14
209159_s_at	NDRG family mem	NDRG4	5.87	5.96	5.82
216941_s_at	TATA box binding	TAF1B	9.13	9.02	9.21
214693_x_at	neuroblastoma br	NBPF10	8.29	8.24	8.22
206153_at	cytochrome P450	CYP4F11	3.95	3.83	3.48

216674_at	hairy and enhance	HES2	4.72	4.62	4.66
207956_x_at	PDS5, regulator of	PDS5B	9.42	9.60	9.61
219728_at	myotilin	MYOT	3.34	3.54	3.08
220555_s_at	PDZ domain conta	PDZD7	5.54	5.41	5.12
217380_s_at	X-prolyl aminopep	XPNPEP1	3.38	3.75	3.67
202717_s_at	cell division cycle	CDC16	9.75	9.75	9.72
212629_s_at	protein kinase N2	PKN2	10.42	10.45	10.46
206405_x_at	ubiquitin specific	USP6	6.98	6.95	7.15
201123_s_at	eukaryotic transla	EIF5A	11.26	11.27	11.29
204941_s_at	aldehyde dehydro	ALDH3B2	3.90	3.88	3.62
218580_x_at	aurora kinase A in	AURKAIP1	11.57	11.51	11.51
211983_x_at	actin, gamma 1	ACTG1	14.22	14.29	14.19
214958_s_at	transmembrane c	TMC6	6.29	6.37	6.19
210954_s_at	TSC22 domain fan	TSC22D2	3.98	4.40	4.28
208359_s_at	potassium inward	KCNJ4	4.45	4.00	4.18
214255_at	ATPase, class V, ty	ATP10A	4.72	4.79	4.55
217185_s_at	zinc finger protein	ZNF259 /// ZNF25	8.59	8.40	8.63
219144_at	dual specificity ph	DUSP26	7.32	6.87	7.04
202386_s_at	KIAA0430	KIAA0430	8.46	8.62	8.72
210799_at	5-hydroxytryptam	HTR1B	5.64	5.81	5.58
207451_at	NK2 homeobox 8	NKX2-8	6.96	6.67	6.62
201500_s_at	protein phosphata	PPP1R11	9.77	9.69	9.75
216886_at	cholinergic recept	CHRNA4	6.11	5.95	5.15
208563_x_at	POU class 3 home	POU3F3	5.47	5.18	5.11
207846_at	POU class 1 home	POU1F1	3.22	3.45	3.24
221372_s_at	purinergic recepto	P2RX2	5.42	5.31	5.20
222046_at	serrate RNA effec	SRRT	6.17	5.75	5.90
217576_x_at	son of sevenless h	SOS2	6.52	6.34	6.06
205756_s_at	coagulation factor	F8	6.66	6.77	6.58
204102_s_at	eukaryotic transla	EEF2	14.20	14.18	14.14
209312_x_at	major histocompa	HLA-DRB1 /// HLA-	6.45	6.40	5.98
206895_at	protein phosphata	PPP1R3A	3.67	3.39	3.48
210264_at	G protein-couplec	GPR35	6.98	6.88	6.55
205749_at	cytochrome P450,	CYP1A1	5.75	5.57	5.74
206433_s_at	sparc/osteonectin	SPOCK3	3.91	4.21	4.14
222261_at	KIAA1609	KIAA1609	6.79	6.69	6.41
220322_at	interleukin 1 fami	IL1F9	4.63	4.92	4.82
211979_at	G protein-couplec	GPR107	7.95	7.84	7.97
216175_at	---	---	6.90	7.05	6.55
38269_at	protein kinase D2	PRKD2	8.03	8.23	8.01
203434_s_at	membrane metall	MME	8.79	8.88	9.05
203714_s_at	tubulin folding co	TBCE	10.28	10.20	10.17
207069_s_at	SMAD family men	SMAD6	7.11	7.23	7.17
204572_s_at	protein (peptidylp	PIN4	5.44	5.55	5.40
203063_at	protein phosphata	PPM1F	7.71	7.71	7.71
206117_at	tropomyosin 1 (al	TPM1	6.41	6.58	6.50
201241_at	DEAD (Asp-Glu-Ala	DDX1	11.82	11.90	11.87

213689_x_at	family with seque	FAM69A	8.26	8.49	8.56
209776_s_at	solute carrier fam	SLC19A1	6.53	6.50	6.28
207555_s_at	thromboxane A2 r	TBXA2R	8.22	7.94	7.84
218602_s_at	HAUS augmin-like	HAUS6	7.84	8.17	8.33
206531_at	D4, zinc and doub	DPF1	5.70	5.82	5.34
201501_s_at	G-rich RNA seque	GRSF1	9.92	9.98	10.20
202476_s_at	tubulin, gamma co	TUBGCP2	7.09	6.84	6.87
205276_s_at	GTP binding prote	GTPBP1	7.79	7.54	7.57
209350_s_at	G protein pathwa	GPS2	7.45	7.37	7.58
221475_s_at	ribosomal protein	RPL15	14.64	14.60	14.66
204059_s_at	malic enzyme 1, N	ME1	11.74	11.69	11.79
209237_s_at	solute carrier fam	SLC23A2	7.03	7.15	7.12
218054_s_at	chromosome 19 o	C19orf50	4.72	4.97	4.75
212334_at	glucosamine (N-ac	GNS	9.74	9.80	10.04
201504_s_at	translin	TSN	8.62	8.70	8.74
218679_s_at	vacuolar protein s	VPS28	9.52	9.30	9.43
221599_at	chromosome 11 o	C11orf67	8.70	8.72	8.77
209547_s_at	splicing factor 4	SF4	7.98	8.05	8.03
210288_at	killer cell lectin-lik	KLRG1	6.47	6.70	6.26
205176_s_at	integrin beta 3 bir	ITGB3BP	10.55	10.48	10.55
221373_x_at	persephin	PSPN	6.78	6.57	6.26
214406_s_at	solute carrier fam	SLC7A4	6.19	6.44	5.95
216990_at	phosphoribosylgly	GART	3.37	3.12	3.32
206903_at	endo/exonuclease	EXOG	6.62	6.32	6.45
202776_at	deoxynucleotidylt	DNTTIP2	10.60	10.75	10.77
215249_at	ribosomal protein	RPL35A	3.87	4.19	3.82
218960_at	transmembrane p	TMPRSS4	6.15	6.39	5.81
213827_at	Rho GTPase activa	ARHGAP33	7.16	7.24	6.82
208990_s_at	heterogeneous nu	HNRNPH3	11.74	11.91	11.91
221320_at	BCL2-like 10 (apo	BCL2L10	5.95	5.64	5.71
209877_at	synuclein, gamma	SNCG	7.05	7.08	6.63
216537_s_at	sialic acid binding	SIGLEC7	7.84	7.82	7.50
204728_s_at	WD repeat and HI	WDHD1	7.89	7.77	7.94
221946_at	chromosome 9 op	C9orf116	6.96	6.90	6.86
219272_at	tripartite motif-co	TRIM62	5.19	5.29	5.16
212476_at	ArfGAP with coile	ACAP2	9.25	9.23	9.51
214338_at	DnaJ (Hsp40) hom	DNAJB12	7.52	7.45	7.48
206100_at	carboxypeptidase	CPM	3.48	3.12	3.07
217801_at	ATP synthase, H+	ATP5E	12.92	12.88	13.08
209640_at	promyelocytic leu	PML	6.46	6.73	6.32
201835_s_at	protein kinase, AM	PRKAB1	6.23	6.02	5.96
78330_at	zinc finger protein	ZNF335	6.44	6.36	6.02
215625_at	hypothetical prote	LOC644450	4.29	4.22	4.45
204109_s_at	nuclear transcript	NFYA	5.83	5.82	5.30
216223_at	carboxypeptidase	CPN2	3.60	3.58	3.74
219099_at	chromosome 12 o	C12orf5	8.60	8.77	8.73
216464_x_at	G protein-couplec	GPR44	5.31	5.44	5.05

210067_at	aquaporin 4	AQP4	3.55	3.49	3.55
220248_x_at	NSFL1 (p97) cofac	NSFL1C	9.24	9.39	9.34
203640_at	muscleblind-like 2	MBNL2	10.95	10.94	11.27
220431_at	transmembrane p	TMPRSS11E	4.13	3.82	3.73
208279_s_at	CMT1A duplicatec	CDRT1	4.77	4.79	4.58
218743_at	chromatin modify	CHMP6	7.49	7.50	7.43
211016_x_at	heat shock 70kDa	HSPA4	7.41	7.26	7.35
219066_at	phosphopantothe	PPCDC	8.76	8.63	8.83
208489_at	gap junction prote	GJA8	5.83	5.65	5.55
215141_at	chromosome 4 op	C4orf10	6.61	6.66	6.78
207925_at	cystatin D	CST5	4.81	4.48	4.51
206434_at	sparc/osteonectir	SPOCK3	3.47	3.66	3.35
204887_s_at	polo-like kinase 4	PLK4	8.52	8.69	8.79
207880_at	ADAM metallopep	ADAM11	5.48	5.47	5.70
219517_at	elongation factor	ELL3 /// SERINC4	6.67	6.75	6.44
219283_at	C1GALT1-specific	C1GALT1C1	8.70	8.97	8.76
210722_at	pancreatic lipase-r	PNLIPRP1	5.37	5.36	5.08
202709_at	fibromodulin	FMOD	5.52	5.63	5.69
219636_s_at	armadillo repeat c	ARMC9	7.19	6.99	7.08
207865_s_at	bone morphogene	BMP8B	4.81	4.46	4.69
203991_s_at	lysine (K)-specific	KDM6A	6.13	6.21	6.29
205978_at	klotho	KL	3.27	3.35	3.32
204166_at	strawberry notch	SBNO2	7.06	7.14	6.84
216745_x_at	---	---	7.12	7.18	7.10
210228_at	colony stimulating	CSF2	7.69	7.71	7.34
214957_at	actin-like 8	ACTL8	7.36	7.24	7.42
209759_s_at	dodecenoyl-CoA i:	DCI	9.63	9.54	9.60
213365_at	ERI1 exoribonucle	ERI2	8.87	8.91	8.94
206638_at	5-hydroxytryptam	HTR2B	3.89	3.93	4.09
1861_at	BCL2-associated a	BAD	7.78	7.83	7.78
221122_at	HRAS-like suppres	HRASLS2	6.20	5.91	5.69
219851_at	zinc finger proteir	ZNF613	3.53	3.51	3.88
209775_x_at	solute carrier fam	SLC19A1	5.70	5.86	5.60
202396_at	transcription elon	TCERG1	11.05	11.20	11.34
213156_at	---	---	9.67	9.79	9.90
212041_at	ATPase, H+ transp	ATP6V0D1	9.48	9.62	9.51
215140_at	---	---	5.23	5.37	5.55
213076_at	inositol 1,4,5-trisp	ITPKC	6.73	6.69	6.99
216437_at	---	---	4.00	4.23	3.89
215838_at	leukocyte immuno	LILRA5	4.67	4.47	4.60
201882_x_at	UDP-Gal:betaGlcN	B4GALT1	7.68	7.58	7.34
220996_s_at	chromosome 1 op	C1orf14	4.48	4.38	4.30
218391_at	SNF8, ESCRT-II cor	SNF8	10.93	10.87	10.84
219407_s_at	laminin, gamma 3	LAMC3	6.91	6.81	6.46
201727_s_at	ELAV (embryonic l	ELAVL1	8.41	8.36	8.49
220602_s_at	hypothetical LOC3	LOC388152 /// LO	8.49	8.13	8.29
208994_s_at	peptidylprolyl isor	PPIG	9.33	9.52	9.50

212620_at	zinc finger proteir	ZNF609	9.14	9.01	8.93
205163_at	myosin light chain	MYLPF	6.50	6.65	6.71
220763_at	heat shock 70kDa	HSPA12A	5.04	4.63	4.62
211433_x_at	KIAA1539	KIAA1539	7.00	6.84	6.79
206710_s_at	erythrocyte meml	EPB41L3	10.00	10.00	10.14
213501_at	acyl-CoA oxidase :	ACOX1	7.34	7.22	7.21
212730_at	synemin, interme	SYNM	6.88	7.25	6.73
214905_at	Ras protein-specif	RASGRF1	4.16	4.23	4.12
205783_at	kallikrein-related	KLK13	5.10	5.09	5.03
215281_x_at	pogo transposable	POGZ	6.07	6.16	6.20
207527_at	potassium inward	KCNJ9	4.49	4.42	4.26
205719_s_at	phenylalanine hyc	PAH	3.39	3.23	3.33
207220_at	ADP-ribosyltransf	ART4	4.01	3.79	3.84
202871_at	cell division cycle	CDC20	8.68	8.55	8.56
204322_at	golgi integral mem	GOLIM4	5.74	5.87	5.70
221897_at	tripartite motif-cc	TRIM52	6.66	7.13	6.94
220583_at	---	---	7.27	6.87	6.71
201388_at	proteasome (pros	PSMD3	9.91	9.96	9.99
219939_s_at	cold shock domair	CSDE1	12.27	12.27	12.35
219637_at	armadillo repeat c	ARMC9	6.53	6.52	6.41
216679_at	tigger transposabl	TIGD1L	5.94	5.80	5.67
215957_at	ubiquitin-conjugat	UBE2D1	3.25	3.11	3.04
208753_s_at	nucleosome asser	NAP1L1	11.76	11.94	12.11
214542_x_at	histone cluster 1,	HIST1H2AI	7.42	7.32	7.29
202338_at	thymidine kinase	TK1	9.23	9.16	9.26
220005_at	purinergic recepto	P2RY13	3.53	3.90	3.42
205965_at	basic leucine zipp	BATF	6.26	6.41	6.15
212027_at	RNA binding moti	RBM25	9.32	9.37	9.46
216790_at	---	---	4.45	4.05	3.94
207565_s_at	major histocompa	MR1	6.03	6.00	6.23
209311_at	BCL2-like 2	BCL2L2	9.24	9.22	9.23
213533_at	DNA segment on c	D4S234E /// FOXP	6.82	6.70	6.45
33494_at	electron-transferr	ETFDH	7.32	7.55	7.58
210227_at	discs, large (Drosc	DLGAP2	3.53	3.82	3.41
217412_at	T cell receptor alp	TRA@	3.50	3.47	3.42
219613_s_at	sirtuin (silent mat	SIRT6	7.74	7.71	7.57
217070_at	methylenetetrahy	MTHFR	5.34	5.39	5.41
208965_s_at	interferon, gamm	IFI16	9.43	9.41	9.39
203495_at	leucine rich repea	LRRC14	8.17	7.97	7.83
208381_s_at	sphingosine-1-phc	SGPL1	5.29	4.99	5.12
211550_at	epidermal growth	EGFR	7.05	7.00	6.76
216564_at	---	---	7.27	6.96	7.11
215763_at	---	---	3.42	3.53	3.32
208531_at	serpin peptidase i	SERPINA2	4.60	4.42	4.43
221012_s_at	tripartite motif-cc	TRIM8	9.57	9.46	9.50
214102_at	ArfGAP with RhoG	ARAP2	7.53	7.63	7.76
218941_at	F-box and WD rep	FBXW2	8.38	8.39	8.41

218352_at	regulator of chror	RCBTB1	7.92	7.99	8.19
217816_s_at	PEST proteolytic s	PCNP	11.66	11.60	11.79
219403_s_at	heparanase	HPSE	8.30	8.18	8.36
202410_x_at	insulin-like growth	IGF2 /// INS-IGF2	6.54	6.42	6.21
205237_at	ficolin (collagen/fi	FCN1	4.53	4.53	4.57
203479_s_at	OTU domain conta	OTUD4	8.15	8.38	8.21
222377_at	T-box 10	TBX10	6.00	5.71	5.71
208140_s_at	leucine rich repea	LRRC48	5.10	5.11	4.77
222223_s_at	interleukin 1 fami	IL1F5	5.46	5.47	5.02
214885_at	MYST histone ace	MYST1	5.57	6.21	5.73
216813_at	---	---	5.14	5.27	4.99
220464_at	MCF.2 cell line de	MCF2L	6.78	6.69	6.75
201910_at	FERM, RhoGEF (A	FARP1	5.16	4.73	4.61
214536_at	secreted LY6/PLAU	SLURP1	6.11	6.21	6.09
219697_at	heparan sulfate (g	HS3ST2	5.63	5.91	5.69
219072_at	B-cell CLL/lympho	BCL7C	8.60	8.47	8.38
216056_at	CD44 molecule (In	CD44	5.30	5.16	5.15
213570_at	eukaryotic transla	EIF4E2	5.88	6.24	5.78
209921_at	solute carrier fam	SLC7A11	12.23	11.93	12.20
220590_at	integrin alpha FG-	ITFG2	6.96	7.08	6.82
202749_at	tryptophan rich ba	WRB	10.07	10.24	10.17
44563_at	WD repeat contai	WRAP53	8.02	8.28	8.07
206314_at	zinc finger protein	ZNF167	3.40	3.53	3.38
213073_at	zinc finger, FYVE c	ZFYVE26	6.96	7.16	6.74
207070_at	retinal G protein c	RGR	5.46	5.18	5.00
211020_at	glucosaminyl (N-a	GCNT2	5.09	5.40	5.05
218053_at	PRP40 pre-mRNA	PRPF40A	11.20	11.35	11.40
203120_at	tumor protein p53	TP53BP2	8.23	8.19	8.30
214424_s_at	Aldolase B, fructo	ALDOB	4.96	4.94	4.79
207993_s_at	calcium binding pı	CHP	7.77	7.69	7.81
206762_at	potassium voltage	KCNA5	4.76	4.77	4.60
216716_at	ABO blood group	ABO	6.67	6.68	6.44
209963_s_at	erythropoietin rec	EPOR	5.89	5.37	5.54
203420_at	family with seque	FAM8A1	8.63	8.88	8.81
219848_s_at	zinc finger protein	ZNF432	7.49	7.55	7.47
AFFX-r2-Ec-bioB	---	---	9.51	9.44	9.54
209900_s_at	solute carrier fam	SLC16A1	9.63	9.61	9.82
209481_at	SNF related kinase	SNRK	8.55	8.73	8.70
216932_at	---	---	5.86	5.64	5.41
211804_s_at	cyclin-dependent	CDK2	7.06	7.21	7.00
211199_s_at	inducible T-cell co	ICOSLG	6.06	5.96	5.80
206356_s_at	guanine nucleotid	GNAL	4.28	4.16	4.32
208044_s_at	peroxisome prolif	PPARD	7.23	7.19	7.15
208360_s_at	---	---	4.59	4.58	4.09
218582_at	membrane-associ	5-Mar	9.31	9.48	9.56
218128_at	nuclear transcript	NFYB	8.38	8.48	8.61
214574_x_at	leukocyte specific	LST1	6.15	6.13	5.96

221978_at	major histocompa	HLA-F	5.59	5.27	5.77
217725_x_at	SERPINE1 mRNA b	SERBP1	11.17	11.28	11.38
220111_s_at	anoctamin 2	ANO2	4.00	3.63	3.78
221925_s_at	centrosome and s	CSPP1	7.16	7.27	7.51
213971_s_at	similar to KIAA016	LOC100292841 ///	9.04	9.07	8.97
205567_at	carbohydrate (ker	CHST1	5.67	5.38	5.44
218964_at	AT rich interactive	ARID3B	6.73	6.71	6.64
215444_s_at	tripartite motif-co	TRIM31	5.59	5.58	5.27
216653_at	Down-regulator o	DR1	3.30	3.68	3.44
205495_s_at	granulysin	GNLY	6.52	6.36	6.39
211230_s_at	phosphoinositide-	PIK3CD	5.50	5.33	5.42
206765_at	potassium inward	KCNJ2	3.64	3.38	3.57
217629_at	---	---	5.96	5.93	5.79
201000_at	alanyl-tRNA synth	AARS	10.99	10.71	10.97
205690_s_at	BUD31 homolog (	BUD31	10.87	10.82	10.99
208068_x_at	chorionic somator	CSH1 /// CSH2 ///	6.74	6.68	6.55
206658_at	uroplakin 3B	UPK3B	6.34	6.12	5.78
212100_s_at	polymerase (DNA	POLDIP3	9.21	9.21	9.22
AFFX-TrpnX-M_a	---	---	3.61	3.78	3.59
202993_at	ilvB (bacterial ace	ILVBL	7.58	7.68	7.53
213790_at	ADAM metallopep	ADAM12	7.99	8.09	8.06
215945_s_at	tripartite motif-co	TRIM2	6.99	7.11	7.30
218651_s_at	La ribonucleoprot	LARP6	9.73	9.31	9.67
209462_at	amyloid beta (A4)	APLP1	5.33	5.43	5.21
204770_at	transporter 2, ATP	TAP2	6.27	6.02	6.17
221433_at	fibroblast growth	FGF21	6.06	5.47	5.88
214438_at	H2.0-like homeob	HLX	6.31	5.87	6.26
217187_at	mucin 5AC, oligon	MUC5AC	5.78	5.56	4.93
218908_at	alveolar soft part	ASPSCR1	7.67	7.63	7.56
202928_s_at	PHD finger protei	PHF1	6.62	6.75	6.79
204339_s_at	regulator of G-pro	RGS4	5.46	5.40	5.36
222023_at	A kinase (PRKA) a	AKAP13	5.49	5.39	5.41
221625_at	---	---	3.79	3.55	3.79
210685_s_at	ubiquitination fac	UBE4B	7.11	7.04	7.30
221982_x_at	---	---	7.88	7.95	7.92
218037_at	family with seque	FAM134A	8.99	8.85	8.77
201589_at	structural mainte	SMC1A	10.90	10.93	11.02
215408_at	---	---	3.67	3.89	3.74
207750_at	---	---	3.06	3.07	3.04
209472_at	cysteine conjugat	CCBL2	9.40	9.46	9.52
205165_at	cadherin, EGF LAG	CELSR3	7.55	7.53	7.48
205028_at	trophinin	TRO	5.00	4.66	4.73
219512_at	DSN1, MIND kinet	DSN1	8.28	8.61	8.83
210165_at	deoxyribonucleas	DNASE1	5.59	5.22	5.44
214313_s_at	Eukaryotic transla	EIF5B	6.39	6.89	6.68
218568_at	acylglycerol kinas	AGK	8.06	8.07	8.23
207897_at	corticotropin rele	CRHR2	4.82	4.85	4.62

207039_at	cyclin-dependent	CDKN2A	3.92	3.94	3.85
210544_s_at	aldehyde dehydrc	ALDH3A2	7.24	7.36	7.10
213422_s_at	matrix-remodellin	MXRA8	6.40	6.34	6.08
214419_s_at	Cytochrome P450	CYP2C9	5.56	5.25	5.22
219995_s_at	zinc finger proteir	ZNF750	3.14	3.05	3.28
205209_at	activin A receptor	ACVR1B	6.45	6.45	6.48
213719_s_at	hypothetical prote	LOC100287659	4.10	4.21	3.99
204961_s_at	neutrophil cytoso	NCF1 /// NCF1B //	5.43	5.58	5.24
212853_at	DCN1, defective ir	DCUN1D4	7.33	7.41	7.49
212607_at	v-akt murine thym	AKT3	8.40	8.36	8.48
207562_at	diacylglycerol kina	DGKQ	6.03	6.22	5.59
220476_s_at	chromosome 1 op	C1orf183	5.98	5.53	5.52
202608_s_at	N-deacetylase/N-s	NDST1	6.13	6.08	5.78
215626_at	---	---	3.92	4.01	3.79
203577_at	general transcript	GTF2H4	8.36	8.45	8.37
213682_at	nucleoporin 50kD	NUP50	10.98	11.11	11.12
217281_x_at	immunoglobulin h	IGH@ /// IGHA1 //	4.10	3.78	3.98
208504_x_at	protocadherin bet	PCDHB11	6.15	6.05	6.20
207446_at	toll-like receptor 6	TLR6	5.26	4.99	4.89
215289_at	zinc finger proteir	ZNF749	3.61	3.52	3.30
209019_s_at	PTEN induced put	PINK1	9.71	9.63	9.77
207793_s_at	erythrocyte meml	EPB41	3.46	3.24	3.20
212710_at	calmodulin regula	CAMSAP1	7.84	7.81	8.05
206735_at	cholinergic recept	CHRNA4	6.86	6.80	6.39
207457_s_at	lymphocyte antige	LY6G6D	6.67	6.58	6.33
220636_at	dynein, axonemal	DNAI2	3.82	3.73	3.25
216324_at	---	---	4.95	4.53	4.72
215115_x_at	neurotrophic tyro	NTRK3	6.00	5.99	5.98
205328_at	claudin 10	CLDN10	4.80	4.70	4.70
221678_at	abhydrolase doma	ABHD6	4.43	4.52	4.21
211001_at	tripartite motif-co	TRIM29	6.58	6.66	6.66
217108_at	---	---	4.02	4.16	4.12
209591_s_at	bone morphogene	BMP7	3.42	3.70	3.48
210062_s_at	zinc finger proteir	ZNF589	5.50	5.48	5.42
211639_x_at	immunoglobulin h	IGH@ /// IGHA1 //	5.40	5.19	5.09
216356_x_at	BAI1-associated p	BAIAP3	7.33	7.19	7.32
202105_at	immunoglobulin (	IGBP1	9.85	9.76	9.80
205790_at	src kinase associat	SKAP1	4.83	4.66	4.19
206488_s_at	CD36 molecule (th	CD36	3.95	3.86	3.93
204425_at	Rho GTPase activa	ARHGAP4	6.48	6.50	6.42
208860_s_at	alpha thalassemia	ATRX	8.02	8.25	8.25
210327_s_at	alanine-glyoxylate	AGXT	5.25	4.96	4.58
208626_s_at	vesicle amine trar	VAT1	9.24	9.25	9.28
210258_at	regulator of G-pro	RGS13	3.10	3.32	3.32
208049_s_at	tachykinin recepto	TACR1	4.64	4.75	4.78
217557_s_at	Carboxypeptidase	CPM	6.30	6.17	5.98
215788_at	chromosome 1 op	C1orf222	6.17	6.11	6.25

217112_at	platelet-derived g	PDGFB	4.92	4.63	4.40
221951_at	transmembrane p	TMEM80	8.27	8.04	8.28
208409_at	solute carrier fam	SLC14A2	3.74	3.87	4.01
204290_s_at	aldehyde dehydrc	ALDH6A1	7.79	7.75	7.71
204061_at	protein kinase, X-l	PRKX	7.41	7.43	7.62
201575_at	SNW domain cont	SNW1	11.28	11.28	11.35
211681_s_at	PDZ and LIM dom	PDLIM5	6.02	6.33	5.68
217120_s_at	hypothetical LOC7	LOC728142	4.64	4.46	4.41
203016_s_at	synovial sarcoma,	SSX2IP	9.27	9.39	9.44
202650_s_at	KIAA0195	KIAA0195	7.29	7.22	6.98
213914_s_at	Spectrin, beta, no	SPTBN1	6.82	6.93	6.56
212991_at	F-box protein 9	FBXO9	6.95	6.83	6.92
204184_s_at	adrenergic, beta, r	ADRBK2	6.35	6.58	6.18
211245_x_at	killer cell immuno	KIR2DL4	4.11	4.16	3.95
216937_s_at	retinoschisin 1	RS1	5.99	5.59	5.56
201864_at	GDP dissociation i	GDI1	8.06	8.01	8.04
222115_x_at	glyoxylate reducta	GLYR1	6.37	6.48	6.29
208841_s_at	GTPase activating	G3BP2	10.95	11.05	11.28
202614_at	solute carrier fam	SLC30A9	10.32	10.42	10.46
216119_s_at	sperm flagellar 1	SPEF1	5.86	5.72	5.74
206484_s_at	X-prolyl aminopep	XPNPEP2	5.64	5.51	5.32
209401_s_at	solute carrier fam	SLC12A4	5.48	5.59	5.99
214475_x_at	calpain 3, (p94)	CAPN3	3.95	4.01	3.71
216733_s_at	glycine amidinotra	GATM	3.64	3.97	3.72
205974_at	homeobox D1	HOXD1	3.68	3.80	3.49
216097_at	---	---	3.54	3.33	3.22
215840_at	dynein, axonemal	DNAH2	5.09	5.48	5.01
204550_x_at	glutathione S-tran	GSTM1	6.58	6.60	6.33
214133_at	mucin 6, oligomer	MUC6	7.30	7.12	7.12
205521_at	endo/exonuclease	EXOG	7.90	7.99	8.01
222174_at	---	---	4.19	4.41	4.57
219655_at	chromosome 7 op	C7orf10	6.80	6.73	6.87
213531_s_at	RAB3 GTPase acti	RAB3GAP1	11.57	11.40	11.52
206437_at	sphingosine-1-pho	S1PR4	7.27	7.34	7.08
219207_at	enhancer of mRN	EDC3	7.24	7.03	7.19
210331_at	HECT, C2 and WW	HECW1	5.51	5.25	4.84
207367_at	ATPase, H+/K+ tra	ATP12A	5.32	5.24	5.22
204144_s_at	phosphatidylinosi	PIGQ	6.68	6.44	6.46
210962_s_at	A kinase (PRKA) a	AKAP9	8.32	8.35	8.59
213856_at	CD47 molecule	CD47	3.51	3.63	3.61
201911_s_at	FERM, RhoGEF (A	FARP1	4.70	4.40	4.60
201106_at	glutathione perox	GPX4	12.22	12.11	12.19
217785_s_at	YKT6 v-SNARE hor	YKT6	8.14	8.21	8.26
219780_at	zinc finger protei	ZNF771	3.77	3.73	3.53
215817_at	serpin peptidase i	SERPINB13	4.17	3.99	4.09
AFFX-BioC-3_at	---	---	11.05	11.05	11.08
38157_at	dom-3 homolog Z	DOM3Z	8.00	8.02	8.20

206942_s_at	pro-melanin-conc	PMCH	5.31	5.08	5.39
202231_at	eukaryotic transla	EIF3M	12.53	12.57	12.63
211571_s_at	versican	VCAN	5.00	5.06	5.15
218959_at	homeobox C10	HOXC10	8.91	8.86	8.71
212103_at	karyopherin alpha	KPNA6	9.15	9.18	9.20
212322_at	sphingosine-1-pho	SGPL1	8.74	8.51	8.79
211348_s_at	CDC14 cell divisio	CDC14B	4.33	4.03	4.31
37005_at	neuroblastoma, su	NBL1	8.76	8.63	8.62
202847_at	phosphatidylinosi	PIGC	9.90	9.47	9.95
216477_at	---	---	4.24	3.95	3.99
204635_at	ribosomal protein	RPS6KA5	5.71	5.80	6.03
207251_at	meprin A, beta	MEP1B	4.22	4.03	4.33
221839_s_at	ubiquitin associat	UBAP2	9.79	9.85	9.92
213203_at	small nuclear RNA	SNAPC5	10.37	10.32	10.38
200633_at	ubiquitin B	UBB	13.03	13.15	13.04
209783_at	D site of albumin	DBP	6.48	6.17	6.38
205851_at	non-metastatic ce	NME6	7.79	7.91	8.15
205335_s_at	signal recognition	SRP19	11.32	11.26	11.53
206003_at	centrosomal prote	CEP135	7.68	7.87	7.94
205362_s_at	prefoldin subunit	PFDN4	6.90	6.93	7.14
214867_at	---	---	6.75	6.99	6.81
207753_at	zinc finger protein	ZNF304	6.99	7.05	7.01
208113_x_at	poly(A) binding pr	PABPC3	13.60	13.57	13.70
219965_s_at	MAGI family mem	MAGIX	4.20	3.94	3.72
202485_s_at	methyl-CpG bindi	MBD2	4.23	4.10	3.84
201658_at	ADP-ribosylation f	ARL1	9.39	9.51	9.37
204609_at	coiled-coil domair	CCDC85B	4.09	3.67	3.95
201329_s_at	v-ets erythroblast	ETS2	9.01	8.92	9.31
218181_s_at	mitogen-activatec	MAP4K4	8.40	8.42	8.51
219591_at	cell cycle exit and	CEND1	7.64	7.52	7.46
203558_at	cullin 7	CUL7	7.17	7.32	7.12
213829_x_at	regulator of telom	RTEL1	7.59	7.47	7.63
218976_at	DnaJ (Hsp40) hom	DNAJC12	9.36	9.15	9.54
207823_s_at	allograft inflamma	AIF1	3.74	4.04	3.85
219820_at	solute carrier fam	SLC6A16	3.95	4.02	4.16
220177_s_at	transmembrane p	TMPRSS3	3.73	3.64	3.60
208046_at	histone cluster 1,	HIST1H4A	4.91	4.67	4.61
220157_x_at	pleckstrin homolo	PLEKHA8 /// PLEKH	7.51	7.41	7.35
216146_at	---	---	3.84	3.58	3.38
50221_at	transcription facto	TFEB	7.97	7.93	8.06
215328_at	EFR3 homolog B (S	EFR3B	6.17	6.26	5.85
221244_s_at	---	---	4.94	5.34	5.12
221422_s_at	chromosome 9 op	C9orf45	5.27	4.72	5.09
217298_at	---	---	5.38	5.02	4.96
216295_s_at	clathrin, light chai	CLTA	11.97	12.00	12.17
214561_at	leukocyte immuno	LILRP2	3.86	3.97	3.42
209961_s_at	hepatocyte growt	HGF	5.90	5.82	5.47

207962_at	calpain 11	CAPN11	5.75	5.64	5.41
211326_x_at	hemochromatosis	HFE	6.52	6.43	6.34
211613_s_at	glycerol-3-phosph	GPD2	5.32	5.75	5.64
212053_at	pyridoxal-depend	PDXDC1	9.05	9.02	8.95
203341_at	CCAAT/enhancer	CEBPZ	10.94	11.09	11.08
216918_s_at	dystonin	DST	7.07	7.05	7.01
214883_at	thyroid hormone	THRA	5.86	5.93	6.07
221789_x_at	ras homolog gene	RHOT2	8.84	8.77	8.77
213669_at	FCH domain only	FCHO1	6.73	6.79	6.64
210370_s_at	lymphocyte antige	LY9	4.31	4.26	4.25
215727_x_at	---	---	6.33	6.28	6.18
221015_s_at	cytidine and dCMI	CDADC1	4.79	4.72	4.62
218423_x_at	vacuolar protein s	VPS54	8.77	8.90	8.68
209300_s_at	NECAP endocytos	NECAP1	9.10	9.21	9.34
207558_s_at	paired-like homec	PITX2	4.75	5.03	4.96
214325_at	glycoprotein 2 (zy	GP2	4.69	4.38	4.79
217123_x_at	pro-melanin-conc	PMCHL1	4.13	4.12	4.05
41858_at	post-GPI attachm	PGAP2	8.05	8.03	8.10
209585_s_at	multiple inositol-p	MINPP1	8.82	8.94	9.07
54051_at	PBX/knotted 1 ho	PKNOX1	7.09	6.85	7.00
222058_at	---	---	5.16	4.88	5.00
221343_at	olfactory receptor	OR11A1	4.37	4.03	3.80
221354_s_at	melanin-concentr	MCHR1	4.34	4.24	4.12
200986_at	serpin peptidase i	SERPING1	5.50	5.34	5.66
204056_s_at	mevalonate kinas	MVK	7.72	7.83	7.66
216173_at	---	---	6.40	6.44	6.31
218652_s_at	phosphatidylinosi	PIGG	9.81	9.95	9.95
217320_at	---	---	3.79	3.86	4.22
217556_at	chloride channel 4	CLCN4	3.65	3.70	3.43
217977_at	selenoprotein X, 1	SEPX1	9.42	9.50	9.34
218038_at	ATP5S-like	ATP5SL	8.91	8.90	8.91
210261_at	potassium channe	KCNK2	4.64	4.50	4.39
218930_s_at	transmembrane p	TMEM106B	10.06	10.10	10.25
207315_at	CD226 molecule	CD226	3.41	3.44	3.19
202409_at	insulin-like growtl	IGF2 /// INS-IGF2	4.39	4.58	4.26
201909_at	ribosomal protein	RPS4Y1	5.86	5.74	5.76
207323_s_at	myelin basic prote	MBP	5.89	5.92	5.66
208023_at	tumor necrosis fa	TNFRSF4	6.18	6.08	6.03
210098_s_at	---	---	7.46	7.46	7.61
208153_s_at	FAT tumor suppre	FAT2	5.79	5.75	5.76
205785_at	integrin, alpha M	ITGAM	6.09	5.82	5.50
215862_at	---	---	4.63	4.60	4.63
209453_at	solute carrier fam	SLC9A1	7.11	7.02	6.98
206179_s_at	tubulin polymeriz	TPPP	5.95	5.82	6.08
201954_at	actin related prot	ARPC1B	10.63	10.52	10.63
207523_at	chromosome 6 op	C6orf10	3.26	3.35	3.00
203155_at	SET domain, bifur	SETDB1	7.80	7.89	7.77

216409_at	Acyl-CoA syntheta	ACSL6	6.91	6.90	6.62
218457_s_at	DNA (cytosine-5-)	DNMT3A	5.87	5.83	5.41
208462_s_at	ATP-binding casse	ABCC9	4.82	4.40	4.46
212660_at	PHD finger protei	PHF15	9.53	9.53	9.43
217605_at	ubiquitin specific	USP27X	4.87	5.05	4.85
211251_x_at	nuclear transcript	NFYC	8.25	8.27	8.11
220091_at	solute carrier fam	SLC2A6	8.37	8.48	8.38
219391_at	3'(2'), 5'-bisphosp	BPNT1	4.46	4.31	3.93
208124_s_at	sema domain, imr	SEMA4F	6.03	6.12	5.87
216270_at	ilvB (bacterial ace	ILVBL	6.02	5.83	5.94
210005_at	phosphoribosylgly	GART	8.20	8.20	8.46
204116_at	interleukin 2 rece	IL2RG	4.83	5.04	4.39
200942_s_at	heat shock factor	HSBP1	11.92	11.96	11.97
204932_at	tumor necrosis fa	TNFRSF11B	3.33	3.33	3.19
221642_at	---	---	6.44	6.64	6.42
206883_x_at	glycoprotein IX (p	GP9	7.89	7.95	7.62
214024_s_at	DiGeorge syndron	DGCR6L	6.42	6.53	6.33
202258_s_at	NEDD4 binding pr	N4BP2L2	10.43	10.37	10.52
218608_at	ATPase type 13A2	ATP13A2	6.27	6.35	5.94
218878_s_at	sirtuin (silent mat	SIRT1	8.57	8.73	8.95
205839_s_at	benzodiazapine re	BZRAP1	5.51	5.56	5.31
201633_s_at	cytochrome b5 ty	CYB5B	8.26	8.40	8.41
214593_at	protein inhibitor c	PIAS2	3.79	3.56	3.55
206457_s_at	deiodinase, iodotl	DIO1	3.95	3.68	3.68
216360_x_at	ribosomal RNA pr	RRP12	6.81	7.02	7.13
209925_at	occludin	OCLN	4.57	4.61	5.04
202150_s_at	neural precursor c	NEDD9	5.79	6.14	5.98
205724_at	plakophilin 1 (ecto	PKP1	5.30	5.09	4.80
208595_s_at	methyl-CpG bindi	MBD1	8.35	8.21	8.37
214636_at	calcitonin-related	CALCB	8.88	8.24	8.84
219801_at	zinc finger protein	ZNF34	6.53	6.24	6.49
211940_x_at	H3 histone, family	H3F3A /// LOC440	13.11	13.15	13.09
206282_at	neurogenic differe	NEUROD1	3.12	3.28	3.31
203194_s_at	nucleoporin 98kD	NUP98	7.76	7.78	7.79
219466_s_at	apolipoprotein A-I	APOA2	4.36	4.59	4.29
202646_s_at	cold shock domai	CSDE1	11.03	11.05	11.12
202136_at	zinc finger, MYND	ZMYND11	10.49	10.51	10.48
209072_at	myelin basic prote	MBP	3.95	3.95	3.99
221141_x_at	epsin 1	EPN1	7.86	7.61	7.48
207602_at	transmembrane p	TMPRSS11D	6.33	6.46	6.07
220652_at	kinesin family me	KIF24	4.03	4.07	3.73
210185_at	calcium channel, v	CACNB1	7.04	6.80	6.90
205611_at	tumor necrosis fa	TNFSF12	6.47	6.27	6.39
211888_x_at	caspase 10, apopt	CASP10	6.47	6.48	6.05
214785_at	vacuolar protein s	VPS13A	7.45	7.38	7.44
222315_at	---	---	4.49	4.67	4.34
208617_s_at	protein tyrosine p	PTP4A2	12.01	11.97	12.06

216177_at	---	---	8.18	8.19	8.32
206654_s_at	polymerase (RNA)	POLR3G	5.63	5.60	5.68
210233_at	interleukin 1 rece	IL1RAP	6.86	6.83	7.17
201984_s_at	epidermal growth	EGFR	9.61	9.35	9.61
41113_at	zinc finger protein	ZNF500	7.34	7.14	7.32
216439_at	tyrosine kinase, n	TNK2	7.50	7.64	7.22
208983_s_at	platelet/endothel	PECAM1	4.44	4.23	4.35
211480_s_at	solute carrier orga	SLCO1A2	5.16	5.21	4.90
212867_at	---	---	8.93	8.93	9.11
202701_at	bone morphogene	BMP1	7.72	7.63	7.68
221255_s_at	transmembrane p	TMEM93	9.98	9.97	9.72
222205_x_at	family with seque	FAM182B	3.87	3.81	3.85
213750_at	ribosomal L1 dom	RSL1D1	8.54	8.57	8.64
208297_s_at	ecotropic viral int	EVI5	5.24	5.56	5.42
213817_at	interleukin-1 rece	IRAK3	3.46	3.31	3.22
207285_x_at	chorionic somato	CSHL1	3.66	3.78	3.85
207159_x_at	CREB regulated tr	CRTC1	7.91	7.58	7.55
206399_x_at	calcium channel, v	CACNA1A	7.87	7.83	7.45
211583_x_at	natural cytotoxicit	NCR3	5.96	6.04	6.14
210807_s_at	solute carrier fam	SLC16A7	7.02	7.04	7.15
212974_at	DENN/MADD dom	DENND3	5.57	5.64	5.58
221941_at	polyamine oxidas	PAOX	6.75	6.79	6.73
204574_s_at	matrix metallope	MMP19	7.59	7.42	7.41
211196_at	dihydrolipoamide	DBT	3.84	3.96	3.85
209326_at	solute carrier fam	SLC35A2	8.79	8.69	8.97
210642_at	calicin	CCIN	6.27	6.28	6.19
210105_s_at	FYN oncogene rel	FYN	9.34	9.03	9.23
215448_at	---	---	3.68	3.39	3.60
208132_x_at	HLA-B associated	BAT2	8.47	8.32	8.58
207895_at	N-acetylated alph	NAALADL1	5.65	5.98	5.95
200646_s_at	nucleobindin 1	NUCB1	5.90	5.48	5.22
214154_s_at	plakophilin 2	PKP2	4.96	4.82	4.93
205182_s_at	zinc finger protein	ZNF324	7.40	7.49	7.36
217273_at	PRAME family me	PRAMEF10	5.59	5.55	5.27
202543_s_at	glia maturation fa	GMFB	8.55	8.85	8.75
207698_at	chromosome 6 op	C6orf123	4.42	4.66	4.16
214340_at	arachidonate 12-l	ALOX12P2	5.37	5.80	5.16
221171_at	coiled-coil domain	CCDC30	4.11	4.41	4.17
212381_at	ubiquitin specific	USP24	8.48	8.46	8.51
213122_at	TSPY-like 5	TSPYL5	3.70	3.60	3.52
201381_x_at	calcyclin binding p	CACYBP	11.62	11.70	11.85
208222_at	activin A receptor	ACVR1B	4.33	4.47	4.36
202629_at	amyloid beta prec	APPBP2	8.50	8.66	8.64
211141_s_at	CCR4-NOT transcr	CNOT3	4.07	4.18	4.23
205418_at	feline sarcoma on	FES	6.39	6.38	6.23
207870_at	A kinase (PRKA) a	AKAP9	3.95	3.75	3.58
208534_s_at	similar to calcium	LOC100132214 ///	4.18	4.19	4.21

209745_at	coenzyme Q7 hon	COQ7	7.45	6.96	7.28
204892_x_at	eukaryotic transla	EEF1A1	15.29	15.27	15.24
219955_at	LINE-1 type transp	L1TD1	4.16	3.92	4.05
222178_s_at	---	---	3.25	3.42	3.32
205417_s_at	dystroglycan 1 (dy	DAG1	10.51	10.44	10.34
205044_at	gamma-aminobut	GABRP	3.37	3.40	3.78
215594_at	pTR7 repetitive se	---	4.53	4.46	4.62
211995_x_at	actin, gamma 1	ACTG1	14.17	14.18	14.06
206629_at	ADAMTS-like 2	ADAMTSL2	6.14	5.99	5.65
213317_at	chloride intracellu	CLIC5	3.30	3.18	3.02
221609_s_at	wingless-type MM	WNT6	5.94	5.77	5.55
207041_at	mannan-binding l	MASP2	5.79	5.52	5.45
210803_at	thioredoxin reduc	TXNRD2	6.62	6.36	6.29
215378_at	---	---	3.52	3.61	3.45
221296_at	tectorin alpha	TECTA	4.70	4.83	4.32
220679_s_at	cadherin 7, type 2	CDH7	4.41	4.49	4.54
219723_x_at	1-acylglycerol-3-p	AGPAT3	6.87	6.74	6.34
208166_at	matrix metallope	MMP16	4.08	4.25	4.23
217342_x_at	hypothetical prot	FLJ11292	6.07	5.82	5.85
207838_x_at	pre-B-cell leukem	PBXIP1	6.14	6.10	5.42
215215_s_at	exonuclease NEF-	LOC81691	7.20	7.57	7.43
206432_at	hyaluronan synth	HAS2	3.43	3.10	3.12
219020_at	HCLS1 binding pro	HS1BP3	8.31	8.29	8.13
217939_s_at	aftiphilin	AFTPH	9.23	9.21	9.22
211157_at	---	---	7.16	6.94	7.01
208490_x_at	histone cluster 1,	HIST1H2BF	7.91	7.73	7.84
205793_x_at	tyrosine kinase, n	TNK1	5.20	5.09	5.21
220654_at	pancreatic polype	PPY2	4.38	4.67	4.68
206701_x_at	endothelin recept	EDNRB	7.62	7.61	7.79
201216_at	endoplasmic retic	ERP29	10.61	10.47	10.73
216248_s_at	nuclear receptor s	NR4A2	5.76	5.78	5.96
207395_at	butyrophilin, subf	BTN1A1	4.07	4.07	3.72
215119_at	myosin XVI	MYO16	4.52	4.12	4.01
211620_x_at	runt-related trans	RUNX1 /// SH3D19	6.05	6.02	5.95
220402_at	tumor protein p5	TP53AIP1	3.36	3.10	3.17
219870_at	activating transcri	ATF7IP2	5.35	5.19	5.43
216433_s_at	PR domain contai	PRDM2	4.85	4.80	4.62
203947_at	cleavage stimulati	CSTF3	9.63	9.78	9.62
217226_s_at	sideroflexin 3	SFXN3	8.48	8.64	8.66
205491_s_at	gap junction prot	GJB3	4.13	4.36	4.03
211272_s_at	diacylglycerol kin	DGKA	5.27	5.33	5.54
211943_x_at	tumor protein, tra	TPT1	14.84	14.81	14.86
217983_s_at	ribonuclease T2	RNASET2	8.29	8.21	8.34
219070_s_at	motile sperm dom	MOSPD3	7.62	7.74	7.65
215662_at	---	---	3.98	4.09	4.50
211102_s_at	leukocyte immun	LILRA2	5.68	5.29	5.33
219402_s_at	Der1-like domain	DERL1	10.21	10.13	10.13

208603_s_at	mitogen-activatec	MAPK8IP2	6.59	6.67	6.08
215687_x_at	phospholipase C, I	PLCB1	4.74	4.80	4.54
215583_at	Transmembrane p	TMEM63A	7.08	6.93	7.04
215001_s_at	glutamate-ammo	GLUL	13.02	13.04	13.10
220848_x_at	odorant binding p	OBP2A	7.49	7.25	6.88
214671_s_at	active BCR-relatec	ABR	5.88	5.56	5.75
208467_at	Kruppel-like facto	KLF12	4.99	4.83	4.58
216393_at	chromosome 10 c	C10orf12	4.91	4.96	4.74
216415_at	Dynein, axonemal	DNAH3	3.17	3.53	3.13
221913_at	sirtuin (silent mat	SIRT3	7.76	7.79	7.50
207521_s_at	ATPase, Ca++ tran	ATP2A3	4.66	4.85	4.92
221281_at	v-src sarcoma (Sch	SRC	6.03	6.01	5.98
208128_x_at	kinesin family mei	KIF25	7.01	7.25	6.86
207955_at	chemokine (C-C m	CCL27	4.99	4.94	4.72
214284_s_at	Fibroblast growth	FGF18	5.17	4.79	4.92
201071_x_at	splicing factor 3b,	SF3B1	11.83	11.94	11.92
209446_s_at	chromosome 7 op	C7orf44	7.66	7.72	7.71
216196_at	---	---	4.20	4.20	3.89
46665_at	sema domain, imr	SEMA4C	8.47	8.49	8.52
218775_s_at	WW and C2 doma	WWC2	7.86	7.76	8.08
206753_at	retinol dehydroge	RDH16	5.29	4.90	4.71
202991_at	StAR-related lipid	STARD3	7.42	7.56	7.36
220053_at	growth differentia	GDF3	5.17	5.54	5.07
204305_at	mitochondrial inte	MIPEP	8.66	8.71	8.71
213571_s_at	eukaryotic transla	EIF4E2	10.15	10.28	10.21
218277_s_at	DEAH (Asp-Glu-Ala	DHX40	9.48	9.63	9.71
207371_at	---	---	3.74	3.69	3.64
208393_s_at	RAD50 homolog (S	RAD50	9.88	9.91	10.10
204115_at	guanine nucleotid	GNG11	10.91	10.99	11.10
221663_x_at	histamine recepto	HRH3	8.31	8.17	7.99
207003_at	guanylate cyclase	GUCA2A	6.42	6.08	5.86
211622_s_at	ADP-ribosylation f	ARF3	7.64	7.76	7.72
201254_x_at	ribosomal protein	RPS6	14.84	14.85	14.93
213890_x_at	ribosomal protein	RPS16	14.94	14.95	14.94
217980_s_at	mitochondrial ribo	MRPL16	10.46	10.47	10.37
212492_s_at	lysine (K)-specific	KDM4B	9.53	9.52	9.33
217151_at	---	---	5.06	4.75	4.38
208207_at	---	---	4.20	3.67	4.13
207474_at	SNF related kinase	SNRK	3.81	4.41	4.11
208392_x_at	SP110 nuclear bod	SP110	6.95	7.10	6.80
211974_x_at	recombination sig	RBPJ	11.35	11.35	11.44
200042_at	chromosome 22 c	C22orf28	10.35	10.39	10.46
209870_s_at	amyloid beta (A4)	APBA2	8.75	8.71	8.70
216017_s_at	NGFI-A binding pr	NAB2	6.20	5.82	6.07
218282_at	ER degradation er	EDEM2	7.79	7.61	7.80
219785_s_at	F-box protein 31 /	FBXO31 /// LOC10	7.22	7.22	7.19
213482_at	dedicator of cytok	DOCK3	4.95	4.53	4.50

205287_s_at	transcription facto	TFAP2C		4.86	5.17	4.72
205879_x_at	ret proto-oncoger	RET		5.97	5.96	5.71
201196_s_at	adenosylmethioni	AMD1		9.29	9.37	9.57
215472_at	PARK2 co-regulate	PACRG		4.64	4.69	4.34
206819_at	POM121 membra	POM121L9P		3.86	4.05	3.44
205739_x_at	zinc finger proteir	ZNF107		5.14	5.75	5.61
41657_at	serine/threonine l	STK11		7.67	7.71	7.59
210024_s_at	ubiquitin-conjugat	UBE2E3		10.92	10.98	10.97
205604_at	homeobox D9	HOXD9		6.63	6.39	6.26
214569_at	interferon, alpha !	IFNA5		3.07	3.20	3.34
211564_s_at	PDZ and LIM dom	PDLIM4		8.76	8.52	8.67
210075_at	membrane-associ		2-Mar	7.02	7.18	7.02
208223_s_at	activin A receptor	ACVR1B		4.24	4.26	3.86
213550_s_at	transmembrane a	TMCO6		7.21	7.30	7.21
220469_at	coatomer protein	COPE		6.12	6.13	5.99
203624_at	splicing factor, arg	SFRS17A		9.03	9.14	9.01
219203_at	family with seque	FAM158A		9.24	9.12	9.12
217731_s_at	integral membran	ITM2B		9.55	9.67	9.74
219589_s_at	transmembrane p	TMEM143		7.13	7.12	7.07
214441_at	syntaxin 6	STX6		6.14	5.96	5.95
211797_s_at	nuclear transcript	NFYC		7.35	7.33	7.41
207013_s_at	matrix metallopep	MMP16		5.66	5.89	5.52
212501_at	CCAAT/enhancer l	CEBPB		12.76	12.64	12.84
209245_s_at	kinesin family mei	KIF1C		5.57	5.42	5.22
203040_s_at	hydroxymethylbila	HMBS		10.42	10.31	10.40
214792_x_at	vesicle-associated	VAMP2		3.79	3.95	3.12
AFFX-PheX-5_at	---	---		7.50	7.25	7.31
37549_g_at	Bardet-Biedl synd	BBS9		6.15	6.24	6.37
208844_at	voltage-depender	VDAC3		6.29	6.47	6.57
214237_x_at	PRKC, apoptosis, \	PAWR		7.56	7.54	7.48
202236_s_at	solute carrier fam	SLC16A1		10.73	10.77	10.93
210599_at	zinc finger proteir	ZNF614		5.56	5.50	5.30
215985_at	Non-protein codir	NCRNA00171		6.33	6.49	6.58
215988_s_at	discs, large homol	DLG1		5.70	5.39	5.47
217577_at	---	---		3.83	3.45	3.38
216453_at	zinc finger proteir	ZNF287		4.27	3.95	4.03
216106_at	hypothetical prote	LOC145678		4.53	4.68	4.34
32836_at	1-acylglycerol-3-p	AGPAT1		9.45	9.53	9.30
216135_at	IQ motif containir	IQCK		3.52	3.52	3.61
215827_x_at	ciliary rootlet coil	CROCCL2		6.35	6.40	6.08
202616_s_at	methyl CpG bindir	MECP2		7.38	7.41	7.20
214765_s_at	N-acylethanolami	NAAA		7.34	7.17	7.48
207420_at	collectin sub-fami	COLEC10		4.36	4.93	4.63
219263_at	ring finger proteir	RNF128		3.38	3.13	3.30
206040_s_at	mitogen-activatec	MAPK11		4.87	4.95	4.99
212592_at	immunoglobulin J	IGJ		3.22	3.29	3.03
204704_s_at	aldolase B, fructos	ALDOB		3.11	3.08	3.22

213319_s_at	Cold shock domai	CSDA	5.95	5.75	5.48
204979_s_at	SH3 domain bindi	SH3BGR	6.93	6.93	6.91
200931_s_at	vinculin	VCL	10.23	10.51	10.37
215910_s_at	fibronectin type II	FNDC3A	5.10	5.35	5.36
216476_at	similar to seven tr	LOC442381 /// LO	5.30	5.43	4.58
211081_s_at	mitogen-activatec	MAP4K5	5.88	6.03	6.06
205718_at	integrin, beta 7	ITGB7	7.63	7.56	7.49
214562_at	histone cluster 1,	HIST1H4L	4.80	4.58	4.46
204395_s_at	G protein-couplec	GRK5	6.64	6.67	6.79
209587_at	paired-like homec	PITX1	7.62	7.22	7.35
219802_at	pyridine nucleotic	PYROXD1	8.85	9.12	9.22
202672_s_at	activating transcri	ATF3	8.59	8.39	8.82
201039_s_at	RAD23 homolog A	RAD23A	9.23	9.10	9.19
215584_at	HECT, C2 and WW	HECW1	6.63	6.78	6.44
217077_s_at	gamma-aminobut	GABBR2	3.81	4.24	3.78
217409_at	---	---	4.80	4.89	4.63
201230_s_at	ariadne homolog :	ARIH2	9.58	9.61	9.63
215277_at	protocadherin 1	PCDH1	4.65	4.69	4.62
207406_at	cytochrome P450,	CYP7A1	3.26	3.04	3.27
202225_at	---	---	9.09	9.13	9.27
218919_at	zinc finger, AN1-ty	ZFAND1	10.22	10.14	10.39
218513_at	chromosome 4 op	C4orf43	7.37	7.33	7.76
208610_s_at	serine/arginine re	SRRM2	10.72	10.77	10.57
61734_at	reticulocalbin 3, E	RCN3	7.62	7.49	7.53
203746_s_at	holocytochrome c	HCCS	9.94	9.88	9.79
208845_at	voltage-depender	VDAC3	12.02	12.19	12.18
219441_s_at	leucine-rich repea	LRRK1	8.50	8.26	8.22
204576_s_at	clusterin associate	CLUAP1	8.64	8.49	8.58
206395_at	diacylglycerol kina	DGKG	4.35	4.48	4.34
201178_at	F-box protein 7	FBXO7	10.38	10.26	10.34
207277_at	CD209 molecule	CD209	6.32	6.38	6.36
210554_s_at	C-terminal binding	CTBP2	11.47	11.41	11.41
218985_at	solute carrier fam	SLC2A8	7.84	7.57	7.79
200758_s_at	nuclear factor (ery	NFE2L1	10.18	9.72	10.13
205635_at	kalirin, RhoGEF kin	KALRN	3.42	3.75	3.47
217154_s_at	endothelin 3	EDN3	4.67	4.53	4.88
207546_at	ATPase, H+/K+ exc	ATP4B	4.48	4.40	4.39
214972_at	Meningioma expr	MGEA5	5.54	5.44	5.74
211303_x_at	folate hydrolase 1	FOLH1B	4.23	4.03	3.87
215264_at	empty spiracles ho	EMX1	4.18	3.85	3.80
204830_x_at	pregnancy specific	PSG5	6.54	6.41	6.53
219275_at	programmed cell	PDCD5	10.56	10.58	10.70
214445_at	elongation factor,	ELL2	4.77	4.74	4.71
208872_s_at	receptor accessor	REEP5	10.95	10.95	10.87
202612_s_at	mediator complex	MED14	3.56	3.46	3.14
206389_s_at	phosphodiesteras	PDE3A	3.81	3.98	3.56
220637_at	family with seque	FAM124B	3.99	3.68	3.48

205770_at	glutathione reduc	GSR	7.25	7.19	7.47
206470_at	plexin C1	PLXNC1	4.14	3.91	4.14
222140_s_at	G protein-couplec	GPR89A /// GPR89	8.54	8.65	8.73
201525_at	apolipoprotein D	APOD	4.38	4.88	5.11
210428_s_at	hepatocyte growt	HGS	8.59	8.48	8.45
211328_x_at	hemochromatosis	HFE	7.36	7.28	6.88
205379_at	carbonyl reductas	CBR3	8.29	8.42	8.33
215049_x_at	CD163 molecule	CD163	5.15	4.68	4.62
208722_s_at	anaphase promot	ANAPC5	9.73	9.77	9.95
201270_x_at	NudC domain con	NUDCD3	9.11	9.06	9.07
220252_x_at	chromosome X op	CXorf21	6.12	6.13	6.26
213411_at	---	---	4.72	4.58	3.95
218329_at	PR domain contai	PRDM4	9.65	9.64	9.59
209671_x_at	T cell receptor alp	TRA@ /// TRAC	6.39	6.51	6.30
222277_at	prostate collagen	PCOTH	5.94	6.04	6.32
200963_x_at	ribosomal protein	RPL31	14.72	14.72	14.78
209342_s_at	inhibitor of kappa	IKBKB	7.63	7.58	7.65
204074_s_at	KIAA0562	KIAA0562	8.16	8.18	8.10
202801_at	solute carrier fam	SLC1A3	8.29	8.37	8.22
222265_at	tensin 4	TNS4	6.65	6.80	6.74
210203_at	CCR4-NOT transcr	CNOT4	5.43	5.68	5.40
222154_s_at	spermatogenesis a	SPATS2L	11.06	11.03	11.12
219328_at	DEAD (Asp-Glu-Ala	DDX31	9.03	8.83	8.88
217648_at	---	---	4.93	5.06	4.46
200930_s_at	vinculin	VCL	5.75	5.46	5.38
221529_s_at	plasmalemma ves	PLVAP	5.39	5.38	5.14
206427_s_at	melan-A	MLANA	4.70	5.22	4.82
207532_at	crystallin, gamma	CRYGD	6.00	6.04	5.79
207811_at	keratin 12	KRT12	3.49	3.21	3.23
212729_at	discs, large homol	DLG3	5.95	5.51	5.79
207969_x_at	acrosomal vesicle	ACRV1	4.86	4.79	4.55
212610_at	protein tyrosine p	PTPN11	12.38	12.60	12.64
201414_s_at	nucleosome asser	NAP1L4	9.51	9.53	9.60
219706_at	chromosome 20 o	C20orf29	8.45	8.20	8.23
221069_s_at	translational activ	TACO1	9.18	9.21	9.20
210507_s_at	advillin	AVIL	5.53	5.09	5.11
220247_at	cancer susceptibil	CASC5	3.97	3.84	3.98
215233_at	jumonji domain co	JMJD6	5.48	5.44	5.48
215120_s_at	sterile alpha moti	SAMD4A	5.87	5.66	5.73
201922_at	NSA2 ribosome bi	NSA2	13.01	13.07	13.09
209365_s_at	extracellular matr	ECM1	6.63	6.78	6.87
202943_s_at	N-acetylgalactosa	NAGA	7.66	7.57	7.63
201363_s_at	influenza virus NS	IVNS1ABP	8.70	8.66	8.81
204398_s_at	echinoderm micro	EML2	7.69	7.34	7.31
216020_at	Interferon induced	IFIH1	3.82	3.72	3.59
203437_at	transmembrane p	TMEM11	8.84	8.75	8.74
213761_at	Mdm1 nuclear pro	MDM1	6.94	7.16	7.37

219206_x_at	transmembrane B	TMBIM4	9.03	9.25	9.09
203887_s_at	thrombomodulin	THBD	8.47	8.43	8.73
218609_s_at	nudix (nucleoside	NUDT2	8.79	8.70	8.72
213726_x_at	tubulin, beta 2C	TUBB2C	11.70	11.89	11.75
213713_s_at	galactosidase, bet	GLB1L2	7.37	7.61	7.02
217794_at	proline rich 13	PRR13	10.36	10.35	10.36
204147_s_at	transcription facto	TFDP1	7.96	7.96	7.90
214646_at	Histone cluster 1,	HIST1H3J	5.09	5.00	5.17
204547_at	RAB40B, member	RAB40B	7.97	8.13	8.16
216917_s_at	synaptonemal cor	SYCP1	3.49	3.08	3.28
220567_at	IKAROS family zin	IKZF2	5.71	5.34	5.53
217875_s_at	prostate transmer	PMEPA1	7.31	7.19	7.25
219037_at	ribosomal RNA pr	RRP15	9.42	9.33	9.53
216358_at	---	---	5.57	5.37	5.69
210563_x_at	CASP8 and FADD-	CFLAR	10.84	10.66	10.79
210952_at	adaptor-related p	AP4S1	6.99	6.93	6.89
213924_at	Metallophosphoe	MPPE1	4.44	4.31	4.22
216474_x_at	tryptase alpha/be	TPSAB1 /// TPSB2	6.31	6.30	6.10
217546_at	metallothionein 1	MT1M	3.57	3.72	3.35
213045_at	microtubule assoc	MAST3	6.94	6.96	6.94
208320_at	calcium binding p	CABP1	5.76	5.30	5.66
216631_s_at	---	---	6.26	6.09	5.91
205515_at	protease, serine, :	PRSS12	4.02	3.94	3.80
210759_s_at	proteasome (pros	PSMA1	11.55	11.59	11.57
200802_at	seryl-tRNA synthe	SARS	11.11	10.79	11.03
211952_at	importin 5	IPO5	8.70	8.66	8.76
205414_s_at	Rho-type GTPase-	RICH2	5.42	5.21	5.04
214925_s_at	spectrin, alpha, no	SPTAN1	6.41	6.29	6.00
220339_s_at	tryptase gamma 1	TPSG1	7.74	7.75	7.37
203299_s_at	adaptor-related p	AP1S2	8.61	8.81	8.79
210106_at	retinol dehydroge	RDH5	7.41	7.45	7.01
203742_s_at	thymine-DNA glyc	TDG	7.61	8.01	7.70
221505_at	acidic (leucine-ricl	ANP32E	10.54	10.65	10.75
221600_s_at	chromosome 11 c	C11orf67	7.90	7.89	8.02
212925_at	chromosome 19 c	C19orf21	6.92	6.85	7.06
36084_at	cullin 7	CUL7	7.93	8.11	7.93
200661_at	cathepsin A	CTSA	9.30	9.29	9.26
208118_x_at	SMG1 homolog, p	LOC100271836 ///	8.23	8.14	8.20
202355_s_at	general transcript	GTF2F1	8.52	8.43	8.55
208154_at	---	---	5.75	5.90	5.64
211679_x_at	gamma-aminobut	GABBR2	6.06	5.47	5.33
218841_at	ankyrin repeat an	ASB8	7.20	7.40	7.50
217739_s_at	nicotinamide pho:	NAMPT	10.40	10.33	10.55
220420_at	lectin, mannose-b	LMAN1L	7.34	7.25	6.83
214766_s_at	AT hook containin	AHCTF1	8.72	8.82	8.94
217782_s_at	G protein pathwa	GPS1	8.22	8.14	8.05
60471_at	Ras and Rab inter	RIN3	7.80	7.61	7.57

206514_s_at	cytochrome P450,	CYP4F2 /// CYP4F3	3.99	3.99	3.63
210808_s_at	NADPH oxidase 1	NOX1	6.17	6.04	6.16
206460_at	adherens junction	AJAP1	6.35	6.37	6.56
65493_at	HEAT repeat cont	HEATR6	8.52	8.36	8.54
66053_at	heterogeneous nu	HNRNPUL2	6.48	6.10	6.31
206630_at	tyrosinase (oculoc	TYR	6.55	6.38	6.15
207157_s_at	guanine nucleotid	GNG5	11.78	11.74	11.84
204135_at	filamin A interacti	FILIP1L	3.88	3.93	3.87
213143_at	chromosome 2 op	C2orf72	6.53	6.30	6.22
205663_at	poly(rC) binding p	PCBP3	6.54	6.48	6.55
221091_at	insulin-like 5	INSL5	3.98	4.04	4.11
218785_s_at	RAB, member RAS	RABL5	5.39	5.63	5.57
218544_s_at	RNA terminal pho	RCL1	8.57	8.45	8.78
206428_s_at	---	---	5.12	4.99	4.68
214383_x_at	kelch domain con	KLHDC3	7.57	7.51	7.43
47571_at	zinc finger protein	ZNF236	7.69	7.89	7.54
204652_s_at	nuclear respirator	NRF1	7.01	6.92	6.87
212798_s_at	ankyrin repeat an	ANKMY2	9.36	9.54	9.38
220738_s_at	ribosomal protein	RPS6KA6	4.04	4.84	4.15
211434_s_at	chemokine (C-C m	CCRL2	5.10	5.36	5.27
217109_at	mucin 4, cell surfa	MUC4	4.54	3.97	4.57
217264_s_at	sodium channel, r	SCNN1A	6.97	6.73	6.86
201855_s_at	ATM interactor	ATMIN	8.40	8.47	8.56
205029_s_at	fatty acid binding	FABP7	3.60	3.80	3.39
213753_x_at	eukaryotic transla	EIF5A	9.72	9.71	9.64
221246_x_at	tensin 1	TNS1	5.58	5.28	5.23
212049_at	WAS/WASL intera	WIPF2	8.02	8.02	8.01
203757_s_at	carcinoembryonic	CEACAM6	4.12	3.98	3.78
214813_at	zinc finger protein	ZNF75D	5.81	5.67	5.31
216390_at	Lecithin-cholester	LCAT	5.77	5.71	5.58
203552_at	mitogen-activatec	MAP4K5	7.36	7.63	7.81
220663_at	interleukin 1 rece	IL1RAPL1	4.40	4.21	4.00
219585_at	coiled-coil domair	CCDC28B	5.90	5.97	5.46
210148_at	homeodomain int	HIPK3	4.67	4.88	4.96
202933_s_at	v-yes-1 Yamaguch	YES1	10.68	10.86	10.98
218145_at	tribbles homolog	TRIB3	11.92	11.38	11.95
215382_x_at	tryptase alpha/be	TPSAB1	6.68	6.59	6.33
202741_at	protein kinase, cA	PRKACB	9.13	9.22	9.29
210409_at	chromosome 6 op	C6orf124	3.39	3.59	3.43
215084_s_at	leucine rich repea	LRRC42	10.04	9.92	9.90
211834_s_at	tumor protein p63	TP63	5.31	5.27	5.25
208819_at	RAB8A, member F	RAB8A	9.36	9.45	9.44
213690_s_at	---	---	7.38	7.12	7.28
217458_at	---	---	5.79	5.53	5.28
211108_s_at	Janus kinase 3	JAK3	4.24	4.05	4.18
209295_at	tumor necrosis fa	TNFRSF10B	11.35	11.13	11.48
205640_at	aldehyde dehydro	ALDH3B1	7.98	7.86	8.01

220896_at	F-box and leucine	FBXL18	8.13	7.83	7.90
206320_s_at	SMAD family men	SMAD9	6.05	6.13	6.25
217952_x_at	PHD finger protei	PHF3	8.86	8.93	9.22
215795_at	myosin, heavy cha	MYH7B	3.83	3.98	3.73
219231_at	trimethylguanosir	TGS1	8.56	8.56	8.70
208830_s_at	suppressor of Ty 6	SUPT6H	6.88	6.81	6.66
216336_x_at	metallothionein 1	MT1E	12.17	12.27	12.22
204625_s_at	integrin, beta 3 (p	ITGB3	6.02	6.15	5.75
215351_at	RNA terminal pho	RTCD1	3.69	4.04	4.07
215516_at	laminin, beta 4	LAMB4	3.33	3.30	3.31
220079_s_at	ubiquitin specific	USP48	9.86	9.93	9.84
203229_s_at	CDC-like kinase 2	CLK2	9.32	9.48	9.33
211552_s_at	aldehyde dehydro	ALDH4A1	7.48	7.52	7.42
212301_at	Rtf1, Paf1/RNA pc	RTF1	9.41	9.54	9.57
204584_at	L1 cell adhesion m	L1CAM	4.44	4.38	4.38
220971_at	interleukin 25	IL25	6.46	6.57	6.35
215960_at	solute carrier fam	SLC5A4	4.26	4.12	3.87
207634_at	programmed cell	PDCD1	7.57	7.55	7.41
204114_at	nidogen 2 (osteon	NID2	4.06	4.20	3.93
203980_at	fatty acid binding	FABP4	5.81	5.60	5.48
215857_at	nicalin homolog (z	NCLN	6.27	6.04	6.15
201800_s_at	oxysterol binding	OSBP	9.50	9.41	9.33
216216_at	slit homolog 3 (Dr	SLIT3	4.58	4.38	4.36
210460_s_at	proteasome (pros	PSMD4	10.94	10.83	10.84
207252_at	inactivation escap	INE1	6.40	6.53	6.36
219292_at	THAP domain con	THAP1	8.66	8.63	8.89
214856_at	spectrin, beta, no	SPTBN1	5.26	5.20	4.99
208648_at	valosin-containing	VCP	8.50	8.88	8.63
213613_s_at	---	---	6.72	6.60	6.29
207012_at	matrix metallopep	MMP16	4.04	4.04	3.96
208516_at	melatonin recepto	MTNR1B	5.89	5.96	5.60
210072_at	chemokine (C-C m	CCL19	5.22	5.35	5.08
203334_at	DEAH (Asp-Glu-Ala	DHX8	8.04	8.08	8.04
205810_s_at	Wiskott-Aldrich sy	WASL	6.32	6.24	5.87
207887_s_at	calcitonin recepto	CALCR	5.60	5.48	4.93
216368_s_at	collagen, type IV,	COL4A3	4.70	4.38	4.17
204051_s_at	secreted frizzled-r	SFRP4	4.53	4.70	4.41
207253_s_at	ubinuclein 1	UBN1	8.99	8.93	8.94
210278_s_at	adaptor-related p	AP4S1	6.56	6.80	6.90
210191_s_at	putative homeodo	PHTF1	7.88	8.02	7.90
214910_s_at	apolipoprotein M	APOM	5.99	5.56	5.92
221705_s_at	suppressor of IKBI	SIKE1	6.65	6.59	6.71
221711_s_at	chromosome 19 c	C19orf62	10.43	10.43	10.34
219840_s_at	T-cell leukemia/ly	TCL6	3.55	3.64	3.65
212768_s_at	olfactomedin 4	OLFM4	3.41	3.60	3.41
215558_at	ubiquitin protein l	UBR2	4.31	4.79	4.49
219538_at	WD repeat domai	WDR5B	7.58	7.80	7.68

216156_at	reversion-inducing	RECK	4.03	3.81	3.71
221398_at	taste receptor, typ	TAS2R8	3.29	3.51	3.27
210034_s_at	ribosomal protein	RPL5	5.70	5.86	5.76
202273_at	platelet-derived g	PDGFRB	7.38	6.98	7.22
222117_s_at	aarF domain conta	ADCK2	4.51	4.80	4.54
213106_at	ATPase, aminopho	ATP8A1	6.38	6.54	6.58
215639_at	SH2 domain conta	SH2D3C	3.69	3.84	3.46
216773_at	---	---	3.67	3.91	4.05
203950_s_at	chloride channel 6	CLCN6	7.77	7.88	7.83
210315_at	synapsin II	SYN2	4.27	4.18	4.17
206584_at	lymphocyte antige	LY96	7.23	7.36	7.44
212591_at	RNA binding moti	RBM34	10.35	10.37	10.46
220017_x_at	cytochrome P450,	CYP2C9	3.55	3.87	3.76
203953_s_at	claudin 3	CLDN3	4.83	4.74	4.80
210269_s_at	splicing factor, arg	SFRS17A	6.43	6.61	6.47
216168_at	---	---	3.66	3.57	3.31
213858_at	zinc finger protein	ZNF250	6.96	6.76	7.14
213605_s_at	---	---	9.49	9.74	9.39
219980_at	chromosome 4 op	C4orf29	6.01	6.10	6.08
216194_s_at	tubulin folding co	TBCB	10.63	10.55	10.60
216616_at	---	---	3.73	3.45	3.82
203934_at	kinase insert dom	KDR	6.93	6.97	6.59
213126_at	mediator complex	MED8	7.58	7.46	7.75
221380_at	---	---	4.83	4.90	5.16
221786_at	chromosome 6 op	C6orf120	9.26	9.27	9.52
207214_at	serine peptidase i	SPINK4	5.95	6.18	5.81
39966_at	chondroitin sulfat	CSPG5	5.48	5.63	5.79
221868_at	poly(A) binding pr	PAIP2B	6.52	6.21	6.22
207140_at	alkaline phosphat	ALPI	6.26	6.12	5.96
221442_at	melanocortin 3 re	MC3R	4.45	4.18	4.57
217098_s_at	zinc finger and SC	ZSCAN12	3.77	3.55	3.82
203045_at	ninjurin 1	NINJ1	8.75	8.67	8.73
216884_at	protein tyrosine p	PTPN12	3.79	3.46	3.50
209973_at	nuclear factor of k	NFKBIL1	7.68	7.50	7.60
210007_s_at	glycerol-3-phosph	GPD2	3.84	3.95	3.91
210415_s_at	outer dense fiber	ODF2	7.66	7.53	7.57
219680_at	NLR family memb	NLRX1	9.55	9.33	9.27
204960_at	protein tyrosine p	PTPRCAP	5.74	5.65	5.60
215374_at	poly(A) polymeras	PAPOLA	6.02	5.92	5.52
216370_s_at	transketolase-like	TKTL1	5.31	5.15	5.13
200736_s_at	glutathione perox	GPX1	12.42	12.28	12.34
214692_s_at	jerky homolog (m	JRK	7.72	7.90	7.86
219406_at	chromosome 1 op	C1orf50	7.97	7.94	8.10
201757_at	NADH dehydrogen	NDUFS5 /// RPL10	13.17	13.12	13.11
218768_at	nucleoporin 107kl	NUP107	10.90	10.94	10.98
220668_s_at	DNA (cytosine-5-)	DNMT3B	5.64	6.28	5.85
201047_x_at	RAB6A, member R	RAB6A	10.77	10.88	11.00

205987_at	CD1c molecule	CD1C	6.69	6.79	6.16
216495_x_at	---	---	6.09	6.28	6.16
210295_at	melanoma antigen	MAGEA10	5.08	5.10	4.75
220879_at	---	---	4.58	4.89	4.46
218804_at	anoctamin 1, calci	ANO1	3.46	3.51	3.58
217595_at	G1 to S phase tran	GSPT1	3.81	3.70	3.81
207691_x_at	ectonucleoside tri	ENTPD1	3.80	3.59	3.87
210932_s_at	ring finger protein	RNF6	3.99	3.52	3.69
209105_at	nuclear receptor c	NCOA1	6.08	5.97	6.11
220215_at	zinc finger protein	ZNF669	6.62	6.47	6.34
55583_at	dedicator of cytok	DOCK6	6.40	6.47	6.35
206952_at	glucose-6-phosph	G6PC	3.03	3.34	2.98
215466_at	---	---	4.71	4.77	4.50
205979_at	secretoglobin, fan	SCGB2A1	3.47	3.39	3.25
218657_at	Rap guanine nucle	RAPGEFL1	6.25	6.29	6.06
206321_at	regulatory factor X	RFX1	6.35	6.39	5.88
212377_s_at	Notch homolog 2	NOTCH2	10.59	10.53	10.63
215423_at	---	---	5.56	5.95	5.78
213680_at	keratin 6B	KRT6B	4.46	4.70	4.66
219413_at	acyl-CoA binding c	ACBD4	7.28	7.16	6.91
209705_at	metal response el	MTF2	6.94	7.26	6.92
205151_s_at	TLR4 interactor w	TRIL	4.68	4.67	4.89
221328_at	claudin 17	CLDN17	6.81	6.85	6.80
213967_at	RALY RNA binding	RALYL	3.94	4.17	3.86
213987_s_at	cyclin-dependent	CDK13	7.09	6.91	6.57
217517_x_at	SFRS protein kinas	SRPK2	5.81	6.05	5.90
221307_at	Kv channel interac	KCNIP1	4.90	4.85	4.69
201165_s_at	pumilio homolog 1	PUM1	10.75	10.68	10.69
211327_x_at	hemochromatosis	HFE	6.00	6.09	5.79
204487_s_at	potassium voltage	KCNQ1	6.89	7.00	6.80
219167_at	RAS-like, family 12	RASL12	4.87	4.87	4.88
207728_at	activating transcri	ATF7IP	4.11	3.85	3.99
207028_at	v-myc myelocytom	MYCNOS	7.04	7.27	6.74
204423_at	muskelin 1, intrac	MKLN1	7.90	7.89	7.80
204365_s_at	receptor accessor	REEP1	4.63	4.47	4.41
217357_at	---	---	6.61	6.31	6.41
205535_s_at	protocadherin 7	PCDH7	5.01	5.20	4.87
201567_s_at	golgin A4	GOLGA4	10.05	10.40	10.33
212251_at	metadherin	MTDH	11.73	11.86	11.91
204251_s_at	centrosomal prote	CEP164	7.29	7.26	6.98
217222_at	Immunoglobulin h	IGHG1	5.35	5.13	4.95
206229_x_at	paired box 2	PAX2	4.90	4.79	4.79
215707_s_at	prion protein	PRNP	8.59	8.47	8.60
205498_at	growth hormone r	GHR	3.74	3.76	3.60
206230_at	LIM homeobox 1	LHX1	5.68	5.47	5.60
216443_at	---	---	3.05	3.39	3.36
216203_at	serine palmitoyltr	SPTLC2	3.69	3.81	3.58

221385_s_at	free fatty acid rec	FFAR3	6.20	5.85	5.57
220582_at	---	---	4.68	4.64	4.96
203136_at	Rab acceptor 1 (pı	RABAC1	10.63	10.72	10.55
207208_at	RNA binding moti	RBMXL2	4.41	4.15	3.92
210174_at	nuclear receptor s	NR5A2	3.70	3.68	3.60
219964_at	suppression of tu	ST7L	5.35	5.58	5.52
214842_s_at	---	---	3.52	3.40	3.40
204086_at	preferentially exp	PRAME	5.34	5.25	5.12
204181_s_at	zinc finger and BT	ZBTB43	8.52	8.63	8.36
210597_x_at	proline-rich prote	PRB1	8.22	8.16	7.65
205599_at	TNF receptor-assc	TRAF1	6.09	6.27	6.05
218925_s_at	chromosome 11 c	C11orf1	3.38	3.48	3.35
215543_s_at	like-glycosyltransf	LARGE	5.82	5.38	5.59
220638_s_at	Cas-Br-M (murine	CBLC	5.70	5.82	5.75
211351_at	chromosome 6 op	C6orf54	4.00	3.90	3.51
216560_x_at	Immunoglobulin l	IGL@	5.97	5.83	5.78
209574_s_at	chromosome 18 c	C18orf1	4.43	4.50	4.38
211257_x_at	zinc finger protein	ZNF638	9.43	9.61	9.68
214907_at	carcinoembryonic	CEACAM21	4.42	4.69	4.38
216863_s_at	MORC family CW-	MORC2	7.17	7.25	7.24
207207_at	RNA binding moti	RBMXL2	4.03	4.03	3.86
218011_at	ubiquitin-like 5	UBL5	11.47	11.45	11.51
214742_at	5-azacytidine indu	AZI1	7.36	7.22	7.33
212061_at	U2-associated SR1	SR140	9.50	9.55	9.66
210304_at	phosphodiesteras	PDE6B	5.38	5.50	5.09
208025_s_at	high mobility grou	HMGA2	6.08	6.00	5.92
221890_at	zinc finger protein	ZNF335	7.37	7.57	7.43
202078_at	COP9 constitutive	COPS3	9.91	9.99	10.15
203168_at	activating transcri	ATF6B	8.27	8.21	8.27
215974_at	similar to major h	LOC100294102	5.37	5.02	5.00
214822_at	family with seque	FAM5B	4.19	3.95	4.04
204486_at	---	---	5.51	5.15	5.42
208634_s_at	microtubule-actin	MACF1	10.13	10.16	10.28
212984_at	activating transcri	ATF2	9.58	9.64	9.76
202090_s_at	ubiquinol-cytochr	UQCR11	12.27	12.31	12.36
218968_s_at	zinc finger protein	ZFP64	9.04	9.18	9.19
214193_s_at	chromosome 1 op	C1orf107	9.27	9.16	9.55
206241_at	karyopherin alpha	KPNA5	4.53	4.75	4.59
214095_at	serine hydroxyme	SHMT2	9.31	9.08	9.22
64440_at	interleukin 17 rec	IL17RC	7.46	7.43	7.30
217387_at	calpain 6	CAPN6	3.69	3.53	3.18
201987_at	mediator complex	MED13	10.80	10.82	10.90
211510_s_at	corticotropin relea	CRHR2	3.76	3.52	3.51
221759_at	glucose 6 phospha	G6PC3	7.69	7.80	7.59
217274_x_at	myosin, light chain	MYL4	6.25	6.14	6.33
217161_x_at	aggrecan	ACAN	6.82	6.43	6.40
204360_s_at	N-acetylglucosam	NAGLU	7.74	7.60	7.60

216986_s_at	interferon regulat	IRF4	5.91	6.09	5.87
222305_at	hexokinase 2	HK2	8.44	8.20	8.36
214215_s_at	La ribonucleoprot	LARP4B	3.99	4.04	3.85
215648_at	---	---	4.23	4.30	4.29
207568_at	cholinergic recept	CHRNA6	6.07	6.19	5.77
210046_s_at	isocitrate dehydrc	IDH2	8.55	8.41	8.37
218637_at	Impact homolog (	IMPACT	7.88	7.98	8.13
208511_at	pituitary tumor-tr	PTTG3P	9.43	9.58	9.57
210930_s_at	v-erb-b2 erythrob	ERBB2	5.30	5.31	5.37
218504_at	fumarylacetoacet	FAHD2A	9.81	9.73	9.82
214854_at	---	---	4.74	5.06	5.17
211390_at	NEDD4 binding pr	N4BP2L1	3.63	3.37	3.51
206622_at	thyrotropin-releas	TRH	5.90	5.78	5.90
222278_at	---	---	4.94	5.15	4.95
204551_s_at	alpha-2-HS-glycop	AHSG	6.73	6.42	6.41
206251_s_at	arginine vasopres	AVPR1A	3.61	3.45	3.46
218894_s_at	mago-nashi homo	MAGOHB	7.35	7.61	7.58
221888_at	coiled-coil and C2	CC2D1A	7.20	6.97	6.91
203831_at	R3H domain conta	R3HDM2	8.75	8.63	8.82
211657_at	carcinoembryonic	CEACAM6	5.18	4.97	5.09
205059_s_at	iduronidase, alpha	IDUA	6.91	6.83	6.73
222134_at	D-aspartate oxida	DDO	6.27	6.23	6.36
215203_at	golgin A4	GOLGA4	7.09	7.26	7.35
205461_at	RAB35, member F	RAB35	5.70	5.94	5.80
211702_s_at	ubiquitin specific		USP32	7.53	7.43	7.55
208224_at	homeobox B1	HOXB1	6.45	6.43	6.23
216383_at	ribosomal protein	RPL18A /// RPL18A	9.29	9.06	9.26
211856_x_at	CD28 molecule	CD28	4.67	4.83	4.58
220901_at	G protein-couplec	GPR157	5.32	4.88	5.33
208348_s_at	Cas-Br-M (murine	CBLB	4.08	3.69	3.71
215124_at	zinc finger protein	ZNF550	3.56	3.98	3.72
204302_s_at	KIAA0427	KIAA0427	7.82	7.58	7.32
218851_s_at	WD repeat domai	WDR33	6.02	5.75	6.20
211218_at	---	---	3.85	3.72	3.72
220392_at	early B-cell factor	EBF2	4.57	4.66	4.78
206222_at	tumor necrosis fa	TNFRSF10C	5.75	5.52	5.27
217341_at	hypothetical prote	LOC100289042	7.29	6.96	7.21
202735_at	emopamil binding	EBP	10.08	10.00	10.10
208580_x_at	histone cluster 1,	HIST1H4J /// HIST1	7.35	7.31	7.42
204437_s_at	folate receptor 1 (	FOLR1	4.62	4.49	4.74
205952_at	potassium channe	KCNK3	4.25	4.00	3.98
209348_s_at	v-maf musculoapo	MAF	3.79	3.59	3.41
200759_x_at	nuclear factor (ery	NFE2L1	10.57	10.21	10.57
212584_at	aquarius homolog	AQR	9.15	9.24	9.22
213450_s_at	inducible T-cell co	ICOSLG	7.73	7.66	7.21
206833_s_at	acylphosphatase 2	ACYP2	8.11	8.21	8.13
212071_s_at	spectrin, beta, no	SPTBN1	12.25	12.14	12.22

202522_at	phosphatidylinosi	PITPNB	10.94	10.97	11.04
206647_at	hemoglobin, zeta	HBZ	5.12	4.88	4.78
216999_at	erythropoietin rec	EPOR	3.40	3.18	2.95
212884_x_at	apolipoprotein E	APOE	7.71	7.41	7.46
216930_at	HNF1 homeobox /	HNF1A	4.77	4.74	4.88
212550_at	signal transducer .	STAT5B	6.87	6.73	6.52
220226_at	transient receptor	TRPM8	5.66	5.67	5.56
206252_s_at	arginine vasopres:	AVPR1A	4.54	4.24	4.39
201576_s_at	galactosidase, bet	GLB1	9.54	9.46	9.44
205246_at	peroxisomal bioge	PEX13	7.65	7.84	7.69
204207_s_at	RNA guanylyltrans	RNGTT	7.11	7.59	7.38
220706_at	ADAM metallopep	ADAMTS7	4.68	4.09	4.39
207977_s_at	dermatopontin	DPT	6.18	6.05	5.50
213368_x_at	protein tyrosine p	PPFIA3	7.30	7.22	7.30
207701_at	chromosome 22 c	C22orf24	6.40	6.23	5.97
208203_x_at	killer cell immuno	KIR2DS5	5.16	5.29	5.10
212163_at	kinase D-interacti	KIDINS220	9.71	9.67	9.81
213496_at	lipid phosphate pl	LPPR4	3.38	3.15	3.27
221607_x_at	actin, gamma 1	ACTG1	14.23	14.30	14.19
205047_s_at	asparagine synthe	ASNS	12.72	12.24	12.72
213197_at	astrotactin 1	ASTN1	3.81	3.68	3.46
210471_s_at	potassium voltage	KCNAB1	5.73	5.56	5.42
207848_at	arginine vasopres:	AVP	5.73	5.67	5.90
205494_at	zinc finger protein	ZNF821	6.50	6.28	6.00
211507_s_at	myotubularin rela	MTMR3	4.86	4.89	4.27
210726_at	cytochrome P450,	CYP3A4	6.72	6.76	6.83
212156_at	vacuolar protein s	VPS39	8.24	8.30	7.99
221822_at	coiled-coil domain	CCDC101	7.41	7.19	7.38
210818_s_at	BTB and CNC hom	BACH1	4.67	4.65	4.41
203018_s_at	synovial sarcoma,	SSX2IP	8.53	8.69	8.75
201419_at	BRCA1 associated	BAP1	8.46	8.41	8.50
205365_at	homeobox B6	HOXB6	4.25	4.10	4.01
208388_at	nuclear receptor s	NR2E3	4.34	4.27	4.02
221865_at	chromosome 9 op	C9orf91	7.91	7.64	8.06
202819_s_at	transcription elon	TCEB3	8.33	8.44	8.44
213127_s_at	mediator complex	MED8	8.84	8.81	8.85
213013_at	mitogen-activated	LOC644172 /// MA	7.93	7.48	7.47
213341_at	fem-1 homolog c (	FEM1C	7.78	7.95	7.93
212611_at	deltex homolog 4	DTX4	4.05	4.38	4.17
205983_at	dipeptidase 1 (rer	DPEP1	6.98	6.74	6.54
218116_at	chromosome 9 op	C9orf78	8.65	8.82	9.16
204973_at	gap junction prote	GJB1	7.82	7.50	7.55
216902_s_at	RRN3 RNA polyme	RRN3 /// RRN3P1 ,	4.74	5.06	4.66
213410_at	chromosome 10 c	C10orf137	8.70	8.87	8.94
218347_at	tRNA-yW synthesi	TYW1	9.06	9.01	9.06
222318_at	zinc finger protein	ZNF324B /// ZNF5	6.41	6.19	6.18
213220_at	non-protein codin	NCRNA00081	9.98	9.96	9.98

210292_s_at	protocadherin 11	PCDH11X /// PCDH	3.04	3.30	3.16
207173_x_at	cadherin 11, type	CDH11	3.39	3.17	3.38
208376_at	chemokine (C-C m	CCR4	5.97	6.13	6.05
218345_at	transmembrane p	TMEM176A	5.50	5.76	5.72
200993_at	importin 7	IPO7	11.67	11.71	11.72
207578_s_at	5-hydroxytryptam	HTR4	7.90	8.00	7.86
213710_s_at	Calmodulin 1 (pho	CALM1	6.70	6.80	6.88
210164_at	granzyme B (gran	GZMB	4.43	4.63	4.53
201581_at	thioredoxin-relate	TMX4	10.91	10.99	11.03
214261_s_at	alcohol dehydroge	ADH6	5.22	5.50	5.22
202229_s_at	calcium homeosta	CHERP	6.79	6.59	6.50
220524_at	erythrocyte meml	EPB41L4B	7.11	7.03	6.78
220274_at	IQ motif containir	IQCA1	4.89	4.98	4.80
206722_s_at	lysophosphatidic a	LPAR2	6.40	6.57	6.41
221116_at	---	---	5.91	5.72	5.87
211932_at	heterogeneous nu	HNRNPA3	11.12	11.16	11.38
36711_at	v-maf musculoapo	MAFF	10.79	10.50	10.85
212287_at	suppressor of zest	SUZ12	11.14	11.32	11.41
203958_s_at	zinc finger and BT	ZBTB40	7.21	7.17	6.82
210588_x_at	heterogeneous nu	HNRNPH3	10.92	11.08	11.06
208040_s_at	myosin binding pr	MYBPC3	7.65	7.51	7.19
AFFX-BioDn-3_a	---	---	12.87	12.91	12.93
208899_x_at	ATPase, H+ transp	ATP6V1D	10.06	10.20	10.10
206161_s_at	synaptotagmin V	SYT5	6.57	6.63	6.44
204766_s_at	nudix (nucleoside	NUDT1	9.63	9.57	9.69
206777_s_at	crystallin, beta B2	CRYBB2 /// CRYBB	7.25	7.05	7.23
213275_x_at	cathepsin B	CTSB	9.13	9.09	9.25
209110_s_at	ral guanine nuclec	RGL2	8.43	8.51	8.44
214397_at	methyl-CpG bindi	MBD2	3.40	3.40	3.48
214023_x_at	tubulin, beta 2B	TUBB2B	5.40	5.22	5.12
35156_at	R3H domain and c	R3HCC1	9.54	9.49	9.42
221118_at	polycystic kidney	PKD2L2	4.02	3.69	3.87
219761_at	C-type lectin dom	CLEC1A	3.84	4.16	3.93
212970_at	amyloid beta (A4)	APBB2	8.76	8.68	8.84
220712_at	chromosome 8 op	C8orf60	6.35	6.59	6.35
208453_s_at	X-prolyl aminopep	XPNPEP1	8.48	8.54	8.39
217735_s_at	eukaryotic transla	EIF2AK1	7.51	7.60	7.31
211398_at	fibroblast growth	FGFR2	5.66	5.51	5.57
207301_at	ephrin-A5	EFNA5	4.84	4.14	4.35
206516_at	anti-Mullerian hor	AMH	5.96	6.23	5.84
206367_at	renin	REN	4.59	4.66	4.64
200750_s_at	RAN, member RAS	RAN	13.37	13.44	13.46
202662_s_at	inositol 1,4,5-tripł	ITPR2	6.05	6.06	6.45
206419_at	RAR-related orpha	RORC	6.82	6.78	6.66
215534_at	---	---	4.33	4.37	3.97
215332_s_at	CD8b molecule	CD8B	3.57	3.71	3.59
208002_s_at	acyl-CoA thioester	ACOT7	10.72	10.65	10.80

216752_at	Phosphoinositide-	PIK3R4	3.34	3.34	3.37
213239_at	progesterone imn	PIBF1	8.68	8.78	8.92
209334_s_at	proteasome (pros	PSMD9	6.97	7.25	7.22
204333_s_at	aspartylglucosami	AGA	6.41	6.34	6.65
207381_at	arachidonate 12-l	ALOX12B	4.02	3.72	3.75
220210_at	cholinergic recept	CHRNA10	5.94	5.64	5.53
214680_at	neurotrophic tyro	NTRK2	4.80	4.63	4.80
201876_at	paraoxonase 2	PON2	11.61	11.74	11.81
220868_s_at	solute carrier fam	SLC7A10	5.65	5.54	5.64
205797_s_at	t-complex 11 (mo	TCP11L1	7.45	7.66	7.18
213418_at	heat shock 70kDa	HSPA6	5.92	5.73	5.80
205107_s_at	ephrin-A4	EFNA4	6.96	7.34	7.01
221391_at	taste receptor, tyı	TAS2R14	4.88	4.94	4.45
207978_s_at	nuclear receptor s	NR4A3	4.44	4.33	4.55
208377_s_at	calcium channel, v	CACNA1F	6.19	6.17	5.86
206872_at	solute carrier fam	SLC17A1	3.73	3.66	3.89
219129_s_at	SAP30-like	SAP30L	7.02	7.01	7.11
216467_s_at	---	---	3.87	4.09	3.91
206061_s_at	dicer 1, ribonucle	DICER1	7.20	7.45	7.67
222264_at	heterogeneous nu	HNRNPUL2	8.03	7.91	7.96
44669_at	succinate dehydro	SDHAF1	7.93	8.02	8.03
219800_s_at	threonine synthas	THNSL1	4.80	5.11	4.97
215058_at	DENN/MADD don	DENND5B	4.62	4.87	4.67
214324_at	glycoprotein 2 (zy	GP2	5.67	5.70	5.43
208623_s_at	ezrin	EZR	10.72	10.76	10.86
212441_at	KIAA0232	KIAA0232	8.39	8.57	8.51
210322_x_at	ubiquitously trans	UTY	5.93	5.76	5.82
200621_at	cysteine and glyci	CSRP1	11.07	11.02	11.03
219717_at	DDB1 and CUL4 as	DCAF16	7.99	8.06	8.08
201795_at	lamin B receptor	LBR	11.60	11.73	11.64
205331_s_at	receptor accessor	REEP2	7.47	7.34	7.14
208543_at	olfactory receptor	OR10H2	3.60	3.85	3.78
220139_at	DNA (cytosine-5-)	DNMT3L	6.15	6.15	5.93
207094_at	chemokine (C-X-C	CXCR1	6.86	6.87	6.65
221523_s_at	Ras-related GTP b	RRAGD	3.86	3.81	3.60
211820_x_at	glycophorin A (MI	GYPA /// GYPB	3.85	3.69	3.55
205962_at	p21 protein (Cdc4	PAK2	4.45	3.95	4.18
220774_at	dymeclin	DYM	5.14	5.29	5.22
207979_s_at	CD8b molecule	CD8B	6.37	6.16	6.55
220888_s_at	Cas scaffolding pr	CASS4	3.72	3.68	3.65
221306_at	G protein-couplec	GPR27	7.01	7.22	6.28
213257_at	sterile alpha and T	SARM1	6.34	6.27	6.29
207928_s_at	glycine receptor, a	GLRA3	3.34	3.27	3.19
219794_at	vacuolar protein s	VPS53	5.70	5.66	5.43
216312_at	ATPase, Ca++ tran	ATP2B3	4.98	4.70	5.01
213573_at	---	---	9.89	9.80	10.02
217671_at	---	---	4.51	4.60	4.75

206286_s_at	teratocarcinoma-(	TDGF1 /// TDGF3	6.07	6.09	5.78
211128_at	ectodysplasin A	EDA	4.03	3.62	3.78
214572_s_at	insulin-like 3 (Leyc	INSL3 /// JAK3	4.52	4.24	4.14
218643_s_at	cysteine-rich PDZ-	CRIPT	8.08	8.23	8.25
207893_at	sex determining r	SRY	3.98	4.07	3.95
222026_at	RNA binding moti	RBM3	8.31	8.43	8.44
205909_at	polymerase (DNA	POLE2	8.22	8.45	8.49
210669_at	transcription facto	TFAP2A	4.34	4.14	3.94
215903_s_at	microtubule assoc	MAST2	8.64	8.47	8.60
217279_x_at	matrix metallopep	MMP14	6.40	6.49	6.20
207763_at	S100 calcium bind	S100A5	6.15	6.24	6.32
214566_at	submaxillary glanc	SMR3A	3.31	3.46	3.71
208896_at	DEAD (Asp-Glu-Al	DDX18	10.19	10.40	10.51
205273_s_at	pitrilysin metallop	PITRM1	9.76	9.78	9.80
217553_at	STEAP family prot	MGC87042	4.47	3.90	3.99
210644_s_at	leukocyte-associa	LAIR1	5.02	5.39	5.20
206362_x_at	mitogen-activatec	MAP3K10	7.49	7.65	7.32
207694_at	POU class 3 home	POU3F4	4.00	4.17	4.08
204521_at	chromosome 12 c	C12orf24	10.81	10.82	10.95
216247_at	---	---	4.76	4.73	4.49
219857_at	chromosome 10 c	C10orf81	3.40	3.51	3.37
215400_x_at	Adenylate cyclase	ADCY9	5.87	5.83	5.86
209670_at	T cell receptor alp	TRAC	4.69	4.58	4.43
222107_x_at	leucine zipper, pu	LZTS1	7.80	7.73	7.65
207385_at	transcription facto	TFDP3	4.89	4.59	4.58
221147_x_at	WW domain cont	WWOX	5.64	5.81	5.59
210344_at	oxysterol binding	OSBPL7	5.11	5.03	4.81
201925_s_at	CD55 molecule, d	CD55	10.32	10.09	10.42
216862_s_at	mature T-cell prol	MTCP1NB	9.63	9.57	9.62
208395_s_at	URB1 ribosome bi	URB1	7.28	7.10	6.82
210019_at	calmodulin-like 3	CALML3	4.64	5.00	4.76
215105_at	hypothetical CG03	CG030	5.26	5.51	5.05
200892_s_at	transformer 2 bet	TRA2B	11.10	11.13	11.22
214207_s_at	Caspase recruitme	CARD10	4.12	4.05	4.09
219704_at	Y box binding prot	YBX2	7.99	8.04	7.42
212490_at	DnaJ (Hsp40) hom	DNAJC8	7.77	7.85	7.79
210666_at	iduronate 2-sulfat	IDS	7.78	7.72	7.33
214558_at	G protein-couplec	GPR12	4.42	4.18	4.28
52940_at	similar to single Ig	LOC100294402 ///	7.17	7.24	7.31
209049_s_at	zinc finger, MYND	ZMYND8	11.12	11.07	11.13
202037_s_at	secreted frizzled-r	SFRP1	4.47	4.60	4.36
221528_s_at	engulfment and c	ELMO2	7.18	7.22	7.31
213235_at	chromosome 16 c	C16orf88	8.94	8.77	8.82
214318_s_at	furry homolog (Dr	FRY	6.62	6.83	6.85
221026_s_at	scratch homolog 1	SCRT1	4.27	4.60	4.18
219721_at	---	---	6.18	6.24	6.11
208387_s_at	matrix metallopep	MMP24	5.65	5.33	5.10

207227_x_at	ret finger protein-	RFPL2	4.62	4.74	4.32
208850_s_at	Thy-1 cell surface	THY1	6.61	6.92	6.58
203525_s_at	adenomatous pol	APC	7.83	8.10	8.06
216172_at	---	---	5.97	5.81	5.45
215087_at	chromosome 15 c	C15orf39	4.89	4.72	4.95
214933_at	calcium channel, v	CACNA1A	4.38	4.23	4.30
211674_x_at	cancer/testis anti	CTAG1A /// CTAG1	5.16	4.89	4.98
210790_s_at	SAR1 homolog A (	SAR1A	7.06	7.21	7.02
40255_at	DEAD (Asp-Glu-Al	DDX28	8.19	8.28	8.20
217565_at	glutamate recepto	GRIA3	3.43	3.55	3.38
215228_at	nescient helix loo	NHLH2	3.37	3.46	3.24
210746_s_at	erythrocyte meml	EPB42	4.28	4.42	4.01
220530_at	non-protein codin	NCRNA00120	3.61	3.59	3.71
218895_at	G patch domain c	GPATCH3	7.31	7.55	7.31
214565_s_at	submaxillary glan	SMR3A /// SMR3B	4.42	4.41	4.28
221655_x_at	EPS8-like 1	EPS8L1	7.73	7.59	7.38
214371_at	testis-specific seri	TSSK2	4.52	4.16	3.93
211923_s_at	zinc finger protei	ZNF471	3.64	3.69	3.72
217204_at	Mitochondrial tra	MTRF1L	3.47	3.52	3.48
219317_at	polymerase (DNA	POLI	8.50	8.69	8.89
203750_s_at	retinoic acid rece	RARA	6.92	6.87	6.54
209276_s_at	glutaredoxin (thio	GLRX	8.68	8.78	8.76
210330_at	sarcoglycan, delta	SGCD	3.81	3.65	3.48
216869_at	phosphodiesteras	PDE1C	5.87	5.54	5.68
209552_at	paired box 8	PAX8	6.03	6.15	6.03
210405_x_at	tumor necrosis fa	TNFRSF10B	7.52	7.33	7.59
219088_s_at	zinc finger protei	ZNF576	7.87	7.86	7.85
208034_s_at	protein Z, vitamin	PROZ	6.43	6.81	6.24
212036_s_at	pinin, desmosome	PNN	10.24	10.44	10.29
206380_s_at	complement facto	CFP	4.03	3.89	4.01
216527_at	---	---	4.48	4.65	4.33
203560_at	gamma-glutamyl l	GGH	10.69	10.78	10.86
204383_at	DiGeorge syndron	DGCR14	6.84	6.85	6.78
211176_s_at	paired box 4	PAX4	4.98	4.96	4.89
210378_s_at	Sjogren syndrome	SSNA1	9.30	9.09	9.27
210641_at	calpain 9	CAPN9	3.76	3.79	3.66
206030_at	aspartoacylase (C	ASPA	4.36	4.30	4.41
221389_at	phospholipase A2	PLA2G2E	5.57	5.71	5.42
215864_at	---	---	3.21	3.74	3.61
203361_s_at	c-myc binding pro	MYCBP	7.70	8.05	7.88
204435_at	nucleoporin like 1	NUPL1	9.51	9.60	9.75
219777_at	GTPase, IMAP fan	GIMAP6	3.03	3.29	3.12
200050_at	zinc finger protei	ZNF146	10.98	10.99	11.14
200975_at	palmitoyl-protein	PPT1	12.02	12.07	12.12
219607_s_at	membrane-spann	MS4A4A	3.29	2.95	3.18
218963_s_at	keratin 23 (histon	KRT23	5.31	5.15	5.08
215562_at	tetratricopeptide	TTC39A	5.67	5.57	5.41

214871_x_at	Hypothetical prot	LOC100287590	6.78	6.51	6.55
220748_s_at	zinc finger protein	ZNF580	8.29	8.19	8.15
212876_at	UDP-Gal:betaGlcN	B4GALT4	8.06	7.97	8.07
205615_at	carboxypeptidase	CPA1	4.04	4.31	4.36
207784_at	arylsulfatase D	ARSD	7.12	7.18	6.90
206814_at	nerve growth fact	NGF	7.30	6.89	7.20
222076_at	Heparin-binding E	HBEGF	5.38	5.65	5.52
214929_s_at	KIAA1109	KIAA1109	5.21	5.40	5.11
218698_at	APAF1 interacting	APIP	10.42	10.36	10.49
202585_s_at	nuclear transcript	NFX1	6.67	6.24	6.44
213282_at	Apolipoprotein O-	APOOL	8.52	8.61	8.65
209475_at	ubiquitin specific	USP15	8.80	8.88	8.87
210054_at	HAUS augmin-like	HAUS3	8.61	8.60	8.71
206534_at	glutamate recepto	GRIN2A	4.49	4.87	4.47
214942_at	RNA binding moti	RBM34	4.19	3.76	4.13
215986_at	---	---	3.85	3.65	3.87
218008_at	chromosome 7 op	C7orf42	10.36	10.35	10.40
218129_s_at	nuclear transcript	NFYB	8.26	8.24	8.46
208977_x_at	tubulin, beta 2C	TUBB2C	11.73	11.91	11.77
203905_at	poly(A)-specific ri	PARN	10.15	10.21	10.21
221580_s_at	TATA box binding	TAF1D	11.61	11.67	11.79
211593_s_at	microtubule asso	MAST2	8.36	8.28	8.20
208888_s_at	nuclear receptor c	NCOR2	6.13	6.22	6.37
204265_s_at	G-protein signalin	GPSM3	8.07	7.97	7.60
213708_s_at	MAX-like protein X	MLX	9.66	9.74	9.68
222078_at	pyruvate kinase, li	PKLR	6.12	6.31	6.36
208719_s_at	DEAD (Asp-Glu-Ala	DDX17	4.78	4.61	4.24
204660_at	growth factor, aug	GFER	6.41	6.30	6.59
203766_s_at	leiomodin 1 (smo	LMOD1	5.59	5.53	5.12
215451_s_at	AF4/FMR2 family,	AFF1	4.99	4.93	4.78
77508_r_at	rabaptin, RAB GTF	RABEP2	8.06	7.82	7.90
209539_at	Rac/Cdc42 guanin	ARHGEF6	7.01	6.98	6.95
210169_at	SEC14-like 5 (S. ce	SEC14L5	5.89	5.85	5.95
220709_at	zinc finger protein	ZNF556	5.68	5.44	5.32
221115_s_at	lens epithelial pro	LENEP	4.51	4.57	4.30
208421_at	---	---	3.86	3.64	3.71
208013_s_at	acrosomal vesicle	ACRV1	4.56	4.91	4.28
221001_at	---	---	4.98	5.08	4.33
207057_at	solute carrier fam	SLC16A7	8.53	8.53	8.63
215405_at	---	---	3.41	3.41	3.39
206480_at	leukotriene C4 syr	LTC4S	6.40	6.55	6.49
220508_at	chaperonin contai	CCT8L2	6.41	6.43	6.06
215705_at	protein phosphata	PPP5C	6.63	7.00	6.90
205664_at	KIN, antigenic det	KIN	8.73	8.91	8.93
221468_at	chemokine (C mot	XCR1	4.66	4.51	4.73
205949_at	carbonic anhydras	CA1	3.83	3.94	3.54
214762_at	ATPase, H+ transp	ATP6V1G2	4.55	4.73	4.66

209953_s_at	cell division cycle	CDC37	9.52	9.54	9.55
217306_at	---	---	3.60	4.07	3.26
221438_s_at	testis expressed 1	TEX12	3.57	3.63	3.51
214832_at	hepatocyte nuclea	HNF4A	6.57	6.33	6.35
214663_at	dual serine/threor	DSTYK	7.89	8.01	7.93
218164_at	spermatogenesis a	SPATA20	7.82	7.81	7.64
203913_s_at	hydroxyprostaglar	HPGD	3.45	3.69	3.46
45714_at	host cell factor C1	HCFC1R1	9.25	9.13	9.16
202232_s_at	eukaryotic transla	EIF3M	11.46	11.56	11.85
217858_s_at	armadillo repeat c	ARMCX3	9.40	9.48	9.54
204137_at	G protein-couplec	GPR137B	6.30	6.36	6.21
211969_at	heat shock protei	HSP90AA1	13.57	13.74	13.66
208903_at	ribosomal protein	RPS28	8.40	8.52	8.33
211882_x_at	fucosyltransferase	FUT6	4.43	4.55	4.70
216957_at	ubiquitin specific		USP22	5.57	5.67	5.45
206518_s_at	regulator of G-prc	RGS9	4.87	5.03	5.01
205691_at	synaptogyrin 3	SYNGR3	6.52	6.78	6.50
207497_s_at	membrane-spann	MS4A2	4.48	4.16	4.38
215210_s_at	dihydrolipoamide	DLST	9.14	8.97	9.07
206291_at	neurotensin	NTS	4.17	4.15	3.96
216171_at	---	---	3.35	3.45	3.28
203690_at	tubulin, gamma cc	TUBGCP3	9.65	9.69	9.83
213655_at	Tyrosine 3-monoc	YWHAE	11.95	11.96	11.95
220003_at	leucine rich repea	LRRC36	7.60	7.56	7.38
217764_s_at	RAB31, member F	RAB31	10.84	10.90	10.97
210878_s_at	lysine (K)-specific	KDM3B	9.24	9.35	9.36
215422_at	---	---	3.56	3.28	3.25
221836_s_at	trafficking protein	TRAPPC9	6.58	6.53	6.38
211572_s_at	solute carrier fam	SLC23A2	4.52	4.58	4.32
210973_s_at	fibroblast growth	FGFR1	6.89	6.96	6.57
208522_s_at	patched homolog	PTCH1	5.90	5.38	5.18
209041_s_at	ubiquitin-conjugat	UBE2G2	8.45	8.50	8.47
207994_s_at	opioid receptor, n	OPRM1	3.92	3.81	3.84
216817_s_at	olfactory receptor	OR2H1	5.06	4.99	4.90
202031_s_at	WD repeat domai	WIPI2	9.77	9.77	9.68
210969_at	protein kinase N2	PKN2	3.26	3.59	3.45
211133_x_at	leukocyte immunc	LILRB3	7.47	7.46	7.09
216736_at	transmembrane 6	TM6SF2	5.22	5.39	5.34
204002_s_at	islet cell autoantig	ICA1	5.38	5.52	5.22
213460_x_at	NOP2/Sun domair	NSUN5P2	9.38	9.44	9.36
215606_s_at	ELKS/RAB6-intera	ERC1	7.56	7.51	7.56
213670_x_at	NOP2/Sun domair	NSUN5P1	9.14	9.21	9.19
206631_at	prostaglandin E re	PTGER2	3.36	3.34	3.50
212057_at	KIAA0182	KIAA0182	9.66	9.49	9.74
203954_x_at	claudin 3	CLDN3	6.21	6.01	6.01
201584_s_at	DEAD (Asp-Glu-Ala	DDX39	10.81	10.91	10.87
206759_at	Fc fragment of IgE	FCER2	4.66	4.81	5.03

215959_at	PTPRF interacting	PPFIBP2	4.87	4.90	4.50
220891_at	chromosome 4 op	C4orf23	4.01	4.08	3.93
201558_at	RAE1 RNA export	RAE1	10.93	10.94	10.93
214659_x_at	YLP motif containi	YLPM1	8.61	8.80	8.58
215430_at	glycerol kinase 2	GK2	4.21	4.14	4.25
207257_at	erythropoietin	EPO	5.97	5.91	5.77
208269_s_at	ADAM metallopep	ADAM28	6.13	6.04	6.12
204064_at	THO complex 1	THOC1	9.46	9.51	9.52
208451_s_at	complement com	C4A /// C4B /// LO	7.24	7.20	6.99
209179_s_at	membrane bound	MBOAT7	8.37	8.51	8.58
211811_s_at	protocadherin alp	PCDHA6	4.61	4.78	4.41
208033_s_at	zinc finger homeo	ZFHX3	5.81	5.60	5.46
213566_at	ribonuclease, RNa	RNASE6	3.78	3.64	3.85
219850_s_at	ets homologous fa	EHF	3.40	3.49	3.38
208565_at	melanocortin 5 re	MC5R	3.74	4.29	4.15
215439_x_at	---	---	6.64	6.59	6.54
220611_at	disabled homolog	DAB1	4.80	4.73	4.88
207425_s_at	septin 9	9-Sep	7.08	7.12	7.15
202824_s_at	transcription elon	TCEB1	12.14	12.19	12.29
214704_at	transcription facto	TCF25	5.04	4.87	4.44
221424_s_at	olfactory receptor	OR51E2	5.49	5.19	5.40
204700_x_at	chromosome 1 op	C1orf107	10.06	9.90	10.19
202796_at	synaptopodin	SYNPO	8.43	8.33	8.47
217405_x_at	glycosylphosphati	GPLD1	5.33	5.11	5.35
220824_at	---	---	6.32	6.59	6.37
219954_s_at	glucosidase, beta,	GBA3	3.91	3.62	3.74
213612_x_at	neuroblastoma br	NBPF10 /// NBPF1	11.58	11.74	11.75
204919_at	proline rich 4 (lacr	PRR4	5.64	5.52	5.85
205801_s_at	RAS guanyl releas	RASGRP3	3.81	3.72	3.31
208016_s_at	angiotensin II rece	AGTR1	3.90	3.89	3.64
218814_s_at	transmembrane p	TMEM206	4.48	4.52	4.63
204711_at	KIAA0753	KIAA0753	8.30	8.28	8.34
201796_s_at	valyl-tRNA synthe	VARS	8.09	8.27	7.91
209430_at	BTAF1 RNA polym	BTAF1	9.29	9.44	9.62
212662_at	poliovirus recepto	PVR	9.61	9.24	9.46
218536_at	MRS2 magnesium	MRS2	8.02	8.13	7.96
217942_at	mitochondrial ribo	MRPS35	10.92	10.87	10.95
206613_s_at	TATA box binding	TAF1A	8.03	7.97	8.11
215126_at	---	---	4.37	4.27	4.31
220657_at	kelch-like 11 (Dros	KLHL11	4.65	5.12	4.52
216857_at	Interleukin 23, alp	IL23A	5.22	4.71	4.39
211499_s_at	mitogen-activated	MAPK11	6.72	6.64	6.96
217965_s_at	SAP30 binding pro	SAP30BP	9.02	8.93	9.16
201207_at	tumor necrosis fac	TNFAIP1	8.52	8.76	8.51
205147_x_at	neutrophil cytoso	NCF4	5.57	5.22	5.13
218232_at	complement com	C1QA	5.63	5.54	4.99
202093_s_at	Paf1, RNA polyme	PAF1	8.48	8.34	8.38

208466_at	RAB3D, member F	RAB3D	6.34	6.45	6.08
213542_at	zinc finger protein	ZNF710	8.10	8.07	8.05
221546_at	PRP18 pre-mRNA	PRPF18	4.87	4.95	4.79
201947_s_at	chaperonin contai	CCT2	12.72	12.76	12.73
202658_at	peroxisomal bioge	PEX11B	8.66	8.86	8.79
210805_x_at	runt-related trans	RUNX1	4.38	4.49	4.35
207102_at	aldo-keto reducta	AKR1D1	3.36	3.26	3.22
200624_s_at	matrin 3	MATR3	11.26	11.42	11.48
221378_at	cerberus 1, cystei	CER1	5.56	5.66	5.39
205193_at	v-maf musculoapo	MAFF	8.06	7.72	8.21
211869_at	---	---	5.59	5.51	5.55
217402_at	---	---	3.56	3.28	3.58
221428_s_at	transducin (beta)-	TBL1XR1	9.70	9.82	9.94
205672_at	xeroderma pigme	XPA	7.77	7.90	7.96
209299_x_at	peptidylprolyl isor	PPIL2	8.00	8.13	8.11
215295_at	dystrobrevin, beta	DTNB	3.57	3.82	3.51
211790_s_at	myeloid/lymphoic	MLL2	3.73	3.38	3.40
211818_s_at	phosphodiesteras	PDE4C	6.21	6.33	6.06
202817_s_at	synovial sarcoma	SS18	8.39	8.60	8.66
220982_s_at	sperm acrosome a	SPACA1	4.24	4.35	4.42
221942_s_at	guanylate cyclase	GUCY1A3	4.58	4.87	4.83
220220_at	leucine rich repea	LRRC37A4	3.96	3.77	3.95
212574_x_at	chromosome 19 o	C19orf6	6.25	5.95	6.05
209840_s_at	leucine rich repea	LRRN3	3.57	3.49	3.47
218861_at	ring finger protein	RNF25	7.38	7.27	7.18
219071_x_at	chromosome 8 op	C8orf30A	8.25	8.09	7.99
217385_at	similar to melanoi	LOC392555	5.02	4.88	4.51
212781_at	retinoblastoma bi	RBBP6	7.71	7.86	7.73
212402_at	zinc finger CCCH-t	ZC3H13	9.87	10.00	9.90
212444_at	---	---	6.28	6.15	6.00
222149_x_at	golgin A8 family, r	GOLGA8C /// GOL(	7.36	7.57	7.44
215563_s_at	macrophage stimu	MST1P9	3.67	3.62	3.22
200994_at	importin 7	IPO7	11.18	11.44	11.72
208364_at	inositol polyphosp	INPP4A	5.26	5.28	4.75
220042_x_at	human immunode	HIVEP3	7.16	7.23	7.06
218372_at	mediator comple)	MED9	6.68	6.74	6.60
215793_at	myotubularin rela	MTMR7	3.41	3.22	3.17
203917_at	coxsackie virus an	CXADR	7.92	8.27	8.33
214226_at	protease, serine, 5	PRSS53	6.58	6.82	6.27
213701_at	chromosome 12 o	C12orf29	10.14	10.10	10.34
218211_s_at	melanophilin	MLPH	8.05	7.87	7.73
203389_at	kinesin family mei	KIF3C	6.61	6.53	6.08
210328_at	glycine N-methylt	GNMT	5.07	5.12	5.13
207648_at	dystrophin relate(	DRP2	6.41	6.59	6.01
203256_at	cadherin 3, type 1	CDH3	4.87	4.98	5.15
201655_s_at	heparan sulfate pi	HSPG2	8.94	8.89	8.81
215640_at	TBC1 domain fam	TBC1D2B	6.83	7.20	6.75

214601_at	tryptophan hydro	TPH1	3.11	3.43	3.19
222056_s_at	fumarylacetoacet	FAHD2A	9.11	9.08	9.23
203923_s_at	cytochrome b-245	CYBB	5.31	4.98	4.96
217451_at	---	---	3.68	3.75	3.65
213176_s_at	latent transformir	LTBP4	6.41	6.49	5.98
215482_s_at	eukaryotic transla	EIF2B4	9.99	9.90	9.85
211879_x_at	protocadherin gar	PCDHGA3	4.78	5.08	4.72
216876_s_at	interleukin 17A	IL17A	3.38	3.26	3.24
209804_at	DNA cross-link rep	DCLRE1A	8.36	8.24	8.38
222180_at	---	---	4.83	5.19	5.11
216587_s_at	frizzled homolog 8	FZD8	5.74	5.52	5.20
203664_s_at	polymerase (RNA)	POLR2D	8.84	8.89	8.99
217173_s_at	low density lipopr	LDLR	7.01	7.22	6.86
200071_at	survival motor ne	SMNDC1	11.03	11.04	11.28
203722_at	aldehyde dehydro	ALDH4A1	6.18	6.34	6.42
201156_s_at	RAB5C, member R	RAB5C	8.40	8.38	8.52
213783_at	MFNG O-fucosylp	MFNG	6.53	6.15	6.20
220588_at	breast carcinoma	BCAS4	7.23	7.25	7.24
217954_s_at	PHD finger protei	PHF3	10.20	10.31	10.41
201851_at	SH3-domain GRB2	SH3GL1	9.99	9.86	9.71
210577_at	calcium-sensing re	CASR	6.18	6.05	5.94
210977_s_at	heat shock transc	HSF4	7.33	7.15	6.93
79005_at	solute carrier fam	SLC35E1	8.21	8.33	8.37
205363_at	butyrobetaine (ga	BBOX1	3.35	3.35	3.31
215460_x_at	bromodomain cor	BRD1	7.89	7.87	7.97
221948_s_at	Kelch-like 22 (Dro	KLHL22	4.43	4.57	4.48
205632_s_at	phosphatidylinosi	PIP5K1B	3.06	3.15	3.36
206145_at	Rh-associated glyc	RHAG	3.30	3.42	2.93
216115_at	neurofibromin 1	NF1	6.00	6.40	6.17
212573_at	endonuclease dor	ENDOD1	7.84	8.10	8.05
213709_at	basic helix-loop-h	BHLHB9	5.26	5.28	5.20
216273_at	zinc finger protein	ZNF460	5.16	5.01	5.10
220995_at	syntaxin binding p	STXBP6	4.53	4.22	4.07
210682_at	lactoperoxidase	LPO	4.48	4.09	4.50
220319_s_at	myosin regulatory	MYLIP	7.68	7.50	7.80
211832_s_at	Mdm2 p53 bindin	MDM2	4.03	4.14	3.88
208330_at	ALX homeobox 4	ALX4	5.99	5.60	5.75
213179_at	RCD1 required for	RQCD1	9.56	9.34	9.46
209440_at	phosphoribosyl py	PRPS1	10.78	10.82	10.76
215866_at	---	---	5.05	4.69	4.63
222145_at	---	---	5.40	5.30	5.17
201018_at	eukaryotic transla	EIF1AX	9.55	9.57	9.73
221360_s_at	growth hormone s	GHSR	4.80	4.76	4.87
204898_at	---	---	4.76	4.97	4.98
220286_at	myotubularin rela	MTMR10	5.10	4.88	4.76
215262_at	---	---	4.16	4.26	4.55
215577_at	---	---	6.76	6.91	6.51

204198_s_at	runt-related trans	RUNX3	4.38	4.43	4.07
37965_at	parvin, beta	PARVB	6.49	6.79	6.57
217377_x_at	neurotrophic tyro	NTRK3	6.21	6.06	5.80
211474_s_at	serpin peptidase i	SERPINB6	10.12	10.04	10.21
220507_s_at	ureidopropionase	UPB1	4.98	4.92	4.37
219362_at	N(alpha)-acetyltra	NAA35	8.81	8.86	8.99
205967_at	histone cluster 1,	HIST1H4C	14.41	14.31	14.42
207188_at	cyclin-dependent	CDK3	6.83	6.82	6.31
217897_at	FXYD domain cont	FXYD6	6.79	6.72	6.24
204925_at	cystinosis, nephro	CTNS	7.00	6.86	6.65
215394_at	phosphoinositide-	PIK3C3	5.42	5.38	5.05
220315_at	poly (ADP-ribose)	PARP11	4.72	4.86	4.88
218996_at	TCF3 (E2A) fusion	TFPT	8.12	7.93	8.03
214436_at	F-box and leucine-	FBXL2	7.25	7.33	7.47
210830_s_at	paraoxonase 2	PON2	10.85	10.97	10.91
213408_s_at	phosphatidylinosi	PI4KA /// PI4KAP1	9.41	9.16	9.32
219677_at	splA/ryanodine re	SPSB1	4.54	4.96	4.69
210099_at	ATP-binding casse	ABCA2	6.65	6.32	6.25
208373_s_at	pyrimidinergic rec	P2RY6	6.59	6.37	5.73
200702_s_at	DEAD (Asp-Glu-Ala	DDX24	9.31	9.51	9.49
219401_at	xylosyltransferase	XYLT2	7.85	7.70	7.68
206162_x_at	synaptotagmin V	SYT5	6.07	5.97	5.77
213779_at	EMI domain conta	EMID1	6.26	6.43	5.93
205963_s_at	DnaJ (Hsp40) hom	DNAJA3	10.22	10.24	10.36
216083_s_at	sialidase 3 (memb	NEU3	5.60	5.53	5.12
201475_x_at	methionyl-tRNA s	MARS	10.98	10.68	10.85
208527_x_at	histone cluster 1,	HIST1H2BE	8.01	7.86	7.86
219917_at	zinc finger, CCHC (	ZCCHC4	6.55	6.71	6.59
208438_s_at	Gardner-Rasheed	FGR	5.51	5.72	5.32
217678_at	solute carrier fam	SLC7A11	11.23	11.11	11.39
204988_at	fibrinogen beta ch	FGB	4.68	4.70	4.41
209851_at	zinc finger CCCH-t	ZC3H13	4.99	5.38	5.38
213828_x_at	H3 histone, family	H3F3A /// LOC440	13.59	13.63	13.61
205667_at	Werner syndrome	WRN	8.31	8.40	8.51
205383_s_at	zinc finger and BT	ZBTB20	9.45	9.49	9.63
220722_s_at	solute carrier fam	SLC5A7	4.31	4.41	4.12
211585_at	nuclear protein, a	NPAT	3.85	3.63	3.66
209294_x_at	tumor necrosis fa	TNFRSF10B	7.43	7.40	7.63
203971_at	solute carrier fam	SLC31A1	9.97	9.85	10.07
216087_at	---	---	3.58	3.35	3.23
220162_s_at	caspase recruitme	CARD9	5.30	4.84	4.64
207024_at	cholinergic recept	CHRND	6.55	6.21	6.10
203593_at	CD2-associated pr	CD2AP	8.93	9.20	9.22
209751_s_at	trafficking protein	TRAPPC2 /// TRAP	8.64	8.54	8.90
214399_s_at	hypothetical prote	LOC100288809	6.65	6.33	5.97
201001_s_at	transmembrane p	TMEM189 /// TME	9.28	9.38	9.50
220544_at	testis-specific seri	TSKS	6.05	5.61	5.47

208339_at	XK, Kell blood gro	XKRY /// XKRY2	3.49	3.68	3.49
216376_x_at	---	---	5.62	5.65	5.51
221210_s_at	N-acetylneuramin	NPL	4.83	4.61	4.64
220308_at	coiled-coil domair	CCDC19	7.19	7.08	6.89
206325_at	serpin peptidase i	SERPINA6	5.00	5.10	4.99
218685_s_at	single-strand-sele	SMUG1	7.54	7.59	7.42
214676_x_at	mucin 3A, cell sur	MUC3A /// MUC3I	6.13	5.73	5.87
217340_at	ribosomal protein	RPL21 /// RPL21P2	4.87	4.67	4.80
206623_at	phosphodiesteras	PDE6A	5.11	4.90	4.57
205556_at	msh homeobox 2	MSX2	3.81	3.64	3.91
209338_at	transcription facto	TFCP2	8.14	8.22	8.39
213490_s_at	mitogen-activatec	MAP2K2	6.55	6.47	6.62
221353_at	olfactory receptor	OR3A1	4.89	5.15	5.04
213505_s_at	splicing factor, arg	SFRS14	8.77	8.61	8.76
220046_s_at	cyclin L1	CCNL1	9.91	9.92	9.87
220656_at	---	---	2.95	3.06	2.99
215956_at	---	---	6.40	6.64	6.43
220059_at	signal transducing	STAP1	3.70	3.74	3.68
205814_at	glutamate recepto	GRM3	4.69	4.92	4.37
201597_at	cytochrome c oxic	COX7A2	12.72	12.79	12.85
203360_s_at	c-myc binding pro	MYCBP	9.43	9.57	9.50
215474_at	---	---	4.07	4.18	3.80
215524_x_at	T cell receptor alp	TRA@ /// TRAC ///	3.95	3.68	3.89
206802_at	paired box 5	PAX5	6.36	6.42	6.44
204158_s_at	T-cell, immune reg	TCIRG1	7.82	7.89	7.85
205772_s_at	---	---	3.89	3.85	3.59
47083_at	chromosome 7 op	C7orf26	9.27	9.13	9.13
212902_at	SEC24 family, mer	SEC24A	7.16	7.23	7.42
206206_at	CD180 molecule	CD180	7.24	7.27	7.06
219421_at	tetratricopeptide	TTC33	7.98	8.21	8.35
218821_at	aminopeptidase-li	NPEPL1	7.18	6.76	6.97
215672_s_at	adenosylhomocys	AHCYL2	5.41	5.26	5.40
212581_x_at	glyceraldehyde-3-	GAPDH	14.71	14.67	14.64
222281_s_at	---	---	5.19	5.23	5.18
201333_s_at	Rho guanine nucle	ARHGEF12	6.68	6.83	6.63
212977_at	chemokine (C-X-C	CXCR7	5.98	6.13	6.20
204991_s_at	neurofibromin 2 (	NF2	6.18	6.19	6.32
221819_at	RAB35, member F	RAB35	9.27	9.22	9.16
212200_at	ankyrin repeat an	ANKLE2	8.10	8.27	8.38
216657_at	ataxin 3	ATXN3	3.56	3.59	3.58
218434_s_at	acetoacetyl-CoA s	AACS	9.28	9.38	9.39
200632_s_at	N-myc downstrea	NDRG1	10.20	10.16	10.22
209957_s_at	natriuretic peptid	NPPA	7.10	6.73	6.90
38149_at	Rho GTPase activa	ARHGAP25	5.63	5.65	5.50
206409_at	T-cell lymphoma i	TIAM1	4.57	4.33	4.11
AFFX-PheX-M_a	---	---	8.12	8.06	7.91
219773_at	NADPH oxidase 4	NOX4	4.46	4.24	4.30

215226_at	Exophilin 5	EXPH5	6.17	5.80	5.93
207516_at	cholinergic recept	CHRNB4	4.49	4.22	4.34
218422_s_at	RNA binding moti	RBM26	10.07	10.27	10.36
202367_at	cut-like homeobo	CUX1	7.17	7.49	6.97
217180_at	---	---	6.73	6.73	6.51
201425_at	aldehyde dehydro	ALDH2	5.86	5.54	5.45
218626_at	eukaryotic transla	EIF4ENIF1	8.82	8.78	8.74
205468_s_at	interferon regulat	IRF5	6.25	6.04	5.79
219114_at	chromosome 3 op	C3orf18	7.59	7.42	7.12
202221_s_at	E1A binding prote	EP300	7.89	7.53	8.08
220460_at	solute carrier orga	SLCO1C1	3.74	3.55	3.31
204236_at	Friend leukemia v	FLI1	3.81	3.81	4.12
220910_at	Fraser syndrome 1	FRAS1	5.16	5.13	5.01
205618_at	proline rich Gla (G	PRRG1	9.34	9.29	9.44
216625_at	---	---	3.14	3.21	3.13
208406_s_at	GRB2-related ada	GRAP2	6.35	6.08	6.35
215744_at	fused in sarcoma	FUS	6.80	6.73	6.83
214788_x_at	dendrin	DDN	6.00	6.01	6.08
218489_s_at	aminolevulinate d	ALAD	6.94	7.14	6.83
219564_at	potassium inward	KCNJ16	3.37	3.48	3.24
213629_x_at	metallothionein 1	MT1F	9.95	10.10	10.01
215963_x_at	similar to ribosom	LOC100294182 ///	14.32	14.30	14.47
221420_at	---	---	3.88	3.79	3.78
207967_at	vacuolar protein s	VPS45	5.86	5.71	5.52
209841_s_at	leucine rich repea	LRRN3	3.93	4.00	4.25
201172_x_at	ATPase, H+ transp	ATP6V0E1	11.85	11.88	11.85
217000_at	---	---	6.03	5.92	6.15
202435_s_at	cytochrome P450,	CYP1B1	4.03	4.01	3.86
201821_s_at	translocase of inn	TIMM17A	10.61	10.84	10.85
209356_x_at	EGF-containing fib	EFEMP2	5.40	5.29	5.24
214090_at	PRKC, apoptosis, V	PAWR	4.76	4.62	4.81
208594_x_at	leukocyte immuno	LILRA6	6.75	6.66	6.40
221597_s_at	transmembrane p	TMEM208	10.67	10.58	10.52
222069_s_at	hypothetical prote	LOC100287697	5.82	5.47	5.57
217643_x_at	---	---	4.87	4.98	5.05
212340_at	Yip1 domain famil	YIPF6	7.37	7.54	7.49
205733_at	Bloom syndrome,	BLM	8.44	8.55	8.62
215077_at	---	---	3.60	3.52	3.49
222280_at	---	---	8.85	8.70	8.81
215844_at	transportin 2	TNPO2	7.57	7.11	7.39
205513_at	transcobalamin I (	TCN1	4.29	4.04	3.99
91952_at	DDB1 and CUL4 as	DCAF15	6.42	6.37	6.22
210755_at	hepatocyte growt	HGF	3.34	3.35	3.33
200628_s_at	tryptophanyl-tRN/	WARS	9.04	8.75	9.01
218648_at	CREB regulated tr	CRTC3	9.94	9.67	9.83
222273_at	poly(A) polymeras	PAPOLG	6.70	6.94	7.25
203529_at	protein phosphata	PPP6C	9.95	10.01	10.01

209816_at	patched homolog	PTCH1	4.25	4.33	3.99
206407_s_at	chemokine (C-C m	CCL13	6.38	6.37	6.12
206216_at	SFRS protein kinas	SRPK3	5.58	5.95	5.56
215755_at	---	---	4.76	4.56	4.50
212101_at	karyopherin alpha	KPNA6	8.35	8.35	8.48
219949_at	leucine rich repea	LRRC2	3.33	3.35	3.17
209867_s_at	latrophilin 3	LPHN3	8.90	8.68	8.78
218047_at	oxysterol binding	OSBPL9	10.43	10.45	10.63
215182_x_at	---	---	7.21	7.01	6.75
208206_s_at	RAS guanyl releasi	RASGRP2	5.28	4.91	4.63
219378_at	N(alpha)-acetyltra	NAA16	8.11	8.22	8.31
217383_at	Phosphoglycerate	PGK1	6.00	5.95	5.89
210704_at	fasciculation and (	FEZ2	4.57	4.63	4.75
206197_at	non-metastatic ce	NME5	3.89	4.11	4.27
216052_x_at	artemin	ARTN	5.65	5.96	5.58
202787_s_at	mitogen-activated	MAPKAPK3	8.65	8.68	8.77
207197_at	Zic family membe	ZIC3	3.97	4.08	4.13
222212_s_at	LAG1 homolog, ce	LASS2	10.83	10.77	10.89
215749_s_at	golgi reassembly s	GORASP1	7.98	8.10	8.17
208604_s_at	homeobox A3	HOXA3	6.46	6.39	6.15
207029_at	KIT ligand	KITLG	4.88	5.12	4.57
220070_at	jumonji domain co	JMJD5	6.23	6.38	6.22
218523_at	phospholysine ph	LHPP	6.96	6.66	6.76
216770_at	---	---	3.76	3.72	3.80
210211_s_at	heat shock protei	HSP90AA1	13.77	13.94	13.87
217057_s_at	GNAS complex loc	GNAS	4.78	4.62	4.15
212155_at	ring finger protein	RNF187	10.14	9.89	10.08
205838_at	glycophorin A (MI	GYPA /// GYPB	4.34	4.29	4.17
204387_x_at	mitochondrial ribo	MRP63	8.84	8.91	8.76
221805_at	neurofilament, lig	NEFL	3.73	3.47	3.32
212647_at	related RAS viral (	RRAS	9.13	9.05	8.97
212929_s_at	family with seque	FAM21A /// FAM2	8.91	8.95	8.98
204600_at	EPH receptor B3	EPHB3	7.40	7.48	7.30
202611_s_at	mediator complex	MED14	7.96	7.90	8.09
211802_x_at	calcium channel, v	CACNA1G	7.05	6.92	6.45
206339_at	CART prepropepti	CARTPT	6.26	6.36	6.32
204710_s_at	WD repeat domai	WIPI2	10.21	10.22	10.13
217195_at	---	---	4.39	4.51	4.34
212888_at	dicer 1, ribonucle	DICER1	8.20	8.43	8.41
222337_at	---	---	6.35	6.08	6.28
221349_at	pre-B lymphocyte	VPREB1	4.52	4.21	4.38
219460_s_at	transmembrane p	TMEM127	8.21	8.36	8.16
220542_s_at	palate, lung and n	PLUNC	4.78	5.02	4.87
220048_at	ectodysplasin A re	EDAR	6.24	6.11	6.02
212295_s_at	solute carrier fam	SLC7A1	12.34	12.00	12.34
203218_at	mitogen-activated	MAPK9	9.54	9.59	9.70
219431_at	Rho GTPase activa	ARHGAP10	8.72	8.52	8.58

208346_at	pro-platelet basic	PPBPL2	3.92	4.11	4.16
215060_at	---	---	3.72	3.64	4.02
215238_s_at	dedicator of cytok	DOCK9	6.41	6.04	6.27
221698_s_at	C-type lectin dom	CLEC7A	4.37	4.30	4.04
206863_x_at	---	---	4.78	4.93	5.09
218466_at	TBC1 domain fam	TBC1D17	7.53	7.49	7.51
222003_s_at	dedicator of cytok	DOCK6	5.69	5.18	5.49
216512_s_at	dopachrome taut	DCT	5.03	4.74	4.62
219852_s_at	MORN repeat con	MORN1	7.85	7.56	7.61
215118_s_at	Immunoglobulin h	IGHA1	3.90	4.32	4.50
211234_x_at	estrogen receptor	ESR1	6.57	6.93	6.60
212604_at	mitochondrial rib	MRPS31	9.59	9.52	9.57
211549_s_at	hydroxyprostaglar	HPGD	5.68	5.11	5.09
207829_s_at	BCL2/adenovirus	BNIP1	8.47	8.56	8.69
211635_x_at	immunoglobulin h	IGH@ /// IGHA1 //	6.92	6.79	6.46
220604_x_at	formiminotransfe	FTCD	6.57	6.22	6.39
220503_at	solute carrier fam	SLC13A1	3.28	3.27	3.11
217203_at	---	---	5.78	5.51	5.49
203666_at	chemokine (C-X-C	CXCL12	5.15	5.22	5.11
200008_s_at	GDP dissociation i	GDI2	10.79	10.78	10.68
214934_at	ATPase, class II, ty	ATP9B	7.13	7.24	7.27
210033_s_at	sperm associated	SPAG6	4.73	4.30	4.07
200097_s_at	heterogeneous nu	HNRNPK	12.55	12.70	12.72
91826_at	EPS8-like 1	EPS8L1	6.92	6.92	6.83
216916_s_at	discs, large (Drosc	DLGAP2	6.01	5.14	5.83
214903_at	synaptotagmin II	SYT2	6.57	6.68	5.96
205428_s_at	calbindin 2	CALB2	6.01	5.81	5.66
221369_at	melatonin recept	MTNR1A	5.04	5.25	5.16
209865_at	solute carrier fam	SLC35A3	6.50	6.94	6.79
219839_x_at	T-cell leukemia/ly	TCL6	4.93	5.07	4.85
208285_at	olfactory receptor	OR7A5	3.91	4.17	3.85
206169_x_at	zinc finger CCCH-t	ZC3H7B	7.57	7.56	7.68
208589_at	transient receptor	TRPC7	3.99	4.08	3.99
213089_at	hypothetical LOC1	LOC100272216	8.39	8.63	8.20
219977_at	aryl hydrocarbon	AIPL1	5.58	5.27	5.33
210848_at	---	---	4.35	4.27	3.91
214878_at	zinc finger protein	ZNF37A /// ZNF37	5.08	5.45	5.26
219933_at	glutaredoxin 2	GLRX2	10.50	10.45	10.58
207176_s_at	CD80 molecule	CD80	4.69	4.61	4.48
207392_x_at	UDP glucuronosyl	UGT2B15	4.08	4.20	4.01
220745_at	interleukin 19	IL19	5.95	6.04	5.96
220207_at	Yip1 interacting fa	YIF1B	7.32	7.50	7.30
202399_s_at	adaptor-related p	AP3S2	9.52	9.36	9.54
219376_at	zinc finger protein	ZNF322B	7.10	7.40	7.34
215263_at	zinc finger, X-linke	ZXDA /// ZXDB	5.05	4.95	4.84
203995_at	chromosome 21 o	C21orf2	6.27	6.32	6.30
206682_at	C-type lectin dom	CLEC10A	5.85	5.45	5.67

200033_at	DEAD (Asp-Glu-Al	DDX5	12.36	12.59	12.53
207031_at	NK3 homeobox 2	NKX3-2	4.06	4.40	4.04
217549_at	---	---	5.99	5.81	6.40
214029_at	Solute carrier fam	SLC25A42	4.78	4.48	4.43
201840_at	neural precursor c	NEDD8	11.38	11.36	11.37
219762_s_at	ribosomal protein	RPL36	14.01	13.95	14.03
211276_at	transcription elon	TCEAL2	3.81	3.68	3.66
207729_at	cadherin 9, type 2	CDH9	3.69	3.46	3.63
211606_at	---	---	3.63	3.47	3.15
208706_s_at	eukaryotic transla	EIF5	11.44	11.36	11.57
45633_at	GINS complex sub	GINS3	8.03	8.01	8.16
203357_s_at	calpain 7	CAPN7	8.02	8.00	8.13
216628_at	---	---	5.22	4.92	5.18
207189_s_at	zinc finger, ZZ-typ	ZZEF1	6.74	6.65	6.03
215872_at	---	---	3.63	3.64	3.66
208776_at	proteasome (pros	PSMD11	7.27	7.67	7.47
222289_at	Potassium voltage	KCNC2	5.57	5.43	5.46
200597_at	eukaryotic transla	EIF3A	9.80	9.76	9.86
214497_s_at	nescient helix loop	NHLH2	4.78	4.67	4.59
202126_at	PRP4 pre-mRNA p	PRPF4B	9.44	9.63	9.75
207957_s_at	protein kinase C, b	PRKCB	4.07	4.23	4.15
206157_at	pentraxin 3, long	PTX3	11.52	11.37	11.68
202904_s_at	LSM5 homolog, U	LSM5	8.46	8.95	8.99
200676_s_at	ubiquitin-conjugat	UBE2L3	7.23	7.38	7.14
208083_s_at	integrin, beta 6	ITGB6	3.51	3.33	3.51
208008_at	TBC1 domain fam	TBC1D29	6.28	5.97	5.95
207661_s_at	SH3 and PX doma	SH3PXD2A	3.84	3.85	3.79
221066_at	relaxin/insulin-like	RXFP3	4.35	4.21	4.23
202707_at	uridine monophos	UMPS	5.58	5.15	5.24
208057_s_at	GLI family zinc fin	GLI2	5.44	5.16	5.11
219204_s_at	serine racemase	SRR	6.31	6.17	6.32
216621_at	---	---	5.42	5.59	5.42
222325_at	---	---	3.31	3.35	3.34
219052_at	Hermansky-Pudlak	HPS6	8.35	8.27	8.34
219781_s_at	zinc finger protein	ZNF771	8.02	8.04	7.72
201885_s_at	cytochrome b5 re	CYB5R3	11.99	11.97	11.82
203681_at	isovaleryl-CoA deh	IVD	4.19	4.20	3.90
209503_s_at	proteasome (pros	PSMC5	11.17	11.18	11.21
206234_s_at	matrix metallopep	MMP17	6.89	6.70	6.25
213308_at	SH3 and multiple	SHANK2	4.27	4.33	4.40
215052_at	FERM and PDZ do	FRMPD4	5.24	4.95	4.68
218839_at	hairy/enhancer-of	HEY1	6.71	6.63	6.77
221032_s_at	transmembrane p	TMPRSS5	7.07	6.76	6.59
210123_s_at	CHRNA7 (choliner	CHRFAM7A /// CH	4.46	4.50	4.72
207259_at	chromosome 17 c	C17orf73	3.60	3.31	3.11
207249_s_at	solute carrier fam	SLC28A2	5.13	4.90	5.03
202517_at	collapsin response	CRMP1	5.40	5.55	5.11

205288_at	CDC14 cell divisio	CDC14A	3.66	3.88	3.74
210728_s_at	calcitonin-related	CALCA	5.66	5.55	5.40
202024_at	arsA arsenite tran	ASNA1	8.44	8.56	8.49
208822_s_at	death associated	DAP3	11.22	11.18	11.33
218715_at	UTP6, small subur	UTP6	10.82	10.77	10.78
219734_at	SID1 transmembr	SIDT1	3.43	3.50	3.18
206094_x_at	UDP glucuronosyl	UGT1A1 /// UGT1/	5.83	5.77	5.90
219549_s_at	reticulon 3	RTN3	10.83	10.85	10.87
203128_at	serine palmitoyltr	SPTLC2	7.20	7.47	7.38
214404_x_at	SAM pointed dom	SPDEF	7.54	7.42	7.26
203205_at	lysine (K)-specific	KDM4A	8.24	8.07	8.62
201484_at	suppressor of Ty 4	SUPT4H1	8.54	8.53	8.68
221345_at	free fatty acid rec	FFAR2	4.66	4.99	4.50
220112_at	ankyrin repeat do	ANKRD55	3.87	3.89	3.74
216078_at	---	---	5.47	5.68	5.52
207073_at	cyclin-dependent	CDKL2	4.95	5.07	4.70
215316_at	---	---	4.95	5.10	4.77
209102_s_at	HMG-box transcri	HBP1	9.14	9.03	9.24
214205_x_at	glutaredoxin 3	GLRX3	9.08	9.15	9.11
217428_s_at	collagen, type X, a	COL10A1	3.55	3.82	3.57
209432_s_at	cAMP responsive	CREB3	9.37	9.34	9.23
212697_at	family with seque	FAM134C	9.27	9.23	9.30
207241_at	chromosome 4 op	C4orf6	3.80	3.65	4.14
201154_x_at	ribosomal protein	RPL4	14.50	14.45	14.55
208598_s_at	HECT, UBA and W	HUWE1	10.05	9.88	9.96
216513_at	dopachrome taut	DCT	3.28	3.63	3.48
201449_at	TIA1 cytotoxic gra	TIA1	7.70	7.89	7.77
219404_at	EPS8-like 3	EPS8L3	4.82	5.12	5.29
215256_x_at	Rho GTPase activa	ARHGAP33	6.60	6.49	6.74
205712_at	protein tyrosine p	PTPRD	4.04	4.29	4.29
214464_at	CDC42 binding pr	CDC42BPA	10.76	10.73	10.80
216539_at	ataxin 3-like	ATXN3L	3.43	3.20	3.48
214761_at	zinc finger protein	ZNF423	3.94	4.14	3.86
221379_at	---	---	8.17	8.08	7.58
218207_s_at	stathmin-like 3	STMN3	4.45	4.71	4.78
202044_at	glucocorticoid rec	GRLF1	6.98	6.90	6.91
202250_s_at	DDB1 and CUL4 as	DCAF8	7.32	7.45	7.44
220630_s_at	chitinase, acidic	CHIA	5.86	5.32	5.99
204987_at	inter-alpha (globu	ITIH2	4.28	4.22	4.17
215873_x_at	ATP-binding casse	ABCC10	7.17	7.42	7.41
219127_at	proline rich 15-lik	PRR15L	4.61	4.74	4.29
202308_at	sterol regulatory e	SREBF1	9.04	8.93	8.90
201684_s_at	TOX high mobility	TOX4	9.38	9.39	9.42
213509_x_at	carboxylesterase 2	CES2	9.30	9.28	9.40
215317_at	---	---	3.77	3.86	3.81
210814_at	transient receptor	TRPC3	3.56	3.84	3.30
202561_at	tankyrase, TRF1-in	TNKS	9.51	9.47	9.49

216795_at	---	---	3.76	3.20	3.12
211437_at	---	---	3.45	3.37	3.45
214343_s_at	ataxin 7-like 1	ATXN7L1	5.13	5.01	4.49
208185_x_at	---	---	8.22	7.92	8.00
213879_at	---	---	8.43	8.42	8.36
204545_at	peroxisomal bioge	PEX6	6.20	6.27	6.14
220193_at	chromosome 1 op	C1orf113	5.17	5.14	4.75
214382_at	unc-93 homolog A	UNC93A	3.62	3.39	3.30
213794_s_at	neuroguidin, EIF4I	NGDN	10.00	10.13	10.17
207281_x_at	variable charge, X	VCX2	6.10	5.82	6.00
214239_x_at	polycomb group r	PCGF2	8.55	8.53	8.58
207093_s_at	oligodendrocyte r	OMG	4.89	4.79	4.51
207631_at	neighbor of BRCA	NBR2	7.35	6.92	6.88
201881_s_at	ariadne homolog,	ARIH1	8.74	8.94	8.82
210158_at	excision repair cro	ERCC4	4.57	4.80	4.47
207022_s_at	lactate dehydroge	LDHC	7.06	6.74	6.67
204420_at	FOS-like antigen 1	FOSL1	9.90	9.81	10.01
220618_s_at	zinc finger, CW typ	ZCWPW1	6.43	6.62	6.39
216638_s_at	prolactin receptor	PRLR	3.81	3.57	3.42
207331_at	---	---	4.56	4.88	4.82
206937_at	spectrin, alpha, er	SPTA1	3.21	3.78	3.48
201223_s_at	RAD23 homolog B	RAD23B	10.94	11.00	11.07
209137_s_at	ubiquitin specific	USP10	9.52	9.55	9.58
215924_at	---	---	3.18	3.14	3.05
207422_at	ADAM metallopep	ADAM20	5.99	6.09	5.99
203639_s_at	fibroblast growth	FGFR2	3.38	3.39	3.84
203644_s_at	MON1 homolog B	MON1B	8.37	8.05	8.12
217124_at	IQ motif containir	IQCE	4.87	4.89	4.91
217201_at	RAS protein activa	RASAL2	3.78	3.69	3.68
220883_at	hypothetical prote	PRO2012	3.71	3.72	3.42
208076_at	histone cluster 1,	HIST1H4D	5.35	5.17	4.96
205178_s_at	retinoblastoma bi	RBBP6	7.91	8.35	8.37
213447_at	imprinted in Prad	IPW	7.88	8.15	8.16
214759_at	Wilms tumor 1 as	WTAP	5.10	5.36	5.21
215579_at	apolipoprotein B r	APOBEC3G	7.16	6.95	7.00
216391_s_at	kelch-like 1 (Dros	KLHL1	6.20	6.23	5.91
202073_at	optineurin	OPTN	8.37	8.59	8.55
200934_at	DEK oncogene	DEK	12.05	12.21	12.27
201368_at	zinc finger protein	ZFP36L2	12.04	11.97	12.06
215145_s_at	contactin associat	CNTNAP2	4.71	4.66	4.82
217833_at	synaptotagmin bi	SYNCRIP	11.29	11.20	11.43
201823_s_at	ring finger protein	RNF14	8.61	8.83	8.87
217588_at	cation channel, sp	CATSPER2 /// CATS	6.14	5.62	5.63
213679_at	tetratricopeptide	TTC30A	5.07	4.91	4.72
203667_at	tubulin folding co	TBCA	12.65	12.67	12.72
219085_s_at	gem (nuclear orga	GEMIN7	6.44	6.14	5.73
213194_at	roundabout, axon	ROBO1	10.49	10.35	10.49

208142_at	epididymal protei	EDDM3A	3.97	3.51	3.43
213435_at	SATB homeobox 2	SATB2	9.09	8.98	9.14
217904_s_at	beta-site APP-clea	BACE1	6.40	6.51	6.51
203924_at	glutathione S-tran	GSTA1	4.11	4.21	4.12
200812_at	chaperonin contai	CCT7	12.62	12.53	12.55
210712_at	lactate dehydroge	LDHAL6B	3.42	3.40	3.44
207400_at	neuropeptide Y re	NPY5R	4.02	4.00	4.06
215550_at	SLIT-ROBO Rho GT	SRGAP3	4.94	5.05	4.70
210787_s_at	calcium/calmodul	CAMKK2	6.76	6.62	6.47
219647_at	popeye domain co	POPDC2	3.84	3.86	3.95
210569_s_at	sialic acid binding	SIGLEC9	4.93	5.34	4.64
221567_at	nucleolar protein	NOL3	8.27	8.26	8.22
217594_at	zinc finger, CCHC	ZCCHC11	4.27	4.28	3.90
210701_at	craniofacial devel	CFDP1	6.56	6.29	6.05
207774_at	ATG10 autophagy	ATG10	5.56	5.49	5.53
203698_s_at	frizzled-related pr	FRZB	3.95	3.90	3.77
212317_at	transportin 3	TNPO3	9.12	9.05	9.21
213859_x_at	SWI/SNF related,	SMARCA5	8.11	8.29	8.16
209990_s_at	gamma-aminobut	GABBR2	3.54	3.63	3.55
216181_at	Synaptojanin 2	SYNJ2	7.14	7.00	6.63
203814_s_at	NAD(P)H dehydro	NQO2	9.40	9.41	9.43
217953_at	PHD finger protei	PHF3	3.85	4.08	3.93
207032_s_at	cysteine-rich secr	CRISP1	4.53	4.40	4.22
215820_x_at	sorting nexin 13	SNX13	5.76	5.85	5.83
214999_s_at	RAB11 family inte	RAB11FIP3	6.41	6.66	6.65
221168_at	PR domain contai	PRDM13	3.65	3.79	3.58
208939_at	selenophosphate	SEPHS1	9.86	9.94	10.05
205274_at	GTP binding prote	GTPBP1	5.64	5.61	5.28
218629_at	smoothened hom	SMO	7.24	7.12	7.21
210305_at	phosphodiesteras	PDE4DIP	4.30	3.98	3.82
206462_s_at	neurotrophic tyro	NTRK3	4.12	3.89	4.10
203688_at	polycystic kidney	PKD2	7.93	8.07	8.18
211886_s_at	T-box 5	TBX5	4.16	3.67	3.74
209560_s_at	delta-like 1 homo	DLK1	4.07	4.07	4.27
213171_s_at	matrix metallope	MMP24	7.12	6.93	6.88
209473_at	ectonucleoside tri	ENTPD1	6.60	6.35	6.15
221903_s_at	cylindromatosis (t	CYLD	7.95	7.92	8.22
202344_at	heat shock transc	HSF1	8.41	8.26	8.23
200723_s_at	cell cycle associat	CAPRIN1	11.80	11.86	11.89
35265_at	fragile X mental re	FXR2	8.52	8.38	8.45
216179_x_at	---	---	4.79	4.72	4.52
208662_s_at	tetratricopeptide	TTC3	10.68	10.77	10.86
219212_at	heat shock 70kDa	HSPA14	9.69	9.84	9.78
209933_s_at	CD300a molecule	CD300A	4.71	4.67	4.31
206414_s_at	ArfGAP with SH3	ASAP2	10.45	10.46	10.57
206835_at	statherin	STATH	3.47	3.45	3.34
214638_s_at	cyclin T2	CCNT2	4.69	4.62	4.82

213916_at	zinc finger proteir	ZNF20 /// ZNF625	4.71	4.97	4.83
220731_s_at	NECAP endocytos	NECAP2	8.78	8.82	9.07
221300_at	chromosome 15 c	C15orf2	4.79	5.22	5.08

159 SH3GL2	159 SH3GL2	159 SH3GL2	**SH3GL2 Mean (Log2)**	**Fold Change (Geometric)**	**p-value (α-level: 0.001)**	**q-value (FDR: 5%)**
8.65	9.00	8.78	**8.81**	**7.5**	3.62E-05	2.10E-02
8.34	8.54	8.70	**8.53**	**5.6**	1.07E-04	3.31E-02
8.21	8.41	8.23	**8.28**	**4.9**	7.43E-05	2.89E-02
8.54	8.66	8.71	**8.64**	**4.8**	2.55E-05	1.89E-02
8.67	8.65	8.80	**8.71**	**4.1**	7.99E-06	1.09E-02
8.41	8.50	8.33	**8.41**	**3.7**	6.54E-05	2.74E-02
8.43	8.56	8.57	**8.52**	**3.4**	2.13E-05	1.83E-02
8.11	8.10	8.04	**8.08**	**3.2**	1.34E-06	7.30E-03
6.85	7.18	7.00	**7.01**	**3.2**	4.15E-04	4.78E-02
7.52	7.51	7.88	**7.64**	**3.0**	2.44E-04	4.19E-02
7.96	8.04	8.15	**8.05**	**2.5**	2.67E-05	1.90E-02
8.36	8.55	8.43	**8.45**	**2.3**	1.01E-04	3.27E-02
7.57	7.54	7.60	**7.57**	**2.0**	5.21E-06	1.05E-02
7.53	7.63	7.68	**7.61**	**2.0**	3.65E-04	4.53E-02
12.50	12.67	12.47	**12.55**	**2.0**	1.56E-04	3.70E-02
5.88	6.01	6.06	**5.98**	**1.8**	8.06E-05	3.06E-02
8.47	8.43	8.44	**8.45**	**1.8**	6.28E-07	7.30E-03
8.71	8.86	8.82	**8.80**	**1.8**	3.40E-04	4.53E-02
7.36	7.41	7.50	**7.42**	**1.7**	3.77E-04	4.53E-02
9.00	8.97	9.00	**8.99**	**1.7**	3.01E-04	4.40E-02
7.96	8.05	7.92	**7.97**	**1.7**	2.23E-04	4.12E-02
7.98	8.06	7.98	**8.01**	**1.6**	3.82E-04	4.53E-02
9.60	9.50	9.60	**9.57**	**1.6**	5.59E-05	2.56E-02
8.53	8.72	8.61	**8.62**	**1.6**	3.10E-04	4.49E-02
10.98	10.97	11.04	**11.00**	**1.6**	1.55E-04	3.70E-02
8.55	8.63	8.70	**8.63**	**1.6**	2.53E-04	4.23E-02
9.98	10.06	10.13	**10.06**	**1.6**	1.17E-04	3.37E-02
9.34	9.38	9.35	**9.36**	**1.6**	1.36E-05	1.39E-02
8.22	8.25	8.23	**8.23**	**1.6**	1.17E-05	1.39E-02
7.47	7.45	7.40	**7.44**	**1.5**	2.51E-05	1.89E-02
10.84	10.97	10.91	**10.91**	**1.5**	1.24E-04	3.49E-02
10.66	10.77	10.72	**10.71**	**1.5**	3.79E-04	4.53E-02
10.12	10.13	10.14	**10.13**	**1.4**	5.58E-06	1.05E-02
7.84	7.86	7.87	**7.86**	**1.4**	2.62E-04	4.23E-02
10.45	10.43	10.48	**10.46**	**1.4**	1.26E-05	1.39E-02
11.88	12.00	11.98	**11.95**	**1.4**	3.70E-04	4.53E-02
7.34	7.40	7.38	**7.37**	**1.4**	9.64E-05	3.22E-02
9.85	9.95	9.96	**9.92**	**1.4**	2.73E-04	4.23E-02
7.65	7.62	7.72	**7.66**	**1.3**	2.04E-04	4.02E-02
13.30	13.25	13.30	**13.28**	**1.3**	2.19E-04	4.11E-02
10.36	10.41	10.37	**10.38**	**1.3**	4.37E-04	4.97E-02
7.43	7.50	7.45	**7.46**	**1.3**	9.21E-05	3.20E-02
12.54	12.49	12.54	**12.52**	**1.3**	3.38E-05	2.08E-02
11.93	11.94	11.97	**11.95**	**1.2**	1.30E-05	1.39E-02

9.46	9.51	9.48	**9.49**	1.2	5.73E-05	2.56E-02
9.32	9.31	9.30	**9.31**	1.2	1.42E-04	3.70E-02
7.02	7.06	7.05	**7.04**	1.2	2.49E-04	4.23E-02
6.14	6.13	6.15	**6.14**	1.2	3.93E-05	2.14E-02
9.60	9.61	9.57	**9.59**	1.2	4.36E-04	4.97E-02
5.63	5.65	5.65	**5.64**	1.2	1.65E-04	3.77E-02
4.32	4.34	4.38	**4.35**	1.2	2.62E-04	4.23E-02
7.01	7.02	6.97	**7.00**	1.2	1.76E-04	3.77E-02
8.54	8.55	8.58	**8.56**	1.2	2.92E-04	4.38E-02
5.57	5.57	5.60	**5.58**	1.2	4.69E-05	2.47E-02
5.24	5.25	5.27	**5.25**	1.1	1.56E-04	3.70E-02
14.62	14.60	14.63	**14.62**	1.1	2.41E-04	4.19E-02
9.36	9.38	9.37	**9.37**	1.1	3.59E-04	4.53E-02
14.91	14.93	14.94	**14.93**	1.1	1.82E-04	3.77E-02
14.87	14.87	14.89	**14.88**	1.0	2.39E-04	4.19E-02
3.59	3.60	3.60	**3.60**	-1.1	3.46E-04	4.53E-02
9.59	9.61	9.59	**9.60**	-1.1	1.40E-04	3.70E-02
11.83	11.81	11.84	**11.83**	-1.1	1.46E-04	3.70E-02
8.20	8.18	8.20	**8.20**	-1.1	2.01E-04	4.02E-02
4.28	4.27	4.28	**4.28**	-1.1	3.76E-04	4.53E-02
7.43	7.45	7.43	**7.43**	-1.1	2.14E-04	4.11E-02
6.08	6.10	6.11	**6.10**	-1.2	1.06E-04	3.31E-02
11.17	11.18	11.22	**11.19**	-1.2	2.10E-04	4.09E-02
11.34	11.39	11.36	**11.36**	-1.2	2.64E-04	4.23E-02
6.38	6.36	6.39	**6.38**	-1.2	3.02E-05	2.06E-02
8.11	8.13	8.11	**8.12**	-1.2	1.78E-04	3.77E-02
10.01	9.96	10.01	**9.99**	-1.2	2.31E-04	4.14E-02
3.90	3.88	3.88	**3.88**	-1.2	3.39E-04	4.53E-02
5.97	5.90	5.94	**5.94**	-1.2	1.61E-04	3.76E-02
9.02	9.10	9.04	**9.05**	-1.2	3.64E-04	4.53E-02
11.58	11.58	11.51	**11.55**	-1.2	4.05E-04	4.73E-02
8.96	9.00	8.99	**8.98**	-1.2	7.36E-05	2.89E-02
10.84	10.87	10.78	**10.83**	-1.3	3.17E-04	4.53E-02
5.54	5.58	5.54	**5.55**	-1.3	1.45E-04	3.70E-02
8.63	8.59	8.61	**8.61**	-1.3	7.54E-06	1.09E-02
8.19	8.17	8.27	**8.21**	-1.3	2.98E-04	4.39E-02
7.96	7.89	7.96	**7.94**	-1.3	3.94E-04	4.64E-02
5.92	5.86	5.87	**5.89**	-1.3	1.74E-04	3.77E-02
7.08	7.12	7.05	**7.08**	-1.3	1.70E-04	3.77E-02
12.44	12.48	12.52	**12.48**	-1.3	6.49E-05	2.74E-02
8.18	8.27	8.26	**8.24**	-1.3	2.03E-04	4.02E-02
9.46	9.50	9.48	**9.48**	-1.3	1.02E-04	3.27E-02
9.13	9.01	9.07	**9.07**	-1.3	3.39E-04	4.53E-02
6.38	6.29	6.31	**6.33**	-1.3	3.58E-04	4.53E-02
6.88	6.92	6.92	**6.91**	-1.4	3.82E-04	4.53E-02
8.57	8.66	8.67	**8.63**	-1.4	1.96E-04	4.00E-02
8.92	8.96	9.00	**8.96**	-1.4	3.30E-04	4.53E-02

9.45	9.50	9.46	**9.47**	-1.4	3.73E-05	2.10E-02
9.00	9.03	9.03	**9.02**	-1.4	2.24E-04	4.12E-02
11.11	11.17	11.16	**11.14**	-1.4	1.17E-04	3.37E-02
11.38	11.30	11.38	**11.35**	-1.4	3.74E-04	4.53E-02
8.94	8.90	9.00	**8.94**	-1.4	1.33E-04	3.68E-02
8.19	8.15	8.23	**8.19**	-1.4	1.72E-04	3.77E-02
4.49	4.46	4.36	**4.44**	-1.4	2.61E-04	4.23E-02
8.37	8.36	8.42	**8.38**	-1.4	1.77E-05	1.61E-02
6.95	6.99	6.99	**6.98**	-1.5	2.64E-04	4.23E-02
9.21	9.22	9.17	**9.20**	-1.5	8.57E-05	3.06E-02
7.33	7.23	7.31	**7.29**	-1.5	3.64E-04	4.53E-02
8.36	8.30	8.38	**8.35**	-1.5	1.53E-04	3.70E-02
10.71	10.77	10.69	**10.72**	-1.5	2.74E-04	4.23E-02
7.35	7.39	7.47	**7.40**	-1.5	3.54E-04	4.53E-02
7.36	7.48	7.51	**7.45**	-1.5	3.80E-04	4.53E-02
3.40	3.30	3.39	**3.36**	-1.5	1.80E-04	3.77E-02
9.69	9.66	9.54	**9.63**	-1.5	3.54E-04	4.53E-02
6.42	6.38	6.30	**6.37**	-1.5	2.70E-04	4.23E-02
6.80	6.75	6.83	**6.79**	-1.5	3.43E-05	2.08E-02
7.26	7.31	7.21	**7.26**	-1.5	1.17E-04	3.37E-02
9.20	9.30	9.26	**9.26**	-1.6	3.34E-04	4.53E-02
8.03	7.98	8.14	**8.05**	-1.6	1.55E-04	3.70E-02
10.01	9.99	9.97	**9.99**	-1.6	1.51E-05	1.46E-02
8.99	9.06	9.05	**9.03**	-1.6	5.14E-05	2.56E-02
8.81	8.83	8.80	**8.81**	-1.7	2.29E-04	4.14E-02
6.02	6.12	6.19	**6.11**	-1.7	2.79E-04	4.24E-02
6.64	6.76	6.77	**6.72**	-1.7	7.29E-05	2.89E-02
6.31	6.39	6.52	**6.40**	-1.7	3.82E-04	4.53E-02
7.60	7.66	7.76	**7.67**	-1.8	4.09E-04	4.75E-02
6.94	6.99	6.95	**6.96**	-1.9	1.18E-06	7.30E-03
8.00	8.26	8.10	**8.12**	-1.9	3.76E-04	4.53E-02
8.90	8.80	8.93	**8.87**	-2.0	5.60E-05	2.56E-02
5.07	4.90	4.94	**4.97**	-2.0	2.97E-04	4.39E-02
9.82	10.00	9.92	**9.91**	-2.0	3.68E-04	4.53E-02
10.99	10.95	10.93	**10.96**	-2.0	2.44E-05	1.89E-02
6.96	6.98	6.95	**6.96**	-2.0	1.78E-04	3.77E-02
8.82	8.90	8.95	**8.89**	-2.0	5.80E-05	2.56E-02
5.98	6.02	6.01	**6.00**	-2.1	1.11E-04	3.35E-02
8.75	8.75	8.79	**8.76**	-2.1	1.98E-06	8.10E-03
7.36	7.42	7.30	**7.36**	-2.1	5.52E-05	2.56E-02
5.70	5.66	5.90	**5.76**	-2.2	2.72E-04	4.23E-02
7.58	7.50	7.43	**7.50**	-2.3	8.23E-05	3.06E-02
9.88	9.83	9.91	**9.87**	-2.3	5.71E-06	1.05E-02
5.34	5.17	5.49	**5.33**	-2.3	2.80E-04	4.24E-02
7.33	7.62	7.33	**7.42**	-2.4	2.17E-04	4.11E-02
5.09	4.93	5.06	**5.03**	-2.4	2.38E-04	4.19E-02
8.82	8.99	9.08	**8.96**	-2.5	1.41E-04	3.70E-02

6.59	6.43	6.42	**6.48**	-3.2	8.61E-05	3.06E-02
4.07	4.01	4.07	**4.05**	-3.4	3.34E-05	2.08E-02
6.71	7.04	6.62	**6.79**	-4.2	9.55E-05	3.22E-02
7.83	8.07	8.00	**7.97**	-7.5	4.65E-06	1.05E-02
9.02	9.36	9.14	**9.17**	-11.0	5.78E-06	1.05E-02
7.66	8.05	7.84	**7.85**	-12.1	6.71E-06	1.09E-02
3.59	3.61	3.58			4.48E-04	5.05E-02
4.26	4.11	4.11			4.57E-04	5.12E-02
9.01	9.23	9.09			4.60E-04	5.12E-02
7.70	7.90	8.10			4.72E-04	5.22E-02
9.95	9.98	9.95			4.75E-04	5.22E-02
11.55	11.59	11.64			4.96E-04	5.35E-02
7.07	7.20	7.10			4.97E-04	5.35E-02
5.84	5.79	5.78			4.97E-04	5.35E-02
10.79	10.91	10.81			5.05E-04	5.40E-02
8.29	8.40	8.35			5.25E-04	5.57E-02
3.60	3.60	3.58			5.35E-04	5.59E-02
10.94	10.97	10.98			5.36E-04	5.59E-02
9.53	9.53	9.55			5.37E-04	5.59E-02
11.68	11.61	11.69			5.57E-04	5.75E-02
10.46	10.41	10.52			5.59E-04	5.75E-02
9.90	9.98	9.99			5.68E-04	5.79E-02
6.61	6.55	6.39			5.75E-04	5.79E-02
9.03	8.98	9.01			5.75E-04	5.79E-02
6.58	6.47	6.75			5.77E-04	5.79E-02
8.18	8.22	8.16			5.81E-04	5.80E-02
12.05	12.09	12.10			5.98E-04	5.92E-02
12.85	12.95	12.92			6.04E-04	5.92E-02
6.80	6.88	7.01			6.05E-04	5.92E-02
8.85	8.96	8.93			6.14E-04	5.94E-02
9.89	10.09	9.92			6.18E-04	5.94E-02
7.89	8.01	7.97			6.19E-04	5.94E-02
3.89	4.00	3.98			6.23E-04	5.94E-02
10.12	10.15	10.18			6.26E-04	5.94E-02
8.31	8.48	8.66			6.28E-04	5.94E-02
4.23	4.80	4.45			6.48E-04	6.03E-02
4.05	4.10	4.11			6.49E-04	6.03E-02
10.05	10.02	10.12			6.50E-04	6.03E-02
7.56	7.69	7.61			6.52E-04	6.03E-02
8.00	7.93	8.11			6.63E-04	6.06E-02
9.64	9.58	9.64			6.69E-04	6.06E-02
7.58	7.60	7.64			6.73E-04	6.06E-02
12.82	12.85	12.84			6.79E-04	6.06E-02
7.60	7.57	7.63			6.80E-04	6.06E-02
5.37	5.38	5.38			6.83E-04	6.06E-02
9.18	9.03	9.04			6.85E-04	6.06E-02

9.37	9.50	9.49	6.92E-04	6.06E-02
12.01	11.96	11.99	6.92E-04	6.06E-02
8.20	8.11	8.11	6.93E-04	6.06E-02
13.29	13.59	13.36	7.09E-04	6.17E-02
8.74	8.86	8.85	7.17E-04	6.21E-02
8.69	8.74	8.79	7.23E-04	6.22E-02
8.38	8.38	8.49	7.31E-04	6.26E-02
12.55	12.84	12.60	7.37E-04	6.28E-02
7.10	7.35	7.34	7.54E-04	6.33E-02
6.86	7.12	6.80	7.54E-04	6.33E-02
7.73	7.78	7.80	7.55E-04	6.33E-02
5.46	5.45	5.43	7.61E-04	6.35E-02
8.44	8.48	8.59	7.68E-04	6.37E-02
11.24	11.29	11.30	7.75E-04	6.38E-02
11.50	11.91	11.53	7.76E-04	6.38E-02
7.75	7.74	7.85	7.82E-04	6.40E-02
6.07	6.09	6.12	7.94E-04	6.44E-02
6.76	7.05	7.14	7.98E-04	6.44E-02
3.44	3.49	3.68	8.02E-04	6.44E-02
3.51	3.52	3.52	8.05E-04	6.44E-02
9.60	9.77	9.75	8.18E-04	6.44E-02
9.89	9.94	9.96	8.19E-04	6.44E-02
10.23	10.18	10.17	8.19E-04	6.44E-02
8.01	8.11	8.21	8.19E-04	6.44E-02
9.00	9.00	9.01	8.30E-04	6.50E-02
8.13	8.31	8.39	8.36E-04	6.51E-02
12.94	13.16	13.02	8.46E-04	6.52E-02
8.32	8.41	8.55	8.48E-04	6.52E-02
9.04	8.91	8.93	8.49E-04	6.52E-02
9.58	9.66	9.72	8.54E-04	6.52E-02
9.13	9.22	9.25	8.78E-04	6.68E-02
10.75	10.73	10.85	8.85E-04	6.68E-02
11.47	11.46	11.46	8.97E-04	6.68E-02
8.05	8.26	8.09	9.01E-04	6.68E-02
4.88	4.83	4.60	9.04E-04	6.68E-02
8.84	8.81	8.87	9.05E-04	6.68E-02
9.66	9.83	9.89	9.15E-04	6.68E-02
9.15	9.16	9.25	9.15E-04	6.68E-02
8.24	8.29	8.24	9.17E-04	6.68E-02
14.73	14.74	14.76	9.18E-04	6.68E-02
6.43	6.47	6.51	9.20E-04	6.68E-02
8.68	8.73	8.76	9.26E-04	6.68E-02
8.35	8.37	8.33	9.34E-04	6.68E-02
6.26	6.41	6.35	9.42E-04	6.68E-02
4.50	4.45	4.54	9.42E-04	6.68E-02
6.00	6.26	6.00	9.44E-04	6.68E-02
7.22	7.28	7.21	9.44E-04	6.68E-02

4.94	4.88	4.84	9.51E-04	6.68E-02
12.24	12.23	12.25	9.51E-04	6.68E-02
11.65	11.63	11.62	9.55E-04	6.68E-02
9.18	9.12	9.17	9.60E-04	6.68E-02
6.50	6.51	6.56	9.76E-04	6.74E-02
9.58	9.59	9.62	9.85E-04	6.74E-02
8.16	8.01	8.29	9.97E-04	6.74E-02
10.40	10.34	10.43	1.00E-03	6.74E-02
5.13	5.13	5.09	1.00E-03	6.74E-02
9.57	9.63	9.57	1.01E-03	6.74E-02
11.37	11.45	11.42	1.01E-03	6.74E-02
3.38	3.36	3.47	1.01E-03	6.74E-02
11.30	11.38	11.33	1.01E-03	6.74E-02
10.22	10.09	10.16	1.02E-03	6.74E-02
13.29	13.64	13.41	1.03E-03	6.74E-02
8.64	8.71	8.66	1.03E-03	6.74E-02
8.68	8.70	8.68	1.03E-03	6.74E-02
10.55	10.49	10.44	1.04E-03	6.74E-02
10.08	10.06	10.08	1.04E-03	6.74E-02
6.81	6.85	6.92	1.04E-03	6.74E-02
7.70	7.57	7.57	1.05E-03	6.74E-02
6.30	6.39	6.31	1.05E-03	6.74E-02
9.85	9.85	9.91	1.05E-03	6.74E-02
9.37	9.41	9.51	1.05E-03	6.74E-02
7.17	7.23	7.18	1.06E-03	6.74E-02
7.39	7.63	7.47	1.07E-03	6.74E-02
7.22	7.48	7.37	1.07E-03	6.74E-02
12.69	12.73	12.70	1.07E-03	6.74E-02
9.10	9.11	9.22	1.07E-03	6.74E-02
7.31	7.52	7.56	1.08E-03	6.75E-02
7.64	7.70	7.68	1.08E-03	6.77E-02
11.94	12.26	12.05	1.09E-03	6.78E-02
8.38	8.37	8.43	1.10E-03	6.82E-02
4.17	4.16	4.18	1.10E-03	6.82E-02
6.71	6.81	6.78	1.11E-03	6.82E-02
9.53	9.36	9.65	1.11E-03	6.82E-02
10.23	10.39	10.40	1.12E-03	6.82E-02
6.17	6.18	6.16	1.12E-03	6.82E-02
9.60	9.57	9.66	1.13E-03	6.82E-02
10.42	10.45	10.39	1.13E-03	6.83E-02
6.91	7.28	7.14	1.14E-03	6.83E-02
10.16	10.15	10.26	1.15E-03	6.92E-02
9.81	10.07	9.83	1.17E-03	6.94E-02
8.85	8.90	8.90	1.17E-03	6.94E-02
6.61	6.68	6.82	1.17E-03	6.94E-02
7.57	7.46	7.51	1.18E-03	6.94E-02
4.70	4.92	4.80	1.19E-03	6.94E-02

10.29	10.29	10.27	1.19E-03	6.94E-02
10.87	10.93	10.86	1.20E-03	6.94E-02
3.83	3.76	3.82	1.20E-03	6.94E-02
6.08	6.21	6.15	1.21E-03	6.94E-02
7.32	7.39	7.39	1.21E-03	6.94E-02
9.17	9.34	9.25	1.21E-03	6.94E-02
12.93	12.93	12.90	1.21E-03	6.94E-02
11.37	11.48	11.45	1.21E-03	6.94E-02
2.98	3.04	3.25	1.22E-03	6.95E-02
6.41	6.41	6.47	1.22E-03	6.95E-02
13.52	13.51	13.46	1.23E-03	6.95E-02
11.38	11.44	11.41	1.23E-03	6.96E-02
3.80	4.19	3.95	1.24E-03	6.97E-02
7.29	7.30	7.21	1.26E-03	7.02E-02
8.80	8.67	8.76	1.26E-03	7.02E-02
7.21	7.48	7.30	1.26E-03	7.02E-02
10.20	10.19	10.21	1.27E-03	7.02E-02
10.58	10.66	10.62	1.27E-03	7.03E-02
10.24	10.22	10.18	1.28E-03	7.05E-02
9.25	9.30	9.32	1.29E-03	7.07E-02
8.51	8.37	8.49	1.30E-03	7.09E-02
6.80	6.72	6.80	1.31E-03	7.09E-02
7.70	7.51	7.50	1.31E-03	7.09E-02
10.20	10.44	10.24	1.31E-03	7.09E-02
8.76	8.75	8.77	1.32E-03	7.09E-02
8.51	8.68	8.54	1.33E-03	7.09E-02
11.19	11.22	11.20	1.33E-03	7.09E-02
9.37	9.35	9.40	1.33E-03	7.09E-02
6.25	6.19	6.01	1.33E-03	7.09E-02
10.47	10.58	10.49	1.34E-03	7.09E-02
4.87	4.96	4.89	1.34E-03	7.09E-02
13.45	13.45	13.52	1.35E-03	7.09E-02
3.88	4.13	4.03	1.36E-03	7.09E-02
8.90	8.90	8.91	1.37E-03	7.09E-02
11.59	11.31	11.36	1.37E-03	7.09E-02
8.51	8.57	8.50	1.37E-03	7.09E-02
8.64	8.21	8.24	1.37E-03	7.09E-02
8.82	8.83	8.78	1.37E-03	7.09E-02
8.57	8.39	8.34	1.37E-03	7.09E-02
3.33	3.37	3.39	1.39E-03	7.14E-02
3.94	4.51	4.15	1.39E-03	7.15E-02
7.95	7.89	7.84	1.40E-03	7.16E-02
9.37	9.39	9.44	1.41E-03	7.20E-02
6.67	7.06	6.76	1.44E-03	7.30E-02
9.06	8.81	9.02	1.45E-03	7.33E-02
8.23	8.37	8.39	1.45E-03	7.33E-02
9.13	9.14	9.13	1.47E-03	7.40E-02

9.48	9.30	9.44	1.47E-03	7.40E-02
7.32	7.66	7.51	1.49E-03	7.42E-02
9.21	9.18	9.36	1.49E-03	7.42E-02
7.91	7.91	7.98	1.50E-03	7.42E-02
11.13	11.19	11.10	1.50E-03	7.42E-02
7.86	7.91	8.00	1.53E-03	7.57E-02
7.09	7.06	7.01	1.55E-03	7.62E-02
5.89	5.91	5.81	1.56E-03	7.68E-02
9.55	9.49	9.56	1.58E-03	7.76E-02
10.91	10.93	10.96	1.59E-03	7.76E-02
8.97	8.94	9.00	1.60E-03	7.79E-02
9.15	9.12	9.08	1.62E-03	7.87E-02
4.12	3.96	3.90	1.63E-03	7.87E-02
4.86	4.89	4.79	1.63E-03	7.87E-02
10.35	10.38	10.38	1.65E-03	7.92E-02
4.85	4.90	4.90	1.65E-03	7.94E-02
11.82	11.79	11.81	1.66E-03	7.95E-02
8.02	7.92	7.97	1.67E-03	7.97E-02
7.94	7.88	7.90	1.68E-03	7.97E-02
9.31	9.45	9.49	1.68E-03	7.97E-02
6.82	6.95	6.82	1.69E-03	7.98E-02
7.90	7.74	7.89	1.70E-03	7.98E-02
7.55	7.54	7.48	1.70E-03	7.98E-02
9.20	9.23	9.31	1.71E-03	7.98E-02
7.08	6.99	7.02	1.71E-03	7.98E-02
8.50	8.55	8.59	1.71E-03	7.98E-02
8.33	8.32	8.45	1.73E-03	7.98E-02
7.24	7.15	7.39	1.73E-03	7.98E-02
8.79	8.90	8.83	1.74E-03	7.98E-02
8.65	8.97	8.97	1.74E-03	7.98E-02
5.26	5.46	5.54	1.74E-03	7.98E-02
4.60	4.52	4.48	1.75E-03	7.99E-02
10.38	10.27	10.43	1.75E-03	7.99E-02
8.47	8.50	8.49	1.77E-03	8.07E-02
6.89	7.04	6.88	1.78E-03	8.07E-02
8.05	7.99	8.09	1.78E-03	8.08E-02
9.05	9.13	9.12	1.79E-03	8.08E-02
12.63	12.63	12.68	1.79E-03	8.09E-02
9.70	9.71	9.64	1.80E-03	8.09E-02
4.10	4.07	4.05	1.81E-03	8.11E-02
8.41	8.10	8.22	1.83E-03	8.17E-02
11.59	11.63	11.62	1.88E-03	8.31E-02
5.13	5.13	5.11	1.88E-03	8.31E-02
11.40	11.43	11.42	1.88E-03	8.31E-02
9.05	9.02	9.13	1.88E-03	8.31E-02
8.30	8.32	8.36	1.92E-03	8.46E-02
5.62	5.76	5.52	1.93E-03	8.47E-02

11.80	11.91	11.85	1.93E-03	8.47E-02
11.03	10.73	11.20	1.94E-03	8.50E-02
8.81	8.74	8.92	1.97E-03	8.56E-02
9.21	9.13	9.20	1.97E-03	8.56E-02
6.98	6.98	7.14	1.97E-03	8.56E-02
9.99	9.91	10.03	1.99E-03	8.61E-02
3.94	3.91	3.88	1.99E-03	8.61E-02
9.03	9.27	9.28	2.01E-03	8.64E-02
10.95	10.82	10.96	2.02E-03	8.64E-02
5.62	5.76	5.97	2.02E-03	8.64E-02
7.04	7.07	7.06	2.03E-03	8.64E-02
12.11	12.06	12.18	2.05E-03	8.64E-02
5.89	6.20	6.38	2.05E-03	8.64E-02
6.27	6.35	6.40	2.05E-03	8.64E-02
7.81	7.91	7.92	2.05E-03	8.64E-02
8.59	8.56	8.53	2.05E-03	8.64E-02
7.41	7.36	7.27	2.05E-03	8.64E-02
7.40	7.32	7.43	2.06E-03	8.64E-02
5.49	5.76	5.60	2.06E-03	8.64E-02
8.57	8.67	8.68	2.07E-03	8.65E-02
5.45	5.53	5.51	2.08E-03	8.65E-02
10.89	10.95	10.91	2.09E-03	8.65E-02
6.73	6.84	6.78	2.09E-03	8.65E-02
8.26	8.21	8.19	2.10E-03	8.65E-02
12.16	12.46	12.27	2.10E-03	8.65E-02
10.72	10.77	10.76	2.11E-03	8.65E-02
11.09	11.08	11.08	2.11E-03	8.65E-02
10.50	10.54	10.49	2.12E-03	8.65E-02
9.13	8.66	9.27	2.12E-03	8.65E-02
8.84	8.49	8.75	2.14E-03	8.67E-02
6.41	6.40	6.49	2.14E-03	8.67E-02
8.42	8.25	8.38	2.15E-03	8.67E-02
9.07	9.16	9.06	2.15E-03	8.67E-02
11.37	11.22	11.38	2.16E-03	8.67E-02
4.63	4.72	4.71	2.16E-03	8.67E-02
4.29	4.22	4.52	2.16E-03	8.67E-02
9.89	9.89	9.86	2.17E-03	8.68E-02
9.18	9.23	9.25	2.18E-03	8.69E-02
7.29	7.38	7.41	2.21E-03	8.80E-02
8.96	9.15	9.19	2.22E-03	8.80E-02
5.09	5.05	5.11	2.22E-03	8.80E-02
9.23	9.41	9.35	2.23E-03	8.80E-02
7.27	7.33	7.49	2.24E-03	8.80E-02
6.79	7.15	6.92	2.24E-03	8.80E-02
6.23	6.12	6.14	2.25E-03	8.80E-02
14.57	14.60	14.56	2.25E-03	8.80E-02
6.43	6.47	6.47	2.25E-03	8.80E-02

4.88	4.82	4.90	2.26E-03	8.80E-02
9.82	9.83	9.75	2.26E-03	8.80E-02
8.96	9.08	8.96	2.27E-03	8.80E-02
5.26	5.23	5.38	2.28E-03	8.80E-02
7.10	7.14	7.40	2.30E-03	8.85E-02
8.03	8.00	8.07	2.30E-03	8.85E-02
6.58	6.69	6.73	2.31E-03	8.85E-02
9.38	9.42	9.60	2.32E-03	8.90E-02
4.31	4.19	4.24	2.33E-03	8.90E-02
7.49	7.52	7.55	2.33E-03	8.90E-02
14.29	14.28	14.31	2.35E-03	8.93E-02
6.43	6.35	6.56	2.35E-03	8.93E-02
10.54	10.68	10.63	2.36E-03	8.95E-02
9.44	9.43	9.43	2.37E-03	8.95E-02
5.33	5.27	5.40	2.39E-03	9.02E-02
3.24	3.24	3.27	2.41E-03	9.05E-02
6.89	7.42	6.86	2.42E-03	9.08E-02
10.99	11.04	11.00	2.43E-03	9.08E-02
4.76	4.70	4.84	2.44E-03	9.08E-02
9.11	9.16	9.17	2.44E-03	9.08E-02
11.45	11.38	11.48	2.45E-03	9.11E-02
10.22	10.28	10.21	2.47E-03	9.11E-02
11.60	11.60	11.60	2.48E-03	9.11E-02
10.75	10.77	10.84	2.48E-03	9.11E-02
8.99	8.99	9.13	2.48E-03	9.11E-02
13.92	13.91	13.93	2.48E-03	9.11E-02
9.68	9.72	9.70	2.54E-03	9.28E-02
3.57	3.60	3.56	2.54E-03	9.28E-02
7.79	7.88	7.98	2.55E-03	9.28E-02
7.69	7.49	7.93	2.55E-03	9.28E-02
6.23	6.28	6.27	2.55E-03	9.28E-02
4.72	5.12	5.11	2.59E-03	9.34E-02
10.08	10.05	10.07	2.60E-03	9.34E-02
9.08	9.19	9.11	2.61E-03	9.34E-02
14.25	14.28	14.29	2.61E-03	9.34E-02
12.79	12.73	12.77	2.61E-03	9.34E-02
4.58	4.61	4.29	2.61E-03	9.34E-02
7.27	7.28	7.38	2.62E-03	9.34E-02
9.40	9.37	9.40	2.62E-03	9.34E-02
4.76	4.92	4.84	2.63E-03	9.34E-02
8.19	8.14	8.25	2.63E-03	9.34E-02
6.93	6.82	6.96	2.64E-03	9.36E-02
11.94	11.86	11.94	2.65E-03	9.39E-02
7.36	7.37	7.30	2.66E-03	9.40E-02
7.69	7.89	8.03	2.67E-03	9.40E-02
8.91	8.74	8.94	2.67E-03	9.40E-02
11.60	11.64	11.70	2.68E-03	9.42E-02

3.48	3.45	3.53	2.70E-03	9.44E-02
10.86	11.18	10.90	2.72E-03	9.52E-02
8.13	8.23	8.30	2.73E-03	9.54E-02
8.29	8.16	8.34	2.76E-03	9.61E-02
8.84	8.82	8.75	2.77E-03	9.61E-02
8.24	8.39	8.35	2.78E-03	9.62E-02
6.63	6.63	6.66	2.79E-03	9.65E-02
10.14	9.66	10.32	2.80E-03	9.65E-02
6.72	6.73	6.72	2.80E-03	9.65E-02
5.41	5.53	5.44	2.83E-03	9.73E-02
6.90	6.97	7.22	2.84E-03	9.73E-02
6.87	6.90	6.87	2.85E-03	9.76E-02
8.32	8.69	8.71	2.87E-03	9.79E-02
9.60	9.63	9.69	2.88E-03	9.81E-02
10.22	10.01	10.10	2.89E-03	9.82E-02
3.58	3.55	3.59	2.90E-03	9.86E-02
6.75	6.79	6.66	2.94E-03	9.93E-02
11.98	12.05	12.09	2.94E-03	9.93E-02
5.28	5.30	5.32	2.94E-03	9.93E-02
7.47	7.66	7.67	2.96E-03	9.95E-02
7.00	6.95	6.98	2.97E-03	9.95E-02
7.19	7.22	7.42	2.97E-03	9.95E-02
9.27	9.31	9.15	2.98E-03	9.95E-02
10.02	9.96	10.01	2.99E-03	9.96E-02
6.72	7.05	7.07	2.99E-03	9.96E-02
8.77	8.74	8.68	3.00E-03	9.96E-02
5.82	5.97	5.99	3.02E-03	9.97E-02
8.70	8.70	8.80	3.02E-03	9.97E-02
6.05	6.03	5.97	3.02E-03	9.97E-02
7.66	7.69	7.60	3.03E-03	9.97E-02
9.22	9.10	9.19	3.03E-03	9.97E-02
7.23	7.07	7.19	3.04E-03	9.97E-02
8.73	8.74	8.78	3.06E-03	9.99E-02
9.52	9.61	9.61	3.06E-03	9.99E-02
9.44	9.54	9.58	3.06E-03	9.99E-02
9.18	9.19	9.21	3.06E-03	9.99E-02
7.59	7.64	7.45	3.07E-03	9.99E-02
6.52	6.72	6.67	3.08E-03	9.99E-02
11.36	11.47	11.47	3.08E-03	9.99E-02
8.41	8.53	8.60	3.09E-03	1.00E-01
9.58	9.60	9.62	3.11E-03	1.00E-01
9.51	9.63	9.55	3.11E-03	1.00E-01
9.23	9.19	9.19	3.13E-03	1.00E-01
3.26	3.30	3.25	3.13E-03	1.00E-01
8.34	8.56	8.46	3.13E-03	1.00E-01
4.69	4.81	4.71	3.15E-03	1.01E-01
6.57	6.50	6.77	3.17E-03	1.01E-01

6.30	6.30	6.37	3.17E-03	1.01E-01
7.58	7.55	7.54	3.18E-03	1.01E-01
5.42	5.72	5.83	3.19E-03	1.01E-01
5.31	5.34	5.25	3.19E-03	1.01E-01
7.55	7.48	7.43	3.24E-03	1.02E-01
7.07	7.00	7.02	3.26E-03	1.03E-01
9.65	9.68	9.57	3.27E-03	1.03E-01
10.22	10.06	10.39	3.28E-03	1.03E-01
7.22	7.13	7.27	3.29E-03	1.03E-01
4.53	4.61	4.61	3.29E-03	1.03E-01
9.34	9.30	9.17	3.29E-03	1.03E-01
6.13	5.71	6.11	3.30E-03	1.03E-01
9.72	9.90	9.72	3.30E-03	1.03E-01
10.17	10.26	10.31	3.31E-03	1.03E-01
9.63	9.70	9.66	3.32E-03	1.03E-01
7.03	7.27	7.22	3.34E-03	1.03E-01
5.85	5.70	5.64	3.37E-03	1.04E-01
5.39	5.43	5.41	3.37E-03	1.04E-01
5.64	5.37	5.49	3.38E-03	1.04E-01
6.13	6.18	6.32	3.39E-03	1.04E-01
5.89	5.94	5.86	3.41E-03	1.04E-01
6.90	6.99	7.17	3.41E-03	1.04E-01
8.43	8.41	8.51	3.43E-03	1.05E-01
10.11	10.36	10.19	3.44E-03	1.05E-01
5.71	5.66	5.64	3.45E-03	1.05E-01
9.59	9.58	9.64	3.47E-03	1.05E-01
9.99	10.11	10.05	3.49E-03	1.06E-01
10.41	10.41	10.22	3.51E-03	1.06E-01
9.27	9.39	9.31	3.52E-03	1.06E-01
8.82	8.80	8.84	3.53E-03	1.06E-01
7.75	7.95	7.82	3.54E-03	1.07E-01
8.70	8.62	8.68	3.55E-03	1.07E-01
4.22	4.20	4.26	3.56E-03	1.07E-01
10.19	10.09	10.22	3.57E-03	1.07E-01
11.11	11.11	11.07	3.60E-03	1.07E-01
8.17	8.20	8.16	3.62E-03	1.08E-01
7.96	8.14	8.08	3.62E-03	1.08E-01
9.38	9.35	9.34	3.63E-03	1.08E-01
6.40	6.38	6.40	3.65E-03	1.08E-01
9.75	9.61	9.72	3.66E-03	1.08E-01
6.04	6.00	5.94	3.67E-03	1.08E-01
5.77	5.65	5.95	3.67E-03	1.08E-01
5.34	5.28	5.26	3.67E-03	1.08E-01
10.02	9.92	10.02	3.67E-03	1.08E-01
9.23	9.32	9.32	3.67E-03	1.08E-01
6.65	6.89	6.99	3.68E-03	1.08E-01
9.48	9.50	9.47	3.70E-03	1.08E-01

11.10	11.09	11.06	3.72E-03	1.09E-01
13.75	13.85	13.81	3.74E-03	1.09E-01
11.89	11.94	11.87	3.75E-03	1.09E-01
5.32	5.22	5.55	3.75E-03	1.09E-01
12.45	12.45	12.42	3.76E-03	1.09E-01
8.48	8.37	8.42	3.77E-03	1.09E-01
8.55	8.49	8.49	3.80E-03	1.09E-01
6.50	6.42	6.38	3.85E-03	1.10E-01
4.24	4.16	4.06	3.85E-03	1.10E-01
14.50	14.53	14.51	3.86E-03	1.10E-01
7.94	8.01	8.15	3.86E-03	1.10E-01
6.09	6.11	6.16	3.86E-03	1.10E-01
10.16	9.95	10.13	3.86E-03	1.10E-01
4.14	4.17	4.14	3.87E-03	1.10E-01
6.38	6.27	6.37	3.88E-03	1.10E-01
11.81	11.80	11.82	3.89E-03	1.10E-01
7.83	7.89	7.88	3.95E-03	1.12E-01
10.07	10.10	10.05	3.95E-03	1.12E-01
8.61	8.68	8.60	3.97E-03	1.12E-01
3.69	3.71	3.70	3.97E-03	1.12E-01
9.21	9.29	9.21	3.97E-03	1.12E-01
8.84	8.93	8.78	3.97E-03	1.12E-01
6.91	6.86	7.03	3.99E-03	1.12E-01
6.59	6.48	6.62	4.00E-03	1.12E-01
6.00	5.92	5.85	4.01E-03	1.12E-01
8.03	7.99	8.06	4.02E-03	1.12E-01
9.52	9.81	9.68	4.03E-03	1.12E-01
8.90	8.62	9.02	4.08E-03	1.13E-01
9.99	10.09	10.00	4.08E-03	1.13E-01
10.78	10.81	10.90	4.09E-03	1.13E-01
7.11	7.23	7.45	4.12E-03	1.14E-01
10.11	10.06	10.14	4.13E-03	1.14E-01
9.54	9.73	9.53	4.15E-03	1.14E-01
5.79	5.85	5.83	4.16E-03	1.14E-01
8.30	8.36	8.26	4.17E-03	1.14E-01
9.97	10.00	10.11	4.18E-03	1.14E-01
12.95	12.98	12.93	4.18E-03	1.14E-01
9.36	9.37	9.53	4.19E-03	1.15E-01
10.97	10.96	10.98	4.21E-03	1.15E-01
9.07	9.11	9.01	4.22E-03	1.15E-01
9.34	9.02	9.23	4.22E-03	1.15E-01
7.72	7.91	7.78	4.23E-03	1.15E-01
9.14	9.17	9.12	4.24E-03	1.15E-01
9.34	9.13	9.19	4.28E-03	1.16E-01
7.06	7.33	7.17	4.30E-03	1.16E-01
9.87	9.89	9.92	4.34E-03	1.17E-01
9.73	9.83	9.79	4.35E-03	1.17E-01

8.53	8.72	8.53	4.36E-03	1.17E-01
5.73	5.57	5.69	4.37E-03	1.17E-01
5.26	5.38	5.21	4.37E-03	1.17E-01
6.76	6.75	6.78	4.37E-03	1.17E-01
6.67	6.65	6.64	4.42E-03	1.18E-01
8.10	8.15	8.23	4.42E-03	1.18E-01
4.05	3.83	3.83	4.42E-03	1.18E-01
7.95	7.82	8.07	4.43E-03	1.18E-01
7.40	7.33	7.36	4.45E-03	1.18E-01
8.16	8.44	7.98	4.49E-03	1.19E-01
6.12	6.02	6.08	4.49E-03	1.19E-01
9.05	8.99	9.12	4.50E-03	1.19E-01
10.03	10.12	10.17	4.52E-03	1.19E-01
6.83	6.70	6.92	4.54E-03	1.19E-01
9.48	9.20	9.54	4.55E-03	1.19E-01
8.71	8.52	8.47	4.55E-03	1.19E-01
8.33	8.32	8.33	4.55E-03	1.19E-01
8.86	8.85	8.81	4.60E-03	1.20E-01
6.33	6.51	6.40	4.60E-03	1.20E-01
5.93	5.82	5.92	4.63E-03	1.21E-01
9.86	9.76	9.73	4.66E-03	1.21E-01
10.01	10.05	10.10	4.66E-03	1.21E-01
6.56	6.29	6.83	4.67E-03	1.21E-01
11.57	11.69	11.65	4.68E-03	1.21E-01
5.64	5.64	5.63	4.68E-03	1.21E-01
8.20	8.11	8.26	4.69E-03	1.21E-01
9.55	9.64	9.64	4.71E-03	1.21E-01
13.03	13.05	13.07	4.71E-03	1.21E-01
10.51	10.46	10.51	4.74E-03	1.21E-01
6.65	6.76	6.65	4.74E-03	1.21E-01
7.69	7.73	7.77	4.74E-03	1.21E-01
5.82	5.76	5.78	4.74E-03	1.21E-01
9.71	9.94	9.80	4.75E-03	1.21E-01
6.49	6.43	6.33	4.75E-03	1.21E-01
10.09	10.05	10.06	4.76E-03	1.21E-01
7.93	7.82	7.81	4.77E-03	1.21E-01
5.17	5.20	5.16	4.80E-03	1.22E-01
8.78	8.93	8.89	4.81E-03	1.22E-01
7.25	7.56	7.34	4.82E-03	1.22E-01
6.70	6.82	6.84	4.82E-03	1.22E-01
8.87	8.69	8.83	4.83E-03	1.22E-01
3.88	3.56	3.69	4.84E-03	1.22E-01
7.79	7.88	7.93	4.85E-03	1.22E-01
7.47	7.47	7.53	4.86E-03	1.22E-01
9.57	9.70	9.61	4.87E-03	1.22E-01
3.50	3.50	3.57	4.88E-03	1.22E-01
11.94	11.71	11.96	4.89E-03	1.22E-01

8.94	8.99	9.02	4.90E-03	1.22E-01
5.24	5.21	5.37	4.93E-03	1.23E-01
9.81	9.94	9.91	4.94E-03	1.23E-01
8.01	8.05	8.04	4.94E-03	1.23E-01
8.21	8.17	8.12	4.99E-03	1.24E-01
8.13	7.93	8.07	4.99E-03	1.24E-01
7.27	7.29	7.52	5.01E-03	1.24E-01
7.29	6.83	7.00	5.03E-03	1.24E-01
11.37	11.35	11.38	5.03E-03	1.24E-01
5.32	5.77	5.41	5.04E-03	1.24E-01
14.57	14.54	14.55	5.05E-03	1.24E-01
9.07	9.27	9.23	5.06E-03	1.24E-01
10.67	10.55	10.56	5.06E-03	1.24E-01
10.04	10.12	10.14	5.09E-03	1.25E-01
4.16	4.08	4.12	5.11E-03	1.25E-01
12.97	12.98	12.92	5.11E-03	1.25E-01
7.14	7.09	7.31	5.13E-03	1.25E-01
9.49	9.50	9.67	5.16E-03	1.26E-01
7.48	7.60	7.57	5.18E-03	1.26E-01
10.26	10.33	10.32	5.19E-03	1.26E-01
10.22	10.06	10.13	5.20E-03	1.26E-01
5.05	5.10	4.99	5.20E-03	1.26E-01
9.94	9.86	9.89	5.22E-03	1.26E-01
3.15	3.15	3.17	5.23E-03	1.26E-01
5.34	5.62	5.60	5.27E-03	1.26E-01
3.71	3.65	3.83	5.27E-03	1.26E-01
8.80	8.89	8.93	5.29E-03	1.26E-01
11.54	11.56	11.50	5.29E-03	1.26E-01
8.90	9.04	9.01	5.30E-03	1.26E-01
6.57	6.39	6.64	5.31E-03	1.26E-01
5.78	5.91	5.97	5.31E-03	1.26E-01
9.00	8.57	9.05	5.31E-03	1.26E-01
6.97	6.96	6.95	5.32E-03	1.26E-01
12.99	13.03	13.00	5.32E-03	1.26E-01
11.12	11.13	11.15	5.33E-03	1.26E-01
12.35	12.40	12.40	5.34E-03	1.26E-01
8.15	8.07	8.18	5.34E-03	1.26E-01
10.74	10.74	10.81	5.35E-03	1.26E-01
7.53	7.53	7.55	5.36E-03	1.26E-01
7.24	7.21	7.40	5.38E-03	1.27E-01
8.65	8.59	8.73	5.40E-03	1.27E-01
4.73	4.69	4.65	5.41E-03	1.27E-01
9.63	9.52	9.59	5.45E-03	1.28E-01
8.42	8.66	8.61	5.46E-03	1.28E-01
3.56	3.62	3.56	5.47E-03	1.28E-01
8.27	8.26	8.26	5.47E-03	1.28E-01
8.05	8.02	8.03	5.48E-03	1.28E-01

10.27	10.10	10.30	5.51E-03	1.28E-01
10.53	10.58	10.58	5.51E-03	1.28E-01
7.53	7.53	7.47	5.51E-03	1.28E-01
7.65	7.50	7.77	5.51E-03	1.28E-01
5.34	5.35	5.31	5.51E-03	1.28E-01
7.79	7.84	7.85	5.52E-03	1.28E-01
9.69	9.63	9.65	5.54E-03	1.28E-01
8.71	8.91	8.81	5.59E-03	1.29E-01
9.54	9.43	9.65	5.59E-03	1.29E-01
6.12	5.81	6.01	5.61E-03	1.29E-01
8.59	8.72	8.71	5.61E-03	1.29E-01
6.01	6.17	6.39	5.62E-03	1.29E-01
8.55	8.60	8.54	5.63E-03	1.29E-01
10.00	9.70	10.06	5.65E-03	1.29E-01
8.29	8.23	8.23	5.67E-03	1.29E-01
9.66	9.48	9.74	5.67E-03	1.29E-01
12.31	11.64	12.20	5.68E-03	1.29E-01
8.34	8.51	8.56	5.69E-03	1.29E-01
7.64	7.66	7.80	5.70E-03	1.29E-01
9.85	9.78	9.85	5.71E-03	1.29E-01
4.78	4.89	4.71	5.71E-03	1.29E-01
8.98	8.76	9.01	5.72E-03	1.29E-01
7.26	7.35	7.28	5.73E-03	1.30E-01
8.74	8.68	8.68	5.75E-03	1.30E-01
7.76	7.60	7.71	5.78E-03	1.30E-01
11.06	11.14	11.12	5.78E-03	1.30E-01
10.04	10.02	10.01	5.81E-03	1.30E-01
6.10	6.27	6.21	5.85E-03	1.31E-01
7.07	7.12	7.13	5.87E-03	1.32E-01
7.46	7.38	7.53	5.88E-03	1.32E-01
11.29	11.40	11.29	5.91E-03	1.32E-01
7.95	7.90	8.00	5.92E-03	1.32E-01
7.71	7.85	7.66	5.92E-03	1.32E-01
4.18	4.10	4.16	5.94E-03	1.32E-01
12.22	12.28	12.28	5.95E-03	1.32E-01
10.42	10.56	10.51	5.98E-03	1.33E-01
4.92	4.83	5.16	6.00E-03	1.33E-01
8.22	8.34	8.22	6.01E-03	1.33E-01
6.74	6.70	6.66	6.02E-03	1.33E-01
12.58	12.66	12.58	6.02E-03	1.33E-01
9.51	9.46	9.60	6.06E-03	1.33E-01
7.22	7.34	7.33	6.07E-03	1.33E-01
10.60	10.62	10.57	6.07E-03	1.33E-01
9.96	10.48	10.12	6.09E-03	1.34E-01
11.15	11.25	11.24	6.10E-03	1.34E-01
7.93	7.85	7.81	6.10E-03	1.34E-01
9.39	9.63	9.39	6.14E-03	1.34E-01

13.33	13.31	13.36	6.16E-03	1.34E-01
6.29	6.33	6.37	6.16E-03	1.34E-01
13.05	13.02	13.06	6.16E-03	1.34E-01
5.10	5.10	5.07	6.18E-03	1.34E-01
11.64	11.73	11.66	6.19E-03	1.34E-01
7.36	7.41	7.48	6.20E-03	1.34E-01
7.90	8.23	7.90	6.22E-03	1.35E-01
7.77	7.72	7.89	6.22E-03	1.35E-01
3.64	3.61	3.69	6.27E-03	1.36E-01
10.29	10.46	10.28	6.32E-03	1.36E-01
5.07	5.00	4.80	6.33E-03	1.36E-01
10.81	10.61	10.83	6.35E-03	1.36E-01
6.00	6.08	6.04	6.35E-03	1.36E-01
3.79	3.83	3.83	6.35E-03	1.36E-01
9.48	9.62	9.55	6.38E-03	1.37E-01
12.04	12.13	12.11	6.39E-03	1.37E-01
6.56	6.53	6.47	6.40E-03	1.37E-01
10.32	10.42	10.39	6.41E-03	1.37E-01
8.47	8.47	8.47	6.42E-03	1.37E-01
8.40	8.56	8.46	6.46E-03	1.38E-01
7.36	7.49	7.35	6.48E-03	1.38E-01
6.78	6.82	6.84	6.50E-03	1.38E-01
9.11	9.07	9.16	6.50E-03	1.38E-01
4.32	4.29	4.20	6.51E-03	1.38E-01
8.57	8.53	8.63	6.51E-03	1.38E-01
10.47	10.62	10.55	6.54E-03	1.38E-01
10.54	10.50	10.55	6.56E-03	1.38E-01
5.18	5.11	5.19	6.60E-03	1.39E-01
9.53	9.80	9.66	6.61E-03	1.39E-01
11.01	10.98	10.92	6.62E-03	1.39E-01
8.72	8.62	8.86	6.62E-03	1.39E-01
9.63	9.53	9.69	6.63E-03	1.39E-01
10.01	9.90	9.96	6.64E-03	1.39E-01
9.94	9.91	9.91	6.65E-03	1.39E-01
9.09	9.28	9.16	6.65E-03	1.39E-01
12.36	12.39	12.45	6.66E-03	1.39E-01
10.17	10.30	10.25	6.67E-03	1.39E-01
8.73	8.72	8.75	6.67E-03	1.39E-01
7.85	7.97	7.88	6.67E-03	1.39E-01
8.39	8.34	8.39	6.67E-03	1.39E-01
6.81	6.50	6.56	6.69E-03	1.39E-01
5.10	5.18	5.03	6.71E-03	1.39E-01
4.29	4.46	4.39	6.72E-03	1.39E-01
4.19	4.17	4.14	6.73E-03	1.39E-01
7.00	7.22	7.11	6.73E-03	1.39E-01
8.49	8.53	8.52	6.75E-03	1.39E-01
5.69	5.60	5.55	6.75E-03	1.39E-01

9.09	9.10	8.97	6.76E-03	1.39E-01
7.80	8.00	7.85	6.78E-03	1.39E-01
5.24	5.22	5.26	6.78E-03	1.39E-01
11.02	11.15	11.12	6.80E-03	1.39E-01
8.50	8.38	8.51	6.83E-03	1.40E-01
5.88	5.84	5.94	6.84E-03	1.40E-01
5.13	5.07	5.06	6.85E-03	1.40E-01
6.10	6.17	6.17	6.89E-03	1.40E-01
9.35	9.39	9.45	6.90E-03	1.40E-01
8.58	8.71	8.60	6.91E-03	1.40E-01
8.05	7.88	8.29	6.94E-03	1.41E-01
7.70	7.76	7.95	6.96E-03	1.41E-01
4.65	4.61	4.65	6.96E-03	1.41E-01
11.02	11.02	11.03	6.97E-03	1.41E-01
7.10	6.83	7.14	6.98E-03	1.41E-01
7.76	7.81	7.80	6.98E-03	1.41E-01
10.16	10.28	10.21	6.99E-03	1.41E-01
4.41	4.34	4.73	7.01E-03	1.41E-01
12.83	12.90	12.86	7.02E-03	1.41E-01
6.23	6.25	6.25	7.05E-03	1.41E-01
10.71	10.79	10.74	7.09E-03	1.42E-01
10.07	9.99	10.13	7.10E-03	1.42E-01
11.58	11.51	11.62	7.10E-03	1.42E-01
9.71	9.67	9.68	7.10E-03	1.42E-01
6.13	6.23	6.31	7.13E-03	1.42E-01
10.55	10.65	10.57	7.14E-03	1.42E-01
8.52	8.36	8.56	7.14E-03	1.42E-01
6.63	6.85	6.84	7.16E-03	1.42E-01
8.55	8.52	8.65	7.17E-03	1.42E-01
8.60	8.63	8.78	7.18E-03	1.42E-01
10.18	10.31	10.35	7.18E-03	1.42E-01
6.75	6.74	6.76	7.19E-03	1.42E-01
6.40	6.42	6.47	7.19E-03	1.42E-01
7.40	7.50	7.32	7.21E-03	1.42E-01
7.36	7.35	7.48	7.23E-03	1.42E-01
12.21	12.21	12.29	7.23E-03	1.42E-01
7.39	7.27	7.42	7.24E-03	1.42E-01
8.26	8.22	8.32	7.24E-03	1.42E-01
7.52	7.55	7.59	7.25E-03	1.42E-01
6.36	6.50	6.44	7.26E-03	1.42E-01
8.76	8.74	8.63	7.26E-03	1.42E-01
10.69	10.83	10.78	7.28E-03	1.42E-01
6.22	6.26	6.39	7.28E-03	1.42E-01
9.04	9.20	9.07	7.29E-03	1.42E-01
8.00	8.07	8.02	7.30E-03	1.42E-01
7.68	7.75	7.72	7.33E-03	1.42E-01
8.45	8.47	8.47	7.34E-03	1.42E-01

6.41	6.38	6.49	7.34E-03	1.42E-01
4.97	4.94	5.02	7.38E-03	1.43E-01
11.36	11.47	11.44	7.41E-03	1.44E-01
6.40	6.94	6.55	7.44E-03	1.44E-01
6.25	6.38	6.32	7.44E-03	1.44E-01
6.17	6.01	6.14	7.46E-03	1.44E-01
9.67	9.78	9.76	7.48E-03	1.44E-01
6.17	6.27	6.20	7.49E-03	1.44E-01
8.36	8.35	8.34	7.49E-03	1.44E-01
9.92	9.89	9.90	7.51E-03	1.44E-01
9.14	9.06	9.06	7.51E-03	1.44E-01
8.47	8.23	8.68	7.55E-03	1.44E-01
8.48	8.56	8.46	7.56E-03	1.44E-01
11.54	11.62	11.49	7.56E-03	1.44E-01
10.34	10.40	10.50	7.57E-03	1.44E-01
10.72	10.63	10.73	7.57E-03	1.44E-01
6.45	6.67	6.59	7.59E-03	1.44E-01
8.03	8.08	8.03	7.59E-03	1.44E-01
6.43	6.11	6.44	7.60E-03	1.44E-01
3.98	4.10	3.98	7.61E-03	1.44E-01
5.88	5.83	5.81	7.64E-03	1.44E-01
11.51	11.64	11.51	7.65E-03	1.44E-01
6.11	6.17	6.12	7.66E-03	1.44E-01
7.76	7.75	7.91	7.67E-03	1.44E-01
8.14	8.17	8.15	7.68E-03	1.44E-01
3.83	3.83	3.85	7.68E-03	1.44E-01
13.27	13.14	13.30	7.68E-03	1.44E-01
8.28	8.42	8.17	7.68E-03	1.44E-01
11.96	11.86	11.96	7.70E-03	1.45E-01
5.87	5.78	5.51	7.71E-03	1.45E-01
6.59	6.36	6.51	7.73E-03	1.45E-01
5.79	5.75	5.78	7.73E-03	1.45E-01
10.76	10.60	10.71	7.74E-03	1.45E-01
5.91	6.03	5.95	7.75E-03	1.45E-01
7.96	7.84	7.91	7.75E-03	1.45E-01
6.08	6.17	6.08	7.77E-03	1.45E-01
10.65	10.75	10.69	7.79E-03	1.45E-01
4.23	4.07	4.20	7.80E-03	1.45E-01
8.01	8.12	8.11	7.80E-03	1.45E-01
6.65	6.53	6.50	7.81E-03	1.45E-01
6.15	6.05	6.32	7.83E-03	1.45E-01
5.37	5.61	5.48	7.86E-03	1.45E-01
8.69	8.81	8.68	7.88E-03	1.45E-01
3.85	3.90	3.87	7.89E-03	1.45E-01
8.84	8.74	8.80	7.89E-03	1.45E-01
8.15	8.37	8.41	7.90E-03	1.45E-01
3.74	3.74	3.64	7.90E-03	1.45E-01

9.21	9.33	9.35	7.90E-03	1.45E-01
5.37	5.47	5.30	7.92E-03	1.45E-01
8.56	8.60	8.54	7.95E-03	1.46E-01
9.34	9.35	9.43	7.96E-03	1.46E-01
5.12	5.13	5.04	7.96E-03	1.46E-01
9.74	9.90	9.77	7.98E-03	1.46E-01
12.95	12.81	13.04	7.98E-03	1.46E-01
12.25	12.31	12.30	8.00E-03	1.46E-01
7.77	7.88	7.85	8.04E-03	1.46E-01
12.11	12.08	12.24	8.04E-03	1.46E-01
7.09	7.05	7.09	8.04E-03	1.46E-01
9.32	9.28	9.41	8.05E-03	1.46E-01
5.44	5.80	5.83	8.06E-03	1.46E-01
7.80	7.81	7.75	8.08E-03	1.46E-01
3.60	3.60	3.60	8.08E-03	1.46E-01
8.84	8.84	8.89	8.11E-03	1.47E-01
10.53	10.62	10.53	8.16E-03	1.47E-01
6.98	7.13	6.96	8.19E-03	1.48E-01
8.24	8.29	8.22	8.20E-03	1.48E-01
11.70	11.65	11.65	8.21E-03	1.48E-01
3.72	3.75	3.76	8.21E-03	1.48E-01
10.14	10.22	10.14	8.23E-03	1.48E-01
9.63	9.50	9.66	8.25E-03	1.48E-01
9.54	9.52	9.59	8.26E-03	1.48E-01
7.25	7.23	7.32	8.27E-03	1.48E-01
5.95	5.84	6.00	8.28E-03	1.48E-01
8.90	8.41	8.79	8.30E-03	1.48E-01
3.78	3.79	3.72	8.31E-03	1.48E-01
7.29	7.21	7.28	8.35E-03	1.48E-01
6.23	6.45	6.74	8.36E-03	1.48E-01
5.34	5.42	5.32	8.38E-03	1.48E-01
9.66	9.47	9.65	8.38E-03	1.48E-01
5.24	5.18	5.08	8.38E-03	1.48E-01
10.87	10.68	10.82	8.39E-03	1.48E-01
10.00	9.97	9.99	8.40E-03	1.48E-01
7.98	8.09	8.04	8.40E-03	1.48E-01
7.32	7.05	7.34	8.41E-03	1.48E-01
5.80	5.78	5.88	8.41E-03	1.48E-01
6.62	6.29	6.64	8.41E-03	1.48E-01
5.91	5.78	5.91	8.43E-03	1.48E-01
5.33	5.22	5.50	8.43E-03	1.48E-01
11.83	11.83	11.86	8.45E-03	1.48E-01
5.84	5.94	5.94	8.47E-03	1.48E-01
5.47	5.66	5.39	8.48E-03	1.48E-01
9.27	9.26	9.27	8.48E-03	1.48E-01
9.41	9.47	9.53	8.49E-03	1.48E-01
10.86	11.04	10.95	8.49E-03	1.48E-01

3.58	3.45	3.60	8.50E-03	1.48E-01
9.30	9.49	9.46	8.53E-03	1.49E-01
4.51	4.58	4.76	8.58E-03	1.49E-01
9.10	8.84	9.22	8.58E-03	1.49E-01
8.60	8.38	8.72	8.61E-03	1.50E-01
6.05	5.85	6.22	8.62E-03	1.50E-01
8.08	8.08	8.19	8.63E-03	1.50E-01
11.04	10.99	11.02	8.69E-03	1.51E-01
5.82	5.74	5.89	8.72E-03	1.51E-01
3.87	3.97	4.00	8.74E-03	1.51E-01
12.30	12.38	12.29	8.75E-03	1.51E-01
6.90	6.79	6.79	8.78E-03	1.51E-01
9.23	9.34	9.23	8.78E-03	1.51E-01
6.44	6.39	6.54	8.81E-03	1.52E-01
5.01	4.87	4.87	8.83E-03	1.52E-01
8.00	7.62	8.15	8.84E-03	1.52E-01
8.28	8.26	8.34	8.92E-03	1.53E-01
4.00	4.00	4.04	8.92E-03	1.53E-01
3.49	3.51	3.55	8.93E-03	1.53E-01
5.28	5.60	5.37	8.93E-03	1.53E-01
7.83	7.79	7.68	8.95E-03	1.53E-01
5.36	5.51	5.43	8.97E-03	1.53E-01
7.84	7.63	7.69	8.98E-03	1.53E-01
3.92	3.96	4.00	8.98E-03	1.53E-01
7.80	7.63	8.13	9.03E-03	1.53E-01
15.14	15.11	15.11	9.03E-03	1.53E-01
8.28	8.49	8.42	9.04E-03	1.53E-01
10.82	10.88	10.97	9.09E-03	1.54E-01
9.26	9.30	9.31	9.09E-03	1.54E-01
4.80	4.76	4.69	9.10E-03	1.54E-01
8.88	9.07	8.89	9.12E-03	1.54E-01
8.28	8.22	8.22	9.15E-03	1.54E-01
10.97	11.12	11.17	9.16E-03	1.54E-01
8.92	8.91	8.89	9.17E-03	1.54E-01
4.19	4.00	4.21	9.17E-03	1.54E-01
8.28	8.28	8.35	9.18E-03	1.55E-01
6.78	6.84	6.84	9.20E-03	1.55E-01
12.87	12.95	12.93	9.22E-03	1.55E-01
11.88	12.00	11.95	9.26E-03	1.55E-01
6.39	6.52	6.30	9.29E-03	1.56E-01
7.52	7.74	7.70	9.30E-03	1.56E-01
7.94	8.00	8.04	9.35E-03	1.56E-01
10.39	10.36	10.42	9.35E-03	1.56E-01
9.28	9.27	9.29	9.37E-03	1.56E-01
10.14	10.27	10.26	9.38E-03	1.56E-01
10.87	11.07	10.95	9.39E-03	1.56E-01
12.59	12.54	12.59	9.40E-03	1.56E-01

8.47	8.62	8.57	9.41E-03	1.56E-01
8.50	8.70	8.58	9.41E-03	1.56E-01
7.82	7.81	7.85	9.41E-03	1.56E-01
5.66	5.64	5.74	9.43E-03	1.56E-01
8.65	8.57	8.63	9.47E-03	1.57E-01
6.86	6.86	6.99	9.47E-03	1.57E-01
5.13	5.11	5.01	9.49E-03	1.57E-01
5.59	5.21	5.29	9.50E-03	1.57E-01
10.93	10.89	10.86	9.51E-03	1.57E-01
6.05	6.03	6.08	9.51E-03	1.57E-01
9.65	9.59	9.59	9.55E-03	1.57E-01
6.05	5.93	5.95	9.58E-03	1.58E-01
7.79	7.95	7.81	9.63E-03	1.58E-01
5.42	5.44	5.34	9.67E-03	1.59E-01
10.53	10.47	10.56	9.68E-03	1.59E-01
6.23	6.20	5.93	9.73E-03	1.59E-01
7.46	7.50	7.42	9.75E-03	1.59E-01
8.31	8.31	8.37	9.77E-03	1.59E-01
3.89	3.68	3.97	9.80E-03	1.59E-01
6.54	6.55	6.54	9.81E-03	1.59E-01
8.48	8.58	8.65	9.82E-03	1.59E-01
6.59	6.54	6.59	9.83E-03	1.59E-01
11.96	11.98	11.92	9.83E-03	1.59E-01
9.63	9.72	9.81	9.84E-03	1.59E-01
6.92	7.00	6.95	9.84E-03	1.59E-01
13.54	13.37	13.39	9.85E-03	1.59E-01
9.08	8.86	9.00	9.85E-03	1.59E-01
4.90	4.72	4.93	9.85E-03	1.59E-01
7.25	7.19	7.26	9.86E-03	1.59E-01
3.97	3.99	3.95	9.86E-03	1.59E-01
7.40	7.64	7.51	9.86E-03	1.59E-01
11.11	11.05	11.04	9.87E-03	1.59E-01
4.79	4.77	4.87	9.89E-03	1.59E-01
6.85	6.89	6.85	9.93E-03	1.60E-01
10.13	10.23	10.25	9.94E-03	1.60E-01
7.58	7.73	7.68	9.99E-03	1.60E-01
9.91	9.81	10.03	1.00E-02	1.60E-01
10.38	10.39	10.33	1.00E-02	1.60E-01
10.38	10.26	10.33	1.01E-02	1.61E-01
10.14	9.72	10.07	1.01E-02	1.61E-01
10.43	10.47	10.49	1.01E-02	1.61E-01
8.90	8.85	8.96	1.01E-02	1.61E-01
7.36	7.42	7.45	1.01E-02	1.61E-01
3.43	3.51	3.43	1.01E-02	1.61E-01
7.64	7.72	7.66	1.02E-02	1.62E-01
7.20	7.31	7.35	1.02E-02	1.62E-01
6.85	6.99	6.95	1.02E-02	1.62E-01

9.14	9.23	9.24	1.02E-02	1.62E-01
12.66	12.74	12.67	1.02E-02	1.62E-01
7.81	8.15	8.08	1.02E-02	1.62E-01
9.51	9.58	9.65	1.02E-02	1.62E-01
9.20	9.19	9.18	1.03E-02	1.63E-01
8.72	8.94	8.78	1.03E-02	1.63E-01
6.58	6.69	6.62	1.04E-02	1.63E-01
11.12	11.13	11.18	1.04E-02	1.63E-01
8.55	8.48	8.59	1.04E-02	1.63E-01
6.20	6.27	6.24	1.04E-02	1.63E-01
4.03	4.08	4.06	1.04E-02	1.63E-01
13.09	13.11	13.08	1.04E-02	1.63E-01
9.57	9.72	9.74	1.04E-02	1.63E-01
7.60	7.85	7.75	1.04E-02	1.63E-01
8.82	9.00	8.62	1.04E-02	1.63E-01
12.48	12.47	12.55	1.04E-02	1.63E-01
9.18	9.51	9.29	1.04E-02	1.63E-01
9.67	9.52	9.75	1.05E-02	1.63E-01
10.94	10.83	10.96	1.05E-02	1.63E-01
4.47	4.32	4.33	1.05E-02	1.63E-01
10.40	10.38	10.37	1.05E-02	1.63E-01
7.08	7.11	7.03	1.05E-02	1.63E-01
8.05	8.00	8.09	1.05E-02	1.63E-01
11.98	12.02	12.05	1.05E-02	1.63E-01
6.24	6.11	6.19	1.06E-02	1.64E-01
12.10	12.10	12.29	1.06E-02	1.64E-01
7.99	7.89	7.92	1.06E-02	1.64E-01
8.02	8.00	8.09	1.06E-02	1.64E-01
6.38	6.23	6.12	1.06E-02	1.64E-01
9.15	9.34	9.24	1.06E-02	1.64E-01
8.56	8.60	8.62	1.06E-02	1.64E-01
8.91	8.89	9.05	1.06E-02	1.64E-01
12.30	12.32	12.35	1.07E-02	1.64E-01
8.70	8.83	8.75	1.07E-02	1.64E-01
7.79	7.78	7.75	1.07E-02	1.64E-01
11.02	11.11	11.12	1.07E-02	1.64E-01
7.46	7.52	7.38	1.07E-02	1.64E-01
6.01	5.92	6.02	1.08E-02	1.65E-01
6.26	6.13	6.17	1.08E-02	1.65E-01
9.02	8.98	9.11	1.08E-02	1.65E-01
4.62	5.01	4.54	1.08E-02	1.65E-01
4.27	4.13	4.01	1.08E-02	1.65E-01
8.16	8.20	8.15	1.08E-02	1.65E-01
5.75	5.80	5.67	1.08E-02	1.65E-01
7.43	7.56	7.48	1.08E-02	1.65E-01
6.37	6.43	6.43	1.09E-02	1.65E-01
5.53	5.56	5.55	1.09E-02	1.65E-01

7.40	7.27	7.25	1.09E-02	1.65E-01
4.42	4.39	4.33	1.09E-02	1.65E-01
7.89	7.94	7.88	1.09E-02	1.65E-01
9.19	8.83	8.99	1.09E-02	1.65E-01
3.51	3.61	3.60	1.09E-02	1.65E-01
10.97	11.10	11.04	1.09E-02	1.65E-01
9.26	9.19	9.32	1.10E-02	1.65E-01
6.30	6.40	6.38	1.10E-02	1.66E-01
7.08	7.08	7.19	1.10E-02	1.66E-01
13.32	13.37	13.37	1.10E-02	1.66E-01
11.45	11.35	11.46	1.10E-02	1.66E-01
7.75	7.99	7.90	1.11E-02	1.66E-01
8.93	9.06	9.03	1.11E-02	1.66E-01
10.64	10.54	10.69	1.11E-02	1.66E-01
7.84	7.85	7.93	1.11E-02	1.66E-01
5.34	5.35	5.27	1.11E-02	1.66E-01
6.77	6.90	6.82	1.11E-02	1.66E-01
7.06	7.25	7.19	1.11E-02	1.66E-01
6.63	6.68	6.61	1.11E-02	1.66E-01
11.37	11.28	11.44	1.12E-02	1.66E-01
9.57	9.56	9.59	1.12E-02	1.66E-01
9.27	9.30	9.23	1.12E-02	1.66E-01
7.88	7.97	7.91	1.12E-02	1.66E-01
10.36	10.41	10.46	1.12E-02	1.66E-01
10.05	10.18	10.02	1.12E-02	1.67E-01
7.28	7.32	7.34	1.12E-02	1.67E-01
7.09	7.19	7.19	1.13E-02	1.67E-01
10.41	10.50	10.45	1.13E-02	1.67E-01
8.57	8.42	8.57	1.13E-02	1.67E-01
10.37	10.44	10.50	1.13E-02	1.67E-01
11.33	11.30	11.34	1.14E-02	1.68E-01
8.12	8.01	8.17	1.14E-02	1.68E-01
10.07	10.18	10.20	1.14E-02	1.68E-01
8.90	8.64	8.87	1.14E-02	1.68E-01
6.65	6.65	6.69	1.14E-02	1.68E-01
9.43	9.55	9.41	1.14E-02	1.68E-01
4.87	4.78	4.85	1.14E-02	1.68E-01
9.06	9.03	8.89	1.14E-02	1.68E-01
10.43	10.26	10.36	1.15E-02	1.68E-01
13.10	12.98	13.10	1.15E-02	1.68E-01
8.00	8.04	8.03	1.15E-02	1.68E-01
4.34	4.24	4.51	1.15E-02	1.68E-01
7.28	7.13	7.21	1.15E-02	1.68E-01
4.04	4.09	4.09	1.16E-02	1.68E-01
7.31	7.25	7.33	1.16E-02	1.68E-01
9.08	9.17	9.04	1.16E-02	1.68E-01
7.97	7.99	8.25	1.16E-02	1.68E-01

6.55	6.52	6.64	1.16E-02	1.68E-01
8.93	9.32	9.13	1.16E-02	1.68E-01
11.50	11.63	11.76	1.16E-02	1.69E-01
9.17	9.45	9.27	1.17E-02	1.69E-01
6.93	7.01	6.91	1.17E-02	1.69E-01
4.69	4.76	4.61	1.17E-02	1.69E-01
9.30	9.25	9.31	1.17E-02	1.69E-01
9.03	9.13	9.12	1.17E-02	1.69E-01
4.41	4.40	4.38	1.17E-02	1.69E-01
10.56	10.56	10.69	1.17E-02	1.69E-01
11.09	11.05	11.08	1.17E-02	1.69E-01
8.19	8.03	8.27	1.18E-02	1.69E-01
3.29	3.22	3.26	1.18E-02	1.70E-01
12.64	12.74	12.65	1.18E-02	1.70E-01
3.62	3.52	3.51	1.19E-02	1.70E-01
11.73	11.75	11.76	1.19E-02	1.70E-01
8.65	8.65	8.68	1.19E-02	1.70E-01
9.88	9.78	9.96	1.19E-02	1.70E-01
9.57	9.73	9.69	1.19E-02	1.70E-01
6.61	6.77	6.89	1.19E-02	1.70E-01
8.24	8.29	8.33	1.19E-02	1.71E-01
10.75	10.80	10.76	1.19E-02	1.71E-01
8.81	8.85	8.89	1.20E-02	1.71E-01
5.34	5.40	5.20	1.20E-02	1.71E-01
9.62	9.67	9.68	1.20E-02	1.71E-01
8.77	8.70	8.73	1.20E-02	1.71E-01
9.54	9.58	9.55	1.20E-02	1.71E-01
9.02	8.96	8.99	1.20E-02	1.71E-01
7.97	8.03	7.84	1.20E-02	1.71E-01
8.29	8.39	8.31	1.20E-02	1.71E-01
10.39	10.50	10.41	1.20E-02	1.71E-01
4.79	4.85	4.75	1.21E-02	1.71E-01
10.38	10.43	10.44	1.21E-02	1.71E-01
10.07	10.42	10.06	1.21E-02	1.71E-01
4.64	4.35	4.50	1.21E-02	1.71E-01
10.57	10.61	10.57	1.21E-02	1.71E-01
9.09	8.72	8.90	1.22E-02	1.71E-01
8.32	8.25	8.21	1.22E-02	1.71E-01
8.07	8.13	8.05	1.22E-02	1.71E-01
7.50	7.64	7.76	1.22E-02	1.71E-01
5.63	5.46	5.47	1.22E-02	1.71E-01
8.87	8.87	8.94	1.22E-02	1.71E-01
3.00	3.26	2.94	1.22E-02	1.71E-01
9.16	9.12	9.12	1.22E-02	1.71E-01
7.19	7.30	7.17	1.23E-02	1.71E-01
8.41	8.42	8.46	1.23E-02	1.71E-01
6.61	6.61	6.84	1.23E-02	1.71E-01

3.33	3.31	3.38	1.23E-02	1.72E-01
8.02	8.18	8.08	1.23E-02	1.72E-01
9.86	9.82	9.66	1.24E-02	1.72E-01
6.34	6.39	6.34	1.24E-02	1.72E-01
9.30	9.24	9.56	1.24E-02	1.72E-01
4.12	4.06	4.16	1.24E-02	1.72E-01
7.84	7.96	7.83	1.24E-02	1.72E-01
6.92	6.96	7.03	1.24E-02	1.72E-01
10.59	10.45	10.62	1.24E-02	1.72E-01
8.76	8.53	8.65	1.24E-02	1.72E-01
9.52	9.54	9.65	1.24E-02	1.72E-01
9.89	9.92	9.85	1.25E-02	1.72E-01
8.71	8.80	8.87	1.25E-02	1.72E-01
10.90	10.93	10.98	1.25E-02	1.72E-01
10.57	10.64	10.62	1.25E-02	1.73E-01
10.28	10.33	10.42	1.25E-02	1.73E-01
10.00	10.21	10.06	1.26E-02	1.73E-01
7.96	7.99	7.89	1.26E-02	1.73E-01
8.59	8.65	8.67	1.26E-02	1.73E-01
4.31	4.12	4.21	1.26E-02	1.73E-01
9.13	9.15	9.20	1.27E-02	1.74E-01
9.92	9.85	9.93	1.27E-02	1.74E-01
9.77	9.95	9.82	1.27E-02	1.74E-01
11.63	11.43	11.59	1.28E-02	1.74E-01
12.30	12.42	12.36	1.28E-02	1.74E-01
6.71	6.53	6.75	1.28E-02	1.74E-01
9.30	9.38	9.35	1.28E-02	1.74E-01
6.97	7.02	6.84	1.28E-02	1.75E-01
7.36	7.36	7.42	1.28E-02	1.75E-01
8.58	8.71	8.54	1.28E-02	1.75E-01
11.50	11.51	11.55	1.29E-02	1.75E-01
11.10	11.27	11.19	1.29E-02	1.75E-01
4.77	4.83	4.63	1.29E-02	1.75E-01
7.12	7.36	7.19	1.29E-02	1.75E-01
9.10	9.23	9.15	1.29E-02	1.75E-01
9.40	9.42	9.38	1.29E-02	1.75E-01
11.36	11.49	11.47	1.30E-02	1.76E-01
4.45	4.45	4.48	1.30E-02	1.76E-01
6.31	6.42	6.32	1.30E-02	1.76E-01
8.47	8.52	8.50	1.30E-02	1.76E-01
7.55	7.43	7.58	1.30E-02	1.76E-01
11.13	11.05	11.23	1.30E-02	1.76E-01
3.11	3.08	3.05	1.30E-02	1.76E-01
9.10	9.13	9.17	1.31E-02	1.76E-01
14.41	14.43	14.38	1.31E-02	1.76E-01
7.13	7.21	7.48	1.31E-02	1.76E-01
10.89	11.01	10.96	1.32E-02	1.77E-01

7.42	7.36	7.34	1.32E-02	1.77E-01
4.65	5.09	5.14	1.32E-02	1.78E-01
7.94	7.95	8.02	1.33E-02	1.78E-01
11.01	10.34	10.92	1.33E-02	1.78E-01
6.97	7.10	7.13	1.33E-02	1.78E-01
9.88	10.11	10.02	1.33E-02	1.78E-01
5.87	5.84	5.79	1.33E-02	1.78E-01
4.84	4.80	4.76	1.33E-02	1.78E-01
6.48	6.57	6.48	1.33E-02	1.78E-01
6.48	6.34	6.37	1.34E-02	1.78E-01
5.08	5.12	5.09	1.34E-02	1.78E-01
9.27	9.56	9.22	1.34E-02	1.78E-01
4.69	4.54	4.72	1.34E-02	1.79E-01
7.58	7.56	7.53	1.35E-02	1.79E-01
12.63	12.77	12.74	1.35E-02	1.79E-01
4.10	3.93	4.12	1.35E-02	1.79E-01
10.35	10.48	10.33	1.35E-02	1.79E-01
5.85	5.83	5.86	1.35E-02	1.79E-01
9.79	9.70	9.92	1.35E-02	1.79E-01
3.40	3.43	3.35	1.35E-02	1.79E-01
11.88	11.83	11.79	1.36E-02	1.79E-01
6.96	6.93	7.02	1.36E-02	1.79E-01
7.17	7.22	7.19	1.36E-02	1.79E-01
9.43	9.32	9.42	1.36E-02	1.79E-01
6.06	6.10	6.08	1.37E-02	1.80E-01
10.70	10.81	10.73	1.37E-02	1.80E-01
8.02	8.13	8.07	1.37E-02	1.80E-01
7.34	7.54	7.51	1.37E-02	1.80E-01
3.57	3.70	3.65	1.37E-02	1.80E-01
5.82	5.65	5.82	1.37E-02	1.80E-01
6.94	6.28	7.73	1.37E-02	1.80E-01
10.49	10.54	10.56	1.38E-02	1.80E-01
4.82	5.01	4.83	1.38E-02	1.80E-01
6.93	7.00	7.10	1.38E-02	1.80E-01
11.05	11.34	11.19	1.38E-02	1.80E-01
9.65	9.73	9.60	1.38E-02	1.80E-01
6.86	6.86	6.75	1.38E-02	1.80E-01
8.42	8.50	8.48	1.39E-02	1.80E-01
6.34	6.38	6.56	1.39E-02	1.80E-01
5.38	5.36	5.52	1.39E-02	1.80E-01
9.55	9.46	9.70	1.39E-02	1.80E-01
7.16	7.20	7.29	1.39E-02	1.80E-01
10.32	10.23	10.28	1.39E-02	1.80E-01
10.81	10.81	10.57	1.39E-02	1.81E-01
9.57	9.46	9.78	1.40E-02	1.81E-01
4.61	4.76	4.75	1.40E-02	1.81E-01
5.96	6.05	5.91	1.40E-02	1.81E-01

6.00	5.86	5.95	1.40E-02	1.82E-01
4.42	4.33	4.33	1.41E-02	1.82E-01
8.05	8.22	8.20	1.41E-02	1.82E-01
3.40	3.49	3.46	1.41E-02	1.82E-01
9.12	9.23	9.10	1.41E-02	1.82E-01
7.56	7.74	7.59	1.41E-02	1.82E-01
13.78	13.74	13.75	1.41E-02	1.82E-01
6.77	6.78	6.71	1.41E-02	1.82E-01
7.78	7.59	7.79	1.42E-02	1.82E-01
7.24	7.34	7.33	1.42E-02	1.82E-01
4.27	4.04	4.15	1.42E-02	1.82E-01
10.79	10.96	10.90	1.42E-02	1.82E-01
11.90	12.12	12.02	1.42E-02	1.82E-01
8.19	8.28	8.28	1.43E-02	1.82E-01
3.77	3.81	3.90	1.43E-02	1.82E-01
6.70	7.13	6.84	1.43E-02	1.82E-01
8.26	8.20	8.19	1.43E-02	1.82E-01
6.50	6.42	6.53	1.43E-02	1.82E-01
11.02	11.02	11.13	1.43E-02	1.82E-01
5.73	5.80	5.80	1.44E-02	1.83E-01
3.08	3.09	3.10	1.44E-02	1.83E-01
4.43	4.32	4.38	1.44E-02	1.83E-01
6.89	6.75	6.82	1.44E-02	1.83E-01
3.25	3.36	3.29	1.44E-02	1.83E-01
7.09	7.13	7.12	1.45E-02	1.83E-01
6.26	6.35	6.46	1.45E-02	1.83E-01
13.31	13.12	13.33	1.45E-02	1.83E-01
6.41	6.35	6.43	1.45E-02	1.83E-01
4.90	5.00	5.16	1.45E-02	1.83E-01
10.11	9.98	10.25	1.45E-02	1.83E-01
7.74	7.97	7.90	1.45E-02	1.83E-01
7.15	7.80	6.82	1.45E-02	1.83E-01
5.89	6.02	6.09	1.45E-02	1.83E-01
8.25	8.38	8.54	1.45E-02	1.83E-01
5.81	5.76	5.85	1.45E-02	1.83E-01
9.98	9.95	9.99	1.46E-02	1.83E-01
13.21	13.25	13.26	1.46E-02	1.83E-01
7.08	6.86	6.81	1.46E-02	1.83E-01
11.42	11.44	11.47	1.46E-02	1.83E-01
10.22	10.24	10.31	1.46E-02	1.84E-01
7.67	7.94	7.84	1.47E-02	1.84E-01
11.61	11.46	11.53	1.47E-02	1.84E-01
9.62	9.72	9.72	1.47E-02	1.84E-01
5.23	5.11	5.20	1.47E-02	1.84E-01
5.18	5.27	5.12	1.48E-02	1.85E-01
7.26	7.29	7.16	1.48E-02	1.85E-01
7.88	7.83	7.94	1.48E-02	1.85E-01

8.83	8.99	8.90	1.48E-02	1.85E-01
9.87	10.06	9.88	1.49E-02	1.85E-01
4.99	5.05	4.92	1.49E-02	1.85E-01
6.53	6.51	6.59	1.49E-02	1.85E-01
11.45	11.16	11.49	1.49E-02	1.85E-01
3.94	3.94	4.07	1.49E-02	1.85E-01
8.98	9.09	9.09	1.50E-02	1.85E-01
7.45	7.24	7.31	1.50E-02	1.85E-01
8.05	7.91	7.95	1.50E-02	1.85E-01
6.58	6.63	6.76	1.50E-02	1.85E-01
5.44	5.45	5.43	1.50E-02	1.85E-01
10.04	10.06	10.08	1.50E-02	1.85E-01
6.02	5.97	6.17	1.50E-02	1.85E-01
13.25	13.24	13.21	1.50E-02	1.85E-01
6.41	5.81	6.21	1.50E-02	1.85E-01
4.53	4.43	4.43	1.51E-02	1.85E-01
5.95	5.90	6.05	1.51E-02	1.85E-01
10.28	10.56	10.50	1.51E-02	1.85E-01
7.90	7.93	7.98	1.51E-02	1.85E-01
8.10	8.18	8.24	1.51E-02	1.85E-01
9.57	9.81	9.68	1.51E-02	1.85E-01
6.89	6.81	6.88	1.51E-02	1.85E-01
11.70	11.64	11.64	1.51E-02	1.85E-01
4.29	4.21	4.17	1.51E-02	1.85E-01
3.81	4.02	3.93	1.52E-02	1.85E-01
8.59	8.58	8.63	1.52E-02	1.85E-01
8.72	8.57	8.65	1.52E-02	1.85E-01
7.09	6.84	7.09	1.52E-02	1.85E-01
9.47	9.79	9.67	1.52E-02	1.85E-01
10.66	10.74	10.75	1.52E-02	1.85E-01
4.23	4.12	4.18	1.52E-02	1.85E-01
9.56	9.38	9.41	1.52E-02	1.85E-01
10.76	10.85	10.67	1.53E-02	1.86E-01
6.48	6.39	6.27	1.53E-02	1.86E-01
12.13	12.22	12.24	1.53E-02	1.86E-01
9.45	9.23	9.35	1.53E-02	1.86E-01
9.26	9.13	9.35	1.53E-02	1.86E-01
9.12	9.32	9.17	1.53E-02	1.86E-01
9.88	10.13	10.01	1.53E-02	1.86E-01
7.60	7.70	7.74	1.53E-02	1.86E-01
10.37	10.26	10.27	1.54E-02	1.86E-01
4.66	4.62	4.52	1.54E-02	1.86E-01
9.78	9.68	9.69	1.54E-02	1.86E-01
6.51	6.34	6.45	1.54E-02	1.86E-01
9.93	10.17	10.10	1.54E-02	1.86E-01
10.94	10.91	11.04	1.54E-02	1.86E-01
5.44	5.43	5.45	1.55E-02	1.86E-01

5.29	5.37	5.29	1.55E-02	1.86E-01
9.17	9.39	9.38	1.55E-02	1.86E-01
7.76	7.68	7.78	1.55E-02	1.86E-01
4.12	4.13	4.18	1.55E-02	1.86E-01
8.10	8.11	8.13	1.55E-02	1.86E-01
6.30	6.49	6.25	1.55E-02	1.86E-01
5.20	5.12	5.15	1.55E-02	1.86E-01
9.40	9.48	9.39	1.55E-02	1.86E-01
6.96	6.79	6.93	1.56E-02	1.86E-01
8.64	8.66	8.76	1.56E-02	1.86E-01
12.55	12.58	12.62	1.56E-02	1.86E-01
10.36	10.13	10.38	1.56E-02	1.86E-01
8.70	8.74	8.45	1.56E-02	1.87E-01
6.59	6.60	6.55	1.57E-02	1.87E-01
6.41	6.39	6.48	1.57E-02	1.87E-01
9.84	9.85	9.78	1.57E-02	1.87E-01
9.08	9.09	9.28	1.57E-02	1.87E-01
3.51	3.56	3.42	1.57E-02	1.87E-01
8.99	9.04	8.95	1.58E-02	1.87E-01
8.57	8.67	8.76	1.58E-02	1.87E-01
4.48	4.40	4.41	1.58E-02	1.88E-01
10.94	10.80	10.89	1.59E-02	1.88E-01
4.05	4.06	4.07	1.59E-02	1.88E-01
7.37	7.45	7.51	1.59E-02	1.88E-01
11.98	12.05	11.99	1.59E-02	1.88E-01
8.38	8.52	8.59	1.59E-02	1.88E-01
7.25	7.31	7.28	1.59E-02	1.88E-01
8.42	8.49	8.49	1.59E-02	1.88E-01
6.57	6.63	6.74	1.59E-02	1.88E-01
5.08	5.15	5.13	1.60E-02	1.88E-01
9.64	9.67	9.74	1.60E-02	1.88E-01
9.37	9.39	9.48	1.60E-02	1.88E-01
6.99	7.20	7.16	1.60E-02	1.88E-01
9.39	9.45	9.42	1.60E-02	1.88E-01
4.31	4.44	4.44	1.60E-02	1.88E-01
7.43	7.54	7.39	1.61E-02	1.88E-01
4.40	4.69	4.42	1.61E-02	1.88E-01
7.74	7.77	7.69	1.61E-02	1.89E-01
11.51	11.34	11.49	1.61E-02	1.89E-01
10.83	10.92	10.95	1.62E-02	1.89E-01
9.30	9.19	9.35	1.62E-02	1.89E-01
8.10	7.99	8.02	1.62E-02	1.89E-01
6.97	6.83	6.99	1.62E-02	1.89E-01
9.25	9.34	9.37	1.62E-02	1.89E-01
7.31	7.26	7.49	1.62E-02	1.89E-01
7.90	7.66	7.92	1.62E-02	1.89E-01
10.56	10.43	10.62	1.62E-02	1.89E-01

8.34	8.39	8.35	1.63E-02	1.89E-01
11.61	11.73	11.71	1.63E-02	1.89E-01
7.22	7.10	7.32	1.63E-02	1.89E-01
5.73	5.54	5.63	1.63E-02	1.89E-01
9.25	9.21	9.37	1.63E-02	1.89E-01
4.29	4.26	4.28	1.64E-02	1.89E-01
7.28	7.39	7.48	1.64E-02	1.90E-01
6.05	5.90	5.87	1.64E-02	1.90E-01
3.75	3.80	3.65	1.64E-02	1.90E-01
4.42	4.40	4.40	1.65E-02	1.90E-01
8.86	8.92	8.78	1.65E-02	1.91E-01
7.62	7.76	7.67	1.65E-02	1.91E-01
10.02	10.00	10.08	1.65E-02	1.91E-01
14.61	14.60	14.63	1.66E-02	1.91E-01
7.28	7.30	7.35	1.66E-02	1.91E-01
6.94	6.95	6.94	1.66E-02	1.91E-01
7.31	7.31	7.40	1.66E-02	1.91E-01
6.47	6.46	6.55	1.67E-02	1.91E-01
10.34	10.21	10.29	1.67E-02	1.91E-01
6.18	6.31	6.13	1.67E-02	1.91E-01
8.67	8.76	8.74	1.67E-02	1.91E-01
9.19	9.22	9.18	1.67E-02	1.91E-01
4.39	4.43	4.53	1.67E-02	1.91E-01
8.49	8.52	8.49	1.67E-02	1.91E-01
5.16	5.29	5.12	1.67E-02	1.91E-01
7.41	7.26	7.34	1.67E-02	1.91E-01
8.07	8.05	8.03	1.68E-02	1.91E-01
4.77	4.72	4.66	1.68E-02	1.91E-01
5.59	5.94	5.65	1.68E-02	1.91E-01
5.68	5.53	5.38	1.68E-02	1.91E-01
6.54	6.74	6.37	1.68E-02	1.91E-01
10.43	10.52	10.50	1.68E-02	1.91E-01
9.89	10.14	10.00	1.68E-02	1.91E-01
5.97	5.91	6.01	1.68E-02	1.91E-01
5.72	5.70	5.71	1.68E-02	1.91E-01
5.37	5.26	5.41	1.68E-02	1.91E-01
5.21	5.22	5.26	1.69E-02	1.91E-01
6.68	6.74	6.45	1.69E-02	1.91E-01
12.30	12.27	12.19	1.69E-02	1.91E-01
11.01	11.13	11.14	1.69E-02	1.91E-01
3.58	3.51	3.47	1.69E-02	1.91E-01
6.87	6.81	6.76	1.69E-02	1.91E-01
10.27	10.34	10.37	1.69E-02	1.91E-01
6.75	6.63	6.62	1.69E-02	1.91E-01
10.56	10.74	10.53	1.69E-02	1.91E-01
8.57	8.41	8.59	1.69E-02	1.91E-01
8.37	8.41	8.42	1.69E-02	1.91E-01

8.39	8.50	8.50	1.69E-02	1.91E-01
11.39	11.55	11.53	1.70E-02	1.91E-01
7.85	7.97	7.79	1.70E-02	1.91E-01
7.70	7.96	7.87	1.71E-02	1.92E-01
4.76	4.83	5.01	1.71E-02	1.92E-01
6.25	6.41	6.34	1.71E-02	1.92E-01
7.94	8.08	7.95	1.71E-02	1.92E-01
6.46	6.44	6.57	1.71E-02	1.92E-01
9.38	9.36	9.27	1.71E-02	1.92E-01
9.01	8.98	8.95	1.72E-02	1.92E-01
10.35	10.36	10.26	1.72E-02	1.92E-01
5.53	5.32	5.37	1.72E-02	1.92E-01
12.65	12.52	12.51	1.72E-02	1.92E-01
10.23	10.20	10.19	1.73E-02	1.92E-01
3.31	3.25	3.33	1.73E-02	1.92E-01
9.27	9.26	9.26	1.73E-02	1.92E-01
7.42	7.55	7.66	1.73E-02	1.93E-01
10.88	11.02	10.98	1.73E-02	1.93E-01
8.64	8.57	8.56	1.73E-02	1.93E-01
8.81	8.91	9.01	1.74E-02	1.93E-01
7.98	7.97	7.94	1.74E-02	1.93E-01
11.47	11.41	11.38	1.74E-02	1.93E-01
7.98	8.23	8.08	1.74E-02	1.93E-01
6.18	6.08	5.99	1.74E-02	1.93E-01
10.38	10.46	10.49	1.74E-02	1.93E-01
7.79	7.81	7.77	1.74E-02	1.93E-01
5.30	5.12	5.07	1.74E-02	1.93E-01
4.32	4.40	4.40	1.75E-02	1.93E-01
9.86	9.95	9.98	1.75E-02	1.93E-01
8.18	8.05	8.18	1.75E-02	1.93E-01
6.20	6.29	6.24	1.75E-02	1.93E-01
10.07	9.98	10.06	1.76E-02	1.94E-01
7.58	7.48	7.46	1.76E-02	1.94E-01
8.97	9.10	9.07	1.76E-02	1.94E-01
9.83	9.83	9.79	1.77E-02	1.94E-01
8.90	8.97	9.05	1.77E-02	1.95E-01
3.20	3.15	3.18	1.78E-02	1.95E-01
4.20	4.36	4.76	1.78E-02	1.95E-01
11.68	11.71	11.61	1.78E-02	1.95E-01
10.49	10.63	10.61	1.78E-02	1.95E-01
7.97	8.16	8.09	1.78E-02	1.95E-01
9.01	9.24	9.05	1.79E-02	1.95E-01
8.05	8.15	8.20	1.79E-02	1.95E-01
7.73	7.73	7.75	1.79E-02	1.95E-01
6.06	6.10	6.03	1.79E-02	1.95E-01
5.93	6.31	6.27	1.79E-02	1.95E-01
7.19	7.21	7.14	1.79E-02	1.95E-01

6.02	6.36	6.04	1.79E-02	1.95E-01
4.02	3.78	3.87	1.80E-02	1.96E-01
5.46	5.50	5.44	1.80E-02	1.96E-01
7.44	7.28	7.38	1.80E-02	1.96E-01
10.10	10.18	10.08	1.80E-02	1.96E-01
9.93	10.03	9.94	1.81E-02	1.96E-01
4.87	4.95	4.86	1.81E-02	1.96E-01
6.08	6.03	6.02	1.81E-02	1.96E-01
8.26	8.39	8.29	1.81E-02	1.96E-01
11.20	11.26	11.18	1.81E-02	1.96E-01
8.52	8.82	8.72	1.82E-02	1.96E-01
8.45	8.66	8.57	1.82E-02	1.96E-01
8.54	8.65	8.56	1.82E-02	1.96E-01
9.99	10.28	10.25	1.82E-02	1.96E-01
9.47	9.73	9.58	1.82E-02	1.96E-01
6.94	7.14	7.31	1.82E-02	1.96E-01
10.37	10.58	10.42	1.82E-02	1.97E-01
6.78	6.88	6.60	1.83E-02	1.97E-01
11.52	11.41	11.43	1.83E-02	1.97E-01
5.75	5.66	5.85	1.83E-02	1.97E-01
6.67	6.50	6.60	1.83E-02	1.97E-01
9.04	9.11	9.22	1.83E-02	1.97E-01
4.74	4.96	4.90	1.84E-02	1.97E-01
6.86	6.79	6.66	1.84E-02	1.97E-01
8.68	8.71	8.73	1.84E-02	1.97E-01
10.94	10.96	10.93	1.84E-02	1.97E-01
9.05	9.25	9.28	1.84E-02	1.97E-01
6.57	6.48	6.55	1.85E-02	1.98E-01
10.36	10.45	10.48	1.85E-02	1.98E-01
8.94	9.07	9.02	1.85E-02	1.98E-01
11.58	11.35	11.41	1.85E-02	1.98E-01
7.46	7.73	7.71	1.85E-02	1.98E-01
10.57	10.60	10.48	1.86E-02	1.98E-01
4.12	4.18	4.24	1.86E-02	1.98E-01
10.66	10.76	10.62	1.86E-02	1.98E-01
10.74	10.79	10.74	1.86E-02	1.98E-01
9.92	9.94	9.94	1.87E-02	1.98E-01
7.96	8.16	8.17	1.87E-02	1.98E-01
8.37	8.30	8.31	1.87E-02	1.98E-01
8.51	8.56	8.63	1.87E-02	1.98E-01
7.81	7.68	7.81	1.87E-02	1.98E-01
6.73	6.59	6.78	1.88E-02	1.99E-01
7.34	7.34	7.38	1.88E-02	1.99E-01
7.60	7.87	7.75	1.88E-02	1.99E-01
9.46	9.34	9.48	1.88E-02	1.99E-01
10.46	10.15	10.48	1.89E-02	2.00E-01
9.98	10.15	10.03	1.89E-02	2.00E-01

10.94	10.99	11.03	1.89E-02	2.00E-01
4.24	4.01	4.21	1.89E-02	2.00E-01
7.19	7.33	7.34	1.89E-02	2.00E-01
14.64	14.69	14.69	1.90E-02	2.00E-01
13.20	12.98	13.21	1.90E-02	2.00E-01
5.64	5.61	5.56	1.90E-02	2.00E-01
9.95	9.83	9.98	1.90E-02	2.00E-01
10.09	9.95	10.12	1.90E-02	2.00E-01
7.79	7.90	7.95	1.90E-02	2.00E-01
8.92	8.57	8.88	1.91E-02	2.00E-01
5.00	4.93	4.84	1.91E-02	2.00E-01
5.90	5.74	5.96	1.91E-02	2.01E-01
6.86	6.87	6.82	1.92E-02	2.01E-01
9.40	9.50	9.46	1.92E-02	2.01E-01
4.66	4.57	4.46	1.92E-02	2.01E-01
9.69	9.45	9.73	1.92E-02	2.01E-01
8.23	8.41	8.37	1.92E-02	2.01E-01
9.79	9.93	9.83	1.92E-02	2.01E-01
9.16	8.88	8.92	1.92E-02	2.01E-01
7.23	7.10	7.16	1.92E-02	2.01E-01
9.79	9.94	9.87	1.93E-02	2.01E-01
6.86	6.70	6.74	1.93E-02	2.01E-01
11.65	11.78	11.73	1.93E-02	2.01E-01
7.82	7.63	7.77	1.93E-02	2.01E-01
6.74	6.74	6.86	1.93E-02	2.01E-01
12.39	12.46	12.47	1.93E-02	2.01E-01
10.23	10.40	10.31	1.94E-02	2.01E-01
10.92	11.09	10.91	1.94E-02	2.01E-01
8.09	8.13	8.14	1.94E-02	2.01E-01
7.70	7.97	7.87	1.94E-02	2.01E-01
9.64	9.69	9.64	1.95E-02	2.02E-01
8.75	8.74	8.75	1.95E-02	2.02E-01
7.49	7.64	7.61	1.95E-02	2.02E-01
11.28	11.26	11.34	1.96E-02	2.03E-01
7.67	7.61	7.68	1.96E-02	2.03E-01
10.69	10.52	10.73	1.96E-02	2.03E-01
8.75	8.98	8.95	1.96E-02	2.03E-01
11.85	11.93	11.98	1.97E-02	2.03E-01
7.57	7.31	7.47	1.97E-02	2.03E-01
5.59	5.65	5.65	1.97E-02	2.03E-01
7.22	7.30	7.04	1.97E-02	2.03E-01
8.19	8.46	8.34	1.98E-02	2.03E-01
3.55	3.72	3.83	1.98E-02	2.03E-01
9.18	9.21	9.18	1.98E-02	2.03E-01
10.30	10.23	10.26	1.98E-02	2.03E-01
6.39	6.51	6.58	1.98E-02	2.03E-01
8.05	8.15	8.30	1.98E-02	2.03E-01

5.09	5.11	5.26	1.98E-02	2.03E-01
7.80	7.97	7.86	1.98E-02	2.03E-01
4.83	4.64	4.63	1.99E-02	2.03E-01
10.54	10.56	10.54	1.99E-02	2.03E-01
9.51	9.45	9.54	1.99E-02	2.03E-01
8.26	8.23	8.23	1.99E-02	2.04E-01
10.01	10.10	10.14	2.00E-02	2.04E-01
8.50	8.35	8.32	2.00E-02	2.04E-01
6.29	6.52	6.49	2.00E-02	2.04E-01
4.69	4.49	4.55	2.01E-02	2.05E-01
7.72	7.95	7.79	2.01E-02	2.05E-01
10.22	10.05	10.12	2.01E-02	2.05E-01
6.47	6.77	6.49	2.01E-02	2.05E-01
3.19	3.23	3.20	2.01E-02	2.05E-01
4.75	4.74	4.74	2.02E-02	2.05E-01
8.61	8.88	8.82	2.02E-02	2.05E-01
5.84	5.79	5.80	2.02E-02	2.05E-01
8.34	8.63	8.53	2.02E-02	2.05E-01
10.97	10.94	10.91	2.02E-02	2.05E-01
4.05	3.89	4.06	2.02E-02	2.05E-01
9.13	9.17	9.13	2.03E-02	2.05E-01
10.17	10.17	10.24	2.03E-02	2.05E-01
4.56	4.74	4.79	2.03E-02	2.05E-01
4.59	4.75	4.83	2.03E-02	2.05E-01
5.63	5.60	5.63	2.03E-02	2.05E-01
8.62	8.38	8.76	2.03E-02	2.05E-01
5.87	5.82	5.73	2.04E-02	2.05E-01
8.36	8.61	8.45	2.04E-02	2.05E-01
6.08	5.83	6.16	2.04E-02	2.05E-01
5.64	5.71	5.77	2.04E-02	2.05E-01
9.56	9.56	9.36	2.04E-02	2.05E-01
5.77	6.01	5.78	2.04E-02	2.05E-01
7.39	7.35	7.43	2.04E-02	2.05E-01
6.85	6.91	6.94	2.04E-02	2.05E-01
8.16	8.15	8.09	2.04E-02	2.05E-01
6.63	6.61	6.54	2.05E-02	2.05E-01
5.13	5.42	5.21	2.05E-02	2.05E-01
9.83	9.84	9.96	2.05E-02	2.05E-01
13.40	13.39	13.52	2.05E-02	2.05E-01
8.48	8.52	8.37	2.05E-02	2.05E-01
9.83	9.78	9.81	2.05E-02	2.05E-01
4.58	4.60	4.61	2.05E-02	2.05E-01
7.79	7.82	7.80	2.05E-02	2.05E-01
7.72	7.84	7.87	2.05E-02	2.05E-01
7.50	7.42	7.46	2.06E-02	2.05E-01
8.75	8.68	8.80	2.06E-02	2.05E-01
10.21	10.29	10.27	2.06E-02	2.05E-01

6.86	7.21	6.94	2.06E-02	2.05E-01
10.41	10.59	10.47	2.06E-02	2.05E-01
8.48	8.44	8.45	2.07E-02	2.06E-01
10.31	10.47	10.34	2.07E-02	2.06E-01
6.17	6.34	6.28	2.07E-02	2.06E-01
5.72	6.06	5.73	2.08E-02	2.06E-01
9.98	9.70	10.11	2.08E-02	2.06E-01
9.67	9.73	9.69	2.08E-02	2.06E-01
7.20	7.19	7.29	2.08E-02	2.06E-01
8.93	9.16	9.02	2.08E-02	2.06E-01
7.31	7.58	7.41	2.08E-02	2.06E-01
9.32	9.58	9.40	2.09E-02	2.06E-01
7.31	7.48	7.40	2.09E-02	2.06E-01
9.01	9.16	9.21	2.09E-02	2.06E-01
7.92	7.95	8.21	2.09E-02	2.06E-01
8.74	8.65	8.82	2.09E-02	2.06E-01
4.92	4.84	4.85	2.09E-02	2.06E-01
9.87	9.82	9.92	2.10E-02	2.06E-01
3.28	3.32	3.12	2.10E-02	2.06E-01
9.20	9.28	9.31	2.10E-02	2.06E-01
11.43	11.36	11.35	2.10E-02	2.06E-01
6.46	6.65	6.31	2.11E-02	2.07E-01
8.05	8.23	8.22	2.11E-02	2.07E-01
8.33	8.11	8.29	2.11E-02	2.08E-01
10.38	10.32	10.48	2.11E-02	2.08E-01
12.19	12.18	12.15	2.11E-02	2.08E-01
7.26	7.20	7.40	2.12E-02	2.08E-01
10.57	10.78	10.53	2.12E-02	2.08E-01
6.85	6.70	7.16	2.12E-02	2.08E-01
6.95	6.98	7.11	2.13E-02	2.08E-01
8.63	8.64	8.51	2.13E-02	2.08E-01
10.73	10.93	10.83	2.13E-02	2.08E-01
6.28	6.28	6.31	2.13E-02	2.08E-01
5.17	5.24	5.27	2.13E-02	2.08E-01
6.13	6.18	6.17	2.13E-02	2.08E-01
7.13	7.22	7.23	2.13E-02	2.08E-01
6.96	7.21	6.83	2.13E-02	2.08E-01
9.36	9.51	9.34	2.13E-02	2.08E-01
3.36	3.52	3.42	2.13E-02	2.08E-01
7.84	8.03	7.98	2.13E-02	2.08E-01
7.29	7.36	7.34	2.14E-02	2.08E-01
9.14	9.35	9.27	2.14E-02	2.08E-01
6.39	6.56	6.23	2.14E-02	2.08E-01
7.40	7.46	7.65	2.15E-02	2.08E-01
7.86	7.65	7.95	2.15E-02	2.08E-01
6.97	7.18	6.92	2.15E-02	2.08E-01
9.84	9.67	9.73	2.15E-02	2.08E-01

8.84	8.95	9.02	2.15E-02	2.08E-01
12.70	12.58	12.69	2.15E-02	2.08E-01
3.43	3.39	3.36	2.15E-02	2.08E-01
7.29	7.51	7.39	2.15E-02	2.08E-01
5.55	5.34	5.48	2.15E-02	2.08E-01
7.89	7.84	7.92	2.15E-02	2.08E-01
7.17	7.12	7.21	2.16E-02	2.08E-01
8.05	8.05	8.10	2.16E-02	2.08E-01
7.15	7.16	7.17	2.17E-02	2.09E-01
5.83	5.95	5.91	2.17E-02	2.09E-01
7.62	7.82	7.76	2.17E-02	2.09E-01
3.69	3.69	3.49	2.17E-02	2.09E-01
10.44	10.52	10.50	2.18E-02	2.09E-01
5.82	6.01	5.96	2.18E-02	2.09E-01
5.33	5.28	5.32	2.18E-02	2.09E-01
7.34	7.35	7.42	2.18E-02	2.09E-01
7.52	7.57	7.39	2.18E-02	2.09E-01
14.22	14.19	14.21	2.19E-02	2.10E-01
6.08	6.11	6.02	2.19E-02	2.10E-01
10.42	10.58	10.53	2.19E-02	2.10E-01
6.78	6.60	6.67	2.19E-02	2.10E-01
7.80	7.75	7.98	2.19E-02	2.10E-01
8.93	9.01	9.06	2.19E-02	2.10E-01
10.62	10.53	10.58	2.19E-02	2.10E-01
4.47	4.57	4.43	2.19E-02	2.10E-01
12.37	12.27	12.38	2.20E-02	2.10E-01
11.09	11.09	11.02	2.20E-02	2.10E-01
3.84	3.70	3.80	2.20E-02	2.10E-01
8.25	8.31	8.35	2.20E-02	2.10E-01
5.46	5.36	5.50	2.20E-02	2.10E-01
8.83	8.97	8.86	2.21E-02	2.10E-01
6.14	6.02	5.86	2.21E-02	2.10E-01
10.88	11.07	10.94	2.21E-02	2.10E-01
5.05	5.08	5.12	2.22E-02	2.11E-01
3.29	3.21	3.21	2.22E-02	2.11E-01
8.58	8.70	8.66	2.23E-02	2.11E-01
12.23	12.25	12.19	2.23E-02	2.11E-01
9.62	9.68	9.72	2.23E-02	2.11E-01
6.31	6.45	6.29	2.23E-02	2.11E-01
6.12	6.27	6.05	2.23E-02	2.11E-01
7.57	7.50	7.52	2.23E-02	2.11E-01
8.74	8.71	8.72	2.23E-02	2.11E-01
4.82	4.89	4.82	2.23E-02	2.11E-01
5.81	5.57	5.75	2.24E-02	2.11E-01
4.64	4.85	4.78	2.24E-02	2.11E-01
4.87	4.90	4.79	2.24E-02	2.11E-01
8.82	8.92	8.86	2.24E-02	2.11E-01

9.16	9.27	9.28	2.24E-02	2.11E-01
10.08	9.96	10.07	2.24E-02	2.11E-01
8.71	8.72	8.72	2.24E-02	2.11E-01
8.18	8.27	8.33	2.25E-02	2.11E-01
12.18	12.28	12.28	2.25E-02	2.11E-01
4.26	4.29	4.31	2.25E-02	2.11E-01
4.33	4.43	4.33	2.25E-02	2.11E-01
9.18	9.26	9.32	2.25E-02	2.11E-01
8.61	8.58	8.54	2.25E-02	2.11E-01
3.70	3.48	3.69	2.26E-02	2.12E-01
11.78	11.65	11.89	2.26E-02	2.12E-01
8.14	8.16	8.19	2.26E-02	2.12E-01
8.90	8.96	8.95	2.26E-02	2.12E-01
6.42	6.31	6.31	2.27E-02	2.12E-01
8.03	7.80	7.90	2.27E-02	2.12E-01
10.65	10.74	10.77	2.27E-02	2.12E-01
11.06	11.19	11.01	2.27E-02	2.12E-01
6.34	6.18	6.43	2.27E-02	2.12E-01
9.51	9.59	9.52	2.27E-02	2.12E-01
10.60	10.75	10.68	2.27E-02	2.12E-01
7.14	7.11	7.15	2.27E-02	2.12E-01
6.85	6.88	7.15	2.28E-02	2.12E-01
7.40	7.37	7.33	2.28E-02	2.12E-01
8.84	8.72	8.92	2.28E-02	2.12E-01
8.49	8.36	8.77	2.28E-02	2.12E-01
5.84	5.98	5.87	2.28E-02	2.12E-01
7.29	7.31	7.40	2.29E-02	2.12E-01
8.13	8.05	8.26	2.29E-02	2.12E-01
7.90	8.04	8.22	2.29E-02	2.12E-01
9.20	9.07	9.10	2.29E-02	2.12E-01
4.04	4.12	4.00	2.29E-02	2.12E-01
8.92	9.01	9.03	2.29E-02	2.12E-01
10.07	10.33	10.26	2.29E-02	2.12E-01
5.08	5.16	4.85	2.29E-02	2.12E-01
5.76	5.62	5.61	2.29E-02	2.12E-01
9.34	9.32	9.38	2.30E-02	2.12E-01
12.44	12.31	12.59	2.30E-02	2.12E-01
12.63	12.50	12.57	2.30E-02	2.12E-01
9.62	9.80	9.60	2.30E-02	2.12E-01
8.48	8.47	8.44	2.30E-02	2.12E-01
14.84	14.82	14.83	2.30E-02	2.12E-01
7.58	7.66	7.68	2.30E-02	2.12E-01
8.63	8.70	8.63	2.31E-02	2.12E-01
8.07	8.25	8.11	2.31E-02	2.12E-01
7.80	8.17	8.08	2.31E-02	2.12E-01
5.25	5.30	5.25	2.31E-02	2.12E-01
10.36	10.24	10.37	2.31E-02	2.12E-01

7.63	7.57	7.68	2.31E-02	2.12E-01
12.55	12.40	12.48	2.31E-02	2.12E-01
9.62	9.74	9.70	2.31E-02	2.12E-01
15.09	15.11	15.09	2.32E-02	2.12E-01
7.41	7.54	7.49	2.32E-02	2.12E-01
3.24	3.34	3.28	2.32E-02	2.12E-01
8.88	8.92	8.72	2.32E-02	2.12E-01
6.64	6.58	6.45	2.32E-02	2.12E-01
8.24	8.29	8.32	2.32E-02	2.12E-01
10.58	10.70	10.64	2.33E-02	2.12E-01
6.58	6.37	6.52	2.33E-02	2.12E-01
6.87	6.71	6.84	2.33E-02	2.12E-01
8.23	8.32	8.31	2.33E-02	2.12E-01
7.69	7.79	7.76	2.33E-02	2.12E-01
10.05	9.96	10.11	2.34E-02	2.12E-01
5.93	5.97	5.81	2.34E-02	2.12E-01
9.72	9.77	9.82	2.34E-02	2.12E-01
3.48	3.29	3.58	2.34E-02	2.12E-01
9.73	9.81	9.79	2.35E-02	2.12E-01
7.64	7.52	7.68	2.35E-02	2.12E-01
3.48	3.43	3.43	2.35E-02	2.12E-01
7.33	7.13	7.15	2.35E-02	2.12E-01
9.64	9.41	9.61	2.35E-02	2.12E-01
4.22	4.27	4.35	2.35E-02	2.12E-01
11.64	11.70	11.69	2.35E-02	2.12E-01
7.48	7.52	7.54	2.35E-02	2.12E-01
9.31	9.57	9.50	2.35E-02	2.12E-01
6.54	6.66	6.68	2.35E-02	2.12E-01
7.08	6.99	7.06	2.36E-02	2.12E-01
11.25	11.36	11.28	2.36E-02	2.12E-01
11.55	11.58	11.61	2.36E-02	2.12E-01
11.88	11.94	11.87	2.36E-02	2.12E-01
6.29	6.50	6.45	2.36E-02	2.12E-01
5.83	6.00	5.93	2.36E-02	2.13E-01
7.21	7.13	7.36	2.37E-02	2.13E-01
4.04	4.12	3.94	2.37E-02	2.13E-01
7.76	7.74	7.79	2.37E-02	2.13E-01
10.32	10.15	10.22	2.37E-02	2.13E-01
7.16	7.13	7.33	2.37E-02	2.13E-01
6.10	6.22	6.30	2.37E-02	2.13E-01
7.97	7.79	7.78	2.37E-02	2.13E-01
10.31	10.10	10.29	2.37E-02	2.13E-01
9.00	9.07	9.05	2.37E-02	2.13E-01
9.47	9.67	9.62	2.38E-02	2.13E-01
7.67	7.77	7.85	2.38E-02	2.13E-01
9.83	9.94	9.92	2.38E-02	2.13E-01
6.70	6.87	7.03	2.38E-02	2.13E-01

3.95	3.90	3.97	2.39E-02	2.13E-01
6.88	7.02	7.01	2.39E-02	2.14E-01
7.99	8.17	7.95	2.39E-02	2.14E-01
9.19	9.03	9.23	2.40E-02	2.14E-01
6.77	6.64	6.68	2.40E-02	2.14E-01
7.12	7.32	7.11	2.40E-02	2.14E-01
9.92	9.90	9.91	2.40E-02	2.14E-01
6.56	6.46	6.50	2.40E-02	2.14E-01
7.88	7.87	8.03	2.41E-02	2.14E-01
8.37	8.27	8.32	2.41E-02	2.14E-01
8.35	8.28	8.30	2.41E-02	2.14E-01
7.58	7.70	7.60	2.41E-02	2.14E-01
4.33	4.48	4.36	2.41E-02	2.14E-01
6.44	6.61	6.50	2.42E-02	2.15E-01
10.64	10.73	10.64	2.42E-02	2.15E-01
6.18	5.95	6.10	2.42E-02	2.15E-01
9.68	9.71	9.73	2.42E-02	2.15E-01
5.55	5.49	5.51	2.43E-02	2.15E-01
9.52	9.60	9.63	2.43E-02	2.15E-01
10.88	11.05	11.00	2.43E-02	2.15E-01
10.76	10.68	10.65	2.44E-02	2.15E-01
6.42	6.64	6.35	2.44E-02	2.15E-01
6.50	6.52	6.81	2.44E-02	2.16E-01
5.82	5.77	5.87	2.44E-02	2.16E-01
6.60	6.55	6.63	2.45E-02	2.16E-01
3.85	3.88	3.97	2.45E-02	2.16E-01
3.52	3.62	3.62	2.45E-02	2.16E-01
13.76	13.56	13.86	2.45E-02	2.16E-01
7.74	7.73	7.68	2.45E-02	2.16E-01
7.59	7.47	7.60	2.45E-02	2.16E-01
8.79	8.44	8.42	2.45E-02	2.16E-01
11.86	11.87	11.87	2.46E-02	2.16E-01
7.78	7.86	7.84	2.46E-02	2.16E-01
13.00	13.11	13.05	2.46E-02	2.16E-01
9.23	9.17	9.29	2.47E-02	2.17E-01
4.34	4.51	4.50	2.47E-02	2.17E-01
8.73	8.58	8.72	2.47E-02	2.17E-01
8.25	8.31	8.37	2.47E-02	2.17E-01
9.02	8.86	9.07	2.48E-02	2.17E-01
7.88	7.89	7.96	2.48E-02	2.17E-01
4.80	4.62	4.71	2.48E-02	2.17E-01
4.30	4.46	4.27	2.48E-02	2.17E-01
6.42	6.33	6.65	2.48E-02	2.17E-01
10.03	9.96	9.99	2.48E-02	2.17E-01
8.67	8.89	8.79	2.48E-02	2.17E-01
6.39	6.38	6.35	2.48E-02	2.17E-01
6.24	6.09	6.14	2.49E-02	2.17E-01

10.07	9.94	9.90	2.49E-02	2.17E-01
5.11	5.24	5.12	2.49E-02	2.17E-01
5.10	5.09	5.10	2.49E-02	2.17E-01
7.41	7.48	7.61	2.50E-02	2.17E-01
10.67	10.83	10.73	2.50E-02	2.17E-01
11.13	11.20	11.14	2.50E-02	2.17E-01
11.80	11.91	11.78	2.50E-02	2.17E-01
9.79	9.73	9.80	2.50E-02	2.17E-01
14.60	14.68	14.66	2.51E-02	2.17E-01
8.70	8.59	8.73	2.51E-02	2.17E-01
3.38	3.48	3.37	2.51E-02	2.17E-01
8.22	8.10	8.24	2.51E-02	2.17E-01
9.42	9.31	9.44	2.51E-02	2.17E-01
4.61	4.47	4.57	2.51E-02	2.18E-01
6.84	6.79	6.76	2.51E-02	2.18E-01
13.98	13.96	13.92	2.52E-02	2.18E-01
7.62	7.80	7.63	2.52E-02	2.18E-01
9.66	9.90	9.71	2.53E-02	2.18E-01
8.04	8.24	8.07	2.53E-02	2.18E-01
7.29	7.14	7.17	2.53E-02	2.18E-01
9.50	9.28	9.55	2.53E-02	2.18E-01
7.08	7.08	7.01	2.53E-02	2.18E-01
9.67	9.73	9.76	2.54E-02	2.18E-01
11.17	11.19	11.42	2.54E-02	2.18E-01
6.48	6.53	6.42	2.54E-02	2.18E-01
9.36	9.23	9.37	2.54E-02	2.18E-01
8.10	8.12	8.00	2.54E-02	2.18E-01
11.02	10.97	11.10	2.54E-02	2.18E-01
8.22	8.13	8.23	2.54E-02	2.18E-01
9.34	9.29	9.17	2.55E-02	2.18E-01
7.04	7.28	7.20	2.55E-02	2.18E-01
9.68	9.75	9.78	2.55E-02	2.18E-01
10.15	10.28	10.16	2.55E-02	2.18E-01
10.45	10.70	10.52	2.55E-02	2.18E-01
10.28	10.24	10.32	2.55E-02	2.18E-01
5.89	5.68	5.80	2.55E-02	2.18E-01
9.32	9.41	9.40	2.56E-02	2.19E-01
6.02	6.04	6.04	2.56E-02	2.19E-01
9.64	9.75	9.65	2.56E-02	2.19E-01
3.71	3.51	3.65	2.56E-02	2.19E-01
8.20	8.25	8.28	2.56E-02	2.19E-01
8.92	9.00	8.95	2.57E-02	2.19E-01
7.00	7.00	7.06	2.57E-02	2.19E-01
6.55	6.66	6.68	2.57E-02	2.19E-01
8.42	8.59	8.51	2.58E-02	2.20E-01
5.80	5.72	5.97	2.58E-02	2.20E-01
3.70	3.73	3.75	2.58E-02	2.20E-01

12.34	12.30	12.30	2.58E-02	2.20E-01
8.30	8.31	8.39	2.59E-02	2.20E-01
7.19	7.56	7.14	2.59E-02	2.20E-01
4.67	4.67	4.63	2.59E-02	2.20E-01
6.49	6.46	6.81	2.59E-02	2.20E-01
7.97	8.06	8.05	2.60E-02	2.20E-01
4.57	4.51	4.55	2.60E-02	2.20E-01
12.62	12.82	12.66	2.61E-02	2.20E-01
7.45	7.65	7.59	2.61E-02	2.20E-01
11.52	11.60	11.55	2.61E-02	2.20E-01
4.42	4.57	4.45	2.61E-02	2.20E-01
6.14	5.92	6.29	2.61E-02	2.20E-01
7.94	7.68	7.87	2.61E-02	2.20E-01
4.48	4.69	4.40	2.62E-02	2.20E-01
10.98	11.15	11.09	2.62E-02	2.20E-01
6.73	6.72	6.79	2.62E-02	2.20E-01
6.73	6.59	6.74	2.62E-02	2.20E-01
4.58	4.74	4.46	2.62E-02	2.20E-01
4.51	4.22	4.64	2.62E-02	2.20E-01
8.90	9.09	9.06	2.62E-02	2.20E-01
5.41	5.52	5.57	2.62E-02	2.20E-01
5.04	4.96	5.12	2.62E-02	2.21E-01
8.41	8.40	8.52	2.62E-02	2.21E-01
6.84	6.88	6.95	2.63E-02	2.21E-01
8.41	8.45	8.49	2.63E-02	2.21E-01
3.54	3.43	3.44	2.63E-02	2.21E-01
5.69	5.74	5.70	2.64E-02	2.21E-01
10.53	10.50	10.69	2.64E-02	2.21E-01
9.48	9.45	9.52	2.64E-02	2.21E-01
6.59	6.88	6.85	2.64E-02	2.21E-01
4.71	4.71	4.61	2.65E-02	2.21E-01
11.90	11.95	12.11	2.65E-02	2.21E-01
5.11	5.17	5.26	2.65E-02	2.21E-01
11.71	11.68	11.67	2.65E-02	2.21E-01
4.01	3.81	3.85	2.65E-02	2.21E-01
4.25	4.27	4.29	2.65E-02	2.21E-01
4.53	4.33	4.24	2.65E-02	2.21E-01
7.33	7.37	7.29	2.65E-02	2.21E-01
4.24	4.23	4.27	2.66E-02	2.21E-01
9.53	9.57	9.60	2.66E-02	2.21E-01
4.39	4.27	4.25	2.66E-02	2.21E-01
3.81	3.63	3.76	2.67E-02	2.22E-01
5.00	5.04	5.15	2.67E-02	2.22E-01
8.52	8.62	8.72	2.67E-02	2.22E-01
4.95	4.93	4.82	2.67E-02	2.22E-01
8.29	8.30	8.28	2.68E-02	2.22E-01
7.25	7.27	7.09	2.68E-02	2.22E-01

7.91	7.97	7.95	2.68E-02	2.22E-01
6.15	6.07	6.13	2.68E-02	2.22E-01
6.98	7.02	7.02	2.68E-02	2.22E-01
10.37	10.42	10.50	2.69E-02	2.22E-01
7.56	7.45	7.58	2.69E-02	2.22E-01
10.01	9.99	10.10	2.69E-02	2.22E-01
4.79	5.44	5.08	2.69E-02	2.23E-01
8.17	8.03	8.40	2.69E-02	2.23E-01
6.43	6.28	6.47	2.70E-02	2.23E-01
6.85	7.01	6.92	2.70E-02	2.23E-01
4.30	4.32	4.28	2.71E-02	2.24E-01
9.46	9.38	9.55	2.71E-02	2.24E-01
5.21	5.19	5.18	2.71E-02	2.24E-01
6.06	6.22	6.24	2.72E-02	2.24E-01
7.09	7.08	7.09	2.72E-02	2.24E-01
10.88	10.71	10.72	2.72E-02	2.24E-01
13.02	13.02	13.03	2.72E-02	2.24E-01
7.68	8.03	7.80	2.72E-02	2.24E-01
6.45	6.56	6.46	2.73E-02	2.24E-01
4.66	4.75	4.80	2.73E-02	2.24E-01
10.33	10.30	10.47	2.73E-02	2.24E-01
11.09	11.11	11.06	2.74E-02	2.25E-01
9.88	9.84	9.98	2.74E-02	2.25E-01
7.90	8.06	8.15	2.75E-02	2.26E-01
8.31	8.50	8.39	2.75E-02	2.26E-01
4.35	4.43	4.42	2.76E-02	2.26E-01
7.86	7.90	8.02	2.76E-02	2.26E-01
3.30	3.21	3.34	2.76E-02	2.26E-01
5.22	5.15	5.33	2.76E-02	2.26E-01
10.50	10.50	10.51	2.76E-02	2.26E-01
8.72	8.42	8.85	2.76E-02	2.26E-01
8.69	8.75	8.83	2.77E-02	2.26E-01
11.15	11.20	11.27	2.77E-02	2.26E-01
5.97	5.97	5.99	2.77E-02	2.26E-01
8.53	8.62	8.67	2.77E-02	2.26E-01
10.92	10.70	10.82	2.77E-02	2.26E-01
8.31	8.50	8.30	2.77E-02	2.26E-01
4.13	4.16	4.30	2.78E-02	2.26E-01
8.49	8.70	8.66	2.78E-02	2.26E-01
8.57	8.65	8.55	2.78E-02	2.26E-01
7.75	7.71	7.77	2.78E-02	2.26E-01
11.11	11.21	11.21	2.78E-02	2.26E-01
7.34	7.30	7.44	2.78E-02	2.26E-01
6.32	6.26	6.21	2.78E-02	2.26E-01
8.63	8.75	8.57	2.79E-02	2.26E-01
10.40	10.48	10.43	2.79E-02	2.26E-01
8.50	8.73	8.53	2.79E-02	2.26E-01

5.84	5.98	5.98	2.79E-02	2.26E-01
6.74	6.68	6.68	2.80E-02	2.26E-01
6.35	6.23	6.23	2.80E-02	2.26E-01
7.31	7.35	7.08	2.81E-02	2.27E-01
4.63	4.54	4.70	2.82E-02	2.28E-01
7.18	6.95	7.47	2.83E-02	2.28E-01
8.93	9.17	8.98	2.83E-02	2.28E-01
13.04	13.11	13.10	2.83E-02	2.28E-01
7.22	7.58	7.21	2.83E-02	2.29E-01
3.90	3.80	3.49	2.83E-02	2.29E-01
5.33	5.33	5.33	2.83E-02	2.29E-01
5.96	6.12	5.37	2.84E-02	2.29E-01
4.73	4.59	4.71	2.84E-02	2.29E-01
7.20	7.14	6.95	2.84E-02	2.29E-01
12.38	12.44	12.45	2.84E-02	2.29E-01
11.90	12.08	11.99	2.85E-02	2.29E-01
14.21	14.16	14.16	2.85E-02	2.29E-01
8.77	8.84	8.77	2.85E-02	2.29E-01
3.39	3.43	3.33	2.85E-02	2.29E-01
14.43	14.42	14.43	2.85E-02	2.29E-01
6.52	6.52	6.42	2.85E-02	2.29E-01
6.90	6.96	6.81	2.85E-02	2.29E-01
9.35	9.56	9.51	2.85E-02	2.29E-01
6.13	6.43	6.25	2.85E-02	2.29E-01
10.71	10.78	10.73	2.86E-02	2.29E-01
11.25	11.33	11.31	2.86E-02	2.29E-01
8.49	8.56	8.61	2.86E-02	2.29E-01
5.00	4.74	4.95	2.86E-02	2.29E-01
4.71	4.62	4.72	2.87E-02	2.29E-01
10.12	10.09	10.08	2.87E-02	2.29E-01
5.71	5.70	5.93	2.87E-02	2.29E-01
9.84	9.91	9.97	2.88E-02	2.29E-01
6.55	6.94	6.58	2.88E-02	2.29E-01
9.47	9.55	9.53	2.88E-02	2.29E-01
5.73	5.74	5.51	2.88E-02	2.29E-01
6.11	6.30	6.21	2.88E-02	2.29E-01
9.74	9.79	9.93	2.88E-02	2.30E-01
6.40	6.48	6.49	2.89E-02	2.30E-01
8.16	8.24	8.23	2.89E-02	2.30E-01
10.48	10.66	10.55	2.89E-02	2.30E-01
7.62	7.77	7.70	2.89E-02	2.30E-01
4.79	4.82	4.67	2.89E-02	2.30E-01
5.78	5.94	5.99	2.89E-02	2.30E-01
5.88	5.90	5.88	2.89E-02	2.30E-01
3.93	4.07	4.02	2.89E-02	2.30E-01
9.04	8.96	9.04	2.90E-02	2.30E-01
5.24	5.20	5.48	2.90E-02	2.30E-01

9.36	9.64	9.56	2.90E-02	2.30E-01
7.31	7.33	7.38	2.90E-02	2.30E-01
5.94	6.26	5.96	2.90E-02	2.30E-01
10.14	10.68	10.23	2.91E-02	2.30E-01
8.02	8.16	8.14	2.91E-02	2.30E-01
7.37	7.22	7.33	2.91E-02	2.30E-01
8.83	8.85	8.97	2.91E-02	2.30E-01
7.03	7.13	7.24	2.92E-02	2.30E-01
3.42	3.42	3.47	2.92E-02	2.30E-01
7.12	7.10	7.11	2.92E-02	2.30E-01
7.38	7.30	7.69	2.92E-02	2.30E-01
8.45	8.60	8.42	2.92E-02	2.30E-01
10.97	10.96	10.91	2.92E-02	2.30E-01
9.62	9.78	9.78	2.93E-02	2.30E-01
4.25	4.15	4.12	2.93E-02	2.31E-01
8.93	8.91	8.94	2.94E-02	2.31E-01
3.85	3.60	3.58	2.94E-02	2.31E-01
12.70	12.58	12.68	2.94E-02	2.31E-01
7.67	7.83	7.87	2.94E-02	2.31E-01
9.73	9.54	9.91	2.95E-02	2.31E-01
7.46	7.45	7.52	2.95E-02	2.31E-01
11.85	12.01	11.99	2.95E-02	2.31E-01
8.32	8.30	8.46	2.95E-02	2.31E-01
5.24	5.23	5.39	2.95E-02	2.31E-01
8.17	8.14	8.29	2.95E-02	2.31E-01
6.37	6.34	6.33	2.95E-02	2.31E-01
6.40	6.83	6.67	2.96E-02	2.31E-01
8.00	8.07	8.03	2.96E-02	2.31E-01
6.35	6.51	6.26	2.96E-02	2.32E-01
8.28	8.21	8.07	2.97E-02	2.32E-01
5.36	5.29	5.26	2.97E-02	2.32E-01
8.66	8.47	8.65	2.97E-02	2.32E-01
7.52	7.63	7.44	2.97E-02	2.32E-01
9.14	8.98	8.97	2.97E-02	2.32E-01
9.25	9.35	9.35	2.97E-02	2.32E-01
7.02	7.07	7.13	2.98E-02	2.32E-01
5.86	6.03	5.98	2.98E-02	2.32E-01
9.95	9.87	9.90	2.98E-02	2.32E-01
6.54	6.58	6.43	2.98E-02	2.32E-01
8.07	8.06	7.97	2.99E-02	2.32E-01
8.52	8.70	8.52	2.99E-02	2.32E-01
5.56	5.91	5.80	2.99E-02	2.32E-01
3.68	3.54	3.63	3.00E-02	2.32E-01
4.99	5.18	5.08	3.00E-02	2.32E-01
3.16	3.15	3.28	3.00E-02	2.32E-01
6.21	6.26	6.03	3.00E-02	2.32E-01
7.49	7.56	7.42	3.00E-02	2.32E-01

6.64	7.18	6.81	3.00E-02	2.32E-01
6.91	6.70	6.83	3.00E-02	2.33E-01
9.26	9.43	9.41	3.01E-02	2.33E-01
9.15	9.20	9.18	3.01E-02	2.33E-01
8.80	9.00	8.81	3.01E-02	2.33E-01
10.11	10.01	10.04	3.02E-02	2.33E-01
7.91	7.91	7.98	3.02E-02	2.33E-01
6.60	6.44	6.42	3.02E-02	2.33E-01
8.04	8.10	8.12	3.03E-02	2.33E-01
6.32	6.25	6.26	3.03E-02	2.33E-01
5.98	5.90	5.84	3.03E-02	2.34E-01
7.76	7.88	7.76	3.04E-02	2.34E-01
9.06	9.13	9.05	3.04E-02	2.34E-01
6.26	6.21	6.09	3.04E-02	2.34E-01
8.18	8.01	8.11	3.04E-02	2.34E-01
8.21	8.01	8.12	3.04E-02	2.34E-01
13.72	13.77	13.74	3.04E-02	2.34E-01
6.13	6.18	6.08	3.04E-02	2.34E-01
7.84	8.07	7.96	3.05E-02	2.34E-01
4.40	4.37	4.46	3.05E-02	2.34E-01
10.29	10.29	10.46	3.05E-02	2.34E-01
13.24	13.19	13.25	3.06E-02	2.34E-01
6.90	7.02	7.13	3.06E-02	2.35E-01
4.64	4.78	4.48	3.06E-02	2.35E-01
8.03	8.05	8.04	3.06E-02	2.35E-01
5.29	5.40	5.58	3.07E-02	2.35E-01
4.50	4.34	4.41	3.07E-02	2.35E-01
7.60	7.79	7.73	3.07E-02	2.35E-01
11.00	11.12	10.99	3.08E-02	2.35E-01
3.52	3.46	3.59	3.08E-02	2.35E-01
7.45	7.22	7.39	3.08E-02	2.35E-01
3.43	3.34	3.42	3.09E-02	2.35E-01
6.35	6.49	6.47	3.09E-02	2.35E-01
9.44	9.24	9.45	3.09E-02	2.35E-01
5.74	5.61	5.75	3.09E-02	2.35E-01
6.43	6.38	6.34	3.09E-02	2.35E-01
5.84	5.76	5.96	3.09E-02	2.36E-01
8.15	8.28	8.02	3.10E-02	2.36E-01
5.21	5.28	5.58	3.10E-02	2.36E-01
6.50	6.62	6.23	3.10E-02	2.36E-01
6.29	6.29	6.41	3.10E-02	2.36E-01
8.96	9.18	8.98	3.11E-02	2.36E-01
9.60	9.62	9.77	3.11E-02	2.37E-01
8.87	8.73	8.94	3.12E-02	2.37E-01
9.63	9.58	9.70	3.12E-02	2.37E-01
9.57	9.35	9.56	3.12E-02	2.37E-01
9.52	9.40	9.58	3.12E-02	2.37E-01

13.19	12.95	13.09	3.12E-02	2.37E-01
5.49	5.44	5.58	3.13E-02	2.37E-01
10.17	10.17	10.28	3.13E-02	2.37E-01
11.50	11.63	11.55	3.13E-02	2.37E-01
9.20	9.27	9.31	3.13E-02	2.37E-01
9.30	9.19	9.15	3.14E-02	2.38E-01
10.76	10.99	10.87	3.15E-02	2.38E-01
9.49	9.65	9.67	3.15E-02	2.38E-01
8.80	8.89	8.70	3.15E-02	2.38E-01
8.74	8.43	8.81	3.16E-02	2.38E-01
4.21	4.28	4.11	3.16E-02	2.38E-01
11.22	11.36	11.33	3.16E-02	2.38E-01
7.48	7.65	7.64	3.17E-02	2.38E-01
8.55	8.49	8.76	3.17E-02	2.38E-01
7.01	7.29	7.12	3.17E-02	2.38E-01
7.94	7.97	8.02	3.17E-02	2.38E-01
7.73	7.95	7.72	3.17E-02	2.38E-01
7.54	7.81	7.62	3.17E-02	2.38E-01
10.01	9.94	10.04	3.17E-02	2.38E-01
6.37	6.51	6.35	3.17E-02	2.38E-01
6.76	6.87	6.74	3.18E-02	2.39E-01
12.45	12.38	12.40	3.18E-02	2.39E-01
6.98	7.00	6.83	3.19E-02	2.39E-01
5.16	5.06	5.27	3.19E-02	2.39E-01
8.97	9.08	9.07	3.20E-02	2.39E-01
9.28	9.44	9.36	3.20E-02	2.39E-01
8.38	8.49	8.50	3.20E-02	2.39E-01
9.84	9.62	9.74	3.20E-02	2.39E-01
9.48	9.32	9.48	3.21E-02	2.40E-01
6.76	6.77	6.78	3.21E-02	2.40E-01
4.47	4.47	4.45	3.21E-02	2.40E-01
7.96	7.99	7.65	3.21E-02	2.40E-01
12.33	12.17	12.19	3.21E-02	2.40E-01
10.12	9.92	9.88	3.21E-02	2.40E-01
11.37	11.27	11.34	3.21E-02	2.40E-01
11.03	11.01	11.06	3.22E-02	2.40E-01
10.07	10.34	10.12	3.22E-02	2.40E-01
5.22	5.28	5.36	3.22E-02	2.40E-01
3.78	3.95	3.69	3.22E-02	2.40E-01
7.63	7.45	7.45	3.22E-02	2.40E-01
4.89	5.18	5.04	3.23E-02	2.40E-01
7.93	7.85	7.89	3.23E-02	2.40E-01
7.95	7.92	7.75	3.23E-02	2.40E-01
11.53	11.67	11.63	3.24E-02	2.41E-01
7.62	7.60	7.47	3.24E-02	2.41E-01
7.78	7.62	7.66	3.24E-02	2.41E-01
13.41	13.21	13.30	3.24E-02	2.41E-01

5.15	5.37	5.12	3.25E-02	2.41E-01
6.49	6.56	6.53	3.25E-02	2.41E-01
7.21	7.16	7.21	3.25E-02	2.41E-01
8.72	8.69	8.81	3.25E-02	2.41E-01
5.90	6.11	5.98	3.25E-02	2.41E-01
6.70	6.97	6.82	3.25E-02	2.41E-01
4.19	4.21	4.31	3.26E-02	2.41E-01
8.59	8.62	8.65	3.26E-02	2.41E-01
12.61	12.72	12.71	3.26E-02	2.41E-01
3.38	3.65	3.47	3.27E-02	2.41E-01
9.15	9.38	9.22	3.27E-02	2.41E-01
9.30	9.01	9.30	3.27E-02	2.41E-01
9.34	9.55	9.36	3.28E-02	2.42E-01
6.68	6.63	6.54	3.29E-02	2.42E-01
4.07	4.11	4.11	3.29E-02	2.42E-01
10.03	10.04	10.07	3.29E-02	2.42E-01
7.20	7.33	7.26	3.29E-02	2.42E-01
8.37	8.22	8.48	3.29E-02	2.42E-01
3.47	3.63	3.47	3.30E-02	2.42E-01
8.37	8.51	8.37	3.30E-02	2.42E-01
5.47	5.42	5.35	3.30E-02	2.42E-01
9.72	9.61	9.86	3.30E-02	2.42E-01
7.87	8.07	8.01	3.30E-02	2.42E-01
11.24	11.43	11.32	3.30E-02	2.42E-01
10.88	10.96	10.92	3.31E-02	2.42E-01
9.22	9.34	9.28	3.31E-02	2.42E-01
7.28	7.02	7.17	3.31E-02	2.42E-01
7.59	7.34	8.04	3.31E-02	2.42E-01
6.30	6.56	6.39	3.31E-02	2.42E-01
5.89	5.85	5.98	3.31E-02	2.42E-01
7.81	7.62	7.82	3.31E-02	2.42E-01
9.57	9.43	9.55	3.32E-02	2.42E-01
9.65	9.71	9.49	3.32E-02	2.42E-01
4.49	4.64	4.60	3.32E-02	2.42E-01
9.25	9.10	9.20	3.32E-02	2.42E-01
7.99	8.37	7.89	3.32E-02	2.43E-01
11.25	11.18	11.19	3.32E-02	2.43E-01
6.54	6.61	6.56	3.33E-02	2.43E-01
8.85	9.24	9.02	3.33E-02	2.43E-01
12.31	12.35	12.36	3.34E-02	2.43E-01
3.36	3.38	3.45	3.34E-02	2.43E-01
4.37	4.06	4.16	3.34E-02	2.43E-01
7.59	7.62	7.56	3.34E-02	2.43E-01
12.12	12.03	12.10	3.35E-02	2.43E-01
8.23	7.98	8.28	3.36E-02	2.44E-01
14.88	14.91	14.86	3.36E-02	2.44E-01
10.61	10.82	10.77	3.36E-02	2.44E-01

9.97	10.18	10.14	3.36E-02	2.44E-01
7.19	7.31	7.26	3.36E-02	2.44E-01
3.61	3.64	3.60	3.37E-02	2.44E-01
7.64	7.72	7.69	3.37E-02	2.44E-01
9.78	9.70	9.82	3.37E-02	2.44E-01
3.57	3.56	3.67	3.37E-02	2.44E-01
11.07	10.92	11.12	3.37E-02	2.44E-01
11.22	11.14	11.15	3.38E-02	2.44E-01
8.31	8.38	8.36	3.38E-02	2.44E-01
10.00	10.05	10.01	3.38E-02	2.45E-01
11.21	11.22	11.28	3.38E-02	2.45E-01
8.07	8.13	8.16	3.39E-02	2.45E-01
4.79	4.75	4.77	3.39E-02	2.45E-01
6.77	6.81	6.85	3.39E-02	2.45E-01
10.45	10.70	10.53	3.39E-02	2.45E-01
7.21	7.25	7.33	3.39E-02	2.45E-01
8.50	8.45	8.44	3.39E-02	2.45E-01
4.34	4.38	4.31	3.39E-02	2.45E-01
5.76	5.47	5.74	3.40E-02	2.45E-01
4.13	4.46	4.31	3.40E-02	2.45E-01
10.39	10.59	10.43	3.40E-02	2.45E-01
10.09	10.20	10.16	3.40E-02	2.45E-01
9.78	9.60	9.84	3.40E-02	2.45E-01
10.23	10.29	10.28	3.40E-02	2.45E-01
10.48	10.52	10.62	3.41E-02	2.45E-01
4.41	4.29	4.36	3.41E-02	2.45E-01
3.59	3.56	3.58	3.41E-02	2.45E-01
9.18	9.34	9.18	3.41E-02	2.45E-01
8.93	8.84	8.90	3.42E-02	2.45E-01
8.60	8.56	8.74	3.42E-02	2.45E-01
5.27	5.32	5.39	3.42E-02	2.45E-01
8.26	8.17	8.34	3.42E-02	2.45E-01
4.06	4.17	4.04	3.42E-02	2.45E-01
10.47	10.55	10.54	3.42E-02	2.45E-01
6.64	6.53	6.59	3.43E-02	2.45E-01
3.27	3.24	3.27	3.43E-02	2.45E-01
10.05	10.22	10.21	3.44E-02	2.45E-01
8.84	8.97	9.00	3.44E-02	2.45E-01
5.25	4.99	5.44	3.44E-02	2.45E-01
5.79	5.87	6.05	3.44E-02	2.45E-01
13.24	13.18	13.21	3.44E-02	2.45E-01
7.34	7.91	7.39	3.44E-02	2.45E-01
9.81	9.87	9.73	3.44E-02	2.45E-01
10.07	9.88	9.92	3.44E-02	2.45E-01
7.36	7.30	7.28	3.45E-02	2.45E-01
3.61	3.43	3.60	3.45E-02	2.45E-01
5.83	5.95	5.84	3.45E-02	2.45E-01

10.93	11.11	10.99	3.45E-02	2.45E-01
4.08	4.07	4.10	3.45E-02	2.45E-01
7.58	7.50	7.47	3.45E-02	2.45E-01
7.01	7.12	7.23	3.46E-02	2.45E-01
6.08	6.12	5.92	3.46E-02	2.45E-01
7.62	7.83	7.67	3.46E-02	2.45E-01
3.41	3.34	3.41	3.46E-02	2.45E-01
4.99	4.63	4.91	3.46E-02	2.45E-01
6.55	6.62	6.76	3.46E-02	2.45E-01
3.94	4.02	4.10	3.46E-02	2.45E-01
9.11	9.11	9.22	3.47E-02	2.45E-01
6.86	6.80	6.64	3.47E-02	2.45E-01
3.51	3.57	3.53	3.47E-02	2.45E-01
6.22	6.35	6.37	3.47E-02	2.45E-01
12.41	12.51	12.44	3.47E-02	2.45E-01
9.02	9.19	9.02	3.48E-02	2.46E-01
6.32	6.20	6.56	3.48E-02	2.46E-01
9.98	9.95	10.07	3.48E-02	2.46E-01
8.65	8.66	8.54	3.48E-02	2.46E-01
8.30	8.12	8.25	3.48E-02	2.46E-01
4.78	4.74	4.76	3.49E-02	2.46E-01
11.31	11.05	11.23	3.49E-02	2.46E-01
9.56	9.67	9.63	3.49E-02	2.46E-01
10.59	10.64	10.67	3.49E-02	2.46E-01
8.22	8.25	8.13	3.50E-02	2.46E-01
9.61	9.73	9.72	3.50E-02	2.46E-01
4.46	4.36	4.59	3.50E-02	2.46E-01
7.87	7.86	7.97	3.50E-02	2.46E-01
7.66	7.71	7.67	3.50E-02	2.46E-01
9.23	9.28	9.38	3.50E-02	2.46E-01
7.41	7.29	7.23	3.50E-02	2.46E-01
9.48	9.50	9.53	3.50E-02	2.46E-01
3.34	3.34	3.50	3.50E-02	2.46E-01
3.95	4.07	4.10	3.50E-02	2.46E-01
6.75	6.84	6.72	3.50E-02	2.46E-01
4.40	4.26	4.38	3.51E-02	2.46E-01
5.63	5.76	5.98	3.52E-02	2.46E-01
7.01	6.98	6.93	3.52E-02	2.46E-01
12.04	12.06	12.12	3.52E-02	2.46E-01
9.77	9.73	9.89	3.52E-02	2.46E-01
7.90	7.97	7.87	3.52E-02	2.46E-01
3.60	3.45	3.47	3.52E-02	2.46E-01
9.87	9.59	10.08	3.54E-02	2.47E-01
8.85	8.99	8.87	3.54E-02	2.47E-01
10.81	10.92	10.92	3.54E-02	2.47E-01
7.34	7.25	7.26	3.54E-02	2.47E-01
8.27	8.13	8.16	3.54E-02	2.47E-01

7.26	7.18	7.18	3.55E-02	2.47E-01
8.35	8.29	8.37	3.55E-02	2.47E-01
4.14	4.14	4.10	3.55E-02	2.47E-01
8.93	8.72	8.82	3.55E-02	2.47E-01
7.71	7.64	7.82	3.56E-02	2.47E-01
6.48	6.57	6.63	3.56E-02	2.47E-01
8.13	7.86	8.04	3.56E-02	2.47E-01
10.73	10.85	10.86	3.56E-02	2.47E-01
4.83	5.14	4.92	3.56E-02	2.47E-01
6.81	6.73	6.76	3.56E-02	2.47E-01
6.52	6.71	6.68	3.57E-02	2.47E-01
9.36	9.34	9.35	3.57E-02	2.48E-01
7.03	7.10	7.00	3.57E-02	2.48E-01
8.91	8.84	8.85	3.57E-02	2.48E-01
7.37	7.45	7.49	3.58E-02	2.48E-01
11.31	11.10	11.33	3.58E-02	2.48E-01
8.75	8.70	8.73	3.58E-02	2.48E-01
4.01	4.11	4.10	3.58E-02	2.48E-01
4.06	4.19	4.09	3.58E-02	2.48E-01
12.37	12.49	12.48	3.59E-02	2.48E-01
10.68	10.71	10.83	3.59E-02	2.48E-01
12.40	12.58	12.50	3.59E-02	2.48E-01
12.31	12.26	12.35	3.60E-02	2.48E-01
7.67	7.43	7.57	3.60E-02	2.49E-01
6.16	6.14	6.36	3.61E-02	2.49E-01
7.24	7.31	7.53	3.61E-02	2.49E-01
7.32	7.13	7.20	3.61E-02	2.49E-01
10.12	9.88	10.03	3.61E-02	2.49E-01
10.73	10.69	10.69	3.61E-02	2.49E-01
5.40	5.33	5.51	3.61E-02	2.49E-01
11.93	11.62	11.92	3.61E-02	2.49E-01
7.96	8.06	7.98	3.62E-02	2.49E-01
8.89	8.97	9.00	3.62E-02	2.49E-01
7.82	7.72	7.80	3.62E-02	2.49E-01
7.15	7.25	7.21	3.62E-02	2.49E-01
3.59	3.47	3.57	3.62E-02	2.49E-01
3.40	3.40	3.27	3.62E-02	2.49E-01
9.54	9.39	9.46	3.62E-02	2.49E-01
10.45	10.45	10.57	3.62E-02	2.49E-01
6.93	6.93	6.79	3.63E-02	2.49E-01
11.06	11.31	11.21	3.63E-02	2.49E-01
10.16	10.27	10.23	3.63E-02	2.49E-01
10.02	10.01	10.04	3.63E-02	2.49E-01
7.87	8.04	7.86	3.64E-02	2.49E-01
11.90	11.94	11.91	3.64E-02	2.49E-01
6.48	6.51	6.58	3.64E-02	2.49E-01
7.19	7.05	7.03	3.64E-02	2.49E-01

3.51	3.44	3.44	3.65E-02	2.49E-01
4.48	4.43	4.68	3.65E-02	2.49E-01
5.04	4.89	4.81	3.65E-02	2.49E-01
3.15	3.14	3.20	3.65E-02	2.49E-01
10.08	10.21	10.23	3.65E-02	2.49E-01
7.70	7.63	7.84	3.65E-02	2.49E-01
10.27	10.20	10.29	3.66E-02	2.49E-01
6.95	7.22	7.25	3.66E-02	2.49E-01
7.30	7.38	7.51	3.66E-02	2.49E-01
8.10	8.14	8.28	3.66E-02	2.49E-01
11.68	12.02	11.67	3.66E-02	2.49E-01
8.97	8.96	9.17	3.66E-02	2.49E-01
5.61	5.63	5.63	3.66E-02	2.49E-01
5.66	5.60	5.75	3.67E-02	2.49E-01
3.98	3.95	4.04	3.67E-02	2.49E-01
7.01	7.46	7.19	3.67E-02	2.49E-01
3.79	3.71	3.70	3.67E-02	2.49E-01
3.87	4.03	3.98	3.68E-02	2.49E-01
9.44	9.44	9.51	3.68E-02	2.49E-01
15.26	15.25	15.24	3.68E-02	2.49E-01
10.04	10.10	10.10	3.68E-02	2.49E-01
7.46	6.98	7.09	3.68E-02	2.49E-01
6.10	6.03	5.94	3.68E-02	2.49E-01
5.16	5.15	5.13	3.69E-02	2.50E-01
6.59	6.61	6.70	3.69E-02	2.50E-01
8.01	7.96	7.87	3.69E-02	2.50E-01
5.10	4.96	5.06	3.70E-02	2.50E-01
4.73	4.60	4.69	3.71E-02	2.50E-01
9.38	9.19	9.35	3.71E-02	2.51E-01
8.66	8.92	8.88	3.71E-02	2.51E-01
9.48	9.40	9.50	3.71E-02	2.51E-01
8.27	8.46	8.44	3.72E-02	2.51E-01
7.29	7.04	7.10	3.72E-02	2.51E-01
5.33	5.19	5.41	3.72E-02	2.51E-01
9.07	9.22	9.03	3.72E-02	2.51E-01
5.49	5.49	5.41	3.72E-02	2.51E-01
8.11	8.14	8.17	3.73E-02	2.51E-01
5.67	5.71	5.88	3.73E-02	2.51E-01
9.64	9.60	9.56	3.73E-02	2.51E-01
10.02	10.19	10.13	3.73E-02	2.51E-01
5.17	5.15	5.07	3.73E-02	2.51E-01
7.80	7.90	7.82	3.74E-02	2.51E-01
11.01	10.88	10.96	3.75E-02	2.51E-01
8.73	8.80	8.78	3.75E-02	2.51E-01
5.60	5.73	5.22	3.75E-02	2.51E-01
4.16	4.06	4.02	3.75E-02	2.51E-01
6.67	6.78	6.69	3.75E-02	2.52E-01

3.52	3.54	3.46	3.76E-02	2.52E-01
10.18	10.19	10.14	3.76E-02	2.52E-01
8.88	9.07	8.99	3.76E-02	2.52E-01
5.92	5.79	5.90	3.76E-02	2.52E-01
8.38	8.32	8.33	3.76E-02	2.52E-01
6.49	6.51	6.29	3.76E-02	2.52E-01
5.13	5.25	5.29	3.77E-02	2.52E-01
8.03	7.99	8.03	3.77E-02	2.52E-01
9.36	9.45	9.46	3.77E-02	2.52E-01
6.19	6.15	6.04	3.77E-02	2.52E-01
5.36	5.29	5.31	3.77E-02	2.52E-01
9.12	9.05	9.13	3.78E-02	2.52E-01
5.79	5.56	5.70	3.78E-02	2.52E-01
4.43	4.43	4.35	3.78E-02	2.52E-01
3.67	3.71	3.69	3.79E-02	2.52E-01
4.39	4.26	4.17	3.79E-02	2.52E-01
7.34	7.57	7.53	3.79E-02	2.52E-01
5.66	5.60	5.62	3.79E-02	2.52E-01
7.44	7.43	7.36	3.79E-02	2.52E-01
11.76	11.55	11.54	3.79E-02	2.52E-01
10.77	10.76	10.74	3.80E-02	2.52E-01
4.27	4.31	4.54	3.80E-02	2.53E-01
10.56	10.75	10.70	3.81E-02	2.53E-01
10.17	10.33	10.24	3.81E-02	2.53E-01
6.82	7.07	6.88	3.81E-02	2.53E-01
10.16	10.30	10.21	3.81E-02	2.53E-01
8.23	8.00	8.23	3.81E-02	2.53E-01
12.14	11.92	12.17	3.81E-02	2.53E-01
12.26	12.35	12.33	3.81E-02	2.53E-01
8.31	8.57	8.42	3.81E-02	2.53E-01
7.77	8.02	7.79	3.82E-02	2.53E-01
13.83	13.87	13.89	3.82E-02	2.53E-01
11.33	11.34	11.43	3.82E-02	2.53E-01
11.97	11.85	12.17	3.82E-02	2.53E-01
6.86	6.92	6.88	3.82E-02	2.53E-01
4.77	4.94	4.85	3.83E-02	2.53E-01
7.95	7.88	7.68	3.83E-02	2.53E-01
6.40	6.45	6.29	3.84E-02	2.53E-01
8.48	8.65	8.52	3.84E-02	2.53E-01
8.18	8.34	8.25	3.84E-02	2.53E-01
7.61	7.77	7.81	3.84E-02	2.53E-01
7.51	7.41	7.37	3.85E-02	2.53E-01
10.69	10.74	10.76	3.85E-02	2.53E-01
13.62	13.67	13.58	3.86E-02	2.54E-01
10.65	10.83	10.67	3.86E-02	2.54E-01
6.89	6.86	6.94	3.86E-02	2.54E-01
4.66	4.46	4.41	3.86E-02	2.54E-01

7.13	7.30	7.21	3.87E-02	2.55E-01
5.60	5.29	5.37	3.87E-02	2.55E-01
4.55	4.38	4.45	3.88E-02	2.55E-01
10.44	10.61	10.57	3.88E-02	2.55E-01
10.23	10.28	10.30	3.88E-02	2.55E-01
10.67	10.57	10.65	3.88E-02	2.55E-01
10.67	10.46	10.58	3.89E-02	2.55E-01
5.63	5.59	5.76	3.89E-02	2.55E-01
10.23	10.27	10.34	3.89E-02	2.55E-01
4.61	4.60	4.55	3.90E-02	2.55E-01
11.89	11.94	11.85	3.90E-02	2.55E-01
8.15	8.07	8.27	3.90E-02	2.55E-01
8.18	8.20	8.13	3.90E-02	2.55E-01
7.82	7.93	7.93	3.90E-02	2.55E-01
8.90	8.87	8.93	3.91E-02	2.55E-01
8.40	8.22	8.40	3.91E-02	2.55E-01
12.04	12.01	12.02	3.91E-02	2.55E-01
5.66	5.67	5.62	3.91E-02	2.55E-01
8.30	8.20	8.15	3.91E-02	2.55E-01
5.76	5.82	5.87	3.91E-02	2.55E-01
6.81	6.86	6.67	3.92E-02	2.55E-01
8.30	8.47	8.49	3.92E-02	2.55E-01
8.07	8.23	8.10	3.92E-02	2.55E-01
9.82	9.47	9.58	3.92E-02	2.55E-01
7.29	7.36	7.32	3.92E-02	2.55E-01
11.49	11.55	11.49	3.93E-02	2.56E-01
7.13	7.18	7.14	3.93E-02	2.56E-01
10.26	10.16	10.44	3.94E-02	2.56E-01
10.65	10.48	10.49	3.94E-02	2.56E-01
9.87	9.67	9.82	3.94E-02	2.56E-01
10.64	10.69	10.75	3.94E-02	2.56E-01
3.19	3.16	3.15	3.94E-02	2.56E-01
8.70	8.69	8.86	3.95E-02	2.56E-01
7.84	8.01	7.93	3.95E-02	2.56E-01
6.60	6.86	6.88	3.95E-02	2.56E-01
7.84	7.56	7.92	3.95E-02	2.56E-01
9.72	9.77	9.90	3.95E-02	2.56E-01
7.74	7.97	7.88	3.95E-02	2.56E-01
9.28	9.53	9.53	3.96E-02	2.56E-01
8.24	8.12	8.17	3.97E-02	2.57E-01
8.29	8.33	8.32	3.97E-02	2.57E-01
10.17	10.20	10.20	3.97E-02	2.57E-01
9.40	9.34	9.38	3.98E-02	2.57E-01
3.94	4.07	3.99	3.98E-02	2.57E-01
11.93	11.92	11.71	3.98E-02	2.57E-01
7.54	7.84	7.55	3.99E-02	2.57E-01
9.22	9.41	9.35	3.99E-02	2.58E-01

5.60	5.15	5.48	4.00E-02	2.58E-01
3.90	3.75	3.92	4.00E-02	2.58E-01
8.10	8.05	8.08	4.00E-02	2.58E-01
9.19	9.37	9.35	4.00E-02	2.58E-01
9.62	9.62	9.64	4.00E-02	2.58E-01
8.97	9.10	8.91	4.00E-02	2.58E-01
3.36	3.29	3.32	4.00E-02	2.58E-01
12.19	12.06	12.15	4.01E-02	2.58E-01
8.66	8.41	8.65	4.01E-02	2.58E-01
3.82	3.92	3.78	4.01E-02	2.58E-01
10.46	10.43	10.47	4.01E-02	2.58E-01
4.84	4.89	4.86	4.01E-02	2.58E-01
10.64	10.72	10.80	4.01E-02	2.58E-01
6.65	6.44	6.52	4.02E-02	2.58E-01
6.78	6.65	6.87	4.03E-02	2.58E-01
7.71	7.70	7.71	4.03E-02	2.58E-01
6.67	6.59	6.78	4.03E-02	2.58E-01
8.65	8.52	8.35	4.03E-02	2.58E-01
9.57	9.74	9.55	4.03E-02	2.58E-01
7.15	7.30	7.16	4.03E-02	2.58E-01
8.30	8.29	8.26	4.03E-02	2.58E-01
5.58	5.43	5.60	4.03E-02	2.58E-01
4.42	4.23	4.44	4.04E-02	2.58E-01
3.70	3.75	3.67	4.04E-02	2.58E-01
8.41	7.95	8.21	4.05E-02	2.58E-01
11.92	12.03	11.97	4.05E-02	2.58E-01
7.71	7.63	7.83	4.05E-02	2.58E-01
5.41	5.32	5.43	4.05E-02	2.58E-01
5.88	5.69	5.93	4.05E-02	2.58E-01
7.68	7.85	7.84	4.06E-02	2.59E-01
8.92	9.16	8.97	4.06E-02	2.59E-01
9.50	9.41	9.50	4.07E-02	2.59E-01
8.68	8.73	8.71	4.07E-02	2.59E-01
5.78	5.79	5.71	4.07E-02	2.59E-01
7.15	7.42	7.14	4.07E-02	2.59E-01
4.69	4.70	4.73	4.08E-02	2.60E-01
7.80	8.05	7.97	4.08E-02	2.60E-01
5.63	5.85	5.44	4.08E-02	2.60E-01
3.80	3.80	3.85	4.09E-02	2.60E-01
5.20	5.06	5.14	4.09E-02	2.60E-01
8.90	9.07	9.02	4.09E-02	2.60E-01
10.65	10.58	10.68	4.09E-02	2.60E-01
5.86	6.09	6.06	4.09E-02	2.60E-01
7.82	7.88	7.93	4.10E-02	2.60E-01
12.30	12.36	12.31	4.10E-02	2.60E-01
13.32	13.29	13.29	4.10E-02	2.60E-01
4.68	5.03	4.80	4.10E-02	2.60E-01

7.37	7.41	7.38	4.10E-02	2.60E-01
10.74	10.69	10.80	4.11E-02	2.60E-01
8.68	8.65	8.78	4.11E-02	2.60E-01
10.42	10.52	10.41	4.11E-02	2.60E-01
10.18	10.39	10.20	4.11E-02	2.60E-01
3.37	3.41	3.38	4.11E-02	2.60E-01
3.40	3.41	3.37	4.12E-02	2.61E-01
7.92	7.88	7.93	4.12E-02	2.61E-01
10.48	10.38	10.34	4.13E-02	2.61E-01
9.20	9.60	9.42	4.13E-02	2.61E-01
6.81	6.86	6.93	4.13E-02	2.61E-01
6.27	6.44	6.45	4.14E-02	2.61E-01
5.75	5.86	5.73	4.14E-02	2.61E-01
8.48	8.41	8.58	4.14E-02	2.61E-01
7.61	7.77	7.49	4.14E-02	2.61E-01
6.08	5.96	6.18	4.15E-02	2.61E-01
10.77	10.94	10.87	4.16E-02	2.62E-01
7.63	7.52	7.51	4.16E-02	2.62E-01
5.13	4.90	5.09	4.16E-02	2.62E-01
8.14	8.13	8.24	4.16E-02	2.62E-01
6.86	6.90	6.85	4.16E-02	2.62E-01
5.74	5.73	5.50	4.16E-02	2.62E-01
5.62	5.54	5.62	4.17E-02	2.62E-01
7.01	6.84	7.07	4.19E-02	2.63E-01
8.89	8.70	8.96	4.19E-02	2.63E-01
6.44	6.51	6.55	4.19E-02	2.63E-01
6.79	6.86	6.84	4.19E-02	2.63E-01
6.46	6.48	6.31	4.20E-02	2.63E-01
8.57	8.79	8.72	4.20E-02	2.63E-01
10.16	10.33	10.14	4.20E-02	2.63E-01
3.81	3.69	3.77	4.20E-02	2.63E-01
6.91	7.05	6.97	4.21E-02	2.63E-01
7.97	8.05	7.94	4.21E-02	2.63E-01
6.35	6.45	6.59	4.21E-02	2.63E-01
7.67	7.79	7.61	4.21E-02	2.63E-01
7.44	7.69	7.65	4.22E-02	2.63E-01
7.08	7.22	7.18	4.22E-02	2.63E-01
12.11	12.11	12.02	4.22E-02	2.63E-01
10.11	10.36	10.31	4.22E-02	2.64E-01
10.16	10.25	10.21	4.23E-02	2.64E-01
4.49	4.54	4.67	4.23E-02	2.64E-01
10.27	10.38	10.35	4.23E-02	2.64E-01
10.84	11.00	10.86	4.23E-02	2.64E-01
7.15	7.26	7.25	4.24E-02	2.64E-01
5.84	5.63	5.74	4.24E-02	2.64E-01
4.41	4.42	4.31	4.24E-02	2.64E-01
8.43	8.62	8.58	4.24E-02	2.64E-01

6.51	6.29	6.49	4.25E-02	2.64E-01
8.05	8.34	8.15	4.25E-02	2.64E-01
11.25	11.18	11.23	4.25E-02	2.64E-01
14.89	14.83	14.87	4.25E-02	2.64E-01
6.65	6.45	6.35	4.26E-02	2.64E-01
9.17	9.08	9.12	4.26E-02	2.64E-01
9.03	8.97	8.78	4.26E-02	2.64E-01
7.64	7.58	7.60	4.26E-02	2.64E-01
4.94	4.92	4.94	4.26E-02	2.64E-01
6.87	6.92	6.95	4.27E-02	2.65E-01
5.58	5.56	5.58	4.27E-02	2.65E-01
8.04	8.15	8.15	4.27E-02	2.65E-01
5.59	5.53	5.52	4.28E-02	2.65E-01
6.70	6.70	6.79	4.28E-02	2.65E-01
7.47	7.52	7.49	4.28E-02	2.65E-01
7.02	6.85	6.93	4.28E-02	2.65E-01
9.60	9.82	9.50	4.28E-02	2.65E-01
7.12	6.66	6.71	4.28E-02	2.65E-01
6.96	6.95	7.04	4.29E-02	2.65E-01
4.28	3.92	4.13	4.29E-02	2.65E-01
8.94	9.11	8.84	4.30E-02	2.65E-01
4.92	5.08	4.86	4.30E-02	2.65E-01
3.80	3.73	3.78	4.30E-02	2.65E-01
5.08	4.99	5.19	4.30E-02	2.65E-01
7.71	7.78	7.77	4.30E-02	2.65E-01
9.61	9.34	9.67	4.30E-02	2.65E-01
7.31	7.17	7.31	4.30E-02	2.65E-01
10.34	10.32	10.26	4.30E-02	2.65E-01
9.44	9.36	9.51	4.30E-02	2.65E-01
14.10	14.09	14.13	4.31E-02	2.65E-01
3.51	3.45	3.47	4.31E-02	2.65E-01
4.58	4.60	4.76	4.31E-02	2.65E-01
10.68	10.93	10.81	4.31E-02	2.65E-01
4.49	4.12	4.33	4.31E-02	2.65E-01
8.67	8.32	8.55	4.31E-02	2.65E-01
3.38	3.16	3.30	4.31E-02	2.65E-01
8.12	8.18	8.24	4.32E-02	2.65E-01
7.29	7.45	7.26	4.32E-02	2.65E-01
8.44	8.48	8.45	4.32E-02	2.65E-01
8.67	8.63	8.70	4.32E-02	2.65E-01
6.71	6.43	6.63	4.33E-02	2.65E-01
3.78	3.77	4.20	4.33E-02	2.65E-01
6.55	6.31	6.58	4.33E-02	2.65E-01
5.70	5.98	5.78	4.33E-02	2.65E-01
9.36	9.34	9.31	4.33E-02	2.65E-01
7.40	7.36	7.43	4.33E-02	2.65E-01
8.88	9.00	8.98	4.33E-02	2.65E-01

9.30	9.32	9.35	4.33E-02	2.65E-01
7.98	8.09	7.87	4.34E-02	2.65E-01
6.44	6.46	6.25	4.34E-02	2.65E-01
11.84	11.97	11.89	4.34E-02	2.65E-01
9.26	9.16	9.27	4.34E-02	2.65E-01
5.97	6.04	5.91	4.34E-02	2.65E-01
10.76	10.95	10.75	4.35E-02	2.65E-01
5.63	5.55	5.55	4.35E-02	2.65E-01
11.28	11.32	11.36	4.35E-02	2.65E-01
7.44	7.67	7.63	4.35E-02	2.65E-01
10.76	10.75	10.95	4.35E-02	2.65E-01
9.74	9.64	9.83	4.35E-02	2.65E-01
8.89	8.94	9.02	4.36E-02	2.65E-01
6.74	6.70	6.80	4.36E-02	2.65E-01
7.68	7.85	7.73	4.36E-02	2.65E-01
6.12	6.10	6.54	4.36E-02	2.65E-01
8.71	8.60	8.56	4.36E-02	2.65E-01
9.27	9.24	9.27	4.37E-02	2.65E-01
5.90	5.84	6.05	4.37E-02	2.65E-01
7.23	7.64	7.49	4.37E-02	2.65E-01
8.34	8.10	8.39	4.37E-02	2.65E-01
8.80	8.70	8.71	4.37E-02	2.65E-01
6.06	6.21	6.15	4.37E-02	2.65E-01
5.70	5.95	5.83	4.38E-02	2.65E-01
7.56	7.56	7.43	4.38E-02	2.65E-01
7.38	7.34	7.22	4.38E-02	2.65E-01
4.53	4.58	4.54	4.38E-02	2.65E-01
9.37	9.57	9.46	4.38E-02	2.65E-01
8.51	8.29	8.49	4.38E-02	2.65E-01
9.93	9.97	9.87	4.38E-02	2.65E-01
5.97	5.95	6.29	4.38E-02	2.65E-01
4.45	4.28	4.46	4.39E-02	2.65E-01
7.88	8.05	7.87	4.39E-02	2.65E-01
6.92	6.73	6.94	4.40E-02	2.65E-01
11.25	11.18	11.28	4.40E-02	2.65E-01
8.11	8.19	8.03	4.40E-02	2.65E-01
9.93	10.00	9.95	4.40E-02	2.65E-01
3.78	3.81	3.67	4.40E-02	2.65E-01
8.97	9.01	9.19	4.40E-02	2.65E-01
8.77	8.72	8.81	4.41E-02	2.65E-01
6.99	6.96	7.01	4.41E-02	2.66E-01
7.01	6.85	7.06	4.42E-02	2.66E-01
7.83	7.92	8.00	4.42E-02	2.66E-01
5.10	5.20	5.07	4.42E-02	2.66E-01
6.29	6.30	6.27	4.42E-02	2.66E-01
7.37	7.24	7.18	4.42E-02	2.66E-01
6.17	6.51	6.64	4.43E-02	2.66E-01

10.36	10.40	10.31	4.43E-02	2.66E-01
4.51	4.46	4.42	4.43E-02	2.66E-01
9.37	9.46	9.49	4.43E-02	2.66E-01
8.37	8.49	8.47	4.43E-02	2.66E-01
9.97	10.11	10.03	4.43E-02	2.66E-01
8.25	8.19	8.26	4.43E-02	2.66E-01
5.15	5.25	5.20	4.44E-02	2.66E-01
4.29	4.26	4.33	4.44E-02	2.66E-01
6.93	6.93	7.04	4.44E-02	2.66E-01
8.77	8.81	8.81	4.44E-02	2.66E-01
6.08	5.88	6.03	4.44E-02	2.66E-01
11.71	11.71	11.74	4.45E-02	2.66E-01
8.71	8.80	8.71	4.46E-02	2.66E-01
10.66	10.48	10.58	4.46E-02	2.66E-01
3.87	3.96	4.01	4.46E-02	2.67E-01
7.27	7.42	7.39	4.46E-02	2.67E-01
6.37	6.08	6.49	4.46E-02	2.67E-01
4.41	4.41	4.40	4.47E-02	2.67E-01
7.59	7.56	7.60	4.47E-02	2.67E-01
9.78	9.88	9.79	4.48E-02	2.67E-01
4.16	4.00	4.15	4.48E-02	2.67E-01
8.54	8.22	8.66	4.48E-02	2.67E-01
3.11	3.19	3.10	4.48E-02	2.67E-01
11.38	10.87	10.96	4.48E-02	2.67E-01
7.60	7.68	7.53	4.48E-02	2.67E-01
8.21	8.13	8.26	4.48E-02	2.67E-01
12.83	12.97	12.91	4.49E-02	2.67E-01
7.14	7.12	7.25	4.49E-02	2.67E-01
8.31	8.35	8.52	4.50E-02	2.67E-01
7.73	7.83	7.86	4.50E-02	2.68E-01
11.74	11.93	11.85	4.51E-02	2.68E-01
8.36	8.35	8.53	4.51E-02	2.68E-01
10.64	10.67	10.58	4.51E-02	2.68E-01
9.73	9.72	9.72	4.52E-02	2.68E-01
7.34	7.26	7.26	4.52E-02	2.68E-01
6.13	6.09	6.20	4.52E-02	2.68E-01
7.64	7.93	7.95	4.52E-02	2.68E-01
3.95	3.92	3.73	4.53E-02	2.68E-01
6.88	6.95	6.95	4.53E-02	2.68E-01
5.47	5.40	5.52	4.53E-02	2.68E-01
6.03	6.06	6.00	4.53E-02	2.68E-01
7.97	8.02	8.07	4.54E-02	2.69E-01
6.44	6.49	6.40	4.54E-02	2.69E-01
9.13	9.24	9.24	4.54E-02	2.69E-01
5.44	5.46	5.38	4.55E-02	2.69E-01
4.75	4.85	4.95	4.55E-02	2.69E-01
6.40	6.40	6.42	4.55E-02	2.69E-01

6.45	6.44	6.40	4.55E-02	2.69E-01
10.79	10.66	10.81	4.56E-02	2.69E-01
14.90	14.92	14.93	4.56E-02	2.69E-01
7.75	7.78	7.95	4.56E-02	2.69E-01
8.64	8.82	8.92	4.56E-02	2.69E-01
10.06	9.99	10.09	4.56E-02	2.69E-01
6.65	6.64	6.60	4.56E-02	2.69E-01
8.87	9.01	8.89	4.57E-02	2.69E-01
7.96	8.06	8.07	4.57E-02	2.69E-01
10.30	10.05	10.25	4.57E-02	2.69E-01
9.93	9.93	9.96	4.57E-02	2.69E-01
7.48	7.91	7.51	4.58E-02	2.69E-01
7.99	7.76	8.25	4.58E-02	2.69E-01
5.61	5.72	5.57	4.58E-02	2.69E-01
9.00	8.83	8.99	4.58E-02	2.69E-01
6.31	6.24	6.34	4.58E-02	2.69E-01
4.13	4.07	3.90	4.59E-02	2.69E-01
8.55	8.67	8.64	4.59E-02	2.69E-01
12.26	12.20	12.17	4.59E-02	2.69E-01
6.55	6.45	6.50	4.59E-02	2.69E-01
7.91	7.75	7.81	4.59E-02	2.69E-01
4.10	4.07	4.08	4.59E-02	2.69E-01
11.23	11.33	11.21	4.59E-02	2.69E-01
10.09	9.99	10.13	4.60E-02	2.69E-01
10.06	9.89	9.95	4.60E-02	2.69E-01
5.08	5.12	5.27	4.60E-02	2.69E-01
8.44	8.48	8.67	4.61E-02	2.69E-01
10.57	10.58	10.66	4.61E-02	2.70E-01
6.48	6.33	6.53	4.61E-02	2.70E-01
8.75	8.65	8.69	4.62E-02	2.70E-01
7.21	7.25	7.42	4.62E-02	2.70E-01
7.52	7.53	7.43	4.62E-02	2.70E-01
9.41	9.37	9.37	4.62E-02	2.70E-01
8.04	8.32	8.12	4.62E-02	2.70E-01
5.47	5.22	5.34	4.63E-02	2.70E-01
7.90	7.96	8.02	4.63E-02	2.70E-01
4.88	4.86	4.79	4.64E-02	2.70E-01
6.69	6.52	6.54	4.64E-02	2.70E-01
8.12	7.90	8.11	4.64E-02	2.70E-01
8.51	8.62	8.59	4.64E-02	2.70E-01
5.23	5.47	4.99	4.64E-02	2.70E-01
9.44	9.25	9.43	4.65E-02	2.70E-01
6.03	5.49	5.96	4.65E-02	2.70E-01
7.17	6.96	7.14	4.65E-02	2.70E-01
13.88	13.84	13.89	4.65E-02	2.70E-01
8.95	9.20	9.01	4.66E-02	2.71E-01
9.22	9.35	9.24	4.66E-02	2.71E-01

8.81	8.94	8.98	4.66E-02	2.71E-01
3.49	3.47	3.35	4.66E-02	2.71E-01
5.30	5.36	5.16	4.67E-02	2.71E-01
4.60	4.58	4.51	4.67E-02	2.71E-01
4.29	4.38	4.33	4.67E-02	2.71E-01
7.35	7.37	7.20	4.67E-02	2.71E-01
8.88	9.16	9.08	4.68E-02	2.71E-01
11.03	10.80	10.97	4.69E-02	2.72E-01
7.06	7.22	7.17	4.69E-02	2.72E-01
10.47	10.38	10.54	4.70E-02	2.72E-01
4.72	4.75	4.67	4.70E-02	2.72E-01
7.01	6.86	7.00	4.71E-02	2.72E-01
7.13	7.05	7.26	4.71E-02	2.72E-01
7.08	7.11	7.05	4.71E-02	2.72E-01
6.09	5.96	6.00	4.72E-02	2.72E-01
7.03	7.23	6.95	4.72E-02	2.72E-01
8.26	8.34	8.32	4.73E-02	2.73E-01
7.53	7.37	7.42	4.73E-02	2.73E-01
8.35	8.50	8.55	4.73E-02	2.73E-01
8.96	9.19	9.05	4.73E-02	2.73E-01
10.39	10.63	10.50	4.74E-02	2.73E-01
9.03	9.10	9.06	4.74E-02	2.73E-01
10.51	10.47	10.64	4.74E-02	2.73E-01
7.01	7.05	6.94	4.74E-02	2.73E-01
8.11	8.18	8.22	4.75E-02	2.73E-01
3.86	3.63	3.64	4.75E-02	2.73E-01
8.42	8.70	8.49	4.75E-02	2.73E-01
9.49	9.25	9.68	4.75E-02	2.73E-01
7.06	7.08	7.03	4.75E-02	2.73E-01
5.02	4.62	5.11	4.75E-02	2.73E-01
6.07	6.23	6.07	4.76E-02	2.73E-01
7.23	7.38	7.29	4.76E-02	2.73E-01
9.65	9.80	9.55	4.76E-02	2.73E-01
8.85	9.00	8.97	4.76E-02	2.73E-01
8.64	8.50	8.49	4.77E-02	2.74E-01
10.97	11.04	11.03	4.78E-02	2.74E-01
13.00	12.99	13.05	4.78E-02	2.74E-01
11.63	11.44	11.63	4.78E-02	2.74E-01
11.24	11.16	11.17	4.78E-02	2.74E-01
9.65	9.64	9.63	4.78E-02	2.74E-01
10.03	10.22	10.15	4.79E-02	2.74E-01
10.66	10.50	10.70	4.79E-02	2.74E-01
4.66	4.51	4.71	4.79E-02	2.74E-01
11.60	11.20	11.65	4.79E-02	2.74E-01
8.09	8.16	8.17	4.79E-02	2.74E-01
5.46	5.35	4.96	4.79E-02	2.74E-01
8.00	8.06	7.98	4.80E-02	2.74E-01

6.83	6.95	6.76	4.80E-02	2.74E-01
9.86	9.77	9.86	4.80E-02	2.74E-01
13.77	13.61	13.81	4.80E-02	2.74E-01
10.88	10.58	10.83	4.80E-02	2.74E-01
7.16	7.18	7.21	4.80E-02	2.74E-01
4.77	4.52	4.62	4.81E-02	2.74E-01
13.87	13.75	13.83	4.81E-02	2.74E-01
5.01	4.98	4.91	4.82E-02	2.74E-01
6.07	6.10	6.19	4.82E-02	2.75E-01
8.18	8.05	8.03	4.82E-02	2.75E-01
10.14	10.16	10.12	4.83E-02	2.75E-01
9.89	9.83	9.85	4.83E-02	2.75E-01
8.65	8.64	8.83	4.83E-02	2.75E-01
8.76	8.71	8.75	4.84E-02	2.75E-01
7.08	7.07	7.10	4.84E-02	2.75E-01
6.54	6.75	6.64	4.84E-02	2.75E-01
3.96	4.21	4.32	4.84E-02	2.75E-01
4.28	4.20	4.28	4.84E-02	2.75E-01
8.76	8.84	8.89	4.85E-02	2.75E-01
9.61	9.70	9.67	4.85E-02	2.75E-01
10.14	10.28	10.21	4.85E-02	2.75E-01
6.10	6.09	5.87	4.86E-02	2.75E-01
4.97	4.87	4.88	4.86E-02	2.75E-01
8.96	8.78	8.83	4.87E-02	2.76E-01
11.81	11.91	11.81	4.87E-02	2.76E-01
9.68	9.66	9.77	4.87E-02	2.76E-01
12.49	12.49	12.53	4.88E-02	2.76E-01
3.98	4.13	3.99	4.88E-02	2.76E-01
7.92	7.89	8.19	4.88E-02	2.76E-01
10.35	10.07	10.38	4.89E-02	2.76E-01
9.30	9.30	9.25	4.89E-02	2.76E-01
8.51	8.40	8.46	4.89E-02	2.76E-01
6.92	6.81	6.82	4.89E-02	2.76E-01
3.44	3.47	3.39	4.90E-02	2.77E-01
6.08	6.02	6.00	4.90E-02	2.77E-01
9.34	9.25	9.17	4.91E-02	2.77E-01
10.76	10.60	10.84	4.91E-02	2.77E-01
11.39	11.43	11.46	4.91E-02	2.77E-01
5.64	5.67	5.55	4.91E-02	2.77E-01
8.09	7.71	8.19	4.92E-02	2.77E-01
8.85	8.67	8.81	4.92E-02	2.77E-01
9.22	9.29	9.46	4.92E-02	2.77E-01
8.62	8.57	8.63	4.92E-02	2.77E-01
8.18	8.20	8.07	4.92E-02	2.77E-01
7.81	7.83	7.96	4.92E-02	2.77E-01
10.20	10.34	10.30	4.92E-02	2.77E-01
6.71	6.74	6.87	4.92E-02	2.77E-01

10.32	10.49	10.54	4.92E-02	2.77E-01
10.70	10.52	10.65	4.93E-02	2.77E-01
5.45	5.79	5.58	4.93E-02	2.77E-01
5.31	5.89	5.34	4.93E-02	2.77E-01
11.09	11.16	11.12	4.94E-02	2.77E-01
8.21	8.18	8.30	4.94E-02	2.77E-01
10.48	10.68	10.60	4.94E-02	2.77E-01
9.85	9.77	9.84	4.94E-02	2.77E-01
8.67	8.93	8.69	4.94E-02	2.77E-01
10.11	10.06	10.03	4.94E-02	2.77E-01
3.86	3.68	3.84	4.94E-02	2.77E-01
10.10	10.23	10.26	4.94E-02	2.77E-01
6.07	5.97	6.15	4.95E-02	2.77E-01
9.55	9.58	9.60	4.95E-02	2.77E-01
12.80	12.90	12.83	4.95E-02	2.77E-01
6.93	7.14	7.17	4.96E-02	2.77E-01
12.04	12.05	12.05	4.96E-02	2.77E-01
5.53	5.54	5.58	4.96E-02	2.77E-01
7.63	7.58	7.66	4.96E-02	2.77E-01
7.75	7.99	7.88	4.96E-02	2.77E-01
11.34	11.27	11.31	4.97E-02	2.77E-01
7.89	8.00	7.97	4.97E-02	2.78E-01
7.39	7.11	7.30	4.98E-02	2.78E-01
8.00	7.87	8.04	4.98E-02	2.78E-01
5.69	5.68	5.21	4.98E-02	2.78E-01
5.51	5.53	5.71	4.98E-02	2.78E-01
8.05	8.24	8.05	5.00E-02	2.78E-01
11.18	11.16	11.41	5.00E-02	2.78E-01
9.37	9.55	9.52	5.00E-02	2.78E-01
5.18	5.22	5.20	5.00E-02	2.78E-01
7.31	7.32	7.26	5.00E-02	2.78E-01
9.71	9.40	9.51	5.01E-02	2.78E-01
6.80	6.76	6.90	5.01E-02	2.78E-01
8.74	8.76	8.84	5.01E-02	2.78E-01
11.26	11.27	11.31	5.01E-02	2.78E-01
6.46	6.29	6.39	5.01E-02	2.78E-01
8.00	8.09	8.08	5.01E-02	2.78E-01
5.53	5.59	5.55	5.01E-02	2.78E-01
8.98	9.21	9.16	5.02E-02	2.78E-01
5.81	5.87	5.66	5.02E-02	2.78E-01
9.43	9.52	9.46	5.02E-02	2.78E-01
11.16	11.00	11.17	5.02E-02	2.78E-01
3.98	3.91	3.84	5.02E-02	2.78E-01
8.95	8.91	9.08	5.02E-02	2.78E-01
12.03	12.07	12.02	5.03E-02	2.78E-01
8.40	8.53	8.47	5.03E-02	2.78E-01
9.70	9.82	9.70	5.03E-02	2.78E-01

5.80	5.76	5.85	5.03E-02	2.78E-01
7.49	7.44	7.62	5.03E-02	2.78E-01
6.59	6.56	6.83	5.03E-02	2.78E-01
10.52	10.63	10.60	5.04E-02	2.79E-01
6.92	6.83	6.81	5.05E-02	2.79E-01
11.60	11.49	11.58	5.05E-02	2.79E-01
6.03	6.00	5.97	5.05E-02	2.79E-01
3.68	3.48	3.60	5.05E-02	2.79E-01
10.35	10.59	10.44	5.06E-02	2.79E-01
8.08	8.14	8.17	5.06E-02	2.79E-01
7.58	7.63	7.62	5.06E-02	2.79E-01
6.47	6.32	6.43	5.06E-02	2.79E-01
10.98	10.92	11.00	5.06E-02	2.79E-01
7.12	7.24	7.09	5.06E-02	2.79E-01
7.84	7.63	7.78	5.06E-02	2.79E-01
6.96	7.11	6.91	5.07E-02	2.79E-01
4.69	4.54	4.61	5.07E-02	2.79E-01
9.50	9.62	9.58	5.07E-02	2.79E-01
8.20	8.17	8.15	5.07E-02	2.79E-01
3.47	3.40	3.41	5.08E-02	2.79E-01
4.62	4.71	4.60	5.08E-02	2.79E-01
6.18	6.14	6.34	5.08E-02	2.79E-01
11.21	11.38	11.32	5.09E-02	2.79E-01
4.24	4.11	4.06	5.09E-02	2.79E-01
4.18	4.07	4.08	5.09E-02	2.79E-01
5.82	5.84	6.03	5.09E-02	2.79E-01
7.75	7.62	7.59	5.09E-02	2.79E-01
7.79	7.85	7.97	5.10E-02	2.79E-01
11.17	11.18	11.25	5.10E-02	2.79E-01
9.80	9.98	9.87	5.10E-02	2.79E-01
6.05	5.87	5.94	5.10E-02	2.79E-01
4.46	4.62	4.47	5.10E-02	2.79E-01
6.88	6.99	6.92	5.10E-02	2.79E-01
3.76	3.81	3.90	5.11E-02	2.79E-01
10.29	10.21	10.17	5.11E-02	2.79E-01
9.85	9.57	9.87	5.11E-02	2.79E-01
5.39	5.40	5.56	5.11E-02	2.79E-01
8.66	8.70	8.77	5.11E-02	2.79E-01
8.67	8.78	8.50	5.11E-02	2.79E-01
4.96	5.00	4.69	5.12E-02	2.79E-01
3.87	3.81	3.63	5.12E-02	2.79E-01
8.53	8.46	8.63	5.12E-02	2.79E-01
8.63	8.58	8.62	5.12E-02	2.79E-01
8.15	8.13	8.25	5.13E-02	2.80E-01
5.10	5.10	5.22	5.14E-02	2.80E-01
11.14	11.33	11.23	5.14E-02	2.80E-01
7.67	7.58	7.56	5.15E-02	2.80E-01

13.40	13.44	13.45	5.15E-02	2.80E-01
7.00	7.05	7.06	5.15E-02	2.80E-01
5.65	5.71	5.49	5.15E-02	2.80E-01
15.01	15.07	15.06	5.15E-02	2.80E-01
6.77	6.69	6.59	5.15E-02	2.80E-01
5.89	6.05	6.07	5.16E-02	2.80E-01
3.93	3.92	3.92	5.16E-02	2.80E-01
6.36	6.20	6.46	5.16E-02	2.80E-01
7.26	7.49	7.47	5.16E-02	2.80E-01
8.86	8.97	8.97	5.16E-02	2.80E-01
9.35	9.48	9.32	5.17E-02	2.80E-01
11.96	11.79	11.85	5.17E-02	2.80E-01
5.12	5.13	5.04	5.17E-02	2.80E-01
5.65	5.52	5.62	5.17E-02	2.80E-01
6.36	6.44	6.29	5.18E-02	2.81E-01
4.33	4.32	4.50	5.18E-02	2.81E-01
10.12	10.14	10.17	5.18E-02	2.81E-01
9.68	9.69	9.56	5.18E-02	2.81E-01
9.69	9.97	9.83	5.19E-02	2.81E-01
7.08	7.20	7.17	5.19E-02	2.81E-01
10.61	10.24	10.26	5.19E-02	2.81E-01
8.11	8.30	8.20	5.19E-02	2.81E-01
8.25	8.11	8.33	5.19E-02	2.81E-01
8.34	8.41	8.30	5.20E-02	2.81E-01
9.27	9.48	9.53	5.20E-02	2.81E-01
6.57	6.54	6.49	5.20E-02	2.81E-01
4.68	4.74	4.80	5.21E-02	2.81E-01
9.51	9.63	9.59	5.21E-02	2.81E-01
8.86	8.81	8.87	5.21E-02	2.81E-01
9.11	9.23	9.17	5.21E-02	2.81E-01
8.15	7.96	8.24	5.21E-02	2.81E-01
10.29	10.49	10.35	5.21E-02	2.81E-01
5.32	5.22	5.20	5.21E-02	2.81E-01
8.34	8.44	8.36	5.22E-02	2.81E-01
11.61	11.36	11.60	5.23E-02	2.81E-01
10.02	10.07	9.95	5.23E-02	2.81E-01
8.95	9.35	9.15	5.23E-02	2.81E-01
6.05	6.01	5.90	5.23E-02	2.81E-01
6.01	6.15	5.84	5.23E-02	2.81E-01
7.44	7.44	7.45	5.24E-02	2.81E-01
9.65	9.69	9.74	5.24E-02	2.81E-01
10.07	10.06	10.05	5.24E-02	2.81E-01
6.04	5.95	6.02	5.24E-02	2.81E-01
8.32	8.17	8.34	5.25E-02	2.81E-01
8.37	8.38	8.48	5.25E-02	2.81E-01
10.90	11.07	11.08	5.25E-02	2.82E-01
4.16	4.10	3.80	5.26E-02	2.82E-01

6.20	6.21	6.19	5.26E-02	2.82E-01
8.46	8.14	8.19	5.26E-02	2.82E-01
4.05	4.08	3.73	5.27E-02	2.82E-01
12.53	12.34	12.52	5.28E-02	2.82E-01
8.81	8.69	8.82	5.28E-02	2.83E-01
10.98	10.98	11.00	5.28E-02	2.83E-01
7.34	7.51	7.49	5.28E-02	2.83E-01
7.84	7.86	7.74	5.29E-02	2.83E-01
9.65	9.81	9.72	5.29E-02	2.83E-01
8.19	7.94	8.22	5.29E-02	2.83E-01
9.64	9.68	9.83	5.29E-02	2.83E-01
7.95	8.11	8.00	5.30E-02	2.83E-01
14.99	14.96	14.93	5.30E-02	2.83E-01
14.52	14.58	14.58	5.30E-02	2.83E-01
7.82	7.77	7.79	5.31E-02	2.83E-01
9.10	8.99	9.17	5.31E-02	2.83E-01
10.93	11.23	10.91	5.31E-02	2.83E-01
7.06	6.96	7.16	5.31E-02	2.83E-01
8.02	7.87	7.94	5.31E-02	2.83E-01
6.92	7.08	7.05	5.31E-02	2.83E-01
10.13	10.11	10.01	5.31E-02	2.83E-01
9.22	9.39	9.39	5.31E-02	2.83E-01
5.69	5.96	5.78	5.31E-02	2.83E-01
9.91	9.95	9.95	5.32E-02	2.83E-01
8.14	8.17	8.23	5.32E-02	2.83E-01
10.03	9.92	10.09	5.32E-02	2.83E-01
9.36	9.71	9.51	5.32E-02	2.83E-01
9.35	9.38	9.46	5.32E-02	2.83E-01
9.15	9.11	9.09	5.32E-02	2.83E-01
8.45	8.60	8.56	5.32E-02	2.83E-01
5.72	5.84	5.81	5.33E-02	2.83E-01
9.72	10.07	9.90	5.33E-02	2.83E-01
5.24	5.26	5.34	5.33E-02	2.83E-01
5.22	5.26	5.26	5.33E-02	2.83E-01
9.94	10.02	9.79	5.33E-02	2.83E-01
8.97	9.30	9.16	5.33E-02	2.83E-01
5.01	5.01	5.14	5.35E-02	2.83E-01
9.75	9.77	9.85	5.35E-02	2.83E-01
9.46	9.49	9.49	5.35E-02	2.83E-01
11.91	11.99	11.97	5.35E-02	2.83E-01
9.55	9.42	9.48	5.36E-02	2.83E-01
5.36	5.46	5.21	5.36E-02	2.83E-01
7.85	7.96	7.77	5.36E-02	2.83E-01
6.55	6.69	6.74	5.36E-02	2.83E-01
5.70	5.59	5.52	5.36E-02	2.83E-01
7.39	7.42	7.28	5.36E-02	2.83E-01
3.35	3.37	3.42	5.36E-02	2.83E-01

7.08	6.97	6.99	5.37E-02	2.83E-01
9.05	8.95	9.14	5.37E-02	2.83E-01
7.88	7.84	8.05	5.37E-02	2.83E-01
3.85	3.81	3.79	5.37E-02	2.83E-01
6.92	6.92	6.94	5.37E-02	2.83E-01
4.67	5.05	4.84	5.39E-02	2.84E-01
8.62	8.80	8.63	5.39E-02	2.84E-01
9.48	9.52	9.49	5.39E-02	2.84E-01
7.86	7.69	7.85	5.39E-02	2.84E-01
10.44	10.52	10.51	5.40E-02	2.84E-01
3.69	3.66	3.60	5.40E-02	2.84E-01
10.35	10.48	10.46	5.40E-02	2.84E-01
10.17	10.30	10.36	5.41E-02	2.84E-01
8.83	8.77	8.88	5.41E-02	2.84E-01
7.31	6.93	7.27	5.41E-02	2.84E-01
6.59	6.75	6.59	5.41E-02	2.84E-01
5.79	5.79	5.71	5.41E-02	2.84E-01
9.54	9.44	9.40	5.41E-02	2.84E-01
5.96	6.00	5.81	5.41E-02	2.84E-01
8.16	7.99	8.12	5.41E-02	2.84E-01
7.38	7.32	7.43	5.42E-02	2.84E-01
4.47	4.31	4.53	5.42E-02	2.84E-01
5.93	5.84	6.10	5.42E-02	2.84E-01
10.29	10.22	10.36	5.42E-02	2.84E-01
7.28	7.35	7.38	5.42E-02	2.84E-01
7.49	7.69	7.71	5.43E-02	2.84E-01
5.87	6.07	5.88	5.43E-02	2.84E-01
11.60	11.58	11.61	5.43E-02	2.84E-01
4.18	4.38	4.37	5.43E-02	2.84E-01
3.13	3.31	3.27	5.43E-02	2.84E-01
9.87	9.82	9.92	5.44E-02	2.84E-01
9.37	9.22	9.52	5.44E-02	2.84E-01
10.73	10.68	10.59	5.45E-02	2.85E-01
6.11	6.01	6.03	5.45E-02	2.85E-01
4.35	4.17	4.28	5.46E-02	2.85E-01
4.86	4.83	4.71	5.46E-02	2.85E-01
6.82	6.79	6.73	5.46E-02	2.85E-01
9.94	10.04	10.10	5.46E-02	2.85E-01
6.14	6.16	6.02	5.47E-02	2.85E-01
7.89	8.00	7.88	5.47E-02	2.85E-01
8.62	8.48	8.56	5.47E-02	2.85E-01
7.43	7.36	7.45	5.47E-02	2.85E-01
9.51	9.58	9.45	5.47E-02	2.85E-01
5.64	5.52	5.57	5.47E-02	2.85E-01
5.50	5.60	5.62	5.47E-02	2.85E-01
8.58	8.38	8.47	5.48E-02	2.85E-01
12.38	12.52	12.43	5.48E-02	2.85E-01

4.52	4.56	4.47	5.48E-02	2.85E-01
3.46	3.39	3.45	5.48E-02	2.85E-01
9.27	9.48	9.37	5.49E-02	2.85E-01
4.18	4.01	3.94	5.49E-02	2.85E-01
8.70	8.71	8.74	5.49E-02	2.85E-01
6.80	7.03	6.86	5.49E-02	2.85E-01
9.36	9.35	9.40	5.50E-02	2.85E-01
5.54	5.53	5.71	5.51E-02	2.86E-01
9.07	8.98	9.08	5.51E-02	2.86E-01
15.18	15.20	15.19	5.51E-02	2.86E-01
7.48	7.43	7.51	5.51E-02	2.86E-01
8.04	8.01	8.15	5.51E-02	2.86E-01
3.54	3.45	3.56	5.52E-02	2.86E-01
9.05	9.07	9.14	5.52E-02	2.86E-01
8.57	8.22	8.15	5.52E-02	2.86E-01
6.80	7.03	6.93	5.54E-02	2.86E-01
10.81	10.82	10.74	5.54E-02	2.86E-01
5.84	6.06	6.16	5.54E-02	2.86E-01
10.19	10.45	10.34	5.54E-02	2.86E-01
8.76	8.79	8.77	5.54E-02	2.86E-01
9.76	9.69	9.62	5.54E-02	2.86E-01
6.70	6.82	6.93	5.55E-02	2.86E-01
7.87	7.85	8.03	5.55E-02	2.86E-01
5.00	4.92	4.94	5.55E-02	2.86E-01
8.24	8.26	8.27	5.55E-02	2.86E-01
11.08	10.95	11.13	5.55E-02	2.86E-01
8.02	8.07	8.10	5.56E-02	2.86E-01
8.93	9.08	8.96	5.56E-02	2.87E-01
9.10	9.36	9.29	5.56E-02	2.87E-01
7.30	7.37	7.31	5.56E-02	2.87E-01
7.48	7.28	7.71	5.57E-02	2.87E-01
7.88	7.84	7.80	5.57E-02	2.87E-01
6.84	6.66	6.49	5.57E-02	2.87E-01
4.20	4.07	4.18	5.57E-02	2.87E-01
9.68	9.80	9.63	5.57E-02	2.87E-01
6.07	6.29	6.47	5.58E-02	2.87E-01
4.88	4.99	5.21	5.58E-02	2.87E-01
9.38	9.33	9.51	5.58E-02	2.87E-01
4.96	4.61	4.98	5.58E-02	2.87E-01
6.70	6.77	6.66	5.58E-02	2.87E-01
6.85	6.62	6.92	5.59E-02	2.87E-01
7.12	7.21	7.15	5.59E-02	2.87E-01
9.72	9.70	9.79	5.59E-02	2.87E-01
4.56	4.74	4.67	5.60E-02	2.87E-01
4.30	4.15	4.08	5.60E-02	2.87E-01
9.15	9.27	9.13	5.60E-02	2.87E-01
6.52	6.69	6.67	5.60E-02	2.87E-01

9.73	9.49	9.48	5.60E-02	2.87E-01
8.96	8.50	8.79	5.60E-02	2.87E-01
5.82	5.91	5.97	5.60E-02	2.87E-01
8.23	7.96	8.21	5.61E-02	2.87E-01
5.78	5.81	6.11	5.61E-02	2.87E-01
11.04	11.15	11.17	5.61E-02	2.87E-01
5.47	5.52	5.58	5.61E-02	2.87E-01
7.32	7.31	7.33	5.62E-02	2.87E-01
3.16	3.31	3.36	5.62E-02	2.87E-01
3.80	3.73	3.91	5.62E-02	2.87E-01
9.32	9.51	9.40	5.62E-02	2.87E-01
3.62	3.56	3.56	5.62E-02	2.87E-01
5.59	5.62	5.64	5.63E-02	2.87E-01
11.23	11.15	11.14	5.63E-02	2.87E-01
3.52	3.43	3.72	5.63E-02	2.87E-01
8.12	8.38	8.24	5.63E-02	2.87E-01
8.57	8.40	8.55	5.64E-02	2.87E-01
5.73	5.88	5.81	5.64E-02	2.87E-01
5.18	5.34	5.26	5.64E-02	2.87E-01
8.55	8.78	8.61	5.64E-02	2.87E-01
11.44	11.14	11.34	5.65E-02	2.87E-01
8.51	8.49	8.52	5.65E-02	2.87E-01
9.48	9.64	9.51	5.65E-02	2.87E-01
8.13	8.50	8.30	5.65E-02	2.87E-01
8.45	8.44	8.52	5.66E-02	2.87E-01
6.64	6.70	6.54	5.66E-02	2.87E-01
7.75	7.51	7.59	5.66E-02	2.87E-01
5.28	5.26	5.10	5.66E-02	2.87E-01
8.93	8.80	9.36	5.66E-02	2.87E-01
9.01	9.11	9.01	5.66E-02	2.87E-01
5.40	5.29	5.45	5.67E-02	2.87E-01
4.60	4.69	4.57	5.67E-02	2.87E-01
6.81	6.76	6.77	5.67E-02	2.88E-01
10.61	10.68	10.64	5.68E-02	2.88E-01
7.35	7.22	7.27	5.68E-02	2.88E-01
8.86	8.82	8.86	5.68E-02	2.88E-01
9.28	9.44	9.40	5.68E-02	2.88E-01
4.87	4.92	4.85	5.68E-02	2.88E-01
8.35	7.68	7.88	5.68E-02	2.88E-01
6.94	6.87	6.79	5.69E-02	2.88E-01
3.83	3.78	3.65	5.69E-02	2.88E-01
10.27	10.39	10.41	5.69E-02	2.88E-01
5.65	5.82	5.36	5.69E-02	2.88E-01
9.38	9.30	9.35	5.69E-02	2.88E-01
8.00	7.89	8.01	5.70E-02	2.88E-01
9.42	9.72	9.55	5.70E-02	2.88E-01
10.05	10.06	10.08	5.70E-02	2.88E-01

4.67	4.52	4.74	5.70E-02	2.88E-01
8.64	8.62	8.69	5.71E-02	2.88E-01
10.67	10.70	10.80	5.71E-02	2.88E-01
10.39	10.57	10.56	5.71E-02	2.88E-01
8.76	9.02	8.87	5.72E-02	2.88E-01
4.76	4.94	4.96	5.72E-02	2.88E-01
5.27	5.37	5.44	5.73E-02	2.89E-01
11.98	12.08	12.01	5.74E-02	2.89E-01
7.47	7.33	7.59	5.74E-02	2.89E-01
4.70	4.43	4.50	5.74E-02	2.89E-01
9.65	9.53	9.67	5.74E-02	2.89E-01
6.87	7.19	6.93	5.74E-02	2.89E-01
10.26	10.32	10.29	5.75E-02	2.89E-01
3.52	3.62	3.66	5.76E-02	2.89E-01
8.37	8.09	8.30	5.76E-02	2.89E-01
3.73	3.76	3.67	5.76E-02	2.89E-01
7.78	7.90	7.88	5.76E-02	2.89E-01
3.44	3.27	3.30	5.76E-02	2.89E-01
4.50	4.40	4.39	5.77E-02	2.90E-01
7.71	7.77	7.87	5.77E-02	2.90E-01
5.84	5.63	5.67	5.77E-02	2.90E-01
12.22	12.21	12.27	5.77E-02	2.90E-01
10.30	10.49	10.43	5.78E-02	2.90E-01
8.18	8.14	8.26	5.78E-02	2.90E-01
7.68	7.43	7.50	5.78E-02	2.90E-01
9.29	9.51	9.46	5.78E-02	2.90E-01
3.60	3.74	3.80	5.79E-02	2.90E-01
7.92	7.86	7.91	5.80E-02	2.90E-01
6.72	7.03	6.90	5.80E-02	2.90E-01
10.12	10.29	10.23	5.80E-02	2.90E-01
7.42	7.29	7.39	5.81E-02	2.91E-01
9.04	9.08	9.07	5.81E-02	2.91E-01
5.95	5.78	5.65	5.81E-02	2.91E-01
7.64	7.54	7.52	5.82E-02	2.91E-01
9.59	9.65	9.66	5.82E-02	2.91E-01
7.40	7.63	7.59	5.82E-02	2.91E-01
9.47	9.35	9.27	5.82E-02	2.91E-01
4.58	4.48	4.84	5.82E-02	2.91E-01
8.29	8.35	8.26	5.82E-02	2.91E-01
6.14	6.02	6.10	5.83E-02	2.91E-01
8.66	8.86	8.80	5.83E-02	2.91E-01
8.00	8.01	8.03	5.83E-02	2.91E-01
11.89	12.00	11.95	5.84E-02	2.91E-01
8.83	8.96	8.97	5.84E-02	2.91E-01
5.63	5.75	5.60	5.84E-02	2.91E-01
3.68	3.85	3.79	5.84E-02	2.91E-01
8.61	8.68	8.78	5.84E-02	2.91E-01

4.00	4.01	4.00	5.84E-02	2.91E-01
4.20	4.16	4.08	5.85E-02	2.91E-01
7.86	8.08	7.95	5.85E-02	2.91E-01
7.76	7.81	7.79	5.85E-02	2.91E-01
7.83	7.95	7.90	5.85E-02	2.91E-01
5.62	5.63	5.49	5.86E-02	2.91E-01
5.12	5.30	5.15	5.87E-02	2.91E-01
8.30	8.20	8.18	5.87E-02	2.91E-01
9.57	9.59	9.36	5.87E-02	2.91E-01
9.63	9.38	9.46	5.87E-02	2.91E-01
6.08	6.01	5.94	5.87E-02	2.91E-01
7.99	8.03	8.13	5.87E-02	2.91E-01
9.02	8.95	8.99	5.87E-02	2.91E-01
8.69	8.83	8.74	5.87E-02	2.91E-01
6.88	6.93	7.32	5.87E-02	2.91E-01
5.66	5.71	5.59	5.88E-02	2.91E-01
4.92	4.99	4.98	5.88E-02	2.91E-01
10.93	11.19	10.92	5.88E-02	2.91E-01
5.53	5.48	5.41	5.88E-02	2.91E-01
10.24	10.13	10.21	5.88E-02	2.91E-01
11.28	11.41	11.34	5.88E-02	2.91E-01
5.19	4.87	5.14	5.88E-02	2.91E-01
8.95	9.05	9.10	5.89E-02	2.91E-01
9.44	9.69	9.49	5.89E-02	2.91E-01
5.43	5.23	5.57	5.89E-02	2.91E-01
9.09	9.27	9.25	5.89E-02	2.91E-01
6.62	6.78	6.68	5.90E-02	2.91E-01
3.53	3.62	3.41	5.90E-02	2.91E-01
8.51	8.34	8.33	5.90E-02	2.91E-01
9.69	9.68	9.65	5.90E-02	2.91E-01
9.63	9.62	9.67	5.90E-02	2.91E-01
9.61	9.72	9.68	5.90E-02	2.91E-01
4.46	4.49	4.49	5.91E-02	2.91E-01
12.20	12.42	12.30	5.91E-02	2.91E-01
6.18	6.25	5.88	5.91E-02	2.91E-01
9.27	9.36	9.40	5.92E-02	2.91E-01
9.33	9.44	9.48	5.92E-02	2.91E-01
3.69	3.85	3.61	5.92E-02	2.91E-01
9.47	9.52	9.60	5.92E-02	2.91E-01
4.07	4.21	4.23	5.92E-02	2.91E-01
4.83	4.83	4.64	5.93E-02	2.91E-01
7.41	7.63	7.44	5.93E-02	2.91E-01
8.92	9.28	9.05	5.93E-02	2.91E-01
10.98	10.98	10.93	5.93E-02	2.91E-01
9.67	9.76	9.87	5.94E-02	2.91E-01
10.17	9.98	10.10	5.94E-02	2.91E-01
11.97	11.97	11.91	5.94E-02	2.91E-01

8.07	8.14	8.27	5.95E-02	2.92E-01
4.75	4.83	4.85	5.95E-02	2.92E-01
7.16	7.14	7.39	5.96E-02	2.92E-01
8.77	8.82	8.79	5.96E-02	2.92E-01
7.89	7.81	7.80	5.96E-02	2.92E-01
9.48	9.28	9.52	5.97E-02	2.92E-01
4.10	4.00	4.12	5.97E-02	2.92E-01
8.81	8.87	8.88	5.97E-02	2.92E-01
3.53	3.47	3.52	5.97E-02	2.92E-01
9.01	8.85	9.03	5.99E-02	2.93E-01
8.38	8.50	8.36	5.99E-02	2.93E-01
8.96	8.95	9.03	5.99E-02	2.93E-01
11.47	11.35	11.59	5.99E-02	2.93E-01
7.94	7.96	8.02	5.99E-02	2.93E-01
8.75	8.68	8.60	6.00E-02	2.93E-01
8.54	8.53	8.48	6.00E-02	2.93E-01
7.10	7.37	7.18	6.00E-02	2.93E-01
5.37	5.31	5.32	6.01E-02	2.93E-01
6.53	6.49	6.38	6.01E-02	2.93E-01
12.26	12.24	12.28	6.01E-02	2.93E-01
12.90	12.94	12.89	6.02E-02	2.93E-01
9.98	10.11	10.06	6.02E-02	2.93E-01
3.95	4.20	3.96	6.03E-02	2.94E-01
11.74	11.79	11.76	6.03E-02	2.94E-01
8.63	8.44	8.77	6.03E-02	2.94E-01
10.43	10.41	10.42	6.03E-02	2.94E-01
9.74	9.95	9.90	6.04E-02	2.94E-01
5.44	5.32	5.28	6.04E-02	2.94E-01
4.66	4.69	4.56	6.05E-02	2.94E-01
7.08	7.27	7.19	6.05E-02	2.94E-01
3.28	3.37	3.44	6.06E-02	2.95E-01
13.08	13.16	13.13	6.07E-02	2.95E-01
10.96	10.79	10.87	6.07E-02	2.95E-01
4.49	4.63	4.69	6.07E-02	2.95E-01
6.13	6.23	6.20	6.07E-02	2.95E-01
5.03	5.05	4.94	6.07E-02	2.95E-01
4.55	4.54	4.66	6.08E-02	2.95E-01
3.26	3.39	3.43	6.08E-02	2.95E-01
10.72	10.72	10.71	6.08E-02	2.95E-01
5.90	5.92	5.95	6.09E-02	2.95E-01
8.81	9.12	8.79	6.09E-02	2.95E-01
10.31	10.40	10.37	6.09E-02	2.95E-01
9.21	9.28	9.17	6.09E-02	2.95E-01
3.84	3.63	3.91	6.09E-02	2.95E-01
8.40	8.69	8.64	6.10E-02	2.95E-01
9.21	9.28	9.30	6.10E-02	2.95E-01
6.23	6.46	6.40	6.10E-02	2.95E-01

6.02	6.00	6.16	6.11E-02	2.95E-01
9.97	10.16	10.12	6.11E-02	2.95E-01
5.01	4.83	4.92	6.11E-02	2.96E-01
8.04	8.12	8.17	6.11E-02	2.96E-01
8.81	8.82	8.80	6.12E-02	2.96E-01
5.81	5.68	5.93	6.12E-02	2.96E-01
4.07	3.96	3.82	6.12E-02	2.96E-01
13.04	13.03	13.01	6.13E-02	2.96E-01
7.99	8.21	8.15	6.14E-02	2.96E-01
7.57	7.52	7.58	6.14E-02	2.96E-01
4.38	4.33	4.31	6.14E-02	2.96E-01
11.58	11.62	11.64	6.15E-02	2.96E-01
7.54	7.57	7.55	6.15E-02	2.96E-01
7.35	7.49	7.29	6.15E-02	2.96E-01
3.57	3.66	3.40	6.15E-02	2.96E-01
12.66	12.72	12.70	6.15E-02	2.96E-01
3.26	3.29	3.39	6.16E-02	2.97E-01
3.50	3.61	3.54	6.16E-02	2.97E-01
3.49	3.45	3.43	6.16E-02	2.97E-01
11.42	11.35	11.40	6.16E-02	2.97E-01
8.23	8.42	8.38	6.17E-02	2.97E-01
5.05	5.25	5.20	6.17E-02	2.97E-01
4.65	4.70	4.50	6.17E-02	2.97E-01
7.35	7.32	7.41	6.18E-02	2.97E-01
6.60	6.53	6.81	6.18E-02	2.97E-01
7.84	8.22	8.01	6.18E-02	2.97E-01
9.67	9.82	9.68	6.18E-02	2.97E-01
7.00	7.01	7.16	6.19E-02	2.97E-01
6.38	6.16	6.28	6.19E-02	2.97E-01
5.22	5.34	5.29	6.19E-02	2.97E-01
7.91	7.99	7.97	6.19E-02	2.97E-01
8.49	8.57	8.62	6.19E-02	2.97E-01
8.93	8.79	9.02	6.20E-02	2.97E-01
4.03	4.16	4.27	6.20E-02	2.97E-01
5.49	5.35	5.44	6.20E-02	2.97E-01
9.18	9.33	9.25	6.20E-02	2.97E-01
8.54	8.55	8.73	6.20E-02	2.97E-01
8.77	8.59	8.65	6.21E-02	2.97E-01
7.28	7.26	7.40	6.21E-02	2.97E-01
4.23	4.08	4.17	6.22E-02	2.97E-01
3.76	3.69	3.80	6.22E-02	2.97E-01
10.89	10.96	10.89	6.22E-02	2.97E-01
3.32	3.17	3.06	6.23E-02	2.98E-01
13.24	13.43	13.27	6.23E-02	2.98E-01
11.83	11.73	11.71	6.24E-02	2.98E-01
5.91	5.86	6.15	6.24E-02	2.98E-01
8.81	8.99	8.84	6.24E-02	2.98E-01

4.64	4.63	4.30	6.25E-02	2.98E-01
11.77	11.90	11.77	6.26E-02	2.98E-01
10.86	11.06	10.98	6.26E-02	2.98E-01
9.90	9.95	9.84	6.26E-02	2.98E-01
11.25	11.02	11.07	6.26E-02	2.98E-01
6.48	6.45	6.33	6.26E-02	2.98E-01
7.38	7.52	7.63	6.26E-02	2.98E-01
4.73	4.66	4.87	6.28E-02	2.99E-01
9.00	9.06	8.91	6.28E-02	2.99E-01
7.86	7.88	7.81	6.28E-02	2.99E-01
12.07	12.12	12.02	6.29E-02	2.99E-01
6.21	6.21	6.23	6.29E-02	2.99E-01
8.83	8.69	8.71	6.29E-02	2.99E-01
7.72	8.03	7.70	6.29E-02	2.99E-01
4.20	4.20	3.99	6.29E-02	2.99E-01
6.71	6.62	6.75	6.30E-02	2.99E-01
8.92	8.93	8.94	6.30E-02	2.99E-01
5.93	5.85	5.87	6.30E-02	2.99E-01
5.03	5.29	5.16	6.30E-02	2.99E-01
7.94	7.91	8.01	6.31E-02	2.99E-01
7.39	7.22	7.38	6.31E-02	2.99E-01
7.63	7.58	7.43	6.31E-02	2.99E-01
9.08	9.29	9.17	6.31E-02	2.99E-01
7.87	8.01	8.05	6.31E-02	2.99E-01
10.33	10.32	10.28	6.32E-02	2.99E-01
10.95	10.94	10.93	6.32E-02	2.99E-01
3.63	3.54	3.73	6.33E-02	2.99E-01
5.98	5.76	5.96	6.33E-02	2.99E-01
7.69	7.80	7.73	6.33E-02	2.99E-01
8.66	8.72	8.71	6.33E-02	2.99E-01
13.17	13.05	13.09	6.33E-02	2.99E-01
5.94	6.00	6.11	6.33E-02	2.99E-01
10.16	10.30	10.17	6.33E-02	2.99E-01
7.28	7.13	7.30	6.33E-02	2.99E-01
5.57	5.40	5.34	6.34E-02	2.99E-01
10.24	9.81	10.42	6.34E-02	2.99E-01
8.90	8.94	8.78	6.34E-02	2.99E-01
5.48	5.70	5.81	6.34E-02	2.99E-01
8.95	9.14	9.12	6.35E-02	3.00E-01
10.63	10.63	10.61	6.35E-02	3.00E-01
4.28	4.53	4.43	6.36E-02	3.00E-01
5.71	5.58	5.86	6.36E-02	3.00E-01
8.22	8.37	8.43	6.36E-02	3.00E-01
8.60	8.66	8.65	6.37E-02	3.00E-01
9.99	9.89	10.07	6.37E-02	3.00E-01
11.79	11.75	11.83	6.37E-02	3.00E-01
5.97	6.07	6.16	6.37E-02	3.00E-01

8.05	8.25	8.08	6.38E-02	3.00E-01
13.80	13.84	13.83	6.38E-02	3.00E-01
8.39	8.53	8.54	6.39E-02	3.01E-01
6.50	6.63	6.50	6.40E-02	3.01E-01
6.91	7.01	7.04	6.40E-02	3.01E-01
7.47	7.34	7.41	6.40E-02	3.01E-01
8.06	8.24	8.21	6.41E-02	3.01E-01
9.72	9.55	9.55	6.41E-02	3.01E-01
7.79	7.82	7.71	6.41E-02	3.01E-01
7.79	7.88	7.89	6.42E-02	3.01E-01
10.73	10.90	10.81	6.42E-02	3.01E-01
12.26	12.26	12.27	6.42E-02	3.01E-01
6.03	5.81	5.76	6.43E-02	3.01E-01
4.25	4.16	4.17	6.43E-02	3.02E-01
8.67	8.93	8.78	6.43E-02	3.02E-01
5.90	5.89	5.69	6.44E-02	3.02E-01
4.58	4.60	4.69	6.44E-02	3.02E-01
3.70	3.90	3.88	6.44E-02	3.02E-01
6.42	6.59	6.57	6.44E-02	3.02E-01
4.02	3.88	3.95	6.45E-02	3.02E-01
10.48	10.54	10.60	6.45E-02	3.02E-01
3.58	3.69	3.65	6.45E-02	3.02E-01
4.66	4.77	4.76	6.46E-02	3.02E-01
6.20	6.08	5.96	6.46E-02	3.02E-01
9.52	9.72	9.54	6.46E-02	3.02E-01
4.14	4.45	4.31	6.47E-02	3.03E-01
3.49	3.50	3.50	6.48E-02	3.03E-01
14.32	14.34	14.39	6.48E-02	3.03E-01
10.55	10.59	10.50	6.48E-02	3.03E-01
7.35	7.28	7.55	6.48E-02	3.03E-01
11.44	11.32	11.35	6.48E-02	3.03E-01
5.35	5.06	5.21	6.49E-02	3.03E-01
10.33	10.35	10.34	6.49E-02	3.03E-01
4.10	4.09	4.11	6.49E-02	3.03E-01
6.37	6.71	6.63	6.50E-02	3.03E-01
6.68	6.69	6.42	6.50E-02	3.03E-01
8.17	8.66	8.33	6.50E-02	3.03E-01
4.18	3.98	4.33	6.50E-02	3.03E-01
4.82	5.03	4.73	6.51E-02	3.03E-01
10.57	10.65	10.68	6.51E-02	3.03E-01
12.12	12.30	12.06	6.51E-02	3.03E-01
6.03	6.46	6.32	6.51E-02	3.03E-01
7.03	7.26	7.12	6.52E-02	3.03E-01
8.42	8.44	8.50	6.52E-02	3.03E-01
9.81	9.78	9.88	6.53E-02	3.03E-01
10.41	10.30	10.47	6.53E-02	3.03E-01
4.29	4.22	4.23	6.53E-02	3.03E-01

7.30	7.64	7.61	6.53E-02	3.03E-01
10.13	9.86	10.07	6.53E-02	3.03E-01
5.18	5.32	5.10	6.54E-02	3.03E-01
5.21	4.74	5.07	6.55E-02	3.04E-01
8.15	8.16	8.17	6.55E-02	3.04E-01
3.56	3.59	3.55	6.56E-02	3.04E-01
6.37	6.41	6.31	6.57E-02	3.04E-01
8.25	8.49	8.50	6.57E-02	3.04E-01
9.37	9.60	9.45	6.57E-02	3.04E-01
5.47	5.42	5.48	6.57E-02	3.04E-01
10.95	11.13	11.15	6.57E-02	3.04E-01
9.31	9.41	9.34	6.57E-02	3.04E-01
7.47	7.31	7.49	6.57E-02	3.04E-01
7.42	7.36	7.48	6.57E-02	3.04E-01
8.05	7.91	7.65	6.57E-02	3.04E-01
3.78	3.76	3.87	6.57E-02	3.04E-01
8.06	7.94	8.05	6.58E-02	3.04E-01
9.07	9.20	9.12	6.58E-02	3.04E-01
4.23	4.20	4.36	6.58E-02	3.04E-01
4.46	4.63	4.58	6.59E-02	3.04E-01
9.01	8.87	8.94	6.60E-02	3.04E-01
5.99	5.77	5.92	6.60E-02	3.04E-01
9.66	9.68	9.74	6.60E-02	3.04E-01
5.23	5.14	5.05	6.60E-02	3.04E-01
11.51	11.56	11.65	6.60E-02	3.04E-01
11.24	11.32	11.26	6.60E-02	3.04E-01
7.72	7.83	7.80	6.60E-02	3.04E-01
12.61	12.56	12.64	6.60E-02	3.04E-01
6.38	6.36	6.36	6.61E-02	3.04E-01
6.31	6.21	6.46	6.61E-02	3.04E-01
8.27	8.15	8.31	6.61E-02	3.04E-01
4.16	4.38	4.10	6.61E-02	3.04E-01
5.66	5.73	5.54	6.61E-02	3.04E-01
8.44	8.63	8.52	6.62E-02	3.04E-01
10.33	9.98	10.24	6.62E-02	3.04E-01
7.37	7.55	7.60	6.62E-02	3.04E-01
6.78	6.63	6.64	6.62E-02	3.04E-01
9.32	9.28	9.31	6.62E-02	3.04E-01
10.70	10.72	10.78	6.63E-02	3.04E-01
10.64	10.67	10.70	6.63E-02	3.05E-01
5.78	5.84	5.88	6.64E-02	3.05E-01
4.54	4.43	4.45	6.64E-02	3.05E-01
8.36	8.53	8.41	6.64E-02	3.05E-01
6.03	6.44	6.37	6.64E-02	3.05E-01
7.51	7.72	7.63	6.65E-02	3.05E-01
8.51	8.60	8.51	6.65E-02	3.05E-01
7.16	7.48	7.30	6.65E-02	3.05E-01

8.52	8.53	8.63	6.66E-02	3.05E-01
9.16	9.09	9.14	6.66E-02	3.05E-01
11.24	11.41	11.35	6.66E-02	3.05E-01
7.35	7.30	7.32	6.66E-02	3.05E-01
8.36	8.41	8.38	6.67E-02	3.06E-01
10.24	10.27	10.13	6.68E-02	3.06E-01
10.48	10.39	10.45	6.68E-02	3.06E-01
8.26	7.92	8.19	6.68E-02	3.06E-01
11.19	11.29	11.19	6.68E-02	3.06E-01
7.43	7.75	7.58	6.68E-02	3.06E-01
7.70	7.66	7.67	6.69E-02	3.06E-01
4.18	4.12	4.30	6.69E-02	3.06E-01
8.72	8.78	8.77	6.70E-02	3.06E-01
9.08	9.07	9.24	6.71E-02	3.06E-01
7.53	7.54	7.59	6.71E-02	3.06E-01
9.70	9.71	9.74	6.71E-02	3.06E-01
6.81	6.46	6.63	6.72E-02	3.06E-01
9.98	9.99	9.98	6.72E-02	3.06E-01
9.89	10.02	9.95	6.72E-02	3.06E-01
4.06	4.10	4.18	6.72E-02	3.06E-01
9.26	9.39	9.32	6.72E-02	3.06E-01
8.57	8.42	8.59	6.73E-02	3.06E-01
7.59	7.72	7.83	6.73E-02	3.06E-01
3.51	3.45	3.21	6.73E-02	3.07E-01
7.75	7.83	7.96	6.73E-02	3.07E-01
10.54	10.64	10.57	6.74E-02	3.07E-01
9.56	9.50	9.44	6.74E-02	3.07E-01
8.41	8.24	8.45	6.74E-02	3.07E-01
4.70	4.53	4.43	6.74E-02	3.07E-01
7.35	7.50	7.52	6.75E-02	3.07E-01
4.94	5.22	5.10	6.75E-02	3.07E-01
9.95	10.15	10.04	6.75E-02	3.07E-01
7.87	7.89	7.88	6.76E-02	3.07E-01
11.48	11.51	11.48	6.77E-02	3.07E-01
7.68	7.72	7.76	6.77E-02	3.07E-01
7.05	6.99	7.07	6.77E-02	3.07E-01
5.42	5.35	5.55	6.77E-02	3.07E-01
5.06	5.16	5.03	6.78E-02	3.07E-01
6.98	7.13	7.07	6.78E-02	3.07E-01
7.45	7.18	7.39	6.78E-02	3.07E-01
8.55	8.83	8.69	6.78E-02	3.07E-01
8.90	8.80	8.63	6.78E-02	3.07E-01
10.30	10.17	10.23	6.78E-02	3.07E-01
5.01	5.00	5.13	6.79E-02	3.07E-01
5.70	5.90	5.69	6.79E-02	3.07E-01
9.76	9.88	9.84	6.79E-02	3.07E-01
11.40	11.43	11.40	6.79E-02	3.07E-01

8.73	8.64	8.56	6.79E-02	3.07E-01
7.90	7.86	7.71	6.80E-02	3.07E-01
8.30	8.23	8.36	6.80E-02	3.08E-01
5.24	5.21	5.07	6.80E-02	3.08E-01
7.29	7.19	7.21	6.81E-02	3.08E-01
7.56	7.38	7.48	6.81E-02	3.08E-01
5.16	5.63	5.45	6.82E-02	3.08E-01
10.46	10.57	10.50	6.82E-02	3.08E-01
9.46	9.62	9.56	6.82E-02	3.08E-01
13.72	13.70	13.79	6.83E-02	3.08E-01
13.26	13.19	13.24	6.84E-02	3.08E-01
11.48	11.50	11.56	6.84E-02	3.08E-01
6.71	6.89	6.90	6.84E-02	3.08E-01
3.47	3.61	3.68	6.84E-02	3.08E-01
7.98	7.88	8.00	6.84E-02	3.08E-01
9.04	9.13	9.10	6.84E-02	3.08E-01
6.41	6.48	6.52	6.84E-02	3.08E-01
3.53	3.45	3.45	6.85E-02	3.09E-01
3.43	3.30	3.37	6.85E-02	3.09E-01
10.70	11.01	10.82	6.86E-02	3.09E-01
3.23	3.17	3.36	6.86E-02	3.09E-01
7.68	8.14	7.93	6.87E-02	3.09E-01
7.85	7.65	7.72	6.88E-02	3.09E-01
14.43	14.50	14.43	6.88E-02	3.09E-01
8.39	8.44	8.41	6.88E-02	3.09E-01
11.44	11.34	11.42	6.88E-02	3.09E-01
7.92	7.91	8.04	6.88E-02	3.09E-01
9.13	9.17	9.24	6.89E-02	3.09E-01
6.40	6.30	6.43	6.89E-02	3.09E-01
7.59	7.38	7.55	6.90E-02	3.10E-01
7.36	7.45	7.46	6.90E-02	3.10E-01
10.90	10.97	10.98	6.90E-02	3.10E-01
9.57	9.55	9.65	6.91E-02	3.10E-01
6.79	6.89	6.85	6.92E-02	3.10E-01
3.93	3.57	4.03	6.92E-02	3.10E-01
7.72	7.79	7.84	6.94E-02	3.11E-01
6.06	6.14	6.17	6.94E-02	3.11E-01
4.15	3.83	3.90	6.94E-02	3.11E-01
7.25	7.41	7.25	6.95E-02	3.11E-01
5.70	5.76	5.57	6.95E-02	3.11E-01
4.11	4.03	4.07	6.95E-02	3.11E-01
8.45	8.28	8.26	6.96E-02	3.11E-01
6.96	6.96	7.04	6.96E-02	3.11E-01
3.69	3.56	3.68	6.96E-02	3.11E-01
7.88	7.95	7.95	6.96E-02	3.11E-01
7.59	7.75	7.56	6.96E-02	3.11E-01
6.95	6.84	6.97	6.96E-02	3.11E-01

10.22	10.07	10.22	6.97E-02	3.11E-01
4.70	4.72	4.75	6.97E-02	3.11E-01
7.45	7.67	7.49	6.97E-02	3.11E-01
4.99	5.26	5.14	6.97E-02	3.11E-01
4.37	4.49	4.59	6.97E-02	3.11E-01
5.91	5.79	5.77	6.98E-02	3.11E-01
5.97	5.79	5.75	6.98E-02	3.11E-01
7.01	6.92	7.07	6.98E-02	3.11E-01
11.07	10.99	11.11	6.98E-02	3.11E-01
9.23	9.02	9.27	6.98E-02	3.11E-01
3.92	3.99	3.94	6.99E-02	3.11E-01
8.11	8.08	8.04	6.99E-02	3.11E-01
4.63	4.44	5.01	6.99E-02	3.11E-01
4.99	4.95	4.96	6.99E-02	3.11E-01
4.35	4.36	4.35	7.00E-02	3.11E-01
8.81	8.66	8.84	7.00E-02	3.11E-01
3.32	3.41	3.41	7.00E-02	3.11E-01
3.81	3.71	3.71	7.01E-02	3.11E-01
4.70	4.80	4.95	7.01E-02	3.11E-01
6.65	7.23	7.09	7.01E-02	3.11E-01
8.54	8.24	8.41	7.01E-02	3.11E-01
3.84	3.86	3.92	7.01E-02	3.11E-01
8.66	8.97	8.68	7.02E-02	3.11E-01
6.12	5.91	5.95	7.03E-02	3.12E-01
10.54	10.40	10.56	7.03E-02	3.12E-01
3.49	3.43	3.47	7.05E-02	3.13E-01
5.74	5.80	5.61	7.05E-02	3.13E-01
10.24	10.29	10.23	7.05E-02	3.13E-01
10.56	10.78	10.57	7.06E-02	3.13E-01
7.24	7.37	7.15	7.06E-02	3.13E-01
7.20	6.86	7.20	7.06E-02	3.13E-01
5.84	5.94	5.95	7.06E-02	3.13E-01
9.15	9.16	9.30	7.07E-02	3.13E-01
13.35	13.05	13.18	7.07E-02	3.13E-01
10.87	10.14	10.50	7.07E-02	3.13E-01
10.33	10.46	10.45	7.07E-02	3.13E-01
9.88	9.90	9.90	7.07E-02	3.13E-01
6.45	6.26	6.26	7.07E-02	3.13E-01
6.88	6.78	6.76	7.08E-02	3.13E-01
12.27	12.27	12.31	7.08E-02	3.13E-01
7.68	7.91	7.94	7.08E-02	3.13E-01
7.52	7.68	7.76	7.08E-02	3.13E-01
7.80	7.73	7.83	7.08E-02	3.13E-01
5.07	5.36	5.18	7.08E-02	3.13E-01
10.98	11.06	11.01	7.09E-02	3.13E-01
7.58	7.70	7.66	7.09E-02	3.13E-01
11.01	11.27	11.09	7.09E-02	3.13E-01

8.85	8.87	8.63	7.09E-02	3.13E-01
6.05	6.01	5.96	7.10E-02	3.13E-01
9.19	9.56	9.28	7.11E-02	3.13E-01
4.56	4.76	4.78	7.11E-02	3.13E-01
4.73	4.56	4.75	7.11E-02	3.13E-01
4.54	4.59	4.41	7.11E-02	3.13E-01
8.86	8.97	8.96	7.11E-02	3.13E-01
6.90	7.07	7.17	7.11E-02	3.13E-01
9.13	9.18	9.16	7.12E-02	3.13E-01
9.82	9.82	9.90	7.12E-02	3.13E-01
12.30	12.23	12.34	7.12E-02	3.13E-01
10.87	10.90	10.92	7.12E-02	3.13E-01
6.34	6.33	6.40	7.13E-02	3.13E-01
11.24	11.37	11.28	7.13E-02	3.13E-01
5.98	5.95	5.93	7.14E-02	3.13E-01
8.70	8.48	8.50	7.14E-02	3.13E-01
11.07	11.03	10.96	7.14E-02	3.13E-01
4.40	4.50	4.62	7.14E-02	3.13E-01
8.07	8.10	8.11	7.15E-02	3.14E-01
7.02	7.09	7.13	7.15E-02	3.14E-01
7.57	7.88	7.85	7.16E-02	3.14E-01
11.45	11.50	11.53	7.16E-02	3.14E-01
9.53	9.74	9.64	7.16E-02	3.14E-01
5.10	5.22	5.25	7.16E-02	3.14E-01
8.42	8.44	8.35	7.17E-02	3.14E-01
8.87	8.95	8.71	7.17E-02	3.14E-01
11.58	11.56	11.63	7.18E-02	3.14E-01
7.19	7.36	7.48	7.18E-02	3.14E-01
9.59	9.76	9.68	7.20E-02	3.15E-01
10.56	10.41	10.39	7.20E-02	3.15E-01
7.71	7.75	7.83	7.20E-02	3.15E-01
8.74	8.92	8.76	7.20E-02	3.15E-01
11.00	11.00	10.95	7.22E-02	3.15E-01
5.68	5.80	5.84	7.22E-02	3.16E-01
8.59	8.61	8.63	7.23E-02	3.16E-01
7.45	7.59	7.61	7.23E-02	3.16E-01
4.58	4.53	4.93	7.23E-02	3.16E-01
9.68	9.77	9.78	7.23E-02	3.16E-01
7.91	8.13	7.93	7.23E-02	3.16E-01
9.90	10.10	10.07	7.24E-02	3.16E-01
9.71	9.75	9.74	7.24E-02	3.16E-01
8.16	8.04	7.99	7.24E-02	3.16E-01
9.94	10.07	9.94	7.25E-02	3.16E-01
10.22	10.42	10.30	7.25E-02	3.16E-01
8.76	8.65	8.81	7.25E-02	3.16E-01
8.90	8.92	8.88	7.25E-02	3.16E-01
4.83	4.51	4.74	7.25E-02	3.16E-01

7.48	7.49	7.40	7.25E-02	3.16E-01
11.35	11.69	11.44	7.26E-02	3.16E-01
4.78	4.35	4.49	7.26E-02	3.16E-01
7.39	7.41	7.50	7.26E-02	3.16E-01
12.30	12.26	12.29	7.26E-02	3.16E-01
6.13	6.14	6.17	7.26E-02	3.16E-01
10.23	10.19	10.31	7.27E-02	3.16E-01
10.64	10.92	10.71	7.27E-02	3.16E-01
3.18	3.06	3.09	7.27E-02	3.16E-01
4.18	4.07	4.23	7.28E-02	3.16E-01
7.27	7.37	7.32	7.28E-02	3.16E-01
9.76	10.12	9.93	7.28E-02	3.16E-01
7.66	7.71	7.73	7.29E-02	3.16E-01
5.07	5.08	5.04	7.29E-02	3.16E-01
10.53	10.67	10.66	7.29E-02	3.16E-01
6.70	6.59	6.66	7.29E-02	3.16E-01
3.56	3.64	3.48	7.30E-02	3.16E-01
6.50	6.51	6.37	7.30E-02	3.16E-01
5.12	5.01	5.00	7.30E-02	3.16E-01
5.41	5.31	5.67	7.30E-02	3.16E-01
6.12	6.09	6.07	7.30E-02	3.16E-01
7.11	7.33	7.24	7.31E-02	3.16E-01
10.49	10.47	10.50	7.31E-02	3.16E-01
12.62	12.58	12.63	7.31E-02	3.16E-01
9.81	9.88	9.88	7.31E-02	3.16E-01
7.13	6.99	7.04	7.31E-02	3.16E-01
11.37	11.45	11.38	7.32E-02	3.17E-01
3.63	3.65	3.69	7.33E-02	3.17E-01
4.99	4.92	4.77	7.33E-02	3.17E-01
10.47	10.54	10.45	7.34E-02	3.17E-01
6.69	6.47	6.53	7.34E-02	3.17E-01
5.41	5.48	5.36	7.34E-02	3.17E-01
3.51	3.56	3.45	7.34E-02	3.17E-01
5.42	5.37	5.36	7.34E-02	3.17E-01
9.98	9.98	10.13	7.34E-02	3.17E-01
6.02	6.13	5.44	7.34E-02	3.17E-01
7.86	7.69	7.79	7.35E-02	3.17E-01
6.60	6.69	6.86	7.35E-02	3.17E-01
5.18	5.50	5.30	7.36E-02	3.17E-01
8.25	8.60	8.18	7.36E-02	3.17E-01
4.50	4.52	4.43	7.36E-02	3.17E-01
7.22	7.50	7.62	7.36E-02	3.17E-01
7.64	7.82	7.82	7.36E-02	3.17E-01
5.74	5.58	5.51	7.36E-02	3.17E-01
4.12	4.36	4.40	7.36E-02	3.17E-01
11.10	11.18	11.20	7.36E-02	3.17E-01
3.61	3.61	3.47	7.36E-02	3.17E-01

7.67	7.84	7.71	7.36E-02	3.17E-01
6.53	6.48	6.45	7.37E-02	3.17E-01
6.85	6.88	6.81	7.37E-02	3.17E-01
6.36	6.19	6.40	7.38E-02	3.17E-01
5.73	5.66	5.65	7.38E-02	3.17E-01
6.32	6.34	6.15	7.38E-02	3.17E-01
7.94	8.16	8.02	7.38E-02	3.17E-01
13.59	13.62	13.68	7.39E-02	3.17E-01
11.47	11.64	11.59	7.39E-02	3.17E-01
8.01	8.14	7.99	7.40E-02	3.17E-01
5.29	5.05	4.97	7.40E-02	3.17E-01
10.97	11.04	10.88	7.41E-02	3.18E-01
8.76	9.14	8.95	7.41E-02	3.18E-01
10.33	10.62	10.53	7.41E-02	3.18E-01
9.17	9.00	9.17	7.41E-02	3.18E-01
3.98	3.90	4.01	7.42E-02	3.18E-01
6.62	6.38	6.38	7.42E-02	3.18E-01
7.27	7.34	7.30	7.42E-02	3.18E-01
8.79	8.83	8.94	7.42E-02	3.18E-01
8.14	8.21	8.19	7.43E-02	3.18E-01
3.57	3.71	3.66	7.43E-02	3.18E-01
6.26	6.57	6.47	7.43E-02	3.18E-01
5.20	5.51	5.43	7.44E-02	3.18E-01
7.92	7.94	7.99	7.44E-02	3.18E-01
6.73	6.96	6.76	7.44E-02	3.18E-01
9.83	10.04	9.98	7.44E-02	3.18E-01
9.14	9.21	9.29	7.44E-02	3.18E-01
8.13	8.21	8.34	7.45E-02	3.18E-01
6.29	6.36	6.10	7.45E-02	3.18E-01
6.54	6.58	6.50	7.45E-02	3.18E-01
9.47	9.21	9.69	7.45E-02	3.18E-01
5.91	6.10	6.05	7.46E-02	3.18E-01
4.70	4.80	4.82	7.46E-02	3.18E-01
11.51	11.65	11.51	7.46E-02	3.18E-01
10.97	10.94	10.97	7.47E-02	3.18E-01
10.19	10.46	10.31	7.47E-02	3.18E-01
10.57	10.78	10.72	7.48E-02	3.18E-01
7.22	7.19	7.20	7.48E-02	3.18E-01
3.20	3.30	3.21	7.48E-02	3.18E-01
6.93	7.07	6.98	7.48E-02	3.18E-01
6.10	6.44	6.33	7.49E-02	3.19E-01
3.00	3.10	3.02	7.49E-02	3.19E-01
6.96	6.93	7.15	7.50E-02	3.19E-01
7.20	7.06	7.24	7.50E-02	3.19E-01
8.35	8.44	8.35	7.50E-02	3.19E-01
4.07	4.04	3.85	7.50E-02	3.19E-01
7.44	7.35	7.50	7.51E-02	3.19E-01

6.17	6.43	6.23	7.51E-02	3.19E-01
8.83	8.93	8.86	7.51E-02	3.19E-01
9.59	9.84	9.70	7.51E-02	3.19E-01
9.27	9.20	9.20	7.52E-02	3.19E-01
5.54	5.52	5.55	7.52E-02	3.19E-01
5.98	6.05	5.85	7.52E-02	3.19E-01
6.69	6.72	6.73	7.52E-02	3.19E-01
10.01	10.19	10.10	7.53E-02	3.19E-01
4.08	4.04	4.03	7.53E-02	3.19E-01
7.49	7.58	7.51	7.53E-02	3.19E-01
9.71	9.70	9.86	7.54E-02	3.19E-01
6.79	7.19	7.14	7.55E-02	3.19E-01
6.79	6.70	6.80	7.55E-02	3.19E-01
8.30	8.38	8.43	7.55E-02	3.19E-01
8.54	8.52	8.73	7.55E-02	3.19E-01
7.44	7.32	7.28	7.55E-02	3.19E-01
8.05	8.13	8.17	7.55E-02	3.19E-01
5.82	5.74	5.84	7.55E-02	3.19E-01
11.13	11.23	11.17	7.55E-02	3.19E-01
6.86	6.79	6.82	7.56E-02	3.20E-01
6.83	6.96	6.85	7.56E-02	3.20E-01
3.72	3.78	3.62	7.56E-02	3.20E-01
6.08	6.10	6.13	7.57E-02	3.20E-01
8.02	7.83	7.58	7.57E-02	3.20E-01
7.45	7.35	7.42	7.58E-02	3.20E-01
5.67	5.60	5.70	7.59E-02	3.20E-01
6.23	6.24	6.27	7.59E-02	3.20E-01
5.15	4.91	4.93	7.59E-02	3.20E-01
10.05	9.89	10.08	7.59E-02	3.20E-01
7.94	7.94	7.86	7.60E-02	3.20E-01
7.96	8.01	8.01	7.60E-02	3.20E-01
8.23	8.07	8.26	7.60E-02	3.20E-01
8.73	8.27	8.60	7.61E-02	3.20E-01
6.89	6.83	6.93	7.61E-02	3.20E-01
3.60	3.58	3.64	7.61E-02	3.20E-01
9.20	9.28	9.28	7.62E-02	3.21E-01
9.10	9.35	9.23	7.63E-02	3.21E-01
3.92	3.92	4.00	7.63E-02	3.21E-01
7.86	8.15	7.98	7.63E-02	3.21E-01
8.39	8.40	8.58	7.64E-02	3.21E-01
4.76	5.12	5.08	7.64E-02	3.21E-01
11.04	10.94	10.87	7.64E-02	3.21E-01
5.40	5.30	5.21	7.65E-02	3.21E-01
6.10	6.12	6.08	7.65E-02	3.21E-01
6.83	6.92	6.63	7.65E-02	3.21E-01
3.24	3.43	3.14	7.65E-02	3.21E-01
6.06	5.98	6.11	7.66E-02	3.21E-01

9.51	9.69	9.63	7.66E-02	3.21E-01
9.28	9.36	9.15	7.66E-02	3.21E-01
10.37	10.58	10.44	7.66E-02	3.21E-01
8.18	8.59	8.27	7.67E-02	3.22E-01
5.86	6.17	6.02	7.67E-02	3.22E-01
3.97	4.22	4.12	7.68E-02	3.22E-01
7.93	7.61	7.63	7.68E-02	3.22E-01
7.31	7.33	7.35	7.68E-02	3.22E-01
6.90	6.96	6.93	7.68E-02	3.22E-01
6.21	6.27	6.17	7.68E-02	3.22E-01
5.93	5.74	5.88	7.69E-02	3.22E-01
8.23	8.02	8.05	7.69E-02	3.22E-01
7.23	7.33	7.43	7.69E-02	3.22E-01
9.22	9.49	9.36	7.69E-02	3.22E-01
5.67	5.56	5.58	7.70E-02	3.22E-01
9.69	9.60	9.66	7.70E-02	3.22E-01
5.44	5.24	5.66	7.70E-02	3.22E-01
4.00	3.96	4.07	7.70E-02	3.22E-01
9.34	9.54	9.51	7.70E-02	3.22E-01
8.66	8.72	8.66	7.71E-02	3.22E-01
4.61	4.63	4.76	7.71E-02	3.22E-01
11.59	11.65	11.57	7.71E-02	3.22E-01
3.73	3.71	3.98	7.71E-02	3.22E-01
7.02	7.25	7.28	7.72E-02	3.22E-01
8.46	8.38	8.57	7.72E-02	3.22E-01
9.74	9.77	9.84	7.73E-02	3.22E-01
9.39	9.57	9.49	7.73E-02	3.22E-01
9.90	9.69	9.94	7.74E-02	3.22E-01
5.81	5.99	5.88	7.74E-02	3.22E-01
8.77	8.91	8.82	7.74E-02	3.22E-01
5.51	5.40	5.36	7.74E-02	3.22E-01
10.56	10.79	10.60	7.75E-02	3.22E-01
4.07	4.04	3.89	7.75E-02	3.22E-01
8.03	7.96	7.98	7.75E-02	3.22E-01
9.73	9.74	9.96	7.75E-02	3.22E-01
5.45	5.50	5.36	7.75E-02	3.22E-01
9.90	9.96	10.06	7.76E-02	3.23E-01
8.57	8.77	8.53	7.76E-02	3.23E-01
3.29	3.35	3.33	7.76E-02	3.23E-01
10.85	10.80	10.86	7.77E-02	3.23E-01
5.31	5.47	5.30	7.77E-02	3.23E-01
13.02	12.76	12.84	7.78E-02	3.23E-01
8.73	8.58	8.66	7.78E-02	3.23E-01
3.83	3.93	4.03	7.78E-02	3.23E-01
8.15	7.85	8.13	7.78E-02	3.23E-01
9.21	9.38	9.29	7.79E-02	3.23E-01
12.06	12.13	12.10	7.80E-02	3.23E-01

7.97	7.97	7.97	7.80E-02	3.23E-01
11.85	11.69	11.84	7.80E-02	3.23E-01
6.64	6.62	6.57	7.80E-02	3.23E-01
3.40	3.40	3.38	7.81E-02	3.23E-01
6.33	6.17	6.09	7.81E-02	3.23E-01
7.10	7.16	7.16	7.81E-02	3.23E-01
3.67	3.84	3.70	7.81E-02	3.23E-01
9.58	9.72	9.70	7.82E-02	3.23E-01
5.79	5.63	5.97	7.82E-02	3.23E-01
10.04	10.22	10.02	7.82E-02	3.23E-01
5.53	5.19	5.33	7.82E-02	3.23E-01
9.28	8.98	9.09	7.82E-02	3.23E-01
4.40	4.17	4.29	7.82E-02	3.23E-01
7.04	6.85	7.03	7.83E-02	3.24E-01
12.52	12.49	12.48	7.83E-02	3.24E-01
6.53	6.60	6.59	7.83E-02	3.24E-01
4.03	4.21	4.20	7.84E-02	3.24E-01
8.46	8.44	8.53	7.84E-02	3.24E-01
7.40	7.39	7.34	7.85E-02	3.24E-01
11.03	10.98	11.03	7.85E-02	3.24E-01
6.83	6.47	6.67	7.85E-02	3.24E-01
9.61	9.57	9.64	7.85E-02	3.24E-01
9.27	9.50	9.35	7.86E-02	3.24E-01
11.46	11.60	11.38	7.86E-02	3.24E-01
8.01	7.92	8.23	7.86E-02	3.24E-01
4.42	4.49	4.22	7.87E-02	3.24E-01
9.64	9.92	9.67	7.87E-02	3.24E-01
6.33	6.86	6.27	7.87E-02	3.24E-01
4.64	4.68	4.75	7.87E-02	3.24E-01
7.77	7.84	7.95	7.87E-02	3.24E-01
7.84	8.09	7.97	7.87E-02	3.24E-01
12.46	12.66	12.66	7.87E-02	3.24E-01
7.71	7.64	7.79	7.87E-02	3.24E-01
4.70	4.68	4.84	7.88E-02	3.24E-01
7.62	7.74	7.83	7.88E-02	3.24E-01
4.67	5.07	5.09	7.88E-02	3.24E-01
6.37	6.40	6.42	7.88E-02	3.24E-01
6.34	6.13	6.14	7.89E-02	3.24E-01
8.34	8.49	8.45	7.89E-02	3.24E-01
9.93	9.85	9.94	7.89E-02	3.24E-01
5.55	5.30	5.37	7.90E-02	3.24E-01
9.59	9.54	9.60	7.90E-02	3.24E-01
7.00	7.25	7.14	7.91E-02	3.24E-01
7.01	6.81	6.91	7.91E-02	3.24E-01
3.18	3.21	3.26	7.91E-02	3.24E-01
3.78	3.60	4.00	7.92E-02	3.24E-01
7.53	7.62	7.50	7.92E-02	3.24E-01

6.81	7.09	7.04	7.92E-02	3.24E-01
3.43	3.42	3.42	7.92E-02	3.24E-01
8.88	8.92	8.98	7.92E-02	3.24E-01
8.88	8.93	9.16	7.92E-02	3.24E-01
5.19	5.20	5.39	7.93E-02	3.25E-01
8.86	8.84	8.93	7.93E-02	3.25E-01
6.63	6.65	6.55	7.93E-02	3.25E-01
4.34	4.33	4.48	7.93E-02	3.25E-01
5.69	5.60	5.67	7.94E-02	3.25E-01
3.64	3.89	3.69	7.95E-02	3.25E-01
9.12	8.98	8.99	7.95E-02	3.25E-01
8.97	9.30	9.13	7.95E-02	3.25E-01
6.80	6.90	6.92	7.95E-02	3.25E-01
9.66	9.69	9.61	7.95E-02	3.25E-01
8.07	7.88	8.00	7.96E-02	3.25E-01
7.42	7.73	7.46	7.96E-02	3.25E-01
7.10	7.06	6.99	7.96E-02	3.25E-01
3.60	3.63	3.54	7.96E-02	3.25E-01
3.61	3.71	3.76	7.96E-02	3.25E-01
10.02	10.14	10.01	7.97E-02	3.25E-01
3.51	3.44	3.57	7.97E-02	3.25E-01
10.66	10.83	10.73	7.97E-02	3.25E-01
9.52	9.78	9.61	7.97E-02	3.25E-01
6.83	6.67	6.75	7.97E-02	3.25E-01
6.90	6.68	7.31	7.97E-02	3.25E-01
6.32	6.52	6.48	7.97E-02	3.25E-01
7.87	8.11	7.97	7.97E-02	3.25E-01
3.28	3.39	3.36	7.98E-02	3.25E-01
6.27	6.28	6.42	7.98E-02	3.25E-01
10.77	10.95	10.82	7.98E-02	3.25E-01
13.74	13.74	13.71	7.98E-02	3.25E-01
6.84	6.49	6.74	7.99E-02	3.25E-01
12.77	12.83	12.81	7.99E-02	3.25E-01
8.96	8.69	8.88	7.99E-02	3.25E-01
6.43	6.12	6.21	7.99E-02	3.25E-01
9.82	9.92	10.02	8.01E-02	3.25E-01
12.79	12.89	12.85	8.01E-02	3.25E-01
7.69	7.89	7.52	8.01E-02	3.25E-01
5.20	5.25	5.30	8.01E-02	3.25E-01
4.12	3.89	4.12	8.01E-02	3.25E-01
4.34	4.38	4.18	8.02E-02	3.25E-01
7.96	7.96	7.99	8.02E-02	3.25E-01
11.14	11.27	11.22	8.02E-02	3.25E-01
7.39	7.25	7.32	8.02E-02	3.25E-01
5.57	5.73	5.64	8.02E-02	3.25E-01
9.91	9.66	9.88	8.03E-02	3.25E-01
9.73	9.52	9.71	8.03E-02	3.25E-01

5.03	4.81	4.92	8.03E-02	3.25E-01
8.51	8.45	8.42	8.03E-02	3.25E-01
4.62	4.72	4.64	8.04E-02	3.25E-01
8.55	8.39	8.55	8.04E-02	3.25E-01
5.42	5.41	5.27	8.04E-02	3.25E-01
8.11	8.11	8.09	8.04E-02	3.25E-01
4.92	5.22	5.12	8.05E-02	3.26E-01
6.44	6.48	6.58	8.05E-02	3.26E-01
4.30	4.46	4.37	8.07E-02	3.26E-01
9.96	10.05	9.92	8.07E-02	3.26E-01
7.90	8.15	8.19	8.08E-02	3.26E-01
5.76	5.96	5.73	8.08E-02	3.26E-01
9.49	9.36	9.37	8.08E-02	3.26E-01
6.03	5.92	6.12	8.08E-02	3.26E-01
12.30	12.32	12.35	8.08E-02	3.26E-01
6.46	6.28	6.38	8.09E-02	3.26E-01
10.86	10.67	10.75	8.09E-02	3.26E-01
10.74	10.71	10.82	8.11E-02	3.27E-01
3.42	3.56	3.72	8.11E-02	3.27E-01
9.27	9.52	9.33	8.11E-02	3.27E-01
10.19	10.28	10.30	8.12E-02	3.27E-01
6.29	6.24	6.20	8.13E-02	3.27E-01
4.37	4.51	4.63	8.13E-02	3.27E-01
6.17	6.23	6.13	8.14E-02	3.27E-01
6.76	7.00	6.83	8.14E-02	3.27E-01
10.34	10.15	10.15	8.14E-02	3.27E-01
6.51	6.54	6.26	8.14E-02	3.27E-01
9.65	9.66	9.68	8.14E-02	3.27E-01
9.45	9.31	9.40	8.14E-02	3.27E-01
4.52	4.44	4.45	8.15E-02	3.28E-01
6.54	6.38	6.74	8.15E-02	3.28E-01
9.79	9.86	9.78	8.16E-02	3.28E-01
9.49	9.66	9.53	8.16E-02	3.28E-01
3.99	3.99	4.12	8.16E-02	3.28E-01
3.92	4.00	4.04	8.17E-02	3.28E-01
9.26	9.21	9.29	8.17E-02	3.28E-01
8.58	8.63	8.61	8.17E-02	3.28E-01
3.27	3.19	3.16	8.17E-02	3.28E-01
9.86	9.72	9.65	8.17E-02	3.28E-01
9.99	10.12	10.13	8.17E-02	3.28E-01
8.29	8.15	8.26	8.18E-02	3.28E-01
3.55	3.44	3.53	8.18E-02	3.28E-01
10.35	10.27	10.28	8.18E-02	3.28E-01
4.79	4.89	4.85	8.18E-02	3.28E-01
7.41	7.47	7.56	8.18E-02	3.28E-01
12.19	12.26	12.27	8.18E-02	3.28E-01
7.51	7.52	7.44	8.18E-02	3.28E-01

7.48	7.29	7.42	8.19E-02	3.28E-01
7.38	7.25	7.37	8.19E-02	3.28E-01
4.58	4.58	5.02	8.20E-02	3.28E-01
4.53	4.67	4.71	8.20E-02	3.28E-01
4.81	4.99	4.98	8.20E-02	3.28E-01
6.18	6.61	6.41	8.20E-02	3.28E-01
9.35	9.53	9.43	8.20E-02	3.28E-01
4.46	4.41	4.43	8.21E-02	3.28E-01
3.83	3.92	3.84	8.21E-02	3.28E-01
7.83	7.82	7.80	8.21E-02	3.28E-01
5.22	5.37	5.14	8.21E-02	3.28E-01
8.13	8.06	8.08	8.21E-02	3.28E-01
5.88	6.04	5.88	8.22E-02	3.28E-01
8.81	8.69	8.78	8.22E-02	3.28E-01
8.82	8.59	8.71	8.22E-02	3.28E-01
8.79	8.90	8.90	8.22E-02	3.28E-01
7.42	7.35	7.35	8.23E-02	3.28E-01
8.81	9.03	8.94	8.23E-02	3.28E-01
3.50	3.50	3.56	8.23E-02	3.28E-01
9.89	9.94	10.07	8.24E-02	3.28E-01
8.85	8.55	8.67	8.24E-02	3.28E-01
8.52	8.51	8.53	8.25E-02	3.28E-01
7.18	7.08	7.07	8.25E-02	3.28E-01
4.46	4.50	4.50	8.25E-02	3.28E-01
4.59	4.65	4.64	8.25E-02	3.28E-01
8.57	8.77	8.54	8.25E-02	3.28E-01
8.84	9.05	8.91	8.26E-02	3.28E-01
8.58	8.49	8.68	8.26E-02	3.28E-01
4.23	4.21	4.21	8.26E-02	3.28E-01
7.09	7.14	7.14	8.26E-02	3.28E-01
11.88	12.01	11.93	8.26E-02	3.28E-01
7.50	7.66	7.42	8.27E-02	3.29E-01
9.25	9.07	9.18	8.27E-02	3.29E-01
7.15	7.31	7.45	8.27E-02	3.29E-01
5.35	5.34	5.44	8.28E-02	3.29E-01
6.16	6.23	6.16	8.28E-02	3.29E-01
5.78	5.68	5.62	8.28E-02	3.29E-01
6.74	7.09	6.94	8.28E-02	3.29E-01
7.60	7.19	7.81	8.29E-02	3.29E-01
3.71	3.44	3.29	8.29E-02	3.29E-01
4.58	4.47	4.49	8.29E-02	3.29E-01
8.93	9.11	9.25	8.29E-02	3.29E-01
6.26	6.37	6.61	8.30E-02	3.29E-01
5.64	5.70	5.57	8.31E-02	3.29E-01
11.21	11.20	11.22	8.31E-02	3.29E-01
5.59	5.54	5.42	8.31E-02	3.29E-01
6.41	6.66	6.70	8.32E-02	3.29E-01

6.71	6.83	6.75	8.32E-02	3.29E-01
7.84	7.80	7.94	8.32E-02	3.29E-01
7.22	7.40	7.36	8.32E-02	3.29E-01
3.30	3.21	3.24	8.32E-02	3.29E-01
7.88	7.98	8.06	8.32E-02	3.29E-01
6.61	6.63	6.70	8.33E-02	3.29E-01
5.33	5.27	5.29	8.33E-02	3.29E-01
9.97	9.91	10.05	8.33E-02	3.29E-01
7.01	6.83	6.91	8.33E-02	3.29E-01
9.04	9.00	9.13	8.33E-02	3.29E-01
11.51	11.59	11.59	8.34E-02	3.29E-01
10.22	10.37	10.28	8.35E-02	3.29E-01
8.30	8.39	8.44	8.35E-02	3.29E-01
8.08	8.23	8.31	8.35E-02	3.29E-01
10.50	10.31	10.50	8.35E-02	3.29E-01
3.37	3.24	3.39	8.36E-02	3.30E-01
7.49	7.46	7.50	8.37E-02	3.30E-01
11.51	11.57	11.54	8.37E-02	3.30E-01
7.22	7.13	6.86	8.37E-02	3.30E-01
9.23	9.37	9.26	8.38E-02	3.30E-01
11.75	11.87	11.78	8.38E-02	3.30E-01
4.75	4.89	4.72	8.38E-02	3.30E-01
4.96	4.74	4.76	8.39E-02	3.30E-01
5.30	5.19	5.36	8.39E-02	3.30E-01
6.73	6.65	6.66	8.39E-02	3.30E-01
6.36	6.72	6.46	8.41E-02	3.31E-01
8.22	8.09	8.13	8.41E-02	3.31E-01
8.70	8.72	8.60	8.41E-02	3.31E-01
9.28	9.25	9.31	8.41E-02	3.31E-01
6.12	6.03	6.01	8.41E-02	3.31E-01
8.88	9.11	9.01	8.42E-02	3.31E-01
3.36	3.29	3.24	8.42E-02	3.31E-01
9.26	9.00	9.14	8.43E-02	3.31E-01
6.57	6.51	6.62	8.43E-02	3.31E-01
7.57	7.47	7.62	8.43E-02	3.31E-01
8.82	9.01	8.93	8.43E-02	3.31E-01
9.69	9.92	9.68	8.43E-02	3.31E-01
6.84	6.78	7.08	8.43E-02	3.31E-01
10.46	10.31	10.39	8.44E-02	3.31E-01
6.08	6.04	6.16	8.45E-02	3.31E-01
7.95	7.98	7.88	8.45E-02	3.31E-01
8.39	8.28	8.58	8.46E-02	3.32E-01
9.02	8.85	9.00	8.47E-02	3.32E-01
3.46	3.38	3.33	8.48E-02	3.32E-01
6.43	6.36	6.47	8.48E-02	3.32E-01
10.36	10.37	10.23	8.48E-02	3.32E-01
10.12	10.21	10.25	8.49E-02	3.32E-01

11.88	12.44	11.85	8.49E-02	3.32E-01
7.98	7.93	8.01	8.49E-02	3.32E-01
7.51	7.61	7.70	8.49E-02	3.32E-01
5.50	5.62	5.54	8.50E-02	3.32E-01
12.64	12.78	12.68	8.50E-02	3.32E-01
4.67	4.62	4.67	8.52E-02	3.32E-01
8.96	8.91	8.82	8.52E-02	3.32E-01
6.19	6.19	6.29	8.52E-02	3.32E-01
3.52	3.66	3.56	8.52E-02	3.32E-01
10.63	10.37	10.53	8.52E-02	3.32E-01
10.28	10.42	10.39	8.52E-02	3.32E-01
4.36	4.14	4.30	8.52E-02	3.32E-01
9.12	9.38	9.15	8.53E-02	3.32E-01
8.71	8.53	8.75	8.53E-02	3.32E-01
10.10	9.96	10.11	8.53E-02	3.32E-01
13.41	13.35	13.42	8.53E-02	3.32E-01
10.59	10.69	10.65	8.53E-02	3.32E-01
5.22	5.25	5.58	8.53E-02	3.33E-01
8.88	8.91	9.00	8.54E-02	3.33E-01
7.47	7.86	7.55	8.54E-02	3.33E-01
6.32	6.57	6.55	8.54E-02	3.33E-01
7.92	8.06	8.07	8.54E-02	3.33E-01
8.72	8.65	8.71	8.54E-02	3.33E-01
3.32	3.43	3.38	8.54E-02	3.33E-01
3.56	3.58	3.56	8.55E-02	3.33E-01
8.43	8.63	8.61	8.55E-02	3.33E-01
8.19	8.40	8.41	8.56E-02	3.33E-01
4.99	4.84	4.97	8.56E-02	3.33E-01
5.18	5.15	5.16	8.56E-02	3.33E-01
12.09	12.32	12.19	8.56E-02	3.33E-01
8.62	8.77	8.68	8.57E-02	3.33E-01
3.67	3.63	3.58	8.57E-02	3.33E-01
11.02	10.95	11.02	8.57E-02	3.33E-01
7.59	7.68	7.74	8.57E-02	3.33E-01
7.59	7.52	7.64	8.57E-02	3.33E-01
5.63	5.45	5.22	8.58E-02	3.33E-01
9.02	8.96	9.07	8.58E-02	3.33E-01
10.94	10.80	10.90	8.58E-02	3.33E-01
7.08	7.07	7.22	8.58E-02	3.33E-01
5.81	5.77	5.99	8.58E-02	3.33E-01
9.18	9.28	9.35	8.59E-02	3.33E-01
6.02	5.94	6.24	8.59E-02	3.33E-01
7.47	7.48	7.49	8.59E-02	3.33E-01
8.98	9.08	9.14	8.59E-02	3.33E-01
7.37	7.48	7.50	8.60E-02	3.33E-01
7.91	7.94	8.15	8.60E-02	3.33E-01
9.01	9.13	9.12	8.61E-02	3.33E-01

12.17	12.05	12.19	8.62E-02	3.33E-01
4.98	5.14	5.09	8.62E-02	3.33E-01
5.14	4.86	4.68	8.62E-02	3.33E-01
5.55	5.38	5.58	8.62E-02	3.33E-01
7.60	8.06	7.96	8.62E-02	3.33E-01
9.00	9.27	9.25	8.63E-02	3.33E-01
10.74	10.77	10.92	8.63E-02	3.33E-01
4.83	4.61	4.52	8.63E-02	3.33E-01
7.17	7.03	7.12	8.64E-02	3.33E-01
5.90	5.96	6.08	8.64E-02	3.33E-01
8.57	8.76	8.68	8.64E-02	3.33E-01
9.80	9.83	9.86	8.65E-02	3.33E-01
9.78	9.67	9.77	8.65E-02	3.33E-01
6.20	6.34	6.16	8.65E-02	3.33E-01
4.39	4.46	4.39	8.65E-02	3.33E-01
6.04	5.98	5.93	8.65E-02	3.33E-01
6.80	6.83	6.76	8.65E-02	3.33E-01
8.37	8.36	8.38	8.66E-02	3.33E-01
10.64	10.49	10.59	8.66E-02	3.33E-01
5.52	5.59	5.52	8.66E-02	3.33E-01
7.30	7.29	7.12	8.66E-02	3.33E-01
8.62	8.69	8.66	8.66E-02	3.33E-01
10.67	10.67	10.74	8.66E-02	3.33E-01
10.38	10.28	10.34	8.66E-02	3.33E-01
10.23	10.21	10.22	8.66E-02	3.33E-01
11.58	11.85	11.71	8.67E-02	3.33E-01
6.53	6.42	6.52	8.67E-02	3.33E-01
6.78	6.80	6.82	8.68E-02	3.34E-01
5.56	5.84	5.61	8.68E-02	3.34E-01
6.18	6.33	6.22	8.68E-02	3.34E-01
7.92	7.96	7.88	8.69E-02	3.34E-01
5.98	5.82	5.81	8.69E-02	3.34E-01
7.09	7.29	7.21	8.70E-02	3.34E-01
9.22	9.36	9.30	8.70E-02	3.34E-01
10.06	10.19	10.02	8.70E-02	3.34E-01
9.99	10.00	10.00	8.71E-02	3.34E-01
8.63	8.70	8.66	8.72E-02	3.35E-01
9.93	9.77	10.03	8.73E-02	3.35E-01
7.99	8.01	8.12	8.73E-02	3.35E-01
9.81	9.76	9.80	8.74E-02	3.35E-01
7.75	7.73	7.70	8.74E-02	3.35E-01
6.12	6.25	6.19	8.75E-02	3.35E-01
4.08	3.86	4.19	8.75E-02	3.35E-01
4.62	4.76	4.75	8.75E-02	3.35E-01
5.34	5.21	5.42	8.75E-02	3.35E-01
4.31	4.42	4.65	8.76E-02	3.35E-01
6.95	7.03	6.91	8.76E-02	3.35E-01

6.54	6.39	6.40	8.76E-02	3.35E-01
9.96	9.66	9.95	8.76E-02	3.35E-01
10.38	10.17	10.41	8.76E-02	3.35E-01
4.30	4.44	4.37	8.77E-02	3.35E-01
3.38	3.44	3.47	8.77E-02	3.35E-01
8.04	8.03	7.96	8.77E-02	3.35E-01
6.14	6.19	6.25	8.78E-02	3.35E-01
9.05	9.01	8.93	8.78E-02	3.35E-01
6.51	6.64	6.57	8.78E-02	3.35E-01
6.92	6.95	6.86	8.79E-02	3.36E-01
10.76	10.67	10.83	8.79E-02	3.36E-01
4.80	4.62	4.59	8.80E-02	3.36E-01
8.04	8.00	8.04	8.80E-02	3.36E-01
8.78	8.89	8.85	8.80E-02	3.36E-01
5.62	5.71	5.74	8.80E-02	3.36E-01
10.26	10.44	10.33	8.80E-02	3.36E-01
11.84	11.59	11.77	8.81E-02	3.36E-01
7.60	7.60	7.71	8.81E-02	3.36E-01
9.58	9.53	9.56	8.81E-02	3.36E-01
5.20	5.06	5.16	8.82E-02	3.36E-01
6.96	7.20	6.99	8.82E-02	3.36E-01
13.46	13.55	13.51	8.83E-02	3.36E-01
7.36	7.29	7.49	8.83E-02	3.36E-01
6.10	6.07	6.02	8.83E-02	3.36E-01
6.06	5.94	6.44	8.83E-02	3.36E-01
12.62	12.64	12.69	8.84E-02	3.36E-01
3.41	3.24	3.47	8.84E-02	3.36E-01
8.81	9.05	8.97	8.84E-02	3.36E-01
5.04	4.90	5.21	8.84E-02	3.36E-01
8.42	8.31	8.51	8.84E-02	3.36E-01
3.44	3.68	3.57	8.84E-02	3.36E-01
8.59	8.86	8.71	8.85E-02	3.36E-01
4.60	4.70	4.47	8.85E-02	3.36E-01
10.54	10.63	10.64	8.85E-02	3.36E-01
12.70	12.78	12.76	8.86E-02	3.36E-01
7.14	7.21	7.29	8.86E-02	3.36E-01
11.00	10.83	11.02	8.87E-02	3.37E-01
7.68	7.53	7.56	8.88E-02	3.37E-01
7.23	7.27	7.20	8.89E-02	3.37E-01
8.60	8.69	8.75	8.89E-02	3.37E-01
5.81	5.73	5.81	8.89E-02	3.37E-01
4.22	4.15	4.13	8.89E-02	3.37E-01
13.47	13.54	13.53	8.90E-02	3.37E-01
8.89	8.64	8.66	8.91E-02	3.38E-01
4.78	4.72	4.56	8.91E-02	3.38E-01
11.42	11.42	11.43	8.92E-02	3.38E-01
9.06	9.05	9.18	8.92E-02	3.38E-01

8.02	8.16	8.12	8.94E-02	3.38E-01
3.32	3.40	3.53	8.94E-02	3.38E-01
11.71	11.62	11.70	8.94E-02	3.38E-01
9.28	9.24	9.32	8.94E-02	3.38E-01
6.32	6.03	6.40	8.95E-02	3.38E-01
5.79	5.81	5.91	8.95E-02	3.38E-01
6.70	6.59	6.65	8.95E-02	3.38E-01
11.58	11.23	11.55	8.95E-02	3.38E-01
5.47	5.25	5.24	8.95E-02	3.38E-01
9.03	9.23	9.03	8.96E-02	3.38E-01
11.68	11.68	11.70	8.97E-02	3.39E-01
4.95	4.79	4.68	8.97E-02	3.39E-01
8.02	8.02	8.06	8.97E-02	3.39E-01
6.44	6.46	6.48	8.98E-02	3.39E-01
10.32	10.20	10.37	8.99E-02	3.39E-01
8.22	8.02	8.08	8.99E-02	3.39E-01
5.12	5.27	5.29	8.99E-02	3.39E-01
9.29	9.40	9.43	9.00E-02	3.39E-01
8.96	8.75	8.97	9.01E-02	3.40E-01
9.40	9.27	9.44	9.02E-02	3.40E-01
7.37	7.48	7.44	9.02E-02	3.40E-01
7.41	7.59	7.38	9.02E-02	3.40E-01
7.05	7.14	7.12	9.02E-02	3.40E-01
3.27	3.22	3.18	9.03E-02	3.40E-01
8.13	8.06	8.21	9.03E-02	3.40E-01
5.77	5.70	5.90	9.04E-02	3.40E-01
6.86	6.85	7.02	9.04E-02	3.40E-01
8.82	8.82	8.98	9.04E-02	3.40E-01
10.89	10.59	10.65	9.05E-02	3.40E-01
12.08	12.17	12.10	9.05E-02	3.40E-01
9.18	9.19	9.16	9.05E-02	3.40E-01
4.01	3.88	3.89	9.06E-02	3.40E-01
5.41	5.31	5.12	9.07E-02	3.41E-01
6.36	6.26	6.07	9.07E-02	3.41E-01
7.70	7.62	7.64	9.08E-02	3.41E-01
7.60	7.53	7.59	9.09E-02	3.41E-01
7.70	7.76	7.66	9.09E-02	3.41E-01
10.47	10.32	10.39	9.09E-02	3.41E-01
4.64	4.48	4.63	9.09E-02	3.41E-01
5.34	5.40	5.50	9.10E-02	3.42E-01
5.47	5.34	5.17	9.11E-02	3.42E-01
3.59	3.73	3.51	9.11E-02	3.42E-01
9.87	10.20	9.85	9.12E-02	3.42E-01
7.04	7.13	7.16	9.13E-02	3.42E-01
9.52	9.37	9.51	9.13E-02	3.42E-01
7.82	7.78	7.92	9.14E-02	3.42E-01
12.74	12.77	12.78	9.14E-02	3.42E-01

3.36	3.44	3.30	9.14E-02	3.42E-01
6.18	6.24	6.22	9.15E-02	3.42E-01
3.03	3.05	3.05	9.15E-02	3.42E-01
9.68	9.88	9.71	9.15E-02	3.42E-01
5.18	5.18	5.03	9.15E-02	3.42E-01
9.33	9.56	9.51	9.16E-02	3.42E-01
7.29	7.45	7.29	9.16E-02	3.42E-01
5.94	6.03	5.91	9.16E-02	3.42E-01
8.50	8.30	8.25	9.17E-02	3.42E-01
6.08	6.06	6.11	9.17E-02	3.42E-01
6.63	7.05	7.12	9.17E-02	3.42E-01
6.80	6.66	6.85	9.17E-02	3.42E-01
4.18	4.24	4.11	9.17E-02	3.42E-01
8.56	8.52	8.64	9.17E-02	3.43E-01
6.30	6.36	6.29	9.19E-02	3.43E-01
11.92	11.66	11.93	9.20E-02	3.43E-01
8.71	8.91	8.81	9.20E-02	3.43E-01
4.71	4.55	4.59	9.20E-02	3.43E-01
6.12	5.99	6.32	9.20E-02	3.43E-01
10.31	10.27	10.16	9.21E-02	3.43E-01
8.03	8.13	8.08	9.21E-02	3.43E-01
5.84	5.92	6.04	9.21E-02	3.43E-01
3.54	3.51	3.51	9.22E-02	3.43E-01
3.69	3.59	3.80	9.22E-02	3.43E-01
5.63	5.63	5.81	9.22E-02	3.43E-01
7.39	7.56	7.45	9.22E-02	3.43E-01
5.31	5.65	5.44	9.23E-02	3.43E-01
5.17	5.29	5.16	9.23E-02	3.43E-01
8.45	8.65	8.64	9.23E-02	3.43E-01
7.45	7.31	7.38	9.23E-02	3.43E-01
10.03	10.19	10.17	9.23E-02	3.43E-01
9.07	9.03	9.08	9.23E-02	3.43E-01
14.66	14.72	14.71	9.23E-02	3.43E-01
6.37	6.40	6.62	9.24E-02	3.43E-01
7.48	7.24	7.24	9.24E-02	3.43E-01
7.06	7.01	7.08	9.24E-02	3.43E-01
7.90	7.92	7.98	9.25E-02	3.43E-01
9.33	9.18	9.08	9.25E-02	3.43E-01
4.30	4.38	4.26	9.25E-02	3.43E-01
3.90	4.00	3.96	9.26E-02	3.44E-01
8.98	8.75	8.98	9.26E-02	3.44E-01
11.84	11.93	11.88	9.27E-02	3.44E-01
13.47	13.48	13.55	9.27E-02	3.44E-01
8.80	8.91	8.87	9.27E-02	3.44E-01
11.31	11.32	11.31	9.27E-02	3.44E-01
7.71	7.68	7.80	9.28E-02	3.44E-01
4.30	4.35	4.27	9.28E-02	3.44E-01

8.80	8.80	8.83	9.29E-02	3.44E-01
6.16	6.25	6.14	9.29E-02	3.44E-01
9.68	9.80	9.74	9.29E-02	3.44E-01
7.11	7.18	7.22	9.29E-02	3.44E-01
13.81	13.84	13.82	9.29E-02	3.44E-01
7.60	7.74	7.70	9.30E-02	3.44E-01
4.37	4.40	4.40	9.30E-02	3.44E-01
5.33	5.35	5.23	9.30E-02	3.44E-01
9.38	9.41	9.45	9.31E-02	3.44E-01
8.56	8.38	8.48	9.31E-02	3.44E-01
4.61	4.62	4.59	9.31E-02	3.44E-01
3.83	3.89	3.85	9.31E-02	3.44E-01
7.12	6.96	7.03	9.33E-02	3.45E-01
10.24	10.17	10.23	9.33E-02	3.45E-01
10.57	10.50	10.49	9.34E-02	3.45E-01
8.16	8.32	8.18	9.34E-02	3.45E-01
7.94	7.98	8.09	9.34E-02	3.45E-01
12.94	12.92	12.94	9.34E-02	3.45E-01
4.53	4.34	4.52	9.35E-02	3.45E-01
8.62	8.76	8.64	9.36E-02	3.45E-01
3.76	3.95	4.00	9.36E-02	3.45E-01
8.36	8.44	8.40	9.36E-02	3.45E-01
8.18	7.97	8.02	9.36E-02	3.45E-01
8.08	8.46	8.20	9.36E-02	3.45E-01
9.67	9.50	9.87	9.37E-02	3.45E-01
5.21	5.06	5.31	9.37E-02	3.45E-01
5.66	5.72	5.53	9.37E-02	3.45E-01
7.79	7.70	7.70	9.37E-02	3.45E-01
9.09	8.97	9.03	9.37E-02	3.45E-01
7.70	7.75	7.61	9.38E-02	3.45E-01
7.19	7.33	7.14	9.38E-02	3.45E-01
9.65	9.68	9.82	9.38E-02	3.45E-01
6.75	7.91	6.76	9.38E-02	3.45E-01
8.46	8.53	8.62	9.39E-02	3.45E-01
3.27	3.24	3.19	9.39E-02	3.45E-01
9.37	9.59	9.60	9.41E-02	3.46E-01
6.62	6.78	6.69	9.41E-02	3.46E-01
9.44	9.21	9.54	9.41E-02	3.46E-01
4.30	4.31	4.55	9.41E-02	3.46E-01
5.46	5.38	5.50	9.41E-02	3.46E-01
4.19	4.20	4.13	9.42E-02	3.46E-01
3.79	3.69	3.60	9.42E-02	3.46E-01
5.78	5.88	5.76	9.42E-02	3.46E-01
11.45	11.48	11.50	9.43E-02	3.46E-01
9.38	9.02	9.20	9.43E-02	3.46E-01
10.45	10.19	10.29	9.43E-02	3.46E-01
9.81	9.38	9.80	9.43E-02	3.46E-01

7.90	7.76	7.89	9.44E-02	3.46E-01
12.88	13.01	12.87	9.44E-02	3.46E-01
7.23	7.32	7.25	9.44E-02	3.46E-01
4.31	4.22	4.39	9.44E-02	3.46E-01
11.69	11.90	11.83	9.44E-02	3.46E-01
10.59	10.65	10.71	9.45E-02	3.46E-01
6.60	6.61	6.40	9.45E-02	3.46E-01
9.20	9.41	9.39	9.45E-02	3.46E-01
9.13	9.04	9.11	9.46E-02	3.46E-01
8.06	7.89	8.24	9.46E-02	3.46E-01
8.91	9.14	9.06	9.47E-02	3.46E-01
8.39	8.41	8.39	9.47E-02	3.46E-01
8.09	7.73	7.76	9.47E-02	3.46E-01
11.06	10.93	11.14	9.48E-02	3.46E-01
4.88	5.01	4.92	9.48E-02	3.46E-01
8.51	8.58	8.64	9.49E-02	3.46E-01
11.92	12.07	12.05	9.49E-02	3.46E-01
7.55	7.41	7.51	9.49E-02	3.46E-01
7.93	8.00	8.10	9.49E-02	3.46E-01
6.30	6.34	6.38	9.49E-02	3.46E-01
9.37	9.20	9.46	9.50E-02	3.46E-01
4.65	4.37	4.78	9.50E-02	3.46E-01
7.39	7.48	7.57	9.50E-02	3.47E-01
11.40	11.42	11.36	9.51E-02	3.47E-01
7.89	8.11	7.90	9.51E-02	3.47E-01
6.26	6.30	6.27	9.51E-02	3.47E-01
4.78	4.84	4.98	9.51E-02	3.47E-01
10.79	10.80	10.88	9.52E-02	3.47E-01
10.62	10.76	10.69	9.52E-02	3.47E-01
5.53	5.45	5.32	9.53E-02	3.47E-01
7.53	7.04	7.39	9.53E-02	3.47E-01
8.34	8.69	8.55	9.53E-02	3.47E-01
10.08	10.32	10.11	9.53E-02	3.47E-01
9.94	10.03	10.14	9.53E-02	3.47E-01
10.56	10.56	10.55	9.53E-02	3.47E-01
8.97	8.82	9.08	9.54E-02	3.47E-01
9.47	9.42	9.46	9.54E-02	3.47E-01
9.91	9.78	9.80	9.54E-02	3.47E-01
4.91	4.88	4.86	9.54E-02	3.47E-01
7.81	7.89	7.99	9.55E-02	3.47E-01
7.39	7.30	7.31	9.55E-02	3.47E-01
6.59	6.45	6.44	9.55E-02	3.47E-01
6.65	6.73	6.66	9.55E-02	3.47E-01
11.03	11.03	11.11	9.56E-02	3.47E-01
5.76	6.06	5.49	9.56E-02	3.47E-01
12.76	12.63	12.74	9.56E-02	3.47E-01
14.95	14.98	14.96	9.56E-02	3.47E-01

3.07	3.08	3.03	9.56E-02	3.47E-01
3.79	3.77	3.79	9.56E-02	3.47E-01
7.92	7.97	7.94	9.57E-02	3.47E-01
5.74	5.44	5.51	9.57E-02	3.47E-01
11.02	11.08	11.07	9.57E-02	3.47E-01
3.72	3.64	3.68	9.57E-02	3.47E-01
4.28	4.00	4.21	9.58E-02	3.47E-01
7.22	7.41	7.21	9.58E-02	3.47E-01
7.64	7.22	7.29	9.59E-02	3.47E-01
5.39	5.26	5.10	9.59E-02	3.47E-01
6.74	6.92	6.77	9.59E-02	3.47E-01
6.98	7.09	7.28	9.59E-02	3.47E-01
8.01	7.94	8.12	9.60E-02	3.47E-01
3.66	3.50	3.80	9.60E-02	3.47E-01
7.73	7.84	7.81	9.60E-02	3.47E-01
4.69	4.57	4.84	9.60E-02	3.47E-01
7.57	7.68	7.68	9.60E-02	3.47E-01
4.83	5.03	5.00	9.60E-02	3.47E-01
10.00	9.95	10.04	9.61E-02	3.47E-01
6.44	6.48	6.41	9.61E-02	3.47E-01
5.82	5.95	5.96	9.61E-02	3.47E-01
8.62	8.61	8.65	9.61E-02	3.47E-01
3.47	3.37	3.58	9.62E-02	3.47E-01
10.28	10.25	10.29	9.62E-02	3.47E-01
3.46	3.37	3.55	9.62E-02	3.47E-01
10.18	10.31	10.29	9.63E-02	3.47E-01
6.51	6.20	6.58	9.63E-02	3.47E-01
8.01	8.26	7.95	9.63E-02	3.47E-01
6.10	6.01	6.20	9.64E-02	3.47E-01
5.68	5.55	5.87	9.64E-02	3.47E-01
7.11	7.06	7.06	9.64E-02	3.47E-01
5.25	5.12	5.13	9.65E-02	3.48E-01
12.64	12.38	12.61	9.66E-02	3.48E-01
8.58	8.53	8.66	9.66E-02	3.48E-01
8.30	8.49	8.50	9.66E-02	3.48E-01
6.22	6.34	6.27	9.67E-02	3.48E-01
5.88	5.98	6.27	9.67E-02	3.48E-01
8.23	8.33	8.46	9.67E-02	3.48E-01
3.43	3.42	3.44	9.67E-02	3.48E-01
9.57	9.55	9.68	9.67E-02	3.48E-01
14.42	14.50	14.41	9.68E-02	3.48E-01
8.35	8.64	8.40	9.68E-02	3.48E-01
8.63	8.78	8.70	9.68E-02	3.48E-01
5.16	5.05	5.09	9.69E-02	3.48E-01
6.57	6.67	6.75	9.69E-02	3.48E-01
6.25	6.32	6.20	9.70E-02	3.48E-01
7.60	7.63	7.73	9.70E-02	3.49E-01

7.63	7.87	7.58	9.71E-02	3.49E-01
7.82	7.85	7.94	9.71E-02	3.49E-01
6.76	6.53	6.71	9.72E-02	3.49E-01
6.25	6.24	6.30	9.73E-02	3.49E-01
8.10	8.34	8.25	9.73E-02	3.49E-01
8.52	8.66	8.68	9.73E-02	3.49E-01
6.45	6.62	6.47	9.73E-02	3.49E-01
7.16	7.20	7.07	9.73E-02	3.49E-01
9.98	9.99	10.03	9.74E-02	3.49E-01
8.72	8.76	8.76	9.74E-02	3.49E-01
11.70	11.78	11.67	9.74E-02	3.49E-01
3.97	3.90	4.02	9.75E-02	3.49E-01
7.41	7.59	7.35	9.75E-02	3.49E-01
6.32	6.32	6.42	9.75E-02	3.49E-01
5.14	5.41	5.57	9.76E-02	3.49E-01
8.71	8.57	8.79	9.76E-02	3.49E-01
9.16	8.99	9.09	9.76E-02	3.49E-01
6.09	5.95	6.22	9.76E-02	3.49E-01
3.62	3.80	3.77	9.76E-02	3.49E-01
6.84	6.53	6.42	9.76E-02	3.49E-01
5.80	5.72	5.84	9.76E-02	3.49E-01
7.69	7.73	7.75	9.76E-02	3.49E-01
5.07	5.32	5.37	9.77E-02	3.49E-01
6.02	5.95	6.00	9.77E-02	3.49E-01
9.55	9.54	9.56	9.77E-02	3.49E-01
10.97	11.04	10.99	9.77E-02	3.49E-01
11.79	12.15	11.99	9.78E-02	3.49E-01
6.53	6.67	6.59	9.78E-02	3.49E-01
7.58	7.31	7.61	9.78E-02	3.49E-01
11.25	11.17	11.27	9.79E-02	3.49E-01
10.12	10.03	10.18	9.79E-02	3.49E-01
14.13	14.14	14.22	9.79E-02	3.49E-01
8.07	8.32	8.19	9.79E-02	3.49E-01
7.65	7.54	7.55	9.79E-02	3.49E-01
10.09	10.19	10.18	9.80E-02	3.49E-01
8.80	8.70	8.80	9.81E-02	3.49E-01
6.15	5.95	5.79	9.81E-02	3.49E-01
7.84	7.97	7.81	9.81E-02	3.49E-01
9.28	9.33	9.26	9.81E-02	3.49E-01
9.23	9.44	9.37	9.82E-02	3.49E-01
4.45	4.56	4.53	9.82E-02	3.50E-01
6.60	6.73	6.70	9.82E-02	3.50E-01
8.45	8.59	8.57	9.83E-02	3.50E-01
4.39	4.08	4.16	9.84E-02	3.50E-01
6.29	6.23	6.32	9.84E-02	3.50E-01
6.68	6.56	6.66	9.85E-02	3.50E-01
6.46	6.53	6.52	9.86E-02	3.50E-01

12.99	12.94	13.03	9.87E-02	3.51E-01
6.44	6.35	6.50	9.87E-02	3.51E-01
5.71	5.65	5.77	9.88E-02	3.51E-01
7.09	7.19	7.21	9.88E-02	3.51E-01
7.15	7.11	7.28	9.88E-02	3.51E-01
9.11	9.12	9.22	9.88E-02	3.51E-01
6.94	6.98	6.99	9.89E-02	3.51E-01
8.03	8.00	8.22	9.89E-02	3.51E-01
7.34	7.45	7.28	9.89E-02	3.51E-01
7.95	8.01	7.96	9.89E-02	3.51E-01
4.74	5.06	4.58	9.90E-02	3.51E-01
8.61	8.63	8.67	9.90E-02	3.51E-01
3.61	3.66	3.76	9.90E-02	3.51E-01
9.04	9.29	9.25	9.90E-02	3.51E-01
3.32	3.35	3.57	9.90E-02	3.51E-01
9.30	9.22	9.31	9.91E-02	3.51E-01
10.94	11.04	11.05	9.91E-02	3.51E-01
3.51	3.52	3.64	9.91E-02	3.51E-01
8.72	8.83	8.84	9.92E-02	3.51E-01
8.99	8.53	9.03	9.92E-02	3.51E-01
4.37	4.21	4.14	9.92E-02	3.51E-01
8.98	9.42	9.18	9.94E-02	3.52E-01
3.62	3.72	3.63	9.94E-02	3.52E-01
9.02	8.97	9.16	9.94E-02	3.52E-01
6.70	6.65	6.84	9.95E-02	3.52E-01
3.87	3.95	3.93	9.95E-02	3.52E-01
4.84	4.73	4.74	9.95E-02	3.52E-01
7.34	7.52	7.60	9.95E-02	3.52E-01
6.33	6.39	6.47	9.96E-02	3.52E-01
7.74	8.23	7.84	9.98E-02	3.52E-01
6.08	6.14	6.13	9.98E-02	3.52E-01
8.12	8.18	8.18	9.99E-02	3.53E-01
9.63	9.68	9.72	1.00E-01	3.53E-01
7.95	8.15	8.17	1.00E-01	3.53E-01
10.00	10.10	10.08	1.00E-01	3.53E-01
12.84	12.88	12.92	1.00E-01	3.53E-01
7.99	8.25	8.14	1.00E-01	3.53E-01
5.22	5.51	5.48	1.00E-01	3.53E-01
10.23	10.53	10.35	1.00E-01	3.53E-01
7.13	7.04	7.14	1.00E-01	3.53E-01
7.73	7.82	7.75	1.00E-01	3.53E-01
12.33	12.57	12.44	1.00E-01	3.53E-01
3.76	3.68	3.63	1.00E-01	3.53E-01
9.41	9.20	9.42	1.00E-01	3.53E-01
8.81	8.73	8.85	1.00E-01	3.53E-01
7.86	7.71	7.78	1.00E-01	3.53E-01
3.68	3.71	3.76	1.00E-01	3.53E-01

5.04	5.07	5.01	1.00E-01	3.53E-01
9.65	9.65	9.71	1.00E-01	3.53E-01
11.06	10.85	11.08	1.00E-01	3.53E-01
5.74	5.60	5.58	1.00E-01	3.53E-01
3.98	4.01	3.85	1.00E-01	3.53E-01
6.83	6.82	6.76	1.00E-01	3.53E-01
10.88	11.09	11.01	1.00E-01	3.53E-01
8.23	8.37	8.19	1.00E-01	3.53E-01
5.67	5.61	5.81	1.00E-01	3.53E-01
14.22	14.24	14.30	1.01E-01	3.53E-01
7.21	7.12	7.04	1.01E-01	3.53E-01
6.27	6.35	6.51	1.01E-01	3.53E-01
7.99	7.61	7.88	1.01E-01	3.53E-01
10.09	10.40	10.19	1.01E-01	3.53E-01
7.53	7.57	7.58	1.01E-01	3.53E-01
8.25	8.39	8.23	1.01E-01	3.53E-01
3.21	3.32	3.24	1.01E-01	3.53E-01
8.09	8.24	8.12	1.01E-01	3.53E-01
9.58	9.57	9.58	1.01E-01	3.53E-01
7.23	7.24	7.26	1.01E-01	3.53E-01
8.85	8.99	8.91	1.01E-01	3.53E-01
4.91	4.50	4.72	1.01E-01	3.53E-01
8.68	8.62	8.61	1.01E-01	3.53E-01
5.86	5.88	5.84	1.01E-01	3.53E-01
3.97	4.02	3.90	1.01E-01	3.54E-01
4.44	4.61	4.58	1.01E-01	3.54E-01
6.12	6.10	6.03	1.01E-01	3.55E-01
7.89	8.12	8.03	1.01E-01	3.55E-01
4.88	4.54	4.49	1.01E-01	3.55E-01
7.94	7.95	8.00	1.01E-01	3.55E-01
5.51	6.24	5.98	1.02E-01	3.55E-01
9.63	9.60	9.64	1.02E-01	3.55E-01
9.51	9.18	9.41	1.02E-01	3.55E-01
10.28	10.40	10.41	1.02E-01	3.55E-01
3.25	3.46	3.20	1.02E-01	3.55E-01
6.52	6.41	6.54	1.02E-01	3.55E-01
9.30	9.31	9.30	1.02E-01	3.56E-01
5.25	5.26	5.29	1.02E-01	3.56E-01
6.71	6.79	6.97	1.02E-01	3.56E-01
9.16	9.16	9.29	1.02E-01	3.56E-01
9.40	9.52	9.64	1.02E-01	3.56E-01
3.46	3.40	3.63	1.02E-01	3.56E-01
7.26	7.17	7.19	1.02E-01	3.56E-01
6.35	6.48	6.66	1.02E-01	3.56E-01
5.86	5.64	5.64	1.02E-01	3.56E-01
7.40	7.40	7.56	1.02E-01	3.56E-01
8.87	8.90	9.04	1.02E-01	3.56E-01

9.47	9.34	9.39	1.02E-01	3.56E-01
12.50	12.46	12.59	1.02E-01	3.56E-01
7.91	8.03	8.02	1.02E-01	3.56E-01
12.09	12.14	12.12	1.02E-01	3.56E-01
6.99	7.23	6.96	1.02E-01	3.56E-01
9.42	9.19	9.30	1.02E-01	3.56E-01
8.50	8.49	8.46	1.02E-01	3.56E-01
7.62	7.45	7.65	1.02E-01	3.56E-01
3.24	3.12	3.19	1.03E-01	3.57E-01
9.68	9.41	9.51	1.03E-01	3.57E-01
7.18	7.38	7.36	1.03E-01	3.57E-01
4.34	4.20	4.16	1.03E-01	3.57E-01
4.72	4.48	4.55	1.03E-01	3.57E-01
9.66	9.46	9.68	1.03E-01	3.57E-01
10.57	10.55	10.52	1.03E-01	3.57E-01
8.03	7.97	8.00	1.03E-01	3.57E-01
9.39	9.40	9.42	1.03E-01	3.57E-01
5.35	5.35	5.24	1.03E-01	3.58E-01
3.34	3.33	3.37	1.03E-01	3.58E-01
7.37	7.56	7.54	1.03E-01	3.58E-01
9.61	9.50	9.60	1.03E-01	3.58E-01
8.18	8.44	8.37	1.03E-01	3.58E-01
9.73	9.86	9.81	1.03E-01	3.58E-01
9.94	9.90	9.87	1.03E-01	3.58E-01
7.59	7.50	7.47	1.03E-01	3.58E-01
8.93	9.02	8.88	1.03E-01	3.58E-01
8.77	8.81	8.73	1.03E-01	3.58E-01
8.05	7.97	8.02	1.03E-01	3.58E-01
4.29	4.36	3.91	1.03E-01	3.58E-01
8.81	8.77	8.97	1.03E-01	3.58E-01
6.50	6.39	6.37	1.03E-01	3.58E-01
6.63	6.76	6.70	1.03E-01	3.58E-01
8.79	8.45	8.81	1.04E-01	3.58E-01
4.26	4.42	4.38	1.04E-01	3.58E-01
9.25	9.29	9.29	1.04E-01	3.58E-01
8.51	8.71	8.57	1.04E-01	3.58E-01
7.75	7.93	7.84	1.04E-01	3.58E-01
7.71	7.63	7.87	1.04E-01	3.58E-01
6.99	7.42	7.21	1.04E-01	3.59E-01
7.50	7.69	7.60	1.04E-01	3.59E-01
9.73	9.97	9.88	1.04E-01	3.59E-01
6.30	6.12	6.38	1.04E-01	3.59E-01
9.68	9.55	9.60	1.04E-01	3.59E-01
3.39	3.16	3.07	1.04E-01	3.59E-01
3.38	3.52	3.44	1.04E-01	3.59E-01
8.65	9.04	8.85	1.04E-01	3.59E-01
6.53	6.51	6.45	1.04E-01	3.59E-01

3.71	3.81	4.01	1.04E-01	3.59E-01
9.29	9.57	9.23	1.04E-01	3.59E-01
9.35	9.36	9.41	1.04E-01	3.59E-01
6.70	6.67	6.64	1.04E-01	3.59E-01
7.67	7.77	7.78	1.04E-01	3.59E-01
5.38	5.29	5.39	1.04E-01	3.59E-01
3.61	3.76	3.75	1.04E-01	3.59E-01
11.33	11.05	11.25	1.04E-01	3.59E-01
6.29	6.02	6.03	1.04E-01	3.59E-01
8.59	8.91	8.70	1.04E-01	3.59E-01
5.76	5.56	5.73	1.05E-01	3.60E-01
9.83	9.93	9.63	1.05E-01	3.60E-01
8.83	8.99	8.96	1.05E-01	3.60E-01
12.28	12.35	12.31	1.05E-01	3.60E-01
4.63	4.70	4.57	1.05E-01	3.60E-01
6.51	6.59	6.61	1.05E-01	3.60E-01
7.93	7.95	8.08	1.05E-01	3.60E-01
4.90	5.04	4.89	1.05E-01	3.60E-01
8.82	8.91	8.94	1.05E-01	3.60E-01
5.56	5.83	5.72	1.05E-01	3.60E-01
6.22	6.08	6.19	1.05E-01	3.60E-01
5.02	5.08	4.94	1.05E-01	3.60E-01
8.30	8.19	8.31	1.05E-01	3.60E-01
5.83	5.95	5.95	1.05E-01	3.60E-01
12.68	12.85	12.80	1.05E-01	3.61E-01
6.48	6.86	6.60	1.05E-01	3.61E-01
6.97	6.76	6.77	1.05E-01	3.61E-01
5.09	5.17	5.29	1.05E-01	3.61E-01
4.96	4.96	5.14	1.05E-01	3.61E-01
7.94	7.67	8.08	1.05E-01	3.61E-01
8.61	8.74	8.61	1.05E-01	3.61E-01
10.37	10.25	10.40	1.05E-01	3.61E-01
12.51	12.45	12.42	1.05E-01	3.61E-01
3.99	3.79	3.69	1.05E-01	3.61E-01
8.06	7.89	7.96	1.05E-01	3.61E-01
9.57	9.39	9.53	1.06E-01	3.61E-01
5.98	6.14	6.01	1.06E-01	3.62E-01
6.54	6.49	6.59	1.06E-01	3.62E-01
9.08	8.67	8.88	1.06E-01	3.62E-01
6.66	6.66	6.43	1.06E-01	3.62E-01
5.80	5.53	5.81	1.06E-01	3.62E-01
6.32	6.60	6.47	1.06E-01	3.62E-01
9.57	9.63	9.71	1.06E-01	3.62E-01
6.22	5.97	6.14	1.06E-01	3.62E-01
8.53	8.80	8.53	1.06E-01	3.62E-01
10.14	10.17	10.15	1.06E-01	3.62E-01
9.70	9.81	9.82	1.06E-01	3.62E-01

15.21	15.17	15.18	1.06E-01	3.62E-01
4.24	4.13	4.15	1.06E-01	3.62E-01
9.49	9.25	9.47	1.06E-01	3.62E-01
4.25	4.46	4.35	1.06E-01	3.62E-01
3.26	3.20	3.35	1.06E-01	3.62E-01
7.65	7.44	7.55	1.06E-01	3.62E-01
6.26	6.36	6.29	1.06E-01	3.62E-01
7.13	7.15	7.15	1.06E-01	3.62E-01
6.62	6.67	6.73	1.06E-01	3.62E-01
5.99	5.98	5.90	1.06E-01	3.62E-01
6.44	6.64	6.81	1.06E-01	3.62E-01
9.77	9.94	9.80	1.06E-01	3.62E-01
7.97	7.97	7.86	1.06E-01	3.62E-01
8.49	8.51	8.54	1.06E-01	3.62E-01
7.52	7.74	7.67	1.06E-01	3.62E-01
4.92	4.98	4.89	1.06E-01	3.62E-01
4.53	4.41	4.44	1.06E-01	3.62E-01
8.47	8.35	8.33	1.06E-01	3.62E-01
8.45	8.73	8.47	1.06E-01	3.62E-01
9.92	9.78	9.92	1.06E-01	3.62E-01
4.98	4.94	4.68	1.06E-01	3.62E-01
5.92	5.89	5.81	1.06E-01	3.62E-01
3.75	3.69	3.73	1.06E-01	3.62E-01
7.27	7.29	7.15	1.06E-01	3.62E-01
14.36	14.36	14.40	1.07E-01	3.62E-01
5.30	5.51	5.39	1.07E-01	3.62E-01
11.46	11.29	11.51	1.07E-01	3.62E-01
6.31	6.55	6.49	1.07E-01	3.62E-01
10.04	10.13	10.08	1.07E-01	3.62E-01
8.51	8.59	8.48	1.07E-01	3.63E-01
9.24	9.22	8.96	1.07E-01	3.63E-01
11.05	11.31	11.14	1.07E-01	3.63E-01
9.62	9.45	9.27	1.07E-01	3.63E-01
10.06	10.08	10.13	1.07E-01	3.63E-01
8.00	7.89	8.01	1.07E-01	3.63E-01
9.15	9.27	9.30	1.07E-01	3.63E-01
6.92	7.13	6.91	1.07E-01	3.63E-01
10.68	10.59	10.68	1.07E-01	3.63E-01
10.08	10.33	10.28	1.07E-01	3.63E-01
7.68	7.75	7.51	1.07E-01	3.63E-01
10.22	10.16	10.13	1.07E-01	3.63E-01
9.21	9.23	9.46	1.07E-01	3.63E-01
10.67	10.56	10.67	1.07E-01	3.63E-01
7.02	6.81	6.74	1.07E-01	3.63E-01
8.69	8.42	8.64	1.07E-01	3.63E-01
7.06	7.01	7.10	1.07E-01	3.63E-01
7.23	7.40	7.22	1.07E-01	3.63E-01

5.07	5.25	5.13	1.07E-01	3.63E-01
10.14	10.40	10.28	1.07E-01	3.63E-01
5.32	5.80	5.42	1.07E-01	3.63E-01
3.30	3.35	3.34	1.07E-01	3.63E-01
7.61	7.74	7.72	1.07E-01	3.63E-01
9.07	9.23	9.22	1.08E-01	3.63E-01
3.91	3.89	3.85	1.08E-01	3.63E-01
8.80	8.87	8.86	1.08E-01	3.63E-01
9.37	9.05	9.16	1.08E-01	3.63E-01
7.61	7.40	7.63	1.08E-01	3.63E-01
10.17	10.20	10.33	1.08E-01	3.63E-01
5.24	4.89	4.99	1.08E-01	3.63E-01
4.53	4.55	4.43	1.08E-01	3.63E-01
9.29	9.60	9.37	1.08E-01	3.63E-01
12.00	12.10	12.11	1.08E-01	3.63E-01
6.92	6.97	6.92	1.08E-01	3.63E-01
8.68	8.70	8.56	1.08E-01	3.63E-01
8.48	8.77	8.56	1.08E-01	3.63E-01
6.94	6.91	7.03	1.08E-01	3.63E-01
4.12	4.21	4.30	1.08E-01	3.63E-01
3.95	3.93	3.85	1.08E-01	3.63E-01
3.56	3.46	3.48	1.08E-01	3.63E-01
8.33	8.37	8.49	1.08E-01	3.63E-01
8.56	8.48	8.70	1.08E-01	3.63E-01
5.45	5.45	5.59	1.08E-01	3.63E-01
6.39	6.32	6.18	1.08E-01	3.63E-01
4.24	4.07	4.11	1.08E-01	3.63E-01
11.63	11.47	11.67	1.08E-01	3.63E-01
3.60	3.50	3.55	1.08E-01	3.63E-01
6.17	6.07	5.93	1.08E-01	3.63E-01
5.65	5.98	5.80	1.08E-01	3.63E-01
7.42	7.27	7.30	1.08E-01	3.63E-01
7.33	7.40	7.39	1.08E-01	3.63E-01
5.87	5.77	5.78	1.08E-01	3.63E-01
7.48	7.37	7.47	1.08E-01	3.63E-01
11.35	11.56	11.36	1.08E-01	3.63E-01
9.79	10.01	9.97	1.08E-01	3.63E-01
9.45	9.53	9.51	1.08E-01	3.63E-01
10.10	9.78	9.84	1.08E-01	3.63E-01
8.88	8.76	8.91	1.08E-01	3.63E-01
9.66	9.91	9.83	1.08E-01	3.63E-01
9.21	9.15	9.36	1.08E-01	3.63E-01
7.11	7.37	7.16	1.08E-01	3.63E-01
8.85	8.98	8.98	1.08E-01	3.63E-01
11.41	11.30	11.40	1.08E-01	3.63E-01
12.33	12.47	12.34	1.08E-01	3.63E-01
8.97	8.94	9.00	1.08E-01	3.63E-01

3.73	3.51	3.75	1.08E-01	3.63E-01
6.62	6.66	6.93	1.08E-01	3.63E-01
6.61	6.57	6.78	1.09E-01	3.63E-01
8.67	8.79	8.80	1.09E-01	3.63E-01
5.37	5.49	5.12	1.09E-01	3.63E-01
4.94	4.92	4.89	1.09E-01	3.63E-01
10.87	10.70	10.91	1.09E-01	3.63E-01
3.77	3.81	3.88	1.09E-01	3.63E-01
11.13	11.37	11.33	1.09E-01	3.63E-01
12.50	12.63	12.55	1.09E-01	3.63E-01
5.44	5.72	5.41	1.09E-01	3.63E-01
4.86	4.79	4.69	1.09E-01	3.63E-01
7.78	7.86	7.73	1.09E-01	3.63E-01
5.74	5.87	5.73	1.09E-01	3.63E-01
8.22	8.42	8.38	1.09E-01	3.63E-01
7.38	7.38	7.42	1.09E-01	3.63E-01
10.71	10.68	10.78	1.09E-01	3.63E-01
8.30	8.20	8.40	1.09E-01	3.63E-01
5.57	5.45	5.67	1.09E-01	3.63E-01
11.23	11.12	11.32	1.09E-01	3.64E-01
4.35	4.37	4.39	1.09E-01	3.64E-01
8.61	8.77	8.66	1.09E-01	3.64E-01
8.39	8.33	8.11	1.09E-01	3.64E-01
6.38	6.38	6.42	1.09E-01	3.64E-01
5.85	5.76	5.88	1.09E-01	3.64E-01
4.07	4.00	4.07	1.09E-01	3.64E-01
7.62	7.66	7.76	1.09E-01	3.64E-01
6.93	6.83	6.82	1.09E-01	3.64E-01
7.03	6.94	7.05	1.09E-01	3.64E-01
6.50	6.40	6.54	1.09E-01	3.64E-01
4.59	4.59	4.55	1.09E-01	3.64E-01
6.61	6.52	6.79	1.09E-01	3.64E-01
8.20	8.19	8.13	1.09E-01	3.64E-01
7.21	7.49	7.41	1.09E-01	3.64E-01
4.81	4.80	4.69	1.09E-01	3.64E-01
6.68	6.82	7.02	1.10E-01	3.64E-01
5.25	4.78	4.97	1.10E-01	3.64E-01
4.04	4.03	4.16	1.10E-01	3.64E-01
9.52	9.46	9.52	1.10E-01	3.64E-01
6.30	6.33	6.35	1.10E-01	3.64E-01
6.96	7.11	7.15	1.10E-01	3.64E-01
9.08	9.16	9.21	1.10E-01	3.64E-01
7.44	7.32	7.47	1.10E-01	3.64E-01
12.33	12.25	12.26	1.10E-01	3.64E-01
9.85	9.93	9.94	1.10E-01	3.64E-01
8.98	8.97	9.01	1.10E-01	3.64E-01
8.56	8.65	8.75	1.10E-01	3.64E-01

8.51	8.24	8.63	1.10E-01	3.64E-01
11.36	11.53	11.40	1.10E-01	3.64E-01
5.39	5.15	5.30	1.10E-01	3.64E-01
5.31	5.25	5.37	1.10E-01	3.64E-01
7.14	7.08	7.06	1.10E-01	3.64E-01
8.14	8.18	8.20	1.10E-01	3.64E-01
7.69	7.42	7.77	1.10E-01	3.64E-01
4.23	4.19	4.03	1.10E-01	3.64E-01
13.28	13.41	13.39	1.10E-01	3.64E-01
10.67	10.38	10.47	1.10E-01	3.64E-01
8.04	8.20	8.14	1.10E-01	3.64E-01
8.16	8.09	8.18	1.10E-01	3.64E-01
4.62	4.44	4.49	1.10E-01	3.64E-01
9.45	9.40	9.49	1.10E-01	3.64E-01
7.20	7.35	7.49	1.10E-01	3.64E-01
8.28	8.44	8.42	1.10E-01	3.64E-01
5.75	5.49	5.62	1.10E-01	3.64E-01
9.33	9.47	9.32	1.10E-01	3.64E-01
3.97	3.97	3.91	1.10E-01	3.64E-01
4.11	3.83	4.25	1.10E-01	3.65E-01
4.27	4.15	4.25	1.10E-01	3.65E-01
9.47	9.57	9.46	1.10E-01	3.65E-01
9.41	9.38	9.35	1.10E-01	3.65E-01
9.36	9.16	9.48	1.11E-01	3.65E-01
9.25	9.29	9.26	1.11E-01	3.65E-01
7.05	7.03	7.18	1.11E-01	3.65E-01
10.05	10.15	10.08	1.11E-01	3.65E-01
7.11	7.44	7.45	1.11E-01	3.65E-01
7.84	8.00	7.94	1.11E-01	3.65E-01
5.17	4.94	5.17	1.11E-01	3.65E-01
4.98	4.78	4.84	1.11E-01	3.65E-01
10.13	10.33	10.21	1.11E-01	3.65E-01
7.46	7.80	7.44	1.11E-01	3.65E-01
6.48	6.54	6.64	1.11E-01	3.65E-01
4.88	5.06	4.84	1.11E-01	3.65E-01
7.18	7.11	7.14	1.11E-01	3.65E-01
5.82	5.73	5.62	1.11E-01	3.65E-01
4.34	4.30	4.29	1.11E-01	3.65E-01
11.37	11.41	11.31	1.11E-01	3.65E-01
7.43	7.56	7.63	1.11E-01	3.66E-01
9.14	9.10	9.29	1.11E-01	3.66E-01
8.29	8.22	8.11	1.11E-01	3.66E-01
8.91	9.01	8.94	1.11E-01	3.66E-01
5.12	4.82	4.98	1.11E-01	3.66E-01
11.27	11.29	11.34	1.11E-01	3.66E-01
10.71	10.70	10.82	1.11E-01	3.66E-01
3.54	3.35	3.28	1.11E-01	3.66E-01

7.54	7.75	7.68	1.11E-01	3.66E-01
9.28	9.32	9.22	1.12E-01	3.66E-01
6.15	6.59	6.18	1.12E-01	3.67E-01
7.09	7.03	7.13	1.12E-01	3.67E-01
6.01	6.11	5.97	1.12E-01	3.67E-01
5.93	5.95	5.91	1.12E-01	3.67E-01
6.80	7.19	7.05	1.12E-01	3.67E-01
9.46	9.43	9.54	1.12E-01	3.67E-01
4.49	4.53	4.53	1.12E-01	3.67E-01
3.49	3.56	3.52	1.12E-01	3.67E-01
5.06	5.05	5.33	1.12E-01	3.67E-01
8.46	8.58	8.59	1.12E-01	3.67E-01
12.15	12.29	12.22	1.12E-01	3.67E-01
4.41	4.27	4.34	1.12E-01	3.67E-01
10.36	10.35	10.39	1.12E-01	3.67E-01
4.96	4.85	5.15	1.12E-01	3.67E-01
8.25	8.48	8.27	1.12E-01	3.67E-01
4.45	4.60	4.44	1.12E-01	3.67E-01
12.24	12.27	12.39	1.12E-01	3.67E-01
11.24	11.28	11.26	1.12E-01	3.67E-01
4.49	4.74	4.45	1.12E-01	3.67E-01
9.50	9.51	9.60	1.12E-01	3.67E-01
5.83	5.98	5.69	1.12E-01	3.67E-01
5.29	5.46	5.50	1.12E-01	3.67E-01
8.32	8.18	8.11	1.12E-01	3.67E-01
7.95	8.04	7.80	1.12E-01	3.67E-01
7.31	7.05	7.47	1.12E-01	3.67E-01
6.27	6.28	6.43	1.12E-01	3.67E-01
13.13	13.14	13.17	1.12E-01	3.67E-01
8.99	8.91	9.07	1.12E-01	3.67E-01
7.78	7.80	7.77	1.13E-01	3.68E-01
6.77	6.73	7.04	1.13E-01	3.68E-01
12.66	12.66	12.60	1.13E-01	3.68E-01
4.46	4.59	4.66	1.13E-01	3.68E-01
9.69	9.99	9.79	1.13E-01	3.68E-01
11.09	11.21	11.03	1.13E-01	3.68E-01
7.87	7.85	8.00	1.13E-01	3.68E-01
9.55	9.71	9.65	1.13E-01	3.68E-01
7.53	7.67	7.61	1.13E-01	3.68E-01
6.97	6.91	6.93	1.13E-01	3.68E-01
8.86	9.30	9.00	1.13E-01	3.68E-01
6.26	6.31	6.50	1.13E-01	3.68E-01
11.53	11.58	11.57	1.13E-01	3.68E-01
4.80	4.63	4.63	1.13E-01	3.68E-01
3.49	3.46	3.35	1.13E-01	3.68E-01
7.32	7.53	7.41	1.13E-01	3.68E-01
6.66	6.23	6.38	1.13E-01	3.68E-01

8.74	8.87	8.80	1.13E-01	3.68E-01
10.66	10.81	10.74	1.13E-01	3.68E-01
7.68	7.66	7.82	1.13E-01	3.68E-01
7.40	6.96	7.43	1.13E-01	3.69E-01
8.26	8.25	8.31	1.13E-01	3.69E-01
10.78	10.78	10.76	1.13E-01	3.69E-01
9.95	9.91	10.04	1.13E-01	3.69E-01
7.92	7.61	7.98	1.14E-01	3.69E-01
8.28	8.22	8.63	1.14E-01	3.69E-01
3.60	3.45	3.82	1.14E-01	3.69E-01
7.49	7.66	7.59	1.14E-01	3.69E-01
11.30	11.26	11.28	1.14E-01	3.69E-01
7.12	6.96	6.96	1.14E-01	3.69E-01
6.62	6.57	6.60	1.14E-01	3.69E-01
10.26	10.19	10.33	1.14E-01	3.69E-01
7.92	7.98	7.85	1.14E-01	3.69E-01
7.57	7.73	7.58	1.14E-01	3.69E-01
8.55	8.53	8.61	1.14E-01	3.69E-01
14.92	14.94	14.93	1.14E-01	3.69E-01
3.13	3.46	3.34	1.14E-01	3.69E-01
4.99	4.54	4.93	1.14E-01	3.69E-01
11.49	11.37	11.45	1.14E-01	3.69E-01
10.46	10.21	10.43	1.14E-01	3.69E-01
4.84	4.66	4.87	1.14E-01	3.69E-01
8.70	8.83	8.72	1.14E-01	3.69E-01
6.08	5.92	5.97	1.14E-01	3.69E-01
8.03	8.12	7.97	1.14E-01	3.69E-01
4.96	5.01	4.87	1.14E-01	3.69E-01
7.04	7.13	7.09	1.14E-01	3.70E-01
11.05	11.11	11.13	1.14E-01	3.70E-01
9.39	9.60	9.48	1.14E-01	3.70E-01
3.98	3.92	4.03	1.14E-01	3.70E-01
8.49	8.52	8.49	1.14E-01	3.70E-01
6.33	5.78	6.26	1.14E-01	3.70E-01
10.72	10.55	10.74	1.14E-01	3.70E-01
8.81	8.55	8.84	1.14E-01	3.70E-01
5.85	5.84	5.82	1.15E-01	3.70E-01
5.19	5.12	5.33	1.15E-01	3.70E-01
5.31	5.15	5.14	1.15E-01	3.70E-01
4.80	5.04	4.69	1.15E-01	3.70E-01
5.01	5.11	5.16	1.15E-01	3.70E-01
4.42	4.43	4.39	1.15E-01	3.70E-01
6.68	6.62	6.60	1.15E-01	3.70E-01
7.31	7.44	7.13	1.15E-01	3.70E-01
5.02	5.04	4.97	1.15E-01	3.70E-01
9.37	9.35	9.39	1.15E-01	3.70E-01
10.07	10.20	10.30	1.15E-01	3.71E-01

7.82	7.69	8.19	1.15E-01	3.71E-01
7.10	7.17	7.18	1.15E-01	3.71E-01
8.15	8.32	8.26	1.15E-01	3.71E-01
5.95	5.81	5.33	1.15E-01	3.71E-01
3.38	3.36	3.29	1.15E-01	3.71E-01
14.16	14.20	14.15	1.15E-01	3.71E-01
4.13	4.63	4.22	1.15E-01	3.71E-01
11.22	11.00	11.17	1.15E-01	3.72E-01
4.43	4.53	4.46	1.15E-01	3.72E-01
7.85	7.83	7.76	1.15E-01	3.72E-01
12.20	12.36	12.26	1.15E-01	3.72E-01
5.29	5.04	5.14	1.15E-01	3.72E-01
7.91	8.12	8.08	1.16E-01	3.72E-01
3.76	4.11	4.05	1.16E-01	3.72E-01
4.62	4.54	4.63	1.16E-01	3.72E-01
7.60	7.67	7.84	1.16E-01	3.72E-01
7.19	7.28	7.27	1.16E-01	3.72E-01
7.55	7.44	7.64	1.16E-01	3.72E-01
7.68	7.74	7.53	1.16E-01	3.72E-01
9.08	8.78	9.18	1.16E-01	3.72E-01
5.77	5.95	5.79	1.16E-01	3.72E-01
5.65	5.53	5.70	1.16E-01	3.72E-01
8.98	9.19	9.25	1.16E-01	3.72E-01
8.34	8.25	8.51	1.16E-01	3.72E-01
14.63	14.62	14.65	1.16E-01	3.72E-01
8.04	7.77	7.96	1.16E-01	3.72E-01
5.90	5.70	5.85	1.16E-01	3.72E-01
6.74	6.81	6.73	1.16E-01	3.72E-01
9.98	10.00	10.02	1.16E-01	3.72E-01
7.69	7.93	7.85	1.16E-01	3.72E-01
6.72	6.91	6.77	1.16E-01	3.72E-01
6.75	6.86	6.85	1.16E-01	3.72E-01
8.59	8.70	8.60	1.16E-01	3.72E-01
10.09	10.16	10.23	1.16E-01	3.72E-01
6.87	7.17	6.79	1.16E-01	3.72E-01
9.81	9.93	9.79	1.16E-01	3.72E-01
13.56	13.80	13.57	1.16E-01	3.72E-01
11.23	11.15	11.17	1.16E-01	3.72E-01
7.44	7.24	7.40	1.16E-01	3.72E-01
10.37	9.93	10.47	1.16E-01	3.72E-01
11.69	11.71	11.70	1.16E-01	3.72E-01
8.98	9.12	9.08	1.16E-01	3.72E-01
7.46	7.41	7.50	1.17E-01	3.73E-01
6.00	6.02	6.17	1.17E-01	3.73E-01
9.10	9.16	9.09	1.17E-01	3.73E-01
4.98	5.06	5.05	1.17E-01	3.73E-01
7.17	6.96	7.04	1.17E-01	3.73E-01

10.15	9.95	10.15	1.17E-01	3.73E-01
6.86	6.94	6.94	1.17E-01	3.73E-01
3.19	3.13	3.26	1.17E-01	3.73E-01
8.33	8.24	8.39	1.17E-01	3.73E-01
9.02	9.33	9.05	1.17E-01	3.73E-01
5.23	5.07	5.21	1.17E-01	3.73E-01
6.39	6.69	6.41	1.17E-01	3.74E-01
4.72	4.79	4.89	1.17E-01	3.74E-01
9.34	9.08	9.50	1.17E-01	3.74E-01
13.45	13.49	13.47	1.17E-01	3.74E-01
4.72	5.01	4.95	1.17E-01	3.74E-01
3.90	3.75	4.08	1.17E-01	3.74E-01
6.84	7.00	6.77	1.17E-01	3.74E-01
4.11	4.22	4.28	1.17E-01	3.74E-01
6.37	6.26	6.08	1.17E-01	3.74E-01
11.93	12.58	11.98	1.17E-01	3.74E-01
6.80	6.66	6.74	1.17E-01	3.74E-01
6.56	6.50	6.59	1.17E-01	3.74E-01
9.86	9.98	9.84	1.18E-01	3.74E-01
4.12	3.96	3.97	1.18E-01	3.74E-01
8.64	8.81	8.65	1.18E-01	3.74E-01
8.06	8.09	8.14	1.18E-01	3.74E-01
10.76	10.78	10.79	1.18E-01	3.74E-01
4.03	3.92	4.01	1.18E-01	3.74E-01
9.96	10.01	10.20	1.18E-01	3.74E-01
10.44	10.22	10.18	1.18E-01	3.74E-01
6.86	6.65	6.82	1.18E-01	3.74E-01
9.59	9.29	9.54	1.18E-01	3.75E-01
10.68	10.66	10.61	1.18E-01	3.75E-01
4.07	3.99	3.93	1.18E-01	3.75E-01
9.35	9.24	9.28	1.18E-01	3.75E-01
8.24	8.11	8.07	1.18E-01	3.75E-01
10.81	10.97	10.89	1.18E-01	3.75E-01
7.72	7.60	7.78	1.18E-01	3.75E-01
5.52	5.50	5.28	1.18E-01	3.75E-01
5.47	5.62	5.54	1.18E-01	3.75E-01
8.44	8.56	8.58	1.18E-01	3.75E-01
6.99	7.01	7.09	1.18E-01	3.75E-01
5.34	5.47	5.63	1.18E-01	3.75E-01
7.69	7.61	7.67	1.18E-01	3.75E-01
7.80	7.95	7.89	1.18E-01	3.75E-01
11.12	10.98	11.17	1.18E-01	3.75E-01
8.18	8.29	8.22	1.18E-01	3.75E-01
9.18	9.38	9.29	1.18E-01	3.75E-01
3.44	3.54	3.47	1.19E-01	3.75E-01
6.84	6.86	6.96	1.19E-01	3.75E-01
3.68	3.65	3.69	1.19E-01	3.75E-01

9.65	9.84	9.83	1.19E-01	3.76E-01
5.45	5.45	5.40	1.19E-01	3.76E-01
8.15	8.16	8.26	1.19E-01	3.76E-01
9.24	9.31	9.39	1.19E-01	3.76E-01
6.24	6.35	6.31	1.19E-01	3.76E-01
7.03	6.91	6.74	1.19E-01	3.76E-01
8.09	8.45	8.22	1.19E-01	3.76E-01
7.64	7.63	7.70	1.19E-01	3.76E-01
10.60	10.56	10.66	1.19E-01	3.76E-01
4.45	4.31	4.21	1.19E-01	3.76E-01
8.16	8.37	8.22	1.19E-01	3.76E-01
7.01	6.78	7.05	1.19E-01	3.76E-01
4.61	4.51	4.61	1.19E-01	3.76E-01
6.48	6.28	6.08	1.19E-01	3.76E-01
7.45	7.32	7.61	1.19E-01	3.76E-01
10.00	9.85	10.02	1.19E-01	3.76E-01
3.50	3.61	3.51	1.19E-01	3.76E-01
8.35	8.61	8.52	1.19E-01	3.76E-01
7.86	7.70	7.90	1.19E-01	3.76E-01
9.12	9.34	9.13	1.19E-01	3.76E-01
10.51	10.82	10.71	1.19E-01	3.76E-01
8.45	8.53	8.65	1.19E-01	3.76E-01
10.60	10.33	10.56	1.19E-01	3.76E-01
8.66	8.52	8.55	1.19E-01	3.76E-01
14.37	14.41	14.40	1.19E-01	3.76E-01
3.68	3.72	3.64	1.19E-01	3.76E-01
11.38	11.54	11.49	1.19E-01	3.76E-01
5.53	5.60	5.70	1.19E-01	3.76E-01
8.34	7.92	8.04	1.19E-01	3.76E-01
4.38	4.49	4.48	1.20E-01	3.76E-01
10.35	10.16	10.45	1.20E-01	3.76E-01
6.47	6.48	6.53	1.20E-01	3.76E-01
6.65	6.72	6.70	1.20E-01	3.76E-01
6.02	5.77	5.89	1.20E-01	3.76E-01
9.67	9.40	9.74	1.20E-01	3.76E-01
9.83	10.09	9.90	1.20E-01	3.76E-01
7.87	8.19	7.96	1.20E-01	3.76E-01
8.22	8.27	8.38	1.20E-01	3.76E-01
9.49	9.09	9.39	1.20E-01	3.76E-01
9.27	9.44	9.37	1.20E-01	3.76E-01
9.57	9.04	9.31	1.20E-01	3.76E-01
4.88	4.78	4.74	1.20E-01	3.76E-01
4.01	3.96	3.80	1.20E-01	3.76E-01
7.42	6.83	7.38	1.20E-01	3.76E-01
4.79	4.75	4.89	1.20E-01	3.76E-01
3.22	3.18	3.06	1.20E-01	3.76E-01
10.96	11.03	10.96	1.20E-01	3.76E-01

14.86	14.87	14.90	1.20E-01	3.76E-01
10.27	10.27	10.41	1.20E-01	3.76E-01
9.31	9.26	9.34	1.20E-01	3.76E-01
5.82	5.75	5.88	1.20E-01	3.76E-01
4.80	4.76	4.99	1.20E-01	3.76E-01
5.34	5.08	5.53	1.20E-01	3.76E-01
14.79	14.78	14.80	1.20E-01	3.76E-01
10.08	9.94	10.19	1.20E-01	3.76E-01
5.55	5.56	5.60	1.20E-01	3.76E-01
7.24	7.12	7.32	1.20E-01	3.76E-01
4.67	4.53	4.71	1.20E-01	3.76E-01
6.47	5.91	6.29	1.20E-01	3.76E-01
6.35	6.20	6.17	1.20E-01	3.76E-01
4.32	4.32	4.19	1.20E-01	3.76E-01
5.19	5.16	5.10	1.20E-01	3.77E-01
6.36	6.32	6.42	1.20E-01	3.77E-01
9.09	9.17	9.19	1.20E-01	3.77E-01
6.43	6.34	6.50	1.21E-01	3.77E-01
7.68	8.11	7.78	1.21E-01	3.77E-01
11.86	11.46	11.89	1.21E-01	3.77E-01
4.70	4.84	4.70	1.21E-01	3.77E-01
4.59	4.56	4.53	1.21E-01	3.77E-01
6.45	6.46	6.53	1.21E-01	3.77E-01
8.57	8.69	8.62	1.21E-01	3.77E-01
4.85	4.45	4.86	1.21E-01	3.77E-01
8.90	8.72	8.93	1.21E-01	3.78E-01
6.32	6.55	6.16	1.21E-01	3.78E-01
7.31	7.37	7.34	1.21E-01	3.78E-01
8.15	8.05	8.41	1.21E-01	3.78E-01
11.48	11.56	11.47	1.21E-01	3.78E-01
7.11	7.07	7.16	1.21E-01	3.78E-01
5.06	5.01	5.03	1.21E-01	3.78E-01
5.45	5.42	5.29	1.21E-01	3.78E-01
9.61	9.63	9.61	1.21E-01	3.78E-01
7.37	7.48	7.42	1.21E-01	3.78E-01
5.24	5.31	5.27	1.21E-01	3.78E-01
8.31	8.19	8.30	1.21E-01	3.78E-01
7.48	7.63	7.53	1.22E-01	3.79E-01
4.28	4.16	4.37	1.22E-01	3.79E-01
10.68	10.78	10.75	1.22E-01	3.79E-01
9.48	9.44	9.38	1.22E-01	3.79E-01
8.69	8.81	8.75	1.22E-01	3.79E-01
5.16	5.32	5.17	1.22E-01	3.79E-01
10.88	11.13	10.98	1.22E-01	3.79E-01
3.88	4.15	4.05	1.22E-01	3.79E-01
5.66	5.89	5.76	1.22E-01	3.79E-01
7.29	7.47	7.47	1.22E-01	3.79E-01

8.92	9.08	9.09	1.22E-01	3.79E-01
6.21	6.30	6.42	1.22E-01	3.79E-01
10.33	10.91	10.19	1.22E-01	3.80E-01
11.49	11.38	11.60	1.22E-01	3.80E-01
6.86	6.92	6.95	1.22E-01	3.80E-01
8.67	8.55	8.60	1.22E-01	3.80E-01
12.71	12.96	12.91	1.22E-01	3.80E-01
3.28	3.23	3.23	1.22E-01	3.80E-01
9.84	10.08	9.90	1.22E-01	3.80E-01
7.88	7.90	8.11	1.22E-01	3.80E-01
5.38	5.35	5.33	1.22E-01	3.80E-01
8.48	8.23	8.16	1.22E-01	3.80E-01
3.98	3.96	4.02	1.22E-01	3.80E-01
9.69	9.99	9.84	1.22E-01	3.80E-01
8.59	8.83	8.72	1.22E-01	3.80E-01
9.68	9.33	9.66	1.22E-01	3.80E-01
6.60	6.75	6.51	1.23E-01	3.80E-01
13.21	13.40	13.30	1.23E-01	3.80E-01
11.69	11.52	11.67	1.23E-01	3.80E-01
3.46	3.71	3.88	1.23E-01	3.80E-01
14.66	14.65	14.66	1.23E-01	3.80E-01
3.79	3.47	3.59	1.23E-01	3.80E-01
7.68	7.69	7.65	1.23E-01	3.80E-01
4.42	4.23	4.25	1.23E-01	3.80E-01
7.79	7.95	7.78	1.23E-01	3.80E-01
8.34	8.42	8.35	1.23E-01	3.80E-01
3.52	3.54	3.55	1.23E-01	3.80E-01
9.89	9.69	9.88	1.23E-01	3.80E-01
6.09	6.15	6.14	1.23E-01	3.80E-01
9.34	9.30	9.45	1.23E-01	3.80E-01
10.41	10.71	10.50	1.23E-01	3.80E-01
3.79	3.65	3.56	1.23E-01	3.80E-01
10.46	10.65	10.73	1.23E-01	3.80E-01
11.09	10.94	11.05	1.23E-01	3.80E-01
8.91	8.76	8.95	1.23E-01	3.80E-01
6.86	6.65	7.10	1.23E-01	3.81E-01
8.48	8.66	8.58	1.23E-01	3.81E-01
3.42	3.52	3.54	1.23E-01	3.81E-01
5.90	6.02	6.03	1.23E-01	3.81E-01
7.89	7.93	8.03	1.23E-01	3.81E-01
10.67	10.82	10.69	1.23E-01	3.81E-01
6.40	6.39	6.44	1.23E-01	3.81E-01
7.05	7.28	7.30	1.23E-01	3.81E-01
8.10	8.10	8.02	1.23E-01	3.81E-01
4.21	4.10	4.17	1.24E-01	3.81E-01
7.71	7.61	7.76	1.24E-01	3.81E-01
6.22	6.43	6.37	1.24E-01	3.81E-01

8.64	8.55	8.56	1.24E-01	3.81E-01
9.30	9.22	9.48	1.24E-01	3.81E-01
9.21	9.29	9.30	1.24E-01	3.81E-01
5.66	5.70	5.52	1.24E-01	3.81E-01
8.35	8.49	8.46	1.24E-01	3.81E-01
3.23	3.30	3.47	1.24E-01	3.81E-01
7.67	7.91	7.62	1.24E-01	3.81E-01
12.18	12.22	12.20	1.24E-01	3.81E-01
14.88	14.90	14.92	1.24E-01	3.82E-01
6.05	6.34	6.25	1.24E-01	3.82E-01
5.40	5.28	5.47	1.24E-01	3.82E-01
6.73	6.55	6.48	1.24E-01	3.82E-01
6.74	6.92	6.64	1.24E-01	3.82E-01
11.17	11.31	11.20	1.24E-01	3.82E-01
5.67	5.95	5.67	1.24E-01	3.82E-01
6.92	7.07	7.03	1.24E-01	3.82E-01
6.60	6.58	6.61	1.24E-01	3.82E-01
9.79	9.76	9.83	1.24E-01	3.82E-01
7.94	8.18	8.07	1.24E-01	3.82E-01
5.66	5.77	5.57	1.24E-01	3.82E-01
9.22	9.06	9.08	1.24E-01	3.82E-01
7.07	7.41	7.23	1.24E-01	3.82E-01
14.99	15.02	15.03	1.24E-01	3.82E-01
9.10	9.21	9.15	1.25E-01	3.82E-01
7.73	7.69	7.72	1.25E-01	3.82E-01
7.56	7.71	7.68	1.25E-01	3.82E-01
8.12	8.12	7.95	1.25E-01	3.82E-01
3.63	3.57	3.51	1.25E-01	3.82E-01
6.32	6.34	6.32	1.25E-01	3.82E-01
13.16	13.28	13.26	1.25E-01	3.82E-01
9.69	9.80	9.67	1.25E-01	3.82E-01
8.27	8.34	8.44	1.25E-01	3.82E-01
8.76	8.82	8.79	1.25E-01	3.82E-01
6.07	6.22	6.18	1.25E-01	3.82E-01
4.54	4.51	4.35	1.25E-01	3.82E-01
3.43	3.60	3.24	1.25E-01	3.82E-01
8.09	8.35	8.06	1.25E-01	3.82E-01
3.28	3.34	3.32	1.25E-01	3.82E-01
8.16	8.26	8.47	1.25E-01	3.82E-01
6.39	6.39	6.28	1.25E-01	3.82E-01
12.21	12.20	12.20	1.25E-01	3.83E-01
11.79	11.74	11.77	1.25E-01	3.83E-01
7.30	7.41	7.31	1.25E-01	3.83E-01
9.24	9.17	9.15	1.25E-01	3.83E-01
11.52	11.62	11.54	1.25E-01	3.83E-01
4.89	5.05	4.79	1.25E-01	3.83E-01
8.83	8.88	8.92	1.25E-01	3.83E-01

9.54	9.69	9.73	1.25E-01	3.83E-01
3.48	3.56	3.39	1.25E-01	3.83E-01
4.35	4.64	4.58	1.25E-01	3.83E-01
8.23	8.39	8.25	1.25E-01	3.83E-01
5.77	6.07	6.07	1.26E-01	3.83E-01
7.97	8.08	7.98	1.26E-01	3.83E-01
6.88	6.69	6.97	1.26E-01	3.83E-01
8.18	8.26	8.33	1.26E-01	3.83E-01
8.11	8.18	8.03	1.26E-01	3.83E-01
11.70	11.53	11.64	1.26E-01	3.83E-01
8.95	9.05	9.05	1.26E-01	3.83E-01
8.62	8.42	8.64	1.26E-01	3.83E-01
8.26	8.25	8.32	1.26E-01	3.83E-01
7.13	6.95	7.03	1.26E-01	3.83E-01
6.64	6.41	6.59	1.26E-01	3.83E-01
13.42	13.30	13.49	1.26E-01	3.83E-01
7.49	7.49	7.31	1.26E-01	3.83E-01
7.08	7.00	7.03	1.26E-01	3.84E-01
11.45	11.14	11.16	1.26E-01	3.84E-01
8.27	8.39	8.29	1.26E-01	3.84E-01
6.52	6.58	6.46	1.26E-01	3.84E-01
9.95	9.88	10.01	1.26E-01	3.84E-01
9.85	10.08	9.83	1.26E-01	3.84E-01
6.10	6.47	6.42	1.26E-01	3.84E-01
3.80	3.81	4.04	1.26E-01	3.84E-01
5.66	5.97	5.89	1.26E-01	3.84E-01
7.53	7.63	7.61	1.26E-01	3.84E-01
9.25	9.29	9.39	1.27E-01	3.85E-01
9.62	9.65	9.48	1.27E-01	3.85E-01
5.20	5.73	5.97	1.27E-01	3.85E-01
7.53	7.64	7.60	1.27E-01	3.85E-01
6.53	6.58	6.47	1.27E-01	3.85E-01
4.09	4.05	3.96	1.27E-01	3.85E-01
4.14	3.99	4.18	1.27E-01	3.85E-01
3.68	3.55	3.98	1.27E-01	3.85E-01
7.32	7.06	7.29	1.27E-01	3.85E-01
6.50	6.27	6.26	1.27E-01	3.85E-01
8.21	8.19	8.20	1.27E-01	3.85E-01
5.43	5.73	5.76	1.27E-01	3.85E-01
13.87	14.06	13.97	1.27E-01	3.85E-01
3.93	3.86	3.92	1.27E-01	3.85E-01
8.12	8.25	8.07	1.27E-01	3.85E-01
5.63	5.66	5.58	1.27E-01	3.85E-01
9.51	9.39	9.48	1.27E-01	3.85E-01
4.48	4.29	4.45	1.27E-01	3.85E-01
5.13	5.12	5.10	1.27E-01	3.85E-01
4.83	4.87	4.88	1.27E-01	3.85E-01

3.60	3.63	3.68	1.27E-01	3.85E-01
6.23	6.23	6.28	1.27E-01	3.85E-01
12.58	12.66	12.71	1.27E-01	3.85E-01
4.98	5.02	5.27	1.27E-01	3.85E-01
9.07	8.88	9.11	1.27E-01	3.85E-01
6.12	6.08	6.10	1.27E-01	3.85E-01
4.26	4.41	4.01	1.27E-01	3.86E-01
10.13	10.18	10.22	1.28E-01	3.86E-01
6.93	6.83	7.01	1.28E-01	3.86E-01
5.53	5.43	5.60	1.28E-01	3.86E-01
10.68	10.95	10.73	1.28E-01	3.86E-01
4.31	4.60	4.42	1.28E-01	3.86E-01
7.44	7.40	7.49	1.28E-01	3.86E-01
6.15	6.39	6.29	1.28E-01	3.86E-01
9.21	8.87	9.27	1.28E-01	3.86E-01
6.42	6.48	6.36	1.28E-01	3.87E-01
3.39	3.46	3.55	1.28E-01	3.87E-01
10.17	10.42	10.02	1.28E-01	3.87E-01
8.07	8.27	8.26	1.28E-01	3.87E-01
7.59	7.96	7.49	1.28E-01	3.87E-01
9.39	9.62	9.46	1.28E-01	3.87E-01
7.48	7.60	7.52	1.28E-01	3.87E-01
9.20	9.39	9.21	1.28E-01	3.87E-01
9.41	9.39	9.58	1.28E-01	3.87E-01
6.64	6.64	6.60	1.28E-01	3.87E-01
11.43	11.53	11.41	1.28E-01	3.87E-01
5.63	5.48	5.53	1.28E-01	3.87E-01
6.66	6.50	6.53	1.28E-01	3.87E-01
8.33	8.46	8.42	1.29E-01	3.87E-01
10.72	10.60	10.67	1.29E-01	3.87E-01
6.18	6.13	6.01	1.29E-01	3.87E-01
6.58	6.35	6.61	1.29E-01	3.87E-01
6.14	6.25	6.26	1.29E-01	3.88E-01
3.50	3.26	3.45	1.29E-01	3.88E-01
3.88	3.91	3.88	1.29E-01	3.88E-01
4.01	3.98	3.68	1.29E-01	3.88E-01
6.61	6.92	6.66	1.29E-01	3.88E-01
6.61	6.56	6.49	1.29E-01	3.88E-01
10.35	10.10	10.15	1.29E-01	3.88E-01
7.86	7.81	7.66	1.29E-01	3.88E-01
3.60	3.71	3.77	1.29E-01	3.88E-01
7.42	7.54	7.59	1.29E-01	3.88E-01
12.26	12.18	12.18	1.29E-01	3.88E-01
5.51	5.50	5.60	1.29E-01	3.88E-01
6.19	6.19	6.12	1.29E-01	3.88E-01
10.47	10.63	10.54	1.29E-01	3.88E-01
5.18	5.54	5.26	1.29E-01	3.88E-01

8.94	9.08	8.99	1.29E-01	3.88E-01
7.32	7.01	7.33	1.29E-01	3.88E-01
3.39	3.32	3.44	1.29E-01	3.88E-01
8.02	8.11	8.15	1.29E-01	3.88E-01
5.74	5.63	5.73	1.29E-01	3.88E-01
8.84	9.03	8.95	1.29E-01	3.88E-01
6.94	6.84	6.88	1.30E-01	3.88E-01
8.99	9.03	9.21	1.30E-01	3.88E-01
4.99	4.91	4.73	1.30E-01	3.88E-01
5.67	5.66	5.60	1.30E-01	3.88E-01
10.71	10.71	10.77	1.30E-01	3.88E-01
5.12	5.66	5.07	1.30E-01	3.88E-01
4.69	4.79	4.77	1.30E-01	3.88E-01
5.67	5.73	5.68	1.30E-01	3.89E-01
8.74	8.48	8.54	1.30E-01	3.89E-01
11.28	10.92	11.19	1.30E-01	3.89E-01
3.88	3.96	4.07	1.30E-01	3.89E-01
5.21	5.06	5.02	1.30E-01	3.89E-01
3.91	3.84	3.99	1.30E-01	3.89E-01
3.52	3.42	3.24	1.30E-01	3.89E-01
15.02	15.03	15.04	1.30E-01	3.89E-01
7.74	7.53	7.66	1.30E-01	3.89E-01
5.84	5.84	6.10	1.30E-01	3.89E-01
6.36	5.82	6.17	1.30E-01	3.89E-01
9.58	10.16	9.44	1.30E-01	3.89E-01
7.59	7.80	7.63	1.30E-01	3.89E-01
8.50	8.60	8.56	1.30E-01	3.89E-01
11.81	11.95	11.92	1.30E-01	3.89E-01
5.87	6.05	6.10	1.30E-01	3.89E-01
4.98	5.02	4.81	1.30E-01	3.89E-01
6.05	6.09	5.99	1.30E-01	3.89E-01
3.34	3.47	3.56	1.30E-01	3.89E-01
10.82	10.74	10.80	1.30E-01	3.89E-01
5.89	5.81	5.87	1.30E-01	3.89E-01
8.64	8.81	8.67	1.30E-01	3.89E-01
7.23	6.98	7.16	1.30E-01	3.89E-01
3.89	4.30	4.12	1.30E-01	3.89E-01
4.18	4.10	4.13	1.30E-01	3.89E-01
9.04	9.05	9.14	1.30E-01	3.89E-01
4.92	4.57	4.87	1.30E-01	3.89E-01
10.10	10.11	10.06	1.30E-01	3.89E-01
11.88	11.88	11.78	1.31E-01	3.89E-01
9.11	9.30	9.09	1.31E-01	3.89E-01
4.24	4.37	4.24	1.31E-01	3.89E-01
5.34	5.37	5.15	1.31E-01	3.89E-01
5.51	5.63	5.72	1.31E-01	3.89E-01
12.17	12.10	12.13	1.31E-01	3.89E-01

5.51	5.64	5.45	1.31E-01	3.89E-01
6.99	7.25	6.89	1.31E-01	3.89E-01
5.77	5.85	5.82	1.31E-01	3.89E-01
8.53	8.61	8.47	1.31E-01	3.89E-01
6.34	6.36	6.45	1.31E-01	3.89E-01
4.29	4.32	4.39	1.31E-01	3.89E-01
10.90	11.03	11.05	1.31E-01	3.89E-01
10.75	10.85	10.72	1.31E-01	3.89E-01
8.07	7.96	8.19	1.31E-01	3.89E-01
7.15	7.14	7.13	1.31E-01	3.89E-01
4.97	5.21	5.16	1.31E-01	3.89E-01
3.50	3.38	3.44	1.31E-01	3.90E-01
3.17	3.62	3.29	1.31E-01	3.90E-01
5.06	5.15	5.25	1.31E-01	3.90E-01
7.82	7.96	7.83	1.31E-01	3.90E-01
5.49	5.90	5.52	1.31E-01	3.90E-01
3.48	3.15	3.38	1.31E-01	3.90E-01
8.53	8.54	8.39	1.31E-01	3.90E-01
6.02	6.08	5.97	1.32E-01	3.90E-01
8.18	8.21	8.27	1.32E-01	3.90E-01
8.95	8.87	9.01	1.32E-01	3.90E-01
9.56	9.34	9.52	1.32E-01	3.90E-01
6.51	6.50	6.22	1.32E-01	3.90E-01
6.44	6.60	6.38	1.32E-01	3.90E-01
5.97	5.85	5.77	1.32E-01	3.91E-01
9.04	9.04	9.16	1.32E-01	3.91E-01
6.89	6.88	6.96	1.32E-01	3.91E-01
7.51	7.42	7.59	1.32E-01	3.91E-01
7.55	7.73	7.69	1.32E-01	3.91E-01
4.49	4.22	4.58	1.32E-01	3.91E-01
3.78	3.42	3.62	1.32E-01	3.91E-01
6.74	6.66	6.71	1.32E-01	3.91E-01
10.98	11.15	11.06	1.32E-01	3.91E-01
9.66	9.63	9.79	1.32E-01	3.91E-01
7.26	7.24	7.13	1.32E-01	3.91E-01
7.08	6.91	6.84	1.32E-01	3.91E-01
6.22	6.76	6.43	1.32E-01	3.91E-01
4.82	4.88	4.82	1.32E-01	3.91E-01
8.35	8.42	8.44	1.32E-01	3.91E-01
7.70	7.82	7.85	1.32E-01	3.91E-01
5.05	5.10	4.91	1.32E-01	3.91E-01
6.32	6.34	6.31	1.32E-01	3.91E-01
9.73	9.62	9.65	1.32E-01	3.91E-01
8.04	8.06	8.23	1.33E-01	3.91E-01
4.72	4.88	4.90	1.33E-01	3.91E-01
4.63	4.40	4.55	1.33E-01	3.91E-01
7.58	7.45	7.65	1.33E-01	3.91E-01

9.57	9.61	9.50	1.33E-01	3.91E-01
7.23	7.34	7.19	1.33E-01	3.91E-01
4.18	4.24	3.87	1.33E-01	3.91E-01
11.83	11.68	11.78	1.33E-01	3.91E-01
8.53	8.62	8.45	1.33E-01	3.91E-01
8.63	9.01	8.70	1.33E-01	3.91E-01
4.55	4.72	4.60	1.33E-01	3.91E-01
4.11	4.21	4.03	1.33E-01	3.91E-01
8.22	8.15	8.17	1.33E-01	3.91E-01
5.10	5.11	5.05	1.33E-01	3.91E-01
8.52	8.80	8.86	1.33E-01	3.91E-01
3.57	3.50	3.60	1.33E-01	3.91E-01
7.77	7.63	7.72	1.33E-01	3.91E-01
7.13	7.30	7.13	1.33E-01	3.91E-01
6.62	6.71	6.59	1.33E-01	3.91E-01
4.55	4.52	4.42	1.33E-01	3.92E-01
9.92	10.16	10.02	1.33E-01	3.92E-01
6.05	6.25	6.00	1.33E-01	3.92E-01
6.77	6.78	6.72	1.33E-01	3.92E-01
3.30	3.33	3.24	1.33E-01	3.92E-01
3.60	3.52	3.47	1.33E-01	3.92E-01
10.73	10.95	10.72	1.34E-01	3.93E-01
7.47	7.57	7.61	1.34E-01	3.93E-01
11.43	11.58	11.56	1.34E-01	3.93E-01
4.48	4.53	4.69	1.34E-01	3.93E-01
8.94	8.87	8.89	1.34E-01	3.93E-01
6.22	6.17	6.28	1.34E-01	3.93E-01
7.98	7.92	7.90	1.34E-01	3.93E-01
6.07	5.91	5.99	1.34E-01	3.93E-01
3.61	3.55	3.58	1.34E-01	3.93E-01
9.43	9.50	9.39	1.34E-01	3.93E-01
6.17	6.13	6.15	1.34E-01	3.93E-01
4.82	4.95	4.87	1.34E-01	3.93E-01
4.21	4.11	4.15	1.34E-01	3.93E-01
8.56	8.29	8.46	1.34E-01	3.93E-01
4.24	4.29	4.15	1.34E-01	3.93E-01
6.22	6.42	6.35	1.34E-01	3.93E-01
9.50	9.48	9.57	1.34E-01	3.93E-01
4.58	4.31	4.81	1.34E-01	3.93E-01
3.58	3.37	3.69	1.34E-01	3.93E-01
3.89	3.69	3.97	1.34E-01	3.93E-01
4.06	4.06	3.97	1.34E-01	3.93E-01
8.67	8.78	8.81	1.34E-01	3.93E-01
8.75	8.58	8.70	1.34E-01	3.93E-01
11.33	11.39	11.22	1.34E-01	3.93E-01
3.58	3.45	3.53	1.34E-01	3.93E-01
3.67	3.85	3.75	1.34E-01	3.93E-01

5.61	5.68	5.55	1.34E-01	3.93E-01
8.71	8.79	8.72	1.34E-01	3.93E-01
5.72	5.74	5.67	1.34E-01	3.93E-01
10.20	9.99	10.35	1.35E-01	3.94E-01
8.43	8.59	8.57	1.35E-01	3.94E-01
6.24	6.11	6.13	1.35E-01	3.94E-01
7.43	7.30	7.47	1.35E-01	3.94E-01
9.99	10.28	10.13	1.35E-01	3.94E-01
9.32	9.24	9.29	1.35E-01	3.94E-01
7.29	7.21	7.29	1.35E-01	3.94E-01
8.50	8.43	8.51	1.35E-01	3.94E-01
6.59	6.67	6.67	1.35E-01	3.94E-01
5.61	5.66	5.61	1.35E-01	3.94E-01
6.92	6.95	6.83	1.35E-01	3.94E-01
7.78	7.93	7.91	1.35E-01	3.94E-01
6.27	6.21	6.15	1.35E-01	3.94E-01
8.95	8.72	8.71	1.35E-01	3.94E-01
3.97	3.90	3.68	1.35E-01	3.94E-01
9.50	9.71	9.51	1.35E-01	3.94E-01
11.09	11.30	11.02	1.35E-01	3.94E-01
9.71	9.81	9.77	1.35E-01	3.94E-01
9.91	9.74	9.83	1.35E-01	3.94E-01
10.14	9.98	10.29	1.35E-01	3.94E-01
8.99	8.89	9.08	1.35E-01	3.94E-01
6.24	6.26	6.05	1.35E-01	3.94E-01
12.18	12.47	12.31	1.35E-01	3.94E-01
5.32	5.44	5.39	1.35E-01	3.94E-01
14.66	14.66	14.68	1.35E-01	3.94E-01
8.06	8.03	8.04	1.35E-01	3.94E-01
4.89	4.85	4.88	1.35E-01	3.94E-01
5.87	5.88	5.84	1.35E-01	3.94E-01
8.70	8.93	8.69	1.35E-01	3.94E-01
4.93	5.21	5.04	1.36E-01	3.94E-01
14.35	14.30	14.38	1.36E-01	3.94E-01
8.09	8.26	8.22	1.36E-01	3.94E-01
4.40	4.58	4.32	1.36E-01	3.94E-01
7.97	7.82	8.01	1.36E-01	3.94E-01
7.17	7.11	7.16	1.36E-01	3.95E-01
6.74	6.74	6.80	1.36E-01	3.95E-01
7.95	8.13	8.01	1.36E-01	3.95E-01
3.10	3.24	3.17	1.36E-01	3.95E-01
9.57	9.53	9.52	1.36E-01	3.95E-01
9.35	9.83	9.57	1.36E-01	3.95E-01
13.43	13.38	13.36	1.36E-01	3.95E-01
8.95	8.65	9.00	1.36E-01	3.95E-01
9.68	8.97	9.96	1.36E-01	3.95E-01
6.16	5.87	6.12	1.36E-01	3.95E-01

6.98	6.88	7.01	1.36E-01	3.95E-01
4.62	5.28	5.37	1.36E-01	3.95E-01
7.35	7.45	7.49	1.36E-01	3.95E-01
8.41	8.36	8.51	1.36E-01	3.95E-01
7.75	7.62	7.64	1.36E-01	3.95E-01
3.78	3.81	4.07	1.36E-01	3.95E-01
8.33	8.53	8.47	1.36E-01	3.95E-01
9.66	9.71	9.90	1.36E-01	3.95E-01
10.37	10.55	10.37	1.36E-01	3.95E-01
9.11	8.90	9.28	1.36E-01	3.95E-01
5.28	5.38	5.26	1.36E-01	3.95E-01
4.46	4.69	4.43	1.36E-01	3.95E-01
13.82	14.07	13.84	1.36E-01	3.95E-01
7.71	7.83	7.85	1.36E-01	3.95E-01
8.29	8.19	8.20	1.36E-01	3.95E-01
9.35	9.50	9.56	1.36E-01	3.95E-01
4.67	4.50	4.58	1.36E-01	3.95E-01
6.54	6.70	6.50	1.37E-01	3.95E-01
9.93	9.62	9.96	1.37E-01	3.95E-01
5.06	4.90	4.87	1.37E-01	3.95E-01
14.53	14.67	14.61	1.37E-01	3.95E-01
5.33	5.41	5.50	1.37E-01	3.95E-01
11.03	10.67	11.25	1.37E-01	3.95E-01
6.52	6.40	6.47	1.37E-01	3.95E-01
12.65	12.27	12.77	1.37E-01	3.95E-01
7.20	7.06	7.33	1.37E-01	3.95E-01
6.49	6.73	6.61	1.37E-01	3.95E-01
5.51	5.57	5.57	1.37E-01	3.95E-01
6.91	6.89	6.95	1.37E-01	3.95E-01
8.03	7.95	7.87	1.37E-01	3.95E-01
4.06	3.90	4.08	1.37E-01	3.95E-01
4.71	4.83	4.58	1.37E-01	3.95E-01
7.39	7.65	7.43	1.37E-01	3.95E-01
4.64	4.71	4.51	1.37E-01	3.95E-01
5.96	5.91	5.87	1.37E-01	3.95E-01
9.72	9.87	9.82	1.37E-01	3.95E-01
3.83	3.75	3.92	1.37E-01	3.95E-01
10.15	10.36	10.29	1.37E-01	3.95E-01
5.61	5.73	5.57	1.37E-01	3.95E-01
4.65	4.32	4.59	1.37E-01	3.95E-01
14.84	14.89	14.88	1.37E-01	3.95E-01
3.93	3.93	3.82	1.37E-01	3.96E-01
9.52	9.55	9.69	1.37E-01	3.96E-01
6.74	6.73	6.66	1.37E-01	3.96E-01
9.00	9.12	9.14	1.37E-01	3.96E-01
12.59	12.74	12.61	1.38E-01	3.96E-01
7.07	7.05	7.22	1.38E-01	3.96E-01

9.80	10.08	9.87	1.38E-01	3.96E-01
9.41	9.38	9.47	1.38E-01	3.96E-01
8.62	8.56	8.71	1.38E-01	3.96E-01
8.49	8.73	8.66	1.38E-01	3.97E-01
7.71	7.70	7.52	1.38E-01	3.97E-01
10.16	10.04	10.12	1.38E-01	3.97E-01
10.69	10.41	10.62	1.38E-01	3.97E-01
8.24	8.05	8.06	1.38E-01	3.97E-01
7.05	7.07	7.38	1.38E-01	3.97E-01
8.79	8.95	8.87	1.38E-01	3.97E-01
10.08	9.95	10.04	1.38E-01	3.97E-01
10.25	10.32	10.33	1.38E-01	3.97E-01
8.74	8.65	8.56	1.38E-01	3.97E-01
8.76	8.69	8.87	1.38E-01	3.97E-01
11.73	11.93	11.73	1.38E-01	3.97E-01
4.77	4.65	4.61	1.38E-01	3.97E-01
9.74	9.90	9.73	1.38E-01	3.97E-01
3.79	3.67	3.74	1.39E-01	3.97E-01
5.80	5.77	5.84	1.39E-01	3.97E-01
10.40	10.12	10.39	1.39E-01	3.97E-01
8.35	8.56	8.35	1.39E-01	3.97E-01
3.99	3.92	3.80	1.39E-01	3.97E-01
8.97	9.08	9.07	1.39E-01	3.97E-01
6.79	6.69	6.89	1.39E-01	3.97E-01
4.42	4.59	4.45	1.39E-01	3.97E-01
4.18	4.17	4.19	1.39E-01	3.97E-01
7.66	7.74	7.71	1.39E-01	3.97E-01
8.48	8.40	8.40	1.39E-01	3.97E-01
7.77	7.85	7.89	1.39E-01	3.97E-01
6.22	6.15	6.26	1.39E-01	3.97E-01
8.53	8.75	8.61	1.39E-01	3.97E-01
9.94	10.28	10.04	1.39E-01	3.97E-01
3.80	3.88	3.98	1.39E-01	3.97E-01
7.18	7.16	7.19	1.39E-01	3.97E-01
5.92	5.98	6.00	1.39E-01	3.97E-01
4.46	4.45	4.45	1.39E-01	3.97E-01
3.95	3.79	3.80	1.39E-01	3.97E-01
5.37	5.26	5.31	1.39E-01	3.98E-01
6.06	6.31	6.32	1.39E-01	3.98E-01
4.73	4.58	4.63	1.39E-01	3.98E-01
7.34	7.38	7.42	1.39E-01	3.98E-01
10.28	10.35	10.23	1.39E-01	3.98E-01
7.16	7.24	7.18	1.39E-01	3.98E-01
3.81	3.76	3.95	1.39E-01	3.98E-01
7.88	7.98	8.16	1.39E-01	3.98E-01
10.04	10.00	10.10	1.39E-01	3.98E-01
6.34	6.13	6.44	1.39E-01	3.98E-01

7.97	8.03	8.06	1.39E-01	3.98E-01
9.10	9.13	9.31	1.39E-01	3.98E-01
5.80	5.78	5.60	1.39E-01	3.98E-01
11.41	11.41	11.42	1.39E-01	3.98E-01
11.50	11.58	11.57	1.40E-01	3.98E-01
6.94	6.84	7.02	1.40E-01	3.98E-01
6.10	6.10	6.23	1.40E-01	3.98E-01
11.25	11.29	11.26	1.40E-01	3.98E-01
4.70	4.99	5.04	1.40E-01	3.98E-01
11.50	11.39	11.49	1.40E-01	3.98E-01
12.80	12.99	12.90	1.40E-01	3.98E-01
10.99	10.96	11.08	1.40E-01	3.98E-01
9.13	9.41	9.10	1.40E-01	3.98E-01
8.05	8.27	8.07	1.40E-01	3.98E-01
9.95	9.98	9.90	1.40E-01	3.98E-01
11.12	11.34	11.17	1.40E-01	3.98E-01
7.38	7.33	7.41	1.40E-01	3.98E-01
4.32	4.09	4.00	1.40E-01	3.98E-01
8.03	8.10	8.12	1.40E-01	3.99E-01
6.71	6.86	6.84	1.40E-01	3.99E-01
8.68	8.66	8.73	1.40E-01	3.99E-01
5.55	5.66	5.53	1.40E-01	3.99E-01
6.70	6.50	6.61	1.40E-01	3.99E-01
7.36	7.41	7.36	1.40E-01	3.99E-01
11.09	11.09	11.23	1.40E-01	3.99E-01
10.25	10.38	10.35	1.40E-01	3.99E-01
7.56	7.90	7.74	1.40E-01	3.99E-01
9.07	8.66	8.59	1.40E-01	3.99E-01
10.44	10.20	10.52	1.40E-01	3.99E-01
7.99	8.10	8.13	1.40E-01	3.99E-01
6.43	6.62	6.34	1.40E-01	3.99E-01
9.86	9.91	9.86	1.41E-01	3.99E-01
9.02	8.92	8.99	1.41E-01	3.99E-01
8.11	8.03	8.13	1.41E-01	3.99E-01
5.17	5.15	4.98	1.41E-01	3.99E-01
8.93	9.42	9.07	1.41E-01	3.99E-01
9.52	9.66	9.54	1.41E-01	3.99E-01
7.73	7.63	7.75	1.41E-01	3.99E-01
6.47	6.49	6.45	1.41E-01	3.99E-01
5.62	5.47	5.54	1.41E-01	3.99E-01
10.28	10.33	10.21	1.41E-01	3.99E-01
8.41	8.44	8.46	1.41E-01	3.99E-01
7.06	7.05	7.11	1.41E-01	3.99E-01
8.30	8.33	8.43	1.41E-01	3.99E-01
5.01	4.90	4.91	1.41E-01	3.99E-01
5.78	5.70	5.93	1.41E-01	3.99E-01
14.29	14.26	14.27	1.41E-01	3.99E-01

3.85	3.91	4.01	1.41E-01	3.99E-01
8.03	7.91	8.07	1.41E-01	3.99E-01
6.05	5.87	5.98	1.41E-01	3.99E-01
5.36	5.09	5.23	1.41E-01	3.99E-01
7.98	8.14	8.01	1.41E-01	3.99E-01
8.22	8.42	8.37	1.41E-01	3.99E-01
6.47	6.50	6.61	1.41E-01	4.00E-01
7.75	7.80	7.85	1.41E-01	4.00E-01
9.11	9.28	9.23	1.41E-01	4.00E-01
4.59	4.49	4.09	1.42E-01	4.00E-01
7.51	7.53	7.51	1.42E-01	4.00E-01
4.06	4.07	4.04	1.42E-01	4.00E-01
7.28	7.20	7.25	1.42E-01	4.00E-01
5.36	5.26	5.24	1.42E-01	4.00E-01
8.49	8.39	8.45	1.42E-01	4.00E-01
3.25	3.21	3.50	1.42E-01	4.00E-01
5.47	5.56	5.53	1.42E-01	4.00E-01
8.33	8.46	8.42	1.42E-01	4.00E-01
4.73	4.30	4.41	1.42E-01	4.00E-01
4.86	5.01	4.78	1.42E-01	4.00E-01
7.08	7.11	7.26	1.42E-01	4.00E-01
9.85	9.97	9.95	1.42E-01	4.00E-01
8.63	8.81	8.70	1.42E-01	4.00E-01
8.88	8.81	8.80	1.42E-01	4.00E-01
8.06	8.15	8.19	1.42E-01	4.00E-01
3.51	3.93	3.77	1.42E-01	4.00E-01
3.19	3.16	3.23	1.42E-01	4.00E-01
12.05	12.00	12.01	1.42E-01	4.00E-01
7.92	8.01	7.99	1.42E-01	4.00E-01
11.62	11.88	11.75	1.42E-01	4.00E-01
3.51	3.47	3.68	1.42E-01	4.00E-01
7.97	8.07	8.17	1.42E-01	4.00E-01
13.66	13.74	13.69	1.42E-01	4.01E-01
6.27	6.21	6.31	1.42E-01	4.01E-01
10.40	10.23	10.31	1.42E-01	4.01E-01
6.16	6.34	6.05	1.42E-01	4.01E-01
8.92	8.83	8.88	1.42E-01	4.01E-01
9.76	9.71	9.69	1.42E-01	4.01E-01
10.39	10.29	10.24	1.43E-01	4.01E-01
5.92	5.60	5.70	1.43E-01	4.01E-01
3.81	3.73	3.78	1.43E-01	4.01E-01
10.08	10.06	10.17	1.43E-01	4.01E-01
7.50	7.59	7.60	1.43E-01	4.01E-01
9.61	9.53	9.62	1.43E-01	4.01E-01
6.53	6.27	6.64	1.43E-01	4.01E-01
9.77	9.80	9.87	1.43E-01	4.01E-01
6.88	6.65	6.80	1.43E-01	4.01E-01

9.32	9.11	9.30	1.43E-01	4.02E-01
4.44	4.31	4.15	1.43E-01	4.02E-01
7.81	7.83	7.76	1.43E-01	4.02E-01
5.94	6.16	5.98	1.43E-01	4.02E-01
11.64	11.76	11.76	1.43E-01	4.02E-01
4.94	5.25	5.09	1.43E-01	4.02E-01
8.28	8.21	8.35	1.43E-01	4.02E-01
8.10	7.40	7.80	1.43E-01	4.02E-01
5.36	5.07	5.24	1.43E-01	4.02E-01
8.33	8.40	8.35	1.43E-01	4.02E-01
3.70	3.52	3.77	1.43E-01	4.02E-01
5.85	5.91	5.95	1.44E-01	4.02E-01
8.92	9.04	8.99	1.44E-01	4.02E-01
5.66	5.33	5.58	1.44E-01	4.02E-01
9.64	9.65	9.72	1.44E-01	4.02E-01
9.76	9.53	9.59	1.44E-01	4.02E-01
8.07	8.06	8.00	1.44E-01	4.02E-01
8.41	8.05	8.22	1.44E-01	4.02E-01
5.80	5.44	5.64	1.44E-01	4.02E-01
7.69	7.56	7.65	1.44E-01	4.02E-01
8.74	8.56	8.63	1.44E-01	4.02E-01
8.33	8.43	8.38	1.44E-01	4.02E-01
5.37	5.30	5.75	1.44E-01	4.03E-01
6.98	7.09	7.05	1.44E-01	4.03E-01
10.29	10.40	10.36	1.44E-01	4.03E-01
5.02	4.97	4.41	1.44E-01	4.03E-01
8.64	8.53	8.71	1.44E-01	4.04E-01
3.24	3.27	3.18	1.44E-01	4.04E-01
3.10	3.38	3.12	1.44E-01	4.04E-01
3.55	3.34	3.25	1.44E-01	4.04E-01
5.71	5.79	5.85	1.45E-01	4.04E-01
11.48	11.23	11.30	1.45E-01	4.04E-01
11.62	11.39	11.59	1.45E-01	4.04E-01
7.70	7.87	7.79	1.45E-01	4.04E-01
3.78	3.87	3.95	1.45E-01	4.04E-01
4.74	4.84	4.53	1.45E-01	4.04E-01
4.23	4.07	4.27	1.45E-01	4.04E-01
8.60	8.70	8.55	1.45E-01	4.04E-01
9.94	9.76	9.84	1.45E-01	4.04E-01
9.18	9.51	9.32	1.45E-01	4.04E-01
11.15	10.97	11.11	1.45E-01	4.04E-01
11.33	11.37	11.38	1.45E-01	4.04E-01
7.97	7.87	7.94	1.45E-01	4.04E-01
4.79	5.12	5.40	1.45E-01	4.04E-01
4.16	3.96	4.56	1.45E-01	4.04E-01
6.39	6.35	6.28	1.45E-01	4.04E-01
4.04	3.86	3.93	1.45E-01	4.04E-01

12.63	12.68	12.61	1.45E-01	4.04E-01
4.17	3.99	4.24	1.45E-01	4.05E-01
10.07	10.04	10.12	1.45E-01	4.05E-01
10.01	10.03	9.98	1.45E-01	4.05E-01
9.66	9.61	9.50	1.46E-01	4.05E-01
9.14	9.09	9.12	1.46E-01	4.05E-01
5.17	4.96	4.90	1.46E-01	4.05E-01
6.16	6.24	6.33	1.46E-01	4.05E-01
4.96	4.74	4.98	1.46E-01	4.05E-01
7.21	7.00	7.23	1.46E-01	4.05E-01
9.38	9.57	9.45	1.46E-01	4.05E-01
8.01	7.87	8.12	1.46E-01	4.05E-01
4.90	5.20	5.02	1.46E-01	4.05E-01
10.06	9.89	10.13	1.46E-01	4.05E-01
7.74	7.68	7.73	1.46E-01	4.05E-01
4.46	4.70	4.47	1.46E-01	4.05E-01
7.72	7.57	7.69	1.46E-01	4.05E-01
9.58	9.33	9.66	1.46E-01	4.05E-01
14.50	14.48	14.50	1.46E-01	4.05E-01
10.37	10.59	10.52	1.46E-01	4.05E-01
6.13	6.00	5.95	1.46E-01	4.05E-01
9.43	9.49	9.44	1.46E-01	4.05E-01
7.64	7.72	7.68	1.46E-01	4.05E-01
6.45	6.16	6.08	1.46E-01	4.05E-01
10.25	10.27	10.07	1.46E-01	4.05E-01
10.89	10.69	10.84	1.46E-01	4.05E-01
6.66	6.60	6.72	1.46E-01	4.05E-01
9.05	9.17	9.18	1.46E-01	4.05E-01
9.52	9.39	9.46	1.46E-01	4.05E-01
5.76	5.63	5.75	1.46E-01	4.05E-01
8.73	8.89	8.77	1.46E-01	4.06E-01
3.57	3.68	3.75	1.46E-01	4.06E-01
7.41	7.28	7.45	1.46E-01	4.06E-01
3.76	3.81	3.50	1.46E-01	4.06E-01
6.98	6.98	7.14	1.47E-01	4.06E-01
4.04	4.08	4.04	1.47E-01	4.06E-01
8.17	8.20	8.24	1.47E-01	4.06E-01
4.17	4.23	4.11	1.47E-01	4.06E-01
7.04	7.13	7.00	1.47E-01	4.06E-01
6.20	6.61	6.39	1.47E-01	4.06E-01
10.30	10.30	10.38	1.47E-01	4.06E-01
4.66	4.66	4.41	1.47E-01	4.06E-01
8.98	8.95	8.80	1.47E-01	4.06E-01
4.31	4.37	4.50	1.47E-01	4.06E-01
10.20	9.96	10.16	1.47E-01	4.06E-01
4.92	5.03	4.73	1.47E-01	4.06E-01
4.98	4.85	5.11	1.47E-01	4.06E-01

4.65	4.93	4.58	1.47E-01	4.06E-01
3.76	3.47	3.60	1.47E-01	4.06E-01
11.34	11.06	11.30	1.47E-01	4.06E-01
7.56	7.85	7.83	1.47E-01	4.06E-01
12.28	12.41	12.33	1.47E-01	4.06E-01
7.99	7.89	8.06	1.47E-01	4.06E-01
8.59	8.73	8.58	1.47E-01	4.06E-01
6.24	6.12	6.04	1.47E-01	4.06E-01
4.22	4.16	4.12	1.47E-01	4.06E-01
12.36	12.27	12.36	1.47E-01	4.06E-01
9.61	9.42	9.62	1.47E-01	4.06E-01
3.64	3.68	3.69	1.47E-01	4.06E-01
7.49	7.53	7.57	1.47E-01	4.06E-01
10.22	10.42	10.28	1.47E-01	4.06E-01
9.39	9.57	9.43	1.47E-01	4.06E-01
7.04	6.91	7.10	1.47E-01	4.06E-01
7.91	7.93	7.95	1.47E-01	4.06E-01
8.74	8.79	8.79	1.48E-01	4.07E-01
4.69	4.93	4.86	1.48E-01	4.07E-01
9.15	9.30	9.34	1.48E-01	4.07E-01
8.95	9.31	8.90	1.48E-01	4.07E-01
7.49	7.54	7.47	1.48E-01	4.07E-01
4.66	4.63	4.69	1.48E-01	4.07E-01
10.70	10.65	10.66	1.48E-01	4.07E-01
8.72	8.89	8.77	1.48E-01	4.07E-01
5.19	5.06	5.04	1.48E-01	4.07E-01
8.52	8.53	8.48	1.48E-01	4.07E-01
8.02	8.11	8.11	1.48E-01	4.07E-01
7.10	7.53	7.29	1.48E-01	4.07E-01
6.10	6.24	6.03	1.48E-01	4.07E-01
12.35	12.32	12.41	1.48E-01	4.07E-01
8.31	7.85	8.04	1.48E-01	4.07E-01
6.01	6.17	6.33	1.48E-01	4.07E-01
8.18	8.46	8.25	1.48E-01	4.07E-01
6.82	6.80	6.98	1.48E-01	4.07E-01
7.14	7.14	7.35	1.48E-01	4.08E-01
10.40	10.57	10.48	1.48E-01	4.08E-01
4.78	4.63	4.51	1.48E-01	4.08E-01
7.01	7.04	7.23	1.48E-01	4.08E-01
5.25	5.05	5.06	1.49E-01	4.08E-01
14.22	14.17	14.20	1.49E-01	4.08E-01
8.21	8.20	8.33	1.49E-01	4.08E-01
8.28	8.32	8.34	1.49E-01	4.08E-01
7.22	7.21	7.31	1.49E-01	4.08E-01
7.64	7.86	7.77	1.49E-01	4.08E-01
10.48	10.60	10.55	1.49E-01	4.08E-01
6.60	6.68	6.69	1.49E-01	4.08E-01

4.99	4.74	4.95	1.49E-01	4.08E-01
7.14	6.70	7.01	1.49E-01	4.08E-01
7.10	7.18	7.06	1.49E-01	4.08E-01
4.99	4.89	4.98	1.49E-01	4.08E-01
11.07	11.35	11.22	1.49E-01	4.08E-01
10.42	10.26	10.36	1.49E-01	4.08E-01
10.52	10.35	10.47	1.49E-01	4.08E-01
6.81	6.70	6.69	1.49E-01	4.09E-01
6.21	6.13	5.99	1.49E-01	4.09E-01
5.28	5.25	5.27	1.49E-01	4.09E-01
6.02	6.00	6.20	1.49E-01	4.09E-01
7.39	7.46	7.31	1.49E-01	4.09E-01
4.51	4.72	4.48	1.49E-01	4.09E-01
4.86	4.90	4.94	1.49E-01	4.09E-01
5.07	4.85	4.82	1.49E-01	4.09E-01
4.72	4.38	4.63	1.50E-01	4.09E-01
6.26	6.15	6.16	1.50E-01	4.09E-01
3.85	3.95	3.84	1.50E-01	4.09E-01
9.97	9.92	10.11	1.50E-01	4.09E-01
9.90	9.64	9.88	1.50E-01	4.09E-01
5.15	4.97	5.05	1.50E-01	4.09E-01
5.09	5.25	5.41	1.50E-01	4.09E-01
3.21	3.02	3.16	1.50E-01	4.09E-01
7.57	7.43	7.43	1.50E-01	4.09E-01
7.80	7.71	7.72	1.50E-01	4.09E-01
10.26	10.02	10.20	1.50E-01	4.09E-01
10.58	10.41	10.56	1.50E-01	4.09E-01
8.08	8.13	8.11	1.50E-01	4.09E-01
4.65	4.70	4.55	1.50E-01	4.09E-01
5.45	5.46	5.28	1.50E-01	4.09E-01
7.03	6.90	7.05	1.50E-01	4.09E-01
12.44	12.44	12.33	1.50E-01	4.09E-01
6.58	6.48	6.76	1.50E-01	4.09E-01
5.67	5.42	5.52	1.50E-01	4.09E-01
6.78	6.62	6.64	1.50E-01	4.09E-01
4.93	4.87	4.79	1.50E-01	4.09E-01
3.99	4.07	3.94	1.50E-01	4.09E-01
7.99	8.24	8.02	1.50E-01	4.09E-01
4.51	4.25	4.27	1.50E-01	4.09E-01
4.81	4.82	4.82	1.50E-01	4.09E-01
5.83	5.51	5.49	1.50E-01	4.10E-01
12.66	12.80	12.75	1.50E-01	4.10E-01
9.69	9.51	9.60	1.51E-01	4.10E-01
8.31	8.51	8.35	1.51E-01	4.10E-01
11.77	11.82	11.86	1.51E-01	4.10E-01
7.16	7.32	7.18	1.51E-01	4.10E-01
5.15	5.17	5.07	1.51E-01	4.10E-01

10.43	10.42	10.58	1.51E-01	4.10E-01
11.04	11.18	11.09	1.51E-01	4.10E-01
9.20	8.98	9.08	1.51E-01	4.10E-01
12.05	11.79	12.14	1.51E-01	4.10E-01
10.40	10.62	10.49	1.51E-01	4.10E-01
7.95	7.94	7.92	1.51E-01	4.11E-01
9.56	9.39	9.32	1.51E-01	4.11E-01
7.63	7.88	7.73	1.51E-01	4.11E-01
5.35	5.91	5.48	1.51E-01	4.11E-01
4.89	4.91	4.90	1.51E-01	4.11E-01
12.10	12.21	12.10	1.51E-01	4.11E-01
8.25	8.45	8.30	1.51E-01	4.11E-01
7.04	6.66	6.87	1.51E-01	4.11E-01
4.89	4.84	5.00	1.51E-01	4.11E-01
5.89	5.73	5.73	1.51E-01	4.11E-01
11.25	11.04	11.23	1.51E-01	4.11E-01
5.91	5.85	5.69	1.51E-01	4.11E-01
14.14	14.09	14.11	1.51E-01	4.11E-01
5.90	5.89	5.73	1.51E-01	4.11E-01
7.06	7.06	7.08	1.52E-01	4.11E-01
8.47	8.53	8.50	1.52E-01	4.11E-01
4.70	4.30	4.62	1.52E-01	4.11E-01
3.51	3.67	3.56	1.52E-01	4.11E-01
9.09	9.05	9.01	1.52E-01	4.11E-01
9.22	9.21	9.29	1.52E-01	4.11E-01
11.56	11.66	11.60	1.52E-01	4.11E-01
10.40	10.47	10.43	1.52E-01	4.11E-01
6.87	7.08	6.94	1.52E-01	4.11E-01
9.05	8.99	9.19	1.52E-01	4.11E-01
4.57	4.61	4.38	1.52E-01	4.12E-01
3.72	3.59	3.59	1.52E-01	4.12E-01
4.66	4.49	4.69	1.52E-01	4.12E-01
3.77	3.74	3.81	1.52E-01	4.12E-01
4.08	3.96	4.08	1.52E-01	4.12E-01
3.57	3.59	3.69	1.52E-01	4.12E-01
11.37	11.62	11.45	1.52E-01	4.12E-01
4.81	4.79	4.65	1.52E-01	4.12E-01
5.94	5.88	5.78	1.52E-01	4.12E-01
3.81	3.66	3.54	1.52E-01	4.12E-01
6.25	6.36	6.23	1.52E-01	4.12E-01
5.75	5.40	5.64	1.52E-01	4.12E-01
6.82	6.66	6.62	1.52E-01	4.12E-01
3.15	3.30	3.32	1.52E-01	4.12E-01
7.25	7.38	7.27	1.52E-01	4.12E-01
7.56	7.54	7.68	1.52E-01	4.12E-01
10.87	11.10	11.01	1.53E-01	4.12E-01
8.19	8.05	8.17	1.53E-01	4.12E-01

5.23	5.18	5.11	1.53E-01	4.12E-01
5.24	5.00	5.35	1.53E-01	4.12E-01
6.18	6.53	6.23	1.53E-01	4.12E-01
4.21	4.37	4.23	1.53E-01	4.12E-01
10.78	10.56	10.73	1.53E-01	4.12E-01
6.71	6.66	6.62	1.53E-01	4.12E-01
10.57	10.64	10.59	1.53E-01	4.12E-01
6.17	6.27	6.20	1.53E-01	4.12E-01
3.81	3.88	3.63	1.53E-01	4.12E-01
8.98	9.22	9.21	1.53E-01	4.12E-01
6.67	6.48	6.65	1.53E-01	4.12E-01
4.61	4.59	4.76	1.53E-01	4.12E-01
9.27	9.09	9.20	1.53E-01	4.12E-01
5.58	5.51	5.48	1.53E-01	4.12E-01
4.48	4.55	4.61	1.53E-01	4.12E-01
9.62	9.60	9.76	1.53E-01	4.12E-01
4.42	4.56	4.59	1.53E-01	4.12E-01
4.10	3.93	4.13	1.53E-01	4.12E-01
6.60	6.57	6.59	1.53E-01	4.13E-01
5.06	4.89	5.14	1.53E-01	4.13E-01
10.02	9.75	9.96	1.53E-01	4.13E-01
12.80	12.84	12.76	1.53E-01	4.13E-01
10.91	10.75	10.81	1.53E-01	4.13E-01
8.55	8.46	8.77	1.53E-01	4.13E-01
7.44	7.39	7.53	1.53E-01	4.13E-01
6.13	6.20	6.15	1.53E-01	4.13E-01
4.16	4.21	4.23	1.54E-01	4.13E-01
10.32	10.26	10.24	1.54E-01	4.13E-01
3.56	3.55	3.57	1.54E-01	4.13E-01
11.74	11.86	11.86	1.54E-01	4.13E-01
8.81	8.71	8.93	1.54E-01	4.13E-01
6.06	6.28	6.33	1.54E-01	4.13E-01
4.68	4.71	4.72	1.54E-01	4.13E-01
7.68	7.35	7.35	1.54E-01	4.13E-01
5.18	5.42	5.15	1.54E-01	4.13E-01
5.80	5.40	5.79	1.54E-01	4.13E-01
3.71	3.67	3.64	1.54E-01	4.13E-01
10.46	10.05	10.46	1.54E-01	4.13E-01
6.17	6.36	6.30	1.54E-01	4.13E-01
3.97	3.73	3.81	1.54E-01	4.13E-01
6.15	6.29	6.09	1.54E-01	4.13E-01
8.47	8.62	8.71	1.54E-01	4.13E-01
6.38	6.37	6.05	1.54E-01	4.13E-01
8.06	8.25	8.11	1.54E-01	4.13E-01
8.54	8.50	8.58	1.54E-01	4.13E-01
9.68	9.62	9.51	1.54E-01	4.13E-01
8.53	8.73	8.66	1.54E-01	4.13E-01

7.91	7.70	7.87	1.54E-01	4.13E-01
11.20	10.71	11.03	1.54E-01	4.13E-01
7.70	7.76	7.62	1.54E-01	4.13E-01
7.40	7.33	7.40	1.54E-01	4.13E-01
8.69	8.81	8.75	1.54E-01	4.13E-01
5.84	5.83	5.84	1.54E-01	4.13E-01
8.92	8.75	8.82	1.54E-01	4.13E-01
9.31	9.21	9.38	1.54E-01	4.13E-01
7.65	7.53	7.83	1.54E-01	4.13E-01
5.67	5.60	5.52	1.54E-01	4.13E-01
4.81	4.70	4.82	1.55E-01	4.13E-01
4.49	4.88	4.50	1.55E-01	4.13E-01
8.78	8.64	8.81	1.55E-01	4.13E-01
7.14	7.05	6.99	1.55E-01	4.14E-01
4.50	4.55	4.64	1.55E-01	4.14E-01
10.52	10.41	10.39	1.55E-01	4.14E-01
8.49	8.53	8.52	1.55E-01	4.14E-01
5.72	5.56	5.74	1.55E-01	4.14E-01
4.18	4.14	4.27	1.55E-01	4.14E-01
7.40	7.57	7.29	1.55E-01	4.14E-01
3.93	4.36	4.10	1.55E-01	4.14E-01
12.67	12.83	12.75	1.55E-01	4.14E-01
9.54	9.49	9.58	1.55E-01	4.14E-01
7.50	7.53	7.50	1.55E-01	4.14E-01
12.02	12.03	11.98	1.55E-01	4.14E-01
5.33	5.34	5.26	1.55E-01	4.14E-01
4.06	4.09	3.93	1.55E-01	4.14E-01
4.83	4.89	4.48	1.55E-01	4.14E-01
10.20	10.46	10.34	1.55E-01	4.15E-01
6.36	6.37	6.24	1.56E-01	4.15E-01
8.38	8.53	8.41	1.56E-01	4.15E-01
4.27	4.23	4.21	1.56E-01	4.15E-01
8.20	8.39	8.39	1.56E-01	4.15E-01
12.14	12.30	12.30	1.56E-01	4.15E-01
7.19	7.51	7.28	1.56E-01	4.15E-01
14.98	15.00	15.02	1.56E-01	4.15E-01
9.27	9.09	9.19	1.56E-01	4.15E-01
6.38	6.23	6.20	1.56E-01	4.15E-01
9.99	10.17	10.06	1.56E-01	4.15E-01
4.11	4.26	4.02	1.56E-01	4.15E-01
6.47	6.42	6.41	1.56E-01	4.15E-01
3.66	3.60	3.76	1.56E-01	4.15E-01
4.19	3.96	4.24	1.56E-01	4.15E-01
7.12	7.24	7.18	1.56E-01	4.15E-01
4.31	4.12	4.24	1.56E-01	4.15E-01
13.08	13.06	12.98	1.56E-01	4.15E-01
9.10	9.17	9.23	1.56E-01	4.15E-01

6.44	6.18	6.11	1.56E-01	4.15E-01
9.79	9.73	9.73	1.56E-01	4.15E-01
6.27	6.34	6.68	1.56E-01	4.15E-01
4.54	4.06	4.17	1.56E-01	4.15E-01
6.57	6.40	6.16	1.56E-01	4.15E-01
4.58	4.49	4.71	1.56E-01	4.15E-01
5.20	5.05	5.17	1.56E-01	4.15E-01
6.24	6.15	6.34	1.56E-01	4.15E-01
14.74	14.74	14.73	1.56E-01	4.15E-01
7.89	8.20	7.92	1.56E-01	4.15E-01
5.51	5.36	5.22	1.56E-01	4.15E-01
8.07	8.19	8.17	1.56E-01	4.15E-01
5.76	5.80	5.77	1.56E-01	4.15E-01
14.90	14.92	14.95	1.57E-01	4.15E-01
8.72	8.61	8.63	1.57E-01	4.15E-01
8.12	8.06	8.09	1.57E-01	4.15E-01
6.47	6.41	6.41	1.57E-01	4.15E-01
3.90	3.94	3.93	1.57E-01	4.15E-01
3.49	3.32	3.35	1.57E-01	4.16E-01
9.13	8.97	9.19	1.57E-01	4.16E-01
9.90	10.07	9.98	1.57E-01	4.16E-01
9.92	9.94	10.00	1.57E-01	4.16E-01
12.75	12.80	12.81	1.57E-01	4.16E-01
10.62	10.77	10.67	1.57E-01	4.16E-01
5.76	5.63	5.96	1.57E-01	4.16E-01
3.57	3.85	3.63	1.57E-01	4.16E-01
5.94	5.94	5.93	1.57E-01	4.16E-01
8.07	8.05	7.90	1.57E-01	4.16E-01
8.14	8.15	8.21	1.57E-01	4.16E-01
9.42	9.31	9.30	1.57E-01	4.16E-01
9.18	9.18	9.17	1.57E-01	4.16E-01
12.21	12.18	12.16	1.57E-01	4.16E-01
3.50	3.72	3.67	1.57E-01	4.16E-01
3.72	3.67	3.64	1.57E-01	4.16E-01
4.64	4.62	4.74	1.57E-01	4.16E-01
8.96	8.95	8.73	1.57E-01	4.16E-01
9.59	9.83	9.74	1.57E-01	4.16E-01
4.42	4.12	4.18	1.58E-01	4.16E-01
9.19	9.34	9.24	1.58E-01	4.16E-01
9.17	9.33	9.25	1.58E-01	4.16E-01
3.58	3.46	3.68	1.58E-01	4.16E-01
9.71	9.98	9.90	1.58E-01	4.16E-01
8.53	8.57	8.68	1.58E-01	4.16E-01
5.39	5.52	5.40	1.58E-01	4.16E-01
3.26	3.24	3.31	1.58E-01	4.16E-01
4.81	4.90	5.05	1.58E-01	4.16E-01
10.35	10.54	10.25	1.58E-01	4.16E-01

4.26	4.12	3.88	1.58E-01	4.16E-01
5.00	4.70	4.90	1.58E-01	4.17E-01
9.20	9.46	9.19	1.58E-01	4.17E-01
8.57	8.73	8.81	1.58E-01	4.17E-01
10.99	10.71	10.91	1.58E-01	4.17E-01
3.77	3.80	3.50	1.58E-01	4.17E-01
9.39	9.39	9.55	1.58E-01	4.17E-01
3.55	3.36	3.49	1.58E-01	4.17E-01
4.61	4.64	4.68	1.58E-01	4.17E-01
5.79	5.79	6.02	1.58E-01	4.17E-01
8.91	8.90	8.86	1.58E-01	4.17E-01
3.78	3.94	3.71	1.58E-01	4.17E-01
8.48	8.37	8.48	1.58E-01	4.17E-01
14.14	14.09	14.11	1.59E-01	4.17E-01
9.15	9.48	9.28	1.59E-01	4.17E-01
4.75	4.94	4.99	1.59E-01	4.17E-01
9.84	9.77	10.04	1.59E-01	4.17E-01
7.25	7.27	7.48	1.59E-01	4.17E-01
7.86	7.64	7.81	1.59E-01	4.17E-01
7.40	7.23	7.31	1.59E-01	4.17E-01
3.59	3.48	3.56	1.59E-01	4.17E-01
5.45	5.24	5.61	1.59E-01	4.17E-01
6.54	6.54	6.49	1.59E-01	4.17E-01
3.62	3.52	3.73	1.59E-01	4.17E-01
6.59	6.27	6.44	1.59E-01	4.17E-01
7.18	6.86	6.91	1.59E-01	4.17E-01
7.15	7.21	7.18	1.59E-01	4.17E-01
3.65	3.58	3.70	1.59E-01	4.17E-01
5.92	5.89	5.87	1.59E-01	4.17E-01
10.78	10.74	10.79	1.59E-01	4.18E-01
9.27	9.48	9.36	1.59E-01	4.18E-01
7.21	7.22	7.36	1.59E-01	4.18E-01
9.57	9.36	9.63	1.59E-01	4.18E-01
4.79	5.26	5.26	1.59E-01	4.18E-01
7.28	7.40	7.36	1.59E-01	4.18E-01
10.22	10.07	10.13	1.59E-01	4.18E-01
8.00	7.90	7.84	1.59E-01	4.18E-01
5.04	5.33	5.15	1.59E-01	4.18E-01
11.39	11.58	11.40	1.59E-01	4.18E-01
10.09	10.21	10.17	1.59E-01	4.18E-01
8.19	8.16	7.88	1.59E-01	4.18E-01
5.35	5.53	5.12	1.60E-01	4.18E-01
11.27	11.26	11.30	1.60E-01	4.18E-01
4.21	4.27	4.23	1.60E-01	4.18E-01
11.01	11.23	11.14	1.60E-01	4.18E-01
5.16	5.21	5.13	1.60E-01	4.18E-01
3.85	3.75	3.51	1.60E-01	4.18E-01

6.19	5.85	5.76	1.60E-01	4.18E-01
12.39	12.53	12.46	1.60E-01	4.18E-01
10.30	10.37	10.40	1.60E-01	4.18E-01
5.08	4.66	4.76	1.60E-01	4.18E-01
8.59	8.65	8.71	1.60E-01	4.18E-01
8.12	8.26	8.05	1.60E-01	4.18E-01
3.79	3.59	3.40	1.60E-01	4.18E-01
3.25	3.16	3.37	1.60E-01	4.18E-01
5.39	5.36	5.41	1.60E-01	4.19E-01
8.00	8.25	8.07	1.60E-01	4.19E-01
4.58	4.38	4.38	1.60E-01	4.19E-01
6.79	7.08	6.75	1.60E-01	4.19E-01
8.61	8.42	8.55	1.60E-01	4.19E-01
7.06	6.69	6.85	1.60E-01	4.19E-01
4.41	4.76	4.53	1.60E-01	4.19E-01
4.67	4.63	4.64	1.60E-01	4.19E-01
10.37	10.61	10.45	1.60E-01	4.19E-01
4.29	4.21	4.51	1.60E-01	4.19E-01
4.04	4.20	4.01	1.61E-01	4.19E-01
7.21	7.39	7.32	1.61E-01	4.19E-01
7.54	7.65	7.66	1.61E-01	4.19E-01
9.25	9.05	9.29	1.61E-01	4.19E-01
9.25	9.16	9.16	1.61E-01	4.19E-01
7.28	7.32	7.33	1.61E-01	4.19E-01
8.91	8.88	9.08	1.61E-01	4.19E-01
3.48	3.61	3.57	1.61E-01	4.19E-01
6.37	6.76	6.53	1.61E-01	4.19E-01
9.19	9.13	9.29	1.61E-01	4.19E-01
8.77	8.98	8.93	1.61E-01	4.19E-01
10.60	10.32	10.49	1.61E-01	4.19E-01
7.89	7.56	7.81	1.61E-01	4.20E-01
5.17	4.88	5.04	1.61E-01	4.20E-01
3.23	3.52	3.23	1.61E-01	4.20E-01
5.67	5.66	5.68	1.61E-01	4.20E-01
7.56	7.75	7.64	1.61E-01	4.20E-01
8.02	7.82	7.67	1.61E-01	4.20E-01
7.24	6.92	7.05	1.61E-01	4.20E-01
8.83	9.00	8.87	1.61E-01	4.20E-01
7.67	7.60	7.77	1.61E-01	4.20E-01
5.33	5.50	5.39	1.61E-01	4.20E-01
6.74	6.75	6.17	1.61E-01	4.20E-01
5.23	5.25	5.04	1.62E-01	4.20E-01
5.57	5.54	5.68	1.62E-01	4.20E-01
7.77	8.00	7.96	1.62E-01	4.20E-01
4.34	4.41	4.43	1.62E-01	4.20E-01
8.90	8.81	8.85	1.62E-01	4.20E-01
8.28	8.31	8.24	1.62E-01	4.20E-01

4.96	4.90	4.76	1.62E-01	4.20E-01
9.59	9.19	9.62	1.62E-01	4.20E-01
9.90	10.04	9.98	1.62E-01	4.20E-01
8.60	8.75	8.71	1.62E-01	4.20E-01
3.77	3.58	3.85	1.62E-01	4.20E-01
6.99	7.03	7.05	1.62E-01	4.20E-01
5.78	5.66	5.78	1.62E-01	4.20E-01
4.70	4.59	5.06	1.62E-01	4.20E-01
7.09	7.09	7.00	1.62E-01	4.20E-01
7.73	7.81	7.91	1.62E-01	4.20E-01
3.24	3.30	3.26	1.62E-01	4.20E-01
3.57	3.47	3.61	1.62E-01	4.20E-01
13.27	13.36	13.28	1.62E-01	4.20E-01
8.21	8.11	8.08	1.62E-01	4.20E-01
13.25	13.41	13.35	1.62E-01	4.20E-01
4.65	4.66	4.63	1.62E-01	4.20E-01
5.49	5.38	5.34	1.62E-01	4.20E-01
8.05	7.85	8.00	1.62E-01	4.20E-01
10.08	10.13	10.06	1.62E-01	4.20E-01
11.40	11.43	11.51	1.62E-01	4.20E-01
9.99	10.02	10.10	1.62E-01	4.20E-01
9.53	9.18	9.48	1.62E-01	4.20E-01
7.00	7.08	7.21	1.62E-01	4.21E-01
4.37	3.98	4.09	1.62E-01	4.21E-01
7.03	7.17	7.03	1.62E-01	4.21E-01
6.37	6.39	6.25	1.62E-01	4.21E-01
8.71	8.58	8.42	1.63E-01	4.21E-01
9.81	9.72	9.93	1.63E-01	4.21E-01
7.76	7.64	7.63	1.63E-01	4.21E-01
4.15	4.20	4.30	1.63E-01	4.21E-01
11.70	11.93	11.71	1.63E-01	4.21E-01
7.32	7.32	7.25	1.63E-01	4.21E-01
10.55	10.53	10.44	1.63E-01	4.21E-01
3.99	3.96	3.89	1.63E-01	4.21E-01
9.87	9.64	9.80	1.63E-01	4.21E-01
8.21	8.31	8.30	1.63E-01	4.21E-01
8.98	8.92	9.00	1.63E-01	4.21E-01
5.75	5.67	6.03	1.63E-01	4.21E-01
9.09	9.11	9.13	1.63E-01	4.21E-01
8.78	8.85	8.84	1.63E-01	4.21E-01
9.06	8.79	9.04	1.63E-01	4.21E-01
8.62	8.63	8.65	1.63E-01	4.21E-01
7.04	7.07	6.94	1.63E-01	4.21E-01
5.72	5.98	5.93	1.63E-01	4.21E-01
8.96	8.77	9.07	1.63E-01	4.21E-01
4.12	4.11	3.88	1.63E-01	4.21E-01
8.62	8.78	8.88	1.63E-01	4.21E-01

5.69	5.61	5.77	1.63E-01	4.21E-01
8.50	8.48	8.55	1.63E-01	4.21E-01
7.09	7.26	7.13	1.63E-01	4.21E-01
7.91	8.17	8.06	1.63E-01	4.21E-01
6.21	6.44	6.41	1.63E-01	4.21E-01
10.36	10.61	10.49	1.63E-01	4.21E-01
3.94	4.22	3.86	1.64E-01	4.21E-01
5.58	5.54	5.63	1.64E-01	4.21E-01
9.68	9.71	9.65	1.64E-01	4.21E-01
4.98	5.05	5.19	1.64E-01	4.21E-01
5.25	5.28	5.34	1.64E-01	4.21E-01
6.32	6.11	6.28	1.64E-01	4.22E-01
9.23	9.16	9.39	1.64E-01	4.22E-01
9.06	9.08	9.18	1.64E-01	4.22E-01
7.98	7.92	7.89	1.64E-01	4.22E-01
6.20	6.19	6.32	1.64E-01	4.22E-01
10.82	10.62	10.66	1.64E-01	4.22E-01
9.59	9.65	9.66	1.64E-01	4.22E-01
5.33	5.32	5.37	1.64E-01	4.22E-01
4.96	4.83	4.92	1.64E-01	4.22E-01
8.45	8.60	8.53	1.64E-01	4.22E-01
9.13	9.16	9.09	1.64E-01	4.22E-01
3.42	3.60	3.62	1.64E-01	4.22E-01
5.66	5.89	6.15	1.64E-01	4.22E-01
3.59	3.61	3.62	1.64E-01	4.22E-01
3.29	3.18	3.19	1.64E-01	4.22E-01
4.68	4.58	4.73	1.64E-01	4.22E-01
7.09	7.03	7.05	1.64E-01	4.22E-01
6.60	6.69	6.67	1.65E-01	4.23E-01
5.63	5.56	5.54	1.65E-01	4.23E-01
9.32	9.47	9.48	1.65E-01	4.23E-01
5.21	5.27	5.50	1.65E-01	4.23E-01
10.68	10.28	10.56	1.65E-01	4.23E-01
9.12	9.32	9.36	1.65E-01	4.23E-01
7.83	7.87	7.87	1.65E-01	4.23E-01
5.97	5.94	5.98	1.65E-01	4.23E-01
3.91	3.91	3.91	1.65E-01	4.23E-01
7.83	7.95	7.99	1.65E-01	4.23E-01
9.76	9.65	9.58	1.65E-01	4.23E-01
8.69	8.94	8.82	1.65E-01	4.23E-01
4.37	4.32	4.26	1.65E-01	4.23E-01
9.04	9.13	9.04	1.65E-01	4.23E-01
8.66	8.44	8.69	1.65E-01	4.23E-01
8.86	9.08	8.96	1.65E-01	4.23E-01
12.12	12.15	12.02	1.65E-01	4.23E-01
12.53	12.65	12.57	1.65E-01	4.23E-01
9.73	9.94	9.86	1.65E-01	4.23E-01

12.12	12.10	12.13	1.65E-01	4.24E-01
5.53	5.60	5.81	1.65E-01	4.24E-01
13.64	13.48	13.64	1.66E-01	4.24E-01
4.22	4.12	4.08	1.66E-01	4.24E-01
6.60	6.55	6.53	1.66E-01	4.24E-01
6.70	6.79	6.39	1.66E-01	4.24E-01
10.36	10.54	10.48	1.66E-01	4.24E-01
3.51	3.55	3.37	1.66E-01	4.24E-01
5.79	5.73	5.65	1.66E-01	4.24E-01
7.81	7.94	7.83	1.66E-01	4.24E-01
7.22	7.59	7.24	1.66E-01	4.24E-01
6.23	6.20	6.14	1.66E-01	4.24E-01
5.54	5.64	5.51	1.66E-01	4.24E-01
8.17	8.49	8.46	1.66E-01	4.24E-01
10.09	10.00	10.05	1.66E-01	4.24E-01
10.06	10.26	9.99	1.66E-01	4.24E-01
5.60	5.98	5.46	1.66E-01	4.24E-01
3.79	3.50	3.62	1.66E-01	4.24E-01
4.03	3.82	3.98	1.66E-01	4.24E-01
12.57	12.78	12.65	1.66E-01	4.24E-01
3.26	3.29	3.29	1.66E-01	4.24E-01
14.38	14.48	14.38	1.66E-01	4.24E-01
5.26	5.24	5.26	1.66E-01	4.24E-01
6.15	6.19	5.99	1.66E-01	4.25E-01
10.03	10.17	10.04	1.67E-01	4.25E-01
8.78	8.71	8.76	1.67E-01	4.25E-01
6.25	6.32	6.31	1.67E-01	4.25E-01
11.16	11.25	11.21	1.67E-01	4.25E-01
8.58	8.49	8.50	1.67E-01	4.25E-01
3.67	3.70	3.56	1.67E-01	4.25E-01
3.66	3.57	3.90	1.67E-01	4.25E-01
5.95	6.14	5.99	1.67E-01	4.25E-01
4.56	4.32	4.44	1.67E-01	4.25E-01
14.44	14.35	14.45	1.67E-01	4.25E-01
8.08	8.01	8.17	1.67E-01	4.25E-01
4.40	4.37	4.53	1.67E-01	4.25E-01
7.53	7.32	7.47	1.67E-01	4.25E-01
10.70	10.49	10.63	1.67E-01	4.25E-01
3.95	4.12	4.12	1.67E-01	4.25E-01
7.62	7.61	7.64	1.67E-01	4.25E-01
8.95	9.01	8.85	1.67E-01	4.25E-01
5.99	6.22	6.05	1.67E-01	4.25E-01
11.33	11.36	11.29	1.67E-01	4.25E-01
6.56	6.41	6.50	1.67E-01	4.25E-01
3.34	3.23	3.20	1.67E-01	4.25E-01
4.76	4.79	4.90	1.67E-01	4.25E-01
6.78	6.55	6.46	1.67E-01	4.25E-01

7.55	7.63	7.61	1.67E-01	4.26E-01
9.56	9.57	9.61	1.68E-01	4.26E-01
7.73	7.90	7.84	1.68E-01	4.26E-01
7.33	7.13	7.24	1.68E-01	4.26E-01
8.33	8.19	8.29	1.68E-01	4.26E-01
10.83	10.68	10.88	1.68E-01	4.26E-01
5.03	5.02	5.00	1.68E-01	4.26E-01
6.45	6.71	6.46	1.68E-01	4.26E-01
5.76	5.79	5.48	1.68E-01	4.26E-01
4.48	4.64	4.73	1.68E-01	4.26E-01
9.18	9.23	9.21	1.68E-01	4.26E-01
6.14	6.15	6.08	1.68E-01	4.26E-01
9.78	9.58	9.79	1.68E-01	4.26E-01
10.47	10.17	10.47	1.68E-01	4.26E-01
5.53	5.34	5.48	1.68E-01	4.26E-01
7.57	7.51	7.64	1.68E-01	4.27E-01
5.71	5.85	5.90	1.68E-01	4.27E-01
4.59	4.48	4.51	1.68E-01	4.27E-01
3.84	3.85	3.71	1.68E-01	4.27E-01
5.55	5.43	5.54	1.68E-01	4.27E-01
8.07	8.03	8.00	1.68E-01	4.27E-01
4.02	3.80	3.83	1.68E-01	4.27E-01
11.07	11.00	11.01	1.68E-01	4.27E-01
5.35	5.31	5.25	1.68E-01	4.27E-01
7.38	7.20	7.23	1.68E-01	4.27E-01
3.72	3.89	4.04	1.69E-01	4.27E-01
7.33	7.26	7.41	1.69E-01	4.27E-01
6.39	6.40	6.42	1.69E-01	4.27E-01
7.14	7.34	7.16	1.69E-01	4.27E-01
6.77	6.73	6.70	1.69E-01	4.27E-01
9.64	9.66	9.70	1.69E-01	4.27E-01
6.31	6.34	6.39	1.69E-01	4.27E-01
11.27	11.56	11.41	1.69E-01	4.27E-01
5.16	5.32	5.21	1.69E-01	4.27E-01
4.40	4.28	4.29	1.69E-01	4.27E-01
6.63	6.82	6.88	1.69E-01	4.27E-01
5.49	5.20	5.68	1.69E-01	4.27E-01
3.91	3.91	3.94	1.69E-01	4.27E-01
4.19	4.11	4.17	1.69E-01	4.27E-01
3.40	3.46	3.53	1.69E-01	4.27E-01
9.79	10.01	9.93	1.69E-01	4.27E-01
12.02	12.02	12.05	1.69E-01	4.27E-01
5.87	5.93	5.75	1.69E-01	4.28E-01
10.01	10.05	9.90	1.69E-01	4.28E-01
11.34	11.27	11.24	1.69E-01	4.28E-01
7.80	7.85	7.91	1.69E-01	4.28E-01
10.45	10.21	10.26	1.69E-01	4.28E-01

3.55	3.53	3.59	1.70E-01	4.28E-01
5.07	4.79	4.82	1.70E-01	4.28E-01
7.62	7.76	7.68	1.70E-01	4.28E-01
3.92	4.28	4.19	1.70E-01	4.28E-01
3.64	3.59	3.49	1.70E-01	4.28E-01
5.56	5.50	5.63	1.70E-01	4.28E-01
9.25	9.00	9.21	1.70E-01	4.28E-01
3.72	3.64	3.66	1.70E-01	4.28E-01
5.92	6.12	6.00	1.70E-01	4.28E-01
8.22	8.20	8.24	1.70E-01	4.28E-01
3.70	3.50	3.35	1.70E-01	4.28E-01
9.56	9.50	9.64	1.70E-01	4.28E-01
4.02	4.03	4.04	1.70E-01	4.28E-01
7.42	7.44	7.54	1.70E-01	4.28E-01
5.24	4.99	5.41	1.70E-01	4.28E-01
8.50	8.71	8.79	1.70E-01	4.28E-01
4.77	5.04	4.70	1.70E-01	4.28E-01
4.57	4.59	4.47	1.70E-01	4.28E-01
5.75	5.53	6.22	1.70E-01	4.28E-01
6.03	6.38	6.09	1.70E-01	4.28E-01
9.06	9.01	9.01	1.70E-01	4.28E-01
5.80	5.93	6.02	1.70E-01	4.28E-01
12.04	11.81	11.99	1.70E-01	4.28E-01
11.64	11.67	11.60	1.70E-01	4.28E-01
7.91	7.97	7.76	1.70E-01	4.28E-01
4.21	4.35	4.10	1.71E-01	4.29E-01
8.75	8.85	8.76	1.71E-01	4.29E-01
6.14	6.33	6.44	1.71E-01	4.29E-01
8.78	9.00	8.92	1.71E-01	4.29E-01
7.67	7.83	7.82	1.71E-01	4.29E-01
6.42	6.28	6.17	1.71E-01	4.29E-01
5.40	5.66	5.49	1.71E-01	4.29E-01
8.83	8.98	8.78	1.71E-01	4.29E-01
8.03	8.14	8.15	1.71E-01	4.29E-01
7.72	7.95	7.83	1.71E-01	4.29E-01
9.14	9.21	9.30	1.71E-01	4.29E-01
9.33	9.28	9.41	1.71E-01	4.29E-01
9.42	9.20	9.24	1.71E-01	4.29E-01
10.76	10.63	10.81	1.71E-01	4.29E-01
6.43	6.65	6.48	1.71E-01	4.29E-01
10.69	10.26	10.49	1.71E-01	4.29E-01
9.59	9.49	9.70	1.71E-01	4.29E-01
4.79	4.86	4.84	1.71E-01	4.29E-01
5.27	5.14	5.23	1.71E-01	4.29E-01
10.80	10.58	10.77	1.71E-01	4.29E-01
7.26	7.26	7.16	1.71E-01	4.29E-01
6.66	6.69	6.80	1.71E-01	4.29E-01

6.93	7.03	7.10	1.71E-01	4.29E-01
8.33	8.62	8.46	1.71E-01	4.29E-01
6.01	5.98	5.88	1.71E-01	4.29E-01
11.56	11.71	11.72	1.72E-01	4.29E-01
3.81	3.81	3.87	1.72E-01	4.29E-01
10.20	10.11	10.03	1.72E-01	4.29E-01
6.91	7.09	6.97	1.72E-01	4.29E-01
4.96	5.29	5.19	1.72E-01	4.29E-01
9.54	9.64	9.66	1.72E-01	4.29E-01
11.11	11.32	11.16	1.72E-01	4.29E-01
6.34	6.19	6.16	1.72E-01	4.29E-01
3.11	3.22	3.29	1.72E-01	4.30E-01
9.92	9.77	9.99	1.72E-01	4.30E-01
9.82	9.58	9.72	1.72E-01	4.30E-01
8.17	8.10	8.06	1.72E-01	4.30E-01
4.24	4.26	3.87	1.72E-01	4.30E-01
7.93	8.09	7.97	1.72E-01	4.30E-01
8.68	8.90	8.80	1.72E-01	4.30E-01
11.43	11.21	11.33	1.72E-01	4.30E-01
5.14	5.18	5.28	1.72E-01	4.30E-01
5.70	5.52	5.60	1.72E-01	4.30E-01
5.20	5.41	5.47	1.72E-01	4.30E-01
8.04	8.12	8.09	1.72E-01	4.30E-01
11.07	10.75	10.92	1.72E-01	4.30E-01
3.91	3.81	3.86	1.73E-01	4.30E-01
9.12	9.26	9.19	1.73E-01	4.30E-01
4.48	4.19	4.30	1.73E-01	4.30E-01
9.11	8.78	8.95	1.73E-01	4.30E-01
10.32	10.14	10.31	1.73E-01	4.30E-01
11.71	11.71	11.65	1.73E-01	4.30E-01
8.65	8.98	8.87	1.73E-01	4.30E-01
9.44	9.35	9.47	1.73E-01	4.30E-01
7.67	7.85	7.83	1.73E-01	4.30E-01
7.41	7.50	7.52	1.73E-01	4.30E-01
6.56	6.63	6.50	1.73E-01	4.30E-01
7.73	7.70	7.86	1.73E-01	4.30E-01
8.49	8.64	8.47	1.73E-01	4.30E-01
4.34	4.45	4.23	1.73E-01	4.30E-01
8.63	8.63	8.77	1.73E-01	4.30E-01
8.30	8.40	8.42	1.73E-01	4.30E-01
7.69	7.83	7.86	1.73E-01	4.30E-01
4.91	4.80	4.73	1.73E-01	4.30E-01
5.53	5.59	5.69	1.73E-01	4.31E-01
6.64	6.74	6.65	1.73E-01	4.31E-01
4.23	4.31	4.09	1.73E-01	4.31E-01
7.81	7.94	7.79	1.73E-01	4.31E-01
5.11	4.89	5.01	1.73E-01	4.31E-01

9.44	9.42	9.61	1.73E-01	4.31E-01
3.44	3.21	3.42	1.73E-01	4.31E-01
10.39	10.36	10.41	1.73E-01	4.31E-01
10.06	10.38	10.24	1.73E-01	4.31E-01
6.35	6.48	6.42	1.73E-01	4.31E-01
7.41	7.23	7.37	1.73E-01	4.31E-01
3.79	3.83	3.88	1.74E-01	4.31E-01
4.78	4.80	4.70	1.74E-01	4.31E-01
8.45	8.15	8.37	1.74E-01	4.31E-01
9.01	9.15	9.07	1.74E-01	4.31E-01
9.47	9.64	9.56	1.74E-01	4.31E-01
10.17	10.24	10.17	1.74E-01	4.31E-01
6.51	6.42	6.61	1.74E-01	4.32E-01
8.36	8.23	8.43	1.74E-01	4.32E-01
3.49	3.37	3.52	1.74E-01	4.32E-01
11.59	11.42	11.60	1.74E-01	4.32E-01
7.61	7.88	7.75	1.74E-01	4.32E-01
7.99	8.09	7.96	1.74E-01	4.32E-01
5.96	5.99	6.05	1.74E-01	4.32E-01
9.37	9.50	9.48	1.74E-01	4.32E-01
8.35	8.33	8.36	1.74E-01	4.32E-01
12.06	12.09	12.02	1.74E-01	4.32E-01
14.12	14.06	14.03	1.74E-01	4.32E-01
7.98	8.19	8.22	1.74E-01	4.32E-01
8.87	9.08	8.98	1.74E-01	4.32E-01
9.88	10.07	10.06	1.74E-01	4.32E-01
5.50	5.63	5.74	1.74E-01	4.32E-01
3.51	3.56	3.73	1.74E-01	4.32E-01
8.20	8.02	8.23	1.74E-01	4.32E-01
8.30	8.62	8.41	1.74E-01	4.32E-01
9.13	9.36	9.29	1.74E-01	4.32E-01
12.48	12.44	12.46	1.74E-01	4.32E-01
9.82	9.79	9.82	1.74E-01	4.32E-01
6.81	6.81	6.78	1.74E-01	4.32E-01
6.03	6.17	5.94	1.75E-01	4.32E-01
3.71	3.74	4.14	1.75E-01	4.32E-01
5.03	4.80	4.85	1.75E-01	4.32E-01
10.10	10.01	10.07	1.75E-01	4.32E-01
11.82	12.01	11.85	1.75E-01	4.32E-01
10.65	10.61	10.65	1.75E-01	4.32E-01
5.61	5.56	5.66	1.75E-01	4.32E-01
5.56	5.45	5.43	1.75E-01	4.32E-01
5.58	5.55	5.55	1.75E-01	4.32E-01
7.28	7.55	7.52	1.75E-01	4.32E-01
5.20	5.11	5.23	1.75E-01	4.32E-01
7.25	7.39	7.34	1.75E-01	4.32E-01
7.66	7.70	7.53	1.75E-01	4.32E-01

7.06	7.28	7.01	1.75E-01	4.32E-01
3.91	3.73	3.83	1.75E-01	4.33E-01
7.98	8.05	8.02	1.75E-01	4.33E-01
11.25	11.34	11.30	1.75E-01	4.33E-01
5.73	5.71	5.66	1.75E-01	4.33E-01
9.61	9.30	9.41	1.75E-01	4.33E-01
4.30	4.18	4.09	1.76E-01	4.33E-01
5.10	5.31	5.35	1.76E-01	4.33E-01
8.23	8.00	8.34	1.76E-01	4.33E-01
4.25	4.17	4.27	1.76E-01	4.33E-01
7.87	7.86	7.87	1.76E-01	4.33E-01
3.75	3.45	3.40	1.76E-01	4.33E-01
7.42	7.48	7.41	1.76E-01	4.33E-01
9.92	10.03	9.97	1.76E-01	4.33E-01
4.65	4.59	4.54	1.76E-01	4.33E-01
6.73	6.71	6.99	1.76E-01	4.33E-01
3.80	3.85	3.75	1.76E-01	4.33E-01
5.79	5.97	5.96	1.76E-01	4.33E-01
5.17	5.32	5.13	1.76E-01	4.33E-01
5.06	5.06	5.07	1.76E-01	4.34E-01
8.65	8.76	8.73	1.76E-01	4.34E-01
3.14	3.30	3.29	1.76E-01	4.34E-01
4.12	4.19	4.42	1.76E-01	4.34E-01
5.91	5.71	5.76	1.76E-01	4.34E-01
3.31	3.17	3.26	1.76E-01	4.34E-01
3.35	3.46	3.51	1.76E-01	4.34E-01
3.89	4.05	3.85	1.76E-01	4.34E-01
3.84	4.06	3.91	1.76E-01	4.34E-01
8.17	8.32	8.28	1.76E-01	4.34E-01
3.30	3.35	3.46	1.76E-01	4.34E-01
9.18	9.11	9.25	1.76E-01	4.34E-01
3.26	3.34	3.33	1.76E-01	4.34E-01
6.74	6.84	6.74	1.77E-01	4.34E-01
7.76	7.73	7.78	1.77E-01	4.34E-01
3.23	3.28	3.23	1.77E-01	4.34E-01
7.72	8.11	7.92	1.77E-01	4.34E-01
9.04	8.98	8.94	1.77E-01	4.34E-01
4.26	4.66	4.32	1.77E-01	4.34E-01
6.82	6.67	6.72	1.77E-01	4.34E-01
12.64	12.68	12.68	1.77E-01	4.34E-01
10.19	10.18	10.17	1.77E-01	4.34E-01
7.57	7.75	7.56	1.77E-01	4.35E-01
3.87	3.87	4.13	1.77E-01	4.35E-01
3.48	3.34	3.70	1.77E-01	4.35E-01
6.04	5.94	5.99	1.77E-01	4.35E-01
4.02	3.92	4.39	1.77E-01	4.35E-01
10.54	10.50	10.53	1.77E-01	4.35E-01

10.67	10.93	10.80	1.77E-01	4.35E-01
4.36	4.55	4.42	1.77E-01	4.35E-01
9.78	9.89	9.81	1.77E-01	4.35E-01
3.64	3.58	3.65	1.77E-01	4.35E-01
6.19	6.29	6.36	1.77E-01	4.35E-01
5.05	5.08	4.82	1.77E-01	4.35E-01
9.32	9.41	9.39	1.77E-01	4.35E-01
5.42	5.29	5.36	1.77E-01	4.35E-01
4.60	4.52	4.47	1.78E-01	4.35E-01
7.32	7.19	7.08	1.78E-01	4.35E-01
8.86	8.92	8.90	1.78E-01	4.35E-01
3.69	3.68	3.77	1.78E-01	4.35E-01
8.99	9.04	8.90	1.78E-01	4.35E-01
5.00	5.11	5.17	1.78E-01	4.35E-01
6.98	6.76	6.99	1.78E-01	4.35E-01
9.34	9.46	9.39	1.78E-01	4.35E-01
10.52	10.53	10.59	1.78E-01	4.35E-01
8.89	8.98	8.87	1.78E-01	4.35E-01
10.55	10.44	10.58	1.78E-01	4.35E-01
6.43	6.00	6.41	1.78E-01	4.35E-01
6.13	6.53	6.31	1.78E-01	4.35E-01
9.39	9.69	9.58	1.78E-01	4.35E-01
4.97	5.51	5.06	1.78E-01	4.35E-01
11.05	11.14	11.07	1.78E-01	4.35E-01
5.92	5.90	6.04	1.78E-01	4.35E-01
4.47	4.39	4.40	1.78E-01	4.35E-01
6.63	6.73	6.64	1.78E-01	4.35E-01
9.05	8.94	9.17	1.78E-01	4.35E-01
3.79	3.68	3.97	1.78E-01	4.35E-01
8.97	8.95	8.97	1.78E-01	4.35E-01
6.17	6.17	5.99	1.78E-01	4.35E-01
3.75	3.90	3.90	1.78E-01	4.35E-01
8.95	8.88	9.04	1.78E-01	4.35E-01
8.17	7.75	8.02	1.78E-01	4.35E-01
7.16	6.98	7.11	1.79E-01	4.35E-01
5.04	4.83	4.88	1.79E-01	4.35E-01
7.10	7.04	7.10	1.79E-01	4.35E-01
9.07	9.06	9.36	1.79E-01	4.35E-01
8.49	8.27	8.39	1.79E-01	4.35E-01
11.39	11.17	11.28	1.79E-01	4.35E-01
5.73	5.71	5.57	1.79E-01	4.35E-01
4.88	4.78	4.58	1.79E-01	4.35E-01
12.36	12.32	12.31	1.79E-01	4.35E-01
8.95	9.11	9.10	1.79E-01	4.35E-01
4.62	4.64	4.66	1.79E-01	4.35E-01
6.41	6.63	6.44	1.79E-01	4.35E-01
5.73	5.49	5.48	1.79E-01	4.35E-01

4.30	4.19	4.15	1.79E-01	4.35E-01
8.12	8.01	8.06	1.79E-01	4.36E-01
8.64	9.03	8.73	1.79E-01	4.36E-01
7.81	7.86	7.97	1.79E-01	4.36E-01
12.11	12.04	12.02	1.79E-01	4.36E-01
5.95	5.24	6.12	1.79E-01	4.36E-01
8.13	8.22	8.14	1.79E-01	4.36E-01
10.22	10.18	10.26	1.79E-01	4.36E-01
4.28	4.37	4.34	1.79E-01	4.36E-01
8.68	8.64	8.64	1.79E-01	4.36E-01
5.66	5.67	5.73	1.79E-01	4.36E-01
6.56	6.61	6.57	1.79E-01	4.36E-01
6.01	6.03	6.12	1.79E-01	4.36E-01
4.27	4.03	4.31	1.79E-01	4.36E-01
7.35	7.23	7.26	1.80E-01	4.36E-01
4.19	4.27	4.05	1.80E-01	4.36E-01
10.95	11.21	11.03	1.80E-01	4.37E-01
6.29	6.23	6.64	1.80E-01	4.37E-01
4.90	5.00	5.10	1.80E-01	4.37E-01
7.22	7.46	7.36	1.80E-01	4.37E-01
8.08	8.09	8.04	1.80E-01	4.37E-01
5.41	5.46	5.40	1.80E-01	4.37E-01
10.14	9.93	10.10	1.80E-01	4.37E-01
5.71	5.95	5.88	1.80E-01	4.37E-01
6.54	6.57	6.47	1.80E-01	4.37E-01
7.63	7.75	7.77	1.80E-01	4.37E-01
9.10	9.21	9.24	1.80E-01	4.37E-01
9.26	9.07	9.23	1.80E-01	4.37E-01
4.25	4.61	4.37	1.80E-01	4.37E-01
13.50	13.64	13.48	1.80E-01	4.37E-01
8.01	7.99	8.09	1.80E-01	4.37E-01
10.77	10.63	10.65	1.80E-01	4.37E-01
6.13	6.16	5.75	1.80E-01	4.37E-01
10.37	10.53	10.45	1.80E-01	4.37E-01
11.38	10.91	11.22	1.81E-01	4.37E-01
9.93	9.69	9.84	1.81E-01	4.37E-01
6.99	6.35	6.88	1.81E-01	4.37E-01
7.67	7.78	7.78	1.81E-01	4.37E-01
3.98	3.95	3.86	1.81E-01	4.37E-01
14.42	14.34	14.39	1.81E-01	4.37E-01
7.09	6.85	6.95	1.81E-01	4.38E-01
7.40	7.46	7.58	1.81E-01	4.38E-01
9.30	9.50	9.36	1.81E-01	4.38E-01
14.38	14.51	14.40	1.81E-01	4.38E-01
9.03	9.06	9.01	1.81E-01	4.38E-01
7.07	6.93	6.91	1.81E-01	4.38E-01
6.89	6.57	6.73	1.81E-01	4.38E-01

10.33	10.62	10.50	1.81E-01	4.38E-01
9.16	8.78	9.34	1.81E-01	4.38E-01
10.83	10.95	10.91	1.81E-01	4.38E-01
8.01	8.21	8.08	1.81E-01	4.38E-01
6.01	6.05	6.17	1.81E-01	4.38E-01
6.15	6.07	6.31	1.81E-01	4.38E-01
7.92	7.72	7.74	1.82E-01	4.38E-01
6.61	6.55	6.52	1.82E-01	4.38E-01
9.67	10.15	9.70	1.82E-01	4.38E-01
9.82	10.10	9.90	1.82E-01	4.38E-01
8.77	8.96	8.81	1.82E-01	4.38E-01
6.18	6.40	6.47	1.82E-01	4.38E-01
6.94	7.08	6.96	1.82E-01	4.38E-01
9.54	9.55	9.39	1.82E-01	4.38E-01
7.01	6.88	6.95	1.82E-01	4.38E-01
5.74	5.60	5.71	1.82E-01	4.38E-01
7.96	7.87	8.02	1.82E-01	4.38E-01
4.97	4.90	5.11	1.82E-01	4.38E-01
13.31	13.38	13.37	1.82E-01	4.38E-01
4.86	4.83	4.74	1.82E-01	4.38E-01
5.46	5.16	5.53	1.82E-01	4.38E-01
7.79	7.91	7.86	1.82E-01	4.39E-01
13.58	13.66	13.64	1.82E-01	4.39E-01
7.02	7.06	7.11	1.82E-01	4.39E-01
9.08	8.90	8.97	1.82E-01	4.39E-01
8.67	8.62	8.75	1.82E-01	4.39E-01
7.43	7.72	7.68	1.82E-01	4.39E-01
7.99	8.08	7.86	1.82E-01	4.39E-01
5.51	5.53	5.34	1.82E-01	4.39E-01
12.01	11.80	11.79	1.82E-01	4.39E-01
5.31	5.53	5.40	1.82E-01	4.39E-01
13.22	13.32	13.28	1.83E-01	4.39E-01
5.28	5.30	5.21	1.83E-01	4.39E-01
8.98	8.98	9.16	1.83E-01	4.39E-01
4.21	4.47	4.44	1.83E-01	4.39E-01
8.75	8.78	8.74	1.83E-01	4.39E-01
4.16	4.32	4.19	1.83E-01	4.40E-01
7.90	7.97	7.98	1.83E-01	4.40E-01
11.17	11.17	11.13	1.83E-01	4.40E-01
9.98	9.93	10.01	1.83E-01	4.40E-01
5.35	5.13	5.09	1.83E-01	4.40E-01
3.24	3.31	3.25	1.83E-01	4.40E-01
8.87	8.93	9.01	1.83E-01	4.40E-01
7.86	7.94	7.90	1.83E-01	4.40E-01
7.98	8.51	8.10	1.83E-01	4.40E-01
3.66	3.55	3.67	1.83E-01	4.40E-01
4.38	4.53	4.66	1.83E-01	4.40E-01

8.76	8.81	8.64	1.83E-01	4.40E-01
4.93	4.89	4.84	1.83E-01	4.40E-01
8.78	9.06	8.81	1.83E-01	4.40E-01
5.66	6.01	5.79	1.83E-01	4.40E-01
11.69	11.69	11.67	1.83E-01	4.40E-01
6.23	6.46	6.54	1.83E-01	4.40E-01
3.86	3.81	3.80	1.83E-01	4.40E-01
5.22	5.04	5.24	1.83E-01	4.40E-01
7.27	7.05	7.06	1.83E-01	4.40E-01
5.52	5.50	5.51	1.83E-01	4.40E-01
3.30	3.26	3.25	1.83E-01	4.40E-01
3.37	3.40	3.53	1.84E-01	4.40E-01
9.20	9.47	9.33	1.84E-01	4.40E-01
4.75	4.59	4.96	1.84E-01	4.40E-01
9.11	9.43	9.22	1.84E-01	4.40E-01
7.59	7.80	7.63	1.84E-01	4.40E-01
10.28	10.08	10.21	1.84E-01	4.40E-01
6.28	6.32	6.37	1.84E-01	4.40E-01
4.20	4.32	4.42	1.84E-01	4.40E-01
6.99	7.14	6.96	1.84E-01	4.40E-01
6.56	6.56	6.63	1.84E-01	4.40E-01
6.91	6.74	6.77	1.84E-01	4.40E-01
15.06	15.06	15.09	1.84E-01	4.40E-01
3.36	3.56	3.51	1.84E-01	4.40E-01
3.62	3.37	3.56	1.84E-01	4.40E-01
3.52	3.62	3.51	1.84E-01	4.40E-01
7.72	7.56	7.58	1.84E-01	4.40E-01
5.55	5.33	5.27	1.84E-01	4.40E-01
7.71	7.87	7.76	1.84E-01	4.40E-01
9.04	9.09	9.11	1.84E-01	4.41E-01
6.54	6.55	6.58	1.84E-01	4.41E-01
8.06	8.12	7.92	1.85E-01	4.41E-01
3.95	4.23	4.10	1.85E-01	4.41E-01
6.46	6.51	6.46	1.85E-01	4.41E-01
4.98	5.04	5.31	1.85E-01	4.41E-01
4.47	4.30	4.30	1.85E-01	4.41E-01
5.61	5.44	5.40	1.85E-01	4.41E-01
4.02	4.02	4.04	1.85E-01	4.41E-01
9.16	9.09	9.06	1.85E-01	4.41E-01
4.00	3.71	3.98	1.85E-01	4.41E-01
4.81	4.85	4.74	1.85E-01	4.41E-01
4.34	4.23	4.28	1.85E-01	4.41E-01
9.47	9.58	9.59	1.85E-01	4.41E-01
5.35	5.62	5.45	1.85E-01	4.41E-01
6.47	6.81	6.92	1.85E-01	4.41E-01
5.51	5.42	5.52	1.85E-01	4.41E-01
7.27	7.22	7.23	1.85E-01	4.41E-01

4.21	4.20	4.23	1.85E-01	4.41E-01
3.58	3.71	3.59	1.85E-01	4.41E-01
14.35	14.35	14.37	1.85E-01	4.41E-01
8.94	8.74	8.97	1.85E-01	4.41E-01
14.30	14.32	14.38	1.85E-01	4.41E-01
7.41	7.36	7.42	1.85E-01	4.41E-01
10.12	10.04	10.19	1.85E-01	4.41E-01
4.64	4.73	4.50	1.85E-01	4.41E-01
8.46	8.17	8.15	1.85E-01	4.41E-01
9.34	9.52	9.50	1.85E-01	4.41E-01
12.10	12.21	12.09	1.85E-01	4.41E-01
7.96	7.99	8.03	1.85E-01	4.41E-01
6.45	6.40	6.45	1.86E-01	4.42E-01
5.80	5.68	5.76	1.86E-01	4.42E-01
7.62	7.41	7.56	1.86E-01	4.42E-01
5.34	5.17	5.16	1.86E-01	4.42E-01
9.08	9.25	9.28	1.86E-01	4.42E-01
8.07	8.08	8.01	1.86E-01	4.42E-01
7.52	7.10	7.49	1.86E-01	4.42E-01
5.58	5.48	5.44	1.86E-01	4.42E-01
5.59	5.36	5.50	1.86E-01	4.42E-01
7.49	7.78	7.84	1.86E-01	4.42E-01
6.03	5.89	5.89	1.86E-01	4.42E-01
7.39	7.48	7.24	1.86E-01	4.42E-01
13.44	13.47	13.44	1.86E-01	4.42E-01
5.77	5.67	5.87	1.86E-01	4.42E-01
6.51	6.53	6.64	1.86E-01	4.43E-01
7.84	7.81	7.71	1.86E-01	4.43E-01
10.17	10.32	10.31	1.86E-01	4.43E-01
5.65	5.43	5.68	1.86E-01	4.43E-01
8.86	9.05	8.79	1.86E-01	4.43E-01
7.17	6.96	7.21	1.87E-01	4.43E-01
6.28	6.28	6.20	1.87E-01	4.43E-01
8.47	8.37	8.50	1.87E-01	4.43E-01
5.94	6.03	5.74	1.87E-01	4.43E-01
9.23	9.24	9.07	1.87E-01	4.43E-01
4.09	4.00	4.31	1.87E-01	4.43E-01
3.66	3.73	3.45	1.87E-01	4.43E-01
5.14	5.24	5.30	1.87E-01	4.43E-01
7.69	7.81	7.65	1.87E-01	4.43E-01
6.41	6.30	6.37	1.87E-01	4.43E-01
7.13	7.17	7.45	1.87E-01	4.43E-01
5.13	5.24	5.44	1.87E-01	4.43E-01
6.15	6.30	6.48	1.87E-01	4.43E-01
4.75	4.58	4.63	1.87E-01	4.43E-01
8.67	8.77	8.71	1.87E-01	4.43E-01
5.90	5.98	6.05	1.87E-01	4.43E-01

4.71	4.60	4.55	1.87E-01	4.43E-01
6.58	6.68	6.58	1.87E-01	4.43E-01
6.98	6.86	6.84	1.87E-01	4.43E-01
8.01	8.25	8.10	1.87E-01	4.43E-01
6.29	6.26	6.38	1.87E-01	4.43E-01
4.00	4.13	3.88	1.87E-01	4.43E-01
11.12	11.33	11.23	1.87E-01	4.43E-01
7.90	7.95	8.17	1.87E-01	4.43E-01
3.85	3.82	3.93	1.87E-01	4.44E-01
8.55	8.76	8.65	1.88E-01	4.44E-01
8.68	9.20	8.94	1.88E-01	4.44E-01
10.86	11.06	10.98	1.88E-01	4.44E-01
8.53	8.82	8.73	1.88E-01	4.44E-01
3.13	3.19	2.97	1.88E-01	4.44E-01
4.52	4.73	4.81	1.88E-01	4.44E-01
9.69	9.74	9.78	1.88E-01	4.44E-01
6.96	7.06	6.82	1.88E-01	4.44E-01
8.74	8.77	8.86	1.88E-01	4.44E-01
6.39	6.42	6.44	1.88E-01	4.44E-01
7.61	7.61	7.66	1.88E-01	4.44E-01
7.05	7.02	6.99	1.88E-01	4.44E-01
4.09	4.05	4.06	1.88E-01	4.44E-01
8.23	8.27	8.45	1.88E-01	4.44E-01
6.26	6.36	6.16	1.88E-01	4.44E-01
12.02	12.23	12.15	1.88E-01	4.44E-01
7.63	7.51	7.72	1.88E-01	4.44E-01
5.49	5.05	5.25	1.88E-01	4.44E-01
11.03	10.69	10.89	1.88E-01	4.44E-01
7.23	7.51	7.33	1.88E-01	4.44E-01
9.72	9.50	9.75	1.88E-01	4.44E-01
4.94	4.89	4.89	1.89E-01	4.45E-01
6.60	6.61	6.69	1.89E-01	4.45E-01
8.06	7.92	7.96	1.89E-01	4.45E-01
5.26	5.28	5.37	1.89E-01	4.45E-01
7.79	7.58	8.10	1.89E-01	4.45E-01
3.42	3.69	3.51	1.89E-01	4.45E-01
3.11	3.33	2.99	1.89E-01	4.45E-01
8.77	8.73	8.89	1.89E-01	4.45E-01
8.51	8.60	8.47	1.89E-01	4.45E-01
10.69	10.73	10.61	1.89E-01	4.45E-01
10.07	10.30	10.11	1.89E-01	4.45E-01
8.47	8.40	8.58	1.89E-01	4.45E-01
7.20	7.36	7.29	1.89E-01	4.45E-01
9.03	8.84	8.98	1.89E-01	4.45E-01
9.26	9.28	9.24	1.89E-01	4.45E-01
5.96	6.02	5.95	1.89E-01	4.45E-01
4.91	4.76	4.94	1.89E-01	4.45E-01

6.21	6.33	6.00	1.89E-01	4.45E-01
9.50	9.62	9.62	1.89E-01	4.45E-01
3.60	3.62	3.65	1.89E-01	4.45E-01
12.52	12.41	12.46	1.89E-01	4.45E-01
4.57	4.64	4.63	1.89E-01	4.45E-01
7.12	7.12	7.15	1.89E-01	4.45E-01
9.23	9.38	9.32	1.90E-01	4.45E-01
4.63	4.86	4.61	1.90E-01	4.45E-01
7.93	8.05	8.04	1.90E-01	4.45E-01
7.59	7.56	7.60	1.90E-01	4.45E-01
9.32	9.23	9.30	1.90E-01	4.45E-01
4.54	4.44	4.61	1.90E-01	4.45E-01
4.57	4.79	5.01	1.90E-01	4.45E-01
13.18	12.84	12.99	1.90E-01	4.45E-01
4.87	4.99	4.97	1.90E-01	4.45E-01
4.27	4.36	4.52	1.90E-01	4.45E-01
7.85	7.73	8.01	1.90E-01	4.45E-01
3.99	3.77	3.92	1.90E-01	4.45E-01
8.10	8.09	8.16	1.90E-01	4.46E-01
7.98	8.34	8.13	1.90E-01	4.46E-01
3.95	4.13	4.17	1.90E-01	4.46E-01
7.02	7.02	7.09	1.90E-01	4.46E-01
6.38	6.35	6.25	1.90E-01	4.46E-01
3.22	3.03	3.26	1.90E-01	4.46E-01
8.60	8.74	8.61	1.90E-01	4.46E-01
7.01	6.97	7.10	1.90E-01	4.46E-01
14.92	14.84	14.86	1.90E-01	4.46E-01
7.87	8.19	7.79	1.90E-01	4.46E-01
7.84	8.02	8.13	1.90E-01	4.46E-01
5.61	5.89	5.83	1.90E-01	4.46E-01
5.75	5.85	5.74	1.90E-01	4.46E-01
5.87	5.75	5.75	1.90E-01	4.46E-01
10.32	10.51	10.43	1.90E-01	4.46E-01
8.74	8.68	8.77	1.91E-01	4.46E-01
5.06	4.84	4.88	1.91E-01	4.46E-01
7.27	7.21	7.21	1.91E-01	4.46E-01
11.54	11.68	11.68	1.91E-01	4.46E-01
8.14	7.81	7.99	1.91E-01	4.46E-01
3.26	3.30	3.08	1.91E-01	4.46E-01
3.87	3.70	3.76	1.91E-01	4.46E-01
6.88	7.04	6.80	1.91E-01	4.46E-01
7.86	7.78	7.85	1.91E-01	4.46E-01
4.85	4.97	4.86	1.91E-01	4.46E-01
9.00	9.05	8.95	1.91E-01	4.46E-01
7.50	7.57	7.64	1.91E-01	4.46E-01
9.28	9.29	9.31	1.91E-01	4.46E-01
9.58	9.75	9.74	1.91E-01	4.46E-01

3.45	3.47	3.31	1.91E-01	4.46E-01
3.36	3.65	3.42	1.91E-01	4.47E-01
11.19	11.42	11.30	1.91E-01	4.47E-01
4.02	3.97	4.02	1.91E-01	4.47E-01
4.01	3.90	3.89	1.91E-01	4.47E-01
8.07	8.32	8.15	1.91E-01	4.47E-01
5.95	5.82	6.05	1.91E-01	4.47E-01
5.40	5.55	5.28	1.91E-01	4.47E-01
3.65	3.62	3.64	1.91E-01	4.47E-01
6.84	6.68	6.71	1.91E-01	4.47E-01
5.04	5.15	5.06	1.91E-01	4.47E-01
7.19	7.34	7.27	1.91E-01	4.47E-01
8.30	8.33	8.19	1.91E-01	4.47E-01
5.35	5.28	5.45	1.92E-01	4.47E-01
4.01	4.08	3.92	1.92E-01	4.47E-01
9.33	9.52	9.50	1.92E-01	4.47E-01
9.45	9.09	9.46	1.92E-01	4.47E-01
8.61	8.63	8.69	1.92E-01	4.47E-01
7.62	7.56	7.56	1.92E-01	4.47E-01
7.69	8.08	7.93	1.92E-01	4.47E-01
6.57	6.68	6.78	1.92E-01	4.47E-01
9.25	8.99	9.58	1.92E-01	4.47E-01
11.00	10.95	10.97	1.92E-01	4.47E-01
5.12	5.53	5.53	1.92E-01	4.47E-01
7.66	7.77	7.83	1.92E-01	4.47E-01
4.55	4.51	4.51	1.92E-01	4.47E-01
3.85	3.66	3.67	1.92E-01	4.47E-01
6.76	6.43	6.54	1.92E-01	4.47E-01
4.04	3.85	3.88	1.92E-01	4.47E-01
10.31	10.01	10.33	1.92E-01	4.47E-01
11.03	11.38	11.09	1.92E-01	4.47E-01
5.34	5.44	5.70	1.92E-01	4.47E-01
6.66	6.68	6.73	1.92E-01	4.47E-01
3.82	3.96	3.76	1.92E-01	4.47E-01
3.52	3.53	3.45	1.92E-01	4.47E-01
10.36	10.21	10.49	1.92E-01	4.47E-01
6.32	6.30	6.40	1.92E-01	4.48E-01
10.80	10.79	10.84	1.93E-01	4.48E-01
10.13	9.89	10.11	1.93E-01	4.48E-01
10.03	9.95	10.07	1.93E-01	4.48E-01
7.86	7.57	7.70	1.93E-01	4.48E-01
10.50	10.28	10.49	1.93E-01	4.48E-01
5.42	5.39	5.51	1.93E-01	4.48E-01
9.38	9.43	9.49	1.93E-01	4.48E-01
8.56	8.22	8.31	1.93E-01	4.48E-01
8.86	9.10	8.77	1.93E-01	4.48E-01
4.07	3.98	4.07	1.93E-01	4.48E-01

7.67	7.52	7.55	1.93E-01	4.48E-01
8.06	8.24	8.18	1.93E-01	4.48E-01
7.28	6.92	7.12	1.93E-01	4.48E-01
11.95	11.96	11.88	1.93E-01	4.48E-01
8.47	8.66	8.49	1.93E-01	4.48E-01
6.08	6.51	6.46	1.93E-01	4.48E-01
7.39	7.47	7.56	1.93E-01	4.48E-01
4.73	4.73	4.51	1.93E-01	4.48E-01
8.35	7.89	8.16	1.93E-01	4.48E-01
11.28	11.30	11.32	1.93E-01	4.48E-01
3.32	3.39	3.12	1.93E-01	4.48E-01
3.45	3.61	3.62	1.93E-01	4.48E-01
11.72	11.61	11.67	1.94E-01	4.48E-01
9.19	9.33	9.35	1.94E-01	4.48E-01
7.59	7.97	7.64	1.94E-01	4.48E-01
11.88	11.57	11.86	1.94E-01	4.48E-01
8.10	8.28	8.37	1.94E-01	4.48E-01
4.80	5.15	5.14	1.94E-01	4.48E-01
7.66	7.81	7.62	1.94E-01	4.48E-01
5.54	5.48	5.50	1.94E-01	4.48E-01
10.11	10.49	10.28	1.94E-01	4.48E-01
5.69	5.81	5.70	1.94E-01	4.48E-01
5.14	5.21	5.26	1.94E-01	4.48E-01
3.79	3.85	3.78	1.94E-01	4.48E-01
4.57	4.65	4.84	1.94E-01	4.48E-01
8.62	8.78	8.71	1.94E-01	4.48E-01
4.19	3.96	3.90	1.94E-01	4.48E-01
5.86	6.15	6.20	1.94E-01	4.48E-01
3.57	3.47	3.48	1.94E-01	4.48E-01
5.47	5.40	5.39	1.94E-01	4.48E-01
3.60	3.56	3.40	1.94E-01	4.48E-01
7.79	7.84	7.81	1.94E-01	4.48E-01
10.19	10.06	10.19	1.94E-01	4.48E-01
14.25	14.21	14.24	1.94E-01	4.48E-01
6.23	6.24	5.99	1.94E-01	4.48E-01
4.76	4.93	4.67	1.94E-01	4.48E-01
6.55	6.36	6.54	1.94E-01	4.48E-01
4.35	4.00	4.20	1.94E-01	4.48E-01
11.00	11.14	11.07	1.94E-01	4.48E-01
9.44	9.31	9.42	1.94E-01	4.48E-01
9.03	8.95	9.01	1.94E-01	4.48E-01
6.59	6.63	6.56	1.94E-01	4.48E-01
3.49	3.57	3.64	1.94E-01	4.48E-01
6.31	6.10	6.03	1.94E-01	4.48E-01
11.29	11.19	11.34	1.94E-01	4.48E-01
7.78	7.71	7.44	1.94E-01	4.48E-01
5.23	5.24	5.37	1.95E-01	4.48E-01

10.13	9.91	10.19	1.95E-01	4.48E-01
11.92	12.22	12.03	1.95E-01	4.48E-01
8.85	9.03	8.90	1.95E-01	4.48E-01
5.02	5.34	4.97	1.95E-01	4.48E-01
12.55	12.74	12.59	1.95E-01	4.48E-01
6.15	6.12	6.02	1.95E-01	4.48E-01
6.14	6.04	6.08	1.95E-01	4.48E-01
8.20	8.20	8.17	1.95E-01	4.48E-01
12.07	12.27	12.14	1.95E-01	4.48E-01
8.84	9.05	9.18	1.95E-01	4.48E-01
7.98	8.21	8.09	1.95E-01	4.48E-01
7.12	7.17	7.24	1.95E-01	4.48E-01
9.31	9.40	9.40	1.95E-01	4.48E-01
3.27	3.46	3.45	1.95E-01	4.48E-01
9.75	9.95	9.89	1.95E-01	4.48E-01
3.17	3.25	3.16	1.95E-01	4.48E-01
9.62	9.28	9.48	1.95E-01	4.48E-01
6.98	6.91	6.93	1.95E-01	4.48E-01
7.47	7.44	7.57	1.95E-01	4.48E-01
4.76	5.03	5.09	1.95E-01	4.48E-01
10.73	10.34	10.52	1.95E-01	4.48E-01
9.13	9.25	9.18	1.95E-01	4.48E-01
5.98	5.81	5.80	1.95E-01	4.48E-01
8.83	8.72	8.96	1.95E-01	4.49E-01
7.92	7.75	7.77	1.95E-01	4.49E-01
4.94	5.04	4.99	1.95E-01	4.49E-01
5.24	5.26	5.11	1.95E-01	4.49E-01
8.99	9.15	9.13	1.95E-01	4.49E-01
6.14	6.27	6.00	1.95E-01	4.49E-01
6.46	6.14	5.86	1.96E-01	4.49E-01
8.34	8.38	8.38	1.96E-01	4.49E-01
3.44	3.48	3.64	1.96E-01	4.49E-01
6.93	7.21	7.01	1.96E-01	4.49E-01
12.67	12.77	12.65	1.96E-01	4.49E-01
3.07	3.35	3.24	1.96E-01	4.49E-01
3.93	4.01	4.01	1.96E-01	4.49E-01
9.56	9.45	9.50	1.96E-01	4.49E-01
9.25	9.11	9.27	1.96E-01	4.49E-01
9.73	9.59	9.78	1.96E-01	4.49E-01
6.96	6.95	7.03	1.96E-01	4.49E-01
5.32	5.55	5.61	1.96E-01	4.49E-01
7.47	7.39	7.50	1.96E-01	4.49E-01
3.47	3.54	3.47	1.96E-01	4.49E-01
6.14	5.98	6.02	1.96E-01	4.49E-01
3.91	3.87	3.84	1.96E-01	4.49E-01
7.52	7.33	7.45	1.96E-01	4.49E-01
7.96	7.91	8.18	1.96E-01	4.49E-01

4.04	3.82	4.07	1.96E-01	4.49E-01
3.36	3.35	3.47	1.96E-01	4.49E-01
8.54	8.63	8.63	1.96E-01	4.50E-01
7.55	7.47	7.62	1.96E-01	4.50E-01
4.62	4.53	4.27	1.96E-01	4.50E-01
5.10	5.25	5.14	1.96E-01	4.50E-01
10.60	10.49	10.63	1.97E-01	4.50E-01
4.19	4.04	4.22	1.97E-01	4.50E-01
6.46	6.23	6.27	1.97E-01	4.50E-01
11.52	11.42	11.38	1.97E-01	4.50E-01
10.89	10.80	10.82	1.97E-01	4.50E-01
9.09	9.29	9.18	1.97E-01	4.50E-01
3.80	3.83	3.72	1.97E-01	4.50E-01
6.93	6.81	6.95	1.97E-01	4.50E-01
5.20	5.28	5.35	1.97E-01	4.50E-01
5.81	5.80	5.85	1.97E-01	4.50E-01
10.10	10.12	10.05	1.97E-01	4.50E-01
14.75	14.90	14.85	1.97E-01	4.50E-01
9.98	9.71	9.88	1.97E-01	4.51E-01
8.29	7.87	7.97	1.97E-01	4.51E-01
9.77	9.87	9.81	1.97E-01	4.51E-01
8.91	8.95	8.92	1.97E-01	4.51E-01
9.44	9.24	9.40	1.97E-01	4.51E-01
10.57	10.47	10.56	1.97E-01	4.51E-01
11.40	11.58	11.49	1.97E-01	4.51E-01
7.74	7.68	7.72	1.98E-01	4.51E-01
5.18	5.18	5.13	1.98E-01	4.51E-01
6.91	7.22	7.03	1.98E-01	4.51E-01
9.66	9.81	9.77	1.98E-01	4.51E-01
9.20	9.41	9.37	1.98E-01	4.51E-01
6.43	6.33	6.34	1.98E-01	4.51E-01
14.69	14.62	14.69	1.98E-01	4.51E-01
8.14	8.10	8.17	1.98E-01	4.51E-01
6.12	6.15	6.25	1.98E-01	4.51E-01
9.74	9.97	9.83	1.98E-01	4.52E-01
7.84	7.96	7.86	1.98E-01	4.52E-01
6.30	6.37	6.30	1.98E-01	4.52E-01
7.90	7.95	7.89	1.98E-01	4.52E-01
9.36	9.43	9.45	1.98E-01	4.52E-01
6.80	6.90	6.76	1.98E-01	4.52E-01
6.23	6.36	6.24	1.98E-01	4.52E-01
5.21	5.56	5.43	1.98E-01	4.52E-01
6.47	6.84	6.51	1.98E-01	4.52E-01
4.56	4.39	4.22	1.98E-01	4.52E-01
5.53	5.13	5.13	1.98E-01	4.52E-01
7.89	7.82	8.00	1.98E-01	4.52E-01
6.22	6.40	6.13	1.98E-01	4.52E-01

5.94	5.75	5.96	1.98E-01	4.52E-01
7.40	7.45	7.49	1.98E-01	4.52E-01
6.54	6.54	6.59	1.98E-01	4.52E-01
9.18	9.27	9.25	1.98E-01	4.52E-01
5.00	4.90	4.95	1.99E-01	4.52E-01
7.79	7.88	7.87	1.99E-01	4.52E-01
5.71	5.67	5.61	1.99E-01	4.52E-01
5.57	5.57	5.62	1.99E-01	4.52E-01
6.83	6.60	6.72	1.99E-01	4.52E-01
5.28	5.48	5.27	1.99E-01	4.52E-01
6.16	6.19	5.95	1.99E-01	4.52E-01
4.72	4.58	4.58	1.99E-01	4.52E-01
4.90	5.00	5.00	1.99E-01	4.52E-01
9.96	9.76	9.81	1.99E-01	4.52E-01
3.88	3.80	3.68	1.99E-01	4.52E-01
7.14	7.20	7.34	1.99E-01	4.52E-01
9.79	9.73	10.01	1.99E-01	4.52E-01
8.63	8.96	8.86	1.99E-01	4.52E-01
13.26	13.14	13.20	1.99E-01	4.52E-01
3.81	3.90	3.88	1.99E-01	4.52E-01
6.76	6.99	6.89	1.99E-01	4.52E-01
12.31	12.34	12.29	1.99E-01	4.52E-01
6.79	6.82	6.77	1.99E-01	4.52E-01
5.39	5.49	5.27	1.99E-01	4.52E-01
8.72	8.50	8.76	1.99E-01	4.52E-01
6.41	6.27	6.16	1.99E-01	4.52E-01
3.27	3.43	3.09	1.99E-01	4.52E-01
10.15	9.86	10.27	1.99E-01	4.52E-01
4.16	4.24	4.22	2.00E-01	4.52E-01
10.77	11.06	10.96	2.00E-01	4.52E-01
5.99	6.10	6.00	2.00E-01	4.52E-01
11.78	11.84	11.86	2.00E-01	4.52E-01
10.47	10.43	10.54	2.00E-01	4.52E-01
3.75	3.87	3.81	2.00E-01	4.52E-01
6.08	6.22	6.06	2.00E-01	4.52E-01
6.04	6.11	6.01	2.00E-01	4.52E-01
11.83	11.91	11.83	2.00E-01	4.52E-01
7.85	7.82	7.81	2.00E-01	4.52E-01
6.93	6.78	6.77	2.00E-01	4.52E-01
8.73	8.78	8.82	2.00E-01	4.52E-01
7.41	7.57	7.49	2.00E-01	4.52E-01
10.17	10.24	10.24	2.00E-01	4.52E-01
10.81	10.69	10.80	2.00E-01	4.52E-01
4.72	4.67	4.66	2.00E-01	4.53E-01
7.03	7.05	7.06	2.00E-01	4.53E-01
4.38	4.35	4.17	2.00E-01	4.53E-01
11.12	11.12	11.04	2.00E-01	4.53E-01

6.59	6.72	6.56	2.00E-01	4.53E-01
5.55	5.21	5.37	2.00E-01	4.53E-01
3.85	3.60	3.70	2.01E-01	4.53E-01
9.61	9.51	9.55	2.01E-01	4.53E-01
4.40	4.44	4.64	2.01E-01	4.53E-01
4.00	4.11	4.03	2.01E-01	4.53E-01
7.98	7.78	8.08	2.01E-01	4.53E-01
7.74	7.75	7.95	2.01E-01	4.53E-01
6.15	5.96	5.88	2.01E-01	4.53E-01
10.51	10.63	10.54	2.01E-01	4.53E-01
11.25	11.20	11.36	2.01E-01	4.54E-01
7.02	6.83	6.90	2.01E-01	4.54E-01
6.12	6.39	6.21	2.01E-01	4.54E-01
7.42	7.51	7.40	2.01E-01	4.54E-01
8.51	8.72	8.58	2.01E-01	4.54E-01
4.79	5.48	4.99	2.01E-01	4.54E-01
3.30	3.39	3.32	2.01E-01	4.54E-01
5.76	5.62	5.77	2.01E-01	4.54E-01
8.50	8.36	8.49	2.01E-01	4.54E-01
7.25	7.01	7.01	2.01E-01	4.54E-01
6.98	6.80	6.77	2.01E-01	4.54E-01
4.38	4.11	4.31	2.01E-01	4.54E-01
4.84	4.98	5.06	2.01E-01	4.54E-01
7.39	7.31	7.07	2.01E-01	4.54E-01
5.52	5.50	5.39	2.01E-01	4.54E-01
6.89	6.62	6.84	2.01E-01	4.54E-01
4.07	4.19	4.22	2.01E-01	4.54E-01
11.53	11.50	11.46	2.01E-01	4.54E-01
6.62	6.66	6.64	2.01E-01	4.54E-01
8.58	8.74	8.68	2.01E-01	4.54E-01
6.81	6.81	6.82	2.01E-01	4.54E-01
11.69	11.70	11.76	2.02E-01	4.54E-01
6.35	6.31	6.39	2.02E-01	4.54E-01
10.35	10.48	10.32	2.02E-01	4.54E-01
3.24	3.35	3.36	2.02E-01	4.54E-01
8.93	8.82	8.94	2.02E-01	4.54E-01
5.10	5.42	5.34	2.02E-01	4.54E-01
5.36	5.42	5.37	2.02E-01	4.54E-01
8.24	8.06	8.14	2.02E-01	4.54E-01
12.29	12.23	12.28	2.02E-01	4.54E-01
3.77	3.90	3.70	2.02E-01	4.54E-01
10.33	10.54	10.40	2.02E-01	4.54E-01
13.24	13.30	13.30	2.02E-01	4.54E-01
8.80	8.78	8.86	2.02E-01	4.55E-01
8.05	8.42	8.01	2.02E-01	4.55E-01
3.84	3.73	3.87	2.02E-01	4.55E-01
8.38	8.13	8.34	2.02E-01	4.55E-01

5.13	5.18	5.09	2.02E-01	4.55E-01
9.37	9.62	9.50	2.02E-01	4.55E-01
9.94	9.93	9.92	2.02E-01	4.55E-01
8.20	8.27	8.32	2.03E-01	4.55E-01
3.58	3.59	3.34	2.03E-01	4.55E-01
12.20	12.39	12.31	2.03E-01	4.55E-01
4.67	4.42	4.33	2.03E-01	4.55E-01
8.15	8.22	8.22	2.03E-01	4.55E-01
5.72	5.89	5.83	2.03E-01	4.55E-01
8.80	9.09	8.84	2.03E-01	4.55E-01
5.37	5.30	5.29	2.03E-01	4.55E-01
5.41	5.23	5.48	2.03E-01	4.55E-01
8.37	8.30	8.41	2.03E-01	4.55E-01
7.99	8.12	8.02	2.03E-01	4.55E-01
6.17	6.46	6.31	2.03E-01	4.55E-01
6.43	6.14	6.25	2.03E-01	4.55E-01
9.49	9.68	9.55	2.03E-01	4.55E-01
6.23	6.14	6.14	2.03E-01	4.55E-01
5.56	5.74	5.64	2.03E-01	4.55E-01
10.88	10.72	10.88	2.03E-01	4.55E-01
6.92	6.79	6.80	2.03E-01	4.55E-01
4.63	4.62	4.51	2.03E-01	4.55E-01
10.35	10.15	10.40	2.03E-01	4.55E-01
8.75	8.90	8.77	2.03E-01	4.55E-01
6.90	6.95	6.88	2.03E-01	4.55E-01
7.31	7.35	7.38	2.03E-01	4.55E-01
5.24	5.28	5.32	2.03E-01	4.55E-01
10.92	10.83	10.75	2.03E-01	4.55E-01
9.89	9.97	10.04	2.03E-01	4.55E-01
4.37	4.17	4.32	2.04E-01	4.55E-01
9.67	9.76	9.75	2.04E-01	4.55E-01
11.31	11.50	11.52	2.04E-01	4.56E-01
4.96	5.00	4.91	2.04E-01	4.56E-01
6.31	6.24	6.22	2.04E-01	4.56E-01
6.88	6.96	7.12	2.04E-01	4.56E-01
8.42	8.49	8.45	2.04E-01	4.56E-01
12.01	12.22	12.14	2.04E-01	4.56E-01
11.13	11.17	11.18	2.04E-01	4.56E-01
6.05	5.93	5.95	2.04E-01	4.56E-01
7.56	7.75	7.80	2.04E-01	4.56E-01
7.29	7.22	7.29	2.04E-01	4.56E-01
4.18	4.22	4.19	2.04E-01	4.56E-01
9.54	9.44	9.47	2.04E-01	4.56E-01
7.95	8.20	8.04	2.04E-01	4.56E-01
11.76	11.80	11.81	2.04E-01	4.56E-01
6.59	6.55	6.47	2.04E-01	4.56E-01
11.07	11.01	11.05	2.04E-01	4.56E-01

8.65	8.63	8.77	2.04E-01	4.56E-01
6.96	7.00	6.96	2.04E-01	4.56E-01
7.09	7.34	7.29	2.04E-01	4.56E-01
9.53	9.59	9.63	2.04E-01	4.56E-01
8.02	8.07	8.14	2.04E-01	4.56E-01
9.08	9.06	9.12	2.05E-01	4.57E-01
3.55	3.36	3.37	2.05E-01	4.57E-01
6.86	6.97	6.81	2.05E-01	4.57E-01
8.15	8.46	8.19	2.05E-01	4.57E-01
3.33	3.43	3.33	2.05E-01	4.57E-01
9.58	9.55	9.67	2.05E-01	4.57E-01
11.46	11.42	11.49	2.05E-01	4.57E-01
4.18	4.21	4.14	2.05E-01	4.57E-01
8.74	8.60	8.76	2.05E-01	4.57E-01
3.97	4.24	4.07	2.05E-01	4.57E-01
5.10	5.06	4.83	2.05E-01	4.57E-01
3.50	3.24	3.28	2.05E-01	4.57E-01
5.78	6.37	6.04	2.05E-01	4.57E-01
3.84	4.33	3.82	2.05E-01	4.57E-01
8.89	8.79	8.87	2.05E-01	4.57E-01
9.26	9.33	9.16	2.05E-01	4.57E-01
4.55	4.57	4.57	2.05E-01	4.57E-01
9.46	9.56	9.50	2.05E-01	4.57E-01
6.10	6.24	6.23	2.06E-01	4.57E-01
4.69	4.64	4.74	2.06E-01	4.57E-01
5.72	5.64	5.73	2.06E-01	4.57E-01
4.85	4.64	5.00	2.06E-01	4.57E-01
5.83	5.88	5.67	2.06E-01	4.57E-01
7.26	7.07	6.95	2.06E-01	4.57E-01
7.82	7.58	7.94	2.06E-01	4.57E-01
5.84	5.83	5.77	2.06E-01	4.57E-01
6.39	6.29	6.07	2.06E-01	4.57E-01
3.01	3.18	3.24	2.06E-01	4.57E-01
3.71	3.87	3.85	2.06E-01	4.57E-01
7.53	7.74	7.60	2.06E-01	4.57E-01
5.04	5.20	5.14	2.06E-01	4.57E-01
5.06	5.04	5.32	2.06E-01	4.57E-01
4.05	3.91	3.79	2.06E-01	4.57E-01
7.73	7.97	7.63	2.06E-01	4.57E-01
4.16	4.10	4.16	2.06E-01	4.57E-01
8.91	9.03	9.09	2.06E-01	4.57E-01
5.04	4.90	5.17	2.06E-01	4.57E-01
4.09	3.96	4.17	2.06E-01	4.57E-01
9.84	9.78	9.97	2.06E-01	4.57E-01
9.14	9.13	9.15	2.06E-01	4.57E-01
5.88	5.85	5.87	2.06E-01	4.57E-01
9.94	10.21	10.04	2.06E-01	4.58E-01

9.02	9.22	9.11	2.06E-01	4.58E-01
8.94	9.28	9.03	2.06E-01	4.58E-01
10.01	10.20	10.14	2.06E-01	4.58E-01
10.98	11.12	11.10	2.06E-01	4.58E-01
6.36	6.34	6.25	2.07E-01	4.58E-01
5.95	5.63	5.68	2.07E-01	4.58E-01
3.94	3.83	3.95	2.07E-01	4.58E-01
7.53	7.90	7.85	2.07E-01	4.58E-01
8.07	8.24	8.22	2.07E-01	4.58E-01
8.94	9.07	9.07	2.07E-01	4.58E-01
7.34	7.29	7.26	2.07E-01	4.58E-01
6.05	5.80	5.83	2.07E-01	4.58E-01
8.16	8.23	8.32	2.07E-01	4.58E-01
8.12	8.28	8.07	2.07E-01	4.58E-01
5.55	5.40	5.28	2.07E-01	4.58E-01
8.24	8.25	8.22	2.07E-01	4.58E-01
8.45	8.51	8.57	2.07E-01	4.58E-01
4.98	5.08	5.12	2.07E-01	4.58E-01
14.00	13.96	13.99	2.07E-01	4.58E-01
5.23	5.02	5.09	2.07E-01	4.58E-01
5.42	5.70	5.68	2.07E-01	4.58E-01
8.54	8.82	8.75	2.07E-01	4.58E-01
10.50	10.55	10.65	2.07E-01	4.58E-01
7.68	7.69	7.74	2.07E-01	4.58E-01
5.47	5.73	5.98	2.07E-01	4.58E-01
4.47	4.42	3.95	2.07E-01	4.58E-01
11.13	11.26	11.19	2.07E-01	4.58E-01
6.76	6.88	6.79	2.07E-01	4.58E-01
6.16	6.04	6.13	2.07E-01	4.58E-01
6.94	7.08	7.21	2.07E-01	4.58E-01
6.58	6.58	6.44	2.08E-01	4.58E-01
8.10	8.11	8.01	2.08E-01	4.58E-01
7.74	7.76	7.85	2.08E-01	4.58E-01
7.83	7.41	7.76	2.08E-01	4.58E-01
10.44	10.55	10.58	2.08E-01	4.58E-01
7.43	7.45	7.47	2.08E-01	4.58E-01
4.34	4.63	4.37	2.08E-01	4.58E-01
7.34	7.19	7.16	2.08E-01	4.58E-01
5.85	6.25	5.95	2.08E-01	4.58E-01
4.35	4.37	4.35	2.08E-01	4.58E-01
5.71	5.44	5.44	2.08E-01	4.58E-01
7.15	7.27	7.26	2.08E-01	4.58E-01
8.81	8.83	8.70	2.08E-01	4.58E-01
5.58	5.59	5.70	2.08E-01	4.58E-01
5.87	5.62	5.48	2.08E-01	4.58E-01
11.05	10.99	11.09	2.08E-01	4.58E-01
7.71	7.90	7.86	2.08E-01	4.58E-01

8.26	8.23	8.18	2.08E-01	4.58E-01
3.75	3.62	3.62	2.08E-01	4.58E-01
3.90	3.93	3.99	2.08E-01	4.58E-01
8.85	9.02	9.06	2.08E-01	4.58E-01
7.67	7.84	7.76	2.08E-01	4.58E-01
9.66	9.46	9.64	2.08E-01	4.58E-01
8.78	8.83	8.76	2.08E-01	4.58E-01
8.31	8.49	8.60	2.08E-01	4.58E-01
4.51	4.59	4.32	2.08E-01	4.58E-01
6.44	6.80	6.66	2.08E-01	4.58E-01
5.41	5.18	5.48	2.08E-01	4.58E-01
5.09	5.44	5.16	2.08E-01	4.58E-01
3.79	3.71	3.76	2.08E-01	4.58E-01
6.43	6.56	6.60	2.08E-01	4.58E-01
9.11	9.08	9.05	2.08E-01	4.58E-01
10.88	11.05	10.85	2.08E-01	4.58E-01
13.48	13.59	13.48	2.08E-01	4.58E-01
12.65	12.70	12.74	2.08E-01	4.58E-01
3.91	3.73	3.84	2.09E-01	4.58E-01
11.14	11.26	11.16	2.09E-01	4.58E-01
7.07	7.49	7.15	2.09E-01	4.59E-01
14.04	13.97	13.99	2.09E-01	4.59E-01
4.49	4.27	4.38	2.09E-01	4.59E-01
5.92	5.77	6.02	2.09E-01	4.59E-01
10.31	10.36	10.23	2.09E-01	4.59E-01
4.79	4.63	4.85	2.09E-01	4.59E-01
7.76	7.89	7.78	2.09E-01	4.59E-01
5.15	5.09	5.14	2.09E-01	4.59E-01
3.51	3.75	3.57	2.09E-01	4.59E-01
5.03	5.09	5.06	2.09E-01	4.59E-01
4.12	4.06	4.13	2.09E-01	4.59E-01
8.53	8.42	8.59	2.09E-01	4.59E-01
10.91	11.05	10.86	2.09E-01	4.59E-01
5.02	5.23	5.41	2.09E-01	4.59E-01
7.56	7.79	7.48	2.09E-01	4.59E-01
3.51	3.34	3.44	2.09E-01	4.59E-01
9.42	9.33	9.43	2.09E-01	4.59E-01
4.04	3.85	3.97	2.09E-01	4.59E-01
7.79	7.50	7.77	2.09E-01	4.59E-01
7.22	7.28	7.12	2.09E-01	4.59E-01
4.60	4.35	4.24	2.10E-01	4.59E-01
5.90	5.95	6.18	2.10E-01	4.60E-01
8.61	8.57	8.61	2.10E-01	4.60E-01
9.84	10.02	10.14	2.10E-01	4.60E-01
9.68	9.64	9.60	2.10E-01	4.60E-01
4.06	4.11	4.24	2.10E-01	4.60E-01
7.53	7.72	7.68	2.10E-01	4.60E-01

4.27	4.32	4.25	2.10E-01	4.60E-01
8.36	8.42	8.18	2.10E-01	4.60E-01
4.76	5.01	5.01	2.10E-01	4.60E-01
7.86	7.62	7.76	2.10E-01	4.60E-01
7.71	7.65	7.74	2.10E-01	4.60E-01
8.70	8.61	8.63	2.10E-01	4.60E-01
10.65	10.81	10.73	2.10E-01	4.60E-01
5.63	5.75	5.54	2.10E-01	4.60E-01
6.32	6.24	6.16	2.10E-01	4.60E-01
10.45	10.29	10.33	2.10E-01	4.60E-01
7.60	7.48	7.82	2.10E-01	4.60E-01
8.06	8.15	8.07	2.10E-01	4.60E-01
11.01	10.55	10.90	2.10E-01	4.60E-01
9.28	9.44	9.42	2.10E-01	4.60E-01
8.21	8.31	8.12	2.10E-01	4.60E-01
6.55	6.65	6.52	2.10E-01	4.60E-01
6.67	6.97	6.64	2.10E-01	4.60E-01
6.34	6.43	6.48	2.11E-01	4.60E-01
5.59	5.72	5.43	2.11E-01	4.60E-01
9.90	9.98	9.97	2.11E-01	4.60E-01
10.40	10.43	10.36	2.11E-01	4.60E-01
3.89	3.89	3.92	2.11E-01	4.60E-01
9.55	9.54	9.46	2.11E-01	4.60E-01
6.07	6.00	5.98	2.11E-01	4.60E-01
4.43	4.29	4.45	2.11E-01	4.60E-01
8.75	8.88	8.87	2.11E-01	4.60E-01
4.77	4.64	4.97	2.11E-01	4.60E-01
3.59	3.38	3.57	2.11E-01	4.60E-01
6.86	7.07	6.98	2.11E-01	4.60E-01
3.66	3.59	3.65	2.11E-01	4.61E-01
12.66	12.77	12.72	2.11E-01	4.61E-01
10.79	11.01	10.99	2.11E-01	4.61E-01
8.22	8.33	8.20	2.11E-01	4.61E-01
7.09	7.11	6.99	2.11E-01	4.61E-01
4.59	4.46	4.47	2.11E-01	4.61E-01
5.12	5.50	5.35	2.11E-01	4.61E-01
6.32	6.19	6.27	2.11E-01	4.61E-01
3.64	3.84	3.80	2.11E-01	4.61E-01
8.05	8.07	7.88	2.11E-01	4.61E-01
4.10	3.85	3.89	2.12E-01	4.61E-01
3.41	3.34	3.25	2.12E-01	4.61E-01
7.91	7.95	7.92	2.12E-01	4.61E-01
9.73	10.04	9.72	2.12E-01	4.61E-01
5.20	5.09	4.78	2.12E-01	4.61E-01
6.14	6.08	6.24	2.12E-01	4.61E-01
7.63	7.91	7.67	2.12E-01	4.61E-01
5.72	5.80	5.73	2.12E-01	4.61E-01

7.78	7.58	7.60	2.12E-01	4.61E-01
5.99	6.17	6.16	2.12E-01	4.61E-01
4.46	5.15	4.37	2.12E-01	4.61E-01
8.81	8.67	8.75	2.12E-01	4.61E-01
7.46	7.69	7.58	2.12E-01	4.61E-01
7.10	6.92	6.85	2.12E-01	4.61E-01
4.69	4.79	4.72	2.12E-01	4.61E-01
6.02	6.05	6.08	2.12E-01	4.61E-01
5.55	5.56	5.37	2.12E-01	4.61E-01
8.12	8.01	8.07	2.12E-01	4.61E-01
3.54	3.41	3.37	2.12E-01	4.61E-01
6.01	6.31	6.26	2.12E-01	4.61E-01
6.01	5.65	5.98	2.12E-01	4.62E-01
4.43	4.68	4.69	2.13E-01	4.62E-01
3.40	3.37	3.38	2.13E-01	4.62E-01
5.88	5.87	5.91	2.13E-01	4.62E-01
10.57	10.70	10.52	2.13E-01	4.62E-01
10.77	10.75	10.81	2.13E-01	4.62E-01
5.16	5.06	5.01	2.13E-01	4.62E-01
4.99	5.24	4.98	2.13E-01	4.62E-01
10.92	10.68	10.88	2.13E-01	4.62E-01
8.11	7.88	8.10	2.13E-01	4.62E-01
8.56	8.85	8.76	2.13E-01	4.62E-01
8.25	8.35	8.17	2.13E-01	4.62E-01
4.59	4.41	4.57	2.13E-01	4.62E-01
5.37	5.31	5.38	2.13E-01	4.62E-01
4.30	4.59	4.44	2.13E-01	4.62E-01
10.20	10.18	10.34	2.13E-01	4.62E-01
7.56	7.51	7.47	2.13E-01	4.62E-01
6.50	6.60	6.64	2.13E-01	4.62E-01
5.78	5.91	5.74	2.13E-01	4.62E-01
7.06	7.19	7.12	2.13E-01	4.62E-01
3.62	3.60	3.48	2.13E-01	4.62E-01
5.05	4.91	4.81	2.13E-01	4.62E-01
10.70	10.42	10.63	2.13E-01	4.62E-01
4.57	4.44	4.60	2.14E-01	4.62E-01
3.90	3.79	3.76	2.14E-01	4.62E-01
10.77	11.01	10.91	2.14E-01	4.62E-01
6.21	6.25	6.14	2.14E-01	4.62E-01
8.70	8.80	8.79	2.14E-01	4.62E-01
10.09	9.99	10.02	2.14E-01	4.62E-01
3.93	3.83	4.06	2.14E-01	4.62E-01
7.80	7.82	7.71	2.14E-01	4.62E-01
4.76	4.87	4.67	2.14E-01	4.62E-01
5.57	5.65	5.58	2.14E-01	4.62E-01
7.22	7.19	7.01	2.14E-01	4.62E-01
8.32	8.52	8.26	2.14E-01	4.62E-01

3.03	3.22	3.08	2.14E-01	4.62E-01
4.34	4.35	4.42	2.14E-01	4.62E-01
6.06	6.01	6.00	2.14E-01	4.63E-01
5.68	5.88	5.91	2.14E-01	4.63E-01
9.26	9.25	9.22	2.14E-01	4.63E-01
5.47	5.37	5.23	2.14E-01	4.63E-01
10.36	10.23	10.40	2.14E-01	4.63E-01
9.16	9.14	9.03	2.14E-01	4.63E-01
9.61	9.75	9.69	2.15E-01	4.63E-01
8.70	8.74	8.67	2.15E-01	4.63E-01
7.53	7.48	7.58	2.15E-01	4.63E-01
6.79	7.20	7.01	2.15E-01	4.64E-01
6.43	6.68	6.69	2.15E-01	4.64E-01
4.88	4.55	4.57	2.15E-01	4.64E-01
8.49	8.17	8.39	2.15E-01	4.64E-01
5.65	5.82	5.74	2.15E-01	4.64E-01
5.48	5.38	5.34	2.15E-01	4.64E-01
5.19	5.65	5.16	2.15E-01	4.64E-01
9.75	9.88	9.71	2.15E-01	4.64E-01
10.63	10.52	10.59	2.15E-01	4.64E-01
7.28	7.22	7.16	2.15E-01	4.64E-01
14.29	14.36	14.37	2.15E-01	4.64E-01
10.11	10.24	10.24	2.15E-01	4.64E-01
7.58	7.69	7.59	2.15E-01	4.64E-01
8.26	8.52	8.25	2.15E-01	4.64E-01
7.69	7.78	7.86	2.15E-01	4.64E-01
8.92	8.69	8.85	2.15E-01	4.64E-01
7.48	7.59	7.48	2.15E-01	4.64E-01
3.40	3.35	3.33	2.15E-01	4.64E-01
10.77	10.81	10.81	2.15E-01	4.64E-01
9.49	9.23	9.47	2.15E-01	4.64E-01
5.05	4.71	4.88	2.15E-01	4.64E-01
5.00	4.88	5.09	2.15E-01	4.64E-01
7.96	7.90	7.90	2.15E-01	4.64E-01
7.26	7.06	7.52	2.15E-01	4.64E-01
7.94	8.61	8.00	2.15E-01	4.64E-01
8.90	8.99	8.97	2.16E-01	4.64E-01
6.61	6.78	6.83	2.16E-01	4.64E-01
4.39	4.46	4.50	2.16E-01	4.64E-01
7.16	7.20	7.24	2.16E-01	4.64E-01
4.17	4.23	4.02	2.16E-01	4.64E-01
8.68	8.81	8.81	2.16E-01	4.64E-01
9.90	9.91	9.89	2.16E-01	4.64E-01
8.37	8.23	8.35	2.16E-01	4.64E-01
9.75	9.80	9.66	2.16E-01	4.64E-01
8.64	8.60	8.55	2.16E-01	4.64E-01
4.76	4.77	4.69	2.16E-01	4.64E-01

3.77	3.67	3.50	2.16E-01	4.64E-01
7.71	7.83	7.85	2.16E-01	4.64E-01
7.57	7.60	7.58	2.16E-01	4.64E-01
3.70	3.71	3.45	2.16E-01	4.65E-01
10.45	10.52	10.43	2.16E-01	4.65E-01
6.69	6.61	6.44	2.16E-01	4.65E-01
5.14	5.15	5.20	2.16E-01	4.65E-01
8.23	8.23	8.23	2.16E-01	4.65E-01
11.57	11.77	11.74	2.17E-01	4.65E-01
6.55	6.82	6.54	2.17E-01	4.65E-01
8.66	8.82	8.74	2.17E-01	4.65E-01
4.50	4.56	4.60	2.17E-01	4.65E-01
9.80	9.97	9.92	2.17E-01	4.65E-01
10.40	10.60	10.45	2.17E-01	4.65E-01
7.97	7.97	8.12	2.17E-01	4.65E-01
7.83	8.15	8.02	2.17E-01	4.65E-01
11.97	12.09	12.02	2.17E-01	4.65E-01
4.39	4.51	4.39	2.17E-01	4.65E-01
3.50	3.30	3.82	2.17E-01	4.65E-01
4.12	4.24	4.08	2.17E-01	4.65E-01
4.40	4.38	4.28	2.17E-01	4.65E-01
5.52	5.46	5.39	2.17E-01	4.65E-01
4.07	4.34	4.13	2.17E-01	4.65E-01
7.73	7.80	7.82	2.17E-01	4.65E-01
9.50	9.28	9.45	2.17E-01	4.65E-01
8.26	8.60	8.36	2.17E-01	4.65E-01
6.25	6.15	6.12	2.17E-01	4.65E-01
8.38	8.51	8.25	2.17E-01	4.65E-01
6.48	6.44	6.43	2.17E-01	4.65E-01
5.51	5.49	5.53	2.17E-01	4.65E-01
10.57	10.68	10.61	2.17E-01	4.65E-01
6.62	6.54	6.59	2.17E-01	4.65E-01
7.63	7.61	7.76	2.17E-01	4.65E-01
9.04	9.08	9.15	2.17E-01	4.65E-01
8.28	8.14	8.41	2.18E-01	4.66E-01
2.95	3.07	3.01	2.18E-01	4.66E-01
5.47	5.71	5.46	2.18E-01	4.66E-01
3.14	3.24	3.42	2.18E-01	4.66E-01
11.82	11.70	11.66	2.18E-01	4.66E-01
5.87	5.96	5.77	2.18E-01	4.66E-01
8.87	8.84	9.05	2.18E-01	4.66E-01
6.99	6.83	6.96	2.18E-01	4.66E-01
5.85	6.08	5.97	2.18E-01	4.66E-01
8.67	8.81	8.69	2.18E-01	4.66E-01
4.92	4.80	4.64	2.18E-01	4.66E-01
7.65	7.85	7.81	2.18E-01	4.66E-01
4.72	4.93	5.55	2.18E-01	4.66E-01

4.95	5.25	5.20	2.18E-01	4.66E-01
5.69	5.50	5.66	2.18E-01	4.66E-01
8.80	9.11	9.02	2.18E-01	4.66E-01
6.41	6.24	6.32	2.18E-01	4.66E-01
5.33	5.26	5.22	2.18E-01	4.66E-01
6.62	6.82	6.81	2.18E-01	4.66E-01
4.25	4.27	4.34	2.18E-01	4.66E-01
10.93	10.85	10.88	2.18E-01	4.66E-01
10.17	10.26	10.12	2.18E-01	4.66E-01
4.30	4.22	4.36	2.18E-01	4.66E-01
7.48	7.52	7.72	2.18E-01	4.66E-01
8.15	8.39	8.26	2.19E-01	4.66E-01
3.69	3.80	3.44	2.19E-01	4.66E-01
4.10	3.86	4.21	2.19E-01	4.66E-01
6.30	6.25	6.40	2.19E-01	4.66E-01
7.53	7.87	7.58	2.19E-01	4.66E-01
8.89	8.65	8.61	2.19E-01	4.66E-01
4.02	4.03	4.36	2.19E-01	4.66E-01
6.59	6.56	6.51	2.19E-01	4.66E-01
6.68	6.41	6.63	2.19E-01	4.66E-01
3.97	4.13	3.95	2.19E-01	4.66E-01
4.34	4.18	4.44	2.19E-01	4.66E-01
7.96	8.12	8.25	2.19E-01	4.66E-01
9.07	9.18	9.06	2.19E-01	4.66E-01
3.26	3.40	3.19	2.19E-01	4.66E-01
10.02	9.80	10.13	2.19E-01	4.66E-01
8.14	8.29	8.22	2.19E-01	4.66E-01
3.84	4.03	4.05	2.19E-01	4.66E-01
8.32	8.20	8.22	2.19E-01	4.66E-01
8.19	8.47	8.46	2.19E-01	4.66E-01
4.38	4.37	4.34	2.19E-01	4.66E-01
4.82	4.92	5.15	2.19E-01	4.66E-01
5.64	5.54	5.60	2.19E-01	4.66E-01
4.42	4.40	4.13	2.19E-01	4.67E-01
7.21	7.32	7.25	2.19E-01	4.67E-01
10.44	10.70	10.59	2.19E-01	4.67E-01
6.33	6.34	6.39	2.20E-01	4.67E-01
5.51	5.47	5.30	2.20E-01	4.67E-01
6.03	6.18	6.08	2.20E-01	4.67E-01
6.21	6.31	6.22	2.20E-01	4.67E-01
5.39	5.54	5.41	2.20E-01	4.67E-01
7.84	8.11	7.96	2.20E-01	4.67E-01
6.41	6.14	6.30	2.20E-01	4.67E-01
7.99	7.89	8.12	2.20E-01	4.67E-01
4.97	4.74	4.93	2.20E-01	4.67E-01
8.83	8.69	8.99	2.20E-01	4.67E-01
3.58	3.55	3.55	2.20E-01	4.67E-01

10.43	10.57	10.47	2.20E-01	4.67E-01
10.78	10.65	10.53	2.20E-01	4.67E-01
4.95	4.85	4.85	2.20E-01	4.67E-01
3.19	3.05	3.05	2.20E-01	4.67E-01
5.52	5.61	5.69	2.20E-01	4.67E-01
10.03	10.12	10.13	2.20E-01	4.67E-01
4.90	4.69	4.67	2.20E-01	4.67E-01
7.96	8.03	8.11	2.20E-01	4.67E-01
3.03	3.31	2.99	2.20E-01	4.67E-01
3.60	3.77	4.02	2.20E-01	4.67E-01
5.51	5.76	5.86	2.20E-01	4.67E-01
3.79	3.81	3.80	2.20E-01	4.67E-01
10.07	10.07	10.08	2.20E-01	4.67E-01
5.32	5.30	5.24	2.20E-01	4.67E-01
4.91	4.68	4.75	2.20E-01	4.67E-01
6.77	6.88	6.87	2.20E-01	4.67E-01
7.64	7.59	7.59	2.20E-01	4.67E-01
10.62	10.56	10.68	2.21E-01	4.67E-01
11.35	11.51	11.42	2.21E-01	4.67E-01
8.50	8.54	8.65	2.21E-01	4.67E-01
8.45	8.55	8.41	2.21E-01	4.67E-01
12.08	12.16	12.23	2.21E-01	4.67E-01
8.33	8.27	8.48	2.21E-01	4.67E-01
7.12	7.17	7.16	2.21E-01	4.67E-01
6.32	5.93	6.20	2.21E-01	4.67E-01
8.58	8.58	8.63	2.21E-01	4.67E-01
5.81	5.69	5.53	2.21E-01	4.67E-01
7.66	7.80	7.72	2.21E-01	4.67E-01
7.22	7.26	7.31	2.21E-01	4.67E-01
5.38	5.47	5.48	2.21E-01	4.67E-01
8.32	8.56	8.49	2.21E-01	4.67E-01
6.60	6.57	6.63	2.21E-01	4.67E-01
6.07	5.98	6.03	2.21E-01	4.67E-01
7.07	7.27	7.32	2.21E-01	4.67E-01
14.78	14.82	14.80	2.21E-01	4.68E-01
8.69	8.45	8.56	2.21E-01	4.68E-01
7.38	7.38	7.38	2.21E-01	4.68E-01
3.13	3.02	3.27	2.21E-01	4.68E-01
5.27	5.27	5.02	2.21E-01	4.68E-01
8.13	7.97	7.97	2.21E-01	4.68E-01
5.13	5.38	5.40	2.21E-01	4.68E-01
5.16	5.27	5.06	2.21E-01	4.68E-01
3.86	4.23	4.07	2.21E-01	4.68E-01
9.36	9.57	9.49	2.22E-01	4.68E-01
8.44	8.46	8.40	2.22E-01	4.68E-01
5.34	5.19	5.48	2.22E-01	4.68E-01
4.25	4.32	4.39	2.22E-01	4.68E-01

5.67	5.93	6.05	2.22E-01	4.68E-01
3.80	3.91	3.84	2.22E-01	4.68E-01
8.73	9.03	8.88	2.22E-01	4.68E-01
9.53	9.53	9.61	2.22E-01	4.68E-01
4.27	4.30	4.23	2.22E-01	4.68E-01
3.68	3.42	3.42	2.22E-01	4.68E-01
6.42	6.41	6.47	2.22E-01	4.68E-01
7.69	7.54	7.55	2.22E-01	4.68E-01
7.47	7.46	7.58	2.22E-01	4.68E-01
5.95	5.84	5.96	2.22E-01	4.68E-01
8.08	8.22	8.15	2.22E-01	4.68E-01
12.25	12.21	12.28	2.22E-01	4.68E-01
9.80	9.79	9.90	2.22E-01	4.68E-01
9.68	9.95	9.79	2.22E-01	4.68E-01
10.59	10.57	10.65	2.22E-01	4.68E-01
3.93	4.03	3.95	2.22E-01	4.68E-01
8.19	8.30	8.23	2.22E-01	4.69E-01
4.39	4.32	4.42	2.23E-01	4.69E-01
9.41	9.02	9.40	2.23E-01	4.69E-01
5.52	5.48	5.47	2.23E-01	4.69E-01
6.47	6.46	6.53	2.23E-01	4.69E-01
10.28	10.24	10.29	2.23E-01	4.69E-01
10.13	9.73	10.00	2.23E-01	4.69E-01
6.89	6.84	6.98	2.23E-01	4.69E-01
9.87	9.93	9.87	2.23E-01	4.69E-01
3.40	3.25	3.32	2.23E-01	4.70E-01
4.22	4.18	4.16	2.23E-01	4.70E-01
5.98	6.02	6.10	2.23E-01	4.70E-01
10.19	10.08	10.13	2.23E-01	4.70E-01
3.30	3.46	3.34	2.23E-01	4.70E-01
9.49	9.48	9.44	2.23E-01	4.70E-01
6.14	6.06	6.22	2.24E-01	4.70E-01
6.05	5.76	5.91	2.24E-01	4.70E-01
10.21	10.42	10.26	2.24E-01	4.70E-01
3.19	3.30	3.23	2.24E-01	4.70E-01
5.24	5.48	5.25	2.24E-01	4.70E-01
3.23	3.10	3.20	2.24E-01	4.70E-01
4.17	4.13	4.08	2.24E-01	4.70E-01
5.86	5.82	5.72	2.24E-01	4.70E-01
6.45	6.30	6.25	2.24E-01	4.70E-01
6.86	7.07	6.94	2.24E-01	4.70E-01
9.43	9.54	9.53	2.24E-01	4.70E-01
6.94	7.23	7.15	2.24E-01	4.70E-01
8.44	8.49	8.38	2.24E-01	4.70E-01
7.14	7.14	7.02	2.24E-01	4.70E-01
5.22	5.15	5.20	2.24E-01	4.70E-01
6.22	6.30	6.28	2.24E-01	4.70E-01

6.75	7.26	6.44	2.24E-01	4.70E-01
7.53	7.62	7.59	2.24E-01	4.70E-01
6.51	6.63	6.49	2.24E-01	4.70E-01
4.48	4.57	4.42	2.24E-01	4.71E-01
6.34	6.47	6.19	2.25E-01	4.71E-01
4.80	4.87	4.75	2.25E-01	4.71E-01
5.27	5.23	5.08	2.25E-01	4.71E-01
7.12	7.14	7.15	2.25E-01	4.71E-01
10.88	10.85	10.86	2.25E-01	4.71E-01
5.19	5.02	4.98	2.25E-01	4.71E-01
7.30	7.29	7.27	2.25E-01	4.71E-01
4.89	4.69	4.72	2.25E-01	4.71E-01
4.62	4.80	4.70	2.25E-01	4.71E-01
8.40	8.32	8.52	2.25E-01	4.71E-01
3.40	3.60	3.86	2.25E-01	4.71E-01
9.92	9.80	10.00	2.25E-01	4.71E-01
6.84	6.90	7.02	2.25E-01	4.71E-01
5.08	5.12	5.33	2.25E-01	4.71E-01
4.38	4.39	4.45	2.25E-01	4.71E-01
3.93	3.95	3.87	2.25E-01	4.71E-01
6.19	5.90	5.96	2.25E-01	4.71E-01
5.75	5.55	5.71	2.25E-01	4.71E-01
8.94	8.55	8.85	2.25E-01	4.71E-01
6.75	6.81	6.91	2.25E-01	4.71E-01
12.44	12.44	12.47	2.25E-01	4.71E-01
7.56	7.36	7.29	2.25E-01	4.71E-01
5.78	5.83	5.77	2.26E-01	4.72E-01
10.26	9.95	10.08	2.26E-01	4.72E-01
10.99	11.08	10.95	2.26E-01	4.72E-01
3.84	3.89	3.90	2.26E-01	4.72E-01
10.45	10.52	10.33	2.26E-01	4.72E-01
7.21	7.74	7.25	2.26E-01	4.72E-01
8.62	8.47	8.56	2.26E-01	4.72E-01
8.85	8.91	9.01	2.26E-01	4.72E-01
7.03	7.06	7.18	2.26E-01	4.72E-01
8.08	7.92	8.22	2.26E-01	4.72E-01
10.91	10.96	11.04	2.26E-01	4.72E-01
2.93	3.13	3.14	2.26E-01	4.72E-01
6.45	6.06	6.27	2.26E-01	4.72E-01
6.01	6.29	5.88	2.26E-01	4.72E-01
9.75	9.51	9.73	2.26E-01	4.72E-01
5.66	5.51	5.41	2.26E-01	4.72E-01
8.22	7.92	8.07	2.26E-01	4.72E-01
11.37	11.30	11.41	2.26E-01	4.72E-01
7.05	6.96	7.00	2.26E-01	4.72E-01
3.63	3.74	3.63	2.26E-01	4.72E-01
3.92	4.07	3.83	2.26E-01	4.72E-01

6.33	6.20	6.15	2.26E-01	4.72E-01
5.46	5.51	5.36	2.26E-01	4.72E-01
4.43	4.20	4.43	2.26E-01	4.72E-01
8.10	8.19	8.23	2.27E-01	4.72E-01
8.99	8.95	9.08	2.27E-01	4.72E-01
5.13	5.12	5.00	2.27E-01	4.72E-01
4.61	4.83	5.16	2.27E-01	4.72E-01
9.16	9.16	9.19	2.27E-01	4.72E-01
5.17	5.17	5.48	2.27E-01	4.72E-01
7.44	7.38	7.38	2.27E-01	4.72E-01
4.19	4.21	4.25	2.27E-01	4.72E-01
6.43	6.23	6.38	2.27E-01	4.72E-01
4.52	4.40	4.38	2.27E-01	4.72E-01
8.56	8.80	8.69	2.27E-01	4.72E-01
6.11	6.10	6.09	2.27E-01	4.72E-01
4.84	4.70	4.70	2.27E-01	4.72E-01
3.34	3.32	3.46	2.27E-01	4.72E-01
8.94	8.78	8.94	2.27E-01	4.72E-01
8.11	8.07	8.12	2.27E-01	4.72E-01
7.75	7.45	7.55	2.27E-01	4.72E-01
13.24	13.37	13.32	2.27E-01	4.72E-01
9.38	9.70	9.60	2.27E-01	4.72E-01
10.33	10.39	10.36	2.27E-01	4.72E-01
11.48	11.77	11.59	2.27E-01	4.72E-01
7.84	7.85	7.97	2.27E-01	4.72E-01
6.95	6.89	6.94	2.27E-01	4.72E-01
10.23	10.32	10.31	2.27E-01	4.72E-01
5.02	5.09	4.92	2.27E-01	4.72E-01
8.22	8.13	7.99	2.27E-01	4.72E-01
9.33	9.50	9.42	2.27E-01	4.73E-01
9.45	9.62	9.48	2.28E-01	4.73E-01
7.65	7.82	7.84	2.28E-01	4.73E-01
4.47	4.58	4.48	2.28E-01	4.73E-01
5.57	5.61	5.59	2.28E-01	4.73E-01
8.53	8.83	8.65	2.28E-01	4.73E-01
14.93	14.86	14.87	2.28E-01	4.73E-01
9.09	8.91	9.02	2.28E-01	4.73E-01
3.49	3.82	3.74	2.28E-01	4.73E-01
6.47	6.42	6.64	2.28E-01	4.73E-01
11.03	11.11	11.04	2.28E-01	4.73E-01
8.39	8.70	8.60	2.28E-01	4.73E-01
5.78	5.63	5.96	2.28E-01	4.73E-01
6.54	6.58	6.55	2.28E-01	4.73E-01
5.97	5.82	5.95	2.28E-01	4.73E-01
9.56	9.57	9.66	2.28E-01	4.73E-01
5.93	5.83	6.00	2.28E-01	4.73E-01
8.77	8.49	8.62	2.28E-01	4.73E-01

11.35	11.50	11.45	2.28E-01	4.73E-01
3.21	3.18	3.08	2.28E-01	4.73E-01
3.59	3.70	3.57	2.28E-01	4.73E-01
6.41	6.53	6.57	2.29E-01	4.74E-01
5.50	5.06	5.48	2.29E-01	4.74E-01
8.51	8.46	8.42	2.29E-01	4.74E-01
6.49	6.46	6.64	2.29E-01	4.74E-01
6.53	6.59	6.37	2.29E-01	4.74E-01
10.26	10.44	10.29	2.29E-01	4.74E-01
5.80	5.73	5.59	2.29E-01	4.74E-01
3.34	3.41	3.40	2.29E-01	4.74E-01
7.20	6.93	6.93	2.29E-01	4.74E-01
8.76	8.65	8.77	2.29E-01	4.74E-01
7.34	7.19	7.63	2.29E-01	4.74E-01
6.82	6.77	6.62	2.29E-01	4.74E-01
7.69	8.19	8.07	2.29E-01	4.74E-01
5.89	5.77	5.84	2.29E-01	4.74E-01
4.25	4.11	4.12	2.29E-01	4.74E-01
6.91	6.94	7.04	2.29E-01	4.74E-01
5.03	4.97	5.22	2.29E-01	4.74E-01
7.13	6.88	6.80	2.29E-01	4.74E-01
4.80	4.86	4.80	2.29E-01	4.74E-01
9.48	9.30	9.45	2.29E-01	4.74E-01
9.80	9.91	9.76	2.29E-01	4.74E-01
4.32	4.25	3.96	2.29E-01	4.74E-01
9.70	9.65	9.65	2.29E-01	4.74E-01
8.43	8.48	8.41	2.29E-01	4.74E-01
7.63	7.71	7.52	2.30E-01	4.74E-01
10.40	10.31	10.44	2.30E-01	4.74E-01
4.74	5.02	4.83	2.30E-01	4.74E-01
12.70	12.61	12.48	2.30E-01	4.74E-01
3.15	3.30	2.98	2.30E-01	4.74E-01
4.99	5.01	4.93	2.30E-01	4.74E-01
8.42	8.32	8.59	2.30E-01	4.74E-01
6.18	6.29	5.93	2.30E-01	4.74E-01
3.90	3.84	3.88	2.30E-01	4.74E-01
5.45	5.28	5.28	2.30E-01	4.74E-01
8.96	8.72	8.92	2.30E-01	4.74E-01
3.61	3.39	3.62	2.30E-01	4.75E-01
9.05	9.12	9.21	2.30E-01	4.75E-01
10.98	10.83	10.96	2.30E-01	4.75E-01
7.91	7.91	7.93	2.30E-01	4.75E-01
9.44	9.50	9.46	2.30E-01	4.75E-01
6.06	6.12	5.95	2.30E-01	4.75E-01
11.70	11.40	11.63	2.30E-01	4.75E-01
8.64	8.70	8.63	2.30E-01	4.75E-01
5.12	5.25	5.11	2.30E-01	4.75E-01

10.07	10.20	10.06	2.30E-01	4.75E-01
6.80	6.66	6.62	2.30E-01	4.75E-01
4.92	4.72	4.98	2.31E-01	4.75E-01
11.83	11.94	11.97	2.31E-01	4.75E-01
7.68	7.71	7.69	2.31E-01	4.75E-01
8.29	8.38	8.43	2.31E-01	4.75E-01
3.32	3.27	3.21	2.31E-01	4.75E-01
6.18	6.15	6.28	2.31E-01	4.75E-01
5.92	6.01	5.84	2.31E-01	4.75E-01
3.54	3.49	3.54	2.31E-01	4.75E-01
7.02	7.00	7.26	2.31E-01	4.75E-01
5.53	5.61	5.50	2.31E-01	4.75E-01
12.06	12.08	12.10	2.31E-01	4.75E-01
9.83	10.03	9.90	2.31E-01	4.75E-01
7.25	7.25	7.05	2.31E-01	4.75E-01
9.02	9.22	9.21	2.31E-01	4.75E-01
9.13	9.29	9.25	2.31E-01	4.75E-01
9.07	8.87	8.91	2.31E-01	4.75E-01
9.84	9.81	9.84	2.31E-01	4.75E-01
9.68	9.63	9.74	2.31E-01	4.75E-01
6.06	5.96	5.87	2.31E-01	4.75E-01
9.98	9.80	9.68	2.31E-01	4.75E-01
5.18	5.12	4.97	2.31E-01	4.75E-01
8.88	8.97	8.96	2.31E-01	4.75E-01
8.34	8.20	8.14	2.31E-01	4.75E-01
5.73	5.92	5.94	2.32E-01	4.75E-01
10.82	10.95	11.01	2.32E-01	4.75E-01
4.17	4.44	4.30	2.32E-01	4.75E-01
8.97	9.12	8.89	2.32E-01	4.75E-01
6.01	6.03	6.05	2.32E-01	4.75E-01
5.90	5.69	5.85	2.32E-01	4.75E-01
8.30	8.29	8.14	2.32E-01	4.75E-01
3.60	3.53	3.45	2.32E-01	4.75E-01
7.37	6.47	7.22	2.32E-01	4.75E-01
9.60	9.63	9.70	2.32E-01	4.75E-01
4.49	4.30	4.31	2.32E-01	4.75E-01
4.04	3.81	3.86	2.32E-01	4.75E-01
14.40	14.42	14.40	2.32E-01	4.75E-01
4.51	4.37	4.31	2.32E-01	4.75E-01
3.78	3.70	3.72	2.32E-01	4.75E-01
8.78	9.09	8.81	2.32E-01	4.75E-01
6.06	6.18	6.06	2.32E-01	4.75E-01
8.71	8.70	8.92	2.32E-01	4.75E-01
7.78	8.03	7.88	2.32E-01	4.75E-01
8.95	8.93	9.11	2.32E-01	4.75E-01
13.40	13.44	13.40	2.32E-01	4.75E-01
7.05	7.08	7.10	2.32E-01	4.75E-01

10.10	10.23	10.12			2.32E-01	4.75E-01
6.81	6.96	7.00			2.32E-01	4.75E-01
4.92	4.98	4.72			2.32E-01	4.75E-01
7.06	6.94	7.06			2.32E-01	4.75E-01
5.75	5.81	5.61			2.32E-01	4.75E-01
4.25	4.43	4.35			2.32E-01	4.75E-01
10.69	10.65	10.82			2.32E-01	4.75E-01
7.11	7.34	7.11			2.32E-01	4.75E-01
7.12	7.01	6.86			2.32E-01	4.75E-01
8.95	9.39	8.92			2.32E-01	4.75E-01
7.47	7.39	7.39			2.32E-01	4.75E-01
9.67	9.63	9.68			2.32E-01	4.75E-01
8.93	8.91	8.86			2.32E-01	4.75E-01
7.78	8.06	7.97			2.32E-01	4.75E-01
10.41	10.46	10.51			2.32E-01	4.75E-01
10.51	10.57	10.59			2.33E-01	4.75E-01
5.26	5.31	5.27			2.33E-01	4.75E-01
8.46	8.58	8.41			2.33E-01	4.75E-01
8.64	8.43	8.47			2.33E-01	4.75E-01
6.61	6.76	6.68			2.33E-01	4.75E-01
5.41	5.42	5.36			2.33E-01	4.75E-01
4.58	4.59	4.49			2.33E-01	4.75E-01
3.61	3.46	3.55			2.33E-01	4.75E-01
10.04	10.43	10.06			2.33E-01	4.75E-01
6.25	6.40	6.25			2.33E-01	4.75E-01
3.60	3.96	3.81			2.33E-01	4.75E-01
8.60	8.37	8.50			2.33E-01	4.75E-01
6.43	6.10	6.27			2.33E-01	4.75E-01
4.56	4.33	4.27			2.33E-01	4.75E-01
4.99	4.94	4.79			2.33E-01	4.75E-01
5.51	5.50	5.50			2.33E-01	4.75E-01
6.50	6.40	6.41			2.33E-01	4.75E-01
7.92	7.66	7.82			2.33E-01	4.75E-01
7.72	7.53	7.56			2.33E-01	4.75E-01
4.59	4.70	5.00			2.33E-01	4.75E-01
8.05	7.88	8.04			2.33E-01	4.75E-01
10.71	10.64	10.73			2.33E-01	4.75E-01
5.47	5.49	5.46			2.33E-01	4.75E-01
10.46	10.50	10.61			2.33E-01	4.75E-01
9.67	9.52	9.71			2.33E-01	4.75E-01
4.78	4.80	4.71			2.33E-01	4.75E-01
6.27	6.41	6.41			2.33E-01	4.75E-01
4.07	3.83	4.26			2.33E-01	4.75E-01
11.22	10.95	11.16			2.33E-01	4.75E-01
8.36	8.45	8.41			2.33E-01	4.75E-01
3.61	3.76	3.69			2.34E-01	4.76E-01
3.95	4.01	4.00			2.34E-01	4.76E-01

6.32	6.41	6.34	2.34E-01	4.76E-01
7.11	7.11	7.11	2.34E-01	4.76E-01
3.84	3.76	3.57	2.34E-01	4.76E-01
8.38	8.58	8.49	2.34E-01	4.76E-01
10.59	10.37	10.69	2.34E-01	4.76E-01
10.91	10.88	10.87	2.34E-01	4.76E-01
4.46	4.33	4.54	2.34E-01	4.76E-01
9.23	8.79	9.14	2.34E-01	4.76E-01
10.51	9.96	10.50	2.34E-01	4.76E-01
7.25	7.10	7.21	2.34E-01	4.76E-01
7.33	7.44	7.39	2.34E-01	4.76E-01
7.82	7.89	7.98	2.34E-01	4.76E-01
6.91	7.06	6.81	2.34E-01	4.76E-01
10.22	9.84	10.12	2.34E-01	4.76E-01
10.68	10.63	10.74	2.34E-01	4.76E-01
8.54	8.43	8.49	2.34E-01	4.76E-01
9.11	9.31	9.15	2.34E-01	4.76E-01
9.14	9.37	9.18	2.34E-01	4.76E-01
12.18	12.09	12.15	2.34E-01	4.76E-01
3.44	3.44	3.12	2.34E-01	4.76E-01
7.75	7.65	7.68	2.34E-01	4.76E-01
6.75	6.83	6.83	2.34E-01	4.76E-01
8.92	8.41	9.15	2.34E-01	4.76E-01
6.23	6.08	6.18	2.34E-01	4.76E-01
7.10	7.40	7.36	2.34E-01	4.76E-01
9.11	8.97	9.27	2.34E-01	4.76E-01
4.02	3.96	3.91	2.35E-01	4.76E-01
10.03	10.02	9.99	2.35E-01	4.76E-01
8.01	8.33	8.27	2.35E-01	4.76E-01
9.69	9.55	9.74	2.35E-01	4.76E-01
12.00	11.91	12.02	2.35E-01	4.76E-01
13.03	13.09	13.17	2.35E-01	4.76E-01
5.58	5.53	5.62	2.35E-01	4.76E-01
6.32	6.43	6.34	2.35E-01	4.76E-01
6.67	6.45	6.54	2.35E-01	4.77E-01
9.98	10.04	10.14	2.35E-01	4.77E-01
4.38	4.54	4.43	2.35E-01	4.77E-01
8.55	8.57	8.62	2.35E-01	4.77E-01
6.16	6.09	6.43	2.35E-01	4.77E-01
4.30	4.36	4.69	2.35E-01	4.77E-01
13.59	13.58	13.64	2.35E-01	4.77E-01
5.71	5.70	5.71	2.35E-01	4.77E-01
4.59	4.75	4.66	2.35E-01	4.77E-01
4.19	4.14	4.18	2.35E-01	4.77E-01
9.45	9.53	9.48	2.36E-01	4.77E-01
5.06	5.15	5.00	2.36E-01	4.77E-01
4.46	4.44	4.48	2.36E-01	4.77E-01

10.32	10.32	10.29	2.36E-01	4.77E-01
12.28	12.10	12.35	2.36E-01	4.77E-01
4.05	4.10	4.05	2.36E-01	4.77E-01
7.89	7.80	7.81	2.36E-01	4.77E-01
3.81	3.88	4.07	2.36E-01	4.77E-01
7.82	7.77	7.88	2.36E-01	4.77E-01
6.80	7.11	6.70	2.36E-01	4.77E-01
4.95	4.96	5.00	2.36E-01	4.77E-01
3.80	3.70	3.92	2.36E-01	4.77E-01
4.85	4.65	5.49	2.36E-01	4.77E-01
8.84	8.81	8.87	2.36E-01	4.77E-01
3.69	3.70	3.80	2.36E-01	4.77E-01
5.27	5.21	5.08	2.36E-01	4.77E-01
4.12	4.12	4.27	2.36E-01	4.77E-01
10.77	10.50	10.71	2.36E-01	4.78E-01
8.63	8.92	8.70	2.36E-01	4.78E-01
7.61	7.63	7.69	2.36E-01	4.78E-01
5.09	4.64	4.68	2.36E-01	4.78E-01
8.88	8.81	8.78	2.37E-01	4.78E-01
11.47	11.60	11.51	2.37E-01	4.78E-01
9.42	9.31	9.38	2.37E-01	4.78E-01
6.71	6.78	6.77	2.37E-01	4.78E-01
9.34	9.05	9.30	2.37E-01	4.78E-01
12.15	12.28	12.27	2.37E-01	4.78E-01
10.60	10.34	10.53	2.37E-01	4.78E-01
6.43	6.38	6.42	2.37E-01	4.78E-01
6.83	6.90	6.85	2.37E-01	4.78E-01
7.94	8.05	7.94	2.37E-01	4.78E-01
9.17	9.08	9.32	2.37E-01	4.78E-01
7.59	7.82	7.62	2.37E-01	4.78E-01
11.69	11.57	11.67	2.37E-01	4.78E-01
11.86	11.82	11.85	2.37E-01	4.79E-01
7.32	7.36	7.32	2.37E-01	4.79E-01
7.34	7.51	7.14	2.37E-01	4.79E-01
3.99	4.14	4.22	2.38E-01	4.79E-01
4.42	4.28	4.29	2.38E-01	4.79E-01
5.58	5.87	5.70	2.38E-01	4.79E-01
7.41	7.37	7.63	2.38E-01	4.79E-01
9.27	9.31	9.34	2.38E-01	4.79E-01
7.04	6.65	7.06	2.38E-01	4.79E-01
6.35	6.33	6.24	2.38E-01	4.79E-01
10.82	10.75	10.91	2.38E-01	4.79E-01
5.89	6.02	6.00	2.38E-01	4.79E-01
8.22	8.11	8.20	2.38E-01	4.79E-01
6.40	6.42	6.34	2.38E-01	4.79E-01
5.34	4.83	4.93	2.38E-01	4.79E-01
6.41	6.45	6.46	2.38E-01	4.79E-01

3.89	3.56	3.50	2.38E-01	4.79E-01
9.48	9.57	9.61	2.38E-01	4.79E-01
8.20	8.36	8.23	2.38E-01	4.79E-01
4.62	4.41	4.27	2.38E-01	4.79E-01
6.26	6.32	6.23	2.38E-01	4.79E-01
3.60	3.81	3.69	2.38E-01	4.79E-01
9.32	9.38	9.27	2.38E-01	4.79E-01
9.63	9.70	9.68	2.38E-01	4.79E-01
6.58	6.65	6.81	2.38E-01	4.79E-01
5.42	5.20	5.20	2.39E-01	4.79E-01
7.25	7.50	7.32	2.39E-01	4.80E-01
7.92	7.71	7.58	2.39E-01	4.80E-01
7.06	7.12	7.27	2.39E-01	4.80E-01
6.53	6.69	6.69	2.39E-01	4.80E-01
8.60	8.66	8.56	2.39E-01	4.80E-01
6.27	6.23	6.30	2.39E-01	4.80E-01
4.64	4.53	4.45	2.39E-01	4.80E-01
9.58	9.39	9.68	2.39E-01	4.80E-01
5.10	5.11	5.07	2.39E-01	4.80E-01
6.47	6.69	6.76	2.39E-01	4.80E-01
3.08	3.05	3.12	2.39E-01	4.80E-01
5.58	5.50	5.50	2.39E-01	4.80E-01
4.43	4.28	4.22	2.39E-01	4.80E-01
7.73	7.67	7.69	2.39E-01	4.80E-01
9.17	9.20	9.28	2.39E-01	4.80E-01
4.76	4.87	4.70	2.39E-01	4.80E-01
8.79	9.20	8.88	2.39E-01	4.80E-01
5.52	5.20	5.40	2.39E-01	4.80E-01
6.88	6.83	6.79	2.39E-01	4.80E-01
8.43	8.40	8.41	2.40E-01	4.80E-01
9.40	9.53	9.55	2.40E-01	4.80E-01
8.83	9.14	8.91	2.40E-01	4.81E-01
6.30	6.32	6.40	2.40E-01	4.81E-01
7.41	7.54	7.48	2.40E-01	4.81E-01
7.20	7.04	7.10	2.40E-01	4.81E-01
6.71	6.51	6.43	2.40E-01	4.81E-01
8.70	8.77	8.67	2.40E-01	4.81E-01
7.43	7.30	7.56	2.40E-01	4.81E-01
4.95	5.01	4.89	2.40E-01	4.81E-01
6.13	6.02	6.21	2.40E-01	4.81E-01
8.36	8.35	8.44	2.40E-01	4.81E-01
5.09	4.88	4.80	2.40E-01	4.81E-01
8.37	8.33	8.21	2.40E-01	4.81E-01
6.13	6.06	6.06	2.40E-01	4.81E-01
4.84	5.05	4.94	2.40E-01	4.81E-01
8.88	8.91	8.82	2.40E-01	4.81E-01
6.24	6.12	6.21	2.40E-01	4.81E-01

5.79	5.78	5.81	2.40E-01	4.81E-01
10.45	10.30	10.36	2.40E-01	4.81E-01
4.29	4.27	4.28	2.40E-01	4.81E-01
14.21	14.19	14.26	2.40E-01	4.81E-01
8.01	8.19	8.18	2.40E-01	4.81E-01
5.48	5.47	5.56	2.40E-01	4.81E-01
7.92	8.06	8.02	2.41E-01	4.81E-01
3.16	3.24	3.26	2.41E-01	4.81E-01
8.57	8.47	8.63	2.41E-01	4.81E-01
15.18	15.16	15.19	2.41E-01	4.81E-01
9.30	9.39	9.43	2.41E-01	4.81E-01
6.66	6.99	6.88	2.41E-01	4.81E-01
3.54	3.89	3.61	2.41E-01	4.81E-01
4.87	4.65	4.80	2.41E-01	4.81E-01
7.38	7.50	7.33	2.41E-01	4.81E-01
3.74	3.62	3.72	2.41E-01	4.81E-01
7.64	7.63	7.62	2.41E-01	4.81E-01
6.86	6.72	6.61	2.41E-01	4.81E-01
8.60	8.64	8.61	2.41E-01	4.81E-01
4.34	4.27	4.06	2.41E-01	4.81E-01
12.36	12.28	12.39	2.41E-01	4.81E-01
7.17	7.26	7.21	2.41E-01	4.81E-01
8.32	8.27	8.33	2.41E-01	4.81E-01
8.02	7.87	7.95	2.41E-01	4.81E-01
9.72	9.83	9.88	2.41E-01	4.81E-01
5.08	5.09	4.99	2.41E-01	4.81E-01
7.51	7.44	7.55	2.41E-01	4.81E-01
10.28	10.38	10.40	2.41E-01	4.81E-01
11.69	11.81	11.74	2.41E-01	4.81E-01
6.48	6.48	6.46	2.41E-01	4.81E-01
10.24	10.34	10.26	2.41E-01	4.81E-01
5.62	5.85	5.93	2.41E-01	4.81E-01
8.25	8.01	8.18	2.41E-01	4.81E-01
5.91	5.97	5.98	2.42E-01	4.81E-01
8.17	8.11	8.17	2.42E-01	4.81E-01
6.40	6.49	6.43	2.42E-01	4.81E-01
3.64	3.43	3.55	2.42E-01	4.81E-01
6.08	5.78	6.00	2.42E-01	4.81E-01
5.92	6.13	6.03	2.42E-01	4.81E-01
11.32	11.17	11.06	2.42E-01	4.81E-01
6.72	6.66	6.62	2.42E-01	4.81E-01
6.14	6.06	6.05	2.42E-01	4.81E-01
5.37	5.44	5.16	2.42E-01	4.81E-01
12.12	12.11	12.05	2.42E-01	4.81E-01
6.56	6.62	6.57	2.42E-01	4.81E-01
9.59	9.43	9.62	2.42E-01	4.81E-01
8.72	8.91	8.82	2.42E-01	4.81E-01

5.02	5.17	4.89	2.42E-01	4.81E-01
8.78	8.76	8.78	2.42E-01	4.81E-01
10.33	10.28	10.37	2.42E-01	4.81E-01
8.70	8.99	8.82	2.42E-01	4.81E-01
8.65	8.62	8.70	2.42E-01	4.81E-01
9.46	9.43	9.54	2.42E-01	4.81E-01
7.03	7.04	6.88	2.42E-01	4.81E-01
5.88	5.84	5.71	2.42E-01	4.81E-01
7.95	8.01	8.11	2.42E-01	4.82E-01
9.75	9.58	9.70	2.42E-01	4.82E-01
4.17	4.15	4.39	2.42E-01	4.82E-01
6.53	6.53	6.48	2.43E-01	4.82E-01
5.19	5.05	5.07	2.43E-01	4.82E-01
4.41	4.05	4.40	2.43E-01	4.82E-01
3.33	3.26	3.26	2.43E-01	4.82E-01
5.50	5.36	5.50	2.43E-01	4.82E-01
8.56	8.49	8.66	2.43E-01	4.82E-01
3.36	3.25	3.50	2.43E-01	4.82E-01
4.85	4.70	4.82	2.43E-01	4.82E-01
5.51	5.57	5.38	2.43E-01	4.82E-01
9.33	9.55	9.38	2.43E-01	4.82E-01
6.19	6.39	6.39	2.43E-01	4.82E-01
9.17	9.19	9.26	2.43E-01	4.82E-01
4.44	4.56	4.28	2.43E-01	4.82E-01
8.57	8.61	8.68	2.43E-01	4.82E-01
4.84	4.78	4.88	2.43E-01	4.82E-01
10.06	9.91	10.19	2.43E-01	4.82E-01
8.03	7.82	7.89	2.43E-01	4.82E-01
4.76	4.46	4.24	2.43E-01	4.82E-01
11.14	10.97	10.99	2.43E-01	4.82E-01
6.05	6.11	6.13	2.43E-01	4.82E-01
4.15	4.23	4.07	2.43E-01	4.82E-01
3.84	3.74	3.93	2.43E-01	4.82E-01
5.55	5.56	5.41	2.43E-01	4.82E-01
6.61	6.51	6.56	2.43E-01	4.82E-01
4.57	4.48	4.55	2.43E-01	4.82E-01
8.42	8.00	8.08	2.43E-01	4.82E-01
3.91	4.01	3.98	2.44E-01	4.82E-01
11.77	11.56	11.58	2.44E-01	4.82E-01
5.91	5.82	6.06	2.44E-01	4.82E-01
7.45	7.64	7.46	2.44E-01	4.82E-01
11.42	11.25	11.34	2.44E-01	4.83E-01
4.46	4.52	4.33	2.44E-01	4.83E-01
12.36	12.40	12.46	2.44E-01	4.83E-01
10.36	10.45	10.41	2.44E-01	4.83E-01
5.95	5.59	5.71	2.44E-01	4.83E-01
7.23	7.55	7.25	2.44E-01	4.83E-01

5.89	5.52	5.84	2.44E-01	4.83E-01
8.17	8.26	8.32	2.44E-01	4.83E-01
8.92	9.18	9.04	2.44E-01	4.83E-01
7.37	7.34	7.30	2.44E-01	4.83E-01
7.98	8.01	7.94	2.44E-01	4.83E-01
5.83	5.87	5.78	2.44E-01	4.83E-01
4.32	4.38	4.47	2.44E-01	4.83E-01
9.67	9.38	9.60	2.44E-01	4.83E-01
7.77	7.61	7.69	2.44E-01	4.83E-01
5.91	6.12	6.23	2.44E-01	4.83E-01
5.23	5.08	5.04	2.44E-01	4.83E-01
10.08	10.10	10.09	2.44E-01	4.83E-01
8.70	8.72	8.57	2.44E-01	4.83E-01
4.53	4.66	4.49	2.44E-01	4.83E-01
9.86	9.50	9.86	2.44E-01	4.83E-01
6.23	6.20	6.33	2.44E-01	4.83E-01
7.89	7.66	8.03	2.45E-01	4.83E-01
9.36	9.31	9.28	2.45E-01	4.83E-01
6.93	7.30	7.08	2.45E-01	4.83E-01
9.68	9.75	9.74	2.45E-01	4.83E-01
8.66	8.58	8.74	2.45E-01	4.83E-01
6.20	6.23	6.13	2.45E-01	4.83E-01
5.49	5.45	6.04	2.45E-01	4.83E-01
6.73	6.79	6.71	2.45E-01	4.83E-01
6.14	6.25	6.13	2.45E-01	4.83E-01
6.52	6.45	6.24	2.45E-01	4.83E-01
6.59	6.67	6.67	2.45E-01	4.83E-01
8.47	8.67	8.54	2.45E-01	4.83E-01
8.21	8.02	8.17	2.45E-01	4.83E-01
7.99	8.14	8.01	2.45E-01	4.83E-01
3.33	3.33	3.29	2.45E-01	4.83E-01
4.93	4.94	4.70	2.45E-01	4.83E-01
4.26	4.19	4.27	2.45E-01	4.84E-01
5.91	5.82	6.13	2.46E-01	4.84E-01
9.48	9.23	9.37	2.46E-01	4.84E-01
7.58	7.77	7.60	2.46E-01	4.84E-01
7.59	7.73	7.73	2.46E-01	4.84E-01
5.15	5.13	5.16	2.46E-01	4.84E-01
8.32	8.20	8.44	2.46E-01	4.84E-01
9.28	9.22	9.29	2.46E-01	4.84E-01
9.29	9.32	9.29	2.46E-01	4.84E-01
10.43	10.72	10.44	2.46E-01	4.84E-01
5.80	5.84	5.84	2.46E-01	4.84E-01
7.91	7.86	7.84	2.46E-01	4.84E-01
14.12	13.91	14.25	2.46E-01	4.84E-01
4.16	4.24	4.32	2.46E-01	4.84E-01
6.55	6.43	6.42	2.46E-01	4.84E-01

7.15	7.15	7.20	2.46E-01	4.84E-01
8.99	9.11	9.05	2.46E-01	4.84E-01
7.87	8.02	7.83	2.46E-01	4.84E-01
12.18	12.36	12.31	2.46E-01	4.84E-01
4.98	4.93	4.89	2.46E-01	4.84E-01
10.09	10.18	10.20	2.46E-01	4.84E-01
7.88	8.14	7.73	2.46E-01	4.84E-01
6.53	6.41	6.49	2.46E-01	4.84E-01
8.24	8.05	8.14	2.46E-01	4.84E-01
6.69	6.66	6.74	2.46E-01	4.84E-01
11.38	11.57	11.40	2.46E-01	4.84E-01
3.93	4.07	3.83	2.46E-01	4.84E-01
6.43	6.87	6.53	2.46E-01	4.84E-01
6.42	6.55	6.29	2.46E-01	4.84E-01
3.35	3.41	3.44	2.46E-01	4.84E-01
5.50	5.56	5.81	2.46E-01	4.84E-01
11.93	11.73	11.90	2.47E-01	4.84E-01
9.53	9.31	9.45	2.47E-01	4.84E-01
6.21	6.48	6.22	2.47E-01	4.84E-01
6.54	6.63	6.66	2.47E-01	4.84E-01
4.57	4.62	4.38	2.47E-01	4.84E-01
7.90	7.66	7.64	2.47E-01	4.84E-01
12.14	12.18	12.17	2.47E-01	4.84E-01
3.42	3.64	3.36	2.47E-01	4.84E-01
9.80	9.83	9.68	2.47E-01	4.84E-01
6.05	6.03	5.94	2.47E-01	4.84E-01
9.68	10.02	9.63	2.47E-01	4.84E-01
10.33	10.45	10.42	2.47E-01	4.84E-01
4.34	4.31	4.46	2.47E-01	4.84E-01
4.36	4.42	4.45	2.47E-01	4.84E-01
7.47	7.47	7.64	2.47E-01	4.84E-01
4.41	4.37	4.39	2.47E-01	4.84E-01
8.06	7.90	7.93	2.47E-01	4.84E-01
5.78	5.60	5.53	2.47E-01	4.84E-01
6.68	6.68	6.93	2.47E-01	4.84E-01
6.20	6.07	6.22	2.47E-01	4.84E-01
6.87	7.18	7.00	2.48E-01	4.85E-01
14.03	14.11	14.14	2.48E-01	4.85E-01
10.00	9.79	9.90	2.48E-01	4.85E-01
8.77	9.05	8.86	2.48E-01	4.85E-01
6.22	6.01	6.29	2.48E-01	4.85E-01
3.61	3.43	3.68	2.48E-01	4.85E-01
10.57	10.61	10.69	2.48E-01	4.85E-01
8.37	8.39	8.59	2.48E-01	4.85E-01
6.94	6.92	6.96	2.48E-01	4.85E-01
5.66	5.48	5.25	2.48E-01	4.85E-01
3.97	3.56	3.71	2.48E-01	4.85E-01

8.22	8.46	8.20	2.48E-01	4.85E-01
11.60	11.67	11.67	2.48E-01	4.85E-01
3.47	3.52	3.51	2.48E-01	4.85E-01
6.57	6.51	6.47	2.48E-01	4.85E-01
6.99	7.14	7.17	2.48E-01	4.85E-01
10.57	10.74	10.62	2.48E-01	4.85E-01
10.82	10.60	10.76	2.48E-01	4.85E-01
11.90	11.98	12.04	2.48E-01	4.85E-01
8.85	9.17	8.99	2.48E-01	4.85E-01
9.13	9.28	9.32	2.48E-01	4.85E-01
7.52	7.51	7.54	2.48E-01	4.85E-01
3.72	3.52	3.73	2.48E-01	4.85E-01
9.98	9.96	10.20	2.48E-01	4.85E-01
5.76	5.58	5.66	2.48E-01	4.85E-01
6.12	6.05	6.05	2.49E-01	4.85E-01
6.60	6.44	6.50	2.49E-01	4.85E-01
3.70	3.51	3.51	2.49E-01	4.85E-01
10.74	10.91	10.78	2.49E-01	4.85E-01
3.80	4.09	3.98	2.49E-01	4.85E-01
9.61	9.48	9.56	2.49E-01	4.85E-01
11.20	11.38	11.32	2.49E-01	4.85E-01
9.17	9.13	9.05	2.49E-01	4.85E-01
4.64	4.63	4.75	2.49E-01	4.85E-01
9.93	10.31	9.91	2.49E-01	4.85E-01
8.55	8.61	8.67	2.49E-01	4.86E-01
6.06	6.04	5.71	2.49E-01	4.86E-01
10.35	10.53	10.47	2.49E-01	4.86E-01
9.45	9.39	9.57	2.49E-01	4.86E-01
7.84	7.94	7.79	2.49E-01	4.86E-01
11.37	11.61	11.48	2.49E-01	4.86E-01
5.66	5.49	5.59	2.49E-01	4.86E-01
6.85	6.77	6.49	2.49E-01	4.86E-01
5.38	5.58	5.65	2.49E-01	4.86E-01
7.74	7.56	7.66	2.49E-01	4.86E-01
12.39	12.48	12.44	2.49E-01	4.86E-01
5.82	6.01	5.67	2.49E-01	4.86E-01
6.20	6.01	5.96	2.50E-01	4.86E-01
12.45	12.17	12.56	2.50E-01	4.86E-01
4.84	4.70	4.86	2.50E-01	4.86E-01
8.93	9.27	8.95	2.50E-01	4.86E-01
9.86	9.83	9.90	2.50E-01	4.86E-01
4.08	3.78	4.37	2.50E-01	4.86E-01
9.01	9.14	8.94	2.50E-01	4.86E-01
7.42	7.64	7.60	2.50E-01	4.86E-01
6.11	5.98	6.00	2.50E-01	4.86E-01
10.44	10.64	10.66	2.50E-01	4.86E-01
6.56	6.69	6.81	2.50E-01	4.86E-01

8.70	8.84	8.65	2.50E-01	4.86E-01
8.73	9.14	8.85	2.50E-01	4.86E-01
6.02	6.29	6.20	2.50E-01	4.86E-01
4.07	3.76	3.92	2.50E-01	4.86E-01
6.36	6.29	6.34	2.50E-01	4.86E-01
4.08	4.01	4.18	2.50E-01	4.86E-01
8.56	8.23	8.27	2.50E-01	4.86E-01
4.22	4.23	4.40	2.50E-01	4.86E-01
3.50	3.44	3.20	2.50E-01	4.86E-01
5.58	5.41	5.24	2.50E-01	4.87E-01
8.12	8.36	8.20	2.50E-01	4.87E-01
9.97	10.04	10.02	2.50E-01	4.87E-01
4.00	4.16	4.24	2.51E-01	4.87E-01
9.88	10.20	10.05	2.51E-01	4.87E-01
6.71	6.52	6.61	2.51E-01	4.87E-01
9.20	9.21	9.28	2.51E-01	4.87E-01
7.41	7.46	7.49	2.51E-01	4.87E-01
9.76	9.74	9.82	2.51E-01	4.87E-01
6.64	6.53	6.56	2.51E-01	4.87E-01
11.59	11.66	11.72	2.51E-01	4.87E-01
4.41	4.39	4.57	2.51E-01	4.87E-01
6.08	6.10	6.02	2.51E-01	4.87E-01
6.24	6.12	6.33	2.51E-01	4.87E-01
11.39	11.52	11.40	2.51E-01	4.87E-01
7.38	7.15	7.19	2.51E-01	4.87E-01
10.01	9.79	10.01	2.51E-01	4.87E-01
4.38	4.23	4.82	2.51E-01	4.87E-01
6.84	6.61	6.83	2.51E-01	4.87E-01
7.92	7.98	8.01	2.51E-01	4.87E-01
4.39	3.85	3.99	2.51E-01	4.87E-01
7.71	7.63	7.64	2.51E-01	4.87E-01
4.38	4.36	4.30	2.51E-01	4.87E-01
10.25	10.42	10.36	2.51E-01	4.87E-01
3.48	3.72	3.33	2.51E-01	4.87E-01
5.11	5.04	5.17	2.51E-01	4.87E-01
10.43	10.38	10.55	2.51E-01	4.87E-01
8.20	8.20	8.23	2.51E-01	4.87E-01
6.20	5.68	5.91	2.51E-01	4.87E-01
7.76	7.46	7.78	2.51E-01	4.87E-01
3.80	3.86	3.68	2.52E-01	4.87E-01
10.23	10.18	10.24	2.52E-01	4.87E-01
14.30	14.34	14.35	2.52E-01	4.87E-01
8.31	8.20	8.44	2.52E-01	4.87E-01
4.97	4.74	4.84	2.52E-01	4.87E-01
5.87	6.06	6.02	2.52E-01	4.87E-01
6.06	6.17	6.06	2.52E-01	4.87E-01
5.55	5.77	5.80	2.52E-01	4.87E-01

7.67	7.57	7.69	2.52E-01	4.87E-01
8.14	8.16	8.20	2.52E-01	4.87E-01
11.11	11.25	11.07	2.52E-01	4.87E-01
5.63	5.33	5.48	2.52E-01	4.87E-01
6.52	6.59	6.58	2.52E-01	4.87E-01
10.45	10.42	10.54	2.52E-01	4.87E-01
3.63	3.60	3.49	2.52E-01	4.87E-01
4.19	4.14	4.21	2.52E-01	4.87E-01
3.68	3.59	3.51	2.52E-01	4.87E-01
7.20	7.02	7.16	2.52E-01	4.87E-01
7.67	7.66	7.72	2.52E-01	4.87E-01
5.38	5.20	4.87	2.52E-01	4.87E-01
8.86	8.69	8.96	2.52E-01	4.87E-01
10.78	10.81	10.84	2.52E-01	4.87E-01
10.06	10.17	10.08	2.52E-01	4.87E-01
13.42	13.51	13.52	2.52E-01	4.87E-01
6.20	6.16	6.23	2.52E-01	4.87E-01
4.38	4.32	4.14	2.53E-01	4.87E-01
4.24	4.12	4.18	2.53E-01	4.87E-01
8.49	8.33	8.72	2.53E-01	4.87E-01
6.01	5.85	5.84	2.53E-01	4.87E-01
6.33	6.55	6.44	2.53E-01	4.87E-01
5.45	5.54	5.50	2.53E-01	4.87E-01
5.19	5.07	5.13	2.53E-01	4.87E-01
6.08	6.10	6.04	2.53E-01	4.87E-01
8.73	8.75	8.90	2.53E-01	4.87E-01
8.71	8.58	8.59	2.53E-01	4.87E-01
6.44	6.53	6.51	2.53E-01	4.87E-01
9.18	9.27	9.24	2.53E-01	4.87E-01
10.02	10.27	10.14	2.53E-01	4.87E-01
6.49	6.48	6.53	2.53E-01	4.87E-01
7.21	7.28	7.23	2.53E-01	4.87E-01
7.80	7.92	7.82	2.53E-01	4.87E-01
6.32	6.42	6.37	2.53E-01	4.87E-01
7.48	7.78	7.60	2.53E-01	4.87E-01
6.49	6.29	6.29	2.53E-01	4.87E-01
4.68	4.55	4.38	2.53E-01	4.87E-01
4.61	4.43	4.63	2.53E-01	4.87E-01
5.34	5.31	5.11	2.53E-01	4.87E-01
3.77	3.83	3.40	2.53E-01	4.87E-01
7.20	6.96	7.03	2.53E-01	4.87E-01
3.92	3.92	3.51	2.53E-01	4.87E-01
3.74	3.97	4.02	2.53E-01	4.87E-01
4.56	4.65	4.54	2.53E-01	4.87E-01
8.25	8.19	8.33	2.53E-01	4.87E-01
6.32	6.17	6.15	2.53E-01	4.87E-01
6.83	7.04	7.06	2.53E-01	4.87E-01

7.23	6.99	7.25	2.53E-01	4.87E-01
6.56	6.69	6.69	2.53E-01	4.87E-01
7.02	7.06	7.02	2.53E-01	4.87E-01
3.82	3.99	3.56	2.54E-01	4.87E-01
3.96	4.18	4.20	2.54E-01	4.87E-01
7.07	7.16	7.23	2.54E-01	4.87E-01
3.53	3.34	3.36	2.54E-01	4.87E-01
9.30	9.24	9.38	2.54E-01	4.87E-01
3.76	3.86	3.94	2.54E-01	4.87E-01
3.37	3.49	3.29	2.54E-01	4.87E-01
3.89	3.76	3.89	2.54E-01	4.87E-01
3.46	3.73	3.11	2.54E-01	4.87E-01
3.66	3.60	3.56	2.54E-01	4.87E-01
6.61	6.80	6.67	2.54E-01	4.87E-01
11.48	11.76	11.46	2.54E-01	4.87E-01
7.77	7.95	7.80	2.54E-01	4.87E-01
10.10	10.01	10.01	2.54E-01	4.88E-01
9.70	9.80	9.77	2.54E-01	4.88E-01
5.21	5.56	5.17	2.54E-01	4.88E-01
4.55	4.59	4.56	2.54E-01	4.88E-01
3.39	3.50	3.36	2.54E-01	4.88E-01
8.81	8.95	8.92	2.54E-01	4.88E-01
6.07	5.96	6.06	2.54E-01	4.88E-01
5.69	5.55	5.35	2.55E-01	4.88E-01
4.21	4.04	4.31	2.55E-01	4.88E-01
8.15	8.31	8.21	2.55E-01	4.88E-01
7.80	7.68	7.87	2.55E-01	4.88E-01
6.10	6.31	6.39	2.55E-01	4.88E-01
6.10	6.47	6.35	2.55E-01	4.88E-01
4.74	4.70	4.70	2.55E-01	4.88E-01
9.81	10.11	9.96	2.55E-01	4.88E-01
10.52	10.49	10.61	2.55E-01	4.88E-01
3.40	3.37	3.43	2.55E-01	4.88E-01
4.86	4.79	4.70	2.55E-01	4.88E-01
10.10	9.89	9.95	2.55E-01	4.88E-01
12.44	12.41	12.38	2.55E-01	4.88E-01
10.33	10.42	10.33	2.55E-01	4.89E-01
8.21	8.29	8.26	2.55E-01	4.89E-01
7.46	7.83	7.51	2.55E-01	4.89E-01
7.16	7.32	7.25	2.55E-01	4.89E-01
9.37	9.47	9.44	2.55E-01	4.89E-01
9.82	9.90	9.82	2.55E-01	4.89E-01
9.31	9.35	9.34	2.55E-01	4.89E-01
4.40	4.39	4.16	2.55E-01	4.89E-01
5.94	6.18	6.16	2.55E-01	4.89E-01
6.92	6.66	6.88	2.55E-01	4.89E-01
6.89	6.79	7.01	2.56E-01	4.89E-01

6.94	6.78	6.94	2.56E-01	4.89E-01
3.64	3.77	3.56	2.56E-01	4.89E-01
7.69	7.57	7.51	2.56E-01	4.89E-01
3.21	3.41	3.17	2.56E-01	4.89E-01
10.76	10.82	10.84	2.56E-01	4.89E-01
6.03	5.87	6.04	2.56E-01	4.89E-01
5.35	5.32	5.31	2.56E-01	4.89E-01
8.88	8.86	8.98	2.56E-01	4.89E-01
5.53	5.36	5.40	2.56E-01	4.89E-01
9.05	9.10	9.08	2.56E-01	4.90E-01
3.21	3.32	3.39	2.56E-01	4.90E-01
4.37	4.31	4.35	2.56E-01	4.90E-01
9.12	8.79	9.04	2.56E-01	4.90E-01
9.65	9.67	9.57	2.56E-01	4.90E-01
7.20	7.24	7.20	2.56E-01	4.90E-01
10.31	10.19	10.16	2.56E-01	4.90E-01
4.61	4.27	4.31	2.57E-01	4.90E-01
8.36	8.76	8.59	2.57E-01	4.90E-01
7.55	7.46	7.41	2.57E-01	4.90E-01
4.48	4.11	4.55	2.57E-01	4.90E-01
9.19	9.41	9.35	2.57E-01	4.90E-01
12.03	12.11	12.04	2.57E-01	4.90E-01
4.53	4.45	4.50	2.57E-01	4.90E-01
8.55	8.68	8.76	2.57E-01	4.90E-01
5.47	5.77	5.80	2.57E-01	4.90E-01
10.19	10.33	10.24	2.57E-01	4.90E-01
3.56	3.68	3.37	2.57E-01	4.90E-01
11.80	11.76	11.74	2.57E-01	4.90E-01
7.78	8.20	7.82	2.57E-01	4.90E-01
8.26	8.34	8.46	2.57E-01	4.90E-01
4.69	4.48	4.45	2.57E-01	4.90E-01
7.22	7.19	7.26	2.57E-01	4.90E-01
8.21	8.13	8.26	2.57E-01	4.90E-01
4.57	4.54	4.62	2.57E-01	4.90E-01
3.36	3.45	3.42	2.57E-01	4.90E-01
4.81	4.87	4.84	2.57E-01	4.90E-01
6.96	6.75	6.95	2.57E-01	4.90E-01
10.08	9.98	10.11	2.57E-01	4.90E-01
4.20	3.86	3.88	2.57E-01	4.90E-01
8.02	7.85	8.13	2.57E-01	4.90E-01
6.71	6.90	7.05	2.57E-01	4.90E-01
6.51	6.45	6.43	2.57E-01	4.90E-01
4.95	4.87	4.46	2.57E-01	4.90E-01
10.44	9.95	10.32	2.58E-01	4.90E-01
3.37	3.16	3.26	2.58E-01	4.91E-01
6.68	6.83	6.64	2.58E-01	4.91E-01
9.23	9.43	9.26	2.58E-01	4.91E-01

6.84	6.92	6.84	2.58E-01	4.91E-01
8.15	8.44	8.16	2.58E-01	4.91E-01
9.02	8.77	8.99	2.58E-01	4.91E-01
6.16	6.10	6.30	2.58E-01	4.91E-01
9.64	9.40	9.64	2.58E-01	4.91E-01
6.83	7.19	6.88	2.58E-01	4.91E-01
7.05	7.11	7.16	2.58E-01	4.91E-01
9.75	9.79	9.75	2.58E-01	4.91E-01
9.98	10.09	10.00	2.58E-01	4.91E-01
3.66	3.88	3.66	2.58E-01	4.91E-01
11.44	11.69	11.61	2.58E-01	4.91E-01
14.19	14.17	14.24	2.58E-01	4.91E-01
8.40	7.96	8.25	2.58E-01	4.91E-01
8.19	8.09	8.15	2.59E-01	4.91E-01
7.87	7.77	7.96	2.59E-01	4.91E-01
3.16	3.38	3.20	2.59E-01	4.91E-01
10.86	10.82	10.78	2.59E-01	4.91E-01
6.83	6.61	6.74	2.59E-01	4.91E-01
6.04	6.11	5.89	2.59E-01	4.91E-01
9.94	9.87	9.91	2.59E-01	4.91E-01
3.62	3.55	3.38	2.59E-01	4.91E-01
6.66	6.77	6.45	2.59E-01	4.91E-01
10.32	10.57	10.45	2.59E-01	4.91E-01
10.21	10.12	10.15	2.59E-01	4.91E-01
4.82	4.88	4.82	2.59E-01	4.91E-01
3.99	3.95	3.96	2.59E-01	4.91E-01
5.25	5.21	5.22	2.59E-01	4.91E-01
5.71	5.81	5.89	2.59E-01	4.91E-01
7.07	6.90	7.14	2.59E-01	4.91E-01
5.62	5.48	5.68	2.59E-01	4.91E-01
9.39	9.14	9.10	2.59E-01	4.91E-01
7.21	7.25	7.42	2.59E-01	4.91E-01
6.88	6.54	6.65	2.59E-01	4.91E-01
3.14	3.10	3.09	2.59E-01	4.91E-01
8.18	8.11	8.23	2.59E-01	4.91E-01
6.99	6.80	6.78	2.59E-01	4.91E-01
5.89	6.14	5.90	2.59E-01	4.91E-01
4.49	4.14	4.43	2.59E-01	4.91E-01
8.47	8.13	8.41	2.59E-01	4.91E-01
8.05	7.90	8.08	2.59E-01	4.91E-01
3.45	3.61	3.53	2.59E-01	4.91E-01
3.13	3.30	3.24	2.60E-01	4.92E-01
7.12	6.95	7.19	2.60E-01	4.92E-01
5.36	5.22	5.42	2.60E-01	4.92E-01
7.10	7.31	7.07	2.60E-01	4.92E-01
11.33	11.36	11.29	2.60E-01	4.92E-01
12.02	11.96	12.15	2.60E-01	4.92E-01

3.87	3.63	3.60	2.60E-01	4.92E-01
6.04	6.09	6.00	2.60E-01	4.92E-01
9.64	9.74	9.64	2.60E-01	4.92E-01
5.71	5.66	6.00	2.60E-01	4.92E-01
4.42	4.27	4.44	2.60E-01	4.92E-01
7.13	7.15	7.03	2.60E-01	4.92E-01
4.24	4.01	4.37	2.60E-01	4.92E-01
7.84	7.85	7.96	2.60E-01	4.92E-01
11.40	11.44	11.46	2.60E-01	4.92E-01
5.10	4.75	5.07	2.60E-01	4.92E-01
7.70	7.88	7.73	2.60E-01	4.92E-01
3.34	3.28	3.35	2.60E-01	4.92E-01
8.65	8.44	8.91	2.60E-01	4.92E-01
13.25	13.14	13.18	2.60E-01	4.92E-01
9.28	9.46	9.46	2.60E-01	4.92E-01
5.13	5.15	4.95	2.61E-01	4.92E-01
5.57	5.74	5.78	2.61E-01	4.92E-01
5.31	5.42	5.23	2.61E-01	4.92E-01
3.29	3.39	3.22	2.61E-01	4.92E-01
4.59	4.70	4.90	2.61E-01	4.92E-01
8.20	8.45	8.18	2.61E-01	4.92E-01
9.93	10.22	10.01	2.61E-01	4.92E-01
3.22	3.38	3.34	2.61E-01	4.92E-01
10.93	10.89	10.91	2.61E-01	4.92E-01
5.62	5.71	5.39	2.61E-01	4.92E-01
5.22	5.07	4.94	2.61E-01	4.92E-01
10.32	9.95	10.19	2.61E-01	4.92E-01
4.48	4.50	4.63	2.61E-01	4.92E-01
4.96	4.99	5.01	2.61E-01	4.92E-01
4.02	4.25	4.18	2.61E-01	4.92E-01
8.18	8.29	8.18	2.61E-01	4.92E-01
8.75	8.79	8.78	2.61E-01	4.92E-01
7.82	7.76	7.91	2.61E-01	4.92E-01
6.45	6.37	6.38	2.61E-01	4.92E-01
5.39	5.46	5.33	2.61E-01	4.92E-01
7.28	7.11	7.24	2.61E-01	4.92E-01
5.75	6.00	5.91	2.61E-01	4.92E-01
9.18	9.02	9.25	2.61E-01	4.92E-01
9.45	9.50	9.57	2.61E-01	4.92E-01
9.99	10.03	10.23	2.61E-01	4.92E-01
7.74	7.37	7.68	2.61E-01	4.92E-01
10.26	10.58	10.41	2.62E-01	4.93E-01
5.12	5.11	4.95	2.62E-01	4.93E-01
9.71	9.89	9.77	2.62E-01	4.93E-01
3.68	3.69	3.60	2.62E-01	4.93E-01
5.54	5.61	5.56	2.62E-01	4.93E-01
10.16	10.42	10.28	2.62E-01	4.93E-01

5.93	5.91	5.92	2.62E-01	4.93E-01
6.86	6.78	6.73	2.62E-01	4.93E-01
8.54	8.38	8.76	2.62E-01	4.93E-01
4.03	4.04	4.07	2.62E-01	4.93E-01
10.50	10.40	10.50	2.62E-01	4.93E-01
9.76	9.57	9.71	2.62E-01	4.93E-01
4.10	4.02	3.80	2.62E-01	4.93E-01
5.52	5.23	5.25	2.62E-01	4.93E-01
6.84	7.06	7.00	2.62E-01	4.93E-01
8.83	8.57	8.82	2.62E-01	4.93E-01
7.22	7.12	6.95	2.62E-01	4.93E-01
5.63	5.43	5.68	2.62E-01	4.93E-01
9.67	9.81	9.78	2.62E-01	4.93E-01
5.24	5.05	5.09	2.62E-01	4.93E-01
9.51	9.85	9.75	2.62E-01	4.93E-01
5.05	4.84	4.99	2.62E-01	4.93E-01
4.18	4.06	4.29	2.62E-01	4.93E-01
5.97	6.00	6.10	2.63E-01	4.93E-01
8.08	8.30	8.10	2.63E-01	4.93E-01
4.03	4.05	3.98	2.63E-01	4.93E-01
3.05	3.34	3.15	2.63E-01	4.93E-01
3.78	3.98	3.94	2.63E-01	4.93E-01
12.23	12.32	12.26	2.63E-01	4.93E-01
5.90	5.98	6.06	2.63E-01	4.93E-01
7.41	7.48	7.21	2.63E-01	4.93E-01
5.77	5.51	5.41	2.63E-01	4.93E-01
8.14	7.94	8.06	2.63E-01	4.93E-01
7.93	7.88	7.85	2.63E-01	4.93E-01
6.84	7.02	6.95	2.63E-01	4.93E-01
4.08	4.22	4.18	2.63E-01	4.93E-01
6.30	6.37	6.31	2.63E-01	4.94E-01
11.63	11.78	11.65	2.63E-01	4.94E-01
10.03	9.70	10.26	2.63E-01	4.94E-01
4.56	4.55	4.44	2.63E-01	4.94E-01
5.64	5.64	5.55	2.63E-01	4.94E-01
4.63	4.71	4.57	2.63E-01	4.94E-01
9.09	8.80	8.90	2.63E-01	4.94E-01
11.83	11.79	11.75	2.63E-01	4.94E-01
6.82	6.40	6.68	2.64E-01	4.94E-01
9.17	8.95	9.06	2.64E-01	4.94E-01
5.66	5.78	5.90	2.64E-01	4.94E-01
3.62	3.83	3.70	2.64E-01	4.94E-01
4.18	4.10	4.12	2.64E-01	4.94E-01
7.24	7.35	7.30	2.64E-01	4.94E-01
8.00	8.01	8.04	2.64E-01	4.94E-01
7.79	7.97	7.76	2.64E-01	4.94E-01
5.08	5.10	5.05	2.64E-01	4.94E-01

7.53	7.72	7.80	2.64E-01	4.94E-01
6.26	6.14	6.35	2.64E-01	4.94E-01
4.38	4.58	4.58	2.64E-01	4.94E-01
10.85	10.85	10.97	2.64E-01	4.94E-01
4.50	4.55	4.55	2.64E-01	4.94E-01
6.56	6.72	6.65	2.64E-01	4.94E-01
4.47	4.52	4.48	2.64E-01	4.94E-01
8.10	7.92	8.19	2.64E-01	4.94E-01
8.54	8.34	8.55	2.64E-01	4.94E-01
8.83	8.68	8.95	2.64E-01	4.94E-01
8.33	8.37	8.26	2.64E-01	4.94E-01
6.59	6.60	6.60	2.64E-01	4.94E-01
8.31	8.12	8.43	2.64E-01	4.94E-01
8.79	8.79	8.92	2.65E-01	4.94E-01
8.94	9.15	8.90	2.65E-01	4.94E-01
4.20	4.23	4.08	2.65E-01	4.94E-01
5.50	5.67	5.65	2.65E-01	4.94E-01
13.47	13.38	13.43	2.65E-01	4.95E-01
7.30	7.13	7.19	2.65E-01	4.95E-01
9.39	9.52	9.47	2.65E-01	4.95E-01
8.67	8.65	8.70	2.65E-01	4.95E-01
7.77	8.10	8.05	2.65E-01	4.95E-01
9.77	10.14	9.86	2.65E-01	4.95E-01
11.80	11.65	11.88	2.65E-01	4.95E-01
9.91	9.73	9.89	2.66E-01	4.96E-01
8.19	8.23	8.28	2.66E-01	4.96E-01
5.13	5.43	5.20	2.66E-01	4.96E-01
9.47	9.59	9.43	2.66E-01	4.96E-01
4.89	4.57	4.61	2.66E-01	4.96E-01
9.60	9.66	9.70	2.66E-01	4.96E-01
10.93	11.37	10.87	2.66E-01	4.96E-01
3.99	3.90	4.05	2.66E-01	4.96E-01
14.48	14.47	14.49	2.66E-01	4.96E-01
9.36	9.53	9.38	2.66E-01	4.96E-01
7.98	8.12	8.16	2.66E-01	4.96E-01
4.83	5.00	4.93	2.66E-01	4.96E-01
10.01	10.18	10.06	2.66E-01	4.96E-01
7.88	8.06	7.89	2.66E-01	4.96E-01
3.46	3.34	3.43	2.66E-01	4.96E-01
5.65	5.51	5.67	2.66E-01	4.96E-01
10.69	10.68	10.75	2.66E-01	4.96E-01
5.17	4.88	5.03	2.66E-01	4.96E-01
9.86	9.95	9.91	2.67E-01	4.97E-01
13.42	13.31	13.43	2.67E-01	4.97E-01
3.82	3.80	3.80	2.67E-01	4.97E-01
7.17	7.32	7.43	2.67E-01	4.97E-01
5.25	5.36	5.31	2.67E-01	4.97E-01

7.55	7.75	7.58	2.67E-01	4.97E-01
7.00	7.25	7.16	2.67E-01	4.97E-01
6.09	5.94	5.98	2.67E-01	4.97E-01
7.12	7.19	7.42	2.67E-01	4.97E-01
10.86	10.63	10.66	2.67E-01	4.97E-01
3.72	3.80	3.75	2.67E-01	4.97E-01
10.28	10.18	10.23	2.67E-01	4.97E-01
6.49	6.29	6.76	2.67E-01	4.97E-01
4.55	4.66	4.37	2.67E-01	4.97E-01
5.26	5.11	5.19	2.67E-01	4.97E-01
6.70	6.97	6.58	2.67E-01	4.97E-01
4.67	4.76	4.52	2.67E-01	4.97E-01
9.36	9.47	9.48	2.67E-01	4.97E-01
7.04	6.84	6.96	2.67E-01	4.97E-01
3.91	3.56	3.45	2.68E-01	4.97E-01
4.40	4.50	4.22	2.68E-01	4.97E-01
3.62	3.70	3.56	2.68E-01	4.98E-01
6.49	6.49	6.49	2.68E-01	4.98E-01
9.11	8.66	9.13	2.68E-01	4.98E-01
7.19	7.19	7.33	2.68E-01	4.98E-01
6.28	5.95	6.33	2.68E-01	4.98E-01
4.83	4.76	4.69	2.68E-01	4.98E-01
9.26	9.40	9.27	2.68E-01	4.98E-01
10.40	10.06	10.28	2.68E-01	4.98E-01
6.88	7.11	6.99	2.68E-01	4.98E-01
7.25	7.13	7.13	2.68E-01	4.98E-01
8.16	7.98	7.95	2.68E-01	4.98E-01
6.43	6.75	6.35	2.68E-01	4.98E-01
5.61	5.39	5.44	2.68E-01	4.98E-01
5.42	5.49	5.87	2.68E-01	4.98E-01
7.83	7.83	8.04	2.69E-01	4.98E-01
6.08	5.85	5.87	2.69E-01	4.98E-01
8.18	8.35	8.21	2.69E-01	4.98E-01
4.03	4.09	3.91	2.69E-01	4.98E-01
12.34	12.48	12.34	2.69E-01	4.98E-01
9.94	10.18	9.82	2.69E-01	4.98E-01
3.68	3.74	3.45	2.69E-01	4.98E-01
5.02	4.63	4.80	2.69E-01	4.98E-01
9.17	9.41	9.44	2.69E-01	4.98E-01
8.30	8.42	8.35	2.69E-01	4.98E-01
3.74	3.77	3.76	2.69E-01	4.98E-01
7.07	7.38	7.40	2.69E-01	4.98E-01
11.11	10.73	10.87	2.69E-01	4.99E-01
6.08	6.12	6.02	2.69E-01	4.99E-01
6.39	6.36	6.37	2.69E-01	4.99E-01
7.59	7.54	7.54	2.69E-01	4.99E-01
5.20	5.39	5.51	2.69E-01	4.99E-01

8.37	8.32	8.42	2.69E-01	4.99E-01
4.54	4.30	4.03	2.69E-01	4.99E-01
5.82	5.90	6.06	2.69E-01	4.99E-01
3.34	3.38	3.23	2.69E-01	4.99E-01
8.12	8.22	8.11	2.69E-01	4.99E-01
3.78	3.85	3.69	2.69E-01	4.99E-01
7.30	7.48	7.34	2.69E-01	4.99E-01
5.69	5.58	5.44	2.70E-01	4.99E-01
3.33	3.48	3.39	2.70E-01	4.99E-01
3.74	3.68	3.67	2.70E-01	4.99E-01
4.22	4.10	4.38	2.70E-01	4.99E-01
3.18	3.29	3.36	2.70E-01	4.99E-01
5.19	5.22	5.43	2.70E-01	4.99E-01
4.10	3.91	4.32	2.70E-01	4.99E-01
6.86	6.80	6.68	2.70E-01	4.99E-01
8.57	8.45	8.59	2.70E-01	4.99E-01
4.38	4.53	4.08	2.70E-01	4.99E-01
2.84	2.88	3.05	2.70E-01	4.99E-01
8.12	8.22	8.08	2.70E-01	4.99E-01
3.65	3.55	3.65	2.70E-01	4.99E-01
5.75	5.53	5.55	2.70E-01	4.99E-01
6.53	6.62	6.39	2.70E-01	4.99E-01
8.17	8.40	8.33	2.70E-01	4.99E-01
10.30	10.45	10.27	2.70E-01	4.99E-01
5.19	5.14	5.13	2.70E-01	4.99E-01
6.73	6.76	7.00	2.70E-01	4.99E-01
7.16	7.30	7.16	2.70E-01	4.99E-01
5.18	5.05	5.37	2.70E-01	4.99E-01
3.88	3.44	3.83	2.70E-01	4.99E-01
8.33	8.64	8.42	2.70E-01	4.99E-01
7.87	7.92	8.05	2.70E-01	4.99E-01
3.84	3.75	3.89	2.70E-01	4.99E-01
11.56	11.51	11.71	2.70E-01	4.99E-01
4.50	4.32	4.35	2.70E-01	4.99E-01
5.04	5.03	5.27	2.71E-01	4.99E-01
8.53	8.72	8.73	2.71E-01	4.99E-01
3.97	4.03	3.97	2.71E-01	4.99E-01
9.87	9.51	9.67	2.71E-01	4.99E-01
3.47	3.30	3.56	2.71E-01	4.99E-01
4.32	4.52	4.40	2.71E-01	4.99E-01
8.20	8.14	8.19	2.71E-01	4.99E-01
6.90	6.76	6.70	2.71E-01	4.99E-01
8.18	7.90	7.95	2.71E-01	4.99E-01
11.28	11.05	11.18	2.71E-01	4.99E-01
6.36	6.30	6.36	2.71E-01	4.99E-01
10.57	10.26	10.70	2.71E-01	4.99E-01
6.31	6.37	6.42	2.71E-01	4.99E-01

8.86	8.92	8.90	2.71E-01	4.99E-01
6.27	6.31	6.40	2.71E-01	4.99E-01
13.24	13.40	13.27	2.71E-01	4.99E-01
11.78	11.82	11.77	2.71E-01	4.99E-01
4.33	4.42	4.01	2.71E-01	4.99E-01
5.05	5.15	5.00	2.71E-01	4.99E-01
6.53	6.57	6.59	2.71E-01	4.99E-01
9.40	9.52	9.44	2.71E-01	4.99E-01
9.64	9.62	9.64	2.71E-01	4.99E-01
8.39	8.44	8.45	2.71E-01	4.99E-01
5.16	5.14	5.11	2.71E-01	4.99E-01
6.44	6.25	6.23	2.71E-01	4.99E-01
6.57	6.54	6.74	2.72E-01	4.99E-01
6.15	6.41	6.29	2.72E-01	4.99E-01
7.27	7.40	7.15	2.72E-01	4.99E-01
4.65	4.48	4.62	2.72E-01	4.99E-01
14.24	14.19	14.26	2.72E-01	4.99E-01
7.39	7.57	7.53	2.72E-01	4.99E-01
7.23	7.01	7.31	2.72E-01	4.99E-01
3.39	3.34	3.16	2.72E-01	4.99E-01
8.54	8.64	8.65	2.72E-01	4.99E-01
8.69	8.78	8.64	2.72E-01	4.99E-01
3.73	3.58	3.65	2.72E-01	4.99E-01
7.21	7.37	7.16	2.72E-01	4.99E-01
4.50	4.50	4.53	2.72E-01	5.00E-01
9.13	8.94	9.19	2.72E-01	5.00E-01
5.43	5.52	5.59	2.72E-01	5.00E-01
7.50	7.62	7.58	2.72E-01	5.00E-01
4.30	4.02	4.33	2.72E-01	5.00E-01
12.07	12.08	12.00	2.72E-01	5.00E-01
5.24	5.01	5.11	2.72E-01	5.00E-01
8.32	8.28	8.19	2.72E-01	5.00E-01
8.43	8.70	8.58	2.72E-01	5.00E-01
8.63	8.43	8.67	2.72E-01	5.00E-01
5.08	5.07	5.12	2.72E-01	5.00E-01
4.43	4.18	4.16	2.72E-01	5.00E-01
6.67	6.93	6.81	2.72E-01	5.00E-01
15.17	15.18	15.19	2.72E-01	5.00E-01
3.79	3.67	3.77	2.73E-01	5.00E-01
6.85	6.81	6.78	2.73E-01	5.00E-01
12.55	12.44	12.49	2.73E-01	5.00E-01
3.11	3.30	3.19	2.73E-01	5.00E-01
8.93	9.05	9.00	2.73E-01	5.00E-01
9.21	9.07	9.21	2.73E-01	5.00E-01
9.18	9.24	9.13	2.73E-01	5.00E-01
8.15	8.10	8.17	2.73E-01	5.00E-01
10.62	10.87	10.70	2.73E-01	5.00E-01

3.23	3.22	3.05	2.73E-01	5.00E-01
7.89	7.99	7.88	2.73E-01	5.00E-01
6.99	7.00	6.88	2.73E-01	5.00E-01
7.46	7.49	7.58	2.73E-01	5.00E-01
7.71	7.86	7.77	2.73E-01	5.00E-01
6.52	6.52	6.55	2.73E-01	5.00E-01
7.82	7.87	7.72	2.73E-01	5.00E-01
3.47	3.28	3.36	2.73E-01	5.00E-01
6.23	5.92	6.19	2.73E-01	5.00E-01
6.93	7.09	6.86	2.73E-01	5.00E-01
10.62	10.75	10.63	2.73E-01	5.00E-01
6.23	6.25	6.50	2.74E-01	5.01E-01
3.73	3.63	3.76	2.74E-01	5.01E-01
7.85	7.79	7.85	2.74E-01	5.01E-01
7.37	7.35	7.36	2.74E-01	5.01E-01
9.82	9.81	9.87	2.74E-01	5.01E-01
11.31	11.14	11.30	2.74E-01	5.01E-01
5.31	5.23	5.28	2.74E-01	5.01E-01
7.57	7.92	7.78	2.74E-01	5.01E-01
6.93	7.27	7.14	2.74E-01	5.01E-01
12.89	13.03	12.98	2.74E-01	5.01E-01
7.17	6.92	6.98	2.74E-01	5.01E-01
5.59	5.59	5.38	2.74E-01	5.01E-01
6.84	6.89	7.06	2.74E-01	5.01E-01
7.83	7.88	8.00	2.74E-01	5.01E-01
7.02	7.16	7.05	2.74E-01	5.01E-01
6.55	6.43	6.61	2.74E-01	5.01E-01
10.03	10.18	9.98	2.74E-01	5.01E-01
4.42	3.93	4.08	2.74E-01	5.01E-01
9.42	9.53	9.51	2.74E-01	5.01E-01
9.23	9.12	9.47	2.74E-01	5.01E-01
6.52	6.39	6.60	2.75E-01	5.01E-01
3.39	3.65	3.34	2.75E-01	5.01E-01
6.72	6.65	6.43	2.75E-01	5.01E-01
8.82	8.73	8.87	2.75E-01	5.01E-01
8.10	8.13	8.28	2.75E-01	5.01E-01
3.90	3.81	4.06	2.75E-01	5.01E-01
5.29	5.24	5.54	2.75E-01	5.01E-01
8.08	8.38	8.40	2.75E-01	5.01E-01
9.09	9.00	8.95	2.75E-01	5.01E-01
11.23	11.51	11.32	2.75E-01	5.02E-01
7.68	9.22	7.59	2.75E-01	5.02E-01
8.87	9.05	9.07	2.75E-01	5.02E-01
5.13	5.24	5.14	2.75E-01	5.02E-01
6.38	6.54	6.57	2.75E-01	5.02E-01
5.87	5.90	5.94	2.75E-01	5.02E-01
14.73	14.69	14.70	2.75E-01	5.02E-01

6.48	6.28	6.41	2.75E-01	5.02E-01
5.43	5.17	5.16	2.75E-01	5.02E-01
9.94	9.75	9.82	2.75E-01	5.02E-01
10.00	10.19	10.10	2.75E-01	5.02E-01
3.95	3.82	3.91	2.75E-01	5.02E-01
3.30	3.20	3.35	2.75E-01	5.02E-01
7.32	7.18	7.27	2.76E-01	5.02E-01
14.62	14.61	14.61	2.76E-01	5.02E-01
10.70	10.78	10.81	2.76E-01	5.02E-01
11.86	12.02	11.94	2.76E-01	5.02E-01
8.54	8.77	8.52	2.76E-01	5.02E-01
4.99	5.07	4.91	2.76E-01	5.02E-01
7.88	7.72	7.80	2.76E-01	5.02E-01
11.26	11.40	11.35	2.76E-01	5.02E-01
14.20	14.14	14.21	2.76E-01	5.02E-01
8.80	8.50	8.93	2.76E-01	5.02E-01
4.47	4.21	4.37	2.76E-01	5.02E-01
6.63	6.44	6.51	2.76E-01	5.02E-01
6.59	6.49	6.61	2.76E-01	5.02E-01
6.33	6.42	6.33	2.76E-01	5.02E-01
5.02	4.86	4.74	2.76E-01	5.02E-01
8.74	8.71	8.78	2.76E-01	5.02E-01
7.71	7.59	7.71	2.76E-01	5.02E-01
11.78	11.66	11.83	2.76E-01	5.02E-01
6.93	7.33	7.05	2.76E-01	5.02E-01
8.45	8.53	8.33	2.76E-01	5.02E-01
9.41	9.23	9.33	2.76E-01	5.02E-01
7.36	7.54	7.59	2.76E-01	5.02E-01
5.31	5.28	5.43	2.76E-01	5.02E-01
6.06	6.02	5.77	2.76E-01	5.02E-01
6.34	6.48	6.24	2.76E-01	5.02E-01
6.94	6.73	6.82	2.76E-01	5.02E-01
8.95	9.03	9.04	2.76E-01	5.02E-01
7.25	7.05	7.21	2.76E-01	5.02E-01
5.25	4.80	4.92	2.76E-01	5.02E-01
10.02	9.81	10.06	2.77E-01	5.02E-01
4.15	3.81	4.05	2.77E-01	5.02E-01
6.69	6.77	6.64	2.77E-01	5.02E-01
11.40	11.17	11.34	2.77E-01	5.02E-01
10.05	10.33	10.14	2.77E-01	5.02E-01
8.13	8.33	8.28	2.77E-01	5.02E-01
7.71	7.59	7.62	2.77E-01	5.02E-01
8.43	8.60	8.59	2.77E-01	5.02E-01
4.34	4.35	4.36	2.77E-01	5.02E-01
7.98	7.56	7.66	2.77E-01	5.02E-01
4.79	5.01	4.69	2.77E-01	5.02E-01
7.68	7.56	7.84	2.77E-01	5.02E-01

7.20	7.32	7.32	2.77E-01	5.02E-01
5.47	5.43	5.62	2.77E-01	5.03E-01
6.37	6.50	6.50	2.77E-01	5.03E-01
4.99	4.73	4.73	2.77E-01	5.03E-01
8.42	8.78	8.67	2.77E-01	5.03E-01
4.55	4.35	4.34	2.77E-01	5.03E-01
9.79	9.73	9.76	2.78E-01	5.03E-01
12.44	12.55	12.50	2.78E-01	5.03E-01
5.18	5.09	5.09	2.78E-01	5.03E-01
5.56	5.53	5.36	2.78E-01	5.03E-01
3.72	3.80	3.70	2.78E-01	5.03E-01
3.73	3.65	3.86	2.78E-01	5.03E-01
7.33	7.20	7.33	2.78E-01	5.03E-01
3.12	3.10	3.00	2.78E-01	5.03E-01
4.56	4.58	4.81	2.78E-01	5.04E-01
6.92	6.65	7.06	2.78E-01	5.04E-01
6.87	6.83	6.78	2.78E-01	5.04E-01
6.38	6.22	6.22	2.78E-01	5.04E-01
5.22	5.23	5.29	2.78E-01	5.04E-01
3.14	3.41	3.25	2.78E-01	5.04E-01
5.66	5.79	5.96	2.79E-01	5.04E-01
11.58	11.83	11.70	2.79E-01	5.04E-01
8.82	9.09	8.93	2.79E-01	5.04E-01
6.65	6.61	6.57	2.79E-01	5.04E-01
4.15	3.99	4.12	2.79E-01	5.04E-01
8.31	8.18	8.35	2.79E-01	5.04E-01
6.17	6.13	6.46	2.79E-01	5.04E-01
9.52	9.68	9.69	2.79E-01	5.04E-01
4.75	4.90	4.55	2.79E-01	5.04E-01
5.10	4.93	4.69	2.79E-01	5.04E-01
5.31	5.20	5.25	2.79E-01	5.04E-01
7.93	8.17	7.99	2.79E-01	5.04E-01
8.61	8.72	8.78	2.79E-01	5.04E-01
4.12	3.88	4.23	2.79E-01	5.04E-01
5.22	5.29	5.54	2.79E-01	5.04E-01
9.61	9.41	9.50	2.79E-01	5.04E-01
5.87	5.73	5.75	2.79E-01	5.05E-01
6.03	5.85	5.98	2.79E-01	5.05E-01
6.98	6.84	6.97	2.79E-01	5.05E-01
4.38	4.58	5.07	2.79E-01	5.05E-01
6.51	6.44	6.57	2.80E-01	5.05E-01
6.51	6.31	6.35	2.80E-01	5.05E-01
5.37	5.56	5.43	2.80E-01	5.05E-01
7.09	6.78	6.96	2.80E-01	5.05E-01
5.77	5.60	5.61	2.80E-01	5.05E-01
12.25	12.55	12.32	2.80E-01	5.05E-01
11.12	11.39	10.96	2.80E-01	5.05E-01

4.28	4.39	4.40	2.80E-01	5.05E-01
3.29	3.49	3.28	2.80E-01	5.05E-01
10.71	10.43	10.62	2.80E-01	5.05E-01
9.55	9.56	9.55	2.80E-01	5.05E-01
7.50	7.80	7.64	2.80E-01	5.05E-01
5.58	5.74	5.67	2.80E-01	5.05E-01
8.45	8.20	7.89	2.80E-01	5.05E-01
8.45	8.47	8.57	2.80E-01	5.05E-01
4.89	5.28	5.05	2.80E-01	5.05E-01
3.28	3.31	3.50	2.80E-01	5.05E-01
5.66	5.46	5.36	2.80E-01	5.05E-01
6.19	6.27	6.15	2.80E-01	5.05E-01
5.81	5.98	6.09	2.80E-01	5.05E-01
7.93	8.06	7.98	2.80E-01	5.05E-01
8.19	8.10	8.20	2.81E-01	5.05E-01
7.33	6.91	7.08	2.81E-01	5.05E-01
7.77	7.66	7.73	2.81E-01	5.05E-01
6.31	6.40	6.32	2.81E-01	5.05E-01
6.01	6.16	5.95	2.81E-01	5.05E-01
5.75	5.57	5.70	2.81E-01	5.05E-01
14.54	14.54	14.57	2.81E-01	5.05E-01
3.91	3.76	3.80	2.81E-01	5.05E-01
6.48	6.32	6.33	2.81E-01	5.05E-01
8.67	9.00	8.89	2.81E-01	5.06E-01
4.48	4.70	4.48	2.81E-01	5.06E-01
4.59	4.94	4.64	2.81E-01	5.06E-01
5.23	4.93	5.11	2.81E-01	5.06E-01
5.84	5.92	6.04	2.81E-01	5.06E-01
6.34	6.15	6.00	2.81E-01	5.06E-01
11.78	11.72	11.77	2.81E-01	5.06E-01
4.69	4.47	4.51	2.81E-01	5.06E-01
5.40	5.05	5.29	2.81E-01	5.06E-01
9.04	9.04	9.13	2.82E-01	5.06E-01
6.57	6.55	6.51	2.82E-01	5.06E-01
3.56	3.65	3.67	2.82E-01	5.06E-01
7.43	7.37	7.43	2.82E-01	5.06E-01
5.13	4.90	5.13	2.82E-01	5.06E-01
12.38	12.52	12.53	2.82E-01	5.06E-01
7.81	8.04	7.90	2.82E-01	5.06E-01
5.52	5.37	5.99	2.82E-01	5.06E-01
6.81	6.88	6.88	2.82E-01	5.06E-01
5.48	5.21	5.30	2.82E-01	5.06E-01
10.85	10.65	10.71	2.82E-01	5.07E-01
13.27	12.89	13.35	2.82E-01	5.07E-01
3.66	3.52	3.68	2.82E-01	5.07E-01
11.03	11.15	11.05	2.82E-01	5.07E-01
8.39	8.72	8.58	2.82E-01	5.07E-01

9.31	9.47	9.36	2.82E-01	5.07E-01
7.80	7.75	7.88	2.82E-01	5.07E-01
4.99	5.06	5.10	2.82E-01	5.07E-01
6.98	7.11	7.22	2.82E-01	5.07E-01
3.54	3.56	3.56	2.82E-01	5.07E-01
5.86	5.87	5.86	2.83E-01	5.07E-01
3.10	3.17	3.17	2.83E-01	5.07E-01
3.88	3.90	3.70	2.83E-01	5.07E-01
9.25	9.11	9.29	2.83E-01	5.07E-01
5.00	5.06	4.98	2.83E-01	5.07E-01
8.50	8.27	8.53	2.83E-01	5.07E-01
6.15	6.14	6.19	2.83E-01	5.07E-01
5.65	5.44	5.63	2.83E-01	5.08E-01
8.86	8.81	8.84	2.83E-01	5.08E-01
4.06	3.87	4.12	2.83E-01	5.08E-01
9.52	9.72	9.62	2.83E-01	5.08E-01
6.29	6.22	6.33	2.83E-01	5.08E-01
10.31	10.05	10.12	2.83E-01	5.08E-01
4.62	4.75	4.64	2.84E-01	5.08E-01
10.47	10.37	10.63	2.84E-01	5.08E-01
4.37	4.40	4.58	2.84E-01	5.08E-01
7.72	7.75	7.59	2.84E-01	5.08E-01
4.79	5.17	5.13	2.84E-01	5.08E-01
7.26	6.90	7.41	2.84E-01	5.08E-01
7.11	7.40	7.05	2.84E-01	5.08E-01
8.20	8.20	8.24	2.84E-01	5.08E-01
5.95	5.97	5.78	2.84E-01	5.08E-01
9.81	10.05	9.94	2.84E-01	5.08E-01
7.24	7.43	7.24	2.84E-01	5.08E-01
7.18	7.20	7.06	2.84E-01	5.08E-01
7.78	7.78	7.82	2.84E-01	5.08E-01
3.54	3.71	3.63	2.84E-01	5.08E-01
8.70	8.82	8.68	2.84E-01	5.08E-01
9.05	9.19	9.03	2.84E-01	5.08E-01
8.56	8.64	8.70	2.84E-01	5.08E-01
8.64	8.84	8.77	2.84E-01	5.08E-01
8.56	8.57	8.59	2.84E-01	5.08E-01
5.17	5.00	5.03	2.84E-01	5.08E-01
4.39	4.58	4.43	2.84E-01	5.08E-01
9.34	9.65	9.51	2.84E-01	5.08E-01
6.06	5.92	5.91	2.84E-01	5.08E-01
3.93	3.91	4.09	2.84E-01	5.08E-01
7.31	7.37	7.40	2.85E-01	5.08E-01
9.09	9.26	9.20	2.85E-01	5.08E-01
7.14	7.12	7.01	2.85E-01	5.08E-01
7.42	7.32	7.57	2.85E-01	5.08E-01
5.37	5.65	5.33	2.85E-01	5.08E-01

8.37	8.31	8.48	2.85E-01	5.08E-01
11.06	11.27	11.09	2.85E-01	5.08E-01
5.33	5.46	5.42	2.85E-01	5.08E-01
9.60	9.53	9.58	2.85E-01	5.09E-01
7.39	7.35	7.40	2.85E-01	5.09E-01
9.34	9.45	9.42	2.85E-01	5.09E-01
3.45	3.51	3.67	2.85E-01	5.09E-01
6.81	6.69	6.83	2.85E-01	5.09E-01
10.63	10.74	10.69	2.85E-01	5.09E-01
5.98	6.00	6.01	2.85E-01	5.09E-01
6.06	6.10	5.99	2.85E-01	5.09E-01
12.17	12.30	12.26	2.85E-01	5.09E-01
4.91	4.63	4.93	2.85E-01	5.09E-01
9.70	9.61	9.72	2.86E-01	5.09E-01
6.38	6.39	6.39	2.86E-01	5.09E-01
11.01	10.77	10.86	2.86E-01	5.09E-01
4.68	4.35	4.64	2.86E-01	5.09E-01
7.25	7.50	7.32	2.86E-01	5.09E-01
8.46	8.50	8.54	2.86E-01	5.09E-01
5.31	5.22	5.14	2.86E-01	5.09E-01
5.18	5.41	5.26	2.86E-01	5.09E-01
4.29	4.16	4.59	2.86E-01	5.09E-01
9.72	9.83	9.95	2.86E-01	5.09E-01
8.55	8.66	8.49	2.86E-01	5.09E-01
5.57	5.75	5.80	2.86E-01	5.09E-01
7.22	7.07	7.25	2.86E-01	5.09E-01
7.21	7.14	6.84	2.86E-01	5.09E-01
8.83	9.15	8.96	2.86E-01	5.09E-01
9.06	9.24	9.02	2.86E-01	5.09E-01
10.12	10.21	10.16	2.86E-01	5.09E-01
9.80	9.67	9.88	2.86E-01	5.09E-01
9.36	9.48	9.38	2.86E-01	5.09E-01
4.67	4.29	4.58	2.86E-01	5.09E-01
8.05	8.12	8.09	2.86E-01	5.09E-01
5.22	5.34	5.21	2.87E-01	5.09E-01
3.30	3.30	3.41	2.87E-01	5.09E-01
4.70	4.87	4.92	2.87E-01	5.09E-01
7.58	7.65	7.88	2.87E-01	5.09E-01
8.36	8.23	8.43	2.87E-01	5.09E-01
6.68	6.57	6.72	2.87E-01	5.09E-01
5.57	5.39	5.52	2.87E-01	5.09E-01
6.76	6.61	6.68	2.87E-01	5.09E-01
7.05	7.07	7.25	2.87E-01	5.09E-01
9.75	9.53	9.70	2.87E-01	5.09E-01
5.03	4.73	4.76	2.87E-01	5.09E-01
5.97	5.88	6.02	2.87E-01	5.09E-01
10.34	10.29	10.45	2.87E-01	5.09E-01

8.24	8.28	8.33	2.87E-01	5.09E-01
10.16	9.86	10.21	2.87E-01	5.09E-01
3.72	3.71	3.96	2.87E-01	5.09E-01
8.79	8.80	8.77	2.87E-01	5.09E-01
9.01	9.05	9.15	2.87E-01	5.09E-01
3.35	3.27	3.25	2.87E-01	5.09E-01
11.58	11.73	11.69	2.87E-01	5.09E-01
4.42	4.72	4.57	2.87E-01	5.09E-01
5.89	5.84	5.92	2.87E-01	5.09E-01
9.51	9.41	9.52	2.87E-01	5.09E-01
9.96	9.85	9.96	2.87E-01	5.09E-01
3.48	3.65	3.45	2.87E-01	5.09E-01
8.22	8.02	8.17	2.87E-01	5.10E-01
5.40	5.19	5.18	2.87E-01	5.10E-01
5.76	5.58	5.64	2.87E-01	5.10E-01
8.84	8.90	8.81	2.87E-01	5.10E-01
7.43	7.58	7.37	2.87E-01	5.10E-01
3.48	3.55	3.28	2.87E-01	5.10E-01
6.59	6.67	6.68	2.87E-01	5.10E-01
3.54	3.48	3.64	2.87E-01	5.10E-01
11.89	11.80	11.89	2.88E-01	5.10E-01
7.32	7.28	7.27	2.88E-01	5.10E-01
8.68	8.81	8.68	2.88E-01	5.10E-01
14.96	15.00	15.00	2.88E-01	5.10E-01
3.14	3.31	3.24	2.88E-01	5.10E-01
6.10	6.05	6.17	2.88E-01	5.10E-01
4.09	4.19	4.02	2.88E-01	5.10E-01
4.46	4.57	4.34	2.88E-01	5.10E-01
5.98	5.87	5.80	2.88E-01	5.10E-01
6.62	6.97	6.49	2.88E-01	5.10E-01
8.78	8.54	8.85	2.88E-01	5.11E-01
5.82	5.91	5.96	2.89E-01	5.11E-01
5.43	5.20	5.01	2.89E-01	5.11E-01
5.44	5.69	5.39	2.89E-01	5.11E-01
10.10	9.84	10.00	2.89E-01	5.11E-01
5.40	5.42	5.45	2.89E-01	5.11E-01
5.83	5.85	5.62	2.89E-01	5.11E-01
3.34	3.14	3.35	2.89E-01	5.11E-01
7.73	7.78	7.82	2.89E-01	5.11E-01
3.34	3.24	3.51	2.89E-01	5.11E-01
6.08	5.66	5.93	2.89E-01	5.11E-01
12.21	12.48	12.30	2.89E-01	5.11E-01
8.30	8.19	8.36	2.89E-01	5.11E-01
8.39	8.35	8.39	2.89E-01	5.11E-01
3.26	3.38	3.54	2.89E-01	5.11E-01
4.32	4.14	4.19	2.89E-01	5.11E-01
7.86	8.03	8.13	2.89E-01	5.11E-01

5.86	5.84	5.76	2.89E-01	5.11E-01
9.24	9.36	9.20	2.89E-01	5.11E-01
8.37	8.41	8.40	2.89E-01	5.11E-01
7.54	7.50	7.63	2.89E-01	5.11E-01
6.75	6.95	6.66	2.89E-01	5.11E-01
7.45	6.92	7.74	2.89E-01	5.11E-01
9.40	9.50	9.43	2.89E-01	5.11E-01
3.77	3.76	3.75	2.89E-01	5.11E-01
9.54	9.63	9.65	2.90E-01	5.11E-01
4.77	4.66	4.77	2.90E-01	5.11E-01
5.60	5.60	5.80	2.90E-01	5.11E-01
5.69	6.16	5.99	2.90E-01	5.11E-01
4.21	4.35	4.44	2.90E-01	5.11E-01
9.61	9.43	9.54	2.90E-01	5.11E-01
4.62	4.21	4.17	2.90E-01	5.11E-01
8.09	8.21	8.17	2.90E-01	5.11E-01
12.20	12.34	12.32	2.90E-01	5.11E-01
5.25	5.04	5.02	2.90E-01	5.11E-01
6.45	6.80	6.62	2.90E-01	5.11E-01
8.07	7.94	8.08	2.90E-01	5.12E-01
6.08	6.12	6.05	2.90E-01	5.12E-01
4.96	4.79	5.24	2.90E-01	5.12E-01
7.57	7.96	7.78	2.90E-01	5.12E-01
4.14	3.84	4.14	2.90E-01	5.12E-01
6.53	6.24	6.43	2.90E-01	5.12E-01
10.95	10.88	10.94	2.90E-01	5.12E-01
3.85	3.74	3.57	2.90E-01	5.12E-01
5.06	4.84	4.76	2.91E-01	5.12E-01
8.84	8.76	8.84	2.91E-01	5.12E-01
8.01	7.99	8.16	2.91E-01	5.12E-01
3.18	3.23	3.20	2.91E-01	5.12E-01
9.83	10.12	9.97	2.91E-01	5.12E-01
7.76	7.82	8.00	2.91E-01	5.12E-01
8.83	9.02	9.00	2.91E-01	5.12E-01
5.25	5.05	5.25	2.91E-01	5.12E-01
3.47	3.53	3.51	2.91E-01	5.12E-01
4.04	4.03	4.16	2.91E-01	5.12E-01
6.64	6.56	6.39	2.91E-01	5.13E-01
4.00	4.20	4.17	2.91E-01	5.13E-01
6.44	6.33	6.69	2.91E-01	5.13E-01
8.56	8.54	8.73	2.91E-01	5.13E-01
5.43	5.42	5.48	2.91E-01	5.13E-01
4.92	4.62	4.58	2.92E-01	5.13E-01
5.52	5.32	5.10	2.92E-01	5.13E-01
7.46	7.71	7.48	2.92E-01	5.13E-01
5.99	5.84	5.93	2.92E-01	5.13E-01
10.67	10.69	10.74	2.92E-01	5.13E-01

6.83	6.94	6.61	2.92E-01	5.13E-01
9.38	9.89	9.73	2.92E-01	5.13E-01
9.43	9.21	9.47	2.92E-01	5.13E-01
6.75	6.42	6.48	2.92E-01	5.13E-01
8.75	9.01	9.03	2.92E-01	5.13E-01
5.76	5.79	5.67	2.92E-01	5.13E-01
5.20	5.41	5.54	2.92E-01	5.13E-01
5.10	5.46	5.32	2.92E-01	5.13E-01
5.30	5.37	5.32	2.92E-01	5.13E-01
7.05	6.97	7.06	2.92E-01	5.13E-01
7.89	8.07	7.81	2.92E-01	5.13E-01
8.67	8.62	8.66	2.92E-01	5.13E-01
3.50	3.30	3.60	2.92E-01	5.13E-01
9.46	9.65	9.67	2.92E-01	5.13E-01
5.30	5.47	5.19	2.92E-01	5.13E-01
9.73	9.53	9.75	2.92E-01	5.13E-01
7.12	7.11	7.17	2.92E-01	5.13E-01
9.18	9.36	9.36	2.92E-01	5.13E-01
5.19	5.55	5.35	2.92E-01	5.13E-01
10.61	10.73	10.69	2.92E-01	5.13E-01
5.98	6.15	6.05	2.92E-01	5.13E-01
3.78	3.72	3.79	2.92E-01	5.13E-01
7.41	7.42	7.44	2.92E-01	5.13E-01
4.10	4.47	4.34	2.92E-01	5.13E-01
9.28	9.23	9.30	2.93E-01	5.13E-01
4.43	4.37	4.40	2.93E-01	5.13E-01
8.45	8.47	8.54	2.93E-01	5.13E-01
7.93	8.13	7.93	2.93E-01	5.13E-01
4.96	4.99	4.88	2.93E-01	5.13E-01
7.28	7.31	7.41	2.93E-01	5.13E-01
4.89	4.91	4.98	2.93E-01	5.13E-01
5.07	5.00	4.94	2.93E-01	5.13E-01
3.85	4.05	3.93	2.93E-01	5.13E-01
4.86	4.99	5.16	2.93E-01	5.13E-01
8.24	8.07	8.30	2.93E-01	5.13E-01
9.07	8.84	9.16	2.93E-01	5.13E-01
12.41	12.59	12.48	2.93E-01	5.13E-01
9.66	9.86	9.86	2.93E-01	5.13E-01
3.63	3.92	3.63	2.93E-01	5.13E-01
8.15	8.18	8.22	2.93E-01	5.13E-01
9.52	9.89	9.57	2.93E-01	5.13E-01
13.25	13.37	13.34	2.93E-01	5.14E-01
6.40	6.60	6.41	2.93E-01	5.14E-01
4.43	4.32	4.40	2.93E-01	5.14E-01
9.14	9.10	9.07	2.94E-01	5.14E-01
3.66	3.46	3.42	2.94E-01	5.14E-01
4.04	3.61	3.91	2.94E-01	5.14E-01

6.21	6.15	5.96	2.94E-01	5.14E-01
10.52	10.77	10.64	2.94E-01	5.14E-01
7.39	6.98	7.03	2.94E-01	5.14E-01
11.56	11.60	11.61	2.94E-01	5.14E-01
6.66	6.89	6.74	2.94E-01	5.14E-01
5.82	5.82	6.00	2.94E-01	5.14E-01
10.50	10.50	10.60	2.94E-01	5.14E-01
9.04	9.04	8.97	2.94E-01	5.14E-01
12.01	11.91	11.90	2.94E-01	5.14E-01
3.57	3.91	3.62	2.94E-01	5.14E-01
13.65	13.71	13.71	2.94E-01	5.14E-01
8.39	8.04	8.45	2.94E-01	5.14E-01
6.91	6.84	6.87	2.94E-01	5.14E-01
11.06	11.19	11.17	2.94E-01	5.14E-01
6.83	6.83	6.74	2.94E-01	5.14E-01
5.10	5.24	5.22	2.94E-01	5.14E-01
5.94	5.93	5.89	2.94E-01	5.14E-01
5.39	5.20	5.34	2.94E-01	5.14E-01
7.79	7.81	7.90	2.95E-01	5.14E-01
7.61	7.71	7.66	2.95E-01	5.14E-01
4.15	4.00	4.12	2.95E-01	5.14E-01
5.56	5.55	5.70	2.95E-01	5.14E-01
5.15	5.15	5.37	2.95E-01	5.14E-01
5.69	5.95	6.01	2.95E-01	5.14E-01
6.89	6.75	6.80	2.95E-01	5.14E-01
3.71	3.61	3.69	2.95E-01	5.14E-01
3.40	3.35	3.21	2.95E-01	5.14E-01
6.05	6.00	5.83	2.95E-01	5.14E-01
6.96	6.90	6.91	2.95E-01	5.14E-01
7.12	7.06	7.00	2.95E-01	5.14E-01
7.90	7.97	8.04	2.95E-01	5.14E-01
6.23	6.36	6.25	2.95E-01	5.14E-01
12.53	12.37	12.43	2.95E-01	5.14E-01
8.07	8.22	8.18	2.95E-01	5.14E-01
5.02	5.21	5.00	2.95E-01	5.14E-01
7.03	6.54	6.93	2.95E-01	5.14E-01
8.78	9.03	8.91	2.95E-01	5.14E-01
3.80	4.09	4.27	2.95E-01	5.14E-01
6.60	6.49	6.60	2.95E-01	5.14E-01
8.95	9.00	8.98	2.95E-01	5.14E-01
4.97	4.77	4.77	2.95E-01	5.14E-01
9.94	10.07	10.06	2.95E-01	5.14E-01
5.08	5.36	5.22	2.95E-01	5.14E-01
5.77	5.84	5.79	2.95E-01	5.14E-01
3.38	3.35	3.23	2.95E-01	5.14E-01
5.45	5.24	5.41	2.95E-01	5.14E-01
9.58	9.65	9.59	2.95E-01	5.14E-01

7.95	7.82	7.99	2.95E-01	5.14E-01
10.28	10.31	10.26	2.96E-01	5.14E-01
8.65	8.94	8.69	2.96E-01	5.14E-01
6.71	6.56	6.83	2.96E-01	5.14E-01
7.75	7.77	7.76	2.96E-01	5.14E-01
8.05	8.00	8.19	2.96E-01	5.14E-01
7.96	8.09	8.09	2.96E-01	5.14E-01
8.93	8.80	8.92	2.96E-01	5.14E-01
5.65	5.75	5.87	2.96E-01	5.14E-01
6.92	7.04	6.88	2.96E-01	5.14E-01
4.06	4.24	4.16	2.96E-01	5.14E-01
5.91	6.03	5.98	2.96E-01	5.14E-01
3.87	3.76	3.71	2.96E-01	5.15E-01
8.84	8.16	8.65	2.96E-01	5.15E-01
4.27	4.28	4.35	2.96E-01	5.15E-01
10.72	10.74	10.76	2.96E-01	5.15E-01
4.41	4.17	4.19	2.96E-01	5.15E-01
3.27	3.19	3.29	2.96E-01	5.15E-01
12.90	12.98	12.99	2.96E-01	5.15E-01
9.86	9.82	9.83	2.96E-01	5.15E-01
4.92	4.92	4.97	2.96E-01	5.15E-01
4.73	4.63	4.63	2.96E-01	5.15E-01
7.09	7.04	6.94	2.96E-01	5.15E-01
7.84	7.81	7.73	2.96E-01	5.15E-01
7.47	7.65	7.54	2.96E-01	5.15E-01
11.10	11.25	11.16	2.96E-01	5.15E-01
8.22	8.34	8.22	2.97E-01	5.15E-01
5.28	4.85	5.24	2.97E-01	5.15E-01
7.74	7.60	7.72	2.97E-01	5.15E-01
9.82	9.61	9.87	2.97E-01	5.15E-01
5.63	5.68	5.65	2.97E-01	5.15E-01
8.73	8.80	8.75	2.97E-01	5.15E-01
5.73	6.03	5.71	2.97E-01	5.15E-01
7.52	7.69	7.72	2.97E-01	5.15E-01
9.66	9.78	9.62	2.97E-01	5.15E-01
8.87	9.08	9.02	2.97E-01	5.15E-01
10.57	10.35	10.52	2.97E-01	5.15E-01
10.79	11.02	10.89	2.97E-01	5.15E-01
6.49	6.55	6.48	2.97E-01	5.15E-01
7.00	7.12	7.10	2.97E-01	5.15E-01
8.72	8.63	8.63	2.97E-01	5.15E-01
7.61	7.85	7.69	2.97E-01	5.15E-01
8.89	9.03	8.97	2.97E-01	5.15E-01
4.61	4.88	4.67	2.97E-01	5.15E-01
6.83	7.24	6.97	2.97E-01	5.15E-01
8.98	8.60	9.03	2.97E-01	5.15E-01
4.70	4.86	4.78	2.97E-01	5.15E-01

7.57	7.72	7.53	2.97E-01	5.15E-01
10.06	10.28	10.07	2.98E-01	5.15E-01
8.35	8.26	8.11	2.98E-01	5.15E-01
6.59	6.82	6.80	2.98E-01	5.15E-01
5.21	5.18	5.38	2.98E-01	5.15E-01
6.40	6.47	6.40	2.98E-01	5.15E-01
8.67	8.66	8.75	2.98E-01	5.15E-01
5.79	6.07	5.77	2.98E-01	5.15E-01
9.17	9.60	9.35	2.98E-01	5.16E-01
3.16	3.20	3.13	2.98E-01	5.16E-01
4.17	3.91	4.08	2.98E-01	5.16E-01
5.93	5.98	6.23	2.98E-01	5.16E-01
6.42	6.58	6.36	2.98E-01	5.16E-01
5.51	5.37	5.51	2.98E-01	5.16E-01
4.15	3.77	3.66	2.98E-01	5.16E-01
8.64	8.52	8.67	2.98E-01	5.16E-01
9.55	9.83	9.66	2.98E-01	5.16E-01
8.23	8.05	8.25	2.98E-01	5.16E-01
5.71	5.55	5.67	2.99E-01	5.16E-01
10.43	10.33	10.44	2.99E-01	5.16E-01
4.42	4.35	4.30	2.99E-01	5.16E-01
6.75	6.62	6.81	2.99E-01	5.16E-01
4.11	4.02	3.83	2.99E-01	5.16E-01
3.79	3.82	3.82	2.99E-01	5.16E-01
11.45	11.10	11.57	2.99E-01	5.16E-01
4.45	4.69	4.45	2.99E-01	5.16E-01
6.61	6.78	6.58	2.99E-01	5.16E-01
10.85	10.88	10.93	2.99E-01	5.16E-01
3.69	3.99	3.96	2.99E-01	5.16E-01
12.03	11.86	11.93	2.99E-01	5.16E-01
8.77	9.03	8.96	2.99E-01	5.16E-01
3.34	3.51	3.31	2.99E-01	5.16E-01
5.51	5.71	5.56	2.99E-01	5.16E-01
6.88	6.58	6.52	2.99E-01	5.16E-01
9.19	9.47	9.35	2.99E-01	5.16E-01
7.43	7.56	7.54	2.99E-01	5.16E-01
5.56	5.44	5.56	2.99E-01	5.16E-01
3.90	3.82	3.87	2.99E-01	5.16E-01
3.33	3.41	3.25	2.99E-01	5.16E-01
3.45	3.47	3.60	2.99E-01	5.16E-01
10.28	9.99	10.12	2.99E-01	5.16E-01
6.81	6.90	6.90	2.99E-01	5.16E-01
7.74	7.83	7.85	3.00E-01	5.16E-01
4.59	4.60	4.63	3.00E-01	5.16E-01
5.87	5.78	5.94	3.00E-01	5.16E-01
7.56	7.76	7.86	3.00E-01	5.16E-01
4.26	4.34	4.29	3.00E-01	5.16E-01

6.03	5.87	6.02	3.00E-01	5.16E-01
4.08	4.19	4.22	3.00E-01	5.16E-01
4.83	5.19	5.08	3.00E-01	5.16E-01
6.19	6.36	6.25	3.00E-01	5.16E-01
5.74	5.71	5.64	3.00E-01	5.16E-01
4.97	4.69	4.73	3.00E-01	5.16E-01
4.74	4.55	4.94	3.00E-01	5.16E-01
3.50	3.35	3.50	3.00E-01	5.16E-01
6.43	6.44	6.43	3.00E-01	5.16E-01
4.17	4.25	4.08	3.00E-01	5.16E-01
8.88	9.03	9.04	3.00E-01	5.16E-01
6.70	6.75	6.83	3.00E-01	5.16E-01
7.78	7.63	7.71	3.00E-01	5.17E-01
9.49	9.40	9.60	3.00E-01	5.17E-01
5.11	4.93	5.00	3.00E-01	5.17E-01
13.07	13.01	13.05	3.00E-01	5.17E-01
7.73	7.82	7.65	3.00E-01	5.17E-01
6.13	6.42	5.80	3.00E-01	5.17E-01
11.28	11.28	11.51	3.01E-01	5.17E-01
3.58	3.55	3.32	3.01E-01	5.17E-01
7.69	7.87	7.71	3.01E-01	5.17E-01
4.06	4.02	3.78	3.01E-01	5.17E-01
4.75	4.73	4.86	3.01E-01	5.17E-01
7.78	7.73	7.65	3.01E-01	5.17E-01
4.47	4.54	4.69	3.01E-01	5.17E-01
5.33	5.05	5.10	3.01E-01	5.17E-01
7.78	8.12	7.91	3.01E-01	5.17E-01
3.49	3.37	3.36	3.01E-01	5.17E-01
4.58	4.84	4.42	3.01E-01	5.17E-01
4.81	4.74	4.48	3.01E-01	5.17E-01
4.82	5.01	5.08	3.01E-01	5.17E-01
5.68	5.68	5.68	3.01E-01	5.17E-01
5.21	5.28	5.29	3.01E-01	5.17E-01
7.52	7.33	7.45	3.01E-01	5.17E-01
3.68	3.65	3.65	3.01E-01	5.18E-01
5.06	4.99	4.93	3.01E-01	5.18E-01
6.26	6.14	6.12	3.01E-01	5.18E-01
8.60	8.81	8.76	3.01E-01	5.18E-01
4.58	4.64	4.64	3.02E-01	5.18E-01
4.81	5.07	4.98	3.02E-01	5.18E-01
5.80	5.75	5.59	3.02E-01	5.18E-01
3.24	3.21	3.21	3.02E-01	5.18E-01
9.77	9.88	9.79	3.02E-01	5.18E-01
6.02	5.96	5.98	3.02E-01	5.18E-01
10.29	10.14	10.23	3.02E-01	5.18E-01
9.81	9.78	9.86	3.02E-01	5.18E-01
8.14	8.14	8.13	3.02E-01	5.18E-01

3.83	3.64	3.49	3.02E-01	5.18E-01
7.52	7.83	7.77	3.02E-01	5.18E-01
4.12	4.12	4.12	3.02E-01	5.18E-01
4.99	4.79	5.03	3.02E-01	5.18E-01
4.52	4.47	4.45	3.02E-01	5.18E-01
3.51	3.41	3.55	3.02E-01	5.18E-01
6.68	6.64	6.25	3.02E-01	5.18E-01
7.20	7.24	7.20	3.02E-01	5.18E-01
8.02	7.92	8.01	3.02E-01	5.18E-01
8.14	8.37	8.30	3.03E-01	5.18E-01
3.38	2.97	3.42	3.03E-01	5.18E-01
8.09	7.81	7.96	3.03E-01	5.18E-01
5.78	5.62	5.78	3.03E-01	5.18E-01
8.57	8.66	8.67	3.03E-01	5.18E-01
8.05	8.04	8.15	3.03E-01	5.18E-01
6.29	6.03	6.08	3.03E-01	5.18E-01
6.28	6.31	6.17	3.03E-01	5.18E-01
10.04	10.00	9.85	3.03E-01	5.18E-01
8.76	8.78	8.87	3.03E-01	5.18E-01
5.39	5.23	5.30	3.03E-01	5.18E-01
9.05	8.99	9.05	3.03E-01	5.18E-01
6.95	6.77	6.67	3.03E-01	5.18E-01
6.81	7.08	6.71	3.03E-01	5.18E-01
4.76	4.44	4.59	3.03E-01	5.18E-01
7.45	7.41	7.46	3.03E-01	5.18E-01
4.73	4.74	4.87	3.03E-01	5.18E-01
7.26	7.19	7.15	3.03E-01	5.18E-01
8.07	8.17	8.22	3.03E-01	5.18E-01
8.78	8.80	8.81	3.03E-01	5.18E-01
6.52	6.65	6.50	3.03E-01	5.18E-01
11.42	11.34	11.45	3.03E-01	5.18E-01
3.27	3.06	3.20	3.03E-01	5.18E-01
8.53	8.54	8.64	3.03E-01	5.18E-01
3.57	3.51	3.75	3.03E-01	5.18E-01
4.19	4.36	4.44	3.03E-01	5.18E-01
4.48	4.48	4.84	3.03E-01	5.18E-01
3.25	3.53	3.41	3.03E-01	5.18E-01
14.90	14.85	14.89	3.03E-01	5.18E-01
10.44	10.66	10.40	3.04E-01	5.18E-01
8.69	8.71	8.72	3.04E-01	5.18E-01
9.84	9.63	9.71	3.04E-01	5.18E-01
8.63	8.83	8.66	3.04E-01	5.18E-01
7.64	7.58	7.63	3.04E-01	5.18E-01
4.91	4.92	4.65	3.04E-01	5.18E-01
7.83	7.93	8.03	3.04E-01	5.18E-01
5.25	5.21	5.36	3.04E-01	5.18E-01
8.34	8.47	8.52	3.04E-01	5.18E-01

6.19	6.15	6.05	3.04E-01	5.18E-01
3.93	4.03	3.90	3.04E-01	5.18E-01
10.67	10.48	10.74	3.04E-01	5.18E-01
11.64	11.76	11.78	3.04E-01	5.18E-01
5.89	6.14	5.97	3.04E-01	5.18E-01
5.78	5.90	5.78	3.04E-01	5.18E-01
10.67	10.44	10.60	3.04E-01	5.19E-01
7.48	7.48	7.49	3.04E-01	5.19E-01
6.14	6.39	6.50	3.04E-01	5.19E-01
4.34	4.27	4.09	3.04E-01	5.19E-01
5.52	5.30	5.56	3.04E-01	5.19E-01
4.27	4.10	4.41	3.04E-01	5.19E-01
6.94	7.06	6.91	3.05E-01	5.19E-01
5.40	5.11	5.19	3.05E-01	5.19E-01
12.09	11.90	12.01	3.05E-01	5.19E-01
3.75	3.67	3.56	3.05E-01	5.19E-01
6.59	6.60	6.47	3.05E-01	5.19E-01
7.53	7.46	7.53	3.05E-01	5.19E-01
4.99	5.00	4.73	3.05E-01	5.19E-01
3.50	3.38	3.52	3.05E-01	5.19E-01
4.22	4.51	4.39	3.05E-01	5.19E-01
6.19	6.37	6.39	3.05E-01	5.19E-01
5.59	5.51	5.43	3.05E-01	5.19E-01
8.25	8.47	8.34	3.05E-01	5.19E-01
3.50	3.55	3.26	3.05E-01	5.19E-01
5.74	5.82	5.29	3.05E-01	5.19E-01
9.09	9.09	9.06	3.05E-01	5.19E-01
10.07	10.08	10.12	3.05E-01	5.19E-01
9.45	9.57	9.46	3.05E-01	5.19E-01
9.54	9.66	9.70	3.05E-01	5.19E-01
6.35	6.36	6.37	3.05E-01	5.19E-01
6.64	6.60	6.38	3.05E-01	5.19E-01
8.69	8.25	8.33	3.05E-01	5.19E-01
6.78	6.60	6.70	3.05E-01	5.19E-01
8.53	9.13	8.59	3.05E-01	5.19E-01
10.48	10.31	10.38	3.05E-01	5.19E-01
7.14	7.11	7.12	3.05E-01	5.19E-01
6.06	5.92	6.03	3.06E-01	5.19E-01
15.08	15.06	15.08	3.06E-01	5.20E-01
5.77	5.60	5.61	3.06E-01	5.20E-01
3.91	3.94	4.19	3.06E-01	5.20E-01
7.21	7.32	7.25	3.06E-01	5.20E-01
7.54	7.44	7.61	3.06E-01	5.20E-01
8.84	8.72	8.93	3.06E-01	5.20E-01
5.40	5.27	5.47	3.06E-01	5.20E-01
11.47	11.34	11.31	3.06E-01	5.20E-01
6.23	5.98	6.24	3.06E-01	5.20E-01

4.56	4.72	4.54	3.06E-01	5.20E-01
5.78	5.72	5.59	3.06E-01	5.20E-01
5.19	5.01	5.05	3.06E-01	5.20E-01
7.64	8.08	8.02	3.06E-01	5.20E-01
9.50	9.68	9.53	3.06E-01	5.20E-01
8.26	8.50	8.44	3.06E-01	5.20E-01
9.32	9.35	9.46	3.06E-01	5.20E-01
5.17	5.29	5.37	3.06E-01	5.20E-01
5.18	5.23	5.20	3.06E-01	5.20E-01
4.61	4.66	4.94	3.06E-01	5.20E-01
8.59	8.51	8.62	3.06E-01	5.20E-01
5.75	5.80	5.55	3.07E-01	5.20E-01
7.61	7.74	7.77	3.07E-01	5.20E-01
6.47	6.57	6.59	3.07E-01	5.20E-01
8.62	8.57	8.61	3.07E-01	5.20E-01
4.26	4.05	4.23	3.07E-01	5.20E-01
13.21	13.28	13.28	3.07E-01	5.20E-01
5.97	6.03	5.94	3.07E-01	5.20E-01
6.06	5.95	5.81	3.07E-01	5.20E-01
4.31	4.15	4.18	3.07E-01	5.20E-01
14.28	14.20	14.27	3.07E-01	5.20E-01
9.35	9.11	9.30	3.07E-01	5.20E-01
4.99	4.62	4.94	3.07E-01	5.20E-01
10.77	10.15	10.64	3.07E-01	5.20E-01
4.19	4.22	3.84	3.07E-01	5.20E-01
7.53	7.34	7.36	3.07E-01	5.20E-01
9.86	9.78	9.94	3.07E-01	5.20E-01
5.31	5.60	5.58	3.07E-01	5.20E-01
3.60	3.47	3.44	3.07E-01	5.20E-01
9.04	9.12	8.97	3.07E-01	5.20E-01
5.22	5.24	5.11	3.07E-01	5.20E-01
3.58	3.66	3.65	3.07E-01	5.20E-01
5.09	4.96	4.90	3.07E-01	5.20E-01
5.50	5.45	5.47	3.07E-01	5.20E-01
12.31	12.34	12.39	3.07E-01	5.20E-01
6.82	6.64	6.92	3.07E-01	5.20E-01
6.45	6.28	6.22	3.08E-01	5.20E-01
4.54	4.27	4.52	3.08E-01	5.20E-01
5.09	4.96	4.92	3.08E-01	5.20E-01
5.44	5.49	5.78	3.08E-01	5.20E-01
4.48	4.33	4.38	3.08E-01	5.20E-01
7.45	7.46	7.45	3.08E-01	5.20E-01
5.51	5.77	5.71	3.08E-01	5.20E-01
6.08	6.02	5.94	3.08E-01	5.20E-01
8.39	8.20	8.20	3.08E-01	5.20E-01
5.62	5.85	5.77	3.08E-01	5.20E-01
4.00	4.07	4.05	3.08E-01	5.20E-01

6.73	6.70	6.76	3.08E-01	5.20E-01
6.40	6.56	6.69	3.08E-01	5.20E-01
5.93	5.74	5.93	3.08E-01	5.20E-01
11.60	11.71	11.77	3.08E-01	5.21E-01
3.98	4.15	4.10	3.08E-01	5.21E-01
8.95	9.16	9.12	3.08E-01	5.21E-01
13.42	13.55	13.59	3.08E-01	5.21E-01
6.81	7.00	6.86	3.08E-01	5.21E-01
4.16	4.31	4.04	3.08E-01	5.21E-01
6.11	6.33	6.52	3.08E-01	5.21E-01
5.98	5.91	5.94	3.08E-01	5.21E-01
3.37	3.53	3.31	3.08E-01	5.21E-01
7.64	7.81	7.91	3.08E-01	5.21E-01
14.66	14.65	14.67	3.08E-01	5.21E-01
9.67	9.45	9.74	3.08E-01	5.21E-01
7.84	7.57	7.89	3.09E-01	5.21E-01
11.27	11.30	11.20	3.09E-01	5.21E-01
9.61	9.32	9.44	3.09E-01	5.21E-01
6.33	6.57	6.43	3.09E-01	5.21E-01
8.94	8.98	8.92	3.09E-01	5.21E-01
8.47	8.63	8.56	3.09E-01	5.21E-01
10.03	10.35	10.22	3.09E-01	5.21E-01
3.45	3.41	3.43	3.09E-01	5.21E-01
9.12	9.13	9.31	3.09E-01	5.21E-01
9.26	9.36	9.45	3.09E-01	5.21E-01
8.89	8.59	8.84	3.09E-01	5.21E-01
14.75	14.74	14.76	3.09E-01	5.21E-01
8.48	8.53	8.56	3.09E-01	5.21E-01
5.15	5.09	5.25	3.09E-01	5.21E-01
10.47	10.42	10.48	3.09E-01	5.21E-01
9.63	9.83	9.77	3.09E-01	5.21E-01
5.88	5.78	5.86	3.09E-01	5.21E-01
6.15	6.07	6.14	3.09E-01	5.21E-01
6.63	6.67	6.56	3.09E-01	5.21E-01
7.27	7.33	7.68	3.09E-01	5.21E-01
8.84	8.90	8.97	3.09E-01	5.21E-01
3.36	3.22	3.28	3.09E-01	5.21E-01
8.80	8.89	8.79	3.09E-01	5.21E-01
8.67	8.97	8.77	3.10E-01	5.21E-01
5.14	4.93	5.39	3.10E-01	5.21E-01
6.00	6.07	5.86	3.10E-01	5.21E-01
6.07	6.09	5.87	3.10E-01	5.21E-01
3.47	3.61	3.84	3.10E-01	5.21E-01
9.07	9.13	9.11	3.10E-01	5.21E-01
7.34	7.57	7.53	3.10E-01	5.21E-01
5.27	5.30	5.33	3.10E-01	5.21E-01
6.93	6.80	6.82	3.10E-01	5.21E-01

7.79	7.82	7.86	3.10E-01	5.21E-01
6.50	6.46	6.72	3.10E-01	5.21E-01
3.96	3.88	3.62	3.10E-01	5.21E-01
6.65	6.87	6.85	3.10E-01	5.21E-01
11.08	11.26	11.10	3.10E-01	5.21E-01
6.05	6.19	6.37	3.10E-01	5.21E-01
6.69	6.86	6.68	3.10E-01	5.21E-01
5.09	5.37	5.14	3.10E-01	5.21E-01
12.38	12.37	12.45	3.10E-01	5.21E-01
7.57	7.62	7.56	3.10E-01	5.21E-01
12.06	11.99	12.16	3.10E-01	5.21E-01
8.74	8.78	8.85	3.10E-01	5.21E-01
7.80	8.03	8.03	3.10E-01	5.21E-01
8.44	8.35	8.40	3.10E-01	5.21E-01
9.27	9.25	9.24	3.10E-01	5.21E-01
6.66	7.02	6.73	3.10E-01	5.21E-01
7.92	7.81	7.68	3.11E-01	5.21E-01
5.00	5.04	4.96	3.11E-01	5.21E-01
9.35	9.33	9.39	3.11E-01	5.21E-01
5.75	5.80	5.70	3.11E-01	5.21E-01
7.16	7.47	7.07	3.11E-01	5.21E-01
8.08	7.88	8.00	3.11E-01	5.21E-01
6.72	6.75	6.75	3.11E-01	5.21E-01
9.19	9.37	9.17	3.11E-01	5.21E-01
3.00	3.47	3.25	3.11E-01	5.21E-01
6.11	5.94	6.26	3.11E-01	5.21E-01
4.88	4.83	4.66	3.11E-01	5.21E-01
8.17	7.89	8.10	3.11E-01	5.21E-01
11.06	10.86	10.97	3.11E-01	5.21E-01
10.42	10.26	10.38	3.11E-01	5.21E-01
9.55	9.86	9.64	3.11E-01	5.21E-01
7.48	7.40	7.40	3.11E-01	5.21E-01
7.19	6.98	7.30	3.11E-01	5.21E-01
4.64	4.49	4.23	3.11E-01	5.21E-01
10.24	10.27	10.27	3.11E-01	5.21E-01
3.42	3.63	3.34	3.11E-01	5.22E-01
6.55	6.37	6.40	3.11E-01	5.22E-01
6.64	6.81	7.03	3.11E-01	5.22E-01
12.25	12.15	12.23	3.11E-01	5.22E-01
7.33	7.43	7.31	3.12E-01	5.22E-01
9.40	9.49	9.43	3.12E-01	5.22E-01
13.32	13.31	13.26	3.12E-01	5.22E-01
3.87	3.87	4.16	3.12E-01	5.22E-01
9.44	9.36	9.56	3.12E-01	5.22E-01
9.90	9.76	9.75	3.12E-01	5.22E-01
9.43	9.17	9.28	3.12E-01	5.22E-01
4.91	4.97	4.80	3.12E-01	5.22E-01

9.15	9.01	9.00	3.12E-01	5.22E-01
4.99	4.91	4.89	3.12E-01	5.22E-01
6.19	6.36	5.97	3.12E-01	5.22E-01
7.96	8.16	8.18	3.12E-01	5.22E-01
4.10	4.30	4.16	3.12E-01	5.22E-01
10.52	10.48	10.59	3.12E-01	5.22E-01
12.88	12.77	12.78	3.12E-01	5.22E-01
5.66	5.59	5.79	3.12E-01	5.22E-01
9.26	9.46	9.35	3.12E-01	5.22E-01
6.03	6.02	6.14	3.12E-01	5.22E-01
8.37	8.33	8.44	3.12E-01	5.22E-01
6.82	7.01	7.00	3.13E-01	5.22E-01
3.69	3.77	3.71	3.13E-01	5.22E-01
8.81	8.74	9.03	3.13E-01	5.22E-01
5.60	5.57	5.65	3.13E-01	5.22E-01
12.11	12.24	12.20	3.13E-01	5.22E-01
9.33	9.61	9.47	3.13E-01	5.22E-01
9.63	9.44	9.57	3.13E-01	5.22E-01
4.07	4.06	4.45	3.13E-01	5.23E-01
7.75	7.70	7.60	3.13E-01	5.23E-01
9.78	10.06	9.94	3.13E-01	5.23E-01
3.56	3.82	3.57	3.13E-01	5.23E-01
6.94	6.93	6.78	3.13E-01	5.23E-01
5.63	5.67	5.76	3.13E-01	5.23E-01
11.17	11.40	11.28	3.13E-01	5.23E-01
7.48	7.34	7.45	3.13E-01	5.23E-01
5.19	5.01	4.94	3.13E-01	5.23E-01
7.49	7.49	7.52	3.13E-01	5.23E-01
8.55	8.65	8.63	3.13E-01	5.23E-01
3.57	3.53	3.75	3.13E-01	5.23E-01
8.22	8.63	8.36	3.13E-01	5.23E-01
4.22	4.33	4.23	3.14E-01	5.23E-01
6.88	6.85	6.65	3.14E-01	5.23E-01
8.18	8.15	8.04	3.14E-01	5.23E-01
5.36	5.21	5.35	3.14E-01	5.23E-01
7.54	7.67	7.65	3.14E-01	5.23E-01
3.70	3.73	3.39	3.14E-01	5.23E-01
8.06	8.18	8.15	3.14E-01	5.23E-01
3.49	3.44	3.36	3.14E-01	5.23E-01
3.56	3.56	3.54	3.14E-01	5.23E-01
5.49	5.48	5.26	3.14E-01	5.23E-01
5.69	5.82	5.56	3.14E-01	5.23E-01
3.63	3.72	3.49	3.14E-01	5.24E-01
10.37	10.15	10.23	3.14E-01	5.24E-01
6.96	7.10	7.12	3.14E-01	5.24E-01
4.60	4.63	4.35	3.14E-01	5.24E-01
10.58	10.67	10.67	3.14E-01	5.24E-01

8.57	8.71	8.65	3.14E-01	5.24E-01
5.89	5.87	5.80	3.14E-01	5.24E-01
9.61	9.70	9.67	3.14E-01	5.24E-01
6.68	6.53	6.38	3.14E-01	5.24E-01
8.05	8.14	7.97	3.15E-01	5.24E-01
5.17	5.59	5.53	3.15E-01	5.24E-01
7.29	7.13	7.17	3.15E-01	5.24E-01
3.84	4.19	3.89	3.15E-01	5.24E-01
4.96	5.02	5.06	3.15E-01	5.24E-01
5.55	5.58	5.38	3.15E-01	5.24E-01
8.15	8.00	8.12	3.15E-01	5.24E-01
4.13	4.21	4.26	3.15E-01	5.24E-01
7.23	7.28	7.13	3.15E-01	5.24E-01
7.09	7.46	7.42	3.15E-01	5.24E-01
5.66	5.50	5.66	3.15E-01	5.24E-01
9.96	9.92	9.75	3.15E-01	5.24E-01
5.08	5.36	5.08	3.15E-01	5.24E-01
9.27	8.90	9.22	3.15E-01	5.24E-01
4.84	4.67	4.53	3.15E-01	5.24E-01
4.06	4.08	3.94	3.15E-01	5.24E-01
9.43	9.63	9.69	3.15E-01	5.24E-01
5.20	5.26	5.26	3.15E-01	5.24E-01
7.89	8.10	7.97	3.15E-01	5.24E-01
14.41	14.42	14.39	3.15E-01	5.24E-01
6.51	6.84	6.48	3.15E-01	5.24E-01
7.03	6.83	7.05	3.15E-01	5.24E-01
9.25	9.43	9.31	3.16E-01	5.24E-01
5.84	6.01	5.77	3.16E-01	5.24E-01
7.23	7.22	7.27	3.16E-01	5.24E-01
5.72	5.55	5.68	3.16E-01	5.24E-01
10.90	10.90	10.92	3.16E-01	5.24E-01
6.03	6.36	6.14	3.16E-01	5.24E-01
10.48	10.39	10.54	3.16E-01	5.24E-01
3.65	3.78	3.95	3.16E-01	5.24E-01
8.29	8.24	8.30	3.16E-01	5.24E-01
3.65	3.95	3.54	3.16E-01	5.24E-01
7.43	7.28	7.44	3.16E-01	5.24E-01
8.87	8.70	8.75	3.16E-01	5.24E-01
9.22	9.21	9.31	3.16E-01	5.24E-01
4.17	4.00	4.25	3.16E-01	5.24E-01
9.73	9.93	9.79	3.16E-01	5.24E-01
7.36	7.10	7.21	3.16E-01	5.24E-01
5.42	5.72	5.40	3.16E-01	5.24E-01
5.24	4.91	5.08	3.16E-01	5.24E-01
4.13	4.11	4.19	3.16E-01	5.24E-01
4.56	4.48	4.30	3.16E-01	5.24E-01
8.77	8.67	8.90	3.16E-01	5.24E-01

8.63	8.71	8.60	3.16E-01	5.24E-01
3.26	3.16	3.30	3.16E-01	5.24E-01
5.25	5.11	5.25	3.16E-01	5.24E-01
5.43	5.64	5.60	3.16E-01	5.24E-01
11.54	11.71	11.63	3.16E-01	5.24E-01
6.77	6.79	6.87	3.16E-01	5.24E-01
7.99	8.39	8.17	3.17E-01	5.24E-01
7.92	7.71	8.08	3.17E-01	5.24E-01
7.16	7.39	7.37	3.17E-01	5.24E-01
9.52	9.69	9.53	3.17E-01	5.25E-01
4.74	4.80	4.60	3.17E-01	5.25E-01
6.07	5.97	6.11	3.17E-01	5.25E-01
7.40	7.37	7.37	3.17E-01	5.25E-01
6.34	6.18	6.18	3.17E-01	5.25E-01
10.31	10.37	10.29	3.17E-01	5.25E-01
3.14	3.24	3.23	3.17E-01	5.25E-01
6.78	6.99	6.96	3.17E-01	5.25E-01
3.48	3.75	3.65	3.17E-01	5.25E-01
11.15	11.13	11.25	3.17E-01	5.25E-01
6.54	6.61	6.64	3.17E-01	5.25E-01
11.77	11.97	11.90	3.17E-01	5.25E-01
5.91	5.92	6.02	3.18E-01	5.25E-01
6.91	7.11	7.02	3.18E-01	5.25E-01
7.87	8.10	7.90	3.18E-01	5.25E-01
7.67	7.66	7.74	3.18E-01	5.25E-01
7.11	7.33	7.28	3.18E-01	5.25E-01
9.22	9.00	9.34	3.18E-01	5.25E-01
5.40	5.51	5.66	3.18E-01	5.25E-01
3.53	3.53	3.68	3.18E-01	5.25E-01
13.00	13.07	13.05	3.18E-01	5.25E-01
11.97	12.05	12.00	3.18E-01	5.25E-01
10.72	10.91	10.86	3.18E-01	5.25E-01
7.90	8.03	7.94	3.18E-01	5.25E-01
12.87	12.91	12.81	3.18E-01	5.25E-01
4.17	4.31	4.24	3.18E-01	5.25E-01
6.62	6.61	6.65	3.18E-01	5.25E-01
4.97	5.08	5.07	3.18E-01	5.25E-01
5.36	5.25	5.45	3.18E-01	5.25E-01
6.29	6.36	6.18	3.18E-01	5.25E-01
4.06	4.12	4.28	3.18E-01	5.25E-01
5.08	5.03	5.17	3.18E-01	5.25E-01
8.67	8.75	8.82	3.18E-01	5.25E-01
5.83	5.97	5.97	3.18E-01	5.25E-01
10.69	10.44	10.57	3.18E-01	5.25E-01
6.48	6.70	6.64	3.18E-01	5.25E-01
9.20	9.50	9.45	3.18E-01	5.25E-01
5.47	5.68	5.42	3.18E-01	5.25E-01

7.36	7.49	7.29	3.18E-01	5.25E-01
8.69	8.83	8.72	3.19E-01	5.25E-01
4.11	4.13	4.46	3.19E-01	5.25E-01
6.89	6.83	6.83	3.19E-01	5.25E-01
10.06	10.12	10.14	3.19E-01	5.25E-01
6.70	6.96	6.84	3.19E-01	5.25E-01
6.12	6.26	6.18	3.19E-01	5.25E-01
13.65	13.69	13.75	3.19E-01	5.26E-01
11.39	11.30	11.32	3.19E-01	5.26E-01
3.33	3.59	3.68	3.19E-01	5.26E-01
4.86	4.94	4.88	3.19E-01	5.26E-01
7.00	7.19	7.15	3.19E-01	5.26E-01
4.19	4.25	3.91	3.19E-01	5.26E-01
5.30	5.15	5.50	3.19E-01	5.26E-01
6.40	6.20	6.31	3.19E-01	5.26E-01
6.98	7.05	6.99	3.19E-01	5.26E-01
9.42	9.31	9.43	3.19E-01	5.26E-01
10.02	10.13	10.14	3.19E-01	5.26E-01
7.03	6.95	6.79	3.19E-01	5.26E-01
7.90	8.11	8.04	3.19E-01	5.26E-01
6.56	6.57	6.39	3.19E-01	5.26E-01
3.71	3.55	3.73	3.19E-01	5.26E-01
7.92	8.15	8.13	3.20E-01	5.26E-01
7.79	8.13	8.02	3.20E-01	5.26E-01
9.38	9.48	9.54	3.20E-01	5.26E-01
3.42	3.58	3.73	3.20E-01	5.26E-01
4.69	4.54	4.71	3.20E-01	5.26E-01
7.40	7.59	7.52	3.20E-01	5.26E-01
10.12	10.16	10.09	3.20E-01	5.26E-01
9.49	9.25	9.22	3.20E-01	5.26E-01
4.97	4.92	5.04	3.20E-01	5.26E-01
7.97	7.87	7.93	3.20E-01	5.26E-01
7.16	7.71	7.32	3.20E-01	5.26E-01
4.93	4.83	4.80	3.20E-01	5.26E-01
6.66	7.05	7.05	3.20E-01	5.26E-01
7.00	7.13	7.08	3.20E-01	5.26E-01
6.02	5.92	6.01	3.20E-01	5.26E-01
6.66	6.76	6.68	3.20E-01	5.26E-01
6.66	6.64	6.51	3.20E-01	5.26E-01
4.25	4.14	4.23	3.20E-01	5.26E-01
3.86	3.69	3.84	3.20E-01	5.26E-01
6.61	6.74	6.66	3.20E-01	5.26E-01
4.00	4.21	4.19	3.20E-01	5.26E-01
10.87	10.97	10.98	3.21E-01	5.26E-01
9.80	10.08	9.88	3.21E-01	5.26E-01
4.70	4.93	4.74	3.21E-01	5.26E-01
6.14	5.92	6.18	3.21E-01	5.26E-01

4.60	4.32	4.43	3.21E-01	5.26E-01
5.40	5.48	5.28	3.21E-01	5.26E-01
6.46	6.41	6.24	3.21E-01	5.26E-01
5.06	5.03	4.96	3.21E-01	5.26E-01
5.17	5.10	5.19	3.21E-01	5.26E-01
6.19	5.94	5.98	3.21E-01	5.26E-01
7.55	7.63	7.53	3.21E-01	5.26E-01
7.76	7.71	7.72	3.21E-01	5.26E-01
7.39	7.06	7.42	3.21E-01	5.26E-01
7.41	7.30	7.63	3.21E-01	5.27E-01
6.27	6.47	6.29	3.21E-01	5.27E-01
5.36	6.01	5.57	3.21E-01	5.27E-01
5.97	5.89	6.15	3.21E-01	5.27E-01
7.91	7.76	7.98	3.21E-01	5.27E-01
6.47	6.32	6.34	3.21E-01	5.27E-01
10.16	10.10	10.24	3.21E-01	5.27E-01
4.75	4.86	4.54	3.21E-01	5.27E-01
9.00	8.70	8.66	3.21E-01	5.27E-01
3.26	3.45	3.08	3.21E-01	5.27E-01
5.64	5.61	5.57	3.21E-01	5.27E-01
9.19	9.27	9.20	3.21E-01	5.27E-01
6.47	6.78	6.69	3.21E-01	5.27E-01
7.98	8.18	8.08	3.21E-01	5.27E-01
8.06	8.06	8.05	3.22E-01	5.27E-01
7.77	7.92	7.94	3.22E-01	5.27E-01
4.65	4.69	4.45	3.22E-01	5.27E-01
8.23	8.12	8.09	3.22E-01	5.27E-01
7.45	7.20	7.58	3.22E-01	5.27E-01
8.59	8.74	8.67	3.22E-01	5.27E-01
5.13	5.10	5.32	3.22E-01	5.27E-01
6.46	6.25	6.36	3.22E-01	5.27E-01
7.47	7.69	7.70	3.22E-01	5.27E-01
6.07	6.13	5.99	3.22E-01	5.27E-01
7.25	6.90	7.03	3.22E-01	5.27E-01
11.71	11.42	11.57	3.22E-01	5.27E-01
4.19	4.09	4.20	3.22E-01	5.27E-01
5.28	5.27	5.33	3.22E-01	5.27E-01
9.68	9.81	9.77	3.22E-01	5.27E-01
8.02	7.99	7.99	3.22E-01	5.27E-01
6.43	6.47	6.45	3.22E-01	5.27E-01
3.94	3.95	3.89	3.22E-01	5.27E-01
4.47	4.29	4.21	3.22E-01	5.27E-01
7.30	6.93	7.13	3.23E-01	5.27E-01
4.06	3.89	4.14	3.23E-01	5.27E-01
3.15	3.10	3.36	3.23E-01	5.27E-01
7.49	7.62	7.51	3.23E-01	5.27E-01
9.32	9.56	9.47	3.23E-01	5.27E-01

8.78	8.56	8.79	3.23E-01	5.28E-01
11.33	11.44	11.44	3.23E-01	5.28E-01
4.96	4.99	4.93	3.23E-01	5.28E-01
4.94	5.08	5.19	3.23E-01	5.28E-01
8.02	8.42	7.98	3.23E-01	5.28E-01
5.13	4.93	5.07	3.23E-01	5.28E-01
3.08	3.07	3.11	3.23E-01	5.28E-01
12.38	12.33	12.33	3.23E-01	5.28E-01
7.94	8.03	7.85	3.23E-01	5.28E-01
6.45	6.37	6.56	3.23E-01	5.28E-01
7.15	7.13	7.03	3.23E-01	5.28E-01
6.16	5.91	6.15	3.23E-01	5.28E-01
12.07	12.34	12.20	3.23E-01	5.28E-01
7.38	7.22	7.64	3.23E-01	5.28E-01
10.00	9.99	10.04	3.23E-01	5.28E-01
4.68	4.79	5.06	3.23E-01	5.28E-01
5.57	5.93	5.67	3.23E-01	5.28E-01
3.79	3.51	3.60	3.24E-01	5.28E-01
6.37	6.54	6.69	3.24E-01	5.28E-01
8.12	8.16	7.98	3.24E-01	5.28E-01
10.22	9.98	10.07	3.24E-01	5.28E-01
5.60	5.40	5.70	3.24E-01	5.28E-01
6.98	6.98	7.08	3.24E-01	5.29E-01
7.43	7.29	7.58	3.24E-01	5.29E-01
9.73	9.94	9.81	3.24E-01	5.29E-01
6.03	6.03	5.95	3.24E-01	5.29E-01
9.44	9.55	9.49	3.24E-01	5.29E-01
7.40	7.29	7.47	3.25E-01	5.29E-01
9.50	9.30	9.49	3.25E-01	5.29E-01
10.90	10.96	11.01	3.25E-01	5.29E-01
8.79	8.65	8.93	3.25E-01	5.29E-01
4.66	4.90	4.70	3.25E-01	5.29E-01
7.39	7.25	7.32	3.25E-01	5.29E-01
5.46	5.22	5.24	3.25E-01	5.29E-01
9.43	9.48	9.51	3.25E-01	5.29E-01
3.12	3.01	3.19	3.25E-01	5.29E-01
10.74	10.85	10.80	3.25E-01	5.29E-01
5.55	5.43	5.59	3.25E-01	5.29E-01
5.54	5.70	5.87	3.25E-01	5.29E-01
3.41	3.75	3.50	3.25E-01	5.29E-01
3.42	3.39	3.22	3.25E-01	5.29E-01
8.42	8.25	8.48	3.25E-01	5.29E-01
8.53	8.69	8.49	3.25E-01	5.29E-01
4.16	4.15	4.22	3.25E-01	5.29E-01
13.25	13.27	13.22	3.25E-01	5.29E-01
5.66	5.36	5.71	3.25E-01	5.29E-01
5.97	6.05	6.05	3.25E-01	5.29E-01

4.11	4.38	4.68	3.25E-01	5.29E-01
4.39	4.38	4.54	3.25E-01	5.29E-01
6.68	6.83	6.90	3.25E-01	5.29E-01
7.50	7.47	7.58	3.26E-01	5.29E-01
5.94	5.96	5.93	3.26E-01	5.29E-01
6.67	6.81	6.74	3.26E-01	5.30E-01
8.25	8.45	8.26	3.26E-01	5.30E-01
6.22	6.19	6.27	3.26E-01	5.30E-01
4.30	4.24	4.26	3.26E-01	5.30E-01
6.20	5.83	6.11	3.26E-01	5.30E-01
8.20	8.13	8.33	3.26E-01	5.30E-01
3.64	3.68	3.67	3.26E-01	5.30E-01
8.58	8.83	8.66	3.26E-01	5.30E-01
10.64	10.90	10.82	3.26E-01	5.30E-01
3.70	3.97	3.84	3.26E-01	5.30E-01
10.13	9.86	9.99	3.26E-01	5.30E-01
10.01	10.05	9.92	3.26E-01	5.30E-01
3.45	3.44	3.30	3.26E-01	5.30E-01
6.79	6.70	6.63	3.26E-01	5.30E-01
11.01	10.62	10.92	3.26E-01	5.30E-01
9.85	9.98	9.91	3.26E-01	5.30E-01
4.15	4.04	4.13	3.26E-01	5.30E-01
10.35	10.37	10.52	3.26E-01	5.30E-01
6.72	6.29	6.62	3.26E-01	5.30E-01
4.09	4.20	4.23	3.26E-01	5.30E-01
7.14	7.17	7.01	3.26E-01	5.30E-01
4.76	4.66	4.60	3.26E-01	5.30E-01
5.70	5.82	5.61	3.26E-01	5.30E-01
9.39	9.30	9.40	3.26E-01	5.30E-01
7.54	7.41	7.61	3.27E-01	5.30E-01
7.67	7.65	7.84	3.27E-01	5.30E-01
4.96	4.96	4.72	3.27E-01	5.30E-01
9.80	9.60	9.82	3.27E-01	5.30E-01
7.27	7.34	7.15	3.27E-01	5.30E-01
6.85	7.13	6.95	3.27E-01	5.30E-01
4.16	4.59	4.48	3.27E-01	5.30E-01
10.00	9.84	9.96	3.27E-01	5.30E-01
3.18	3.21	3.18	3.27E-01	5.30E-01
5.51	5.53	5.49	3.27E-01	5.30E-01
11.91	11.91	11.87	3.27E-01	5.30E-01
6.87	6.79	6.79	3.27E-01	5.30E-01
8.34	8.30	8.48	3.27E-01	5.30E-01
8.42	8.48	8.54	3.27E-01	5.30E-01
8.10	8.17	8.12	3.27E-01	5.30E-01
8.09	8.20	8.10	3.27E-01	5.30E-01
4.44	4.37	4.48	3.27E-01	5.30E-01
3.61	3.57	3.49	3.27E-01	5.30E-01

8.57	8.32	8.57	3.27E-01	5.30E-01
7.91	8.05	7.98	3.27E-01	5.30E-01
5.48	5.61	5.35	3.27E-01	5.30E-01
5.93	5.82	6.03	3.27E-01	5.30E-01
7.05	7.00	7.20	3.27E-01	5.30E-01
6.09	6.11	6.02	3.27E-01	5.30E-01
8.64	8.97	8.76	3.28E-01	5.30E-01
5.91	5.82	5.98	3.28E-01	5.30E-01
3.82	3.86	4.07	3.28E-01	5.30E-01
7.67	7.94	7.72	3.28E-01	5.30E-01
12.52	12.68	12.56	3.28E-01	5.30E-01
5.91	6.00	6.03	3.28E-01	5.30E-01
6.13	6.32	6.23	3.28E-01	5.30E-01
9.10	9.00	8.99	3.28E-01	5.30E-01
5.86	5.93	5.90	3.28E-01	5.30E-01
7.09	7.11	6.91	3.28E-01	5.30E-01
3.55	3.55	3.63	3.28E-01	5.30E-01
8.02	7.99	8.27	3.28E-01	5.30E-01
4.87	4.76	4.96	3.28E-01	5.30E-01
9.66	9.65	9.53	3.28E-01	5.30E-01
8.73	8.96	8.85	3.28E-01	5.30E-01
11.88	12.13	11.85	3.28E-01	5.30E-01
6.19	6.24	6.23	3.28E-01	5.30E-01
10.31	10.94	10.16	3.28E-01	5.30E-01
8.24	8.02	8.27	3.28E-01	5.30E-01
7.68	7.66	7.73	3.28E-01	5.30E-01
3.62	3.64	3.55	3.28E-01	5.30E-01
4.86	4.80	5.13	3.28E-01	5.30E-01
7.44	7.34	7.07	3.28E-01	5.30E-01
4.43	4.39	4.32	3.28E-01	5.30E-01
9.94	10.21	10.08	3.28E-01	5.30E-01
3.84	3.60	3.58	3.28E-01	5.30E-01
6.77	6.68	6.72	3.29E-01	5.30E-01
14.51	14.51	14.54	3.29E-01	5.30E-01
9.68	9.75	9.54	3.29E-01	5.30E-01
5.30	5.11	5.12	3.29E-01	5.30E-01
4.10	4.31	4.15	3.29E-01	5.30E-01
4.45	4.69	4.58	3.29E-01	5.30E-01
10.16	10.44	10.31	3.29E-01	5.30E-01
10.47	9.77	10.64	3.29E-01	5.30E-01
8.74	8.84	8.72	3.29E-01	5.30E-01
6.46	6.39	6.53	3.29E-01	5.31E-01
7.27	7.19	7.24	3.29E-01	5.31E-01
8.38	7.90	8.13	3.29E-01	5.31E-01
3.74	3.87	3.80	3.29E-01	5.31E-01
8.84	8.69	8.83	3.29E-01	5.31E-01
4.90	4.93	5.15	3.29E-01	5.31E-01

9.67	9.87	9.81	3.29E-01	5.31E-01
8.13	7.84	7.89	3.30E-01	5.31E-01
4.75	4.94	4.86	3.30E-01	5.31E-01
7.40	7.37	7.14	3.30E-01	5.31E-01
9.57	9.50	9.48	3.30E-01	5.31E-01
6.17	5.88	6.37	3.30E-01	5.31E-01
7.98	8.13	7.99	3.30E-01	5.31E-01
4.73	4.78	4.87	3.30E-01	5.31E-01
6.09	6.24	6.33	3.30E-01	5.31E-01
6.43	6.42	6.43	3.30E-01	5.31E-01
3.66	3.76	3.72	3.30E-01	5.31E-01
9.16	9.31	9.30	3.30E-01	5.31E-01
4.30	4.19	4.29	3.30E-01	5.31E-01
6.36	6.59	6.32	3.30E-01	5.31E-01
7.64	7.59	7.63	3.30E-01	5.31E-01
7.87	7.95	7.91	3.30E-01	5.31E-01
6.30	6.29	6.24	3.30E-01	5.31E-01
8.84	8.43	8.70	3.30E-01	5.31E-01
8.07	7.78	8.03	3.30E-01	5.31E-01
3.82	3.59	3.78	3.30E-01	5.31E-01
7.85	8.07	8.01	3.30E-01	5.31E-01
6.34	6.12	6.19	3.30E-01	5.31E-01
7.05	7.29	7.07	3.30E-01	5.31E-01
7.07	6.98	6.99	3.30E-01	5.31E-01
7.64	8.02	7.69	3.31E-01	5.32E-01
7.40	7.44	7.48	3.31E-01	5.32E-01
4.97	5.15	4.95	3.31E-01	5.32E-01
5.80	5.76	5.67	3.31E-01	5.32E-01
4.35	4.34	4.47	3.31E-01	5.32E-01
7.66	7.56	7.98	3.31E-01	5.32E-01
9.84	10.06	9.94	3.31E-01	5.32E-01
8.76	8.89	9.02	3.31E-01	5.32E-01
7.90	8.03	8.10	3.31E-01	5.32E-01
10.32	10.63	10.34	3.31E-01	5.32E-01
4.04	3.95	4.33	3.31E-01	5.32E-01
10.03	9.84	9.81	3.31E-01	5.32E-01
14.43	14.44	14.43	3.31E-01	5.32E-01
7.23	7.45	7.33	3.31E-01	5.32E-01
5.54	5.67	5.49	3.32E-01	5.32E-01
6.04	6.02	5.98	3.32E-01	5.32E-01
3.93	3.93	3.94	3.32E-01	5.32E-01
3.24	3.11	3.05	3.32E-01	5.33E-01
4.25	4.29	4.01	3.32E-01	5.33E-01
4.31	4.05	4.22	3.32E-01	5.33E-01
4.23	4.25	4.28	3.32E-01	5.33E-01
5.55	5.77	5.59	3.32E-01	5.33E-01
6.40	6.32	6.38	3.32E-01	5.33E-01

4.24	4.44	4.39	3.32E-01	5.33E-01
4.70	4.81	4.62	3.32E-01	5.33E-01
10.95	10.69	10.91	3.32E-01	5.33E-01
9.62	9.73	9.62	3.32E-01	5.33E-01
8.31	8.07	8.20	3.32E-01	5.33E-01
5.06	5.00	4.86	3.32E-01	5.33E-01
10.17	10.53	10.27	3.32E-01	5.33E-01
7.09	6.94	7.44	3.32E-01	5.33E-01
4.52	4.41	4.31	3.33E-01	5.33E-01
5.93	5.92	5.87	3.33E-01	5.33E-01
12.60	12.65	12.71	3.33E-01	5.33E-01
3.97	4.12	4.02	3.33E-01	5.33E-01
7.87	7.72	7.62	3.33E-01	5.33E-01
6.86	6.89	6.94	3.33E-01	5.33E-01
6.96	6.56	6.80	3.33E-01	5.33E-01
8.26	8.35	8.41	3.33E-01	5.33E-01
7.31	7.12	7.18	3.33E-01	5.33E-01
3.29	3.22	3.15	3.33E-01	5.33E-01
7.81	8.31	7.88	3.33E-01	5.33E-01
6.77	6.41	6.54	3.33E-01	5.33E-01
3.48	3.50	3.59	3.33E-01	5.33E-01
6.40	6.49	6.49	3.34E-01	5.34E-01
7.57	7.86	7.49	3.34E-01	5.34E-01
5.45	5.39	5.65	3.34E-01	5.34E-01
7.94	7.84	7.95	3.34E-01	5.34E-01
7.91	8.13	8.15	3.34E-01	5.34E-01
7.20	7.47	7.57	3.34E-01	5.34E-01
5.97	5.89	5.98	3.34E-01	5.34E-01
6.06	6.12	6.02	3.34E-01	5.34E-01
7.83	7.91	8.02	3.34E-01	5.34E-01
11.12	10.97	11.09	3.34E-01	5.34E-01
9.46	9.69	9.58	3.34E-01	5.34E-01
6.63	6.75	6.70	3.34E-01	5.34E-01
3.71	3.74	3.87	3.34E-01	5.34E-01
6.13	6.06	6.24	3.34E-01	5.34E-01
9.00	8.96	9.07	3.34E-01	5.34E-01
4.00	3.96	3.98	3.34E-01	5.34E-01
6.51	6.26	6.40	3.34E-01	5.34E-01
3.61	3.60	3.56	3.34E-01	5.34E-01
7.36	7.18	7.26	3.34E-01	5.34E-01
6.49	6.52	6.32	3.34E-01	5.34E-01
6.52	6.49	6.54	3.34E-01	5.34E-01
6.30	6.19	6.38	3.35E-01	5.35E-01
3.86	3.92	3.97	3.35E-01	5.35E-01
11.88	11.96	11.95	3.35E-01	5.35E-01
6.90	6.94	6.94	3.35E-01	5.35E-01
4.50	4.61	4.56	3.35E-01	5.35E-01

10.67	10.79	10.71	3.35E-01	5.35E-01
12.31	12.31	12.43	3.35E-01	5.35E-01
5.71	5.59	5.78	3.35E-01	5.35E-01
3.81	3.77	3.78	3.35E-01	5.35E-01
5.65	6.02	5.99	3.35E-01	5.35E-01
7.83	8.38	8.02	3.35E-01	5.35E-01
8.74	8.57	8.69	3.35E-01	5.35E-01
7.10	7.01	6.89	3.35E-01	5.35E-01
5.82	5.79	5.83	3.35E-01	5.35E-01
5.38	5.35	5.20	3.35E-01	5.35E-01
3.65	3.66	4.00	3.35E-01	5.35E-01
10.04	10.21	10.16	3.35E-01	5.35E-01
7.22	7.29	7.34	3.35E-01	5.35E-01
7.66	7.70	7.92	3.35E-01	5.35E-01
3.65	3.53	3.76	3.35E-01	5.35E-01
6.21	6.35	6.35	3.35E-01	5.35E-01
8.46	8.64	8.56	3.36E-01	5.35E-01
5.61	5.45	5.81	3.36E-01	5.35E-01
9.81	9.94	10.00	3.36E-01	5.35E-01
9.29	9.30	9.34	3.36E-01	5.35E-01
7.87	7.94	7.95	3.36E-01	5.35E-01
4.81	5.18	5.16	3.36E-01	5.36E-01
10.98	10.97	11.03	3.36E-01	5.36E-01
8.13	7.82	8.03	3.36E-01	5.36E-01
4.15	4.49	4.51	3.36E-01	5.36E-01
4.17	4.17	4.16	3.36E-01	5.36E-01
6.73	6.83	6.78	3.36E-01	5.36E-01
6.05	6.00	5.96	3.36E-01	5.36E-01
8.41	8.53	8.56	3.36E-01	5.36E-01
5.22	5.13	5.41	3.36E-01	5.36E-01
7.14	7.12	6.96	3.36E-01	5.36E-01
9.96	10.09	10.06	3.36E-01	5.36E-01
9.37	9.25	9.39	3.37E-01	5.36E-01
7.81	7.80	7.89	3.37E-01	5.36E-01
5.77	5.63	6.04	3.37E-01	5.36E-01
4.66	4.36	4.49	3.37E-01	5.36E-01
8.26	8.11	8.11	3.37E-01	5.36E-01
8.07	8.21	8.18	3.37E-01	5.36E-01
9.43	9.66	9.45	3.37E-01	5.36E-01
5.24	5.17	5.43	3.37E-01	5.36E-01
9.09	9.13	9.16	3.37E-01	5.36E-01
8.22	8.35	8.44	3.37E-01	5.36E-01
4.70	4.70	4.48	3.37E-01	5.36E-01
5.06	4.70	5.12	3.37E-01	5.36E-01
3.11	3.24	3.33	3.37E-01	5.36E-01
3.45	3.58	3.56	3.37E-01	5.36E-01
4.44	4.64	4.73	3.37E-01	5.36E-01

5.19	4.98	5.12	3.38E-01	5.36E-01
4.26	4.31	4.08	3.38E-01	5.36E-01
8.45	8.49	8.45	3.38E-01	5.36E-01
9.32	9.16	9.34	3.38E-01	5.36E-01
5.39	5.40	5.73	3.38E-01	5.36E-01
10.95	11.03	10.99	3.38E-01	5.37E-01
6.07	6.21	6.09	3.38E-01	5.37E-01
10.07	10.17	10.23	3.38E-01	5.37E-01
6.37	6.32	6.33	3.38E-01	5.37E-01
6.45	6.69	6.75	3.38E-01	5.37E-01
6.21	5.93	5.87	3.38E-01	5.37E-01
5.09	5.00	5.04	3.38E-01	5.37E-01
9.14	8.95	8.99	3.38E-01	5.37E-01
5.03	5.16	4.97	3.38E-01	5.37E-01
7.17	7.55	7.48	3.38E-01	5.37E-01
8.72	8.58	8.57	3.38E-01	5.37E-01
6.74	6.82	6.73	3.38E-01	5.37E-01
7.86	7.79	7.84	3.38E-01	5.37E-01
9.92	9.59	9.81	3.38E-01	5.37E-01
6.18	6.05	6.05	3.39E-01	5.37E-01
7.98	8.25	8.06	3.39E-01	5.37E-01
5.80	5.55	5.34	3.39E-01	5.37E-01
8.04	7.66	8.11	3.39E-01	5.37E-01
11.18	11.40	11.26	3.39E-01	5.37E-01
4.63	4.43	4.36	3.39E-01	5.37E-01
7.31	7.17	7.36	3.39E-01	5.37E-01
6.82	6.84	6.83	3.39E-01	5.37E-01
9.92	10.11	10.03	3.39E-01	5.37E-01
9.25	9.40	9.34	3.39E-01	5.37E-01
6.66	6.51	6.32	3.39E-01	5.37E-01
7.76	7.67	7.75	3.39E-01	5.37E-01
7.40	7.45	7.40	3.39E-01	5.37E-01
7.14	7.25	6.93	3.39E-01	5.37E-01
3.61	3.91	3.49	3.39E-01	5.37E-01
6.79	6.59	6.68	3.39E-01	5.37E-01
7.67	8.05	7.66	3.39E-01	5.37E-01
3.67	3.42	3.61	3.39E-01	5.37E-01
7.25	7.35	7.29	3.39E-01	5.37E-01
6.14	5.63	5.65	3.39E-01	5.37E-01
5.41	5.22	5.28	3.39E-01	5.37E-01
4.15	4.22	4.23	3.39E-01	5.37E-01
5.39	5.11	5.05	3.39E-01	5.37E-01
3.57	3.57	3.60	3.39E-01	5.37E-01
8.23	8.15	8.31	3.39E-01	5.37E-01
7.04	7.25	6.96	3.39E-01	5.37E-01
5.97	5.78	5.90	3.40E-01	5.37E-01
6.62	6.48	6.45	3.40E-01	5.37E-01

8.33	8.48	8.33	3.40E-01	5.37E-01
5.16	5.51	5.55	3.40E-01	5.37E-01
6.36	6.45	6.39	3.40E-01	5.37E-01
4.62	4.95	4.96	3.40E-01	5.37E-01
7.76	7.88	7.86	3.40E-01	5.37E-01
6.79	6.75	6.87	3.40E-01	5.37E-01
6.66	7.16	6.98	3.40E-01	5.37E-01
8.17	8.00	8.15	3.40E-01	5.38E-01
8.34	8.11	8.43	3.40E-01	5.38E-01
8.74	8.72	8.78	3.40E-01	5.38E-01
3.53	3.68	3.47	3.40E-01	5.38E-01
11.01	11.12	10.99	3.40E-01	5.38E-01
3.26	3.28	3.47	3.40E-01	5.38E-01
6.88	6.62	6.77	3.40E-01	5.38E-01
9.27	9.35	9.44	3.40E-01	5.38E-01
11.88	11.62	11.89	3.40E-01	5.38E-01
4.68	4.72	4.75	3.40E-01	5.38E-01
8.07	8.11	8.35	3.40E-01	5.38E-01
5.38	5.42	5.48	3.40E-01	5.38E-01
7.14	6.95	7.15	3.40E-01	5.38E-01
6.38	6.66	6.42	3.40E-01	5.38E-01
7.00	7.11	6.94	3.41E-01	5.38E-01
6.40	6.51	6.31	3.41E-01	5.38E-01
7.02	7.30	7.01	3.41E-01	5.38E-01
9.79	9.99	9.83	3.41E-01	5.38E-01
9.11	8.99	9.08	3.41E-01	5.38E-01
3.90	3.54	3.90	3.41E-01	5.38E-01
5.48	5.66	5.63	3.41E-01	5.38E-01
9.51	9.70	9.63	3.41E-01	5.38E-01
8.32	8.20	8.37	3.41E-01	5.38E-01
10.68	10.74	10.81	3.41E-01	5.38E-01
11.14	10.91	11.13	3.41E-01	5.38E-01
8.94	8.82	9.05	3.41E-01	5.38E-01
9.94	9.83	9.83	3.41E-01	5.38E-01
10.28	10.04	10.19	3.41E-01	5.38E-01
4.37	4.44	4.57	3.41E-01	5.38E-01
3.60	3.66	3.57	3.41E-01	5.38E-01
6.95	6.83	6.95	3.41E-01	5.38E-01
4.84	4.76	4.57	3.41E-01	5.38E-01
5.01	4.93	4.80	3.41E-01	5.38E-01
8.83	8.77	8.77	3.41E-01	5.38E-01
6.20	6.20	6.19	3.41E-01	5.38E-01
4.88	4.83	4.82	3.41E-01	5.38E-01
6.08	5.80	5.75	3.42E-01	5.38E-01
8.28	8.24	8.29	3.42E-01	5.38E-01
4.68	4.53	4.82	3.42E-01	5.38E-01
6.07	6.18	6.17	3.42E-01	5.38E-01

10.81	11.04	10.93	3.42E-01	5.38E-01
4.21	4.13	4.04	3.42E-01	5.38E-01
10.45	10.70	10.66	3.42E-01	5.38E-01
7.94	8.07	8.10	3.42E-01	5.38E-01
4.66	4.74	4.78	3.42E-01	5.38E-01
11.26	11.36	11.30	3.42E-01	5.38E-01
4.27	4.40	4.05	3.42E-01	5.38E-01
3.05	3.31	3.26	3.42E-01	5.38E-01
3.74	3.36	3.66	3.42E-01	5.39E-01
8.02	7.98	8.19	3.42E-01	5.39E-01
3.64	3.82	3.78	3.42E-01	5.39E-01
9.79	9.48	9.77	3.42E-01	5.39E-01
8.64	8.87	8.87	3.42E-01	5.39E-01
10.77	10.94	10.83	3.42E-01	5.39E-01
9.05	9.32	9.17	3.42E-01	5.39E-01
7.44	7.37	7.43	3.43E-01	5.39E-01
12.48	12.35	12.36	3.43E-01	5.39E-01
3.67	3.71	3.75	3.43E-01	5.39E-01
6.48	6.75	6.57	3.43E-01	5.39E-01
6.70	6.92	6.92	3.43E-01	5.39E-01
8.17	8.21	8.23	3.43E-01	5.39E-01
5.32	5.65	5.37	3.43E-01	5.39E-01
8.79	9.02	8.76	3.43E-01	5.39E-01
8.48	8.57	8.51	3.43E-01	5.39E-01
9.21	8.89	9.09	3.43E-01	5.39E-01
12.80	13.07	12.98	3.43E-01	5.39E-01
3.82	3.74	3.84	3.43E-01	5.39E-01
4.55	4.46	4.76	3.43E-01	5.39E-01
5.81	5.72	5.68	3.43E-01	5.39E-01
7.85	7.92	7.88	3.43E-01	5.39E-01
3.83	3.62	3.65	3.43E-01	5.39E-01
8.82	8.70	8.75	3.43E-01	5.39E-01
11.75	11.72	11.78	3.43E-01	5.39E-01
12.31	12.20	12.23	3.43E-01	5.39E-01
7.60	7.58	7.59	3.43E-01	5.39E-01
3.60	3.45	3.40	3.43E-01	5.39E-01
7.27	7.56	7.29	3.44E-01	5.39E-01
7.51	7.39	7.59	3.44E-01	5.39E-01
7.31	7.34	7.17	3.44E-01	5.39E-01
6.63	6.98	6.82	3.44E-01	5.39E-01
8.59	8.61	8.62	3.44E-01	5.39E-01
11.26	11.27	11.19	3.44E-01	5.39E-01
6.07	5.78	5.83	3.44E-01	5.39E-01
6.47	6.67	6.79	3.44E-01	5.39E-01
6.63	6.41	6.32	3.44E-01	5.39E-01
9.68	10.04	9.94	3.44E-01	5.39E-01
6.78	6.81	6.83	3.44E-01	5.39E-01

6.06	6.11	6.05	3.44E-01	5.39E-01
3.33	3.33	3.36	3.44E-01	5.39E-01
9.70	9.39	9.46	3.44E-01	5.39E-01
9.23	8.74	8.94	3.44E-01	5.39E-01
3.79	3.56	3.52	3.44E-01	5.39E-01
8.67	8.80	8.81	3.44E-01	5.39E-01
3.93	4.05	3.91	3.44E-01	5.40E-01
8.79	8.60	8.73	3.44E-01	5.40E-01
7.92	8.01	8.06	3.44E-01	5.40E-01
12.11	12.26	12.22	3.44E-01	5.40E-01
9.02	9.16	9.18	3.45E-01	5.40E-01
12.52	12.72	12.65	3.45E-01	5.40E-01
6.95	7.02	6.90	3.45E-01	5.40E-01
10.43	10.28	10.33	3.45E-01	5.40E-01
3.72	3.30	3.55	3.45E-01	5.40E-01
7.22	6.95	7.01	3.45E-01	5.40E-01
6.28	6.30	6.16	3.45E-01	5.40E-01
7.34	7.56	7.47	3.45E-01	5.40E-01
11.36	11.49	11.41	3.45E-01	5.40E-01
4.28	4.30	4.60	3.45E-01	5.40E-01
4.59	4.57	4.95	3.45E-01	5.40E-01
12.11	12.44	12.02	3.45E-01	5.40E-01
6.50	6.69	6.61	3.45E-01	5.40E-01
4.38	4.27	4.45	3.45E-01	5.40E-01
8.28	8.48	8.35	3.45E-01	5.40E-01
8.87	8.90	9.00	3.45E-01	5.40E-01
6.53	6.63	6.56	3.45E-01	5.40E-01
5.25	5.07	5.11	3.45E-01	5.40E-01
6.92	6.84	6.91	3.46E-01	5.40E-01
5.40	5.23	5.53	3.46E-01	5.40E-01
5.78	5.79	5.66	3.46E-01	5.40E-01
7.64	7.80	7.72	3.46E-01	5.40E-01
5.64	5.36	5.44	3.46E-01	5.41E-01
10.84	10.94	10.85	3.46E-01	5.41E-01
8.71	8.70	8.81	3.46E-01	5.41E-01
4.04	4.00	4.12	3.46E-01	5.41E-01
4.64	4.86	4.48	3.46E-01	5.41E-01
5.81	5.66	5.84	3.46E-01	5.41E-01
7.66	7.79	7.55	3.46E-01	5.41E-01
6.45	6.31	6.35	3.46E-01	5.41E-01
7.50	7.48	7.78	3.46E-01	5.41E-01
10.65	10.46	10.46	3.46E-01	5.41E-01
5.02	4.88	4.97	3.46E-01	5.41E-01
10.82	10.89	11.01	3.46E-01	5.41E-01
3.47	3.53	3.82	3.46E-01	5.41E-01
9.73	9.78	9.78	3.46E-01	5.41E-01
6.04	6.03	5.92	3.47E-01	5.41E-01

7.13	7.17	7.09	3.47E-01	5.41E-01
8.09	8.26	8.15	3.47E-01	5.41E-01
10.32	10.07	10.31	3.47E-01	5.41E-01
6.62	6.53	6.82	3.47E-01	5.41E-01
4.70	4.81	4.88	3.47E-01	5.41E-01
8.35	8.77	8.47	3.47E-01	5.41E-01
3.59	3.44	3.65	3.47E-01	5.41E-01
8.97	9.28	9.02	3.47E-01	5.41E-01
5.41	5.33	5.25	3.47E-01	5.41E-01
4.54	4.83	4.63	3.47E-01	5.41E-01
5.56	5.57	5.51	3.47E-01	5.41E-01
8.36	8.26	8.45	3.47E-01	5.41E-01
5.23	5.18	5.14	3.47E-01	5.41E-01
6.22	6.06	5.93	3.47E-01	5.41E-01
6.70	6.42	6.63	3.47E-01	5.41E-01
3.61	3.76	3.42	3.47E-01	5.41E-01
7.93	7.93	8.05	3.47E-01	5.41E-01
4.91	5.11	4.85	3.47E-01	5.41E-01
6.08	5.97	5.86	3.47E-01	5.41E-01
6.93	6.98	6.97	3.47E-01	5.41E-01
7.56	7.36	7.47	3.47E-01	5.41E-01
11.58	11.50	11.50	3.47E-01	5.41E-01
8.25	8.29	8.21	3.47E-01	5.41E-01
5.82	6.11	5.93	3.48E-01	5.41E-01
6.46	6.19	6.43	3.48E-01	5.41E-01
3.38	3.29	3.48	3.48E-01	5.41E-01
6.85	6.93	6.83	3.48E-01	5.41E-01
10.30	10.39	10.48	3.48E-01	5.41E-01
9.90	10.17	10.07	3.48E-01	5.42E-01
4.35	4.27	4.78	3.48E-01	5.42E-01
9.41	9.06	9.29	3.48E-01	5.42E-01
11.24	10.94	11.20	3.48E-01	5.42E-01
7.64	7.91	7.85	3.48E-01	5.42E-01
8.27	8.29	8.34	3.48E-01	5.42E-01
9.12	9.07	9.16	3.48E-01	5.42E-01
7.40	7.42	7.43	3.48E-01	5.42E-01
12.98	12.84	12.97	3.48E-01	5.42E-01
9.19	9.33	9.32	3.48E-01	5.42E-01
9.54	9.33	9.43	3.48E-01	5.42E-01
12.17	12.21	12.28	3.48E-01	5.42E-01
5.95	5.75	5.60	3.48E-01	5.42E-01
10.03	10.39	10.10	3.48E-01	5.42E-01
3.52	3.61	3.64	3.48E-01	5.42E-01
9.97	9.80	9.93	3.48E-01	5.42E-01
7.41	7.59	7.33	3.49E-01	5.42E-01
9.21	9.39	9.29	3.49E-01	5.42E-01
3.96	4.04	4.09	3.49E-01	5.42E-01

4.61	4.53	4.71	3.49E-01	5.42E-01
4.32	4.36	4.42	3.49E-01	5.42E-01
10.24	10.08	10.02	3.49E-01	5.42E-01
3.01	3.12	3.15	3.49E-01	5.42E-01
7.40	7.35	7.25	3.49E-01	5.42E-01
6.44	6.75	6.63	3.49E-01	5.42E-01
9.55	9.75	9.62	3.49E-01	5.42E-01
8.70	8.92	8.70	3.49E-01	5.42E-01
5.79	5.59	5.66	3.49E-01	5.42E-01
12.35	12.49	12.39	3.49E-01	5.42E-01
3.44	3.50	3.51	3.49E-01	5.42E-01
7.19	7.20	7.04	3.49E-01	5.42E-01
9.84	9.85	9.98	3.49E-01	5.42E-01
5.27	5.20	5.40	3.49E-01	5.42E-01
6.81	6.85	6.76	3.49E-01	5.42E-01
3.60	3.93	3.59	3.49E-01	5.42E-01
9.24	9.35	9.30	3.49E-01	5.42E-01
4.49	4.40	4.42	3.49E-01	5.42E-01
5.37	5.14	5.41	3.49E-01	5.42E-01
4.06	4.13	3.86	3.49E-01	5.42E-01
3.49	3.48	3.57	3.49E-01	5.42E-01
6.45	6.06	6.14	3.49E-01	5.42E-01
6.60	6.68	6.42	3.49E-01	5.42E-01
11.53	11.56	11.53	3.49E-01	5.42E-01
14.71	14.73	14.75	3.49E-01	5.42E-01
8.86	8.89	9.00	3.49E-01	5.42E-01
3.74	3.56	3.59	3.49E-01	5.42E-01
6.99	6.92	6.70	3.49E-01	5.42E-01
6.29	6.12	5.96	3.49E-01	5.42E-01
3.83	3.83	3.90	3.50E-01	5.42E-01
5.10	4.78	5.15	3.50E-01	5.42E-01
9.06	9.25	9.21	3.50E-01	5.42E-01
4.66	4.57	4.61	3.50E-01	5.42E-01
13.96	14.05	13.96	3.50E-01	5.42E-01
7.68	8.03	7.79	3.50E-01	5.42E-01
5.23	5.23	5.01	3.50E-01	5.42E-01
3.76	3.80	3.57	3.50E-01	5.42E-01
11.50	11.15	11.47	3.50E-01	5.42E-01
7.62	7.52	7.69	3.50E-01	5.42E-01
14.30	14.40	14.32	3.50E-01	5.42E-01
5.34	5.26	5.11	3.50E-01	5.42E-01
7.96	7.93	8.15	3.50E-01	5.42E-01
4.42	4.34	4.41	3.50E-01	5.42E-01
7.25	7.36	6.98	3.50E-01	5.42E-01
8.91	8.94	8.90	3.51E-01	5.43E-01
3.24	3.30	3.36	3.51E-01	5.43E-01
5.25	5.17	5.31	3.51E-01	5.43E-01

7.60	7.62	7.69	3.51E-01	5.43E-01
9.15	9.22	9.36	3.51E-01	5.43E-01
6.84	7.03	6.99	3.51E-01	5.43E-01
3.60	3.68	4.39	3.51E-01	5.43E-01
10.37	10.57	10.46	3.51E-01	5.43E-01
6.52	6.46	6.61	3.51E-01	5.43E-01
5.72	5.83	5.86	3.51E-01	5.43E-01
4.21	4.13	4.21	3.51E-01	5.43E-01
8.14	8.30	8.22	3.51E-01	5.43E-01
8.87	8.60	8.78	3.51E-01	5.43E-01
3.88	3.54	3.55	3.51E-01	5.43E-01
6.61	6.51	6.50	3.51E-01	5.43E-01
3.83	3.75	3.81	3.51E-01	5.43E-01
3.90	3.88	3.99	3.51E-01	5.43E-01
5.32	5.32	5.26	3.51E-01	5.43E-01
5.14	5.08	5.17	3.51E-01	5.43E-01
4.14	3.77	4.13	3.52E-01	5.43E-01
10.98	10.98	10.98	3.52E-01	5.43E-01
8.76	8.55	8.66	3.52E-01	5.43E-01
7.10	6.89	6.96	3.52E-01	5.43E-01
5.03	4.81	4.82	3.52E-01	5.43E-01
8.31	8.23	8.15	3.52E-01	5.44E-01
3.89	3.96	3.95	3.52E-01	5.44E-01
5.29	5.18	5.30	3.52E-01	5.44E-01
5.59	5.55	5.65	3.52E-01	5.44E-01
9.08	9.27	9.00	3.52E-01	5.44E-01
6.09	6.11	6.20	3.52E-01	5.44E-01
7.93	7.98	8.01	3.52E-01	5.44E-01
7.00	6.77	6.94	3.52E-01	5.44E-01
4.72	5.65	4.84	3.53E-01	5.44E-01
10.51	10.41	10.43	3.53E-01	5.44E-01
10.45	10.49	10.53	3.53E-01	5.44E-01
4.98	5.07	5.03	3.53E-01	5.44E-01
9.12	9.18	9.26	3.53E-01	5.44E-01
4.10	4.11	3.96	3.53E-01	5.44E-01
6.69	6.81	6.79	3.53E-01	5.44E-01
3.58	3.63	3.80	3.53E-01	5.44E-01
7.05	6.98	7.00	3.53E-01	5.44E-01
5.69	5.31	5.38	3.53E-01	5.44E-01
8.35	8.45	8.45	3.53E-01	5.44E-01
8.76	9.05	8.86	3.53E-01	5.44E-01
7.28	7.31	7.31	3.53E-01	5.44E-01
6.86	7.03	7.20	3.53E-01	5.44E-01
7.75	7.78	7.77	3.53E-01	5.44E-01
6.18	6.41	6.22	3.53E-01	5.44E-01
9.83	9.64	9.82	3.53E-01	5.44E-01
6.46	6.12	6.44	3.53E-01	5.44E-01

11.41	11.45	11.50	3.53E-01	5.44E-01
6.91	7.14	6.82	3.53E-01	5.44E-01
5.00	4.78	4.62	3.53E-01	5.44E-01
8.32	8.08	8.18	3.53E-01	5.44E-01
4.39	4.65	4.57	3.53E-01	5.44E-01
10.54	10.49	10.60	3.54E-01	5.44E-01
4.89	4.86	4.79	3.54E-01	5.44E-01
6.54	6.31	6.80	3.54E-01	5.44E-01
7.13	6.96	7.08	3.54E-01	5.44E-01
8.41	8.35	8.34	3.54E-01	5.44E-01
7.43	7.42	7.69	3.54E-01	5.44E-01
3.95	4.00	3.97	3.54E-01	5.44E-01
3.94	4.32	4.07	3.54E-01	5.45E-01
5.45	5.25	5.29	3.54E-01	5.45E-01
6.43	6.72	6.68	3.54E-01	5.45E-01
6.21	6.20	6.24	3.54E-01	5.45E-01
6.57	6.58	6.73	3.54E-01	5.45E-01
7.71	7.49	7.72	3.54E-01	5.45E-01
3.98	3.86	4.07	3.54E-01	5.45E-01
3.46	3.36	3.47	3.54E-01	5.45E-01
3.73	3.82	3.75	3.54E-01	5.45E-01
6.12	6.27	5.71	3.54E-01	5.45E-01
3.85	4.05	4.08	3.54E-01	5.45E-01
11.08	11.19	11.11	3.54E-01	5.45E-01
4.43	4.36	4.36	3.54E-01	5.45E-01
4.53	4.40	4.52	3.55E-01	5.45E-01
8.28	8.59	8.32	3.55E-01	5.45E-01
5.00	5.28	5.10	3.55E-01	5.45E-01
6.06	5.80	6.06	3.55E-01	5.45E-01
3.63	3.65	3.63	3.55E-01	5.45E-01
8.61	8.75	8.82	3.55E-01	5.45E-01
3.08	3.25	3.36	3.55E-01	5.45E-01
3.11	3.12	3.05	3.55E-01	5.45E-01
3.19	3.32	3.33	3.55E-01	5.45E-01
3.96	3.86	3.76	3.55E-01	5.45E-01
4.97	4.89	4.79	3.55E-01	5.45E-01
4.51	4.38	4.49	3.55E-01	5.45E-01
5.59	5.47	5.45	3.55E-01	5.45E-01
5.51	5.41	5.38	3.55E-01	5.45E-01
8.35	8.19	8.30	3.55E-01	5.45E-01
4.25	4.24	4.37	3.55E-01	5.45E-01
4.05	3.99	3.76	3.55E-01	5.45E-01
11.47	11.68	11.65	3.55E-01	5.45E-01
7.61	7.56	7.88	3.55E-01	5.45E-01
3.94	4.00	3.65	3.55E-01	5.45E-01
7.67	7.72	7.64	3.55E-01	5.45E-01
6.85	6.74	6.81	3.55E-01	5.45E-01

9.31	9.19	9.56	3.55E-01	5.45E-01
9.17	9.37	9.34	3.55E-01	5.45E-01
5.30	5.36	5.37	3.55E-01	5.45E-01
6.47	6.31	6.47	3.55E-01	5.45E-01
7.09	7.12	7.07	3.56E-01	5.45E-01
3.94	3.89	3.85	3.56E-01	5.45E-01
10.44	10.64	10.53	3.56E-01	5.45E-01
3.34	3.28	3.70	3.56E-01	5.45E-01
8.13	8.12	8.00	3.56E-01	5.45E-01
8.86	9.06	8.87	3.56E-01	5.45E-01
9.51	9.34	9.52	3.56E-01	5.45E-01
4.45	4.36	4.21	3.56E-01	5.45E-01
11.21	11.38	11.25	3.56E-01	5.45E-01
7.94	7.66	7.80	3.56E-01	5.45E-01
11.42	11.34	11.34	3.56E-01	5.45E-01
10.03	10.16	10.11	3.56E-01	5.45E-01
9.26	9.14	9.56	3.56E-01	5.45E-01
13.28	13.26	13.29	3.56E-01	5.45E-01
4.97	4.93	4.98	3.56E-01	5.45E-01
4.42	4.10	4.34	3.56E-01	5.45E-01
5.50	5.59	5.51	3.56E-01	5.45E-01
7.50	7.41	7.58	3.56E-01	5.45E-01
5.27	5.55	5.31	3.56E-01	5.45E-01
6.86	6.93	6.92	3.56E-01	5.45E-01
13.20	13.20	13.25	3.56E-01	5.45E-01
4.47	4.50	4.51	3.56E-01	5.45E-01
8.63	8.78	8.69	3.56E-01	5.45E-01
6.72	6.36	6.52	3.56E-01	5.45E-01
9.94	9.96	10.09	3.56E-01	5.45E-01
6.12	6.20	6.05	3.57E-01	5.45E-01
4.74	4.78	4.61	3.57E-01	5.45E-01
8.53	8.30	8.63	3.57E-01	5.45E-01
11.59	11.65	11.57	3.57E-01	5.45E-01
8.58	8.83	8.57	3.57E-01	5.45E-01
6.00	6.29	6.15	3.57E-01	5.45E-01
6.11	6.26	6.20	3.57E-01	5.45E-01
4.84	5.02	5.05	3.57E-01	5.45E-01
5.19	5.30	5.03	3.57E-01	5.45E-01
10.25	9.87	10.30	3.57E-01	5.46E-01
5.84	5.78	5.77	3.57E-01	5.46E-01
7.53	7.71	7.64	3.57E-01	5.46E-01
10.28	10.21	10.38	3.57E-01	5.46E-01
6.48	6.50	6.39	3.57E-01	5.46E-01
9.02	9.17	9.07	3.57E-01	5.46E-01
8.39	8.64	8.51	3.57E-01	5.46E-01
6.26	6.14	6.38	3.57E-01	5.46E-01
9.71	9.89	9.89	3.58E-01	5.46E-01

4.35	4.51	4.30	3.58E-01	5.46E-01
6.98	6.86	6.88	3.58E-01	5.46E-01
7.01	6.88	6.94	3.58E-01	5.46E-01
6.05	6.33	6.22	3.58E-01	5.46E-01
7.40	7.31	7.45	3.58E-01	5.46E-01
3.64	3.74	3.85	3.58E-01	5.46E-01
4.01	3.79	3.70	3.58E-01	5.46E-01
4.17	4.10	4.17	3.58E-01	5.46E-01
3.39	3.38	3.36	3.58E-01	5.46E-01
6.32	6.15	6.19	3.58E-01	5.46E-01
3.78	3.52	3.49	3.58E-01	5.46E-01
6.63	6.58	6.74	3.58E-01	5.46E-01
3.66	3.56	3.71	3.58E-01	5.46E-01
5.07	4.98	4.91	3.58E-01	5.46E-01
6.83	7.11	7.18	3.58E-01	5.46E-01
7.86	7.78	8.02	3.58E-01	5.46E-01
3.84	3.72	3.97	3.58E-01	5.46E-01
6.82	6.73	6.92	3.58E-01	5.46E-01
7.50	7.56	7.59	3.58E-01	5.46E-01
7.41	7.57	7.48	3.58E-01	5.46E-01
6.72	6.54	6.66	3.58E-01	5.46E-01
9.13	8.60	9.01	3.58E-01	5.46E-01
6.65	6.54	6.45	3.58E-01	5.46E-01
5.12	4.99	5.15	3.58E-01	5.46E-01
8.78	8.95	8.90	3.58E-01	5.46E-01
13.39	13.57	13.50	3.58E-01	5.46E-01
3.53	3.39	3.47	3.59E-01	5.46E-01
6.30	6.55	6.31	3.59E-01	5.46E-01
6.72	6.58	6.81	3.59E-01	5.46E-01
3.39	3.32	3.46	3.59E-01	5.46E-01
10.74	10.55	10.75	3.59E-01	5.46E-01
4.99	4.52	4.76	3.59E-01	5.46E-01
3.78	3.70	3.82	3.59E-01	5.46E-01
4.49	4.42	4.69	3.59E-01	5.46E-01
4.41	4.24	4.17	3.59E-01	5.46E-01
6.71	6.56	6.63	3.59E-01	5.46E-01
6.29	6.28	6.24	3.59E-01	5.46E-01
9.77	9.54	9.84	3.59E-01	5.46E-01
4.38	4.32	4.60	3.59E-01	5.46E-01
6.69	6.68	6.64	3.59E-01	5.46E-01
6.70	6.46	6.74	3.59E-01	5.46E-01
8.93	9.00	8.99	3.59E-01	5.46E-01
4.58	4.89	4.51	3.59E-01	5.46E-01
7.60	7.43	7.60	3.59E-01	5.46E-01
9.98	9.98	10.03	3.59E-01	5.46E-01
5.06	5.09	5.21	3.59E-01	5.46E-01
5.72	5.85	5.56	3.59E-01	5.46E-01

7.82	8.09	8.03				3.60E-01	5.46E-01
7.86	7.95	7.90				3.60E-01	5.46E-01
6.73	6.58	6.88				3.60E-01	5.46E-01
7.08	7.06	7.02				3.60E-01	5.47E-01
6.78	6.93	6.81				3.60E-01	5.47E-01
7.09	7.15	7.20				3.60E-01	5.47E-01
8.34	8.17	8.29				3.60E-01	5.47E-01
7.35	7.02	7.24				3.60E-01	5.47E-01
7.07	8.16	6.88				3.60E-01	5.47E-01
3.10	3.10	3.21				3.60E-01	5.47E-01
8.54	8.60	8.50				3.60E-01	5.47E-01
4.28	4.58	4.37				3.60E-01	5.47E-01
6.10	5.94	5.93				3.60E-01	5.47E-01
4.80	4.67	4.89				3.60E-01	5.47E-01
6.31	6.30	6.23				3.60E-01	5.47E-01
9.62	9.62	9.59				3.60E-01	5.47E-01
4.64	4.68	4.71				3.60E-01	5.47E-01
7.45	7.37	7.41				3.60E-01	5.47E-01
13.49	13.37	13.39				3.60E-01	5.47E-01
7.25	7.25	7.32				3.60E-01	5.47E-01
9.63	9.66	9.76				3.60E-01	5.47E-01
6.90	6.51	6.78				3.60E-01	5.47E-01
4.98	5.02	4.69				3.61E-01	5.47E-01
4.98	4.93	5.01				3.61E-01	5.47E-01
4.49	4.32	4.34				3.61E-01	5.47E-01
9.68	9.86	9.65				3.61E-01	5.47E-01
3.48	3.68	3.53				3.61E-01	5.47E-01
8.75	8.75	8.92				3.61E-01	5.47E-01
9.94	10.08	9.89				3.61E-01	5.47E-01
11.97	11.66	12.08				3.61E-01	5.47E-01
4.62	4.74	4.69				3.61E-01	5.47E-01
3.67	3.85	3.64				3.61E-01	5.47E-01
6.48	6.46	6.49				3.61E-01	5.47E-01
10.08	10.34	10.24				3.61E-01	5.47E-01
5.54	5.40	5.40				3.61E-01	5.47E-01
7.77	7.74	7.89				3.61E-01	5.47E-01
3.98	3.90	3.81				3.61E-01	5.47E-01
6.43	6.39	6.47				3.61E-01	5.47E-01
11.65	11.55	11.65				3.61E-01	5.47E-01
5.91	5.73	5.75				3.61E-01	5.47E-01
6.61	6.46	6.47				3.61E-01	5.47E-01
9.55	9.52	9.55				3.61E-01	5.47E-01
10.54	10.61	10.56				3.61E-01	5.47E-01
4.06	4.00	4.07				3.61E-01	5.47E-01
5.93	5.96	5.91				3.62E-01	5.47E-01
10.57	10.79	10.60				3.62E-01	5.48E-01
9.13	8.96	9.09				3.62E-01	5.48E-01

11.49	11.77	11.58	3.62E-01	5.48E-01
6.20	6.31	6.26	3.62E-01	5.48E-01
8.52	8.44	8.56	3.62E-01	5.48E-01
5.05	4.71	5.03	3.62E-01	5.48E-01
3.84	3.61	3.74	3.62E-01	5.48E-01
9.03	9.15	9.05	3.62E-01	5.48E-01
5.47	5.32	5.45	3.62E-01	5.48E-01
4.65	4.73	4.75	3.62E-01	5.48E-01
11.01	10.93	11.04	3.62E-01	5.48E-01
9.27	9.41	9.27	3.62E-01	5.48E-01
3.90	4.00	4.01	3.62E-01	5.48E-01
5.57	5.30	5.58	3.62E-01	5.48E-01
7.27	7.45	7.40	3.62E-01	5.48E-01
3.64	3.76	3.71	3.63E-01	5.48E-01
8.99	9.17	8.93	3.63E-01	5.48E-01
8.59	8.46	8.58	3.63E-01	5.48E-01
6.93	6.85	7.06	3.63E-01	5.48E-01
5.20	5.07	5.17	3.63E-01	5.48E-01
4.49	4.41	4.45	3.63E-01	5.48E-01
6.02	5.65	5.95	3.63E-01	5.48E-01
6.47	6.43	6.09	3.63E-01	5.48E-01
5.28	4.99	4.67	3.63E-01	5.48E-01
3.12	3.27	3.42	3.63E-01	5.48E-01
8.72	8.57	8.90	3.63E-01	5.48E-01
10.11	9.64	10.18	3.63E-01	5.49E-01
7.14	7.46	7.25	3.63E-01	5.49E-01
5.80	5.89	5.78	3.63E-01	5.49E-01
9.34	9.00	9.21	3.63E-01	5.49E-01
5.55	5.40	5.51	3.63E-01	5.49E-01
3.48	3.47	3.51	3.63E-01	5.49E-01
3.72	3.69	4.01	3.63E-01	5.49E-01
11.90	11.72	11.82	3.63E-01	5.49E-01
14.34	14.32	14.36	3.64E-01	5.49E-01
4.95	4.76	4.62	3.64E-01	5.49E-01
8.09	7.80	7.75	3.64E-01	5.49E-01
3.44	3.26	3.27	3.64E-01	5.49E-01
7.46	7.15	7.15	3.64E-01	5.49E-01
2.99	3.16	3.17	3.64E-01	5.49E-01
9.04	9.43	9.21	3.64E-01	5.49E-01
6.51	6.56	6.65	3.64E-01	5.49E-01
6.61	6.59	6.57	3.64E-01	5.49E-01
3.87	3.88	3.97	3.64E-01	5.49E-01
8.77	8.68	8.75	3.64E-01	5.49E-01
10.99	11.07	11.02	3.64E-01	5.49E-01
8.75	8.77	8.85	3.64E-01	5.49E-01
6.26	6.39	6.17	3.64E-01	5.49E-01
10.66	11.03	10.83	3.64E-01	5.49E-01

7.78	7.84	7.76	3.65E-01	5.49E-01
8.22	8.10	8.19	3.65E-01	5.49E-01
7.51	7.62	7.76	3.65E-01	5.49E-01
8.20	8.10	8.40	3.65E-01	5.49E-01
4.82	4.75	4.61	3.65E-01	5.49E-01
8.51	8.45	8.58	3.65E-01	5.49E-01
5.17	5.20	5.31	3.65E-01	5.49E-01
9.13	9.22	9.24	3.65E-01	5.49E-01
13.06	13.28	13.17	3.65E-01	5.49E-01
9.48	9.36	9.42	3.65E-01	5.49E-01
9.75	9.82	9.65	3.65E-01	5.49E-01
6.73	6.55	6.81	3.65E-01	5.49E-01
6.50	6.41	6.58	3.65E-01	5.49E-01
3.68	3.91	4.20	3.65E-01	5.49E-01
5.47	5.47	5.46	3.65E-01	5.49E-01
4.48	4.57	4.09	3.65E-01	5.49E-01
10.42	10.34	10.45	3.65E-01	5.49E-01
8.87	8.97	8.83	3.65E-01	5.49E-01
10.60	10.77	10.66	3.65E-01	5.49E-01
7.28	7.15	7.36	3.65E-01	5.49E-01
10.96	11.12	11.09	3.65E-01	5.49E-01
3.67	3.65	3.72	3.65E-01	5.49E-01
5.87	5.73	5.68	3.65E-01	5.49E-01
10.21	10.09	10.34	3.65E-01	5.49E-01
9.24	9.20	9.36	3.65E-01	5.49E-01
10.52	10.59	10.71	3.65E-01	5.49E-01
4.44	4.42	4.56	3.65E-01	5.49E-01
10.26	10.29	10.32	3.65E-01	5.49E-01
8.43	8.13	8.43	3.65E-01	5.49E-01
4.51	4.44	4.46	3.65E-01	5.49E-01
3.34	3.34	3.29	3.65E-01	5.49E-01
10.71	10.54	10.64	3.65E-01	5.49E-01
11.36	11.25	11.37	3.66E-01	5.49E-01
6.47	6.90	6.54	3.66E-01	5.49E-01
8.83	9.01	8.99	3.66E-01	5.49E-01
3.76	3.41	3.56	3.66E-01	5.49E-01
4.06	3.86	4.15	3.66E-01	5.49E-01
4.01	4.01	4.15	3.66E-01	5.49E-01
3.24	3.24	3.36	3.66E-01	5.49E-01
4.24	4.26	3.88	3.66E-01	5.50E-01
9.33	9.32	9.50	3.66E-01	5.50E-01
3.58	3.75	3.84	3.66E-01	5.50E-01
10.40	10.54	10.53	3.66E-01	5.50E-01
7.32	7.37	7.42	3.66E-01	5.50E-01
8.73	8.58	8.70	3.66E-01	5.50E-01
3.96	3.74	3.84	3.66E-01	5.50E-01
7.07	7.11	7.14	3.66E-01	5.50E-01

8.11	8.13	8.13	3.66E-01	5.50E-01
9.65	9.58	9.64	3.67E-01	5.50E-01
7.89	7.72	7.73	3.67E-01	5.50E-01
5.57	5.49	5.52	3.67E-01	5.50E-01
6.65	6.59	6.59	3.67E-01	5.50E-01
9.52	9.83	9.60	3.67E-01	5.50E-01
7.81	8.37	7.74	3.67E-01	5.50E-01
11.22	11.26	11.28	3.67E-01	5.50E-01
10.45	10.71	10.54	3.67E-01	5.50E-01
6.74	6.69	6.69	3.67E-01	5.50E-01
8.06	8.50	8.17	3.67E-01	5.50E-01
5.12	4.83	4.64	3.67E-01	5.51E-01
3.88	3.80	4.03	3.67E-01	5.51E-01
4.17	4.25	4.59	3.68E-01	5.51E-01
6.96	6.70	6.80	3.68E-01	5.51E-01
4.89	4.77	4.53	3.68E-01	5.51E-01
6.56	6.73	6.80	3.68E-01	5.51E-01
8.11	8.36	8.40	3.68E-01	5.51E-01
7.19	7.11	7.05	3.68E-01	5.51E-01
9.39	9.11	9.40	3.68E-01	5.51E-01
8.30	8.34	8.19	3.68E-01	5.51E-01
5.75	5.70	5.76	3.68E-01	5.51E-01
6.85	7.07	6.65	3.68E-01	5.51E-01
5.50	5.46	5.27	3.68E-01	5.51E-01
9.85	9.75	9.88	3.68E-01	5.51E-01
7.52	7.69	7.74	3.68E-01	5.51E-01
5.42	5.73	5.90	3.68E-01	5.51E-01
5.64	5.76	5.94	3.68E-01	5.51E-01
4.74	4.74	4.78	3.68E-01	5.51E-01
7.84	8.09	8.04	3.68E-01	5.51E-01
4.44	4.48	4.58	3.68E-01	5.51E-01
4.82	4.57	5.07	3.68E-01	5.51E-01
5.68	5.65	5.66	3.69E-01	5.51E-01
8.89	8.75	8.80	3.69E-01	5.51E-01
9.14	9.42	9.35	3.69E-01	5.51E-01
3.92	3.68	3.68	3.69E-01	5.51E-01
9.47	9.42	9.44	3.69E-01	5.51E-01
10.63	10.98	10.65	3.69E-01	5.51E-01
5.51	5.45	5.38	3.69E-01	5.51E-01
7.69	7.77	7.74	3.69E-01	5.51E-01
10.18	10.35	10.26	3.69E-01	5.51E-01
4.19	4.32	4.14	3.69E-01	5.51E-01
9.10	9.42	9.19	3.69E-01	5.51E-01
3.34	3.30	3.49	3.69E-01	5.51E-01
7.17	7.08	7.02	3.69E-01	5.51E-01
4.06	3.80	3.82	3.69E-01	5.51E-01
3.54	3.58	3.60	3.69E-01	5.51E-01

9.91	9.73	9.81	3.69E-01	5.51E-01
5.70	5.86	5.95	3.69E-01	5.51E-01
11.56	11.65	11.63	3.69E-01	5.51E-01
10.38	10.10	10.38	3.69E-01	5.51E-01
7.23	7.27	7.29	3.69E-01	5.51E-01
8.12	8.27	8.21	3.69E-01	5.51E-01
5.99	5.54	5.74	3.69E-01	5.51E-01
5.70	5.86	5.50	3.69E-01	5.51E-01
11.85	11.77	11.94	3.69E-01	5.51E-01
11.63	11.79	11.70	3.69E-01	5.51E-01
9.51	9.71	9.67	3.69E-01	5.51E-01
3.25	3.19	3.43	3.69E-01	5.51E-01
6.71	6.27	6.73	3.69E-01	5.51E-01
7.50	7.63	7.51	3.69E-01	5.51E-01
5.58	5.63	5.66	3.69E-01	5.51E-01
3.36	3.34	3.60	3.70E-01	5.51E-01
9.39	9.63	9.56	3.70E-01	5.51E-01
4.19	4.20	4.24	3.70E-01	5.52E-01
4.66	4.74	4.56	3.70E-01	5.52E-01
7.18	7.20	7.19	3.70E-01	5.52E-01
10.45	10.42	10.44	3.70E-01	5.52E-01
6.66	6.73	6.76	3.70E-01	5.52E-01
4.58	4.35	4.53	3.70E-01	5.52E-01
4.71	4.37	4.40	3.70E-01	5.52E-01
9.32	9.08	9.18	3.70E-01	5.52E-01
7.28	7.34	7.42	3.70E-01	5.52E-01
4.30	4.42	4.08	3.70E-01	5.52E-01
6.96	6.74	6.87	3.70E-01	5.52E-01
4.89	4.89	4.81	3.70E-01	5.52E-01
4.52	4.49	4.32	3.70E-01	5.52E-01
4.41	4.40	4.48	3.70E-01	5.52E-01
3.33	3.51	3.49	3.70E-01	5.52E-01
9.51	9.77	9.66	3.70E-01	5.52E-01
6.45	6.27	6.53	3.71E-01	5.52E-01
6.48	6.21	6.44	3.71E-01	5.52E-01
9.44	9.21	9.22	3.71E-01	5.52E-01
3.68	3.81	3.84	3.71E-01	5.52E-01
8.91	9.14	8.87	3.71E-01	5.52E-01
9.61	9.80	9.64	3.71E-01	5.52E-01
10.14	10.09	10.14	3.71E-01	5.52E-01
6.81	6.87	6.73	3.71E-01	5.52E-01
10.60	10.50	10.58	3.71E-01	5.52E-01
10.17	10.25	10.27	3.71E-01	5.53E-01
4.31	4.63	4.43	3.71E-01	5.53E-01
10.58	10.35	10.44	3.71E-01	5.53E-01
6.66	6.75	6.78	3.72E-01	5.53E-01
4.14	3.87	3.98	3.72E-01	5.53E-01

4.37	4.13	4.05	3.72E-01	5.53E-01
4.13	4.06	4.16	3.72E-01	5.53E-01
4.82	4.64	4.79	3.72E-01	5.53E-01
4.00	4.20	4.36	3.72E-01	5.53E-01
5.14	5.16	5.09	3.72E-01	5.53E-01
3.86	3.66	3.69	3.72E-01	5.53E-01
7.20	6.97	7.30	3.72E-01	5.53E-01
9.57	9.24	9.43	3.72E-01	5.53E-01
9.82	9.68	9.95	3.72E-01	5.53E-01
3.56	3.50	3.34	3.72E-01	5.53E-01
9.80	10.03	9.98	3.72E-01	5.53E-01
8.54	8.52	8.61	3.72E-01	5.53E-01
5.70	5.49	5.45	3.72E-01	5.53E-01
6.80	6.66	6.42	3.72E-01	5.53E-01
10.41	10.29	10.25	3.73E-01	5.53E-01
7.73	7.70	7.66	3.73E-01	5.53E-01
6.35	6.24	6.58	3.73E-01	5.53E-01
8.81	8.78	8.81	3.73E-01	5.53E-01
7.56	7.46	7.58	3.73E-01	5.53E-01
8.82	9.00	8.86	3.73E-01	5.53E-01
4.88	4.84	4.70	3.73E-01	5.53E-01
3.87	3.80	3.81	3.73E-01	5.53E-01
8.60	8.69	8.75	3.73E-01	5.53E-01
5.38	5.42	5.50	3.73E-01	5.53E-01
7.27	7.23	7.18	3.73E-01	5.53E-01
7.66	7.82	7.68	3.73E-01	5.53E-01
3.37	3.51	3.34	3.73E-01	5.53E-01
4.09	4.18	4.09	3.73E-01	5.53E-01
8.25	8.21	8.35	3.73E-01	5.53E-01
9.86	9.90	9.99	3.73E-01	5.53E-01
5.98	5.87	5.87	3.73E-01	5.53E-01
6.88	6.92	7.08	3.73E-01	5.53E-01
8.86	8.93	8.97	3.73E-01	5.53E-01
3.63	4.02	4.07	3.73E-01	5.53E-01
4.83	4.62	4.66	3.73E-01	5.53E-01
4.55	4.73	4.59	3.73E-01	5.53E-01
5.82	5.70	5.92	3.73E-01	5.53E-01
4.68	4.60	4.33	3.73E-01	5.54E-01
7.92	7.95	7.96	3.73E-01	5.54E-01
3.43	3.21	3.54	3.73E-01	5.54E-01
8.22	8.46	8.35	3.74E-01	5.54E-01
4.42	4.36	4.36	3.74E-01	5.54E-01
5.33	5.11	5.07	3.74E-01	5.54E-01
6.57	6.70	6.68	3.74E-01	5.54E-01
9.53	9.19	9.26	3.74E-01	5.54E-01
6.00	6.00	6.02	3.74E-01	5.54E-01
9.27	9.20	9.35	3.74E-01	5.54E-01

9.63	9.40	9.65	3.74E-01	5.54E-01
12.36	12.11	12.33	3.74E-01	5.54E-01
3.60	3.70	3.39	3.74E-01	5.54E-01
8.72	8.92	8.73	3.74E-01	5.54E-01
10.48	10.60	10.47	3.74E-01	5.54E-01
5.97	5.84	6.10	3.74E-01	5.54E-01
10.33	10.58	10.43	3.74E-01	5.54E-01
10.41	10.57	10.51	3.74E-01	5.54E-01
5.11	5.22	4.85	3.74E-01	5.54E-01
6.08	6.34	5.78	3.74E-01	5.54E-01
7.70	7.72	7.74	3.74E-01	5.54E-01
9.97	9.88	9.76	3.74E-01	5.54E-01
11.48	11.60	11.56	3.75E-01	5.54E-01
7.21	7.46	7.24	3.75E-01	5.54E-01
6.18	5.87	6.23	3.75E-01	5.54E-01
4.97	5.24	4.81	3.75E-01	5.54E-01
3.83	3.79	4.17	3.75E-01	5.54E-01
10.26	10.19	10.08	3.75E-01	5.54E-01
9.45	9.45	9.48	3.75E-01	5.54E-01
8.19	8.55	8.37	3.75E-01	5.54E-01
4.78	5.11	4.94	3.75E-01	5.54E-01
10.05	10.02	10.05	3.75E-01	5.54E-01
5.47	5.68	5.53	3.75E-01	5.54E-01
4.04	4.08	4.08	3.75E-01	5.54E-01
5.99	6.11	6.41	3.75E-01	5.54E-01
3.50	3.50	3.41	3.75E-01	5.54E-01
9.64	9.56	9.66	3.75E-01	5.54E-01
6.09	6.04	5.91	3.75E-01	5.54E-01
6.24	6.32	6.28	3.75E-01	5.54E-01
6.94	6.94	6.87	3.75E-01	5.54E-01
5.79	5.65	5.86	3.75E-01	5.54E-01
4.54	4.67	4.60	3.75E-01	5.54E-01
3.70	3.94	3.72	3.75E-01	5.54E-01
9.59	9.91	9.69	3.75E-01	5.54E-01
9.28	9.14	9.28	3.75E-01	5.54E-01
8.47	8.66	8.61	3.75E-01	5.54E-01
4.28	4.24	4.37	3.75E-01	5.54E-01
12.45	12.72	12.54	3.76E-01	5.54E-01
4.53	4.30	4.52	3.76E-01	5.54E-01
5.91	5.79	5.74	3.76E-01	5.54E-01
14.53	14.44	14.55	3.76E-01	5.54E-01
8.25	8.54	8.37	3.76E-01	5.54E-01
7.87	8.16	7.79	3.76E-01	5.54E-01
7.39	7.33	7.35	3.76E-01	5.55E-01
12.33	12.39	12.26	3.76E-01	5.55E-01
8.89	9.12	8.94	3.76E-01	5.55E-01
10.34	10.48	10.37	3.76E-01	5.55E-01

4.69	4.56	4.40	3.76E-01	5.55E-01
6.24	6.36	6.22	3.76E-01	5.55E-01
8.43	8.53	8.52	3.76E-01	5.55E-01
9.58	9.73	9.55	3.76E-01	5.55E-01
8.41	8.30	8.26	3.76E-01	5.55E-01
4.33	4.26	4.46	3.76E-01	5.55E-01
9.38	9.58	9.45	3.77E-01	5.55E-01
4.95	4.83	4.55	3.77E-01	5.55E-01
9.49	9.36	9.44	3.77E-01	5.55E-01
6.23	6.41	6.32	3.77E-01	5.55E-01
3.89	3.69	3.77	3.77E-01	5.55E-01
8.60	9.05	8.84	3.77E-01	5.55E-01
8.91	9.01	8.89	3.77E-01	5.55E-01
14.42	14.44	14.45	3.77E-01	5.55E-01
4.73	4.55	4.44	3.77E-01	5.55E-01
7.10	7.08	7.06	3.77E-01	5.55E-01
5.29	5.23	5.16	3.77E-01	5.55E-01
7.94	8.11	7.91	3.77E-01	5.55E-01
4.83	5.16	5.02	3.77E-01	5.55E-01
10.51	10.84	10.69	3.77E-01	5.55E-01
9.68	9.84	9.68	3.77E-01	5.55E-01
6.36	6.13	6.28	3.77E-01	5.55E-01
6.19	6.13	6.03	3.77E-01	5.55E-01
5.55	5.29	5.48	3.77E-01	5.55E-01
5.40	5.11	5.34	3.77E-01	5.55E-01
4.24	4.21	4.05	3.77E-01	5.55E-01
6.85	7.00	6.76	3.77E-01	5.55E-01
6.84	6.94	6.79	3.77E-01	5.55E-01
7.57	8.25	7.71	3.77E-01	5.55E-01
3.91	3.88	3.89	3.77E-01	5.55E-01
6.27	6.13	6.27	3.77E-01	5.55E-01
6.44	6.60	6.45	3.77E-01	5.55E-01
8.06	8.10	8.06	3.77E-01	5.55E-01
3.43	3.27	3.60	3.78E-01	5.55E-01
9.45	9.53	9.53	3.78E-01	5.55E-01
5.41	5.69	5.52	3.78E-01	5.55E-01
9.16	9.46	9.41	3.78E-01	5.56E-01
7.19	6.99	6.98	3.78E-01	5.56E-01
6.52	6.39	6.58	3.78E-01	5.56E-01
14.54	14.69	14.56	3.78E-01	5.56E-01
6.36	6.26	6.32	3.78E-01	5.56E-01
6.06	6.02	6.14	3.78E-01	5.56E-01
6.98	7.19	7.17	3.78E-01	5.56E-01
5.12	5.11	5.18	3.78E-01	5.56E-01
7.70	7.60	7.71	3.78E-01	5.56E-01
3.67	3.50	3.85	3.78E-01	5.56E-01
9.94	9.71	9.81	3.78E-01	5.56E-01

8.76	8.47	8.51	3.78E-01	5.56E-01
10.62	10.36	10.52	3.78E-01	5.56E-01
6.49	6.39	6.02	3.78E-01	5.56E-01
8.04	8.14	8.08	3.78E-01	5.56E-01
9.66	9.88	9.76	3.78E-01	5.56E-01
9.98	9.81	9.96	3.78E-01	5.56E-01
12.97	13.26	13.13	3.78E-01	5.56E-01
6.32	6.23	6.25	3.78E-01	5.56E-01
4.62	4.79	4.85	3.79E-01	5.56E-01
10.12	9.95	10.03	3.79E-01	5.56E-01
4.53	4.46	4.27	3.79E-01	5.56E-01
8.03	8.06	8.12	3.79E-01	5.56E-01
6.59	6.59	6.63	3.79E-01	5.56E-01
8.64	8.77	8.81	3.79E-01	5.56E-01
8.25	8.12	8.38	3.79E-01	5.56E-01
8.46	8.51	8.60	3.79E-01	5.56E-01
5.93	5.81	5.81	3.79E-01	5.56E-01
3.30	3.25	3.48	3.79E-01	5.56E-01
6.25	6.30	6.26	3.79E-01	5.56E-01
4.80	4.80	4.91	3.79E-01	5.56E-01
4.91	5.06	4.63	3.79E-01	5.56E-01
8.35	8.55	8.51	3.79E-01	5.56E-01
7.23	7.16	7.29	3.79E-01	5.56E-01
12.10	12.29	12.16	3.79E-01	5.56E-01
9.79	9.86	9.81	3.79E-01	5.56E-01
5.62	5.36	5.25	3.79E-01	5.56E-01
5.98	5.94	5.85	3.80E-01	5.56E-01
6.46	6.51	6.60	3.80E-01	5.56E-01
9.56	9.92	9.75	3.80E-01	5.56E-01
7.15	7.25	7.14	3.80E-01	5.57E-01
5.08	4.92	5.02	3.80E-01	5.57E-01
7.52	7.60	7.47	3.80E-01	5.57E-01
6.17	6.39	6.37	3.80E-01	5.57E-01
6.06	6.24	6.25	3.80E-01	5.57E-01
10.94	11.03	10.92	3.80E-01	5.57E-01
7.64	7.92	7.90	3.80E-01	5.57E-01
8.67	8.87	8.73	3.80E-01	5.57E-01
7.60	7.93	7.40	3.80E-01	5.57E-01
7.46	7.53	7.68	3.81E-01	5.57E-01
9.58	9.64	9.47	3.81E-01	5.57E-01
3.76	3.76	3.90	3.81E-01	5.57E-01
4.31	4.22	4.31	3.81E-01	5.57E-01
12.13	12.13	12.20	3.81E-01	5.57E-01
5.80	5.41	5.49	3.81E-01	5.57E-01
7.51	7.55	7.53	3.81E-01	5.57E-01
13.66	13.71	13.73	3.81E-01	5.57E-01
5.16	5.41	5.27	3.81E-01	5.57E-01

5.70	5.92	5.69	3.81E-01	5.57E-01
5.28	5.66	5.46	3.81E-01	5.57E-01
3.02	3.17	3.16	3.81E-01	5.57E-01
4.82	4.64	4.85	3.81E-01	5.57E-01
3.47	3.68	3.27	3.81E-01	5.57E-01
6.60	6.67	6.71	3.81E-01	5.57E-01
6.49	6.14	6.38	3.81E-01	5.57E-01
9.75	9.94	9.85	3.81E-01	5.57E-01
6.58	6.56	6.93	3.81E-01	5.57E-01
9.62	9.32	9.50	3.81E-01	5.57E-01
3.21	3.28	3.08	3.81E-01	5.57E-01
9.25	9.31	9.30	3.81E-01	5.57E-01
5.37	5.53	5.45	3.81E-01	5.57E-01
9.22	8.86	9.08	3.82E-01	5.57E-01
3.26	3.57	3.51	3.82E-01	5.57E-01
3.65	3.57	3.53	3.82E-01	5.58E-01
6.35	6.41	6.25	3.82E-01	5.58E-01
6.21	6.40	6.51	3.82E-01	5.58E-01
8.22	8.35	8.30	3.82E-01	5.58E-01
12.37	12.51	12.47	3.82E-01	5.58E-01
8.27	8.40	8.44	3.82E-01	5.58E-01
7.52	7.60	7.63	3.82E-01	5.58E-01
5.75	5.80	5.97	3.82E-01	5.58E-01
8.90	8.69	8.87	3.82E-01	5.58E-01
4.62	4.88	4.64	3.82E-01	5.58E-01
6.94	6.95	7.02	3.82E-01	5.58E-01
5.09	5.20	5.24	3.82E-01	5.58E-01
7.05	6.88	7.12	3.83E-01	5.58E-01
5.87	6.15	6.35	3.83E-01	5.58E-01
8.75	8.55	8.40	3.83E-01	5.58E-01
9.23	9.18	9.10	3.83E-01	5.58E-01
8.74	9.14	8.95	3.83E-01	5.58E-01
4.56	4.63	4.48	3.83E-01	5.58E-01
4.39	4.42	4.27	3.83E-01	5.58E-01
9.06	9.27	9.29	3.83E-01	5.58E-01
8.97	9.12	9.06	3.83E-01	5.58E-01
4.67	4.44	4.64	3.83E-01	5.58E-01
3.98	3.91	3.93	3.83E-01	5.58E-01
4.18	3.86	4.10	3.83E-01	5.58E-01
4.48	4.46	4.38	3.83E-01	5.58E-01
9.11	9.14	9.08	3.83E-01	5.58E-01
4.29	4.47	4.37	3.83E-01	5.58E-01
6.94	6.87	6.91	3.83E-01	5.58E-01
5.73	5.54	5.85	3.83E-01	5.58E-01
5.61	5.89	5.48	3.83E-01	5.58E-01
6.59	6.56	6.50	3.83E-01	5.58E-01
7.22	7.17	7.24	3.83E-01	5.58E-01

6.33	6.16	6.36	3.83E-01	5.58E-01
10.07	10.13	10.01	3.83E-01	5.58E-01
9.39	9.61	9.61	3.83E-01	5.58E-01
7.89	8.08	8.06	3.84E-01	5.58E-01
8.53	8.74	8.72	3.84E-01	5.58E-01
4.08	4.06	3.95	3.84E-01	5.58E-01
3.30	3.56	3.37	3.84E-01	5.58E-01
6.51	6.42	6.46	3.84E-01	5.58E-01
3.26	3.24	2.98	3.84E-01	5.58E-01
5.62	5.30	5.79	3.84E-01	5.58E-01
3.34	3.23	3.43	3.84E-01	5.58E-01
3.79	3.95	3.93	3.84E-01	5.58E-01
5.42	5.40	5.74	3.84E-01	5.58E-01
6.72	6.46	6.71	3.84E-01	5.58E-01
7.94	8.12	8.00	3.84E-01	5.58E-01
5.26	5.29	5.30	3.84E-01	5.58E-01
8.54	8.43	8.39	3.84E-01	5.59E-01
11.67	11.42	11.44	3.84E-01	5.59E-01
5.14	5.08	5.18	3.84E-01	5.59E-01
4.80	4.66	4.53	3.84E-01	5.59E-01
8.60	8.57	8.65	3.84E-01	5.59E-01
15.05	15.07	15.08	3.84E-01	5.59E-01
8.86	8.90	8.87	3.84E-01	5.59E-01
9.51	9.28	9.50	3.84E-01	5.59E-01
5.24	5.29	5.45	3.84E-01	5.59E-01
4.74	4.84	4.64	3.85E-01	5.59E-01
8.38	8.17	8.28	3.85E-01	5.59E-01
9.11	8.86	8.96	3.85E-01	5.59E-01
5.48	5.74	5.24	3.85E-01	5.59E-01
11.56	11.79	11.70	3.85E-01	5.59E-01
7.43	7.58	7.51	3.85E-01	5.59E-01
3.83	3.53	3.97	3.85E-01	5.59E-01
6.46	6.56	6.59	3.85E-01	5.59E-01
5.37	5.16	5.22	3.85E-01	5.59E-01
7.09	7.22	7.23	3.85E-01	5.59E-01
8.05	8.48	8.02	3.85E-01	5.59E-01
4.06	4.03	4.03	3.85E-01	5.59E-01
4.41	4.64	4.58	3.85E-01	5.59E-01
5.66	5.76	5.34	3.85E-01	5.59E-01
6.35	6.23	6.29	3.85E-01	5.59E-01
11.48	11.65	11.55	3.85E-01	5.59E-01
4.29	5.08	4.31	3.85E-01	5.59E-01
5.17	5.02	5.16	3.85E-01	5.59E-01
10.46	10.35	10.43	3.85E-01	5.59E-01
4.12	4.01	4.00	3.85E-01	5.59E-01
10.19	10.19	10.17	3.85E-01	5.59E-01
7.94	8.16	7.94	3.85E-01	5.59E-01

3.75	3.72	3.81	3.85E-01	5.59E-01
5.60	5.73	5.75	3.86E-01	5.59E-01
9.36	8.83	9.08	3.86E-01	5.59E-01
8.30	7.98	8.01	3.86E-01	5.59E-01
4.39	4.27	4.15	3.86E-01	5.59E-01
8.51	7.97	8.59	3.86E-01	5.59E-01
5.01	4.92	5.02	3.86E-01	5.59E-01
8.86	8.95	8.89	3.86E-01	5.59E-01
7.98	8.10	7.89	3.86E-01	5.59E-01
9.38	9.58	9.46	3.86E-01	5.59E-01
7.72	7.85	7.92	3.86E-01	5.59E-01
5.97	6.02	5.98	3.86E-01	5.59E-01
10.56	10.75	10.65	3.86E-01	5.59E-01
3.30	3.02	3.22	3.86E-01	5.59E-01
6.61	6.35	6.76	3.86E-01	5.59E-01
9.44	9.44	9.41	3.86E-01	5.59E-01
3.42	3.70	3.25	3.86E-01	5.59E-01
4.77	4.74	4.69	3.86E-01	5.59E-01
5.29	5.45	5.41	3.86E-01	5.59E-01
6.85	6.81	7.16	3.86E-01	5.59E-01
8.75	8.82	8.80	3.86E-01	5.59E-01
5.26	5.32	5.40	3.86E-01	5.59E-01
6.64	6.25	6.28	3.86E-01	5.60E-01
4.57	4.60	4.50	3.87E-01	5.60E-01
8.69	9.11	8.77	3.87E-01	5.60E-01
9.23	9.46	9.41	3.87E-01	5.60E-01
5.40	5.34	5.32	3.87E-01	5.60E-01
4.96	4.68	4.58	3.87E-01	5.60E-01
3.93	4.08	4.06	3.87E-01	5.60E-01
4.94	4.98	4.91	3.87E-01	5.60E-01
11.15	10.96	11.01	3.87E-01	5.60E-01
4.22	4.07	3.95	3.87E-01	5.60E-01
5.50	5.52	5.44	3.87E-01	5.60E-01
4.67	4.95	5.04	3.87E-01	5.60E-01
8.56	8.81	8.67	3.87E-01	5.60E-01
4.20	4.19	4.22	3.87E-01	5.60E-01
6.50	6.44	6.59	3.87E-01	5.60E-01
5.68	5.78	6.00	3.87E-01	5.60E-01
10.06	10.50	9.89	3.87E-01	5.60E-01
8.29	8.68	8.38	3.87E-01	5.60E-01
9.73	10.14	9.84	3.87E-01	5.60E-01
11.35	11.31	11.27	3.87E-01	5.60E-01
5.34	5.21	5.03	3.87E-01	5.60E-01
13.06	13.32	13.08	3.88E-01	5.60E-01
7.93	7.83	7.95	3.88E-01	5.60E-01
4.81	5.10	5.02	3.88E-01	5.60E-01
8.02	8.01	7.92	3.88E-01	5.60E-01

3.94	3.64	4.00	3.88E-01	5.60E-01
8.13	7.80	7.89	3.88E-01	5.60E-01
3.69	3.69	3.57	3.88E-01	5.60E-01
6.50	6.27	6.54	3.88E-01	5.60E-01
10.31	10.20	10.30	3.88E-01	5.60E-01
4.16	4.07	3.98	3.88E-01	5.60E-01
4.13	3.90	4.19	3.88E-01	5.60E-01
7.37	7.56	7.42	3.88E-01	5.60E-01
4.32	4.49	4.21	3.88E-01	5.60E-01
9.06	9.32	9.11	3.88E-01	5.60E-01
10.54	10.54	10.54	3.88E-01	5.60E-01
13.06	13.16	13.12	3.88E-01	5.60E-01
9.31	9.63	9.52	3.88E-01	5.60E-01
5.04	4.73	4.60	3.88E-01	5.60E-01
5.30	5.09	5.15	3.88E-01	5.60E-01
8.83	9.09	9.04	3.88E-01	5.60E-01
10.21	10.35	10.34	3.88E-01	5.60E-01
10.45	10.55	10.57	3.89E-01	5.60E-01
6.07	6.12	6.11	3.89E-01	5.60E-01
3.34	3.34	3.31	3.89E-01	5.60E-01
6.47	6.24	6.60	3.89E-01	5.60E-01
5.39	5.47	5.37	3.89E-01	5.60E-01
7.58	7.68	7.67	3.89E-01	5.60E-01
5.39	5.07	5.12	3.89E-01	5.60E-01
9.22	9.08	9.22	3.89E-01	5.60E-01
4.29	4.32	4.36	3.89E-01	5.60E-01
6.78	6.77	6.96	3.89E-01	5.60E-01
6.27	6.27	6.25	3.89E-01	5.60E-01
6.59	6.66	6.84	3.89E-01	5.60E-01
9.49	9.73	9.61	3.89E-01	5.60E-01
5.14	5.17	5.37	3.89E-01	5.60E-01
6.06	5.97	5.92	3.89E-01	5.60E-01
3.92	3.70	3.77	3.89E-01	5.60E-01
11.07	11.07	11.15	3.89E-01	5.60E-01
5.88	5.89	6.04	3.89E-01	5.60E-01
6.39	6.44	6.47	3.89E-01	5.60E-01
12.14	12.07	12.16	3.89E-01	5.60E-01
3.91	3.63	3.89	3.89E-01	5.61E-01
3.55	3.38	3.59	3.90E-01	5.61E-01
6.58	6.66	6.73	3.90E-01	5.61E-01
8.30	8.11	8.33	3.90E-01	5.61E-01
4.29	4.45	4.43	3.90E-01	5.61E-01
14.42	14.40	14.46	3.90E-01	5.61E-01
9.10	9.23	9.16	3.90E-01	5.61E-01
15.16	15.17	15.18	3.90E-01	5.61E-01
4.41	4.91	4.56	3.90E-01	5.61E-01
11.86	12.08	11.97	3.90E-01	5.61E-01

5.45	5.16	5.32	3.90E-01	5.61E-01
7.21	7.19	7.30	3.90E-01	5.61E-01
4.65	4.69	4.72	3.90E-01	5.61E-01
5.91	6.15	5.99	3.90E-01	5.61E-01
7.82	7.86	7.95	3.90E-01	5.61E-01
5.12	5.29	4.98	3.90E-01	5.61E-01
7.87	8.02	8.06	3.90E-01	5.61E-01
5.88	5.67	5.77	3.90E-01	5.61E-01
5.22	5.31	5.15	3.90E-01	5.61E-01
8.72	8.44	8.73	3.91E-01	5.61E-01
5.67	5.89	5.92	3.91E-01	5.61E-01
4.84	4.96	4.97	3.91E-01	5.61E-01
6.20	6.36	6.32	3.91E-01	5.61E-01
6.78	6.66	6.50	3.91E-01	5.62E-01
11.46	10.87	11.41	3.91E-01	5.62E-01
4.57	4.56	4.49	3.91E-01	5.62E-01
7.90	7.92	7.84	3.91E-01	5.62E-01
9.59	9.58	9.63	3.91E-01	5.62E-01
9.51	9.36	9.48	3.91E-01	5.62E-01
6.50	6.58	6.38	3.91E-01	5.62E-01
6.67	6.64	6.72	3.91E-01	5.62E-01
12.04	11.98	11.93	3.91E-01	5.62E-01
6.83	6.84	6.88	3.91E-01	5.62E-01
7.15	7.15	6.97	3.91E-01	5.62E-01
8.27	8.39	8.41	3.91E-01	5.62E-01
4.69	4.47	4.77	3.91E-01	5.62E-01
9.32	9.10	9.48	3.92E-01	5.62E-01
8.23	8.60	8.34	3.92E-01	5.62E-01
7.05	7.19	6.92	3.92E-01	5.62E-01
8.18	8.16	8.15	3.92E-01	5.62E-01
4.24	4.18	4.35	3.92E-01	5.62E-01
3.34	3.49	3.66	3.92E-01	5.62E-01
10.20	9.99	10.15	3.92E-01	5.62E-01
9.53	9.49	9.57	3.92E-01	5.62E-01
8.36	8.47	8.34	3.92E-01	5.62E-01
9.05	9.19	9.06	3.92E-01	5.62E-01
8.19	8.00	8.20	3.92E-01	5.62E-01
8.43	8.57	8.53	3.92E-01	5.63E-01
8.17	8.06	8.24	3.92E-01	5.63E-01
6.84	6.64	6.55	3.92E-01	5.63E-01
9.03	9.02	9.01	3.93E-01	5.63E-01
5.09	5.26	5.04	3.93E-01	5.63E-01
3.53	3.47	3.50	3.93E-01	5.63E-01
3.01	3.08	3.20	3.93E-01	5.63E-01
10.75	10.94	10.87	3.93E-01	5.63E-01
5.13	4.87	5.04	3.93E-01	5.63E-01
6.08	5.96	6.04	3.93E-01	5.63E-01

9.15	9.38	9.27	3.93E-01	5.63E-01
3.48	3.18	3.42	3.93E-01	5.63E-01
6.70	6.39	6.75	3.93E-01	5.63E-01
6.77	6.65	6.73	3.93E-01	5.63E-01
6.13	6.08	6.15	3.93E-01	5.63E-01
5.50	5.67	5.26	3.93E-01	5.63E-01
5.91	5.99	5.80	3.93E-01	5.63E-01
5.94	5.66	5.85	3.93E-01	5.63E-01
7.05	7.12	6.90	3.93E-01	5.63E-01
6.78	6.89	6.76	3.93E-01	5.63E-01
6.11	6.16	6.25	3.93E-01	5.63E-01
7.27	7.46	7.32	3.93E-01	5.63E-01
6.39	6.08	6.42	3.93E-01	5.63E-01
6.76	6.59	6.59	3.93E-01	5.63E-01
8.91	8.69	9.03	3.93E-01	5.63E-01
3.94	3.93	3.90	3.93E-01	5.63E-01
7.13	7.06	7.09	3.93E-01	5.63E-01
4.04	3.86	4.03	3.94E-01	5.63E-01
7.06	7.13	7.11	3.94E-01	5.63E-01
3.52	3.57	3.58	3.94E-01	5.63E-01
4.31	4.14	4.23	3.94E-01	5.63E-01
5.73	5.44	5.60	3.94E-01	5.63E-01
12.35	12.54	12.26	3.94E-01	5.63E-01
8.25	8.10	8.20	3.94E-01	5.63E-01
4.33	4.34	4.21	3.94E-01	5.63E-01
4.32	4.16	4.00	3.94E-01	5.63E-01
4.52	4.63	4.39	3.94E-01	5.63E-01
3.80	4.26	3.89	3.94E-01	5.63E-01
3.51	3.59	3.51	3.94E-01	5.63E-01
6.48	6.35	6.21	3.94E-01	5.63E-01
10.27	10.18	10.14	3.94E-01	5.63E-01
9.53	9.27	9.40	3.94E-01	5.63E-01
5.89	5.90	6.02	3.94E-01	5.63E-01
6.99	6.94	7.11	3.95E-01	5.64E-01
5.34	5.06	4.78	3.95E-01	5.64E-01
8.62	8.82	8.74	3.95E-01	5.64E-01
6.66	6.73	6.61	3.95E-01	5.64E-01
13.99	13.98	13.93	3.95E-01	5.64E-01
7.07	6.79	6.86	3.95E-01	5.64E-01
12.21	12.20	12.25	3.95E-01	5.64E-01
3.92	3.74	4.16	3.95E-01	5.64E-01
10.01	9.99	10.11	3.95E-01	5.64E-01
10.94	11.12	11.12	3.95E-01	5.64E-01
4.69	5.03	4.87	3.95E-01	5.64E-01
8.53	8.76	8.66	3.95E-01	5.64E-01
9.18	9.14	9.22	3.95E-01	5.64E-01
6.31	6.28	6.18	3.95E-01	5.64E-01

3.80	3.78	3.61	3.95E-01	5.64E-01
6.62	6.76	6.69	3.95E-01	5.64E-01
7.44	7.69	7.49	3.95E-01	5.64E-01
8.84	8.91	8.81	3.95E-01	5.64E-01
6.95	6.90	6.96	3.95E-01	5.64E-01
6.25	6.44	6.30	3.95E-01	5.64E-01
12.69	12.88	12.81	3.95E-01	5.64E-01
7.16	6.93	7.14	3.95E-01	5.64E-01
9.27	9.12	9.34	3.95E-01	5.64E-01
5.23	4.75	5.09	3.95E-01	5.64E-01
4.31	4.35	4.67	3.95E-01	5.64E-01
11.35	11.53	11.38	3.95E-01	5.64E-01
7.87	8.10	7.95	3.95E-01	5.64E-01
5.87	5.54	5.40	3.95E-01	5.64E-01
9.48	9.09	9.48	3.96E-01	5.64E-01
6.08	6.34	5.99	3.96E-01	5.64E-01
9.22	9.20	9.27	3.96E-01	5.64E-01
3.36	3.72	3.73	3.96E-01	5.64E-01
4.95	4.95	4.89	3.96E-01	5.64E-01
10.02	9.78	9.98	3.96E-01	5.64E-01
8.55	8.46	8.56	3.96E-01	5.64E-01
9.96	10.02	10.02	3.96E-01	5.64E-01
8.67	8.70	8.80	3.96E-01	5.64E-01
3.76	3.80	3.60	3.96E-01	5.64E-01
3.47	3.60	3.66	3.96E-01	5.64E-01
7.01	7.25	7.17	3.96E-01	5.64E-01
5.53	5.44	5.75	3.96E-01	5.64E-01
6.43	6.38	6.26	3.96E-01	5.64E-01
5.88	5.91	5.96	3.96E-01	5.64E-01
4.66	4.78	4.99	3.96E-01	5.64E-01
3.23	3.35	3.27	3.96E-01	5.64E-01
3.96	4.13	4.19	3.96E-01	5.64E-01
6.59	6.57	6.48	3.96E-01	5.64E-01
5.58	5.42	5.33	3.96E-01	5.64E-01
7.47	7.58	7.57	3.96E-01	5.64E-01
6.02	6.20	6.11	3.96E-01	5.64E-01
8.05	8.07	8.03	3.96E-01	5.64E-01
6.66	6.46	6.67	3.96E-01	5.64E-01
6.77	6.99	6.93	3.96E-01	5.64E-01
6.97	7.00	6.98	3.96E-01	5.64E-01
11.09	10.88	11.05	3.97E-01	5.64E-01
5.37	5.43	5.48	3.97E-01	5.64E-01
3.58	3.59	3.32	3.97E-01	5.64E-01
8.42	8.06	8.36	3.97E-01	5.64E-01
7.67	7.44	7.47	3.97E-01	5.64E-01
3.47	3.50	3.37	3.97E-01	5.64E-01
5.91	5.80	5.81	3.97E-01	5.64E-01

9.89	10.03	10.00	3.97E-01	5.64E-01
5.03	4.54	4.80	3.97E-01	5.64E-01
5.98	5.74	5.79	3.97E-01	5.64E-01
9.58	9.43	9.58	3.97E-01	5.64E-01
5.58	5.51	5.67	3.97E-01	5.64E-01
3.16	3.35	3.43	3.97E-01	5.64E-01
11.00	11.28	11.17	3.97E-01	5.64E-01
4.35	4.32	4.19	3.97E-01	5.64E-01
9.60	9.89	9.65	3.97E-01	5.64E-01
9.75	9.96	9.79	3.97E-01	5.64E-01
5.23	5.43	5.48	3.97E-01	5.64E-01
14.42	14.45	14.46	3.97E-01	5.64E-01
6.30	6.30	6.41	3.97E-01	5.64E-01
5.82	5.53	5.59	3.97E-01	5.64E-01
11.83	11.92	11.85	3.98E-01	5.64E-01
8.42	8.80	8.75	3.98E-01	5.64E-01
6.69	6.67	6.74	3.98E-01	5.64E-01
10.26	9.86	10.26	3.98E-01	5.64E-01
8.40	8.57	8.51	3.98E-01	5.64E-01
5.98	5.83	5.94	3.98E-01	5.64E-01
11.16	11.38	11.28	3.98E-01	5.64E-01
6.07	5.91	5.90	3.98E-01	5.64E-01
8.13	8.28	8.14	3.98E-01	5.64E-01
4.06	3.95	3.97	3.98E-01	5.64E-01
5.21	5.15	5.28	3.98E-01	5.64E-01
6.35	6.21	6.23	3.98E-01	5.64E-01
5.08	4.79	4.86	3.98E-01	5.64E-01
3.79	3.64	3.67	3.98E-01	5.64E-01
7.55	7.74	7.72	3.98E-01	5.64E-01
7.43	7.50	7.56	3.98E-01	5.64E-01
3.43	3.69	3.40	3.98E-01	5.64E-01
6.21	6.39	6.43	3.98E-01	5.64E-01
5.13	5.40	4.98	3.98E-01	5.64E-01
5.49	5.63	5.38	3.98E-01	5.64E-01
4.84	4.88	4.57	3.98E-01	5.64E-01
3.13	3.27	3.27	3.98E-01	5.64E-01
5.35	5.58	5.68	3.98E-01	5.64E-01
5.76	5.57	5.66	3.98E-01	5.64E-01
5.12	4.91	4.78	3.98E-01	5.64E-01
6.94	7.02	7.42	3.99E-01	5.64E-01
7.26	7.18	7.23	3.99E-01	5.64E-01
3.91	3.84	3.85	3.99E-01	5.64E-01
3.76	3.44	3.66	3.99E-01	5.64E-01
12.59	12.87	12.67	3.99E-01	5.64E-01
5.38	4.91	5.28	3.99E-01	5.64E-01
6.20	6.23	6.41	3.99E-01	5.64E-01
12.33	12.12	12.37	3.99E-01	5.64E-01

7.25	7.35	7.23	3.99E-01	5.64E-01
3.73	3.55	3.41	3.99E-01	5.64E-01
4.39	4.37	4.40	3.99E-01	5.64E-01
6.26	6.23	6.22	3.99E-01	5.64E-01
7.39	7.50	7.64	3.99E-01	5.64E-01
8.62	8.77	8.64	3.99E-01	5.64E-01
8.99	9.17	9.15	3.99E-01	5.64E-01
4.00	3.93	3.92	3.99E-01	5.65E-01
7.37	7.34	7.42	3.99E-01	5.65E-01
10.14	10.36	10.17	3.99E-01	5.65E-01
7.42	7.37	7.58	3.99E-01	5.65E-01
11.29	11.51	11.32	3.99E-01	5.65E-01
9.09	9.01	9.01	3.99E-01	5.65E-01
3.28	3.31	3.18	3.99E-01	5.65E-01
4.39	4.22	4.49	3.99E-01	5.65E-01
9.30	8.81	9.12	4.00E-01	5.65E-01
9.11	9.29	9.17	4.00E-01	5.65E-01
5.74	5.86	5.84	4.00E-01	5.65E-01
7.41	7.55	7.63	4.00E-01	5.65E-01
8.18	8.35	8.19	4.00E-01	5.65E-01
3.86	4.16	4.24	4.00E-01	5.65E-01
7.28	7.36	7.18	4.00E-01	5.65E-01
9.17	9.42	9.31	4.00E-01	5.65E-01
4.61	4.48	4.68	4.00E-01	5.65E-01
6.70	6.68	6.75	4.00E-01	5.65E-01
4.95	4.86	4.67	4.00E-01	5.65E-01
6.69	6.65	6.83	4.00E-01	5.65E-01
5.13	5.36	5.27	4.00E-01	5.65E-01
6.93	6.64	7.01	4.00E-01	5.65E-01
7.09	7.31	7.04	4.00E-01	5.65E-01
8.63	9.04	8.77	4.00E-01	5.65E-01
4.35	4.43	4.49	4.00E-01	5.65E-01
4.58	4.53	4.20	4.00E-01	5.65E-01
6.62	6.46	6.50	4.00E-01	5.65E-01
10.87	11.23	11.07	4.00E-01	5.65E-01
4.91	4.86	4.73	4.00E-01	5.65E-01
8.82	9.44	9.29	4.00E-01	5.65E-01
8.50	8.60	8.46	4.00E-01	5.65E-01
9.85	9.92	9.99	4.00E-01	5.65E-01
4.87	4.78	4.84	4.01E-01	5.65E-01
5.22	5.62	5.17	4.01E-01	5.65E-01
5.25	5.23	4.79	4.01E-01	5.65E-01
4.20	4.07	4.11	4.01E-01	5.65E-01
7.67	7.69	7.56	4.01E-01	5.65E-01
5.64	5.60	5.69	4.01E-01	5.65E-01
7.48	7.61	7.70	4.01E-01	5.65E-01
9.65	9.64	9.80	4.01E-01	5.65E-01

8.39	8.72	8.46	4.01E-01	5.65E-01
5.02	5.24	5.07	4.01E-01	5.65E-01
3.38	3.52	3.36	4.01E-01	5.65E-01
6.22	6.12	6.19	4.01E-01	5.65E-01
3.32	3.38	3.60	4.01E-01	5.65E-01
7.26	7.18	7.13	4.01E-01	5.65E-01
5.79	5.76	5.80	4.01E-01	5.65E-01
7.55	7.65	7.59	4.01E-01	5.65E-01
8.78	8.88	8.77	4.01E-01	5.65E-01
6.30	6.33	6.21	4.01E-01	5.65E-01
8.78	8.82	8.94	4.01E-01	5.65E-01
9.90	9.84	10.16	4.02E-01	5.65E-01
6.91	6.91	6.97	4.02E-01	5.65E-01
9.69	10.03	9.86	4.02E-01	5.65E-01
6.52	6.65	6.31	4.02E-01	5.65E-01
8.53	8.65	8.55	4.02E-01	5.65E-01
3.72	3.57	3.57	4.02E-01	5.65E-01
7.70	7.81	7.80	4.02E-01	5.65E-01
4.20	3.89	4.21	4.02E-01	5.65E-01
5.10	4.87	4.98	4.02E-01	5.66E-01
4.70	4.87	4.48	4.02E-01	5.66E-01
5.87	6.08	6.02	4.02E-01	5.66E-01
6.64	6.43	6.71	4.02E-01	5.66E-01
9.81	9.74	9.83	4.02E-01	5.66E-01
3.67	3.73	4.00	4.02E-01	5.66E-01
5.03	4.48	4.83	4.02E-01	5.66E-01
7.99	7.75	7.91	4.02E-01	5.66E-01
8.18	8.09	7.96	4.02E-01	5.66E-01
6.12	6.08	6.50	4.02E-01	5.66E-01
3.63	3.55	3.59	4.03E-01	5.66E-01
9.32	9.49	9.28	4.03E-01	5.66E-01
7.99	8.18	8.02	4.03E-01	5.66E-01
6.26	5.87	6.58	4.03E-01	5.66E-01
3.40	3.35	3.38	4.03E-01	5.66E-01
4.22	3.85	4.12	4.03E-01	5.66E-01
7.82	7.98	7.90	4.03E-01	5.66E-01
9.57	9.23	9.66	4.03E-01	5.66E-01
3.78	3.66	3.70	4.03E-01	5.66E-01
3.94	4.18	4.00	4.03E-01	5.66E-01
8.38	8.35	8.39	4.03E-01	5.66E-01
6.35	6.49	6.53	4.03E-01	5.66E-01
9.07	9.07	9.11	4.03E-01	5.66E-01
8.04	7.89	8.16	4.03E-01	5.66E-01
7.81	7.96	7.82	4.03E-01	5.66E-01
7.85	7.79	7.73	4.03E-01	5.66E-01
5.85	5.48	5.83	4.03E-01	5.66E-01
10.99	11.29	11.16	4.03E-01	5.66E-01

8.28	8.31	8.36	4.03E-01	5.66E-01
8.54	8.84	8.58	4.03E-01	5.66E-01
6.14	5.92	6.05	4.03E-01	5.66E-01
7.26	7.23	7.40	4.03E-01	5.66E-01
4.17	3.90	3.95	4.04E-01	5.66E-01
4.28	4.33	3.86	4.04E-01	5.66E-01
9.20	9.18	9.26	4.04E-01	5.66E-01
5.18	5.29	5.21	4.04E-01	5.66E-01
6.29	6.12	6.13	4.04E-01	5.66E-01
8.30	8.47	8.36	4.04E-01	5.66E-01
6.24	6.32	6.30	4.04E-01	5.66E-01
5.27	5.12	5.22	4.04E-01	5.66E-01
8.38	8.39	8.41	4.04E-01	5.66E-01
7.15	7.04	7.36	4.04E-01	5.66E-01
10.99	10.66	11.05	4.04E-01	5.66E-01
10.39	10.37	10.46	4.04E-01	5.66E-01
5.63	5.54	5.56	4.04E-01	5.66E-01
7.38	7.47	7.40	4.04E-01	5.66E-01
6.50	6.42	6.38	4.04E-01	5.66E-01
5.68	5.85	5.80	4.04E-01	5.66E-01
4.56	4.37	4.49	4.04E-01	5.66E-01
5.78	5.90	5.86	4.04E-01	5.66E-01
3.51	3.59	3.38	4.04E-01	5.66E-01
7.01	7.04	7.05	4.04E-01	5.66E-01
7.44	7.26	7.41	4.04E-01	5.66E-01
12.64	12.62	12.67	4.04E-01	5.67E-01
4.58	4.72	4.57	4.05E-01	5.67E-01
6.82	7.11	7.14	4.05E-01	5.67E-01
14.89	14.87	14.86	4.05E-01	5.67E-01
4.39	4.42	4.06	4.05E-01	5.67E-01
4.97	4.97	4.78	4.05E-01	5.67E-01
6.54	6.58	6.35	4.05E-01	5.67E-01
5.02	5.10	5.01	4.05E-01	5.67E-01
9.70	9.83	9.74	4.05E-01	5.67E-01
7.83	8.06	7.96	4.05E-01	5.67E-01
6.74	6.82	6.72	4.05E-01	5.67E-01
4.17	4.27	4.26	4.05E-01	5.67E-01
7.48	7.48	7.38	4.05E-01	5.67E-01
7.06	6.84	6.93	4.05E-01	5.67E-01
3.37	3.29	3.44	4.05E-01	5.67E-01
9.38	9.36	9.37	4.05E-01	5.67E-01
7.63	7.68	7.55	4.05E-01	5.67E-01
6.13	5.90	6.17	4.05E-01	5.67E-01
4.89	4.71	4.75	4.05E-01	5.67E-01
10.31	10.15	10.28	4.05E-01	5.67E-01
5.96	5.92	5.90	4.05E-01	5.67E-01
10.38	10.26	10.35	4.06E-01	5.67E-01

6.10	6.09	6.11	4.06E-01	5.67E-01
6.72	6.54	6.89	4.06E-01	5.67E-01
3.56	3.53	3.50	4.06E-01	5.67E-01
3.62	3.53	3.62	4.06E-01	5.67E-01
5.94	5.76	5.83	4.06E-01	5.67E-01
4.80	4.80	4.40	4.06E-01	5.67E-01
9.36	9.21	9.37	4.06E-01	5.67E-01
8.70	8.86	8.64	4.06E-01	5.67E-01
5.92	5.57	5.64	4.06E-01	5.67E-01
11.63	11.73	11.74	4.06E-01	5.67E-01
6.03	6.06	6.13	4.06E-01	5.67E-01
10.49	10.35	10.51	4.06E-01	5.67E-01
10.18	10.34	10.31	4.06E-01	5.67E-01
4.41	4.22	4.52	4.06E-01	5.67E-01
5.54	5.60	5.56	4.06E-01	5.67E-01
5.07	5.31	5.37	4.06E-01	5.67E-01
9.52	9.79	9.68	4.06E-01	5.67E-01
6.45	6.39	6.41	4.06E-01	5.67E-01
8.75	8.59	8.81	4.06E-01	5.67E-01
6.93	6.90	6.98	4.07E-01	5.67E-01
4.63	4.69	4.47	4.07E-01	5.67E-01
6.91	7.07	6.87	4.07E-01	5.67E-01
8.31	8.26	8.35	4.07E-01	5.67E-01
6.47	6.53	6.58	4.07E-01	5.67E-01
6.15	6.12	6.13	4.07E-01	5.67E-01
8.09	8.19	8.06	4.07E-01	5.67E-01
3.68	3.75	3.70	4.07E-01	5.67E-01
3.88	3.65	3.90	4.07E-01	5.67E-01
8.45	8.63	8.59	4.07E-01	5.67E-01
3.75	3.66	3.83	4.07E-01	5.67E-01
7.63	7.79	7.69	4.07E-01	5.67E-01
9.45	9.62	9.42	4.07E-01	5.67E-01
3.90	4.22	4.30	4.07E-01	5.67E-01
4.71	4.71	4.66	4.07E-01	5.67E-01
7.89	7.76	7.90	4.07E-01	5.67E-01
5.25	5.24	5.13	4.07E-01	5.67E-01
7.12	6.69	7.12	4.07E-01	5.67E-01
3.31	3.37	3.43	4.07E-01	5.67E-01
8.63	8.75	8.78	4.07E-01	5.67E-01
7.17	7.43	7.25	4.07E-01	5.68E-01
6.75	6.92	7.06	4.07E-01	5.68E-01
8.27	8.37	8.32	4.08E-01	5.68E-01
6.45	6.63	6.61	4.08E-01	5.68E-01
9.05	8.91	9.05	4.08E-01	5.68E-01
5.58	5.54	5.70	4.08E-01	5.68E-01
3.30	3.44	3.21	4.08E-01	5.68E-01
5.50	5.45	5.29	4.08E-01	5.68E-01

11.98	12.03	11.92	4.08E-01	5.68E-01
9.69	9.73	9.86	4.08E-01	5.68E-01
3.97	4.05	4.08	4.08E-01	5.68E-01
4.41	4.39	4.26	4.08E-01	5.68E-01
3.59	3.31	3.29	4.08E-01	5.68E-01
7.50	7.50	7.55	4.08E-01	5.68E-01
4.16	4.07	3.91	4.08E-01	5.68E-01
4.74	4.41	4.63	4.08E-01	5.68E-01
4.32	4.30	4.35	4.08E-01	5.68E-01
4.22	3.95	4.27	4.08E-01	5.68E-01
5.61	5.66	5.63	4.08E-01	5.68E-01
4.35	4.47	4.58	4.08E-01	5.68E-01
6.77	6.64	6.70	4.08E-01	5.68E-01
7.32	7.41	7.44	4.08E-01	5.68E-01
10.36	10.24	10.24	4.09E-01	5.68E-01
8.79	8.80	8.84	4.09E-01	5.68E-01
13.25	13.44	13.25	4.09E-01	5.68E-01
12.60	12.75	12.70	4.09E-01	5.68E-01
7.89	7.91	7.91	4.09E-01	5.68E-01
5.64	5.81	5.87	4.09E-01	5.68E-01
9.02	8.75	9.04	4.09E-01	5.68E-01
3.54	3.72	3.65	4.09E-01	5.68E-01
7.72	8.03	7.88	4.09E-01	5.68E-01
5.55	5.58	5.73	4.09E-01	5.68E-01
8.52	8.56	8.36	4.09E-01	5.68E-01
11.23	11.26	11.20	4.09E-01	5.68E-01
4.93	4.97	4.71	4.09E-01	5.68E-01
4.92	4.59	5.04	4.09E-01	5.68E-01
10.95	10.92	10.88	4.09E-01	5.68E-01
4.71	4.67	4.62	4.09E-01	5.68E-01
4.57	4.42	4.68	4.09E-01	5.68E-01
5.70	6.15	5.90	4.09E-01	5.68E-01
7.06	7.13	7.03	4.09E-01	5.68E-01
7.50	7.34	7.61	4.10E-01	5.69E-01
8.79	8.85	8.81	4.10E-01	5.69E-01
11.48	11.41	11.59	4.10E-01	5.69E-01
8.32	8.48	8.44	4.10E-01	5.69E-01
4.42	4.41	4.51	4.10E-01	5.69E-01
5.37	5.42	5.47	4.10E-01	5.69E-01
10.50	10.47	10.69	4.10E-01	5.69E-01
6.53	6.60	6.50	4.10E-01	5.69E-01
3.41	3.34	3.27	4.10E-01	5.69E-01
6.19	6.11	6.36	4.10E-01	5.69E-01
3.37	3.66	3.45	4.10E-01	5.69E-01
10.61	10.85	10.63	4.10E-01	5.69E-01
3.55	3.61	3.61	4.10E-01	5.69E-01
3.60	3.60	3.23	4.10E-01	5.69E-01

8.17	8.35	8.35	4.10E-01	5.69E-01
6.77	6.79	6.70	4.10E-01	5.69E-01
9.62	9.38	9.59	4.10E-01	5.69E-01
4.39	4.36	4.37	4.10E-01	5.69E-01
3.28	3.33	3.41	4.10E-01	5.69E-01
9.14	9.41	9.33	4.10E-01	5.69E-01
4.56	4.33	4.40	4.10E-01	5.69E-01
3.33	3.20	3.46	4.11E-01	5.69E-01
14.42	14.45	14.48	4.11E-01	5.69E-01
3.70	3.43	3.42	4.11E-01	5.69E-01
3.97	3.53	4.00	4.11E-01	5.69E-01
8.18	8.25	8.35	4.11E-01	5.69E-01
7.70	7.49	7.67	4.11E-01	5.69E-01
6.63	6.95	6.94	4.11E-01	5.69E-01
12.35	12.25	12.41	4.11E-01	5.69E-01
5.89	6.31	6.10	4.11E-01	5.69E-01
4.56	4.51	4.61	4.11E-01	5.69E-01
6.94	7.11	7.16	4.11E-01	5.69E-01
4.30	4.37	4.07	4.11E-01	5.69E-01
6.35	6.35	6.45	4.11E-01	5.70E-01
5.94	5.87	5.91	4.11E-01	5.70E-01
10.82	10.97	10.91	4.12E-01	5.70E-01
11.97	11.72	11.87	4.12E-01	5.70E-01
6.05	6.17	6.18	4.12E-01	5.70E-01
8.64	9.02	8.78	4.12E-01	5.70E-01
3.50	3.95	3.97	4.12E-01	5.70E-01
6.32	6.53	6.53	4.12E-01	5.70E-01
5.21	4.97	5.30	4.12E-01	5.70E-01
8.84	8.85	8.95	4.12E-01	5.70E-01
8.46	8.12	8.34	4.12E-01	5.70E-01
7.12	7.13	7.32	4.12E-01	5.70E-01
6.12	6.43	6.32	4.12E-01	5.70E-01
7.98	8.20	8.04	4.12E-01	5.70E-01
5.93	5.72	5.72	4.12E-01	5.70E-01
6.98	7.15	7.04	4.12E-01	5.70E-01
10.08	10.35	10.22	4.12E-01	5.70E-01
3.58	4.09	3.96	4.12E-01	5.70E-01
5.44	5.53	5.23	4.12E-01	5.70E-01
6.73	6.38	6.73	4.13E-01	5.70E-01
5.20	4.91	5.04	4.13E-01	5.70E-01
3.58	3.75	3.92	4.13E-01	5.71E-01
7.79	7.56	7.66	4.13E-01	5.71E-01
3.75	3.69	3.61	4.13E-01	5.71E-01
6.61	6.39	6.37	4.13E-01	5.71E-01
8.67	8.65	8.70	4.13E-01	5.71E-01
5.81	5.70	5.74	4.13E-01	5.71E-01
10.29	9.93	10.15	4.13E-01	5.71E-01

4.40	4.53	4.41	4.13E-01	5.71E-01
6.98	7.22	7.27	4.13E-01	5.71E-01
6.43	6.39	6.46	4.13E-01	5.71E-01
3.18	3.33	3.28	4.13E-01	5.71E-01
6.28	6.12	6.32	4.13E-01	5.71E-01
3.82	3.88	3.77	4.13E-01	5.71E-01
5.91	5.85	5.98	4.13E-01	5.71E-01
6.66	6.52	6.44	4.13E-01	5.71E-01
6.46	6.74	6.44	4.14E-01	5.71E-01
6.40	6.35	6.34	4.14E-01	5.71E-01
4.16	3.99	3.99	4.14E-01	5.71E-01
11.12	10.94	11.10	4.14E-01	5.71E-01
8.90	8.93	9.04	4.14E-01	5.71E-01
3.79	3.79	3.80	4.14E-01	5.71E-01
3.43	3.35	3.09	4.14E-01	5.71E-01
4.46	4.30	4.34	4.14E-01	5.71E-01
3.69	3.98	3.76	4.14E-01	5.71E-01
6.86	6.96	6.96	4.14E-01	5.71E-01
7.65	7.59	7.80	4.14E-01	5.71E-01
5.07	4.70	5.22	4.14E-01	5.71E-01
4.14	3.88	4.17	4.14E-01	5.71E-01
7.79	7.92	7.93	4.14E-01	5.71E-01
9.27	9.24	9.16	4.14E-01	5.71E-01
8.33	8.24	8.40	4.14E-01	5.71E-01
10.28	10.63	10.33	4.14E-01	5.71E-01
3.97	3.95	3.95	4.14E-01	5.71E-01
4.91	4.75	4.71	4.14E-01	5.71E-01
5.76	5.38	5.79	4.14E-01	5.71E-01
10.83	10.47	10.81	4.14E-01	5.71E-01
5.31	5.21	5.13	4.14E-01	5.71E-01
10.87	10.65	10.85	4.14E-01	5.71E-01
3.39	3.22	3.46	4.14E-01	5.71E-01
7.15	7.13	7.07	4.14E-01	5.71E-01
5.53	5.45	5.44	4.15E-01	5.71E-01
3.90	3.77	3.85	4.15E-01	5.71E-01
7.42	7.27	7.49	4.15E-01	5.71E-01
9.89	10.13	10.01	4.15E-01	5.71E-01
9.77	9.73	9.88	4.15E-01	5.71E-01
10.89	10.57	10.83	4.15E-01	5.71E-01
4.50	4.73	4.69	4.15E-01	5.71E-01
6.31	6.33	6.14	4.15E-01	5.71E-01
10.27	10.18	10.12	4.15E-01	5.71E-01
5.28	5.15	5.42	4.15E-01	5.71E-01
5.81	5.66	6.29	4.15E-01	5.71E-01
4.30	4.34	4.03	4.15E-01	5.71E-01
4.73	4.73	4.70	4.15E-01	5.71E-01
5.94	5.84	5.94	4.15E-01	5.71E-01

4.47	4.64	4.59	4.15E-01	5.71E-01
3.84	3.87	3.87	4.15E-01	5.71E-01
5.42	5.62	5.35	4.15E-01	5.71E-01
8.91	9.05	9.13	4.15E-01	5.71E-01
4.31	4.45	4.46	4.15E-01	5.71E-01
8.07	8.09	8.24	4.15E-01	5.71E-01
4.53	4.48	4.57	4.15E-01	5.71E-01
6.54	6.78	6.72	4.15E-01	5.71E-01
7.41	7.53	7.41	4.15E-01	5.71E-01
7.67	7.55	7.57	4.15E-01	5.71E-01
5.44	5.54	5.66	4.15E-01	5.71E-01
10.25	10.28	10.24	4.15E-01	5.71E-01
3.94	3.90	3.72	4.16E-01	5.71E-01
6.75	6.71	6.98	4.16E-01	5.71E-01
5.20	4.93	4.95	4.16E-01	5.71E-01
6.53	6.80	6.60	4.16E-01	5.71E-01
8.36	8.28	8.21	4.16E-01	5.71E-01
6.47	6.66	6.30	4.16E-01	5.71E-01
11.21	11.43	11.37	4.16E-01	5.71E-01
8.98	8.69	8.81	4.16E-01	5.71E-01
7.91	7.95	7.94	4.16E-01	5.71E-01
4.39	4.50	4.25	4.16E-01	5.71E-01
12.14	12.18	12.15	4.16E-01	5.71E-01
5.64	5.55	5.63	4.16E-01	5.71E-01
6.11	5.98	6.10	4.16E-01	5.71E-01
8.53	8.55	8.26	4.16E-01	5.71E-01
3.21	3.36	3.29	4.16E-01	5.71E-01
3.62	3.63	3.38	4.16E-01	5.71E-01
7.18	7.29	7.18	4.16E-01	5.71E-01
7.88	7.93	7.94	4.16E-01	5.71E-01
7.30	7.14	7.19	4.16E-01	5.71E-01
4.10	3.97	3.95	4.16E-01	5.71E-01
7.21	7.16	7.30	4.16E-01	5.71E-01
7.79	8.10	7.97	4.16E-01	5.71E-01
5.49	5.42	5.49	4.16E-01	5.71E-01
5.49	5.30	5.11	4.16E-01	5.71E-01
3.44	3.23	3.35	4.16E-01	5.71E-01
6.37	6.33	6.10	4.16E-01	5.71E-01
6.53	6.82	6.71	4.17E-01	5.71E-01
3.49	3.63	3.44	4.17E-01	5.71E-01
3.47	3.34	3.31	4.17E-01	5.71E-01
3.85	3.95	3.98	4.17E-01	5.71E-01
11.79	11.86	11.81	4.17E-01	5.71E-01
6.94	6.66	6.73	4.17E-01	5.71E-01
9.12	9.17	9.18	4.17E-01	5.71E-01
8.26	8.52	8.41	4.17E-01	5.71E-01
10.81	10.81	10.71	4.17E-01	5.71E-01

8.95	9.22	9.08	4.17E-01	5.71E-01
3.87	3.85	3.98	4.17E-01	5.71E-01
6.23	6.54	6.53	4.17E-01	5.71E-01
6.81	6.75	6.75	4.17E-01	5.71E-01
5.73	5.79	5.88	4.17E-01	5.71E-01
3.95	3.99	3.88	4.17E-01	5.71E-01
11.80	11.94	11.79	4.17E-01	5.71E-01
9.59	9.88	9.78	4.17E-01	5.71E-01
6.82	6.56	6.62	4.17E-01	5.71E-01
4.39	4.42	4.51	4.17E-01	5.71E-01
7.33	6.99	7.08	4.17E-01	5.71E-01
7.50	7.27	7.69	4.17E-01	5.71E-01
11.35	11.04	11.18	4.17E-01	5.71E-01
3.82	3.60	3.72	4.17E-01	5.71E-01
7.32	7.47	7.44	4.17E-01	5.71E-01
7.91	7.78	7.82	4.17E-01	5.71E-01
7.89	7.74	7.80	4.18E-01	5.71E-01
3.05	3.30	3.20	4.18E-01	5.71E-01
9.62	9.73	9.63	4.18E-01	5.71E-01
4.42	4.34	4.50	4.18E-01	5.71E-01
10.40	10.66	10.56	4.18E-01	5.71E-01
4.57	4.67	4.75	4.18E-01	5.71E-01
8.77	8.63	8.90	4.18E-01	5.71E-01
5.37	5.69	5.56	4.18E-01	5.71E-01
7.40	7.64	7.77	4.18E-01	5.71E-01
6.85	7.00	6.72	4.18E-01	5.71E-01
10.01	10.11	10.05	4.18E-01	5.71E-01
7.06	7.23	7.02	4.18E-01	5.71E-01
5.56	5.68	5.52	4.18E-01	5.71E-01
5.04	4.86	4.91	4.18E-01	5.71E-01
3.72	3.90	3.56	4.18E-01	5.71E-01
10.36	10.62	10.46	4.18E-01	5.71E-01
7.69	7.91	7.85	4.18E-01	5.71E-01
8.03	8.18	8.10	4.18E-01	5.71E-01
8.18	8.37	8.34	4.18E-01	5.71E-01
6.27	6.63	6.74	4.18E-01	5.71E-01
5.07	5.23	5.05	4.18E-01	5.71E-01
5.19	5.42	5.19	4.18E-01	5.71E-01
7.74	7.58	7.78	4.18E-01	5.71E-01
7.15	7.04	7.17	4.18E-01	5.71E-01
8.48	8.57	8.44	4.18E-01	5.71E-01
8.33	8.10	8.18	4.18E-01	5.72E-01
9.55	9.54	9.62	4.19E-01	5.72E-01
8.41	8.39	8.52	4.19E-01	5.72E-01
10.25	10.12	10.13	4.19E-01	5.72E-01
5.37	5.27	5.54	4.19E-01	5.72E-01
4.33	4.12	4.33	4.19E-01	5.72E-01

7.04	6.81	6.96	4.19E-01	5.72E-01
10.93	11.19	10.85	4.19E-01	5.72E-01
7.08	6.67	7.02	4.19E-01	5.72E-01
4.62	4.38	4.12	4.19E-01	5.72E-01
3.85	4.17	3.90	4.19E-01	5.72E-01
4.66	4.22	4.67	4.19E-01	5.72E-01
6.97	6.95	7.09	4.19E-01	5.72E-01
10.08	10.15	10.03	4.19E-01	5.72E-01
10.95	10.91	10.97	4.19E-01	5.72E-01
7.47	7.65	7.42	4.19E-01	5.72E-01
5.01	5.28	5.12	4.20E-01	5.72E-01
12.26	12.26	12.32	4.20E-01	5.72E-01
5.26	5.16	5.12	4.20E-01	5.72E-01
8.65	8.48	8.67	4.20E-01	5.72E-01
7.58	7.63	7.61	4.20E-01	5.72E-01
10.00	10.17	10.11	4.20E-01	5.72E-01
9.76	9.66	9.77	4.20E-01	5.72E-01
7.23	7.26	7.34	4.20E-01	5.72E-01
5.43	5.40	5.43	4.20E-01	5.72E-01
8.46	8.71	8.53	4.20E-01	5.72E-01
3.88	3.75	3.84	4.20E-01	5.72E-01
4.13	4.24	4.45	4.20E-01	5.73E-01
9.83	10.01	9.95	4.20E-01	5.73E-01
4.68	4.67	4.80	4.20E-01	5.73E-01
8.57	8.30	8.69	4.20E-01	5.73E-01
5.17	5.18	5.33	4.20E-01	5.73E-01
5.58	5.10	5.25	4.20E-01	5.73E-01
10.33	10.06	10.17	4.21E-01	5.73E-01
5.52	5.62	5.57	4.21E-01	5.73E-01
8.04	8.22	8.36	4.21E-01	5.73E-01
9.28	9.66	9.46	4.21E-01	5.73E-01
8.59	8.35	8.68	4.21E-01	5.73E-01
8.68	8.77	8.74	4.21E-01	5.73E-01
10.59	10.91	10.76	4.21E-01	5.73E-01
3.70	3.84	3.74	4.21E-01	5.73E-01
3.32	3.36	3.33	4.21E-01	5.73E-01
5.87	5.86	5.90	4.21E-01	5.73E-01
5.64	5.54	5.79	4.21E-01	5.73E-01
8.80	8.75	8.83	4.21E-01	5.73E-01
8.27	8.49	8.16	4.21E-01	5.73E-01
5.35	5.26	5.20	4.21E-01	5.73E-01
8.22	8.30	8.30	4.21E-01	5.73E-01
7.06	7.01	7.19	4.21E-01	5.73E-01
5.50	5.54	5.61	4.21E-01	5.73E-01
6.20	6.30	6.20	4.21E-01	5.73E-01
7.97	8.08	8.27	4.21E-01	5.73E-01
6.26	6.35	6.33	4.21E-01	5.73E-01

[truncated: 394,810 more chars]
